# Leisure time television watching, computer use and risks of breast, colorectal and prostate cancer: A Mendelian randomisation analysis

**DOI:** 10.1002/cam4.6732

**Published:** 2023-12-28

**Authors:** Nikos Papadimitriou, Nabila Kazmi, Niki Dimou, Konstantinos K. Tsilidis, Richard M. Martin, Sarah J. Lewis, Brigid M. Lynch, Michael Hoffmeister, Sun‐Seog Kweon, Li Li, Roger L. Milne, Lori C. Sakoda, Robert E. Schoen, Amanda I. Phipps, Jane C. Figueiredo, Ulrike Peters, Suzanne C. Dixon‐Suen, Marc J. Gunter, Neil Murphy

**Affiliations:** ^1^ Nutrition and Metabolism Branch International Agency for Research on Cancer Lyon France; ^2^ MRC Integrative Epidemiology Unit (IEU) Population Health Sciences, Bristol Medical School, University of Bristol Bristol UK; ^3^ Department of Hygiene and Epidemiology University of Ioannina School of Medicine Ioannina Greece; ^4^ Department of Epidemiology and Biostatistics School of Public Health, Imperial College London London UK; ^5^ Department of Population Health Sciences Bristol Medical School, University of Bristol Bristol UK; ^6^ National Institute for Health Research (NIHR) Bristol Biomedical Research Centre University Hospitals Bristol, Weston NHS Foundation Trust, University of Bristol Bristol UK; ^7^ Cancer Epidemiology Division Cancer Council Victoria Melbourne Victoria Australia; ^8^ Centre for Epidemiology and Biostatistics Melbourne School of Population and Global Health, The University of Melbourne Melbourne Victoria Australia; ^9^ Physical Activity Laboratory Baker Heart and Diabetes Institute Melbourne Victoria Australia; ^10^ Division of Clinical Epidemiology and Aging Research German Cancer Research Center (DKFZ) Heidelberg Germany; ^11^ Department of Preventive Medicine Chonnam National University Medical School Gwangju Korea; ^12^ Jeonnam Regional Cancer Center Chonnam National University Hwasun Hospital Gwangju Korea; ^13^ Department of Family Medicine University of Virginia Charlottesville Virginia USA; ^14^ Precision Medicine School of Clinical Sciences at Monash Health, Monash University Clayton Victoria Australia; ^15^ Division of Research Kaiser Permanente Northern California Oakland California USA; ^16^ Public Health Sciences Division Fred Hutchinson Cancer Center Seattle Washington USA; ^17^ Department of Medicine and Epidemiology University of Pittsburgh Medical Center Pittsburgh Pennsylvania USA; ^18^ Department of Epidemiology University of Washington Seattle Washington USA; ^19^ Department of Medicine Samuel Oschin Comprehensive Cancer Institute, Cedars‐Sinai Medical Center Los Angeles California USA; ^20^ Department of Preventive Medicine Keck School of Medicine, University of Southern California Los Angeles California USA; ^21^ Institute for Physical Activity and Nutrition, School of Exercise and Nutrition Sciences, Deakin University Geelong Victoria Australia

**Keywords:** breast cancer, colorectal cancer, Mendelian randomisation, prostate cancer, sedentary activities

## Abstract

**Background:**

Sedentary behaviours have been associated with increased risks of some common cancers in epidemiological studies; however, it is unclear if these associations are causal.

**Methods:**

We used univariable and multivariable two‐sample Mendelian randomisation (MR) to examine potential causal relationships between sedentary behaviours and risks of breast, colorectal and prostate cancer. Genetic variants associated with self‐reported leisure television watching and computer use were identified from a recent genome‐wide association study (GWAS). Data related to cancer risk were obtained from cancer GWAS consortia. A series of sensitivity analyses were applied to examine the robustness of the results to the presence of confounding.

**Results:**

A 1‐standard deviation (SD: 1.5 h/day) increment in hours of television watching increased risk of breast cancer (OR per 1‐SD: 1.15, 95% confidence interval [CI]: 1.05–1.26) and colorectal cancer (OR per 1‐SD: 1.32, 95% CI: 1.16–1.49) while there was little evidence of an association for prostate cancer risk (OR per 1‐SD: 0.94, 95% CI: 0.84–1.06). After adjusting for years of education, the effect estimates for television watching were attenuated (breast cancer, OR per 1‐SD: 1.08, 95% CI: 0.92–1.27; colorectal cancer, OR per 1‐SD: 1.08, 95% CI: 0.90–1.31). Post hoc analyses showed that years of education might have a possible confounding and mediating role in the association between television watching with breast and colorectal cancer. Consistent results were observed for each cancer site according to sex (colorectal cancer), anatomical subsites and cancer subtypes. There was little evidence of associations between genetically predicted computer use and cancer risk.

**Conclusions:**

Our univariable analysis identified some positive associations between hours of television watching and risks of breast and colorectal cancer. However, further adjustment for additional lifestyle factors especially years of education attenuated these results. Future studies using objective measures of exposure can provide new insights into the possible role of sedentary behaviour in cancer development.

## 1 INTRODUCTION

Breast, colorectal and prostate cancer are three of the most common malignancies collectively accounting for an estimated 29% of new cancer cases in 2020.^1^ Sedentary behaviour is defined as any waking behaviour characterised by energy expenditure ≤1.5 metabolic equivalents while in a sitting, reclining or lying posture.^2^ The most common sedentary activities are television watching and computer use; these are more accurately recalled than total sedentary time and are therefore commonly used as surrogates of sedentary behaviour.^3^ A recent US study reported that approximately two‐thirds of adults spent two or more hours each day watching television and around 50% spend more than 1 h using their computer outside work.^4^ Studies in the United Kingdom and in the United States estimated that adults on average spend 5–6 h per day sitting.^4^, ^5^ Given such a high prevalence, sedentary behaviours represent an important public health challenge as they have been linked with multiple adverse health outcomes.^6^, ^7^

Numerous observational studies have examined the associations between sedentary behaviours and the risks of breast, colorectal and prostate cancer.^8^ A meta‐analysis of case–control and cohort studies reported that sedentary behaviour was not associated with colorectal cancer risk.^8^ More recently, however, a UK Biobank analysis found that greater volumes of television watching were associated with elevated colon cancer risk.^9^The aforementioned meta‐analysis did not observe any significant associations between sedentary behaviour and risk of prostate cancer.^8^ For breast cancer, when the meta‐analysis included cohort studies only, sedentary behaviour was associated with a higher breast cancer risk.^8^ Clarifying causal associations from such observational evidence is hampered by inherent biases of the study design, such as residual confounding and reverse causality.^10^, ^11^, ^12^ Mendelian randomisation (MR) is an alternative way to investigate potential causal associations. MR uses germline genetic variants as proxies (or instrumental variables) for exposures of interest to make causal inferences between an exposure and an outcome.^13^ Unlike traditional observational epidemiology, if all underlying assumptions are satisfied, MR can reduce conventional confounding owing to the random independent assignment of alleles during meiosis.^14^ In addition, multivariable MR methods have been developed to adjust for confounding if found to be present or for possible pleiotropy bias due to horizontal pleiotropy of a specific effect. MR studies should be less prone to reverse causation, as germline genetic variants are fixed at conception and are consequently unaffected by the disease process.^14^ Recent MR analyses reported a positive effect estimate for television watching and overall sedentary time with breast cancer risk.^15^, ^16^ However, these analyses either relied on a small number of instruments or were not very detailed in terms of cancer subtype. Furthermore, there is less evidence for colorectal and prostate cancers.^15^

We used a two‐sample MR framework to examine potential causal associations between self‐reported sedentary behaviours and risks of breast, colorectal and prostate cancer. Genetic variants associated with leisure television watching and computer use were identified from a recent genome‐wide association study (GWAS),^17^ and we then examined how these genetic variants related to risks of breast, colorectal and prostate cancer using large‐scale GWAS consortia data.^18^, ^19^, ^20^

## 2 MATERIALS AND METHODS

### 2.1 Data on leisure sedentary behaviours

Summary‐level data on duration of leisure sedentary behaviours for men and women combined were obtained from a recently published GWAS conducted in 408,815 participants of European ancestry from the UK Biobank using BOLT‐LMM v2.3beta2, using a mixed linear model correcting for population structure and cryptic relatedness.^17^ To ascertain the duration of the sedentary behaviours, participants within the UK Biobank were asked three questions, ‘In a typical DAY, how many hours do you spend watching television?’, ‘In a typical DAY, how many hours do you spend using the computer? (Do not include using a computer at work)’ and ‘In a typical DAY, how many hours do you spend driving?’.^17^ This GWAS identified 209 and 52 genome‐wide‐significant single nucleotide polymorphisms (SNPs) (*p* < 5 × 10^−8^) for leisure television watching and computer use, respectively, using a linkage disequilibrium (LD) of *R* ^2^ < 0.005 within a five megabase window (Tables S1 and S2). The GWAS also identified five genetic variants associated with driving; however, we did not include these instruments in our MR analyses due to low statistical power (see Statistical power, below). The 261 SNPs included in both instruments were identified in 204 loci demonstrating a partial overlap between the two phenotypes with 22 common loci. The selected SNPs explained approximately 2% and 0.5% of the variability in television watching and computer use, respectively.

### 2.2 Data on breast, colorectal and prostate cancer

Summary data for the associations of the above genetic variants with breast cancer were obtained from a GWAS of 247,173 women (133,384 breast cancer cases and 113,789 controls) of European ancestry from the Breast Cancer Association Consortium.^20^ We included six related outcomes in our analyses (overall, luminal A, luminal B, luminal B HER2 negative, HER2 enriched and triple negative breast cancer).

For colorectal cancer, summary data from 98,715 participants (52,775 colorectal cancer cases and 45,940 controls) were drawn from a meta‐analysis within the ColoRectal Transdisciplinary Study, the Colon Cancer Family Registry, and the Genetics and Epidemiology of Colorectal Cancer consortia.^18^ We included five outcomes in our analyses (overall colorectal cancer, colorectal cancer for men, colorectal cancer for women, colon cancer and rectal cancer). The summary statistics did not include UK Biobank study to avoid potential overlap with the leisure sedentary behaviours GWAS.

For prostate cancer, summary data from a meta‐analysis of 140,254 (79,148 prostate cancer cases and 61,106 controls) men of European ancestry in the Prostate Cancer Association Group to Investigate Cancer‐Associated Alterations in the Genome and the Genetic Associations and Mechanisms in Oncology/Elucidating Loci Involved in Prostate Cancer Susceptibility consortia.^19^ The same consortia also conducted a GWAS of aggressive prostate cancer involving 15,167 cases and 58,308 controls, in which cancer cases were defined as aggressive based on the following characteristics: Gleason score ≥8, Prostate‐Specific Antigen >100 ng/mL, metastatic disease (M1) or death from prostate cancer.^19^

All cancer estimates for the two exposures of interest are provided in Tables S3–S8. All participants provided written informed consent. Ethics were approved by respective institutional review boards.^17^, ^18^, ^19^, ^20^

### 2.3 Statistical power

The statistical power was calculated a priori using an online tool at http://cnsgenomics.com/shiny/mRnd/.^21^ Under the scenario of a type 1 error of 5%, for leisure television use an expected OR per 1 standard deviation (SD) ≥ 1.09, ≥ 1.14 and ≥1.11 was needed to have adequate statistical power (>80%) for overall breast, colorectal and prostate cancer, respectively. Table S9 presents the power estimates for the three exposures by breast, colorectal and prostate cancer.

### 2.4 Statistical analysis

A two‐sample MR approach using summary data and the fixed‐effect IVW method was implemented. All results correspond to an OR per 1‐SD increment in genetically predicted hours of leisure sedentary behaviour (television watching: 1.5 h/day; computer use: 1.2 h/day). The heterogeneity of the causal estimates by cancer subtype (breast cancer), subsite (colorectal cancer) and sex (colorectal cancer only) was investigated by calculating the *I* ^2^ metric using a fixed‐effect meta‐analysis model.^22^ Since some genetic variants were also associated with adiposity or education‐related phenotypes, we performed multivariable MR to investigate whether associations for sedentary behaviour are confounded by body mass index (BMI) and years of education, as well as lifetime smoking and alcohol consumption which have previously been linked with cancer risk.^23^, ^24^, ^25^

For BMI, summary data from a GWAS meta‐analysis of about 700,000 participants of European descent within the Genetic Investigation of ANthropometric Traits (GIANT) consortium and UK Biobank were obtained.^26^ For years of educational attainment, we obtained summary‐level data from a published GWAS of 1.1 million participants of European descent within the Social Science Genetic Association Consortium and which measured the number of completed years of schooling among those individuals.^27^ Data on alcohol consumption (drinks per week) were drawn from a GWAS of 1.2 million individuals.^28^ The data for lifetime smoking were obtained from a recent GWAS and MR study on causal effects of lifetime smoking on risk for depression and schizophrenia.^29^ In the current analysis, we used data from 766,345 participants which were publicly available. All relevant summary statistics for the multivariable MR analyses is given in Tables S10–S25. MR studies have three main assumptions that must be satisfied in order for their causal estimates to be valid, which in the context of this study are as follows: (1) The genetic instrument is strongly associated with the levels of exposure (sedentary behaviour); (2) the genetic instrument is not associated with any potential confounder of the exposure (sedentary behaviour)—outcome (cancer) association; and (3) the genetic instrument does not affect the outcome (cancer) independently of the exposure (sedentary behaviour) (i.e. exclusion of horizontal pleiotropy). The strength of each genetic instrument can be evaluated through the F‐statistic (provided by the initial GWAS).^17^ For multivariable MR, we also calculated the conditional F statistics which can be used to examine whether the genetic variants strongly predict each of the main (sedentary behaviours) and secondary exposures (e.g. years of education) conditional on the other exposure in the model; similar to univariable MR, *F* values over 10 suggest little evidence of weak instrument bias.^30^

### 2.5 Sensitivity analyses

Several sensitivity analyses were conducted to identify and correct for the presence of horizontal pleiotropy in the results from the main analysis. Cochran's *Q* was computed to quantify heterogeneity across the individual causal effects, with a *p* ≤ 0.05 indicating the presence of pleiotropy, and consequently, a random effects IVW MR analysis was used.^22^, ^31^ MR‐Egger regression provides valid MR estimates in the presence of horizontal pleiotropy when the pleiotropic effects of the genetic variants are independent from the genetic associations with the exposure.^32^ Large deviations from zero for the intercept test represent the presence of horizontal pleiotropic effects across the genetic variants. In such a case, the slope of the MR‐Egger regression provides valid MR estimates when the pleiotropic effects of the genetic variants are independent from the genetic associations with the exposure.^32^, ^33^ Moreover, causal estimates were also computed using the weighted‐median method that can give valid MR estimates under the presence of horizontal pleiotropy when up to 50% of the included instruments are invalid.^34^ The MR pleiotropy residual sum and outlier test (MR‐PRESSO) was also used to assess the presence of pleiotropy. The MR‐PRESSO test relies on a regression framework to identify outlying genetic variants which may potentially be pleiotropic, we then reran the analysis after excluding these outlying variants.^35^ We also examined the selected genetic instruments and their proxies (*r* ^2^ > 0.8) and their associations with secondary phenotypes (*p*‐value < 5 × 10^−8^) in populations of European descent in Phenoscanner (http://www.phenoscanner.medschl.cam.ac.uk/) to explore potential pleiotropy of the included SNPs. Finally, as a post hoc analysis based on the results from the multivariable MR and trying to understand the observed attenuation, we also conducted a bidirectional MR study to examine the associations between sedentary behaviours and the four secondary traits (BMI, years of education, alcohol consumption and lifetime smoking) (Tables S26–S33).

All the analyses were conducted using the MendelianRandomization and TwoSampleMR packages, while the LD clumping (LD < 0.001) in the multivariable MR analyses between SNPs of sedentary behaviour phenotypes with those for the secondary traits was done using the ieugwasr R package (https://mrcieu.github.io/ieugwasr/) and the R programming language (version 4.1.2).^36^, ^37^, ^38^ Reporting guidelines for MR studies were followed.^39^, ^40^

## 3 RESULTS

### 3.1 Baseline characteristics

For the sedentary behaviour GWAS, the average age of the participants was 57.4 (SD: 8.0) years old, and 45.7% were men. Mean daily reported time of leisure television watching and leisure computer use was 2.8 (SD: 1.5) and 1.0 (SD: 1.2) h, respectively. The mean BMI was 27.4 kg/m^2^, 55% were never smokers or quit >12 months ago, and 67% were physically active (i.e. ≥150 min/week moderate or ≥75 min/week vigorous or 150 min/week mixed [moderate and vigorous] activity) behaviour.^17^

### 3.2 MR estimates for leisure television watching

A 1 SD (1.5 h/day) increment in genetically predicted duration of leisure television watching increased breast cancer risk (OR per 1 SD: 1.15, 95% confidence interval [CI]: 1.05–1.26, *p*‐value: 0.002) (Table 1). Similar magnitude positive effect estimates were found for all molecular subtypes of breast cancer (*I* ^2^ = 0%, *p*‐heterogeneity = 0.98) (Table 1).

**TABLE 1:** Mendelian randomisation estimates for sedentary behaviour and breast cancer risk.

Methods	Leisure television watching				Leisure computer use			
	Estimates (OR)^a^	95% CI	*p*‐value	*p*‐value for pleiotropy^b^ or heterogeneity^c^	Estimates (OR)^a^	95% CI	*p*‐value	*p*‐value for pleiotropy^b^ or heterogeneity^c^
Breast cancer								
Inverse‐variance weighted	1.15	1.05–1.26	0.002	1 × 10^−17^	1.01	0.84–1.23	0.89	1 × 10^−9^
MR‐Egger	1.48	0.98–2.23	0.06	0.22	0.69	0.19–2.48	0.57	0.55
Weighted median	1.16	1.05–1.27	0.003		1.06	0.87–1.28	0.57	
MR‐PRESSO	1.12	1.03–1.20	0.008	3 × 10^−8^	1.04	0.88–1.23	0.62	8 × 10^−4^
Luminal A breast cancer								
Inverse‐variance weighted	1.20	1.06–1.35	0.002	6 × 10^−19^	1.06	0.84–1.34	0.62	4 × 10^−6^
MR‐Egger	1.55	0.90–2.69	0.11	0.34	1.58	0.35–7.10	0.55	0.60
Weighted median	1.15	1.01–1.31	0.03		1.06	0.83–1.35	0.66	
MR‐PRESSO	1.14	1.03–1.26	0.01	3 × 10^−7^	1.06	0.87–1.31	0.54	0.003
Luminal B breast cancer								
Inverse‐variance weighted	1.14	0.94–1.38	0.19	0.03	0.89	0.58–1.36	0.58	0.02
MR‐Egger	1.16	0.47–2.89	0.74	0.96	1.95	0.12–30.3	0.63	0.57
Weighted median	1.13	0.86–1.48	0.40		0.97	0.57–1.67	0.92	
MR‐PRESSO					0.82	0.57–1.17	0.28	0.11
Luminal B HER2 negative breast cancer								
Inverse‐variance weighted	1.14	0.96–1.36	0.13	0.004	1.03	0.76–1.40	0.84	0.19
MR‐Egger	1.07	0.48–2.39	0.86	0.88	0.27	0.04–2.25	0.23	0.22
Weighted median	1.30	1.03–1.63	0.03		1.15	0.76–1.75	0.52	
MR‐PRESSO								
HER2 enriched breast cancer								
Inverse‐variance weighted	1.21	0.91–1.60	0.19	0.02	0.67	0.40–1.13	0.13	0.69
MR‐Egger	1.31	0.35–4.95	0.68	0.90	0.08	0.00–2.16	0.13	0.20
Weighted median	1.25	0.84–1.86	0.28		0.65	0.31–1.35	0.25	
MR‐ PRESSO								
Triple negative breast cancer								
Inverse‐variance weighted	1.16	0.99–1.35	0.06	0.10	0.68	0.50–0.93	0.02	0.24
MR‐Egger	1.54	0.72–3.29	0.27	0.45	0.41	0.05–3.35	0.40	0.63
Weighted median	1.31	1.04–1.67	0.02		0.73	0.47–1.14	0.16	
MR‐PRESSO								

Abbreviations: CI, confidence interval; MR, Mendelian randomisation; OR, odds ratio; MR‐PRESSO, MR pleiotropy residual sum and outlier test.

^a^ The estimates correspond to a standard deviation increase in duration of sedentary activity.

^b^ *p*‐value or pleiotropy based on MR‐Egger intercept.

^c^ *p*‐value for heterogeneity based on Q statistic.

A 1 SD increment in genetically predicted duration of leisure television watching increased colorectal cancer risk (OR per 1 SD: 1.32, 95% CI: 1.16–1.49, *p*‐value: 2 × 10^−5^) with similar significant estimates being observed for men and women (*I* ^2^ = 42%, *p*‐heterogeneity = 0.19) and by subsite (*I* ^2^ = 45%, *p*‐heterogeneity = 0.17) (Table 2).

**TABLE 2:** Mendelian randomisation estimates for sedentary behaviour and colorectal cancer risk.

Methods	Leisure television watching				Leisure computer use			
	Estimates (OR)^a^	95% CI	*p*‐value	*p*‐value for pleiotropy^b^ or heterogeneity^c^	Estimates (OR)^a^	95% CI	*p*‐value	*p*‐value for pleiotropy^b^ or heterogeneity^c^
Colorectal cancer								
Inverse‐variance weighted	1.32	1.16–1.49	2 × 10^−5^	9 × 10^−9^	0.90	0.70–1.13	0.33	0.02
MR‐Egger	1.35	0.76–2.39	0.31	0.94	0.35	0.08–1.55	0.17	0.21
Weighted median	1.40	1.20–1.63	2 × 10^−5^		1.08	0.81–1.45	0.59	
MR‐PRESSO								
Colorectal cancer in men								
Inverse‐variance weighted	1.45	1.23–1.67	5 × 10^−6^	3 × 10^−3^	0.79	0.61–1.04	0.10	0.2
MR‐Egger	1.72	0.84–3.53	0.14	0.63	0.61	0.09–4.06	0.61	0.79
Weighted median	1.52	1.23–1.88	9 × 10^−5^		0.76	0.51–1.13	0.17	
MR‐PRESSO								
Colorectal cancer in women								
Inverse‐variance weighted	1.25	1.06–1.46	0.007	0.003	1.02	0.74–1.40	0.89	0.05
MR‐Egger	1.02	0.50–2.08	0.96	0.57	0.31	0.04–2.29	0.25	0.24
Weighted median	1.25	1.01–1.54	0.04		1.20	0.81–1.79	0.36	
MR‐PRESSO					1.08	0.83–1.42	0.58	0.27
Colon cancer								
Inverse‐variance weighted	1.36	1.19–1.57	2 × 10^−5^	5 × 10^−5^	0.90	0.72–1.14	0.42	0.06
MR‐Egger	1.48	0.78–2.80	0.24	0.80	0.26	0.05–1.42	0.12	0.14
Weighted median	1.49	1.25–1.79	2 × 10^−5^		0.96	0.68–1.34	0.82	
MR‐PRESSO								
Rectal cancer								
Inverse‐variance weighted	1.60	1.32–1.93	2 × 10^−6^	8 × 10^−7^	0.66	0.49–0.89	0.006	0.57
MR‐Egger	1.97	0.82–4.71	0.13	0.63	0.88	0.13–6.05	0.90	0.76
Weighted median	1.86	1.48–2.36	3 × 10^−7^		0.81	0.53–1.25	0.34	
MR‐PRESSO								

Abbreviations: CI, confidence interval; MR, Mendelian randomisation; OR, odds ratio; MR‐PRESSO, MR pleiotropy residual sum and outlier test.

^a^ The estimates correspond to a standard deviation increase in duration of sedentary activity.

^b^ *p*‐value or pleiotropy based on MR‐Egger intercept.

^c^ *p*‐value for heterogeneity based on *Q* statistic.

There was little evidence that a 1 SD increment in genetically predicted duration of leisure television watching was associated with risk of overall (OR per 1 SD: 0.94, 95% CI: 0.84–1.06, *p*‐value: 0.34) or aggressive (OR per 1 SD: 0.95, 95% CI: 0.81–1.13, *p*‐value: 0.59) prostate cancer (overall vs aggressive; *I* ^2^ = 0%, *p*‐heterogeneity = 0.92) (Table 3).

**TABLE 3:** Mendelian randomisation estimates for sedentary behaviour and prostate cancer risk.

Methods	Leisure television watching				Leisure computer use			
	Estimates (OR)^a^	95% CI	*p*‐value	*p*‐value for pleiotropy^b^ or heterogeneity^c^	Estimates (OR)^a^	95% CI	*p*‐value	*p*‐value for pleiotropy^b^ or heterogeneity^c^
Prostate cancer								
Inverse‐variance weighted	0.94	0.84–1.06	0.34	3 × 10^−12^	1.08	0.89–1.34	0.42	0.01
MR‐Egger	1.19	0.71–1.99	0.51	0.37	0.70	0.19–2.56	0.59	0.5
Weighted median	0.94	0.83–1.08	0.41		1.13	0.88–1.46	0.33	
MR‐PRESSO	0.92	0.84–1.02	0.13	1 × 10^−5^	1.14	0.96–1.35	0.13	0.09
Advanced prostate cancer								
Inverse‐variance weighted	0.95	0.81–1.13	0.59	3 × 10^−4^	0.91	0.69–1.22	0.54	0.1
MR‐Egger	1.46	0.68–3.16	0.33	0.26	1.05	0.14–8.17	0.96	0.89
Weighted median	0.82	0.66–1.02	0.07		0.96	0.62–11.48	0.84	
MR‐PRESSO								

Abbreviations: CI, confidence intervals; MR, Mendelian randomisation; OR, odds ratio; MR‐PRESSO, MR pleiotropy residual sum and outlier test.

^a^ The estimates correspond to a standard deviation increase in duration of sedentary activity.

^b^ *p*‐value or pleiotropy based on MR‐Egger intercept.

^c^ *p*‐value for heterogeneity based on Q statistic.

**FIGURE 1:**
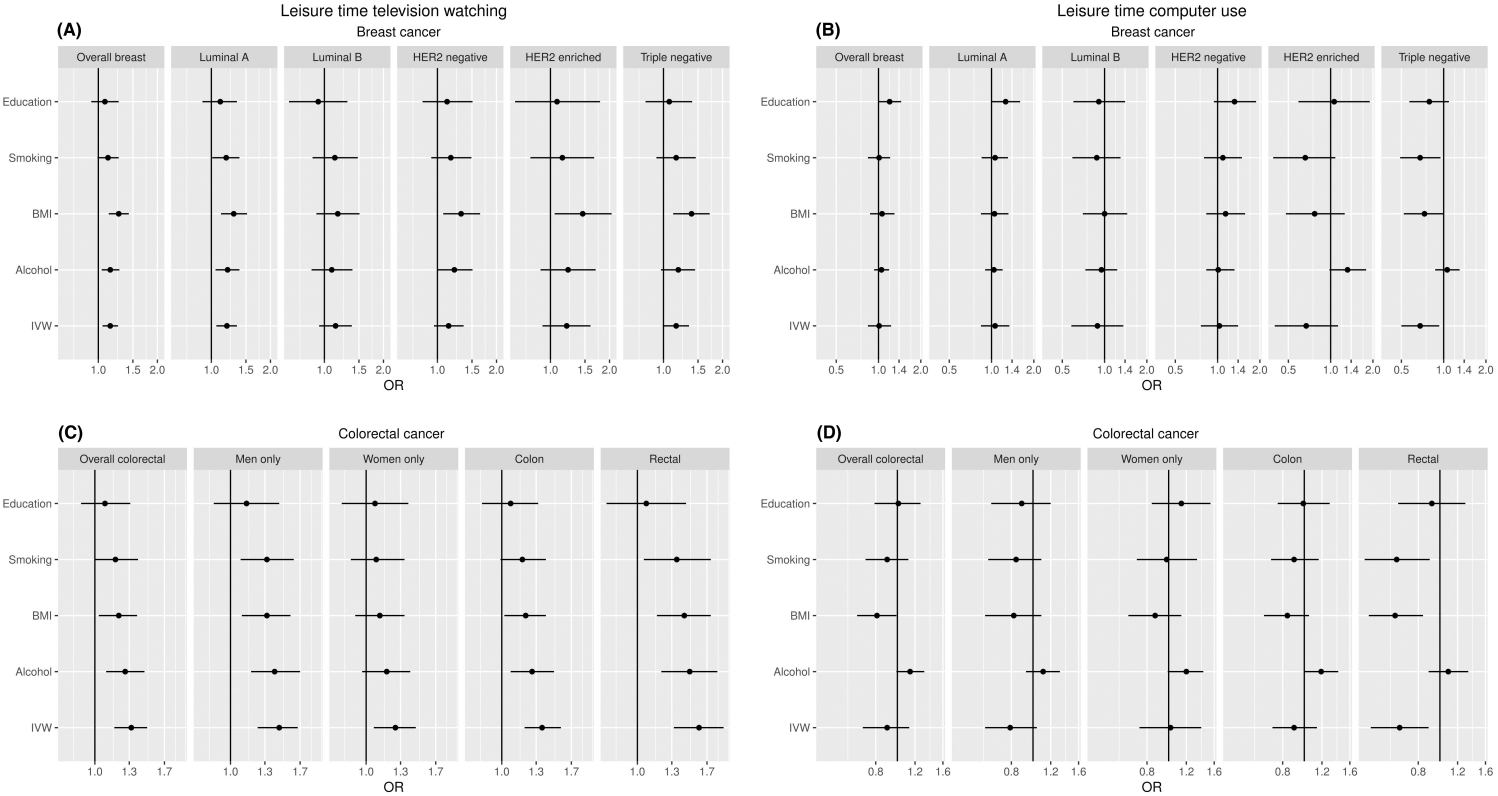
Associations of leisure time television watching and computer use with breast and colorectal cancer after adjusting for the four secondary traits. The black dot corresponds to the 1‐SD odds ratio and the corresponding error bar to the 95% confidence interval. Each error bar refers to the associations of leisure time television watching or computer use with breast or colorectal cancer after adjusting for the specific trait on the left side of the graph. (A) leisure time television watching‐breast cancer associations; (B) leisure time computer use‐breast cancer associations; (C) leisure time television watching‐colorectal cancer associations; (D) leisure time computer use‐colorectal cancer associations. BMI, body mass index; IVW, inverse‐variance weighting.

The multivariable MR analysis adjusting for years of education led to the attenuation of all effect estimates between genetically predicted television watching and the risk of breast (OR per 1 SD: 1.08, 95% CI: 0.92–1.27) and colorectal cancer (OR per 1 SD: 1.08, 95% CI: 0.90–1.31) (Figure 1A, C, Table S22). Additional attenuations were observed for the models adjusting for lifetime smoking. For women, risk estimates for colorectal cancer were attenuated towards the null in all multivariable MR models adjusting for each of the four secondary traits (Figure 1C, Table S34). Finally, genetically predicted television watching was associated with HER2 negative, HER2 positive and triple negative breast cancer after adjusting for BMI in the multivariable MR models with effect sizes ranging from 1.32 to 1.46 per SD (Figure 1A).

Based on the Cochran's *Q* values, there was evidence of heterogeneity of SNP effects for most outcomes except for triple negative breast cancer (Tables 1, 2, 3). Scatter plots (with coloured lines representing the slopes of the different regression analyses) and funnel plots of the association between leisure television watching and the risk of breast, colorectal and prostate cancer risk are presented in Figures S1–S6.

### 3.3 MR estimates for leisure computer use

There was little evidence of any causal effect of longer duration of genetically predicted leisure computer use with overall breast, colorectal and prostate cancer (Tables 1, 2, 3). Inverse effect estimates were found for triple negative breast cancer (OR per 1 SD: 0.68, 95% CI: 0.50–0.93, *p*‐value: 0.02) and rectal cancer (OR per 1 SD: 0.66, 95% CI: 0.49–0.89, *p*‐value: 6 × 10^−3^) (Tables 1 and 2). Despite this, little evidence of heterogeneity was found by breast cancer subtype (*I* ^2^ = 36%, *p*‐heterogeneity = 0.17), colorectal cancer subsite (*I* ^2^ = 45%, *p*‐heterogeneity = 0.15), or by prostate cancer status (overall vs aggressive; *I* ^2^ = 0%, *p*‐heterogeneity = 0.34), or sex (colorectal cancer: *I* ^2^ = 31%, *p*‐heterogeneity = 0.23).

In the multivariable MR analysis for triple negative breast cancer, after adjusting separately for years of education, alcohol and BMI the inverse effect estimates for genetically predicted computer use found in the univariable MR analysis were no longer statistically significant with the new attenuated effect sizes ranging from 0.73 to 1.06 per SD (Figure 1B,D, Table S34). Similarly, the inverse effect estimates for rectal cancer observed in the univariable analysis were attenuated after adjusting for years of education or alcohol consumption (Figure 1D, Table S34).

Based on Cochran's *Q* values, heterogeneity in SNP effects was found for overall breast cancer, luminal A breast cancer, luminal B breast cancer and colorectal cancer. Scatter plots (with coloured lines representing the slopes of the different regression analyses) and funnel plots of the association between leisure computer use and risks of breast, colorectal and prostate cancer are presented in Figures S7–S12.

### 3.4 Evaluation of assumptions and sensitivity analyses

The strength of the genetic instruments according to the F‐statistic was ≥10 for both exposures of interest and ranged between 23 and 164 (Tables S1–S3). In the multivariable MR framework, the conditional F statistics were mainly above 10 (indicating little evidence of weak instrument bias) for both our exposures of interest and the adjusting factors. For models including television watching and years of education, conditional *F* statistics for both variables were below 10. Also, adjusting for BMI or years of education resulted in low *F* statistics (<10) for computer use. Little evidence of directional pleiotropy was observed based on the MR‐Egger's test (MR‐Egger intercept *p* > 0.05) (Tables 1, 2, 3). The effect estimates from MR Egger regression models were generally in the same direction with those from the main analysis but with wider confidence intervals (Tables 1, 2, 3). Similarly, the weighted‐median approach effect estimates were consistent in direction and magnitude to the IVW models (Tables 1, 2, 3). The MR‐PRESSO analysis identified several (10 in total) outlying SNPs (Table S35); however, no major differences were observed when these outlying genetic variants were excluded from the analyses (Tables 1, 2, 3). After examining Phenoscanner, we found that several of the genetic variants were also associated with adiposity or education‐related phenotypes, such as BMI and highest qualification (Table S36).

### 3.5 MR estimates for the bidirectional MR

**FIGURE 2:**
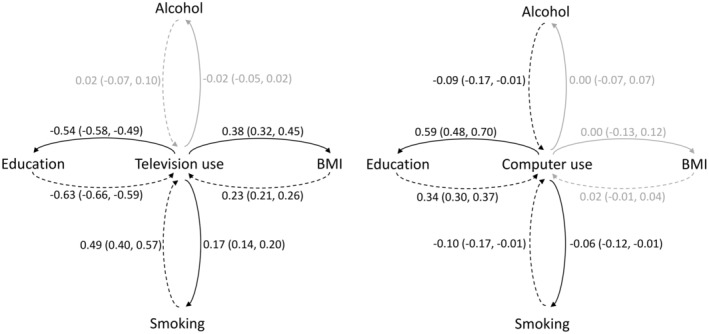
Bidirectional associations of leisure time television watching and computer use with the four secondary traits: ΒΜΙ, years of education, smoking and alcohol. The solid lines correspond to the effects of time television watching and computer use on the four secondary traits while the dashed lines correspond to the effects of the four secondary traits on time television watching and computer use. The black colour corresponds to statistically significant associations and the grey colour to non‐significant. All the results, odds ratios and 95% confidence intervals correspond to a 1‐SD change in the levels of the variables. BMI, body mass index.

In post hoc analyses, inverse bidirectional associations were observed between the genetically predicted duration of leisure television watching and years of education. A one SD increase in genetically predicted duration of leisure television watching reduced years of education by 0.54 SD (95% CI: −0.58 to −0.49). Similarly, a one SD increase in genetically predicted years of education reduced duration of leisure television watching by 0.63 SD (95% CI: −0.66 to −0.59) (Figure 2, Tables S37 and S38). These observations taken together with the inverse effect estimate found for years of education with breast and colorectal cancer (Table S39) point to education having a complex dual confounding and mediating role in the association between television watching with breast and colorectal cancer risk. Contrary to this, positive bidirectional associations were observed for genetically predicted duration of leisure computer use (${\text{beta}}_{\text{computer}\;\mathrm{use}\to \text{education}}$: 0.59; 95% CI: 0.48–0.70 and ${\text{beta}}_{\text{education}\to \text{computer}\;\mathrm{use}}$: 0.34; 95% CI: 0.30–0.37). Additionally, positive bidirectional associations were observed between the genetically predicted duration of leisure television watching with BMI and smoking status while inverse bidirectional associations were observed between the genetically predicted duration of leisure computer use and smoking status. Finally, alcohol consumption was inversely associated with computer use (Figure 2, Tables S37 and S38).

## 4 DISCUSSION

The univariable MR analyses showed that a high level of genetically predicted television watching increased risks of breast and colorectal cancer but after multivariable MR adjustment for years of education, the positive effects were attenuated. Our post hoc analyses further suggested that education has a complex dual confounding and mediating role in the association between television watching with these cancers. The effect estimates for television watching were robust according to most of the univariable sensitivity analyses conducted to assess the influence of pleiotropy. We found little evidence that genetically predicted leisure computer use was associated with breast, colorectal and prostate cancer.

Inconsistent results have been reported in prospective cohort studies that have examined the association between sedentary behaviours and breast cancer risk. A recent meta‐analysis reported a statistically significant 10% higher risk for the highest sedentary behaviour group when compared with the lowest group (relative risk: 1.10, 95% CI: 1.02–1.18).^8^ However, a recent study in UK Biobank found little evidence of any association between hours spent watching television and the risk of breast cancer (OR per 1 h increase: 1.01, 95% CI: 0.99–1.03).^9^ In our analysis, we initially observed positive associations between hours of television watching and the risk of breast cancer. However, these positive effect estimates were attenuated towards the null in our multivariable MR models adjusting for other risk factors, particularly years of education.

Numerous observational studies have investigated the associations between sedentary behaviours and colorectal cancer risk. Results from the most recent meta‐analysis of case–control and cohort studies reported a non‐significant 10% risk increase for colorectal cancer for the highest sedentary behaviour group when compared with the lowest group (RR = 1.10, 95% CI: 0.96–1.26).^8^ Television viewing time has been the most investigated sedentary behaviour trait, and positive associations have been found with colon cancer.^9^, ^41^ A recent UK Biobank analysis reported that higher levels of television watching time were associated with greater colon cancer risk (HR per 1‐hour increase, 1.04, 95% CI: 1.01–1.07; *p*‐value = 0.016), but not rectal cancer.^9^ The same UK Biobank study found no association between leisure computer use and colorectal cancer risk.^9^ Results from our univariable MR analyses were generally consistent with this prior observational evidence, with positive effect estimates found for television watching, and little evidence of an association between computer use and colorectal cancer risk, except of rectal cancer. However, these positive associations attenuated towards the null in multivariable MR models adjusted for years of education and smoking (colorectal; television watching) or alcohol (rectal; computer use).

We found little evidence of any associations between sedentary behaviours and prostate cancer risk, consistent with prior observational evidence.^9^, ^41^ The null effects we found were similar for overall and aggressive prostate cancer risk.

Recently, two MR studies investigated the associations between sedentary behaviours and the risks of breast, colon and prostate cancer.^15^, ^16^ The first included six SNPs associated with the probability of engaging in sedentary behaviours and found that longer genetically predicted sedentary time was associated with higher hormone‐receptor‐negative breast cancer risk (OR per‐SD [~7% time spent sedentary] = 1.77; 95% CI: 1.07–2.92) with an attenuated effect observed for overall breast cancer (OR per‐SD =1.20; 95% CI: 0.93–1.55).^16^ These results are in general agreement with our study in which we observed positive effects estimates for both HER2 enriched and triple negative breast cancers. However, the earlier MR analysis did not include multivariable analyses to adjust for other risk factors. The second MR study used the same instruments as our study and similarly identified the positive effects of television watching with overall breast cancer and similarly observed an attenuation of the estimates after adjusting for years of education.^15^ However, no positive effects were observed for television watching and colon cancer in this study, most likely due to the small number of colon cancer cases included (*n* = 2437).

Current literature suggests that the mechanisms connecting sedentary behaviours with cancer risk overlap at least partially with those underpinning the physical activity relationship and include interrelated pathways such as excess adiposity, metabolic dysfunction and alterations in sex hormone and inflammatory pathways.^8^, ^16^

Strong genetic correlations have been reported between television watching (inverse) and computer use (positive) with years of education ($r_{g}^{\mathrm{TV}}$ = −0.79 and $r_{g}^{\mathrm{PC}}$ = 0.53).^17^ The low conditional F statistics in our multivariable models including the sedentary behaviour traits with years of education provided a further indicator of strong correlations. A recent MR study reported an inverse association between years of education and breast (OR: 0.89, 95% CI: 0.83–0.96; *p*‐value = 0.001) and a positive association for prostate cancer (OR: 1.10, 95% CI: 1.01–1.21; *p*‐value = 0.035).^42^ In agreement with that, we observed inverse effect estimates for years of education in our multivariable models for breast and colorectal cancer. An additional MR study found that higher educational attainment levels were further inversely associated with smoking, BMI and sedentary behaviours, and positively with vigorous physical activity levels and alcohol consumption.^43^ Therefore, education may be a proxy for overall lifestyle, with higher educated individuals practising healthier lifestyle behaviours and actively participating in screening programmes that lower their risk of developing cancer.^42^ Additionally, traits like sedentary behaviours, education, smoking, alcohol consumption and obesity are correlated and it is therefore difficult to disentangle their complex interrelationships. As an example, in our post hoc analyses we found evidence of education having a dual confounding and mediating role in the association between television watching with breast and colorectal cancers. Previous studies and ours have shown that education plays an important role in cancer incidence of these three cancer types. However, the role of other lifestyle factors in these relationships is unclear, and further studies are needed to disentangle these complex interrelationships.

The main strength of the current study is the use of large‐scale summary genetic data from consortia and the UK Biobank that allowed us to investigate the role of leisure sedentary behaviours on risk of developing breast, colorectal and prostate cancer. A limitation of our study is that leisure sedentary behaviours were derived from self‐reported questionnaires that are prone to measurement error.^44^, ^45^ An alternative approach is to use genetic instruments derived from objectively measured levels of physical activity using accelerometer data from the UK Biobank.^46^, ^47^ However, a current limitation is that the number of genetic instruments is comparatively small as the GWAS on accelerometer data was analysed in a subset of 91,000 participants. Analysing two highly correlated phenotypes together, like sedentary behaviours and years of education may have introduced collinearity which leads to greater imprecision and possible bias. Furthermore, caution is needed regarding the results from the analyses for leisure computer use as the genetic instruments explained a small proportion of the phenotypic variance resulting in a lower powered analysis. Also, our analyses focused solely on leisure sedentary exposures so non‐leisure sedentary behaviours were unaccounted for. The genetic correlation between television watching and objectively measured sedentary behaviour in UK Biobank was weak ($r_{g}^{\mathrm{TV}}$ = 0.14) while the correlation for computer use was higher ($r_{g}^{\mathrm{PC}}$ = 0.46).^17^ This can be at least partially explained from the fact that accelerometers measure total but not domain‐specific sedentary time (e.g. television watching) that has been studied in previous observational studies.^3^, ^48^ Therefore, our results cannot be generalised to overall sedentary behaviour. The genetic instruments were derived from UK Biobank which is not without limitations. For example, the average age of the participants in UK Biobank was 57 years, an age group that spends most time watching television.^17^ Consequently, the results cannot be generalised to younger ages as the habits of younger people are not included in the analysis and similarly the phenotype of leisure time computer use perhaps is not optimal to capture sedentary behaviours of this population. In addition, large biobanks like UK Biobank often suffer from participation bias since the participants are not representative of their target population and it has been shown to distort genome‐wide findings and downstream analyses particularly for socio‐behavioural traits.^49^ Furthermore, we cannot exclude the possibility of confounding due to population stratification in our dataset. The genetic instruments were derived from a sex combined population while some of the outcomes were sex specific which could introduce some bias in our results if the effects of the genetic instruments differ between two sexes. Additionally, we cannot exclude potential dynastic and assortative mating effects as it has been reported that the estimates of education could be attributed at least partially to parental effects to the child's characteristics.^43^ Moreover, parents do not mate randomly but assort on characteristics such as educational level.^43^ These cross‐generational effects could also have biased our results.^50^, ^51^ Finally, the results cannot be generalised to diverse populations due to the lack of ancestral diversity in UK Biobank.

### 4.1 Conclusions

In conclusion, after adjusting for lifestyle factors, especially years of education, leisure time television watching no longer increased the risks of breast and colorectal cancer and demonstrated how highly intercorrelated these exposures are. These multivariable results should be interpreted cautiously as we detected evidence of education having a dual confounding and mediating role in the associations between television watching with risks of breast and colorectal cancer. Future analyses utilising objective measures of exposure (e.g. accelerometers) and novel analytic frameworks (e.g. target trial emulation) are required to provide new insights into the possible role of sedentary behaviour in cancer development.

## AUTHOR CONTRIBUTIONS

**Nikos Papadimitriou:** Formal analysis (lead); writing – original draft (lead); writing – review and editing (equal). **Nabila Kazmi:** Data curation (equal); formal analysis (supporting); writing – review and editing (equal). **Niki Dimou:** Writing – review and editing (equal). **Konstantinos K Tsilidis:** Methodology (equal); writing – review and editing (equal). **Richard Martin:** Methodology (equal); writing – review and editing (equal). **Sarah Lewis:** Methodology (equal); writing – review and editing (equal). **Brigid Lynch:** Methodology (equal); writing – review and editing (equal). **Michael Hoffmeister:** Writing – review and editing (equal). **Sun‐Seog Kweon:** Writing – review and editing (equal). **Li Li:** Writing – review and editing (equal). **Roger Milne:** Writing – review and editing (equal). **Lori C. Sakoda:** Writing – review and editing (equal). **Robert Schoen:** Writing – review and editing (equal). **Amanda I. Phipps:** Writing – review and editing (equal). **Jane Figueiredo:** Writing – review and editing (equal). **Ulrike Peters:** Data curation (equal); writing – review and editing (equal). **Suzanne Dixon‐Suen:** Writing – review and editing (equal). **Marc Gunter:** Writing – review and editing (equal). **Neil Murphy:** Conceptualization (lead); methodology (equal); writing – review and editing (equal).

## CONFLICT OF INTEREST STATEMENT

The authors declare that they have no conflicts of interest.

## FUNDING INFORMATION

Funding for grant WCRF_2020_019 was obtained from Wereld Kanker Onderzoek Fonds (WKOF) as part of the World Cancer Research Fund International grant programme. RMM is supported by a Cancer Research UK Programme Grant, the Integrative Cancer Epidemiology Programme (C18281/A29019). RMM is a member of the MRC IEU which is supported by the Medical Research Council and the University of Bristol (MC_UU_12013/1‐9). RMM is supported by the National Institute for Health Research (NIHR) Bristol Biomedical Research Centre which is funded by the National Institute for Health Research and is a partnership between University Hospitals Bristol NHS Trust, Weston NHS Foundation Trust and the University of Bristol. The views expressed in this publication are those of the author(s) and not necessarily those of the NIHR or the UK Department of Health and Social Care. BML is supported by the Victorian Cancer Agency (MCRF‐18005).

## CONSORTIA FUNDING

GECCO: Genetics and Epidemiology of Colorectal Cancer Consortium: National Cancer Institute, National Institutes of Health, U.S. Department of Health and Human Services (U01 CA164930, U01 CA137088, R01 CA059045, R21 CA191312, R01201407). Genotyping/Sequencing services were provided by the Center for Inherited Disease Research (CIDR) contract number HHSN268201700006I and HHSN268201200008I. This research was funded in part through the NIH/NCI Cancer Center Support Grant P30 CA015704. Scientific Computing Infrastructure at Fred Hutch funded by ORIP grant S10OD028685. ASTERISK: a Hospital Clinical Research Programme (PHRC‐BRD09/C) from the University Hospital Center of Nantes (CHU de Nantes) and supported by the Regional Council of Pays de la Loire, the Groupement des Entreprises Françaises dans la Lutte contre le Cancer (GEFLUC), the Association Anne de Bretagne Génétique and the Ligue Régionale Contre le Cancer (LRCC). The ATBC Study is supported by the Intramural Research Programme of the U.S. National Cancer Institute, National Institutes of Health. CLUE II funding was from the National Cancer Institute (U01 CA86308, Early Detection Research Network; P30 CA006973), National Institute on Aging (U01 AG18033) and the American Institute for Cancer Research. The content of this publication does not necessarily reflect the views or policies of the Department of Health and Human Services, nor does mention of trade names, commercial products, or organisations imply endorsement by the US government. Maryland Cancer Registry (MCR) Cancer data were provided by the Maryland Cancer Registry, Center for Cancer Prevention and Control, Maryland Department of Health, with funding from the State of Maryland and the Maryland Cigarette Restitution Fund. The collection and availability of cancer registry data are also supported by the Cooperative Agreement NU58DP006333, funded by the Centers for Disease Control and Prevention. Its contents are solely the responsibility of the authors and do not necessarily represent the official views of the Centers for Disease Control and Prevention or the Department of Health and Human Services. ColoCare: This work was supported by the National Institutes of Health (grant numbers R01 CA189184 (Li/Ulrich), U01 CA206110 (Ulrich/Li/Siegel/Figueireido/Colditz, 2P30CA015704–40 (Gilliland), R01 CA207371 (Ulrich/Li)), the Matthias Lackas‐Foundation, the German Consortium for Translational Cancer Research and the EU TRANSCAN initiative. The Colon Cancer Family Registry (CCFR, www.coloncfr.org) is supported in part by funding from the National Cancer Institute (NCI), National Institutes of Health (NIH) (award U01 CA167551). Support for case ascertainment was provided in part from the Surveillance, Epidemiology and End Results (SEER) Programme and the following U.S. state cancer registries: AZ, CO, MN, NC and NH; and by the Victoria Cancer Registry (Australia) and Ontario Cancer Registry (Canada). The CCFR Set‐1 (Illumina 1M/1M‐Duo) was supported by NIH awards U01 CA122839 and R01 CA143247 (to GC). The CCFR Set‐3 (Affymetrix Axiom CORECT Set array) was supported by NIH award U19 CA148107 and R01 CA81488 (to SBG). The CCFR Set‐4 (Illumina OncoArray 600K SNP array) was supported by NIH award U19 CA148107 (to SBG) and by the Center for Inherited Disease Research (CIDR), which is funded by the NIH to the Johns Hopkins University, contract number HHSN268201200008I. Additional funding for the The content of this manuscript does not necessarily reflect the views or policies of the NCI, NIH or any of the collaborating centres in the Colon Cancer Family Registry (CCFR), nor does mention of trade names, commercial products or organisations imply endorsement by the US Government, any cancer registry, or the CCFR. COLON: The COLON study is sponsored by Wereld Kanker Onderzoek Fonds, including funds from grant 2014/1179 as part of the World Cancer Research Fund International Regular Grant Programme, by Alpe d'Huzes and the Dutch Cancer Society (UM 2012–5653, UW 2013‐5927, UW2015‐7946), and by TRANSCAN (JTC2012‐MetaboCCC, JTC2013‐FOCUS). The Nqplus study is sponsored by a ZonMW investment grant (98‐10030); by PREVIEW, the project PREVention of diabetes through lifestyle intervention and population studies in Europe and around the World (PREVIEW) project which received funding from the European Union Seventh Framework Programme (FP7/2007–2013) under grant no. 312057; by funds from TI Food and Nutrition (cardiovascular health theme), a public–private partnership on precompetitive research in food and nutrition; and by FOODBALL, the Food Biomarker Alliance, a project from JPI Healthy Diet for a Healthy Life. Colorectal Cancer Transdisciplinary (CORECT) Study: The CORECT Study was supported by the National Cancer Institute, National Institutes of Health (NCI/NIH), U.S. Department of Health and Human Services (grant numbers U19 CA148107, R01 CA81488, P30 CA014089, R01 CA197350; P01 CA196569; R01 CA201407) and National Institutes of Environmental Health Sciences, National Institutes of Health (grant number T32 ES013678). CORSA: ‘Österreichische Nationalbank Jubiläumsfondsprojekt’ (12511) and Austrian Research Funding Agency (FFG) grant 829,675. CPS‐II: The American Cancer Society funds the creation, maintenance and updating of the Cancer Prevention Study‐II (CPS‐II) cohort. This study was conducted with Institutional Review Board approval. CRCGEN: Colorectal Cancer Genetics & Genomics, Spanish study was supported by Instituto de Salud Carlos III, co‐funded by FEDER funds—a way to build Europe—(grants PI14‐613 and PI09‐1286), Agency for Management of University and Research Grants (AGAUR) of the Catalan Government (grant 2017SGR723), and Junta de Castilla y León (grant LE22A10–2). Sample collection of this work was supported by the Xarxa de Bancs de Tumors de Catalunya sponsored by Pla Director d'Oncología de Catalunya (XBTC), Plataforma Biobancos PT13/0010/0013 and ICOBIOBANC, sponsored by the Catalan Institute of Oncology. Czech Republic CCS: This work was supported by the Czech Science Foundation (20‐03997S) and by the Grant Agency of the Ministry of Health of the Czech Republic (grants NV18/03/00199 and NU21‐07‐00247). DACHS: This work was supported by the German Research Council (BR 1704/6‐1, BR 1704/6‐3, BR 1704/6‐4, CH 117/1‐1, HO 5117/2‐1, HE 5998/2‐1, KL 2354/3‐1, RO 2270/8‐1 and BR 1704/17‐1), the Interdisciplinary Research Programme of the National Center for Tumor Diseases (NCT), Germany, and the German Federal Ministry of Education and Research (01KH0404, 01ER0814, 01ER0815, 01ER1505A and 01ER1505B). DALS: National Institutes of Health (R01 CA48998 to M. L. Slattery). EDRN: This work is funded and supported by the NCI, EDRN Grant (U01CA152753). EPIC: The coordination of EPIC is financially supported by the European Commission (DGSANCO) and the International Agency for Research on Cancer. The national cohorts are supported by Danish Cancer Society (Denmark); Ligue Contre le Cancer, Institut Gustave Roussy, Mutuelle Générale de l'Education Nationale, Institut National de la Santé et de la Recherche Médicale (INSERM) (France); German Cancer Aid, German Cancer Research Center (DKFZ), Federal Ministry of Education and Research (BMBF), Deutsche Krebshilfe, Deutsches Krebsforschungszentrum and Federal Ministry of Education and Research (Germany); the Hellenic Health Foundation (Greece); Associazione Italiana per la Ricerca sul Cancro‐AIRCItaly and National Research Council (Italy); Dutch Ministry of Public Health, Welfare and Sports (VWS), Netherlands Cancer Registry (NKR), LK Research Funds, Dutch Prevention Funds, Dutch ZON (Zorg Onderzoek Nederland), World Cancer Research Fund (WCRF), Statistics Netherlands (The Netherlands); ERC‐2009‐AdG 232997 and Nordforsk, Nordic Centre of Excellence programme on Food, Nutrition and Health (Norway); Health Research Fund (FIS), PI13/00061 to Granada, PI13/01162 to EPIC‐Murcia, Regional Governments of Andalucía, Asturias, Basque Country, Murcia and Navarra, ISCIII RETIC (RD06/0020) (Spain); Swedish Cancer Society, Swedish Research Council and County Councils of Skåne and Västerbotten (Sweden); Cancer Research UK (14136 to EPIC‐Norfolk; C570/A16491 and C8221/A19170 to EPIC‐Oxford), Medical Research Council (1000143 to EPIC‐Norfolk, MR/M012190/1 to EPICOxford) (United Kingdom). The EPIC‐Norfolk study (https://doi.org/10.22025/2019.10.105.00004) has received funding from the Medical Research Council (MR/N003284/1 and MC‐UU_12015/1) and Cancer Research UK (C864/A14136). The genetics work in the EPIC‐Norfolk study was funded by the Medical Research Council (MC_PC_13048). Metabolite measurements in the EPIC‐Norfolk study were supported by the MRC Cambridge Initiative in Metabolic Science (MR/L00002/1) and the Innovative Medicines Initiative Joint Undertaking under EMIF grant agreement no. 115372. EPICOLON: This work was supported by grants from Fondo de Investigación Sanitaria/FEDER (PI08/0024, PI08/1276, PS09/02368, PI11/00219, PI11/00681, PI14/00173, PI14/00230, PI17/00509, 17/00878, PI20/00113, PI20/00226, Acción Transversal de Cáncer), Xunta de Galicia (PGIDIT07PXIB9101209PR), Ministerio de Economia y Competitividad (SAF07‐64873, SAF 2010‐19273, SAF2014‐54453R), Fundación Científica de la Asociación Española contra el Cáncer (GCB13131592CAST), Beca Grupo de Trabajo ‘Oncología’ AEG (Asociación Española de Gastroenterología), Fundación Privada Olga Torres, FP7 CHIBCHA Consortium, Agència de Gestió d'Ajuts Universitaris i de Recerca (AGAUR, Generalitat de Catalunya, 2014SGR135, 2014SGR255, 2017SGR21, 2017SGR653), Catalan Tumour Bank Network (Pla Director d'Oncologia, Generalitat de Catalunya), PERIS (SLT002/16/00398, Generalitat de Catalunya), CERCA Programme (Generalitat de Catalunya) and COST Actions BM1206 and CA17118. CIBERehd is funded by the Instituto de Salud Carlos III. ESTHER/VERDI. This work was supported by grants from the Baden‐Württemberg Ministry of Science, Research and Arts and the German Cancer Aid. Harvard cohorts (HPFS, NHS, PHS): HPFS is supported by the National Institutes of Health (P01 CA055075, UM1 CA167552, U01 CA167552, R01 CA137178, R01 CA151993, R35 CA197735, K07 CA190673, and P50 CA127003), NHS by the National Institutes of Health (R01 CA137178, P01 CA087969, UM1 CA186107, R01 CA151993, R35 CA197735, K07CA190673, and P50 CA127003) and PHS by the National Institutes of Health (R01 CA042182). Hawaii Adenoma Study: NCI grants R01 CA72520. HCES‐CRC: the Hwasun Cancer Epidemiology Study–Colon and Rectum Cancer (HCES‐CRC; grants from Chonnam National University Hwasun Hospital, HCRI21019). Kentucky: This work was supported by the following grant support: Clinical Investigator Award from Damon Runyon Cancer Research Foundation (CI‐8); NCI R01CA136726. LCCS: The Leeds Colorectal Cancer Study was funded by the Food Standards Agency and Cancer Research UK Programme Award (C588/A19167). Melbourne Collaborative Cohort Study (MCCS) cohort recruitment was funded by VicHealth and Cancer Council Victoria. The MCCS was further augmented by Australian National Health and Medical Research Council grants 209057, 396414 and 1074383 and by infrastructure provided by Cancer Council Victoria. Cases and their vital status were ascertained through the Victorian Cancer Registry and the Australian Institute of Health and Welfare, including the National Death Index and the Australian Cancer Database. Multiethnic Cohort (MEC) Study: National Institutes of Health (R37 CA54281, P01 CA033619, R01 CA063464 and U01 CA164973). MECC: This work was supported by the National Institutes of Health, U.S. Department of Health and Human Services (R01 CA81488 to SBG and GR). MSKCC: The work at Sloan Kettering in New York was supported by the Robert and Kate Niehaus Center for Inherited Cancer Genomics and the Romeo Milio Foundation. Moffitt: This work was supported by funding from the National Institutes of Health (grant numbers R01 CA189184, P30 CA076292), Florida Department of Health Bankhead‐Coley Grant 09BN‐13 and the University of South Florida Oehler Foundation. Moffitt contributions were supported in part by the Total Cancer Care Initiative, Collaborative Data Services Core and Tissue Core at the H. Lee Moffitt Cancer Center & Research Institute, a National Cancer Institute‐designated Comprehensive Cancer Center (grant number P30 CA076292). NCCCS I & II: We acknowledge funding support for this project from the National Institutes of Health, R01 CA66635 and P30 DK034987. NFCCR: This work was supported by an Interdisciplinary Health Research Team award from the Canadian Institutes of Health Research (CRT 43821); the National Institutes of Health, U.S. Department of Health and Human Serivces (U01 CA74783); and National Cancer Institute of Canada grants (18223 and 18226). The authors wish to acknowledge the contribution of Alexandre Belisle and the genotyping team of the McGill University and Génome Québec Innovation Centre, Montréal, Canada, for genotyping the Sequenom panel in the NFCCR samples. Funding was provided to Michael O. Woods by the Canadian Cancer Society Research Institute. NSHDS: Swedish Research Council; Swedish Cancer Society; Cutting‐Edge Research Grant and other grants from Region Västerbotten; Knut and Alice Wallenberg Foundation; Lion's Cancer Research Foundation at Umeå University; the Cancer Research Foundation in Northern Sweden; and the Faculty of Medicine, Umeå University, Umeå, Sweden. OSUMC: OCCPI funding was provided by Pelotonia and HNPCC funding was provided by the NCI (CA16058 and CA67941). PLCO: Intramural Research Programme of the Division of Cancer Epidemiology and Genetics and supported by contracts from the Division of Cancer Prevention, National Cancer Institute, NIH, DHHS. Funding was provided by National Institutes of Health (NIH), Genes, Environment and Health Initiative (GEI) Z01 CP 010200, NIH U01 HG004446 and NIH GEI U01 HG 004438. SEARCH: The University of Cambridge has received salary support in respect of PDPP from the NHS in the East of England through the Clinical Academic Reserve. Cancer Research UK (C490/A16561); the UK National Institute for Health Research Biomedical Research Centres at the University of Cambridge. SELECT: Research reported in this publication was supported in part by the National Cancer Institute of the National Institutes of Health under Award Numbers U10 CA37429 (CD Blanke), and UM1 CA182883 (CM Tangen/IM Thompson). The content is solely the responsibility of the authors and does not necessarily represent the official views of the National Institutes of Health. SMS and REACH: This work was supported by the National Cancer Institute (grant P01 CA074184 to J.D.P. and P.A.N., grants R01 CA097325, R03 CA153323 and K05 CA152715 to P.A.N., and the National Center for Advancing Translational Sciences at the National Institutes of Health (grant KL2 TR000421 to A.N.B.‐H.). The Swedish Low‐risk Colorectal Cancer Study: The study was supported by grants from the Swedish research council; K2015‐55X‐22674‐01‐4, K2008‐55X‐20157‐03‐3, K2006‐72X‐20157‐01‐2 and the Stockholm County Council (ALF project). Swedish Mammography Cohort and Cohort of Swedish Men: This work is supported by the Swedish Research Council /Infrastructure grant, the Swedish Cancer Foundation, and the Karolinska Institute's Distinguished Professor Award to Alicja Wolk. UK Biobank: This research has been conducted using the UK Biobank Resource under Application Number 8614. VITAL: National Institutes of Health (K05 CA154337). WHI: The WHI programme is funded by the National Heart, Lung and Blood Institute, National Institutes of Health, U.S. Department of Health and Human Services through contracts HHSN268201100046C, HHSN268201100001C, HHSN268201100002C, HHSN268201100003C, HHSN268201100004C and HHSN271201100004C.

## ETHICS APPROVAL

All analyses were conducted using summary‐level data generated by previous studies that have described their relevant ethical approvals.

## DISCLAIMER

Where authors are identified as personnel of the International Agency for Research on Cancer/World Health Organization, the authors alone are responsible for the views expressed in this article and they do not necessarily represent the decisions, policy or views of the International Agency for Research on Cancer/World Health Organization.

## Supporting information


Appendix S1.

## Data Availability

The datasets supporting the conclusions of this article are included within the supplemental tables.
