# Supplementary material for: Leisure time television watching, computer use and risks of breast, colorectal and prostate cancer: A Mendelian randomisation analysis
Source: Cancer Med. 2023 Dec 28;13(1):e6732. doi: 10.1002/cam4.6732 (PMC10807615; doi:10.1002/cam4.6732)
Supplement: Supplementary file 1 — Appendix S1. [file CAM4-13-e6732-s001.docx]

**Supplemental material**

**Table S1.** Summary information on leisure television watching SNPs used as genetic instruments in the Mendelian randomisation analyses

**Table S2.** Summary information on leisure computer use SNPs used as genetic instruments used in the Mendelian randomisation analyses

**Table S3.** Summary information on breast cancer for the leisure television watching SNPs used in the Mendelian randomisation analyses

**Table S4.** Summary information on breast cancer for the leisure computer use SNPs used in the Mendelian randomisation analyses

**Table S5.** Summary information on colorectal cancer for the leisure television watching SNPs used in the Mendelian randomisation analyses

**Table S6.** Summary information on colorectal cancer for the leisure computer use SNPs used in the Mendelian randomisation analyses

**Table S7.** Summary information on prostate cancer for the leisure television watching SNPs used in the Mendelian randomisation analyses

**Table S8.** Summary information on prostate cancer for the leisure computer use SNPs used in the Mendelian randomisation analyses

**Table S9.** Sample size and power calculations in Mendelian randomization study of sedentary behaviour and cancer risk

**Table S10.** Beta estimates for the SNPs included in the multivariable MR of alcohol consumption, leisure computer use, and breast cancer risk

**Table S11.** Beta estimates for the SNPs included in the multivariable MR of alcohol consumption, leisure computer use, and colorectal cancer risk

**Table S12.** Beta estimates for the SNPs included in the multivariable MR of alcohol consumption, television use, and breast cancer risk

**Table S13.** Beta estimates for the SNPs included in the multivariable MR of alcohol consumption, television use, and colorectal cancer risk

**Table S14.** Beta estimates for the SNPs included in the multivariable MR of BMI, leisure computer use, and breast cancer risk

**Table S15.** Beta estimates for the SNPs included in the multivariable MR of BMI, leisure computer use, and colorectal cancer risk

**Table S16.** Beta estimates for the SNPs included in the multivariable MR of BMI, leisure television watching, and breast cancer risk

**Table S17.** Beta estimates for the SNPs included in the multivariable MR of BMI, leisure television watching, and colorectal cancer risk **Table S18.** Beta estimates for the SNPs included in the multivariable MR of years of education, leisure computer use, and breast cancer risk

**Table S19.** Beta estimates for the SNPs included in the multivariable MR of years of education, leisure computer use, and colorectal cancer risk

**Table S20.** Beta estimates for the SNPs included in the multivariable MR of years of education, leisure television watching, and breast cancer risk

**Table S21.** Beta estimates for the SNPs included in the multivariable MR of years of education, leisure television watching, and colorectal cancer risk

**Table S22.** Beta estimates for the SNPs included in the multivariable MR of smoking, leisure computer use, and breast cancer risk

**Table S23.** Beta estimates for the SNPs included in the multivariable MR of smoking, leisure computer use, and colorectal cancer risk

**Table S24.** Beta estimates for the SNPs included in the multivariable MR of smoking, leisure television use, and breast cancer risk

**Table S25.** Beta estimates for the SNPs included in the multivariable MR of smoking, leisure television use, and colorectal cancer risk

**Table S26.** Betas of the bidirectional MR between alcohol and leisure computer use

**Table S27.** Betas of the bidirectional MR between alcohol and leisure television use

**Table S28.** Betas of the bidirectional MR between BMI and leisure computer use

**Table S29.** Betas of the bidirectional MR between BMI and leisure television use

**Table S30.** Betas of the bidirectional MR between years of education and leisure computer use

**Table S31.** Betas of the bidirectional MR between years of education and leisure television use

**Table S32.** Betas of the bidirectional MR between smoking and leisure computer use

**Table S33.** Betas of the bidirectional MR between smoking and leisure television use

**Table S34.** Multivariable Mendelian randomization estimates between leisure television watching, leisure computer use and cancer risk

**Table S35.** Outlying SNPs identified by MR-PRESSO

**Table S36.** Evidence of association (p<5*10-8) with secondary traits of the SNPs (or their proxies) used as genetic instruments for Mendelian randomization analyses of sedentary behaviours and risk of breast and colorectal cancer

**Table S37.** Mendelian Randomization estimates for the effects of sedentary behaviour on the secondary traits

**Table S38.** Mendelian Randomization estimates for the effects of the secondary traits on sedentary behaviour

**Table S39.** Multivariable Mendelian randomization estimates for years of education and cancer risk

**Figure S1.** Scatter plots showing the correlation of genetic associations of leisure television watching with genetic associations with breast cancer. Coloured lines represent the slopes of the different regression analyses

**Figure S2.** Scatter plots showing the correlation of genetic associations of leisure television watching with genetic associations with colorectal cancer. Coloured lines represent the slopes of the different regression analyses

**Figure S3.** Scatter plots showing the correlation of genetic associations of leisure television watching with genetic associations with prostate cancer. Coloured lines represent the slopes of the different regression analyses

**Figure S4.** Funnel plots of risk estimates of leisure television watching and breast cancer against instrumental strength

**Figure S5.** Funnel plots of risk estimates of leisure television watching and colorectal cancer against instrumental strength

**Figure S6.** Funnel plots of risk estimates of leisure television watching and prostate cancer against instrumental strength

**Figure S7.** Scatter plots showing the correlation of genetic associations of leisure computer use with genetic associations with breast cancer. Coloured lines represent the slopes of the different regression analyses

**Figure S8.** Scatter plots showing the correlation of genetic associations of leisure computer use with genetic associations with colorectal cancer. Coloured lines represent the slopes of the different regression analyses

**Figure S9.** Scatter plots showing the correlation of genetic associations of leisure computer use with genetic associations with prostate cancer. Coloured lines represent the slopes of the different regression analyses

**Figure S10.** Funnel plots of risk estimates of leisure computer use and breast cancer against instrumental strength

**Figure S11.** Funnel plots of risk estimates of leisure computer use and colorectal cancer against instrumental strength

**Figure S12.** Funnel plots of risk estimates of leisure computer use and prostate cancer against instrumental strength

| **Table S1: Summary information on leisure television watching SNPs used as genetic instruments in the Mendelian randomisation analyses** | | | | | | | | | | |
| --- | --- | --- | --- | --- | --- | --- | --- | --- | --- | --- |
| SNP | CHR | Position^a^ | EA | OA | EAF | beta | se | Pvalue | R^2^ | F-statistic |
| rs11810109 | 1 | 44186812 | A | T | 0.701 | 0.016 | 0.002 | 5.40E-12 | 1.31E-04 | 54.8 |
| rs112632642 | 1 | 51115634 | A | G | 0.065 | 0.024 | 0.004 | 3.60E-08 | 6.78E-05 | 28.3 |
| rs6670915 | 1 | 61109968 | C | T | 0.533 | 0.014 | 0.002 | 2.40E-10 | 9.41E-05 | 39.3 |
| rs12725114 | 1 | 62432641 | G | A | 0.802 | 0.015 | 0.003 | 1.60E-08 | 7.79E-05 | 32.5 |
| rs984409 | 1 | 67020440 | A | G | 0.637 | 0.015 | 0.002 | 4.10E-11 | 1.06E-04 | 44.2 |
| rs10890123 | 1 | 74802123 | C | T | 0.770 | 0.014 | 0.003 | 3.30E-08 | 7.86E-05 | 32.8 |
| rs71658797 | 1 | 77967507 | A | T | 0.123 | 0.020 | 0.003 | 6.30E-10 | 9.05E-05 | 37.8 |
| rs114328297 | 1 | 91190854 | T | G | 0.778 | 0.014 | 0.003 | 4.40E-08 | 8.17E-05 | 34.1 |
| rs631130 | 1 | 96043010 | A | T | 0.631 | 0.014 | 0.002 | 5.00E-09 | 9.42E-05 | 39.4 |
| rs148648822 | 1 | 97815048 | T | G | 0.157 | 0.018 | 0.003 | 4.20E-09 | 8.29E-05 | 34.6 |
| rs17379561 | 1 | 98340139 | T | A | 0.144 | 0.025 | 0.003 | 1.10E-16 | 1.65E-04 | 68.9 |
| rs55909997 | 1 | 107581039 | A | G | 0.350 | 0.014 | 0.002 | 2.40E-09 | 8.31E-05 | 34.7 |
| rs1278847 | 1 | 110037483 | C | A | 0.694 | 0.016 | 0.002 | 1.20E-11 | 1.14E-04 | 47.4 |
| rs141425667 | 1 | 114930465 | A | G | 0.065 | 0.024 | 0.004 | 4.00E-08 | 7.94E-05 | 33.1 |
| rs4845364 | 1 | 154141908 | G | A | 0.505 | 0.015 | 0.002 | 1.30E-12 | 1.29E-04 | 53.9 |
| rs6673341 | 1 | 184655649 | G | T | 0.535 | 0.015 | 0.002 | 2.20E-11 | 1.13E-04 | 47.3 |
| rs72725224 | 1 | 188115858 | C | A | 0.020 | 0.046 | 0.008 | 3.70E-09 | 7.37E-05 | 30.8 |
| rs10737620 | 1 | 193057971 | T | A | 0.274 | 0.014 | 0.002 | 2.60E-09 | 9.94E-05 | 41.5 |
| rs6721975 | 2 | 5832667 | C | T | 0.768 | 0.017 | 0.003 | 1.70E-10 | 1.07E-04 | 44.7 |
| rs11896615 | 2 | 17407744 | G | A | 0.852 | 0.017 | 0.003 | 3.10E-08 | 8.10E-05 | 33.8 |
| rs72781699 | 2 | 24277709 | A | G | 0.203 | 0.019 | 0.003 | 3.00E-12 | 1.42E-04 | 59.5 |
| rs12105701 | 2 | 41737200 | T | C | 0.604 | 0.013 | 0.002 | 5.40E-09 | 7.30E-05 | 30.5 |
| rs7564130 | 2 | 50606642 | C | T | 0.359 | 0.015 | 0.002 | 2.80E-11 | 1.12E-04 | 46.6 |
| rs11125515 | 2 | 53886799 | A | C | 0.271 | 0.014 | 0.002 | 2.20E-08 | 8.16E-05 | 34.1 |
| rs12475168 | 2 | 58992704 | T | G | 0.131 | 0.018 | 0.003 | 1.70E-08 | 6.98E-05 | 29.1 |
| rs183566425 | 2 | 59325356 | A | T | 0.982 | 0.046 | 0.008 | 1.90E-08 | 9.09E-05 | 38.0 |
| rs10189857 | 2 | 60713235 | G | A | 0.432 | 0.020 | 0.002 | 6.20E-21 | 2.28E-04 | 95.3 |
| rs62641636 | 2 | 68425427 | A | G | 0.692 | 0.014 | 0.002 | 7.00E-10 | 1.02E-04 | 42.6 |
| rs59210467 | 2 | 97097291 | T | C | 0.307 | 0.013 | 0.002 | 1.00E-08 | 7.86E-05 | 32.8 |
| rs11689199 | 2 | 100820421 | A | G | 0.598 | 0.018 | 0.002 | 5.50E-17 | 1.81E-04 | 75.6 |
| rs2916251 | 2 | 104447052 | T | G | 0.510 | 0.017 | 0.002 | 1.50E-13 | 1.35E-04 | 56.5 |
| rs1451533 | 2 | 105466005 | A | G | 0.275 | 0.016 | 0.002 | 1.10E-10 | 1.04E-04 | 43.3 |
| rs2971640 | 2 | 107624618 | A | G | 0.495 | 0.013 | 0.002 | 4.20E-09 | 9.05E-05 | 37.8 |
| rs12476388 | 2 | 116330983 | C | T | 0.709 | 0.013 | 0.002 | 2.10E-08 | 9.10E-05 | 38.0 |
| rs374722 | 2 | 147839830 | G | A | 0.150 | 0.024 | 0.003 | 5.50E-16 | 1.79E-04 | 74.7 |
| rs3754970 | 2 | 162091836 | C | T | 0.504 | 0.015 | 0.002 | 4.80E-12 | 1.46E-04 | 61.1 |
| rs1913808 | 2 | 162804678 | G | A | 0.713 | 0.015 | 0.002 | 3.90E-10 | 8.72E-05 | 36.4 |
| rs2060199 | 2 | 166196681 | T | A | 0.446 | 0.012 | 0.002 | 2.40E-08 | 7.46E-05 | 31.2 |
| rs263771 | 2 | 185921692 | A | C | 0.233 | 0.017 | 0.003 | 3.10E-11 | 1.24E-04 | 51.7 |
| rs4577309 | 2 | 191288833 | A | G | 0.469 | 0.016 | 0.002 | 1.60E-13 | 1.24E-04 | 51.7 |
| rs34224261 | 2 | 193855452 | C | G | 0.531 | 0.018 | 0.002 | 4.60E-16 | 1.60E-04 | 66.8 |
| rs4675246 | 2 | 202864487 | T | G | 0.203 | 0.015 | 0.003 | 2.90E-08 | 7.37E-05 | 30.8 |
| rs13029509 | 2 | 215374209 | A | G | 0.480 | 0.018 | 0.002 | 2.00E-17 | 1.83E-04 | 76.3 |
| rs10932837 | 2 | 221044785 | T | C | 0.506 | 0.013 | 0.002 | 1.30E-09 | 9.41E-05 | 39.3 |
| rs4973576 | 2 | 233806771 | A | C | 0.702 | 0.015 | 0.002 | 1.00E-09 | 8.93E-05 | 37.3 |
| rs138256022 | 3 | 11603090 | T | C | 0.041 | 0.031 | 0.006 | 2.90E-08 | 7.63E-05 | 31.9 |
| rs9834970 | 3 | 36856030 | T | C | 0.501 | 0.013 | 0.002 | 3.30E-09 | 8.92E-05 | 37.3 |
| rs1352890 | 3 | 49652156 | T | C | 0.525 | 0.012 | 0.002 | 1.30E-08 | 8.87E-05 | 37.1 |
| rs3796386 | 3 | 49899795 | A | G | 0.428 | 0.026 | 0.002 | 3.20E-33 | 3.93E-04 | 164.3 |
| rs182355396 | 3 | 50920457 | A | C | 0.010 | 0.080 | 0.013 | 2.10E-10 | 0.00012 | 51.6 |
| rs11917871 | 3 | 54233476 | C | T | 0.575 | 0.017 | 0.002 | 4.50E-14 | 1.20E-04 | 50.3 |
| rs11130793 | 3 | 60884659 | C | T | 0.602 | 0.013 | 0.002 | 5.00E-09 | 9.51E-05 | 39.7 |
| rs11714337 | 3 | 71582521 | G | A | 0.569 | 0.014 | 0.002 | 4.70E-11 | 1.27E-04 | 53.1 |
| rs12496084 | 3 | 75174777 | G | A | 0.247 | 0.014 | 0.003 | 4.60E-08 | 7.10E-05 | 29.7 |
| rs6797840 | 3 | 85656569 | C | A | 0.544 | 0.016 | 0.002 | 1.70E-13 | 1.35E-04 | 56.4 |
| rs56398417 | 3 | 88024986 | C | T | 0.687 | 0.014 | 0.002 | 5.90E-10 | 1.04E-04 | 43.6 |
| rs2034768 | 3 | 93986371 | A | G | 0.487 | 0.015 | 0.002 | 8.60E-12 | 1.20E-04 | 50.0 |
| rs12632778 | 3 | 106452359 | G | T | 0.046 | 0.028 | 0.005 | 3.90E-08 | 7.06E-05 | 29.5 |
| rs9867121 | 3 | 114631548 | C | A | 0.818 | 0.019 | 0.003 | 3.90E-12 | 1.15E-04 | 47.9 |
| rs12491503 | 3 | 165706855 | A | G | 0.330 | 0.014 | 0.002 | 5.50E-10 | 9.14E-05 | 38.2 |
| rs114600294 | 3 | 181419367 | C | G | 0.211 | 0.016 | 0.003 | 7.90E-10 | 9.69E-05 | 40.5 |
| rs66852340 | 4 | 3311070 | T | C | 0.224 | 0.018 | 0.003 | 7.90E-12 | 1.24E-04 | 51.8 |
| rs7700107 | 4 | 17880416 | C | A | 0.137 | 0.021 | 0.003 | 1.90E-11 | 1.15E-04 | 47.9 |
| rs34811474 | 4 | 25408838 | G | A | 0.768 | 0.015 | 0.003 | 2.30E-09 | 9.89E-05 | 41.3 |
| rs77215114 | 4 | 45187658 | A | T | 0.930 | 0.024 | 0.004 | 1.50E-08 | 9.42E-05 | 39.3 |
| rs28971796 | 4 | 49151982 | A | G | 0.378 | 0.015 | 0.002 | 2.10E-09 | 8.60E-05 | 35.9 |
| rs6850494 | 4 | 82291771 | C | A | 0.384 | 0.014 | 0.002 | 1.10E-10 | 1.08E-04 | 45.0 |
| rs13107325 | 4 | 103188709 | T | C | 0.074 | 0.029 | 0.004 | 1.50E-12 | 1.36E-04 | 56.7 |
| rs62332760 | 4 | 106135316 | T | G | 0.387 | 0.014 | 0.002 | 8.00E-11 | 1.02E-04 | 42.8 |
| rs7693703 | 4 | 118357405 | G | A | 0.910 | 0.023 | 0.004 | 2.70E-09 | 8.32E-05 | 34.8 |
| rs4334769 | 4 | 130245233 | G | T | 0.474 | 0.012 | 0.002 | 2.10E-08 | 9.13E-05 | 38.1 |
| rs6825241 | 4 | 152543783 | A | C | 0.464 | 0.017 | 0.002 | 4.90E-15 | 1.52E-04 | 63.4 |
| rs7693082 | 4 | 159857819 | G | C | 0.300 | 0.015 | 0.002 | 1.70E-10 | 1.10E-04 | 46.1 |
| rs10940659 | 5 | 59360341 | G | A | 0.469 | 0.013 | 0.002 | 1.50E-08 | 9.00E-05 | 37.6 |
| rs2409838 | 5 | 60204410 | A | T | 0.555 | 0.017 | 0.002 | 3.40E-15 | 1.60E-04 | 67.0 |
| rs262890 | 5 | 62930015 | G | A | 0.301 | 0.019 | 0.002 | 3.20E-15 | 1.54E-04 | 64.4 |
| rs7716447 | 5 | 88800331 | G | A | 0.361 | 0.013 | 0.002 | 3.60E-09 | 9.29E-05 | 38.8 |
| rs1031423 | 5 | 93276883 | C | T | 0.785 | 0.019 | 0.003 | 1.80E-12 | 1.22E-04 | 50.9 |
| rs57585211 | 5 | 107197725 | G | T | 0.173 | 0.017 | 0.003 | 4.30E-09 | 8.21E-05 | 34.3 |
| rs9326862 | 5 | 112054547 | A | T | 0.468 | 0.012 | 0.002 | 2.60E-08 | 5.59E-05 | 23.3 |
| rs10041724 | 5 | 124273520 | T | C | 0.808 | 0.018 | 0.003 | 3.90E-11 | 1.18E-04 | 49.4 |
| rs10057545 | 5 | 138431008 | A | G | 0.445 | 0.014 | 0.002 | 2.00E-10 | 8.89E-05 | 37.1 |
| rs62379379 | 5 | 141082015 | T | G | 0.071 | 0.026 | 0.004 | 7.80E-10 | 8.77E-05 | 36.6 |
| rs10054327 | 5 | 147871137 | G | A | 0.576 | 0.017 | 0.002 | 3.40E-15 | 1.57E-04 | 65.5 |
| rs42210 | 5 | 166408788 | C | G | 0.711 | 0.014 | 0.002 | 7.30E-09 | 8.67E-05 | 36.2 |
| rs2336895 | 5 | 166990695 | G | A | 0.360 | 0.012 | 0.002 | 4.60E-08 | 6.39E-05 | 26.7 |
| rs72828890 | 5 | 167806836 | C | T | 0.869 | 0.019 | 0.003 | 4.90E-09 | 9.51E-05 | 39.7 |
| rs255292 | 5 | 172580866 | A | C | 0.598 | 0.012 | 0.002 | 1.70E-08 | 7.47E-05 | 31.2 |
| rs72834698 | 6 | 26176517 | G | A | 0.858 | 0.023 | 0.003 | 2.70E-13 | 1.62E-04 | 67.8 |
| rs3135044 | 6 | 31592820 | T | C | 0.360 | 0.015 | 0.002 | 8.70E-11 | 1.02E-04 | 42.8 |
| rs576432906 | 6 | 33443967 | C | A | 0.289 | 0.014 | 0.002 | 9.60E-09 | 0.00009 | 37.1 |
| rs10947452 | 6 | 33803752 | T | C | 0.349 | 0.014 | 0.002 | 6.40E-10 | 9.46E-05 | 39.5 |
| rs9471333 | 6 | 40362023 | C | T | 0.450 | 0.013 | 0.002 | 1.50E-09 | 1.05E-04 | 43.8 |
| rs4523073 | 6 | 67550288 | G | A | 0.409 | 0.014 | 0.002 | 5.00E-10 | 9.68E-05 | 40.4 |
| rs77589760 | 6 | 89827716 | C | G | 0.961 | 0.037 | 0.006 | 4.10E-10 | 8.31E-05 | 34.7 |
| rs6905544 | 6 | 98411631 | G | A | 0.601 | 0.019 | 0.002 | 8.50E-18 | 2.22E-04 | 92.8 |
| rs17789218 | 6 | 100600097 | T | C | 0.756 | 0.019 | 0.003 | 1.40E-13 | 1.36E-04 | 57.0 |
| rs78394231 | 6 | 107649123 | C | T | 0.098 | 0.021 | 0.004 | 9.70E-09 | 1.03E-04 | 42.8 |
| rs2184364 | 6 | 143183596 | A | G | 0.782 | 0.016 | 0.003 | 3.00E-09 | 8.97E-05 | 37.4 |
| rs9718104 | 6 | 166170539 | G | T | 0.058 | 0.041 | 0.005 | 9.30E-19 | 1.81E-04 | 75.4 |
| rs17568389 | 7 | 8091876 | T | A | 0.487 | 0.015 | 0.002 | 8.60E-13 | 1.43E-04 | 59.9 |
| rs10234444 | 7 | 41828527 | G | A | 0.823 | 0.016 | 0.003 | 2.90E-08 | 8.33E-05 | 34.8 |
| rs6973656 | 7 | 77422583 | G | A | 0.397 | 0.013 | 0.002 | 1.00E-09 | 9.08E-05 | 37.9 |
| rs973734 | 7 | 89387578 | C | A | 0.152 | 0.017 | 0.003 | 1.50E-08 | 8.05E-05 | 33.6 |
| rs3137 | 7 | 99130875 | G | A | 0.846 | 0.017 | 0.003 | 1.40E-08 | 7.58E-05 | 31.6 |
| rs2694151 | 7 | 101902180 | C | T | 0.058 | 0.026 | 0.005 | 2.30E-08 | 8.77E-05 | 36.6 |
| rs62471080 | 7 | 109128060 | C | G | 0.459 | 0.013 | 0.002 | 3.70E-09 | 9.08E-05 | 37.9 |
| rs7788008 | 7 | 112972483 | G | A | 0.566 | 0.013 | 0.002 | 5.90E-09 | 8.43E-05 | 35.2 |
| rs10246289 | 7 | 115544763 | A | G | 0.113 | 0.019 | 0.003 | 1.70E-08 | 7.71E-05 | 32.2 |
| rs11763734 | 7 | 126400910 | C | A | 0.490 | 0.012 | 0.002 | 8.20E-09 | 8.64E-05 | 36.1 |
| rs7834121 | 8 | 10770255 | T | G | 0.496 | 0.014 | 0.002 | 1.10E-10 | 1.03E-04 | 43.1 |
| rs1421334 | 8 | 30865733 | A | C | 0.450 | 0.017 | 0.002 | 2.20E-15 | 1.58E-04 | 66.0 |
| rs6996198 | 8 | 65463442 | T | C | 0.157 | 0.016 | 0.003 | 3.50E-08 | 8.30E-05 | 34.6 |
| rs6472942 | 8 | 76827190 | C | T | 0.432 | 0.013 | 0.002 | 1.80E-09 | 8.53E-05 | 35.6 |
| rs11985394 | 8 | 92667521 | T | A | 0.545 | 0.012 | 0.002 | 1.30E-08 | 7.39E-05 | 30.8 |
| rs72671494 | 8 | 93195457 | C | T | 0.141 | 0.017 | 0.003 | 4.50E-08 | 6.92E-05 | 28.9 |
| rs12541615 | 8 | 118870151 | C | T | 0.183 | 0.018 | 0.003 | 3.90E-10 | 9.26E-05 | 38.7 |
| rs2717559 | 8 | 143882420 | A | G | 0.559 | 0.012 | 0.002 | 1.50E-08 | 6.94E-05 | 29.0 |
| rs2616830 | 9 | 1721385 | G | A | 0.462 | 0.016 | 0.002 | 2.90E-14 | 1.27E-04 | 53.0 |
| rs2291681 | 9 | 14790841 | G | T | 0.584 | 0.013 | 0.002 | 7.90E-09 | 7.73E-05 | 32.3 |
| rs34864022 | 9 | 22609110 | G | A | 0.066 | 0.026 | 0.004 | 1.40E-09 | 9.14E-05 | 38.2 |
| rs12554512 | 9 | 23352293 | T | C | 0.584 | 0.021 | 0.002 | 3.80E-21 | 2.41E-04 | 100.7 |
| rs2791574 | 9 | 82212094 | G | T | 0.343 | 0.013 | 0.002 | 3.00E-08 | 7.55E-05 | 31.5 |
| rs141184308 | 9 | 86463339 | A | G | 0.978 | 0.043 | 0.008 | 2.60E-08 | 7.91E-05 | 33.0 |
| rs870151 | 9 | 92230747 | A | T | 0.472 | 0.016 | 0.002 | 8.80E-13 | 1.41E-04 | 58.9 |
| rs2787374 | 9 | 103054951 | T | C | 0.408 | 0.012 | 0.002 | 1.20E-08 | 7.07E-05 | 29.5 |
| rs10739499 | 9 | 120514261 | C | G | 0.337 | 0.014 | 0.002 | 1.50E-09 | 9.39E-05 | 39.2 |
| rs7043521 | 9 | 124602779 | A | T | 0.431 | 0.014 | 0.002 | 6.50E-11 | 1.15E-04 | 48.1 |
| rs12342024 | 9 | 126134232 | T | G | 0.114 | 0.021 | 0.003 | 1.70E-09 | 8.60E-05 | 35.9 |
| rs4382592 | 9 | 134870755 | T | G | 0.301 | 0.014 | 0.002 | 6.70E-09 | 9.15E-05 | 38.2 |
| rs2073869 | 9 | 135763816 | C | T | 0.834 | 0.019 | 0.003 | 1.50E-10 | 1.05E-04 | 43.7 |
| 9:139926402_T_C | 9 | 139926402 | C | T | 0.634 | 0.013 | 0.002 | 2.00E-08 | 0.00007 | 30.7 |
| rs1623025 | 10 | 11164813 | C | G | 0.471 | 0.012 | 0.002 | 1.40E-08 | 8.98E-05 | 37.5 |
| rs1243182 | 10 | 21916728 | T | C | 0.310 | 0.019 | 0.002 | 2.00E-15 | 1.61E-04 | 67.3 |
| rs2045147 | 10 | 56660437 | A | G | 0.449 | 0.013 | 0.002 | 5.90E-09 | 6.84E-05 | 28.6 |
| rs10994943 | 10 | 63591413 | T | G | 0.581 | 0.013 | 0.002 | 3.10E-09 | 9.21E-05 | 38.5 |
| rs11201422 | 10 | 86924483 | T | C | 0.669 | 0.013 | 0.002 | 3.40E-08 | 6.74E-05 | 28.1 |
| rs10786658 | 10 | 103737193 | T | A | 0.586 | 0.014 | 0.002 | 4.60E-11 | 1.11E-04 | 46.5 |
| rs7089973 | 10 | 116569565 | A | C | 0.381 | 0.013 | 0.002 | 2.40E-09 | 8.35E-05 | 34.9 |
| rs11245482 | 10 | 126733546 | C | T | 0.387 | 0.013 | 0.002 | 2.60E-09 | 8.79E-05 | 36.7 |
| rs17727474 | 10 | 127182959 | C | T | 0.832 | 0.018 | 0.003 | 3.10E-09 | 8.69E-05 | 36.3 |
| rs12289262 | 11 | 12894758 | T | C | 0.269 | 0.014 | 0.002 | 2.00E-08 | 8.63E-05 | 36.0 |
| rs801733 | 11 | 65934549 | A | C | 0.641 | 0.017 | 0.002 | 7.30E-14 | 1.44E-04 | 60.2 |
| rs116175338 | 11 | 72467875 | T | G | 0.246 | 0.014 | 0.003 | 1.40E-08 | 7.32E-05 | 30.6 |
| rs12272012 | 11 | 84861286 | C | G | 0.044 | 0.030 | 0.005 | 1.90E-08 | 7.18E-05 | 30.0 |
| rs11020045 | 11 | 92487907 | C | A | 0.331 | 0.013 | 0.002 | 1.60E-08 | 8.68E-05 | 36.2 |
| rs17207890 | 11 | 95490754 | G | A | 0.657 | 0.016 | 0.002 | 6.70E-12 | 1.26E-04 | 52.4 |
| rs10791546 | 11 | 101830126 | C | T | 0.589 | 0.012 | 0.002 | 4.10E-08 | 6.74E-05 | 28.1 |
| rs113584870 | 11 | 107243922 | G | A | 0.824 | 0.017 | 0.003 | 1.40E-08 | 0.00008 | 33.2 |
| rs328277 | 11 | 110414195 | C | A | 0.398 | 0.012 | 0.002 | 4.50E-08 | 5.70E-05 | 23.8 |
| rs648044 | 11 | 114030799 | G | A | 0.605 | 0.014 | 0.002 | 1.00E-09 | 1.08E-04 | 45.0 |
| rs11218575 | 11 | 122154576 | C | T | 0.566 | 0.015 | 0.002 | 2.10E-12 | 1.10E-04 | 46.1 |
| rs74802478 | 11 | 131966907 | G | A | 0.819 | 0.017 | 0.003 | 3.50E-09 | 7.72E-05 | 32.2 |
| rs4937842 | 11 | 133734542 | C | G | 0.366 | 0.013 | 0.002 | 1.40E-08 | 7.94E-05 | 33.2 |
| rs10772643 | 12 | 13415288 | C | T | 0.108 | 0.025 | 0.003 | 1.30E-12 | 1.17E-04 | 48.8 |
| rs74996610 | 12 | 24075007 | G | C | 0.046 | 0.031 | 0.005 | 4.10E-09 | 7.97E-05 | 33.3 |
| rs10771746 | 12 | 30791864 | T | C | 0.283 | 0.014 | 0.002 | 2.50E-09 | 9.61E-05 | 40.1 |
| rs10876864 | 12 | 56401085 | A | G | 0.573 | 0.013 | 0.002 | 1.00E-09 | 9.57E-05 | 40.0 |
| rs8756 | 12 | 66359752 | A | C | 0.515 | 0.013 | 0.002 | 5.30E-10 | 9.62E-05 | 40.2 |
| rs11179838 | 12 | 74321123 | C | G | 0.644 | 0.013 | 0.002 | 2.10E-08 | 7.70E-05 | 32.1 |
| rs2164744 | 12 | 109872039 | C | T | 0.365 | 0.013 | 0.002 | 1.10E-08 | 7.67E-05 | 32.0 |
| rs2173650 | 12 | 117525235 | G | T | 0.852 | 0.018 | 0.003 | 4.60E-09 | 8.91E-05 | 37.2 |
| rs1790135 | 12 | 123669235 | T | C | 0.756 | 0.014 | 0.003 | 2.60E-08 | 8.66E-05 | 36.2 |
| rs9563168 | 13 | 54247827 | G | A | 0.791 | 0.018 | 0.003 | 4.30E-11 | 1.11E-04 | 46.3 |
| rs9569734 | 13 | 58319476 | A | G | 0.844 | 0.019 | 0.003 | 3.40E-10 | 1.11E-04 | 46.4 |
| rs9528023 | 13 | 60226991 | C | T | 0.724 | 0.013 | 0.002 | 3.80E-08 | 8.54E-05 | 35.6 |
| rs56858768 | 13 | 86511730 | A | G | 0.299 | 0.015 | 0.002 | 3.40E-10 | 1.04E-04 | 43.4 |
| rs553397860 | 13 | 99051118 | A | G | 0.845 | 0.017 | 0.003 | 8.90E-09 | 7.82E-05 | 32.7 |
| rs7991062 | 13 | 100713194 | G | C | 0.341 | 0.018 | 0.002 | 8.80E-15 | 1.55E-04 | 64.8 |
| rs11629120 | 14 | 23458457 | C | T | 0.414 | 0.013 | 0.002 | 1.10E-08 | 8.37E-05 | 34.9 |
| rs405797 | 14 | 26972834 | A | T | 0.753 | 0.015 | 0.003 | 2.80E-09 | 9.92E-05 | 41.4 |
| rs1022785 | 14 | 29571653 | G | A | 0.142 | 0.018 | 0.003 | 7.10E-09 | 1.01E-04 | 42.4 |
| rs10145592 | 14 | 94287215 | G | C | 0.591 | 0.015 | 0.002 | 1.80E-11 | 1.13E-04 | 47.0 |
| rs7157001 | 14 | 99749484 | G | A | 0.256 | 0.014 | 0.002 | 2.30E-08 | 7.76E-05 | 32.4 |
| rs8043253 | 15 | 41503730 | T | C | 0.427 | 0.012 | 0.002 | 3.40E-08 | 7.98E-05 | 33.3 |
| rs2460 | 15 | 53073084 | A | G | 0.264 | 0.015 | 0.002 | 5.00E-10 | 1.02E-04 | 42.4 |
| rs4775373 | 15 | 61476936 | T | C | 0.361 | 0.013 | 0.002 | 4.70E-09 | 9.02E-05 | 37.7 |
| rs61331678 | 15 | 77995949 | G | C | 0.568 | 0.015 | 0.002 | 6.10E-12 | 1.13E-04 | 47.2 |
| rs35574015 | 16 | 15146061 | C | T | 0.294 | 0.013 | 0.002 | 3.00E-08 | 7.84E-05 | 32.7 |
| rs7189927 | 16 | 28913787 | T | C | 0.356 | 0.015 | 0.002 | 3.40E-11 | 1.17E-04 | 48.7 |
| rs749671 | 16 | 31088347 | G | A | 0.629 | 0.016 | 0.002 | 2.70E-12 | 1.26E-04 | 52.5 |
| rs7184800 | 16 | 53509131 | G | A | 0.697 | 0.017 | 0.002 | 8.20E-13 | 1.38E-04 | 57.7 |
| rs142710267 | 16 | 72169262 | T | G | 0.650 | 0.016 | 0.002 | 6.60E-12 | 1.08E-04 | 45.1 |
| rs2447098 | 17 | 2277720 | A | C | 0.525 | 0.015 | 0.002 | 6.80E-12 | 1.19E-04 | 49.7 |
| rs11654952 | 17 | 4813799 | G | T | 0.139 | 0.017 | 0.003 | 4.30E-08 | 6.59E-05 | 27.5 |
| rs2584597 | 17 | 61941284 | T | C | 0.662 | 0.015 | 0.002 | 2.90E-10 | 9.49E-05 | 39.6 |
| rs9902312 | 17 | 65070304 | T | C | 0.683 | 0.015 | 0.002 | 4.50E-11 | 9.79E-05 | 40.9 |
| rs11657730 | 17 | 79372489 | C | T | 0.637 | 0.013 | 0.002 | 1.10E-08 | 9.44E-05 | 39.4 |
| rs303753 | 18 | 21074922 | A | G | 0.347 | 0.014 | 0.002 | 2.70E-10 | 1.08E-04 | 45.3 |
| rs71362609 | 18 | 23104382 | C | T | 0.486 | 0.012 | 0.002 | 2.80E-08 | 7.49E-05 | 31.3 |
| rs11081851 | 18 | 31783262 | A | G | 0.562 | 0.013 | 0.002 | 1.00E-08 | 7.61E-05 | 31.8 |
| rs9964724 | 18 | 35159124 | C | T | 0.318 | 0.018 | 0.002 | 3.30E-14 | 1.70E-04 | 71.2 |
| rs1156541 | 18 | 39952989 | C | T | 0.223 | 0.014 | 0.003 | 2.80E-08 | 6.15E-05 | 25.7 |
| rs148544378 | 18 | 40323567 | C | T | 0.979 | 0.053 | 0.008 | 2.20E-12 | 1.30E-04 | 54.4 |
| rs17512836 | 18 | 53194961 | T | C | 0.974 | 0.042 | 0.007 | 7.00E-10 | 9.30E-05 | 38.8 |
| rs7248205 | 19 | 10770305 | C | T | 0.398 | 0.014 | 0.002 | 3.40E-10 | 1.09E-04 | 45.4 |
| rs111901094 | 19 | 19513570 | T | G | 0.183 | 0.017 | 0.003 | 2.00E-09 | 1.00E-04 | 41.7 |
| rs6131281 | 20 | 11891724 | C | T | 0.597 | 0.016 | 0.002 | 3.10E-13 | 1.33E-04 | 55.4 |
| rs6141814 | 20 | 31368960 | A | C | 0.387 | 0.014 | 0.002 | 1.30E-09 | 9.34E-05 | 39.0 |
| rs4810312 | 20 | 39968707 | G | C | 0.421 | 0.013 | 0.002 | 1.20E-08 | 8.45E-05 | 35.3 |
| rs6124476 | 20 | 41202830 | T | C | 0.589 | 0.012 | 0.002 | 1.80E-08 | 8.06E-05 | 33.6 |
| rs6031440 | 20 | 42809317 | A | T | 0.398 | 0.012 | 0.002 | 1.80E-08 | 7.75E-05 | 32.4 |
| rs55700114 | 20 | 43717080 | A | G | 0.287 | 0.014 | 0.002 | 2.10E-09 | 1.00E-04 | 41.9 |
| rs6125906 | 20 | 48729803 | A | C | 0.092 | 0.021 | 0.004 | 3.90E-08 | 7.16E-05 | 29.9 |
| rs56103247 | 20 | 62483184 | C | T | 0.944 | 0.030 | 0.005 | 3.80E-10 | 9.35E-05 | 39.1 |
| rs10427502 | 21 | 40654840 | G | A | 0.622 | 0.014 | 0.002 | 8.60E-10 | 8.34E-05 | 34.8 |
| rs7609 | 22 | 29951650 | A | T | 0.231 | 0.015 | 0.003 | 3.60E-09 | 8.58E-05 | 35.8 |
| rs2092829 | 22 | 31780514 | G | A | 0.706 | 0.014 | 0.002 | 1.30E-08 | 7.24E-05 | 30.2 |
| rs150399729 | 22 | 42969453 | C | G | 0.969 | 0.039 | 0.007 | 3.60E-08 | 6.98E-05 | 29.2 |
| Chr chromosome, EA effect allele, EAF effect allele frequency, OA other allele, se standard error, SNP single nucleotide polymorphism  ^a^ Position based on GRCh37 | | | | | | | | | | |

| **Table S2: Summary information on leisure computer use SNPs used as genetic instruments used in the Mendelian randomisation analyses** | | | | | | | | | | |
| --- | --- | --- | --- | --- | --- | --- | --- | --- | --- | --- |
| SNP | CHR | POS | EA | OA | EAF | beta | se | P_value | R^2^ | F_statistic |
| rs2748985 | 1 | 1853184 | C | T | 0.545 | 0.015 | 0.002 | 4.10E-12 | 1.22E-04 | 50.7 |
| rs12145677 | 1 | 110023610 | A | G | 0.298 | 0.017 | 0.002 | 1.20E-12 | 1.27E-04 | 52.8 |
| rs7526456 | 1 | 197773734 | C | T | 0.587 | 0.013 | 0.002 | 1.20E-08 | 8.36E-05 | 34.7 |
| rs10754920 | 2 | 146456888 | C | G | 0.112 | 0.020 | 0.003 | 6.70E-09 | 7.59E-05 | 31.5 |
| rs6744254 | 2 | 155986195 | T | C | 0.529 | 0.016 | 0.002 | 5.40E-13 | 1.27E-04 | 52.5 |
| rs11274218 | 2 | 215035001 | T | C | 0.389 | 0.014 | 0.002 | 2.50E-09 | 8.63E-05 | 35.8 |
| rs4592851 | 2 | 215364232 | T | G | 0.250 | 0.014 | 0.003 | 1.40E-08 | 8.99E-05 | 37.3 |
| rs10208088 | 2 | 221055873 | C | T | 0.419 | 0.013 | 0.002 | 4.40E-09 | 9.21E-05 | 38.2 |
| rs11708955 | 3 | 49540114 | C | T | 0.305 | 0.016 | 0.002 | 4.20E-11 | 1.14E-04 | 47.2 |
| rs6774533 | 3 | 62471086 | T | C | 0.706 | 0.015 | 0.002 | 1.20E-09 | 9.14E-05 | 37.9 |
| rs78082503 | 3 | 116556913 | C | G | 0.214 | 0.015 | 0.003 | 1.10E-08 | 7.38E-05 | 30.6 |
| rs6857629 | 4 | 39684977 | G | A | 0.755 | 0.015 | 0.003 | 1.90E-09 | 9.25E-05 | 38.4 |
| rs2068625 | 4 | 159856739 | C | T | 0.698 | 0.016 | 0.002 | 4.00E-11 | 1.08E-04 | 44.8 |
| rs2220599 | 5 | 7378854 | G | C | 0.367 | 0.016 | 0.002 | 2.40E-12 | 1.15E-04 | 47.6 |
| rs11749912 | 5 | 88065628 | A | G | 0.423 | 0.014 | 0.002 | 4.90E-10 | 9.48E-05 | 39.3 |
| rs1469249 | 5 | 113837198 | G | A | 0.789 | 0.016 | 0.003 | 9.50E-09 | 8.09E-05 | 33.6 |
| rs162894 | 5 | 131611872 | T | G | 0.332 | 0.013 | 0.002 | 1.10E-08 | 7.66E-05 | 31.8 |
| rs56158102 | 5 | 144027803 | C | T | 0.344 | 0.013 | 0.002 | 3.10E-08 | 7.49E-05 | 31.1 |
| rs9477970 | 6 | 19082663 | A | T | 0.192 | 0.016 | 0.003 | 1.00E-08 | 8.15E-05 | 33.8 |
| rs2179517 | 6 | 26198845 | C | G | 0.449 | 0.012 | 0.002 | 1.90E-08 | 9.25E-05 | 38.4 |
| rs9372625 | 6 | 98344031 | A | G | 0.381 | 0.018 | 0.002 | 5.70E-16 | 1.65E-04 | 68.3 |
| rs17789218 | 6 | 100600097 | C | T | 0.244 | 0.015 | 0.003 | 4.20E-09 | 8.57E-05 | 35.6 |
| rs6935828 | 6 | 140811367 | T | C | 0.554 | 0.012 | 0.002 | 4.30E-08 | 7.02E-05 | 29.1 |
| rs55772938 | 7 | 70025113 | G | A | 0.295 | 0.015 | 0.002 | 3.90E-10 | 1.02E-04 | 42.4 |
| rs12706626 | 7 | 124531370 | A | G | 0.383 | 0.013 | 0.002 | 1.40E-08 | 8.13E-05 | 33.7 |
| rs3944151 | 7 | 126970005 | A | G | 0.275 | 0.014 | 0.002 | 3.10E-08 | 7.20E-05 | 29.9 |
| rs2345941 | 7 | 133337635 | A | G | 0.550 | 0.015 | 0.002 | 4.00E-11 | 1.16E-04 | 48.3 |
| rs13262595 | 8 | 143316970 | G | A | 0.560 | 0.016 | 0.002 | 1.20E-12 | 1.23E-04 | 51.1 |
| rs4977839 | 9 | 23355310 | A | G | 0.416 | 0.020 | 0.002 | 4.70E-19 | 2.01E-04 | 83.5 |
| rs113851275 | 9 | 98297220 | A | G | 0.108 | 0.021 | 0.004 | 3.20E-09 | 8.60E-05 | 35.7 |
| rs7020477 | 9 | 116827760 | A | G | 0.735 | 0.014 | 0.002 | 1.20E-08 | 7.81E-05 | 32.4 |
| rs73578186 | 9 | 126334485 | C | T | 0.675 | 0.015 | 0.002 | 2.30E-10 | 9.66E-05 | 40.1 |
| rs2734849 | 11 | 113270160 | G | A | 0.509 | 0.014 | 0.002 | 8.20E-10 | 9.70E-05 | 40.3 |
| rs1448355 | 11 | 131286685 | T | C | 0.619 | 0.015 | 0.002 | 1.30E-10 | 9.83E-05 | 40.8 |
| rs35933007 | 12 | 38929341 | A | G | 0.228 | 0.015 | 0.003 | 9.20E-09 | 8.79E-05 | 36.5 |
| rs1319734 | 12 | 40367517 | A | G | 0.601 | 0.013 | 0.002 | 1.90E-08 | 7.72E-05 | 32.1 |
| rs206965 | 12 | 120856332 | T | C | 0.207 | 0.016 | 0.003 | 9.10E-09 | 8.19E-05 | 34.0 |
| rs12874776 | 13 | 57794345 | A | C | 0.107 | 0.023 | 0.004 | 8.00E-11 | 1.01E-04 | 41.8 |
| rs198262 | 14 | 57283283 | C | T | 0.044 | 0.030 | 0.005 | 3.90E-08 | 7.19E-05 | 29.8 |
| rs166835 | 15 | 47716037 | C | T | 0.443 | 0.013 | 0.002 | 3.40E-09 | 8.96E-05 | 37.2 |
| rs4702 | 15 | 91426560 | G | A | 0.444 | 0.012 | 0.002 | 3.80E-08 | 7.30E-05 | 30.3 |
| rs6498759 | 16 | 18020295 | T | C | 0.310 | 0.013 | 0.002 | 2.70E-08 | 8.10E-05 | 33.6 |
| rs3730399 | 16 | 67229019 | A | G | 0.935 | 0.025 | 0.004 | 3.30E-08 | 7.72E-05 | 32.0 |
| rs4073003 | 17 | 19148305 | A | G | 0.874 | 0.020 | 0.003 | 1.20E-09 | 9.54E-05 | 39.6 |
| rs7209653 | 17 | 19882084 | T | C | 0.704 | 0.016 | 0.002 | 9.10E-11 | 9.97E-05 | 41.4 |
| rs12603813 | 17 | 43196584 | T | C | 0.746 | 0.014 | 0.003 | 1.30E-08 | 7.98E-05 | 33.1 |
| rs66643547 | 17 | 79375315 | T | C | 0.353 | 0.015 | 0.002 | 3.20E-11 | 1.09E-04 | 45.3 |
| rs784256 | 18 | 53398626 | G | A | 0.188 | 0.019 | 0.003 | 6.20E-11 | 1.04E-04 | 43.3 |
| rs6129084 | 20 | 59828857 | T | A | 0.641 | 0.014 | 0.002 | 9.30E-10 | 8.92E-05 | 37.0 |
| rs9608249 | 22 | 24613952 | G | A | 0.851 | 0.017 | 0.003 | 2.20E-08 | 7.49E-05 | 31.1 |
| rs136553 | 22 | 27255675 | T | C | 0.376 | 0.015 | 0.002 | 2.30E-11 | 1.11E-04 | 46.1 |
| rs7288455 | 22 | 39966547 | A | G | 0.436 | 0.013 | 0.002 | 4.00E-09 | 8.55E-05 | 35.5 |
| Chr chromosome, EA effect allele, EAF effect allele frequency, OA other allele, se standard error, SNP single nucleotide polymorphism  ^a^ Position based on GRCh37 | | | | | | | | | | |

| **Table S3: Summary information on breast cancer for the leisure television watching SNPs used in the Mendelian randomisation analyses** | | | | | | | | | | | | | | |
| --- | --- | --- | --- | --- | --- | --- | --- | --- | --- | --- | --- | --- | --- | --- |
| SNP | EA | OA | brst_beta | brst_se | Lum_A_beta | Lum_A_se | Lum_B_beta | Lum_B_se | HER2Neg_beta | HER2Neg_se | HER2_Enriched_beta | HER2_Enriched_se | 3N_beta | 3N_se |
| rs11810109 | A | T | 0.005 | 0.007 | -0.001 | 0.009 | 0.020 | 0.019 | 0.016 | 0.016 | 0.000 | 0.028 | 0.026 | 0.017 |
| rs112632642 | A | G | 0.007 | 0.012 | 0.011 | 0.016 | -0.002 | 0.035 | -0.052 | 0.031 | 0.076 | 0.049 | 0.005 | 0.030 |
| rs6670915 | C | T | 0.005 | 0.006 | -0.002 | 0.008 | 0.004 | 0.018 | 0.014 | 0.015 | -0.027 | 0.026 | 0.011 | 0.015 |
| rs12725114 | G | A | 0.007 | 0.007 | 0.016 | 0.010 | 0.029 | 0.022 | 0.002 | 0.018 | 0.040 | 0.032 | -0.004 | 0.019 |
| rs984409 | A | G | 0.015 | 0.006 | 0.019 | 0.008 | 0.006 | 0.019 | 0.032 | 0.016 | -0.022 | 0.027 | 0.025 | 0.016 |
| rs10890123 | C | T | 0.002 | 0.008 | 0.019 | 0.010 | -0.012 | 0.023 | 0.012 | 0.019 | -0.019 | 0.033 | -0.016 | 0.020 |
| rs71658797 | A | T | 0.004 | 0.010 | 0.003 | 0.013 | 0.034 | 0.029 | -0.059 | 0.025 | 0.038 | 0.042 | -0.002 | 0.025 |
| rs114328297 | T | G | -0.006 | 0.008 | 0.001 | 0.010 | -0.021 | 0.023 | -0.019 | 0.020 | 0.053 | 0.034 | -0.033 | 0.020 |
| rs631130 | A | T | 0.006 | 0.007 | 0.013 | 0.009 | 0.007 | 0.021 | -0.006 | 0.018 | -0.042 | 0.030 | 0.016 | 0.018 |
| rs148648822 | T | G | 0.005 | 0.009 | -0.004 | 0.011 | 0.006 | 0.025 | 0.011 | 0.021 | -0.022 | 0.037 | 0.008 | 0.021 |
| rs17379561 | T | A | 0.002 | 0.009 | 0.001 | 0.011 | -0.052 | 0.025 | 0.029 | 0.021 | -0.010 | 0.036 | 0.008 | 0.021 |
| rs55909997 | A | G | 0.002 | 0.006 | -0.002 | 0.008 | -0.016 | 0.019 | 0.024 | 0.016 | 0.005 | 0.027 | 0.024 | 0.016 |
| rs1278847 | C | A | -0.012 | 0.007 | -0.016 | 0.009 | -0.008 | 0.019 | -0.004 | 0.017 | -0.017 | 0.028 | 0.011 | 0.017 |
| rs141425667 | A | G | -0.016 | 0.014 | -0.017 | 0.018 | -0.013 | 0.038 | -0.047 | 0.034 | -0.029 | 0.057 | 0.032 | 0.033 |
| rs4845364 | G | A | -0.005 | 0.006 | -0.007 | 0.008 | -0.002 | 0.018 | 0.002 | 0.015 | -0.018 | 0.026 | -0.020 | 0.015 |
| rs6673341 | G | T | 0.000 | 0.006 | 0.006 | 0.008 | 0.004 | 0.018 | -0.007 | 0.015 | 0.005 | 0.026 | 0.026 | 0.015 |
| rs72725224 | C | A | -0.034 | 0.025 | -0.023 | 0.032 | -0.056 | 0.070 | -0.038 | 0.061 | -0.046 | 0.101 | -0.042 | 0.062 |
| rs10737620 | T | A | 0.011 | 0.007 | 0.022 | 0.009 | 0.017 | 0.020 | 0.012 | 0.017 | 0.012 | 0.029 | -0.017 | 0.017 |
| rs6721975 | C | T | 0.012 | 0.008 | 0.008 | 0.010 | -0.018 | 0.022 | -0.008 | 0.019 | 0.031 | 0.033 | 0.025 | 0.020 |
| rs11896615 | G | A | -0.002 | 0.009 | 0.004 | 0.011 | 0.020 | 0.025 | -0.022 | 0.021 | -0.019 | 0.036 | -0.024 | 0.021 |
| rs72781699 | A | G | -0.011 | 0.008 | -0.006 | 0.010 | -0.016 | 0.022 | 0.015 | 0.019 | 0.007 | 0.032 | -0.043 | 0.019 |
| rs12105701 | T | C | 0.003 | 0.006 | 0.004 | 0.008 | 0.020 | 0.018 | -0.043 | 0.016 | -0.026 | 0.026 | 0.027 | 0.016 |
| rs7564130 | C | T | 0.002 | 0.006 | 0.010 | 0.008 | 0.016 | 0.018 | 0.005 | 0.016 | -0.005 | 0.026 | -0.006 | 0.016 |
| rs11125515 | A | C | 0.005 | 0.007 | -0.001 | 0.009 | 0.016 | 0.020 | 0.017 | 0.017 | 0.057 | 0.028 | 0.013 | 0.017 |
| rs12475168 | T | G | 0.008 | 0.009 | 0.002 | 0.012 | 0.052 | 0.025 | 0.017 | 0.022 | 0.036 | 0.037 | -0.001 | 0.022 |
| rs183566425 | A | T | -0.023 | 0.031 | -0.076 | 0.039 | 0.088 | 0.094 | 0.066 | 0.080 | 0.324 | 0.156 | -0.082 | 0.075 |
| rs10189857 | G | A | 0.017 | 0.006 | 0.019 | 0.008 | 0.011 | 0.018 | 0.022 | 0.015 | 0.008 | 0.026 | 0.015 | 0.015 |
| rs62641636 | A | G | 0.001 | 0.007 | -0.003 | 0.009 | 0.039 | 0.019 | 0.009 | 0.016 | -0.042 | 0.028 | 0.016 | 0.017 |
| rs59210467 | T | C | 0.000 | 0.007 | 0.011 | 0.009 | -0.025 | 0.020 | -0.007 | 0.017 | 0.049 | 0.028 | 0.012 | 0.017 |
| rs11689199 | A | G | -0.010 | 0.006 | -0.008 | 0.008 | -0.016 | 0.018 | -0.007 | 0.016 | -0.014 | 0.027 | -0.017 | 0.016 |
| rs2916251 | T | G | 0.010 | 0.006 | 0.008 | 0.008 | -0.018 | 0.018 | 0.003 | 0.016 | 0.004 | 0.027 | 0.018 | 0.016 |
| rs1451533 | A | G | 0.004 | 0.007 | 0.015 | 0.009 | -0.032 | 0.021 | 0.001 | 0.018 | -0.018 | 0.031 | 0.005 | 0.018 |
| rs2971640 | A | G | -0.010 | 0.006 | -0.008 | 0.008 | -0.016 | 0.018 | -0.018 | 0.015 | -0.013 | 0.026 | -0.002 | 0.015 |
| rs12476388 | C | T | -0.002 | 0.007 | 0.002 | 0.009 | 0.009 | 0.019 | -0.004 | 0.017 | -0.035 | 0.028 | -0.014 | 0.017 |
| rs374722 | G | A | 0.011 | 0.009 | 0.018 | 0.012 | 0.036 | 0.025 | -0.012 | 0.022 | -0.080 | 0.039 | 0.001 | 0.022 |
| rs3754970 | C | T | -0.007 | 0.006 | 0.000 | 0.008 | 0.003 | 0.018 | -0.006 | 0.015 | 0.017 | 0.026 | -0.023 | 0.016 |
| rs1913808 | G | A | 0.010 | 0.007 | 0.003 | 0.009 | 0.052 | 0.019 | -0.012 | 0.016 | 0.001 | 0.028 | 0.001 | 0.016 |
| rs2060199 | T | A | -0.007 | 0.006 | -0.007 | 0.008 | 0.010 | 0.018 | 0.019 | 0.016 | 0.011 | 0.026 | -0.021 | 0.016 |
| rs263771 | A | C | -0.003 | 0.007 | -0.001 | 0.010 | -0.016 | 0.021 | -0.007 | 0.018 | -0.008 | 0.031 | -0.028 | 0.018 |
| rs4577309 | A | G | -0.001 | 0.006 | 0.004 | 0.008 | -0.014 | 0.018 | 0.008 | 0.015 | -0.006 | 0.026 | -0.015 | 0.015 |
| rs34224261 | C | G | 0.000 | 0.006 | 0.006 | 0.008 | -0.004 | 0.017 | 0.026 | 0.015 | 0.060 | 0.026 | 0.001 | 0.015 |
| rs4675246 | T | G | -0.006 | 0.008 | -0.013 | 0.010 | 0.005 | 0.022 | -0.038 | 0.019 | 0.077 | 0.032 | 0.040 | 0.019 |
| rs13029509 | A | G | 0.009 | 0.006 | 0.008 | 0.008 | -0.008 | 0.018 | 0.019 | 0.015 | -0.017 | 0.026 | 0.017 | 0.015 |
| rs10932837 | T | C | -0.001 | 0.006 | -0.003 | 0.008 | 0.011 | 0.018 | -0.009 | 0.015 | 0.023 | 0.026 | 0.000 | 0.015 |
| rs4973576 | A | C | -0.012 | 0.007 | -0.022 | 0.009 | -0.031 | 0.020 | 0.015 | 0.017 | -0.018 | 0.029 | -0.026 | 0.017 |
| rs138256022 | T | C | 0.016 | 0.017 | 0.025 | 0.023 | 0.024 | 0.050 | 0.043 | 0.043 | 0.061 | 0.072 | 0.005 | 0.044 |
| rs9834970 | T | C | 0.001 | 0.006 | 0.001 | 0.008 | 0.005 | 0.018 | 0.004 | 0.016 | -0.035 | 0.027 | 0.011 | 0.016 |
| rs1352890 | T | C | 0.010 | 0.006 | 0.009 | 0.008 | 0.004 | 0.018 | 0.019 | 0.015 | 0.034 | 0.026 | 0.014 | 0.015 |
| rs3796386 | A | G | 0.018 | 0.006 | 0.014 | 0.008 | 0.038 | 0.018 | 0.012 | 0.015 | 0.063 | 0.026 | 0.043 | 0.015 |
| rs11917871 | C | T | 0.005 | 0.006 | 0.017 | 0.008 | 0.009 | 0.019 | -0.015 | 0.016 | -0.047 | 0.027 | 0.025 | 0.016 |
| rs11130793 | C | T | 0.001 | 0.006 | 0.006 | 0.008 | 0.012 | 0.018 | -0.021 | 0.016 | 0.004 | 0.027 | 0.002 | 0.016 |
| rs11714337 | G | A | 0.032 | 0.006 | 0.045 | 0.008 | 0.036 | 0.018 | 0.029 | 0.015 | 0.000 | 0.026 | 0.014 | 0.015 |
| rs12496084 | G | A | -0.002 | 0.007 | -0.008 | 0.010 | -0.018 | 0.021 | 0.015 | 0.018 | -0.069 | 0.031 | 0.016 | 0.018 |
| rs6797840 | C | A | 0.004 | 0.006 | 0.007 | 0.008 | 0.015 | 0.018 | 0.022 | 0.015 | -0.024 | 0.026 | -0.005 | 0.015 |
| rs56398417 | C | T | -0.018 | 0.007 | -0.019 | 0.009 | -0.040 | 0.019 | -0.033 | 0.016 | -0.014 | 0.028 | -0.026 | 0.016 |
| rs2034768 | A | G | -0.003 | 0.006 | -0.006 | 0.008 | 0.010 | 0.018 | 0.016 | 0.015 | 0.016 | 0.025 | -0.025 | 0.015 |
| rs12632778 | G | T | 0.023 | 0.015 | 0.044 | 0.019 | -0.025 | 0.044 | 0.012 | 0.037 | -0.100 | 0.066 | 0.026 | 0.037 |
| rs9867121 | C | A | 0.013 | 0.008 | 0.015 | 0.010 | 0.020 | 0.023 | 0.001 | 0.020 | 0.052 | 0.034 | 0.009 | 0.020 |
| rs12491503 | A | G | 0.001 | 0.007 | -0.008 | 0.009 | 0.018 | 0.019 | 0.029 | 0.016 | 0.025 | 0.028 | -0.009 | 0.017 |
| rs114600294 | C | G | -0.017 | 0.010 | -0.002 | 0.013 | -0.029 | 0.028 | 0.015 | 0.024 | -0.035 | 0.042 | -0.007 | 0.025 |
| rs66852340 | T | C | 0.016 | 0.007 | 0.019 | 0.009 | -0.027 | 0.021 | 0.046 | 0.018 | 0.011 | 0.031 | 0.022 | 0.018 |
| rs7700107 | C | A | -0.005 | 0.009 | -0.033 | 0.012 | 0.019 | 0.025 | 0.026 | 0.022 | 0.066 | 0.036 | 0.009 | 0.022 |
| rs34811474 | G | A | 0.006 | 0.008 | -0.002 | 0.011 | 0.015 | 0.025 | 0.012 | 0.021 | 0.045 | 0.036 | 0.005 | 0.021 |
| rs77215114 | A | T | -0.016 | 0.013 | -0.022 | 0.018 | -0.019 | 0.039 | 0.021 | 0.034 | 0.022 | 0.057 | -0.051 | 0.033 |
| rs28971796 | A | G | 0.010 | 0.009 | 0.017 | 0.012 | 0.042 | 0.026 | 0.005 | 0.022 | 0.049 | 0.037 | 0.017 | 0.022 |
| rs6850494 | C | A | -0.009 | 0.006 | -0.004 | 0.008 | -0.030 | 0.018 | -0.002 | 0.015 | 0.045 | 0.026 | -0.020 | 0.016 |
| rs13107325 | T | C | 0.019 | 0.012 | 0.021 | 0.016 | 0.051 | 0.034 | 0.007 | 0.030 | -0.051 | 0.051 | 0.025 | 0.030 |
| rs62332760 | T | G | -0.017 | 0.006 | -0.018 | 0.008 | -0.016 | 0.018 | -0.024 | 0.016 | 0.039 | 0.026 | 0.021 | 0.016 |
| rs7693703 | G | A | -0.007 | 0.010 | -0.015 | 0.014 | -0.012 | 0.030 | 0.009 | 0.026 | 0.052 | 0.046 | 0.005 | 0.026 |
| rs4334769 | G | T | -0.008 | 0.006 | -0.007 | 0.008 | -0.016 | 0.018 | -0.032 | 0.015 | 0.012 | 0.026 | 0.015 | 0.015 |
| rs6825241 | A | C | -0.002 | 0.006 | -0.009 | 0.008 | 0.008 | 0.018 | 0.018 | 0.015 | -0.008 | 0.026 | -0.006 | 0.016 |
| rs7693082 | G | C | -0.004 | 0.007 | -0.013 | 0.009 | -0.023 | 0.020 | -0.001 | 0.017 | 0.044 | 0.028 | -0.013 | 0.017 |
| rs10940659 | G | A | -0.008 | 0.006 | -0.020 | 0.008 | 0.061 | 0.018 | -0.014 | 0.015 | 0.028 | 0.026 | -0.003 | 0.015 |
| rs2409838 | A | T | -0.002 | 0.006 | -0.010 | 0.008 | 0.007 | 0.018 | 0.009 | 0.015 | 0.015 | 0.026 | -0.011 | 0.016 |
| rs262890 | G | A | 0.003 | 0.007 | 0.002 | 0.009 | -0.032 | 0.019 | 0.006 | 0.016 | 0.021 | 0.028 | 0.008 | 0.017 |
| rs7716447 | G | A | -0.007 | 0.007 | -0.009 | 0.009 | 0.013 | 0.019 | -0.011 | 0.016 | -0.034 | 0.028 | 0.011 | 0.017 |
| rs1031423 | C | T | 0.002 | 0.008 | 0.007 | 0.010 | -0.013 | 0.022 | -0.013 | 0.019 | 0.010 | 0.032 | -0.021 | 0.019 |
| rs57585211 | G | T | -0.001 | 0.008 | 0.000 | 0.011 | -0.021 | 0.024 | 0.036 | 0.020 | -0.044 | 0.035 | 0.026 | 0.020 |
| rs9326862 | A | T | 0.005 | 0.006 | 0.008 | 0.008 | 0.008 | 0.018 | -0.002 | 0.015 | 0.025 | 0.026 | 0.005 | 0.015 |
| rs10041724 | T | C | 0.008 | 0.008 | 0.006 | 0.010 | 0.020 | 0.022 | -0.011 | 0.019 | 0.065 | 0.033 | -0.021 | 0.019 |
| rs10057545 | A | G | 0.006 | 0.006 | -0.002 | 0.008 | 0.030 | 0.018 | 0.007 | 0.016 | 0.003 | 0.027 | 0.014 | 0.016 |
| rs62379379 | T | G | 0.001 | 0.012 | 0.014 | 0.016 | -0.074 | 0.037 | -0.019 | 0.031 | -0.122 | 0.055 | 0.031 | 0.030 |
| rs10054327 | G | A | -0.010 | 0.006 | -0.013 | 0.008 | 0.000 | 0.018 | 0.004 | 0.015 | 0.003 | 0.026 | -0.022 | 0.016 |
| rs42210 | C | G | 0.020 | 0.007 | 0.008 | 0.010 | 0.046 | 0.021 | 0.046 | 0.018 | 0.083 | 0.031 | 0.019 | 0.018 |
| rs2336895 | G | A | 0.007 | 0.007 | 0.013 | 0.009 | -0.008 | 0.020 | 0.020 | 0.017 | -0.019 | 0.029 | 0.001 | 0.017 |
| rs72828890 | C | T | -0.005 | 0.011 | 0.006 | 0.014 | -0.010 | 0.032 | 0.027 | 0.028 | 0.002 | 0.047 | 0.012 | 0.028 |
| rs255292 | A | C | -0.003 | 0.006 | 0.001 | 0.008 | 0.001 | 0.018 | -0.003 | 0.016 | 0.018 | 0.026 | -0.020 | 0.016 |
| rs72834698 | G | A | 0.007 | 0.009 | 0.019 | 0.012 | -0.001 | 0.026 | -0.022 | 0.022 | 0.030 | 0.038 | 0.019 | 0.023 |
| rs3135044 | T | C | 0.004 | 0.006 | -0.003 | 0.008 | 0.018 | 0.018 | -0.002 | 0.015 | 0.010 | 0.026 | -0.021 | 0.016 |
| rs576432906 | C | A | 0.007 | 0.007 | -0.001 | 0.009 | 0.023 | 0.020 | 0.011 | 0.017 | 0.027 | 0.029 | 0.025 | 0.017 |
| rs10947452 | T | C | 0.011 | 0.007 | 0.011 | 0.009 | -0.016 | 0.019 | 0.032 | 0.016 | -0.005 | 0.028 | 0.014 | 0.016 |
| rs9471333 | C | T | 0.000 | 0.006 | 0.012 | 0.009 | -0.008 | 0.019 | -0.039 | 0.016 | -0.044 | 0.027 | 0.011 | 0.016 |
| rs4523073 | G | A | 0.002 | 0.006 | 0.004 | 0.008 | -0.025 | 0.018 | 0.006 | 0.016 | 0.003 | 0.027 | -0.008 | 0.016 |
| rs77589760 | C | G | 0.057 | 0.024 | 0.060 | 0.031 | 0.124 | 0.071 | 0.011 | 0.058 | 0.029 | 0.101 | 0.124 | 0.062 |
| rs6905544 | G | A | 0.011 | 0.006 | 0.016 | 0.008 | 0.007 | 0.018 | 0.012 | 0.015 | -0.005 | 0.026 | 0.012 | 0.016 |
| rs17789218 | T | C | -0.013 | 0.008 | -0.025 | 0.011 | -0.002 | 0.023 | 0.030 | 0.020 | 0.068 | 0.034 | 0.014 | 0.020 |
| rs78394231 | C | T | 0.003 | 0.010 | 0.001 | 0.013 | -0.011 | 0.030 | 0.018 | 0.025 | 0.017 | 0.043 | 0.004 | 0.026 |
| rs2184364 | A | G | 0.007 | 0.007 | 0.009 | 0.010 | 0.009 | 0.022 | -0.004 | 0.018 | -0.035 | 0.031 | 0.023 | 0.019 |
| rs9718104 | G | T | 0.010 | 0.014 | 0.009 | 0.018 | -0.009 | 0.041 | -0.004 | 0.034 | -0.041 | 0.060 | 0.054 | 0.034 |
| rs17568389 | T | A | -0.003 | 0.006 | -0.008 | 0.008 | -0.012 | 0.018 | -0.014 | 0.015 | 0.038 | 0.026 | 0.010 | 0.016 |
| rs10234444 | G | A | 0.011 | 0.008 | 0.001 | 0.011 | 0.031 | 0.025 | 0.005 | 0.021 | -0.069 | 0.035 | 0.021 | 0.022 |
| rs6973656 | G | A | 0.000 | 0.006 | -0.006 | 0.009 | 0.013 | 0.019 | 0.019 | 0.016 | 0.013 | 0.027 | -0.003 | 0.016 |
| rs973734 | C | A | 0.013 | 0.009 | 0.015 | 0.011 | 0.032 | 0.025 | 0.015 | 0.021 | 0.017 | 0.036 | 0.023 | 0.022 |
| rs3137 | G | A | 0.010 | 0.008 | 0.002 | 0.011 | 0.018 | 0.024 | 0.004 | 0.021 | 0.037 | 0.035 | 0.019 | 0.021 |
| rs2694151 | C | T | -0.003 | 0.013 | -0.008 | 0.017 | 0.039 | 0.036 | 0.028 | 0.031 | -0.046 | 0.055 | -0.046 | 0.033 |
| rs62471080 | C | G | -0.005 | 0.006 | -0.006 | 0.008 | -0.027 | 0.018 | -0.008 | 0.015 | -0.004 | 0.026 | 0.004 | 0.015 |
| rs7788008 | G | A | 0.000 | 0.006 | 0.002 | 0.008 | -0.027 | 0.018 | 0.013 | 0.015 | -0.019 | 0.026 | 0.005 | 0.015 |
| rs10246289 | A | G | 0.021 | 0.009 | 0.018 | 0.012 | 0.021 | 0.027 | 0.014 | 0.024 | 0.032 | 0.040 | 0.048 | 0.024 |
| rs11763734 | C | A | -0.003 | 0.006 | 0.003 | 0.008 | -0.011 | 0.018 | -0.009 | 0.015 | 0.006 | 0.026 | -0.024 | 0.015 |
| rs7834121 | T | G | 0.008 | 0.007 | 0.009 | 0.009 | -0.008 | 0.021 | -0.005 | 0.017 | 0.013 | 0.028 | 0.008 | 0.018 |
| rs1421334 | A | C | 0.005 | 0.006 | 0.002 | 0.008 | 0.031 | 0.018 | -0.006 | 0.015 | 0.018 | 0.026 | 0.009 | 0.016 |
| rs6996198 | T | C | 0.001 | 0.008 | 0.001 | 0.011 | 0.002 | 0.024 | 0.002 | 0.020 | -0.065 | 0.036 | 0.036 | 0.020 |
| rs6472942 | C | T | 0.012 | 0.006 | 0.020 | 0.008 | -0.023 | 0.018 | 0.013 | 0.015 | 0.031 | 0.025 | 0.006 | 0.015 |
| rs11985394 | T | A | 0.005 | 0.006 | 0.010 | 0.008 | 0.002 | 0.018 | 0.005 | 0.015 | -0.012 | 0.026 | 0.001 | 0.016 |
| rs72671494 | C | T | -0.001 | 0.009 | 0.006 | 0.012 | 0.034 | 0.027 | -0.030 | 0.024 | 0.007 | 0.040 | -0.025 | 0.024 |
| rs12541615 | C | T | -0.029 | 0.008 | -0.037 | 0.011 | -0.035 | 0.023 | -0.048 | 0.020 | -0.039 | 0.034 | -0.004 | 0.020 |
| rs2717559 | A | G | 0.000 | 0.007 | -0.009 | 0.009 | 0.018 | 0.019 | -0.022 | 0.016 | 0.030 | 0.028 | -0.003 | 0.017 |
| rs2616830 | G | A | 0.003 | 0.006 | 0.006 | 0.008 | -0.001 | 0.018 | 0.012 | 0.015 | -0.052 | 0.026 | 0.001 | 0.015 |
| rs2291681 | G | T | 0.009 | 0.006 | 0.007 | 0.008 | 0.010 | 0.019 | 0.019 | 0.016 | -0.023 | 0.027 | 0.027 | 0.016 |
| rs34864022 | G | A | -0.008 | 0.013 | -0.002 | 0.017 | -0.001 | 0.038 | -0.043 | 0.032 | 0.046 | 0.054 | -0.011 | 0.033 |
| rs12554512 | T | C | -0.006 | 0.006 | -0.001 | 0.008 | -0.038 | 0.019 | -0.006 | 0.016 | 0.017 | 0.027 | -0.018 | 0.016 |
| rs2791574 | G | T | -0.003 | 0.006 | -0.011 | 0.008 | -0.009 | 0.018 | 0.004 | 0.016 | -0.026 | 0.027 | 0.029 | 0.016 |
| rs141184308 | A | G | 0.012 | 0.033 | 0.058 | 0.044 | 0.147 | 0.102 | -0.082 | 0.080 | -0.132 | 0.135 | -0.049 | 0.082 |
| rs870151 | A | T | -0.005 | 0.006 | 0.008 | 0.008 | -0.014 | 0.018 | 0.005 | 0.015 | -0.043 | 0.026 | -0.021 | 0.015 |
| rs2787374 | T | C | -0.004 | 0.006 | -0.010 | 0.008 | 0.001 | 0.018 | 0.042 | 0.015 | -0.015 | 0.026 | 0.000 | 0.015 |
| rs10739499 | C | G | 0.001 | 0.007 | 0.014 | 0.009 | -0.003 | 0.019 | -0.010 | 0.016 | -0.017 | 0.028 | -0.014 | 0.017 |
| rs7043521 | A | T | 0.003 | 0.006 | 0.003 | 0.008 | -0.019 | 0.018 | 0.016 | 0.015 | 0.037 | 0.026 | -0.019 | 0.015 |
| rs12342024 | T | G | 0.002 | 0.010 | -0.001 | 0.013 | 0.000 | 0.028 | -0.015 | 0.025 | 0.012 | 0.041 | -0.013 | 0.025 |
| rs4382592 | T | G | 0.011 | 0.007 | 0.004 | 0.009 | 0.030 | 0.019 | 0.002 | 0.016 | 0.045 | 0.028 | 0.006 | 0.017 |
| rs2073869 | C | T | 0.011 | 0.008 | 0.001 | 0.011 | 0.012 | 0.023 | 0.029 | 0.020 | 0.019 | 0.034 | -0.013 | 0.020 |
| 9:139926402_T_C | C | T | -0.002 | 0.006 | 0.002 | 0.008 | -0.006 | 0.018 | -0.029 | 0.016 | -0.026 | 0.027 | 0.015 | 0.016 |
| rs1623025 | C | G | 0.009 | 0.006 | 0.012 | 0.008 | 0.011 | 0.018 | 0.013 | 0.015 | 0.044 | 0.026 | 0.000 | 0.015 |
| rs1243182 | T | C | 0.056 | 0.006 | 0.081 | 0.008 | 0.059 | 0.019 | 0.051 | 0.016 | 0.016 | 0.027 | -0.042 | 0.016 |
| rs2045147 | A | G | 0.002 | 0.006 | -0.002 | 0.008 | 0.023 | 0.018 | 0.032 | 0.015 | 0.045 | 0.026 | 0.001 | 0.015 |
| rs10994943 | T | G | 0.011 | 0.006 | 0.010 | 0.008 | -0.009 | 0.018 | 0.015 | 0.015 | 0.028 | 0.026 | 0.032 | 0.015 |
| rs11201422 | T | C | -0.015 | 0.007 | -0.007 | 0.009 | -0.025 | 0.019 | -0.027 | 0.016 | -0.059 | 0.028 | 0.007 | 0.017 |
| rs10786658 | T | A | -0.001 | 0.006 | -0.002 | 0.008 | 0.032 | 0.018 | -0.022 | 0.015 | 0.011 | 0.026 | -0.014 | 0.016 |
| rs7089973 | A | C | 0.005 | 0.006 | 0.008 | 0.009 | -0.025 | 0.019 | 0.007 | 0.016 | 0.045 | 0.027 | -0.002 | 0.016 |
| rs11245482 | C | T | 0.005 | 0.006 | 0.003 | 0.008 | 0.037 | 0.018 | -0.004 | 0.015 | 0.016 | 0.026 | -0.022 | 0.016 |
| rs17727474 | C | T | -0.013 | 0.008 | -0.020 | 0.011 | 0.021 | 0.025 | -0.048 | 0.021 | -0.078 | 0.035 | 0.025 | 0.022 |
| rs12289262 | T | C | -0.002 | 0.007 | -0.008 | 0.009 | 0.064 | 0.021 | 0.013 | 0.018 | 0.001 | 0.030 | -0.012 | 0.018 |
| rs801733 | A | C | 0.023 | 0.006 | 0.031 | 0.008 | 0.016 | 0.019 | 0.001 | 0.016 | 0.057 | 0.027 | -0.002 | 0.016 |
| rs116175338 | T | G | 0.017 | 0.007 | 0.028 | 0.009 | -0.007 | 0.021 | 0.004 | 0.018 | 0.016 | 0.030 | 0.025 | 0.018 |
| rs12272012 | C | G | 0.023 | 0.015 | 0.017 | 0.019 | -0.098 | 0.046 | 0.003 | 0.037 | 0.045 | 0.063 | 0.040 | 0.037 |
| rs11020045 | C | A | 0.009 | 0.006 | 0.003 | 0.008 | 0.000 | 0.019 | 0.017 | 0.016 | 0.019 | 0.027 | 0.022 | 0.016 |
| rs17207890 | G | A | 0.017 | 0.006 | 0.033 | 0.008 | 0.000 | 0.019 | 0.039 | 0.016 | -0.011 | 0.027 | 0.019 | 0.016 |
| rs10791546 | C | T | -0.001 | 0.006 | 0.007 | 0.008 | 0.005 | 0.018 | -0.014 | 0.015 | 0.000 | 0.026 | -0.016 | 0.015 |
| rs113584870 | G | A | 0.009 | 0.009 | 0.004 | 0.011 | 0.022 | 0.025 | 0.011 | 0.022 | 0.068 | 0.038 | 0.011 | 0.022 |
| rs328277 | C | A | -0.007 | 0.006 | -0.013 | 0.008 | 0.005 | 0.018 | 0.001 | 0.015 | 0.012 | 0.026 | 0.008 | 0.015 |
| rs648044 | G | A | 0.005 | 0.007 | 0.004 | 0.009 | 0.024 | 0.020 | 0.009 | 0.017 | 0.004 | 0.030 | -0.003 | 0.018 |
| rs11218575 | C | T | 0.009 | 0.006 | 0.000 | 0.009 | 0.024 | 0.019 | 0.001 | 0.016 | 0.063 | 0.027 | 0.008 | 0.016 |
| rs74802478 | G | A | -0.007 | 0.008 | -0.002 | 0.011 | 0.011 | 0.024 | -0.007 | 0.020 | -0.012 | 0.034 | -0.030 | 0.020 |
| rs4937842 | C | G | -0.009 | 0.006 | -0.012 | 0.008 | 0.005 | 0.018 | -0.017 | 0.016 | -0.020 | 0.027 | -0.010 | 0.016 |
| rs10772643 | C | T | 0.008 | 0.010 | 0.022 | 0.013 | -0.012 | 0.030 | -0.005 | 0.025 | -0.044 | 0.044 | -0.017 | 0.026 |
| rs74996610 | G | C | 0.023 | 0.015 | 0.032 | 0.019 | -0.067 | 0.046 | 0.038 | 0.037 | 0.070 | 0.062 | -0.005 | 0.038 |
| rs10771746 | T | C | 0.024 | 0.007 | 0.015 | 0.009 | 0.037 | 0.019 | 0.053 | 0.016 | 0.018 | 0.028 | -0.013 | 0.017 |
| rs10876864 | A | G | -0.004 | 0.006 | -0.008 | 0.008 | -0.040 | 0.018 | 0.011 | 0.015 | -0.008 | 0.026 | 0.024 | 0.015 |
| rs8756 | A | C | 0.003 | 0.006 | -0.004 | 0.008 | -0.009 | 0.018 | 0.007 | 0.015 | -0.019 | 0.025 | 0.023 | 0.015 |
| rs11179838 | C | G | -0.004 | 0.006 | -0.009 | 0.008 | -0.009 | 0.018 | 0.003 | 0.016 | -0.013 | 0.026 | 0.000 | 0.016 |
| rs2164744 | C | T | -0.019 | 0.006 | -0.009 | 0.008 | -0.024 | 0.018 | -0.042 | 0.016 | -0.072 | 0.026 | -0.011 | 0.016 |
| rs2173650 | G | T | 0.016 | 0.009 | 0.014 | 0.014 | 0.012 | 0.028 | 0.009 | 0.025 | 0.036 | 0.042 | 0.043 | 0.026 |
| rs1790135 | T | C | 0.003 | 0.007 | 0.000 | 0.009 | 0.006 | 0.020 | 0.019 | 0.017 | 0.021 | 0.029 | -0.003 | 0.017 |
| rs9563168 | G | A | 0.003 | 0.008 | 0.011 | 0.010 | 0.010 | 0.022 | 0.003 | 0.019 | -0.004 | 0.032 | -0.013 | 0.019 |
| rs9569734 | A | G | 0.002 | 0.008 | 0.010 | 0.011 | -0.034 | 0.024 | 0.014 | 0.021 | 0.002 | 0.036 | 0.023 | 0.021 |
| rs9528023 | C | T | 0.002 | 0.007 | -0.004 | 0.009 | -0.003 | 0.021 | 0.015 | 0.018 | -0.003 | 0.030 | -0.020 | 0.018 |
| rs56858768 | A | G | -0.012 | 0.007 | -0.008 | 0.009 | -0.012 | 0.020 | -0.017 | 0.017 | 0.002 | 0.029 | -0.004 | 0.017 |
| rs553397860 | A | G | -0.013 | 0.009 | -0.023 | 0.011 | -0.004 | 0.025 | 0.006 | 0.021 | 0.045 | 0.037 | -0.005 | 0.022 |
| rs7991062 | G | C | 0.004 | 0.006 | 0.012 | 0.008 | 0.001 | 0.019 | 0.001 | 0.016 | 0.036 | 0.027 | 0.005 | 0.016 |
| rs11629120 | C | T | 0.012 | 0.006 | 0.016 | 0.008 | 0.007 | 0.018 | 0.016 | 0.015 | 0.028 | 0.026 | 0.012 | 0.015 |
| rs405797 | A | T | -0.015 | 0.008 | -0.013 | 0.010 | -0.017 | 0.022 | -0.036 | 0.019 | -0.027 | 0.032 | -0.004 | 0.019 |
| rs1022785 | G | A | 0.008 | 0.009 | 0.009 | 0.011 | 0.007 | 0.025 | -0.014 | 0.022 | -0.026 | 0.037 | 0.032 | 0.022 |
| rs10145592 | G | C | -0.003 | 0.006 | 0.015 | 0.009 | 0.009 | 0.019 | -0.006 | 0.016 | -0.030 | 0.027 | -0.020 | 0.016 |
| rs7157001 | G | A | 0.003 | 0.008 | 0.007 | 0.011 | 0.020 | 0.024 | -0.027 | 0.021 | -0.018 | 0.035 | -0.013 | 0.021 |
| rs8043253 | T | C | 0.004 | 0.006 | 0.018 | 0.008 | -0.016 | 0.018 | -0.012 | 0.015 | 0.004 | 0.026 | 0.008 | 0.015 |
| rs2460 | A | G | 0.000 | 0.007 | 0.006 | 0.010 | 0.001 | 0.021 | 0.007 | 0.018 | -0.024 | 0.031 | -0.024 | 0.019 |
| rs4775373 | T | C | 0.002 | 0.006 | 0.000 | 0.008 | -0.001 | 0.019 | 0.004 | 0.016 | 0.034 | 0.027 | -0.026 | 0.016 |
| rs61331678 | G | C | 0.002 | 0.006 | 0.002 | 0.008 | 0.027 | 0.018 | -0.034 | 0.015 | -0.027 | 0.026 | 0.017 | 0.016 |
| rs35574015 | C | T | 0.007 | 0.007 | 0.000 | 0.009 | 0.023 | 0.020 | 0.008 | 0.017 | -0.026 | 0.030 | 0.015 | 0.018 |
| rs7189927 | T | C | 0.001 | 0.006 | 0.006 | 0.008 | -0.010 | 0.019 | -0.001 | 0.016 | 0.014 | 0.027 | -0.026 | 0.016 |
| rs749671 | G | A | -0.017 | 0.006 | -0.014 | 0.008 | -0.010 | 0.018 | -0.010 | 0.015 | -0.002 | 0.026 | -0.015 | 0.016 |
| rs7184800 | G | A | -0.005 | 0.006 | -0.010 | 0.008 | -0.014 | 0.019 | 0.005 | 0.016 | 0.026 | 0.028 | -0.011 | 0.016 |
| rs142710267 | T | G | 0.003 | 0.007 | 0.007 | 0.009 | 0.026 | 0.019 | -0.014 | 0.016 | 0.030 | 0.028 | 0.019 | 0.017 |
| rs2447098 | A | C | -0.008 | 0.006 | -0.009 | 0.009 | -0.012 | 0.019 | -0.036 | 0.016 | 0.005 | 0.027 | -0.020 | 0.016 |
| rs11654952 | G | T | -0.007 | 0.009 | -0.009 | 0.012 | -0.031 | 0.026 | -0.037 | 0.022 | -0.017 | 0.038 | 0.027 | 0.022 |
| rs2584597 | T | C | -0.006 | 0.007 | -0.008 | 0.009 | 0.007 | 0.020 | -0.013 | 0.017 | -0.016 | 0.028 | 0.010 | 0.017 |
| rs9902312 | T | C | -0.009 | 0.006 | -0.024 | 0.008 | -0.026 | 0.019 | 0.016 | 0.016 | -0.021 | 0.027 | 0.009 | 0.016 |
| rs11657730 | C | T | 0.021 | 0.007 | 0.021 | 0.009 | 0.007 | 0.019 | 0.024 | 0.017 | 0.062 | 0.029 | 0.023 | 0.017 |
| rs303753 | A | G | 0.004 | 0.006 | 0.010 | 0.009 | 0.033 | 0.019 | -0.005 | 0.016 | -0.026 | 0.028 | 0.051 | 0.016 |
| rs71362609 | C | T | 0.014 | 0.006 | 0.011 | 0.008 | 0.019 | 0.018 | 0.045 | 0.015 | -0.009 | 0.026 | -0.001 | 0.015 |
| rs11081851 | A | G | 0.003 | 0.006 | -0.001 | 0.008 | -0.004 | 0.018 | 0.003 | 0.015 | -0.009 | 0.026 | 0.029 | 0.015 |
| rs9964724 | C | T | 0.007 | 0.007 | 0.002 | 0.009 | 0.029 | 0.019 | 0.007 | 0.017 | -0.043 | 0.029 | 0.009 | 0.017 |
| rs1156541 | C | T | -0.018 | 0.007 | -0.012 | 0.009 | -0.043 | 0.021 | -0.029 | 0.018 | -0.010 | 0.030 | -0.044 | 0.018 |
| rs17512836 | T | C | -0.008 | 0.019 | -0.034 | 0.025 | 0.048 | 0.056 | -0.022 | 0.047 | -0.007 | 0.079 | 0.035 | 0.049 |
| rs7248205 | C | T | 0.012 | 0.006 | 0.012 | 0.008 | 0.030 | 0.019 | 0.021 | 0.016 | -0.012 | 0.027 | -0.022 | 0.016 |
| rs111901094 | T | G | 0.032 | 0.009 | 0.038 | 0.012 | 0.011 | 0.027 | 0.024 | 0.023 | 0.092 | 0.039 | 0.064 | 0.023 |
| rs6131281 | C | T | 0.002 | 0.007 | -0.006 | 0.009 | -0.007 | 0.019 | 0.008 | 0.016 | 0.016 | 0.027 | 0.010 | 0.016 |
| rs6141814 | A | C | -0.004 | 0.006 | -0.005 | 0.008 | -0.029 | 0.018 | 0.000 | 0.015 | -0.006 | 0.026 | 0.006 | 0.016 |
| rs4810312 | G | C | 0.009 | 0.006 | 0.014 | 0.008 | 0.007 | 0.018 | 0.006 | 0.015 | -0.005 | 0.026 | -0.017 | 0.015 |
| rs6124476 | T | C | -0.004 | 0.006 | 0.007 | 0.008 | -0.022 | 0.018 | 0.004 | 0.016 | 0.011 | 0.027 | -0.013 | 0.016 |
| rs6031440 | A | T | 0.005 | 0.006 | 0.013 | 0.008 | 0.006 | 0.019 | -0.007 | 0.016 | 0.011 | 0.027 | -0.012 | 0.016 |
| rs55700114 | A | G | 0.002 | 0.006 | 0.007 | 0.009 | -0.035 | 0.019 | 0.019 | 0.016 | -0.018 | 0.027 | 0.008 | 0.016 |
| rs6125906 | A | C | 0.004 | 0.011 | -0.002 | 0.014 | 0.029 | 0.031 | -0.040 | 0.027 | -0.041 | 0.047 | 0.023 | 0.027 |
| rs56103247 | C | T | -0.006 | 0.015 | -0.007 | 0.020 | 0.020 | 0.045 | -0.013 | 0.037 | -0.068 | 0.061 | 0.014 | 0.038 |
| rs10427502 | G | A | -0.012 | 0.006 | -0.013 | 0.008 | -0.003 | 0.018 | -0.021 | 0.016 | -0.067 | 0.027 | -0.014 | 0.016 |
| rs7609 | A | T | -0.003 | 0.007 | -0.001 | 0.010 | -0.014 | 0.021 | -0.024 | 0.018 | -0.029 | 0.031 | -0.010 | 0.019 |
| rs2092829 | G | A | -0.002 | 0.007 | -0.010 | 0.009 | -0.005 | 0.019 | -0.014 | 0.017 | -0.001 | 0.028 | 0.010 | 0.017 |
| rs150399729 | C | G | -0.011 | 0.017 | 0.012 | 0.022 | -0.044 | 0.049 | -0.052 | 0.041 | -0.069 | 0.069 | -0.032 | 0.042 |
| brst breast, EA effect allele, Lum luminal, Neg negative OA other allele, se standard error, SNP single nucleotide polymorphism, 3N triple negative | | | | | | | | | | | | | | |

| **Table S4: Summary information on breast cancer for the leisure computer use SNPs used in the Mendelian randomisation analyses** | | | | | | | | | | | | | | |
| --- | --- | --- | --- | --- | --- | --- | --- | --- | --- | --- | --- | --- | --- | --- |
| SNP | EA | OA | brst_beta | brst_se | Lum_A_beta | Lum_A_se | Lum_B_beta | Lum_B_se | HER2Neg_beta | HER2Neg_se | HER2_Enriched_beta | HER2_Enriched_se | 3N_beta | 3N_se |
| rs2748985 | C | T | -0.003 | 0.007 | -0.007 | 0.009 | -0.017 | 0.019 | 0.002 | 0.017 | 0.011 | 0.028 | -0.014 | 0.017 |
| rs12145677 | A | G | 0.011 | 0.007 | 0.016 | 0.009 | 0.012 | 0.020 | 0.003 | 0.017 | 0.009 | 0.028 | -0.013 | 0.017 |
| rs7526456 | C | T | -0.005 | 0.006 | -0.005 | 0.009 | -0.012 | 0.019 | -0.002 | 0.016 | 0.039 | 0.028 | -0.014 | 0.016 |
| rs10754920 | C | G | 0.004 | 0.010 | 0.016 | 0.013 | 0.005 | 0.028 | 0.015 | 0.024 | 0.014 | 0.040 | -0.064 | 0.025 |
| rs6744254 | T | C | -0.007 | 0.006 | -0.002 | 0.008 | 0.014 | 0.018 | -0.005 | 0.015 | -0.024 | 0.026 | -0.004 | 0.015 |
| rs11274218 | T | C | -0.014 | 0.006 | -0.008 | 0.008 | -0.030 | 0.018 | -0.003 | 0.016 | -0.006 | 0.027 | -0.021 | 0.016 |
| rs4592851 | T | G | -0.010 | 0.007 | -0.001 | 0.009 | 0.031 | 0.020 | -0.008 | 0.017 | 0.007 | 0.030 | -0.044 | 0.018 |
| rs10208088 | C | T | 0.001 | 0.006 | 0.002 | 0.008 | -0.021 | 0.018 | 0.012 | 0.015 | 0.000 | 0.026 | 0.006 | 0.015 |
| rs11708955 | C | T | -0.023 | 0.007 | -0.018 | 0.009 | -0.016 | 0.019 | -0.025 | 0.016 | -0.062 | 0.028 | -0.053 | 0.016 |
| rs6774533 | T | C | 0.012 | 0.007 | 0.022 | 0.009 | 0.004 | 0.021 | 0.004 | 0.018 | 0.047 | 0.031 | -0.018 | 0.018 |
| rs78082503 | C | G | -0.004 | 0.008 | 0.001 | 0.010 | -0.022 | 0.022 | -0.013 | 0.019 | -0.054 | 0.032 | -0.012 | 0.019 |
| rs6857629 | G | A | 0.009 | 0.007 | 0.014 | 0.009 | 0.003 | 0.021 | 0.023 | 0.018 | 0.005 | 0.030 | 0.015 | 0.018 |
| rs2068625 | C | T | 0.005 | 0.007 | 0.013 | 0.009 | 0.022 | 0.020 | 0.002 | 0.017 | -0.041 | 0.028 | 0.014 | 0.017 |
| rs2220599 | G | C | -0.008 | 0.007 | -0.014 | 0.009 | -0.018 | 0.019 | -0.005 | 0.016 | 0.005 | 0.027 | 0.015 | 0.016 |
| rs11749912 | A | G | 0.002 | 0.006 | -0.001 | 0.008 | 0.010 | 0.018 | 0.019 | 0.015 | -0.015 | 0.026 | 0.006 | 0.015 |
| rs1469249 | G | A | -0.011 | 0.007 | -0.008 | 0.010 | -0.023 | 0.021 | -0.006 | 0.018 | -0.013 | 0.031 | -0.019 | 0.019 |
| rs162894 | T | G | -0.024 | 0.006 | -0.034 | 0.008 | -0.048 | 0.019 | -0.031 | 0.016 | 0.013 | 0.027 | -0.030 | 0.016 |
| rs56158102 | C | T | -0.015 | 0.006 | -0.012 | 0.008 | -0.042 | 0.019 | -0.016 | 0.016 | -0.054 | 0.027 | -0.015 | 0.016 |
| rs9477970 | A | T | 0.002 | 0.008 | -0.001 | 0.010 | 0.001 | 0.023 | -0.017 | 0.020 | -0.007 | 0.033 | 0.002 | 0.020 |
| rs2179517 | C | G | 0.013 | 0.006 | 0.006 | 0.008 | 0.000 | 0.018 | 0.019 | 0.015 | 0.044 | 0.025 | 0.006 | 0.015 |
| rs9372625 | A | G | -0.005 | 0.006 | -0.009 | 0.008 | -0.003 | 0.019 | -0.003 | 0.016 | 0.008 | 0.027 | -0.006 | 0.016 |
| rs17789218 | C | T | 0.013 | 0.008 | 0.025 | 0.011 | 0.002 | 0.023 | -0.030 | 0.020 | -0.068 | 0.034 | -0.014 | 0.020 |
| rs6935828 | T | C | 0.004 | 0.006 | 0.003 | 0.008 | -0.018 | 0.018 | -0.007 | 0.015 | 0.028 | 0.026 | -0.001 | 0.015 |
| rs55772938 | G | A | -0.003 | 0.007 | 0.003 | 0.009 | -0.020 | 0.019 | 0.024 | 0.016 | -0.042 | 0.028 | -0.015 | 0.017 |
| rs12706626 | A | G | 0.009 | 0.006 | 0.016 | 0.008 | -0.012 | 0.018 | 0.015 | 0.015 | -0.006 | 0.026 | -0.004 | 0.016 |
| rs3944151 | A | G | 0.016 | 0.007 | 0.013 | 0.009 | 0.003 | 0.020 | 0.015 | 0.017 | 0.037 | 0.028 | 0.010 | 0.017 |
| rs2345941 | A | G | 0.007 | 0.006 | 0.002 | 0.008 | 0.036 | 0.018 | 0.007 | 0.015 | 0.019 | 0.026 | -0.002 | 0.015 |
| rs13262595 | G | A | 0.001 | 0.006 | -0.005 | 0.008 | 0.016 | 0.018 | -0.013 | 0.015 | -0.043 | 0.026 | 0.005 | 0.015 |
| rs4977839 | A | G | 0.006 | 0.006 | 0.001 | 0.008 | 0.037 | 0.019 | 0.006 | 0.016 | -0.019 | 0.027 | 0.022 | 0.016 |
| rs113851275 | A | G | 0.028 | 0.010 | 0.044 | 0.012 | 0.025 | 0.028 | 0.009 | 0.024 | -0.010 | 0.041 | -0.008 | 0.024 |
| rs7020477 | A | G | -0.005 | 0.008 | -0.011 | 0.010 | -0.012 | 0.022 | 0.007 | 0.019 | 0.007 | 0.033 | 0.037 | 0.020 |
| rs73578186 | C | T | -0.003 | 0.006 | -0.011 | 0.008 | 0.011 | 0.019 | -0.021 | 0.016 | -0.002 | 0.027 | -0.028 | 0.016 |
| rs2734849 | G | A | -0.003 | 0.006 | -0.001 | 0.008 | -0.023 | 0.018 | 0.002 | 0.015 | -0.004 | 0.025 | 0.005 | 0.015 |
| rs1448355 | T | C | 0.001 | 0.006 | 0.001 | 0.008 | -0.011 | 0.018 | 0.002 | 0.015 | -0.007 | 0.026 | 0.006 | 0.016 |
| rs35933007 | A | G | -0.003 | 0.008 | -0.017 | 0.011 | 0.000 | 0.024 | -0.018 | 0.020 | 0.017 | 0.034 | 0.030 | 0.021 |
| rs1319734 | A | G | 0.017 | 0.006 | 0.014 | 0.008 | 0.031 | 0.018 | 0.012 | 0.016 | -0.007 | 0.026 | 0.011 | 0.016 |
| rs206965 | T | C | 0.010 | 0.007 | 0.008 | 0.010 | 0.014 | 0.022 | 0.032 | 0.019 | -0.008 | 0.032 | 0.014 | 0.019 |
| rs12874776 | A | C | -0.001 | 0.010 | -0.007 | 0.013 | -0.036 | 0.030 | -0.007 | 0.026 | 0.005 | 0.044 | -0.018 | 0.026 |
| rs198262 | C | T | 0.002 | 0.015 | 0.011 | 0.020 | 0.041 | 0.044 | -0.020 | 0.038 | -0.019 | 0.065 | -0.010 | 0.039 |
| rs166835 | C | T | -0.013 | 0.006 | -0.023 | 0.008 | -0.038 | 0.018 | 0.002 | 0.015 | 0.001 | 0.026 | -0.015 | 0.015 |
| rs4702 | G | A | 0.029 | 0.006 | 0.016 | 0.008 | 0.057 | 0.018 | 0.071 | 0.015 | -0.002 | 0.026 | 0.007 | 0.015 |
| rs6498759 | T | C | 0.012 | 0.007 | 0.021 | 0.009 | 0.034 | 0.020 | -0.028 | 0.017 | 0.021 | 0.029 | 0.017 | 0.018 |
| rs3730399 | A | G | -0.015 | 0.012 | 0.005 | 0.016 | -0.038 | 0.035 | 0.021 | 0.030 | -0.032 | 0.051 | -0.015 | 0.030 |
| rs4073003 | A | G | -0.007 | 0.009 | -0.005 | 0.012 | -0.015 | 0.027 | -0.025 | 0.023 | -0.047 | 0.039 | -0.006 | 0.023 |
| rs7209653 | T | C | -0.007 | 0.007 | 0.000 | 0.009 | -0.035 | 0.020 | -0.012 | 0.017 | 0.012 | 0.029 | -0.023 | 0.017 |
| rs12603813 | T | C | 0.005 | 0.007 | 0.017 | 0.009 | 0.036 | 0.020 | -0.003 | 0.017 | 0.033 | 0.030 | -0.032 | 0.017 |
| rs66643547 | T | C | -0.020 | 0.007 | -0.021 | 0.009 | -0.005 | 0.019 | -0.024 | 0.017 | -0.061 | 0.029 | -0.019 | 0.017 |
| rs784256 | G | A | -0.005 | 0.008 | 0.006 | 0.010 | -0.020 | 0.023 | 0.009 | 0.020 | -0.015 | 0.033 | -0.034 | 0.020 |
| rs6129084 | T | A | 0.001 | 0.006 | -0.005 | 0.008 | 0.004 | 0.019 | 0.010 | 0.016 | -0.015 | 0.027 | 0.001 | 0.016 |
| rs9608249 | G | A | 0.008 | 0.009 | 0.019 | 0.012 | -0.015 | 0.025 | -0.011 | 0.022 | 0.027 | 0.037 | 0.024 | 0.022 |
| rs136553 | T | C | 0.004 | 0.006 | 0.001 | 0.008 | 0.000 | 0.018 | 0.005 | 0.015 | -0.014 | 0.027 | -0.002 | 0.016 |
| rs7288455 | A | G | 0.001 | 0.006 | 0.005 | 0.008 | -0.018 | 0.019 | 0.004 | 0.016 | -0.025 | 0.027 | -0.007 | 0.016 |
| EA effect allele, ER estrogen receptor, OA other allele, se standard error, SNP single nucleotide polymorphism | | | | | | | | | | | | | | |

| **Table S5: Summary information on colorectal cancer for the leisure television watching SNPs used in the Mendelian randomisation analyses** | | | | | | | | | | | | |
| --- | --- | --- | --- | --- | --- | --- | --- | --- | --- | --- | --- | --- |
| SNP | ΕΑ | ΟΑ | beta_crc | se_crc | beta_crc_men | se_crc_men | beta_crc_women | se_crc_women | beta_colon | se_colon | beta_re | se_re |
| rs11810109 | A | T | 0.011 | 0.011 | 0.000 | 0.015 | 0.022 | 0.015 | 0.004 | 0.013 | 0.016 | 0.017 |
| rs112632642 | A | G | -0.003 | 0.020 | 0.008 | 0.029 | -0.011 | 0.028 | -0.009 | 0.024 | 0.065 | 0.032 |
| rs6670915 | C | T | 0.005 | 0.010 | 0.011 | 0.014 | 0.001 | 0.014 | 0.000 | 0.012 | 0.012 | 0.015 |
| rs12725114 | G | A | -0.016 | 0.012 | -0.023 | 0.017 | -0.007 | 0.017 | 0.003 | 0.014 | -0.060 | 0.018 |
| rs984409 | A | G | 0.019 | 0.010 | 0.010 | 0.015 | 0.028 | 0.015 | 0.011 | 0.012 | 0.030 | 0.016 |
| rs10890123 | C | T | 0.007 | 0.012 | -0.004 | 0.017 | 0.015 | 0.017 | -0.004 | 0.014 | 0.022 | 0.019 |
| rs71658797 | A | T | 0.005 | 0.016 | 0.002 | 0.023 | 0.009 | 0.023 | 0.009 | 0.019 | 0.009 | 0.025 |
| rs114328297 | T | G | 0.002 | 0.012 | 0.018 | 0.017 | -0.018 | 0.018 | 0.016 | 0.015 | -0.056 | 0.019 |
| rs631130 | A | T | 0.000 | 0.011 | -0.020 | 0.015 | 0.020 | 0.015 | 0.011 | 0.013 | -0.014 | 0.017 |
| rs148648822 | T | G | 0.050 | 0.013 | 0.046 | 0.019 | 0.054 | 0.019 | 0.049 | 0.017 | 0.042 | 0.022 |
| rs17379561 | T | A | -0.016 | 0.014 | -0.004 | 0.020 | -0.024 | 0.020 | -0.016 | 0.017 | -0.016 | 0.022 |
| rs55909997 | A | G | 0.027 | 0.010 | 0.037 | 0.014 | 0.017 | 0.014 | 0.011 | 0.012 | 0.033 | 0.016 |
| rs1278847 | C | A | -0.006 | 0.011 | 0.008 | 0.015 | -0.018 | 0.015 | -0.006 | 0.013 | -0.004 | 0.017 |
| rs141425667 | A | G | 0.018 | 0.021 | 0.020 | 0.029 | 0.020 | 0.030 | 0.037 | 0.025 | 0.000 | 0.032 |
| rs4845364 | G | A | 0.006 | 0.010 | 0.014 | 0.014 | -0.001 | 0.014 | 0.008 | 0.012 | 0.013 | 0.015 |
| rs6673341 | G | T | 0.000 | 0.010 | 0.012 | 0.014 | -0.012 | 0.014 | -0.002 | 0.012 | 0.014 | 0.015 |
| rs72725224 | C | A | 0.075 | 0.038 | 0.044 | 0.053 | 0.098 | 0.055 | 0.056 | 0.045 | 0.106 | 0.057 |
| rs10737620 | T | A | -0.010 | 0.011 | -0.008 | 0.015 | -0.011 | 0.015 | 0.000 | 0.013 | -0.024 | 0.017 |
| rs11896615 | G | A | 0.020 | 0.014 | 0.011 | 0.020 | 0.029 | 0.020 | 0.005 | 0.016 | 0.037 | 0.022 |
| rs72781699 | A | G | -0.009 | 0.012 | -0.008 | 0.017 | -0.008 | 0.017 | 0.012 | 0.014 | -0.042 | 0.019 |
| rs12105701 | T | C | 0.009 | 0.010 | -0.013 | 0.014 | 0.030 | 0.014 | 0.007 | 0.012 | 0.017 | 0.015 |
| rs7564130 | C | T | 0.021 | 0.010 | 0.019 | 0.014 | 0.022 | 0.014 | 0.032 | 0.012 | 0.002 | 0.016 |
| rs11125515 | A | C | 0.005 | 0.011 | 0.001 | 0.015 | 0.008 | 0.015 | 0.006 | 0.013 | 0.019 | 0.017 |
| rs12475168 | T | G | -0.012 | 0.013 | -0.021 | 0.019 | -0.003 | 0.019 | -0.012 | 0.016 | 0.006 | 0.021 |
| rs183566425 | A | T | -0.031 | 0.043 | -0.043 | 0.062 | -0.015 | 0.061 | -0.060 | 0.050 | 0.153 | 0.071 |
| rs10189857 | G | A | 0.022 | 0.010 | 0.022 | 0.014 | 0.021 | 0.014 | 0.020 | 0.012 | 0.019 | 0.015 |
| rs62641636 | A | G | 0.010 | 0.010 | 0.000 | 0.015 | 0.018 | 0.015 | 0.022 | 0.012 | 0.015 | 0.016 |
| rs59210467 | T | C | -0.006 | 0.011 | -0.001 | 0.015 | -0.012 | 0.015 | -0.007 | 0.013 | 0.010 | 0.017 |
| rs11689199 | A | G | 0.025 | 0.010 | 0.039 | 0.014 | 0.012 | 0.014 | 0.020 | 0.012 | 0.034 | 0.016 |
| rs2916251 | T | G | 0.006 | 0.010 | 0.016 | 0.014 | -0.004 | 0.014 | 0.008 | 0.012 | 0.018 | 0.015 |
| rs1451533 | A | G | -0.009 | 0.011 | -0.009 | 0.015 | -0.011 | 0.015 | -0.002 | 0.013 | -0.013 | 0.017 |
| rs2971640 | A | G | 0.013 | 0.010 | 0.014 | 0.014 | 0.013 | 0.014 | 0.007 | 0.012 | 0.022 | 0.015 |
| rs12476388 | C | T | -0.002 | 0.010 | -0.005 | 0.015 | 0.000 | 0.015 | -0.012 | 0.013 | 0.001 | 0.017 |
| rs374722 | G | A | 0.026 | 0.013 | 0.002 | 0.019 | 0.049 | 0.019 | 0.038 | 0.016 | 0.017 | 0.021 |
| rs3754970 | C | T | -0.006 | 0.010 | -0.003 | 0.014 | -0.007 | 0.014 | -0.011 | 0.012 | 0.011 | 0.015 |
| rs1913808 | G | A | 0.014 | 0.010 | 0.025 | 0.015 | 0.005 | 0.015 | 0.003 | 0.013 | 0.022 | 0.017 |
| rs2060199 | T | A | 0.016 | 0.010 | 0.039 | 0.014 | -0.002 | 0.014 | 0.012 | 0.012 | 0.028 | 0.015 |
| rs263771 | A | C | 0.006 | 0.012 | 0.001 | 0.017 | 0.016 | 0.017 | 0.001 | 0.014 | 0.009 | 0.019 |
| rs4577309 | A | G | 0.001 | 0.010 | 0.015 | 0.014 | -0.012 | 0.014 | 0.004 | 0.012 | 0.009 | 0.015 |
| rs34224261 | C | G | -0.001 | 0.010 | 0.002 | 0.014 | -0.005 | 0.014 | 0.002 | 0.012 | 0.000 | 0.015 |
| rs4675246 | T | G | -0.037 | 0.013 | -0.043 | 0.018 | -0.031 | 0.018 | -0.028 | 0.015 | -0.047 | 0.020 |
| rs13029509 | A | G | 0.005 | 0.010 | 0.007 | 0.014 | 0.004 | 0.014 | 0.001 | 0.012 | 0.011 | 0.015 |
| rs10932837 | T | C | 0.003 | 0.010 | -0.013 | 0.014 | 0.020 | 0.014 | 0.005 | 0.012 | 0.016 | 0.015 |
| rs4973576 | A | C | -0.013 | 0.011 | 0.005 | 0.015 | -0.034 | 0.015 | -0.013 | 0.013 | 0.001 | 0.017 |
| rs138256022 | T | C | -0.043 | 0.031 | -0.025 | 0.045 | -0.065 | 0.044 | -0.031 | 0.037 | -0.046 | 0.049 |
| rs9834970 | T | C | 0.013 | 0.010 | 0.002 | 0.014 | 0.024 | 0.014 | 0.010 | 0.012 | 0.017 | 0.015 |
| rs1352890 | T | C | -0.006 | 0.010 | 0.007 | 0.014 | -0.014 | 0.014 | -0.016 | 0.012 | 0.029 | 0.015 |
| rs3796386 | A | G | 0.015 | 0.010 | 0.014 | 0.014 | 0.018 | 0.014 | 0.012 | 0.012 | 0.040 | 0.015 |
| rs182355396 | A | C | -0.002 | 0.061 | 0.094 | 0.087 | -0.098 | 0.084 | 0.047 | 0.072 | -0.052 | 0.097 |
| rs11917871 | C | T | -0.012 | 0.010 | -0.013 | 0.014 | -0.006 | 0.014 | -0.012 | 0.012 | 0.005 | 0.016 |
| rs11130793 | C | T | 0.008 | 0.010 | 0.018 | 0.014 | -0.003 | 0.014 | -0.004 | 0.012 | 0.011 | 0.016 |
| rs11714337 | G | A | -0.021 | 0.010 | -0.010 | 0.014 | -0.029 | 0.014 | -0.029 | 0.012 | -0.011 | 0.015 |
| rs12496084 | G | A | 0.007 | 0.011 | 0.010 | 0.016 | 0.006 | 0.016 | 0.003 | 0.014 | 0.006 | 0.018 |
| rs6797840 | C | A | -0.009 | 0.010 | 0.004 | 0.014 | -0.018 | 0.014 | -0.016 | 0.012 | -0.015 | 0.015 |
| rs56398417 | C | T | 0.015 | 0.011 | 0.028 | 0.015 | 0.001 | 0.015 | 0.011 | 0.013 | 0.013 | 0.017 |
| rs2034768 | A | G | -0.013 | 0.010 | -0.017 | 0.014 | -0.007 | 0.014 | -0.014 | 0.012 | -0.007 | 0.015 |
| rs12632778 | G | T | -0.029 | 0.021 | -0.045 | 0.030 | -0.021 | 0.030 | -0.035 | 0.028 | 0.026 | 0.036 |
| rs9867121 | C | A | 0.020 | 0.012 | 0.041 | 0.017 | -0.004 | 0.017 | 0.018 | 0.015 | 0.014 | 0.019 |
| rs12491503 | A | G | 0.001 | 0.010 | 0.011 | 0.014 | -0.008 | 0.014 | 0.005 | 0.012 | 0.006 | 0.016 |
| rs114600294 | C | G | -0.013 | 0.014 | -0.022 | 0.020 | -0.005 | 0.020 | -0.021 | 0.017 | -0.007 | 0.022 |
| rs66852340 | T | C | 0.004 | 0.011 | 0.013 | 0.016 | -0.002 | 0.016 | 0.000 | 0.014 | 0.030 | 0.018 |
| rs7700107 | C | A | 0.003 | 0.014 | 0.025 | 0.019 | -0.017 | 0.019 | -0.018 | 0.017 | 0.011 | 0.022 |
| rs34811474 | G | A | 0.022 | 0.013 | 0.009 | 0.019 | 0.035 | 0.019 | 0.038 | 0.016 | 0.016 | 0.021 |
| rs77215114 | A | T | 0.011 | 0.019 | -0.017 | 0.027 | 0.036 | 0.027 | 0.031 | 0.024 | 0.017 | 0.032 |
| rs28971796 | A | G | 0.010 | 0.012 | 0.004 | 0.017 | 0.018 | 0.017 | 0.003 | 0.015 | 0.037 | 0.019 |
| rs6850494 | C | A | 0.011 | 0.010 | 0.019 | 0.014 | 0.002 | 0.014 | 0.029 | 0.012 | 0.008 | 0.016 |
| rs13107325 | T | C | -0.008 | 0.019 | 0.009 | 0.026 | -0.028 | 0.027 | -0.012 | 0.022 | 0.006 | 0.029 |
| rs62332760 | T | G | 0.031 | 0.010 | 0.032 | 0.014 | 0.029 | 0.014 | 0.031 | 0.012 | 0.026 | 0.016 |
| rs7693703 | G | A | 0.022 | 0.017 | -0.004 | 0.024 | 0.050 | 0.024 | 0.018 | 0.020 | 0.070 | 0.027 |
| rs4334769 | G | T | -0.015 | 0.010 | -0.010 | 0.014 | -0.016 | 0.014 | -0.023 | 0.012 | -0.015 | 0.015 |
| rs6825241 | A | C | 0.008 | 0.010 | 0.003 | 0.014 | 0.012 | 0.014 | 0.005 | 0.012 | 0.016 | 0.015 |
| rs7693082 | G | C | 0.017 | 0.011 | 0.012 | 0.015 | 0.020 | 0.015 | 0.026 | 0.013 | 0.020 | 0.017 |
| rs10940659 | G | A | 0.011 | 0.010 | 0.027 | 0.014 | -0.003 | 0.014 | 0.011 | 0.012 | 0.020 | 0.015 |
| rs2409838 | A | T | 0.002 | 0.010 | -0.007 | 0.014 | 0.010 | 0.014 | 0.006 | 0.012 | -0.010 | 0.015 |
| rs262890 | G | A | -0.031 | 0.011 | -0.036 | 0.015 | -0.028 | 0.015 | -0.028 | 0.013 | -0.043 | 0.017 |
| rs7716447 | G | A | 0.016 | 0.010 | 0.018 | 0.014 | 0.009 | 0.014 | 0.007 | 0.012 | 0.021 | 0.016 |
| rs1031423 | C | T | -0.020 | 0.012 | -0.023 | 0.017 | -0.017 | 0.017 | -0.022 | 0.014 | -0.017 | 0.019 |
| rs57585211 | G | T | 0.003 | 0.013 | 0.020 | 0.018 | -0.012 | 0.018 | 0.008 | 0.016 | 0.009 | 0.021 |
| rs9326862 | A | T | 0.001 | 0.010 | -0.011 | 0.014 | 0.014 | 0.014 | 0.006 | 0.012 | -0.010 | 0.015 |
| rs10041724 | T | C | 0.015 | 0.012 | 0.021 | 0.017 | 0.010 | 0.017 | 0.016 | 0.014 | 0.012 | 0.019 |
| rs10057545 | A | G | 0.013 | 0.010 | 0.021 | 0.014 | 0.005 | 0.014 | 0.013 | 0.012 | 0.003 | 0.015 |
| rs62379379 | T | G | 0.032 | 0.019 | 0.025 | 0.027 | 0.038 | 0.026 | 0.029 | 0.023 | 0.022 | 0.031 |
| rs10054327 | G | A | -0.002 | 0.010 | 0.003 | 0.014 | -0.005 | 0.014 | -0.013 | 0.012 | -0.002 | 0.016 |
| rs42210 | C | G | 0.007 | 0.011 | 0.016 | 0.015 | -0.004 | 0.015 | -0.004 | 0.013 | 0.024 | 0.017 |
| rs2336895 | G | A | 0.008 | 0.010 | 0.020 | 0.014 | -0.003 | 0.014 | 0.016 | 0.012 | 0.020 | 0.016 |
| rs72828890 | C | T | 0.012 | 0.016 | 0.026 | 0.023 | -0.002 | 0.023 | 0.017 | 0.019 | 0.003 | 0.025 |
| rs255292 | A | C | 0.006 | 0.010 | 0.015 | 0.014 | -0.002 | 0.014 | 0.012 | 0.012 | -0.004 | 0.015 |
| rs72834698 | G | A | 0.044 | 0.014 | 0.060 | 0.020 | 0.031 | 0.020 | 0.059 | 0.017 | 0.069 | 0.022 |
| rs3135044 | T | C | 0.014 | 0.010 | 0.018 | 0.014 | 0.009 | 0.014 | 0.020 | 0.012 | 0.011 | 0.016 |
| rs10947452 | T | C | 0.019 | 0.010 | 0.034 | 0.014 | 0.005 | 0.014 | 0.011 | 0.012 | 0.042 | 0.016 |
| rs9471333 | C | T | 0.010 | 0.010 | 0.020 | 0.014 | 0.001 | 0.014 | 0.007 | 0.012 | 0.018 | 0.015 |
| rs4523073 | G | A | -0.006 | 0.010 | -0.002 | 0.014 | -0.009 | 0.014 | -0.002 | 0.012 | -0.022 | 0.016 |
| rs6905544 | G | A | 0.029 | 0.010 | 0.037 | 0.014 | 0.023 | 0.014 | 0.024 | 0.012 | 0.035 | 0.015 |
| rs17789218 | T | C | 0.004 | 0.012 | 0.016 | 0.016 | -0.006 | 0.016 | -0.006 | 0.014 | 0.011 | 0.018 |
| rs78394231 | C | T | 0.024 | 0.016 | 0.010 | 0.023 | 0.040 | 0.023 | 0.034 | 0.019 | 0.056 | 0.025 |
| rs2184364 | A | G | 0.023 | 0.012 | 0.025 | 0.017 | 0.022 | 0.017 | 0.024 | 0.015 | 0.017 | 0.019 |
| rs9718104 | G | T | 0.002 | 0.022 | -0.002 | 0.032 | 0.013 | 0.031 | -0.009 | 0.027 | -0.005 | 0.036 |
| rs17568389 | T | A | 0.001 | 0.010 | 0.012 | 0.014 | -0.012 | 0.014 | 0.000 | 0.012 | 0.012 | 0.015 |
| rs10234444 | G | A | -0.017 | 0.013 | -0.018 | 0.018 | -0.018 | 0.018 | -0.023 | 0.016 | -0.004 | 0.021 |
| rs6973656 | G | A | -0.029 | 0.010 | -0.025 | 0.014 | -0.031 | 0.014 | -0.028 | 0.012 | -0.036 | 0.015 |
| rs973734 | C | A | 0.002 | 0.014 | -0.022 | 0.020 | 0.031 | 0.020 | 0.013 | 0.016 | -0.009 | 0.021 |
| rs3137 | G | A | 0.013 | 0.013 | -0.005 | 0.018 | 0.034 | 0.018 | 0.013 | 0.016 | -0.003 | 0.021 |
| rs2694151 | C | T | 0.011 | 0.020 | 0.008 | 0.028 | 0.016 | 0.028 | 0.003 | 0.023 | 0.047 | 0.030 |
| rs62471080 | C | G | 0.005 | 0.010 | 0.007 | 0.014 | 0.004 | 0.014 | -0.010 | 0.012 | 0.012 | 0.015 |
| rs7788008 | G | A | 0.001 | 0.010 | 0.001 | 0.014 | 0.002 | 0.014 | 0.005 | 0.012 | -0.027 | 0.015 |
| rs10246289 | A | G | 0.041 | 0.015 | 0.039 | 0.022 | 0.042 | 0.022 | 0.033 | 0.019 | 0.033 | 0.024 |
| rs11763734 | C | A | 0.001 | 0.010 | -0.001 | 0.014 | 0.005 | 0.014 | 0.002 | 0.012 | -0.005 | 0.015 |
| rs7834121 | T | G | -0.007 | 0.010 | -0.015 | 0.015 | 0.000 | 0.015 | 0.007 | 0.012 | -0.028 | 0.016 |
| rs1421334 | A | C | 0.017 | 0.010 | 0.017 | 0.014 | 0.018 | 0.014 | 0.022 | 0.012 | -0.005 | 0.015 |
| rs6996198 | T | C | 0.016 | 0.013 | 0.009 | 0.018 | 0.025 | 0.018 | 0.011 | 0.016 | 0.017 | 0.020 |
| rs6472942 | C | T | 0.000 | 0.010 | 0.006 | 0.014 | -0.004 | 0.014 | 0.001 | 0.012 | -0.015 | 0.015 |
| rs11985394 | T | A | 0.010 | 0.010 | 0.017 | 0.014 | -0.001 | 0.014 | 0.020 | 0.012 | 0.000 | 0.015 |
| rs72671494 | C | T | 0.006 | 0.015 | -0.007 | 0.022 | 0.018 | 0.022 | 0.011 | 0.018 | -0.011 | 0.024 |
| rs12541615 | C | T | 0.003 | 0.012 | -0.001 | 0.017 | 0.008 | 0.018 | 0.013 | 0.015 | -0.006 | 0.019 |
| rs2717559 | A | G | 0.000 | 0.010 | -0.018 | 0.014 | 0.018 | 0.014 | -0.006 | 0.012 | 0.003 | 0.015 |
| rs2616830 | G | A | 0.013 | 0.010 | 0.014 | 0.014 | 0.008 | 0.014 | 0.016 | 0.012 | 0.031 | 0.015 |
| rs2291681 | G | T | -0.003 | 0.010 | -0.004 | 0.014 | -0.003 | 0.014 | -0.002 | 0.012 | -0.010 | 0.015 |
| rs34864022 | G | A | -0.013 | 0.021 | -0.033 | 0.030 | 0.010 | 0.030 | -0.015 | 0.025 | 0.026 | 0.033 |
| rs12554512 | T | C | -0.002 | 0.010 | -0.001 | 0.014 | -0.002 | 0.014 | 0.011 | 0.012 | -0.005 | 0.016 |
| rs2791574 | G | T | 0.020 | 0.010 | 0.017 | 0.014 | 0.019 | 0.014 | 0.021 | 0.012 | 0.014 | 0.016 |
| rs870151 | A | T | -0.010 | 0.010 | -0.005 | 0.014 | -0.018 | 0.014 | -0.011 | 0.012 | -0.005 | 0.015 |
| rs2787374 | T | C | 0.012 | 0.010 | -0.002 | 0.014 | 0.025 | 0.014 | 0.005 | 0.012 | 0.028 | 0.015 |
| rs10739499 | C | G | -0.006 | 0.010 | 0.002 | 0.014 | -0.016 | 0.015 | 0.001 | 0.012 | 0.024 | 0.016 |
| rs7043521 | A | T | -0.021 | 0.010 | -0.014 | 0.014 | -0.027 | 0.014 | -0.021 | 0.012 | -0.043 | 0.015 |
| rs12342024 | T | G | 0.033 | 0.015 | 0.045 | 0.022 | 0.019 | 0.022 | 0.048 | 0.018 | 0.015 | 0.024 |
| rs4382592 | T | G | 0.009 | 0.010 | 0.013 | 0.015 | 0.005 | 0.015 | 0.006 | 0.013 | 0.000 | 0.016 |
| rs2073869 | C | T | -0.011 | 0.013 | -0.002 | 0.018 | -0.018 | 0.018 | -0.018 | 0.015 | -0.019 | 0.020 |
| rs1623025 | C | G | -0.018 | 0.010 | -0.019 | 0.014 | -0.017 | 0.014 | -0.019 | 0.012 | -0.006 | 0.015 |
| rs1243182 | T | C | 0.029 | 0.011 | 0.039 | 0.015 | 0.020 | 0.015 | 0.025 | 0.013 | 0.054 | 0.016 |
| rs2045147 | A | G | 0.009 | 0.010 | 0.018 | 0.014 | -0.001 | 0.014 | 0.006 | 0.012 | 0.019 | 0.015 |
| rs10994943 | T | G | 0.007 | 0.010 | 0.017 | 0.014 | -0.006 | 0.014 | 0.000 | 0.012 | 0.014 | 0.015 |
| rs11201422 | T | C | 0.019 | 0.011 | 0.005 | 0.015 | 0.036 | 0.015 | 0.022 | 0.012 | 0.027 | 0.016 |
| rs10786658 | T | A | -0.003 | 0.010 | -0.011 | 0.014 | 0.007 | 0.014 | -0.002 | 0.012 | 0.001 | 0.015 |
| rs7089973 | A | C | 0.021 | 0.010 | 0.022 | 0.015 | 0.020 | 0.014 | 0.028 | 0.012 | 0.036 | 0.016 |
| rs11245482 | C | T | 0.004 | 0.010 | -0.006 | 0.014 | 0.016 | 0.014 | 0.002 | 0.012 | 0.008 | 0.015 |
| rs17727474 | C | T | 0.001 | 0.013 | -0.001 | 0.019 | 0.005 | 0.019 | -0.009 | 0.016 | 0.010 | 0.021 |
| rs12289262 | T | C | 0.028 | 0.011 | 0.029 | 0.016 | 0.029 | 0.016 | 0.030 | 0.014 | 0.010 | 0.018 |
| rs801733 | A | C | 0.027 | 0.010 | 0.028 | 0.014 | 0.026 | 0.014 | 0.023 | 0.012 | 0.048 | 0.016 |
| rs116175338 | T | G | 0.001 | 0.011 | -0.011 | 0.016 | 0.011 | 0.016 | 0.002 | 0.014 | -0.020 | 0.018 |
| rs12272012 | C | G | -0.031 | 0.024 | 0.000 | 0.034 | -0.062 | 0.033 | -0.036 | 0.028 | -0.064 | 0.037 |
| rs11020045 | C | A | -0.007 | 0.010 | -0.021 | 0.015 | 0.006 | 0.015 | -0.018 | 0.012 | 0.003 | 0.016 |
| rs17207890 | G | A | 0.000 | 0.010 | 0.012 | 0.015 | -0.010 | 0.015 | 0.016 | 0.012 | -0.021 | 0.016 |
| rs10791546 | C | T | 0.018 | 0.010 | 0.023 | 0.014 | 0.020 | 0.014 | 0.025 | 0.012 | 0.002 | 0.015 |
| rs328277 | C | A | 0.000 | 0.010 | -0.004 | 0.014 | 0.003 | 0.014 | 0.005 | 0.012 | -0.017 | 0.016 |
| rs648044 | G | A | -0.002 | 0.011 | 0.003 | 0.015 | -0.008 | 0.015 | -0.002 | 0.013 | 0.016 | 0.017 |
| rs11218575 | C | T | 0.001 | 0.010 | 0.013 | 0.014 | -0.010 | 0.014 | 0.001 | 0.012 | -0.001 | 0.015 |
| rs74802478 | G | A | -0.028 | 0.013 | -0.029 | 0.018 | -0.029 | 0.018 | -0.017 | 0.015 | -0.052 | 0.020 |
| rs4937842 | C | G | 0.012 | 0.010 | 0.018 | 0.014 | 0.008 | 0.014 | 0.018 | 0.012 | 0.023 | 0.016 |
| rs10772643 | C | T | 0.027 | 0.016 | 0.041 | 0.022 | 0.014 | 0.022 | 0.024 | 0.019 | 0.056 | 0.024 |
| rs74996610 | G | C | -0.013 | 0.024 | -0.004 | 0.034 | -0.020 | 0.034 | 0.001 | 0.030 | -0.019 | 0.039 |
| rs10771746 | T | C | -0.006 | 0.010 | 0.008 | 0.015 | -0.017 | 0.015 | -0.001 | 0.013 | -0.031 | 0.017 |
| rs10876864 | A | G | 0.010 | 0.010 | 0.005 | 0.014 | 0.018 | 0.014 | 0.029 | 0.012 | -0.021 | 0.015 |
| rs8756 | A | C | 0.009 | 0.010 | 0.008 | 0.014 | 0.009 | 0.014 | 0.005 | 0.012 | 0.016 | 0.015 |
| rs11179838 | C | G | 0.006 | 0.010 | -0.012 | 0.014 | 0.023 | 0.014 | 0.019 | 0.012 | 0.006 | 0.016 |
| rs2164744 | C | T | -0.014 | 0.010 | -0.013 | 0.014 | -0.015 | 0.014 | -0.007 | 0.012 | -0.027 | 0.015 |
| rs2173650 | G | T | -0.006 | 0.013 | 0.016 | 0.019 | -0.028 | 0.019 | -0.002 | 0.016 | 0.010 | 0.021 |
| rs1790135 | T | C | 0.030 | 0.011 | 0.022 | 0.016 | 0.037 | 0.016 | 0.039 | 0.013 | 0.034 | 0.017 |
| rs9563168 | G | A | 0.006 | 0.012 | -0.004 | 0.017 | 0.016 | 0.017 | 0.010 | 0.014 | -0.013 | 0.018 |
| rs9569734 | A | G | 0.010 | 0.013 | 0.024 | 0.019 | -0.011 | 0.019 | 0.012 | 0.016 | 0.037 | 0.021 |
| rs9528023 | C | T | 0.005 | 0.011 | 0.013 | 0.016 | -0.005 | 0.016 | 0.018 | 0.013 | 0.000 | 0.017 |
| rs56858768 | A | G | 0.010 | 0.011 | 0.006 | 0.015 | 0.015 | 0.015 | 0.012 | 0.013 | 0.019 | 0.017 |
| rs553397860 | A | G | 0.004 | 0.014 | 0.004 | 0.019 | 0.008 | 0.019 | 0.002 | 0.016 | 0.020 | 0.021 |
| rs7991062 | G | C | 0.013 | 0.010 | 0.011 | 0.014 | 0.015 | 0.014 | 0.012 | 0.012 | 0.017 | 0.016 |
| rs11629120 | C | T | 0.027 | 0.012 | 0.027 | 0.014 | 0.044 | 0.014 | 0.038 | 0.012 | 0.037 | 0.015 |
| rs405797 | A | T | 0.014 | 0.013 | 0.009 | 0.016 | 0.023 | 0.016 | 0.017 | 0.013 | 0.006 | 0.017 |
| rs1022785 | G | A | -0.003 | 0.016 | 0.027 | 0.019 | -0.024 | 0.019 | -0.005 | 0.016 | 0.035 | 0.021 |
| rs10145592 | G | C | 0.023 | 0.012 | 0.017 | 0.014 | 0.014 | 0.014 | 0.019 | 0.012 | 0.029 | 0.015 |
| rs7157001 | G | A | 0.007 | 0.014 | 0.005 | 0.017 | 0.013 | 0.017 | 0.003 | 0.014 | 0.051 | 0.019 |
| rs8043253 | T | C | -0.017 | 0.010 | -0.013 | 0.014 | -0.019 | 0.014 | -0.028 | 0.012 | 0.002 | 0.015 |
| rs2460 | A | G | 0.015 | 0.011 | -0.004 | 0.016 | 0.032 | 0.016 | 0.021 | 0.013 | -0.015 | 0.018 |
| rs4775373 | T | C | 0.006 | 0.010 | 0.002 | 0.014 | 0.009 | 0.014 | 0.007 | 0.012 | 0.017 | 0.016 |
| rs61331678 | G | C | -0.011 | 0.010 | 0.004 | 0.014 | -0.025 | 0.014 | -0.015 | 0.012 | -0.011 | 0.015 |
| rs35574015 | C | T | -0.020 | 0.010 | -0.028 | 0.015 | -0.011 | 0.015 | -0.018 | 0.013 | 0.001 | 0.016 |
| rs7189927 | T | C | 0.009 | 0.010 | 0.006 | 0.015 | 0.012 | 0.015 | 0.016 | 0.012 | 0.009 | 0.016 |
| rs749671 | G | A | 0.023 | 0.010 | 0.018 | 0.014 | 0.028 | 0.014 | 0.023 | 0.012 | 0.015 | 0.016 |
| rs7184800 | G | A | 0.005 | 0.011 | -0.003 | 0.015 | 0.011 | 0.015 | 0.003 | 0.013 | 0.024 | 0.016 |
| rs2447098 | A | C | 0.011 | 0.010 | 0.034 | 0.014 | -0.009 | 0.014 | 0.010 | 0.012 | 0.003 | 0.015 |
| rs11654952 | G | T | -0.025 | 0.014 | -0.005 | 0.019 | -0.047 | 0.020 | -0.018 | 0.017 | -0.001 | 0.022 |
| rs9902312 | T | C | -0.001 | 0.010 | -0.005 | 0.015 | 0.004 | 0.015 | 0.000 | 0.012 | 0.010 | 0.016 |
| rs11657730 | C | T | 0.010 | 0.011 | 0.007 | 0.015 | 0.012 | 0.015 | 0.010 | 0.012 | 0.016 | 0.016 |
| rs303753 | A | G | 0.013 | 0.010 | 0.029 | 0.015 | -0.001 | 0.015 | 0.008 | 0.012 | 0.022 | 0.016 |
| rs11081851 | A | G | 0.001 | 0.010 | 0.007 | 0.014 | -0.004 | 0.014 | 0.005 | 0.012 | -0.012 | 0.015 |
| rs9964724 | C | T | 0.009 | 0.010 | 0.014 | 0.015 | 0.004 | 0.015 | 0.011 | 0.012 | 0.012 | 0.016 |
| rs1156541 | C | T | 0.017 | 0.011 | 0.016 | 0.016 | 0.019 | 0.016 | 0.015 | 0.014 | 0.043 | 0.018 |
| rs148544378 | C | T | 0.046 | 0.047 | 0.153 | 0.067 | -0.060 | 0.067 | 0.045 | 0.056 | 0.068 | 0.073 |
| rs17512836 | T | C | -0.014 | 0.028 | -0.018 | 0.040 | -0.009 | 0.040 | 0.019 | 0.033 | -0.075 | 0.043 |
| rs7248205 | C | T | -0.029 | 0.010 | -0.046 | 0.014 | -0.010 | 0.014 | -0.021 | 0.012 | -0.023 | 0.016 |
| rs111901094 | T | G | 0.011 | 0.013 | -0.022 | 0.018 | 0.044 | 0.018 | -0.003 | 0.016 | 0.022 | 0.020 |
| rs6131281 | C | T | 0.017 | 0.010 | 0.021 | 0.014 | 0.011 | 0.014 | 0.020 | 0.012 | 0.014 | 0.015 |
| rs6141814 | A | C | -0.013 | 0.010 | -0.020 | 0.014 | -0.004 | 0.014 | -0.015 | 0.012 | -0.003 | 0.016 |
| rs4810312 | G | C | -0.007 | 0.010 | -0.010 | 0.014 | -0.003 | 0.014 | -0.010 | 0.012 | 0.008 | 0.015 |
| rs6124476 | T | C | -0.005 | 0.010 | -0.015 | 0.014 | 0.003 | 0.014 | -0.013 | 0.012 | 0.024 | 0.015 |
| rs6031440 | A | T | -0.013 | 0.010 | -0.018 | 0.014 | -0.009 | 0.014 | -0.015 | 0.012 | 0.005 | 0.016 |
| rs55700114 | A | G | 0.001 | 0.011 | 0.006 | 0.015 | -0.006 | 0.015 | 0.002 | 0.013 | 0.006 | 0.016 |
| rs6125906 | A | C | -0.019 | 0.017 | -0.021 | 0.024 | -0.019 | 0.024 | -0.016 | 0.020 | -0.009 | 0.026 |
| rs56103247 | C | T | -0.077 | 0.024 | -0.075 | 0.035 | -0.080 | 0.034 | -0.074 | 0.029 | -0.074 | 0.038 |
| rs10427502 | G | A | 0.001 | 0.010 | 0.024 | 0.014 | -0.017 | 0.014 | -0.006 | 0.012 | 0.000 | 0.016 |
| rs7609 | A | T | 0.001 | 0.011 | -0.016 | 0.016 | 0.014 | 0.016 | 0.003 | 0.014 | -0.014 | 0.018 |
| rs2092829 | G | A | -0.023 | 0.011 | -0.027 | 0.015 | -0.016 | 0.015 | -0.026 | 0.013 | -0.029 | 0.017 |
| crc colorectal, EA effect allele, OA other allele, re rectal, se standard error, SNP single nucleotide polymorphism | | | | | | | | | | | | |

| **Table S6: Summary information on colorectal cancer for the leisure computer use SNPs used in the Mendelian randomisation analyses** | | | | | | | | | | | | |
| --- | --- | --- | --- | --- | --- | --- | --- | --- | --- | --- | --- | --- |
| SNP | ΕΑ | ΟΑ | beta_crc | se_crc | beta_crc_men | se_crc_men | beta_crc_women | se_crc_women | beta_colon | se_colon | beta_re | se_re |
| rs2748985 | C | T | -0.006 | 0.010 | -0.017 | 0.014 | 0.003 | 0.014 | -0.016 | 0.012 | 0.003 | 0.015 |
| rs12145677 | A | G | 0.006 | 0.011 | -0.012 | 0.015 | 0.023 | 0.015 | 0.007 | 0.013 | 0.004 | 0.017 |
| rs7526456 | C | T | 0.005 | 0.010 | 0.001 | 0.014 | 0.009 | 0.014 | 0.004 | 0.012 | 0.012 | 0.015 |
| rs10754920 | C | G | 0.002 | 0.014 | -0.012 | 0.020 | 0.011 | 0.020 | 0.007 | 0.018 | 0.011 | 0.023 |
| rs6744254 | T | C | -0.014 | 0.010 | -0.041 | 0.014 | 0.012 | 0.014 | -0.020 | 0.012 | -0.021 | 0.015 |
| rs11274218 | T | C | 0.007 | 0.010 | -0.005 | 0.014 | 0.016 | 0.014 | 0.009 | 0.012 | -0.001 | 0.015 |
| rs4592851 | T | G | -0.015 | 0.011 | -0.020 | 0.016 | -0.010 | 0.016 | -0.018 | 0.013 | -0.009 | 0.017 |
| rs10208088 | C | T | 0.002 | 0.010 | 0.007 | 0.014 | -0.006 | 0.014 | 0.003 | 0.012 | -0.019 | 0.015 |
| rs11708955 | C | T | 0.001 | 0.010 | -0.007 | 0.015 | 0.006 | 0.015 | 0.007 | 0.012 | -0.029 | 0.016 |
| rs6774533 | T | C | 0.004 | 0.011 | 0.008 | 0.015 | 0.001 | 0.015 | 0.009 | 0.013 | -0.009 | 0.017 |
| rs78082503 | C | G | 0.007 | 0.011 | 0.020 | 0.016 | -0.004 | 0.016 | 0.002 | 0.014 | -0.033 | 0.018 |
| rs6857629 | G | A | 0.021 | 0.011 | 0.034 | 0.016 | 0.012 | 0.016 | 0.014 | 0.013 | 0.026 | 0.017 |
| rs2068625 | C | T | -0.017 | 0.011 | -0.011 | 0.015 | -0.020 | 0.015 | -0.025 | 0.013 | -0.020 | 0.017 |
| rs2220599 | G | C | 0.003 | 0.010 | 0.010 | 0.014 | 0.000 | 0.014 | -0.001 | 0.012 | 0.002 | 0.016 |
| rs11749912 | A | G | 0.015 | 0.010 | 0.010 | 0.014 | 0.023 | 0.014 | 0.019 | 0.012 | -0.001 | 0.015 |
| rs1469249 | G | A | 0.009 | 0.012 | 0.017 | 0.017 | 0.005 | 0.017 | -0.001 | 0.014 | 0.038 | 0.018 |
| rs162894 | T | G | -0.012 | 0.010 | -0.017 | 0.014 | -0.007 | 0.014 | -0.018 | 0.012 | -0.012 | 0.016 |
| rs56158102 | C | T | -0.006 | 0.010 | 0.003 | 0.014 | -0.014 | 0.014 | -0.001 | 0.012 | -0.001 | 0.016 |
| rs9477970 | A | T | 0.017 | 0.012 | 0.032 | 0.018 | 0.004 | 0.018 | 0.016 | 0.015 | 0.005 | 0.020 |
| rs2179517 | C | G | -0.020 | 0.010 | -0.024 | 0.014 | -0.014 | 0.014 | -0.030 | 0.012 | -0.016 | 0.015 |
| rs9372625 | A | G | -0.031 | 0.010 | -0.033 | 0.014 | -0.030 | 0.014 | -0.026 | 0.012 | -0.041 | 0.016 |
| rs17789218 | C | T | -0.004 | 0.012 | -0.016 | 0.016 | 0.006 | 0.016 | 0.006 | 0.014 | -0.011 | 0.018 |
| rs6935828 | T | C | 0.002 | 0.010 | -0.005 | 0.014 | 0.004 | 0.014 | -0.002 | 0.012 | -0.022 | 0.015 |
| rs55772938 | G | A | 0.008 | 0.011 | -0.001 | 0.015 | 0.017 | 0.015 | 0.001 | 0.013 | 0.006 | 0.016 |
| rs12706626 | A | G | 0.007 | 0.010 | 0.029 | 0.014 | -0.016 | 0.014 | 0.007 | 0.012 | -0.003 | 0.016 |
| rs3944151 | A | G | 0.021 | 0.011 | 0.020 | 0.015 | 0.019 | 0.015 | 0.029 | 0.013 | 0.016 | 0.017 |
| rs2345941 | A | G | -0.023 | 0.010 | -0.013 | 0.014 | -0.033 | 0.014 | -0.025 | 0.012 | -0.012 | 0.015 |
| rs13262595 | G | A | 0.001 | 0.010 | -0.004 | 0.014 | 0.008 | 0.014 | 0.007 | 0.012 | -0.012 | 0.015 |
| rs4977839 | A | G | 0.004 | 0.010 | 0.002 | 0.014 | 0.005 | 0.014 | -0.010 | 0.012 | 0.006 | 0.016 |
| rs113851275 | A | G | -0.022 | 0.015 | -0.001 | 0.022 | -0.043 | 0.022 | -0.010 | 0.019 | 0.001 | 0.024 |
| rs7020477 | A | G | 0.016 | 0.012 | -0.004 | 0.017 | 0.033 | 0.017 | 0.020 | 0.014 | 0.011 | 0.018 |
| rs73578186 | C | T | -0.015 | 0.010 | -0.022 | 0.014 | -0.006 | 0.015 | -0.018 | 0.012 | -0.003 | 0.016 |
| rs2734849 | G | A | 0.003 | 0.010 | -0.004 | 0.014 | 0.010 | 0.014 | 0.009 | 0.012 | -0.012 | 0.015 |
| rs1448355 | T | C | -0.012 | 0.010 | 0.008 | 0.014 | -0.030 | 0.014 | -0.004 | 0.012 | -0.013 | 0.016 |
| rs35933007 | A | G | 0.009 | 0.013 | 0.005 | 0.019 | 0.017 | 0.019 | 0.011 | 0.016 | 0.002 | 0.021 |
| rs1319734 | A | G | 0.014 | 0.010 | 0.008 | 0.014 | 0.021 | 0.014 | 0.016 | 0.012 | 0.002 | 0.016 |
| rs206965 | T | C | -0.008 | 0.012 | -0.014 | 0.017 | 0.001 | 0.017 | -0.001 | 0.014 | -0.029 | 0.019 |
| rs12874776 | A | C | 0.010 | 0.016 | 0.008 | 0.023 | 0.009 | 0.022 | 0.008 | 0.019 | 0.012 | 0.025 |
| rs198262 | C | T | -0.023 | 0.030 | 0.005 | 0.035 | -0.003 | 0.034 | 0.015 | 0.029 | -0.038 | 0.038 |
| rs166835 | C | T | -0.007 | 0.010 | -0.013 | 0.014 | 0.002 | 0.014 | -0.001 | 0.012 | -0.012 | 0.015 |
| rs4702 | G | A | 0.018 | 0.010 | 0.022 | 0.014 | 0.014 | 0.014 | 0.020 | 0.012 | 0.013 | 0.015 |
| rs6498759 | T | C | -0.003 | 0.010 | 0.001 | 0.014 | -0.009 | 0.014 | -0.004 | 0.012 | 0.000 | 0.016 |
| rs3730399 | A | G | -0.054 | 0.020 | -0.003 | 0.028 | -0.104 | 0.029 | -0.075 | 0.024 | -0.048 | 0.031 |
| rs4073003 | A | G | 0.009 | 0.015 | 0.016 | 0.021 | -0.001 | 0.021 | -0.002 | 0.018 | 0.013 | 0.023 |
| rs7209653 | T | C | -0.006 | 0.011 | -0.010 | 0.015 | 0.001 | 0.015 | -0.016 | 0.013 | 0.011 | 0.017 |
| rs12603813 | T | C | -0.003 | 0.011 | -0.024 | 0.016 | 0.018 | 0.016 | 0.012 | 0.013 | -0.019 | 0.017 |
| rs66643547 | T | C | -0.011 | 0.010 | -0.010 | 0.015 | -0.011 | 0.015 | -0.009 | 0.012 | -0.016 | 0.016 |
| rs784256 | G | A | 0.011 | 0.013 | 0.010 | 0.018 | 0.013 | 0.018 | 0.006 | 0.015 | 0.006 | 0.020 |
| rs6129084 | T | A | -0.015 | 0.010 | -0.019 | 0.014 | -0.009 | 0.014 | -0.003 | 0.012 | -0.051 | 0.016 |
| rs9608249 | G | A | 0.014 | 0.014 | -0.019 | 0.019 | 0.046 | 0.019 | 0.015 | 0.016 | 0.002 | 0.021 |
| rs136553 | T | C | -0.014 | 0.010 | -0.024 | 0.014 | -0.005 | 0.014 | -0.007 | 0.012 | -0.011 | 0.016 |
| rs7288455 | A | G | -0.002 | 0.010 | -0.004 | 0.014 | -0.004 | 0.014 | 0.006 | 0.012 | -0.010 | 0.016 |
| crc colorectal, EA effect allele, OA other allele, re rectal, se standard error, SNP single nucleotide polymorphism | | | | | | | | | | | | |

| **Table S7: Summary information on prostate cancer for the leisure television watching SNPs used in the Mendelian randomisation analyses** | | | | | | |
| --- | --- | --- | --- | --- | --- | --- |
| SNP | ΕΑ | ΟΑ | beta_prst | se_prst | beta_adv | se_adv |
| rs11810109 | A | T | -0.018 | 0.009 | -0.005 | 0.015 |
| rs112632642 | A | G | 0.022 | 0.016 | -0.005 | 0.028 |
| rs6670915 | C | T | -0.016 | 0.008 | -0.017 | 0.014 |
| rs12725114 | G | A | 0.011 | 0.010 | 0.025 | 0.017 |
| rs984409 | A | G | -0.008 | 0.008 | 0.009 | 0.014 |
| rs10890123 | C | T | 0.011 | 0.010 | 0.019 | 0.018 |
| rs71658797 | A | T | 0.008 | 0.013 | -0.001 | 0.022 |
| rs114328297 | T | G | -0.007 | 0.011 | -0.021 | 0.018 |
| rs631130 | A | T | 0.000 | 0.009 | -0.001 | 0.016 |
| rs148648822 | T | G | -0.012 | 0.012 | -0.007 | 0.020 |
| rs17379561 | T | A | -0.010 | 0.011 | -0.008 | 0.019 |
| rs55909997 | A | G | 0.003 | 0.009 | -0.002 | 0.015 |
| rs1278847 | C | A | -0.002 | 0.009 | -0.010 | 0.015 |
| rs141425667 | A | G | -0.028 | 0.018 | 0.013 | 0.031 |
| rs4845364 | G | A | 0.033 | 0.008 | 0.044 | 0.014 |
| rs6673341 | G | T | 0.004 | 0.008 | -0.010 | 0.014 |
| rs72725224 | C | A | -0.067 | 0.033 | -0.061 | 0.059 |
| rs10737620 | T | A | 0.004 | 0.009 | 0.014 | 0.016 |
| rs6721975 | C | T | NA | NA | -0.010 | 0.018 |
| rs11896615 | G | A | 0.002 | 0.012 | 0.022 | 0.020 |
| rs72781699 | A | G | -0.029 | 0.010 | -0.028 | 0.018 |
| rs12105701 | T | C | 0.001 | 0.008 | -0.017 | 0.014 |
| rs7564130 | C | T | -0.005 | 0.008 | -0.010 | 0.014 |
| rs11125515 | A | C | 0.001 | 0.009 | 0.001 | 0.016 |
| rs12475168 | T | G | 0.008 | 0.012 | -0.004 | 0.020 |
| rs183566425 | A | T | -0.044 | 0.042 | -0.015 | 0.074 |
| rs10189857 | G | A | -0.001 | 0.008 | 0.033 | 0.014 |
| rs62641636 | A | G | -0.006 | 0.009 | -0.008 | 0.015 |
| rs59210467 | T | C | 0.006 | 0.009 | 0.028 | 0.015 |
| rs11689199 | A | G | -0.004 | 0.009 | 0.001 | 0.015 |
| rs1451533 | A | G | -0.008 | 0.009 | 0.004 | 0.016 |
| rs2971640 | A | G | 0.003 | 0.008 | 0.007 | 0.014 |
| rs12476388 | C | T | 0.009 | 0.009 | -0.012 | 0.015 |
| rs374722 | G | A | 0.005 | 0.012 | -0.003 | 0.020 |
| rs3754970 | C | T | 0.001 | 0.008 | -0.006 | 0.014 |
| rs1913808 | G | A | 0.003 | 0.009 | -0.009 | 0.015 |
| rs2060199 | T | A | -0.010 | 0.008 | -0.013 | 0.014 |
| rs263771 | A | C | 0.009 | 0.010 | 0.006 | 0.017 |
| rs4577309 | A | G | NA | NA | -0.006 | 0.014 |
| rs34224261 | C | G | -0.002 | 0.008 | 0.015 | 0.014 |
| rs4675246 | T | G | -0.005 | 0.010 | -0.010 | 0.017 |
| rs13029509 | A | G | 0.000 | 0.008 | -0.001 | 0.014 |
| rs10932837 | T | C | 0.007 | 0.008 | 0.007 | 0.014 |
| rs4973576 | A | C | -0.021 | 0.009 | -0.011 | 0.015 |
| rs138256022 | T | C | 0.011 | 0.023 | 0.058 | 0.039 |
| rs9834970 | T | C | -0.005 | 0.009 | -0.010 | 0.015 |
| rs1352890 | T | C | -0.006 | 0.008 | -0.021 | 0.014 |
| rs3796386 | A | G | 0.027 | 0.008 | 0.025 | 0.014 |
| rs182355396 | A | C | 0.070 | 0.083 | -0.063 | 0.149 |
| rs11917871 | C | T | 0.011 | 0.009 | 0.005 | 0.015 |
| rs11130793 | C | T | 0.009 | 0.008 | 0.008 | 0.014 |
| rs11714337 | G | A | -0.016 | 0.008 | -0.033 | 0.014 |
| rs12496084 | G | A | -0.013 | 0.010 | -0.026 | 0.017 |
| rs6797840 | C | A | -0.007 | 0.008 | -0.023 | 0.014 |
| rs56398417 | C | T | -0.005 | 0.009 | 0.005 | 0.015 |
| rs2034768 | A | G | -0.007 | 0.008 | -0.008 | 0.014 |
| rs12632778 | G | T | -0.030 | 0.020 | -0.055 | 0.033 |
| rs9867121 | C | A | NA | NA | -0.007 | 0.018 |
| rs12491503 | A | G | NA | NA | 0.004 | 0.015 |
| rs114600294 | C | G | -0.002 | 0.013 | -0.008 | 0.022 |
| rs66852340 | T | C | -0.001 | 0.010 | 0.010 | 0.017 |
| rs7700107 | C | A | -0.004 | 0.012 | -0.010 | 0.021 |
| rs34811474 | G | A | 0.018 | 0.011 | 0.005 | 0.019 |
| rs77215114 | A | T | -0.027 | 0.017 | -0.024 | 0.030 |
| rs28971796 | A | G | -0.014 | 0.012 | -0.018 | 0.020 |
| rs6850494 | C | A | 0.011 | 0.008 | 0.007 | 0.014 |
| rs13107325 | T | C | -0.015 | 0.016 | -0.009 | 0.028 |
| rs62332760 | T | G | 0.053 | 0.008 | 0.041 | 0.014 |
| rs7693703 | G | A | 0.022 | 0.014 | 0.034 | 0.024 |
| rs4334769 | G | T | 0.013 | 0.008 | 0.007 | 0.014 |
| rs6825241 | A | C | -0.011 | 0.008 | -0.021 | 0.014 |
| rs7693082 | G | C | -0.004 | 0.009 | 0.035 | 0.015 |
| rs10940659 | G | A | -0.005 | 0.008 | -0.017 | 0.014 |
| rs2409838 | A | T | -0.013 | 0.008 | -0.009 | 0.014 |
| rs262890 | G | A | -0.002 | 0.009 | -0.011 | 0.015 |
| rs7716447 | G | A | -0.007 | 0.009 | 0.008 | 0.015 |
| rs1031423 | C | T | 0.008 | 0.010 | 0.013 | 0.018 |
| rs57585211 | G | T | -0.011 | 0.011 | -0.012 | 0.018 |
| rs9326862 | A | T | -0.002 | 0.008 | -0.008 | 0.014 |
| rs10041724 | T | C | 0.004 | 0.010 | 0.028 | 0.018 |
| rs10057545 | A | G | -0.008 | 0.009 | -0.011 | 0.015 |
| rs62379379 | T | G | -0.004 | 0.016 | -0.023 | 0.028 |
| rs10054327 | G | A | 0.008 | 0.008 | -0.003 | 0.014 |
| rs42210 | C | G | -0.010 | 0.010 | 0.008 | 0.016 |
| rs2336895 | G | A | -0.003 | 0.009 | 0.003 | 0.015 |
| rs72828890 | C | T | 0.023 | 0.014 | -0.019 | 0.024 |
| rs255292 | A | C | -0.003 | 0.008 | 0.012 | 0.014 |
| rs72834698 | G | A | -0.034 | 0.012 | -0.034 | 0.020 |
| rs3135044 | T | C | 0.022 | 0.008 | 0.023 | 0.014 |
| rs10947452 | T | C | 0.000 | 0.009 | 0.017 | 0.015 |
| rs9471333 | C | T | 0.002 | 0.009 | -0.023 | 0.015 |
| rs4523073 | G | A | -0.005 | 0.008 | -0.008 | 0.015 |
| rs77589760 | C | G | NA | NA | 0.001 | 0.053 |
| rs6905544 | G | A | 0.001 | 0.008 | 0.013 | 0.014 |
| rs17789218 | T | C | -0.008 | 0.011 | -0.019 | 0.018 |
| rs78394231 | C | T | 0.042 | 0.014 | 0.006 | 0.024 |
| rs2184364 | A | G | -0.020 | 0.010 | -0.013 | 0.017 |
| rs9718104 | G | T | 0.002 | 0.018 | -0.021 | 0.032 |
| rs17568389 | T | A | 0.005 | 0.008 | -0.006 | 0.014 |
| rs10234444 | G | A | 0.010 | 0.012 | 0.022 | 0.020 |
| rs6973656 | G | A | 0.005 | 0.009 | 0.014 | 0.015 |
| rs973734 | C | A | 0.014 | 0.011 | 0.013 | 0.020 |
| rs3137 | G | A | 0.002 | 0.011 | 0.007 | 0.019 |
| rs2694151 | C | T | 0.012 | 0.018 | -0.032 | 0.031 |
| rs62471080 | C | G | -0.003 | 0.008 | -0.001 | 0.014 |
| rs7788008 | G | A | -0.006 | 0.008 | -0.003 | 0.014 |
| rs10246289 | A | G | 0.002 | 0.013 | -0.004 | 0.021 |
| rs11763734 | C | A | 0.007 | 0.008 | -0.002 | 0.014 |
| rs7834121 | T | G | -0.017 | 0.008 | -0.030 | 0.014 |
| rs1421334 | A | C | 0.010 | 0.008 | 0.028 | 0.014 |
| rs6996198 | T | C | -0.011 | 0.011 | -0.009 | 0.019 |
| rs6472942 | C | T | -0.010 | 0.008 | -0.011 | 0.014 |
| rs11985394 | T | A | 0.000 | 0.008 | 0.014 | 0.014 |
| rs72671494 | C | T | 0.010 | 0.013 | 0.031 | 0.021 |
| rs12541615 | C | T | -0.033 | 0.011 | -0.050 | 0.018 |
| rs2717559 | A | G | NA | NA | -0.016 | 0.015 |
| rs2616830 | G | A | -0.004 | 0.008 | -0.002 | 0.014 |
| rs2291681 | G | T | 0.008 | 0.009 | 0.028 | 0.014 |
| rs34864022 | G | A | -0.024 | 0.017 | 0.024 | 0.028 |
| rs12554512 | T | C | -0.014 | 0.009 | -0.008 | 0.014 |
| rs2791574 | G | T | -0.001 | 0.009 | 0.010 | 0.015 |
| rs141184308 | A | G | NA | NA | 0.065 | 0.074 |
| rs870151 | A | T | -0.013 | 0.008 | -0.011 | 0.014 |
| rs2787374 | T | C | -0.015 | 0.008 | 0.003 | 0.014 |
| rs10739499 | C | G | -0.007 | 0.009 | -0.003 | 0.015 |
| rs7043521 | A | T | -0.004 | 0.008 | -0.016 | 0.014 |
| rs12342024 | T | G | -0.037 | 0.013 | -0.045 | 0.022 |
| rs4382592 | T | G | -0.013 | 0.009 | -0.027 | 0.015 |
| rs2073869 | C | T | -0.006 | 0.011 | -0.022 | 0.018 |
| 9:139926402_T_C | C | T | 0.004 | 0.008 | NA | NA |
| rs1623025 | C | G | 0.010 | 0.008 | 0.023 | 0.014 |
| rs1243182 | T | C | -0.010 | 0.009 | -0.008 | 0.015 |
| rs2045147 | A | G | -0.002 | 0.008 | 0.003 | 0.014 |
| rs10994943 | T | G | -0.017 | 0.008 | -0.022 | 0.014 |
| rs11201422 | T | C | 0.011 | 0.009 | 0.017 | 0.015 |
| rs10786658 | T | A | -0.006 | 0.008 | -0.017 | 0.014 |
| rs7089973 | A | C | -0.007 | 0.009 | -0.048 | 0.015 |
| rs11245482 | C | T | 0.023 | 0.008 | 0.021 | 0.014 |
| rs17727474 | C | T | 0.004 | 0.011 | 0.037 | 0.020 |
| rs12289262 | T | C | -0.015 | 0.009 | -0.011 | 0.016 |
| rs801733 | A | C | 0.002 | 0.009 | -0.004 | 0.015 |
| rs116175338 | T | G | NA | NA | 0.002 | 0.016 |
| rs12272012 | C | G | 0.011 | 0.020 | 0.006 | 0.034 |
| rs11020045 | C | A | 0.010 | 0.009 | -0.015 | 0.015 |
| rs17207890 | G | A | -0.001 | 0.009 | 0.018 | 0.015 |
| rs10791546 | C | T | 0.008 | 0.008 | -0.010 | 0.015 |
| rs113584870 | G | A | -0.028 | 0.012 | NA | NA |
| rs328277 | C | A | 0.002 | 0.008 | 0.030 | 0.014 |
| rs648044 | G | A | -0.003 | 0.009 | -0.010 | 0.016 |
| rs11218575 | C | T | 0.002 | 0.009 | 0.009 | 0.015 |
| rs74802478 | G | A | 0.002 | 0.011 | -0.041 | 0.018 |
| rs4937842 | C | G | -0.004 | 0.008 | -0.011 | 0.014 |
| rs10772643 | C | T | 0.029 | 0.013 | NA | NA |
| rs74996610 | G | C | 0.017 | 0.020 | NA | NA |
| rs10771746 | T | C | 0.021 | 0.009 | NA | NA |
| rs10876864 | A | G | -0.017 | 0.008 | -0.026 | 0.014 |
| rs8756 | A | C | 0.002 | 0.008 | 0.006 | 0.014 |
| rs11179838 | C | G | 0.004 | 0.008 | NA | NA |
| rs2164744 | C | T | -0.018 | 0.009 | NA | NA |
| rs2173650 | G | T | 0.010 | 0.012 | NA | NA |
| rs1790135 | T | C | -0.005 | 0.009 | NA | NA |
| rs9563168 | G | A | 0.007 | 0.009 | 0.030 | 0.017 |
| rs9569734 | A | G | 0.007 | 0.012 | 0.026 | 0.019 |
| rs9528023 | C | T | -0.012 | 0.010 | -0.017 | 0.017 |
| rs56858768 | A | G | 0.000 | 0.009 | -0.011 | 0.015 |
| rs553397860 | A | G | -0.010 | 0.012 | -0.010 | 0.020 |
| rs7991062 | G | C | -0.010 | 0.009 | -0.010 | 0.015 |
| rs11629120 | C | T | 0.024 | 0.008 | 0.035 | 0.014 |
| rs405797 | A | T | 0.008 | 0.010 | -0.007 | 0.018 |
| rs1022785 | G | A | -0.011 | 0.012 | -0.018 | 0.020 |
| rs10145592 | G | C | 0.006 | 0.009 | 0.020 | 0.015 |
| rs7157001 | G | A | NA | NA | -0.007 | 0.019 |
| rs8043253 | T | C | 0.000 | 0.008 | -0.015 | 0.014 |
| rs2460 | A | G | -0.020 | 0.010 | -0.039 | 0.017 |
| rs4775373 | T | C | 0.006 | 0.009 | 0.010 | 0.015 |
| rs61331678 | G | C | 0.012 | 0.008 | 0.023 | 0.014 |
| rs35574015 | C | T | -0.016 | 0.009 | -0.031 | 0.015 |
| rs7189927 | T | C | -0.003 | 0.009 | -0.013 | 0.015 |
| rs749671 | G | A | -0.012 | 0.008 | -0.015 | 0.014 |
| rs7184800 | G | A | -0.015 | 0.009 | -0.013 | 0.015 |
| rs142710267 | T | G | NA | NA | 0.024 | 0.015 |
| rs2447098 | A | C | 0.005 | 0.009 | 0.015 | 0.015 |
| rs11654952 | G | T | NA | NA | 0.011 | 0.020 |
| rs2584597 | T | C | 0.008 | 0.009 | -0.025 | 0.015 |
| rs9902312 | T | C | -0.003 | 0.009 | -0.009 | 0.015 |
| rs11657730 | C | T | 0.009 | 0.009 | -0.003 | 0.016 |
| rs303753 | A | G | 0.006 | 0.009 | 0.008 | 0.015 |
| rs71362609 | C | T | -0.013 | 0.008 | -0.029 | 0.014 |
| rs11081851 | A | G | -0.012 | 0.008 | 0.000 | 0.014 |
| rs9964724 | C | T | 0.003 | 0.009 | -0.023 | 0.015 |
| rs1156541 | C | T | 0.016 | 0.010 | 0.021 | 0.017 |
| rs148544378 | C | T | -0.003 | 0.043 | 0.079 | 0.072 |
| rs17512836 | T | C | 0.058 | 0.026 | 0.060 | 0.045 |
| rs7248205 | C | T | -0.002 | 0.009 | 0.009 | 0.015 |
| rs111901094 | T | G | 0.024 | 0.012 | 0.026 | 0.021 |
| rs6131281 | C | T | 0.009 | 0.009 | 0.019 | 0.015 |
| rs6141814 | A | C | -0.035 | 0.008 | -0.038 | 0.014 |
| rs4810312 | G | C | -0.010 | 0.008 | -0.032 | 0.014 |
| rs6124476 | T | C | 0.001 | 0.009 | 0.009 | 0.014 |
| rs6031440 | A | T | 0.006 | 0.009 | 0.016 | 0.015 |
| rs55700114 | A | G | -0.001 | 0.009 | 0.009 | 0.015 |
| rs6125906 | A | C | 0.024 | 0.015 | 0.054 | 0.025 |
| rs56103247 | C | T | 0.004 | 0.020 | 0.079 | 0.035 |
| rs10427502 | G | A | -0.004 | 0.009 | 0.017 | 0.015 |
| rs7609 | A | T | -0.020 | 0.010 | 0.015 | 0.017 |
| rs2092829 | G | A | -0.012 | 0.009 | -0.008 | 0.015 |
| rs150399729 | C | G | 0.026 | 0.025 | -0.006 | 0.040 |
| adv adcanced, EA effect allele, OA other allele, NA not available, prst prostate, se standard error, SNP single nucleotide polymorphism | | | | | | |

| **Table S8: Summary information on prostate cancer for the leisure computer use SNPs used in the Mendelian randomisation analyses** | | | | | | |
| --- | --- | --- | --- | --- | --- | --- |
| SNP | ΕΑ | ΟΑ | beta_prst | se_prst | beta_adv | se_adv |
| rs2748985 | C | T | -0.021 | 0.009 | -0.022 | 0.016 |
| rs12145677 | A | G | 0.000 | 0.009 | 0.007 | 0.015 |
| rs7526456 | C | T | 0.015 | 0.009 | -0.002 | 0.015 |
| rs10754920 | C | G | 0.003 | 0.013 | 0.003 | 0.022 |
| rs6744254 | T | C | -0.006 | 0.008 | -0.017 | 0.014 |
| rs11274218 | T | C | 0.002 | 0.008 | 0.000 | 0.015 |
| rs4592851 | T | G | 0.004 | 0.009 | 0.003 | 0.016 |
| rs10208088 | C | T | -0.003 | 0.008 | -0.005 | 0.014 |
| rs11708955 | C | T | -0.027 | 0.009 | -0.016 | 0.015 |
| rs6774533 | T | C | 0.000 | 0.009 | -0.008 | 0.016 |
| rs78082503 | C | G | 0.003 | 0.010 | -0.004 | 0.017 |
| rs6857629 | G | A | -0.008 | 0.010 | -0.023 | 0.016 |
| rs2068625 | C | T | -0.006 | 0.009 | -0.034 | 0.015 |
| rs2220599 | G | C | -0.011 | 0.009 | -0.017 | 0.015 |
| rs11749912 | A | G | -0.003 | 0.008 | -0.020 | 0.014 |
| rs1469249 | G | A | 0.009 | 0.010 | 0.015 | 0.017 |
| rs162894 | T | G | -0.013 | 0.008 | -0.005 | 0.014 |
| rs56158102 | C | T | NA | NA | 0.003 | 0.015 |
| rs9477970 | A | T | -0.004 | 0.010 | -0.017 | 0.018 |
| rs2179517 | C | G | 0.009 | 0.008 | 0.017 | 0.014 |
| rs9372625 | A | G | 0.003 | 0.009 | -0.012 | 0.015 |
| rs17789218 | C | T | 0.008 | 0.011 | 0.019 | 0.018 |
| rs6935828 | T | C | 0.015 | 0.008 | 0.009 | 0.014 |
| rs55772938 | G | A | 0.005 | 0.009 | 0.007 | 0.016 |
| rs12706626 | A | G | 0.009 | 0.008 | -0.015 | 0.014 |
| rs3944151 | A | G | 0.001 | 0.009 | -0.005 | 0.015 |
| rs2345941 | A | G | -0.008 | 0.008 | -0.028 | 0.014 |
| rs13262595 | G | A | 0.020 | 0.008 | 0.019 | 0.014 |
| rs4977839 | A | G | 0.014 | 0.009 | 0.010 | 0.014 |
| rs113851275 | A | G | -0.008 | 0.013 | -0.009 | 0.022 |
| rs7020477 | A | G | -0.007 | 0.010 | 0.000 | 0.017 |
| rs73578186 | C | T | 0.017 | 0.009 | 0.034 | 0.015 |
| rs2734849 | G | A | -0.001 | 0.008 | -0.001 | 0.014 |
| rs1448355 | T | C | -0.005 | 0.008 | -0.024 | 0.015 |
| rs35933007 | A | G | 0.003 | 0.011 | NA | NA |
| rs1319734 | A | G | -0.008 | 0.008 | NA | NA |
| rs206965 | T | C | -0.017 | 0.010 | -0.013 | 0.017 |
| rs12874776 | A | C | 0.009 | 0.014 | 0.021 | 0.024 |
| rs198262 | C | T | -0.020 | 0.021 | -0.030 | 0.035 |
| rs166835 | C | T | -0.002 | 0.008 | -0.012 | 0.014 |
| rs4702 | G | A | 0.018 | 0.008 | 0.025 | 0.014 |
| rs6498759 | T | C | 0.010 | 0.009 | -0.020 | 0.016 |
| rs3730399 | A | G | 0.031 | 0.016 | 0.048 | 0.027 |
| rs4073003 | A | G | 0.012 | 0.012 | 0.035 | 0.021 |
| rs7209653 | T | C | 0.002 | 0.009 | 0.014 | 0.016 |
| rs12603813 | T | C | 0.007 | 0.009 | 0.020 | 0.016 |
| rs66643547 | T | C | -0.007 | 0.009 | 0.002 | 0.016 |
| rs784256 | G | A | -0.018 | 0.011 | -0.046 | 0.018 |
| rs6129084 | T | A | 0.013 | 0.009 | 0.016 | 0.015 |
| rs9608249 | G | A | NA | NA | 0.006 | 0.020 |
| rs136553 | T | C | 0.015 | 0.008 | 0.015 | 0.014 |
| rs7288455 | A | G | 0.006 | 0.009 | 0.013 | 0.015 |
| adv adcanced, EA effect allele, OA other allele, NA not available, prst prostate, se standard error, SNP single nucleotide polymorphism | | | | | | |

| **Table S9: Sample size and power calculations in Mendelian randomization study of sedentary behaviour and cancer risk** | | | | | | | | | |
| --- | --- | --- | --- | --- | --- | --- | --- | --- | --- |
| **Exposure** | **Outcome** | **Sample size** | **Proportion of cases** | **Selected scenarios^*^** | | | |  | |
|  |  |  |  | **OR=1.05** | **OR=1.1** | **OR=1.15** | **OR=1.2** | **OR=1.25** |  |
| **Leisure television watching** | Breast cancer | 228,951 | 0.54 | 0.38 | 0.89 | 1 | 1 | 1 |  |
|  | Colorectal cancer | 98,715 | 0.53 | 0.19 | 0.56 | 0.87 | 0.98 | 1 |  |
|  | Prostate cancer | 140,254 | 0.56 | 0.25 | 0.7 | 0.95 | 1 | 1 |  |
|  |  |  |  |  |  |  |  |  |  |
| **Leisure computer use** | Breast cancer | 228,951 | 0.54 | 0.13 | 0.36 | 0.65 | 0.86 | 0.96 |  |
|  | Colorectal cancer | 98,715 | 0.53 | 0.08 | 0.18 | 0.34 | 0.52 | 0.69 |  |
|  | Prostate cancer | 140,254 | 0.56 | 0.1 | 0.24 | 0.45 | 0.66 | 0.83 |  |
|  |  |  |  |  |  |  |  |  |  |
| Abbreviations: OR, odds ratio  ^*^ Type 1 error of 5% and a proportion of variance explained equal to 2% and 0.5%, are assumed for leisure television watching and leisure computer use respectively | | | | | | | |  |  |

| **Table S10. Beta estimates for the SNPs included in the multivariable MR of alcohol consumption, computer use, and breast cancer risk** | | | | | | | | | | | | | | | | | | | | | | | |
| --- | --- | --- | --- | --- | --- | --- | --- | --- | --- | --- | --- | --- | --- | --- | --- | --- | --- | --- | --- | --- | --- | --- | --- |
| SNP | Chr | POS | EA | OA | beta_  alc | se_  alc | pval_  alc | beta_  pc | se_pc | pval_pc | phenotype | beta_br | se_br | Lum_A_  beta | Lum_A_  se | Lum_B_  beta | Lum_B_  se | HER2Neg_  beta | HER2Neg_  se | HER2_  Enriched_beta | HER2_  Enriched_se | 3N_beta | 3N_se |
| rs2748985 | 1 | 1853184 | T | C | 0.002 | 0.002 | 2.94E-01 | -0.015 | 0.002 | 4.10E-12 | PC | 0.003 | 0.007 | 0.007 | 0.009 | 0.017 | 0.019 | -0.002 | 0.017 | -0.011 | 0.028 | 0.014 | 0.017 |
| rs58107686 | 1 | 33837334 | A | C | -0.010 | 0.002 | 7.79E-10 | 0.000 | 0.002 | 9.40E-01 | Alcohol | -0.001 | 0.006 | -0.008 | 0.008 | -0.017 | 0.019 | 0.036 | 0.016 | -0.003 | 0.027 | 0.000 | 0.016 |
| rs12088813 | 1 | 66407700 | C | A | -0.009 | 0.002 | 1.58E-08 | 0.004 | 0.002 | 7.30E-02 | Alcohol | 0.018 | 0.007 | 0.025 | 0.009 | 0.018 | 0.020 | 0.040 | 0.017 | -0.028 | 0.030 | 0.029 | 0.018 |
| rs5024204 | 1 | 71491890 | T | A | 0.010 | 0.002 | 2.55E-09 | 0.002 | 0.002 | 5.10E-01 | Alcohol | 0.003 | 0.007 | 0.001 | 0.009 | 0.013 | 0.020 | -0.023 | 0.017 | 0.008 | 0.029 | 0.018 | 0.017 |
| rs12145677 | 1 | 110023610 | G | A | 0.005 | 0.002 | 2.43E-02 | -0.017 | 0.002 | 1.20E-12 | PC | -0.011 | 0.007 | -0.016 | 0.009 | -0.012 | 0.020 | -0.003 | 0.017 | -0.009 | 0.028 | 0.013 | 0.017 |
| rs10753661 | 1 | 165119792 | A | G | -0.009 | 0.002 | 3.76E-08 | 0.003 | 0.002 | 1.80E-01 | Alcohol | 0.010 | 0.007 | 0.010 | 0.009 | 0.011 | 0.019 | 0.006 | 0.017 | -0.031 | 0.028 | 0.024 | 0.017 |
| rs28680958 | 1 | 173848808 | A | G | -0.011 | 0.002 | 5.13E-10 | -0.005 | 0.003 | 4.50E-02 | Alcohol | -0.004 | 0.007 | 0.000 | 0.010 | 0.024 | 0.021 | -0.012 | 0.018 | 0.026 | 0.031 | -0.004 | 0.018 |
| rs7526456 | 1 | 197773734 | T | C | 0.000 | 0.002 | 9.48E-01 | -0.013 | 0.002 | 1.20E-08 | PC | 0.005 | 0.007 | 0.005 | 0.009 | 0.012 | 0.019 | 0.002 | 0.016 | -0.039 | 0.028 | 0.014 | 0.016 |
| rs823114 | 1 | 205719532 | A | G | 0.009 | 0.001 | 2.31E-09 | -0.005 | 0.002 | 1.70E-02 | Alcohol | -0.012 | 0.006 | -0.013 | 0.008 | -0.010 | 0.018 | 0.000 | 0.015 | 0.001 | 0.026 | -0.029 | 0.015 |
| rs77165542 | 2 | 430975 | T | C | -0.026 | 0.004 | 5.63E-11 | -0.013 | 0.006 | 3.60E-02 | Alcohol | 0.004 | 0.020 | 0.024 | 0.027 | -0.027 | 0.058 | 0.070 | 0.048 | -0.068 | 0.085 | -0.007 | 0.050 |
| rs2178197 | 2 | 27860551 | G | A | -0.009 | 0.001 | 2.45E-09 | -0.003 | 0.002 | 2.00E-01 | Alcohol | -0.006 | 0.006 | -0.009 | 0.008 | -0.003 | 0.018 | -0.007 | 0.015 | -0.012 | 0.026 | 0.011 | 0.015 |
| rs1004787 | 2 | 45159091 | A | G | 0.008 | 0.001 | 8.40E-09 | 0.002 | 0.002 | 3.90E-01 | Alcohol | -0.001 | 0.006 | -0.005 | 0.008 | 0.047 | 0.018 | -0.008 | 0.015 | 0.002 | 0.026 | 0.002 | 0.015 |
| rs13032049 | 2 | 63581507 | G | A | 0.010 | 0.002 | 3.00E-10 | 0.002 | 0.002 | 3.40E-01 | Alcohol | 0.007 | 0.007 | -0.005 | 0.009 | 0.012 | 0.019 | 0.015 | 0.017 | -0.025 | 0.028 | 0.049 | 0.017 |
| rs828867 | 2 | 74334462 | A | G | 0.009 | 0.001 | 2.15E-09 | 0.002 | 0.002 | 4.30E-01 | Alcohol | -0.003 | 0.007 | -0.007 | 0.009 | 0.036 | 0.019 | -0.053 | 0.017 | -0.020 | 0.028 | 0.000 | 0.017 |
| rs11692435 | 2 | 98275354 | A | G | 0.017 | 0.003 | 2.53E-11 | -0.010 | 0.004 | 1.70E-02 | Alcohol | -0.010 | 0.010 | 0.000 | 0.013 | -0.003 | 0.030 | -0.017 | 0.026 | 0.050 | 0.043 | -0.022 | 0.026 |
| rs10754920 | 2 | 146456888 | C | G | 0.004 | 0.003 | 1.53E-01 | 0.020 | 0.003 | 6.70E-09 | PC | 0.004 | 0.010 | 0.016 | 0.013 | 0.005 | 0.028 | 0.015 | 0.024 | 0.014 | 0.040 | -0.064 | 0.025 |
| rs6744254 | 2 | 155986195 | C | T | -0.003 | 0.002 | 1.11E-01 | -0.016 | 0.002 | 5.40E-13 | PC | 0.007 | 0.006 | 0.002 | 0.008 | -0.014 | 0.018 | 0.005 | 0.015 | 0.024 | 0.026 | 0.004 | 0.015 |
| rs11274218 | 2 | 215035001 | C | T | -0.004 | 0.002 | 6.92E-02 | -0.014 | 0.002 | 2.50E-09 | PC | 0.014 | 0.006 | 0.008 | 0.008 | 0.030 | 0.018 | 0.004 | 0.016 | 0.006 | 0.027 | 0.021 | 0.016 |
| rs10208088 | 2 | 221055873 | C | T | 0.000 | 0.002 | 8.85E-01 | 0.013 | 0.002 | 4.40E-09 | PC | 0.001 | 0.006 | 0.002 | 0.008 | -0.021 | 0.018 | 0.012 | 0.015 | 0.000 | 0.026 | 0.006 | 0.016 |
| rs11708955 | 3 | 49540114 | T | C | 0.000 | 0.002 | 9.85E-01 | -0.016 | 0.002 | 4.20E-11 | PC | 0.023 | 0.007 | 0.018 | 0.009 | 0.016 | 0.019 | 0.025 | 0.016 | 0.062 | 0.028 | 0.053 | 0.017 |
| rs13094887 | 3 | 70968431 | T | A | -0.010 | 0.002 | 8.57E-11 | 0.000 | 0.002 | 9.10E-01 | Alcohol | 0.012 | 0.007 | 0.021 | 0.009 | -0.007 | 0.019 | 0.002 | 0.016 | 0.037 | 0.028 | -0.002 | 0.017 |
| rs74664784 | 3 | 85475292 | C | T | -0.013 | 0.002 | 1.58E-14 | 0.149 | 0.063 | 1.80E-02 | Alcohol | 0.012 | 0.007 | 0.011 | 0.009 | -0.009 | 0.019 | 0.011 | 0.016 | 0.086 | 0.028 | 0.032 | 0.016 |
| rs78082503 | 3 | 116556913 | G | C | 0.001 | 0.002 | 7.55E-01 | -0.015 | 0.003 | 1.10E-08 | PC | 0.004 | 0.008 | -0.001 | 0.010 | 0.022 | 0.022 | 0.013 | 0.019 | 0.054 | 0.032 | 0.012 | 0.019 |
| rs9838144 | 3 | 131576287 | C | G | -0.010 | 0.002 | 2.65E-08 | -0.002 | 0.003 | 3.90E-01 | Alcohol | 0.000 | 0.008 | -0.005 | 0.010 | -0.027 | 0.023 | -0.002 | 0.019 | -0.003 | 0.033 | 0.014 | 0.020 |
| rs2011092 | 3 | 141124607 | C | T | -0.009 | 0.002 | 7.35E-09 | 0.011 | 0.002 | 2.40E-06 | Alcohol | 0.042 | 0.006 | 0.046 | 0.008 | 0.070 | 0.018 | 0.075 | 0.016 | 0.077 | 0.027 | 0.025 | 0.016 |
| rs6787172 | 3 | 158187811 | G | T | -0.008 | 0.001 | 4.27E-08 | -0.001 | 0.002 | 7.40E-01 | Alcohol | -0.020 | 0.006 | -0.016 | 0.008 | -0.044 | 0.018 | -0.030 | 0.015 | -0.035 | 0.026 | -0.002 | 0.015 |
| rs3748034 | 4 | 3446091 | T | G | -0.012 | 0.002 | 1.67E-08 | -0.006 | 0.003 | 8.10E-02 | Alcohol | 0.006 | 0.009 | 0.016 | 0.012 | -0.032 | 0.026 | -0.008 | 0.022 | -0.012 | 0.038 | 0.013 | 0.022 |
| rs6857629 | 4 | 39684977 | G | A | -0.001 | 0.002 | 5.62E-01 | 0.015 | 0.003 | 1.90E-09 | PC | 0.009 | 0.007 | 0.014 | 0.009 | 0.003 | 0.021 | 0.023 | 0.018 | 0.005 | 0.030 | 0.015 | 0.018 |
| rs4501255 | 4 | 42151306 | G | C | 0.011 | 0.002 | 4.83E-10 | 0.002 | 0.003 | 5.00E-01 | Alcohol | -0.010 | 0.007 | -0.012 | 0.010 | -0.004 | 0.022 | -0.019 | 0.019 | -0.014 | 0.032 | -0.019 | 0.019 |
| rs1229984 | 4 | 100239319 | C | T | 0.151 | 0.004 | <2.2e-308 | -0.031 | 0.007 | 2.40E-05 | Alcohol | -0.023 | 0.016 | -0.010 | 0.021 | -0.105 | 0.042 | -0.017 | 0.038 | 0.080 | 0.064 | -0.033 | 0.037 |
| rs2165670 | 4 | 100286085 | A | G | 0.023 | 0.002 | 1.67E-22 | -0.009 | 0.004 | 1.00E-02 | Alcohol | 0.023 | 0.010 | 0.039 | 0.013 | 0.017 | 0.029 | 0.025 | 0.025 | -0.026 | 0.043 | 0.006 | 0.025 |
| rs17029090 | 4 | 100443853 | G | A | -0.049 | 0.005 | 4.75E-21 | 0.007 | 0.008 | 3.60E-01 | Alcohol | 0.011 | 0.022 | -0.004 | 0.029 | -0.071 | 0.066 | 0.017 | 0.054 | 0.008 | 0.093 | 0.038 | 0.054 |
| rs4699791 | 4 | 101243023 | A | G | 0.019 | 0.002 | 6.58E-14 | -0.003 | 0.004 | 4.00E-01 | Alcohol | 0.014 | 0.011 | -0.009 | 0.014 | 0.017 | 0.031 | 0.020 | 0.026 | 0.065 | 0.043 | 0.014 | 0.027 |
| rs10004020 | 4 | 152968372 | A | G | 0.009 | 0.002 | 2.43E-08 | 0.006 | 0.002 | 1.00E-02 | Alcohol | -0.012 | 0.007 | -0.025 | 0.009 | -0.015 | 0.020 | -0.039 | 0.017 | 0.016 | 0.029 | 0.038 | 0.017 |
| rs2068625 | 4 | 159856739 | T | C | 0.003 | 0.002 | 1.14E-01 | -0.016 | 0.002 | 4.00E-11 | PC | -0.005 | 0.007 | -0.014 | 0.009 | -0.022 | 0.020 | -0.002 | 0.017 | 0.041 | 0.028 | -0.014 | 0.017 |
| rs12651313 | 4 | 171086393 | G | C | -0.009 | 0.001 | 3.79E-09 | -0.001 | 0.002 | 5.20E-01 | Alcohol | -0.004 | 0.006 | 0.002 | 0.008 | 0.010 | 0.018 | -0.005 | 0.016 | 0.030 | 0.026 | 0.011 | 0.016 |
| rs2220599 | 5 | 7378854 | C | G | 0.000 | 0.002 | 8.88E-01 | -0.016 | 0.002 | 2.40E-12 | PC | 0.008 | 0.007 | 0.014 | 0.009 | 0.019 | 0.019 | 0.005 | 0.016 | -0.005 | 0.027 | -0.015 | 0.016 |
| rs11749912 | 5 | 88065628 | A | G | 0.003 | 0.002 | 1.59E-01 | 0.014 | 0.002 | 4.90E-10 | PC | 0.002 | 0.006 | -0.001 | 0.008 | 0.010 | 0.018 | 0.019 | 0.015 | -0.015 | 0.026 | 0.006 | 0.015 |
| rs1469249 | 5 | 113837198 | G | A | 0.002 | 0.002 | 4.19E-01 | 0.016 | 0.003 | 9.50E-09 | PC | -0.011 | 0.007 | -0.008 | 0.010 | -0.023 | 0.021 | -0.006 | 0.018 | -0.013 | 0.031 | -0.019 | 0.019 |
| rs162894 | 5 | 131611872 | T | G | 0.001 | 0.002 | 6.32E-01 | 0.013 | 0.002 | 1.10E-08 | PC | -0.024 | 0.006 | -0.034 | 0.008 | -0.048 | 0.019 | -0.031 | 0.016 | 0.013 | 0.027 | -0.030 | 0.016 |
| rs56158102 | 5 | 144027803 | C | T | -0.003 | 0.002 | 1.95E-01 | 0.013 | 0.002 | 3.10E-08 | PC | -0.015 | 0.006 | -0.012 | 0.008 | -0.042 | 0.019 | -0.016 | 0.016 | -0.054 | 0.027 | -0.015 | 0.016 |
| rs9477970 | 6 | 19082663 | T | A | 0.000 | 0.002 | 9.08E-01 | -0.016 | 0.003 | 1.00E-08 | PC | -0.002 | 0.008 | 0.001 | 0.010 | -0.001 | 0.023 | 0.017 | 0.020 | 0.007 | 0.033 | -0.002 | 0.020 |
| rs9372625 | 6 | 98344031 | G | A | -0.008 | 0.002 | 2.17E-05 | -0.018 | 0.002 | 5.70E-16 | PC | 0.005 | 0.006 | 0.009 | 0.008 | 0.003 | 0.019 | 0.003 | 0.016 | -0.008 | 0.027 | 0.007 | 0.016 |
| rs6935828 | 6 | 140811367 | C | T | 0.004 | 0.002 | 3.72E-02 | -0.012 | 0.002 | 4.30E-08 | PC | -0.004 | 0.006 | -0.003 | 0.008 | 0.018 | 0.018 | 0.007 | 0.015 | -0.028 | 0.026 | 0.001 | 0.015 |
| rs55772938 | 7 | 70025113 | A | G | -0.003 | 0.002 | 1.37E-01 | -0.015 | 0.002 | 3.90E-10 | PC | 0.003 | 0.007 | -0.003 | 0.009 | 0.020 | 0.019 | -0.024 | 0.016 | 0.042 | 0.028 | 0.015 | 0.017 |
| rs6460047 | 7 | 73042443 | C | T | 0.012 | 0.002 | 9.69E-11 | -0.006 | 0.003 | 1.80E-02 | Alcohol | 0.002 | 0.008 | -0.002 | 0.011 | -0.021 | 0.024 | 0.008 | 0.020 | -0.003 | 0.034 | 0.011 | 0.021 |
| rs10236149 | 7 | 98977515 | G | A | -0.013 | 0.002 | 1.18E-09 | -0.013 | 0.003 | 5.50E-05 | Alcohol | 0.001 | 0.009 | 0.017 | 0.012 | 0.002 | 0.026 | 0.012 | 0.022 | -0.025 | 0.038 | -0.029 | 0.023 |
| rs35034355 | 7 | 103840115 | A | G | -0.008 | 0.001 | 2.87E-08 | -0.003 | 0.002 | 1.80E-01 | Alcohol | -0.003 | 0.006 | -0.009 | 0.008 | -0.001 | 0.018 | -0.014 | 0.015 | 0.001 | 0.026 | -0.004 | 0.015 |
| rs12706626 | 7 | 124531370 | G | A | 0.000 | 0.002 | 8.44E-01 | -0.013 | 0.002 | 1.40E-08 | PC | -0.009 | 0.006 | -0.016 | 0.008 | 0.012 | 0.018 | -0.015 | 0.015 | 0.006 | 0.026 | 0.004 | 0.016 |
| rs3944151 | 7 | 126970005 | A | G | -0.003 | 0.002 | 2.24E-01 | 0.014 | 0.002 | 3.10E-08 | PC | 0.016 | 0.007 | 0.013 | 0.009 | 0.003 | 0.020 | 0.015 | 0.017 | 0.037 | 0.028 | 0.010 | 0.017 |
| rs2345941 | 7 | 133337635 | A | G | -0.002 | 0.002 | 3.62E-01 | 0.015 | 0.002 | 4.00E-11 | PC | 0.007 | 0.006 | 0.002 | 0.008 | 0.036 | 0.018 | 0.007 | 0.015 | 0.019 | 0.026 | -0.002 | 0.015 |
| rs6951574 | 7 | 153489744 | C | T | 0.013 | 0.001 | 1.58E-19 | -0.002 | 0.002 | 4.40E-01 | Alcohol | 0.007 | 0.006 | 0.010 | 0.008 | -0.019 | 0.018 | 0.027 | 0.016 | -0.055 | 0.027 | 0.038 | 0.016 |
| rs13250583 | 8 | 20949917 | T | C | -0.010 | 0.002 | 4.70E-08 | -0.006 | 0.003 | 2.10E-02 | Alcohol | -0.012 | 0.008 | -0.012 | 0.010 | -0.029 | 0.022 | 0.014 | 0.018 | -0.060 | 0.032 | 0.024 | 0.019 |
| rs1217091 | 8 | 64527399 | C | T | 0.012 | 0.002 | 7.05E-11 | -0.005 | 0.003 | 6.30E-02 | Alcohol | 0.007 | 0.008 | 0.008 | 0.011 | 0.003 | 0.024 | 0.033 | 0.021 | 0.019 | 0.035 | -0.001 | 0.021 |
| rs28601761 | 8 | 126500031 | G | C | 0.009 | 0.001 | 7.17E-10 | -0.001 | 0.002 | 7.00E-01 | Alcohol | 0.009 | 0.006 | 0.012 | 0.008 | -0.003 | 0.018 | 0.005 | 0.016 | 0.042 | 0.027 | 0.029 | 0.016 |
| rs13262595 | 8 | 143316970 | A | G | -0.005 | 0.002 | 1.45E-02 | -0.016 | 0.002 | 1.20E-12 | PC | -0.001 | 0.006 | 0.005 | 0.008 | -0.016 | 0.018 | 0.013 | 0.015 | 0.043 | 0.026 | -0.005 | 0.015 |
| rs4977839 | 9 | 23355310 | G | A | 0.000 | 0.002 | 9.45E-01 | -0.020 | 0.002 | 4.70E-19 | PC | -0.006 | 0.006 | -0.001 | 0.009 | -0.037 | 0.019 | -0.006 | 0.016 | 0.019 | 0.027 | -0.022 | 0.016 |
| rs113851275 | 9 | 98297220 | G | A | 0.004 | 0.003 | 1.75E-01 | -0.021 | 0.004 | 3.20E-09 | PC | -0.028 | 0.010 | -0.044 | 0.012 | -0.025 | 0.028 | -0.009 | 0.024 | 0.011 | 0.041 | 0.008 | 0.024 |
| rs55932213 | 9 | 108755622 | G | A | 0.009 | 0.002 | 9.55E-09 | -0.004 | 0.003 | 9.70E-02 | Alcohol | 0.009 | 0.007 | -0.001 | 0.009 | 0.026 | 0.021 | 0.040 | 0.018 | 0.040 | 0.030 | -0.004 | 0.018 |
| rs7020477 | 9 | 116827760 | A | G | -0.002 | 0.002 | 3.27E-01 | 0.014 | 0.002 | 1.20E-08 | PC | -0.005 | 0.008 | -0.011 | 0.010 | -0.012 | 0.022 | 0.007 | 0.019 | 0.007 | 0.033 | 0.037 | 0.020 |
| rs73578186 | 9 | 126334485 | C | T | 0.002 | 0.002 | 4.03E-01 | 0.015 | 0.002 | 2.30E-10 | PC | -0.003 | 0.006 | -0.011 | 0.008 | 0.011 | 0.019 | -0.021 | 0.016 | -0.002 | 0.027 | -0.028 | 0.016 |
| rs7074871 | 10 | 110507806 | A | G | -0.009 | 0.002 | 1.86E-08 | 0.005 | 0.003 | 2.90E-02 | Alcohol | -0.018 | 0.007 | -0.016 | 0.009 | -0.010 | 0.020 | -0.016 | 0.017 | -0.020 | 0.030 | -0.012 | 0.018 |
| rs17665139 | 10 | 125093880 | T | C | -0.012 | 0.002 | 1.59E-08 | 0.001 | 0.003 | 7.20E-01 | Alcohol | -0.002 | 0.008 | -0.009 | 0.011 | 0.019 | 0.025 | -0.007 | 0.021 | -0.053 | 0.037 | 0.005 | 0.021 |
| rs11030084 | 11 | 27643725 | T | C | -0.011 | 0.002 | 1.72E-08 | -0.012 | 0.003 | 3.70E-05 | Alcohol | -0.010 | 0.008 | -0.005 | 0.010 | -0.010 | 0.022 | 0.006 | 0.019 | -0.012 | 0.033 | -0.004 | 0.019 |
| rs56030824 | 11 | 47397353 | A | G | -0.012 | 0.002 | 1.15E-13 | 0.010 | 0.002 | 3.80E-05 | Alcohol | 0.005 | 0.006 | -0.006 | 0.008 | 0.000 | 0.019 | -0.001 | 0.016 | -0.001 | 0.027 | -0.007 | 0.016 |
| rs2734849 | 11 | 113270160 | A | G | 0.011 | 0.002 | 6.73E-09 | -0.014 | 0.002 | 8.20E-10 | PC | 0.003 | 0.006 | 0.001 | 0.008 | 0.023 | 0.018 | -0.002 | 0.015 | 0.004 | 0.025 | -0.005 | 0.015 |
| rs1713676 | 11 | 113660576 | G | A | -0.008 | 0.001 | 4.29E-08 | -0.002 | 0.002 | 2.70E-01 | Alcohol | 0.016 | 0.006 | 0.019 | 0.008 | 0.013 | 0.018 | 0.020 | 0.015 | 0.013 | 0.026 | -0.012 | 0.015 |
| rs1448355 | 11 | 131286685 | C | T | 0.000 | 0.002 | 9.03E-01 | -0.015 | 0.002 | 1.30E-10 | PC | -0.001 | 0.006 | -0.001 | 0.008 | 0.011 | 0.018 | -0.002 | 0.015 | 0.008 | 0.026 | -0.006 | 0.016 |
| rs35933007 | 12 | 38929341 | G | A | 0.010 | 0.002 | 1.10E-05 | -0.015 | 0.003 | 9.20E-09 | PC | 0.003 | 0.008 | 0.017 | 0.011 | -0.001 | 0.024 | 0.018 | 0.021 | -0.017 | 0.035 | -0.030 | 0.021 |
| rs3809162 | 12 | 54674235 | G | A | 0.009 | 0.001 | 1.19E-09 | 0.004 | 0.002 | 8.30E-02 | Alcohol | -0.012 | 0.006 | -0.015 | 0.008 | -0.015 | 0.018 | -0.011 | 0.015 | 0.043 | 0.026 | 0.016 | 0.016 |
| rs10506274 | 12 | 81601464 | T | G | -0.009 | 0.001 | 5.78E-10 | 0.005 | 0.002 | 1.40E-02 | Alcohol | 0.006 | 0.006 | -0.011 | 0.008 | 0.020 | 0.018 | 0.029 | 0.015 | 0.001 | 0.026 | 0.008 | 0.015 |
| rs4842786 | 12 | 92170791 | A | G | -0.009 | 0.001 | 2.73E-09 | -0.004 | 0.002 | 9.70E-02 | Alcohol | 0.005 | 0.006 | 0.001 | 0.008 | 0.008 | 0.018 | 0.023 | 0.015 | -0.001 | 0.026 | 0.009 | 0.015 |
| rs206965 | 12 | 120856332 | T | C | -0.003 | 0.002 | 2.90E-01 | 0.016 | 0.003 | 9.10E-09 | PC | 0.010 | 0.008 | 0.008 | 0.010 | 0.014 | 0.022 | 0.033 | 0.019 | -0.008 | 0.032 | 0.014 | 0.019 |
| rs500321 | 13 | 27124360 | T | A | -0.010 | 0.002 | 4.92E-09 | 0.000 | 0.002 | 9.00E-01 | Alcohol | -0.011 | 0.007 | -0.013 | 0.009 | -0.008 | 0.020 | -0.029 | 0.018 | 0.009 | 0.030 | 0.031 | 0.018 |
| rs12874776 | 13 | 57794345 | C | A | -0.002 | 0.003 | 4.62E-01 | -0.023 | 0.004 | 8.00E-11 | PC | 0.001 | 0.010 | 0.007 | 0.013 | 0.036 | 0.030 | 0.007 | 0.026 | -0.005 | 0.044 | 0.018 | 0.026 |
| rs198262 | 14 | 57283283 | C | T | -0.012 | 0.005 | 9.85E-03 | 0.030 | 0.005 | 3.90E-08 | PC | 0.002 | 0.015 | 0.011 | 0.020 | 0.041 | 0.044 | -0.020 | 0.038 | -0.020 | 0.066 | -0.010 | 0.039 |
| rs2180870 | 14 | 58782779 | C | T | -0.012 | 0.002 | 1.12E-08 | -0.005 | 0.003 | 1.60E-01 | Alcohol | -0.007 | 0.009 | -0.019 | 0.012 | 0.040 | 0.025 | -0.030 | 0.022 | -0.014 | 0.037 | -0.004 | 0.022 |
| rs28929474 | 14 | 94844947 | T | C | -0.037 | 0.005 | 1.34E-11 | 0.016 | 0.008 | 3.60E-02 | Alcohol | -0.056 | 0.023 | -0.070 | 0.030 | -0.146 | 0.072 | -0.195 | 0.062 | 0.017 | 0.097 | -0.089 | 0.061 |
| rs166835 | 15 | 47716037 | C | T | 0.005 | 0.002 | 1.69E-02 | 0.013 | 0.002 | 3.40E-09 | PC | -0.013 | 0.006 | -0.023 | 0.008 | -0.038 | 0.018 | 0.002 | 0.015 | 0.001 | 0.026 | -0.015 | 0.015 |
| rs2472297 | 15 | 75027880 | T | C | 0.011 | 0.002 | 3.10E-10 | -0.007 | 0.002 | 7.50E-03 | Alcohol | 0.019 | 0.007 | 0.032 | 0.010 | -0.016 | 0.022 | 0.016 | 0.019 | 0.034 | 0.032 | 0.028 | 0.019 |
| rs12907323 | 15 | 86796012 | G | A | 0.008 | 0.001 | 9.93E-09 | 0.004 | 0.002 | 5.20E-02 | Alcohol | 0.005 | 0.006 | 0.018 | 0.008 | -0.008 | 0.018 | -0.026 | 0.015 | -0.019 | 0.026 | 0.023 | 0.015 |
| rs4702 | 15 | 91426560 | G | A | 0.005 | 0.002 | 1.34E-02 | 0.012 | 0.002 | 3.80E-08 | PC | 0.029 | 0.006 | 0.016 | 0.008 | 0.057 | 0.018 | 0.071 | 0.015 | -0.002 | 0.026 | 0.007 | 0.015 |
| rs6498759 | 16 | 18020295 | T | C | 0.003 | 0.002 | 1.48E-01 | 0.013 | 0.002 | 2.70E-08 | PC | 0.012 | 0.007 | 0.021 | 0.009 | 0.034 | 0.020 | -0.028 | 0.018 | 0.021 | 0.029 | 0.017 | 0.018 |
| rs17177078 | 16 | 24810681 | T | C | -0.022 | 0.003 | 1.27E-13 | 0.004 | 0.005 | 4.10E-01 | Alcohol | 0.014 | 0.012 | 0.018 | 0.016 | -0.001 | 0.035 | 0.044 | 0.030 | 0.071 | 0.050 | 0.012 | 0.031 |
| rs378421 | 16 | 28754684 | A | G | -0.011 | 0.001 | 4.83E-14 | -0.005 | 0.002 | 2.90E-02 | Alcohol | 0.001 | 0.007 | -0.001 | 0.009 | 0.006 | 0.020 | 0.005 | 0.017 | -0.032 | 0.028 | -0.012 | 0.017 |
| rs62044525 | 16 | 64872590 | G | C | -0.012 | 0.002 | 1.03E-10 | 0.002 | 0.003 | 3.80E-01 | Alcohol | -0.010 | 0.008 | -0.011 | 0.010 | -0.044 | 0.023 | -0.016 | 0.020 | -0.018 | 0.034 | -0.018 | 0.020 |
| rs3730399 | 16 | 67229019 | A | G | -0.002 | 0.004 | 6.45E-01 | 0.025 | 0.004 | 3.30E-08 | PC | -0.015 | 0.012 | 0.005 | 0.016 | -0.038 | 0.035 | 0.021 | 0.030 | -0.032 | 0.051 | -0.015 | 0.030 |
| rs1104608 | 16 | 73912588 | C | G | -0.011 | 0.001 | 1.05E-13 | 0.008 | 0.002 | 2.50E-04 | Alcohol | 0.000 | 0.006 | 0.002 | 0.008 | 0.016 | 0.019 | -0.018 | 0.016 | 0.000 | 0.027 | 0.009 | 0.016 |
| rs4548913 | 17 | 2209888 | A | G | -0.008 | 0.002 | 3.11E-08 | -0.002 | 0.002 | 3.20E-01 | Alcohol | -0.015 | 0.006 | -0.021 | 0.008 | 0.003 | 0.019 | -0.050 | 0.016 | -0.004 | 0.027 | 0.004 | 0.016 |
| rs3803800 | 17 | 7462969 | G | A | 0.011 | 0.002 | 1.50E-10 | 0.007 | 0.003 | 7.90E-03 | Alcohol | -0.023 | 0.007 | -0.024 | 0.010 | -0.020 | 0.022 | -0.025 | 0.019 | -0.052 | 0.032 | 0.007 | 0.019 |
| rs7209653 | 17 | 19882084 | T | C | 0.001 | 0.002 | 5.16E-01 | 0.016 | 0.002 | 9.10E-11 | PC | -0.007 | 0.007 | 0.000 | 0.009 | -0.035 | 0.020 | -0.012 | 0.017 | 0.012 | 0.029 | -0.023 | 0.017 |
| rs2854334 | 17 | 29715500 | G | A | 0.009 | 0.001 | 7.51E-10 | -0.002 | 0.002 | 3.40E-01 | Alcohol | -0.014 | 0.006 | -0.018 | 0.008 | 0.000 | 0.018 | -0.015 | 0.016 | -0.014 | 0.026 | -0.011 | 0.016 |
| rs12603813 | 17 | 43196584 | T | C | -0.002 | 0.002 | 3.69E-01 | 0.014 | 0.003 | 1.30E-08 | PC | 0.005 | 0.007 | 0.017 | 0.009 | 0.036 | 0.020 | -0.003 | 0.017 | 0.033 | 0.030 | -0.032 | 0.017 |
| rs66643547 | 17 | 79375315 | C | T | 0.001 | 0.002 | 4.71E-01 | -0.015 | 0.002 | 3.20E-11 | PC | 0.020 | 0.007 | 0.021 | 0.009 | 0.005 | 0.019 | 0.024 | 0.017 | 0.061 | 0.029 | 0.019 | 0.017 |
| rs784256 | 18 | 53398626 | G | A | 0.006 | 0.002 | 2.39E-02 | 0.019 | 0.003 | 6.20E-11 | PC | -0.005 | 0.008 | 0.006 | 0.010 | -0.020 | 0.023 | 0.009 | 0.020 | -0.015 | 0.033 | -0.034 | 0.020 |
| rs281379 | 19 | 49214274 | A | G | 0.014 | 0.001 | 4.91E-21 | 0.004 | 0.002 | 1.00E-01 | Alcohol | 0.013 | 0.006 | 0.007 | 0.008 | 0.069 | 0.018 | 0.001 | 0.015 | 0.004 | 0.026 | -0.018 | 0.015 |
| rs4815364 | 20 | 25035711 | A | G | 0.009 | 0.001 | 1.02E-08 | -0.001 | 0.002 | 5.80E-01 | Alcohol | -0.001 | 0.006 | 0.001 | 0.008 | -0.011 | 0.019 | 0.002 | 0.016 | 0.025 | 0.027 | -0.016 | 0.016 |
| rs6129084 | 20 | 59828857 | A | T | -0.007 | 0.002 | 5.53E-04 | -0.014 | 0.002 | 9.30E-10 | PC | -0.001 | 0.006 | 0.005 | 0.008 | -0.004 | 0.019 | -0.010 | 0.016 | 0.015 | 0.027 | -0.001 | 0.016 |
| rs136553 | 22 | 27255675 | C | T | 0.003 | 0.002 | 9.73E-02 | -0.015 | 0.002 | 2.30E-11 | PC | -0.004 | 0.006 | -0.001 | 0.008 | 0.000 | 0.018 | -0.005 | 0.016 | 0.014 | 0.027 | 0.003 | 0.016 |
| rs7288455 | 22 | 39966547 | A | G | 0.000 | 0.002 | 8.19E-01 | 0.013 | 0.002 | 4.00E-09 | PC | 0.001 | 0.006 | 0.005 | 0.008 | -0.018 | 0.019 | 0.004 | 0.016 | -0.025 | 0.027 | -0.007 | 0.016 |
| rs9607814 | 22 | 41946519 | A | C | -0.010 | 0.002 | 4.31E-08 | 0.005 | 0.003 | 8.80E-02 | Alcohol | 0.000 | 0.008 | 0.016 | 0.010 | 0.038 | 0.022 | -0.041 | 0.019 | -0.015 | 0.032 | -0.026 | 0.019 |

| **Table S11. Beta estimates for the SNPs included in the multivariable MR of alcohol consumption, computer use, and colorectal cancer risk** | | | | | | | | | | | | | | | | | | | | | |
| --- | --- | --- | --- | --- | --- | --- | --- | --- | --- | --- | --- | --- | --- | --- | --- | --- | --- | --- | --- | --- | --- |
| SNP | Chr | POS | EA | OA | beta_alc | se_alc | pval_alc | beta_pc | se_pc | pval_pc | phenotype | beta_crc | se_crc | beta_crc  _men | se_crc  _men | beta_crc  _women | se_crc  _women | beta_co | se_co | beta_re | se_re |
| rs2748985 | 1 | 1853184 | T | C | 0.002 | 0.002 | 2.94E-01 | -0.015 | 0.002 | 4.10E-12 | PC | 0.006 | 0.010 | 0.017 | 0.014 | -0.003 | 0.014 | 0.016 | 0.012 | -0.003 | 0.015 |
| rs58107686 | 1 | 33837334 | A | C | -0.010 | 0.002 | 7.79E-10 | 0.000 | 0.002 | 9.40E-01 | Alcohol | 0.007 | 0.010 | 0.018 | 0.014 | -0.002 | 0.014 | 0.007 | 0.012 | 0.009 | 0.016 |
| rs12088813 | 1 | 66407700 | C | A | -0.009 | 0.002 | 1.58E-08 | 0.004 | 0.002 | 7.30E-02 | Alcohol | 0.017 | 0.011 | 0.019 | 0.015 | 0.015 | 0.015 | -0.001 | 0.013 | 0.031 | 0.017 |
| rs5024204 | 1 | 71491890 | T | A | 0.010 | 0.002 | 2.55E-09 | 0.002 | 0.002 | 5.10E-01 | Alcohol | -0.005 | 0.011 | 0.005 | 0.015 | -0.015 | 0.015 | 0.006 | 0.013 | -0.008 | 0.017 |
| rs12145677 | 1 | 110023610 | G | A | 0.005 | 0.002 | 2.43E-02 | -0.017 | 0.002 | 1.20E-12 | PC | -0.006 | 0.011 | 0.012 | 0.015 | -0.023 | 0.015 | -0.007 | 0.013 | -0.004 | 0.017 |
| rs10753661 | 1 | 165119792 | A | G | -0.009 | 0.002 | 3.76E-08 | 0.003 | 0.002 | 1.80E-01 | Alcohol | 0.006 | 0.011 | -0.008 | 0.015 | 0.020 | 0.015 | 0.003 | 0.012 | 0.022 | 0.016 |
| rs28680958 | 1 | 173848808 | A | G | -0.011 | 0.002 | 5.13E-10 | -0.005 | 0.003 | 4.50E-02 | Alcohol | -0.013 | 0.011 | -0.013 | 0.016 | -0.018 | 0.016 | -0.024 | 0.014 | -0.006 | 0.018 |
| rs7526456 | 1 | 197773734 | T | C | 0.000 | 0.002 | 9.48E-01 | -0.013 | 0.002 | 1.20E-08 | PC | -0.005 | 0.010 | -0.001 | 0.014 | -0.009 | 0.014 | -0.004 | 0.012 | -0.012 | 0.015 |
| rs823114 | 1 | 205719532 | A | G | 0.009 | 0.001 | 2.31E-09 | -0.005 | 0.002 | 1.70E-02 | Alcohol | -0.031 | 0.010 | -0.033 | 0.014 | -0.030 | 0.014 | -0.033 | 0.012 | -0.032 | 0.015 |
| rs77165542 | 2 | 430975 | T | C | -0.026 | 0.004 | 5.63E-11 | -0.013 | 0.006 | 3.60E-02 | Alcohol | -0.058 | 0.030 | -0.033 | 0.043 | -0.085 | 0.043 | -0.060 | 0.036 | -0.026 | 0.047 |
| rs2178197 | 2 | 27860551 | G | A | -0.009 | 0.001 | 2.45E-09 | -0.003 | 0.002 | 2.00E-01 | Alcohol | -0.002 | 0.010 | -0.006 | 0.014 | -0.001 | 0.014 | -0.007 | 0.012 | 0.004 | 0.015 |
| rs1004787 | 2 | 45159091 | A | G | 0.008 | 0.001 | 8.40E-09 | 0.002 | 0.002 | 3.90E-01 | Alcohol | -0.020 | 0.010 | -0.019 | 0.014 | -0.018 | 0.014 | -0.020 | 0.012 | -0.023 | 0.015 |
| rs13032049 | 2 | 63581507 | G | A | 0.010 | 0.002 | 3.00E-10 | 0.002 | 0.002 | 3.40E-01 | Alcohol | -0.006 | 0.011 | 0.011 | 0.015 | -0.022 | 0.015 | -0.015 | 0.013 | -0.010 | 0.016 |
| rs828867 | 2 | 74334462 | A | G | 0.009 | 0.001 | 2.15E-09 | 0.002 | 0.002 | 4.30E-01 | Alcohol | 0.010 | 0.010 | 0.008 | 0.014 | 0.009 | 0.014 | 0.008 | 0.012 | 0.022 | 0.016 |
| rs11692435 | 2 | 98275354 | A | G | 0.017 | 0.003 | 2.53E-11 | -0.010 | 0.004 | 1.70E-02 | Alcohol | -0.074 | 0.018 | -0.054 | 0.025 | -0.102 | 0.026 | -0.098 | 0.022 | -0.036 | 0.028 |
| rs10754920 | 2 | 146456888 | C | G | 0.004 | 0.003 | 1.53E-01 | 0.020 | 0.003 | 6.70E-09 | PC | 0.002 | 0.014 | -0.012 | 0.020 | 0.011 | 0.020 | 0.007 | 0.018 | 0.011 | 0.023 |
| rs6744254 | 2 | 155986195 | C | T | -0.003 | 0.002 | 1.11E-01 | -0.016 | 0.002 | 5.40E-13 | PC | 0.014 | 0.010 | 0.041 | 0.014 | -0.012 | 0.014 | 0.020 | 0.012 | 0.021 | 0.015 |
| rs11274218 | 2 | 215035001 | C | T | -0.004 | 0.002 | 6.92E-02 | -0.014 | 0.002 | 2.50E-09 | PC | -0.007 | 0.010 | 0.005 | 0.014 | -0.016 | 0.014 | -0.009 | 0.012 | 0.001 | 0.015 |
| rs10208088 | 2 | 221055873 | C | T | 0.000 | 0.002 | 8.85E-01 | 0.013 | 0.002 | 4.40E-09 | PC | 0.002 | 0.010 | 0.007 | 0.014 | -0.006 | 0.014 | 0.003 | 0.012 | -0.019 | 0.015 |
| rs11708955 | 3 | 49540114 | T | C | 0.000 | 0.002 | 9.85E-01 | -0.016 | 0.002 | 4.20E-11 | PC | -0.001 | 0.010 | 0.007 | 0.015 | -0.006 | 0.015 | -0.007 | 0.012 | 0.029 | 0.016 |
| rs13094887 | 3 | 70968431 | T | A | -0.010 | 0.002 | 8.57E-11 | 0.000 | 0.002 | 9.10E-01 | Alcohol | -0.014 | 0.011 | -0.017 | 0.015 | -0.010 | 0.015 | -0.006 | 0.013 | -0.054 | 0.016 |
| rs74664784 | 3 | 85475292 | C | T | -0.013 | 0.002 | 1.58E-14 | 0.149 | 0.063 | 1.80E-02 | Alcohol | 0.030 | 0.010 | 0.029 | 0.014 | 0.033 | 0.014 | 0.035 | 0.012 | 0.039 | 0.015 |
| rs78082503 | 3 | 116556913 | G | C | 0.001 | 0.002 | 7.55E-01 | -0.015 | 0.003 | 1.10E-08 | PC | -0.007 | 0.011 | -0.020 | 0.016 | 0.004 | 0.016 | -0.002 | 0.014 | 0.033 | 0.018 |
| rs9838144 | 3 | 131576287 | C | G | -0.010 | 0.002 | 2.65E-08 | -0.002 | 0.003 | 3.90E-01 | Alcohol | -0.002 | 0.012 | -0.023 | 0.017 | 0.022 | 0.017 | -0.009 | 0.014 | 0.013 | 0.019 |
| rs2011092 | 3 | 141124607 | C | T | -0.009 | 0.002 | 7.35E-09 | 0.011 | 0.002 | 2.40E-06 | Alcohol | 0.021 | 0.010 | 0.022 | 0.015 | 0.023 | 0.015 | 0.028 | 0.012 | 0.021 | 0.016 |
| rs6787172 | 3 | 158187811 | G | T | -0.008 | 0.001 | 4.27E-08 | -0.001 | 0.002 | 7.40E-01 | Alcohol | -0.013 | 0.010 | -0.018 | 0.014 | -0.011 | 0.014 | -0.014 | 0.012 | -0.003 | 0.015 |
| rs3748034 | 4 | 3446091 | T | G | -0.012 | 0.002 | 1.67E-08 | -0.006 | 0.003 | 8.10E-02 | Alcohol | 0.032 | 0.014 | 0.034 | 0.019 | 0.030 | 0.019 | 0.024 | 0.017 | 0.053 | 0.022 |
| rs6857629 | 4 | 39684977 | G | A | -0.001 | 0.002 | 5.62E-01 | 0.015 | 0.003 | 1.90E-09 | PC | 0.021 | 0.011 | 0.034 | 0.016 | 0.012 | 0.016 | 0.014 | 0.013 | 0.026 | 0.017 |
| rs4501255 | 4 | 42151306 | G | C | 0.011 | 0.002 | 4.83E-10 | 0.002 | 0.003 | 5.00E-01 | Alcohol | 0.014 | 0.012 | 0.021 | 0.016 | 0.005 | 0.016 | 0.025 | 0.014 | -0.001 | 0.018 |
| rs1229984 | 4 | 100239319 | C | T | 0.151 | 0.004 | <2.2e-308 | -0.031 | 0.007 | 2.40E-05 | Alcohol | 0.039 | 0.021 | 0.040 | 0.029 | 0.036 | 0.030 | 0.039 | 0.026 | 0.063 | 0.035 |
| rs2165670 | 4 | 100286085 | A | G | 0.023 | 0.002 | 1.67E-22 | -0.009 | 0.004 | 1.00E-02 | Alcohol | 0.009 | 0.016 | -0.005 | 0.023 | 0.027 | 0.023 | 0.010 | 0.020 | -0.001 | 0.026 |
| rs17029090 | 4 | 100443853 | G | A | -0.049 | 0.005 | 4.75E-21 | 0.007 | 0.008 | 3.60E-01 | Alcohol | 0.027 | 0.031 | 0.015 | 0.045 | 0.040 | 0.044 | 0.012 | 0.042 | 0.062 | 0.054 |
| rs4699791 | 4 | 101243023 | A | G | 0.019 | 0.002 | 6.58E-14 | -0.003 | 0.004 | 4.00E-01 | Alcohol | -0.005 | 0.016 | -0.003 | 0.023 | -0.004 | 0.023 | -0.005 | 0.020 | -0.005 | 0.026 |
| rs10004020 | 4 | 152968372 | A | G | 0.009 | 0.002 | 2.43E-08 | 0.006 | 0.002 | 1.00E-02 | Alcohol | -0.028 | 0.011 | -0.038 | 0.015 | -0.020 | 0.015 | -0.029 | 0.013 | -0.026 | 0.017 |
| rs2068625 | 4 | 159856739 | T | C | 0.003 | 0.002 | 1.14E-01 | -0.016 | 0.002 | 4.00E-11 | PC | 0.017 | 0.011 | 0.011 | 0.015 | 0.020 | 0.015 | 0.025 | 0.013 | 0.020 | 0.017 |
| rs12651313 | 4 | 171086393 | G | C | -0.009 | 0.001 | 3.79E-09 | -0.001 | 0.002 | 5.20E-01 | Alcohol | -0.011 | 0.010 | 0.008 | 0.014 | -0.029 | 0.014 | -0.017 | 0.012 | -0.007 | 0.015 |
| rs2220599 | 5 | 7378854 | C | G | 0.000 | 0.002 | 8.88E-01 | -0.016 | 0.002 | 2.40E-12 | PC | -0.003 | 0.010 | -0.010 | 0.014 | 0.000 | 0.014 | 0.001 | 0.012 | -0.002 | 0.016 |
| rs11749912 | 5 | 88065628 | A | G | 0.003 | 0.002 | 1.59E-01 | 0.014 | 0.002 | 4.90E-10 | PC | 0.015 | 0.010 | 0.010 | 0.014 | 0.023 | 0.014 | 0.019 | 0.012 | -0.001 | 0.015 |
| rs1469249 | 5 | 113837198 | G | A | 0.002 | 0.002 | 4.19E-01 | 0.016 | 0.003 | 9.50E-09 | PC | 0.009 | 0.012 | 0.017 | 0.017 | 0.005 | 0.017 | -0.001 | 0.014 | 0.038 | 0.018 |
| rs162894 | 5 | 131611872 | T | G | 0.001 | 0.002 | 6.32E-01 | 0.013 | 0.002 | 1.10E-08 | PC | -0.012 | 0.010 | -0.017 | 0.014 | -0.007 | 0.014 | -0.018 | 0.012 | -0.012 | 0.016 |
| rs56158102 | 5 | 144027803 | C | T | -0.003 | 0.002 | 1.95E-01 | 0.013 | 0.002 | 3.10E-08 | PC | -0.006 | 0.010 | 0.003 | 0.014 | -0.014 | 0.014 | -0.001 | 0.012 | -0.001 | 0.016 |
| rs9477970 | 6 | 19082663 | T | A | 0.000 | 0.002 | 9.08E-01 | -0.016 | 0.003 | 1.00E-08 | PC | -0.017 | 0.012 | -0.032 | 0.018 | -0.004 | 0.018 | -0.016 | 0.015 | -0.005 | 0.020 |
| rs9372625 | 6 | 98344031 | G | A | -0.008 | 0.002 | 2.17E-05 | -0.018 | 0.002 | 5.70E-16 | PC | 0.031 | 0.010 | 0.033 | 0.014 | 0.030 | 0.014 | 0.026 | 0.012 | 0.041 | 0.016 |
| rs6935828 | 6 | 140811367 | C | T | 0.004 | 0.002 | 3.72E-02 | -0.012 | 0.002 | 4.30E-08 | PC | -0.002 | 0.010 | 0.005 | 0.014 | -0.004 | 0.014 | 0.002 | 0.012 | 0.022 | 0.015 |
| rs55772938 | 7 | 70025113 | A | G | -0.003 | 0.002 | 1.37E-01 | -0.015 | 0.002 | 3.90E-10 | PC | -0.008 | 0.011 | 0.001 | 0.015 | -0.017 | 0.015 | -0.001 | 0.013 | -0.006 | 0.016 |
| rs6460047 | 7 | 73042443 | C | T | 0.012 | 0.002 | 9.69E-11 | -0.006 | 0.003 | 1.80E-02 | Alcohol | -0.002 | 0.012 | 0.004 | 0.017 | -0.008 | 0.017 | -0.004 | 0.014 | 0.019 | 0.019 |
| rs10236149 | 7 | 98977515 | G | A | -0.013 | 0.002 | 1.18E-09 | -0.013 | 0.003 | 5.50E-05 | Alcohol | -0.007 | 0.014 | 0.005 | 0.020 | -0.025 | 0.020 | -0.012 | 0.017 | 0.015 | 0.022 |
| rs35034355 | 7 | 103840115 | A | G | -0.008 | 0.001 | 2.87E-08 | -0.003 | 0.002 | 1.80E-01 | Alcohol | -0.027 | 0.010 | -0.019 | 0.014 | -0.032 | 0.014 | -0.031 | 0.012 | -0.032 | 0.015 |
| rs12706626 | 7 | 124531370 | G | A | 0.000 | 0.002 | 8.44E-01 | -0.013 | 0.002 | 1.40E-08 | PC | -0.007 | 0.010 | -0.029 | 0.014 | 0.016 | 0.014 | -0.007 | 0.012 | 0.003 | 0.016 |
| rs3944151 | 7 | 126970005 | A | G | -0.003 | 0.002 | 2.24E-01 | 0.014 | 0.002 | 3.10E-08 | PC | 0.021 | 0.011 | 0.020 | 0.015 | 0.019 | 0.015 | 0.029 | 0.013 | 0.016 | 0.017 |
| rs2345941 | 7 | 133337635 | A | G | -0.002 | 0.002 | 3.62E-01 | 0.015 | 0.002 | 4.00E-11 | PC | -0.023 | 0.010 | -0.013 | 0.014 | -0.033 | 0.014 | -0.025 | 0.012 | -0.012 | 0.015 |
| rs6951574 | 7 | 153489744 | C | T | 0.013 | 0.001 | 1.58E-19 | -0.002 | 0.002 | 4.40E-01 | Alcohol | -0.006 | 0.010 | -0.001 | 0.014 | -0.015 | 0.014 | -0.010 | 0.012 | 0.004 | 0.016 |
| rs13250583 | 8 | 20949917 | T | C | -0.010 | 0.002 | 4.70E-08 | -0.006 | 0.003 | 2.10E-02 | Alcohol | -0.015 | 0.012 | -0.038 | 0.017 | 0.010 | 0.017 | -0.017 | 0.014 | 0.000 | 0.019 |
| rs1217091 | 8 | 64527399 | C | T | 0.012 | 0.002 | 7.05E-11 | -0.005 | 0.003 | 6.30E-02 | Alcohol | 0.010 | 0.012 | 0.010 | 0.018 | 0.011 | 0.018 | 0.018 | 0.015 | -0.014 | 0.019 |
| rs28601761 | 8 | 126500031 | G | C | 0.009 | 0.001 | 7.17E-10 | -0.001 | 0.002 | 7.00E-01 | Alcohol | -0.016 | 0.010 | -0.014 | 0.014 | -0.019 | 0.014 | -0.024 | 0.012 | 0.006 | 0.015 |
| rs13262595 | 8 | 143316970 | A | G | -0.005 | 0.002 | 1.45E-02 | -0.016 | 0.002 | 1.20E-12 | PC | -0.001 | 0.010 | 0.004 | 0.014 | -0.008 | 0.014 | -0.007 | 0.012 | 0.012 | 0.015 |
| rs4977839 | 9 | 23355310 | G | A | 0.000 | 0.002 | 9.45E-01 | -0.020 | 0.002 | 4.70E-19 | PC | -0.004 | 0.010 | -0.002 | 0.014 | -0.005 | 0.014 | 0.010 | 0.012 | -0.006 | 0.016 |
| rs113851275 | 9 | 98297220 | G | A | 0.004 | 0.003 | 1.75E-01 | -0.021 | 0.004 | 3.20E-09 | PC | 0.022 | 0.015 | 0.001 | 0.022 | 0.043 | 0.022 | 0.010 | 0.019 | -0.001 | 0.024 |
| rs55932213 | 9 | 108755622 | G | A | 0.009 | 0.002 | 9.55E-09 | -0.004 | 0.003 | 9.70E-02 | Alcohol | -0.014 | 0.011 | -0.010 | 0.016 | -0.016 | 0.016 | -0.008 | 0.013 | -0.020 | 0.017 |
| rs7020477 | 9 | 116827760 | A | G | -0.002 | 0.002 | 3.27E-01 | 0.014 | 0.002 | 1.20E-08 | PC | 0.016 | 0.012 | -0.004 | 0.017 | 0.033 | 0.017 | 0.020 | 0.014 | 0.011 | 0.018 |
| rs73578186 | 9 | 126334485 | C | T | 0.002 | 0.002 | 4.03E-01 | 0.015 | 0.002 | 2.30E-10 | PC | -0.015 | 0.010 | -0.022 | 0.014 | -0.006 | 0.015 | -0.018 | 0.012 | -0.003 | 0.016 |
| rs7074871 | 10 | 110507806 | A | G | -0.009 | 0.002 | 1.86E-08 | 0.005 | 0.003 | 2.90E-02 | Alcohol | -0.011 | 0.011 | -0.011 | 0.015 | -0.012 | 0.015 | -0.015 | 0.013 | 0.000 | 0.017 |
| rs17665139 | 10 | 125093880 | T | C | -0.012 | 0.002 | 1.59E-08 | 0.001 | 0.003 | 7.20E-01 | Alcohol | 0.002 | 0.014 | 0.019 | 0.019 | -0.012 | 0.019 | 0.021 | 0.016 | -0.041 | 0.022 |
| rs11030084 | 11 | 27643725 | T | C | -0.011 | 0.002 | 1.72E-08 | -0.012 | 0.003 | 3.70E-05 | Alcohol | -0.003 | 0.012 | -0.017 | 0.017 | 0.011 | 0.017 | -0.007 | 0.015 | -0.012 | 0.019 |
| rs56030824 | 11 | 47397353 | A | G | -0.012 | 0.002 | 1.15E-13 | 0.010 | 0.002 | 3.80E-05 | Alcohol | 0.009 | 0.010 | 0.013 | 0.014 | 0.005 | 0.014 | 0.005 | 0.012 | -0.004 | 0.016 |
| rs2734849 | 11 | 113270160 | A | G | 0.011 | 0.002 | 6.73E-09 | -0.014 | 0.002 | 8.20E-10 | PC | -0.003 | 0.010 | 0.004 | 0.014 | -0.010 | 0.014 | -0.009 | 0.012 | 0.012 | 0.015 |
| rs1713676 | 11 | 113660576 | G | A | -0.008 | 0.001 | 4.29E-08 | -0.002 | 0.002 | 2.70E-01 | Alcohol | 0.002 | 0.010 | 0.005 | 0.014 | 0.001 | 0.014 | -0.003 | 0.012 | -0.005 | 0.015 |
| rs1448355 | 11 | 131286685 | C | T | 0.000 | 0.002 | 9.03E-01 | -0.015 | 0.002 | 1.30E-10 | PC | 0.012 | 0.010 | -0.008 | 0.014 | 0.030 | 0.014 | 0.004 | 0.012 | 0.013 | 0.016 |
| rs35933007 | 12 | 38929341 | G | A | 0.010 | 0.002 | 1.10E-05 | -0.015 | 0.003 | 9.20E-09 | PC | -0.009 | 0.013 | -0.005 | 0.019 | -0.017 | 0.019 | -0.011 | 0.016 | -0.002 | 0.021 |
| rs3809162 | 12 | 54674235 | G | A | 0.009 | 0.001 | 1.19E-09 | 0.004 | 0.002 | 8.30E-02 | Alcohol | 0.010 | 0.010 | 0.008 | 0.014 | 0.011 | 0.014 | 0.011 | 0.012 | 0.017 | 0.016 |
| rs10506274 | 12 | 81601464 | T | G | -0.009 | 0.001 | 5.78E-10 | 0.005 | 0.002 | 1.40E-02 | Alcohol | 0.014 | 0.010 | 0.003 | 0.014 | 0.023 | 0.014 | 0.012 | 0.012 | 0.024 | 0.015 |
| rs4842786 | 12 | 92170791 | A | G | -0.009 | 0.001 | 2.73E-09 | -0.004 | 0.002 | 9.70E-02 | Alcohol | -0.013 | 0.010 | -0.011 | 0.014 | -0.018 | 0.014 | -0.011 | 0.012 | -0.030 | 0.015 |
| rs206965 | 12 | 120856332 | T | C | -0.003 | 0.002 | 2.90E-01 | 0.016 | 0.003 | 9.10E-09 | PC | -0.008 | 0.012 | -0.014 | 0.017 | 0.001 | 0.017 | -0.001 | 0.014 | -0.029 | 0.019 |
| rs500321 | 13 | 27124360 | T | A | -0.010 | 0.002 | 4.92E-09 | 0.000 | 0.002 | 9.00E-01 | Alcohol | 0.008 | 0.011 | 0.024 | 0.016 | -0.010 | 0.016 | 0.001 | 0.013 | 0.020 | 0.017 |
| rs12874776 | 13 | 57794345 | C | A | -0.002 | 0.003 | 4.62E-01 | -0.023 | 0.004 | 8.00E-11 | PC | -0.010 | 0.016 | -0.008 | 0.023 | -0.009 | 0.022 | -0.008 | 0.019 | -0.012 | 0.025 |
| rs198262 | 14 | 57283283 | C | T | -0.012 | 0.005 | 9.85E-03 | 0.030 | 0.005 | 3.90E-08 | PC | -0.023 | 0.030 | 0.005 | 0.035 | -0.003 | 0.034 | 0.015 | 0.029 | -0.038 | 0.038 |
| rs2180870 | 14 | 58782779 | C | T | -0.012 | 0.002 | 1.12E-08 | -0.005 | 0.003 | 1.60E-01 | Alcohol | 0.007 | 0.017 | 0.008 | 0.020 | 0.010 | 0.020 | 0.016 | 0.016 | -0.041 | 0.022 |
| rs28929474 | 14 | 94844947 | T | C | -0.037 | 0.005 | 1.34E-11 | 0.016 | 0.008 | 3.60E-02 | Alcohol | -0.102 | 0.047 | -0.147 | 0.058 | -0.050 | 0.055 | -0.105 | 0.047 | -0.124 | 0.063 |
| rs166835 | 15 | 47716037 | C | T | 0.005 | 0.002 | 1.69E-02 | 0.013 | 0.002 | 3.40E-09 | PC | -0.007 | 0.010 | -0.013 | 0.014 | 0.002 | 0.014 | -0.001 | 0.012 | -0.012 | 0.015 |
| rs2472297 | 15 | 75027880 | T | C | 0.011 | 0.002 | 3.10E-10 | -0.007 | 0.002 | 7.50E-03 | Alcohol | -0.006 | 0.013 | 0.004 | 0.018 | -0.017 | 0.018 | -0.018 | 0.015 | 0.015 | 0.020 |
| rs12907323 | 15 | 86796012 | G | A | 0.008 | 0.001 | 9.93E-09 | 0.004 | 0.002 | 5.20E-02 | Alcohol | -0.002 | 0.010 | -0.005 | 0.014 | 0.003 | 0.014 | 0.001 | 0.012 | -0.016 | 0.015 |
| rs4702 | 15 | 91426560 | G | A | 0.005 | 0.002 | 1.34E-02 | 0.012 | 0.002 | 3.80E-08 | PC | 0.018 | 0.010 | 0.022 | 0.014 | 0.014 | 0.014 | 0.020 | 0.012 | 0.013 | 0.015 |
| rs6498759 | 16 | 18020295 | T | C | 0.003 | 0.002 | 1.48E-01 | 0.013 | 0.002 | 2.70E-08 | PC | -0.003 | 0.010 | 0.001 | 0.014 | -0.009 | 0.014 | -0.004 | 0.012 | 0.000 | 0.016 |
| rs17177078 | 16 | 24810681 | T | C | -0.022 | 0.003 | 1.27E-13 | 0.004 | 0.005 | 4.10E-01 | Alcohol | -0.041 | 0.019 | -0.053 | 0.027 | -0.029 | 0.027 | -0.040 | 0.023 | -0.024 | 0.029 |
| rs378421 | 16 | 28754684 | A | G | -0.011 | 0.001 | 4.83E-14 | -0.005 | 0.002 | 2.90E-02 | Alcohol | 0.008 | 0.010 | 0.008 | 0.014 | 0.010 | 0.014 | 0.016 | 0.012 | -0.003 | 0.016 |
| rs62044525 | 16 | 64872590 | G | C | -0.012 | 0.002 | 1.03E-10 | 0.002 | 0.003 | 3.80E-01 | Alcohol | 0.001 | 0.013 | -0.007 | 0.018 | 0.011 | 0.018 | -0.006 | 0.015 | -0.014 | 0.020 |
| rs3730399 | 16 | 67229019 | A | G | -0.002 | 0.004 | 6.45E-01 | 0.025 | 0.004 | 3.30E-08 | PC | -0.054 | 0.020 | -0.003 | 0.028 | -0.104 | 0.029 | -0.075 | 0.024 | -0.048 | 0.031 |
| rs1104608 | 16 | 73912588 | C | G | -0.011 | 0.001 | 1.05E-13 | 0.008 | 0.002 | 2.50E-04 | Alcohol | 0.007 | 0.010 | 0.010 | 0.015 | 0.003 | 0.015 | 0.005 | 0.013 | 0.023 | 0.016 |
| rs4548913 | 17 | 2209888 | A | G | -0.008 | 0.002 | 3.11E-08 | -0.002 | 0.002 | 3.20E-01 | Alcohol | -0.004 | 0.010 | -0.014 | 0.014 | 0.005 | 0.014 | -0.004 | 0.012 | 0.000 | 0.016 |
| rs3803800 | 17 | 7462969 | G | A | 0.011 | 0.002 | 1.50E-10 | 0.007 | 0.003 | 7.90E-03 | Alcohol | 0.018 | 0.012 | 0.010 | 0.017 | 0.018 | 0.017 | 0.019 | 0.014 | 0.008 | 0.019 |
| rs7209653 | 17 | 19882084 | T | C | 0.001 | 0.002 | 5.16E-01 | 0.016 | 0.002 | 9.10E-11 | PC | -0.006 | 0.011 | -0.010 | 0.015 | 0.001 | 0.015 | -0.016 | 0.013 | 0.011 | 0.017 |
| rs2854334 | 17 | 29715500 | G | A | 0.009 | 0.001 | 7.51E-10 | -0.002 | 0.002 | 3.40E-01 | Alcohol | -0.029 | 0.010 | -0.028 | 0.014 | -0.029 | 0.014 | -0.042 | 0.012 | -0.042 | 0.016 |
| rs12603813 | 17 | 43196584 | T | C | -0.002 | 0.002 | 3.69E-01 | 0.014 | 0.003 | 1.30E-08 | PC | -0.003 | 0.011 | -0.024 | 0.016 | 0.018 | 0.016 | 0.012 | 0.013 | -0.019 | 0.017 |
| rs66643547 | 17 | 79375315 | C | T | 0.001 | 0.002 | 4.71E-01 | -0.015 | 0.002 | 3.20E-11 | PC | 0.011 | 0.010 | 0.010 | 0.015 | 0.011 | 0.015 | 0.009 | 0.012 | 0.016 | 0.016 |
| rs784256 | 18 | 53398626 | G | A | 0.006 | 0.002 | 2.39E-02 | 0.019 | 0.003 | 6.20E-11 | PC | 0.011 | 0.013 | 0.010 | 0.018 | 0.013 | 0.018 | 0.006 | 0.015 | 0.006 | 0.020 |
| rs281379 | 19 | 49214274 | A | G | 0.014 | 0.001 | 4.91E-21 | 0.004 | 0.002 | 1.00E-01 | Alcohol | 0.010 | 0.010 | -0.004 | 0.014 | 0.025 | 0.014 | 0.019 | 0.012 | -0.004 | 0.015 |
| rs4815364 | 20 | 25035711 | A | G | 0.009 | 0.001 | 1.02E-08 | -0.001 | 0.002 | 5.80E-01 | Alcohol | -0.016 | 0.010 | -0.009 | 0.014 | -0.022 | 0.014 | -0.009 | 0.012 | -0.025 | 0.016 |
| rs6129084 | 20 | 59828857 | A | T | -0.007 | 0.002 | 5.53E-04 | -0.014 | 0.002 | 9.30E-10 | PC | 0.015 | 0.010 | 0.019 | 0.014 | 0.009 | 0.014 | 0.003 | 0.012 | 0.051 | 0.016 |
| rs136553 | 22 | 27255675 | C | T | 0.003 | 0.002 | 9.73E-02 | -0.015 | 0.002 | 2.30E-11 | PC | 0.014 | 0.010 | 0.024 | 0.014 | 0.005 | 0.014 | 0.007 | 0.012 | 0.011 | 0.016 |
| rs7288455 | 22 | 39966547 | A | G | 0.000 | 0.002 | 8.19E-01 | 0.013 | 0.002 | 4.00E-09 | PC | -0.002 | 0.010 | -0.004 | 0.014 | -0.004 | 0.014 | 0.006 | 0.012 | -0.010 | 0.016 |
| rs9607814 | 22 | 41946519 | A | C | -0.010 | 0.002 | 4.31E-08 | 0.005 | 0.003 | 8.80E-02 | Alcohol | 0.001 | 0.013 | -0.010 | 0.018 | 0.015 | 0.018 | -0.004 | 0.015 | 0.014 | 0.019 |

| **Table S12. Beta estimates for the SNPs included in the multivariable MR of alcohol consumption, television use, and breast cancer risk** | | | | | | | | | | | | | | | | | | | | | | | |
| --- | --- | --- | --- | --- | --- | --- | --- | --- | --- | --- | --- | --- | --- | --- | --- | --- | --- | --- | --- | --- | --- | --- | --- |
| SNP | Chr | POS | EA | OA | beta_  alc | se_  alc | pval_  alc | beta_  pc | se_pc | pval_pc | phenotype | beta_br | se_br | Lum_A_  beta | Lum_A_  se | Lum_B_  beta | Lum_B_  se | HER2Neg_  beta | HER2Neg_  se | HER2_  Enriched_beta | HER2_  Enriched_se | 3N_beta | 3N_se |
| rs705687 | 1 | 4548453 | G | A | -0.011 | 0.002 | 8.15E-10 | 0.002 | 0.003 | 5.30E-01 | Alcohol | 0.012 | 0.008 | 0.011 | 0.010 | -0.008 | 0.022 | 0.027 | 0.019 | 0.033 | 0.033 | 0.014 | 0.019 |
| rs58107686 | 1 | 33837334 | A | C | -0.010 | 0.002 | 7.79E-10 | 0.001 | 0.002 | 6.40E-01 | Alcohol | 0.000 | 0.006 | -0.008 | 0.008 | -0.017 | 0.019 | 0.036 | 0.016 | -0.002 | 0.027 | 0.000 | 0.016 |
| rs11810109 | 1 | 44186812 | A | T | 0.003 | 0.002 | 1.44E-01 | 0.016 | 0.002 | 5.40E-12 | TV | 0.005 | 0.007 | -0.001 | 0.009 | 0.020 | 0.019 | 0.016 | 0.016 | 0.000 | 0.028 | 0.026 | 0.017 |
| rs12725114 | 1 | 62432641 | G | A | 0.001 | 0.002 | 7.98E-01 | 0.015 | 0.003 | 1.60E-08 | TV | 0.007 | 0.007 | 0.016 | 0.010 | 0.029 | 0.022 | 0.002 | 0.018 | 0.040 | 0.032 | -0.004 | 0.019 |
| rs984409 | 1 | 67020440 | G | A | -0.002 | 0.002 | 2.14E-01 | -0.015 | 0.002 | 4.10E-11 | TV | -0.015 | 0.006 | -0.019 | 0.008 | -0.006 | 0.019 | -0.032 | 0.016 | 0.022 | 0.027 | -0.025 | 0.016 |
| rs5024204 | 1 | 71491890 | T | A | 0.010 | 0.002 | 2.55E-09 | -0.008 | 0.002 | 1.70E-03 | Alcohol | 0.003 | 0.007 | 0.001 | 0.009 | 0.013 | 0.020 | -0.023 | 0.017 | 0.008 | 0.029 | 0.018 | 0.017 |
| rs71658797 | 1 | 77967507 | T | A | 0.003 | 0.003 | 3.40E-01 | -0.020 | 0.003 | 6.30E-10 | TV | -0.004 | 0.010 | -0.003 | 0.013 | -0.034 | 0.029 | 0.059 | 0.025 | -0.038 | 0.042 | 0.002 | 0.025 |
| rs114328297 | 1 | 91190854 | T | G | 0.008 | 0.002 | 3.24E-04 | 0.014 | 0.003 | 4.40E-08 | TV | -0.006 | 0.008 | 0.001 | 0.010 | -0.021 | 0.023 | -0.019 | 0.020 | 0.053 | 0.034 | -0.033 | 0.020 |
| rs17379561 | 1 | 98340139 | A | T | -0.003 | 0.003 | 3.25E-01 | -0.025 | 0.003 | 1.10E-16 | TV | -0.002 | 0.009 | -0.001 | 0.011 | 0.052 | 0.025 | -0.029 | 0.021 | 0.010 | 0.036 | -0.008 | 0.021 |
| rs55909997 | 1 | 107581039 | G | A | -0.002 | 0.002 | 3.61E-01 | -0.014 | 0.002 | 2.40E-09 | TV | -0.002 | 0.006 | 0.002 | 0.008 | 0.016 | 0.019 | -0.024 | 0.016 | -0.005 | 0.027 | -0.024 | 0.016 |
| rs1278847 | 1 | 110037483 | C | A | 0.004 | 0.002 | 5.09E-02 | 0.016 | 0.002 | 1.20E-11 | TV | -0.012 | 0.007 | -0.016 | 0.009 | -0.008 | 0.019 | -0.004 | 0.017 | -0.017 | 0.028 | 0.011 | 0.017 |
| rs4845364 | 1 | 154141908 | A | G | -0.001 | 0.002 | 7.34E-01 | -0.015 | 0.002 | 1.30E-12 | TV | 0.005 | 0.006 | 0.007 | 0.008 | 0.002 | 0.018 | -0.002 | 0.015 | 0.018 | 0.026 | 0.020 | 0.015 |
| rs10753661 | 1 | 165119792 | A | G | -0.009 | 0.002 | 3.76E-08 | 0.002 | 0.002 | 3.00E-01 | Alcohol | 0.010 | 0.007 | 0.010 | 0.009 | 0.011 | 0.019 | 0.006 | 0.017 | -0.031 | 0.028 | 0.024 | 0.017 |
| rs28680958 | 1 | 173848808 | A | G | -0.011 | 0.002 | 5.13E-10 | 0.000 | 0.003 | 9.50E-01 | Alcohol | -0.004 | 0.007 | 0.000 | 0.010 | 0.024 | 0.021 | -0.012 | 0.018 | 0.026 | 0.031 | -0.004 | 0.018 |
| rs6673341 | 1 | 184655649 | T | G | -0.001 | 0.002 | 5.32E-01 | -0.015 | 0.002 | 2.20E-11 | TV | 0.000 | 0.006 | -0.006 | 0.008 | -0.004 | 0.018 | 0.007 | 0.015 | -0.005 | 0.026 | -0.026 | 0.015 |
| rs10737620 | 1 | 193057971 | T | A | 0.001 | 0.002 | 6.71E-01 | 0.014 | 0.002 | 2.60E-09 | TV | 0.011 | 0.007 | 0.022 | 0.009 | 0.017 | 0.020 | 0.012 | 0.017 | 0.012 | 0.029 | -0.017 | 0.017 |
| rs823114 | 1 | 205719532 | A | G | 0.009 | 0.001 | 2.31E-09 | -0.001 | 0.002 | 5.00E-01 | Alcohol | -0.012 | 0.006 | -0.013 | 0.008 | -0.010 | 0.018 | 0.000 | 0.015 | 0.001 | 0.026 | -0.028 | 0.015 |
| rs77165542 | 2 | 430975 | T | C | -0.026 | 0.004 | 5.63E-11 | 0.003 | 0.006 | 5.90E-01 | Alcohol | 0.004 | 0.020 | 0.024 | 0.026 | -0.027 | 0.058 | 0.070 | 0.048 | -0.068 | 0.085 | -0.007 | 0.050 |
| rs72781699 | 2 | 24277709 | G | A | 0.007 | 0.002 | 3.84E-03 | -0.019 | 0.003 | 3.00E-12 | TV | 0.011 | 0.008 | 0.006 | 0.010 | 0.016 | 0.022 | -0.015 | 0.019 | -0.007 | 0.032 | 0.043 | 0.019 |
| rs2178197 | 2 | 27860551 | G | A | -0.009 | 0.001 | 2.45E-09 | -0.007 | 0.002 | 6.30E-04 | Alcohol | -0.006 | 0.006 | -0.009 | 0.008 | -0.003 | 0.018 | -0.007 | 0.015 | -0.012 | 0.026 | 0.011 | 0.015 |
| rs12105701 | 2 | 41737200 | C | T | 0.000 | 0.002 | 9.36E-01 | -0.013 | 0.002 | 5.40E-09 | TV | -0.003 | 0.006 | -0.004 | 0.008 | -0.020 | 0.018 | 0.043 | 0.016 | 0.026 | 0.026 | -0.027 | 0.016 |
| rs1004787 | 2 | 45159091 | A | G | 0.008 | 0.001 | 8.40E-09 | 0.002 | 0.002 | 4.40E-01 | Alcohol | -0.001 | 0.006 | -0.005 | 0.008 | 0.047 | 0.018 | -0.008 | 0.015 | 0.002 | 0.026 | 0.002 | 0.015 |
| rs7564130 | 2 | 50606642 | T | C | -0.005 | 0.002 | 1.89E-02 | -0.015 | 0.002 | 2.80E-11 | TV | -0.002 | 0.006 | -0.010 | 0.008 | -0.016 | 0.018 | -0.005 | 0.016 | 0.005 | 0.026 | 0.006 | 0.016 |
| rs10189857 | 2 | 60713235 | A | G | 0.006 | 0.002 | 1.57E-03 | -0.020 | 0.002 | 6.20E-21 | TV | -0.017 | 0.006 | -0.019 | 0.008 | -0.011 | 0.018 | -0.022 | 0.015 | -0.008 | 0.026 | -0.015 | 0.015 |
| rs62641636 | 2 | 68425427 | A | G | 0.002 | 0.002 | 2.65E-01 | 0.014 | 0.002 | 7.00E-10 | TV | 0.001 | 0.007 | -0.003 | 0.009 | 0.039 | 0.019 | 0.009 | 0.016 | -0.042 | 0.028 | 0.016 | 0.017 |
| rs11689199 | 2 | 100820421 | A | G | 0.004 | 0.002 | 6.86E-02 | 0.018 | 0.002 | 5.50E-17 | TV | -0.010 | 0.006 | -0.008 | 0.008 | -0.016 | 0.018 | -0.007 | 0.016 | -0.014 | 0.027 | -0.017 | 0.016 |
| rs1451533 | 2 | 105466005 | G | A | 0.000 | 0.002 | 9.17E-01 | -0.016 | 0.002 | 1.10E-10 | TV | -0.004 | 0.007 | -0.015 | 0.009 | 0.032 | 0.021 | -0.001 | 0.018 | 0.018 | 0.031 | -0.005 | 0.018 |
| rs12476388 | 2 | 116330983 | C | T | 0.000 | 0.002 | 9.96E-01 | 0.013 | 0.002 | 2.10E-08 | TV | -0.002 | 0.007 | 0.002 | 0.009 | 0.009 | 0.019 | -0.004 | 0.017 | -0.035 | 0.028 | -0.014 | 0.017 |
| rs374722 | 2 | 147839830 | G | A | -0.005 | 0.003 | 8.71E-02 | 0.024 | 0.003 | 5.50E-16 | TV | 0.011 | 0.009 | 0.018 | 0.012 | 0.036 | 0.025 | -0.012 | 0.022 | -0.080 | 0.039 | 0.001 | 0.022 |
| rs3754970 | 2 | 162091836 | T | C | -0.006 | 0.002 | 1.14E-03 | -0.015 | 0.002 | 4.80E-12 | TV | 0.007 | 0.006 | 0.000 | 0.008 | -0.003 | 0.018 | 0.006 | 0.015 | -0.017 | 0.026 | 0.023 | 0.016 |
| rs2060199 | 2 | 166196681 | T | A | -0.003 | 0.002 | 1.82E-01 | 0.012 | 0.002 | 2.40E-08 | TV | -0.007 | 0.006 | -0.007 | 0.008 | 0.010 | 0.018 | 0.019 | 0.016 | 0.011 | 0.026 | -0.021 | 0.016 |
| rs4577309 | 2 | 191288833 | A | G | -0.001 | 0.002 | 7.79E-01 | 0.016 | 0.002 | 1.60E-13 | TV | -0.001 | 0.006 | 0.004 | 0.008 | -0.014 | 0.018 | 0.008 | 0.015 | -0.006 | 0.026 | -0.015 | 0.015 |
| rs34224261 | 2 | 193855452 | G | C | 0.004 | 0.002 | 3.27E-02 | -0.018 | 0.002 | 4.60E-16 | TV | 0.000 | 0.006 | -0.006 | 0.008 | 0.004 | 0.017 | -0.026 | 0.015 | -0.060 | 0.026 | -0.001 | 0.015 |
| rs4675246 | 2 | 202864487 | G | T | -0.005 | 0.002 | 3.61E-02 | -0.015 | 0.003 | 2.90E-08 | TV | 0.006 | 0.008 | 0.013 | 0.010 | -0.005 | 0.022 | 0.038 | 0.019 | -0.077 | 0.032 | -0.040 | 0.019 |
| rs13029509 | 2 | 215374209 | G | A | 0.001 | 0.002 | 5.73E-01 | -0.018 | 0.002 | 2.00E-17 | TV | -0.009 | 0.006 | -0.008 | 0.008 | 0.008 | 0.018 | -0.019 | 0.015 | 0.017 | 0.026 | -0.017 | 0.015 |
| rs10932837 | 2 | 221044785 | C | T | 0.000 | 0.002 | 9.44E-01 | -0.013 | 0.002 | 1.30E-09 | TV | 0.001 | 0.006 | 0.003 | 0.008 | -0.011 | 0.018 | 0.009 | 0.015 | -0.023 | 0.026 | 0.000 | 0.015 |
| rs4973576 | 2 | 233806771 | C | A | 0.005 | 0.002 | 3.73E-02 | -0.015 | 0.002 | 1.00E-09 | TV | 0.012 | 0.007 | 0.022 | 0.009 | 0.031 | 0.020 | -0.015 | 0.017 | 0.018 | 0.029 | 0.026 | 0.017 |
| rs138256022 | 3 | 11603090 | C | T | 0.004 | 0.005 | 4.32E-01 | -0.031 | 0.006 | 2.90E-08 | TV | -0.016 | 0.017 | -0.025 | 0.023 | -0.024 | 0.050 | -0.043 | 0.043 | -0.061 | 0.072 | -0.005 | 0.044 |
| rs9834970 | 3 | 36856030 | T | C | -0.001 | 0.002 | 5.90E-01 | 0.013 | 0.002 | 3.30E-09 | TV | 0.001 | 0.006 | 0.001 | 0.008 | 0.005 | 0.018 | 0.004 | 0.016 | -0.035 | 0.027 | 0.011 | 0.016 |
| rs3796386 | 3 | 49899795 | G | A | 0.001 | 0.002 | 5.67E-01 | -0.026 | 0.002 | 3.20E-33 | TV | -0.018 | 0.006 | -0.014 | 0.008 | -0.038 | 0.018 | -0.012 | 0.015 | -0.063 | 0.026 | -0.043 | 0.015 |
| rs11130793 | 3 | 60884659 | C | T | -0.002 | 0.002 | 2.82E-01 | 0.013 | 0.002 | 5.00E-09 | TV | 0.001 | 0.006 | 0.006 | 0.008 | 0.012 | 0.018 | -0.021 | 0.016 | 0.004 | 0.027 | 0.002 | 0.016 |
| rs11714337 | 3 | 71582521 | G | A | -0.006 | 0.002 | 3.41E-03 | 0.014 | 0.002 | 4.70E-11 | TV | 0.032 | 0.006 | 0.045 | 0.008 | 0.036 | 0.018 | 0.029 | 0.015 | 0.000 | 0.026 | 0.014 | 0.015 |
| rs6797840 | 3 | 85656569 | A | C | -0.007 | 0.002 | 3.54E-04 | -0.016 | 0.002 | 1.70E-13 | TV | -0.004 | 0.006 | -0.007 | 0.008 | -0.015 | 0.018 | -0.022 | 0.015 | 0.024 | 0.026 | 0.005 | 0.015 |
| rs2034768 | 3 | 93986371 | A | G | 0.005 | 0.002 | 6.70E-03 | 0.015 | 0.002 | 8.60E-12 | TV | -0.003 | 0.006 | -0.006 | 0.008 | 0.010 | 0.018 | 0.016 | 0.015 | 0.016 | 0.025 | -0.025 | 0.015 |
| rs9867121 | 3 | 114631548 | C | A | 0.004 | 0.002 | 9.28E-02 | 0.019 | 0.003 | 3.90E-12 | TV | 0.013 | 0.008 | 0.015 | 0.010 | 0.020 | 0.023 | 0.001 | 0.020 | 0.052 | 0.034 | 0.009 | 0.020 |
| rs9838144 | 3 | 131576287 | C | G | -0.010 | 0.002 | 2.65E-08 | 0.005 | 0.003 | 8.80E-02 | Alcohol | 0.000 | 0.008 | -0.005 | 0.010 | -0.027 | 0.023 | -0.002 | 0.019 | -0.003 | 0.033 | 0.014 | 0.019 |
| rs60654199 | 3 | 141267295 | A | C | -0.017 | 0.003 | 2.85E-08 | 0.013 | 0.004 | 1.90E-03 | Alcohol | 0.006 | 0.012 | 0.011 | 0.016 | 0.037 | 0.035 | -0.024 | 0.031 | -0.092 | 0.054 | 0.056 | 0.030 |
| rs6787172 | 3 | 158187811 | G | T | -0.008 | 0.001 | 4.27E-08 | 0.001 | 0.002 | 7.40E-01 | Alcohol | -0.020 | 0.006 | -0.016 | 0.008 | -0.044 | 0.018 | -0.030 | 0.015 | -0.035 | 0.025 | -0.002 | 0.015 |
| rs12491503 | 3 | 165706855 | G | A | 0.001 | 0.002 | 6.46E-01 | -0.014 | 0.002 | 5.50E-10 | TV | -0.001 | 0.007 | 0.008 | 0.009 | -0.018 | 0.019 | -0.029 | 0.016 | -0.025 | 0.028 | 0.009 | 0.017 |
| rs114600294 | 3 | 181419367 | G | C | 0.006 | 0.002 | 2.37E-02 | -0.016 | 0.003 | 7.90E-10 | TV | 0.017 | 0.010 | 0.002 | 0.013 | 0.029 | 0.028 | -0.015 | 0.024 | 0.035 | 0.042 | 0.007 | 0.025 |
| rs66852340 | 4 | 3311070 | C | T | 0.004 | 0.002 | 8.69E-02 | -0.018 | 0.003 | 7.90E-12 | TV | -0.016 | 0.007 | -0.019 | 0.009 | 0.027 | 0.021 | -0.046 | 0.018 | -0.011 | 0.031 | -0.022 | 0.018 |
| rs7700107 | 4 | 17880416 | A | C | -0.002 | 0.003 | 4.30E-01 | -0.021 | 0.003 | 1.90E-11 | TV | 0.005 | 0.009 | 0.033 | 0.012 | -0.019 | 0.025 | -0.026 | 0.022 | -0.066 | 0.036 | -0.009 | 0.022 |
| rs11940694 | 4 | 39414993 | G | A | 0.026 | 0.001 | 3.03E-68 | 0.004 | 0.002 | 9.50E-02 | Alcohol | -0.008 | 0.006 | -0.009 | 0.008 | -0.045 | 0.018 | -0.009 | 0.016 | -0.005 | 0.027 | -0.005 | 0.016 |
| rs28971796 | 4 | 49151982 | A | G | -0.004 | 0.002 | 5.96E-02 | 0.015 | 0.002 | 2.10E-09 | TV | 0.010 | 0.009 | 0.017 | 0.012 | 0.042 | 0.026 | 0.005 | 0.022 | 0.049 | 0.037 | 0.017 | 0.022 |
| rs6850494 | 4 | 82291771 | A | C | -0.001 | 0.002 | 6.62E-01 | -0.014 | 0.002 | 1.10E-10 | TV | 0.009 | 0.006 | 0.004 | 0.008 | 0.030 | 0.018 | 0.002 | 0.015 | -0.045 | 0.026 | 0.020 | 0.016 |
| rs1229984 | 4 | 100239319 | C | T | 0.151 | 0.004 | 0.00E+00 | 0.023 | 0.007 | 2.00E-03 | Alcohol | -0.023 | 0.016 | -0.010 | 0.021 | -0.105 | 0.042 | -0.017 | 0.038 | 0.080 | 0.064 | -0.033 | 0.037 |
| rs2165670 | 4 | 100286085 | A | G | 0.023 | 0.002 | 1.67E-22 | 0.004 | 0.004 | 3.30E-01 | Alcohol | 0.023 | 0.010 | 0.039 | 0.013 | 0.017 | 0.029 | 0.025 | 0.025 | -0.026 | 0.043 | 0.006 | 0.025 |
| rs13107325 | 4 | 103188709 | T | C | -0.028 | 0.003 | 1.53E-22 | 0.029 | 0.004 | 1.50E-12 | both | 0.019 | 0.012 | 0.021 | 0.016 | 0.051 | 0.034 | 0.007 | 0.030 | -0.051 | 0.051 | 0.025 | 0.030 |
| rs7693703 | 4 | 118357405 | G | A | -0.008 | 0.003 | 1.32E-02 | 0.023 | 0.004 | 2.70E-09 | TV | -0.007 | 0.010 | -0.015 | 0.014 | -0.012 | 0.030 | 0.009 | 0.026 | 0.052 | 0.046 | 0.005 | 0.026 |
| rs4334769 | 4 | 130245233 | G | T | -0.003 | 0.002 | 1.23E-01 | 0.012 | 0.002 | 2.10E-08 | TV | -0.008 | 0.006 | -0.007 | 0.008 | -0.016 | 0.018 | -0.032 | 0.015 | 0.012 | 0.026 | 0.015 | 0.015 |
| rs4690727 | 4 | 143648579 | G | C | 0.011 | 0.002 | 2.43E-11 | 0.001 | 0.002 | 7.90E-01 | Alcohol | 0.009 | 0.007 | 0.013 | 0.009 | -0.028 | 0.020 | 0.021 | 0.017 | 0.039 | 0.030 | 0.039 | 0.018 |
| rs6825241 | 4 | 152543783 | C | A | 0.002 | 0.002 | 2.41E-01 | -0.017 | 0.002 | 4.90E-15 | TV | 0.002 | 0.006 | 0.009 | 0.008 | -0.008 | 0.018 | -0.018 | 0.015 | 0.008 | 0.026 | 0.006 | 0.016 |
| rs7693082 | 4 | 159857819 | G | C | 0.003 | 0.002 | 1.08E-01 | 0.015 | 0.002 | 1.70E-10 | TV | -0.004 | 0.007 | -0.013 | 0.009 | -0.023 | 0.020 | -0.001 | 0.017 | 0.044 | 0.028 | -0.013 | 0.017 |
| rs12651313 | 4 | 171086393 | G | C | -0.009 | 0.001 | 3.79E-09 | 0.006 | 0.002 | 3.10E-03 | Alcohol | -0.004 | 0.006 | 0.002 | 0.008 | 0.010 | 0.018 | -0.005 | 0.016 | 0.030 | 0.026 | 0.011 | 0.016 |
| rs10940659 | 5 | 59360341 | A | G | 0.005 | 0.002 | 1.36E-02 | -0.013 | 0.002 | 1.50E-08 | TV | 0.008 | 0.006 | 0.020 | 0.008 | -0.061 | 0.018 | 0.014 | 0.015 | -0.028 | 0.026 | 0.003 | 0.015 |
| rs262890 | 5 | 62930015 | A | G | -0.003 | 0.002 | 1.60E-01 | -0.019 | 0.002 | 3.20E-15 | TV | -0.003 | 0.007 | -0.002 | 0.009 | 0.032 | 0.019 | -0.006 | 0.016 | -0.021 | 0.028 | -0.008 | 0.017 |
| rs7716447 | 5 | 88800331 | A | G | 0.001 | 0.002 | 7.36E-01 | -0.013 | 0.002 | 3.60E-09 | TV | 0.007 | 0.007 | 0.009 | 0.009 | -0.013 | 0.019 | 0.011 | 0.016 | 0.034 | 0.028 | -0.011 | 0.017 |
| rs1031423 | 5 | 93276883 | T | C | 0.003 | 0.002 | 2.70E-01 | -0.019 | 0.003 | 1.80E-12 | TV | -0.002 | 0.008 | -0.007 | 0.010 | 0.013 | 0.022 | 0.013 | 0.019 | -0.010 | 0.032 | 0.021 | 0.019 |
| rs57585211 | 5 | 107197725 | T | G | 0.004 | 0.003 | 1.71E-01 | -0.017 | 0.003 | 4.30E-09 | TV | 0.001 | 0.008 | 0.000 | 0.011 | 0.021 | 0.024 | -0.036 | 0.020 | 0.044 | 0.035 | -0.026 | 0.020 |
| rs9326862 | 5 | 112054547 | A | T | 0.002 | 0.002 | 2.05E-01 | 0.012 | 0.002 | 2.60E-08 | TV | 0.005 | 0.006 | 0.008 | 0.008 | 0.008 | 0.018 | -0.002 | 0.015 | 0.025 | 0.026 | 0.005 | 0.015 |
| rs10041724 | 5 | 124273520 | T | C | -0.001 | 0.002 | 8.33E-01 | 0.018 | 0.003 | 3.90E-11 | TV | 0.008 | 0.008 | 0.006 | 0.010 | 0.020 | 0.022 | -0.011 | 0.019 | 0.065 | 0.033 | -0.021 | 0.019 |
| rs62379379 | 5 | 141082015 | G | T | 0.001 | 0.004 | 8.54E-01 | -0.026 | 0.004 | 7.80E-10 | TV | -0.001 | 0.012 | -0.014 | 0.016 | 0.074 | 0.037 | 0.019 | 0.031 | 0.122 | 0.055 | -0.031 | 0.030 |
| rs10054327 | 5 | 147871137 | G | A | -0.002 | 0.002 | 3.26E-01 | 0.017 | 0.002 | 3.40E-15 | TV | -0.010 | 0.006 | -0.013 | 0.008 | 0.000 | 0.018 | 0.004 | 0.015 | 0.003 | 0.026 | -0.022 | 0.016 |
| rs42210 | 5 | 166408788 | G | C | -0.002 | 0.002 | 3.85E-01 | -0.014 | 0.002 | 7.30E-09 | TV | -0.020 | 0.007 | -0.008 | 0.010 | -0.046 | 0.021 | -0.046 | 0.018 | -0.083 | 0.031 | -0.019 | 0.018 |
| rs72828890 | 5 | 167806836 | C | T | 0.004 | 0.003 | 1.27E-01 | 0.019 | 0.003 | 4.90E-09 | TV | -0.005 | 0.011 | 0.006 | 0.014 | -0.010 | 0.032 | 0.027 | 0.028 | 0.002 | 0.047 | 0.012 | 0.028 |
| rs72834698 | 6 | 26176517 | G | A | 0.001 | 0.003 | 7.42E-01 | 0.023 | 0.003 | 2.70E-13 | TV | 0.007 | 0.009 | 0.019 | 0.012 | -0.001 | 0.026 | -0.022 | 0.022 | 0.030 | 0.038 | 0.019 | 0.023 |
| rs9471333 | 6 | 40362023 | C | T | 0.002 | 0.002 | 2.09E-01 | 0.013 | 0.002 | 1.50E-09 | TV | 0.000 | 0.006 | 0.012 | 0.009 | -0.008 | 0.019 | -0.039 | 0.016 | -0.044 | 0.027 | 0.011 | 0.016 |
| rs4523073 | 6 | 67550288 | A | G | -0.005 | 0.002 | 1.59E-02 | -0.014 | 0.002 | 5.00E-10 | TV | -0.002 | 0.006 | -0.004 | 0.008 | 0.025 | 0.018 | -0.006 | 0.016 | -0.003 | 0.027 | 0.008 | 0.016 |
| rs6905544 | 6 | 98411631 | A | G | 0.008 | 0.002 | 8.95E-05 | -0.019 | 0.002 | 8.50E-18 | TV | -0.011 | 0.006 | -0.016 | 0.008 | -0.007 | 0.018 | -0.012 | 0.015 | 0.005 | 0.026 | -0.012 | 0.016 |
| rs17789218 | 6 | 100600097 | T | C | 0.003 | 0.002 | 2.33E-01 | 0.019 | 0.003 | 1.40E-13 | TV | -0.013 | 0.008 | -0.025 | 0.011 | -0.002 | 0.023 | 0.030 | 0.020 | 0.068 | 0.034 | 0.014 | 0.020 |
| rs2184364 | 6 | 143183596 | A | G | 0.006 | 0.002 | 1.69E-02 | 0.016 | 0.003 | 3.00E-09 | TV | 0.007 | 0.007 | 0.009 | 0.010 | 0.009 | 0.022 | -0.004 | 0.018 | -0.035 | 0.031 | 0.023 | 0.019 |
| rs9718104 | 6 | 166170539 | T | G | 0.013 | 0.004 | 1.68E-03 | -0.041 | 0.005 | 9.30E-19 | TV | -0.010 | 0.014 | -0.009 | 0.018 | 0.009 | 0.041 | 0.004 | 0.034 | 0.041 | 0.060 | -0.054 | 0.034 |
| rs17568389 | 7 | 8091876 | T | A | 0.004 | 0.002 | 4.20E-02 | 0.015 | 0.002 | 8.60E-13 | TV | -0.003 | 0.006 | -0.008 | 0.008 | -0.012 | 0.018 | -0.014 | 0.015 | 0.038 | 0.026 | 0.010 | 0.016 |
| rs10234444 | 7 | 41828527 | G | A | 0.002 | 0.003 | 5.06E-01 | 0.016 | 0.003 | 2.90E-08 | TV | 0.011 | 0.008 | 0.001 | 0.011 | 0.031 | 0.025 | 0.005 | 0.021 | -0.069 | 0.035 | 0.021 | 0.022 |
| rs6460047 | 7 | 73042443 | C | T | 0.012 | 0.002 | 9.69E-11 | 0.000 | 0.003 | 9.50E-01 | Alcohol | 0.002 | 0.008 | -0.002 | 0.011 | -0.021 | 0.023 | 0.008 | 0.020 | -0.003 | 0.034 | 0.011 | 0.020 |
| rs6973656 | 7 | 77422583 | A | G | -0.001 | 0.002 | 6.44E-01 | -0.013 | 0.002 | 1.00E-09 | TV | 0.000 | 0.006 | 0.006 | 0.009 | -0.013 | 0.019 | -0.019 | 0.016 | -0.013 | 0.027 | 0.003 | 0.016 |
| rs973734 | 7 | 89387578 | C | A | 0.004 | 0.003 | 1.88E-01 | 0.017 | 0.003 | 1.50E-08 | TV | 0.013 | 0.009 | 0.015 | 0.011 | 0.032 | 0.025 | 0.015 | 0.021 | 0.017 | 0.036 | 0.023 | 0.022 |
| rs10236149 | 7 | 98977515 | G | A | -0.013 | 0.002 | 1.18E-09 | -0.014 | 0.003 | 1.40E-05 | Alcohol | 0.001 | 0.009 | 0.017 | 0.012 | 0.002 | 0.026 | 0.011 | 0.022 | -0.025 | 0.038 | -0.029 | 0.023 |
| rs62471080 | 7 | 109128060 | G | C | -0.001 | 0.002 | 7.32E-01 | -0.013 | 0.002 | 3.70E-09 | TV | 0.005 | 0.006 | 0.006 | 0.008 | 0.027 | 0.018 | 0.008 | 0.015 | 0.004 | 0.026 | -0.004 | 0.015 |
| rs10246289 | 7 | 115544763 | A | G | 0.001 | 0.003 | 7.01E-01 | 0.019 | 0.003 | 1.70E-08 | TV | 0.021 | 0.009 | 0.018 | 0.012 | 0.021 | 0.027 | 0.014 | 0.024 | 0.032 | 0.040 | 0.048 | 0.024 |
| rs11763734 | 7 | 126400910 | A | C | -0.005 | 0.002 | 5.86E-03 | -0.012 | 0.002 | 8.20E-09 | TV | 0.003 | 0.006 | -0.003 | 0.008 | 0.011 | 0.018 | 0.009 | 0.015 | -0.006 | 0.026 | 0.024 | 0.015 |
| rs6951574 | 7 | 153489744 | C | T | 0.013 | 0.001 | 1.58E-19 | 0.004 | 0.002 | 7.20E-02 | Alcohol | 0.007 | 0.006 | 0.010 | 0.008 | -0.019 | 0.018 | 0.027 | 0.016 | -0.055 | 0.027 | 0.038 | 0.016 |
| rs7834121 | 8 | 10770255 | G | T | -0.005 | 0.002 | 1.89E-02 | -0.014 | 0.002 | 1.10E-10 | TV | -0.008 | 0.007 | -0.009 | 0.009 | 0.008 | 0.021 | 0.005 | 0.017 | -0.013 | 0.028 | -0.008 | 0.018 |
| rs13250583 | 8 | 20949917 | T | C | -0.010 | 0.002 | 4.70E-08 | 0.002 | 0.003 | 4.00E-01 | Alcohol | -0.012 | 0.007 | -0.011 | 0.010 | -0.029 | 0.022 | 0.014 | 0.018 | -0.060 | 0.032 | 0.024 | 0.019 |
| rs1421334 | 8 | 30865733 | A | C | -0.005 | 0.002 | 9.72E-03 | 0.017 | 0.002 | 2.20E-15 | TV | 0.005 | 0.006 | 0.002 | 0.008 | 0.031 | 0.018 | -0.006 | 0.015 | 0.018 | 0.026 | 0.009 | 0.016 |
| rs6996198 | 8 | 65463442 | C | T | 0.007 | 0.003 | 1.30E-02 | -0.016 | 0.003 | 3.50E-08 | TV | -0.001 | 0.008 | -0.001 | 0.011 | -0.002 | 0.024 | -0.002 | 0.020 | 0.065 | 0.036 | -0.036 | 0.020 |
| rs6472942 | 8 | 76827190 | T | C | 0.000 | 0.002 | 8.98E-01 | -0.013 | 0.002 | 1.80E-09 | TV | -0.012 | 0.006 | -0.020 | 0.008 | 0.023 | 0.018 | -0.013 | 0.015 | -0.031 | 0.025 | -0.006 | 0.015 |
| rs11985394 | 8 | 92667521 | T | A | 0.005 | 0.002 | 1.63E-02 | 0.012 | 0.002 | 1.30E-08 | TV | 0.005 | 0.006 | 0.010 | 0.008 | 0.002 | 0.018 | 0.005 | 0.015 | -0.012 | 0.026 | 0.001 | 0.016 |
| rs12541615 | 8 | 118870151 | T | C | -0.002 | 0.002 | 3.69E-01 | -0.018 | 0.003 | 3.90E-10 | TV | 0.029 | 0.008 | 0.037 | 0.011 | 0.035 | 0.023 | 0.048 | 0.020 | 0.039 | 0.034 | 0.004 | 0.020 |
| rs2717559 | 8 | 143882420 | A | G | -0.001 | 0.002 | 5.86E-01 | 0.012 | 0.002 | 1.50E-08 | TV | 0.000 | 0.007 | -0.009 | 0.009 | 0.018 | 0.019 | -0.022 | 0.016 | 0.030 | 0.028 | -0.003 | 0.017 |
| rs2616830 | 9 | 1721385 | G | A | -0.004 | 0.002 | 3.60E-02 | 0.016 | 0.002 | 2.90E-14 | TV | 0.003 | 0.006 | 0.006 | 0.008 | -0.001 | 0.018 | 0.012 | 0.015 | -0.052 | 0.026 | 0.001 | 0.015 |
| rs34864022 | 9 | 22609110 | A | G | 0.011 | 0.004 | 4.36E-03 | -0.026 | 0.004 | 1.40E-09 | TV | 0.008 | 0.013 | 0.002 | 0.017 | 0.001 | 0.038 | 0.043 | 0.032 | -0.046 | 0.054 | 0.011 | 0.033 |
| rs12554512 | 9 | 23352293 | T | C | 0.000 | 0.002 | 9.84E-01 | 0.021 | 0.002 | 3.80E-21 | TV | -0.006 | 0.006 | -0.001 | 0.008 | -0.038 | 0.019 | -0.006 | 0.016 | 0.017 | 0.027 | -0.018 | 0.016 |
| rs2791574 | 9 | 82212094 | T | G | 0.004 | 0.002 | 4.65E-02 | -0.013 | 0.002 | 3.00E-08 | TV | 0.003 | 0.006 | 0.011 | 0.008 | 0.009 | 0.018 | -0.004 | 0.016 | 0.026 | 0.027 | -0.029 | 0.016 |
| rs870151 | 9 | 92230747 | T | A | 0.005 | 0.002 | 1.14E-02 | -0.016 | 0.002 | 8.80E-13 | TV | 0.005 | 0.006 | -0.008 | 0.008 | 0.014 | 0.018 | -0.005 | 0.015 | 0.043 | 0.026 | 0.021 | 0.015 |
| rs2787374 | 9 | 103054951 | T | C | 0.001 | 0.002 | 6.60E-01 | 0.012 | 0.002 | 1.20E-08 | TV | -0.004 | 0.006 | -0.010 | 0.008 | 0.001 | 0.018 | 0.042 | 0.015 | -0.015 | 0.026 | 0.000 | 0.015 |
| rs7043521 | 9 | 124602779 | A | T | 0.001 | 0.002 | 7.61E-01 | 0.014 | 0.002 | 6.50E-11 | TV | 0.003 | 0.006 | 0.003 | 0.008 | -0.019 | 0.018 | 0.016 | 0.015 | 0.037 | 0.026 | -0.019 | 0.015 |
| rs4382592 | 9 | 134870755 | T | G | 0.002 | 0.002 | 3.75E-01 | 0.014 | 0.002 | 6.70E-09 | TV | 0.011 | 0.007 | 0.004 | 0.009 | 0.030 | 0.019 | 0.002 | 0.016 | 0.045 | 0.028 | 0.006 | 0.017 |
| rs2073869 | 9 | 135763816 | C | T | 0.001 | 0.003 | 6.77E-01 | 0.019 | 0.003 | 1.50E-10 | TV | 0.011 | 0.008 | 0.001 | 0.011 | 0.012 | 0.023 | 0.029 | 0.020 | 0.019 | 0.034 | -0.013 | 0.020 |
| rs1623025 | 10 | 11164813 | G | C | -0.004 | 0.002 | 6.59E-02 | -0.012 | 0.002 | 1.40E-08 | TV | -0.009 | 0.006 | -0.012 | 0.008 | -0.011 | 0.018 | -0.013 | 0.015 | -0.044 | 0.026 | 0.000 | 0.015 |
| rs1243182 | 10 | 21916728 | C | T | 0.000 | 0.002 | 9.78E-01 | -0.019 | 0.002 | 2.00E-15 | TV | -0.056 | 0.006 | -0.081 | 0.008 | -0.059 | 0.019 | -0.051 | 0.016 | -0.016 | 0.027 | 0.042 | 0.016 |
| rs2045147 | 10 | 56660437 | A | G | 0.004 | 0.002 | 3.23E-02 | 0.013 | 0.002 | 5.90E-09 | TV | 0.002 | 0.006 | -0.002 | 0.008 | 0.023 | 0.018 | 0.032 | 0.015 | 0.045 | 0.026 | 0.001 | 0.015 |
| rs10994943 | 10 | 63591413 | T | G | 0.001 | 0.002 | 4.50E-01 | 0.013 | 0.002 | 3.10E-09 | TV | 0.011 | 0.006 | 0.010 | 0.008 | -0.009 | 0.018 | 0.015 | 0.015 | 0.028 | 0.026 | 0.032 | 0.015 |
| rs11201422 | 10 | 86924483 | T | C | -0.003 | 0.002 | 2.21E-01 | 0.013 | 0.002 | 3.40E-08 | TV | -0.015 | 0.007 | -0.007 | 0.009 | -0.025 | 0.019 | -0.027 | 0.016 | -0.059 | 0.028 | 0.007 | 0.017 |
| rs10786658 | 10 | 103737193 | A | T | 0.001 | 0.002 | 6.64E-01 | -0.014 | 0.002 | 4.60E-11 | TV | 0.001 | 0.006 | 0.002 | 0.008 | -0.032 | 0.018 | 0.022 | 0.015 | -0.011 | 0.026 | 0.014 | 0.016 |
| rs7089973 | 10 | 116569565 | C | A | 0.005 | 0.002 | 2.30E-02 | -0.013 | 0.002 | 2.40E-09 | TV | -0.005 | 0.006 | -0.008 | 0.009 | 0.025 | 0.019 | -0.007 | 0.016 | -0.045 | 0.027 | 0.002 | 0.016 |
| rs17665139 | 10 | 125093880 | T | C | -0.012 | 0.002 | 1.59E-08 | -0.006 | 0.003 | 3.30E-02 | Alcohol | -0.002 | 0.008 | -0.009 | 0.011 | 0.019 | 0.024 | -0.007 | 0.021 | -0.053 | 0.037 | 0.005 | 0.021 |
| rs11245482 | 10 | 126733546 | T | C | 0.002 | 0.002 | 4.27E-01 | -0.013 | 0.002 | 2.60E-09 | TV | -0.005 | 0.006 | -0.003 | 0.008 | -0.037 | 0.018 | 0.004 | 0.015 | -0.016 | 0.026 | 0.022 | 0.016 |
| rs17727474 | 10 | 127182959 | C | T | 0.006 | 0.003 | 1.28E-02 | 0.018 | 0.003 | 3.10E-09 | TV | -0.013 | 0.008 | -0.020 | 0.011 | 0.021 | 0.025 | -0.048 | 0.021 | -0.078 | 0.035 | 0.025 | 0.022 |
| rs7950166 | 11 | 8642218 | T | C | -0.010 | 0.002 | 9.89E-11 | 0.003 | 0.002 | 1.70E-01 | Alcohol | 0.002 | 0.006 | 0.008 | 0.008 | 0.006 | 0.018 | -0.011 | 0.015 | 0.018 | 0.026 | -0.003 | 0.016 |
| rs12289262 | 11 | 12894758 | C | T | 0.000 | 0.002 | 8.95E-01 | -0.014 | 0.002 | 2.00E-08 | TV | 0.002 | 0.007 | 0.008 | 0.009 | -0.064 | 0.021 | -0.013 | 0.018 | -0.001 | 0.030 | 0.012 | 0.018 |
| rs11030084 | 11 | 27643725 | T | C | -0.011 | 0.002 | 1.72E-08 | 0.004 | 0.003 | 1.70E-01 | Alcohol | -0.010 | 0.008 | -0.005 | 0.010 | -0.010 | 0.022 | 0.006 | 0.019 | -0.012 | 0.033 | -0.004 | 0.019 |
| rs56030824 | 11 | 47397353 | A | G | -0.012 | 0.002 | 1.15E-13 | 0.005 | 0.002 | 2.00E-02 | Alcohol | 0.004 | 0.006 | -0.006 | 0.008 | 0.000 | 0.019 | -0.001 | 0.016 | -0.001 | 0.027 | -0.007 | 0.016 |
| rs801733 | 11 | 65934549 | A | C | -0.004 | 0.002 | 3.86E-02 | 0.017 | 0.002 | 7.30E-14 | TV | 0.023 | 0.006 | 0.031 | 0.008 | 0.016 | 0.019 | 0.001 | 0.016 | 0.057 | 0.027 | -0.002 | 0.016 |
| rs116175338 | 11 | 72467875 | G | T | 0.003 | 0.002 | 1.36E-01 | -0.014 | 0.003 | 1.40E-08 | TV | -0.017 | 0.007 | -0.028 | 0.009 | 0.007 | 0.021 | -0.004 | 0.018 | -0.016 | 0.030 | -0.025 | 0.018 |
| rs12272012 | 11 | 84861286 | G | C | 0.000 | 0.005 | 9.22E-01 | -0.030 | 0.005 | 1.90E-08 | TV | -0.023 | 0.015 | -0.017 | 0.019 | 0.098 | 0.046 | -0.003 | 0.037 | -0.045 | 0.063 | -0.040 | 0.037 |
| rs11020045 | 11 | 92487907 | A | C | 0.000 | 0.002 | 8.94E-01 | -0.013 | 0.002 | 1.60E-08 | TV | -0.009 | 0.006 | -0.003 | 0.008 | 0.000 | 0.019 | -0.017 | 0.016 | -0.019 | 0.027 | -0.022 | 0.016 |
| rs17207890 | 11 | 95490754 | G | A | 0.002 | 0.002 | 2.74E-01 | 0.016 | 0.002 | 6.70E-12 | TV | 0.017 | 0.006 | 0.033 | 0.008 | 0.000 | 0.019 | 0.039 | 0.016 | -0.011 | 0.027 | 0.019 | 0.016 |
| rs10750025 | 11 | 113424042 | T | C | 0.010 | 0.002 | 4.89E-11 | -0.005 | 0.002 | 4.40E-02 | Alcohol | 0.000 | 0.007 | 0.011 | 0.009 | -0.001 | 0.019 | -0.017 | 0.016 | -0.001 | 0.028 | -0.035 | 0.016 |
| rs682011 | 11 | 121544285 | C | T | 0.008 | 0.001 | 2.22E-08 | 0.008 | 0.002 | 2.70E-04 | Alcohol | 0.001 | 0.006 | -0.003 | 0.008 | 0.015 | 0.018 | 0.018 | 0.015 | -0.019 | 0.026 | 0.004 | 0.015 |
| rs11218575 | 11 | 122154576 | C | T | 0.000 | 0.002 | 9.54E-01 | 0.015 | 0.002 | 2.10E-12 | TV | 0.009 | 0.006 | 0.000 | 0.009 | 0.024 | 0.019 | 0.001 | 0.016 | 0.063 | 0.027 | 0.008 | 0.016 |
| rs12795042 | 11 | 133658168 | C | A | -0.008 | 0.002 | 3.25E-08 | 0.001 | 0.002 | 6.70E-01 | Alcohol | -0.009 | 0.006 | -0.006 | 0.008 | -0.021 | 0.019 | -0.023 | 0.016 | 0.010 | 0.027 | -0.022 | 0.016 |
| rs4937842 | 11 | 133734542 | G | C | 0.002 | 0.002 | 3.05E-01 | -0.013 | 0.002 | 1.40E-08 | TV | 0.009 | 0.006 | 0.012 | 0.008 | -0.005 | 0.018 | 0.017 | 0.016 | 0.020 | 0.027 | 0.010 | 0.016 |
| rs10772643 | 12 | 13415288 | C | T | 0.003 | 0.003 | 2.82E-01 | 0.025 | 0.003 | 1.30E-12 | TV | 0.008 | 0.010 | 0.022 | 0.013 | -0.012 | 0.030 | -0.005 | 0.025 | -0.044 | 0.044 | -0.017 | 0.026 |
| rs10771746 | 12 | 30791864 | C | T | 0.001 | 0.002 | 6.00E-01 | -0.014 | 0.002 | 2.50E-09 | TV | -0.024 | 0.007 | -0.015 | 0.009 | -0.037 | 0.019 | -0.053 | 0.016 | -0.018 | 0.028 | 0.013 | 0.017 |
| rs10876188 | 12 | 51895882 | T | C | -0.008 | 0.001 | 4.84E-08 | -0.006 | 0.002 | 7.10E-03 | Alcohol | 0.000 | 0.006 | -0.006 | 0.008 | 0.029 | 0.018 | -0.029 | 0.015 | -0.008 | 0.025 | 0.008 | 0.015 |
| rs10876864 | 12 | 56401085 | G | A | 0.004 | 0.002 | 3.26E-02 | -0.013 | 0.002 | 1.00E-09 | TV | 0.004 | 0.006 | 0.008 | 0.008 | 0.040 | 0.018 | -0.011 | 0.015 | 0.008 | 0.026 | -0.024 | 0.015 |
| rs8756 | 12 | 66359752 | C | A | 0.003 | 0.002 | 9.39E-02 | -0.013 | 0.002 | 5.30E-10 | TV | -0.003 | 0.006 | 0.004 | 0.008 | 0.009 | 0.018 | -0.007 | 0.015 | 0.019 | 0.025 | -0.023 | 0.015 |
| rs10506274 | 12 | 81601464 | T | G | -0.009 | 0.001 | 5.78E-10 | 0.003 | 0.002 | 1.90E-01 | Alcohol | 0.006 | 0.006 | -0.011 | 0.008 | 0.020 | 0.018 | 0.029 | 0.015 | 0.001 | 0.026 | 0.008 | 0.015 |
| rs4842786 | 12 | 92170791 | A | G | -0.009 | 0.001 | 2.73E-09 | 0.005 | 0.002 | 3.70E-02 | Alcohol | 0.005 | 0.006 | 0.001 | 0.008 | 0.008 | 0.018 | 0.023 | 0.015 | -0.001 | 0.026 | 0.009 | 0.015 |
| rs2164744 | 12 | 109872039 | T | C | -0.003 | 0.002 | 1.26E-01 | -0.013 | 0.002 | 1.10E-08 | TV | 0.019 | 0.006 | 0.009 | 0.008 | 0.024 | 0.018 | 0.042 | 0.016 | 0.072 | 0.026 | 0.011 | 0.016 |
| rs2173650 | 12 | 117525235 | G | T | 0.001 | 0.003 | 6.30E-01 | 0.018 | 0.003 | 4.60E-09 | TV | 0.016 | 0.009 | 0.014 | 0.014 | 0.012 | 0.028 | 0.009 | 0.025 | 0.036 | 0.042 | 0.043 | 0.026 |
| rs500321 | 13 | 27124360 | T | A | -0.010 | 0.002 | 4.92E-09 | -0.001 | 0.002 | 5.90E-01 | Alcohol | -0.011 | 0.007 | -0.013 | 0.009 | -0.008 | 0.020 | -0.029 | 0.017 | 0.009 | 0.030 | 0.031 | 0.018 |
| rs9563168 | 13 | 54247827 | G | A | -0.005 | 0.002 | 2.96E-02 | 0.018 | 0.003 | 4.30E-11 | TV | 0.003 | 0.008 | 0.011 | 0.010 | 0.010 | 0.022 | 0.003 | 0.019 | -0.004 | 0.032 | -0.013 | 0.019 |
| rs9569734 | 13 | 58319476 | A | G | 0.003 | 0.003 | 1.97E-01 | 0.019 | 0.003 | 3.40E-10 | TV | 0.002 | 0.008 | 0.010 | 0.011 | -0.034 | 0.024 | 0.014 | 0.021 | 0.002 | 0.036 | 0.023 | 0.021 |
| rs56858768 | 13 | 86511730 | G | A | 0.000 | 0.002 | 8.78E-01 | -0.015 | 0.002 | 3.40E-10 | TV | 0.012 | 0.007 | 0.008 | 0.009 | 0.012 | 0.020 | 0.017 | 0.017 | -0.002 | 0.029 | 0.004 | 0.017 |
| rs7991062 | 13 | 100713194 | C | G | -0.008 | 0.002 | 1.45E-04 | -0.018 | 0.002 | 8.80E-15 | TV | -0.004 | 0.006 | -0.012 | 0.008 | -0.001 | 0.019 | -0.001 | 0.016 | -0.036 | 0.027 | -0.005 | 0.016 |
| rs405797 | 14 | 26972834 | T | A | 0.005 | 0.002 | 3.65E-02 | -0.015 | 0.003 | 2.80E-09 | TV | 0.015 | 0.008 | 0.013 | 0.010 | 0.017 | 0.022 | 0.036 | 0.019 | 0.027 | 0.032 | 0.004 | 0.019 |
| rs1022785 | 14 | 29571653 | G | A | 0.002 | 0.003 | 3.71E-01 | 0.018 | 0.003 | 7.10E-09 | TV | 0.008 | 0.009 | 0.009 | 0.011 | 0.007 | 0.025 | -0.014 | 0.022 | -0.026 | 0.037 | 0.032 | 0.022 |
| rs1123285 | 14 | 57274519 | G | C | -0.009 | 0.002 | 8.14E-09 | -0.006 | 0.002 | 1.30E-02 | Alcohol | 0.004 | 0.006 | 0.015 | 0.008 | 0.016 | 0.019 | -0.004 | 0.016 | 0.016 | 0.027 | 0.001 | 0.016 |
| rs10145592 | 14 | 94287215 | C | G | 0.008 | 0.002 | 5.41E-05 | -0.015 | 0.002 | 1.80E-11 | TV | 0.003 | 0.006 | -0.015 | 0.009 | -0.009 | 0.019 | 0.006 | 0.016 | 0.030 | 0.027 | 0.020 | 0.016 |
| rs7157001 | 14 | 99749484 | A | G | -0.003 | 0.002 | 1.16E-01 | -0.014 | 0.002 | 2.30E-08 | TV | -0.003 | 0.008 | -0.007 | 0.011 | -0.020 | 0.024 | 0.027 | 0.021 | 0.018 | 0.035 | 0.013 | 0.021 |
| rs8043253 | 15 | 41503730 | C | T | 0.006 | 0.002 | 1.66E-03 | -0.012 | 0.002 | 3.40E-08 | TV | -0.004 | 0.006 | -0.018 | 0.008 | 0.016 | 0.018 | 0.012 | 0.015 | -0.004 | 0.026 | -0.008 | 0.015 |
| rs2460 | 15 | 53073084 | G | A | 0.000 | 0.002 | 9.83E-01 | -0.015 | 0.002 | 5.00E-10 | TV | 0.000 | 0.007 | -0.006 | 0.010 | -0.001 | 0.021 | -0.007 | 0.018 | 0.024 | 0.031 | 0.024 | 0.019 |
| rs4775373 | 15 | 61476936 | T | C | -0.002 | 0.002 | 3.92E-01 | 0.013 | 0.002 | 4.70E-09 | TV | 0.002 | 0.006 | 0.000 | 0.008 | -0.001 | 0.019 | 0.004 | 0.016 | 0.034 | 0.027 | -0.026 | 0.016 |
| rs2472297 | 15 | 75027880 | T | C | 0.011 | 0.002 | 3.10E-10 | 0.003 | 0.002 | 2.30E-01 | Alcohol | 0.019 | 0.007 | 0.032 | 0.010 | -0.016 | 0.022 | 0.016 | 0.019 | 0.034 | 0.032 | 0.028 | 0.019 |
| rs61331678 | 15 | 77995949 | G | C | 0.000 | 0.002 | 8.35E-01 | 0.015 | 0.002 | 6.10E-12 | TV | 0.002 | 0.006 | 0.002 | 0.008 | 0.027 | 0.018 | -0.034 | 0.015 | -0.027 | 0.026 | 0.017 | 0.016 |
| rs12907323 | 15 | 86796012 | G | A | 0.008 | 0.001 | 9.93E-09 | -0.007 | 0.002 | 1.10E-03 | Alcohol | 0.005 | 0.006 | 0.018 | 0.008 | -0.008 | 0.018 | -0.026 | 0.015 | -0.019 | 0.026 | 0.023 | 0.015 |
| rs35574015 | 16 | 15146061 | T | C | -0.001 | 0.002 | 7.98E-01 | -0.013 | 0.002 | 3.00E-08 | TV | -0.007 | 0.007 | 0.000 | 0.009 | -0.023 | 0.020 | -0.008 | 0.017 | 0.026 | 0.030 | -0.015 | 0.018 |
| rs7189927 | 16 | 28913787 | T | C | -0.013 | 0.002 | 5.93E-11 | 0.015 | 0.002 | 3.40E-11 | TV | 0.001 | 0.006 | 0.006 | 0.008 | -0.010 | 0.019 | -0.001 | 0.016 | 0.014 | 0.027 | -0.026 | 0.016 |
| rs749671 | 16 | 31088347 | G | A | -0.004 | 0.002 | 2.94E-02 | 0.016 | 0.002 | 2.70E-12 | TV | -0.017 | 0.006 | -0.014 | 0.008 | -0.010 | 0.018 | -0.010 | 0.015 | -0.002 | 0.026 | -0.015 | 0.016 |
| rs7184800 | 16 | 53509131 | G | A | -0.002 | 0.002 | 2.51E-01 | 0.017 | 0.002 | 8.20E-13 | TV | -0.005 | 0.006 | -0.010 | 0.008 | -0.014 | 0.019 | 0.005 | 0.016 | 0.026 | 0.028 | -0.011 | 0.016 |
| rs7185555 | 16 | 69131281 | C | G | -0.011 | 0.002 | 4.24E-08 | -0.006 | 0.003 | 3.20E-02 | Alcohol | 0.012 | 0.009 | 0.011 | 0.011 | 0.011 | 0.025 | 0.010 | 0.021 | 0.008 | 0.036 | 0.011 | 0.022 |
| rs79616692 | 16 | 72338507 | C | G | 0.016 | 0.002 | 4.11E-12 | -0.008 | 0.004 | 1.70E-02 | Alcohol | 0.022 | 0.010 | 0.007 | 0.013 | 0.025 | 0.029 | -0.006 | 0.025 | 0.020 | 0.042 | 0.070 | 0.024 |
| rs2447098 | 17 | 2277720 | C | A | 0.000 | 0.002 | 9.08E-01 | -0.015 | 0.002 | 6.80E-12 | TV | 0.008 | 0.006 | 0.009 | 0.009 | 0.012 | 0.019 | 0.036 | 0.016 | -0.005 | 0.027 | 0.020 | 0.016 |
| rs11654952 | 17 | 4813799 | T | G | 0.003 | 0.003 | 2.49E-01 | -0.017 | 0.003 | 4.30E-08 | TV | 0.007 | 0.009 | 0.009 | 0.012 | 0.031 | 0.026 | 0.037 | 0.022 | 0.017 | 0.038 | -0.027 | 0.022 |
| rs2854334 | 17 | 29715500 | G | A | 0.009 | 0.001 | 7.51E-10 | -0.001 | 0.002 | 6.00E-01 | Alcohol | -0.014 | 0.006 | -0.017 | 0.008 | 0.000 | 0.018 | -0.015 | 0.015 | -0.014 | 0.026 | -0.011 | 0.016 |
| rs9902312 | 17 | 65070304 | T | C | 0.002 | 0.002 | 2.98E-01 | 0.015 | 0.002 | 4.50E-11 | TV | -0.009 | 0.006 | -0.024 | 0.008 | -0.026 | 0.019 | 0.016 | 0.016 | -0.021 | 0.027 | 0.009 | 0.016 |
| rs11657730 | 17 | 79372489 | C | T | 0.001 | 0.002 | 4.96E-01 | 0.013 | 0.002 | 1.10E-08 | TV | 0.021 | 0.007 | 0.021 | 0.009 | 0.007 | 0.019 | 0.024 | 0.017 | 0.062 | 0.029 | 0.023 | 0.017 |
| rs303753 | 18 | 21074922 | G | A | 0.007 | 0.002 | 3.24E-04 | -0.014 | 0.002 | 2.70E-10 | TV | -0.004 | 0.006 | -0.010 | 0.009 | -0.033 | 0.019 | 0.005 | 0.016 | 0.026 | 0.028 | -0.051 | 0.016 |
| rs9964724 | 18 | 35159124 | C | T | 0.002 | 0.002 | 3.50E-01 | 0.018 | 0.002 | 3.30E-14 | TV | 0.007 | 0.007 | 0.002 | 0.009 | 0.029 | 0.019 | 0.007 | 0.017 | -0.043 | 0.029 | 0.009 | 0.017 |
| rs1156541 | 18 | 39952989 | C | T | 0.000 | 0.002 | 9.54E-01 | 0.014 | 0.003 | 2.80E-08 | TV | -0.018 | 0.007 | -0.012 | 0.009 | -0.043 | 0.021 | -0.029 | 0.018 | -0.010 | 0.030 | -0.044 | 0.018 |
| rs17512836 | 18 | 53194961 | T | C | -0.005 | 0.006 | 4.40E-01 | 0.042 | 0.007 | 7.00E-10 | TV | -0.008 | 0.019 | -0.034 | 0.025 | 0.048 | 0.056 | -0.022 | 0.047 | -0.007 | 0.079 | 0.035 | 0.049 |
| rs7248205 | 19 | 10770305 | C | T | 0.002 | 0.002 | 3.57E-01 | 0.014 | 0.002 | 3.40E-10 | TV | 0.012 | 0.006 | 0.012 | 0.008 | 0.030 | 0.019 | 0.021 | 0.016 | -0.012 | 0.027 | -0.022 | 0.016 |
| rs111901094 | 19 | 19513570 | G | T | -0.003 | 0.003 | 2.16E-01 | -0.017 | 0.003 | 2.00E-09 | TV | -0.032 | 0.009 | -0.038 | 0.012 | -0.011 | 0.027 | -0.024 | 0.023 | -0.092 | 0.039 | -0.064 | 0.023 |
| rs281379 | 19 | 49214274 | A | G | 0.014 | 0.001 | 4.91E-21 | 0.001 | 0.002 | 5.60E-01 | Alcohol | 0.013 | 0.006 | 0.007 | 0.008 | 0.068 | 0.018 | 0.001 | 0.015 | 0.004 | 0.026 | -0.018 | 0.015 |
| rs6131281 | 20 | 11891724 | C | T | 0.006 | 0.002 | 1.36E-03 | 0.016 | 0.002 | 3.10E-13 | TV | 0.002 | 0.007 | -0.006 | 0.009 | -0.007 | 0.019 | 0.008 | 0.016 | 0.016 | 0.027 | 0.010 | 0.016 |
| rs6141814 | 20 | 31368960 | C | A | -0.005 | 0.002 | 1.55E-02 | -0.014 | 0.002 | 1.30E-09 | TV | 0.004 | 0.006 | 0.005 | 0.008 | 0.029 | 0.018 | 0.000 | 0.015 | 0.006 | 0.026 | -0.006 | 0.016 |
| rs55700114 | 20 | 43717080 | G | A | -0.001 | 0.002 | 5.01E-01 | -0.014 | 0.002 | 2.10E-09 | TV | -0.002 | 0.006 | -0.007 | 0.009 | 0.035 | 0.019 | -0.019 | 0.016 | 0.018 | 0.027 | -0.008 | 0.016 |
| rs56103247 | 20 | 62483184 | C | T | 0.005 | 0.004 | 2.74E-01 | 0.030 | 0.005 | 3.80E-10 | TV | -0.006 | 0.015 | -0.007 | 0.020 | 0.020 | 0.045 | -0.013 | 0.037 | -0.068 | 0.061 | 0.014 | 0.038 |
| rs10427502 | 21 | 40654840 | G | A | -0.002 | 0.002 | 2.27E-01 | 0.014 | 0.002 | 8.60E-10 | TV | -0.012 | 0.006 | -0.013 | 0.008 | -0.003 | 0.018 | -0.021 | 0.016 | -0.067 | 0.027 | -0.014 | 0.016 |
| rs2092829 | 22 | 31780514 | G | A | 0.004 | 0.002 | 3.89E-02 | 0.014 | 0.002 | 1.30E-08 | TV | -0.002 | 0.007 | -0.010 | 0.009 | -0.005 | 0.019 | -0.014 | 0.017 | -0.001 | 0.028 | 0.010 | 0.017 |
| rs9607814 | 22 | 41946519 | A | C | -0.010 | 0.002 | 4.31E-08 | 0.006 | 0.003 | 2.90E-02 | Alcohol | 0.000 | 0.008 | 0.016 | 0.010 | 0.038 | 0.022 | -0.041 | 0.019 | -0.015 | 0.032 | -0.026 | 0.019 |

| **Table S13. Beta estimates for the SNPs included in the multivariable MR of alcohol consumption, television use, and colorectal cancer risk** | | | | | | | | | | | | | | | | | | | | | |
| --- | --- | --- | --- | --- | --- | --- | --- | --- | --- | --- | --- | --- | --- | --- | --- | --- | --- | --- | --- | --- | --- |
| SNP | Chr | Position | EA | OA | beta_alc | se_alc | pval_alc | beta_tv | se_tv | pval_tv | phenotype | beta_crc | se_crc | beta_crc_men | se_crc_men | beta_crc_women | se_crc_women | beta_co | se_co | beta_re | se_re |
| rs705687 | 1 | 4548453 | G | A | -0.011 | 0.002 | 8.15E-10 | 0.002 | 0.003 | 5.30E-01 | Alcohol | 0.003 | 0.012 | 0.002 | 0.017 | -0.001 | 0.017 | -0.003 | 0.014 | -0.002 | 0.019 |
| rs58107686 | 1 | 33837334 | A | C | -0.010 | 0.002 | 7.79E-10 | 0.001 | 0.002 | 6.40E-01 | Alcohol | 0.007 | 0.010 | 0.018 | 0.014 | -0.002 | 0.014 | 0.007 | 0.012 | 0.009 | 0.016 |
| rs11810109 | 1 | 44186812 | A | T | 0.003 | 0.002 | 1.44E-01 | 0.016 | 0.002 | 5.40E-12 | TV | 0.011 | 0.011 | 0.000 | 0.015 | 0.022 | 0.015 | 0.004 | 0.013 | 0.016 | 0.017 |
| rs12725114 | 1 | 62432641 | G | A | 0.001 | 0.002 | 7.98E-01 | 0.015 | 0.003 | 1.60E-08 | TV | -0.016 | 0.012 | -0.023 | 0.017 | -0.007 | 0.017 | 0.003 | 0.014 | -0.060 | 0.018 |
| rs984409 | 1 | 67020440 | G | A | -0.002 | 0.002 | 2.14E-01 | -0.015 | 0.002 | 4.10E-11 | TV | -0.019 | 0.010 | -0.010 | 0.015 | -0.028 | 0.015 | -0.011 | 0.012 | -0.030 | 0.016 |
| rs5024204 | 1 | 71491890 | T | A | 0.010 | 0.002 | 2.55E-09 | -0.008 | 0.002 | 1.70E-03 | Alcohol | -0.005 | 0.011 | 0.005 | 0.015 | -0.015 | 0.015 | 0.006 | 0.013 | -0.008 | 0.017 |
| rs71658797 | 1 | 77967507 | T | A | 0.003 | 0.003 | 3.40E-01 | -0.020 | 0.003 | 6.30E-10 | TV | -0.005 | 0.016 | -0.002 | 0.023 | -0.009 | 0.023 | -0.009 | 0.019 | -0.009 | 0.025 |
| rs114328297 | 1 | 91190854 | T | G | 0.008 | 0.002 | 3.24E-04 | 0.014 | 0.003 | 4.40E-08 | TV | 0.002 | 0.012 | 0.018 | 0.017 | -0.018 | 0.018 | 0.016 | 0.015 | -0.056 | 0.019 |
| rs17379561 | 1 | 98340139 | A | T | -0.003 | 0.003 | 3.25E-01 | -0.025 | 0.003 | 1.10E-16 | TV | 0.016 | 0.014 | 0.004 | 0.020 | 0.024 | 0.020 | 0.016 | 0.017 | 0.016 | 0.022 |
| rs55909997 | 1 | 107581039 | G | A | -0.002 | 0.002 | 3.61E-01 | -0.014 | 0.002 | 2.40E-09 | TV | -0.027 | 0.010 | -0.037 | 0.014 | -0.017 | 0.014 | -0.011 | 0.012 | -0.033 | 0.016 |
| rs1278847 | 1 | 110037483 | C | A | 0.004 | 0.002 | 5.09E-02 | 0.016 | 0.002 | 1.20E-11 | TV | -0.006 | 0.011 | 0.008 | 0.015 | -0.018 | 0.015 | -0.006 | 0.013 | -0.004 | 0.017 |
| rs4845364 | 1 | 154141908 | A | G | -0.001 | 0.002 | 7.34E-01 | -0.015 | 0.002 | 1.30E-12 | TV | -0.006 | 0.010 | -0.014 | 0.014 | 0.001 | 0.014 | -0.008 | 0.012 | -0.013 | 0.015 |
| rs10753661 | 1 | 165119792 | A | G | -0.009 | 0.002 | 3.76E-08 | 0.002 | 0.002 | 3.00E-01 | Alcohol | 0.006 | 0.011 | -0.008 | 0.015 | 0.020 | 0.015 | 0.003 | 0.012 | 0.022 | 0.016 |
| rs28680958 | 1 | 173848808 | A | G | -0.011 | 0.002 | 5.13E-10 | 0.000 | 0.003 | 9.50E-01 | Alcohol | -0.013 | 0.011 | -0.013 | 0.016 | -0.018 | 0.016 | -0.024 | 0.014 | -0.006 | 0.018 |
| rs6673341 | 1 | 184655649 | T | G | -0.001 | 0.002 | 5.32E-01 | -0.015 | 0.002 | 2.20E-11 | TV | 0.000 | 0.010 | -0.012 | 0.014 | 0.012 | 0.014 | 0.002 | 0.012 | -0.014 | 0.015 |
| rs10737620 | 1 | 193057971 | T | A | 0.001 | 0.002 | 6.71E-01 | 0.014 | 0.002 | 2.60E-09 | TV | -0.010 | 0.011 | -0.008 | 0.015 | -0.011 | 0.015 | 0.000 | 0.013 | -0.024 | 0.017 |
| rs823114 | 1 | 205719532 | A | G | 0.009 | 0.001 | 2.31E-09 | -0.001 | 0.002 | 5.00E-01 | Alcohol | -0.031 | 0.010 | -0.033 | 0.014 | -0.030 | 0.014 | -0.033 | 0.012 | -0.032 | 0.015 |
| rs77165542 | 2 | 430975 | T | C | -0.026 | 0.004 | 5.63E-11 | 0.003 | 0.006 | 5.90E-01 | Alcohol | -0.058 | 0.030 | -0.033 | 0.043 | -0.085 | 0.043 | -0.060 | 0.036 | -0.026 | 0.047 |
| rs72781699 | 2 | 24277709 | G | A | 0.007 | 0.002 | 3.84E-03 | -0.019 | 0.003 | 3.00E-12 | TV | 0.009 | 0.012 | 0.008 | 0.017 | 0.008 | 0.017 | -0.012 | 0.014 | 0.042 | 0.019 |
| rs2178197 | 2 | 27860551 | G | A | -0.009 | 0.001 | 2.45E-09 | -0.007 | 0.002 | 6.30E-04 | Alcohol | -0.002 | 0.010 | -0.006 | 0.014 | -0.001 | 0.014 | -0.007 | 0.012 | 0.004 | 0.015 |
| rs12105701 | 2 | 41737200 | C | T | 0.000 | 0.002 | 9.36E-01 | -0.013 | 0.002 | 5.40E-09 | TV | -0.009 | 0.010 | 0.013 | 0.014 | -0.030 | 0.014 | -0.007 | 0.012 | -0.017 | 0.015 |
| rs1004787 | 2 | 45159091 | A | G | 0.008 | 0.001 | 8.40E-09 | 0.002 | 0.002 | 4.40E-01 | Alcohol | -0.020 | 0.010 | -0.019 | 0.014 | -0.018 | 0.014 | -0.020 | 0.012 | -0.023 | 0.015 |
| rs7564130 | 2 | 50606642 | T | C | -0.005 | 0.002 | 1.89E-02 | -0.015 | 0.002 | 2.80E-11 | TV | -0.021 | 0.010 | -0.019 | 0.014 | -0.022 | 0.014 | -0.032 | 0.012 | -0.002 | 0.016 |
| rs10189857 | 2 | 60713235 | A | G | 0.006 | 0.002 | 1.57E-03 | -0.020 | 0.002 | 6.20E-21 | TV | -0.022 | 0.010 | -0.022 | 0.014 | -0.021 | 0.014 | -0.020 | 0.012 | -0.019 | 0.015 |
| rs62641636 | 2 | 68425427 | A | G | 0.002 | 0.002 | 2.65E-01 | 0.014 | 0.002 | 7.00E-10 | TV | 0.010 | 0.010 | 0.000 | 0.015 | 0.018 | 0.015 | 0.022 | 0.012 | 0.015 | 0.016 |
| rs11689199 | 2 | 100820421 | A | G | 0.004 | 0.002 | 6.86E-02 | 0.018 | 0.002 | 5.50E-17 | TV | 0.025 | 0.010 | 0.039 | 0.014 | 0.012 | 0.014 | 0.020 | 0.012 | 0.034 | 0.016 |
| rs1451533 | 2 | 105466005 | G | A | 0.000 | 0.002 | 9.17E-01 | -0.016 | 0.002 | 1.10E-10 | TV | 0.009 | 0.011 | 0.009 | 0.015 | 0.011 | 0.015 | 0.002 | 0.013 | 0.013 | 0.017 |
| rs12476388 | 2 | 116330983 | C | T | 0.000 | 0.002 | 9.96E-01 | 0.013 | 0.002 | 2.10E-08 | TV | -0.002 | 0.010 | -0.005 | 0.015 | 0.000 | 0.015 | -0.012 | 0.013 | 0.001 | 0.017 |
| rs374722 | 2 | 147839830 | G | A | -0.005 | 0.003 | 8.71E-02 | 0.024 | 0.003 | 5.50E-16 | TV | 0.026 | 0.013 | 0.002 | 0.019 | 0.049 | 0.019 | 0.038 | 0.016 | 0.017 | 0.021 |
| rs3754970 | 2 | 162091836 | T | C | -0.006 | 0.002 | 1.14E-03 | -0.015 | 0.002 | 4.80E-12 | TV | 0.006 | 0.010 | 0.003 | 0.014 | 0.007 | 0.014 | 0.011 | 0.012 | -0.011 | 0.015 |
| rs2060199 | 2 | 166196681 | T | A | -0.003 | 0.002 | 1.82E-01 | 0.012 | 0.002 | 2.40E-08 | TV | 0.016 | 0.010 | 0.039 | 0.014 | -0.002 | 0.014 | 0.012 | 0.012 | 0.028 | 0.015 |
| rs4577309 | 2 | 191288833 | A | G | -0.001 | 0.002 | 7.79E-01 | 0.016 | 0.002 | 1.60E-13 | TV | 0.001 | 0.010 | 0.015 | 0.014 | -0.012 | 0.014 | 0.004 | 0.012 | 0.009 | 0.015 |
| rs34224261 | 2 | 193855452 | G | C | 0.004 | 0.002 | 3.27E-02 | -0.018 | 0.002 | 4.60E-16 | TV | 0.001 | 0.010 | -0.002 | 0.014 | 0.005 | 0.014 | -0.002 | 0.012 | 0.000 | 0.015 |
| rs4675246 | 2 | 202864487 | G | T | -0.005 | 0.002 | 3.61E-02 | -0.015 | 0.003 | 2.90E-08 | TV | 0.037 | 0.013 | 0.043 | 0.018 | 0.031 | 0.018 | 0.028 | 0.015 | 0.047 | 0.020 |
| rs13029509 | 2 | 215374209 | G | A | 0.001 | 0.002 | 5.73E-01 | -0.018 | 0.002 | 2.00E-17 | TV | -0.005 | 0.010 | -0.007 | 0.014 | -0.004 | 0.014 | -0.001 | 0.012 | -0.011 | 0.015 |
| rs10932837 | 2 | 221044785 | C | T | 0.000 | 0.002 | 9.44E-01 | -0.013 | 0.002 | 1.30E-09 | TV | -0.003 | 0.010 | 0.013 | 0.014 | -0.020 | 0.014 | -0.005 | 0.012 | -0.016 | 0.015 |
| rs4973576 | 2 | 233806771 | C | A | 0.005 | 0.002 | 3.73E-02 | -0.015 | 0.002 | 1.00E-09 | TV | 0.013 | 0.011 | -0.005 | 0.015 | 0.034 | 0.015 | 0.013 | 0.013 | -0.001 | 0.017 |
| rs138256022 | 3 | 11603090 | C | T | 0.004 | 0.005 | 4.32E-01 | -0.031 | 0.006 | 2.90E-08 | TV | 0.043 | 0.031 | 0.025 | 0.045 | 0.065 | 0.044 | 0.031 | 0.037 | 0.046 | 0.049 |
| rs9834970 | 3 | 36856030 | T | C | -0.001 | 0.002 | 5.90E-01 | 0.013 | 0.002 | 3.30E-09 | TV | 0.013 | 0.010 | 0.002 | 0.014 | 0.024 | 0.014 | 0.010 | 0.012 | 0.017 | 0.015 |
| rs3796386 | 3 | 49899795 | G | A | 0.001 | 0.002 | 5.67E-01 | -0.026 | 0.002 | 3.20E-33 | TV | -0.015 | 0.010 | -0.014 | 0.014 | -0.018 | 0.014 | -0.012 | 0.012 | -0.040 | 0.015 |
| rs11130793 | 3 | 60884659 | C | T | -0.002 | 0.002 | 2.82E-01 | 0.013 | 0.002 | 5.00E-09 | TV | 0.008 | 0.010 | 0.018 | 0.014 | -0.003 | 0.014 | -0.004 | 0.012 | 0.011 | 0.016 |
| rs11714337 | 3 | 71582521 | G | A | -0.006 | 0.002 | 3.41E-03 | 0.014 | 0.002 | 4.70E-11 | TV | -0.021 | 0.010 | -0.010 | 0.014 | -0.029 | 0.014 | -0.029 | 0.012 | -0.011 | 0.015 |
| rs6797840 | 3 | 85656569 | A | C | -0.007 | 0.002 | 3.54E-04 | -0.016 | 0.002 | 1.70E-13 | TV | 0.009 | 0.010 | -0.004 | 0.014 | 0.018 | 0.014 | 0.016 | 0.012 | 0.015 | 0.015 |
| rs2034768 | 3 | 93986371 | A | G | 0.005 | 0.002 | 6.70E-03 | 0.015 | 0.002 | 8.60E-12 | TV | -0.013 | 0.010 | -0.017 | 0.014 | -0.007 | 0.014 | -0.014 | 0.012 | -0.007 | 0.015 |
| rs9867121 | 3 | 114631548 | C | A | 0.004 | 0.002 | 9.28E-02 | 0.019 | 0.003 | 3.90E-12 | TV | 0.020 | 0.012 | 0.041 | 0.017 | -0.004 | 0.017 | 0.018 | 0.015 | 0.014 | 0.019 |
| rs9838144 | 3 | 131576287 | C | G | -0.010 | 0.002 | 2.65E-08 | 0.005 | 0.003 | 8.80E-02 | Alcohol | -0.002 | 0.012 | -0.023 | 0.017 | 0.022 | 0.017 | -0.009 | 0.014 | 0.013 | 0.019 |
| rs60654199 | 3 | 141267295 | A | C | -0.017 | 0.003 | 2.85E-08 | 0.013 | 0.004 | 1.90E-03 | Alcohol | -0.006 | 0.019 | -0.035 | 0.026 | 0.026 | 0.026 | 0.001 | 0.024 | -0.001 | 0.031 |
| rs6787172 | 3 | 158187811 | G | T | -0.008 | 0.001 | 4.27E-08 | 0.001 | 0.002 | 7.40E-01 | Alcohol | -0.013 | 0.010 | -0.018 | 0.014 | -0.011 | 0.014 | -0.014 | 0.012 | -0.003 | 0.015 |
| rs12491503 | 3 | 165706855 | G | A | 0.001 | 0.002 | 6.46E-01 | -0.014 | 0.002 | 5.50E-10 | TV | -0.001 | 0.010 | -0.011 | 0.014 | 0.008 | 0.014 | -0.005 | 0.012 | -0.006 | 0.016 |
| rs114600294 | 3 | 181419367 | G | C | 0.006 | 0.002 | 2.37E-02 | -0.016 | 0.003 | 7.90E-10 | TV | 0.013 | 0.014 | 0.022 | 0.020 | 0.005 | 0.020 | 0.021 | 0.017 | 0.007 | 0.022 |
| rs66852340 | 4 | 3311070 | C | T | 0.004 | 0.002 | 8.69E-02 | -0.018 | 0.003 | 7.90E-12 | TV | -0.004 | 0.011 | -0.013 | 0.016 | 0.002 | 0.016 | 0.000 | 0.014 | -0.030 | 0.018 |
| rs7700107 | 4 | 17880416 | A | C | -0.002 | 0.003 | 4.30E-01 | -0.021 | 0.003 | 1.90E-11 | TV | -0.003 | 0.014 | -0.025 | 0.019 | 0.017 | 0.019 | 0.018 | 0.017 | -0.011 | 0.022 |
| rs11940694 | 4 | 39414993 | G | A | 0.026 | 0.001 | 3.03E-68 | 0.004 | 0.002 | 9.50E-02 | Alcohol | -0.005 | 0.010 | -0.015 | 0.014 | 0.007 | 0.014 | -0.011 | 0.012 | 0.006 | 0.015 |
| rs28971796 | 4 | 49151982 | A | G | -0.004 | 0.002 | 5.96E-02 | 0.015 | 0.002 | 2.10E-09 | TV | 0.010 | 0.012 | 0.004 | 0.017 | 0.018 | 0.017 | 0.003 | 0.015 | 0.037 | 0.019 |
| rs6850494 | 4 | 82291771 | A | C | -0.001 | 0.002 | 6.62E-01 | -0.014 | 0.002 | 1.10E-10 | TV | -0.011 | 0.010 | -0.019 | 0.014 | -0.002 | 0.014 | -0.029 | 0.012 | -0.008 | 0.016 |
| rs1229984 | 4 | 100239319 | C | T | 0.151 | 0.004 | 0.00E+00 | 0.023 | 0.007 | 2.00E-03 | Alcohol | 0.039 | 0.021 | 0.040 | 0.029 | 0.036 | 0.030 | 0.039 | 0.026 | 0.063 | 0.035 |
| rs2165670 | 4 | 100286085 | A | G | 0.023 | 0.002 | 1.67E-22 | 0.004 | 0.004 | 3.30E-01 | Alcohol | 0.009 | 0.016 | -0.005 | 0.023 | 0.027 | 0.023 | 0.010 | 0.020 | -0.001 | 0.026 |
| rs13107325 | 4 | 103188709 | T | C | -0.028 | 0.003 | 1.53E-22 | 0.029 | 0.004 | 1.50E-12 | both | -0.008 | 0.019 | 0.009 | 0.026 | -0.028 | 0.027 | -0.012 | 0.022 | 0.006 | 0.029 |
| rs7693703 | 4 | 118357405 | G | A | -0.008 | 0.003 | 1.32E-02 | 0.023 | 0.004 | 2.70E-09 | TV | 0.022 | 0.017 | -0.004 | 0.024 | 0.050 | 0.024 | 0.018 | 0.020 | 0.070 | 0.027 |
| rs4334769 | 4 | 130245233 | G | T | -0.003 | 0.002 | 1.23E-01 | 0.012 | 0.002 | 2.10E-08 | TV | -0.015 | 0.010 | -0.010 | 0.014 | -0.016 | 0.014 | -0.023 | 0.012 | -0.015 | 0.015 |
| rs4690727 | 4 | 143648579 | G | C | 0.011 | 0.002 | 2.43E-11 | 0.001 | 0.002 | 7.90E-01 | Alcohol | 0.030 | 0.011 | 0.037 | 0.015 | 0.026 | 0.015 | 0.033 | 0.013 | 0.013 | 0.017 |
| rs6825241 | 4 | 152543783 | C | A | 0.002 | 0.002 | 2.41E-01 | -0.017 | 0.002 | 4.90E-15 | TV | -0.008 | 0.010 | -0.003 | 0.014 | -0.012 | 0.014 | -0.005 | 0.012 | -0.016 | 0.015 |
| rs7693082 | 4 | 159857819 | G | C | 0.003 | 0.002 | 1.08E-01 | 0.015 | 0.002 | 1.70E-10 | TV | 0.017 | 0.011 | 0.012 | 0.015 | 0.020 | 0.015 | 0.026 | 0.013 | 0.020 | 0.017 |
| rs12651313 | 4 | 171086393 | G | C | -0.009 | 0.001 | 3.79E-09 | 0.006 | 0.002 | 3.10E-03 | Alcohol | -0.011 | 0.010 | 0.008 | 0.014 | -0.029 | 0.014 | -0.017 | 0.012 | -0.007 | 0.015 |
| rs10940659 | 5 | 59360341 | A | G | 0.005 | 0.002 | 1.36E-02 | -0.013 | 0.002 | 1.50E-08 | TV | -0.011 | 0.010 | -0.027 | 0.014 | 0.003 | 0.014 | -0.011 | 0.012 | -0.020 | 0.015 |
| rs262890 | 5 | 62930015 | A | G | -0.003 | 0.002 | 1.60E-01 | -0.019 | 0.002 | 3.20E-15 | TV | 0.031 | 0.011 | 0.036 | 0.015 | 0.028 | 0.015 | 0.028 | 0.013 | 0.043 | 0.017 |
| rs7716447 | 5 | 88800331 | A | G | 0.001 | 0.002 | 7.36E-01 | -0.013 | 0.002 | 3.60E-09 | TV | -0.016 | 0.010 | -0.018 | 0.014 | -0.009 | 0.014 | -0.007 | 0.012 | -0.021 | 0.016 |
| rs1031423 | 5 | 93276883 | T | C | 0.003 | 0.002 | 2.70E-01 | -0.019 | 0.003 | 1.80E-12 | TV | 0.020 | 0.012 | 0.023 | 0.017 | 0.017 | 0.017 | 0.022 | 0.014 | 0.017 | 0.019 |
| rs57585211 | 5 | 107197725 | T | G | 0.004 | 0.003 | 1.71E-01 | -0.017 | 0.003 | 4.30E-09 | TV | -0.003 | 0.013 | -0.020 | 0.018 | 0.012 | 0.018 | -0.008 | 0.016 | -0.009 | 0.021 |
| rs9326862 | 5 | 112054547 | A | T | 0.002 | 0.002 | 2.05E-01 | 0.012 | 0.002 | 2.60E-08 | TV | 0.001 | 0.010 | -0.011 | 0.014 | 0.014 | 0.014 | 0.006 | 0.012 | -0.010 | 0.015 |
| rs10041724 | 5 | 124273520 | T | C | -0.001 | 0.002 | 8.33E-01 | 0.018 | 0.003 | 3.90E-11 | TV | 0.015 | 0.012 | 0.021 | 0.017 | 0.010 | 0.017 | 0.016 | 0.014 | 0.012 | 0.019 |
| rs62379379 | 5 | 141082015 | G | T | 0.001 | 0.004 | 8.54E-01 | -0.026 | 0.004 | 7.80E-10 | TV | -0.032 | 0.019 | -0.025 | 0.027 | -0.038 | 0.026 | -0.029 | 0.023 | -0.022 | 0.031 |
| rs10054327 | 5 | 147871137 | G | A | -0.002 | 0.002 | 3.26E-01 | 0.017 | 0.002 | 3.40E-15 | TV | -0.002 | 0.010 | 0.003 | 0.014 | -0.005 | 0.014 | -0.013 | 0.012 | -0.002 | 0.016 |
| rs42210 | 5 | 166408788 | G | C | -0.002 | 0.002 | 3.85E-01 | -0.014 | 0.002 | 7.30E-09 | TV | -0.007 | 0.011 | -0.016 | 0.015 | 0.004 | 0.015 | 0.004 | 0.013 | -0.024 | 0.017 |
| rs72828890 | 5 | 167806836 | C | T | 0.004 | 0.003 | 1.27E-01 | 0.019 | 0.003 | 4.90E-09 | TV | 0.012 | 0.016 | 0.026 | 0.023 | -0.002 | 0.023 | 0.017 | 0.019 | 0.003 | 0.025 |
| rs72834698 | 6 | 26176517 | G | A | 0.001 | 0.003 | 7.42E-01 | 0.023 | 0.003 | 2.70E-13 | TV | 0.044 | 0.014 | 0.060 | 0.020 | 0.031 | 0.020 | 0.059 | 0.017 | 0.069 | 0.022 |
| rs9471333 | 6 | 40362023 | C | T | 0.002 | 0.002 | 2.09E-01 | 0.013 | 0.002 | 1.50E-09 | TV | 0.010 | 0.010 | 0.020 | 0.014 | 0.001 | 0.014 | 0.007 | 0.012 | 0.018 | 0.015 |
| rs4523073 | 6 | 67550288 | A | G | -0.005 | 0.002 | 1.59E-02 | -0.014 | 0.002 | 5.00E-10 | TV | 0.006 | 0.010 | 0.002 | 0.014 | 0.009 | 0.014 | 0.002 | 0.012 | 0.022 | 0.016 |
| rs6905544 | 6 | 98411631 | A | G | 0.008 | 0.002 | 8.95E-05 | -0.019 | 0.002 | 8.50E-18 | TV | -0.029 | 0.010 | -0.037 | 0.014 | -0.023 | 0.014 | -0.024 | 0.012 | -0.035 | 0.015 |
| rs17789218 | 6 | 100600097 | T | C | 0.003 | 0.002 | 2.33E-01 | 0.019 | 0.003 | 1.40E-13 | TV | 0.004 | 0.012 | 0.016 | 0.016 | -0.006 | 0.016 | -0.006 | 0.014 | 0.011 | 0.018 |
| rs2184364 | 6 | 143183596 | A | G | 0.006 | 0.002 | 1.69E-02 | 0.016 | 0.003 | 3.00E-09 | TV | 0.023 | 0.012 | 0.025 | 0.017 | 0.022 | 0.017 | 0.024 | 0.015 | 0.017 | 0.019 |
| rs9718104 | 6 | 166170539 | T | G | 0.013 | 0.004 | 1.68E-03 | -0.041 | 0.005 | 9.30E-19 | TV | -0.002 | 0.022 | 0.002 | 0.032 | -0.013 | 0.031 | 0.009 | 0.027 | 0.005 | 0.036 |
| rs17568389 | 7 | 8091876 | T | A | 0.004 | 0.002 | 4.20E-02 | 0.015 | 0.002 | 8.60E-13 | TV | 0.001 | 0.010 | 0.012 | 0.014 | -0.012 | 0.014 | 0.000 | 0.012 | 0.012 | 0.015 |
| rs10234444 | 7 | 41828527 | G | A | 0.002 | 0.003 | 5.06E-01 | 0.016 | 0.003 | 2.90E-08 | TV | -0.017 | 0.013 | -0.018 | 0.018 | -0.018 | 0.018 | -0.023 | 0.016 | -0.004 | 0.021 |
| rs6460047 | 7 | 73042443 | C | T | 0.012 | 0.002 | 9.69E-11 | 0.000 | 0.003 | 9.50E-01 | Alcohol | -0.002 | 0.012 | 0.004 | 0.017 | -0.008 | 0.017 | -0.004 | 0.014 | 0.019 | 0.019 |
| rs6973656 | 7 | 77422583 | A | G | -0.001 | 0.002 | 6.44E-01 | -0.013 | 0.002 | 1.00E-09 | TV | 0.029 | 0.010 | 0.025 | 0.014 | 0.031 | 0.014 | 0.028 | 0.012 | 0.036 | 0.015 |
| rs973734 | 7 | 89387578 | C | A | 0.004 | 0.003 | 1.88E-01 | 0.017 | 0.003 | 1.50E-08 | TV | 0.002 | 0.014 | -0.022 | 0.020 | 0.031 | 0.020 | 0.013 | 0.016 | -0.009 | 0.021 |
| rs10236149 | 7 | 98977515 | G | A | -0.013 | 0.002 | 1.18E-09 | -0.014 | 0.003 | 1.40E-05 | Alcohol | -0.007 | 0.014 | 0.005 | 0.020 | -0.025 | 0.020 | -0.012 | 0.017 | 0.015 | 0.022 |
| rs62471080 | 7 | 109128060 | G | C | -0.001 | 0.002 | 7.32E-01 | -0.013 | 0.002 | 3.70E-09 | TV | -0.005 | 0.010 | -0.007 | 0.014 | -0.004 | 0.014 | 0.010 | 0.012 | -0.012 | 0.015 |
| rs10246289 | 7 | 115544763 | A | G | 0.001 | 0.003 | 7.01E-01 | 0.019 | 0.003 | 1.70E-08 | TV | 0.041 | 0.015 | 0.039 | 0.022 | 0.042 | 0.022 | 0.033 | 0.019 | 0.033 | 0.024 |
| rs11763734 | 7 | 126400910 | A | C | -0.005 | 0.002 | 5.86E-03 | -0.012 | 0.002 | 8.20E-09 | TV | -0.001 | 0.010 | 0.001 | 0.014 | -0.005 | 0.014 | -0.002 | 0.012 | 0.005 | 0.015 |
| rs6951574 | 7 | 153489744 | C | T | 0.013 | 0.001 | 1.58E-19 | 0.004 | 0.002 | 7.20E-02 | Alcohol | -0.006 | 0.010 | -0.001 | 0.014 | -0.015 | 0.014 | -0.010 | 0.012 | 0.004 | 0.016 |
| rs7834121 | 8 | 10770255 | G | T | -0.005 | 0.002 | 1.89E-02 | -0.014 | 0.002 | 1.10E-10 | TV | 0.007 | 0.010 | 0.015 | 0.015 | 0.000 | 0.015 | -0.007 | 0.012 | 0.028 | 0.016 |
| rs13250583 | 8 | 20949917 | T | C | -0.010 | 0.002 | 4.70E-08 | 0.002 | 0.003 | 4.00E-01 | Alcohol | -0.015 | 0.012 | -0.038 | 0.017 | 0.010 | 0.017 | -0.017 | 0.014 | 0.000 | 0.019 |
| rs1421334 | 8 | 30865733 | A | C | -0.005 | 0.002 | 9.72E-03 | 0.017 | 0.002 | 2.20E-15 | TV | 0.017 | 0.010 | 0.017 | 0.014 | 0.018 | 0.014 | 0.022 | 0.012 | -0.005 | 0.015 |
| rs6996198 | 8 | 65463442 | C | T | 0.007 | 0.003 | 1.30E-02 | -0.016 | 0.003 | 3.50E-08 | TV | -0.016 | 0.013 | -0.009 | 0.018 | -0.025 | 0.018 | -0.011 | 0.016 | -0.017 | 0.020 |
| rs6472942 | 8 | 76827190 | T | C | 0.000 | 0.002 | 8.98E-01 | -0.013 | 0.002 | 1.80E-09 | TV | 0.000 | 0.010 | -0.006 | 0.014 | 0.004 | 0.014 | -0.001 | 0.012 | 0.015 | 0.015 |
| rs11985394 | 8 | 92667521 | T | A | 0.005 | 0.002 | 1.63E-02 | 0.012 | 0.002 | 1.30E-08 | TV | 0.010 | 0.010 | 0.017 | 0.014 | -0.001 | 0.014 | 0.020 | 0.012 | 0.000 | 0.015 |
| rs12541615 | 8 | 118870151 | T | C | -0.002 | 0.002 | 3.69E-01 | -0.018 | 0.003 | 3.90E-10 | TV | -0.003 | 0.012 | 0.001 | 0.017 | -0.008 | 0.018 | -0.013 | 0.015 | 0.006 | 0.019 |
| rs2717559 | 8 | 143882420 | A | G | -0.001 | 0.002 | 5.86E-01 | 0.012 | 0.002 | 1.50E-08 | TV | 0.000 | 0.010 | -0.018 | 0.014 | 0.018 | 0.014 | -0.006 | 0.012 | 0.003 | 0.015 |
| rs2616830 | 9 | 1721385 | G | A | -0.004 | 0.002 | 3.60E-02 | 0.016 | 0.002 | 2.90E-14 | TV | 0.013 | 0.010 | 0.014 | 0.014 | 0.008 | 0.014 | 0.016 | 0.012 | 0.031 | 0.015 |
| rs34864022 | 9 | 22609110 | A | G | 0.011 | 0.004 | 4.36E-03 | -0.026 | 0.004 | 1.40E-09 | TV | 0.013 | 0.021 | 0.033 | 0.030 | -0.010 | 0.030 | 0.015 | 0.025 | -0.026 | 0.033 |
| rs12554512 | 9 | 23352293 | T | C | 0.000 | 0.002 | 9.84E-01 | 0.021 | 0.002 | 3.80E-21 | TV | -0.002 | 0.010 | -0.001 | 0.014 | -0.002 | 0.014 | 0.011 | 0.012 | -0.005 | 0.016 |
| rs2791574 | 9 | 82212094 | T | G | 0.004 | 0.002 | 4.65E-02 | -0.013 | 0.002 | 3.00E-08 | TV | -0.020 | 0.010 | -0.017 | 0.014 | -0.019 | 0.014 | -0.021 | 0.012 | -0.014 | 0.016 |
| rs870151 | 9 | 92230747 | T | A | 0.005 | 0.002 | 1.14E-02 | -0.016 | 0.002 | 8.80E-13 | TV | 0.010 | 0.010 | 0.005 | 0.014 | 0.018 | 0.014 | 0.011 | 0.012 | 0.005 | 0.015 |
| rs2787374 | 9 | 103054951 | T | C | 0.001 | 0.002 | 6.60E-01 | 0.012 | 0.002 | 1.20E-08 | TV | 0.012 | 0.010 | -0.002 | 0.014 | 0.025 | 0.014 | 0.005 | 0.012 | 0.028 | 0.015 |
| rs7043521 | 9 | 124602779 | A | T | 0.001 | 0.002 | 7.61E-01 | 0.014 | 0.002 | 6.50E-11 | TV | -0.021 | 0.010 | -0.014 | 0.014 | -0.027 | 0.014 | -0.021 | 0.012 | -0.043 | 0.015 |
| rs4382592 | 9 | 134870755 | T | G | 0.002 | 0.002 | 3.75E-01 | 0.014 | 0.002 | 6.70E-09 | TV | 0.009 | 0.010 | 0.013 | 0.015 | 0.005 | 0.015 | 0.006 | 0.013 | 0.000 | 0.016 |
| rs2073869 | 9 | 135763816 | C | T | 0.001 | 0.003 | 6.77E-01 | 0.019 | 0.003 | 1.50E-10 | TV | -0.011 | 0.013 | -0.002 | 0.018 | -0.018 | 0.018 | -0.018 | 0.015 | -0.019 | 0.020 |
| rs1623025 | 10 | 11164813 | G | C | -0.004 | 0.002 | 6.59E-02 | -0.012 | 0.002 | 1.40E-08 | TV | 0.018 | 0.010 | 0.019 | 0.014 | 0.017 | 0.014 | 0.019 | 0.012 | 0.006 | 0.015 |
| rs1243182 | 10 | 21916728 | C | T | 0.000 | 0.002 | 9.78E-01 | -0.019 | 0.002 | 2.00E-15 | TV | -0.029 | 0.011 | -0.039 | 0.015 | -0.020 | 0.015 | -0.025 | 0.013 | -0.054 | 0.016 |
| rs2045147 | 10 | 56660437 | A | G | 0.004 | 0.002 | 3.23E-02 | 0.013 | 0.002 | 5.90E-09 | TV | 0.009 | 0.010 | 0.018 | 0.014 | -0.001 | 0.014 | 0.006 | 0.012 | 0.019 | 0.015 |
| rs10994943 | 10 | 63591413 | T | G | 0.001 | 0.002 | 4.50E-01 | 0.013 | 0.002 | 3.10E-09 | TV | 0.007 | 0.010 | 0.017 | 0.014 | -0.006 | 0.014 | 0.000 | 0.012 | 0.014 | 0.015 |
| rs11201422 | 10 | 86924483 | T | C | -0.003 | 0.002 | 2.21E-01 | 0.013 | 0.002 | 3.40E-08 | TV | 0.019 | 0.011 | 0.005 | 0.015 | 0.036 | 0.015 | 0.022 | 0.012 | 0.027 | 0.016 |
| rs10786658 | 10 | 103737193 | A | T | 0.001 | 0.002 | 6.64E-01 | -0.014 | 0.002 | 4.60E-11 | TV | 0.003 | 0.010 | 0.011 | 0.014 | -0.007 | 0.014 | 0.002 | 0.012 | -0.001 | 0.015 |
| rs7089973 | 10 | 116569565 | C | A | 0.005 | 0.002 | 2.30E-02 | -0.013 | 0.002 | 2.40E-09 | TV | -0.021 | 0.010 | -0.022 | 0.015 | -0.020 | 0.014 | -0.028 | 0.012 | -0.036 | 0.016 |
| rs17665139 | 10 | 125093880 | T | C | -0.012 | 0.002 | 1.59E-08 | -0.006 | 0.003 | 3.30E-02 | Alcohol | 0.002 | 0.014 | 0.019 | 0.019 | -0.012 | 0.019 | 0.021 | 0.016 | -0.041 | 0.022 |
| rs11245482 | 10 | 126733546 | T | C | 0.002 | 0.002 | 4.27E-01 | -0.013 | 0.002 | 2.60E-09 | TV | -0.004 | 0.010 | 0.006 | 0.014 | -0.016 | 0.014 | -0.002 | 0.012 | -0.008 | 0.015 |
| rs17727474 | 10 | 127182959 | C | T | 0.006 | 0.003 | 1.28E-02 | 0.018 | 0.003 | 3.10E-09 | TV | 0.001 | 0.013 | -0.001 | 0.019 | 0.005 | 0.019 | -0.009 | 0.016 | 0.010 | 0.021 |
| rs7950166 | 11 | 8642218 | T | C | -0.010 | 0.002 | 9.89E-11 | 0.003 | 0.002 | 1.70E-01 | Alcohol | 0.000 | 0.010 | -0.003 | 0.014 | 0.007 | 0.014 | 0.004 | 0.012 | -0.003 | 0.016 |
| rs12289262 | 11 | 12894758 | C | T | 0.000 | 0.002 | 8.95E-01 | -0.014 | 0.002 | 2.00E-08 | TV | -0.028 | 0.011 | -0.029 | 0.016 | -0.029 | 0.016 | -0.030 | 0.014 | -0.010 | 0.018 |
| rs11030084 | 11 | 27643725 | T | C | -0.011 | 0.002 | 1.72E-08 | 0.004 | 0.003 | 1.70E-01 | Alcohol | -0.003 | 0.012 | -0.017 | 0.017 | 0.011 | 0.017 | -0.007 | 0.015 | -0.012 | 0.019 |
| rs56030824 | 11 | 47397353 | A | G | -0.012 | 0.002 | 1.15E-13 | 0.005 | 0.002 | 2.00E-02 | Alcohol | 0.009 | 0.010 | 0.013 | 0.014 | 0.005 | 0.014 | 0.005 | 0.012 | -0.004 | 0.016 |
| rs801733 | 11 | 65934549 | A | C | -0.004 | 0.002 | 3.86E-02 | 0.017 | 0.002 | 7.30E-14 | TV | 0.027 | 0.010 | 0.028 | 0.014 | 0.026 | 0.014 | 0.023 | 0.012 | 0.048 | 0.016 |
| rs116175338 | 11 | 72467875 | G | T | 0.003 | 0.002 | 1.36E-01 | -0.014 | 0.003 | 1.40E-08 | TV | -0.001 | 0.011 | 0.011 | 0.016 | -0.011 | 0.016 | -0.002 | 0.014 | 0.020 | 0.018 |
| rs12272012 | 11 | 84861286 | G | C | 0.000 | 0.005 | 9.22E-01 | -0.030 | 0.005 | 1.90E-08 | TV | 0.031 | 0.024 | 0.000 | 0.034 | 0.062 | 0.033 | 0.036 | 0.028 | 0.064 | 0.037 |
| rs11020045 | 11 | 92487907 | A | C | 0.000 | 0.002 | 8.94E-01 | -0.013 | 0.002 | 1.60E-08 | TV | 0.007 | 0.010 | 0.021 | 0.015 | -0.006 | 0.015 | 0.018 | 0.012 | -0.003 | 0.016 |
| rs17207890 | 11 | 95490754 | G | A | 0.002 | 0.002 | 2.74E-01 | 0.016 | 0.002 | 6.70E-12 | TV | 0.000 | 0.010 | 0.012 | 0.015 | -0.010 | 0.015 | 0.016 | 0.012 | -0.021 | 0.016 |
| rs10750025 | 11 | 113424042 | T | C | 0.010 | 0.002 | 4.89E-11 | -0.005 | 0.002 | 4.40E-02 | Alcohol | -0.006 | 0.010 | -0.023 | 0.015 | 0.008 | 0.015 | -0.010 | 0.012 | -0.017 | 0.016 |
| rs682011 | 11 | 121544285 | C | T | 0.008 | 0.001 | 2.22E-08 | 0.008 | 0.002 | 2.70E-04 | Alcohol | -0.021 | 0.010 | -0.021 | 0.014 | -0.024 | 0.014 | -0.022 | 0.012 | -0.007 | 0.015 |
| rs11218575 | 11 | 122154576 | C | T | 0.000 | 0.002 | 9.54E-01 | 0.015 | 0.002 | 2.10E-12 | TV | 0.001 | 0.010 | 0.013 | 0.014 | -0.010 | 0.014 | 0.001 | 0.012 | -0.001 | 0.015 |
| rs12795042 | 11 | 133658168 | C | A | -0.008 | 0.002 | 3.25E-08 | 0.001 | 0.002 | 6.70E-01 | Alcohol | -0.010 | 0.010 | -0.002 | 0.014 | -0.019 | 0.014 | -0.017 | 0.012 | 0.009 | 0.016 |
| rs4937842 | 11 | 133734542 | G | C | 0.002 | 0.002 | 3.05E-01 | -0.013 | 0.002 | 1.40E-08 | TV | -0.012 | 0.010 | -0.018 | 0.014 | -0.008 | 0.014 | -0.018 | 0.012 | -0.023 | 0.016 |
| rs10772643 | 12 | 13415288 | C | T | 0.003 | 0.003 | 2.82E-01 | 0.025 | 0.003 | 1.30E-12 | TV | 0.027 | 0.016 | 0.041 | 0.022 | 0.014 | 0.022 | 0.024 | 0.019 | 0.056 | 0.024 |
| rs10771746 | 12 | 30791864 | C | T | 0.001 | 0.002 | 6.00E-01 | -0.014 | 0.002 | 2.50E-09 | TV | 0.006 | 0.010 | -0.008 | 0.015 | 0.017 | 0.015 | 0.001 | 0.013 | 0.031 | 0.017 |
| rs10876188 | 12 | 51895882 | T | C | -0.008 | 0.001 | 4.84E-08 | -0.006 | 0.002 | 7.10E-03 | Alcohol | 0.020 | 0.010 | 0.023 | 0.014 | 0.017 | 0.014 | 0.031 | 0.012 | 0.010 | 0.015 |
| rs10876864 | 12 | 56401085 | G | A | 0.004 | 0.002 | 3.26E-02 | -0.013 | 0.002 | 1.00E-09 | TV | -0.010 | 0.010 | -0.005 | 0.014 | -0.018 | 0.014 | -0.029 | 0.012 | 0.021 | 0.015 |
| rs8756 | 12 | 66359752 | C | A | 0.003 | 0.002 | 9.39E-02 | -0.013 | 0.002 | 5.30E-10 | TV | -0.009 | 0.010 | -0.008 | 0.014 | -0.009 | 0.014 | -0.005 | 0.012 | -0.016 | 0.015 |
| rs10506274 | 12 | 81601464 | T | G | -0.009 | 0.001 | 5.78E-10 | 0.003 | 0.002 | 1.90E-01 | Alcohol | 0.014 | 0.010 | 0.003 | 0.014 | 0.023 | 0.014 | 0.012 | 0.012 | 0.024 | 0.015 |
| rs4842786 | 12 | 92170791 | A | G | -0.009 | 0.001 | 2.73E-09 | 0.005 | 0.002 | 3.70E-02 | Alcohol | -0.013 | 0.010 | -0.011 | 0.014 | -0.018 | 0.014 | -0.011 | 0.012 | -0.030 | 0.015 |
| rs2164744 | 12 | 109872039 | T | C | -0.003 | 0.002 | 1.26E-01 | -0.013 | 0.002 | 1.10E-08 | TV | 0.014 | 0.010 | 0.013 | 0.014 | 0.015 | 0.014 | 0.007 | 0.012 | 0.027 | 0.015 |
| rs2173650 | 12 | 117525235 | G | T | 0.001 | 0.003 | 6.30E-01 | 0.018 | 0.003 | 4.60E-09 | TV | -0.006 | 0.013 | 0.016 | 0.019 | -0.028 | 0.019 | -0.002 | 0.016 | 0.010 | 0.021 |
| rs500321 | 13 | 27124360 | T | A | -0.010 | 0.002 | 4.92E-09 | -0.001 | 0.002 | 5.90E-01 | Alcohol | 0.008 | 0.011 | 0.024 | 0.016 | -0.010 | 0.016 | 0.001 | 0.013 | 0.020 | 0.017 |
| rs9563168 | 13 | 54247827 | G | A | -0.005 | 0.002 | 2.96E-02 | 0.018 | 0.003 | 4.30E-11 | TV | 0.006 | 0.012 | -0.004 | 0.017 | 0.016 | 0.017 | 0.010 | 0.014 | -0.013 | 0.018 |
| rs9569734 | 13 | 58319476 | A | G | 0.003 | 0.003 | 1.97E-01 | 0.019 | 0.003 | 3.40E-10 | TV | 0.010 | 0.013 | 0.024 | 0.019 | -0.011 | 0.019 | 0.012 | 0.016 | 0.037 | 0.021 |
| rs56858768 | 13 | 86511730 | G | A | 0.000 | 0.002 | 8.78E-01 | -0.015 | 0.002 | 3.40E-10 | TV | -0.010 | 0.011 | -0.006 | 0.015 | -0.015 | 0.015 | -0.012 | 0.013 | -0.019 | 0.017 |
| rs7991062 | 13 | 100713194 | C | G | -0.008 | 0.002 | 1.45E-04 | -0.018 | 0.002 | 8.80E-15 | TV | -0.013 | 0.010 | -0.011 | 0.014 | -0.015 | 0.014 | -0.012 | 0.012 | -0.017 | 0.016 |
| rs405797 | 14 | 26972834 | T | A | 0.005 | 0.002 | 3.65E-02 | -0.015 | 0.003 | 2.80E-09 | TV | -0.014 | 0.013 | -0.009 | 0.016 | -0.023 | 0.016 | -0.017 | 0.013 | -0.006 | 0.017 |
| rs1022785 | 14 | 29571653 | G | A | 0.002 | 0.003 | 3.71E-01 | 0.018 | 0.003 | 7.10E-09 | TV | -0.003 | 0.016 | 0.027 | 0.019 | -0.024 | 0.019 | -0.005 | 0.016 | 0.035 | 0.021 |
| rs1123285 | 14 | 57274519 | G | C | -0.009 | 0.002 | 8.14E-09 | -0.006 | 0.002 | 1.30E-02 | Alcohol | -0.013 | 0.012 | -0.015 | 0.014 | -0.012 | 0.015 | -0.015 | 0.012 | -0.006 | 0.016 |
| rs10145592 | 14 | 94287215 | C | G | 0.008 | 0.002 | 5.41E-05 | -0.015 | 0.002 | 1.80E-11 | TV | -0.023 | 0.012 | -0.017 | 0.014 | -0.014 | 0.014 | -0.019 | 0.012 | -0.029 | 0.015 |
| rs7157001 | 14 | 99749484 | A | G | -0.003 | 0.002 | 1.16E-01 | -0.014 | 0.002 | 2.30E-08 | TV | -0.007 | 0.014 | -0.005 | 0.017 | -0.013 | 0.017 | -0.003 | 0.014 | -0.051 | 0.019 |
| rs8043253 | 15 | 41503730 | C | T | 0.006 | 0.002 | 1.66E-03 | -0.012 | 0.002 | 3.40E-08 | TV | 0.017 | 0.010 | 0.013 | 0.014 | 0.019 | 0.014 | 0.028 | 0.012 | -0.002 | 0.015 |
| rs2460 | 15 | 53073084 | G | A | 0.000 | 0.002 | 9.83E-01 | -0.015 | 0.002 | 5.00E-10 | TV | -0.015 | 0.011 | 0.004 | 0.016 | -0.032 | 0.016 | -0.021 | 0.013 | 0.015 | 0.018 |
| rs4775373 | 15 | 61476936 | T | C | -0.002 | 0.002 | 3.92E-01 | 0.013 | 0.002 | 4.70E-09 | TV | 0.006 | 0.010 | 0.002 | 0.014 | 0.009 | 0.014 | 0.007 | 0.012 | 0.017 | 0.016 |
| rs2472297 | 15 | 75027880 | T | C | 0.011 | 0.002 | 3.10E-10 | 0.003 | 0.002 | 2.30E-01 | Alcohol | -0.006 | 0.013 | 0.004 | 0.018 | -0.017 | 0.018 | -0.018 | 0.015 | 0.015 | 0.020 |
| rs61331678 | 15 | 77995949 | G | C | 0.000 | 0.002 | 8.35E-01 | 0.015 | 0.002 | 6.10E-12 | TV | -0.011 | 0.010 | 0.004 | 0.014 | -0.025 | 0.014 | -0.015 | 0.012 | -0.011 | 0.015 |
| rs12907323 | 15 | 86796012 | G | A | 0.008 | 0.001 | 9.93E-09 | -0.007 | 0.002 | 1.10E-03 | Alcohol | -0.002 | 0.010 | -0.005 | 0.014 | 0.003 | 0.014 | 0.001 | 0.012 | -0.016 | 0.015 |
| rs35574015 | 16 | 15146061 | T | C | -0.001 | 0.002 | 7.98E-01 | -0.013 | 0.002 | 3.00E-08 | TV | 0.020 | 0.010 | 0.028 | 0.015 | 0.011 | 0.015 | 0.018 | 0.013 | -0.001 | 0.016 |
| rs7189927 | 16 | 28913787 | T | C | -0.013 | 0.002 | 5.93E-11 | 0.015 | 0.002 | 3.40E-11 | TV | 0.009 | 0.010 | 0.006 | 0.015 | 0.012 | 0.015 | 0.016 | 0.012 | 0.009 | 0.016 |
| rs749671 | 16 | 31088347 | G | A | -0.004 | 0.002 | 2.94E-02 | 0.016 | 0.002 | 2.70E-12 | TV | 0.023 | 0.010 | 0.018 | 0.014 | 0.028 | 0.014 | 0.023 | 0.012 | 0.015 | 0.016 |
| rs7184800 | 16 | 53509131 | G | A | -0.002 | 0.002 | 2.51E-01 | 0.017 | 0.002 | 8.20E-13 | TV | 0.005 | 0.011 | -0.003 | 0.015 | 0.011 | 0.015 | 0.003 | 0.013 | 0.024 | 0.016 |
| rs7185555 | 16 | 69131281 | C | G | -0.011 | 0.002 | 4.24E-08 | -0.006 | 0.003 | 3.20E-02 | Alcohol | -0.004 | 0.014 | 0.012 | 0.019 | -0.018 | 0.019 | -0.006 | 0.016 | -0.003 | 0.021 |
| rs79616692 | 16 | 72338507 | C | G | 0.016 | 0.002 | 4.11E-12 | -0.008 | 0.004 | 1.70E-02 | Alcohol | -0.017 | 0.016 | -0.045 | 0.022 | 0.010 | 0.022 | -0.023 | 0.019 | -0.038 | 0.025 |
| rs2447098 | 17 | 2277720 | C | A | 0.000 | 0.002 | 9.08E-01 | -0.015 | 0.002 | 6.80E-12 | TV | -0.011 | 0.010 | -0.034 | 0.014 | 0.009 | 0.014 | -0.010 | 0.012 | -0.003 | 0.015 |
| rs11654952 | 17 | 4813799 | T | G | 0.003 | 0.003 | 2.49E-01 | -0.017 | 0.003 | 4.30E-08 | TV | 0.025 | 0.014 | 0.005 | 0.019 | 0.047 | 0.020 | 0.018 | 0.017 | 0.001 | 0.022 |
| rs2854334 | 17 | 29715500 | G | A | 0.009 | 0.001 | 7.51E-10 | -0.001 | 0.002 | 6.00E-01 | Alcohol | -0.029 | 0.010 | -0.028 | 0.014 | -0.029 | 0.014 | -0.042 | 0.012 | -0.042 | 0.016 |
| rs9902312 | 17 | 65070304 | T | C | 0.002 | 0.002 | 2.98E-01 | 0.015 | 0.002 | 4.50E-11 | TV | -0.001 | 0.010 | -0.005 | 0.015 | 0.004 | 0.015 | 0.000 | 0.012 | 0.010 | 0.016 |
| rs11657730 | 17 | 79372489 | C | T | 0.001 | 0.002 | 4.96E-01 | 0.013 | 0.002 | 1.10E-08 | TV | 0.010 | 0.011 | 0.007 | 0.015 | 0.012 | 0.015 | 0.010 | 0.012 | 0.016 | 0.016 |
| rs303753 | 18 | 21074922 | G | A | 0.007 | 0.002 | 3.24E-04 | -0.014 | 0.002 | 2.70E-10 | TV | -0.013 | 0.010 | -0.029 | 0.015 | 0.001 | 0.015 | -0.008 | 0.012 | -0.022 | 0.016 |
| rs9964724 | 18 | 35159124 | C | T | 0.002 | 0.002 | 3.50E-01 | 0.018 | 0.002 | 3.30E-14 | TV | 0.009 | 0.010 | 0.014 | 0.015 | 0.004 | 0.015 | 0.011 | 0.012 | 0.012 | 0.016 |
| rs1156541 | 18 | 39952989 | C | T | 0.000 | 0.002 | 9.54E-01 | 0.014 | 0.003 | 2.80E-08 | TV | 0.017 | 0.011 | 0.016 | 0.016 | 0.019 | 0.016 | 0.015 | 0.014 | 0.043 | 0.018 |
| rs17512836 | 18 | 53194961 | T | C | -0.005 | 0.006 | 4.40E-01 | 0.042 | 0.007 | 7.00E-10 | TV | -0.014 | 0.028 | -0.018 | 0.040 | -0.009 | 0.040 | 0.019 | 0.033 | -0.075 | 0.043 |
| rs7248205 | 19 | 10770305 | C | T | 0.002 | 0.002 | 3.57E-01 | 0.014 | 0.002 | 3.40E-10 | TV | -0.029 | 0.010 | -0.046 | 0.014 | -0.010 | 0.014 | -0.021 | 0.012 | -0.023 | 0.016 |
| rs111901094 | 19 | 19513570 | G | T | -0.003 | 0.003 | 2.16E-01 | -0.017 | 0.003 | 2.00E-09 | TV | -0.011 | 0.013 | 0.022 | 0.018 | -0.044 | 0.018 | 0.003 | 0.016 | -0.022 | 0.020 |
| rs281379 | 19 | 49214274 | A | G | 0.014 | 0.001 | 4.91E-21 | 0.001 | 0.002 | 5.60E-01 | Alcohol | 0.010 | 0.010 | -0.004 | 0.014 | 0.025 | 0.014 | 0.019 | 0.012 | -0.004 | 0.015 |
| rs6131281 | 20 | 11891724 | C | T | 0.006 | 0.002 | 1.36E-03 | 0.016 | 0.002 | 3.10E-13 | TV | 0.017 | 0.010 | 0.021 | 0.014 | 0.011 | 0.014 | 0.020 | 0.012 | 0.014 | 0.015 |
| rs6141814 | 20 | 31368960 | C | A | -0.005 | 0.002 | 1.55E-02 | -0.014 | 0.002 | 1.30E-09 | TV | 0.013 | 0.010 | 0.020 | 0.014 | 0.004 | 0.014 | 0.015 | 0.012 | 0.003 | 0.016 |
| rs55700114 | 20 | 43717080 | G | A | -0.001 | 0.002 | 5.01E-01 | -0.014 | 0.002 | 2.10E-09 | TV | -0.001 | 0.011 | -0.006 | 0.015 | 0.006 | 0.015 | -0.002 | 0.013 | -0.006 | 0.016 |
| rs56103247 | 20 | 62483184 | C | T | 0.005 | 0.004 | 2.74E-01 | 0.030 | 0.005 | 3.80E-10 | TV | -0.077 | 0.024 | -0.075 | 0.035 | -0.080 | 0.034 | -0.074 | 0.029 | -0.074 | 0.038 |
| rs10427502 | 21 | 40654840 | G | A | -0.002 | 0.002 | 2.27E-01 | 0.014 | 0.002 | 8.60E-10 | TV | 0.001 | 0.010 | 0.024 | 0.014 | -0.017 | 0.014 | -0.006 | 0.012 | 0.000 | 0.016 |
| rs2092829 | 22 | 31780514 | G | A | 0.004 | 0.002 | 3.89E-02 | 0.014 | 0.002 | 1.30E-08 | TV | -0.023 | 0.011 | -0.027 | 0.015 | -0.016 | 0.015 | -0.026 | 0.013 | -0.029 | 0.017 |
| rs9607814 | 22 | 41946519 | A | C | -0.010 | 0.002 | 4.31E-08 | 0.006 | 0.003 | 2.90E-02 | Alcohol | 0.001 | 0.013 | -0.010 | 0.018 | 0.015 | 0.018 | -0.004 | 0.015 | 0.014 | 0.019 |

| **Table S14. Beta estimates for the SNPs included in the multivariable MR of BMI, computer use, and breast cancer risk** | | | | | | | | | | | | | | | | | | | | | | | |
| --- | --- | --- | --- | --- | --- | --- | --- | --- | --- | --- | --- | --- | --- | --- | --- | --- | --- | --- | --- | --- | --- | --- | --- |
| SNP | CHR | POS | EA | OA | beta_bmi | se_bmi | pval_bmi | beta_pc | se_pc | pval | phenotype | beta_  br | se_br | Lum_A  _beta | Lum_A  _se | Lum_B  _beta | Lum_B  _se | HER2Neg  _beta | HER2Neg  _se | HER2_  Enriched  _beta | HER2_  Enriched  _se | 3N_  beta | 3N_se |
| rs2748985 | 1 | 1853184 | T | C | -0.015 | 0.003 | 3.64E-08 | -0.015 | 0.002 | 4.10E-12 | PC | 0.003 | 0.007 | 0.007 | 0.009 | 0.017 | 0.019 | -0.002 | 0.017 | -0.011 | 0.028 | 0.014 | 0.017 |
| rs10909880 | 1 | 2727804 | T | C | -0.012 | 0.002 | 1.97E-08 | -0.006 | 0.002 | 7.30E-03 | BMI | 0.008 | 0.006 | 0.011 | 0.008 | -0.006 | 0.018 | -0.010 | 0.015 | -0.019 | 0.026 | 0.008 | 0.015 |
| rs78886584 | 1 | 16859325 | A | G | -0.015 | 0.003 | 1.30E-08 | 0.002 | 0.002 | 4.30E-01 | BMI | -0.002 | 0.007 | -0.006 | 0.009 | 0.016 | 0.019 | 0.006 | 0.016 | -0.031 | 0.028 | 0.011 | 0.017 |
| rs4295917 | 1 | 32173360 | A | G | 0.014 | 0.002 | 1.62E-08 | 0.000 | 0.002 | 9.10E-01 | BMI | -0.009 | 0.007 | -0.019 | 0.009 | -0.006 | 0.019 | -0.001 | 0.017 | -0.013 | 0.028 | -0.011 | 0.017 |
| rs112646560 | 1 | 39560250 | T | C | 0.020 | 0.003 | 1.14E-09 | 0.003 | 0.003 | 3.40E-01 | BMI | 0.010 | 0.008 | 0.005 | 0.010 | -0.019 | 0.022 | 0.028 | 0.019 | 0.113 | 0.031 | -0.032 | 0.019 |
| rs2984618 | 1 | 47690438 | T | G | 0.017 | 0.002 | 2.21E-14 | 0.004 | 0.002 | 9.30E-02 | BMI | -0.002 | 0.006 | -0.004 | 0.008 | -0.007 | 0.018 | -0.024 | 0.015 | 0.018 | 0.026 | -0.012 | 0.016 |
| rs657452 | 1 | 49589847 | A | G | 0.020 | 0.002 | 1.14E-19 | -0.005 | 0.002 | 4.00E-02 | BMI | -0.006 | 0.006 | -0.010 | 0.008 | 0.007 | 0.018 | -0.005 | 0.015 | -0.001 | 0.026 | -0.024 | 0.016 |
| rs12140153 | 1 | 62579891 | T | G | -0.033 | 0.005 | 2.87E-12 | 0.004 | 0.004 | 3.20E-01 | BMI | 0.014 | 0.011 | 0.035 | 0.015 | -0.001 | 0.033 | 0.011 | 0.028 | -0.076 | 0.049 | -0.011 | 0.029 |
| rs2568958 | 1 | 72765116 | A | G | 0.023 | 0.002 | 1.32E-24 | 0.007 | 0.002 | 1.20E-03 | BMI | -0.014 | 0.006 | -0.012 | 0.008 | -0.027 | 0.018 | -0.016 | 0.015 | -0.031 | 0.026 | -0.021 | 0.016 |
| rs6669189 | 1 | 75011358 | T | C | 0.021 | 0.002 | 4.69E-21 | -0.004 | 0.002 | 5.80E-02 | BMI | -0.004 | 0.006 | -0.001 | 0.008 | -0.016 | 0.018 | 0.011 | 0.015 | 0.004 | 0.026 | -0.001 | 0.016 |
| rs34517439 | 1 | 78450517 | A | C | 0.039 | 0.004 | 2.00E-21 | 0.003 | 0.003 | 3.90E-01 | BMI | 0.004 | 0.010 | 0.014 | 0.013 | -0.003 | 0.030 | -0.049 | 0.026 | 0.011 | 0.043 | -0.003 | 0.026 |
| rs12089815 | 1 | 91189933 | A | G | -0.015 | 0.003 | 6.02E-09 | 0.003 | 0.002 | 1.90E-01 | BMI | 0.008 | 0.006 | 0.000 | 0.009 | 0.024 | 0.019 | 0.016 | 0.016 | -0.019 | 0.027 | 0.007 | 0.016 |
| rs11165643 | 1 | 96924097 | T | C | 0.018 | 0.002 | 1.71E-16 | 0.003 | 0.002 | 1.30E-01 | BMI | -0.005 | 0.006 | -0.008 | 0.008 | -0.006 | 0.018 | -0.030 | 0.016 | 0.007 | 0.027 | 0.001 | 0.016 |
| rs4421623 | 1 | 98316285 | T | G | 0.018 | 0.003 | 1.13E-11 | -0.002 | 0.003 | 4.70E-01 | BMI | 0.003 | 0.007 | 0.007 | 0.009 | -0.009 | 0.021 | 0.001 | 0.018 | -0.034 | 0.030 | 0.011 | 0.018 |
| rs12145677 | 1 | 110023610 | G | A | -0.011 | 0.002 | 1.50E-05 | -0.017 | 0.002 | 1.20E-12 | PC | -0.011 | 0.007 | -0.016 | 0.009 | -0.012 | 0.020 | -0.003 | 0.017 | -0.009 | 0.028 | 0.013 | 0.017 |
| rs61813324 | 1 | 156049877 | T | C | 0.030 | 0.004 | 3.60E-14 | 0.000 | 0.003 | 9.50E-01 | BMI | -0.019 | 0.010 | -0.031 | 0.013 | -0.019 | 0.028 | -0.032 | 0.024 | -0.042 | 0.042 | -0.003 | 0.025 |
| rs1750307 | 1 | 156488420 | A | T | 0.014 | 0.002 | 8.22E-09 | -0.002 | 0.002 | 3.00E-01 | BMI | -0.001 | 0.006 | 0.005 | 0.008 | 0.011 | 0.018 | -0.013 | 0.016 | 0.039 | 0.026 | -0.027 | 0.016 |
| rs12564992 | 1 | 174478100 | A | G | -0.021 | 0.004 | 2.37E-09 | -0.013 | 0.004 | 1.70E-04 | BMI | -0.002 | 0.009 | -0.005 | 0.012 | 0.051 | 0.028 | -0.022 | 0.023 | 0.022 | 0.040 | -0.007 | 0.024 |
| rs543874 | 1 | 177889480 | A | G | -0.058 | 0.003 | 8.70E-100 | 0.002 | 0.003 | 4.50E-01 | BMI | 0.034 | 0.008 | 0.033 | 0.010 | 0.059 | 0.023 | 0.028 | 0.019 | 0.025 | 0.033 | 0.011 | 0.019 |
| rs10920678 | 1 | 190239907 | A | G | 0.016 | 0.002 | 7.46E-14 | 0.001 | 0.002 | 7.70E-01 | BMI | -0.003 | 0.006 | -0.009 | 0.008 | 0.009 | 0.018 | 0.001 | 0.016 | 0.001 | 0.026 | 0.013 | 0.016 |
| rs4474229 | 1 | 195037001 | A | G | -0.017 | 0.003 | 1.64E-10 | -0.002 | 0.002 | 4.80E-01 | BMI | 0.003 | 0.006 | 0.006 | 0.008 | 0.009 | 0.018 | -0.004 | 0.015 | -0.035 | 0.026 | 0.005 | 0.016 |
| rs7526456 | 1 | 197773734 | T | C | 0.010 | 0.002 | 3.75E-05 | -0.013 | 0.002 | 1.20E-08 | PC | 0.005 | 0.006 | 0.005 | 0.009 | 0.012 | 0.019 | 0.002 | 0.016 | -0.039 | 0.028 | 0.014 | 0.016 |
| rs2820295 | 1 | 201800868 | A | G | 0.021 | 0.002 | 8.03E-19 | -0.006 | 0.002 | 1.00E-02 | BMI | -0.006 | 0.006 | -0.007 | 0.008 | 0.009 | 0.019 | 0.026 | 0.016 | 0.030 | 0.027 | -0.017 | 0.016 |
| rs17014375 | 1 | 209543560 | T | G | -0.019 | 0.003 | 1.46E-08 | 0.009 | 0.003 | 6.10E-03 | BMI | 0.000 | 0.009 | -0.007 | 0.012 | 0.012 | 0.026 | 0.003 | 0.022 | 0.012 | 0.038 | -0.007 | 0.022 |
| rs12042959 | 1 | 243533273 | A | G | 0.019 | 0.003 | 2.33E-09 | -0.008 | 0.003 | 1.70E-02 | BMI | 0.020 | 0.009 | 0.015 | 0.011 | 0.020 | 0.025 | 0.040 | 0.022 | 0.060 | 0.037 | 0.010 | 0.022 |
| rs62106258 | 2 | 417167 | T | C | 0.099 | 0.006 | 8.72E-58 | 0.009 | 0.005 | 7.80E-02 | BMI | -0.006 | 0.017 | -0.020 | 0.023 | 0.002 | 0.049 | -0.082 | 0.041 | 0.084 | 0.074 | -0.027 | 0.042 |
| rs6548237 | 2 | 621461 | A | C | -0.058 | 0.003 | 4.43E-89 | -0.008 | 0.003 | 3.50E-03 | BMI | 0.020 | 0.010 | 0.020 | 0.013 | 0.017 | 0.029 | 0.023 | 0.025 | 0.128 | 0.041 | 0.026 | 0.025 |
| rs7571496 | 2 | 6169351 | A | G | 0.015 | 0.003 | 1.45E-08 | -0.002 | 0.003 | 3.80E-01 | BMI | -0.007 | 0.007 | -0.013 | 0.009 | -0.003 | 0.020 | -0.023 | 0.017 | -0.002 | 0.030 | 0.009 | 0.018 |
| rs7601870 | 2 | 15384392 | A | G | -0.013 | 0.002 | 1.23E-08 | 0.003 | 0.002 | 2.10E-01 | BMI | 0.006 | 0.006 | 0.004 | 0.008 | -0.011 | 0.018 | 0.008 | 0.015 | 0.001 | 0.026 | 0.002 | 0.015 |
| rs10182181 | 2 | 25150296 | A | G | -0.036 | 0.002 | 5.00E-63 | -0.004 | 0.002 | 1.10E-01 | BMI | 0.035 | 0.006 | 0.031 | 0.008 | 0.047 | 0.018 | 0.013 | 0.016 | 0.070 | 0.027 | 0.050 | 0.016 |
| rs10048652 | 2 | 35449306 | T | C | 0.014 | 0.002 | 3.47E-09 | 0.000 | 0.002 | 9.30E-01 | BMI | 0.002 | 0.006 | 0.009 | 0.008 | -0.044 | 0.018 | 0.004 | 0.015 | -0.011 | 0.026 | 0.009 | 0.015 |
| rs786420 | 2 | 44719893 | T | C | 0.016 | 0.003 | 8.84E-09 | 0.007 | 0.003 | 4.70E-03 | BMI | -0.014 | 0.007 | -0.010 | 0.009 | -0.004 | 0.021 | -0.027 | 0.017 | -0.036 | 0.030 | 0.019 | 0.018 |
| rs35722922 | 2 | 47002226 | A | G | 0.019 | 0.003 | 3.65E-12 | 0.002 | 0.002 | 4.40E-01 | BMI | 0.000 | 0.006 | 0.008 | 0.008 | 0.022 | 0.018 | -0.018 | 0.016 | 0.032 | 0.027 | -0.017 | 0.016 |
| rs1421598 | 2 | 50264710 | A | C | -0.019 | 0.003 | 6.78E-10 | 0.001 | 0.003 | 7.80E-01 | BMI | -0.012 | 0.008 | -0.025 | 0.011 | -0.002 | 0.024 | -0.033 | 0.020 | 0.042 | 0.035 | -0.021 | 0.021 |
| rs7601895 | 2 | 55281901 | C | G | 0.016 | 0.003 | 9.61E-11 | 0.004 | 0.002 | 9.70E-02 | BMI | 0.006 | 0.007 | 0.005 | 0.009 | 0.018 | 0.020 | 0.033 | 0.017 | -0.016 | 0.029 | -0.005 | 0.017 |
| rs1861412 | 2 | 58893065 | A | G | -0.022 | 0.002 | 9.13E-21 | -0.005 | 0.002 | 1.90E-02 | BMI | 0.004 | 0.006 | 0.007 | 0.008 | -0.020 | 0.018 | -0.016 | 0.015 | 0.031 | 0.026 | -0.011 | 0.015 |
| rs6728037 | 2 | 59297346 | A | G | 0.021 | 0.002 | 6.49E-19 | -0.001 | 0.002 | 6.50E-01 | BMI | 0.000 | 0.006 | 0.008 | 0.008 | -0.012 | 0.018 | -0.006 | 0.015 | -0.006 | 0.026 | 0.025 | 0.016 |
| rs2861685 | 2 | 67837553 | T | C | 0.018 | 0.003 | 1.05E-11 | 0.003 | 0.002 | 1.40E-01 | BMI | 0.018 | 0.006 | 0.017 | 0.008 | 0.008 | 0.018 | -0.002 | 0.015 | -0.013 | 0.026 | 0.038 | 0.015 |
| rs12714199 | 2 | 86812549 | T | C | -0.015 | 0.002 | 5.12E-10 | -0.001 | 0.002 | 7.90E-01 | BMI | 0.007 | 0.006 | 0.015 | 0.008 | 0.010 | 0.018 | 0.009 | 0.016 | -0.060 | 0.026 | 0.020 | 0.016 |
| rs11677607 | 2 | 100751150 | T | C | -0.024 | 0.003 | 2.87E-19 | 0.006 | 0.003 | 3.10E-02 | BMI | 0.012 | 0.007 | 0.003 | 0.009 | 0.019 | 0.021 | 0.007 | 0.018 | -0.016 | 0.030 | 0.029 | 0.018 |
| rs1457457 | 2 | 104340148 | A | T | 0.014 | 0.002 | 3.79E-10 | -0.004 | 0.002 | 7.60E-02 | BMI | 0.015 | 0.006 | 0.012 | 0.008 | -0.015 | 0.018 | 0.008 | 0.015 | 0.002 | 0.026 | 0.017 | 0.015 |
| rs6739199 | 2 | 105452326 | T | C | 0.016 | 0.003 | 3.20E-10 | -0.007 | 0.002 | 4.30E-03 | BMI | 0.000 | 0.007 | 0.012 | 0.009 | -0.027 | 0.021 | -0.002 | 0.018 | -0.033 | 0.031 | -0.002 | 0.018 |
| rs1984559 | 2 | 135678888 | A | G | 0.016 | 0.003 | 3.27E-08 | 0.001 | 0.003 | 6.70E-01 | BMI | 0.019 | 0.007 | 0.028 | 0.010 | 0.047 | 0.021 | 0.012 | 0.018 | 0.027 | 0.030 | 0.027 | 0.018 |
| rs13002158 | 2 | 144021712 | A | G | -0.021 | 0.003 | 1.15E-12 | -0.006 | 0.003 | 3.60E-02 | BMI | 0.016 | 0.008 | 0.010 | 0.011 | -0.010 | 0.023 | 0.015 | 0.020 | 0.063 | 0.035 | 0.028 | 0.021 |
| rs76900466 | 2 | 145057760 | T | G | 0.023 | 0.004 | 4.53E-08 | -0.001 | 0.003 | 8.00E-01 | BMI | -0.018 | 0.012 | -0.013 | 0.016 | -0.004 | 0.035 | 0.008 | 0.031 | 0.023 | 0.052 | -0.008 | 0.031 |
| rs10754920 | 2 | 146456888 | C | G | 0.008 | 0.004 | 3.86E-02 | 0.020 | 0.003 | 6.70E-09 | PC | 0.004 | 0.010 | 0.016 | 0.013 | 0.005 | 0.028 | 0.015 | 0.024 | 0.014 | 0.040 | -0.064 | 0.025 |
| rs6744254 | 2 | 155986195 | C | T | -0.005 | 0.002 | 4.60E-02 | -0.016 | 0.002 | 5.40E-13 | PC | 0.007 | 0.006 | 0.002 | 0.008 | -0.014 | 0.018 | 0.005 | 0.015 | 0.024 | 0.026 | 0.004 | 0.015 |
| rs7588437 | 2 | 181575281 | A | G | -0.018 | 0.002 | 8.38E-15 | 0.002 | 0.002 | 3.50E-01 | BMI | 0.015 | 0.006 | 0.009 | 0.008 | 0.027 | 0.018 | 0.025 | 0.016 | 0.019 | 0.027 | 0.036 | 0.016 |
| rs7594237 | 2 | 198187164 | A | T | -0.014 | 0.003 | 1.81E-08 | 0.003 | 0.002 | 1.90E-01 | BMI | -0.017 | 0.007 | -0.010 | 0.009 | -0.032 | 0.020 | -0.058 | 0.017 | -0.032 | 0.029 | -0.023 | 0.018 |
| rs4482463 | 2 | 205375909 | A | C | -0.037 | 0.004 | 5.21E-18 | -0.005 | 0.004 | 2.60E-01 | BMI | 0.012 | 0.011 | 0.016 | 0.015 | -0.047 | 0.033 | 0.035 | 0.029 | 0.070 | 0.050 | -0.012 | 0.029 |
| rs4673553 | 2 | 211608379 | T | G | -0.015 | 0.002 | 2.59E-11 | 0.000 | 0.002 | 9.00E-01 | BMI | 0.004 | 0.006 | 0.012 | 0.008 | 0.010 | 0.018 | -0.002 | 0.015 | -0.048 | 0.026 | -0.013 | 0.015 |
| rs11274218 | 2 | 215035001 | C | T | 0.004 | 0.002 | 8.24E-02 | -0.014 | 0.002 | 2.50E-09 | PC | 0.014 | 0.006 | 0.008 | 0.008 | 0.030 | 0.018 | 0.003 | 0.016 | 0.006 | 0.027 | 0.021 | 0.016 |
| rs10208088 | 2 | 221055873 | C | T | 0.000 | 0.003 | 9.66E-01 | 0.013 | 0.002 | 4.40E-09 | PC | 0.001 | 0.006 | 0.002 | 0.008 | -0.021 | 0.018 | 0.012 | 0.015 | 0.000 | 0.026 | 0.006 | 0.015 |
| rs12612009 | 2 | 228982603 | T | G | -0.014 | 0.002 | 3.78E-09 | 0.003 | 0.002 | 2.80E-01 | BMI | 0.001 | 0.007 | 0.002 | 0.009 | -0.031 | 0.019 | 0.007 | 0.017 | -0.070 | 0.028 | 0.010 | 0.017 |
| rs6720868 | 2 | 230663576 | T | C | 0.017 | 0.002 | 4.65E-12 | 0.000 | 0.002 | 9.40E-01 | BMI | -0.012 | 0.006 | -0.002 | 0.008 | -0.019 | 0.019 | -0.015 | 0.016 | -0.012 | 0.027 | -0.009 | 0.016 |
| rs1801282 | 3 | 12393125 | C | G | -0.018 | 0.003 | 4.72E-08 | 0.000 | 0.003 | 9.80E-01 | BMI | 0.016 | 0.009 | 0.000 | 0.012 | 0.076 | 0.027 | 0.030 | 0.023 | -0.052 | 0.038 | 0.012 | 0.023 |
| rs2470520 | 3 | 15762920 | T | C | -0.013 | 0.002 | 4.16E-08 | -0.002 | 0.002 | 3.50E-01 | BMI | -0.003 | 0.006 | -0.007 | 0.008 | -0.033 | 0.018 | 0.012 | 0.015 | -0.012 | 0.026 | 0.015 | 0.015 |
| rs4858223 | 3 | 20581149 | T | C | -0.016 | 0.003 | 8.26E-11 | 0.000 | 0.002 | 9.50E-01 | BMI | 0.005 | 0.007 | 0.011 | 0.009 | 0.022 | 0.019 | 0.014 | 0.016 | -0.037 | 0.028 | 0.025 | 0.017 |
| rs13062093 | 3 | 35667057 | T | G | -0.014 | 0.002 | 8.00E-09 | 0.000 | 0.002 | 8.40E-01 | BMI | -0.003 | 0.006 | -0.012 | 0.008 | -0.015 | 0.018 | -0.005 | 0.016 | -0.021 | 0.026 | -0.001 | 0.016 |
| rs11708955 | 3 | 49540114 | T | C | 0.015 | 0.003 | 2.36E-09 | -0.016 | 0.002 | 4.20E-11 | PC | 0.023 | 0.007 | 0.018 | 0.009 | 0.016 | 0.019 | 0.025 | 0.016 | 0.062 | 0.028 | 0.053 | 0.016 |
| rs6774533 | 3 | 62471086 | C | T | -0.016 | 0.003 | 2.30E-08 | -0.015 | 0.002 | 1.20E-09 | PC | -0.012 | 0.007 | -0.022 | 0.009 | -0.004 | 0.021 | -0.004 | 0.018 | -0.047 | 0.031 | 0.018 | 0.018 |
| rs7647242 | 3 | 82642734 | T | C | 0.015 | 0.002 | 3.92E-10 | -0.003 | 0.002 | 1.80E-01 | BMI | 0.003 | 0.006 | 0.005 | 0.008 | -0.008 | 0.018 | 0.015 | 0.016 | 0.008 | 0.027 | 0.008 | 0.016 |
| rs13068138 | 3 | 85809425 | T | C | 0.023 | 0.003 | 3.60E-17 | 0.005 | 0.003 | 7.70E-02 | BMI | -0.010 | 0.008 | -0.018 | 0.010 | 0.002 | 0.022 | 0.019 | 0.019 | -0.024 | 0.032 | -0.010 | 0.019 |
| rs1949197 | 3 | 86273850 | A | G | 0.014 | 0.002 | 3.04E-09 | 0.003 | 0.002 | 1.40E-01 | BMI | -0.008 | 0.006 | -0.008 | 0.008 | 0.026 | 0.018 | -0.009 | 0.015 | -0.070 | 0.026 | -0.024 | 0.016 |
| rs1492014 | 3 | 94071481 | T | C | -0.015 | 0.002 | 2.84E-10 | 0.002 | 0.002 | 2.80E-01 | BMI | 0.006 | 0.006 | 0.002 | 0.008 | 0.003 | 0.018 | -0.001 | 0.015 | 0.001 | 0.026 | 0.019 | 0.015 |
| rs1436348 | 3 | 104612668 | A | G | -0.013 | 0.002 | 6.31E-09 | 0.004 | 0.002 | 6.90E-02 | BMI | 0.000 | 0.006 | -0.001 | 0.008 | 0.003 | 0.018 | -0.002 | 0.015 | -0.026 | 0.026 | 0.014 | 0.016 |
| rs1289736 | 3 | 107950892 | T | C | 0.014 | 0.002 | 1.43E-09 | -0.004 | 0.002 | 7.80E-02 | BMI | -0.005 | 0.006 | -0.001 | 0.008 | 0.029 | 0.018 | 0.007 | 0.015 | -0.042 | 0.026 | -0.015 | 0.015 |
| rs17619973 | 3 | 114417675 | A | G | 0.024 | 0.004 | 1.29E-08 | 0.000 | 0.004 | 9.20E-01 | BMI | 0.018 | 0.012 | 0.020 | 0.016 | 0.025 | 0.036 | 0.009 | 0.031 | 0.008 | 0.052 | 0.022 | 0.031 |
| rs78082503 | 3 | 116556913 | G | C | -0.001 | 0.003 | 7.24E-01 | -0.015 | 0.003 | 1.10E-08 | PC | 0.004 | 0.008 | -0.001 | 0.010 | 0.022 | 0.022 | 0.013 | 0.019 | 0.054 | 0.032 | 0.012 | 0.019 |
| rs7428670 | 3 | 131579810 | C | G | 0.020 | 0.003 | 3.09E-15 | -0.004 | 0.002 | 1.30E-01 | BMI | -0.004 | 0.007 | -0.012 | 0.009 | -0.010 | 0.020 | -0.005 | 0.017 | -0.017 | 0.029 | 0.017 | 0.017 |
| rs7621025 | 3 | 136272246 | T | C | -0.020 | 0.003 | 1.62E-15 | -0.008 | 0.003 | 1.10E-03 | BMI | 0.013 | 0.007 | -0.001 | 0.009 | 0.015 | 0.020 | -0.012 | 0.017 | 0.042 | 0.029 | 0.032 | 0.017 |
| rs16851483 | 3 | 141275436 | T | G | 0.036 | 0.005 | 2.28E-15 | 0.006 | 0.004 | 1.80E-01 | BMI | 0.006 | 0.012 | 0.011 | 0.016 | 0.039 | 0.035 | -0.024 | 0.031 | -0.085 | 0.054 | 0.053 | 0.030 |
| rs355754 | 3 | 154024165 | T | C | -0.014 | 0.002 | 4.26E-10 | 0.004 | 0.002 | 1.20E-01 | BMI | 0.000 | 0.006 | 0.006 | 0.008 | -0.031 | 0.018 | 0.005 | 0.016 | -0.031 | 0.026 | 0.006 | 0.016 |
| rs247975 | 3 | 173107443 | T | C | -0.018 | 0.002 | 3.62E-15 | 0.006 | 0.002 | 5.60E-03 | BMI | 0.002 | 0.007 | -0.003 | 0.009 | 0.012 | 0.019 | 0.012 | 0.016 | 0.027 | 0.028 | 0.000 | 0.017 |
| rs10513801 | 3 | 185822353 | T | G | 0.035 | 0.003 | 1.26E-27 | -0.004 | 0.003 | 2.50E-01 | BMI | -0.009 | 0.009 | -0.017 | 0.012 | -0.001 | 0.027 | 0.018 | 0.023 | 0.007 | 0.039 | -0.020 | 0.023 |
| rs4677812 | 3 | 194863794 | A | C | -0.014 | 0.003 | 9.38E-09 | 0.000 | 0.002 | 9.90E-01 | BMI | -0.002 | 0.007 | -0.002 | 0.009 | 0.002 | 0.020 | -0.014 | 0.017 | 0.023 | 0.029 | -0.013 | 0.017 |
| rs994596 | 4 | 18459828 | T | C | 0.015 | 0.003 | 1.02E-09 | 0.002 | 0.002 | 4.40E-01 | BMI | 0.002 | 0.006 | -0.001 | 0.009 | -0.004 | 0.019 | 0.035 | 0.016 | -0.005 | 0.027 | -0.017 | 0.016 |
| rs34811474 | 4 | 25408838 | A | G | -0.032 | 0.003 | 5.19E-24 | 0.003 | 0.003 | 2.80E-01 | BMI | -0.006 | 0.008 | 0.002 | 0.011 | -0.015 | 0.025 | -0.012 | 0.021 | -0.045 | 0.036 | -0.005 | 0.021 |
| rs73213484 | 4 | 28489339 | A | T | 0.025 | 0.004 | 2.28E-11 | 0.003 | 0.003 | 3.20E-01 | BMI | 0.003 | 0.008 | 0.002 | 0.011 | 0.024 | 0.025 | 0.019 | 0.021 | 0.053 | 0.036 | -0.026 | 0.021 |
| rs337637 | 4 | 38604470 | A | G | -0.013 | 0.002 | 3.04E-08 | 0.005 | 0.002 | 1.90E-02 | BMI | -0.001 | 0.006 | -0.009 | 0.008 | 0.023 | 0.018 | -0.006 | 0.016 | 0.034 | 0.027 | 0.030 | 0.016 |
| rs6857629 | 4 | 39684977 | G | A | -0.004 | 0.003 | 1.99E-01 | 0.015 | 0.003 | 1.90E-09 | PC | 0.009 | 0.007 | 0.014 | 0.009 | 0.003 | 0.021 | 0.023 | 0.018 | 0.005 | 0.030 | 0.015 | 0.018 |
| rs2271046 | 4 | 52752812 | A | T | -0.014 | 0.003 | 2.38E-08 | 0.004 | 0.002 | 6.50E-02 | BMI | -0.016 | 0.007 | -0.017 | 0.009 | -0.026 | 0.020 | -0.034 | 0.017 | -0.035 | 0.028 | -0.004 | 0.017 |
| rs11727273 | 4 | 55509748 | T | C | -0.017 | 0.003 | 1.28E-10 | -0.002 | 0.002 | 3.00E-01 | BMI | 0.002 | 0.006 | 0.016 | 0.008 | -0.010 | 0.018 | -0.009 | 0.016 | -0.012 | 0.027 | 0.004 | 0.016 |
| rs11945861 | 4 | 65700865 | A | G | -0.015 | 0.003 | 3.13E-08 | 0.004 | 0.003 | 9.50E-02 | BMI | -0.008 | 0.007 | -0.012 | 0.009 | -0.010 | 0.021 | -0.023 | 0.018 | 0.038 | 0.030 | -0.006 | 0.018 |
| rs10002111 | 4 | 67815504 | A | G | 0.017 | 0.003 | 9.26E-10 | -0.001 | 0.003 | 7.50E-01 | BMI | -0.016 | 0.007 | -0.013 | 0.009 | 0.010 | 0.021 | -0.006 | 0.018 | 0.010 | 0.031 | -0.028 | 0.018 |
| rs4148155 | 4 | 89054667 | A | G | 0.020 | 0.004 | 6.20E-09 | -0.009 | 0.003 | 6.90E-03 | BMI | 0.043 | 0.010 | 0.019 | 0.013 | 0.023 | 0.029 | 0.060 | 0.025 | 0.011 | 0.042 | 0.078 | 0.026 |
| rs12645001 | 4 | 96018337 | A | G | -0.014 | 0.002 | 4.11E-09 | 0.002 | 0.002 | 3.30E-01 | BMI | -0.009 | 0.006 | -0.005 | 0.008 | -0.034 | 0.019 | 0.005 | 0.016 | -0.035 | 0.027 | 0.003 | 0.016 |
| rs13107325 | 4 | 103188709 | T | C | 0.045 | 0.004 | 9.33E-25 | 0.001 | 0.004 | 7.60E-01 | BMI | 0.019 | 0.012 | 0.021 | 0.016 | 0.051 | 0.034 | 0.007 | 0.030 | -0.051 | 0.051 | 0.025 | 0.030 |
| rs4834272 | 4 | 113313986 | T | C | -0.014 | 0.002 | 8.24E-10 | 0.000 | 0.002 | 8.80E-01 | BMI | -0.002 | 0.007 | -0.002 | 0.009 | -0.004 | 0.019 | -0.006 | 0.017 | -0.029 | 0.028 | -0.009 | 0.017 |
| rs1403846 | 4 | 119101723 | T | C | 0.016 | 0.003 | 1.62E-08 | 0.000 | 0.003 | 9.80E-01 | BMI | 0.007 | 0.008 | 0.010 | 0.010 | -0.023 | 0.023 | 0.038 | 0.020 | 0.032 | 0.034 | 0.006 | 0.020 |
| rs4864201 | 4 | 130731284 | T | C | 0.013 | 0.002 | 6.34E-09 | 0.000 | 0.002 | 9.30E-01 | BMI | 0.003 | 0.006 | 0.003 | 0.008 | -0.004 | 0.018 | 0.013 | 0.016 | 0.014 | 0.027 | -0.009 | 0.016 |
| rs57800857 | 4 | 140863365 | A | C | 0.019 | 0.003 | 1.07E-11 | 0.006 | 0.002 | 1.40E-02 | BMI | 0.011 | 0.006 | 0.007 | 0.008 | 0.019 | 0.019 | 0.011 | 0.016 | 0.007 | 0.027 | 0.050 | 0.016 |
| rs35107973 | 4 | 143125948 | T | C | 0.023 | 0.004 | 7.66E-09 | -0.004 | 0.003 | 2.70E-01 | BMI | 0.012 | 0.010 | 0.010 | 0.013 | 0.013 | 0.029 | 0.007 | 0.025 | 0.028 | 0.042 | -0.003 | 0.025 |
| rs2068625 | 4 | 159856739 | T | C | 0.010 | 0.003 | 3.92E-05 | -0.016 | 0.002 | 4.00E-11 | PC | -0.005 | 0.007 | -0.013 | 0.009 | -0.022 | 0.020 | -0.002 | 0.017 | 0.041 | 0.028 | -0.014 | 0.017 |
| rs13110266 | 4 | 162129844 | A | G | -0.014 | 0.002 | 1.55E-10 | -0.001 | 0.002 | 7.30E-01 | BMI | -0.002 | 0.006 | 0.005 | 0.008 | 0.000 | 0.018 | 0.012 | 0.015 | -0.013 | 0.026 | -0.010 | 0.016 |
| rs1491333 | 4 | 172186288 | T | G | -0.015 | 0.003 | 9.85E-09 | 0.002 | 0.003 | 4.60E-01 | BMI | -0.001 | 0.007 | 0.001 | 0.009 | -0.015 | 0.021 | 0.009 | 0.018 | -0.013 | 0.030 | -0.001 | 0.018 |
| rs2220599 | 5 | 7378854 | C | G | 0.006 | 0.002 | 8.26E-03 | -0.016 | 0.002 | 2.40E-12 | PC | 0.008 | 0.007 | 0.014 | 0.009 | 0.018 | 0.019 | 0.005 | 0.016 | -0.005 | 0.027 | -0.015 | 0.016 |
| rs6890310 | 5 | 27193573 | A | G | -0.015 | 0.003 | 1.38E-09 | 0.001 | 0.002 | 7.10E-01 | BMI | 0.008 | 0.007 | 0.013 | 0.009 | 0.022 | 0.020 | -0.018 | 0.017 | -0.010 | 0.030 | 0.010 | 0.018 |
| rs782971 | 5 | 43124688 | A | G | -0.017 | 0.003 | 4.89E-12 | -0.002 | 0.003 | 4.70E-01 | BMI | -0.014 | 0.007 | -0.013 | 0.009 | 0.013 | 0.021 | -0.016 | 0.018 | 0.004 | 0.031 | -0.009 | 0.018 |
| rs1812587 | 5 | 62991802 | T | G | 0.015 | 0.003 | 6.95E-09 | 0.002 | 0.002 | 4.60E-01 | BMI | 0.006 | 0.006 | 0.012 | 0.008 | 0.004 | 0.018 | -0.003 | 0.015 | 0.040 | 0.026 | -0.005 | 0.015 |
| rs10471636 | 5 | 63979856 | A | G | -0.017 | 0.002 | 4.66E-13 | -0.004 | 0.002 | 8.40E-02 | BMI | 0.000 | 0.006 | -0.004 | 0.008 | -0.036 | 0.019 | 0.004 | 0.016 | 0.009 | 0.027 | -0.015 | 0.016 |
| rs2112347 | 5 | 75015242 | T | G | 0.031 | 0.002 | 2.13E-42 | -0.005 | 0.002 | 4.70E-02 | BMI | -0.020 | 0.006 | -0.017 | 0.008 | 0.019 | 0.018 | -0.023 | 0.015 | -0.065 | 0.026 | -0.030 | 0.016 |
| rs11749912 | 5 | 88065628 | A | G | 0.006 | 0.003 | 1.79E-02 | 0.014 | 0.002 | 4.90E-10 | PC | 0.002 | 0.006 | -0.001 | 0.008 | 0.010 | 0.018 | 0.019 | 0.015 | -0.015 | 0.026 | 0.006 | 0.015 |
| rs158186 | 5 | 107440856 | A | G | -0.029 | 0.003 | 3.23E-20 | -0.003 | 0.003 | 3.90E-01 | BMI | -0.005 | 0.008 | -0.017 | 0.011 | 0.047 | 0.023 | -0.024 | 0.020 | 0.052 | 0.033 | -0.019 | 0.020 |
| rs1469249 | 5 | 113837198 | G | A | -0.012 | 0.003 | 3.12E-05 | 0.016 | 0.003 | 9.50E-09 | PC | -0.011 | 0.007 | -0.008 | 0.010 | -0.023 | 0.021 | -0.006 | 0.018 | -0.013 | 0.031 | -0.019 | 0.019 |
| rs162894 | 5 | 131611872 | T | G | 0.003 | 0.002 | 2.43E-01 | 0.013 | 0.002 | 1.10E-08 | PC | -0.024 | 0.006 | -0.034 | 0.008 | -0.048 | 0.019 | -0.031 | 0.016 | 0.013 | 0.027 | -0.030 | 0.016 |
| rs56158102 | 5 | 144027803 | C | T | 0.000 | 0.002 | 9.11E-01 | 0.013 | 0.002 | 3.10E-08 | PC | -0.015 | 0.006 | -0.012 | 0.008 | -0.042 | 0.019 | -0.016 | 0.016 | -0.054 | 0.027 | -0.015 | 0.016 |
| rs4569924 | 5 | 153540025 | T | C | 0.016 | 0.002 | 7.44E-13 | -0.004 | 0.002 | 1.00E-01 | BMI | -0.010 | 0.006 | -0.010 | 0.008 | -0.014 | 0.018 | -0.013 | 0.015 | -0.025 | 0.026 | -0.030 | 0.015 |
| rs6556810 | 5 | 164585949 | T | C | -0.012 | 0.002 | 4.98E-08 | -0.002 | 0.002 | 3.20E-01 | BMI | -0.008 | 0.006 | -0.002 | 0.008 | -0.004 | 0.018 | -0.030 | 0.015 | 0.021 | 0.026 | -0.029 | 0.016 |
| rs2053682 | 5 | 170599327 | A | C | 0.018 | 0.003 | 1.89E-13 | 0.004 | 0.002 | 6.30E-02 | BMI | -0.001 | 0.007 | 0.007 | 0.009 | -0.011 | 0.019 | 0.012 | 0.016 | 0.001 | 0.028 | -0.012 | 0.017 |
| rs2228213 | 6 | 12124855 | A | G | -0.015 | 0.002 | 9.21E-11 | -0.003 | 0.002 | 2.70E-01 | BMI | 0.001 | 0.006 | 0.006 | 0.008 | -0.004 | 0.018 | -0.013 | 0.016 | -0.037 | 0.027 | 0.025 | 0.016 |
| rs9367369 | 6 | 13189941 | A | G | 0.017 | 0.003 | 2.32E-09 | 0.003 | 0.002 | 2.00E-01 | BMI | 0.001 | 0.007 | 0.002 | 0.009 | -0.026 | 0.019 | -0.008 | 0.016 | -0.009 | 0.028 | 0.041 | 0.017 |
| rs9477970 | 6 | 19082663 | T | A | -0.013 | 0.003 | 7.03E-06 | -0.016 | 0.003 | 1.00E-08 | PC | -0.002 | 0.008 | 0.001 | 0.010 | -0.001 | 0.023 | 0.017 | 0.020 | 0.007 | 0.033 | -0.002 | 0.020 |
| rs2178899 | 6 | 31606756 | A | T | 0.025 | 0.003 | 7.16E-14 | 0.001 | 0.003 | 7.20E-01 | BMI | -0.001 | 0.009 | -0.002 | 0.012 | -0.001 | 0.028 | -0.010 | 0.023 | 0.008 | 0.040 | -0.002 | 0.024 |
| rs11751591 | 6 | 33794215 | A | G | -0.019 | 0.003 | 1.16E-08 | -0.002 | 0.003 | 4.60E-01 | BMI | 0.002 | 0.009 | -0.004 | 0.011 | 0.011 | 0.025 | -0.040 | 0.022 | 0.006 | 0.036 | 0.001 | 0.022 |
| rs2814992 | 6 | 34617144 | A | G | -0.029 | 0.002 | 1.25E-35 | -0.005 | 0.002 | 3.90E-02 | BMI | 0.004 | 0.006 | 0.002 | 0.008 | 0.018 | 0.018 | 0.005 | 0.016 | -0.004 | 0.027 | 0.006 | 0.016 |
| rs1579557 | 6 | 40371918 | T | C | 0.021 | 0.003 | 3.09E-17 | -0.001 | 0.002 | 8.30E-01 | BMI | -0.003 | 0.007 | 0.003 | 0.009 | -0.003 | 0.021 | -0.017 | 0.018 | -0.030 | 0.030 | 0.010 | 0.018 |
| rs943005 | 6 | 50865820 | T | C | 0.040 | 0.003 | 1.99E-43 | 0.003 | 0.003 | 3.30E-01 | BMI | 0.011 | 0.008 | 0.020 | 0.010 | 0.024 | 0.023 | 0.005 | 0.020 | -0.036 | 0.034 | -0.015 | 0.020 |
| rs2465043 | 6 | 51180765 | A | G | -0.014 | 0.002 | 3.22E-09 | 0.001 | 0.002 | 6.50E-01 | BMI | 0.007 | 0.006 | -0.007 | 0.008 | 0.045 | 0.018 | 0.025 | 0.016 | 0.011 | 0.027 | 0.007 | 0.016 |
| rs62412826 | 6 | 61881167 | T | C | 0.033 | 0.006 | 7.98E-09 | -0.003 | 0.005 | 5.40E-01 | BMI | 0.027 | 0.014 | 0.028 | 0.018 | 0.099 | 0.038 | 0.013 | 0.034 | -0.035 | 0.058 | 0.001 | 0.034 |
| rs209416 | 6 | 83534729 | A | T | 0.017 | 0.002 | 8.71E-12 | -0.001 | 0.002 | 6.10E-01 | BMI | -0.013 | 0.006 | 0.001 | 0.009 | -0.067 | 0.019 | 0.004 | 0.016 | -0.010 | 0.028 | -0.006 | 0.016 |
| rs9372625 | 6 | 98344031 | G | A | 0.013 | 0.002 | 1.20E-07 | -0.018 | 0.002 | 5.70E-16 | PC | 0.005 | 0.006 | 0.009 | 0.008 | 0.003 | 0.019 | 0.003 | 0.016 | -0.008 | 0.027 | 0.006 | 0.016 |
| rs768023 | 6 | 108876002 | A | G | 0.019 | 0.002 | 1.38E-16 | 0.001 | 0.002 | 6.60E-01 | BMI | 0.011 | 0.006 | 0.018 | 0.008 | 0.005 | 0.018 | 0.004 | 0.015 | 0.029 | 0.026 | -0.004 | 0.016 |
| rs2357760 | 6 | 120213880 | A | G | 0.016 | 0.002 | 1.09E-11 | 0.004 | 0.002 | 9.60E-02 | BMI | -0.007 | 0.006 | 0.003 | 0.008 | -0.005 | 0.019 | 0.016 | 0.016 | -0.029 | 0.027 | -0.016 | 0.016 |
| rs6935828 | 6 | 140811367 | C | T | -0.002 | 0.002 | 3.92E-01 | -0.012 | 0.002 | 4.30E-08 | PC | -0.004 | 0.006 | -0.003 | 0.008 | 0.018 | 0.018 | 0.007 | 0.015 | -0.028 | 0.026 | 0.001 | 0.015 |
| rs11754747 | 6 | 141494602 | T | C | 0.015 | 0.003 | 1.27E-08 | -0.004 | 0.003 | 1.40E-01 | BMI | 0.004 | 0.007 | 0.003 | 0.010 | 0.003 | 0.021 | -0.007 | 0.018 | 0.064 | 0.031 | -0.015 | 0.019 |
| rs2185027 | 6 | 153381622 | A | C | -0.015 | 0.002 | 1.11E-10 | -0.002 | 0.002 | 5.20E-01 | BMI | 0.002 | 0.007 | 0.007 | 0.009 | -0.004 | 0.019 | -0.023 | 0.016 | -0.030 | 0.028 | 0.020 | 0.017 |
| rs12207125 | 6 | 154301575 | A | G | 0.018 | 0.003 | 7.67E-09 | 0.006 | 0.003 | 3.50E-02 | BMI | -0.010 | 0.007 | -0.010 | 0.010 | -0.005 | 0.022 | -0.040 | 0.018 | 0.045 | 0.032 | -0.024 | 0.019 |
| rs13213408 | 6 | 162998049 | A | T | 0.020 | 0.003 | 4.33E-09 | -0.004 | 0.003 | 1.80E-01 | BMI | 0.008 | 0.009 | -0.005 | 0.012 | -0.028 | 0.026 | -0.004 | 0.022 | 0.055 | 0.039 | 0.020 | 0.023 |
| rs6973700 | 7 | 1856669 | A | G | 0.019 | 0.003 | 1.77E-09 | 0.004 | 0.003 | 2.00E-01 | BMI | 0.007 | 0.007 | 0.002 | 0.010 | -0.010 | 0.021 | -0.003 | 0.018 | 0.022 | 0.031 | 0.028 | 0.019 |
| rs836525 | 7 | 6465909 | T | C | 0.018 | 0.003 | 9.75E-09 | 0.004 | 0.003 | 2.20E-01 | BMI | -0.014 | 0.008 | -0.018 | 0.011 | -0.021 | 0.024 | -0.027 | 0.021 | -0.066 | 0.036 | 0.009 | 0.021 |
| rs4307239 | 7 | 24354300 | A | G | -0.014 | 0.002 | 1.31E-09 | 0.003 | 0.002 | 1.40E-01 | BMI | 0.012 | 0.006 | 0.001 | 0.008 | 0.026 | 0.018 | 0.025 | 0.016 | 0.026 | 0.026 | 0.027 | 0.016 |
| rs11971098 | 7 | 26699061 | A | G | -0.024 | 0.004 | 6.49E-09 | -0.009 | 0.004 | 2.10E-02 | BMI | -0.015 | 0.011 | -0.016 | 0.014 | -0.026 | 0.031 | -0.032 | 0.027 | 0.042 | 0.046 | 0.041 | 0.028 |
| rs10499694 | 7 | 50614173 | A | G | 0.014 | 0.002 | 3.99E-10 | 0.001 | 0.002 | 5.50E-01 | BMI | 0.003 | 0.006 | 0.003 | 0.008 | 0.005 | 0.018 | 0.010 | 0.015 | -0.001 | 0.026 | 0.012 | 0.015 |
| rs55772938 | 7 | 70025113 | A | G | -0.008 | 0.003 | 4.34E-03 | -0.015 | 0.002 | 3.90E-10 | PC | 0.003 | 0.007 | -0.003 | 0.009 | 0.020 | 0.019 | -0.024 | 0.016 | 0.042 | 0.028 | 0.015 | 0.017 |
| rs2267812 | 7 | 74138121 | A | C | -0.017 | 0.003 | 1.74E-09 | -0.001 | 0.003 | 6.90E-01 | BMI | -0.035 | 0.007 | -0.030 | 0.010 | -0.041 | 0.021 | -0.020 | 0.018 | -0.082 | 0.031 | -0.039 | 0.019 |
| rs7805441 | 7 | 78121458 | T | C | 0.015 | 0.002 | 2.01E-10 | 0.000 | 0.002 | 9.50E-01 | BMI | -0.003 | 0.006 | 0.002 | 0.008 | -0.010 | 0.018 | -0.002 | 0.015 | -0.041 | 0.026 | 0.001 | 0.015 |
| rs2528531 | 7 | 93236510 | A | C | -0.015 | 0.002 | 1.35E-09 | 0.000 | 0.002 | 8.90E-01 | BMI | 0.007 | 0.006 | -0.001 | 0.008 | -0.004 | 0.018 | 0.020 | 0.016 | 0.002 | 0.027 | 0.022 | 0.016 |
| rs10271582 | 7 | 99121950 | A | G | 0.019 | 0.003 | 8.41E-09 | 0.012 | 0.003 | 7.40E-05 | BMI | 0.010 | 0.008 | 0.002 | 0.011 | 0.018 | 0.024 | 0.004 | 0.021 | 0.036 | 0.035 | 0.019 | 0.021 |
| rs2299383 | 7 | 103418846 | T | C | 0.018 | 0.002 | 2.73E-16 | 0.007 | 0.002 | 3.00E-03 | BMI | -0.013 | 0.006 | -0.005 | 0.008 | -0.034 | 0.018 | -0.017 | 0.015 | -0.003 | 0.026 | -0.025 | 0.016 |
| rs7788008 | 7 | 112972483 | A | G | -0.017 | 0.002 | 1.09E-13 | 0.004 | 0.002 | 5.20E-02 | BMI | 0.000 | 0.006 | -0.002 | 0.008 | 0.027 | 0.018 | -0.013 | 0.015 | 0.019 | 0.026 | -0.005 | 0.015 |
| rs2045293 | 7 | 114350102 | T | C | 0.019 | 0.002 | 4.06E-16 | -0.005 | 0.002 | 2.30E-02 | BMI | -0.019 | 0.006 | -0.021 | 0.008 | -0.019 | 0.018 | -0.010 | 0.015 | -0.012 | 0.026 | 0.004 | 0.016 |
| rs12706626 | 7 | 124531370 | G | A | 0.001 | 0.003 | 8.39E-01 | -0.013 | 0.002 | 1.40E-08 | PC | -0.009 | 0.006 | -0.016 | 0.008 | 0.012 | 0.018 | -0.015 | 0.015 | 0.006 | 0.026 | 0.004 | 0.016 |
| rs3944151 | 7 | 126970005 | A | G | -0.001 | 0.003 | 7.41E-01 | 0.014 | 0.002 | 3.10E-08 | PC | 0.016 | 0.007 | 0.013 | 0.009 | 0.003 | 0.020 | 0.015 | 0.017 | 0.037 | 0.028 | 0.010 | 0.017 |
| rs2345941 | 7 | 133337635 | A | G | -0.001 | 0.002 | 8.00E-01 | 0.015 | 0.002 | 4.00E-11 | PC | 0.007 | 0.006 | 0.002 | 0.008 | 0.036 | 0.018 | 0.007 | 0.015 | 0.019 | 0.026 | -0.002 | 0.015 |
| rs2968864 | 7 | 150622162 | T | C | 0.016 | 0.003 | 9.35E-10 | 0.003 | 0.003 | 2.50E-01 | BMI | -0.013 | 0.007 | -0.022 | 0.009 | -0.017 | 0.021 | -0.003 | 0.018 | -0.009 | 0.030 | -0.004 | 0.018 |
| rs4240673 | 8 | 10787612 | T | C | 0.018 | 0.002 | 1.03E-15 | 0.002 | 0.002 | 4.40E-01 | BMI | -0.007 | 0.007 | -0.003 | 0.010 | -0.006 | 0.022 | 0.012 | 0.017 | -0.021 | 0.029 | 0.008 | 0.018 |
| rs2959592 | 8 | 17002725 | T | C | 0.017 | 0.003 | 6.26E-10 | -0.001 | 0.003 | 7.00E-01 | BMI | 0.002 | 0.008 | 0.001 | 0.010 | 0.033 | 0.023 | -0.001 | 0.020 | -0.009 | 0.033 | 0.008 | 0.020 |
| rs1362910 | 8 | 30856464 | A | G | 0.013 | 0.002 | 3.03E-09 | -0.001 | 0.002 | 7.10E-01 | BMI | 0.001 | 0.006 | 0.000 | 0.008 | 0.017 | 0.018 | -0.002 | 0.015 | -0.028 | 0.026 | -0.003 | 0.016 |
| rs4320543 | 8 | 34422967 | A | G | -0.013 | 0.002 | 2.95E-08 | 0.004 | 0.002 | 1.20E-01 | BMI | 0.004 | 0.006 | 0.002 | 0.008 | 0.002 | 0.019 | 0.026 | 0.016 | 0.002 | 0.027 | 0.006 | 0.016 |
| rs12681792 | 8 | 62054463 | A | C | 0.018 | 0.003 | 2.61E-10 | 0.005 | 0.003 | 5.80E-02 | BMI | 0.010 | 0.008 | 0.016 | 0.011 | 0.020 | 0.023 | 0.025 | 0.020 | -0.045 | 0.035 | -0.005 | 0.020 |
| rs7827848 | 8 | 64790774 | A | T | 0.015 | 0.002 | 1.88E-11 | 0.001 | 0.002 | 5.40E-01 | BMI | 0.003 | 0.006 | -0.005 | 0.008 | 0.002 | 0.018 | 0.019 | 0.016 | -0.001 | 0.027 | -0.011 | 0.016 |
| rs1808629 | 8 | 73435964 | A | G | -0.023 | 0.003 | 5.76E-16 | -0.002 | 0.002 | 3.30E-01 | BMI | -0.006 | 0.007 | -0.011 | 0.009 | 0.006 | 0.019 | 0.012 | 0.017 | 0.002 | 0.028 | -0.012 | 0.017 |
| rs17405819 | 8 | 76806584 | T | C | 0.022 | 0.002 | 7.81E-20 | 0.002 | 0.002 | 4.60E-01 | BMI | -0.013 | 0.007 | -0.019 | 0.009 | 0.015 | 0.019 | -0.011 | 0.016 | -0.030 | 0.027 | -0.002 | 0.016 |
| rs12680842 | 8 | 95582606 | A | G | 0.013 | 0.002 | 3.36E-08 | 0.000 | 0.002 | 8.60E-01 | BMI | -0.002 | 0.006 | -0.003 | 0.008 | 0.019 | 0.019 | 0.012 | 0.016 | -0.013 | 0.027 | 0.001 | 0.016 |
| rs2721965 | 8 | 116662038 | A | C | 0.019 | 0.002 | 2.10E-14 | 0.002 | 0.002 | 3.50E-01 | BMI | 0.020 | 0.006 | 0.026 | 0.008 | -0.008 | 0.019 | 0.038 | 0.016 | -0.024 | 0.027 | -0.008 | 0.016 |
| rs13262595 | 8 | 143316970 | A | G | -0.006 | 0.002 | 1.61E-02 | -0.016 | 0.002 | 1.20E-12 | PC | -0.001 | 0.006 | 0.005 | 0.008 | -0.016 | 0.018 | 0.013 | 0.015 | 0.043 | 0.026 | -0.005 | 0.015 |
| rs10124645 | 9 | 11321119 | A | G | 0.014 | 0.002 | 4.98E-09 | 0.002 | 0.002 | 4.10E-01 | BMI | -0.006 | 0.006 | -0.012 | 0.008 | -0.024 | 0.018 | 0.017 | 0.016 | 0.032 | 0.027 | 0.008 | 0.016 |
| rs10962550 | 9 | 16720329 | C | G | 0.019 | 0.003 | 1.70E-10 | -0.002 | 0.003 | 4.00E-01 | BMI | 0.013 | 0.008 | 0.016 | 0.011 | -0.013 | 0.024 | 0.014 | 0.020 | -0.024 | 0.035 | 0.041 | 0.020 |
| rs4977839 | 9 | 23355310 | G | A | -0.011 | 0.003 | 6.06E-05 | -0.020 | 0.002 | 4.70E-19 | PC | -0.006 | 0.006 | -0.001 | 0.008 | -0.037 | 0.019 | -0.006 | 0.016 | 0.019 | 0.027 | -0.022 | 0.016 |
| rs16912921 | 9 | 28413461 | A | C | 0.025 | 0.002 | 1.56E-26 | 0.005 | 0.002 | 4.30E-02 | BMI | -0.001 | 0.006 | 0.011 | 0.008 | -0.015 | 0.019 | 0.003 | 0.016 | -0.002 | 0.027 | -0.015 | 0.016 |
| rs973345 | 9 | 29713169 | T | C | 0.014 | 0.002 | 1.09E-09 | 0.003 | 0.002 | 2.00E-01 | BMI | 0.008 | 0.006 | 0.022 | 0.008 | 0.004 | 0.018 | -0.016 | 0.015 | 0.025 | 0.026 | 0.021 | 0.015 |
| rs563132 | 9 | 37245476 | A | T | 0.018 | 0.002 | 1.96E-14 | 0.000 | 0.002 | 9.80E-01 | BMI | -0.014 | 0.006 | -0.021 | 0.008 | -0.006 | 0.018 | -0.030 | 0.015 | -0.014 | 0.026 | -0.008 | 0.016 |
| rs10116186 | 9 | 80532374 | A | G | -0.015 | 0.003 | 4.10E-08 | 0.000 | 0.002 | 9.90E-01 | BMI | 0.015 | 0.006 | 0.017 | 0.008 | 0.025 | 0.018 | 0.030 | 0.015 | 0.029 | 0.026 | 0.017 | 0.015 |
| rs10992867 | 9 | 96461013 | A | G | 0.019 | 0.003 | 8.89E-13 | -0.001 | 0.003 | 7.00E-01 | BMI | -0.003 | 0.007 | 0.004 | 0.009 | -0.017 | 0.020 | -0.027 | 0.017 | -0.002 | 0.029 | -0.013 | 0.017 |
| rs113851275 | 9 | 98297220 | G | A | 0.005 | 0.004 | 2.79E-01 | -0.021 | 0.004 | 3.20E-09 | PC | -0.028 | 0.010 | -0.044 | 0.012 | -0.025 | 0.028 | -0.009 | 0.024 | 0.010 | 0.041 | 0.008 | 0.024 |
| rs10118701 | 9 | 103061366 | A | G | -0.018 | 0.002 | 2.20E-13 | 0.002 | 0.002 | 3.00E-01 | BMI | 0.002 | 0.006 | 0.007 | 0.008 | -0.012 | 0.019 | -0.035 | 0.016 | 0.044 | 0.027 | -0.003 | 0.016 |
| rs7024334 | 9 | 109072075 | T | G | 0.016 | 0.003 | 1.63E-09 | 0.001 | 0.003 | 7.90E-01 | BMI | 0.016 | 0.007 | 0.032 | 0.009 | -0.063 | 0.021 | 0.054 | 0.018 | 0.007 | 0.030 | 0.029 | 0.018 |
| rs7020477 | 9 | 116827760 | A | G | 0.003 | 0.003 | 3.01E-01 | 0.014 | 0.002 | 1.20E-08 | PC | -0.005 | 0.008 | -0.011 | 0.010 | -0.012 | 0.022 | 0.007 | 0.019 | 0.007 | 0.033 | 0.037 | 0.020 |
| rs73578186 | 9 | 126334485 | C | T | -0.009 | 0.003 | 1.79E-03 | 0.015 | 0.002 | 2.30E-10 | PC | -0.003 | 0.006 | -0.011 | 0.008 | 0.011 | 0.019 | -0.021 | 0.016 | -0.002 | 0.027 | -0.028 | 0.016 |
| rs3829849 | 9 | 129390800 | T | C | 0.014 | 0.002 | 1.93E-09 | 0.001 | 0.002 | 6.60E-01 | BMI | 0.023 | 0.006 | 0.023 | 0.008 | 0.011 | 0.018 | 0.007 | 0.016 | 0.037 | 0.027 | 0.015 | 0.016 |
| rs10733682 | 9 | 129460914 | A | G | 0.016 | 0.002 | 1.85E-12 | -0.002 | 0.002 | 4.50E-01 | BMI | -0.023 | 0.006 | -0.025 | 0.008 | -0.025 | 0.018 | -0.034 | 0.015 | -0.053 | 0.026 | -0.019 | 0.015 |
| rs1009473 | 9 | 138777312 | T | C | -0.013 | 0.002 | 3.36E-08 | -0.001 | 0.002 | 6.40E-01 | BMI | 0.009 | 0.006 | 0.009 | 0.008 | -0.017 | 0.018 | 0.005 | 0.015 | -0.003 | 0.026 | 0.013 | 0.015 |
| rs7084454 | 10 | 21821274 | A | G | 0.023 | 0.003 | 7.43E-20 | 0.011 | 0.002 | 6.40E-06 | BMI | 0.044 | 0.006 | 0.069 | 0.008 | 0.056 | 0.018 | 0.058 | 0.016 | 0.007 | 0.027 | -0.056 | 0.016 |
| rs34930419 | 10 | 33987329 | T | C | -0.046 | 0.008 | 1.37E-09 | -0.008 | 0.006 | 1.90E-01 | BMI | -0.010 | 0.018 | -0.030 | 0.023 | -0.019 | 0.052 | 0.014 | 0.045 | 0.052 | 0.079 | -0.009 | 0.045 |
| rs10823893 | 10 | 53677313 | A | G | 0.018 | 0.003 | 5.22E-11 | -0.004 | 0.002 | 5.10E-02 | BMI | -0.018 | 0.006 | -0.013 | 0.008 | -0.044 | 0.018 | -0.010 | 0.015 | -0.015 | 0.026 | 0.000 | 0.016 |
| rs10761785 | 10 | 65318766 | T | G | -0.017 | 0.002 | 4.17E-15 | 0.001 | 0.002 | 6.30E-01 | BMI | -0.025 | 0.006 | -0.030 | 0.008 | -0.039 | 0.018 | -0.020 | 0.015 | 0.008 | 0.026 | -0.014 | 0.015 |
| rs10824347 | 10 | 77645229 | A | T | 0.018 | 0.003 | 5.47E-11 | -0.003 | 0.003 | 3.10E-01 | BMI | 0.006 | 0.007 | 0.013 | 0.009 | -0.008 | 0.021 | 0.018 | 0.018 | -0.034 | 0.030 | -0.004 | 0.018 |
| rs7899106 | 10 | 87410904 | A | G | -0.038 | 0.005 | 7.65E-14 | -0.003 | 0.005 | 5.20E-01 | BMI | 0.023 | 0.014 | 0.030 | 0.019 | 0.056 | 0.042 | 0.072 | 0.036 | -0.006 | 0.061 | -0.032 | 0.035 |
| rs563296 | 10 | 99772404 | A | G | 0.018 | 0.002 | 8.85E-14 | -0.001 | 0.002 | 7.60E-01 | BMI | 0.005 | 0.006 | 0.003 | 0.008 | 0.002 | 0.018 | -0.012 | 0.016 | -0.021 | 0.027 | 0.008 | 0.016 |
| rs3977755 | 10 | 104420210 | T | C | -0.014 | 0.003 | 8.50E-09 | 0.010 | 0.002 | 3.10E-05 | BMI | 0.015 | 0.007 | 0.003 | 0.009 | 0.023 | 0.020 | 0.011 | 0.017 | -0.005 | 0.029 | -0.009 | 0.017 |
| rs6585198 | 10 | 114762237 | A | G | -0.015 | 0.002 | 1.22E-12 | -0.004 | 0.002 | 8.60E-02 | BMI | 0.041 | 0.006 | 0.045 | 0.008 | 0.003 | 0.018 | 0.013 | 0.015 | -0.021 | 0.025 | 0.081 | 0.015 |
| rs1681743 | 10 | 118565501 | T | C | -0.013 | 0.002 | 3.70E-08 | -0.004 | 0.002 | 1.20E-01 | BMI | -0.011 | 0.006 | -0.005 | 0.008 | -0.020 | 0.018 | -0.024 | 0.015 | -0.039 | 0.026 | -0.016 | 0.016 |
| rs1561589 | 10 | 126695673 | A | G | 0.016 | 0.002 | 4.24E-12 | 0.003 | 0.002 | 2.50E-01 | BMI | -0.005 | 0.006 | -0.002 | 0.008 | 0.019 | 0.018 | 0.000 | 0.016 | -0.025 | 0.027 | -0.017 | 0.016 |
| rs4880341 | 10 | 133992689 | T | C | -0.014 | 0.002 | 4.53E-10 | -0.003 | 0.002 | 2.40E-01 | BMI | -0.001 | 0.006 | -0.003 | 0.008 | 0.016 | 0.018 | 0.028 | 0.015 | -0.019 | 0.026 | -0.004 | 0.015 |
| rs7395632 | 11 | 958789 | T | C | -0.013 | 0.002 | 3.87E-08 | -0.003 | 0.002 | 1.80E-01 | BMI | -0.001 | 0.006 | 0.012 | 0.008 | -0.020 | 0.018 | -0.040 | 0.016 | -0.030 | 0.027 | -0.006 | 0.016 |
| rs4929923 | 11 | 8639200 | T | C | -0.022 | 0.002 | 2.51E-22 | 0.000 | 0.002 | 9.70E-01 | BMI | -0.002 | 0.006 | -0.008 | 0.008 | -0.006 | 0.018 | 0.010 | 0.016 | -0.018 | 0.026 | 0.004 | 0.016 |
| rs11022762 | 11 | 13335926 | T | C | -0.015 | 0.002 | 5.56E-11 | -0.008 | 0.002 | 2.20E-04 | BMI | -0.001 | 0.006 | 0.000 | 0.008 | 0.014 | 0.018 | -0.001 | 0.015 | 0.002 | 0.026 | 0.007 | 0.015 |
| rs6265 | 11 | 27679916 | T | C | -0.037 | 0.003 | 7.95E-40 | -0.011 | 0.003 | 4.70E-05 | BMI | -0.009 | 0.008 | -0.005 | 0.010 | -0.013 | 0.022 | 0.011 | 0.019 | -0.005 | 0.032 | -0.002 | 0.019 |
| rs10742752 | 11 | 45438374 | T | C | -0.013 | 0.002 | 3.98E-09 | -0.003 | 0.002 | 1.30E-01 | BMI | 0.009 | 0.006 | 0.016 | 0.008 | -0.006 | 0.018 | 0.014 | 0.016 | 0.000 | 0.027 | 0.014 | 0.016 |
| rs7124681 | 11 | 47529947 | A | C | 0.025 | 0.002 | 9.27E-29 | 0.009 | 0.002 | 7.20E-05 | BMI | -0.005 | 0.006 | -0.018 | 0.008 | -0.011 | 0.018 | -0.009 | 0.015 | -0.013 | 0.026 | -0.006 | 0.015 |
| rs893006 | 11 | 64365796 | A | C | 0.017 | 0.002 | 4.05E-13 | -0.001 | 0.002 | 7.00E-01 | BMI | 0.002 | 0.007 | 0.000 | 0.009 | 0.011 | 0.019 | -0.013 | 0.017 | -0.013 | 0.028 | 0.005 | 0.017 |
| rs625652 | 11 | 65641931 | A | T | -0.020 | 0.003 | 9.05E-13 | -0.002 | 0.002 | 4.00E-01 | BMI | -0.031 | 0.006 | -0.036 | 0.008 | -0.031 | 0.018 | -0.041 | 0.016 | -0.027 | 0.027 | -0.005 | 0.016 |
| rs7107409 | 11 | 76492195 | T | C | -0.018 | 0.003 | 2.05E-08 | 0.000 | 0.003 | 9.10E-01 | BMI | 0.017 | 0.007 | 0.026 | 0.012 | 0.023 | 0.024 | 0.029 | 0.021 | -0.011 | 0.036 | 0.007 | 0.022 |
| rs2605603 | 11 | 93221105 | A | G | -0.013 | 0.002 | 1.40E-09 | 0.001 | 0.002 | 7.70E-01 | BMI | 0.000 | 0.006 | -0.003 | 0.008 | -0.030 | 0.018 | 0.009 | 0.015 | 0.061 | 0.026 | -0.004 | 0.015 |
| rs2734849 | 11 | 113270160 | A | G | 0.008 | 0.002 | 2.10E-04 | -0.014 | 0.002 | 8.20E-10 | PC | 0.003 | 0.006 | 0.001 | 0.008 | 0.023 | 0.018 | -0.002 | 0.015 | 0.004 | 0.025 | -0.005 | 0.015 |
| rs1003081 | 11 | 118913993 | T | C | 0.014 | 0.002 | 6.27E-11 | -0.004 | 0.002 | 6.20E-02 | BMI | -0.016 | 0.006 | -0.010 | 0.008 | -0.001 | 0.018 | -0.036 | 0.015 | -0.015 | 0.026 | -0.024 | 0.016 |
| rs4459316 | 11 | 130756176 | T | C | -0.015 | 0.002 | 2.33E-11 | 0.002 | 0.002 | 3.10E-01 | BMI | 0.002 | 0.006 | -0.003 | 0.008 | 0.013 | 0.018 | -0.020 | 0.015 | 0.001 | 0.026 | 0.003 | 0.016 |
| rs1448355 | 11 | 131286685 | C | T | 0.001 | 0.002 | 6.03E-01 | -0.015 | 0.002 | 1.30E-10 | PC | -0.001 | 0.006 | -0.001 | 0.008 | 0.011 | 0.018 | -0.002 | 0.015 | 0.007 | 0.026 | -0.006 | 0.016 |
| rs329651 | 11 | 133767622 | T | G | 0.018 | 0.003 | 1.96E-10 | -0.009 | 0.003 | 1.90E-03 | BMI | -0.017 | 0.008 | -0.017 | 0.010 | -0.019 | 0.023 | -0.021 | 0.020 | -0.003 | 0.033 | -0.022 | 0.020 |
| rs12364470 | 11 | 134601012 | T | G | -0.020 | 0.003 | 2.54E-11 | -0.001 | 0.003 | 7.50E-01 | BMI | 0.011 | 0.009 | 0.019 | 0.012 | -0.029 | 0.027 | 0.013 | 0.023 | 0.036 | 0.040 | 0.016 | 0.024 |
| rs11611246 | 12 | 939480 | T | G | 0.021 | 0.003 | 1.27E-14 | 0.002 | 0.003 | 3.80E-01 | BMI | 0.010 | 0.007 | 0.007 | 0.010 | 0.036 | 0.022 | 0.029 | 0.018 | -0.018 | 0.032 | 0.009 | 0.019 |
| rs7976757 | 12 | 19207948 | T | C | -0.019 | 0.003 | 2.94E-10 | -0.002 | 0.003 | 5.30E-01 | BMI | -0.006 | 0.008 | 0.008 | 0.011 | -0.029 | 0.024 | 0.015 | 0.020 | -0.024 | 0.035 | 0.007 | 0.021 |
| rs16926318 | 12 | 23678745 | A | G | -0.023 | 0.004 | 4.23E-08 | 0.001 | 0.004 | 7.60E-01 | BMI | 0.005 | 0.011 | -0.008 | 0.014 | -0.010 | 0.031 | 0.022 | 0.027 | 0.005 | 0.045 | 0.028 | 0.027 |
| rs16926778 | 12 | 24002125 | C | G | 0.026 | 0.004 | 1.41E-09 | 0.006 | 0.004 | 1.30E-01 | BMI | 0.006 | 0.011 | 0.028 | 0.015 | 0.039 | 0.033 | -0.038 | 0.029 | 0.028 | 0.048 | 0.012 | 0.029 |
| rs35933007 | 12 | 38929341 | G | A | 0.007 | 0.003 | 3.97E-02 | -0.015 | 0.003 | 9.20E-09 | PC | 0.003 | 0.008 | 0.017 | 0.011 | 0.000 | 0.024 | 0.018 | 0.020 | -0.017 | 0.034 | -0.030 | 0.021 |
| rs7138803 | 12 | 50247468 | A | G | 0.029 | 0.002 | 9.70E-38 | 0.000 | 0.002 | 9.80E-01 | BMI | -0.004 | 0.006 | -0.010 | 0.008 | -0.004 | 0.018 | -0.004 | 0.015 | -0.008 | 0.026 | -0.006 | 0.016 |
| rs2271189 | 12 | 56494991 | A | G | -0.015 | 0.002 | 6.35E-10 | 0.005 | 0.002 | 1.60E-02 | BMI | -0.016 | 0.006 | -0.024 | 0.008 | -0.010 | 0.019 | 0.008 | 0.016 | 0.012 | 0.027 | -0.033 | 0.016 |
| rs11173522 | 12 | 60953472 | A | C | 0.017 | 0.003 | 9.01E-10 | -0.002 | 0.003 | 5.40E-01 | BMI | -0.006 | 0.007 | -0.017 | 0.010 | 0.004 | 0.022 | -0.025 | 0.019 | 0.002 | 0.031 | 0.019 | 0.019 |
| rs645026 | 12 | 69757931 | A | G | -0.016 | 0.003 | 4.79E-10 | 0.001 | 0.002 | 7.30E-01 | BMI | 0.017 | 0.007 | 0.018 | 0.009 | 0.009 | 0.020 | 0.022 | 0.018 | -0.001 | 0.030 | 0.050 | 0.018 |
| rs10746306 | 12 | 84477374 | T | G | 0.018 | 0.003 | 8.74E-09 | 0.002 | 0.003 | 4.00E-01 | BMI | 0.011 | 0.007 | 0.007 | 0.009 | 0.031 | 0.020 | 0.005 | 0.017 | 0.015 | 0.029 | 0.002 | 0.017 |
| rs2279574 | 12 | 89745477 | A | C | -0.017 | 0.002 | 1.96E-12 | 0.001 | 0.002 | 6.90E-01 | BMI | 0.003 | 0.007 | 0.009 | 0.009 | 0.006 | 0.019 | -0.007 | 0.017 | 0.035 | 0.028 | -0.018 | 0.017 |
| rs2712643 | 12 | 99638276 | A | C | -0.015 | 0.003 | 1.47E-09 | 0.004 | 0.003 | 1.00E-01 | BMI | 0.000 | 0.007 | -0.002 | 0.009 | 0.005 | 0.020 | 0.015 | 0.017 | -0.033 | 0.030 | -0.022 | 0.018 |
| rs17033633 | 12 | 103656343 | T | C | -0.021 | 0.003 | 7.26E-12 | 0.001 | 0.003 | 6.50E-01 | BMI | 0.019 | 0.008 | 0.013 | 0.010 | -0.003 | 0.023 | 0.006 | 0.020 | -0.002 | 0.034 | 0.011 | 0.020 |
| rs17508240 | 12 | 117577358 | A | G | 0.019 | 0.003 | 2.83E-09 | 0.002 | 0.003 | 3.60E-01 | BMI | 0.004 | 0.007 | 0.003 | 0.012 | 0.006 | 0.025 | -0.016 | 0.022 | -0.031 | 0.038 | 0.026 | 0.023 |
| rs206965 | 12 | 120856332 | T | C | 0.000 | 0.003 | 9.16E-01 | 0.016 | 0.003 | 9.10E-09 | PC | 0.010 | 0.007 | 0.008 | 0.010 | 0.014 | 0.022 | 0.032 | 0.019 | -0.008 | 0.032 | 0.014 | 0.019 |
| rs6489156 | 12 | 123100397 | T | C | 0.022 | 0.003 | 9.97E-18 | -0.001 | 0.002 | 6.40E-01 | BMI | 0.013 | 0.007 | 0.009 | 0.009 | 0.033 | 0.020 | 0.035 | 0.017 | 0.057 | 0.029 | 0.001 | 0.017 |
| rs9579775 | 13 | 20616557 | A | C | -0.025 | 0.004 | 3.66E-10 | -0.001 | 0.003 | 8.80E-01 | BMI | 0.023 | 0.011 | 0.044 | 0.015 | -0.028 | 0.031 | -0.007 | 0.027 | 0.003 | 0.046 | 0.067 | 0.028 |
| rs12872889 | 13 | 28674628 | T | C | -0.021 | 0.003 | 1.52E-11 | -0.009 | 0.003 | 9.80E-04 | BMI | 0.006 | 0.007 | 0.010 | 0.009 | 0.006 | 0.021 | 0.033 | 0.018 | 0.023 | 0.031 | -0.017 | 0.018 |
| rs12429545 | 13 | 54102206 | A | G | 0.029 | 0.003 | 1.09E-18 | -0.005 | 0.003 | 1.20E-01 | BMI | 0.005 | 0.009 | -0.005 | 0.012 | 0.030 | 0.027 | -0.012 | 0.023 | 0.032 | 0.039 | 0.035 | 0.023 |
| rs12874776 | 13 | 57794345 | C | A | 0.001 | 0.003 | 8.91E-01 | -0.023 | 0.004 | 8.00E-11 | PC | 0.001 | 0.010 | 0.007 | 0.013 | 0.036 | 0.030 | 0.007 | 0.026 | -0.005 | 0.044 | 0.018 | 0.026 |
| rs4055791 | 13 | 59266053 | T | C | -0.017 | 0.003 | 1.77E-10 | -0.003 | 0.002 | 2.30E-01 | BMI | -0.011 | 0.006 | -0.020 | 0.008 | 0.002 | 0.018 | -0.008 | 0.016 | 0.031 | 0.026 | -0.019 | 0.016 |
| rs1441264 | 13 | 79580919 | A | G | 0.017 | 0.002 | 5.19E-14 | 0.002 | 0.002 | 3.20E-01 | BMI | -0.008 | 0.007 | -0.024 | 0.009 | 0.006 | 0.019 | 0.000 | 0.016 | -0.033 | 0.027 | -0.009 | 0.017 |
| rs9531786 | 13 | 85983968 | C | G | -0.014 | 0.002 | 8.39E-09 | -0.006 | 0.002 | 6.70E-03 | BMI | -0.010 | 0.006 | -0.007 | 0.008 | 0.008 | 0.018 | -0.013 | 0.016 | -0.051 | 0.027 | 0.006 | 0.016 |
| rs77432547 | 13 | 86494817 | A | G | -0.018 | 0.003 | 2.28E-09 | 0.000 | 0.002 | 9.90E-01 | BMI | 0.013 | 0.007 | 0.007 | 0.009 | 0.008 | 0.020 | 0.019 | 0.017 | 0.001 | 0.029 | 0.008 | 0.017 |
| rs1927790 | 13 | 96922191 | T | C | -0.014 | 0.002 | 1.27E-09 | -0.004 | 0.002 | 4.70E-02 | BMI | 0.002 | 0.006 | 0.000 | 0.008 | -0.018 | 0.018 | 0.012 | 0.016 | 0.032 | 0.026 | -0.012 | 0.016 |
| rs4586287 | 13 | 99245334 | C | G | 0.014 | 0.003 | 2.95E-08 | -0.005 | 0.002 | 3.00E-02 | BMI | -0.012 | 0.007 | -0.011 | 0.009 | -0.021 | 0.019 | 0.002 | 0.016 | 0.005 | 0.028 | -0.001 | 0.016 |
| rs7335249 | 13 | 109962579 | T | C | 0.014 | 0.002 | 4.91E-09 | 0.003 | 0.002 | 1.70E-01 | BMI | 0.002 | 0.006 | -0.002 | 0.008 | 0.006 | 0.018 | 0.009 | 0.016 | 0.005 | 0.026 | 0.001 | 0.016 |
| rs2479958 | 13 | 111984244 | A | G | 0.014 | 0.002 | 1.38E-08 | -0.003 | 0.002 | 1.60E-01 | BMI | -0.004 | 0.006 | -0.001 | 0.008 | 0.028 | 0.018 | 0.008 | 0.016 | -0.033 | 0.027 | -0.013 | 0.016 |
| rs2754079 | 14 | 25886140 | A | G | -0.016 | 0.002 | 2.31E-11 | -0.002 | 0.002 | 2.90E-01 | BMI | 0.014 | 0.006 | 0.022 | 0.008 | 0.001 | 0.018 | -0.020 | 0.016 | 0.054 | 0.027 | -0.014 | 0.016 |
| rs1569979 | 14 | 29681294 | A | G | 0.023 | 0.003 | 1.57E-17 | 0.000 | 0.003 | 9.10E-01 | BMI | -0.002 | 0.007 | 0.001 | 0.010 | -0.021 | 0.021 | 0.001 | 0.018 | -0.020 | 0.030 | 0.010 | 0.018 |
| rs17522122 | 14 | 33302882 | T | G | 0.017 | 0.002 | 6.46E-14 | -0.001 | 0.002 | 5.10E-01 | BMI | -0.010 | 0.006 | -0.005 | 0.008 | -0.029 | 0.018 | 0.004 | 0.015 | 0.037 | 0.026 | -0.016 | 0.016 |
| rs198262 | 14 | 57283283 | C | T | 0.003 | 0.006 | 6.11E-01 | 0.030 | 0.005 | 3.90E-08 | PC | 0.002 | 0.015 | 0.011 | 0.020 | 0.041 | 0.044 | -0.020 | 0.038 | -0.019 | 0.065 | -0.010 | 0.039 |
| rs35020117 | 14 | 73341655 | T | G | 0.017 | 0.003 | 1.23E-09 | 0.000 | 0.002 | 9.90E-01 | BMI | -0.005 | 0.006 | -0.003 | 0.009 | 0.019 | 0.019 | -0.025 | 0.016 | 0.005 | 0.028 | -0.007 | 0.016 |
| rs7141420 | 14 | 79899454 | T | C | 0.021 | 0.002 | 7.84E-22 | -0.006 | 0.002 | 1.00E-02 | BMI | 0.012 | 0.006 | 0.007 | 0.008 | 0.034 | 0.018 | -0.002 | 0.016 | 0.000 | 0.027 | 0.021 | 0.016 |
| rs1285997 | 14 | 91513029 | C | G | -0.015 | 0.003 | 3.18E-09 | -0.002 | 0.002 | 3.20E-01 | BMI | 0.001 | 0.007 | 0.007 | 0.009 | 0.003 | 0.020 | 0.012 | 0.017 | -0.004 | 0.028 | -0.037 | 0.017 |
| rs942066 | 14 | 94031914 | A | G | -0.020 | 0.003 | 1.04E-13 | 0.003 | 0.002 | 2.10E-01 | BMI | 0.002 | 0.006 | -0.002 | 0.008 | -0.023 | 0.018 | 0.027 | 0.016 | 0.006 | 0.027 | -0.016 | 0.016 |
| rs3803286 | 14 | 103246470 | A | G | 0.017 | 0.002 | 8.29E-13 | -0.004 | 0.002 | 7.50E-02 | BMI | -0.020 | 0.006 | -0.032 | 0.008 | -0.018 | 0.019 | 0.002 | 0.016 | 0.013 | 0.027 | 0.015 | 0.016 |
| rs709400 | 14 | 104149475 | A | G | 0.018 | 0.002 | 4.41E-16 | -0.001 | 0.002 | 5.20E-01 | BMI | -0.020 | 0.006 | -0.021 | 0.008 | -0.016 | 0.018 | -0.019 | 0.015 | -0.044 | 0.026 | 0.005 | 0.016 |
| rs12101625 | 15 | 26972431 | C | G | 0.014 | 0.002 | 5.54E-09 | 0.004 | 0.002 | 5.60E-02 | BMI | -0.018 | 0.006 | -0.021 | 0.008 | -0.021 | 0.018 | -0.018 | 0.016 | 0.020 | 0.027 | -0.024 | 0.016 |
| rs166835 | 15 | 47716037 | C | T | -0.001 | 0.002 | 8.32E-01 | 0.013 | 0.002 | 3.40E-09 | PC | -0.013 | 0.006 | -0.023 | 0.008 | -0.038 | 0.018 | 0.002 | 0.015 | 0.001 | 0.026 | -0.015 | 0.015 |
| rs6493498 | 15 | 51754451 | T | C | 0.016 | 0.002 | 1.53E-13 | 0.004 | 0.002 | 8.70E-02 | BMI | -0.010 | 0.006 | -0.011 | 0.008 | -0.016 | 0.018 | -0.028 | 0.015 | 0.016 | 0.026 | 0.011 | 0.015 |
| rs8024806 | 15 | 53473990 | T | C | 0.027 | 0.005 | 9.76E-09 | -0.006 | 0.005 | 2.00E-01 | BMI | -0.010 | 0.013 | -0.024 | 0.017 | -0.025 | 0.036 | 0.012 | 0.032 | 0.031 | 0.055 | -0.015 | 0.032 |
| rs8033510 | 15 | 61445514 | T | C | 0.014 | 0.002 | 1.13E-08 | -0.003 | 0.002 | 2.00E-01 | BMI | 0.001 | 0.006 | 0.004 | 0.008 | -0.003 | 0.018 | -0.035 | 0.016 | -0.039 | 0.027 | -0.024 | 0.016 |
| rs4776982 | 15 | 68114974 | A | G | 0.031 | 0.003 | 4.25E-33 | -0.006 | 0.003 | 3.00E-02 | BMI | -0.006 | 0.007 | 0.007 | 0.009 | -0.033 | 0.021 | -0.002 | 0.018 | 0.017 | 0.031 | -0.007 | 0.018 |
| rs12914489 | 15 | 74187937 | A | G | 0.023 | 0.004 | 1.64E-10 | 0.000 | 0.004 | 9.10E-01 | BMI | 0.022 | 0.010 | 0.026 | 0.013 | 0.026 | 0.029 | 0.051 | 0.024 | 0.026 | 0.042 | -0.030 | 0.026 |
| rs62007782 | 15 | 78029797 | A | G | -0.018 | 0.003 | 1.94E-09 | 0.001 | 0.002 | 6.20E-01 | BMI | -0.004 | 0.007 | -0.009 | 0.009 | -0.009 | 0.020 | 0.036 | 0.017 | 0.053 | 0.029 | -0.029 | 0.018 |
| rs12593036 | 15 | 81058652 | A | G | 0.015 | 0.003 | 6.65E-10 | 0.006 | 0.002 | 1.90E-02 | BMI | -0.001 | 0.007 | 0.000 | 0.009 | -0.002 | 0.019 | -0.008 | 0.017 | -0.009 | 0.028 | 0.017 | 0.017 |
| rs4702 | 15 | 91426560 | G | A | 0.000 | 0.002 | 9.14E-01 | 0.012 | 0.002 | 3.80E-08 | PC | 0.029 | 0.006 | 0.016 | 0.008 | 0.057 | 0.018 | 0.071 | 0.015 | -0.002 | 0.026 | 0.007 | 0.015 |
| rs7498044 | 15 | 92573639 | A | G | -0.018 | 0.003 | 2.73E-08 | 0.002 | 0.003 | 5.70E-01 | BMI | 0.005 | 0.008 | 0.002 | 0.010 | 0.027 | 0.022 | -0.015 | 0.019 | 0.000 | 0.033 | -0.003 | 0.019 |
| rs214249 | 16 | 348687 | T | G | 0.017 | 0.002 | 1.37E-12 | -0.003 | 0.002 | 1.30E-01 | BMI | 0.000 | 0.006 | 0.003 | 0.008 | -0.004 | 0.018 | 0.016 | 0.015 | 0.015 | 0.026 | 0.003 | 0.015 |
| rs12448257 | 16 | 3599655 | A | G | 0.015 | 0.003 | 3.13E-08 | 0.005 | 0.003 | 5.70E-02 | BMI | 0.002 | 0.008 | 0.001 | 0.010 | -0.012 | 0.022 | 0.027 | 0.019 | -0.043 | 0.033 | 0.009 | 0.019 |
| rs879620 | 16 | 4015729 | T | C | 0.025 | 0.002 | 5.45E-26 | -0.003 | 0.002 | 1.70E-01 | BMI | 0.009 | 0.006 | 0.014 | 0.008 | 0.020 | 0.018 | 0.011 | 0.016 | 0.025 | 0.027 | 0.013 | 0.016 |
| rs6498759 | 16 | 18020295 | T | C | -0.001 | 0.003 | 5.94E-01 | 0.013 | 0.002 | 2.70E-08 | PC | 0.012 | 0.007 | 0.021 | 0.009 | 0.034 | 0.020 | -0.028 | 0.017 | 0.021 | 0.029 | 0.017 | 0.018 |
| rs9926784 | 16 | 19941968 | T | C | 0.025 | 0.003 | 6.01E-20 | 0.000 | 0.003 | 9.40E-01 | BMI | -0.011 | 0.008 | -0.006 | 0.010 | 0.011 | 0.023 | -0.023 | 0.019 | -0.039 | 0.032 | -0.018 | 0.020 |
| rs17767765 | 16 | 24539793 | T | C | -0.013 | 0.002 | 2.60E-08 | 0.000 | 0.002 | 9.30E-01 | BMI | 0.001 | 0.007 | 0.001 | 0.009 | 0.000 | 0.019 | 0.015 | 0.016 | 0.012 | 0.027 | 0.006 | 0.016 |
| rs7498665 | 16 | 28883241 | A | G | -0.026 | 0.002 | 1.94E-32 | 0.005 | 0.002 | 1.90E-02 | BMI | -0.005 | 0.006 | -0.007 | 0.008 | 0.003 | 0.018 | -0.014 | 0.015 | -0.006 | 0.026 | 0.015 | 0.016 |
| rs2289292 | 16 | 30097630 | T | C | -0.019 | 0.003 | 1.13E-14 | 0.001 | 0.002 | 5.90E-01 | BMI | -0.002 | 0.007 | 0.000 | 0.009 | 0.024 | 0.019 | 0.006 | 0.017 | -0.008 | 0.029 | -0.028 | 0.017 |
| rs4889606 | 16 | 31011183 | A | G | 0.027 | 0.002 | 1.25E-32 | 0.001 | 0.002 | 6.70E-01 | BMI | -0.014 | 0.006 | -0.011 | 0.008 | -0.001 | 0.018 | -0.010 | 0.015 | -0.007 | 0.026 | -0.013 | 0.016 |
| rs1558902 | 16 | 53803574 | A | T | 0.070 | 0.002 | 1.10E-215 | -0.004 | 0.002 | 4.60E-02 | BMI | -0.059 | 0.006 | -0.063 | 0.008 | -0.072 | 0.018 | -0.053 | 0.015 | -0.079 | 0.026 | -0.088 | 0.015 |
| rs3730399 | 16 | 67229019 | A | G | -0.009 | 0.005 | 7.35E-02 | 0.025 | 0.004 | 3.30E-08 | PC | -0.015 | 0.012 | 0.005 | 0.016 | -0.038 | 0.035 | 0.021 | 0.030 | -0.032 | 0.051 | -0.015 | 0.030 |
| rs2012502 | 16 | 81728081 | A | C | 0.015 | 0.002 | 2.97E-11 | -0.004 | 0.002 | 9.40E-02 | BMI | -0.008 | 0.006 | -0.001 | 0.008 | -0.009 | 0.019 | -0.017 | 0.016 | -0.034 | 0.027 | -0.007 | 0.016 |
| rs4790292 | 17 | 1824305 | A | C | -0.026 | 0.003 | 1.65E-15 | 0.004 | 0.003 | 1.60E-01 | BMI | 0.013 | 0.009 | 0.009 | 0.012 | 0.027 | 0.026 | 0.027 | 0.022 | 0.054 | 0.038 | 0.003 | 0.023 |
| rs3826408 | 17 | 7101292 | T | C | 0.012 | 0.002 | 4.48E-08 | -0.003 | 0.002 | 1.60E-01 | BMI | 0.014 | 0.006 | 0.023 | 0.008 | -0.016 | 0.018 | 0.011 | 0.015 | 0.006 | 0.026 | 0.026 | 0.015 |
| rs7209653 | 17 | 19882084 | T | C | 0.002 | 0.003 | 4.02E-01 | 0.016 | 0.002 | 9.10E-11 | PC | -0.007 | 0.007 | 0.000 | 0.009 | -0.035 | 0.020 | -0.012 | 0.017 | 0.012 | 0.029 | -0.023 | 0.017 |
| rs4986044 | 17 | 21261560 | T | C | -0.017 | 0.002 | 2.20E-15 | -0.002 | 0.002 | 2.70E-01 | BMI | -0.002 | 0.006 | -0.002 | 0.008 | 0.027 | 0.018 | -0.001 | 0.015 | -0.015 | 0.026 | 0.014 | 0.015 |
| rs12603813 | 17 | 43196584 | T | C | -0.003 | 0.003 | 2.94E-01 | 0.014 | 0.003 | 1.30E-08 | PC | 0.005 | 0.007 | 0.017 | 0.009 | 0.036 | 0.020 | -0.003 | 0.017 | 0.033 | 0.030 | -0.032 | 0.017 |
| rs56161855 | 17 | 46288649 | A | T | -0.026 | 0.004 | 1.48E-11 | 0.001 | 0.003 | 7.90E-01 | BMI | 0.003 | 0.009 | -0.008 | 0.011 | 0.062 | 0.025 | 0.009 | 0.021 | -0.008 | 0.036 | 0.005 | 0.022 |
| rs9906944 | 17 | 47091420 | T | C | -0.019 | 0.002 | 7.25E-15 | 0.003 | 0.002 | 2.00E-01 | BMI | -0.004 | 0.007 | -0.002 | 0.009 | -0.028 | 0.019 | -0.017 | 0.017 | -0.039 | 0.028 | -0.007 | 0.017 |
| rs7211966 | 17 | 54374611 | T | C | -0.047 | 0.008 | 4.89E-09 | 0.003 | 0.008 | 7.10E-01 | BMI | 0.001 | 0.026 | 0.007 | 0.034 | 0.033 | 0.074 | -0.178 | 0.071 | 0.019 | 0.108 | -0.052 | 0.067 |
| rs8075273 | 17 | 61728881 | A | C | -0.016 | 0.002 | 5.56E-11 | 0.003 | 0.002 | 2.30E-01 | BMI | 0.011 | 0.007 | 0.006 | 0.009 | 0.011 | 0.019 | 0.018 | 0.017 | 0.042 | 0.028 | -0.001 | 0.017 |
| rs2619976 | 17 | 71754545 | T | C | 0.015 | 0.002 | 6.17E-10 | 0.001 | 0.002 | 6.00E-01 | BMI | 0.006 | 0.007 | 0.010 | 0.009 | -0.031 | 0.019 | -0.001 | 0.016 | 0.015 | 0.028 | 0.008 | 0.017 |
| rs4969387 | 17 | 79081724 | C | G | -0.015 | 0.003 | 6.90E-09 | -0.001 | 0.002 | 7.60E-01 | BMI | -0.013 | 0.007 | -0.010 | 0.009 | -0.011 | 0.021 | 0.023 | 0.018 | -0.024 | 0.030 | -0.036 | 0.018 |
| rs66643547 | 17 | 79375315 | C | T | 0.001 | 0.003 | 6.52E-01 | -0.015 | 0.002 | 3.20E-11 | PC | 0.020 | 0.007 | 0.021 | 0.009 | 0.005 | 0.019 | 0.024 | 0.017 | 0.061 | 0.029 | 0.019 | 0.017 |
| rs9955276 | 18 | 1839339 | T | C | 0.022 | 0.003 | 8.02E-12 | -0.008 | 0.003 | 1.10E-02 | BMI | -0.002 | 0.009 | -0.003 | 0.011 | 0.034 | 0.025 | -0.021 | 0.021 | -0.044 | 0.036 | 0.045 | 0.021 |
| rs1808579 | 18 | 21104888 | T | C | -0.020 | 0.002 | 9.44E-20 | 0.007 | 0.002 | 3.10E-03 | BMI | -0.009 | 0.006 | -0.016 | 0.008 | -0.043 | 0.018 | 0.001 | 0.015 | 0.043 | 0.026 | -0.048 | 0.015 |
| rs16975921 | 18 | 39914870 | A | T | -0.016 | 0.003 | 3.87E-11 | 0.006 | 0.002 | 7.80E-03 | BMI | 0.009 | 0.006 | 0.005 | 0.008 | 0.011 | 0.019 | 0.004 | 0.016 | 0.022 | 0.027 | 0.026 | 0.016 |
| rs1518159 | 18 | 40760146 | T | C | -0.019 | 0.003 | 6.66E-13 | -0.003 | 0.003 | 2.70E-01 | BMI | -0.005 | 0.007 | -0.008 | 0.009 | -0.008 | 0.020 | -0.011 | 0.017 | 0.054 | 0.029 | 0.001 | 0.018 |
| rs784256 | 18 | 53398626 | G | A | -0.018 | 0.003 | 1.77E-09 | 0.019 | 0.003 | 6.20E-11 | PC | -0.005 | 0.008 | 0.006 | 0.010 | -0.020 | 0.023 | 0.009 | 0.020 | -0.015 | 0.033 | -0.034 | 0.020 |
| rs6567160 | 18 | 57829135 | T | C | -0.057 | 0.003 | 8.74E-108 | -0.004 | 0.003 | 1.60E-01 | BMI | 0.021 | 0.007 | 0.001 | 0.009 | 0.024 | 0.021 | 0.025 | 0.018 | -0.002 | 0.030 | 0.012 | 0.018 |
| rs17066856 | 18 | 58049656 | T | C | 0.040 | 0.004 | 1.39E-27 | 0.006 | 0.004 | 1.40E-01 | BMI | -0.012 | 0.010 | -0.004 | 0.014 | -0.006 | 0.030 | -0.030 | 0.026 | -0.005 | 0.043 | -0.007 | 0.026 |
| rs12981256 | 19 | 1865901 | A | G | 0.018 | 0.002 | 3.93E-15 | -0.005 | 0.002 | 4.00E-02 | BMI | 0.006 | 0.006 | 0.009 | 0.008 | -0.005 | 0.018 | 0.018 | 0.015 | -0.022 | 0.026 | -0.009 | 0.016 |
| rs895330 | 19 | 4060707 | C | G | 0.018 | 0.003 | 6.65E-10 | 0.005 | 0.003 | 5.10E-02 | BMI | 0.021 | 0.008 | 0.031 | 0.011 | 0.016 | 0.023 | 0.033 | 0.020 | -0.011 | 0.034 | 0.025 | 0.021 |
| rs273504 | 19 | 18215247 | A | G | -0.015 | 0.002 | 1.27E-10 | -0.003 | 0.002 | 2.50E-01 | BMI | -0.017 | 0.006 | -0.019 | 0.008 | 0.012 | 0.018 | -0.039 | 0.015 | -0.041 | 0.026 | -0.016 | 0.015 |
| rs17724992 | 19 | 18454825 | A | G | 0.017 | 0.003 | 6.65E-12 | -0.006 | 0.002 | 1.20E-02 | BMI | 0.002 | 0.007 | 0.010 | 0.009 | 0.006 | 0.020 | -0.008 | 0.017 | 0.025 | 0.029 | -0.007 | 0.017 |
| rs11880870 | 19 | 18830704 | A | G | 0.020 | 0.002 | 3.31E-18 | 0.001 | 0.002 | 7.20E-01 | BMI | 0.014 | 0.006 | 0.022 | 0.008 | 0.040 | 0.018 | 0.007 | 0.016 | 0.047 | 0.027 | -0.022 | 0.016 |
| rs17751061 | 19 | 19413092 | T | C | -0.018 | 0.003 | 1.29E-08 | -0.001 | 0.003 | 7.20E-01 | BMI | 0.032 | 0.008 | 0.016 | 0.011 | 0.029 | 0.024 | 0.058 | 0.020 | -0.002 | 0.035 | 0.028 | 0.021 |
| rs12462975 | 19 | 30272202 | A | G | 0.020 | 0.003 | 2.63E-16 | 0.003 | 0.002 | 2.00E-01 | BMI | 0.020 | 0.006 | 0.011 | 0.008 | 0.045 | 0.019 | 0.037 | 0.016 | 0.022 | 0.027 | 0.054 | 0.016 |
| rs11667280 | 19 | 30705747 | C | G | 0.016 | 0.003 | 3.14E-08 | 0.004 | 0.003 | 1.40E-01 | BMI | 0.010 | 0.008 | 0.021 | 0.011 | -0.005 | 0.023 | 0.005 | 0.020 | 0.034 | 0.034 | 0.020 | 0.020 |
| rs7250833 | 19 | 33937277 | T | C | 0.015 | 0.002 | 7.22E-10 | 0.009 | 0.002 | 3.00E-04 | BMI | -0.010 | 0.007 | -0.018 | 0.009 | 0.006 | 0.020 | -0.014 | 0.017 | -0.012 | 0.028 | -0.015 | 0.017 |
| rs185350 | 19 | 34306816 | T | C | 0.013 | 0.002 | 7.14E-09 | -0.001 | 0.002 | 6.00E-01 | BMI | -0.012 | 0.006 | -0.011 | 0.008 | -0.011 | 0.018 | -0.004 | 0.015 | -0.012 | 0.026 | -0.017 | 0.015 |
| rs429358 | 19 | 45411941 | T | C | 0.028 | 0.004 | 3.17E-14 | 0.011 | 0.003 | 2.60E-04 | BMI | 0.015 | 0.009 | 0.017 | 0.012 | 0.002 | 0.026 | 0.000 | 0.022 | 0.064 | 0.038 | 0.024 | 0.022 |
| rs11672660 | 19 | 46180184 | T | C | -0.032 | 0.003 | 5.93E-31 | -0.004 | 0.003 | 1.80E-01 | BMI | 0.045 | 0.008 | 0.050 | 0.010 | 0.038 | 0.022 | 0.060 | 0.019 | 0.051 | 0.032 | 0.021 | 0.019 |
| rs9304665 | 19 | 47602577 | A | T | 0.020 | 0.003 | 1.84E-14 | 0.004 | 0.003 | 1.10E-01 | BMI | -0.019 | 0.007 | -0.015 | 0.009 | -0.008 | 0.021 | -0.014 | 0.018 | 0.005 | 0.030 | -0.005 | 0.018 |
| rs1884389 | 20 | 1410582 | T | C | -0.014 | 0.002 | 1.20E-09 | -0.002 | 0.002 | 3.30E-01 | BMI | 0.010 | 0.006 | 0.015 | 0.008 | 0.020 | 0.018 | 0.007 | 0.015 | 0.011 | 0.026 | 0.002 | 0.015 |
| rs1884897 | 20 | 6612832 | A | G | -0.017 | 0.002 | 9.06E-14 | -0.001 | 0.002 | 6.40E-01 | BMI | 0.005 | 0.006 | -0.003 | 0.008 | 0.015 | 0.018 | -0.004 | 0.016 | 0.001 | 0.027 | 0.048 | 0.016 |
| rs1409818 | 20 | 21381121 | T | C | 0.025 | 0.004 | 1.58E-11 | 0.003 | 0.004 | 4.90E-01 | BMI | -0.018 | 0.010 | -0.024 | 0.013 | 0.036 | 0.029 | -0.040 | 0.026 | -0.013 | 0.043 | -0.011 | 0.026 |
| rs6050446 | 20 | 25195509 | A | G | -0.037 | 0.006 | 6.20E-09 | -0.014 | 0.006 | 2.30E-02 | BMI | 0.014 | 0.019 | -0.001 | 0.024 | 0.019 | 0.055 | 0.023 | 0.046 | 0.073 | 0.077 | -0.016 | 0.048 |
| rs6142096 | 20 | 32686658 | A | G | 0.013 | 0.002 | 4.17E-09 | 0.001 | 0.002 | 7.40E-01 | BMI | -0.017 | 0.006 | -0.008 | 0.008 | -0.004 | 0.018 | -0.018 | 0.015 | 0.010 | 0.026 | -0.026 | 0.015 |
| rs16989232 | 20 | 39291784 | A | G | 0.013 | 0.002 | 1.52E-08 | -0.001 | 0.002 | 7.30E-01 | BMI | -0.010 | 0.006 | -0.027 | 0.009 | -0.013 | 0.019 | 0.015 | 0.016 | 0.050 | 0.027 | -0.002 | 0.016 |
| rs17806224 | 20 | 51065854 | A | G | -0.029 | 0.003 | 3.52E-22 | -0.007 | 0.003 | 1.00E-02 | BMI | -0.019 | 0.008 | -0.005 | 0.011 | -0.039 | 0.024 | -0.020 | 0.020 | -0.071 | 0.036 | -0.055 | 0.021 |
| rs6129084 | 20 | 59828857 | A | T | -0.005 | 0.003 | 4.80E-02 | -0.014 | 0.002 | 9.30E-10 | PC | -0.001 | 0.006 | 0.005 | 0.008 | -0.004 | 0.019 | -0.010 | 0.016 | 0.015 | 0.027 | -0.001 | 0.016 |
| rs6010784 | 20 | 61540319 | T | C | 0.013 | 0.002 | 1.08E-08 | 0.010 | 0.002 | 5.40E-06 | BMI | 0.006 | 0.006 | 0.006 | 0.008 | 0.009 | 0.018 | 0.000 | 0.015 | 0.021 | 0.025 | -0.036 | 0.015 |
| rs2836754 | 21 | 40291740 | T | C | -0.015 | 0.002 | 3.73E-11 | 0.001 | 0.002 | 8.20E-01 | BMI | 0.002 | 0.006 | 0.003 | 0.008 | 0.009 | 0.018 | 0.006 | 0.015 | 0.000 | 0.026 | -0.001 | 0.016 |
| rs915814 | 21 | 46493003 | A | G | -0.018 | 0.003 | 5.88E-09 | 0.001 | 0.003 | 5.70E-01 | BMI | -0.001 | 0.007 | -0.018 | 0.009 | 0.045 | 0.021 | 0.017 | 0.018 | -0.028 | 0.030 | 0.039 | 0.018 |
| rs427943 | 21 | 46570896 | A | C | -0.018 | 0.002 | 1.37E-14 | 0.001 | 0.002 | 5.00E-01 | BMI | 0.002 | 0.006 | -0.001 | 0.008 | 0.010 | 0.018 | 0.031 | 0.015 | 0.029 | 0.026 | 0.020 | 0.015 |
| rs136553 | 22 | 27255675 | C | T | -0.010 | 0.003 | 1.86E-04 | -0.015 | 0.002 | 2.30E-11 | PC | -0.004 | 0.006 | -0.001 | 0.008 | 0.000 | 0.018 | -0.005 | 0.015 | 0.014 | 0.027 | 0.002 | 0.016 |
| rs7288455 | 22 | 39966547 | A | G | 0.001 | 0.003 | 7.53E-01 | 0.013 | 0.002 | 4.00E-09 | PC | 0.001 | 0.006 | 0.005 | 0.008 | -0.018 | 0.019 | 0.004 | 0.016 | -0.025 | 0.027 | -0.007 | 0.016 |
| rs13053342 | 22 | 48871624 | A | G | 0.015 | 0.002 | 1.32E-10 | -0.001 | 0.002 | 7.60E-01 | BMI | 0.000 | 0.006 | 0.000 | 0.009 | -0.047 | 0.019 | 0.004 | 0.016 | -0.012 | 0.027 | 0.012 | 0.016 |

| **Table S15. Beta estimates for the SNPs included in the multivariable MR of BMI, computer use, and colorectal cancer risk** | | | | | | | | | | | | | | | | | | | |
| --- | --- | --- | --- | --- | --- | --- | --- | --- | --- | --- | --- | --- | --- | --- | --- | --- | --- | --- | --- |
| **1) Analysis of colorectal cancer in men and women combined** | | | | | | | | | | | | | | | | | | | |
| SNP | CHR | POS | EA | OA | EAF | beta_bmi | se_bmi | pval_bmi | beta_pc | se_pc | pval_pc | Phenotype | beta_crc | se_crc | beta_co | | se_co | beta_re | se_re |
| rs2748985 | 1 | 1853184 | T | C | 0.455 | -0.014 | 0.002 | 5.53E-13 | -0.015 | 0.002 | 4.10E-12 | PC | 0.006 | 0.010 | 0.016 | | 0.012 | -0.003 | 0.015 |
| rs10909880 | 1 | 2727804 | T | C | 0.4522 | -0.014 | 0.002 | 2.21E-16 | -0.006 | 0.002 | 7.30E-03 | BMI | -0.024 | 0.010 | -0.019 | | 0.012 | -0.032 | 0.015 |
| rs11121210 | 1 | 8708529 | T | C | 0.3445 | -0.011 | 0.002 | 2.37E-10 | 0.007 | 0.002 | 2.60E-03 | BMI | -0.004 | 0.010 | -0.005 | | 0.012 | -0.005 | 0.016 |
| rs10779751 | 1 | 11284336 | A | G | 0.2787 | 0.013 | 0.002 | 2.66E-13 | 0.003 | 0.002 | 2.00E-01 | BMI | -0.002 | 0.011 | 0.006 | | 0.013 | -0.019 | 0.017 |
| rs61740466 | 1 | 19934900 | A | G | 0.2346 | -0.014 | 0.002 | 2.24E-10 | 0.001 | 0.003 | 6.80E-01 | BMI | -0.010 | 0.011 | -0.028 | | 0.013 | 0.010 | 0.017 |
| rs4655141 | 1 | 23312025 | T | C | 0.8362 | -0.017 | 0.002 | 6.20E-15 | 0.010 | 0.003 | 1.00E-03 | BMI | -0.027 | 0.013 | -0.035 | | 0.015 | -0.043 | 0.020 |
| rs2228552 | 1 | 32165495 | T | G | 0.5663 | 0.012 | 0.002 | 1.80E-11 | -0.001 | 0.002 | 7.50E-01 | BMI | -0.008 | 0.010 | -0.011 | | 0.012 | -0.015 | 0.016 |
| rs4653017 | 1 | 33776728 | T | C | 0.6679 | 0.012 | 0.002 | 1.11E-10 | 0.000 | 0.002 | 9.00E-01 | BMI | -0.003 | 0.010 | -0.002 | | 0.012 | -0.009 | 0.016 |
| rs7512146 | 1 | 34283008 | T | G | 0.5272 | -0.010 | 0.002 | 1.29E-08 | 0.000 | 0.002 | 8.80E-01 | BMI | -0.012 | 0.010 | -0.013 | | 0.012 | -0.012 | 0.015 |
| rs11577094 | 1 | 38026600 | T | C | 0.0791 | 0.019 | 0.003 | 3.28E-10 | -0.009 | 0.004 | 2.10E-02 | BMI | -0.038 | 0.019 | -0.021 | | 0.022 | -0.051 | 0.029 |
| rs112646560 | 1 | 39560250 | T | C | 0.2119 | 0.018 | 0.002 | 1.29E-14 | 0.003 | 0.003 | 3.40E-01 | BMI | 0.008 | 0.012 | 0.003 | | 0.014 | 0.024 | 0.019 |
| rs2984618 | 1 | 47690438 | T | G | 0.4408 | 0.017 | 0.002 | 3.80E-24 | 0.004 | 0.002 | 9.30E-02 | BMI | -0.019 | 0.010 | -0.022 | | 0.012 | -0.037 | 0.015 |
| rs657452 | 1 | 49589847 | A | G | 0.4042 | 0.019 | 0.002 | 3.17E-30 | -0.005 | 0.002 | 4.00E-02 | BMI | 0.015 | 0.010 | 0.015 | | 0.012 | 0.010 | 0.016 |
| rs587271 | 1 | 54743111 | T | C | 0.69 | 0.012 | 0.002 | 6.21E-11 | 0.010 | 0.002 | 7.00E-05 | BMI | -0.002 | 0.011 | 0.007 | | 0.013 | -0.024 | 0.017 |
| rs79906980 | 1 | 57887985 | T | C | 0.1555 | 0.016 | 0.003 | 1.69E-09 | 0.001 | 0.003 | 7.30E-01 | BMI | -0.016 | 0.014 | -0.012 | | 0.016 | -0.015 | 0.021 |
| rs12140153 | 1 | 62579891 | T | G | 0.0911 | -0.035 | 0.003 | 1.44E-25 | 0.004 | 0.004 | 3.20E-01 | BMI | -0.015 | 0.019 | -0.026 | | 0.022 | 0.024 | 0.029 |
| rs2503185 | 1 | 66461401 | A | G | 0.5097 | 0.013 | 0.002 | 1.33E-14 | 0.000 | 0.002 | 8.70E-01 | BMI | -0.010 | 0.010 | -0.003 | | 0.012 | -0.003 | 0.015 |
| rs3101336 | 1 | 72751185 | T | C | 0.3825 | -0.025 | 0.002 | 4.80E-54 | -0.007 | 0.002 | 1.20E-03 | BMI | 0.016 | 0.010 | 0.011 | | 0.012 | 0.034 | 0.016 |
| rs6656785 | 1 | 75005776 | A | G | 0.6125 | -0.018 | 0.002 | 3.06E-27 | 0.004 | 0.002 | 1.10E-01 | BMI | -0.004 | 0.010 | -0.003 | | 0.012 | -0.026 | 0.016 |
| rs34517439 | 1 | 78450517 | A | C | 0.1168 | 0.039 | 0.003 | 3.40E-39 | 0.003 | 0.003 | 3.90E-01 | BMI | 0.007 | 0.017 | 0.016 | | 0.020 | 0.015 | 0.026 |
| rs321237 | 1 | 96478125 | A | G | 0.7498 | 0.013 | 0.002 | 1.49E-10 | 0.010 | 0.003 | 1.10E-04 | BMI | -0.004 | 0.011 | 0.004 | | 0.013 | -0.034 | 0.017 |
| rs11165643 | 1 | 96924097 | T | C | 0.5796 | 0.019 | 0.002 | 4.49E-30 | 0.003 | 0.002 | 1.30E-01 | BMI | -0.003 | 0.010 | 0.003 | | 0.012 | -0.016 | 0.015 |
| rs12072739 | 1 | 98315893 | A | G | 0.7762 | -0.017 | 0.002 | 1.50E-13 | 0.002 | 0.003 | 4.50E-01 | BMI | 0.008 | 0.012 | 0.008 | | 0.014 | 0.014 | 0.018 |
| rs12145677 | 1 | 110023610 | G | A | 0.702 | -0.011 | 0.002 | 4.93E-09 | -0.017 | 0.002 | 1.20E-12 | PC | -0.006 | 0.011 | -0.007 | | 0.013 | -0.004 | 0.017 |
| rs7534091 | 1 | 118864616 | A | G | 0.7182 | -0.012 | 0.002 | 6.65E-11 | 0.003 | 0.003 | 2.00E-01 | BMI | 0.031 | 0.011 | 0.030 | | 0.013 | 0.042 | 0.017 |
| rs74887628 | 1 | 147032779 | A | G | 0.0339 | 0.031 | 0.005 | 1.76E-08 | -0.008 | 0.006 | 2.00E-01 | BMI | -0.003 | 0.030 | -0.004 | | 0.036 | -0.014 | 0.047 |
| rs4970991 | 1 | 151004003 | T | C | 0.2151 | 0.012 | 0.002 | 1.81E-08 | 0.007 | 0.003 | 1.20E-02 | BMI | 0.015 | 0.012 | 0.013 | | 0.014 | 0.010 | 0.019 |
| rs61813324 | 1 | 156049877 | T | C | 0.1312 | 0.029 | 0.003 | 3.20E-24 | 0.000 | 0.003 | 9.50E-01 | BMI | 0.047 | 0.017 | 0.045 | | 0.020 | 0.047 | 0.027 |
| rs1750307 | 1 | 156488420 | A | T | 0.3655 | 0.013 | 0.002 | 2.78E-13 | -0.002 | 0.002 | 3.00E-01 | BMI | 0.021 | 0.010 | 0.020 | | 0.012 | 0.025 | 0.016 |
| rs10733051 | 1 | 167280354 | A | G | 0.512 | 0.009 | 0.002 | 6.96E-09 | -0.003 | 0.002 | 1.60E-01 | BMI | 0.027 | 0.010 | 0.029 | | 0.012 | 0.022 | 0.015 |
| rs61828641 | 1 | 174321997 | A | G | 0.1092 | 0.022 | 0.003 | 2.49E-13 | 0.014 | 0.004 | 1.10E-04 | BMI | -0.006 | 0.015 | 0.005 | | 0.018 | -0.011 | 0.024 |
| rs543874 | 1 | 177889480 | A | G | 0.7786 | -0.048 | 0.002 | 3.06E-125 | 0.002 | 0.003 | 4.50E-01 | BMI | -0.003 | 0.012 | 0.002 | | 0.015 | 0.002 | 0.019 |
| rs10920678 | 1 | 190239907 | A | G | 0.425 | 0.015 | 0.002 | 7.15E-20 | 0.001 | 0.002 | 7.70E-01 | BMI | -0.015 | 0.010 | -0.016 | | 0.012 | -0.017 | 0.015 |
| rs2400414 | 1 | 194965200 | T | C | 0.3461 | -0.013 | 0.002 | 5.52E-13 | -0.002 | 0.002 | 3.60E-01 | BMI | -0.028 | 0.010 | -0.028 | | 0.012 | -0.033 | 0.016 |
| rs7526456 | 1 | 197773734 | T | C | 0.413 | 0.007 | 0.002 | 1.71E-04 | -0.013 | 0.002 | 1.20E-08 | PC | -0.005 | 0.010 | -0.004 | | 0.012 | -0.012 | 0.015 |
| rs2820295 | 1 | 201800868 | A | G | 0.3266 | 0.024 | 0.002 | 5.56E-39 | -0.006 | 0.002 | 1.00E-02 | BMI | 0.027 | 0.010 | 0.034 | | 0.012 | 0.005 | 0.016 |
| rs10920336 | 1 | 202115945 | A | G | 0.5327 | -0.010 | 0.002 | 4.20E-09 | -0.001 | 0.002 | 7.80E-01 | BMI | -0.025 | 0.010 | -0.027 | | 0.012 | -0.005 | 0.015 |
| rs1006317 | 1 | 209552636 | T | G | 0.1268 | 0.016 | 0.003 | 3.75E-10 | -0.009 | 0.003 | 6.50E-03 | BMI | -0.034 | 0.014 | -0.021 | | 0.017 | -0.039 | 0.023 |
| rs6661316 | 1 | 210095527 | T | C | 0.5893 | 0.012 | 0.002 | 1.72E-13 | 0.000 | 0.002 | 9.60E-01 | BMI | 0.009 | 0.010 | 0.013 | | 0.012 | 0.013 | 0.015 |
| rs10864728 | 1 | 230304914 | A | G | 0.3978 | 0.011 | 0.002 | 1.20E-08 | 0.000 | 0.002 | 8.40E-01 | BMI | 0.001 | 0.010 | -0.009 | | 0.012 | 0.008 | 0.015 |
| rs946824 | 1 | 243684019 | T | C | 0.1355 | 0.020 | 0.003 | 4.82E-15 | -0.007 | 0.003 | 3.10E-02 | BMI | 0.004 | 0.014 | 0.005 | | 0.017 | 0.022 | 0.022 |
| rs77165542 | 2 | 430975 | T | C | 0.0341 | -0.094 | 0.005 | 1.51E-70 | -0.013 | 0.006 | 3.60E-02 | BMI | -0.058 | 0.030 | -0.060 | | 0.036 | -0.026 | 0.047 |
| rs13021737 | 2 | 632348 | A | G | 0.1567 | -0.058 | 0.002 | 2.89E-161 | -0.009 | 0.003 | 2.80E-03 | BMI | -0.018 | 0.013 | -0.024 | | 0.015 | -0.024 | 0.020 |
| rs10929925 | 2 | 6155557 | A | C | 0.4105 | -0.014 | 0.002 | 3.05E-18 | 0.002 | 0.002 | 4.80E-01 | BMI | -0.010 | 0.010 | -0.012 | | 0.012 | 0.006 | 0.015 |
| rs11902450 | 2 | 12845368 | T | C | 0.1052 | 0.017 | 0.003 | 1.06E-09 | 0.002 | 0.004 | 6.60E-01 | BMI | 0.000 | 0.016 | 0.004 | | 0.019 | 0.007 | 0.025 |
| rs10182181 | 2 | 25150296 | A | G | 0.5052 | -0.033 | 0.002 | 2.45E-91 | -0.004 | 0.002 | 1.10E-01 | BMI | -0.004 | 0.010 | -0.010 | | 0.012 | -0.013 | 0.015 |
| rs935166 | 2 | 26949366 | A | G | 0.4948 | -0.015 | 0.002 | 8.69E-16 | -0.004 | 0.002 | 5.50E-02 | BMI | -0.023 | 0.010 | -0.026 | | 0.012 | -0.027 | 0.015 |
| rs10168563 | 2 | 35552173 | A | G | 0.7031 | 0.013 | 0.002 | 9.33E-12 | -0.005 | 0.002 | 3.30E-02 | BMI | 0.008 | 0.011 | 0.016 | | 0.013 | 0.000 | 0.017 |
| rs3770890 | 2 | 36657992 | T | G | 0.9725 | -0.030 | 0.005 | 2.10E-08 | -0.010 | 0.007 | 1.40E-01 | BMI | 0.026 | 0.035 | -0.007 | | 0.043 | 0.099 | 0.059 |
| rs10185199 | 2 | 40282202 | A | G | 0.286 | -0.014 | 0.002 | 3.18E-13 | -0.002 | 0.003 | 4.50E-01 | BMI | 0.009 | 0.011 | 0.008 | | 0.013 | 0.006 | 0.017 |
| rs10169594 | 2 | 41637688 | T | C | 0.6475 | -0.012 | 0.002 | 1.34E-11 | 0.001 | 0.002 | 6.90E-01 | BMI | -0.006 | 0.010 | -0.006 | | 0.012 | -0.008 | 0.016 |
| rs7561278 | 2 | 48954905 | T | C | 0.7668 | 0.017 | 0.002 | 4.89E-16 | -0.001 | 0.003 | 7.30E-01 | BMI | -0.015 | 0.012 | -0.024 | | 0.014 | -0.032 | 0.019 |
| rs930295 | 2 | 50233352 | A | C | 0.1548 | 0.021 | 0.002 | 2.03E-19 | -0.001 | 0.003 | 7.10E-01 | BMI | 0.000 | 0.013 | 0.007 | | 0.016 | -0.014 | 0.021 |
| rs805412 | 2 | 54120820 | A | G | 0.4337 | -0.010 | 0.002 | 1.14E-08 | 0.000 | 0.002 | 9.90E-01 | BMI | 0.014 | 0.010 | 0.009 | | 0.012 | 0.026 | 0.015 |
| rs4671328 | 2 | 58935282 | T | G | 0.4496 | 0.021 | 0.002 | 3.42E-36 | 0.002 | 0.002 | 4.70E-01 | BMI | -0.007 | 0.010 | -0.007 | | 0.012 | 0.013 | 0.015 |
| rs6545714 | 2 | 59307725 | A | G | 0.6112 | -0.019 | 0.002 | 4.01E-32 | 0.001 | 0.002 | 7.30E-01 | BMI | -0.013 | 0.010 | -0.006 | | 0.012 | -0.036 | 0.015 |
| rs2861685 | 2 | 67837553 | T | C | 0.5864 | 0.017 | 0.002 | 7.82E-18 | 0.003 | 0.002 | 1.40E-01 | BMI | -0.010 | 0.010 | -0.016 | | 0.012 | -0.031 | 0.015 |
| rs17020497 | 2 | 81826131 | A | G | 0.1341 | 0.014 | 0.003 | 2.04E-08 | -0.001 | 0.003 | 6.90E-01 | BMI | 0.008 | 0.014 | 0.018 | | 0.017 | 0.014 | 0.022 |
| rs12714199 | 2 | 86812549 | T | C | 0.6146 | -0.014 | 0.002 | 3.22E-16 | -0.001 | 0.002 | 7.90E-01 | BMI | 0.000 | 0.010 | 0.009 | | 0.012 | -0.003 | 0.016 |
| rs4303732 | 2 | 100830040 | T | C | 0.6137 | 0.017 | 0.002 | 1.26E-22 | -0.006 | 0.002 | 4.20E-03 | BMI | 0.024 | 0.010 | 0.019 | | 0.012 | 0.033 | 0.016 |
| rs264941 | 2 | 104297420 | A | C | 0.4679 | -0.012 | 0.002 | 1.84E-13 | 0.004 | 0.002 | 8.90E-02 | BMI | -0.004 | 0.010 | -0.010 | | 0.012 | -0.018 | 0.015 |
| rs10197031 | 2 | 105454590 | T | C | 0.7262 | -0.016 | 0.002 | 5.05E-18 | 0.007 | 0.002 | 3.60E-03 | BMI | 0.012 | 0.011 | 0.010 | | 0.013 | 0.008 | 0.017 |
| rs13033310 | 2 | 133523605 | A | G | 0.2519 | 0.015 | 0.002 | 3.40E-11 | 0.000 | 0.003 | 9.60E-01 | BMI | 0.001 | 0.011 | 0.005 | | 0.013 | -0.002 | 0.017 |
| rs4988235 | 2 | 136608646 | A | G | 0.7206 | 0.012 | 0.002 | 7.09E-13 | 0.002 | 0.003 | 5.30E-01 | BMI | -0.043 | 0.011 | -0.049 | | 0.013 | -0.054 | 0.017 |
| rs17814208 | 2 | 144037998 | A | G | 0.7575 | -0.013 | 0.002 | 7.24E-11 | -0.003 | 0.003 | 2.20E-01 | BMI | -0.003 | 0.011 | 0.003 | | 0.013 | -0.006 | 0.017 |
| rs10754920 | 2 | 146456888 | C | G | 0.112 | 0.007 | 0.003 | 1.13E-02 | 0.020 | 0.003 | 6.70E-09 | PC | 0.002 | 0.014 | 0.007 | | 0.018 | 0.011 | 0.023 |
| rs2119753 | 2 | 151224579 | A | G | 0.6229 | 0.010 | 0.002 | 1.00E-08 | -0.001 | 0.002 | 8.00E-01 | BMI | 0.001 | 0.010 | 0.000 | | 0.012 | 0.008 | 0.016 |
| rs6744254 | 2 | 155986195 | C | T | 0.471 | -0.006 | 0.002 | 8.39E-04 | -0.016 | 0.002 | 5.40E-13 | PC | 0.014 | 0.010 | 0.020 | | 0.012 | 0.021 | 0.015 |
| rs3764835 | 2 | 159519368 | A | G | 0.1505 | -0.013 | 0.002 | 3.84E-08 | -0.008 | 0.003 | 5.70E-03 | BMI | -0.003 | 0.013 | 0.003 | | 0.016 | 0.000 | 0.021 |
| rs62176243 | 2 | 166190881 | A | T | 0.7571 | 0.015 | 0.002 | 2.41E-11 | 0.001 | 0.003 | 7.40E-01 | BMI | 0.012 | 0.011 | 0.012 | | 0.014 | 0.023 | 0.018 |
| rs56133507 | 2 | 172818467 | T | G | 0.8065 | -0.013 | 0.002 | 4.31E-08 | 0.004 | 0.003 | 1.10E-01 | BMI | 0.020 | 0.012 | 0.024 | | 0.015 | 0.015 | 0.019 |
| rs34234296 | 2 | 175166636 | A | G | 0.3876 | -0.015 | 0.002 | 2.01E-13 | -0.005 | 0.002 | 1.90E-02 | BMI | -0.011 | 0.010 | 0.003 | | 0.012 | -0.032 | 0.016 |
| rs7588437 | 2 | 181575281 | A | G | 0.3664 | -0.017 | 0.002 | 2.32E-22 | 0.002 | 0.002 | 3.50E-01 | BMI | -0.002 | 0.010 | -0.009 | | 0.012 | 0.008 | 0.016 |
| rs6716898 | 2 | 198944271 | A | G | 0.4873 | 0.013 | 0.002 | 2.33E-11 | -0.008 | 0.002 | 2.70E-04 | BMI | 0.003 | 0.010 | 0.007 | | 0.012 | 0.002 | 0.015 |
| rs7593917 | 2 | 203931012 | A | G | 0.4535 | -0.012 | 0.002 | 9.67E-13 | -0.003 | 0.002 | 1.50E-01 | BMI | -0.003 | 0.010 | -0.003 | | 0.012 | 0.005 | 0.015 |
| rs4482463 | 2 | 205375909 | A | C | 0.917 | -0.031 | 0.003 | 4.85E-23 | -0.005 | 0.004 | 2.60E-01 | BMI | -0.001 | 0.017 | -0.006 | | 0.021 | 0.018 | 0.028 |
| rs11274218 | 2 | 215035001 | C | T | 0.611 | 0.004 | 0.002 | 1.46E-02 | -0.014 | 0.002 | 2.50E-09 | PC | -0.007 | 0.010 | -0.009 | | 0.012 | 0.001 | 0.015 |
| rs12987009 | 2 | 219658170 | A | T | 0.5851 | -0.011 | 0.002 | 1.83E-10 | 0.001 | 0.002 | 5.60E-01 | BMI | 0.006 | 0.010 | 0.006 | | 0.012 | 0.010 | 0.015 |
| rs10208088 | 2 | 221055873 | C | T | 0.419 | 0.002 | 0.002 | 3.66E-01 | 0.013 | 0.002 | 4.40E-09 | PC | 0.002 | 0.010 | 0.003 | | 0.012 | -0.019 | 0.015 |
| rs4973618 | 2 | 229002620 | A | G | 0.6623 | -0.015 | 0.002 | 1.25E-16 | 0.002 | 0.002 | 2.90E-01 | BMI | -0.005 | 0.010 | -0.001 | | 0.012 | -0.013 | 0.016 |
| rs6720868 | 2 | 230663576 | T | C | 0.3083 | 0.015 | 0.002 | 1.82E-17 | 0.000 | 0.002 | 9.40E-01 | BMI | -0.003 | 0.010 | -0.005 | | 0.012 | 0.003 | 0.016 |
| rs7568228 | 2 | 236848488 | C | G | 0.5401 | -0.010 | 0.002 | 1.00E-09 | 0.001 | 0.002 | 7.30E-01 | BMI | -0.018 | 0.010 | -0.026 | | 0.012 | 0.006 | 0.015 |
| rs6783054 | 3 | 11672805 | A | C | 0.5028 | 0.010 | 0.002 | 6.22E-09 | 0.001 | 0.002 | 5.00E-01 | BMI | 0.008 | 0.010 | 0.002 | | 0.012 | 0.017 | 0.015 |
| rs10510419 | 3 | 12426936 | T | G | 0.1467 | -0.017 | 0.002 | 2.23E-13 | 0.000 | 0.003 | 8.90E-01 | BMI | 0.037 | 0.014 | 0.034 | | 0.016 | 0.047 | 0.021 |
| rs2600226 | 3 | 12928762 | T | C | 0.6712 | -0.012 | 0.002 | 1.42E-10 | 0.001 | 0.002 | 6.80E-01 | BMI | 0.016 | 0.010 | 0.003 | | 0.012 | 0.016 | 0.016 |
| rs1048637 | 3 | 13358171 | T | G | 0.5474 | -0.009 | 0.002 | 2.94E-08 | -0.004 | 0.002 | 4.50E-02 | BMI | 0.018 | 0.010 | 0.014 | | 0.012 | 0.023 | 0.015 |
| rs4858193 | 3 | 20441050 | T | C | 0.7199 | 0.013 | 0.002 | 2.44E-12 | -0.001 | 0.002 | 6.80E-01 | BMI | 0.002 | 0.011 | -0.011 | | 0.013 | -0.006 | 0.017 |
| rs6804842 | 3 | 25106437 | A | G | 0.4301 | -0.014 | 0.002 | 7.57E-18 | 0.001 | 0.002 | 5.80E-01 | BMI | 0.008 | 0.010 | 0.011 | | 0.012 | -0.007 | 0.015 |
| rs11921432 | 3 | 35117776 | T | C | 0.8906 | -0.019 | 0.003 | 5.38E-12 | 0.003 | 0.003 | 3.20E-01 | BMI | -0.008 | 0.016 | -0.018 | | 0.018 | 0.006 | 0.024 |
| rs4017425 | 3 | 44028764 | T | C | 0.4676 | -0.012 | 0.002 | 2.91E-12 | -0.001 | 0.002 | 6.20E-01 | BMI | 0.015 | 0.010 | 0.021 | | 0.012 | 0.014 | 0.015 |
| rs11708955 | 3 | 49540114 | T | C | 0.695 | 0.015 | 0.002 | 3.34E-15 | -0.016 | 0.002 | 4.20E-11 | PC | -0.001 | 0.010 | -0.007 | | 0.012 | 0.029 | 0.016 |
| rs6445258 | 3 | 62112198 | T | C | 0.207 | 0.013 | 0.002 | 2.48E-08 | 0.004 | 0.003 | 1.60E-01 | BMI | 0.002 | 0.013 | 0.010 | | 0.015 | -0.017 | 0.020 |
| rs6774533 | 3 | 62471086 | C | T | 0.294 | -0.012 | 0.002 | 5.87E-09 | -0.015 | 0.002 | 1.20E-09 | PC | -0.004 | 0.011 | -0.009 | | 0.013 | 0.009 | 0.017 |
| rs12636480 | 3 | 82719412 | T | G | 0.3507 | 0.013 | 0.002 | 6.96E-13 | -0.003 | 0.002 | 1.80E-01 | BMI | 0.005 | 0.010 | 0.000 | | 0.012 | 0.003 | 0.016 |
| rs9827823 | 3 | 84221774 | T | C | 0.8552 | 0.018 | 0.002 | 3.39E-15 | -0.005 | 0.003 | 8.60E-02 | BMI | -0.004 | 0.014 | -0.016 | | 0.016 | 0.003 | 0.021 |
| rs9818122 | 3 | 85861064 | T | C | 0.7933 | -0.023 | 0.002 | 3.97E-30 | -0.006 | 0.003 | 2.70E-02 | BMI | 0.021 | 0.012 | 0.026 | | 0.014 | 0.013 | 0.018 |
| rs34184235 | 3 | 86192846 | T | C | 0.4353 | -0.012 | 0.002 | 2.14E-09 | -0.001 | 0.002 | 6.10E-01 | BMI | 0.009 | 0.010 | 0.009 | | 0.012 | 0.016 | 0.015 |
| rs11128021 | 3 | 88139016 | A | G | 0.1615 | -0.018 | 0.002 | 1.63E-14 | -0.004 | 0.003 | 1.40E-01 | BMI | -0.011 | 0.014 | -0.005 | | 0.017 | -0.026 | 0.022 |
| rs1492014 | 3 | 94071481 | T | C | 0.5674 | -0.017 | 0.002 | 1.44E-23 | 0.002 | 0.002 | 2.80E-01 | BMI | 0.010 | 0.010 | 0.012 | | 0.012 | 0.010 | 0.015 |
| rs1436344 | 3 | 104606144 | C | G | 0.5703 | 0.015 | 0.002 | 1.07E-17 | -0.004 | 0.002 | 7.60E-02 | BMI | 0.011 | 0.010 | 0.016 | | 0.012 | -0.004 | 0.015 |
| rs78082503 | 3 | 116556913 | G | C | 0.786 | -0.001 | 0.002 | 6.07E-01 | -0.015 | 0.003 | 1.10E-08 | PC | -0.007 | 0.011 | -0.002 | | 0.014 | 0.033 | 0.018 |
| rs7631156 | 3 | 131751628 | A | G | 0.3091 | 0.022 | 0.002 | 3.33E-32 | -0.002 | 0.002 | 4.80E-01 | BMI | 0.001 | 0.010 | -0.010 | | 0.012 | 0.032 | 0.016 |
| rs687339 | 3 | 135932359 | T | C | 0.7697 | 0.019 | 0.002 | 4.32E-22 | 0.006 | 0.003 | 1.60E-02 | BMI | 0.014 | 0.011 | 0.015 | | 0.014 | 0.007 | 0.018 |
| rs16851483 | 3 | 141275436 | T | G | 0.0717 | 0.035 | 0.003 | 4.87E-25 | 0.006 | 0.004 | 1.80E-01 | BMI | -0.003 | 0.018 | 0.005 | | 0.023 | -0.001 | 0.031 |
| rs355777 | 3 | 154034950 | C | G | 0.3984 | 0.015 | 0.002 | 2.13E-18 | -0.004 | 0.002 | 1.20E-01 | BMI | 0.001 | 0.010 | -0.005 | | 0.012 | 0.012 | 0.015 |
| rs9826775 | 3 | 156295341 | A | G | 0.8513 | 0.016 | 0.002 | 6.61E-11 | -0.001 | 0.003 | 8.70E-01 | BMI | 0.013 | 0.014 | 0.020 | | 0.016 | 0.018 | 0.021 |
| rs2047648 | 3 | 157033438 | A | T | 0.7465 | -0.013 | 0.002 | 7.95E-12 | -0.004 | 0.003 | 1.00E-01 | BMI | -0.005 | 0.011 | -0.013 | | 0.013 | 0.010 | 0.017 |
| rs3732927 | 3 | 170586057 | T | C | 0.2907 | 0.010 | 0.002 | 1.13E-08 | -0.002 | 0.002 | 3.60E-01 | BMI | -0.008 | 0.010 | -0.006 | | 0.012 | 0.015 | 0.016 |
| rs12635553 | 3 | 171113714 | A | T | 0.486 | 0.010 | 0.002 | 4.10E-09 | 0.003 | 0.002 | 2.20E-01 | BMI | -0.005 | 0.010 | -0.012 | | 0.012 | 0.016 | 0.015 |
| rs39654 | 3 | 173095123 | A | G | 0.4513 | -0.016 | 0.002 | 1.75E-21 | 0.006 | 0.002 | 1.20E-02 | BMI | 0.001 | 0.010 | 0.002 | | 0.012 | -0.012 | 0.015 |
| rs6443750 | 3 | 181329682 | T | C | 0.1994 | -0.015 | 0.002 | 7.25E-13 | -0.001 | 0.003 | 6.50E-01 | BMI | -0.010 | 0.014 | -0.026 | | 0.016 | 0.004 | 0.021 |
| rs865809 | 3 | 183997735 | A | G | 0.2246 | 0.012 | 0.002 | 6.50E-10 | 0.006 | 0.003 | 1.50E-02 | BMI | 0.009 | 0.012 | 0.009 | | 0.014 | -0.005 | 0.018 |
| rs9816226 | 3 | 185834499 | A | T | 0.1755 | -0.032 | 0.002 | 1.45E-50 | 0.002 | 0.003 | 4.90E-01 | BMI | 0.020 | 0.013 | 0.020 | | 0.015 | 0.001 | 0.020 |
| rs7616009 | 3 | 194881756 | A | G | 0.1595 | -0.016 | 0.002 | 4.33E-11 | 0.000 | 0.003 | 9.80E-01 | BMI | 0.000 | 0.013 | 0.003 | | 0.017 | -0.019 | 0.022 |
| rs6803161 | 3 | 196205694 | T | C | 0.3945 | 0.011 | 0.002 | 2.71E-08 | 0.001 | 0.002 | 8.00E-01 | BMI | 0.003 | 0.010 | 0.012 | | 0.012 | -0.005 | 0.016 |
| rs2051559 | 4 | 3298800 | T | C | 0.8588 | -0.017 | 0.003 | 3.78E-11 | 0.009 | 0.003 | 8.30E-03 | BMI | -0.017 | 0.014 | -0.010 | | 0.017 | -0.059 | 0.022 |
| rs12642970 | 4 | 16601492 | C | G | 0.44 | -0.011 | 0.002 | 1.71E-08 | -0.005 | 0.002 | 1.60E-02 | BMI | 0.007 | 0.010 | 0.010 | | 0.012 | -0.017 | 0.015 |
| rs994596 | 4 | 18459828 | T | C | 0.3247 | 0.013 | 0.002 | 1.06E-12 | 0.002 | 0.002 | 4.40E-01 | BMI | 0.006 | 0.010 | 0.007 | | 0.012 | 0.014 | 0.016 |
| rs34811474 | 4 | 25408838 | A | G | 0.2224 | -0.029 | 0.002 | 8.50E-38 | 0.003 | 0.003 | 2.80E-01 | BMI | -0.022 | 0.013 | -0.038 | | 0.016 | -0.016 | 0.021 |
| rs73213484 | 4 | 28489339 | A | T | 0.8565 | 0.021 | 0.003 | 2.04E-14 | 0.003 | 0.003 | 3.20E-01 | BMI | 0.008 | 0.013 | 0.014 | | 0.016 | 0.036 | 0.021 |
| rs337637 | 4 | 38604470 | A | G | 0.359 | -0.014 | 0.002 | 6.24E-16 | 0.005 | 0.002 | 1.90E-02 | BMI | -0.008 | 0.010 | -0.009 | | 0.012 | -0.013 | 0.016 |
| rs6857629 | 4 | 39684977 | G | A | 0.755 | -0.004 | 0.002 | 8.48E-02 | 0.015 | 0.003 | 1.90E-09 | PC | 0.021 | 0.011 | 0.014 | | 0.013 | 0.026 | 0.017 |
| rs2271046 | 4 | 52752812 | A | T | 0.6948 | -0.012 | 0.002 | 3.03E-10 | 0.004 | 0.002 | 6.50E-02 | BMI | -0.011 | 0.011 | -0.003 | | 0.013 | -0.030 | 0.017 |
| rs1492767 | 4 | 55221467 | T | C | 0.4681 | 0.010 | 0.002 | 3.55E-09 | 0.002 | 0.002 | 4.00E-01 | BMI | 0.000 | 0.010 | -0.011 | | 0.012 | 0.022 | 0.015 |
| rs2192158 | 4 | 55505360 | A | G | 0.4492 | 0.014 | 0.002 | 7.42E-16 | 0.003 | 0.002 | 1.70E-01 | BMI | -0.029 | 0.010 | -0.005 | | 0.012 | -0.064 | 0.015 |
| rs1346841 | 4 | 65651730 | A | G | 0.4116 | -0.013 | 0.002 | 3.18E-13 | -0.001 | 0.002 | 5.50E-01 | BMI | -0.004 | 0.010 | 0.000 | | 0.012 | 0.020 | 0.015 |
| rs10002111 | 4 | 67815504 | A | G | 0.2227 | 0.013 | 0.002 | 1.26E-09 | -0.001 | 0.003 | 7.50E-01 | BMI | 0.000 | 0.012 | 0.006 | | 0.014 | -0.007 | 0.018 |
| rs10033843 | 4 | 77028783 | A | G | 0.215 | 0.014 | 0.002 | 1.07E-11 | 0.001 | 0.003 | 7.30E-01 | BMI | -0.001 | 0.012 | -0.007 | | 0.014 | 0.008 | 0.018 |
| rs72649373 | 4 | 80609966 | T | C | 0.8619 | -0.017 | 0.003 | 3.20E-09 | 0.005 | 0.003 | 1.10E-01 | BMI | 0.005 | 0.014 | -0.009 | | 0.017 | 0.033 | 0.022 |
| rs4148155 | 4 | 89054667 | A | G | 0.8867 | 0.019 | 0.003 | 1.34E-13 | -0.009 | 0.003 | 6.90E-03 | BMI | 0.004 | 0.015 | 0.014 | | 0.019 | 0.018 | 0.025 |
| rs4286488 | 4 | 94440026 | A | G | 0.7608 | 0.012 | 0.002 | 1.70E-09 | -0.007 | 0.003 | 1.00E-02 | BMI | 0.018 | 0.011 | 0.022 | | 0.014 | 0.036 | 0.018 |
| rs7678054 | 4 | 95093855 | A | G | 0.4788 | -0.010 | 0.002 | 4.57E-09 | 0.004 | 0.002 | 7.90E-02 | BMI | -0.025 | 0.010 | -0.025 | | 0.012 | -0.028 | 0.015 |
| rs3796432 | 4 | 96030402 | T | G | 0.3672 | -0.011 | 0.002 | 2.21E-10 | 0.002 | 0.002 | 4.30E-01 | BMI | 0.000 | 0.010 | 0.002 | | 0.012 | -0.011 | 0.016 |
| rs13107325 | 4 | 103188709 | T | C | 0.082 | 0.047 | 0.003 | 3.81E-47 | 0.001 | 0.004 | 7.60E-01 | BMI | -0.008 | 0.019 | -0.012 | | 0.022 | 0.006 | 0.029 |
| rs326893 | 4 | 112691776 | T | C | 0.5812 | 0.012 | 0.002 | 1.89E-12 | -0.001 | 0.002 | 6.40E-01 | BMI | -0.010 | 0.010 | -0.006 | | 0.012 | -0.015 | 0.015 |
| rs7696649 | 4 | 120322177 | A | G | 0.278 | 0.012 | 0.002 | 4.73E-10 | 0.000 | 0.002 | 8.50E-01 | BMI | 0.036 | 0.011 | 0.026 | | 0.013 | 0.056 | 0.017 |
| rs4864201 | 4 | 130731284 | T | C | 0.3471 | 0.014 | 0.002 | 4.30E-16 | 0.000 | 0.002 | 9.30E-01 | BMI | -0.001 | 0.010 | -0.002 | | 0.012 | -0.002 | 0.016 |
| rs1296328 | 4 | 137083193 | A | C | 0.4464 | 0.017 | 0.002 | 3.49E-22 | -0.002 | 0.002 | 2.90E-01 | BMI | -0.012 | 0.010 | -0.001 | | 0.012 | -0.025 | 0.015 |
| rs17367750 | 4 | 140782542 | T | C | 0.3117 | -0.012 | 0.002 | 1.97E-11 | -0.006 | 0.002 | 1.50E-02 | BMI | -0.013 | 0.010 | -0.018 | | 0.012 | -0.019 | 0.016 |
| rs9992189 | 4 | 144060728 | C | G | 0.6083 | -0.010 | 0.002 | 2.04E-08 | 0.000 | 0.002 | 9.50E-01 | BMI | 0.010 | 0.010 | 0.004 | | 0.012 | 0.042 | 0.015 |
| rs3914628 | 4 | 147438019 | T | C | 0.8593 | 0.017 | 0.002 | 6.91E-13 | 0.005 | 0.003 | 9.10E-02 | BMI | -0.010 | 0.013 | -0.002 | | 0.016 | -0.006 | 0.021 |
| rs2068625 | 4 | 159856739 | T | C | 0.302 | 0.007 | 0.002 | 1.39E-04 | -0.016 | 0.002 | 4.00E-11 | PC | 0.017 | 0.011 | 0.025 | | 0.013 | 0.020 | 0.017 |
| rs13110266 | 4 | 162129844 | A | G | 0.4037 | -0.012 | 0.002 | 3.96E-14 | -0.001 | 0.002 | 7.30E-01 | BMI | -0.018 | 0.010 | -0.022 | | 0.012 | -0.013 | 0.016 |
| rs7685628 | 4 | 165310133 | A | T | 0.4014 | 0.010 | 0.002 | 6.15E-09 | 0.004 | 0.002 | 9.30E-02 | BMI | 0.027 | 0.010 | 0.021 | | 0.012 | 0.042 | 0.015 |
| rs1522569 | 4 | 171632637 | T | G | 0.8183 | 0.014 | 0.002 | 1.60E-10 | 0.002 | 0.003 | 5.80E-01 | BMI | 0.000 | 0.012 | 0.000 | | 0.015 | -0.001 | 0.019 |
| rs1437842 | 4 | 173597016 | A | G | 0.4907 | -0.011 | 0.002 | 8.49E-10 | 0.002 | 0.002 | 4.10E-01 | BMI | -0.011 | 0.010 | -0.020 | | 0.012 | -0.008 | 0.015 |
| rs6850421 | 4 | 180187034 | A | G | 0.4564 | 0.011 | 0.002 | 3.66E-09 | 0.007 | 0.002 | 2.60E-03 | BMI | 0.002 | 0.010 | 0.002 | | 0.012 | -0.020 | 0.015 |
| rs35408866 | 4 | 187743245 | A | G | 0.1339 | 0.016 | 0.003 | 1.50E-08 | 0.000 | 0.003 | 9.10E-01 | BMI | 0.004 | 0.015 | 0.015 | | 0.017 | -0.003 | 0.023 |
| rs698147 | 5 | 3513485 | A | G | 0.447 | 0.012 | 0.002 | 9.67E-12 | 0.005 | 0.002 | 2.60E-02 | BMI | -0.003 | 0.010 | -0.011 | | 0.012 | 0.001 | 0.015 |
| rs2220599 | 5 | 7378854 | C | G | 0.633 | 0.004 | 0.002 | 1.27E-02 | -0.016 | 0.002 | 2.40E-12 | PC | -0.003 | 0.010 | 0.001 | | 0.012 | -0.002 | 0.016 |
| rs6890310 | 5 | 27193573 | A | G | 0.2923 | -0.012 | 0.002 | 3.29E-10 | 0.001 | 0.002 | 7.10E-01 | BMI | 0.019 | 0.011 | 0.027 | | 0.013 | 0.012 | 0.017 |
| rs7730004 | 5 | 43191033 | T | C | 0.6614 | 0.014 | 0.002 | 1.46E-14 | -0.002 | 0.002 | 5.10E-01 | BMI | 0.024 | 0.010 | 0.021 | | 0.012 | 0.043 | 0.016 |
| rs116374395 | 5 | 50723410 | A | G | 0.0348 | 0.032 | 0.005 | 7.05E-10 | 0.002 | 0.006 | 7.50E-01 | BMI | -0.014 | 0.024 | -0.013 | | 0.029 | 0.027 | 0.037 |
| rs150215901 | 5 | 50935903 | A | T | 0.0414 | -0.028 | 0.005 | 1.54E-08 | 0.002 | 0.005 | 6.60E-01 | BMI | 0.013 | 0.026 | 0.004 | | 0.031 | 0.035 | 0.040 |
| rs4865796 | 5 | 53272664 | A | G | 0.7044 | -0.010 | 0.002 | 4.21E-08 | -0.001 | 0.002 | 8.10E-01 | BMI | 0.012 | 0.010 | 0.007 | | 0.013 | 0.017 | 0.016 |
| rs13186194 | 5 | 60795485 | T | C | 0.6169 | 0.010 | 0.002 | 2.20E-09 | 0.003 | 0.002 | 2.00E-01 | BMI | 0.006 | 0.010 | 0.011 | | 0.012 | 0.007 | 0.016 |
| rs4700608 | 5 | 63026280 | T | C | 0.5236 | -0.016 | 0.002 | 4.32E-20 | -0.002 | 0.002 | 3.90E-01 | BMI | 0.025 | 0.010 | 0.021 | | 0.012 | 0.018 | 0.015 |
| rs10050620 | 5 | 63927239 | T | C | 0.3308 | -0.013 | 0.002 | 1.20E-10 | 0.003 | 0.002 | 2.80E-01 | BMI | -0.004 | 0.010 | -0.005 | | 0.012 | -0.012 | 0.016 |
| rs1159692 | 5 | 63977815 | A | C | 0.4752 | 0.014 | 0.002 | 5.54E-15 | 0.003 | 0.002 | 1.20E-01 | BMI | 0.005 | 0.010 | 0.012 | | 0.012 | -0.021 | 0.015 |
| rs2112347 | 5 | 75015242 | T | G | 0.6298 | 0.028 | 0.002 | 1.17E-61 | -0.005 | 0.002 | 4.70E-02 | BMI | 0.010 | 0.010 | 0.005 | | 0.012 | -0.002 | 0.016 |
| rs2962334 | 5 | 86879056 | T | G | 0.0272 | 0.040 | 0.006 | 1.78E-11 | 0.011 | 0.008 | 1.80E-01 | BMI | 0.007 | 0.032 | 0.009 | | 0.037 | -0.077 | 0.050 |
| rs11749912 | 5 | 88065628 | A | G | 0.423 | 0.008 | 0.002 | 2.34E-05 | 0.014 | 0.002 | 4.90E-10 | PC | 0.015 | 0.010 | 0.019 | | 0.012 | -0.001 | 0.015 |
| rs12652212 | 5 | 88808594 | A | G | 0.5611 | -0.013 | 0.002 | 1.63E-15 | 0.003 | 0.002 | 1.40E-01 | BMI | -0.019 | 0.010 | -0.007 | | 0.012 | -0.022 | 0.015 |
| rs11739877 | 5 | 105876806 | T | C | 0.6231 | 0.012 | 0.002 | 4.01E-11 | 0.001 | 0.002 | 6.70E-01 | BMI | 0.003 | 0.010 | 0.000 | | 0.012 | 0.006 | 0.015 |
| rs6888194 | 5 | 106910657 | T | C | 0.8443 | -0.013 | 0.002 | 3.58E-08 | -0.003 | 0.003 | 3.80E-01 | BMI | 0.017 | 0.014 | 0.004 | | 0.016 | 0.038 | 0.021 |
| rs40067 | 5 | 107439012 | A | G | 0.1701 | -0.025 | 0.002 | 9.73E-29 | -0.002 | 0.003 | 4.00E-01 | BMI | -0.008 | 0.013 | -0.008 | | 0.015 | -0.014 | 0.020 |
| rs459552 | 5 | 112176756 | A | T | 0.7747 | -0.013 | 0.002 | 8.36E-12 | 0.003 | 0.003 | 2.40E-01 | BMI | 0.004 | 0.012 | -0.002 | | 0.014 | 0.010 | 0.018 |
| rs1469249 | 5 | 113837198 | G | A | 0.789 | -0.010 | 0.002 | 1.08E-06 | 0.016 | 0.003 | 9.50E-09 | PC | 0.009 | 0.012 | -0.001 | | 0.014 | 0.038 | 0.018 |
| rs162894 | 5 | 131611872 | T | G | 0.332 | 0.004 | 0.002 | 3.71E-02 | 0.013 | 0.002 | 1.10E-08 | PC | -0.012 | 0.010 | -0.018 | | 0.012 | -0.012 | 0.016 |
| rs56158102 | 5 | 144027803 | C | T | 0.344 | -0.001 | 0.002 | 4.22E-01 | 0.013 | 0.002 | 3.10E-08 | PC | -0.006 | 0.010 | -0.001 | | 0.012 | -0.001 | 0.016 |
| rs2910026 | 5 | 152529936 | T | C | 0.7222 | -0.013 | 0.002 | 5.69E-10 | 0.004 | 0.002 | 1.20E-01 | BMI | 0.012 | 0.011 | 0.016 | | 0.013 | 0.000 | 0.017 |
| rs7715256 | 5 | 153537893 | T | G | 0.5644 | -0.016 | 0.002 | 3.98E-22 | 0.004 | 0.002 | 1.10E-01 | BMI | -0.011 | 0.010 | -0.017 | | 0.012 | 0.011 | 0.015 |
| rs7734385 | 5 | 158460212 | A | G | 0.4449 | -0.010 | 0.002 | 6.08E-10 | 0.002 | 0.002 | 4.90E-01 | BMI | -0.025 | 0.010 | -0.021 | | 0.012 | -0.041 | 0.015 |
| rs4921301 | 5 | 159984492 | T | C | 0.2127 | -0.013 | 0.002 | 4.03E-08 | 0.001 | 0.003 | 7.50E-01 | BMI | -0.010 | 0.012 | -0.019 | | 0.014 | 0.001 | 0.019 |
| rs2861089 | 5 | 164557954 | A | T | 0.3806 | 0.011 | 0.002 | 1.55E-09 | 0.004 | 0.002 | 1.00E-01 | BMI | 0.015 | 0.010 | 0.011 | | 0.012 | 0.019 | 0.015 |
| rs7727781 | 5 | 165185571 | T | C | 0.5164 | 0.009 | 0.002 | 4.27E-08 | 0.004 | 0.002 | 6.70E-02 | BMI | -0.006 | 0.010 | -0.012 | | 0.012 | -0.014 | 0.015 |
| rs2053682 | 5 | 170599327 | A | C | 0.6784 | 0.017 | 0.002 | 2.59E-20 | 0.004 | 0.002 | 6.30E-02 | BMI | -0.005 | 0.010 | -0.008 | | 0.012 | 0.009 | 0.016 |
| rs6556301 | 5 | 176527577 | T | G | 0.3707 | -0.011 | 0.002 | 8.14E-11 | -0.001 | 0.002 | 7.70E-01 | BMI | -0.012 | 0.010 | -0.007 | | 0.012 | 0.004 | 0.016 |
| rs2228213 | 6 | 12124855 | A | G | 0.3374 | -0.014 | 0.002 | 5.50E-17 | -0.003 | 0.002 | 2.70E-01 | BMI | -0.007 | 0.010 | -0.010 | | 0.012 | 0.001 | 0.016 |
| rs11757278 | 6 | 13180454 | T | C | 0.6961 | 0.013 | 0.002 | 6.92E-13 | 0.003 | 0.002 | 1.80E-01 | BMI | 0.006 | 0.010 | 0.005 | | 0.013 | 0.016 | 0.016 |
| rs9477970 | 6 | 19082663 | T | A | 0.808 | -0.010 | 0.002 | 2.41E-06 | -0.016 | 0.003 | 1.00E-08 | PC | -0.017 | 0.012 | -0.016 | | 0.015 | -0.005 | 0.020 |
| rs2066295 | 6 | 26168903 | A | G | 0.7577 | 0.014 | 0.002 | 2.29E-12 | -0.009 | 0.003 | 9.80E-04 | BMI | 0.018 | 0.011 | 0.030 | | 0.013 | 0.034 | 0.018 |
| rs3115667 | 6 | 31643399 | T | C | 0.2821 | -0.018 | 0.002 | 5.92E-21 | 0.004 | 0.002 | 6.60E-02 | BMI | -0.044 | 0.012 | -0.055 | | 0.014 | -0.050 | 0.018 |
| rs2281819 | 6 | 33771673 | A | T | 0.2293 | -0.015 | 0.002 | 2.09E-14 | -0.003 | 0.003 | 2.80E-01 | BMI | -0.009 | 0.011 | -0.002 | | 0.014 | -0.027 | 0.018 |
| rs2744974 | 6 | 34579431 | T | C | 0.3239 | 0.026 | 0.002 | 1.28E-51 | 0.005 | 0.002 | 3.00E-02 | BMI | -0.010 | 0.010 | -0.005 | | 0.012 | -0.043 | 0.016 |
| rs2436728 | 6 | 40365601 | A | G | 0.4073 | 0.019 | 0.002 | 1.97E-29 | -0.004 | 0.002 | 9.30E-02 | BMI | 0.010 | 0.010 | 0.009 | | 0.012 | 0.019 | 0.015 |
| rs1358980 | 6 | 43764551 | T | C | 0.4735 | -0.013 | 0.002 | 5.15E-15 | -0.004 | 0.002 | 1.10E-01 | BMI | 0.003 | 0.010 | 0.006 | | 0.012 | 0.006 | 0.015 |
| rs2206277 | 6 | 50798526 | T | C | 0.1557 | 0.041 | 0.002 | 1.82E-83 | 0.002 | 0.003 | 4.90E-01 | BMI | -0.010 | 0.012 | -0.007 | | 0.015 | -0.026 | 0.020 |
| rs1327259 | 6 | 51177811 | A | G | 0.6096 | 0.016 | 0.002 | 1.47E-19 | -0.001 | 0.002 | 7.40E-01 | BMI | 0.012 | 0.010 | 0.013 | | 0.012 | 0.004 | 0.016 |
| rs6915002 | 6 | 54028069 | T | C | 0.4113 | 0.010 | 0.002 | 8.71E-09 | 0.009 | 0.002 | 6.00E-05 | BMI | 0.004 | 0.010 | 0.007 | | 0.012 | 0.001 | 0.015 |
| rs9370410 | 6 | 55171842 | A | G | 0.7293 | 0.011 | 0.002 | 2.58E-08 | -0.002 | 0.002 | 3.50E-01 | BMI | 0.007 | 0.011 | 0.017 | | 0.013 | -0.008 | 0.017 |
| rs2622274 | 6 | 64240516 | T | G | 0.4553 | -0.011 | 0.002 | 3.23E-10 | -0.003 | 0.002 | 1.80E-01 | BMI | -0.005 | 0.010 | -0.002 | | 0.012 | 0.001 | 0.015 |
| rs1293037 | 6 | 70248345 | T | C | 0.7544 | 0.013 | 0.002 | 5.73E-09 | -0.004 | 0.003 | 9.70E-02 | BMI | -0.010 | 0.011 | -0.018 | | 0.013 | 0.010 | 0.018 |
| rs6921533 | 6 | 73742334 | T | C | 0.2933 | 0.010 | 0.002 | 1.37E-08 | 0.001 | 0.002 | 7.00E-01 | BMI | -0.001 | 0.011 | -0.007 | | 0.013 | 0.008 | 0.017 |
| rs9294260 | 6 | 83433228 | A | G | 0.4689 | 0.014 | 0.002 | 8.16E-18 | 0.000 | 0.002 | 9.40E-01 | BMI | -0.005 | 0.010 | -0.006 | | 0.012 | 0.005 | 0.015 |
| rs6909685 | 6 | 97753952 | T | C | 0.3295 | -0.015 | 0.002 | 2.78E-16 | 0.001 | 0.002 | 6.30E-01 | BMI | -0.011 | 0.010 | -0.015 | | 0.012 | 0.007 | 0.016 |
| rs9372625 | 6 | 98344031 | G | A | 0.619 | 0.015 | 0.002 | 5.41E-17 | -0.018 | 0.002 | 5.70E-16 | PC | 0.031 | 0.010 | 0.026 | | 0.012 | 0.041 | 0.016 |
| rs768023 | 6 | 108876002 | A | G | 0.6033 | 0.016 | 0.002 | 1.13E-22 | 0.001 | 0.002 | 6.60E-01 | BMI | 0.015 | 0.010 | 0.019 | | 0.012 | 0.006 | 0.015 |
| rs2357760 | 6 | 120213880 | A | G | 0.6735 | 0.014 | 0.002 | 2.11E-16 | 0.004 | 0.002 | 9.60E-02 | BMI | 0.001 | 0.010 | 0.000 | | 0.012 | -0.001 | 0.016 |
| rs2875762 | 6 | 124925032 | C | G | 0.252 | 0.013 | 0.002 | 1.07E-10 | 0.001 | 0.003 | 7.40E-01 | BMI | -0.003 | 0.011 | 0.002 | | 0.014 | -0.001 | 0.018 |
| rs1268065 | 6 | 126042783 | A | G | 0.5 | -0.010 | 0.002 | 7.12E-10 | -0.003 | 0.002 | 1.70E-01 | BMI | -0.001 | 0.010 | 0.006 | | 0.012 | -0.004 | 0.015 |
| rs9375702 | 6 | 130384187 | T | C | 0.6785 | -0.011 | 0.002 | 5.52E-09 | -0.005 | 0.002 | 5.10E-02 | BMI | 0.004 | 0.010 | 0.004 | | 0.013 | 0.002 | 0.016 |
| rs6935828 | 6 | 140811367 | C | T | 0.446 | -0.001 | 0.002 | 7.11E-01 | -0.012 | 0.002 | 4.30E-08 | PC | -0.002 | 0.010 | 0.002 | | 0.012 | 0.022 | 0.015 |
| rs6922607 | 6 | 142703483 | A | G | 0.808 | -0.013 | 0.002 | 1.85E-09 | 0.004 | 0.003 | 1.10E-01 | BMI | 0.012 | 0.012 | 0.016 | | 0.015 | -0.002 | 0.019 |
| rs7760482 | 6 | 147354276 | A | G | 0.6159 | -0.010 | 0.002 | 6.25E-09 | 0.003 | 0.002 | 1.60E-01 | BMI | 0.007 | 0.010 | 0.010 | | 0.012 | 0.012 | 0.016 |
| rs12527426 | 6 | 153392002 | A | G | 0.3 | 0.015 | 0.002 | 5.54E-16 | 0.002 | 0.002 | 5.20E-01 | BMI | 0.012 | 0.010 | -0.003 | | 0.013 | 0.024 | 0.016 |
| rs9478496 | 6 | 154333183 | T | C | 0.8453 | -0.016 | 0.002 | 8.17E-12 | -0.003 | 0.003 | 3.80E-01 | BMI | -0.018 | 0.013 | -0.012 | | 0.016 | -0.007 | 0.020 |
| rs13191362 | 6 | 163033350 | A | G | 0.8572 | 0.024 | 0.003 | 4.08E-21 | -0.005 | 0.003 | 1.10E-01 | BMI | 0.021 | 0.015 | 0.036 | | 0.018 | -0.008 | 0.023 |
| rs9458814 | 6 | 163771305 | T | C | 0.7708 | -0.011 | 0.002 | 2.33E-08 | -0.005 | 0.003 | 3.90E-02 | BMI | -0.021 | 0.011 | -0.027 | | 0.014 | -0.020 | 0.018 |
| rs6950388 | 7 | 1270699 | A | G | 0.7782 | 0.014 | 0.002 | 1.59E-09 | -0.001 | 0.003 | 7.50E-01 | BMI | 0.010 | 0.012 | 0.012 | | 0.015 | 0.026 | 0.019 |
| rs4721089 | 7 | 1872921 | T | C | 0.783 | 0.017 | 0.002 | 5.60E-13 | 0.003 | 0.003 | 2.10E-01 | BMI | 0.007 | 0.011 | 0.005 | | 0.014 | 0.013 | 0.018 |
| rs6463489 | 7 | 5542513 | T | C | 0.0959 | 0.017 | 0.003 | 2.50E-10 | 0.003 | 0.003 | 3.20E-01 | BMI | -0.007 | 0.016 | -0.011 | | 0.019 | -0.020 | 0.025 |
| rs4307239 | 7 | 24354300 | A | G | 0.5361 | -0.012 | 0.002 | 1.47E-11 | 0.003 | 0.002 | 1.40E-01 | BMI | -0.008 | 0.010 | -0.017 | | 0.012 | 0.016 | 0.015 |
| rs213518 | 7 | 26941065 | T | C | 0.8498 | -0.015 | 0.002 | 2.49E-10 | -0.008 | 0.003 | 1.40E-02 | BMI | -0.001 | 0.014 | -0.003 | | 0.017 | 0.028 | 0.022 |
| rs215669 | 7 | 32378979 | A | G | 0.6024 | -0.015 | 0.002 | 8.94E-18 | 0.004 | 0.002 | 8.20E-02 | BMI | 0.004 | 0.010 | 0.011 | | 0.012 | 0.010 | 0.015 |
| rs2108719 | 7 | 39481056 | A | G | 0.727 | 0.011 | 0.002 | 2.28E-08 | -0.003 | 0.003 | 2.90E-01 | BMI | -0.003 | 0.011 | 0.003 | | 0.013 | -0.007 | 0.017 |
| rs217433 | 7 | 44553496 | T | C | 0.8031 | -0.012 | 0.002 | 3.00E-08 | -0.002 | 0.003 | 3.60E-01 | BMI | 0.011 | 0.012 | 0.017 | | 0.015 | 0.027 | 0.019 |
| rs2289379 | 7 | 44804225 | T | C | 0.3917 | -0.014 | 0.002 | 7.02E-15 | 0.007 | 0.002 | 2.50E-03 | BMI | -0.015 | 0.010 | -0.009 | | 0.012 | 0.000 | 0.015 |
| rs10499694 | 7 | 50614173 | A | G | 0.4935 | 0.013 | 0.002 | 1.29E-15 | 0.001 | 0.002 | 5.50E-01 | BMI | -0.001 | 0.010 | -0.010 | | 0.012 | 0.017 | 0.015 |
| rs55772938 | 7 | 70025113 | A | G | 0.705 | -0.009 | 0.002 | 4.43E-05 | -0.015 | 0.002 | 3.90E-10 | PC | -0.008 | 0.011 | -0.001 | | 0.013 | -0.006 | 0.016 |
| rs11772246 | 7 | 71603692 | T | C | 0.8234 | 0.015 | 0.002 | 3.69E-11 | 0.003 | 0.003 | 3.60E-01 | BMI | 0.036 | 0.012 | 0.039 | | 0.015 | 0.020 | 0.019 |
| rs17149254 | 7 | 76634463 | T | C | 0.2051 | 0.024 | 0.002 | 2.99E-25 | -0.003 | 0.003 | 3.70E-01 | BMI | 0.012 | 0.012 | 0.025 | | 0.015 | 0.004 | 0.020 |
| rs274628 | 7 | 86265855 | A | C | 0.3366 | -0.010 | 0.002 | 1.36E-08 | -0.009 | 0.002 | 1.80E-04 | BMI | -0.013 | 0.010 | -0.010 | | 0.012 | -0.013 | 0.016 |
| rs2283006 | 7 | 93085722 | A | G | 0.4881 | 0.013 | 0.002 | 8.06E-15 | 0.000 | 0.002 | 8.80E-01 | BMI | -0.013 | 0.010 | -0.021 | | 0.012 | 0.003 | 0.015 |
| rs13240600 | 7 | 99064466 | A | G | 0.8343 | 0.018 | 0.002 | 7.90E-16 | 0.012 | 0.003 | 5.80E-05 | BMI | 0.011 | 0.013 | 0.011 | | 0.016 | -0.006 | 0.021 |
| rs1721447 | 7 | 109214139 | T | G | 0.5096 | -0.010 | 0.002 | 3.79E-09 | 0.000 | 0.002 | 9.10E-01 | BMI | 0.009 | 0.010 | 0.020 | | 0.012 | 0.011 | 0.015 |
| rs2396625 | 7 | 113028634 | A | T | 0.4183 | -0.018 | 0.002 | 2.81E-24 | 0.004 | 0.002 | 8.30E-02 | BMI | -0.010 | 0.010 | -0.011 | | 0.012 | 0.018 | 0.015 |
| rs13245051 | 7 | 113362799 | A | G | 0.4557 | 0.015 | 0.002 | 1.12E-18 | -0.002 | 0.002 | 4.80E-01 | BMI | -0.006 | 0.010 | -0.002 | | 0.012 | -0.012 | 0.016 |
| rs10261050 | 7 | 114337652 | T | C | 0.4754 | 0.011 | 0.002 | 4.53E-11 | -0.006 | 0.002 | 1.20E-02 | BMI | -0.005 | 0.010 | -0.010 | | 0.012 | 0.011 | 0.015 |
| rs12706626 | 7 | 124531370 | G | A | 0.617 | -0.002 | 0.002 | 4.25E-01 | -0.013 | 0.002 | 1.40E-08 | PC | -0.007 | 0.010 | -0.007 | | 0.012 | 0.003 | 0.016 |
| rs3944151 | 7 | 126970005 | A | G | 0.275 | 0.000 | 0.002 | 9.81E-01 | 0.014 | 0.002 | 3.10E-08 | PC | 0.021 | 0.011 | 0.029 | | 0.013 | 0.016 | 0.017 |
| rs2345941 | 7 | 133337635 | A | G | 0.55 | -0.002 | 0.002 | 3.27E-01 | 0.015 | 0.002 | 4.00E-11 | PC | -0.023 | 0.010 | -0.025 | | 0.012 | -0.012 | 0.015 |
| rs7802342 | 7 | 137435925 | T | G | 0.7048 | -0.012 | 0.002 | 6.23E-11 | -0.001 | 0.002 | 6.90E-01 | BMI | -0.013 | 0.011 | -0.014 | | 0.013 | -0.025 | 0.018 |
| rs11773362 | 7 | 147668180 | T | C | 0.3396 | -0.011 | 0.002 | 6.39E-09 | 0.001 | 0.002 | 6.60E-01 | BMI | -0.009 | 0.010 | -0.022 | | 0.012 | 0.002 | 0.016 |
| rs2907948 | 7 | 150638484 | A | G | 0.244 | -0.015 | 0.002 | 1.95E-14 | -0.003 | 0.003 | 3.10E-01 | BMI | 0.002 | 0.011 | -0.002 | | 0.014 | 0.014 | 0.018 |
| rs56211164 | 7 | 158016764 | A | G | 0.2395 | -0.013 | 0.002 | 8.90E-09 | -0.003 | 0.003 | 1.90E-01 | BMI | -0.012 | 0.012 | -0.020 | | 0.014 | -0.024 | 0.018 |
| rs1700082 | 8 | 4121727 | C | G | 0.659 | 0.009 | 0.002 | 3.38E-08 | -0.001 | 0.002 | 7.20E-01 | BMI | -0.009 | 0.010 | -0.014 | | 0.012 | -0.009 | 0.016 |
| rs4240673 | 8 | 10787612 | T | C | 0.4545 | 0.018 | 0.002 | 1.58E-26 | 0.002 | 0.002 | 4.40E-01 | BMI | 0.010 | 0.011 | -0.012 | | 0.013 | 0.033 | 0.017 |
| rs13263601 | 8 | 14095900 | A | C | 0.6585 | -0.015 | 0.002 | 4.87E-16 | -0.003 | 0.002 | 1.50E-01 | BMI | 0.009 | 0.010 | 0.018 | | 0.012 | -0.005 | 0.016 |
| rs10110189 | 8 | 15393380 | T | C | 0.1057 | -0.016 | 0.003 | 2.20E-08 | 0.004 | 0.004 | 3.30E-01 | BMI | 0.014 | 0.016 | 0.003 | | 0.019 | -0.001 | 0.026 |
| rs10101364 | 8 | 20634888 | T | C | 0.6801 | 0.012 | 0.002 | 5.61E-11 | 0.005 | 0.002 | 2.60E-02 | BMI | 0.016 | 0.011 | 0.017 | | 0.013 | 0.016 | 0.016 |
| rs73225274 | 8 | 21088909 | A | G | 0.8649 | -0.016 | 0.003 | 2.33E-08 | 0.002 | 0.003 | 6.30E-01 | BMI | -0.009 | 0.014 | -0.023 | | 0.017 | -0.005 | 0.022 |
| rs13249650 | 8 | 25664336 | A | T | 0.4443 | 0.011 | 0.002 | 1.02E-08 | -0.007 | 0.002 | 1.80E-03 | BMI | 0.002 | 0.010 | 0.006 | | 0.012 | -0.001 | 0.015 |
| rs1421334 | 8 | 30865733 | A | C | 0.4546 | 0.014 | 0.002 | 3.11E-15 | -0.002 | 0.002 | 4.40E-01 | BMI | 0.017 | 0.010 | 0.022 | | 0.012 | -0.005 | 0.015 |
| rs2466103 | 8 | 32412304 | T | G | 0.6928 | -0.012 | 0.002 | 8.00E-12 | -0.002 | 0.002 | 5.00E-01 | BMI | -0.010 | 0.011 | -0.009 | | 0.012 | -0.009 | 0.016 |
| rs6468266 | 8 | 34380276 | A | T | 0.4176 | -0.011 | 0.002 | 7.71E-11 | 0.004 | 0.002 | 9.10E-02 | BMI | -0.011 | 0.010 | -0.011 | | 0.012 | -0.005 | 0.016 |
| rs12681792 | 8 | 62054463 | A | C | 0.2024 | 0.015 | 0.002 | 2.88E-12 | 0.005 | 0.003 | 5.80E-02 | BMI | 0.019 | 0.012 | 0.010 | | 0.015 | 0.015 | 0.020 |
| rs12334877 | 8 | 67194171 | A | G | 0.1913 | -0.014 | 0.002 | 2.17E-11 | -0.003 | 0.003 | 2.20E-01 | BMI | 0.009 | 0.012 | 0.010 | | 0.014 | 0.012 | 0.019 |
| rs1808629 | 8 | 73435964 | A | G | 0.674 | -0.020 | 0.002 | 2.19E-23 | -0.002 | 0.002 | 3.30E-01 | BMI | 0.020 | 0.010 | 0.023 | | 0.013 | 0.027 | 0.016 |
| rs17405819 | 8 | 76806584 | T | C | 0.6845 | 0.021 | 0.002 | 6.04E-33 | 0.002 | 0.002 | 4.60E-01 | BMI | -0.001 | 0.010 | -0.003 | | 0.013 | 0.025 | 0.017 |
| rs2196618 | 8 | 85089437 | A | G | 0.2634 | -0.014 | 0.002 | 1.49E-12 | -0.003 | 0.003 | 2.10E-01 | BMI | 0.001 | 0.011 | 0.002 | | 0.013 | -0.001 | 0.017 |
| rs12386885 | 8 | 87766769 | T | C | 0.1564 | 0.015 | 0.003 | 9.90E-09 | -0.003 | 0.003 | 3.10E-01 | BMI | -0.011 | 0.013 | -0.006 | | 0.016 | -0.031 | 0.021 |
| rs2120710 | 8 | 93210803 | A | G | 0.6529 | 0.010 | 0.002 | 1.98E-08 | -0.003 | 0.002 | 1.60E-01 | BMI | 0.003 | 0.010 | -0.002 | | 0.012 | 0.015 | 0.016 |
| rs12680842 | 8 | 95582606 | A | G | 0.6839 | 0.014 | 0.002 | 3.41E-16 | 0.000 | 0.002 | 8.60E-01 | BMI | 0.009 | 0.010 | 0.004 | | 0.012 | -0.001 | 0.016 |
| rs1383592 | 8 | 106430676 | A | G | 0.212 | 0.012 | 0.002 | 4.92E-09 | 0.001 | 0.003 | 6.00E-01 | BMI | -0.013 | 0.012 | -0.004 | | 0.014 | -0.018 | 0.018 |
| rs3808477 | 8 | 116670347 | T | C | 0.274 | -0.018 | 0.002 | 8.73E-22 | -0.001 | 0.002 | 6.70E-01 | BMI | -0.007 | 0.011 | -0.014 | | 0.013 | -0.009 | 0.017 |
| rs72673947 | 8 | 118884379 | A | G | 0.8902 | -0.022 | 0.003 | 4.11E-13 | 0.000 | 0.004 | 9.60E-01 | BMI | -0.006 | 0.015 | 0.002 | | 0.018 | -0.006 | 0.024 |
| rs6470144 | 8 | 124152245 | T | G | 0.6503 | 0.010 | 0.002 | 3.06E-08 | -0.001 | 0.002 | 7.20E-01 | BMI | -0.002 | 0.010 | -0.006 | | 0.012 | -0.002 | 0.016 |
| rs7842934 | 8 | 132838921 | T | C | 0.9211 | -0.018 | 0.003 | 1.55E-08 | 0.001 | 0.004 | 7.40E-01 | BMI | 0.016 | 0.018 | 0.026 | | 0.023 | 0.050 | 0.030 |
| rs13262595 | 8 | 143316970 | A | G | 0.44 | -0.005 | 0.002 | 4.97E-03 | -0.016 | 0.002 | 1.20E-12 | PC | -0.001 | 0.010 | -0.007 | | 0.012 | 0.012 | 0.015 |
| rs10975933 | 9 | 6954557 | C | G | 0.6634 | 0.011 | 0.002 | 2.07E-10 | -0.003 | 0.002 | 1.40E-01 | BMI | 0.014 | 0.010 | 0.011 | | 0.012 | 0.014 | 0.016 |
| rs1948080 | 9 | 11852043 | T | G | 0.6303 | 0.014 | 0.002 | 1.13E-14 | 0.003 | 0.002 | 2.30E-01 | BMI | -0.019 | 0.010 | -0.022 | | 0.012 | -0.007 | 0.016 |
| rs10962550 | 9 | 16720329 | C | G | 0.1756 | 0.019 | 0.002 | 5.68E-17 | -0.002 | 0.003 | 4.00E-01 | BMI | 0.016 | 0.012 | 0.013 | | 0.015 | -0.015 | 0.019 |
| rs4977839 | 9 | 23355310 | G | A | 0.584 | -0.010 | 0.002 | 7.01E-07 | -0.020 | 0.002 | 4.70E-19 | PC | -0.004 | 0.010 | 0.010 | | 0.012 | -0.006 | 0.016 |
| rs10968114 | 9 | 27800007 | A | C | 0.5331 | 0.011 | 0.002 | 3.30E-11 | 0.001 | 0.002 | 6.30E-01 | BMI | 0.019 | 0.010 | 0.027 | | 0.012 | 0.022 | 0.015 |
| rs1412235 | 9 | 28410996 | C | G | 0.3151 | 0.024 | 0.002 | 2.28E-42 | 0.005 | 0.002 | 4.60E-02 | BMI | 0.027 | 0.011 | 0.023 | | 0.013 | 0.018 | 0.017 |
| rs2275003 | 9 | 34124860 | A | G | 0.5007 | 0.011 | 0.002 | 9.44E-12 | -0.001 | 0.002 | 7.90E-01 | BMI | 0.008 | 0.010 | 0.010 | | 0.012 | 0.019 | 0.015 |
| rs13296413 | 9 | 37258105 | T | C | 0.3811 | -0.015 | 0.002 | 2.49E-17 | 0.000 | 0.002 | 8.60E-01 | BMI | 0.015 | 0.010 | 0.006 | | 0.012 | 0.032 | 0.015 |
| rs2134858 | 9 | 73837155 | T | C | 0.5119 | -0.012 | 0.002 | 5.87E-12 | 0.004 | 0.002 | 5.10E-02 | BMI | -0.005 | 0.010 | -0.003 | | 0.012 | -0.033 | 0.015 |
| rs7861160 | 9 | 80799579 | T | C | 0.5941 | 0.010 | 0.002 | 3.68E-08 | 0.002 | 0.002 | 3.10E-01 | BMI | -0.008 | 0.010 | -0.009 | | 0.012 | 0.002 | 0.015 |
| rs1999433 | 9 | 81371441 | T | C | 0.4403 | -0.011 | 0.002 | 2.90E-10 | 0.000 | 0.002 | 8.70E-01 | BMI | -0.004 | 0.010 | -0.005 | | 0.012 | -0.011 | 0.015 |
| rs2777768 | 9 | 84186734 | A | G | 0.7228 | 0.012 | 0.002 | 6.38E-10 | -0.001 | 0.002 | 5.50E-01 | BMI | 0.005 | 0.011 | 0.011 | | 0.013 | -0.028 | 0.017 |
| rs10992867 | 9 | 96461013 | A | G | 0.2687 | 0.016 | 0.002 | 3.20E-17 | -0.001 | 0.003 | 7.00E-01 | BMI | -0.006 | 0.011 | -0.009 | | 0.013 | 0.005 | 0.017 |
| rs113851275 | 9 | 98297220 | G | A | 0.892 | -0.002 | 0.003 | 6.23E-01 | -0.021 | 0.004 | 3.20E-09 | PC | 0.022 | 0.015 | 0.010 | | 0.019 | -0.001 | 0.024 |
| rs7025938 | 9 | 103088321 | C | G | 0.6798 | -0.016 | 0.002 | 1.47E-19 | 0.002 | 0.002 | 3.60E-01 | BMI | -0.016 | 0.010 | -0.011 | | 0.012 | -0.036 | 0.016 |
| rs7024334 | 9 | 109072075 | T | G | 0.2254 | 0.014 | 0.002 | 4.71E-12 | 0.001 | 0.003 | 7.90E-01 | BMI | 0.011 | 0.011 | 0.003 | | 0.014 | 0.018 | 0.018 |
| rs7020477 | 9 | 116827760 | A | G | 0.735 | 0.002 | 0.002 | 4.96E-01 | 0.014 | 0.002 | 1.20E-08 | PC | 0.016 | 0.012 | 0.020 | | 0.014 | 0.011 | 0.018 |
| rs73578186 | 9 | 126334485 | C | T | 0.675 | -0.005 | 0.002 | 2.79E-02 | 0.015 | 0.002 | 2.30E-10 | PC | -0.015 | 0.010 | -0.018 | | 0.012 | -0.003 | 0.016 |
| rs10733682 | 9 | 129460914 | A | G | 0.4594 | 0.015 | 0.002 | 1.76E-19 | -0.002 | 0.002 | 4.50E-01 | BMI | 0.003 | 0.010 | 0.008 | | 0.012 | 0.001 | 0.015 |
| rs10858334 | 9 | 137989785 | C | G | 0.8484 | -0.015 | 0.003 | 4.91E-09 | -0.011 | 0.003 | 7.10E-04 | BMI | 0.014 | 0.014 | 0.016 | | 0.017 | 0.038 | 0.023 |
| rs7907470 | 10 | 10268989 | A | G | 0.9161 | -0.018 | 0.003 | 7.36E-09 | 0.003 | 0.004 | 5.30E-01 | BMI | -0.010 | 0.018 | -0.005 | | 0.021 | -0.032 | 0.028 |
| rs7893571 | 10 | 16750129 | T | G | 0.6736 | 0.013 | 0.002 | 5.83E-12 | 0.006 | 0.002 | 1.30E-02 | BMI | -0.001 | 0.010 | -0.005 | | 0.012 | 0.005 | 0.016 |
| rs76638898 | 10 | 21099584 | A | G | 0.0234 | -0.038 | 0.007 | 8.15E-09 | -0.001 | 0.007 | 8.80E-01 | BMI | -0.017 | 0.034 | -0.035 | | 0.041 | -0.042 | 0.054 |
| rs7084454 | 10 | 21821274 | A | G | 0.3086 | 0.020 | 0.002 | 4.51E-27 | 0.011 | 0.002 | 6.40E-06 | BMI | 0.026 | 0.011 | 0.022 | | 0.013 | 0.052 | 0.016 |
| rs10829164 | 10 | 27318370 | T | C | 0.1468 | 0.015 | 0.002 | 2.37E-10 | 0.008 | 0.003 | 9.20E-03 | BMI | 0.006 | 0.013 | 0.001 | | 0.016 | 0.066 | 0.021 |
| rs4097319 | 10 | 33860515 | T | G | 0.5641 | 0.011 | 0.002 | 4.32E-10 | -0.007 | 0.002 | 1.80E-03 | BMI | -0.005 | 0.010 | -0.017 | | 0.012 | 0.019 | 0.015 |
| rs12765914 | 10 | 34013507 | T | C | 0.0801 | 0.023 | 0.003 | 1.96E-13 | 0.009 | 0.004 | 2.40E-02 | BMI | 0.012 | 0.017 | 0.023 | | 0.021 | 0.039 | 0.027 |
| rs1624134 | 10 | 34834482 | C | G | 0.3925 | 0.010 | 0.002 | 6.51E-09 | 0.004 | 0.002 | 9.70E-02 | BMI | 0.009 | 0.010 | 0.009 | | 0.012 | 0.003 | 0.015 |
| rs12259464 | 10 | 53680099 | A | G | 0.4811 | 0.011 | 0.002 | 1.48E-10 | -0.005 | 0.002 | 3.80E-02 | BMI | 0.007 | 0.010 | 0.015 | | 0.012 | -0.005 | 0.015 |
| rs7070670 | 10 | 61842645 | T | C | 0.3228 | -0.013 | 0.002 | 7.14E-10 | -0.002 | 0.002 | 3.20E-01 | BMI | -0.014 | 0.010 | -0.009 | | 0.012 | -0.026 | 0.016 |
| rs10761785 | 10 | 65318766 | T | G | 0.5136 | -0.013 | 0.002 | 3.47E-16 | 0.001 | 0.002 | 6.30E-01 | BMI | 0.009 | 0.010 | 0.013 | | 0.012 | -0.001 | 0.015 |
| rs12098284 | 10 | 76047464 | T | C | 0.1212 | 0.018 | 0.003 | 9.87E-13 | 0.006 | 0.003 | 6.10E-02 | BMI | -0.010 | 0.015 | -0.025 | | 0.018 | 0.035 | 0.023 |
| rs11001259 | 10 | 76807650 | A | T | 0.1767 | -0.014 | 0.002 | 1.22E-08 | -0.001 | 0.003 | 7.50E-01 | BMI | -0.005 | 0.015 | -0.005 | | 0.018 | 0.006 | 0.024 |
| rs7899106 | 10 | 87410904 | A | G | 0.9496 | -0.033 | 0.004 | 1.72E-18 | -0.003 | 0.005 | 5.20E-01 | BMI | 0.037 | 0.023 | 0.043 | | 0.027 | 0.031 | 0.035 |
| rs10788494 | 10 | 88110925 | C | G | 0.4835 | 0.013 | 0.002 | 5.51E-15 | 0.000 | 0.002 | 9.70E-01 | BMI | -0.008 | 0.010 | -0.015 | | 0.012 | 0.004 | 0.015 |
| rs147568678 | 10 | 93061851 | T | C | 0.7684 | 0.013 | 0.002 | 3.42E-09 | 0.002 | 0.003 | 3.40E-01 | BMI | -0.021 | 0.012 | -0.016 | | 0.014 | -0.019 | 0.019 |
| rs2439823 | 10 | 99778226 | A | G | 0.4514 | -0.017 | 0.002 | 6.51E-22 | 0.000 | 0.002 | 9.20E-01 | BMI | -0.001 | 0.010 | 0.002 | | 0.012 | 0.002 | 0.015 |
| rs17094222 | 10 | 102395440 | T | C | 0.7912 | -0.017 | 0.002 | 4.04E-18 | 0.000 | 0.003 | 9.60E-01 | BMI | -0.005 | 0.012 | -0.011 | | 0.014 | -0.003 | 0.018 |
| rs11594179 | 10 | 104392580 | T | C | 0.2328 | -0.011 | 0.002 | 2.16E-08 | 0.007 | 0.003 | 8.60E-03 | BMI | -0.002 | 0.012 | -0.008 | | 0.014 | 0.016 | 0.018 |
| rs79780963 | 10 | 104952499 | T | C | 0.0804 | 0.024 | 0.004 | 2.63E-12 | 0.004 | 0.004 | 2.80E-01 | BMI | 0.048 | 0.016 | 0.062 | | 0.020 | 0.017 | 0.027 |
| rs7903146 | 10 | 114758349 | T | C | 0.2787 | -0.018 | 0.002 | 1.67E-23 | 0.000 | 0.002 | 8.40E-01 | BMI | 0.003 | 0.011 | 0.004 | | 0.013 | -0.001 | 0.017 |
| rs2257791 | 10 | 118643670 | A | G | 0.756 | -0.014 | 0.002 | 1.63E-12 | -0.005 | 0.003 | 6.90E-02 | BMI | 0.023 | 0.011 | 0.013 | | 0.013 | 0.009 | 0.018 |
| rs845084 | 10 | 125220036 | A | G | 0.2666 | 0.014 | 0.002 | 3.22E-12 | 0.001 | 0.003 | 7.20E-01 | BMI | -0.011 | 0.011 | -0.009 | | 0.013 | -0.011 | 0.017 |
| rs17636031 | 10 | 126594078 | T | C | 0.7244 | -0.015 | 0.002 | 3.87E-17 | -0.002 | 0.002 | 5.10E-01 | BMI | 0.004 | 0.011 | 0.005 | | 0.013 | 0.003 | 0.017 |
| rs4880341 | 10 | 133992689 | T | C | 0.5725 | -0.013 | 0.002 | 3.06E-14 | -0.003 | 0.002 | 2.40E-01 | BMI | -0.006 | 0.010 | -0.002 | | 0.012 | -0.015 | 0.015 |
| rs11246136 | 11 | 371265 | A | C | 0.1021 | -0.017 | 0.003 | 2.03E-08 | -0.002 | 0.004 | 6.10E-01 | BMI | 0.008 | 0.017 | 0.019 | | 0.021 | -0.003 | 0.027 |
| rs12421848 | 11 | 891338 | A | G | 0.3962 | -0.014 | 0.002 | 3.99E-13 | -0.005 | 0.002 | 2.00E-02 | BMI | 0.018 | 0.010 | 0.020 | | 0.012 | 0.016 | 0.016 |
| rs4256980 | 11 | 8673939 | C | G | 0.3305 | -0.019 | 0.002 | 8.63E-29 | 0.000 | 0.002 | 9.30E-01 | BMI | -0.001 | 0.010 | -0.004 | | 0.012 | 0.001 | 0.016 |
| rs900144 | 11 | 13294268 | T | C | 0.5784 | 0.015 | 0.002 | 1.53E-18 | 0.006 | 0.002 | 1.20E-02 | BMI | -0.016 | 0.010 | -0.012 | | 0.012 | -0.037 | 0.015 |
| rs2074314 | 11 | 17411821 | T | C | 0.6356 | 0.011 | 0.002 | 1.37E-09 | 0.002 | 0.002 | 4.10E-01 | BMI | -0.007 | 0.010 | -0.009 | | 0.012 | -0.003 | 0.016 |
| rs6265 | 11 | 27679916 | T | C | 0.1836 | -0.041 | 0.002 | 7.40E-89 | -0.011 | 0.003 | 4.70E-05 | BMI | -0.008 | 0.012 | -0.015 | | 0.015 | -0.010 | 0.019 |
| rs570463 | 11 | 28739318 | A | C | 0.3266 | -0.012 | 0.002 | 4.33E-11 | 0.005 | 0.002 | 3.00E-02 | BMI | -0.017 | 0.011 | -0.005 | | 0.012 | -0.007 | 0.016 |
| rs11030618 | 11 | 29243293 | T | C | 0.5786 | 0.011 | 0.002 | 1.67E-10 | -0.002 | 0.002 | 3.80E-01 | BMI | -0.016 | 0.010 | -0.014 | | 0.012 | -0.025 | 0.015 |
| rs2065418 | 11 | 30422068 | T | G | 0.646 | 0.014 | 0.002 | 6.07E-15 | 0.001 | 0.002 | 7.50E-01 | BMI | 0.016 | 0.010 | 0.005 | | 0.012 | 0.008 | 0.016 |
| rs2862996 | 11 | 43653833 | T | G | 0.7012 | -0.022 | 0.002 | 3.60E-35 | 0.000 | 0.002 | 9.50E-01 | BMI | -0.009 | 0.010 | -0.013 | | 0.012 | 0.010 | 0.016 |
| rs10742752 | 11 | 45438374 | T | C | 0.3761 | -0.012 | 0.002 | 1.20E-13 | -0.003 | 0.002 | 1.30E-01 | BMI | 0.015 | 0.010 | 0.012 | | 0.012 | 0.025 | 0.015 |
| rs118081010 | 11 | 46174948 | T | C | 0.0165 | 0.052 | 0.008 | 5.28E-12 | -0.005 | 0.009 | 5.70E-01 | BMI | 0.033 | 0.040 | 0.029 | | 0.050 | 0.064 | 0.065 |
| rs7124681 | 11 | 47529947 | A | C | 0.4162 | 0.026 | 0.002 | 3.96E-55 | 0.009 | 0.002 | 7.20E-05 | BMI | 0.007 | 0.010 | 0.004 | | 0.012 | 0.003 | 0.015 |
| rs6591407 | 11 | 56914157 | A | C | 0.1984 | -0.012 | 0.002 | 3.58E-09 | 0.001 | 0.003 | 7.20E-01 | BMI | -0.035 | 0.012 | -0.035 | | 0.015 | -0.035 | 0.019 |
| rs562664 | 11 | 63823619 | T | C | 0.1873 | -0.014 | 0.002 | 4.15E-10 | 0.000 | 0.003 | 8.70E-01 | BMI | -0.001 | 0.013 | 0.011 | | 0.015 | -0.005 | 0.020 |
| rs7102454 | 11 | 65594820 | T | C | 0.6437 | -0.017 | 0.002 | 3.84E-21 | -0.002 | 0.002 | 4.20E-01 | BMI | -0.024 | 0.010 | -0.032 | | 0.012 | -0.019 | 0.016 |
| rs592483 | 11 | 69445173 | T | C | 0.5933 | -0.014 | 0.002 | 1.53E-16 | 0.000 | 0.002 | 9.90E-01 | BMI | -0.015 | 0.010 | -0.024 | | 0.012 | 0.000 | 0.015 |
| rs12282785 | 11 | 76476030 | A | C | 0.2204 | -0.016 | 0.002 | 1.43E-11 | 0.000 | 0.003 | 9.70E-01 | BMI | 0.001 | 0.012 | -0.003 | | 0.014 | -0.013 | 0.018 |
| rs349088 | 11 | 84814393 | A | C | 0.4777 | -0.013 | 0.002 | 3.50E-14 | -0.002 | 0.002 | 4.50E-01 | BMI | -0.008 | 0.010 | 0.004 | | 0.012 | -0.024 | 0.015 |
| rs10741329 | 11 | 89997796 | A | G | 0.6944 | 0.012 | 0.002 | 3.91E-10 | 0.000 | 0.002 | 8.40E-01 | BMI | -0.006 | 0.010 | -0.006 | | 0.012 | -0.013 | 0.016 |
| rs3019466 | 11 | 92476178 | T | C | 0.1677 | -0.013 | 0.002 | 4.91E-08 | 0.001 | 0.003 | 6.30E-01 | BMI | -0.005 | 0.013 | -0.004 | | 0.016 | -0.001 | 0.021 |
| rs2605603 | 11 | 93221105 | A | G | 0.4812 | -0.010 | 0.002 | 2.04E-10 | 0.001 | 0.002 | 7.70E-01 | BMI | -0.013 | 0.010 | -0.016 | | 0.012 | 0.017 | 0.015 |
| rs2513999 | 11 | 103019633 | A | G | 0.1593 | -0.015 | 0.003 | 3.07E-09 | 0.005 | 0.003 | 1.00E-01 | BMI | -0.012 | 0.014 | -0.014 | | 0.016 | 0.004 | 0.021 |
| rs2734849 | 11 | 113270160 | A | G | 0.491 | 0.009 | 0.002 | 2.61E-08 | -0.014 | 0.002 | 8.20E-10 | PC | -0.003 | 0.010 | -0.009 | | 0.012 | 0.012 | 0.015 |
| rs12286929 | 11 | 115022404 | A | G | 0.5007 | -0.018 | 0.002 | 1.93E-27 | 0.002 | 0.002 | 3.40E-01 | BMI | 0.011 | 0.010 | 0.015 | | 0.012 | 0.006 | 0.015 |
| rs76942203 | 11 | 116973247 | A | G | 0.0587 | 0.026 | 0.004 | 9.08E-11 | -0.010 | 0.005 | 4.60E-02 | BMI | -0.005 | 0.020 | -0.002 | | 0.024 | -0.031 | 0.032 |
| rs3825061 | 11 | 118944675 | T | C | 0.3863 | 0.014 | 0.002 | 6.15E-16 | -0.005 | 0.002 | 4.00E-02 | BMI | 0.018 | 0.010 | 0.013 | | 0.012 | 0.028 | 0.016 |
| rs7944782 | 11 | 130795698 | T | G | 0.5016 | -0.014 | 0.002 | 3.61E-17 | 0.002 | 0.002 | 4.80E-01 | BMI | -0.010 | 0.010 | -0.004 | | 0.012 | -0.022 | 0.015 |
| rs1448355 | 11 | 131286685 | C | T | 0.381 | 0.001 | 0.002 | 4.80E-01 | -0.015 | 0.002 | 1.30E-10 | PC | 0.012 | 0.010 | 0.004 | | 0.012 | 0.013 | 0.016 |
| rs2007518 | 11 | 132639606 | A | G | 0.5618 | -0.013 | 0.002 | 1.96E-14 | -0.006 | 0.002 | 3.40E-03 | BMI | 0.006 | 0.010 | 0.012 | | 0.012 | 0.007 | 0.015 |
| rs329651 | 11 | 133767622 | T | G | 0.806 | 0.016 | 0.002 | 2.13E-14 | -0.009 | 0.003 | 1.90E-03 | BMI | 0.006 | 0.013 | 0.001 | | 0.015 | 0.005 | 0.020 |
| rs12364470 | 11 | 134601012 | T | G | 0.8535 | -0.019 | 0.002 | 2.18E-17 | -0.001 | 0.003 | 7.50E-01 | BMI | -0.011 | 0.014 | -0.001 | | 0.016 | -0.055 | 0.021 |
| rs11611246 | 12 | 939480 | T | G | 0.199 | 0.022 | 0.002 | 2.04E-28 | 0.002 | 0.003 | 3.80E-01 | BMI | 0.014 | 0.012 | 0.026 | | 0.014 | -0.004 | 0.019 |
| rs765125 | 12 | 2156207 | T | C | 0.5855 | -0.010 | 0.002 | 1.27E-08 | -0.003 | 0.002 | 1.50E-01 | BMI | -0.003 | 0.010 | -0.008 | | 0.012 | -0.007 | 0.015 |
| rs10772983 | 12 | 17141582 | T | C | 0.5394 | -0.010 | 0.002 | 6.21E-10 | 0.002 | 0.002 | 2.90E-01 | BMI | 0.001 | 0.010 | 0.006 | | 0.012 | 0.014 | 0.015 |
| rs11044430 | 12 | 19287416 | A | T | 0.844 | 0.016 | 0.002 | 3.77E-12 | 0.000 | 0.003 | 9.20E-01 | BMI | 0.023 | 0.013 | 0.002 | | 0.016 | 0.041 | 0.021 |
| rs11046972 | 12 | 23705969 | T | C | 0.0714 | 0.018 | 0.003 | 3.72E-08 | -0.004 | 0.004 | 3.20E-01 | BMI | -0.013 | 0.017 | -0.009 | | 0.021 | 0.005 | 0.027 |
| rs10842240 | 12 | 24060075 | C | G | 0.1256 | 0.020 | 0.003 | 1.21E-14 | 0.003 | 0.003 | 4.20E-01 | BMI | -0.001 | 0.014 | -0.003 | | 0.017 | -0.008 | 0.022 |
| rs35933007 | 12 | 38929341 | G | A | 0.772 | 0.008 | 0.002 | 4.91E-04 | -0.015 | 0.003 | 9.20E-09 | PC | -0.009 | 0.013 | -0.011 | | 0.016 | -0.002 | 0.021 |
| rs1350430 | 12 | 41819215 | T | C | 0.5328 | -0.013 | 0.002 | 2.08E-13 | -0.003 | 0.002 | 2.20E-01 | BMI | 0.001 | 0.010 | 0.008 | | 0.012 | 0.000 | 0.015 |
| rs1126930 | 12 | 49399132 | C | G | 0.0339 | 0.034 | 0.005 | 1.00E-10 | -0.003 | 0.006 | 6.00E-01 | BMI | 0.004 | 0.029 | 0.020 | | 0.035 | 0.034 | 0.046 |
| rs7138803 | 12 | 50247468 | A | G | 0.387 | 0.030 | 0.002 | 3.10E-71 | 0.000 | 0.002 | 9.80E-01 | BMI | 0.011 | 0.010 | 0.004 | | 0.012 | 0.005 | 0.016 |
| rs2271189 | 12 | 56494991 | A | G | 0.396 | -0.014 | 0.002 | 9.26E-16 | 0.005 | 0.002 | 1.60E-02 | BMI | -0.011 | 0.010 | -0.023 | | 0.012 | -0.009 | 0.016 |
| rs7975187 | 12 | 60964108 | A | G | 0.7712 | -0.014 | 0.002 | 3.86E-11 | 0.002 | 0.003 | 5.30E-01 | BMI | -0.010 | 0.012 | -0.010 | | 0.014 | -0.015 | 0.018 |
| rs650198 | 12 | 69674595 | T | C | 0.7294 | -0.014 | 0.002 | 6.43E-13 | 0.003 | 0.002 | 2.40E-01 | BMI | 0.020 | 0.011 | 0.023 | | 0.013 | 0.008 | 0.017 |
| rs11115176 | 12 | 82465797 | T | C | 0.7839 | 0.013 | 0.002 | 6.79E-12 | -0.002 | 0.003 | 4.60E-01 | BMI | 0.001 | 0.011 | 0.003 | | 0.014 | -0.019 | 0.018 |
| rs10506971 | 12 | 89757937 | A | G | 0.5521 | -0.014 | 0.002 | 4.69E-17 | 0.001 | 0.002 | 6.90E-01 | BMI | 0.004 | 0.010 | 0.006 | | 0.012 | -0.020 | 0.015 |
| rs11105839 | 12 | 91237920 | A | T | 0.3729 | -0.011 | 0.002 | 1.25E-11 | -0.001 | 0.002 | 6.30E-01 | BMI | 0.027 | 0.010 | 0.038 | | 0.012 | -0.010 | 0.016 |
| rs2712665 | 12 | 99594947 | T | C | 0.6968 | -0.011 | 0.002 | 5.40E-09 | 0.001 | 0.002 | 7.60E-01 | BMI | 0.003 | 0.011 | -0.008 | | 0.013 | 0.039 | 0.017 |
| rs6539064 | 12 | 103706754 | C | G | 0.7468 | 0.019 | 0.002 | 1.14E-23 | -0.002 | 0.003 | 5.30E-01 | BMI | 0.005 | 0.011 | 0.008 | | 0.013 | -0.009 | 0.017 |
| rs1860561 | 12 | 110783241 | A | G | 0.2087 | 0.016 | 0.002 | 1.71E-16 | -0.006 | 0.003 | 2.10E-02 | BMI | 0.007 | 0.012 | 0.016 | | 0.015 | 0.000 | 0.019 |
| rs11066188 | 12 | 112610714 | A | G | 0.3762 | -0.011 | 0.002 | 3.05E-12 | 0.001 | 0.002 | 5.70E-01 | BMI | -0.062 | 0.010 | -0.072 | | 0.012 | -0.060 | 0.015 |
| rs206965 | 12 | 120856332 | T | C | 0.207 | 0.003 | 0.002 | 2.07E-01 | 0.016 | 0.003 | 9.10E-09 | PC | -0.008 | 0.012 | -0.001 | | 0.014 | -0.029 | 0.019 |
| rs11060853 | 12 | 123424071 | A | G | 0.591 | -0.011 | 0.002 | 3.10E-08 | -0.002 | 0.002 | 2.70E-01 | BMI | -0.008 | 0.010 | -0.022 | | 0.012 | -0.007 | 0.017 |
| rs11614340 | 12 | 133426483 | T | C | 0.7066 | -0.012 | 0.002 | 1.87E-10 | 0.000 | 0.002 | 9.50E-01 | BMI | -0.016 | 0.011 | -0.009 | | 0.013 | -0.032 | 0.017 |
| rs9512648 | 13 | 27933910 | A | G | 0.4741 | 0.010 | 0.002 | 4.11E-08 | 0.003 | 0.002 | 1.10E-01 | BMI | 0.010 | 0.010 | 0.011 | | 0.012 | 0.016 | 0.015 |
| rs7323 | 13 | 28009031 | C | G | 0.268 | -0.017 | 0.002 | 2.18E-18 | -0.005 | 0.002 | 2.60E-02 | BMI | 0.007 | 0.011 | 0.004 | | 0.013 | 0.017 | 0.017 |
| rs4771218 | 13 | 28655311 | A | G | 0.6244 | -0.014 | 0.002 | 3.18E-15 | -0.004 | 0.002 | 7.00E-02 | BMI | 0.009 | 0.010 | 0.012 | | 0.012 | -0.006 | 0.016 |
| rs9595908 | 13 | 33184288 | T | C | 0.6427 | 0.015 | 0.002 | 3.73E-20 | -0.001 | 0.002 | 8.00E-01 | BMI | -0.002 | 0.010 | 0.004 | | 0.012 | -0.013 | 0.016 |
| rs9603697 | 13 | 40783323 | T | C | 0.3184 | 0.013 | 0.002 | 1.69E-13 | -0.001 | 0.002 | 7.00E-01 | BMI | -0.011 | 0.010 | -0.018 | | 0.012 | -0.015 | 0.016 |
| rs12429545 | 13 | 54102206 | A | G | 0.1218 | 0.031 | 0.002 | 1.42E-37 | -0.005 | 0.003 | 1.20E-01 | BMI | -0.005 | 0.014 | -0.015 | | 0.017 | -0.020 | 0.023 |
| rs962796 | 13 | 54385284 | T | C | 0.1937 | 0.014 | 0.002 | 1.88E-11 | -0.002 | 0.003 | 4.00E-01 | BMI | 0.017 | 0.012 | 0.016 | | 0.014 | 0.011 | 0.019 |
| rs12874776 | 13 | 57794345 | C | A | 0.893 | -0.001 | 0.003 | 6.51E-01 | -0.023 | 0.004 | 8.00E-11 | PC | -0.010 | 0.016 | -0.008 | | 0.019 | -0.012 | 0.025 |
| rs9527895 | 13 | 59367767 | T | C | 0.819 | -0.016 | 0.002 | 3.69E-12 | 0.001 | 0.003 | 7.80E-01 | BMI | -0.007 | 0.013 | -0.009 | | 0.015 | 0.005 | 0.020 |
| rs8181823 | 13 | 65477940 | A | C | 0.2344 | -0.013 | 0.002 | 4.36E-10 | -0.004 | 0.003 | 1.60E-01 | BMI | 0.002 | 0.011 | 0.013 | | 0.014 | -0.018 | 0.018 |
| rs1441264 | 13 | 79580919 | A | G | 0.5846 | 0.017 | 0.002 | 7.60E-25 | 0.002 | 0.002 | 3.20E-01 | BMI | 0.017 | 0.010 | 0.020 | | 0.012 | 0.010 | 0.016 |
| rs9531786 | 13 | 85983968 | C | G | 0.3715 | -0.011 | 0.002 | 4.11E-10 | -0.006 | 0.002 | 6.70E-03 | BMI | 0.002 | 0.010 | -0.013 | | 0.012 | 0.000 | 0.016 |
| rs77432547 | 13 | 86494817 | A | G | 0.7229 | -0.017 | 0.002 | 1.44E-15 | 0.000 | 0.002 | 9.90E-01 | BMI | -0.014 | 0.011 | -0.014 | | 0.013 | -0.026 | 0.017 |
| rs1927790 | 13 | 96922191 | T | C | 0.606 | -0.014 | 0.002 | 1.57E-17 | -0.004 | 0.002 | 4.70E-02 | BMI | 0.002 | 0.010 | -0.016 | | 0.012 | 0.025 | 0.015 |
| rs9168 | 13 | 99101583 | A | C | 0.2752 | -0.014 | 0.002 | 1.08E-13 | 0.003 | 0.002 | 2.80E-01 | BMI | 0.019 | 0.011 | 0.034 | | 0.013 | 0.012 | 0.017 |
| rs9888533 | 13 | 107854612 | T | C | 0.5302 | 0.013 | 0.002 | 1.38E-10 | -0.001 | 0.002 | 6.50E-01 | BMI | -0.018 | 0.010 | -0.010 | | 0.012 | -0.029 | 0.016 |
| rs9522183 | 13 | 111977280 | T | G | 0.5492 | -0.014 | 0.002 | 2.10E-12 | 0.003 | 0.002 | 1.60E-01 | BMI | -0.017 | 0.010 | -0.014 | | 0.012 | 0.009 | 0.015 |
| rs12868881 | 13 | 112218924 | A | T | 0.4066 | 0.014 | 0.002 | 1.40E-15 | -0.007 | 0.002 | 1.60E-03 | BMI | -0.002 | 0.010 | -0.002 | | 0.012 | 0.008 | 0.015 |
| rs10132280 | 14 | 25928179 | A | C | 0.311 | -0.021 | 0.002 | 2.28E-33 | 0.000 | 0.002 | 9.40E-01 | BMI | 0.010 | 0.012 | 0.008 | | 0.013 | 0.013 | 0.016 |
| rs4981693 | 14 | 29680331 | A | G | 0.7743 | 0.020 | 0.002 | 7.89E-24 | 0.000 | 0.003 | 8.70E-01 | BMI | -0.003 | 0.013 | 0.023 | | 0.014 | 0.000 | 0.018 |
| rs225882 | 14 | 30480123 | T | C | 0.7513 | 0.011 | 0.002 | 8.56E-10 | -0.005 | 0.003 | 5.20E-02 | BMI | -0.008 | 0.012 | -0.017 | | 0.013 | -0.004 | 0.017 |
| rs1958898 | 14 | 40886886 | C | G | 0.2098 | -0.015 | 0.002 | 4.23E-12 | -0.001 | 0.003 | 6.10E-01 | BMI | -0.004 | 0.014 | -0.010 | | 0.015 | 0.001 | 0.019 |
| rs61983990 | 14 | 41475003 | A | G | 0.0802 | 0.020 | 0.004 | 1.91E-08 | -0.005 | 0.004 | 2.60E-01 | BMI | 0.048 | 0.023 | 0.038 | | 0.023 | 0.087 | 0.029 |
| rs198262 | 14 | 57283283 | C | T | 0.044 | -0.001 | 0.004 | 8.31E-01 | 0.030 | 0.005 | 3.90E-08 | PC | -0.023 | 0.030 | 0.015 | | 0.029 | -0.038 | 0.038 |
| rs217669 | 14 | 62360075 | T | C | 0.7224 | -0.017 | 0.002 | 6.27E-16 | -0.002 | 0.002 | 4.80E-01 | BMI | -0.003 | 0.013 | -0.011 | | 0.013 | -0.002 | 0.017 |
| rs4430672 | 14 | 63094407 | T | C | 0.1958 | 0.012 | 0.002 | 5.00E-09 | 0.001 | 0.003 | 8.20E-01 | BMI | 0.004 | 0.014 | 0.012 | | 0.014 | -0.007 | 0.019 |
| rs3902951 | 14 | 69789755 | T | G | 0.7691 | -0.013 | 0.002 | 2.88E-12 | 0.005 | 0.003 | 6.60E-02 | BMI | 0.010 | 0.013 | 0.019 | | 0.014 | 0.013 | 0.018 |
| rs17182027 | 14 | 73348130 | A | G | 0.5647 | -0.011 | 0.002 | 1.46E-10 | 0.002 | 0.002 | 3.10E-01 | BMI | -0.018 | 0.012 | -0.041 | | 0.012 | 0.017 | 0.015 |
| rs7144011 | 14 | 79940383 | T | G | 0.2334 | 0.026 | 0.002 | 2.37E-40 | -0.007 | 0.003 | 5.80E-03 | BMI | 0.009 | 0.015 | 0.002 | | 0.014 | -0.006 | 0.019 |
| rs4517716 | 14 | 83013876 | C | G | 0.7924 | -0.012 | 0.002 | 4.27E-09 | -0.001 | 0.003 | 6.50E-01 | BMI | 0.013 | 0.014 | 0.018 | | 0.014 | 0.004 | 0.019 |
| rs12888545 | 14 | 88308044 | A | G | 0.7464 | -0.013 | 0.002 | 1.76E-11 | -0.002 | 0.003 | 5.50E-01 | BMI | 0.014 | 0.014 | 0.006 | | 0.014 | -0.006 | 0.018 |
| rs1951455 | 14 | 91512339 | T | C | 0.2813 | -0.015 | 0.002 | 6.05E-15 | -0.003 | 0.002 | 2.80E-01 | BMI | 0.001 | 0.013 | -0.006 | | 0.013 | 0.007 | 0.017 |
| rs942066 | 14 | 94031914 | A | G | 0.373 | -0.020 | 0.002 | 2.36E-24 | 0.003 | 0.002 | 2.10E-01 | BMI | -0.006 | 0.012 | 0.012 | | 0.012 | -0.007 | 0.016 |
| rs7161194 | 14 | 101529005 | A | G | 0.3437 | 0.019 | 0.002 | 2.23E-24 | 0.000 | 0.002 | 9.30E-01 | BMI | 0.002 | 0.012 | 0.017 | | 0.013 | -0.016 | 0.017 |
| rs10145749 | 14 | 102782109 | T | C | 0.1549 | 0.016 | 0.003 | 8.33E-10 | -0.007 | 0.003 | 3.70E-02 | BMI | 0.005 | 0.016 | -0.011 | | 0.017 | -0.023 | 0.022 |
| rs4906263 | 14 | 103249127 | C | G | 0.6562 | -0.018 | 0.002 | 8.11E-23 | 0.003 | 0.002 | 1.60E-01 | BMI | 0.012 | 0.012 | 0.005 | | 0.012 | 0.012 | 0.016 |
| rs2273175 | 14 | 104160141 | T | C | 0.6791 | -0.012 | 0.002 | 3.69E-11 | -0.006 | 0.002 | 1.50E-02 | BMI | 0.000 | 0.012 | 0.002 | | 0.012 | -0.026 | 0.016 |
| rs12594043 | 15 | 27034988 | C | G | 0.5205 | 0.010 | 0.002 | 1.31E-09 | 0.001 | 0.002 | 5.50E-01 | BMI | 0.004 | 0.010 | 0.006 | | 0.012 | 0.010 | 0.015 |
| rs7172627 | 15 | 31877690 | A | G | 0.523 | -0.011 | 0.002 | 1.81E-11 | -0.002 | 0.002 | 3.40E-01 | BMI | 0.018 | 0.010 | 0.033 | | 0.012 | 0.021 | 0.015 |
| rs11636611 | 15 | 36391965 | T | C | 0.4981 | 0.010 | 0.002 | 8.85E-10 | -0.001 | 0.002 | 6.70E-01 | BMI | 0.018 | 0.010 | 0.015 | | 0.012 | 0.019 | 0.015 |
| rs9944219 | 15 | 46500612 | A | G | 0.6044 | -0.012 | 0.002 | 8.14E-13 | -0.005 | 0.002 | 3.80E-02 | BMI | -0.003 | 0.010 | -0.003 | | 0.012 | 0.005 | 0.015 |
| rs12912198 | 15 | 47103953 | T | C | 0.2731 | -0.010 | 0.002 | 3.44E-08 | 0.001 | 0.002 | 5.80E-01 | BMI | -0.002 | 0.011 | -0.006 | | 0.013 | -0.002 | 0.017 |
| rs166835 | 15 | 47716037 | C | T | 0.443 | 0.001 | 0.002 | 6.55E-01 | 0.013 | 0.002 | 3.40E-09 | PC | -0.007 | 0.010 | -0.001 | | 0.012 | -0.012 | 0.015 |
| rs6493498 | 15 | 51754451 | T | C | 0.4465 | 0.014 | 0.002 | 4.84E-17 | 0.004 | 0.002 | 8.70E-02 | BMI | 0.023 | 0.010 | 0.023 | | 0.012 | 0.025 | 0.015 |
| rs10518694 | 15 | 53072673 | A | C | 0.1394 | 0.014 | 0.002 | 3.43E-09 | 0.000 | 0.003 | 9.60E-01 | BMI | 0.021 | 0.014 | 0.035 | | 0.017 | 0.002 | 0.022 |
| rs12439632 | 15 | 59103963 | C | G | 0.1682 | 0.015 | 0.002 | 3.68E-10 | 0.007 | 0.003 | 1.50E-02 | BMI | -0.025 | 0.014 | -0.033 | | 0.017 | -0.029 | 0.022 |
| rs339991 | 15 | 60913637 | A | G | 0.4201 | -0.013 | 0.002 | 3.51E-13 | -0.001 | 0.002 | 8.00E-01 | BMI | -0.022 | 0.010 | -0.026 | | 0.012 | -0.025 | 0.015 |
| rs1559673 | 15 | 62156514 | A | C | 0.9688 | 0.036 | 0.005 | 2.21E-13 | 0.004 | 0.008 | 5.80E-01 | BMI | 0.030 | 0.027 | 0.039 | | 0.036 | -0.004 | 0.046 |
| rs2241423 | 15 | 68086838 | A | G | 0.2285 | -0.030 | 0.002 | 3.60E-54 | 0.006 | 0.003 | 3.30E-02 | BMI | -0.044 | 0.011 | -0.043 | | 0.014 | -0.012 | 0.018 |
| rs7171864 | 15 | 73227249 | A | G | 0.6935 | 0.013 | 0.002 | 2.60E-14 | 0.001 | 0.002 | 7.80E-01 | BMI | 0.021 | 0.010 | 0.033 | | 0.012 | 0.009 | 0.016 |
| rs2470893 | 15 | 75019449 | T | C | 0.3049 | 0.011 | 0.002 | 9.43E-10 | -0.005 | 0.002 | 2.30E-02 | BMI | -0.011 | 0.011 | -0.018 | | 0.013 | -0.006 | 0.017 |
| rs56151256 | 15 | 78024806 | A | C | 0.7555 | 0.017 | 0.002 | 6.19E-14 | -0.001 | 0.003 | 7.20E-01 | BMI | -0.012 | 0.011 | -0.022 | | 0.013 | -0.014 | 0.018 |
| rs12914623 | 15 | 80993570 | C | G | 0.2722 | -0.016 | 0.002 | 2.00E-16 | -0.004 | 0.002 | 1.10E-01 | BMI | 0.035 | 0.011 | 0.026 | | 0.013 | 0.048 | 0.017 |
| rs4702 | 15 | 91426560 | G | A | 0.444 | 0.005 | 0.002 | 4.15E-03 | 0.012 | 0.002 | 3.80E-08 | PC | 0.018 | 0.010 | 0.020 | | 0.012 | 0.013 | 0.015 |
| rs72757415 | 15 | 92572762 | T | G | 0.2123 | -0.016 | 0.002 | 1.34E-11 | 0.002 | 0.003 | 5.30E-01 | BMI | -0.009 | 0.012 | -0.007 | | 0.014 | -0.009 | 0.019 |
| rs6496248 | 15 | 98262923 | A | T | 0.6352 | 0.010 | 0.002 | 2.93E-09 | 0.002 | 0.002 | 4.90E-01 | BMI | -0.015 | 0.010 | -0.014 | | 0.012 | -0.022 | 0.016 |
| rs12591120 | 15 | 99236869 | T | C | 0.7397 | 0.012 | 0.002 | 3.84E-08 | 0.000 | 0.003 | 8.50E-01 | BMI | -0.010 | 0.011 | -0.007 | | 0.013 | -0.025 | 0.017 |
| rs2715423 | 15 | 99511873 | A | G | 0.2805 | -0.012 | 0.002 | 1.90E-09 | 0.000 | 0.002 | 9.50E-01 | BMI | -0.010 | 0.011 | -0.009 | | 0.013 | -0.004 | 0.017 |
| rs214249 | 16 | 348687 | T | G | 0.6071 | 0.014 | 0.002 | 2.72E-15 | -0.003 | 0.002 | 1.30E-01 | BMI | 0.010 | 0.010 | 0.004 | | 0.012 | 0.016 | 0.016 |
| rs12448257 | 16 | 3599655 | A | G | 0.2185 | 0.016 | 0.002 | 9.04E-16 | 0.005 | 0.003 | 5.70E-02 | BMI | 0.003 | 0.012 | 0.000 | | 0.015 | -0.016 | 0.019 |
| rs879620 | 16 | 4015729 | T | C | 0.5966 | 0.023 | 0.002 | 8.45E-39 | -0.003 | 0.002 | 1.70E-01 | BMI | 0.010 | 0.010 | 0.004 | | 0.012 | 0.012 | 0.015 |
| rs2058527 | 16 | 6704749 | T | G | 0.2715 | -0.012 | 0.002 | 1.80E-09 | -0.002 | 0.002 | 4.20E-01 | BMI | 0.029 | 0.011 | 0.037 | | 0.013 | 0.046 | 0.017 |
| rs6498759 | 16 | 18020295 | T | C | 0.31 | -0.001 | 0.002 | 7.31E-01 | 0.013 | 0.002 | 2.70E-08 | PC | -0.003 | 0.010 | -0.004 | | 0.012 | 0.000 | 0.016 |
| rs9926784 | 16 | 19941968 | T | C | 0.8007 | 0.024 | 0.002 | 1.08E-30 | 0.000 | 0.003 | 9.40E-01 | BMI | -0.012 | 0.012 | -0.017 | | 0.015 | 0.001 | 0.020 |
| rs194809 | 16 | 23804956 | A | G | 0.1871 | 0.013 | 0.002 | 4.86E-09 | 0.008 | 0.003 | 2.90E-03 | BMI | 0.019 | 0.012 | 0.010 | | 0.014 | 0.020 | 0.019 |
| rs2342892 | 16 | 24540806 | T | G | 0.4855 | 0.013 | 0.002 | 1.33E-13 | 0.000 | 0.002 | 9.40E-01 | BMI | 0.009 | 0.010 | 0.006 | | 0.012 | 0.021 | 0.015 |
| rs7498665 | 16 | 28883241 | A | G | 0.6179 | -0.029 | 0.002 | 1.14E-66 | 0.005 | 0.002 | 1.90E-02 | BMI | -0.011 | 0.010 | -0.016 | | 0.012 | -0.007 | 0.016 |
| rs3814883 | 16 | 29994922 | T | C | 0.4795 | 0.023 | 0.002 | 1.47E-40 | 0.000 | 0.002 | 9.50E-01 | BMI | 0.002 | 0.010 | 0.006 | | 0.012 | -0.015 | 0.015 |
| rs2193101 | 16 | 49035172 | C | G | 0.1947 | 0.014 | 0.002 | 3.33E-11 | 0.002 | 0.003 | 6.00E-01 | BMI | 0.015 | 0.013 | 0.028 | | 0.015 | 0.010 | 0.020 |
| rs16952482 | 16 | 53771583 | T | C | 0.8876 | -0.015 | 0.003 | 3.06E-08 | 0.001 | 0.004 | 7.70E-01 | BMI | 0.028 | 0.016 | 0.037 | | 0.019 | 0.021 | 0.025 |
| rs9937053 | 16 | 53799507 | A | G | 0.4372 | 0.072 | 0.002 | 0.00E+00 | -0.004 | 0.002 | 6.80E-02 | BMI | -0.006 | 0.010 | 0.001 | | 0.012 | -0.008 | 0.015 |
| rs8057911 | 16 | 54143352 | T | C | 0.234 | 0.013 | 0.002 | 3.66E-08 | 0.002 | 0.003 | 4.90E-01 | BMI | 0.006 | 0.012 | -0.008 | | 0.014 | 0.036 | 0.018 |
| rs3730399 | 16 | 67229019 | A | G | 0.935 | -0.011 | 0.004 | 2.45E-03 | 0.025 | 0.004 | 3.30E-08 | PC | -0.054 | 0.020 | -0.075 | | 0.024 | -0.048 | 0.031 |
| rs889398 | 16 | 69556715 | T | C | 0.4151 | -0.020 | 0.002 | 3.23E-32 | -0.002 | 0.002 | 4.90E-01 | BMI | -0.016 | 0.010 | -0.020 | | 0.012 | 0.003 | 0.015 |
| rs35949039 | 16 | 70572605 | T | G | 0.1056 | -0.022 | 0.003 | 6.30E-12 | -0.008 | 0.004 | 2.00E-02 | BMI | 0.024 | 0.016 | 0.023 | | 0.020 | 0.007 | 0.026 |
| rs4390583 | 16 | 81694835 | A | C | 0.5714 | -0.010 | 0.002 | 1.63E-08 | -0.001 | 0.002 | 7.50E-01 | BMI | -0.010 | 0.010 | -0.008 | | 0.012 | -0.015 | 0.015 |
| rs12922346 | 16 | 82438337 | C | G | 0.2641 | 0.013 | 0.002 | 1.48E-11 | 0.001 | 0.003 | 6.80E-01 | BMI | 0.003 | 0.011 | 0.002 | | 0.013 | -0.010 | 0.017 |
| rs7206608 | 16 | 82872628 | C | G | 0.6751 | -0.013 | 0.002 | 1.20E-12 | -0.003 | 0.002 | 2.60E-01 | BMI | -0.028 | 0.011 | -0.029 | | 0.013 | -0.032 | 0.016 |
| rs3923783 | 17 | 1843189 | A | C | 0.1784 | -0.022 | 0.002 | 4.12E-23 | 0.003 | 0.003 | 3.20E-01 | BMI | -0.003 | 0.013 | -0.008 | | 0.015 | -0.025 | 0.020 |
| rs11078883 | 17 | 2138828 | C | G | 0.654 | -0.013 | 0.002 | 1.30E-12 | -0.003 | 0.002 | 2.00E-01 | BMI | 0.002 | 0.010 | 0.004 | | 0.012 | 0.003 | 0.016 |
| rs7774 | 17 | 4801163 | A | C | 0.3197 | 0.013 | 0.002 | 4.22E-10 | 0.001 | 0.002 | 5.90E-01 | BMI | -0.010 | 0.010 | -0.011 | | 0.013 | -0.014 | 0.016 |
| rs7209653 | 17 | 19882084 | T | C | 0.704 | 0.003 | 0.002 | 1.01E-01 | 0.016 | 0.002 | 9.10E-11 | PC | -0.006 | 0.011 | -0.016 | | 0.013 | 0.011 | 0.017 |
| rs4986044 | 17 | 21261560 | T | C | 0.455 | -0.018 | 0.002 | 1.27E-27 | -0.002 | 0.002 | 2.70E-01 | BMI | -0.018 | 0.010 | -0.021 | | 0.012 | 0.002 | 0.015 |
| rs8065172 | 17 | 31456969 | A | G | 0.2383 | -0.012 | 0.002 | 5.29E-10 | -0.003 | 0.003 | 2.90E-01 | BMI | 0.025 | 0.011 | 0.023 | | 0.013 | 0.023 | 0.018 |
| rs12603813 | 17 | 43196584 | T | C | 0.746 | -0.001 | 0.002 | 6.96E-01 | 0.014 | 0.003 | 1.30E-08 | PC | -0.003 | 0.011 | 0.012 | | 0.013 | -0.019 | 0.017 |
| rs56161855 | 17 | 46288649 | A | T | 0.8678 | -0.023 | 0.003 | 1.41E-16 | 0.001 | 0.003 | 7.90E-01 | BMI | -0.021 | 0.013 | -0.029 | | 0.016 | -0.011 | 0.021 |
| rs11649864 | 17 | 56093061 | A | G | 0.0859 | 0.019 | 0.003 | 2.52E-10 | -0.010 | 0.004 | 1.30E-02 | BMI | 0.006 | 0.017 | 0.021 | | 0.021 | -0.015 | 0.027 |
| rs12602912 | 17 | 65870073 | T | C | 0.212 | 0.017 | 0.002 | 2.90E-16 | 0.003 | 0.003 | 2.90E-01 | BMI | 0.008 | 0.012 | 0.010 | | 0.014 | 0.012 | 0.019 |
| rs312750 | 17 | 68343539 | A | G | 0.4986 | 0.010 | 0.002 | 2.57E-09 | -0.004 | 0.002 | 9.20E-02 | BMI | 0.002 | 0.010 | -0.005 | | 0.012 | 0.001 | 0.015 |
| rs35867081 | 17 | 79047278 | A | G | 0.4871 | 0.015 | 0.002 | 6.65E-15 | 0.001 | 0.002 | 5.30E-01 | BMI | -0.003 | 0.010 | 0.003 | | 0.012 | -0.020 | 0.016 |
| rs66643547 | 17 | 79375315 | C | T | 0.647 | 0.002 | 0.002 | 2.59E-01 | -0.015 | 0.002 | 3.20E-11 | PC | 0.011 | 0.010 | 0.009 | | 0.012 | 0.016 | 0.016 |
| rs8097672 | 18 | 1839601 | A | T | 0.8545 | -0.021 | 0.002 | 3.26E-18 | 0.008 | 0.003 | 8.50E-03 | BMI | -0.010 | 0.014 | 0.001 | | 0.016 | -0.020 | 0.021 |
| rs478707 | 18 | 7543207 | T | C | 0.2032 | -0.015 | 0.002 | 1.98E-10 | -0.001 | 0.003 | 8.60E-01 | BMI | 0.006 | 0.012 | -0.006 | | 0.014 | 0.008 | 0.019 |
| rs891387 | 18 | 21103909 | T | C | 0.4951 | 0.021 | 0.002 | 9.26E-35 | -0.007 | 0.002 | 6.80E-04 | BMI | 0.017 | 0.010 | 0.012 | | 0.012 | 0.027 | 0.015 |
| rs1945160 | 18 | 22164216 | A | G | 0.3819 | -0.010 | 0.002 | 5.65E-09 | 0.002 | 0.002 | 4.60E-01 | BMI | -0.003 | 0.010 | 0.004 | | 0.012 | -0.009 | 0.016 |
| rs1941696 | 18 | 31252129 | A | G | 0.5174 | 0.011 | 0.002 | 4.74E-11 | 0.004 | 0.002 | 8.50E-02 | BMI | -0.003 | 0.010 | 0.003 | | 0.012 | -0.011 | 0.015 |
| rs474605 | 18 | 39612720 | A | G | 0.4679 | -0.013 | 0.002 | 2.72E-13 | 0.001 | 0.002 | 6.40E-01 | BMI | 0.005 | 0.010 | 0.009 | | 0.012 | -0.006 | 0.015 |
| rs1356506 | 18 | 40708038 | T | C | 0.6287 | 0.014 | 0.002 | 8.39E-15 | 0.004 | 0.002 | 9.50E-02 | BMI | 0.018 | 0.010 | 0.030 | | 0.012 | 0.016 | 0.016 |
| rs2612576 | 18 | 42967950 | A | T | 0.2945 | -0.011 | 0.002 | 8.49E-09 | -0.003 | 0.002 | 2.80E-01 | BMI | -0.010 | 0.011 | -0.008 | | 0.013 | 0.004 | 0.017 |
| rs784256 | 18 | 53398626 | G | A | 0.188 | -0.015 | 0.002 | 5.35E-11 | 0.019 | 0.003 | 6.20E-11 | PC | 0.011 | 0.013 | 0.006 | | 0.015 | 0.006 | 0.020 |
| rs9951619 | 18 | 56882326 | T | G | 0.2239 | -0.015 | 0.002 | 2.33E-15 | -0.001 | 0.003 | 7.90E-01 | BMI | -0.002 | 0.011 | 0.001 | | 0.013 | -0.013 | 0.018 |
| rs6567160 | 18 | 57829135 | T | C | 0.7518 | -0.055 | 0.002 | 7.82E-184 | -0.004 | 0.003 | 1.60E-01 | BMI | -0.019 | 0.011 | -0.011 | | 0.014 | -0.023 | 0.018 |
| rs17066856 | 18 | 58049656 | T | C | 0.8915 | 0.035 | 0.003 | 7.10E-38 | 0.006 | 0.004 | 1.40E-01 | BMI | 0.023 | 0.017 | 0.018 | | 0.020 | 0.040 | 0.026 |
| rs17783165 | 18 | 63461638 | T | C | 0.671 | -0.013 | 0.002 | 2.74E-13 | 0.003 | 0.002 | 2.20E-01 | BMI | -0.007 | 0.010 | -0.009 | | 0.012 | 0.008 | 0.016 |
| rs11150911 | 18 | 73498528 | A | C | 0.2848 | 0.012 | 0.002 | 3.72E-11 | -0.005 | 0.002 | 3.10E-02 | BMI | -0.005 | 0.010 | -0.013 | | 0.013 | 0.008 | 0.016 |
| rs12981256 | 19 | 1865901 | A | G | 0.5284 | 0.015 | 0.002 | 1.43E-18 | -0.005 | 0.002 | 4.00E-02 | BMI | 0.006 | 0.010 | 0.012 | | 0.012 | -0.001 | 0.016 |
| rs45486197 | 19 | 2244849 | A | G | 0.0635 | 0.028 | 0.004 | 1.80E-12 | 0.007 | 0.004 | 1.20E-01 | BMI | -0.002 | 0.024 | -0.015 | | 0.028 | -0.054 | 0.037 |
| rs895330 | 19 | 4060707 | C | G | 0.806 | 0.020 | 0.002 | 1.59E-19 | 0.005 | 0.003 | 5.10E-02 | BMI | -0.032 | 0.012 | -0.021 | | 0.015 | -0.042 | 0.019 |
| rs273512 | 19 | 18224729 | T | C | 0.409 | 0.016 | 0.002 | 4.48E-19 | 0.002 | 0.002 | 3.20E-01 | BMI | -0.008 | 0.010 | -0.009 | | 0.012 | 0.004 | 0.015 |
| rs17724992 | 19 | 18454825 | A | G | 0.7197 | 0.017 | 0.002 | 5.23E-21 | -0.006 | 0.002 | 1.20E-02 | BMI | 0.006 | 0.011 | 0.000 | | 0.013 | 0.007 | 0.017 |
| rs7258722 | 19 | 18808915 | A | T | 0.4131 | -0.020 | 0.002 | 1.81E-30 | 0.001 | 0.002 | 6.60E-01 | BMI | -0.023 | 0.010 | -0.032 | | 0.012 | -0.024 | 0.016 |
| rs12611148 | 19 | 19865077 | A | C | 0.1429 | -0.014 | 0.002 | 1.42E-08 | -0.001 | 0.003 | 8.20E-01 | BMI | 0.012 | 0.013 | 0.007 | | 0.016 | 0.017 | 0.021 |
| rs12462975 | 19 | 30272202 | A | G | 0.3243 | 0.019 | 0.002 | 1.47E-25 | 0.003 | 0.002 | 2.00E-01 | BMI | 0.025 | 0.010 | 0.028 | | 0.012 | 0.028 | 0.016 |
| rs7245985 | 19 | 30710410 | T | G | 0.7956 | 0.012 | 0.002 | 1.88E-08 | 0.004 | 0.003 | 1.80E-01 | BMI | 0.015 | 0.012 | 0.021 | | 0.014 | 0.002 | 0.019 |
| rs11882409 | 19 | 34019685 | A | C | 0.303 | 0.012 | 0.002 | 3.31E-10 | 0.009 | 0.002 | 4.00E-04 | BMI | -0.020 | 0.011 | -0.026 | | 0.013 | -0.021 | 0.017 |
| rs185350 | 19 | 34306816 | T | C | 0.4902 | 0.014 | 0.002 | 9.10E-17 | -0.001 | 0.002 | 6.00E-01 | BMI | 0.009 | 0.010 | 0.015 | | 0.012 | 0.003 | 0.015 |
| rs429358 | 19 | 45411941 | T | C | 0.8459 | 0.026 | 0.003 | 2.60E-22 | 0.011 | 0.003 | 2.60E-04 | BMI | 0.005 | 0.014 | 0.017 | | 0.017 | 0.007 | 0.022 |
| rs11672660 | 19 | 46180184 | T | C | 0.1871 | -0.034 | 0.002 | 6.83E-60 | -0.004 | 0.003 | 1.80E-01 | BMI | 0.005 | 0.012 | 0.002 | | 0.014 | 0.007 | 0.019 |
| rs9304665 | 19 | 47602577 | A | T | 0.7423 | 0.023 | 0.002 | 2.20E-31 | 0.004 | 0.003 | 1.10E-01 | BMI | -0.003 | 0.012 | -0.005 | | 0.014 | 0.001 | 0.018 |
| rs3764625 | 19 | 49649051 | T | G | 0.3989 | 0.010 | 0.002 | 2.42E-08 | -0.004 | 0.002 | 5.90E-02 | BMI | 0.005 | 0.010 | 0.001 | | 0.012 | -0.015 | 0.015 |
| rs1884389 | 20 | 1410582 | T | C | 0.4386 | -0.011 | 0.002 | 3.72E-10 | -0.002 | 0.002 | 3.30E-01 | BMI | -0.002 | 0.010 | 0.006 | | 0.012 | -0.015 | 0.015 |
| rs4813619 | 20 | 2815715 | T | G | 0.5305 | -0.011 | 0.002 | 1.70E-09 | -0.005 | 0.002 | 1.90E-02 | BMI | 0.001 | 0.010 | 0.000 | | 0.012 | 0.003 | 0.015 |
| rs1884897 | 20 | 6612832 | A | G | 0.3747 | -0.018 | 0.002 | 2.70E-28 | -0.001 | 0.002 | 6.40E-01 | BMI | 0.053 | 0.010 | 0.037 | | 0.012 | 0.063 | 0.016 |
| rs2423668 | 20 | 12430673 | T | C | 0.4196 | 0.011 | 0.002 | 7.84E-09 | 0.001 | 0.002 | 5.50E-01 | BMI | -0.005 | 0.010 | -0.005 | | 0.012 | -0.006 | 0.015 |
| rs852056 | 20 | 17102860 | T | C | 0.2538 | 0.012 | 0.002 | 2.15E-10 | 0.005 | 0.003 | 5.60E-02 | BMI | -0.002 | 0.011 | 0.015 | | 0.014 | -0.009 | 0.018 |
| rs1409818 | 20 | 21381121 | T | C | 0.1074 | 0.020 | 0.003 | 2.59E-12 | 0.003 | 0.004 | 4.90E-01 | BMI | 0.011 | 0.016 | 0.021 | | 0.019 | -0.034 | 0.025 |
| rs8122855 | 20 | 25192049 | A | G | 0.3377 | 0.014 | 0.002 | 4.11E-14 | 0.003 | 0.002 | 2.10E-01 | BMI | -0.018 | 0.010 | -0.010 | | 0.012 | -0.025 | 0.016 |
| rs4812405 | 20 | 35276585 | A | C | 0.0763 | -0.020 | 0.003 | 1.28E-09 | -0.004 | 0.004 | 2.90E-01 | BMI | -0.025 | 0.021 | -0.048 | | 0.026 | 0.029 | 0.033 |
| rs742748 | 20 | 39293397 | T | C | 0.5864 | -0.011 | 0.002 | 1.13E-11 | 0.000 | 0.002 | 8.50E-01 | BMI | -0.001 | 0.010 | 0.004 | | 0.012 | -0.009 | 0.015 |
| rs79186842 | 20 | 47689036 | A | G | 0.8597 | 0.020 | 0.003 | 2.60E-13 | 0.007 | 0.003 | 2.40E-02 | BMI | -0.013 | 0.015 | -0.023 | | 0.017 | 0.009 | 0.023 |
| rs17806224 | 20 | 51065854 | A | G | 0.1808 | -0.026 | 0.002 | 7.91E-32 | -0.007 | 0.003 | 1.00E-02 | BMI | -0.023 | 0.013 | -0.026 | | 0.015 | -0.022 | 0.020 |
| rs6129084 | 20 | 59828857 | A | T | 0.359 | -0.005 | 0.002 | 1.68E-02 | -0.014 | 0.002 | 9.30E-10 | PC | 0.015 | 0.010 | 0.003 | | 0.012 | 0.051 | 0.016 |
| rs6010784 | 20 | 61540319 | T | C | 0.5062 | 0.011 | 0.002 | 6.91E-11 | 0.010 | 0.002 | 5.40E-06 | BMI | 0.003 | 0.010 | -0.002 | | 0.012 | -0.006 | 0.015 |
| rs2064044 | 21 | 22119890 | A | C | 0.8062 | -0.012 | 0.002 | 1.02E-08 | 0.003 | 0.003 | 2.80E-01 | BMI | -0.017 | 0.012 | -0.022 | | 0.014 | 0.005 | 0.019 |
| rs2832283 | 21 | 30690558 | A | G | 0.2254 | 0.012 | 0.002 | 4.72E-09 | -0.003 | 0.003 | 2.90E-01 | BMI | -0.031 | 0.012 | -0.039 | | 0.014 | -0.015 | 0.018 |
| rs13047416 | 21 | 40309436 | C | G | 0.6263 | 0.015 | 0.002 | 6.93E-18 | 0.000 | 0.002 | 8.50E-01 | BMI | -0.017 | 0.010 | -0.003 | | 0.012 | -0.038 | 0.016 |
| rs8134638 | 21 | 40644170 | T | C | 0.6273 | -0.013 | 0.002 | 1.57E-11 | 0.003 | 0.002 | 2.20E-01 | BMI | 0.000 | 0.010 | -0.002 | | 0.012 | 0.018 | 0.016 |
| rs8126575 | 21 | 46435610 | T | G | 0.8673 | 0.015 | 0.003 | 5.88E-10 | -0.001 | 0.003 | 7.20E-01 | BMI | -0.006 | 0.015 | -0.009 | | 0.018 | -0.001 | 0.024 |
| rs427943 | 21 | 46570896 | A | C | 0.4288 | -0.018 | 0.002 | 3.60E-25 | 0.001 | 0.002 | 5.00E-01 | BMI | 0.006 | 0.010 | 0.008 | | 0.012 | 0.016 | 0.015 |
| rs11538 | 22 | 18220831 | A | G | 0.8284 | -0.014 | 0.002 | 1.09E-09 | 0.002 | 0.003 | 4.90E-01 | BMI | 0.000 | 0.013 | -0.013 | | 0.016 | -0.017 | 0.020 |
| rs136553 | 22 | 27255675 | C | T | 0.624 | -0.007 | 0.002 | 3.19E-04 | -0.015 | 0.002 | 2.30E-11 | PC | 0.014 | 0.010 | 0.007 | | 0.012 | 0.011 | 0.016 |
| rs7288455 | 22 | 39966547 | A | G | 0.436 | -0.001 | 0.002 | 5.60E-01 | 0.013 | 0.002 | 4.00E-09 | PC | -0.002 | 0.010 | 0.006 | | 0.012 | -0.010 | 0.016 |
| rs12628051 | 22 | 40654276 | T | C | 0.6407 | 0.016 | 0.002 | 2.90E-19 | -0.002 | 0.002 | 3.30E-01 | BMI | 0.019 | 0.010 | 0.012 | | 0.012 | 0.024 | 0.016 |
| **2) Analysis of colorectal cancer in men only** | | | | | | | | | | | | | | | | | | | |
| SNP | CHR | POS | EA | OA | EAF | beta_bmi | se_bmi | pval_bmi | beta_pc | se_pc | pval_pc | phenotype | beta_crc_men | se_crc_men | |  |  |  |  |
| rs2748985 | 1 | 1853184 | T | C | 0.455 | -0.0134 | 0.0029 | 3.29E-06 | -0.01534 | 0.002213 | 4.1E-12 | PC | 0.0168 | 0.0136 |  | |  |  |  |
| rs3762444 | 1 | 2427712 | T | C | 0.4607 | -0.0194 | 0.0025 | 1.47E-14 | -0.00302 | 0.002223 | 0.17 | BMI | -0.0213 | 0.0143 |  | |  |  |  |
| rs11121210 | 1 | 8708529 | T | C | 0.3424 | -0.0149 | 0.0026 | 7.86E-09 | 0.006937 | 0.0023 | 0.0026 | BMI | -0.0168 | 0.014 |  | |  |  |  |
| rs11581010 | 1 | 11212458 | A | G | 0.7632 | -0.0178 | 0.0029 | 1.01E-09 | -0.00411 | 0.002588 | 0.11 | BMI | 0.0034 | 0.0159 |  | |  |  |  |
| rs2235549 | 1 | 23391775 | T | G | 0.8395 | -0.0213 | 0.0033 | 8.27E-11 | 0.00979 | 0.002972 | 0.00099 | BMI | -0.0466 | 0.0181 |  | |  |  |  |
| rs2271928 | 1 | 32127953 | A | G | 0.3751 | -0.0136 | 0.0024 | 9.92E-09 | -0.00136 | 0.002232 | 0.54 | BMI | 0.0098 | 0.0137 |  | |  |  |  |
| rs12061406 | 1 | 40073479 | T | C | 0.0779 | -0.0273 | 0.0045 | 1.7E-09 | -0.00314 | 0.003945 | 0.43 | BMI | 0.0328 | 0.0255 |  | |  |  |  |
| rs1768808 | 1 | 46503217 | T | C | 0.6241 | 0.0143 | 0.0026 | 3.54E-08 | -0.00074 | 0.0023 | 0.75 | BMI | -0.0113 | 0.0144 |  | |  |  |  |
| rs977747 | 1 | 47684677 | T | G | 0.4386 | 0.0159 | 0.0024 | 1.35E-11 | 0.003652 | 0.002234 | 0.1 | BMI | -0.0163 | 0.0141 |  | |  |  |  |
| rs7531656 | 1 | 49828663 | A | G | 0.3299 | 0.0198 | 0.0025 | 2.32E-15 | -0.00263 | 0.002351 | 0.26 | BMI | 0.0199 | 0.0144 |  | |  |  |  |
| rs2481665 | 1 | 62594677 | T | C | 0.5827 | 0.0187 | 0.0024 | 2.03E-15 | -0.00375 | 0.002212 | 0.09 | BMI | -0.02 | 0.0138 |  | |  |  |  |
| rs11208779 | 1 | 66417145 | C | G | 0.516 | 0.0192 | 0.0028 | 1.53E-11 | -0.00039 | 0.00221 | 0.86 | BMI | -0.0123 | 0.0136 |  | |  |  |  |
| rs1993709 | 1 | 72838529 | A | G | 0.1779 | -0.0413 | 0.003 | 1.49E-42 | -0.00043 | 0.002764 | 0.88 | BMI | -0.0112 | 0.0184 |  | |  |  |  |
| rs12566985 | 1 | 75002193 | A | G | 0.5584 | -0.0181 | 0.0023 | 1.09E-14 | 0.004057 | 0.002219 | 0.068 | BMI | -0.0118 | 0.0138 |  | |  |  |  |
| rs12049202 | 1 | 77967523 | T | C | 0.1858 | 0.0226 | 0.0031 | 5.52E-13 | 0.004581 | 0.002755 | 0.096 | BMI | -0.0074 | 0.0173 |  | |  |  |  |
| rs12034762 | 1 | 96290845 | T | C | 0.3923 | 0.0168 | 0.0026 | 4.8E-11 | 0.000551 | 0.002258 | 0.81 | BMI | -0.0133 | 0.0139 |  | |  |  |  |
| rs11165643 | 1 | 96924097 | T | C | 0.5793 | 0.0201 | 0.0024 | 1.69E-17 | 0.003409 | 0.002237 | 0.13 | BMI | -0.0244 | 0.0137 |  | |  |  |  |
| rs12145677 | 1 | 110023610 | G | A | 0.702 | -0.0114 | 0.0026 | 1.18E-05 | -0.01708 | 0.002405 | 1.2E-12 | PC | 0.0116 | 0.0152 |  | |  |  |  |
| rs61813324 | 1 | 156049877 | T | C | 0.1317 | 0.0276 | 0.0043 | 1.29E-10 | -0.00021 | 0.003257 | 0.95 | BMI | 0.0374 | 0.0236 |  | |  |  |  |
| rs76194251 | 1 | 173931908 | C | G | 0.0233 | -0.0599 | 0.0096 | 4.04E-10 | -0.00366 | 0.007373 | 0.62 | BMI | 0.0241 | 0.0464 |  | |  |  |  |
| rs543874 | 1 | 177889480 | A | G | 0.7749 | -0.036 | 0.0029 | 3.7E-35 | 0.002035 | 0.00271 | 0.45 | BMI | 0.0132 | 0.0173 |  | |  |  |  |
| rs10920678 | 1 | 190239907 | A | G | 0.4222 | 0.0133 | 0.0024 | 1.9E-08 | 0.000647 | 0.002217 | 0.77 | BMI | -0.0288 | 0.0137 |  | |  |  |  |
| rs7526456 | 1 | 197773734 | T | C | 0.413 | 0.0028 | 0.0025 | 0.2666 | -0.0127 | 0.00223 | 1.2E-08 | PC | -0.001 | 0.0138 |  | |  |  |  |
| rs2172935 | 1 | 201826340 | T | C | 0.3213 | 0.0267 | 0.0026 | 3.56E-24 | -0.00566 | 0.002324 | 0.015 | BMI | 0.0306 | 0.0148 |  | |  |  |  |
| rs823094 | 1 | 205689807 | T | G | 0.5526 | 0.0148 | 0.0024 | 3.77E-10 | 0.0038 | 0.002229 | 0.088 | BMI | 0.0405 | 0.014 |  | |  |  |  |
| rs4653942 | 1 | 228505204 | A | G | 0.209 | -0.016 | 0.0029 | 4.41E-08 | 0.003706 | 0.00283 | 0.19 | BMI | 0.0158 | 0.0164 |  | |  |  |  |
| rs2125231 | 1 | 243762299 | T | C | 0.3229 | 0.0171 | 0.0026 | 5.5E-11 | -0.00459 | 0.002366 | 0.052 | BMI | -0.0054 | 0.0144 |  | |  |  |  |
| rs76111507 | 2 | 455477 | T | C | 0.0355 | -0.0811 | 0.0077 | 1.13E-25 | -0.01062 | 0.005826 | 0.068 | BMI | -0.0379 | 0.0421 |  | |  |  |  |
| rs12714415 | 2 | 651430 | T | C | 0.8495 | 0.0604 | 0.0032 | 1.09E-80 | 0.008277 | 0.003031 | 0.0063 | BMI | 0.031 | 0.0184 |  | |  |  |  |
| rs2693826 | 2 | 6160943 | A | G | 0.4287 | -0.0164 | 0.0025 | 4.96E-11 | 0.002046 | 0.002219 | 0.36 | BMI | -0.0064 | 0.0136 |  | |  |  |  |
| rs10182181 | 2 | 25150296 | A | G | 0.5048 | -0.0273 | 0.0023 | 5.55E-32 | -0.00352 | 0.0022 | 0.11 | BMI | 0.0024 | 0.0135 |  | |  |  |  |
| rs1260326 | 2 | 27730940 | T | C | 0.3924 | -0.0183 | 0.0024 | 2.05E-14 | -0.00137 | 0.00225 | 0.54 | BMI | -0.047 | 0.0138 |  | |  |  |  |
| rs2888172 | 2 | 35439812 | A | G | 0.5467 | -0.0141 | 0.0025 | 1.42E-08 | 0.00063 | 0.002217 | 0.78 | BMI | 0.0075 | 0.0138 |  | |  |  |  |
| rs10185199 | 2 | 40282202 | A | G | 0.2868 | -0.0159 | 0.0029 | 3.79E-08 | -0.0019 | 0.002517 | 0.45 | BMI | 0.0073 | 0.0155 |  | |  |  |  |
| rs7561278 | 2 | 48954905 | T | C | 0.7636 | 0.0175 | 0.0031 | 1.06E-08 | -0.00093 | 0.00268 | 0.73 | BMI | -0.0035 | 0.0165 |  | |  |  |  |
| rs12986742 | 2 | 58975143 | T | C | 0.5174 | -0.0197 | 0.0025 | 3.22E-15 | -0.00198 | 0.002215 | 0.37 | BMI | 0.0137 | 0.0135 |  | |  |  |  |
| rs887912 | 2 | 59302877 | T | C | 0.2987 | 0.0229 | 0.0026 | 2.75E-19 | -0.0014 | 0.002405 | 0.56 | BMI | 0.0032 | 0.0152 |  | |  |  |  |
| rs11685481 | 2 | 67736749 | T | C | 0.8499 | 0.0197 | 0.0034 | 8.99E-09 | -0.00121 | 0.003027 | 0.69 | BMI | -0.015 | 0.0191 |  | |  |  |  |
| rs4410328 | 2 | 69600683 | T | C | 0.4108 | -0.0199 | 0.0029 | 7.75E-12 | -0.00309 | 0.002238 | 0.17 | BMI | 0.01 | 0.0137 |  | |  |  |  |
| rs10209325 | 2 | 86806356 | T | C | 0.6212 | -0.0178 | 0.0029 | 1.49E-09 | -0.00059 | 0.002269 | 0.79 | BMI | -0.0091 | 0.0142 |  | |  |  |  |
| rs1437377 | 2 | 100744924 | T | G | 0.8539 | -0.0244 | 0.0036 | 1.47E-11 | 0.001498 | 0.003246 | 0.64 | BMI | -0.04 | 0.02 |  | |  |  |  |
| rs10197031 | 2 | 105454590 | T | C | 0.7292 | -0.0168 | 0.0027 | 7.69E-10 | 0.007083 | 0.002433 | 0.0036 | BMI | 0.0042 | 0.0153 |  | |  |  |  |
| rs13405033 | 2 | 113994595 | T | C | 0.1521 | 0.0263 | 0.004 | 4.37E-11 | -0.00103 | 0.003058 | 0.74 | BMI | 0.0459 | 0.0196 |  | |  |  |  |
| rs11687120 | 2 | 119579558 | A | G | 0.4462 | 0.0137 | 0.0025 | 4.74E-08 | -0.00175 | 0.002211 | 0.43 | BMI | -0.0168 | 0.0138 |  | |  |  |  |
| rs961360 | 2 | 136393658 | A | G | 0.8898 | 0.0213 | 0.0034 | 3.75E-10 | 0.001627 | 0.003547 | 0.65 | BMI | -0.0228 | 0.0172 |  | |  |  |  |
| rs10754920 | 2 | 146456888 | C | G | 0.112 | 0.0055 | 0.0043 | 0.2034 | 0.020241 | 0.00349 | 6.7E-09 | PC | -0.0119 | 0.0202 |  | |  |  |  |
| rs6744254 | 2 | 155986195 | C | T | 0.471 | -0.007 | 0.0025 | 0.004607 | -0.0159 | 0.002204 | 5.4E-13 | PC | 0.0406 | 0.0136 |  | |  |  |  |
| rs34234296 | 2 | 175166636 | A | G | 0.3877 | -0.0176 | 0.003 | 2.86E-09 | -0.00533 | 0.002276 | 0.019 | BMI | -0.011 | 0.0147 |  | |  |  |  |
| rs1019612 | 2 | 181602526 | T | C | 0.6635 | 0.0179 | 0.0026 | 1.4E-11 | -0.00099 | 0.002326 | 0.67 | BMI | 0.0036 | 0.0144 |  | |  |  |  |
| rs10497807 | 2 | 198585087 | C | G | 0.5024 | -0.0146 | 0.0023 | 4.95E-10 | 0.01072 | 0.002206 | 1.2E-06 | BMI | 0.0082 | 0.0136 |  | |  |  |  |
| rs11274218 | 2 | 215035001 | C | T | 0.611 | 0.0052 | 0.0025 | 0.04163 | -0.01375 | 0.002308 | 2.5E-09 | PC | 0.0047 | 0.0139 |  | |  |  |  |
| rs1541777 | 2 | 219587291 | A | G | 0.5514 | -0.0156 | 0.0023 | 1.82E-11 | -0.00082 | 0.0022 | 0.71 | BMI | -0.001 | 0.0139 |  | |  |  |  |
| rs10208088 | 2 | 221055873 | C | T | 0.419 | 0.0055 | 0.0029 | 0.05843 | 0.013103 | 0.002232 | 4.4E-09 | PC | 0.007 | 0.0138 |  | |  |  |  |
| rs2596902 | 3 | 12955982 | A | G | 0.6501 | -0.015 | 0.0026 | 1.3E-08 | -0.00091 | 0.002343 | 0.7 | BMI | 0.011 | 0.0144 |  | |  |  |  |
| rs6804842 | 3 | 25106437 | A | G | 0.4295 | -0.0182 | 0.0024 | 1.29E-14 | 0.001243 | 0.002229 | 0.58 | BMI | 0.012 | 0.0136 |  | |  |  |  |
| rs762318 | 3 | 38516369 | A | C | 0.6276 | 0.0153 | 0.0026 | 2.13E-09 | 0.003981 | 0.002255 | 0.078 | BMI | -0.0014 | 0.0138 |  | |  |  |  |
| rs1524887 | 3 | 44016371 | T | C | 0.4191 | -0.0139 | 0.0025 | 3.6E-08 | -0.00203 | 0.00223 | 0.36 | BMI | 0.0172 | 0.0137 |  | |  |  |  |
| rs11708955 | 3 | 49540114 | T | C | 0.695 | 0.0124 | 0.0027 | 3.85E-06 | -0.01575 | 0.002387 | 4.2E-11 | PC | 0.0071 | 0.0147 |  | |  |  |  |
| rs6774533 | 3 | 62471086 | C | T | 0.294 | -0.0082 | 0.0032 | 0.01038 | -0.01494 | 0.002458 | 1.2E-09 | PC | -0.0081 | 0.0152 |  | |  |  |  |
| rs13078509 | 3 | 70881429 | T | C | 0.0275 | 0.0481 | 0.0084 | 8.37E-09 | -0.00857 | 0.00723 | 0.24 | BMI | 0.0599 | 0.0479 |  | |  |  |  |
| rs3849570 | 3 | 81792112 | A | C | 0.3544 | 0.0149 | 0.0025 | 2.56E-09 | -0.00542 | 0.002309 | 0.019 | BMI | -0.0071 | 0.0143 |  | |  |  |  |
| rs12495178 | 3 | 85886077 | T | C | 0.6413 | 0.0226 | 0.0024 | 1.54E-20 | -0.00244 | 0.002298 | 0.29 | BMI | -0.0291 | 0.0141 |  | |  |  |  |
| rs73140133 | 3 | 88067498 | T | C | 0.3287 | -0.0213 | 0.003 | 2.56E-12 | 0.000972 | 0.002326 | 0.68 | BMI | -0.0289 | 0.0145 |  | |  |  |  |
| rs1492014 | 3 | 94071481 | T | C | 0.5681 | -0.0213 | 0.0025 | 1.64E-17 | 0.002406 | 0.002216 | 0.28 | BMI | 0.022 | 0.0139 |  | |  |  |  |
| rs1436351 | 3 | 104617973 | T | G | 0.7372 | 0.0178 | 0.0027 | 3.39E-11 | -0.00368 | 0.002531 | 0.15 | BMI | -0.0044 | 0.0155 |  | |  |  |  |
| rs6768228 | 3 | 108120008 | A | G | 0.6323 | -0.0149 | 0.0026 | 6.77E-09 | 0.004068 | 0.002276 | 0.074 | BMI | -0.014 | 0.0142 |  | |  |  |  |
| rs2918217 | 3 | 115546380 | T | C | 0.1526 | 0.0221 | 0.0036 | 7.18E-10 | 0.002181 | 0.003186 | 0.49 | BMI | -0.0234 | 0.0198 |  | |  |  |  |
| rs78082503 | 3 | 116556913 | G | C | 0.786 | -0.0019 | 0.0035 | 0.5922 | -0.01536 | 0.002688 | 1.1E-08 | PC | -0.0198 | 0.0162 |  | |  |  |  |
| rs1320903 | 3 | 131758077 | A | G | 0.309 | 0.0255 | 0.0027 | 1.65E-21 | -0.00163 | 0.002363 | 0.49 | BMI | 0.0033 | 0.0146 |  | |  |  |  |
| rs645040 | 3 | 135926622 | T | G | 0.7697 | 0.0187 | 0.0028 | 3.16E-11 | 0.00644 | 0.002627 | 0.014 | BMI | -0.0022 | 0.0163 |  | |  |  |  |
| rs2035935 | 3 | 141306013 | A | G | 0.9243 | -0.0333 | 0.0046 | 3.99E-13 | -0.00616 | 0.004409 | 0.16 | BMI | 0.0308 | 0.0258 |  | |  |  |  |
| rs355777 | 3 | 154034950 | C | G | 0.3968 | 0.017 | 0.0025 | 1.81E-11 | -0.00352 | 0.002245 | 0.12 | BMI | -0.008 | 0.0137 |  | |  |  |  |
| rs9870575 | 3 | 156296559 | A | G | 0.1409 | -0.0237 | 0.0035 | 1.48E-11 | 0.000574 | 0.003072 | 0.85 | BMI | -0.037 | 0.0194 |  | |  |  |  |
| rs7356034 | 3 | 170732599 | A | G | 0.2854 | 0.0218 | 0.0032 | 6.24E-12 | 0.001294 | 0.002444 | 0.6 | BMI | -0.0158 | 0.0149 |  | |  |  |  |
| rs2606227 | 3 | 183536836 | T | C | 0.3679 | 0.0142 | 0.0026 | 4.61E-08 | -0.00542 | 0.002307 | 0.019 | BMI | -0.0137 | 0.0145 |  | |  |  |  |
| rs9816226 | 3 | 185834499 | A | T | 0.1738 | -0.0341 | 0.0031 | 5.05E-29 | 0.001952 | 0.002844 | 0.49 | BMI | 0.028 | 0.0181 |  | |  |  |  |
| rs13135416 | 4 | 18346297 | T | C | 0.3032 | 0.0177 | 0.0031 | 1.56E-08 | 0.000508 | 0.002407 | 0.83 | BMI | 0.0145 | 0.0148 |  | |  |  |  |
| rs34811474 | 4 | 25408838 | A | G | 0.2229 | -0.0278 | 0.0034 | 6.48E-16 | 0.002815 | 0.002605 | 0.28 | BMI | -0.0086 | 0.0191 |  | |  |  |  |
| rs2242189 | 4 | 38691024 | T | C | 0.6328 | 0.017 | 0.0026 | 5.55E-11 | -0.00617 | 0.002282 | 0.0069 | BMI | -0.0088 | 0.0145 |  | |  |  |  |
| rs6857629 | 4 | 39684977 | G | A | 0.755 | -0.0039 | 0.0033 | 0.2321 | 0.015386 | 0.00256 | 1.9E-09 | PC | 0.0344 | 0.0155 |  | |  |  |  |
| rs2237025 | 4 | 55541879 | T | C | 0.4302 | 0.0141 | 0.0025 | 3.1E-08 | 0.000936 | 0.002233 | 0.68 | BMI | -0.0525 | 0.0138 |  | |  |  |  |
| rs13107325 | 4 | 103188709 | T | C | 0.0852 | 0.0495 | 0.0047 | 3.22E-26 | 0.001276 | 0.004199 | 0.76 | BMI | 0.0093 | 0.026 |  | |  |  |  |
| rs2613726 | 4 | 112597321 | A | G | 0.3698 | -0.0162 | 0.0026 | 4.34E-10 | -0.00116 | 0.002319 | 0.62 | BMI | 0.0211 | 0.0143 |  | |  |  |  |
| rs2391518 | 4 | 130817060 | T | C | 0.315 | 0.0159 | 0.0025 | 1.74E-10 | -0.00436 | 0.002374 | 0.067 | BMI | 0.0049 | 0.0146 |  | |  |  |  |
| rs1296328 | 4 | 137083193 | A | C | 0.4434 | 0.02 | 0.0025 | 2.07E-15 | -0.00233 | 0.002226 | 0.29 | BMI | -0.0283 | 0.014 |  | |  |  |  |
| rs6537285 | 4 | 145310147 | A | G | 0.2801 | -0.0178 | 0.0028 | 1.24E-10 | -0.00423 | 0.002444 | 0.083 | BMI | 0.0245 | 0.0151 |  | |  |  |  |
| rs6827083 | 4 | 153075491 | A | G | 0.5791 | -0.0152 | 0.0024 | 1.34E-10 | -0.00733 | 0.002223 | 0.00098 | BMI | 0.0244 | 0.014 |  | |  |  |  |
| rs2068625 | 4 | 159856739 | T | C | 0.302 | 0.0038 | 0.0027 | 0.1606 | -0.01584 | 0.002398 | 4E-11 | PC | 0.0111 | 0.015 |  | |  |  |  |
| rs2220599 | 5 | 7378854 | C | G | 0.633 | 0.0018 | 0.0026 | 0.4959 | -0.01605 | 0.00229 | 2.4E-12 | PC | -0.0095 | 0.014 |  | |  |  |  |
| rs1503526 | 5 | 63020706 | T | C | 0.5296 | -0.014 | 0.0024 | 5.74E-09 | -0.00171 | 0.002202 | 0.44 | BMI | 0.0187 | 0.0136 |  | |  |  |  |
| rs253414 | 5 | 74956517 | T | C | 0.6567 | 0.0259 | 0.0025 | 1.1E-24 | -0.00586 | 0.002342 | 0.012 | BMI | 0.0075 | 0.0142 |  | |  |  |  |
| rs4532349 | 5 | 77473165 | A | G | 0.7831 | 0.0162 | 0.0028 | 6.64E-09 | 0.007505 | 0.002585 | 0.0037 | BMI | -0.0169 | 0.0166 |  | |  |  |  |
| rs11749912 | 5 | 88065628 | A | G | 0.423 | 0.0109 | 0.0029 | 0.000169 | 0.01392 | 0.002237 | 4.9E-10 | PC | 0.0102 | 0.0137 |  | |  |  |  |
| rs1469249 | 5 | 113837198 | G | A | 0.789 | -0.0083 | 0.0031 | 0.007374 | 0.015558 | 0.002711 | 9.5E-09 | PC | 0.0166 | 0.0165 |  | |  |  |  |
| rs162894 | 5 | 131611872 | T | G | 0.332 | 0.0046 | 0.0026 | 0.08134 | 0.013385 | 0.00234 | 1.1E-08 | PC | -0.0174 | 0.0142 |  | |  |  |  |
| rs56158102 | 5 | 144027803 | C | T | 0.344 | -0.0034 | 0.0026 | 0.2019 | 0.01321 | 0.002385 | 3.1E-08 | PC | 0.003 | 0.0144 |  | |  |  |  |
| rs7715256 | 5 | 153537893 | T | G | 0.5631 | -0.0163 | 0.0024 | 4.52E-12 | 0.003583 | 0.002223 | 0.11 | BMI | -0.0086 | 0.0139 |  | |  |  |  |
| rs7730898 | 5 | 170459675 | A | G | 0.7314 | 0.0169 | 0.0026 | 9.94E-11 | 0.00212 | 0.002474 | 0.39 | BMI | 0.0007 | 0.0153 |  | |  |  |  |
| rs6556301 | 5 | 176527577 | T | G | 0.3704 | -0.0143 | 0.0025 | 1.57E-08 | -0.00067 | 0.002293 | 0.77 | BMI | -0.003 | 0.0143 |  | |  |  |  |
| rs2228213 | 6 | 12124855 | A | G | 0.3366 | -0.0151 | 0.0025 | 6.77E-10 | -0.00253 | 0.00231 | 0.27 | BMI | -0.011 | 0.0142 |  | |  |  |  |
| rs9477970 | 6 | 19082663 | T | A | 0.808 | -0.0042 | 0.0032 | 0.188 | -0.01604 | 0.0028 | 1E-08 | PC | -0.0323 | 0.0175 |  | |  |  |  |
| rs9366639 | 6 | 26167613 | C | G | 0.804 | 0.0194 | 0.0033 | 3.82E-09 | -0.00842 | 0.002869 | 0.0033 | BMI | 0.0348 | 0.0177 |  | |  |  |  |
| rs2269553 | 6 | 28876509 | A | G | 0.2114 | -0.019 | 0.0028 | 2.23E-11 | 0.006481 | 0.002683 | 0.016 | BMI | 0.0063 | 0.016 |  | |  |  |  |
| rs9277992 | 6 | 33312455 | A | G | 0.1883 | 0.0275 | 0.0037 | 8.69E-14 | 0.004172 | 0.002827 | 0.14 | BMI | -0.0114 | 0.0175 |  | |  |  |  |
| rs2744974 | 6 | 34579431 | T | C | 0.3217 | 0.0229 | 0.0025 | 8.48E-20 | 0.0051 | 0.002347 | 0.03 | BMI | 0.0045 | 0.0142 |  | |  |  |  |
| rs34045288 | 6 | 40369081 | T | C | 0.3304 | 0.0249 | 0.0031 | 3.95E-16 | -0.00177 | 0.00233 | 0.45 | BMI | 0.0276 | 0.0145 |  | |  |  |  |
| rs2206277 | 6 | 50798526 | T | C | 0.1508 | 0.0454 | 0.0031 | 5.35E-50 | 0.001994 | 0.002878 | 0.49 | BMI | -0.0041 | 0.0172 |  | |  |  |  |
| rs1327259 | 6 | 51177811 | A | G | 0.6096 | 0.0159 | 0.0026 | 5.96E-10 | -0.00074 | 0.002268 | 0.74 | BMI | 0.0147 | 0.0139 |  | |  |  |  |
| rs7773045 | 6 | 81443423 | T | G | 0.9215 | 0.0264 | 0.0044 | 1.6E-09 | -0.0056 | 0.004008 | 0.16 | BMI | -0.0025 | 0.0273 |  | |  |  |  |
| rs12663742 | 6 | 97792735 | A | G | 0.3719 | -0.0181 | 0.0026 | 1.69E-12 | 0.002798 | 0.00228 | 0.22 | BMI | -0.0057 | 0.0139 |  | |  |  |  |
| rs9372625 | 6 | 98344031 | G | A | 0.619 | 0.0194 | 0.0026 | 5.05E-14 | -0.01839 | 0.002272 | 5.7E-16 | PC | 0.0327 | 0.014 |  | |  |  |  |
| rs768023 | 6 | 108876002 | A | G | 0.6018 | 0.0133 | 0.0024 | 3.05E-08 | 0.001009 | 0.002277 | 0.66 | BMI | 0.0275 | 0.0139 |  | |  |  |  |
| rs1159974 | 6 | 126090277 | T | C | 0.4922 | -0.0139 | 0.0025 | 2.03E-08 | -0.0033 | 0.002203 | 0.13 | BMI | -0.0178 | 0.0135 |  | |  |  |  |
| rs6569648 | 6 | 130349119 | T | C | 0.7649 | -0.0152 | 0.0028 | 3.66E-08 | -0.00628 | 0.00257 | 0.014 | BMI | 0.0247 | 0.0164 |  | |  |  |  |
| rs6935828 | 6 | 140811367 | C | T | 0.446 | 0.0009 | 0.0025 | 0.7269 | -0.01212 | 0.002213 | 4.3E-08 | PC | 0.0045 | 0.0137 |  | |  |  |  |
| rs13191362 | 6 | 163033350 | A | G | 0.8527 | 0.0265 | 0.0036 | 2.34E-13 | -0.00533 | 0.003332 | 0.11 | BMI | 0.0227 | 0.0214 |  | |  |  |  |
| rs9364687 | 6 | 163817911 | T | G | 0.3979 | -0.014 | 0.0024 | 3.97E-09 | -0.00879 | 0.002236 | 0.000084 | BMI | -0.0057 | 0.0137 |  | |  |  |  |
| rs9638713 | 7 | 14645949 | A | G | 0.0267 | 0.045 | 0.0075 | 2.32E-09 | -0.00409 | 0.007038 | 0.56 | BMI | -0.0011 | 0.0434 |  | |  |  |  |
| rs215635 | 7 | 32370220 | T | C | 0.6215 | -0.0183 | 0.0026 | 1.16E-12 | 0.003151 | 0.002285 | 0.17 | BMI | 0.0123 | 0.0139 |  | |  |  |  |
| rs77095142 | 7 | 39096572 | A | C | 0.0314 | 0.0459 | 0.0082 | 2.37E-08 | 0.015732 | 0.006135 | 0.01 | BMI | 0.0692 | 0.0435 |  | |  |  |  |
| rs10269783 | 7 | 49616203 | A | G | 0.4227 | 0.0172 | 0.0024 | 7.59E-13 | 0.003668 | 0.002261 | 0.1 | BMI | 0.0013 | 0.0138 |  | |  |  |  |
| rs55772938 | 7 | 70025113 | A | G | 0.705 | -0.0084 | 0.0032 | 0.007678 | -0.01512 | 0.002417 | 3.9E-10 | PC | 0.0009 | 0.0148 |  | |  |  |  |
| rs2245368 | 7 | 76608143 | T | C | 0.8104 | -0.0328 | 0.0033 | 4.39E-23 | 0.00137 | 0.002942 | 0.64 | BMI | -0.0103 | 0.0176 |  | |  |  |  |
| rs1701829 | 7 | 113033426 | A | G | 0.3356 | 0.0214 | 0.0026 | 6.76E-16 | -0.00208 | 0.002334 | 0.37 | BMI | 0.0001 | 0.0145 |  | |  |  |  |
| rs12706626 | 7 | 124531370 | G | A | 0.617 | -0.0042 | 0.003 | 0.1566 | -0.01287 | 0.002266 | 1.4E-08 | PC | -0.0294 | 0.0141 |  | |  |  |  |
| rs3944151 | 7 | 126970005 | A | G | 0.275 | 0.0015 | 0.0028 | 0.6056 | 0.013673 | 0.002469 | 3.1E-08 | PC | 0.02 | 0.0153 |  | |  |  |  |
| rs2345941 | 7 | 133337635 | A | G | 0.55 | -0.0021 | 0.0025 | 0.4073 | 0.014647 | 0.002217 | 4E-11 | PC | -0.0126 | 0.0136 |  | |  |  |  |
| rs13228237 | 7 | 138780222 | C | G | 0.2131 | -0.0164 | 0.003 | 4.59E-08 | -0.00339 | 0.002669 | 0.2 | BMI | -0.0175 | 0.0166 |  | |  |  |  |
| rs4841504 | 8 | 11024663 | A | C | 0.5023 | -0.0193 | 0.0024 | 4.24E-16 | -0.00106 | 0.002209 | 0.63 | BMI | -0.0038 | 0.0151 |  | |  |  |  |
| rs4123853 | 8 | 14091025 | T | C | 0.3732 | 0.0166 | 0.0026 | 1.1E-10 | 0.002292 | 0.002271 | 0.31 | BMI | 0.0049 | 0.0139 |  | |  |  |  |
| rs354510 | 8 | 15534749 | T | C | 0.8657 | 0.0236 | 0.0042 | 1.98E-08 | -0.00536 | 0.003291 | 0.1 | BMI | -0.0244 | 0.0198 |  | |  |  |  |
| rs7827210 | 8 | 30871656 | A | G | 0.3969 | 0.0179 | 0.0026 | 2.34E-12 | -0.00744 | 0.002259 | 0.00098 | BMI | 0.0219 | 0.0142 |  | |  |  |  |
| rs7012923 | 8 | 33630937 | A | C | 0.4412 | 0.0163 | 0.0025 | 4.92E-11 | 0.0045 | 0.00223 | 0.044 | BMI | -0.0132 | 0.014 |  | |  |  |  |
| rs35957544 | 8 | 73440371 | T | G | 0.5597 | -0.018 | 0.0029 | 4.14E-10 | -0.00492 | 0.002232 | 0.028 | BMI | 0.0178 | 0.0138 |  | |  |  |  |
| rs2588785 | 8 | 76636191 | A | G | 0.2841 | -0.0211 | 0.0026 | 1.74E-15 | 0.001875 | 0.00248 | 0.45 | BMI | -0.0174 | 0.0151 |  | |  |  |  |
| rs957447 | 8 | 95541223 | A | C | 0.2385 | -0.0225 | 0.003 | 8.56E-14 | -0.00112 | 0.002594 | 0.67 | BMI | -0.0163 | 0.0153 |  | |  |  |  |
| rs2960151 | 8 | 116718620 | A | C | 0.7641 | 0.0225 | 0.0034 | 3.22E-11 | -0.0007 | 0.002574 | 0.79 | BMI | 0.0294 | 0.0161 |  | |  |  |  |
| rs72673947 | 8 | 118884379 | A | G | 0.8896 | -0.0278 | 0.0046 | 1.76E-09 | -0.00018 | 0.003614 | 0.96 | BMI | -0.0091 | 0.021 |  | |  |  |  |
| rs7826388 | 8 | 128109776 | T | C | 0.0365 | -0.0374 | 0.0066 | 1.4E-08 | -0.00129 | 0.006125 | 0.83 | BMI | -0.0276 | 0.0347 |  | |  |  |  |
| rs13262595 | 8 | 143316970 | A | G | 0.44 | -0.004 | 0.0025 | 0.1145 | -0.01573 | 0.002215 | 1.2E-12 | PC | 0.0043 | 0.0138 |  | |  |  |  |
| rs10738451 | 9 | 16713666 | T | C | 0.7153 | -0.0163 | 0.0027 | 2.22E-09 | -0.00125 | 0.002457 | 0.61 | BMI | -0.021 | 0.0148 |  | |  |  |  |
| rs4977839 | 9 | 23355310 | G | A | 0.584 | -0.0081 | 0.0029 | 0.005508 | -0.01992 | 0.002233 | 4.7E-19 | PC | -0.0019 | 0.0139 |  | |  |  |  |
| rs10968114 | 9 | 27800007 | A | C | 0.534 | 0.0153 | 0.0025 | 8.7E-10 | 0.001081 | 0.002214 | 0.63 | BMI | 0.0168 | 0.0137 |  | |  |  |  |
| rs1412235 | 9 | 28410996 | C | G | 0.3137 | 0.0234 | 0.0025 | 9.85E-21 | 0.004698 | 0.00235 | 0.046 | BMI | 0.0243 | 0.0148 |  | |  |  |  |
| rs10746862 | 9 | 73780912 | T | G | 0.5804 | -0.0155 | 0.0025 | 8.63E-10 | 0.003109 | 0.002229 | 0.16 | BMI | -0.0043 | 0.0137 |  | |  |  |  |
| rs1187351 | 9 | 87295077 | A | C | 0.3985 | -0.0139 | 0.0025 | 4.51E-08 | -0.0031 | 0.002248 | 0.17 | BMI | -0.0002 | 0.0138 |  | |  |  |  |
| rs6559921 | 9 | 88872039 | A | T | 0.7711 | 0.0181 | 0.003 | 1.19E-09 | -0.00433 | 0.002654 | 0.1 | BMI | 0.0055 | 0.0165 |  | |  |  |  |
| rs113851275 | 9 | 98297220 | G | A | 0.892 | -0.0085 | 0.0046 | 0.06279 | -0.02094 | 0.003537 | 3.2E-09 | PC | 0.0008 | 0.0217 |  | |  |  |  |
| rs7020477 | 9 | 116827760 | A | G | 0.735 | -0.001 | 0.0033 | 0.7631 | 0.014234 | 0.002498 | 1.2E-08 | PC | -0.0035 | 0.0165 |  | |  |  |  |
| rs10760279 | 9 | 126105291 | T | G | 0.3832 | 0.0154 | 0.0027 | 7.49E-09 | -0.00463 | 0.002311 | 0.045 | BMI | 0.0174 | 0.0139 |  | |  |  |  |
| rs73578186 | 9 | 126334485 | C | T | 0.675 | 0.0024 | 0.0031 | 0.4412 | 0.01499 | 0.002365 | 2.3E-10 | PC | -0.0224 | 0.0144 |  | |  |  |  |
| rs11251291 | 10 | 2561766 | A | C | 0.4671 | -0.0141 | 0.0025 | 1.65E-08 | 0.001359 | 0.002209 | 0.54 | BMI | -0.0097 | 0.0137 |  | |  |  |  |
| rs12251016 | 10 | 21821918 | A | T | 0.6575 | -0.0194 | 0.003 | 1.43E-10 | -0.01155 | 0.00232 | 6.3E-07 | BMI | -0.0285 | 0.0146 |  | |  |  |  |
| rs3758438 | 10 | 27400661 | T | C | 0.0782 | 0.0274 | 0.0047 | 7.27E-09 | 0.006032 | 0.004086 | 0.14 | BMI | -0.0226 | 0.0243 |  | |  |  |  |
| rs12761779 | 10 | 63782043 | C | G | 0.6501 | 0.0173 | 0.0031 | 1.48E-08 | -0.0015 | 0.00234 | 0.52 | BMI | 0.0245 | 0.0145 |  | |  |  |  |
| rs7072873 | 10 | 77210191 | T | C | 0.5346 | -0.0147 | 0.0025 | 5.45E-09 | 0.000447 | 0.002218 | 0.84 | BMI | -0.0134 | 0.0136 |  | |  |  |  |
| rs10788494 | 10 | 88110925 | C | G | 0.4834 | 0.0155 | 0.0025 | 5.39E-10 | -7.7E-05 | 0.002209 | 0.97 | BMI | -0.0009 | 0.0137 |  | |  |  |  |
| rs10883026 | 10 | 99793865 | T | C | 0.5201 | -0.0189 | 0.0029 | 6.23E-11 | 0.001363 | 0.002217 | 0.54 | BMI | -0.0112 | 0.0138 |  | |  |  |  |
| rs17094222 | 10 | 102395440 | T | C | 0.7914 | -0.0255 | 0.0029 | 1.14E-18 | 0.000145 | 0.002697 | 0.96 | BMI | -0.0122 | 0.0165 |  | |  |  |  |
| rs11191356 | 10 | 104393489 | A | G | 0.8942 | -0.026 | 0.0041 | 2.48E-10 | 0.003631 | 0.004089 | 0.37 | BMI | -0.033 | 0.0224 |  | |  |  |  |
| rs79780963 | 10 | 104952499 | T | C | 0.0808 | 0.0371 | 0.0052 | 1.55E-12 | 0.004472 | 0.00414 | 0.28 | BMI | 0.0664 | 0.0231 |  | |  |  |  |
| rs7903146 | 10 | 114758349 | T | C | 0.2775 | -0.0231 | 0.0026 | 3.57E-19 | -0.00048 | 0.002421 | 0.84 | BMI | -0.0079 | 0.0151 |  | |  |  |  |
| rs845084 | 10 | 125220036 | A | G | 0.2687 | 0.0157 | 0.0029 | 3.85E-08 | 0.00092 | 0.002527 | 0.72 | BMI | -0.0085 | 0.0151 |  | |  |  |  |
| rs7113874 | 11 | 8688016 | T | C | 0.3274 | -0.0144 | 0.0024 | 3.3E-09 | -0.00042 | 0.002322 | 0.86 | BMI | 0 | 0.0141 |  | |  |  |  |
| rs900144 | 11 | 13294268 | T | C | 0.5839 | 0.0154 | 0.0025 | 5.54E-10 | 0.005599 | 0.002222 | 0.012 | BMI | -0.0167 | 0.0136 |  | |  |  |  |
| rs6265 | 11 | 27679916 | T | C | 0.1832 | -0.0478 | 0.003 | 6.25E-58 | -0.01143 | 0.002809 | 0.000047 | BMI | -0.0172 | 0.0167 |  | |  |  |  |
| rs1765139 | 11 | 30353725 | T | C | 0.7669 | 0.0221 | 0.003 | 1.8E-13 | 0.003647 | 0.00262 | 0.16 | BMI | 0.001 | 0.0167 |  | |  |  |  |
| rs2862996 | 11 | 43653833 | T | G | 0.7043 | -0.0199 | 0.0025 | 2.49E-15 | 0.000141 | 0.00238 | 0.95 | BMI | 0.0005 | 0.0147 |  | |  |  |  |
| rs7124681 | 11 | 47529947 | A | C | 0.4187 | 0.0275 | 0.0024 | 5.1E-31 | 0.008866 | 0.002235 | 0.000072 | BMI | 0.0127 | 0.0137 |  | |  |  |  |
| rs7102454 | 11 | 65594820 | T | C | 0.6417 | -0.0177 | 0.0026 | 1.29E-11 | -0.00186 | 0.002289 | 0.42 | BMI | -0.0238 | 0.0142 |  | |  |  |  |
| rs11228371 | 11 | 68594174 | A | C | 0.4066 | 0.0165 | 0.0029 | 1.31E-08 | 0.001656 | 0.00227 | 0.47 | BMI | -0.0189 | 0.0139 |  | |  |  |  |
| rs349071 | 11 | 84776849 | A | G | 0.4989 | -0.0177 | 0.0029 | 7.53E-10 | -0.00167 | 0.002205 | 0.45 | BMI | 0.0036 | 0.0135 |  | |  |  |  |
| rs647248 | 11 | 89917699 | A | G | 0.5444 | 0.0161 | 0.0025 | 2.59E-10 | 0.001306 | 0.002234 | 0.56 | BMI | -0.0234 | 0.0135 |  | |  |  |  |
| rs2734849 | 11 | 113270160 | A | G | 0.491 | 0.0113 | 0.0025 | 5.34E-06 | -0.01351 | 0.0022 | 8.2E-10 | PC | 0.0039 | 0.0137 |  | |  |  |  |
| rs12286929 | 11 | 115022404 | A | G | 0.5071 | -0.0178 | 0.0023 | 2.34E-14 | 0.002107 | 0.002206 | 0.34 | BMI | 0.0001 | 0.0136 |  | |  |  |  |
| rs7933085 | 11 | 130796248 | A | G | 0.4904 | -0.0174 | 0.0029 | 1.62E-09 | 0.001219 | 0.002213 | 0.58 | BMI | -0.0116 | 0.0137 |  | |  |  |  |
| rs1448355 | 11 | 131286685 | C | T | 0.381 | 0.0008 | 0.0026 | 0.7523 | -0.01461 | 0.002274 | 1.3E-10 | PC | -0.0081 | 0.014 |  | |  |  |  |
| rs11611246 | 12 | 939480 | T | G | 0.1962 | 0.0246 | 0.003 | 7.99E-17 | 0.002373 | 0.002689 | 0.38 | BMI | 0.0099 | 0.0167 |  | |  |  |  |
| rs76895963 | 12 | 4384844 | T | G | 0.9798 | -0.0768 | 0.0113 | 1.07E-11 | -0.01795 | 0.008522 | 0.035 | BMI | -0.2321 | 0.0563 |  | |  |  |  |
| rs10772983 | 12 | 17141582 | T | C | 0.544 | -0.014 | 0.0023 | 2.04E-09 | 0.002337 | 0.002204 | 0.29 | BMI | 0.0016 | 0.0135 |  | |  |  |  |
| rs10842240 | 12 | 24060075 | C | G | 0.1253 | 0.0258 | 0.0037 | 4.56E-12 | 0.002779 | 0.003455 | 0.42 | BMI | 0.0081 | 0.0196 |  | |  |  |  |
| rs35933007 | 12 | 38929341 | G | A | 0.772 | 0.0088 | 0.0035 | 0.01172 | -0.01519 | 0.002644 | 9.2E-09 | PC | -0.0048 | 0.0188 |  | |  |  |  |
| rs11181001 | 12 | 41948196 | A | G | 0.4822 | 0.0144 | 0.0024 | 1.25E-09 | 0.002444 | 0.002227 | 0.27 | BMI | 0.0167 | 0.0137 |  | |  |  |  |
| rs7132908 | 12 | 50263148 | A | G | 0.3923 | 0.0346 | 0.0027 | 1.35E-38 | -0.00089 | 0.002264 | 0.69 | BMI | -0.001 | 0.0139 |  | |  |  |  |
| rs705704 | 12 | 56435412 | A | G | 0.3222 | -0.0163 | 0.0026 | 1.52E-10 | 0.007229 | 0.002317 | 0.0018 | BMI | 0 | 0.0146 |  | |  |  |  |
| rs1689437 | 12 | 89758046 | A | G | 0.9475 | -0.0297 | 0.0049 | 2.04E-09 | -0.00582 | 0.004577 | 0.2 | BMI | 0.034 | 0.0284 |  | |  |  |  |
| rs7488867 | 12 | 103699685 | T | C | 0.2574 | -0.022 | 0.0029 | 5.69E-14 | 0.001692 | 0.002532 | 0.5 | BMI | -0.0039 | 0.0151 |  | |  |  |  |
| rs10850184 | 12 | 114064977 | T | G | 0.3214 | 0.0167 | 0.0027 | 3.53E-10 | 0.004793 | 0.002364 | 0.043 | BMI | 0.0182 | 0.0144 |  | |  |  |  |
| rs206965 | 12 | 120856332 | T | C | 0.207 | 0.0043 | 0.0031 | 0.1572 | 0.015601 | 0.002715 | 9.1E-09 | PC | -0.0141 | 0.017 |  | |  |  |  |
| rs10902480 | 12 | 132414769 | T | G | 0.8236 | 0.022 | 0.0038 | 5.6E-09 | 0.001998 | 0.002894 | 0.49 | BMI | -0.0018 | 0.0182 |  | |  |  |  |
| rs2012835 | 13 | 27987643 | T | G | 0.2675 | -0.0205 | 0.0033 | 3.06E-10 | -0.00546 | 0.002469 | 0.027 | BMI | 0.0124 | 0.0159 |  | |  |  |  |
| rs2504236 | 13 | 28613298 | T | C | 0.586 | -0.0151 | 0.0025 | 1.94E-09 | -0.00442 | 0.002242 | 0.049 | BMI | 0.0065 | 0.0135 |  | |  |  |  |
| rs9595893 | 13 | 33175793 | T | G | 0.3595 | -0.0199 | 0.0026 | 1.24E-14 | 0.000537 | 0.002282 | 0.81 | BMI | -0.0019 | 0.014 |  | |  |  |  |
| rs9603697 | 13 | 40783323 | T | C | 0.3168 | 0.0197 | 0.0027 | 1.34E-13 | -0.00091 | 0.002347 | 0.7 | BMI | -0.0024 | 0.0145 |  | |  |  |  |
| rs12429545 | 13 | 54102206 | A | G | 0.1206 | 0.0336 | 0.0035 | 2.03E-21 | -0.00512 | 0.003305 | 0.12 | BMI | -0.0092 | 0.0199 |  | |  |  |  |
| rs12874776 | 13 | 57794345 | C | A | 0.893 | -0.0008 | 0.0037 | 0.8316 | -0.02335 | 0.003592 | 8E-11 | PC | -0.0083 | 0.0225 |  | |  |  |  |
| rs7338127 | 13 | 59260511 | A | T | 0.3304 | 0.0197 | 0.0027 | 1.39E-13 | 0.000741 | 0.002355 | 0.75 | BMI | -0.0052 | 0.0144 |  | |  |  |  |
| rs1441264 | 13 | 79580919 | A | G | 0.5821 | 0.0181 | 0.0024 | 1.35E-13 | 0.00228 | 0.002288 | 0.32 | BMI | 0.0171 | 0.0141 |  | |  |  |  |
| rs912690 | 13 | 97020705 | C | G | 0.5566 | -0.0173 | 0.0025 | 6.92E-12 | -0.00257 | 0.002229 | 0.25 | BMI | -0.0137 | 0.0136 |  | |  |  |  |
| rs9168 | 13 | 99101583 | A | C | 0.2727 | -0.015 | 0.0027 | 4.62E-08 | 0.002608 | 0.002417 | 0.28 | BMI | 0.0397 | 0.0151 |  | |  |  |  |
| rs9522279 | 13 | 112221296 | T | C | 0.4155 | 0.0224 | 0.0029 | 1.65E-14 | -0.00713 | 0.00223 | 0.0014 | BMI | 0.0194 | 0.0138 |  | |  |  |  |
| rs10132280 | 14 | 25928179 | A | C | 0.3128 | -0.0299 | 0.0026 | 1.38E-31 | -0.00018 | 0.002413 | 0.94 | BMI | 0.0121 | 0.0149 |  | |  |  |  |
| rs1811859 | 14 | 29677727 | T | C | 0.2168 | -0.0204 | 0.0035 | 6.48E-09 | -0.00085 | 0.002725 | 0.76 | BMI | -0.0019 | 0.0168 |  | |  |  |  |
| rs1955540 | 14 | 40801751 | T | C | 0.1942 | -0.02 | 0.0032 | 2.89E-10 | -0.00033 | 0.002796 | 0.91 | BMI | -0.0075 | 0.0173 |  | |  |  |  |
| rs198262 | 14 | 57283283 | C | T | 0.044 | -0.0065 | 0.0063 | 0.3 | 0.030005 | 0.00546 | 3.9E-08 | PC | 0.0046 | 0.0352 |  | |  |  |  |
| rs217672 | 14 | 62361021 | A | C | 0.7243 | -0.0201 | 0.0032 | 4.4E-10 | -0.00113 | 0.002478 | 0.65 | BMI | 0.0121 | 0.0154 |  | |  |  |  |
| rs3902951 | 14 | 69789755 | T | G | 0.7709 | -0.0151 | 0.0028 | 4.4E-08 | 0.004805 | 0.002613 | 0.066 | BMI | 0.0031 | 0.016 |  | |  |  |  |
| rs17109256 | 14 | 79939993 | A | G | 0.2374 | 0.0308 | 0.0028 | 2.81E-27 | -0.00742 | 0.002682 | 0.0056 | BMI | -0.0052 | 0.0173 |  | |  |  |  |
| rs34918753 | 14 | 88312341 | A | G | 0.2337 | 0.0204 | 0.0034 | 1.77E-09 | 0.002577 | 0.002567 | 0.32 | BMI | -0.0044 | 0.0165 |  | |  |  |  |
| rs11160119 | 14 | 93932258 | A | G | 0.4105 | -0.0222 | 0.0029 | 2.85E-14 | 0.001385 | 0.002238 | 0.54 | BMI | -0.0169 | 0.0139 |  | |  |  |  |
| rs7161194 | 14 | 101529005 | A | G | 0.3445 | 0.0215 | 0.0027 | 3.32E-15 | 0.000222 | 0.002436 | 0.93 | BMI | 0.0211 | 0.0151 |  | |  |  |  |
| rs7143963 | 14 | 103304425 | T | C | 0.1647 | 0.0211 | 0.003 | 3.35E-12 | 0.001005 | 0.00293 | 0.73 | BMI | -0.0076 | 0.0172 |  | |  |  |  |
| rs11636536 | 15 | 36391772 | T | C | 0.4972 | 0.0148 | 0.0025 | 2.8E-09 | -0.00091 | 0.002204 | 0.68 | BMI | 0.0144 | 0.0136 |  | |  |  |  |
| rs166835 | 15 | 47716037 | C | T | 0.443 | 0.0027 | 0.0025 | 0.2768 | 0.013136 | 0.002222 | 3.4E-09 | PC | -0.0125 | 0.0137 |  | |  |  |  |
| rs4776970 | 15 | 68080886 | A | T | 0.6427 | 0.0279 | 0.0024 | 1.52E-30 | -0.00462 | 0.002298 | 0.044 | BMI | 0.0261 | 0.0141 |  | |  |  |  |
| rs58429335 | 15 | 70199927 | T | C | 0.9286 | -0.0306 | 0.0056 | 4.85E-08 | -0.00048 | 0.00436 | 0.91 | BMI | 0.0147 | 0.0252 |  | |  |  |  |
| rs4777541 | 15 | 73082240 | T | C | 0.7844 | 0.021 | 0.003 | 1.15E-12 | -0.0017 | 0.002605 | 0.51 | BMI | 0.0152 | 0.0166 |  | |  |  |  |
| rs8041222 | 15 | 79465549 | A | G | 0.41 | -0.0194 | 0.0029 | 3.28E-11 | 0.001104 | 0.002234 | 0.62 | BMI | -0.0402 | 0.0138 |  | |  |  |  |
| rs4702 | 15 | 91426560 | G | A | 0.444 | 0.0107 | 0.0024 | 9.88E-06 | 0.012178 | 0.002215 | 3.8E-08 | PC | 0.0218 | 0.0137 |  | |  |  |  |
| rs12448257 | 16 | 3599655 | A | G | 0.2191 | 0.0186 | 0.0029 | 1.17E-10 | 0.005134 | 0.002693 | 0.057 | BMI | 0.0109 | 0.017 |  | |  |  |  |
| rs879620 | 16 | 4015729 | T | C | 0.5967 | 0.0209 | 0.0026 | 3.11E-16 | -0.00311 | 0.00227 | 0.17 | BMI | 0.0105 | 0.0139 |  | |  |  |  |
| rs6498759 | 16 | 18020295 | T | C | 0.31 | 0.0003 | 0.0027 | 0.9238 | 0.013265 | 0.002386 | 2.7E-08 | PC | 0.001 | 0.0144 |  | |  |  |  |
| rs9926784 | 16 | 19941968 | T | C | 0.8007 | 0.0224 | 0.003 | 5.74E-14 | -0.00022 | 0.002848 | 0.94 | BMI | -0.008 | 0.0172 |  | |  |  |  |
| rs3888190 | 16 | 28889486 | A | C | 0.3801 | 0.0315 | 0.0024 | 6.96E-40 | -0.00535 | 0.002245 | 0.017 | BMI | 0.0088 | 0.0142 |  | |  |  |  |
| rs57149692 | 16 | 30142021 | C | G | 0.3992 | -0.0267 | 0.0029 | 1.11E-19 | 0.003486 | 0.002256 | 0.12 | BMI | -0.0076 | 0.014 |  | |  |  |  |
| rs1558902 | 16 | 53803574 | A | T | 0.4152 | 0.0815 | 0.0024 | 1.2E-257 | -0.00448 | 0.002244 | 0.046 | BMI | 0 | 0.0138 |  | |  |  |  |
| rs3730399 | 16 | 67229019 | A | G | 0.935 | -0.0127 | 0.0056 | 0.02477 | 0.024651 | 0.004461 | 3.3E-08 | PC | -0.0031 | 0.028 |  | |  |  |  |
| rs889398 | 16 | 69556715 | T | C | 0.4179 | -0.0209 | 0.0024 | 1.54E-18 | -0.00155 | 0.002244 | 0.49 | BMI | -0.0136 | 0.0138 |  | |  |  |  |
| rs75950518 | 16 | 70378917 | T | C | 0.1154 | -0.0266 | 0.0045 | 3.51E-09 | -0.00926 | 0.003439 | 0.0071 | BMI | 0.0115 | 0.023 |  | |  |  |  |
| rs7206608 | 16 | 82872628 | C | G | 0.6743 | -0.015 | 0.0027 | 1.85E-08 | -0.00266 | 0.002356 | 0.26 | BMI | -0.0161 | 0.015 |  | |  |  |  |
| rs3923783 | 17 | 1843189 | A | C | 0.178 | -0.0226 | 0.0033 | 7.47E-12 | 0.002835 | 0.002832 | 0.32 | BMI | -0.0288 | 0.0181 |  | |  |  |  |
| rs12450045 | 17 | 4742112 | A | G | 0.4576 | 0.0143 | 0.0025 | 1.68E-08 | -0.00012 | 0.002219 | 0.96 | BMI | 0.0032 | 0.0142 |  | |  |  |  |
| rs7209653 | 17 | 19882084 | T | C | 0.704 | 0.004 | 0.0027 | 0.1428 | 0.015638 | 0.002413 | 9.1E-11 | PC | -0.0096 | 0.0151 |  | |  |  |  |
| rs4986044 | 17 | 21261560 | T | C | 0.454 | -0.0181 | 0.0024 | 1.45E-14 | -0.00245 | 0.002215 | 0.27 | BMI | -0.0054 | 0.0137 |  | |  |  |  |
| rs8065172 | 17 | 31456969 | A | G | 0.2384 | -0.0167 | 0.0029 | 1.23E-08 | -0.00278 | 0.002601 | 0.29 | BMI | 0.0236 | 0.0156 |  | |  |  |  |
| rs12603813 | 17 | 43196584 | T | C | 0.746 | 0.0017 | 0.0027 | 0.5388 | 0.014406 | 0.002534 | 1.3E-08 | PC | -0.0236 | 0.0158 |  | |  |  |  |
| rs208015 | 17 | 46252346 | T | C | 0.0703 | 0.0365 | 0.0049 | 8.72E-14 | -0.00691 | 0.004387 | 0.12 | BMI | 0.003 | 0.0242 |  | |  |  |  |
| rs78369934 | 17 | 61739101 | T | C | 0.9478 | 0.0369 | 0.0065 | 1.47E-08 | -0.01832 | 0.004887 | 0.00018 | BMI | 0.0197 | 0.0321 |  | |  |  |  |
| rs66643547 | 17 | 79375315 | C | T | 0.647 | 0.0045 | 0.003 | 0.1418 | -0.01539 | 0.002318 | 3.2E-11 | PC | 0.0097 | 0.0147 |  | |  |  |  |
| rs7226371 | 18 | 1850771 | A | G | 0.8436 | -0.0186 | 0.0032 | 7.73E-09 | 0.005368 | 0.003085 | 0.082 | BMI | 0.0037 | 0.0188 |  | |  |  |  |
| rs891387 | 18 | 21103909 | T | C | 0.4956 | 0.0227 | 0.0025 | 6.81E-20 | -0.00749 | 0.002203 | 0.00068 | BMI | 0.0172 | 0.0138 |  | |  |  |  |
| rs11874191 | 18 | 36164084 | T | C | 0.7278 | -0.0169 | 0.0029 | 3.06E-09 | 0.002179 | 0.002528 | 0.39 | BMI | -0.0318 | 0.0153 |  | |  |  |  |
| rs2612575 | 18 | 42967340 | A | G | 0.2763 | -0.016 | 0.0028 | 1.45E-08 | 4.49E-05 | 0.002481 | 0.99 | BMI | -0.0145 | 0.0155 |  | |  |  |  |
| rs784256 | 18 | 53398626 | G | A | 0.188 | -0.0108 | 0.0033 | 0.000905 | 0.018508 | 0.00283 | 6.2E-11 | PC | 0.0097 | 0.0177 |  | |  |  |  |
| rs9951619 | 18 | 56882326 | T | G | 0.222 | -0.0186 | 0.0028 | 1.75E-11 | -0.00068 | 0.00263 | 0.79 | BMI | -0.0128 | 0.0159 |  | |  |  |  |
| rs6567160 | 18 | 57829135 | T | C | 0.7486 | -0.0533 | 0.0028 | 2.11E-82 | -0.00363 | 0.0026 | 0.16 | BMI | -0.0188 | 0.0159 |  | |  |  |  |
| rs17066856 | 18 | 58049656 | T | C | 0.8896 | 0.0301 | 0.0039 | 2.65E-14 | 0.005579 | 0.003826 | 0.14 | BMI | 0.0446 | 0.0234 |  | |  |  |  |
| rs17710386 | 18 | 63461201 | T | C | 0.6644 | -0.0164 | 0.0025 | 3.75E-11 | 0.003471 | 0.002379 | 0.14 | BMI | 0.0093 | 0.0146 |  | |  |  |  |
| rs1787267 | 18 | 76742544 | C | G | 0.0629 | -0.0327 | 0.0052 | 2.49E-10 | 0.003056 | 0.004588 | 0.51 | BMI | 0.0283 | 0.0296 |  | |  |  |  |
| rs45486197 | 19 | 2244849 | A | G | 0.0635 | 0.0352 | 0.006 | 3.49E-09 | 0.006913 | 0.004463 | 0.12 | BMI | -0.025 | 0.0337 |  | |  |  |  |
| rs7258309 | 19 | 4016005 | T | G | 0.784 | 0.0233 | 0.0031 | 6.54E-14 | 0.006682 | 0.002725 | 0.014 | BMI | -0.0249 | 0.0174 |  | |  |  |  |
| rs273496 | 19 | 18241857 | T | C | 0.3881 | 0.0197 | 0.0029 | 2.5E-11 | 0.002149 | 0.002257 | 0.34 | BMI | -0.012 | 0.0138 |  | |  |  |  |
| rs7258722 | 19 | 18808915 | A | T | 0.4114 | -0.0199 | 0.0026 | 8.61E-15 | 0.000995 | 0.002252 | 0.66 | BMI | -0.0361 | 0.0142 |  | |  |  |  |
| rs17513613 | 19 | 30286822 | T | C | 0.6946 | -0.0179 | 0.0025 | 1.5E-12 | -0.0029 | 0.002351 | 0.22 | BMI | -0.0205 | 0.0145 |  | |  |  |  |
| rs11084553 | 19 | 31019780 | A | G | 0.8486 | 0.0237 | 0.0035 | 9.41E-12 | -0.00223 | 0.003035 | 0.46 | BMI | 0.0308 | 0.0201 |  | |  |  |  |
| rs769449 | 19 | 45410002 | A | G | 0.1019 | -0.0242 | 0.004 | 1.58E-09 | -0.01 | 0.003304 | 0.0025 | BMI | 0.0165 | 0.022 |  | |  |  |  |
| rs1800437 | 19 | 46181392 | C | G | 0.1861 | -0.0365 | 0.003 | 1.73E-33 | -0.00388 | 0.002779 | 0.16 | BMI | -0.0064 | 0.0165 |  | |  |  |  |
| rs9304665 | 19 | 47602577 | A | T | 0.7407 | 0.0274 | 0.0029 | 4.78E-21 | 0.004207 | 0.002602 | 0.11 | BMI | -0.0124 | 0.0165 |  | |  |  |  |
| rs7249149 | 19 | 51774538 | A | G | 0.6956 | 0.0152 | 0.0028 | 4.41E-08 | 0.00145 | 0.002426 | 0.55 | BMI | 0.0054 | 0.0156 |  | |  |  |  |
| rs2145270 | 20 | 6621685 | T | C | 0.6163 | 0.0208 | 0.0024 | 6.97E-18 | 0.000411 | 0.002267 | 0.86 | BMI | -0.0254 | 0.0142 |  | |  |  |  |
| rs8123881 | 20 | 15819495 | A | G | 0.8643 | -0.0192 | 0.0034 | 2.53E-08 | 0.007672 | 0.003261 | 0.019 | BMI | 0.0396 | 0.0204 |  | |  |  |  |
| rs7272093 | 20 | 49810724 | A | G | 0.7001 | -0.0172 | 0.0028 | 1.1E-09 | 0.002831 | 0.002419 | 0.24 | BMI | 0.01 | 0.0155 |  | |  |  |  |
| rs35388084 | 20 | 50960490 | A | G | 0.1814 | -0.0277 | 0.0037 | 1.12E-13 | -0.00663 | 0.002838 | 0.02 | BMI | -0.0313 | 0.0176 |  | |  |  |  |
| rs6092143 | 20 | 54181875 | T | C | 0.3676 | 0.0149 | 0.0026 | 1.1E-08 | 0.001662 | 0.002288 | 0.47 | BMI | -0.0046 | 0.0141 |  | |  |  |  |
| rs6129084 | 20 | 59828857 | A | T | 0.359 | -0.0038 | 0.003 | 0.2088 | -0.01409 | 0.002303 | 9.3E-10 | PC | 0.019 | 0.0141 |  | |  |  |  |
| rs13047416 | 21 | 40309436 | C | G | 0.6277 | 0.0157 | 0.0026 | 9.84E-10 | -0.00044 | 0.002281 | 0.85 | BMI | -0.0213 | 0.014 |  | |  |  |  |
| rs427943 | 21 | 46570896 | A | C | 0.4265 | -0.0175 | 0.0025 | 2.19E-12 | 0.001489 | 0.002225 | 0.5 | BMI | 0.0031 | 0.0137 |  | |  |  |  |
| rs136553 | 22 | 27255675 | C | T | 0.624 | -0.0019 | 0.003 | 0.5128 | -0.01523 | 0.002277 | 2.3E-11 | PC | 0.0239 | 0.014 |  | |  |  |  |
| rs138383 | 22 | 38799861 | T | C | 0.5144 | -0.0155 | 0.0026 | 1.39E-09 | -0.00556 | 0.002222 | 0.012 | BMI | -0.0118 | 0.0139 |  | |  |  |  |
| rs7288455 | 22 | 39966547 | A | G | 0.436 | -0.0044 | 0.0029 | 0.1277 | 0.013109 | 0.002227 | 4E-09 | PC | -0.0036 | 0.0142 |  | |  |  |  |
| rs733381 | 22 | 40669648 | A | G | 0.7669 | 0.0206 | 0.0029 | 5.2E-13 | -0.00486 | 0.002687 | 0.071 | BMI | 0.0265 | 0.0164 |  | |  |  |  |
| **3) Analysis of colorectal cancer in women only** | | | | | | | | | | | | | | | | | | | |
| SNP | CHR | POS | EA | OA | EAF | beta_bmi | se_bmi | pval_bmi | beta_pc | se_pc | pval | phenotype | beta_crc_women | se_crc_women |  | |  |  |  |
| rs2748985 | 1 | 1853184 | T | C | 0.455 | -0.015 | 0.003 | 3.64E-08 | -0.015 | 0.002 | 4.10E-12 | PC | -0.003 | 0.014 |  | |  |  |  |
| rs10909880 | 1 | 2727804 | T | C | 0.4531 | -0.012 | 0.002 | 1.97E-08 | -0.006 | 0.002 | 7.30E-03 | BMI | -0.013 | 0.014 |  | |  |  |  |
| rs78886584 | 1 | 16859325 | A | G | 0.5011 | -0.015 | 0.003 | 1.30E-08 | 0.002 | 0.002 | 4.30E-01 | BMI | -0.024 | 0.016 |  | |  |  |  |
| rs4295917 | 1 | 32173360 | A | G | 0.6522 | 0.014 | 0.002 | 1.62E-08 | 0.000 | 0.002 | 9.10E-01 | BMI | -0.001 | 0.014 |  | |  |  |  |
| rs112646560 | 1 | 39560250 | T | C | 0.212 | 0.020 | 0.003 | 1.14E-09 | 0.003 | 0.003 | 3.40E-01 | BMI | -0.005 | 0.017 |  | |  |  |  |
| rs2984618 | 1 | 47690438 | T | G | 0.4426 | 0.017 | 0.002 | 2.21E-14 | 0.004 | 0.002 | 9.30E-02 | BMI | -0.017 | 0.014 |  | |  |  |  |
| rs657452 | 1 | 49589847 | A | G | 0.4045 | 0.020 | 0.002 | 1.14E-19 | -0.005 | 0.002 | 4.00E-02 | BMI | 0.001 | 0.014 |  | |  |  |  |
| rs12140153 | 1 | 62579891 | T | G | 0.0905 | -0.033 | 0.005 | 2.87E-12 | 0.004 | 0.004 | 3.20E-01 | BMI | -0.028 | 0.027 |  | |  |  |  |
| rs2568958 | 1 | 72765116 | A | G | 0.6187 | 0.023 | 0.002 | 1.32E-24 | 0.007 | 0.002 | 1.20E-03 | BMI | -0.005 | 0.014 |  | |  |  |  |
| rs6669189 | 1 | 75011358 | T | C | 0.4051 | 0.021 | 0.002 | 4.69E-21 | -0.004 | 0.002 | 5.80E-02 | BMI | -0.008 | 0.014 |  | |  |  |  |
| rs34517439 | 1 | 78450517 | A | C | 0.1173 | 0.039 | 0.004 | 2.00E-21 | 0.003 | 0.003 | 3.90E-01 | BMI | 0.007 | 0.024 |  | |  |  |  |
| rs12089815 | 1 | 91189933 | A | G | 0.5527 | -0.015 | 0.003 | 6.02E-09 | 0.003 | 0.002 | 1.90E-01 | BMI | 0.015 | 0.014 |  | |  |  |  |
| rs11165643 | 1 | 96924097 | T | C | 0.5793 | 0.018 | 0.002 | 1.71E-16 | 0.003 | 0.002 | 1.30E-01 | BMI | 0.017 | 0.014 |  | |  |  |  |
| rs4421623 | 1 | 98316285 | T | G | 0.2103 | 0.018 | 0.003 | 1.13E-11 | -0.002 | 0.003 | 4.70E-01 | BMI | -0.022 | 0.017 |  | |  |  |  |
| rs12145677 | 1 | 110023610 | G | A | 0.702 | -0.011 | 0.002 | 1.50E-05 | -0.017 | 0.002 | 1.20E-12 | PC | -0.023 | 0.015 |  | |  |  |  |
| rs61813324 | 1 | 156049877 | T | C | 0.1308 | 0.030 | 0.004 | 3.60E-14 | 0.000 | 0.003 | 9.50E-01 | BMI | 0.063 | 0.024 |  | |  |  |  |
| rs1750307 | 1 | 156488420 | A | T | 0.365 | 0.014 | 0.002 | 8.22E-09 | -0.002 | 0.002 | 3.00E-01 | BMI | 0.017 | 0.014 |  | |  |  |  |
| rs12564992 | 1 | 174478100 | A | G | 0.8875 | -0.021 | 0.004 | 2.37E-09 | -0.013 | 0.004 | 1.70E-04 | BMI | -0.012 | 0.021 |  | |  |  |  |
| rs543874 | 1 | 177889480 | A | G | 0.7768 | -0.058 | 0.003 | 8.70E-100 | 0.002 | 0.003 | 4.50E-01 | BMI | -0.017 | 0.017 |  | |  |  |  |
| rs10920678 | 1 | 190239907 | A | G | 0.4244 | 0.016 | 0.002 | 7.46E-14 | 0.001 | 0.002 | 7.70E-01 | BMI | -0.001 | 0.014 |  | |  |  |  |
| rs4474229 | 1 | 195037001 | A | G | 0.3694 | -0.017 | 0.003 | 1.64E-10 | -0.002 | 0.002 | 4.80E-01 | BMI | -0.009 | 0.014 |  | |  |  |  |
| rs7526456 | 1 | 197773734 | T | C | 0.413 | 0.010 | 0.002 | 3.75E-05 | -0.013 | 0.002 | 1.20E-08 | PC | -0.009 | 0.014 |  | |  |  |  |
| rs2820295 | 1 | 201800868 | A | G | 0.3253 | 0.021 | 0.002 | 8.03E-19 | -0.006 | 0.002 | 1.00E-02 | BMI | 0.026 | 0.015 |  | |  |  |  |
| rs17014375 | 1 | 209543560 | T | G | 0.8704 | -0.019 | 0.003 | 1.46E-08 | 0.009 | 0.003 | 6.10E-03 | BMI | 0.049 | 0.020 |  | |  |  |  |
| rs12042959 | 1 | 243533273 | A | G | 0.8646 | 0.019 | 0.003 | 2.33E-09 | -0.008 | 0.003 | 1.70E-02 | BMI | -0.002 | 0.019 |  | |  |  |  |
| rs62106258 | 2 | 417167 | T | C | 0.9532 | 0.099 | 0.006 | 8.72E-58 | 0.009 | 0.005 | 7.80E-02 | BMI | 0.041 | 0.036 |  | |  |  |  |
| rs6548237 | 2 | 621461 | A | C | 0.1598 | -0.058 | 0.003 | 4.43E-89 | -0.008 | 0.003 | 3.50E-03 | BMI | -0.004 | 0.018 |  | |  |  |  |
| rs7571496 | 2 | 6169351 | A | G | 0.7433 | 0.015 | 0.003 | 1.45E-08 | -0.002 | 0.003 | 3.80E-01 | BMI | 0.008 | 0.015 |  | |  |  |  |
| rs7601870 | 2 | 15384392 | A | G | 0.4526 | -0.013 | 0.002 | 1.23E-08 | 0.003 | 0.002 | 2.10E-01 | BMI | -0.013 | 0.014 |  | |  |  |  |
| rs10182181 | 2 | 25150296 | A | G | 0.5049 | -0.036 | 0.002 | 5.00E-63 | -0.004 | 0.002 | 1.10E-01 | BMI | -0.011 | 0.014 |  | |  |  |  |
| rs10048652 | 2 | 35449306 | T | C | 0.4495 | 0.014 | 0.002 | 3.47E-09 | 0.000 | 0.002 | 9.30E-01 | BMI | 0.010 | 0.014 |  | |  |  |  |
| rs786420 | 2 | 44719893 | T | C | 0.7715 | 0.016 | 0.003 | 8.84E-09 | 0.007 | 0.003 | 4.70E-03 | BMI | 0.009 | 0.016 |  | |  |  |  |
| rs35722922 | 2 | 47002226 | A | G | 0.6255 | 0.019 | 0.003 | 3.65E-12 | 0.002 | 0.002 | 4.40E-01 | BMI | -0.018 | 0.014 |  | |  |  |  |
| rs1421598 | 2 | 50264710 | A | C | 0.8411 | -0.019 | 0.003 | 6.78E-10 | 0.001 | 0.003 | 7.80E-01 | BMI | -0.019 | 0.018 |  | |  |  |  |
| rs7601895 | 2 | 55281901 | C | G | 0.6892 | 0.016 | 0.003 | 9.61E-11 | 0.004 | 0.002 | 9.70E-02 | BMI | 0.005 | 0.015 |  | |  |  |  |
| rs1861412 | 2 | 58893065 | A | G | 0.4233 | -0.022 | 0.002 | 9.13E-21 | -0.005 | 0.002 | 1.90E-02 | BMI | 0.009 | 0.014 |  | |  |  |  |
| rs6728037 | 2 | 59297346 | A | G | 0.3856 | 0.021 | 0.002 | 6.49E-19 | -0.001 | 0.002 | 6.50E-01 | BMI | 0.006 | 0.014 |  | |  |  |  |
| rs2861685 | 2 | 67837553 | T | C | 0.5861 | 0.018 | 0.003 | 1.05E-11 | 0.003 | 0.002 | 1.40E-01 | BMI | -0.002 | 0.014 |  | |  |  |  |
| rs12714199 | 2 | 86812549 | T | C | 0.6145 | -0.015 | 0.002 | 5.12E-10 | -0.001 | 0.002 | 7.90E-01 | BMI | 0.007 | 0.014 |  | |  |  |  |
| rs11677607 | 2 | 100751150 | T | C | 0.246 | -0.024 | 0.003 | 2.87E-19 | 0.006 | 0.003 | 3.10E-02 | BMI | 0.005 | 0.016 |  | |  |  |  |
| rs1457457 | 2 | 104340148 | A | T | 0.5319 | 0.014 | 0.002 | 3.79E-10 | -0.004 | 0.002 | 7.60E-02 | BMI | -0.002 | 0.014 |  | |  |  |  |
| rs6739199 | 2 | 105452326 | T | C | 0.2695 | 0.016 | 0.003 | 3.20E-10 | -0.007 | 0.002 | 4.30E-03 | BMI | -0.023 | 0.015 |  | |  |  |  |
| rs1984559 | 2 | 135678888 | A | G | 0.8083 | 0.016 | 0.003 | 3.27E-08 | 0.001 | 0.003 | 6.70E-01 | BMI | -0.004 | 0.016 |  | |  |  |  |
| rs13002158 | 2 | 144021712 | A | G | 0.8125 | -0.021 | 0.003 | 1.15E-12 | -0.006 | 0.003 | 3.60E-02 | BMI | -0.001 | 0.017 |  | |  |  |  |
| rs76900466 | 2 | 145057760 | T | G | 0.8829 | 0.023 | 0.004 | 4.53E-08 | -0.001 | 0.003 | 8.00E-01 | BMI | 0.022 | 0.024 |  | |  |  |  |
| rs10754920 | 2 | 146456888 | C | G | 0.112 | 0.008 | 0.004 | 3.86E-02 | 0.020 | 0.003 | 6.70E-09 | PC | 0.011 | 0.020 |  | |  |  |  |
| rs6744254 | 2 | 155986195 | C | T | 0.471 | -0.005 | 0.002 | 4.60E-02 | -0.016 | 0.002 | 5.40E-13 | PC | -0.012 | 0.014 |  | |  |  |  |
| rs7588437 | 2 | 181575281 | A | G | 0.3672 | -0.018 | 0.002 | 8.38E-15 | 0.002 | 0.002 | 3.50E-01 | BMI | 0.010 | 0.014 |  | |  |  |  |
| rs7594237 | 2 | 198187164 | A | T | 0.277 | -0.014 | 0.003 | 1.81E-08 | 0.003 | 0.002 | 1.90E-01 | BMI | 0.012 | 0.015 |  | |  |  |  |
| rs4482463 | 2 | 205375909 | A | C | 0.9169 | -0.037 | 0.004 | 5.21E-18 | -0.005 | 0.004 | 2.60E-01 | BMI | 0.024 | 0.024 |  | |  |  |  |
| rs4673553 | 2 | 211608379 | T | G | 0.5552 | -0.015 | 0.002 | 2.59E-11 | 0.000 | 0.002 | 9.00E-01 | BMI | 0.004 | 0.014 |  | |  |  |  |
| rs11274218 | 2 | 215035001 | C | T | 0.611 | 0.004 | 0.002 | 8.24E-02 | -0.014 | 0.002 | 2.50E-09 | PC | -0.016 | 0.014 |  | |  |  |  |
| rs10208088 | 2 | 221055873 | C | T | 0.419 | 0.000 | 0.003 | 9.66E-01 | 0.013 | 0.002 | 4.40E-09 | PC | -0.006 | 0.014 |  | |  |  |  |
| rs12612009 | 2 | 228982603 | T | G | 0.6627 | -0.014 | 0.002 | 3.78E-09 | 0.003 | 0.002 | 2.80E-01 | BMI | -0.004 | 0.014 |  | |  |  |  |
| rs6720868 | 2 | 230663576 | T | C | 0.3063 | 0.017 | 0.002 | 4.65E-12 | 0.000 | 0.002 | 9.40E-01 | BMI | -0.013 | 0.014 |  | |  |  |  |
| rs1801282 | 3 | 12393125 | C | G | 0.8974 | -0.018 | 0.003 | 4.72E-08 | 0.000 | 0.003 | 9.80E-01 | BMI | 0.004 | 0.021 |  | |  |  |  |
| rs2470520 | 3 | 15762920 | T | C | 0.5774 | -0.013 | 0.002 | 4.16E-08 | -0.002 | 0.002 | 3.50E-01 | BMI | -0.010 | 0.014 |  | |  |  |  |
| rs4858223 | 3 | 20581149 | T | C | 0.3263 | -0.016 | 0.003 | 8.26E-11 | 0.000 | 0.002 | 9.50E-01 | BMI | -0.003 | 0.015 |  | |  |  |  |
| rs13062093 | 3 | 35667057 | T | G | 0.632 | -0.014 | 0.002 | 8.00E-09 | 0.000 | 0.002 | 8.40E-01 | BMI | 0.003 | 0.014 |  | |  |  |  |
| rs11708955 | 3 | 49540114 | T | C | 0.695 | 0.015 | 0.003 | 2.36E-09 | -0.016 | 0.002 | 4.20E-11 | PC | -0.006 | 0.015 |  | |  |  |  |
| rs6774533 | 3 | 62471086 | C | T | 0.294 | -0.016 | 0.003 | 2.30E-08 | -0.015 | 0.002 | 1.20E-09 | PC | -0.001 | 0.015 |  | |  |  |  |
| rs7647242 | 3 | 82642734 | T | C | 0.3941 | 0.015 | 0.002 | 3.92E-10 | -0.003 | 0.002 | 1.80E-01 | BMI | 0.005 | 0.014 |  | |  |  |  |
| rs13068138 | 3 | 85809425 | T | C | 0.1915 | 0.023 | 0.003 | 3.60E-17 | 0.005 | 0.003 | 7.70E-02 | BMI | -0.021 | 0.017 |  | |  |  |  |
| rs1949197 | 3 | 86273850 | A | G | 0.4013 | 0.014 | 0.002 | 3.04E-09 | 0.003 | 0.002 | 1.40E-01 | BMI | -0.001 | 0.014 |  | |  |  |  |
| rs1492014 | 3 | 94071481 | T | C | 0.5681 | -0.015 | 0.002 | 2.84E-10 | 0.002 | 0.002 | 2.80E-01 | BMI | -0.002 | 0.014 |  | |  |  |  |
| rs1436348 | 3 | 104612668 | A | G | 0.4318 | -0.013 | 0.002 | 6.31E-09 | 0.004 | 0.002 | 6.90E-02 | BMI | -0.030 | 0.014 |  | |  |  |  |
| rs1289736 | 3 | 107950892 | T | C | 0.5434 | 0.014 | 0.002 | 1.43E-09 | -0.004 | 0.002 | 7.80E-02 | BMI | 0.008 | 0.014 |  | |  |  |  |
| rs17619973 | 3 | 114417675 | A | G | 0.9361 | 0.024 | 0.004 | 1.29E-08 | 0.000 | 0.004 | 9.20E-01 | BMI | -0.027 | 0.027 |  | |  |  |  |
| rs78082503 | 3 | 116556913 | G | C | 0.786 | -0.001 | 0.003 | 7.24E-01 | -0.015 | 0.003 | 1.10E-08 | PC | 0.004 | 0.016 |  | |  |  |  |
| rs7428670 | 3 | 131579810 | C | G | 0.2691 | 0.020 | 0.003 | 3.09E-15 | -0.004 | 0.002 | 1.30E-01 | BMI | 0.023 | 0.016 |  | |  |  |  |
| rs7621025 | 3 | 136272246 | T | C | 0.2608 | -0.020 | 0.003 | 1.62E-15 | -0.008 | 0.003 | 1.10E-03 | BMI | -0.029 | 0.016 |  | |  |  |  |
| rs16851483 | 3 | 141275436 | T | G | 0.0728 | 0.036 | 0.005 | 2.28E-15 | 0.006 | 0.004 | 1.80E-01 | BMI | 0.028 | 0.026 |  | |  |  |  |
| rs355754 | 3 | 154024165 | T | C | 0.6041 | -0.014 | 0.002 | 4.26E-10 | 0.004 | 0.002 | 1.20E-01 | BMI | -0.008 | 0.014 |  | |  |  |  |
| rs247975 | 3 | 173107443 | T | C | 0.4659 | -0.018 | 0.002 | 3.62E-15 | 0.006 | 0.002 | 5.60E-03 | BMI | 0.016 | 0.014 |  | |  |  |  |
| rs10513801 | 3 | 185822353 | T | G | 0.8778 | 0.035 | 0.003 | 1.26E-27 | -0.004 | 0.003 | 2.50E-01 | BMI | -0.019 | 0.021 |  | |  |  |  |
| rs4677812 | 3 | 194863794 | A | C | 0.2929 | -0.014 | 0.003 | 9.38E-09 | 0.000 | 0.002 | 9.90E-01 | BMI | -0.026 | 0.015 |  | |  |  |  |
| rs994596 | 4 | 18459828 | T | C | 0.3254 | 0.015 | 0.003 | 1.02E-09 | 0.002 | 0.002 | 4.40E-01 | BMI | 0.003 | 0.014 |  | |  |  |  |
| rs34811474 | 4 | 25408838 | A | G | 0.222 | -0.032 | 0.003 | 5.19E-24 | 0.003 | 0.003 | 2.80E-01 | BMI | -0.035 | 0.019 |  | |  |  |  |
| rs73213484 | 4 | 28489339 | A | T | 0.8566 | 0.025 | 0.004 | 2.28E-11 | 0.003 | 0.003 | 3.20E-01 | BMI | 0.014 | 0.019 |  | |  |  |  |
| rs337637 | 4 | 38604470 | A | G | 0.3576 | -0.013 | 0.002 | 3.04E-08 | 0.005 | 0.002 | 1.90E-02 | BMI | -0.031 | 0.014 |  | |  |  |  |
| rs6857629 | 4 | 39684977 | G | A | 0.755 | -0.004 | 0.003 | 1.99E-01 | 0.015 | 0.003 | 1.90E-09 | PC | 0.012 | 0.016 |  | |  |  |  |
| rs2271046 | 4 | 52752812 | A | T | 0.6942 | -0.014 | 0.003 | 2.38E-08 | 0.004 | 0.002 | 6.50E-02 | BMI | -0.019 | 0.015 |  | |  |  |  |
| rs11727273 | 4 | 55509748 | T | C | 0.5549 | -0.017 | 0.003 | 1.28E-10 | -0.002 | 0.002 | 3.00E-01 | BMI | 0.022 | 0.014 |  | |  |  |  |
| rs11945861 | 4 | 65700865 | A | G | 0.2466 | -0.015 | 0.003 | 3.13E-08 | 0.004 | 0.003 | 9.50E-02 | BMI | -0.005 | 0.016 |  | |  |  |  |
| rs10002111 | 4 | 67815504 | A | G | 0.2244 | 0.017 | 0.003 | 9.26E-10 | -0.001 | 0.003 | 7.50E-01 | BMI | 0.022 | 0.016 |  | |  |  |  |
| rs4148155 | 4 | 89054667 | A | G | 0.8866 | 0.020 | 0.004 | 6.20E-09 | -0.009 | 0.003 | 6.90E-03 | BMI | -0.015 | 0.022 |  | |  |  |  |
| rs12645001 | 4 | 96018337 | A | G | 0.3556 | -0.014 | 0.002 | 4.11E-09 | 0.002 | 0.002 | 3.30E-01 | BMI | 0.011 | 0.014 |  | |  |  |  |
| rs13107325 | 4 | 103188709 | T | C | 0.0826 | 0.045 | 0.004 | 9.33E-25 | 0.001 | 0.004 | 7.60E-01 | BMI | -0.028 | 0.027 |  | |  |  |  |
| rs4834272 | 4 | 113313986 | T | C | 0.6866 | -0.014 | 0.002 | 8.24E-10 | 0.000 | 0.002 | 8.80E-01 | BMI | -0.001 | 0.014 |  | |  |  |  |
| rs1403846 | 4 | 119101723 | T | C | 0.8331 | 0.016 | 0.003 | 1.62E-08 | 0.000 | 0.003 | 9.80E-01 | BMI | 0.028 | 0.018 |  | |  |  |  |
| rs4864201 | 4 | 130731284 | T | C | 0.3461 | 0.013 | 0.002 | 6.34E-09 | 0.000 | 0.002 | 9.30E-01 | BMI | 0.013 | 0.014 |  | |  |  |  |
| rs57800857 | 4 | 140863365 | A | C | 0.6334 | 0.019 | 0.003 | 1.07E-11 | 0.006 | 0.002 | 1.40E-02 | BMI | 0.001 | 0.014 |  | |  |  |  |
| rs35107973 | 4 | 143125948 | T | C | 0.1173 | 0.023 | 0.004 | 7.66E-09 | -0.004 | 0.003 | 2.70E-01 | BMI | 0.026 | 0.022 |  | |  |  |  |
| rs2068625 | 4 | 159856739 | T | C | 0.302 | 0.010 | 0.003 | 3.92E-05 | -0.016 | 0.002 | 4.00E-11 | PC | 0.020 | 0.015 |  | |  |  |  |
| rs13110266 | 4 | 162129844 | A | G | 0.4027 | -0.014 | 0.002 | 1.55E-10 | -0.001 | 0.002 | 7.30E-01 | BMI | -0.003 | 0.014 |  | |  |  |  |
| rs1491333 | 4 | 172186288 | T | G | 0.2634 | -0.015 | 0.003 | 9.85E-09 | 0.002 | 0.003 | 4.60E-01 | BMI | 0.000 | 0.015 |  | |  |  |  |
| rs2220599 | 5 | 7378854 | C | G | 0.633 | 0.006 | 0.002 | 8.26E-03 | -0.016 | 0.002 | 2.40E-12 | PC | 0.000 | 0.014 |  | |  |  |  |
| rs6890310 | 5 | 27193573 | A | G | 0.294 | -0.015 | 0.003 | 1.38E-09 | 0.001 | 0.002 | 7.10E-01 | BMI | 0.029 | 0.015 |  | |  |  |  |
| rs782971 | 5 | 43124688 | A | G | 0.7544 | -0.017 | 0.003 | 4.89E-12 | -0.002 | 0.003 | 4.70E-01 | BMI | -0.023 | 0.016 |  | |  |  |  |
| rs1812587 | 5 | 62991802 | T | G | 0.4578 | 0.015 | 0.003 | 6.95E-09 | 0.002 | 0.002 | 4.60E-01 | BMI | -0.026 | 0.014 |  | |  |  |  |
| rs10471636 | 5 | 63979856 | A | G | 0.5248 | -0.017 | 0.002 | 4.66E-13 | -0.004 | 0.002 | 8.40E-02 | BMI | -0.006 | 0.014 |  | |  |  |  |
| rs2112347 | 5 | 75015242 | T | G | 0.6296 | 0.031 | 0.002 | 2.13E-42 | -0.005 | 0.002 | 4.70E-02 | BMI | 0.003 | 0.014 |  | |  |  |  |
| rs11749912 | 5 | 88065628 | A | G | 0.423 | 0.006 | 0.003 | 1.79E-02 | 0.014 | 0.002 | 4.90E-10 | PC | 0.023 | 0.014 |  | |  |  |  |
| rs158186 | 5 | 107440856 | A | G | 0.166 | -0.029 | 0.003 | 3.23E-20 | -0.003 | 0.003 | 3.90E-01 | BMI | -0.011 | 0.018 |  | |  |  |  |
| rs1469249 | 5 | 113837198 | G | A | 0.789 | -0.012 | 0.003 | 3.12E-05 | 0.016 | 0.003 | 9.50E-09 | PC | 0.005 | 0.017 |  | |  |  |  |
| rs162894 | 5 | 131611872 | T | G | 0.332 | 0.003 | 0.002 | 2.43E-01 | 0.013 | 0.002 | 1.10E-08 | PC | -0.007 | 0.014 |  | |  |  |  |
| rs56158102 | 5 | 144027803 | C | T | 0.344 | 0.000 | 0.002 | 9.11E-01 | 0.013 | 0.002 | 3.10E-08 | PC | -0.014 | 0.014 |  | |  |  |  |
| rs4569924 | 5 | 153540025 | T | C | 0.4354 | 0.016 | 0.002 | 7.44E-13 | -0.004 | 0.002 | 1.00E-01 | BMI | 0.014 | 0.014 |  | |  |  |  |
| rs6556810 | 5 | 164585949 | T | C | 0.5335 | -0.012 | 0.002 | 4.98E-08 | -0.002 | 0.002 | 3.20E-01 | BMI | -0.027 | 0.014 |  | |  |  |  |
| rs2053682 | 5 | 170599327 | A | C | 0.6776 | 0.018 | 0.003 | 1.89E-13 | 0.004 | 0.002 | 6.30E-02 | BMI | -0.008 | 0.015 |  | |  |  |  |
| rs2228213 | 6 | 12124855 | A | G | 0.3374 | -0.015 | 0.002 | 9.21E-11 | -0.003 | 0.002 | 2.70E-01 | BMI | -0.004 | 0.014 |  | |  |  |  |
| rs9367369 | 6 | 13189941 | A | G | 0.7095 | 0.017 | 0.003 | 2.32E-09 | 0.003 | 0.002 | 2.00E-01 | BMI | -0.005 | 0.015 |  | |  |  |  |
| rs9477970 | 6 | 19082663 | T | A | 0.808 | -0.013 | 0.003 | 7.03E-06 | -0.016 | 0.003 | 1.00E-08 | PC | -0.004 | 0.018 |  | |  |  |  |
| rs2178899 | 6 | 31606756 | A | T | 0.8853 | 0.025 | 0.003 | 7.16E-14 | 0.001 | 0.003 | 7.20E-01 | BMI | 0.068 | 0.022 |  | |  |  |  |
| rs11751591 | 6 | 33794215 | A | G | 0.1526 | -0.019 | 0.003 | 1.16E-08 | -0.002 | 0.003 | 4.60E-01 | BMI | -0.007 | 0.019 |  | |  |  |  |
| rs2814992 | 6 | 34617144 | A | G | 0.6666 | -0.029 | 0.002 | 1.25E-35 | -0.005 | 0.002 | 3.90E-02 | BMI | 0.021 | 0.014 |  | |  |  |  |
| rs1579557 | 6 | 40371918 | T | C | 0.288 | 0.021 | 0.003 | 3.09E-17 | -0.001 | 0.002 | 8.30E-01 | BMI | 0.010 | 0.015 |  | |  |  |  |
| rs943005 | 6 | 50865820 | T | C | 0.1472 | 0.040 | 0.003 | 1.99E-43 | 0.003 | 0.003 | 3.30E-01 | BMI | -0.012 | 0.018 |  | |  |  |  |
| rs2465043 | 6 | 51180765 | A | G | 0.3512 | -0.014 | 0.002 | 3.22E-09 | 0.001 | 0.002 | 6.50E-01 | BMI | -0.012 | 0.014 |  | |  |  |  |
| rs62412826 | 6 | 61881167 | T | C | 0.0591 | 0.033 | 0.006 | 7.98E-09 | -0.003 | 0.005 | 5.40E-01 | BMI | 0.043 | 0.036 |  | |  |  |  |
| rs209416 | 6 | 83534729 | A | T | 0.3236 | 0.017 | 0.002 | 8.71E-12 | -0.001 | 0.002 | 6.10E-01 | BMI | 0.004 | 0.015 |  | |  |  |  |
| rs9372625 | 6 | 98344031 | G | A | 0.619 | 0.013 | 0.002 | 1.20E-07 | -0.018 | 0.002 | 5.70E-16 | PC | 0.030 | 0.014 |  | |  |  |  |
| rs768023 | 6 | 108876002 | A | G | 0.6025 | 0.019 | 0.002 | 1.38E-16 | 0.001 | 0.002 | 6.60E-01 | BMI | 0.001 | 0.014 |  | |  |  |  |
| rs2357760 | 6 | 120213880 | A | G | 0.6732 | 0.016 | 0.002 | 1.09E-11 | 0.004 | 0.002 | 9.60E-02 | BMI | -0.005 | 0.014 |  | |  |  |  |
| rs6935828 | 6 | 140811367 | C | T | 0.446 | -0.002 | 0.002 | 3.92E-01 | -0.012 | 0.002 | 4.30E-08 | PC | -0.004 | 0.014 |  | |  |  |  |
| rs11754747 | 6 | 141494602 | T | C | 0.2714 | 0.015 | 0.003 | 1.27E-08 | -0.004 | 0.003 | 1.40E-01 | BMI | -0.007 | 0.017 |  | |  |  |  |
| rs2185027 | 6 | 153381622 | A | C | 0.6977 | -0.015 | 0.002 | 1.11E-10 | -0.002 | 0.002 | 5.20E-01 | BMI | -0.002 | 0.015 |  | |  |  |  |
| rs12207125 | 6 | 154301575 | A | G | 0.7755 | 0.018 | 0.003 | 7.67E-09 | 0.006 | 0.003 | 3.50E-02 | BMI | -0.039 | 0.016 |  | |  |  |  |
| rs13213408 | 6 | 162998049 | A | T | 0.8471 | 0.020 | 0.003 | 4.33E-09 | -0.004 | 0.003 | 1.80E-01 | BMI | 0.030 | 0.021 |  | |  |  |  |
| rs6973700 | 7 | 1856669 | A | G | 0.7887 | 0.019 | 0.003 | 1.77E-09 | 0.004 | 0.003 | 2.00E-01 | BMI | -0.012 | 0.016 |  | |  |  |  |
| rs836525 | 7 | 6465909 | T | C | 0.1728 | 0.018 | 0.003 | 9.75E-09 | 0.004 | 0.003 | 2.20E-01 | BMI | -0.024 | 0.019 |  | |  |  |  |
| rs4307239 | 7 | 24354300 | A | G | 0.5357 | -0.014 | 0.002 | 1.31E-09 | 0.003 | 0.002 | 1.40E-01 | BMI | 0.004 | 0.014 |  | |  |  |  |
| rs11971098 | 7 | 26699061 | A | G | 0.9093 | -0.024 | 0.004 | 6.49E-09 | -0.009 | 0.004 | 2.10E-02 | BMI | -0.019 | 0.024 |  | |  |  |  |
| rs10499694 | 7 | 50614173 | A | G | 0.4938 | 0.014 | 0.002 | 3.99E-10 | 0.001 | 0.002 | 5.50E-01 | BMI | 0.009 | 0.014 |  | |  |  |  |
| rs55772938 | 7 | 70025113 | A | G | 0.705 | -0.008 | 0.003 | 4.34E-03 | -0.015 | 0.002 | 3.90E-10 | PC | -0.017 | 0.015 |  | |  |  |  |
| rs2267812 | 7 | 74138121 | A | C | 0.8071 | -0.017 | 0.003 | 1.74E-09 | -0.001 | 0.003 | 6.90E-01 | BMI | 0.020 | 0.017 |  | |  |  |  |
| rs7805441 | 7 | 78121458 | T | C | 0.5471 | 0.015 | 0.002 | 2.01E-10 | 0.000 | 0.002 | 9.50E-01 | BMI | 0.009 | 0.014 |  | |  |  |  |
| rs2528531 | 7 | 93236510 | A | C | 0.6506 | -0.015 | 0.002 | 1.35E-09 | 0.000 | 0.002 | 8.90E-01 | BMI | -0.007 | 0.014 |  | |  |  |  |
| rs10271582 | 7 | 99121950 | A | G | 0.8277 | 0.019 | 0.003 | 8.41E-09 | 0.012 | 0.003 | 7.40E-05 | BMI | 0.033 | 0.018 |  | |  |  |  |
| rs2299383 | 7 | 103418846 | T | C | 0.3952 | 0.018 | 0.002 | 2.73E-16 | 0.007 | 0.002 | 3.00E-03 | BMI | -0.008 | 0.014 |  | |  |  |  |
| rs7788008 | 7 | 112972483 | A | G | 0.4299 | -0.017 | 0.002 | 1.09E-13 | 0.004 | 0.002 | 5.20E-02 | BMI | -0.002 | 0.014 |  | |  |  |  |
| rs2045293 | 7 | 114350102 | T | C | 0.417 | 0.019 | 0.002 | 4.06E-16 | -0.005 | 0.002 | 2.30E-02 | BMI | 0.000 | 0.014 |  | |  |  |  |
| rs12706626 | 7 | 124531370 | G | A | 0.617 | 0.001 | 0.003 | 8.39E-01 | -0.013 | 0.002 | 1.40E-08 | PC | 0.016 | 0.014 |  | |  |  |  |
| rs3944151 | 7 | 126970005 | A | G | 0.275 | -0.001 | 0.003 | 7.41E-01 | 0.014 | 0.002 | 3.10E-08 | PC | 0.019 | 0.015 |  | |  |  |  |
| rs2345941 | 7 | 133337635 | A | G | 0.55 | -0.001 | 0.002 | 8.00E-01 | 0.015 | 0.002 | 4.00E-11 | PC | -0.033 | 0.014 |  | |  |  |  |
| rs2968864 | 7 | 150622162 | T | C | 0.7563 | 0.016 | 0.003 | 9.35E-10 | 0.003 | 0.003 | 2.50E-01 | BMI | 0.001 | 0.016 |  | |  |  |  |
| rs4240673 | 8 | 10787612 | T | C | 0.4555 | 0.018 | 0.002 | 1.03E-15 | 0.002 | 0.002 | 4.40E-01 | BMI | 0.006 | 0.015 |  | |  |  |  |
| rs2959592 | 8 | 17002725 | T | C | 0.8094 | 0.017 | 0.003 | 6.26E-10 | -0.001 | 0.003 | 7.00E-01 | BMI | 0.004 | 0.017 |  | |  |  |  |
| rs1362910 | 8 | 30856464 | A | G | 0.4125 | 0.013 | 0.002 | 3.03E-09 | -0.001 | 0.002 | 7.10E-01 | BMI | 0.009 | 0.014 |  | |  |  |  |
| rs4320543 | 8 | 34422967 | A | G | 0.4188 | -0.013 | 0.002 | 2.95E-08 | 0.004 | 0.002 | 1.20E-01 | BMI | -0.010 | 0.014 |  | |  |  |  |
| rs12681792 | 8 | 62054463 | A | C | 0.2036 | 0.018 | 0.003 | 2.61E-10 | 0.005 | 0.003 | 5.80E-02 | BMI | 0.003 | 0.018 |  | |  |  |  |
| rs7827848 | 8 | 64790774 | A | T | 0.5104 | 0.015 | 0.002 | 1.88E-11 | 0.001 | 0.002 | 5.40E-01 | BMI | 0.016 | 0.014 |  | |  |  |  |
| rs1808629 | 8 | 73435964 | A | G | 0.6744 | -0.023 | 0.003 | 5.76E-16 | -0.002 | 0.002 | 3.30E-01 | BMI | 0.023 | 0.015 |  | |  |  |  |
| rs17405819 | 8 | 76806584 | T | C | 0.683 | 0.022 | 0.002 | 7.81E-20 | 0.002 | 0.002 | 4.60E-01 | BMI | -0.006 | 0.015 |  | |  |  |  |
| rs12680842 | 8 | 95582606 | A | G | 0.6841 | 0.013 | 0.002 | 3.36E-08 | 0.000 | 0.002 | 8.60E-01 | BMI | 0.007 | 0.014 |  | |  |  |  |
| rs2721965 | 8 | 116662038 | A | C | 0.669 | 0.019 | 0.002 | 2.10E-14 | 0.002 | 0.002 | 3.50E-01 | BMI | -0.002 | 0.015 |  | |  |  |  |
| rs13262595 | 8 | 143316970 | A | G | 0.44 | -0.006 | 0.002 | 1.61E-02 | -0.016 | 0.002 | 1.20E-12 | PC | -0.008 | 0.014 |  | |  |  |  |
| rs10124645 | 9 | 11321119 | A | G | 0.592 | 0.014 | 0.002 | 4.98E-09 | 0.002 | 0.002 | 4.10E-01 | BMI | -0.012 | 0.014 |  | |  |  |  |
| rs10962550 | 9 | 16720329 | C | G | 0.1739 | 0.019 | 0.003 | 1.70E-10 | -0.002 | 0.003 | 4.00E-01 | BMI | 0.014 | 0.017 |  | |  |  |  |
| rs4977839 | 9 | 23355310 | G | A | 0.584 | -0.011 | 0.003 | 6.06E-05 | -0.020 | 0.002 | 4.70E-19 | PC | -0.005 | 0.014 |  | |  |  |  |
| rs16912921 | 9 | 28413461 | A | C | 0.3133 | 0.025 | 0.002 | 1.56E-26 | 0.005 | 0.002 | 4.30E-02 | BMI | 0.027 | 0.015 |  | |  |  |  |
| rs973345 | 9 | 29713169 | T | C | 0.6107 | 0.014 | 0.002 | 1.09E-09 | 0.003 | 0.002 | 2.00E-01 | BMI | 0.006 | 0.014 |  | |  |  |  |
| rs563132 | 9 | 37245476 | A | T | 0.605 | 0.018 | 0.002 | 1.96E-14 | 0.000 | 0.002 | 9.80E-01 | BMI | -0.006 | 0.014 |  | |  |  |  |
| rs10116186 | 9 | 80532374 | A | G | 0.4587 | -0.015 | 0.003 | 4.10E-08 | 0.000 | 0.002 | 9.90E-01 | BMI | 0.005 | 0.014 |  | |  |  |  |
| rs10992867 | 9 | 96461013 | A | G | 0.2692 | 0.019 | 0.003 | 8.89E-13 | -0.001 | 0.003 | 7.00E-01 | BMI | -0.007 | 0.015 |  | |  |  |  |
| rs113851275 | 9 | 98297220 | G | A | 0.892 | 0.005 | 0.004 | 2.79E-01 | -0.021 | 0.004 | 3.20E-09 | PC | 0.043 | 0.022 |  | |  |  |  |
| rs10118701 | 9 | 103061366 | A | G | 0.6718 | -0.018 | 0.002 | 2.20E-13 | 0.002 | 0.002 | 3.00E-01 | BMI | -0.022 | 0.015 |  | |  |  |  |
| rs7024334 | 9 | 109072075 | T | G | 0.226 | 0.016 | 0.003 | 1.63E-09 | 0.001 | 0.003 | 7.90E-01 | BMI | 0.020 | 0.016 |  | |  |  |  |
| rs7020477 | 9 | 116827760 | A | G | 0.735 | 0.003 | 0.003 | 3.01E-01 | 0.014 | 0.002 | 1.20E-08 | PC | 0.033 | 0.017 |  | |  |  |  |
| rs73578186 | 9 | 126334485 | C | T | 0.675 | -0.009 | 0.003 | 1.79E-03 | 0.015 | 0.002 | 2.30E-10 | PC | -0.006 | 0.015 |  | |  |  |  |
| rs3829849 | 9 | 129390800 | T | C | 0.3503 | 0.014 | 0.002 | 1.93E-09 | 0.001 | 0.002 | 6.60E-01 | BMI | 0.015 | 0.014 |  | |  |  |  |
| rs10733682 | 9 | 129460914 | A | G | 0.4583 | 0.016 | 0.002 | 1.85E-12 | -0.002 | 0.002 | 4.50E-01 | BMI | 0.000 | 0.014 |  | |  |  |  |
| rs1009473 | 9 | 138777312 | T | C | 0.5262 | -0.013 | 0.002 | 3.36E-08 | -0.001 | 0.002 | 6.40E-01 | BMI | -0.009 | 0.014 |  | |  |  |  |
| rs7084454 | 10 | 21821274 | A | G | 0.3064 | 0.023 | 0.003 | 7.43E-20 | 0.011 | 0.002 | 6.40E-06 | BMI | 0.027 | 0.015 |  | |  |  |  |
| rs34930419 | 10 | 33987329 | T | C | 0.9682 | -0.046 | 0.008 | 1.37E-09 | -0.008 | 0.006 | 1.90E-01 | BMI | -0.028 | 0.039 |  | |  |  |  |
| rs10823893 | 10 | 53677313 | A | G | 0.4056 | 0.018 | 0.003 | 5.22E-11 | -0.004 | 0.002 | 5.10E-02 | BMI | 0.028 | 0.014 |  | |  |  |  |
| rs10761785 | 10 | 65318766 | T | G | 0.5139 | -0.017 | 0.002 | 4.17E-15 | 0.001 | 0.002 | 6.30E-01 | BMI | 0.018 | 0.014 |  | |  |  |  |
| rs10824347 | 10 | 77645229 | A | T | 0.7707 | 0.018 | 0.003 | 5.47E-11 | -0.003 | 0.003 | 3.10E-01 | BMI | -0.007 | 0.016 |  | |  |  |  |
| rs7899106 | 10 | 87410904 | A | G | 0.9495 | -0.038 | 0.005 | 7.65E-14 | -0.003 | 0.005 | 5.20E-01 | BMI | 0.042 | 0.032 |  | |  |  |  |
| rs563296 | 10 | 99772404 | A | G | 0.5578 | 0.018 | 0.002 | 8.85E-14 | -0.001 | 0.002 | 7.60E-01 | BMI | 0.001 | 0.014 |  | |  |  |  |
| rs3977755 | 10 | 104420210 | T | C | 0.2869 | -0.014 | 0.003 | 8.50E-09 | 0.010 | 0.002 | 3.10E-05 | BMI | -0.013 | 0.015 |  | |  |  |  |
| rs6585198 | 10 | 114762237 | A | G | 0.4436 | -0.015 | 0.002 | 1.22E-12 | -0.004 | 0.002 | 8.60E-02 | BMI | 0.004 | 0.014 |  | |  |  |  |
| rs1681743 | 10 | 118565501 | T | C | 0.4013 | -0.013 | 0.002 | 3.70E-08 | -0.004 | 0.002 | 1.20E-01 | BMI | 0.004 | 0.014 |  | |  |  |  |
| rs1561589 | 10 | 126695673 | A | G | 0.3609 | 0.016 | 0.002 | 4.24E-12 | 0.003 | 0.002 | 2.50E-01 | BMI | 0.014 | 0.014 |  | |  |  |  |
| rs4880341 | 10 | 133992689 | T | C | 0.5716 | -0.014 | 0.002 | 4.53E-10 | -0.003 | 0.002 | 2.40E-01 | BMI | 0.004 | 0.014 |  | |  |  |  |
| rs7395632 | 11 | 958789 | T | C | 0.357 | -0.013 | 0.002 | 3.87E-08 | -0.003 | 0.002 | 1.80E-01 | BMI | 0.025 | 0.014 |  | |  |  |  |
| rs4929923 | 11 | 8639200 | T | C | 0.3365 | -0.022 | 0.002 | 2.51E-22 | 0.000 | 0.002 | 9.70E-01 | BMI | -0.008 | 0.014 |  | |  |  |  |
| rs11022762 | 11 | 13335926 | T | C | 0.3677 | -0.015 | 0.002 | 5.56E-11 | -0.008 | 0.002 | 2.20E-04 | BMI | 0.018 | 0.014 |  | |  |  |  |
| rs6265 | 11 | 27679916 | T | C | 0.1829 | -0.037 | 0.003 | 7.95E-40 | -0.011 | 0.003 | 4.70E-05 | BMI | 0.001 | 0.017 |  | |  |  |  |
| rs10742752 | 11 | 45438374 | T | C | 0.3753 | -0.013 | 0.002 | 3.98E-09 | -0.003 | 0.002 | 1.30E-01 | BMI | 0.023 | 0.014 |  | |  |  |  |
| rs7124681 | 11 | 47529947 | A | C | 0.4175 | 0.025 | 0.002 | 9.27E-29 | 0.009 | 0.002 | 7.20E-05 | BMI | 0.001 | 0.014 |  | |  |  |  |
| rs893006 | 11 | 64365796 | A | C | 0.721 | 0.017 | 0.002 | 4.05E-13 | -0.001 | 0.002 | 7.00E-01 | BMI | 0.013 | 0.015 |  | |  |  |  |
| rs625652 | 11 | 65641931 | A | T | 0.6403 | -0.020 | 0.003 | 9.05E-13 | -0.002 | 0.002 | 4.00E-01 | BMI | -0.029 | 0.014 |  | |  |  |  |
| rs7107409 | 11 | 76492195 | T | C | 0.2198 | -0.018 | 0.003 | 2.05E-08 | 0.000 | 0.003 | 9.10E-01 | BMI | 0.022 | 0.017 |  | |  |  |  |
| rs2605603 | 11 | 93221105 | A | G | 0.4805 | -0.013 | 0.002 | 1.40E-09 | 0.001 | 0.002 | 7.70E-01 | BMI | -0.030 | 0.014 |  | |  |  |  |
| rs2734849 | 11 | 113270160 | A | G | 0.491 | 0.008 | 0.002 | 2.10E-04 | -0.014 | 0.002 | 8.20E-10 | PC | -0.010 | 0.014 |  | |  |  |  |
| rs1003081 | 11 | 118913993 | T | C | 0.4349 | 0.014 | 0.002 | 6.27E-11 | -0.004 | 0.002 | 6.20E-02 | BMI | 0.019 | 0.014 |  | |  |  |  |
| rs4459316 | 11 | 130756176 | T | C | 0.5508 | -0.015 | 0.002 | 2.33E-11 | 0.002 | 0.002 | 3.10E-01 | BMI | 0.006 | 0.014 |  | |  |  |  |
| rs1448355 | 11 | 131286685 | C | T | 0.381 | 0.001 | 0.002 | 6.03E-01 | -0.015 | 0.002 | 1.30E-10 | PC | 0.030 | 0.014 |  | |  |  |  |
| rs329651 | 11 | 133767622 | T | G | 0.8054 | 0.018 | 0.003 | 1.96E-10 | -0.009 | 0.003 | 1.90E-03 | BMI | 0.004 | 0.018 |  | |  |  |  |
| rs12364470 | 11 | 134601012 | T | G | 0.855 | -0.020 | 0.003 | 2.54E-11 | -0.001 | 0.003 | 7.50E-01 | BMI | -0.013 | 0.020 |  | |  |  |  |
| rs11611246 | 12 | 939480 | T | G | 0.1991 | 0.021 | 0.003 | 1.27E-14 | 0.002 | 0.003 | 3.80E-01 | BMI | 0.017 | 0.017 |  | |  |  |  |
| rs7976757 | 12 | 19207948 | T | C | 0.1768 | -0.019 | 0.003 | 2.94E-10 | -0.002 | 0.003 | 5.30E-01 | BMI | -0.001 | 0.018 |  | |  |  |  |
| rs16926318 | 12 | 23678745 | A | G | 0.9208 | -0.023 | 0.004 | 4.23E-08 | 0.001 | 0.004 | 7.60E-01 | BMI | -0.005 | 0.023 |  | |  |  |  |
| rs16926778 | 12 | 24002125 | C | G | 0.0771 | 0.026 | 0.004 | 1.41E-09 | 0.006 | 0.004 | 1.30E-01 | BMI | 0.008 | 0.024 |  | |  |  |  |
| rs35933007 | 12 | 38929341 | G | A | 0.772 | 0.007 | 0.003 | 3.97E-02 | -0.015 | 0.003 | 9.20E-09 | PC | -0.017 | 0.019 |  | |  |  |  |
| rs7138803 | 12 | 50247468 | A | G | 0.3881 | 0.029 | 0.002 | 9.70E-38 | 0.000 | 0.002 | 9.80E-01 | BMI | 0.022 | 0.014 |  | |  |  |  |
| rs2271189 | 12 | 56494991 | A | G | 0.3958 | -0.015 | 0.002 | 6.35E-10 | 0.005 | 0.002 | 1.60E-02 | BMI | -0.011 | 0.014 |  | |  |  |  |
| rs11173522 | 12 | 60953472 | A | C | 0.2325 | 0.017 | 0.003 | 9.01E-10 | -0.002 | 0.003 | 5.40E-01 | BMI | 0.017 | 0.016 |  | |  |  |  |
| rs645026 | 12 | 69757931 | A | G | 0.7435 | -0.016 | 0.003 | 4.79E-10 | 0.001 | 0.002 | 7.30E-01 | BMI | 0.013 | 0.015 |  | |  |  |  |
| rs10746306 | 12 | 84477374 | T | G | 0.7673 | 0.018 | 0.003 | 8.74E-09 | 0.002 | 0.003 | 4.00E-01 | BMI | -0.014 | 0.016 |  | |  |  |  |
| rs2279574 | 12 | 89745477 | A | C | 0.5485 | -0.017 | 0.002 | 1.96E-12 | 0.001 | 0.002 | 6.90E-01 | BMI | 0.006 | 0.014 |  | |  |  |  |
| rs2712643 | 12 | 99638276 | A | C | 0.239 | -0.015 | 0.003 | 1.47E-09 | 0.004 | 0.003 | 1.00E-01 | BMI | -0.004 | 0.016 |  | |  |  |  |
| rs17033633 | 12 | 103656343 | T | C | 0.1692 | -0.021 | 0.003 | 7.26E-12 | 0.001 | 0.003 | 6.50E-01 | BMI | -0.004 | 0.018 |  | |  |  |  |
| rs17508240 | 12 | 117577358 | A | G | 0.2135 | 0.019 | 0.003 | 2.83E-09 | 0.002 | 0.003 | 3.60E-01 | BMI | 0.079 | 0.017 |  | |  |  |  |
| rs206965 | 12 | 120856332 | T | C | 0.207 | 0.000 | 0.003 | 9.16E-01 | 0.016 | 0.003 | 9.10E-09 | PC | 0.001 | 0.017 |  | |  |  |  |
| rs6489156 | 12 | 123100397 | T | C | 0.7336 | 0.022 | 0.003 | 9.97E-18 | -0.001 | 0.002 | 6.40E-01 | BMI | 0.019 | 0.015 |  | |  |  |  |
| rs9579775 | 13 | 20616557 | A | C | 0.8669 | -0.025 | 0.004 | 3.66E-10 | -0.001 | 0.003 | 8.80E-01 | BMI | -0.039 | 0.023 |  | |  |  |  |
| rs12872889 | 13 | 28674628 | T | C | 0.76 | -0.021 | 0.003 | 1.52E-11 | -0.009 | 0.003 | 9.80E-04 | BMI | -0.011 | 0.017 |  | |  |  |  |
| rs12429545 | 13 | 54102206 | A | G | 0.1204 | 0.029 | 0.003 | 1.09E-18 | -0.005 | 0.003 | 1.20E-01 | BMI | -0.003 | 0.020 |  | |  |  |  |
| rs12874776 | 13 | 57794345 | C | A | 0.893 | 0.001 | 0.003 | 8.91E-01 | -0.023 | 0.004 | 8.00E-11 | PC | -0.009 | 0.022 |  | |  |  |  |
| rs4055791 | 13 | 59266053 | T | C | 0.4142 | -0.017 | 0.003 | 1.77E-10 | -0.003 | 0.002 | 2.30E-01 | BMI | -0.001 | 0.014 |  | |  |  |  |
| rs1441264 | 13 | 79580919 | A | G | 0.5828 | 0.017 | 0.002 | 5.19E-14 | 0.002 | 0.002 | 3.20E-01 | BMI | 0.017 | 0.014 |  | |  |  |  |
| rs9531786 | 13 | 85983968 | C | G | 0.371 | -0.014 | 0.002 | 8.39E-09 | -0.006 | 0.002 | 6.70E-03 | BMI | 0.003 | 0.014 |  | |  |  |  |
| rs77432547 | 13 | 86494817 | A | G | 0.7236 | -0.018 | 0.003 | 2.28E-09 | 0.000 | 0.002 | 9.90E-01 | BMI | -0.019 | 0.016 |  | |  |  |  |
| rs1927790 | 13 | 96922191 | T | C | 0.6083 | -0.014 | 0.002 | 1.27E-09 | -0.004 | 0.002 | 4.70E-02 | BMI | 0.012 | 0.014 |  | |  |  |  |
| rs4586287 | 13 | 99245334 | C | G | 0.6743 | 0.014 | 0.003 | 2.95E-08 | -0.005 | 0.002 | 3.00E-02 | BMI | 0.027 | 0.015 |  | |  |  |  |
| rs7335249 | 13 | 109962579 | T | C | 0.5578 | 0.014 | 0.002 | 4.91E-09 | 0.003 | 0.002 | 1.70E-01 | BMI | -0.008 | 0.014 |  | |  |  |  |
| rs2479958 | 13 | 111984244 | A | G | 0.4857 | 0.014 | 0.002 | 1.38E-08 | -0.003 | 0.002 | 1.60E-01 | BMI | 0.034 | 0.014 |  | |  |  |  |
| rs2754079 | 14 | 25886140 | A | G | 0.5713 | -0.016 | 0.002 | 2.31E-11 | -0.002 | 0.002 | 2.90E-01 | BMI | 0.014 | 0.014 |  | |  |  |  |
| rs1569979 | 14 | 29681294 | A | G | 0.774 | 0.023 | 0.003 | 1.57E-17 | 0.000 | 0.003 | 9.10E-01 | BMI | -0.003 | 0.016 |  | |  |  |  |
| rs17522122 | 14 | 33302882 | T | G | 0.4667 | 0.017 | 0.002 | 6.46E-14 | -0.001 | 0.002 | 5.10E-01 | BMI | -0.006 | 0.014 |  | |  |  |  |
| rs198262 | 14 | 57283283 | C | T | 0.044 | 0.003 | 0.006 | 6.11E-01 | 0.030 | 0.005 | 3.90E-08 | PC | -0.003 | 0.034 |  | |  |  |  |
| rs35020117 | 14 | 73341655 | T | G | 0.5464 | 0.017 | 0.003 | 1.23E-09 | 0.000 | 0.002 | 9.90E-01 | BMI | 0.013 | 0.014 |  | |  |  |  |
| rs7141420 | 14 | 79899454 | T | C | 0.5493 | 0.021 | 0.002 | 7.84E-22 | -0.006 | 0.002 | 1.00E-02 | BMI | 0.002 | 0.014 |  | |  |  |  |
| rs1285997 | 14 | 91513029 | C | G | 0.2884 | -0.015 | 0.003 | 3.18E-09 | -0.002 | 0.002 | 3.20E-01 | BMI | 0.005 | 0.015 |  | |  |  |  |
| rs942066 | 14 | 94031914 | A | G | 0.3742 | -0.020 | 0.003 | 1.04E-13 | 0.003 | 0.002 | 2.10E-01 | BMI | 0.009 | 0.014 |  | |  |  |  |
| rs3803286 | 14 | 103246470 | A | G | 0.3412 | 0.017 | 0.002 | 8.29E-13 | -0.004 | 0.002 | 7.50E-02 | BMI | -0.009 | 0.014 |  | |  |  |  |
| rs709400 | 14 | 104149475 | A | G | 0.6212 | 0.018 | 0.002 | 4.41E-16 | -0.001 | 0.002 | 5.20E-01 | BMI | -0.002 | 0.014 |  | |  |  |  |
| rs12101625 | 15 | 26972431 | C | G | 0.6142 | 0.014 | 0.002 | 5.54E-09 | 0.004 | 0.002 | 5.60E-02 | BMI | 0.006 | 0.014 |  | |  |  |  |
| rs166835 | 15 | 47716037 | C | T | 0.443 | -0.001 | 0.002 | 8.32E-01 | 0.013 | 0.002 | 3.40E-09 | PC | 0.002 | 0.014 |  | |  |  |  |
| rs6493498 | 15 | 51754451 | T | C | 0.4455 | 0.016 | 0.002 | 1.53E-13 | 0.004 | 0.002 | 8.70E-02 | BMI | 0.024 | 0.014 |  | |  |  |  |
| rs8024806 | 15 | 53473990 | T | C | 0.9366 | 0.027 | 0.005 | 9.76E-09 | -0.006 | 0.005 | 2.00E-01 | BMI | 0.041 | 0.028 |  | |  |  |  |
| rs8033510 | 15 | 61445514 | T | C | 0.3635 | 0.014 | 0.002 | 1.13E-08 | -0.003 | 0.002 | 2.00E-01 | BMI | 0.013 | 0.014 |  | |  |  |  |
| rs4776982 | 15 | 68114974 | A | G | 0.7694 | 0.031 | 0.003 | 4.25E-33 | -0.006 | 0.003 | 3.00E-02 | BMI | 0.028 | 0.016 |  | |  |  |  |
| rs12914489 | 15 | 74187937 | A | G | 0.0809 | 0.023 | 0.004 | 1.64E-10 | 0.000 | 0.004 | 9.10E-01 | BMI | 0.023 | 0.023 |  | |  |  |  |
| rs62007782 | 15 | 78029797 | A | G | 0.2585 | -0.018 | 0.003 | 1.94E-09 | 0.001 | 0.002 | 6.20E-01 | BMI | 0.003 | 0.016 |  | |  |  |  |
| rs12593036 | 15 | 81058652 | A | G | 0.6899 | 0.015 | 0.003 | 6.65E-10 | 0.006 | 0.002 | 1.90E-02 | BMI | -0.008 | 0.015 |  | |  |  |  |
| rs4702 | 15 | 91426560 | G | A | 0.444 | 0.000 | 0.002 | 9.14E-01 | 0.012 | 0.002 | 3.80E-08 | PC | 0.014 | 0.014 |  | |  |  |  |
| rs7498044 | 15 | 92573639 | A | G | 0.2143 | -0.018 | 0.003 | 2.73E-08 | 0.002 | 0.003 | 5.70E-01 | BMI | -0.006 | 0.017 |  | |  |  |  |
| rs214249 | 16 | 348687 | T | G | 0.6068 | 0.017 | 0.002 | 1.37E-12 | -0.003 | 0.002 | 1.30E-01 | BMI | 0.018 | 0.014 |  | |  |  |  |
| rs12448257 | 16 | 3599655 | A | G | 0.2187 | 0.015 | 0.003 | 3.13E-08 | 0.005 | 0.003 | 5.70E-02 | BMI | -0.003 | 0.017 |  | |  |  |  |
| rs879620 | 16 | 4015729 | T | C | 0.5962 | 0.025 | 0.002 | 5.45E-26 | -0.003 | 0.002 | 1.70E-01 | BMI | 0.011 | 0.014 |  | |  |  |  |
| rs6498759 | 16 | 18020295 | T | C | 0.31 | -0.001 | 0.003 | 5.94E-01 | 0.013 | 0.002 | 2.70E-08 | PC | -0.009 | 0.014 |  | |  |  |  |
| rs9926784 | 16 | 19941968 | T | C | 0.7995 | 0.025 | 0.003 | 6.01E-20 | 0.000 | 0.003 | 9.40E-01 | BMI | -0.015 | 0.017 |  | |  |  |  |
| rs17767765 | 16 | 24539793 | T | C | 0.5145 | -0.013 | 0.002 | 2.60E-08 | 0.000 | 0.002 | 9.30E-01 | BMI | -0.018 | 0.014 |  | |  |  |  |
| rs7498665 | 16 | 28883241 | A | G | 0.6194 | -0.026 | 0.002 | 1.94E-32 | 0.005 | 0.002 | 1.90E-02 | BMI | -0.012 | 0.014 |  | |  |  |  |
| rs2289292 | 16 | 30097630 | T | C | 0.3315 | -0.019 | 0.003 | 1.13E-14 | 0.001 | 0.002 | 5.90E-01 | BMI | -0.002 | 0.015 |  | |  |  |  |
| rs4889606 | 16 | 31011183 | A | G | 0.6307 | 0.027 | 0.002 | 1.25E-32 | 0.001 | 0.002 | 6.70E-01 | BMI | 0.030 | 0.014 |  | |  |  |  |
| rs1558902 | 16 | 53803574 | A | T | 0.4094 | 0.070 | 0.002 | 1.10E-215 | -0.004 | 0.002 | 4.60E-02 | BMI | -0.005 | 0.014 |  | |  |  |  |
| rs3730399 | 16 | 67229019 | A | G | 0.935 | -0.009 | 0.005 | 7.35E-02 | 0.025 | 0.004 | 3.30E-08 | PC | -0.104 | 0.029 |  | |  |  |  |
| rs2012502 | 16 | 81728081 | A | C | 0.3841 | 0.015 | 0.002 | 2.97E-11 | -0.004 | 0.002 | 9.40E-02 | BMI | 0.008 | 0.014 |  | |  |  |  |
| rs4790292 | 17 | 1824305 | A | C | 0.1474 | -0.026 | 0.003 | 1.65E-15 | 0.004 | 0.003 | 1.60E-01 | BMI | 0.023 | 0.020 |  | |  |  |  |
| rs3826408 | 17 | 7101292 | T | C | 0.4179 | 0.012 | 0.002 | 4.48E-08 | -0.003 | 0.002 | 1.60E-01 | BMI | -0.005 | 0.014 |  | |  |  |  |
| rs7209653 | 17 | 19882084 | T | C | 0.704 | 0.002 | 0.003 | 4.02E-01 | 0.016 | 0.002 | 9.10E-11 | PC | 0.001 | 0.015 |  | |  |  |  |
| rs4986044 | 17 | 21261560 | T | C | 0.4533 | -0.017 | 0.002 | 2.20E-15 | -0.002 | 0.002 | 2.70E-01 | BMI | -0.030 | 0.014 |  | |  |  |  |
| rs12603813 | 17 | 43196584 | T | C | 0.746 | -0.003 | 0.003 | 2.94E-01 | 0.014 | 0.003 | 1.30E-08 | PC | 0.018 | 0.016 |  | |  |  |  |
| rs56161855 | 17 | 46288649 | A | T | 0.8674 | -0.026 | 0.004 | 1.48E-11 | 0.001 | 0.003 | 7.90E-01 | BMI | -0.039 | 0.019 |  | |  |  |  |
| rs9906944 | 17 | 47091420 | T | C | 0.355 | -0.019 | 0.002 | 7.25E-15 | 0.003 | 0.002 | 2.00E-01 | BMI | 0.016 | 0.015 |  | |  |  |  |
| rs7211966 | 17 | 54374611 | T | C | 0.0287 | -0.047 | 0.008 | 4.89E-09 | 0.003 | 0.008 | 7.10E-01 | BMI | 0.013 | 0.056 |  | |  |  |  |
| rs8075273 | 17 | 61728881 | A | C | 0.2895 | -0.016 | 0.002 | 5.56E-11 | 0.003 | 0.002 | 2.30E-01 | BMI | 0.013 | 0.015 |  | |  |  |  |
| rs2619976 | 17 | 71754545 | T | C | 0.3993 | 0.015 | 0.002 | 6.17E-10 | 0.001 | 0.002 | 6.00E-01 | BMI | -0.013 | 0.014 |  | |  |  |  |
| rs4969387 | 17 | 79081724 | C | G | 0.7383 | -0.015 | 0.003 | 6.90E-09 | -0.001 | 0.002 | 7.60E-01 | BMI | -0.005 | 0.016 |  | |  |  |  |
| rs66643547 | 17 | 79375315 | C | T | 0.647 | 0.001 | 0.003 | 6.52E-01 | -0.015 | 0.002 | 3.20E-11 | PC | 0.011 | 0.015 |  | |  |  |  |
| rs9955276 | 18 | 1839339 | T | C | 0.1441 | 0.022 | 0.003 | 8.02E-12 | -0.008 | 0.003 | 1.10E-02 | BMI | 0.025 | 0.020 |  | |  |  |  |
| rs1808579 | 18 | 21104888 | T | C | 0.4805 | -0.020 | 0.002 | 9.44E-20 | 0.007 | 0.002 | 3.10E-03 | BMI | -0.023 | 0.014 |  | |  |  |  |
| rs16975921 | 18 | 39914870 | A | T | 0.6949 | -0.016 | 0.003 | 3.87E-11 | 0.006 | 0.002 | 7.80E-03 | BMI | -0.020 | 0.014 |  | |  |  |  |
| rs1518159 | 18 | 40760146 | T | C | 0.2502 | -0.019 | 0.003 | 6.66E-13 | -0.003 | 0.003 | 2.70E-01 | BMI | -0.015 | 0.016 |  | |  |  |  |
| rs784256 | 18 | 53398626 | G | A | 0.188 | -0.018 | 0.003 | 1.77E-09 | 0.019 | 0.003 | 6.20E-11 | PC | 0.013 | 0.018 |  | |  |  |  |
| rs6567160 | 18 | 57829135 | T | C | 0.7509 | -0.057 | 0.003 | 8.74E-108 | -0.004 | 0.003 | 1.60E-01 | BMI | -0.020 | 0.016 |  | |  |  |  |
| rs17066856 | 18 | 58049656 | T | C | 0.8905 | 0.040 | 0.004 | 1.39E-27 | 0.006 | 0.004 | 1.40E-01 | BMI | -0.002 | 0.023 |  | |  |  |  |
| rs12981256 | 19 | 1865901 | A | G | 0.5271 | 0.018 | 0.002 | 3.93E-15 | -0.005 | 0.002 | 4.00E-02 | BMI | 0.031 | 0.014 |  | |  |  |  |
| rs895330 | 19 | 4060707 | C | G | 0.807 | 0.018 | 0.003 | 6.65E-10 | 0.005 | 0.003 | 5.10E-02 | BMI | -0.045 | 0.018 |  | |  |  |  |
| rs273504 | 19 | 18215247 | A | G | 0.5763 | -0.015 | 0.002 | 1.27E-10 | -0.003 | 0.002 | 2.50E-01 | BMI | -0.006 | 0.014 |  | |  |  |  |
| rs17724992 | 19 | 18454825 | A | G | 0.7186 | 0.017 | 0.003 | 6.65E-12 | -0.006 | 0.002 | 1.20E-02 | BMI | 0.029 | 0.015 |  | |  |  |  |
| rs11880870 | 19 | 18830704 | A | G | 0.5189 | 0.020 | 0.002 | 3.31E-18 | 0.001 | 0.002 | 7.20E-01 | BMI | 0.018 | 0.014 |  | |  |  |  |
| rs17751061 | 19 | 19413092 | T | C | 0.1594 | -0.018 | 0.003 | 1.29E-08 | -0.001 | 0.003 | 7.20E-01 | BMI | 0.043 | 0.019 |  | |  |  |  |
| rs12462975 | 19 | 30272202 | A | G | 0.3247 | 0.020 | 0.003 | 2.63E-16 | 0.003 | 0.002 | 2.00E-01 | BMI | 0.025 | 0.015 |  | |  |  |  |
| rs11667280 | 19 | 30705747 | C | G | 0.7971 | 0.016 | 0.003 | 3.14E-08 | 0.004 | 0.003 | 1.40E-01 | BMI | 0.017 | 0.017 |  | |  |  |  |
| rs7250833 | 19 | 33937277 | T | C | 0.3107 | 0.015 | 0.002 | 7.22E-10 | 0.009 | 0.002 | 3.00E-04 | BMI | -0.029 | 0.015 |  | |  |  |  |
| rs185350 | 19 | 34306816 | T | C | 0.4901 | 0.013 | 0.002 | 7.14E-09 | -0.001 | 0.002 | 6.00E-01 | BMI | 0.011 | 0.014 |  | |  |  |  |
| rs429358 | 19 | 45411941 | T | C | 0.8459 | 0.028 | 0.004 | 3.17E-14 | 0.011 | 0.003 | 2.60E-04 | BMI | 0.019 | 0.020 |  | |  |  |  |
| rs11672660 | 19 | 46180184 | T | C | 0.1867 | -0.032 | 0.003 | 5.93E-31 | -0.004 | 0.003 | 1.80E-01 | BMI | 0.011 | 0.017 |  | |  |  |  |
| rs9304665 | 19 | 47602577 | A | T | 0.7404 | 0.020 | 0.003 | 1.84E-14 | 0.004 | 0.003 | 1.10E-01 | BMI | 0.009 | 0.016 |  | |  |  |  |
| rs1884389 | 20 | 1410582 | T | C | 0.4397 | -0.014 | 0.002 | 1.20E-09 | -0.002 | 0.002 | 3.30E-01 | BMI | -0.007 | 0.014 |  | |  |  |  |
| rs1884897 | 20 | 6612832 | A | G | 0.3749 | -0.017 | 0.002 | 9.06E-14 | -0.001 | 0.002 | 6.40E-01 | BMI | 0.074 | 0.014 |  | |  |  |  |
| rs1409818 | 20 | 21381121 | T | C | 0.1082 | 0.025 | 0.004 | 1.58E-11 | 0.003 | 0.004 | 4.90E-01 | BMI | 0.009 | 0.022 |  | |  |  |  |
| rs6050446 | 20 | 25195509 | A | G | 0.0317 | -0.037 | 0.006 | 6.20E-09 | -0.014 | 0.006 | 2.30E-02 | BMI | -0.009 | 0.045 |  | |  |  |  |
| rs6142096 | 20 | 32686658 | A | G | 0.5198 | 0.013 | 0.002 | 4.17E-09 | 0.001 | 0.002 | 7.40E-01 | BMI | -0.004 | 0.014 |  | |  |  |  |
| rs16989232 | 20 | 39291784 | A | G | 0.3851 | 0.013 | 0.002 | 1.52E-08 | -0.001 | 0.002 | 7.30E-01 | BMI | 0.009 | 0.014 |  | |  |  |  |
| rs17806224 | 20 | 51065854 | A | G | 0.1809 | -0.029 | 0.003 | 3.52E-22 | -0.007 | 0.003 | 1.00E-02 | BMI | -0.012 | 0.018 |  | |  |  |  |
| rs6129084 | 20 | 59828857 | A | T | 0.359 | -0.005 | 0.003 | 4.80E-02 | -0.014 | 0.002 | 9.30E-10 | PC | 0.009 | 0.014 |  | |  |  |  |
| rs6010784 | 20 | 61540319 | T | C | 0.5084 | 0.013 | 0.002 | 1.08E-08 | 0.010 | 0.002 | 5.40E-06 | BMI | 0.009 | 0.014 |  | |  |  |  |
| rs2836754 | 21 | 40291740 | T | C | 0.3691 | -0.015 | 0.002 | 3.73E-11 | 0.001 | 0.002 | 8.20E-01 | BMI | 0.003 | 0.014 |  | |  |  |  |
| rs915814 | 21 | 46493003 | A | G | 0.2456 | -0.018 | 0.003 | 5.88E-09 | 0.001 | 0.003 | 5.70E-01 | BMI | 0.022 | 0.016 |  | |  |  |  |
| rs427943 | 21 | 46570896 | A | C | 0.4277 | -0.018 | 0.002 | 1.37E-14 | 0.001 | 0.002 | 5.00E-01 | BMI | 0.008 | 0.014 |  | |  |  |  |
| rs136553 | 22 | 27255675 | C | T | 0.624 | -0.010 | 0.003 | 1.86E-04 | -0.015 | 0.002 | 2.30E-11 | PC | 0.005 | 0.014 |  | |  |  |  |
| rs7288455 | 22 | 39966547 | A | G | 0.436 | 0.001 | 0.003 | 7.53E-01 | 0.013 | 0.002 | 4.00E-09 | PC | -0.004 | 0.014 |  | |  |  |  |
| rs13053342 | 22 | 48871624 | A | G | 0.3997 | 0.015 | 0.002 | 1.32E-10 | -0.001 | 0.002 | 7.60E-01 | BMI | 0.008 | 0.014 |  | |  |  |  |

| **Table S16. Beta estimates for the SNPs included in the multivariable MR of BMI, leisure television watching, and breast cancer risk** | | | | | | | | | | | | | | | | | | | | | | | |
| --- | --- | --- | --- | --- | --- | --- | --- | --- | --- | --- | --- | --- | --- | --- | --- | --- | --- | --- | --- | --- | --- | --- | --- |
| SNP | CHR | POS | EA | OA | beta_  bmi | se_  bmi | PvaL  _bmi | beta  _tv | se  _tv | pval  _tv | phenotype | beta_bρ | se_br | Luminal  _A_beta | Luminal_  A_se | Luminal_  B_beta | Lumina  l_B_se | Luminal_B_  HER2Neg  _beta | Luminal_B_  HER2Neg  _se | HER2_  Enriched  _beta | HER2_  Enriched  _se | Triple_Neg_beta | Triple_Neg_se |
| rs79113395 | 1 | 1590521 | A | G | -0.021 | 0.003 | 1.45E-12 | -0.007 | 0.002 | 4.50E-03 | BMI | -0.009 | 0.010 | -0.005 | 0.012 | -0.021 | 0.026 | -0.011 | 0.023 | 0.062 | 0.038 | -0.035 | 0.023 |
| rs10909880 | 1 | 2727804 | T | C | -0.012 | 0.002 | 1.97E-08 | 0.000 | 0.002 | 9.30E-01 | BMI | 0.008 | 0.006 | 0.011 | 0.008 | -0.006 | 0.018 | -0.010 | 0.015 | -0.019 | 0.026 | 0.008 | 0.015 |
| rs78886584 | 1 | 16859325 | A | G | -0.015 | 0.003 | 1.30E-08 | -0.007 | 0.002 | 2.40E-03 | BMI | -0.002 | 0.007 | -0.006 | 0.009 | 0.016 | 0.019 | 0.006 | 0.016 | -0.031 | 0.028 | 0.011 | 0.017 |
| rs4295917 | 1 | 32173360 | A | G | 0.014 | 0.002 | 1.62E-08 | 0.006 | 0.002 | 8.50E-03 | BMI | -0.009 | 0.007 | -0.019 | 0.009 | -0.006 | 0.019 | -0.001 | 0.017 | -0.013 | 0.028 | -0.011 | 0.017 |
| rs11810109 | 1 | 44186812 | A | T | -0.002 | 0.003 | 5.76E-01 | 0.016 | 0.002 | 5.40E-12 | TV | 0.005 | 0.007 | -0.001 | 0.009 | 0.020 | 0.019 | 0.016 | 0.016 | 0.000 | 0.028 | 0.026 | 0.017 |
| rs2984618 | 1 | 47690438 | T | G | 0.017 | 0.002 | 2.21E-14 | 0.001 | 0.002 | 6.40E-01 | BMI | -0.002 | 0.006 | -0.004 | 0.008 | -0.007 | 0.018 | -0.024 | 0.015 | 0.018 | 0.026 | -0.012 | 0.016 |
| rs657452 | 1 | 49589847 | A | G | 0.020 | 0.002 | 1.14E-19 | -0.004 | 0.002 | 7.00E-02 | BMI | -0.006 | 0.006 | -0.010 | 0.008 | 0.007 | 0.018 | -0.005 | 0.015 | -0.001 | 0.026 | -0.024 | 0.016 |
| rs12725114 | 1 | 62432641 | G | A | 0.008 | 0.003 | 1.01E-02 | 0.015 | 0.003 | 1.60E-08 | TV | 0.007 | 0.007 | 0.016 | 0.010 | 0.029 | 0.022 | 0.002 | 0.018 | 0.040 | 0.032 | -0.004 | 0.019 |
| rs984409 | 1 | 67020440 | G | A | -0.010 | 0.003 | 2.25E-04 | -0.015 | 0.002 | 4.10E-11 | TV | -0.015 | 0.006 | -0.019 | 0.008 | -0.006 | 0.019 | -0.032 | 0.016 | 0.022 | 0.027 | -0.025 | 0.016 |
| rs71658797 | 1 | 77967507 | T | A | -0.038 | 0.004 | 1.24E-20 | -0.020 | 0.003 | 6.30E-10 | TV | -0.004 | 0.010 | -0.003 | 0.013 | -0.034 | 0.029 | 0.059 | 0.025 | -0.038 | 0.042 | 0.002 | 0.025 |
| rs114328297 | 1 | 91190854 | T | G | 0.003 | 0.003 | 4.34E-01 | 0.014 | 0.003 | 4.40E-08 | TV | -0.006 | 0.008 | 0.001 | 0.010 | -0.021 | 0.023 | -0.019 | 0.020 | 0.053 | 0.034 | -0.033 | 0.020 |
| rs17379561 | 1 | 98340139 | A | T | -0.022 | 0.003 | 1.48E-11 | -0.025 | 0.003 | 1.10E-16 | TV | -0.002 | 0.009 | -0.001 | 0.011 | 0.052 | 0.025 | -0.029 | 0.021 | 0.010 | 0.036 | -0.008 | 0.021 |
| rs55909997 | 1 | 1.08E+08 | G | A | -0.013 | 0.002 | 1.72E-07 | -0.014 | 0.002 | 2.40E-09 | TV | -0.002 | 0.006 | 0.002 | 0.008 | 0.016 | 0.019 | -0.024 | 0.016 | -0.005 | 0.027 | -0.024 | 0.016 |
| rs1278847 | 1 | 1.1E+08 | C | A | -0.010 | 0.003 | 4.05E-04 | 0.016 | 0.002 | 1.20E-11 | TV | -0.012 | 0.007 | -0.016 | 0.009 | -0.008 | 0.019 | -0.004 | 0.017 | -0.017 | 0.028 | 0.011 | 0.017 |
| rs4845364 | 1 | 1.54E+08 | A | G | -0.002 | 0.002 | 5.13E-01 | -0.015 | 0.002 | 1.30E-12 | TV | 0.005 | 0.006 | 0.007 | 0.008 | 0.002 | 0.018 | -0.002 | 0.015 | 0.018 | 0.026 | 0.020 | 0.015 |
| rs61813324 | 1 | 1.56E+08 | T | C | 0.030 | 0.004 | 3.60E-14 | 0.015 | 0.003 | 4.30E-06 | BMI | -0.019 | 0.010 | -0.031 | 0.013 | -0.019 | 0.028 | -0.032 | 0.024 | -0.042 | 0.042 | -0.003 | 0.025 |
| rs12564992 | 1 | 1.74E+08 | A | G | -0.021 | 0.004 | 2.37E-09 | 0.008 | 0.003 | 2.00E-02 | BMI | -0.002 | 0.009 | -0.005 | 0.012 | 0.051 | 0.028 | -0.022 | 0.023 | 0.022 | 0.040 | -0.007 | 0.024 |
| rs6673341 | 1 | 1.85E+08 | T | G | -0.011 | 0.002 | 1.15E-06 | -0.015 | 0.002 | 2.20E-11 | TV | 0.000 | 0.006 | -0.006 | 0.008 | -0.004 | 0.018 | 0.007 | 0.015 | -0.005 | 0.026 | -0.026 | 0.015 |
| rs10737620 | 1 | 1.93E+08 | T | A | 0.013 | 0.003 | 1.16E-07 | 0.014 | 0.002 | 2.60E-09 | TV | 0.011 | 0.007 | 0.022 | 0.009 | 0.017 | 0.020 | 0.012 | 0.017 | 0.012 | 0.029 | -0.017 | 0.017 |
| rs4474229 | 1 | 1.95E+08 | A | G | -0.017 | 0.003 | 1.64E-10 | -0.003 | 0.002 | 1.20E-01 | BMI | 0.003 | 0.006 | 0.006 | 0.008 | 0.009 | 0.018 | -0.004 | 0.015 | -0.035 | 0.026 | 0.005 | 0.016 |
| rs2820295 | 1 | 2.02E+08 | A | G | 0.021 | 0.002 | 8.03E-19 | 0.010 | 0.002 | 8.70E-06 | BMI | -0.006 | 0.006 | -0.007 | 0.008 | 0.009 | 0.019 | 0.026 | 0.016 | 0.030 | 0.027 | -0.017 | 0.016 |
| rs17014375 | 1 | 2.1E+08 | T | G | -0.019 | 0.003 | 1.46E-08 | -0.001 | 0.003 | 7.20E-01 | BMI | 0.000 | 0.009 | -0.007 | 0.012 | 0.012 | 0.026 | 0.003 | 0.022 | 0.012 | 0.038 | -0.007 | 0.022 |
| rs12042959 | 1 | 2.44E+08 | A | G | 0.019 | 0.003 | 2.33E-09 | 0.004 | 0.003 | 2.20E-01 | BMI | 0.020 | 0.009 | 0.015 | 0.011 | 0.020 | 0.025 | 0.040 | 0.022 | 0.060 | 0.037 | 0.010 | 0.022 |
| rs62106258 | 2 | 417167 | T | C | 0.099 | 0.006 | 8.72E-58 | -0.007 | 0.005 | 1.90E-01 | BMI | -0.006 | 0.017 | -0.020 | 0.023 | 0.002 | 0.049 | -0.082 | 0.041 | 0.084 | 0.074 | -0.027 | 0.042 |
| rs6548237 | 2 | 621461 | A | C | -0.058 | 0.003 | 4.43E-89 | -0.005 | 0.003 | 1.00E-01 | BMI | 0.020 | 0.010 | 0.020 | 0.013 | 0.017 | 0.029 | 0.023 | 0.025 | 0.128 | 0.041 | 0.026 | 0.025 |
| rs7571496 | 2 | 6169351 | A | G | 0.015 | 0.003 | 1.45E-08 | 0.004 | 0.002 | 1.20E-01 | BMI | -0.007 | 0.007 | -0.013 | 0.009 | -0.003 | 0.020 | -0.023 | 0.017 | -0.002 | 0.030 | 0.009 | 0.018 |
| rs7601870 | 2 | 15384392 | A | G | -0.013 | 0.002 | 1.23E-08 | -0.006 | 0.002 | 9.10E-03 | BMI | 0.006 | 0.006 | 0.004 | 0.008 | -0.011 | 0.018 | 0.008 | 0.015 | 0.001 | 0.026 | 0.002 | 0.015 |
| rs72781699 | 2 | 24277709 | G | A | -0.002 | 0.003 | 4.85E-01 | -0.019 | 0.003 | 3.00E-12 | TV | 0.011 | 0.008 | 0.006 | 0.010 | 0.016 | 0.022 | -0.015 | 0.019 | -0.007 | 0.032 | 0.043 | 0.019 |
| rs10048652 | 2 | 35449306 | T | C | 0.014 | 0.002 | 3.47E-09 | 0.001 | 0.002 | 7.30E-01 | BMI | 0.002 | 0.006 | 0.009 | 0.008 | -0.044 | 0.018 | 0.004 | 0.015 | -0.011 | 0.026 | 0.009 | 0.015 |
| rs12105701 | 2 | 41737200 | C | T | -0.009 | 0.002 | 6.21E-05 | -0.013 | 0.002 | 5.40E-09 | TV | -0.003 | 0.006 | -0.004 | 0.008 | -0.020 | 0.018 | 0.043 | 0.016 | 0.026 | 0.026 | -0.027 | 0.016 |
| rs786420 | 2 | 44719893 | T | C | 0.016 | 0.003 | 8.84E-09 | -0.001 | 0.003 | 6.90E-01 | BMI | -0.014 | 0.007 | -0.010 | 0.009 | -0.004 | 0.021 | -0.027 | 0.017 | -0.036 | 0.030 | 0.019 | 0.018 |
| rs7564130 | 2 | 50606642 | T | C | -0.001 | 0.002 | 5.70E-01 | -0.015 | 0.002 | 2.80E-11 | TV | -0.002 | 0.006 | -0.010 | 0.008 | -0.016 | 0.018 | -0.005 | 0.016 | 0.005 | 0.026 | 0.006 | 0.016 |
| rs10189857 | 2 | 60713235 | A | G | -0.005 | 0.002 | 2.16E-02 | -0.020 | 0.002 | 6.20E-21 | TV | -0.017 | 0.006 | -0.019 | 0.008 | -0.011 | 0.018 | -0.022 | 0.015 | -0.008 | 0.026 | -0.015 | 0.015 |
| rs62641636 | 2 | 68425427 | A | G | 0.003 | 0.003 | 2.52E-01 | 0.014 | 0.002 | 7.00E-10 | TV | 0.001 | 0.007 | -0.003 | 0.009 | 0.039 | 0.019 | 0.009 | 0.016 | -0.042 | 0.028 | 0.016 | 0.017 |
| rs12714199 | 2 | 86812549 | T | C | -0.015 | 0.002 | 5.12E-10 | -0.002 | 0.002 | 4.80E-01 | BMI | 0.007 | 0.006 | 0.015 | 0.008 | 0.010 | 0.018 | 0.009 | 0.016 | -0.060 | 0.026 | 0.020 | 0.016 |
| rs11689199 | 2 | 1.01E+08 | A | G | 0.019 | 0.002 | 1.45E-15 | 0.018 | 0.002 | 5.50E-17 | TV | -0.010 | 0.006 | -0.008 | 0.008 | -0.016 | 0.018 | -0.007 | 0.016 | -0.014 | 0.027 | -0.017 | 0.016 |
| rs1457457 | 2 | 1.04E+08 | A | T | 0.014 | 0.002 | 3.79E-10 | 0.012 | 0.002 | 1.20E-08 | BMI | 0.015 | 0.006 | 0.012 | 0.008 | -0.015 | 0.018 | 0.008 | 0.015 | 0.002 | 0.026 | 0.017 | 0.015 |
| rs1451533 | 2 | 1.05E+08 | G | A | -0.015 | 0.003 | 3.95E-09 | -0.016 | 0.002 | 1.10E-10 | TV | -0.004 | 0.007 | -0.015 | 0.009 | 0.032 | 0.021 | -0.001 | 0.018 | 0.018 | 0.031 | -0.005 | 0.018 |
| rs12476388 | 2 | 1.16E+08 | C | T | 0.006 | 0.003 | 1.70E-02 | 0.013 | 0.002 | 2.10E-08 | TV | -0.002 | 0.007 | 0.002 | 0.009 | 0.009 | 0.019 | -0.004 | 0.017 | -0.035 | 0.028 | -0.014 | 0.017 |
| rs1984559 | 2 | 1.36E+08 | A | G | 0.016 | 0.003 | 3.27E-08 | 0.007 | 0.003 | 9.40E-03 | BMI | 0.019 | 0.007 | 0.028 | 0.010 | 0.047 | 0.021 | 0.012 | 0.018 | 0.027 | 0.030 | 0.027 | 0.018 |
| rs13002158 | 2 | 1.44E+08 | A | G | -0.021 | 0.003 | 1.15E-12 | -0.003 | 0.003 | 2.90E-01 | BMI | 0.016 | 0.008 | 0.010 | 0.011 | -0.010 | 0.023 | 0.015 | 0.020 | 0.063 | 0.035 | 0.028 | 0.021 |
| rs374722 | 2 | 1.48E+08 | G | A | 0.013 | 0.003 | 5.05E-05 | 0.024 | 0.003 | 5.50E-16 | TV | 0.011 | 0.009 | 0.018 | 0.012 | 0.036 | 0.025 | -0.012 | 0.022 | -0.080 | 0.039 | 0.001 | 0.022 |
| rs3754970 | 2 | 1.62E+08 | T | C | -0.003 | 0.003 | 2.07E-01 | -0.015 | 0.002 | 4.80E-12 | TV | 0.007 | 0.006 | 0.000 | 0.008 | -0.003 | 0.018 | 0.006 | 0.015 | -0.017 | 0.026 | 0.023 | 0.016 |
| rs2060199 | 2 | 1.66E+08 | T | A | 0.003 | 0.002 | 1.88E-01 | 0.012 | 0.002 | 2.40E-08 | TV | -0.007 | 0.006 | -0.007 | 0.008 | 0.010 | 0.018 | 0.019 | 0.016 | 0.011 | 0.026 | -0.021 | 0.016 |
| rs7588437 | 2 | 1.82E+08 | A | G | -0.018 | 0.002 | 8.38E-15 | -0.006 | 0.002 | 1.40E-02 | BMI | 0.015 | 0.006 | 0.009 | 0.008 | 0.027 | 0.018 | 0.025 | 0.016 | 0.019 | 0.027 | 0.036 | 0.016 |
| rs4577309 | 2 | 1.91E+08 | A | G | 0.002 | 0.003 | 3.61E-01 | 0.016 | 0.002 | 1.60E-13 | TV | -0.001 | 0.006 | 0.004 | 0.008 | -0.014 | 0.018 | 0.008 | 0.015 | -0.006 | 0.026 | -0.015 | 0.015 |
| rs34224261 | 2 | 1.94E+08 | G | C | -0.010 | 0.003 | 1.97E-04 | -0.018 | 0.002 | 4.60E-16 | TV | 0.000 | 0.006 | -0.006 | 0.008 | 0.004 | 0.017 | -0.026 | 0.015 | -0.060 | 0.026 | -0.001 | 0.015 |
| rs4675246 | 2 | 2.03E+08 | G | T | 0.002 | 0.003 | 4.91E-01 | -0.015 | 0.003 | 2.90E-08 | TV | 0.006 | 0.008 | 0.013 | 0.010 | -0.005 | 0.022 | 0.038 | 0.019 | -0.077 | 0.032 | -0.040 | 0.019 |
| rs13029509 | 2 | 2.15E+08 | G | A | -0.007 | 0.002 | 2.60E-03 | -0.018 | 0.002 | 2.00E-17 | TV | -0.009 | 0.006 | -0.008 | 0.008 | 0.008 | 0.018 | -0.019 | 0.015 | 0.017 | 0.026 | -0.017 | 0.015 |
| rs10932837 | 2 | 2.21E+08 | C | T | 0.001 | 0.002 | 7.82E-01 | -0.013 | 0.002 | 1.30E-09 | TV | 0.001 | 0.006 | 0.003 | 0.008 | -0.011 | 0.018 | 0.009 | 0.015 | -0.023 | 0.026 | 0.000 | 0.015 |
| rs6720868 | 2 | 2.31E+08 | T | C | 0.017 | 0.002 | 4.65E-12 | 0.006 | 0.002 | 1.10E-02 | BMI | -0.012 | 0.006 | -0.002 | 0.008 | -0.019 | 0.019 | -0.015 | 0.016 | -0.012 | 0.027 | -0.009 | 0.016 |
| rs4973576 | 2 | 2.34E+08 | C | A | 0.000 | 0.003 | 8.92E-01 | -0.015 | 0.002 | 1.00E-09 | TV | 0.012 | 0.007 | 0.022 | 0.009 | 0.031 | 0.020 | -0.015 | 0.017 | 0.018 | 0.029 | 0.026 | 0.017 |
| rs138256022 | 3 | 11603090 | C | T | 0.000 | 0.007 | 9.75E-01 | -0.031 | 0.006 | 2.90E-08 | TV | -0.016 | 0.017 | -0.025 | 0.023 | -0.024 | 0.050 | -0.043 | 0.043 | -0.061 | 0.072 | -0.005 | 0.044 |
| rs13062093 | 3 | 35667057 | T | G | -0.014 | 0.002 | 8.00E-09 | 0.001 | 0.002 | 6.20E-01 | BMI | -0.003 | 0.006 | -0.012 | 0.008 | -0.015 | 0.018 | -0.005 | 0.016 | -0.021 | 0.026 | -0.001 | 0.016 |
| rs9834970 | 3 | 36856030 | T | C | 0.006 | 0.002 | 1.14E-02 | 0.013 | 0.002 | 3.30E-09 | TV | 0.001 | 0.006 | 0.001 | 0.008 | 0.005 | 0.018 | 0.004 | 0.016 | -0.035 | 0.027 | 0.011 | 0.016 |
| rs3796386 | 3 | 49899795 | G | A | -0.017 | 0.002 | 9.78E-14 | -0.026 | 0.002 | 3.20E-33 | TV | -0.018 | 0.006 | -0.014 | 0.008 | -0.038 | 0.018 | -0.012 | 0.015 | -0.063 | 0.026 | -0.043 | 0.015 |
| rs11130793 | 3 | 60884659 | C | T | 0.011 | 0.003 | 6.46E-05 | 0.013 | 0.002 | 5.00E-09 | TV | 0.001 | 0.006 | 0.006 | 0.008 | 0.012 | 0.018 | -0.021 | 0.016 | 0.004 | 0.027 | 0.002 | 0.016 |
| rs1452075 | 3 | 62481063 | T | C | 0.016 | 0.003 | 1.47E-10 | 0.002 | 0.002 | 3.70E-01 | BMI | 0.012 | 0.007 | 0.019 | 0.009 | -0.008 | 0.020 | 0.017 | 0.018 | 0.067 | 0.030 | -0.024 | 0.018 |
| rs11714337 | 3 | 71582521 | G | A | 0.009 | 0.003 | 1.28E-03 | 0.014 | 0.002 | 4.70E-11 | TV | 0.032 | 0.006 | 0.045 | 0.008 | 0.036 | 0.018 | 0.029 | 0.015 | 0.000 | 0.026 | 0.014 | 0.015 |
| rs7647242 | 3 | 82642734 | T | C | 0.015 | 0.002 | 3.92E-10 | 0.002 | 0.002 | 4.50E-01 | BMI | 0.003 | 0.006 | 0.005 | 0.008 | -0.008 | 0.018 | 0.015 | 0.016 | 0.008 | 0.027 | 0.008 | 0.016 |
| rs6797840 | 3 | 85656569 | A | C | -0.012 | 0.003 | 8.70E-06 | -0.016 | 0.002 | 1.70E-13 | TV | -0.004 | 0.006 | -0.007 | 0.008 | -0.015 | 0.018 | -0.022 | 0.015 | 0.024 | 0.026 | 0.005 | 0.015 |
| rs2034768 | 3 | 93986371 | A | G | 0.019 | 0.003 | 9.80E-14 | 0.015 | 0.002 | 8.60E-12 | TV | -0.003 | 0.006 | -0.006 | 0.008 | 0.010 | 0.018 | 0.016 | 0.015 | 0.016 | 0.025 | -0.025 | 0.015 |
| rs1436348 | 3 | 1.05E+08 | A | G | -0.013 | 0.002 | 6.31E-09 | -0.004 | 0.002 | 9.70E-02 | BMI | 0.000 | 0.006 | -0.001 | 0.008 | 0.003 | 0.018 | -0.002 | 0.015 | -0.026 | 0.026 | 0.014 | 0.016 |
| rs1289736 | 3 | 1.08E+08 | T | C | 0.014 | 0.002 | 1.43E-09 | 0.007 | 0.002 | 9.50E-04 | BMI | -0.005 | 0.006 | -0.001 | 0.008 | 0.029 | 0.018 | 0.007 | 0.015 | -0.042 | 0.026 | -0.015 | 0.015 |
| rs9867121 | 3 | 1.15E+08 | C | A | 0.009 | 0.003 | 5.94E-03 | 0.019 | 0.003 | 3.90E-12 | TV | 0.013 | 0.008 | 0.015 | 0.010 | 0.020 | 0.023 | 0.001 | 0.020 | 0.052 | 0.034 | 0.009 | 0.020 |
| rs7428670 | 3 | 1.32E+08 | C | G | 0.020 | 0.003 | 3.09E-15 | 0.003 | 0.002 | 2.20E-01 | BMI | -0.004 | 0.007 | -0.012 | 0.009 | -0.010 | 0.020 | -0.005 | 0.017 | -0.017 | 0.029 | 0.017 | 0.017 |
| rs7621025 | 3 | 1.36E+08 | T | C | -0.020 | 0.003 | 1.62E-15 | -0.004 | 0.002 | 1.30E-01 | BMI | 0.013 | 0.007 | -0.001 | 0.009 | 0.015 | 0.020 | -0.012 | 0.017 | 0.042 | 0.029 | 0.032 | 0.017 |
| rs16851483 | 3 | 1.41E+08 | T | G | 0.036 | 0.005 | 2.28E-15 | 0.013 | 0.004 | 2.90E-03 | BMI | 0.006 | 0.012 | 0.011 | 0.016 | 0.039 | 0.035 | -0.024 | 0.031 | -0.085 | 0.054 | 0.053 | 0.030 |
| rs355754 | 3 | 1.54E+08 | T | C | -0.014 | 0.002 | 4.26E-10 | -0.003 | 0.002 | 1.60E-01 | BMI | 0.000 | 0.006 | 0.006 | 0.008 | -0.031 | 0.018 | 0.005 | 0.016 | -0.031 | 0.026 | 0.006 | 0.016 |
| rs12491503 | 3 | 1.66E+08 | G | A | -0.005 | 0.002 | 3.29E-02 | -0.014 | 0.002 | 5.50E-10 | TV | -0.001 | 0.007 | 0.008 | 0.009 | -0.018 | 0.019 | -0.029 | 0.016 | -0.025 | 0.028 | 0.009 | 0.017 |
| rs247975 | 3 | 1.73E+08 | T | C | -0.018 | 0.002 | 3.62E-15 | -0.005 | 0.002 | 1.80E-02 | BMI | 0.002 | 0.007 | -0.003 | 0.009 | 0.012 | 0.019 | 0.012 | 0.016 | 0.027 | 0.028 | 0.000 | 0.017 |
| rs114600294 | 3 | 1.81E+08 | G | C | -0.007 | 0.003 | 3.65E-02 | -0.016 | 0.003 | 7.90E-10 | TV | 0.017 | 0.010 | 0.002 | 0.013 | 0.029 | 0.028 | -0.015 | 0.024 | 0.035 | 0.042 | 0.007 | 0.025 |
| rs10513801 | 3 | 1.86E+08 | T | G | 0.035 | 0.003 | 1.26E-27 | -0.009 | 0.003 | 3.70E-03 | BMI | -0.009 | 0.009 | -0.017 | 0.012 | -0.001 | 0.027 | 0.018 | 0.023 | 0.007 | 0.039 | -0.020 | 0.023 |
| rs4677812 | 3 | 1.95E+08 | A | C | -0.014 | 0.003 | 9.38E-09 | -0.009 | 0.002 | 1.10E-04 | BMI | -0.002 | 0.007 | -0.002 | 0.009 | 0.002 | 0.020 | -0.014 | 0.017 | 0.023 | 0.029 | -0.013 | 0.017 |
| rs66852340 | 4 | 3311070 | C | T | -0.009 | 0.003 | 1.10E-03 | -0.018 | 0.003 | 7.90E-12 | TV | -0.016 | 0.007 | -0.019 | 0.009 | 0.027 | 0.021 | -0.046 | 0.018 | -0.011 | 0.031 | -0.022 | 0.018 |
| rs7700107 | 4 | 17880416 | A | C | -0.016 | 0.003 | 3.40E-06 | -0.021 | 0.003 | 1.90E-11 | TV | 0.005 | 0.009 | 0.033 | 0.012 | -0.019 | 0.025 | -0.026 | 0.022 | -0.066 | 0.036 | -0.009 | 0.022 |
| rs994596 | 4 | 18459828 | T | C | 0.015 | 0.003 | 1.02E-09 | 0.001 | 0.002 | 5.30E-01 | BMI | 0.002 | 0.006 | -0.001 | 0.009 | -0.004 | 0.019 | 0.035 | 0.016 | -0.005 | 0.027 | -0.017 | 0.016 |
| rs73213484 | 4 | 28489339 | A | T | 0.025 | 0.004 | 2.28E-11 | 0.002 | 0.003 | 4.40E-01 | BMI | 0.003 | 0.008 | 0.002 | 0.011 | 0.024 | 0.025 | 0.019 | 0.021 | 0.053 | 0.036 | -0.026 | 0.021 |
| rs337637 | 4 | 38604470 | A | G | -0.013 | 0.002 | 3.04E-08 | -0.002 | 0.002 | 3.40E-01 | BMI | -0.001 | 0.006 | -0.009 | 0.008 | 0.023 | 0.018 | -0.006 | 0.016 | 0.034 | 0.027 | 0.030 | 0.016 |
| rs10938397 | 4 | 45182527 | A | G | -0.032 | 0.002 | 1.66E-45 | -0.011 | 0.002 | 9.30E-07 | BMI | 0.015 | 0.006 | 0.016 | 0.008 | -0.012 | 0.018 | 0.007 | 0.015 | 0.003 | 0.026 | 0.025 | 0.015 |
| rs28971796 | 4 | 49151982 | A | G | 0.012 | 0.003 | 3.93E-05 | 0.015 | 0.002 | 2.10E-09 | TV | 0.010 | 0.009 | 0.017 | 0.012 | 0.042 | 0.026 | 0.005 | 0.022 | 0.049 | 0.037 | 0.017 | 0.022 |
| rs11945861 | 4 | 65700865 | A | G | -0.015 | 0.003 | 3.13E-08 | 0.002 | 0.003 | 3.40E-01 | BMI | -0.008 | 0.007 | -0.012 | 0.009 | -0.010 | 0.021 | -0.023 | 0.018 | 0.038 | 0.030 | -0.006 | 0.018 |
| rs10002111 | 4 | 67815504 | A | G | 0.017 | 0.003 | 9.26E-10 | 0.007 | 0.003 | 4.30E-03 | BMI | -0.016 | 0.007 | -0.013 | 0.009 | 0.010 | 0.021 | -0.006 | 0.018 | 0.010 | 0.031 | -0.028 | 0.018 |
| rs6850494 | 4 | 82291771 | A | C | 0.003 | 0.003 | 2.38E-01 | -0.014 | 0.002 | 1.10E-10 | TV | 0.009 | 0.006 | 0.004 | 0.008 | 0.030 | 0.018 | 0.002 | 0.015 | -0.045 | 0.026 | 0.020 | 0.016 |
| rs4148155 | 4 | 89054667 | A | G | 0.020 | 0.004 | 6.20E-09 | 0.009 | 0.003 | 6.60E-03 | BMI | 0.043 | 0.010 | 0.019 | 0.013 | 0.023 | 0.029 | 0.060 | 0.025 | 0.011 | 0.042 | 0.078 | 0.026 |
| rs12645001 | 4 | 96018337 | A | G | -0.014 | 0.002 | 4.11E-09 | 0.001 | 0.002 | 7.30E-01 | BMI | -0.009 | 0.006 | -0.005 | 0.008 | -0.034 | 0.019 | 0.005 | 0.016 | -0.035 | 0.027 | 0.003 | 0.016 |
| rs13107325 | 4 | 1.03E+08 | T | C | 0.045 | 0.004 | 9.33E-25 | 0.029 | 0.004 | 1.50E-12 | both | 0.019 | 0.012 | 0.021 | 0.016 | 0.051 | 0.034 | 0.007 | 0.030 | -0.051 | 0.051 | 0.025 | 0.030 |
| rs4834272 | 4 | 1.13E+08 | T | C | -0.014 | 0.002 | 8.24E-10 | 0.002 | 0.002 | 4.40E-01 | BMI | -0.002 | 0.007 | -0.002 | 0.009 | -0.004 | 0.019 | -0.006 | 0.017 | -0.029 | 0.028 | -0.009 | 0.017 |
| rs7693703 | 4 | 1.18E+08 | G | A | 0.004 | 0.004 | 3.03E-01 | 0.023 | 0.004 | 2.70E-09 | TV | -0.007 | 0.010 | -0.015 | 0.014 | -0.012 | 0.030 | 0.009 | 0.026 | 0.052 | 0.046 | 0.005 | 0.026 |
| rs1403846 | 4 | 1.19E+08 | T | C | 0.016 | 0.003 | 1.62E-08 | 0.008 | 0.003 | 3.50E-03 | BMI | 0.007 | 0.008 | 0.010 | 0.010 | -0.023 | 0.023 | 0.038 | 0.020 | 0.032 | 0.034 | 0.006 | 0.020 |
| rs4334769 | 4 | 1.3E+08 | G | T | 0.004 | 0.002 | 1.22E-01 | 0.012 | 0.002 | 2.10E-08 | TV | -0.008 | 0.006 | -0.007 | 0.008 | -0.016 | 0.018 | -0.032 | 0.015 | 0.012 | 0.026 | 0.015 | 0.015 |
| rs57800857 | 4 | 1.41E+08 | A | C | 0.019 | 0.003 | 1.07E-11 | 0.004 | 0.002 | 5.20E-02 | BMI | 0.011 | 0.006 | 0.007 | 0.008 | 0.019 | 0.019 | 0.011 | 0.016 | 0.007 | 0.027 | 0.050 | 0.016 |
| rs35107973 | 4 | 1.43E+08 | T | C | 0.023 | 0.004 | 7.66E-09 | 0.002 | 0.003 | 5.50E-01 | BMI | 0.012 | 0.010 | 0.010 | 0.013 | 0.013 | 0.029 | 0.007 | 0.025 | 0.028 | 0.042 | -0.003 | 0.025 |
| rs6825241 | 4 | 1.53E+08 | C | A | -0.003 | 0.002 | 2.30E-01 | -0.017 | 0.002 | 4.90E-15 | TV | 0.002 | 0.006 | 0.009 | 0.008 | -0.008 | 0.018 | -0.018 | 0.015 | 0.008 | 0.026 | 0.006 | 0.016 |
| rs7693082 | 4 | 1.6E+08 | G | C | 0.012 | 0.003 | 3.71E-05 | 0.015 | 0.002 | 1.70E-10 | TV | -0.004 | 0.007 | -0.013 | 0.009 | -0.023 | 0.020 | -0.001 | 0.017 | 0.044 | 0.028 | -0.013 | 0.017 |
| rs13110266 | 4 | 1.62E+08 | A | G | -0.014 | 0.002 | 1.55E-10 | -0.006 | 0.002 | 7.90E-03 | BMI | -0.002 | 0.006 | 0.005 | 0.008 | 0.000 | 0.018 | 0.012 | 0.015 | -0.013 | 0.026 | -0.010 | 0.016 |
| rs1491333 | 4 | 1.72E+08 | T | G | -0.015 | 0.003 | 9.85E-09 | -0.002 | 0.003 | 4.90E-01 | BMI | -0.001 | 0.007 | 0.001 | 0.009 | -0.015 | 0.021 | 0.009 | 0.018 | -0.013 | 0.030 | -0.001 | 0.018 |
| rs6890310 | 5 | 27193573 | A | G | -0.015 | 0.003 | 1.38E-09 | -0.003 | 0.002 | 1.90E-01 | BMI | 0.008 | 0.007 | 0.013 | 0.009 | 0.022 | 0.020 | -0.018 | 0.017 | -0.010 | 0.030 | 0.010 | 0.018 |
| rs782971 | 5 | 43124688 | A | G | -0.017 | 0.003 | 4.89E-12 | -0.001 | 0.002 | 5.90E-01 | BMI | -0.014 | 0.007 | -0.013 | 0.009 | 0.013 | 0.021 | -0.016 | 0.018 | 0.004 | 0.031 | -0.009 | 0.018 |
| rs10940659 | 5 | 59360341 | A | G | -0.007 | 0.002 | 4.25E-03 | -0.013 | 0.002 | 1.50E-08 | TV | 0.008 | 0.006 | 0.020 | 0.008 | -0.061 | 0.018 | 0.014 | 0.015 | -0.028 | 0.026 | 0.003 | 0.015 |
| rs262890 | 5 | 62930015 | A | G | -0.013 | 0.003 | 7.07E-06 | -0.019 | 0.002 | 3.20E-15 | TV | -0.003 | 0.007 | -0.002 | 0.009 | 0.032 | 0.019 | -0.006 | 0.016 | -0.021 | 0.028 | -0.008 | 0.017 |
| rs2112347 | 5 | 75015242 | T | G | 0.031 | 0.002 | 2.13E-42 | 0.007 | 0.002 | 2.10E-03 | BMI | -0.020 | 0.006 | -0.017 | 0.008 | 0.019 | 0.018 | -0.023 | 0.015 | -0.065 | 0.026 | -0.030 | 0.016 |
| rs7716447 | 5 | 88800331 | A | G | -0.015 | 0.002 | 1.46E-10 | -0.013 | 0.002 | 3.60E-09 | TV | 0.007 | 0.007 | 0.009 | 0.009 | -0.013 | 0.019 | 0.011 | 0.016 | 0.034 | 0.028 | -0.011 | 0.017 |
| rs1031423 | 5 | 93276883 | T | C | 0.007 | 0.003 | 1.55E-02 | -0.019 | 0.003 | 1.80E-12 | TV | -0.002 | 0.008 | -0.007 | 0.010 | 0.013 | 0.022 | 0.013 | 0.019 | -0.010 | 0.032 | 0.021 | 0.019 |
| rs2161097 | 5 | 1.04E+08 | T | C | 0.013 | 0.002 | 2.31E-08 | 0.004 | 0.002 | 8.40E-02 | BMI | 0.006 | 0.006 | 0.012 | 0.008 | -0.001 | 0.018 | 0.023 | 0.015 | 0.026 | 0.026 | -0.006 | 0.015 |
| rs57585211 | 5 | 1.07E+08 | T | G | -0.003 | 0.003 | 3.66E-01 | -0.017 | 0.003 | 4.30E-09 | TV | 0.001 | 0.008 | 0.000 | 0.011 | 0.021 | 0.024 | -0.036 | 0.020 | 0.044 | 0.035 | -0.026 | 0.020 |
| rs158186 | 5 | 1.07E+08 | A | G | -0.029 | 0.003 | 3.23E-20 | -0.002 | 0.003 | 5.30E-01 | BMI | -0.005 | 0.008 | -0.017 | 0.011 | 0.047 | 0.023 | -0.024 | 0.020 | 0.052 | 0.033 | -0.019 | 0.020 |
| rs9326862 | 5 | 1.12E+08 | A | T | 0.005 | 0.002 | 4.43E-02 | 0.012 | 0.002 | 2.60E-08 | TV | 0.005 | 0.006 | 0.008 | 0.008 | 0.008 | 0.018 | -0.002 | 0.015 | 0.025 | 0.026 | 0.005 | 0.015 |
| rs4895231 | 5 | 1.19E+08 | C | G | 0.014 | 0.002 | 9.26E-10 | 0.008 | 0.002 | 4.60E-04 | BMI | 0.007 | 0.006 | 0.010 | 0.008 | 0.026 | 0.018 | 0.007 | 0.016 | -0.004 | 0.027 | 0.020 | 0.016 |
| rs10041724 | 5 | 1.24E+08 | T | C | 0.005 | 0.003 | 1.17E-01 | 0.018 | 0.003 | 3.90E-11 | TV | 0.008 | 0.008 | 0.006 | 0.010 | 0.020 | 0.022 | -0.011 | 0.019 | 0.065 | 0.033 | -0.021 | 0.019 |
| rs62379379 | 5 | 1.41E+08 | G | T | -0.011 | 0.005 | 3.92E-02 | -0.026 | 0.004 | 7.80E-10 | TV | -0.001 | 0.012 | -0.014 | 0.016 | 0.074 | 0.037 | 0.019 | 0.031 | 0.122 | 0.055 | -0.031 | 0.030 |
| rs10054327 | 5 | 1.48E+08 | G | A | 0.004 | 0.003 | 1.83E-01 | 0.017 | 0.002 | 3.40E-15 | TV | -0.010 | 0.006 | -0.013 | 0.008 | 0.000 | 0.018 | 0.004 | 0.015 | 0.003 | 0.026 | -0.022 | 0.016 |
| rs42210 | 5 | 1.66E+08 | G | C | -0.004 | 0.003 | 1.81E-01 | -0.014 | 0.002 | 7.30E-09 | TV | -0.020 | 0.007 | -0.008 | 0.010 | -0.046 | 0.021 | -0.046 | 0.018 | -0.083 | 0.031 | -0.019 | 0.018 |
| rs72828890 | 5 | 1.68E+08 | C | T | 0.016 | 0.004 | 1.25E-04 | 0.019 | 0.003 | 4.90E-09 | TV | -0.005 | 0.011 | 0.006 | 0.014 | -0.010 | 0.032 | 0.027 | 0.028 | 0.002 | 0.047 | 0.012 | 0.028 |
| rs2228213 | 6 | 12124855 | A | G | -0.015 | 0.002 | 9.21E-11 | -0.005 | 0.002 | 3.30E-02 | BMI | 0.001 | 0.006 | 0.006 | 0.008 | -0.004 | 0.018 | -0.013 | 0.016 | -0.037 | 0.027 | 0.025 | 0.016 |
| rs9367369 | 6 | 13189941 | A | G | 0.017 | 0.003 | 2.32E-09 | 0.003 | 0.002 | 2.40E-01 | BMI | 0.001 | 0.007 | 0.002 | 0.009 | -0.026 | 0.019 | -0.008 | 0.016 | -0.009 | 0.028 | 0.041 | 0.017 |
| rs3806114 | 6 | 20482335 | A | G | -0.014 | 0.002 | 4.49E-09 | -0.009 | 0.002 | 1.30E-04 | BMI | -0.019 | 0.007 | -0.004 | 0.009 | -0.036 | 0.019 | -0.024 | 0.016 | -0.047 | 0.028 | -0.015 | 0.017 |
| rs72834698 | 6 | 26176517 | G | A | 0.013 | 0.004 | 9.74E-04 | 0.023 | 0.003 | 2.70E-13 | TV | 0.007 | 0.009 | 0.019 | 0.012 | -0.001 | 0.026 | -0.022 | 0.022 | 0.030 | 0.038 | 0.019 | 0.023 |
| rs9471333 | 6 | 40362023 | C | T | 0.021 | 0.003 | 4.04E-15 | 0.013 | 0.002 | 1.50E-09 | TV | 0.000 | 0.006 | 0.012 | 0.009 | -0.008 | 0.019 | -0.039 | 0.016 | -0.044 | 0.027 | 0.011 | 0.016 |
| rs943005 | 6 | 50865820 | T | C | 0.040 | 0.003 | 1.99E-43 | 0.003 | 0.003 | 3.10E-01 | BMI | 0.011 | 0.008 | 0.020 | 0.010 | 0.024 | 0.023 | 0.005 | 0.020 | -0.036 | 0.034 | -0.015 | 0.020 |
| rs2465043 | 6 | 51180765 | A | G | -0.014 | 0.002 | 3.22E-09 | -0.002 | 0.002 | 2.90E-01 | BMI | 0.007 | 0.006 | -0.007 | 0.008 | 0.045 | 0.018 | 0.025 | 0.016 | 0.011 | 0.027 | 0.007 | 0.016 |
| rs62412826 | 6 | 61881167 | T | C | 0.033 | 0.006 | 7.98E-09 | -0.003 | 0.005 | 5.10E-01 | BMI | 0.027 | 0.014 | 0.028 | 0.018 | 0.099 | 0.038 | 0.013 | 0.034 | -0.035 | 0.058 | 0.001 | 0.034 |
| rs4523073 | 6 | 67550288 | A | G | -0.004 | 0.003 | 1.07E-01 | -0.014 | 0.002 | 5.00E-10 | TV | -0.002 | 0.006 | -0.004 | 0.008 | 0.025 | 0.018 | -0.006 | 0.016 | -0.003 | 0.027 | 0.008 | 0.016 |
| rs209416 | 6 | 83534729 | A | T | 0.017 | 0.002 | 8.71E-12 | -0.003 | 0.002 | 2.50E-01 | BMI | -0.013 | 0.006 | 0.001 | 0.009 | -0.067 | 0.019 | 0.004 | 0.016 | -0.010 | 0.028 | -0.006 | 0.016 |
| rs6905544 | 6 | 98411631 | A | G | -0.015 | 0.002 | 1.28E-10 | -0.019 | 0.002 | 8.50E-18 | TV | -0.011 | 0.006 | -0.016 | 0.008 | -0.007 | 0.018 | -0.012 | 0.015 | 0.005 | 0.026 | -0.012 | 0.016 |
| rs17789218 | 6 | 1.01E+08 | T | C | -0.007 | 0.003 | 1.07E-02 | 0.019 | 0.003 | 1.40E-13 | TV | -0.013 | 0.008 | -0.025 | 0.011 | -0.002 | 0.023 | 0.030 | 0.020 | 0.068 | 0.034 | 0.014 | 0.020 |
| rs768023 | 6 | 1.09E+08 | A | G | 0.019 | 0.002 | 1.38E-16 | 0.005 | 0.002 | 2.00E-02 | BMI | 0.011 | 0.006 | 0.018 | 0.008 | 0.005 | 0.018 | 0.004 | 0.015 | 0.029 | 0.026 | -0.004 | 0.016 |
| rs2357760 | 6 | 1.2E+08 | A | G | 0.016 | 0.002 | 1.09E-11 | 0.006 | 0.002 | 1.60E-02 | BMI | -0.007 | 0.006 | 0.003 | 0.008 | -0.005 | 0.019 | 0.016 | 0.016 | -0.029 | 0.027 | -0.016 | 0.016 |
| rs2184364 | 6 | 1.43E+08 | A | G | 0.011 | 0.003 | 2.43E-04 | 0.016 | 0.003 | 3.00E-09 | TV | 0.007 | 0.007 | 0.009 | 0.010 | 0.009 | 0.022 | -0.004 | 0.018 | -0.035 | 0.031 | 0.023 | 0.019 |
| rs2185027 | 6 | 1.53E+08 | A | C | -0.015 | 0.002 | 1.11E-10 | -0.009 | 0.002 | 2.10E-04 | BMI | 0.002 | 0.007 | 0.007 | 0.009 | -0.004 | 0.019 | -0.023 | 0.016 | -0.030 | 0.028 | 0.020 | 0.017 |
| rs12207125 | 6 | 1.54E+08 | A | G | 0.018 | 0.003 | 7.67E-09 | -0.003 | 0.003 | 2.00E-01 | BMI | -0.010 | 0.007 | -0.010 | 0.010 | -0.005 | 0.022 | -0.040 | 0.018 | 0.045 | 0.032 | -0.024 | 0.019 |
| rs9718104 | 6 | 1.66E+08 | T | G | 0.002 | 0.006 | 7.51E-01 | -0.041 | 0.005 | 9.30E-19 | TV | -0.010 | 0.014 | -0.009 | 0.018 | 0.009 | 0.041 | 0.004 | 0.034 | 0.041 | 0.060 | -0.054 | 0.034 |
| rs836525 | 7 | 6465909 | T | C | 0.018 | 0.003 | 9.75E-09 | 0.003 | 0.003 | 3.60E-01 | BMI | -0.014 | 0.008 | -0.018 | 0.011 | -0.021 | 0.024 | -0.027 | 0.021 | -0.066 | 0.036 | 0.009 | 0.021 |
| rs17568389 | 7 | 8091876 | T | A | 0.002 | 0.002 | 3.46E-01 | 0.015 | 0.002 | 8.60E-13 | TV | -0.003 | 0.006 | -0.008 | 0.008 | -0.012 | 0.018 | -0.014 | 0.015 | 0.038 | 0.026 | 0.010 | 0.016 |
| rs4307239 | 7 | 24354300 | A | G | -0.014 | 0.002 | 1.31E-09 | -0.001 | 0.002 | 8.00E-01 | BMI | 0.012 | 0.006 | 0.001 | 0.008 | 0.026 | 0.018 | 0.025 | 0.016 | 0.026 | 0.026 | 0.027 | 0.016 |
| rs11971098 | 7 | 26699061 | A | G | -0.024 | 0.004 | 6.49E-09 | 0.005 | 0.004 | 2.40E-01 | BMI | -0.015 | 0.011 | -0.016 | 0.014 | -0.026 | 0.031 | -0.032 | 0.027 | 0.042 | 0.046 | 0.041 | 0.028 |
| rs10234444 | 7 | 41828527 | G | A | 0.006 | 0.003 | 5.90E-02 | 0.016 | 0.003 | 2.90E-08 | TV | 0.011 | 0.008 | 0.001 | 0.011 | 0.031 | 0.025 | 0.005 | 0.021 | -0.069 | 0.035 | 0.021 | 0.022 |
| rs10237317 | 7 | 70045941 | A | G | -0.014 | 0.002 | 6.82E-10 | 0.000 | 0.002 | 9.20E-01 | BMI | 0.001 | 0.006 | -0.003 | 0.008 | 0.006 | 0.018 | -0.015 | 0.016 | 0.027 | 0.027 | 0.007 | 0.016 |
| rs1167800 | 7 | 75176196 | A | G | 0.020 | 0.002 | 3.66E-19 | 0.008 | 0.002 | 1.10E-04 | BMI | 0.012 | 0.006 | 0.009 | 0.008 | 0.019 | 0.018 | 0.016 | 0.015 | 0.040 | 0.026 | 0.001 | 0.015 |
| rs6973656 | 7 | 77422583 | A | G | -0.011 | 0.002 | 1.55E-06 | -0.013 | 0.002 | 1.00E-09 | TV | 0.000 | 0.006 | 0.006 | 0.009 | -0.013 | 0.019 | -0.019 | 0.016 | -0.013 | 0.027 | 0.003 | 0.016 |
| rs7805441 | 7 | 78121458 | T | C | 0.015 | 0.002 | 2.01E-10 | 0.007 | 0.002 | 1.60E-03 | BMI | -0.003 | 0.006 | 0.002 | 0.008 | -0.010 | 0.018 | -0.002 | 0.015 | -0.041 | 0.026 | 0.001 | 0.015 |
| rs973734 | 7 | 89387578 | C | A | 0.001 | 0.003 | 7.34E-01 | 0.017 | 0.003 | 1.50E-08 | TV | 0.013 | 0.009 | 0.015 | 0.011 | 0.032 | 0.025 | 0.015 | 0.021 | 0.017 | 0.036 | 0.023 | 0.022 |
| rs2528531 | 7 | 93236510 | A | C | -0.015 | 0.002 | 1.35E-09 | 0.004 | 0.002 | 1.00E-01 | BMI | 0.007 | 0.006 | -0.001 | 0.008 | -0.004 | 0.018 | 0.020 | 0.016 | 0.002 | 0.027 | 0.022 | 0.016 |
| rs10271582 | 7 | 99121950 | A | G | 0.019 | 0.003 | 8.41E-09 | 0.017 | 0.003 | 1.90E-08 | BMI | 0.010 | 0.008 | 0.002 | 0.011 | 0.018 | 0.024 | 0.004 | 0.021 | 0.036 | 0.035 | 0.019 | 0.021 |
| rs2299383 | 7 | 1.03E+08 | T | C | 0.018 | 0.002 | 2.73E-16 | -0.002 | 0.002 | 2.90E-01 | BMI | -0.013 | 0.006 | -0.005 | 0.008 | -0.034 | 0.018 | -0.017 | 0.015 | -0.003 | 0.026 | -0.025 | 0.016 |
| rs62471080 | 7 | 1.09E+08 | G | C | -0.009 | 0.003 | 9.05E-04 | -0.013 | 0.002 | 3.70E-09 | TV | 0.005 | 0.006 | 0.006 | 0.008 | 0.027 | 0.018 | 0.008 | 0.015 | 0.004 | 0.026 | -0.004 | 0.015 |
| rs10246289 | 7 | 1.16E+08 | A | G | 0.004 | 0.004 | 3.87E-01 | 0.019 | 0.003 | 1.70E-08 | TV | 0.021 | 0.009 | 0.018 | 0.012 | 0.021 | 0.027 | 0.014 | 0.024 | 0.032 | 0.040 | 0.048 | 0.024 |
| rs11763734 | 7 | 1.26E+08 | A | C | 0.000 | 0.002 | 9.14E-01 | -0.012 | 0.002 | 8.20E-09 | TV | 0.003 | 0.006 | -0.003 | 0.008 | 0.011 | 0.018 | 0.009 | 0.015 | -0.006 | 0.026 | 0.024 | 0.015 |
| rs2968864 | 7 | 1.51E+08 | T | C | 0.016 | 0.003 | 9.35E-10 | 0.004 | 0.002 | 7.70E-02 | BMI | -0.013 | 0.007 | -0.022 | 0.009 | -0.017 | 0.021 | -0.003 | 0.018 | -0.009 | 0.030 | -0.004 | 0.018 |
| rs7834121 | 8 | 10770255 | G | T | 0.017 | 0.003 | 1.79E-10 | -0.014 | 0.002 | 1.10E-10 | TV | -0.008 | 0.007 | -0.009 | 0.009 | 0.008 | 0.021 | 0.005 | 0.017 | -0.013 | 0.028 | -0.008 | 0.018 |
| rs2959592 | 8 | 17002725 | T | C | 0.017 | 0.003 | 6.26E-10 | 0.006 | 0.003 | 3.30E-02 | BMI | 0.002 | 0.008 | 0.001 | 0.010 | 0.033 | 0.023 | -0.001 | 0.020 | -0.009 | 0.033 | 0.008 | 0.020 |
| rs1421334 | 8 | 30865733 | A | C | 0.013 | 0.002 | 2.39E-08 | 0.017 | 0.002 | 2.20E-15 | TV | 0.005 | 0.006 | 0.002 | 0.008 | 0.031 | 0.018 | -0.006 | 0.015 | 0.018 | 0.026 | 0.009 | 0.016 |
| rs4320543 | 8 | 34422967 | A | G | -0.013 | 0.002 | 2.95E-08 | -0.010 | 0.002 | 6.30E-06 | BMI | 0.004 | 0.006 | 0.002 | 0.008 | 0.002 | 0.019 | 0.026 | 0.016 | 0.002 | 0.027 | 0.006 | 0.016 |
| rs12681792 | 8 | 62054463 | A | C | 0.018 | 0.003 | 2.61E-10 | 0.007 | 0.003 | 1.50E-02 | BMI | 0.010 | 0.008 | 0.016 | 0.011 | 0.020 | 0.023 | 0.025 | 0.020 | -0.045 | 0.035 | -0.005 | 0.020 |
| rs7827848 | 8 | 64790774 | A | T | 0.015 | 0.002 | 1.88E-11 | 0.009 | 0.002 | 8.20E-05 | BMI | 0.003 | 0.006 | -0.005 | 0.008 | 0.002 | 0.018 | 0.019 | 0.016 | -0.001 | 0.027 | -0.011 | 0.016 |
| rs6996198 | 8 | 65463442 | C | T | -0.002 | 0.003 | 4.82E-01 | -0.016 | 0.003 | 3.50E-08 | TV | -0.001 | 0.008 | -0.001 | 0.011 | -0.002 | 0.024 | -0.002 | 0.020 | 0.065 | 0.036 | -0.036 | 0.020 |
| rs6472942 | 8 | 76827190 | T | C | 0.017 | 0.002 | 3.53E-13 | -0.013 | 0.002 | 1.80E-09 | TV | -0.012 | 0.006 | -0.020 | 0.008 | 0.023 | 0.018 | -0.013 | 0.015 | -0.031 | 0.025 | -0.006 | 0.015 |
| rs11985394 | 8 | 92667521 | T | A | 0.003 | 0.002 | 2.74E-01 | 0.012 | 0.002 | 1.30E-08 | TV | 0.005 | 0.006 | 0.010 | 0.008 | 0.002 | 0.018 | 0.005 | 0.015 | -0.012 | 0.026 | 0.001 | 0.016 |
| rs2721965 | 8 | 1.17E+08 | A | C | 0.019 | 0.002 | 2.10E-14 | 0.006 | 0.002 | 6.60E-03 | BMI | 0.020 | 0.006 | 0.026 | 0.008 | -0.008 | 0.019 | 0.038 | 0.016 | -0.024 | 0.027 | -0.008 | 0.016 |
| rs12541615 | 8 | 1.19E+08 | T | C | -0.010 | 0.003 | 3.99E-03 | -0.018 | 0.003 | 3.90E-10 | TV | 0.029 | 0.008 | 0.037 | 0.011 | 0.035 | 0.023 | 0.048 | 0.020 | 0.039 | 0.034 | 0.004 | 0.020 |
| rs10099330 | 8 | 1.43E+08 | A | G | -0.013 | 0.002 | 9.94E-09 | -0.007 | 0.002 | 9.80E-04 | BMI | -0.002 | 0.006 | 0.009 | 0.008 | -0.026 | 0.018 | 0.001 | 0.015 | -0.008 | 0.026 | 0.002 | 0.015 |
| rs2717559 | 8 | 1.44E+08 | A | G | 0.005 | 0.003 | 8.28E-02 | 0.012 | 0.002 | 1.50E-08 | TV | 0.000 | 0.007 | -0.009 | 0.009 | 0.018 | 0.019 | -0.022 | 0.016 | 0.030 | 0.028 | -0.003 | 0.017 |
| rs2616830 | 9 | 1721385 | G | A | 0.003 | 0.002 | 2.41E-01 | 0.016 | 0.002 | 2.90E-14 | TV | 0.003 | 0.006 | 0.006 | 0.008 | -0.001 | 0.018 | 0.012 | 0.015 | -0.052 | 0.026 | 0.001 | 0.015 |
| rs10124645 | 9 | 11321119 | A | G | 0.014 | 0.002 | 4.98E-09 | 0.007 | 0.002 | 2.70E-03 | BMI | -0.006 | 0.006 | -0.012 | 0.008 | -0.024 | 0.018 | 0.017 | 0.016 | 0.032 | 0.027 | 0.008 | 0.016 |
| rs34864022 | 9 | 22609110 | A | G | 0.001 | 0.005 | 9.19E-01 | -0.026 | 0.004 | 1.40E-09 | TV | 0.008 | 0.013 | 0.002 | 0.017 | 0.001 | 0.038 | 0.043 | 0.032 | -0.046 | 0.054 | 0.011 | 0.033 |
| rs12554512 | 9 | 23352293 | T | C | -0.012 | 0.003 | 4.61E-06 | 0.021 | 0.002 | 3.80E-21 | TV | -0.006 | 0.006 | -0.001 | 0.008 | -0.038 | 0.019 | -0.006 | 0.016 | 0.017 | 0.027 | -0.018 | 0.016 |
| rs16912921 | 9 | 28413461 | A | C | 0.025 | 0.002 | 1.56E-26 | 0.005 | 0.002 | 4.70E-02 | BMI | -0.001 | 0.006 | 0.011 | 0.008 | -0.015 | 0.019 | 0.003 | 0.016 | -0.002 | 0.027 | -0.015 | 0.016 |
| rs973345 | 9 | 29713169 | T | C | 0.014 | 0.002 | 1.09E-09 | 0.005 | 0.002 | 2.20E-02 | BMI | 0.008 | 0.006 | 0.022 | 0.008 | 0.004 | 0.018 | -0.016 | 0.015 | 0.025 | 0.026 | 0.021 | 0.015 |
| rs563132 | 9 | 37245476 | A | T | 0.018 | 0.002 | 1.96E-14 | -0.006 | 0.002 | 4.50E-03 | BMI | -0.014 | 0.006 | -0.021 | 0.008 | -0.006 | 0.018 | -0.030 | 0.015 | -0.014 | 0.026 | -0.008 | 0.016 |
| rs10116186 | 9 | 80532374 | A | G | -0.015 | 0.003 | 4.10E-08 | -0.003 | 0.002 | 1.70E-01 | BMI | 0.015 | 0.006 | 0.017 | 0.008 | 0.025 | 0.018 | 0.030 | 0.015 | 0.029 | 0.026 | 0.017 | 0.015 |
| rs2791574 | 9 | 82212094 | T | G | -0.004 | 0.002 | 8.01E-02 | -0.013 | 0.002 | 3.00E-08 | TV | 0.003 | 0.006 | 0.011 | 0.008 | 0.009 | 0.018 | -0.004 | 0.016 | 0.026 | 0.027 | -0.029 | 0.016 |
| rs870151 | 9 | 92230747 | T | A | -0.014 | 0.002 | 4.62E-09 | -0.016 | 0.002 | 8.80E-13 | TV | 0.005 | 0.006 | -0.008 | 0.008 | 0.014 | 0.018 | -0.005 | 0.015 | 0.043 | 0.026 | 0.021 | 0.015 |
| rs2787374 | 9 | 1.03E+08 | T | C | 0.013 | 0.002 | 5.57E-08 | 0.012 | 0.002 | 1.20E-08 | TV | -0.004 | 0.006 | -0.010 | 0.008 | 0.001 | 0.018 | 0.042 | 0.015 | -0.015 | 0.026 | 0.000 | 0.015 |
| rs7024334 | 9 | 1.09E+08 | T | G | 0.016 | 0.003 | 1.63E-09 | 0.000 | 0.003 | 8.90E-01 | BMI | 0.016 | 0.007 | 0.032 | 0.009 | -0.063 | 0.021 | 0.054 | 0.018 | 0.007 | 0.030 | 0.029 | 0.018 |
| rs1928295 | 9 | 1.2E+08 | T | C | 0.016 | 0.002 | 2.85E-13 | 0.006 | 0.002 | 3.90E-03 | BMI | 0.002 | 0.006 | 0.006 | 0.008 | -0.001 | 0.018 | -0.005 | 0.015 | -0.010 | 0.026 | 0.003 | 0.015 |
| rs7043521 | 9 | 1.25E+08 | A | T | 0.013 | 0.003 | 1.97E-06 | 0.014 | 0.002 | 6.50E-11 | TV | 0.003 | 0.006 | 0.003 | 0.008 | -0.019 | 0.018 | 0.016 | 0.015 | 0.037 | 0.026 | -0.019 | 0.015 |
| rs4382592 | 9 | 1.35E+08 | T | G | 0.007 | 0.003 | 4.41E-03 | 0.014 | 0.002 | 6.70E-09 | TV | 0.011 | 0.007 | 0.004 | 0.009 | 0.030 | 0.019 | 0.002 | 0.016 | 0.045 | 0.028 | 0.006 | 0.017 |
| rs2073869 | 9 | 1.36E+08 | C | T | 0.008 | 0.003 | 1.17E-02 | 0.019 | 0.003 | 1.50E-10 | TV | 0.011 | 0.008 | 0.001 | 0.011 | 0.012 | 0.023 | 0.029 | 0.020 | 0.019 | 0.034 | -0.013 | 0.020 |
| rs1623025 | 10 | 11164813 | G | C | -0.003 | 0.003 | 2.47E-01 | -0.012 | 0.002 | 1.40E-08 | TV | -0.009 | 0.006 | -0.012 | 0.008 | -0.011 | 0.018 | -0.013 | 0.015 | -0.044 | 0.026 | 0.000 | 0.015 |
| rs1243182 | 10 | 21916728 | C | T | -0.020 | 0.003 | 3.15E-15 | -0.019 | 0.002 | 2.00E-15 | TV | -0.056 | 0.006 | -0.081 | 0.008 | -0.059 | 0.019 | -0.051 | 0.016 | -0.016 | 0.027 | 0.042 | 0.016 |
| rs34930419 | 10 | 33987329 | T | C | -0.046 | 0.008 | 1.37E-09 | 0.000 | 0.006 | 9.70E-01 | BMI | -0.010 | 0.018 | -0.030 | 0.023 | -0.019 | 0.052 | 0.014 | 0.045 | 0.052 | 0.079 | -0.009 | 0.045 |
| rs2045147 | 10 | 56660437 | A | G | 0.003 | 0.002 | 1.72E-01 | 0.013 | 0.002 | 5.90E-09 | TV | 0.002 | 0.006 | -0.002 | 0.008 | 0.023 | 0.018 | 0.032 | 0.015 | 0.045 | 0.026 | 0.001 | 0.015 |
| rs10994943 | 10 | 63591413 | T | G | 0.007 | 0.003 | 1.48E-02 | 0.013 | 0.002 | 3.10E-09 | TV | 0.011 | 0.006 | 0.010 | 0.008 | -0.009 | 0.018 | 0.015 | 0.015 | 0.028 | 0.026 | 0.032 | 0.015 |
| rs10824347 | 10 | 77645229 | A | T | 0.018 | 0.003 | 5.47E-11 | 0.006 | 0.003 | 1.10E-02 | BMI | 0.006 | 0.007 | 0.013 | 0.009 | -0.008 | 0.021 | 0.018 | 0.018 | -0.034 | 0.030 | -0.004 | 0.018 |
| rs11201422 | 10 | 86924483 | T | C | 0.012 | 0.003 | 1.45E-06 | 0.013 | 0.002 | 3.40E-08 | TV | -0.015 | 0.007 | -0.007 | 0.009 | -0.025 | 0.019 | -0.027 | 0.016 | -0.059 | 0.028 | 0.007 | 0.017 |
| rs563296 | 10 | 99772404 | A | G | 0.018 | 0.002 | 8.85E-14 | 0.007 | 0.002 | 9.30E-04 | BMI | 0.005 | 0.006 | 0.003 | 0.008 | 0.002 | 0.018 | -0.012 | 0.016 | -0.021 | 0.027 | 0.008 | 0.016 |
| rs10786658 | 10 | 1.04E+08 | A | T | 0.002 | 0.003 | 3.88E-01 | -0.014 | 0.002 | 4.60E-11 | TV | 0.001 | 0.006 | 0.002 | 0.008 | -0.032 | 0.018 | 0.022 | 0.015 | -0.011 | 0.026 | 0.014 | 0.016 |
| rs7089973 | 10 | 1.17E+08 | C | A | 0.000 | 0.002 | 9.06E-01 | -0.013 | 0.002 | 2.40E-09 | TV | -0.005 | 0.006 | -0.008 | 0.009 | 0.025 | 0.019 | -0.007 | 0.016 | -0.045 | 0.027 | 0.002 | 0.016 |
| rs11245482 | 10 | 1.27E+08 | T | C | -0.011 | 0.002 | 4.63E-06 | -0.013 | 0.002 | 2.60E-09 | TV | -0.005 | 0.006 | -0.003 | 0.008 | -0.037 | 0.018 | 0.004 | 0.015 | -0.016 | 0.026 | 0.022 | 0.016 |
| rs17727474 | 10 | 1.27E+08 | C | T | -0.004 | 0.003 | 2.43E-01 | 0.018 | 0.003 | 3.10E-09 | TV | -0.013 | 0.008 | -0.020 | 0.011 | 0.021 | 0.025 | -0.048 | 0.021 | -0.078 | 0.035 | 0.025 | 0.022 |
| rs7395632 | 11 | 958789 | T | C | -0.013 | 0.002 | 3.87E-08 | 0.000 | 0.002 | 9.00E-01 | BMI | -0.001 | 0.006 | 0.012 | 0.008 | -0.020 | 0.018 | -0.040 | 0.016 | -0.030 | 0.027 | -0.006 | 0.016 |
| rs4929923 | 11 | 8639200 | T | C | -0.022 | 0.002 | 2.51E-22 | -0.003 | 0.002 | 1.30E-01 | BMI | -0.002 | 0.006 | -0.008 | 0.008 | -0.006 | 0.018 | 0.010 | 0.016 | -0.018 | 0.026 | 0.004 | 0.016 |
| rs12289262 | 11 | 12894758 | C | T | -0.009 | 0.003 | 4.88E-04 | -0.014 | 0.002 | 2.00E-08 | TV | 0.002 | 0.007 | 0.008 | 0.009 | -0.064 | 0.021 | -0.013 | 0.018 | -0.001 | 0.030 | 0.012 | 0.018 |
| rs11022762 | 11 | 13335926 | T | C | -0.015 | 0.002 | 5.56E-11 | -0.005 | 0.002 | 1.30E-02 | BMI | -0.001 | 0.006 | 0.000 | 0.008 | 0.014 | 0.018 | -0.001 | 0.015 | 0.002 | 0.026 | 0.007 | 0.015 |
| rs6265 | 11 | 27679916 | T | C | -0.037 | 0.003 | 7.95E-40 | 0.003 | 0.003 | 2.20E-01 | BMI | -0.009 | 0.008 | -0.005 | 0.010 | -0.013 | 0.022 | 0.011 | 0.019 | -0.005 | 0.032 | -0.002 | 0.019 |
| rs2862961 | 11 | 43656535 | A | G | -0.024 | 0.003 | 5.30E-22 | -0.004 | 0.002 | 1.10E-01 | BMI | -0.007 | 0.007 | -0.021 | 0.009 | 0.009 | 0.019 | 0.004 | 0.016 | -0.031 | 0.028 | 0.019 | 0.017 |
| rs10742752 | 11 | 45438374 | T | C | -0.013 | 0.002 | 3.98E-09 | 0.002 | 0.002 | 3.80E-01 | BMI | 0.009 | 0.006 | 0.016 | 0.008 | -0.006 | 0.018 | 0.014 | 0.016 | 0.000 | 0.027 | 0.014 | 0.016 |
| rs7124681 | 11 | 47529947 | A | C | 0.025 | 0.002 | 9.27E-29 | 0.004 | 0.002 | 5.10E-02 | BMI | -0.005 | 0.006 | -0.018 | 0.008 | -0.011 | 0.018 | -0.009 | 0.015 | -0.013 | 0.026 | -0.006 | 0.015 |
| rs801733 | 11 | 65934549 | A | C | 0.009 | 0.002 | 1.67E-04 | 0.017 | 0.002 | 7.30E-14 | TV | 0.023 | 0.006 | 0.031 | 0.008 | 0.016 | 0.019 | 0.001 | 0.016 | 0.057 | 0.027 | -0.002 | 0.016 |
| rs116175338 | 11 | 72467875 | G | T | -0.008 | 0.003 | 2.76E-03 | -0.014 | 0.003 | 1.40E-08 | TV | -0.017 | 0.007 | -0.028 | 0.009 | 0.007 | 0.021 | -0.004 | 0.018 | -0.016 | 0.030 | -0.025 | 0.018 |
| rs12272012 | 11 | 84861286 | G | C | -0.010 | 0.007 | 1.20E-01 | -0.030 | 0.005 | 1.90E-08 | TV | -0.023 | 0.015 | -0.017 | 0.019 | 0.098 | 0.046 | -0.003 | 0.037 | -0.045 | 0.063 | -0.040 | 0.037 |
| rs11020045 | 11 | 92487907 | A | C | 0.002 | 0.002 | 5.41E-01 | -0.013 | 0.002 | 1.60E-08 | TV | -0.009 | 0.006 | -0.003 | 0.008 | 0.000 | 0.019 | -0.017 | 0.016 | -0.019 | 0.027 | -0.022 | 0.016 |
| rs17207890 | 11 | 95490754 | G | A | -0.002 | 0.002 | 5.51E-01 | 0.016 | 0.002 | 6.70E-12 | TV | 0.017 | 0.006 | 0.033 | 0.008 | 0.000 | 0.019 | 0.039 | 0.016 | -0.011 | 0.027 | 0.019 | 0.016 |
| rs1003081 | 11 | 1.19E+08 | T | C | 0.014 | 0.002 | 6.27E-11 | 0.003 | 0.002 | 2.40E-01 | BMI | -0.016 | 0.006 | -0.010 | 0.008 | -0.001 | 0.018 | -0.036 | 0.015 | -0.015 | 0.026 | -0.024 | 0.016 |
| rs11218575 | 11 | 1.22E+08 | C | T | 0.005 | 0.002 | 4.42E-02 | 0.015 | 0.002 | 2.10E-12 | TV | 0.009 | 0.006 | 0.000 | 0.009 | 0.024 | 0.019 | 0.001 | 0.016 | 0.063 | 0.027 | 0.008 | 0.016 |
| rs4937842 | 11 | 1.34E+08 | G | C | -0.009 | 0.002 | 2.80E-04 | -0.013 | 0.002 | 1.40E-08 | TV | 0.009 | 0.006 | 0.012 | 0.008 | -0.005 | 0.018 | 0.017 | 0.016 | 0.020 | 0.027 | 0.010 | 0.016 |
| rs11611246 | 12 | 939480 | T | G | 0.021 | 0.003 | 1.27E-14 | -0.002 | 0.003 | 4.20E-01 | BMI | 0.010 | 0.007 | 0.007 | 0.010 | 0.036 | 0.022 | 0.029 | 0.018 | -0.018 | 0.032 | 0.009 | 0.019 |
| rs10772643 | 12 | 13415288 | C | T | 0.013 | 0.004 | 1.86E-03 | 0.025 | 0.003 | 1.30E-12 | TV | 0.008 | 0.010 | 0.022 | 0.013 | -0.012 | 0.030 | -0.005 | 0.025 | -0.044 | 0.044 | -0.017 | 0.026 |
| rs7976757 | 12 | 19207948 | T | C | -0.019 | 0.003 | 2.94E-10 | -0.006 | 0.003 | 2.90E-02 | BMI | -0.006 | 0.008 | 0.008 | 0.011 | -0.029 | 0.024 | 0.015 | 0.020 | -0.024 | 0.035 | 0.007 | 0.021 |
| rs16926318 | 12 | 23678745 | A | G | -0.023 | 0.004 | 4.23E-08 | -0.011 | 0.004 | 5.50E-03 | BMI | 0.005 | 0.011 | -0.008 | 0.014 | -0.010 | 0.031 | 0.022 | 0.027 | 0.005 | 0.045 | 0.028 | 0.027 |
| rs16926778 | 12 | 24002125 | C | G | 0.026 | 0.004 | 1.41E-09 | 0.003 | 0.004 | 5.30E-01 | BMI | 0.006 | 0.011 | 0.028 | 0.015 | 0.039 | 0.033 | -0.038 | 0.029 | 0.028 | 0.048 | 0.012 | 0.029 |
| rs10771746 | 12 | 30791864 | C | T | -0.003 | 0.003 | 3.36E-01 | -0.014 | 0.002 | 2.50E-09 | TV | -0.024 | 0.007 | -0.015 | 0.009 | -0.037 | 0.019 | -0.053 | 0.016 | -0.018 | 0.028 | 0.013 | 0.017 |
| rs7138803 | 12 | 50247468 | A | G | 0.029 | 0.002 | 9.70E-38 | 0.004 | 0.002 | 1.10E-01 | BMI | -0.004 | 0.006 | -0.010 | 0.008 | -0.004 | 0.018 | -0.004 | 0.015 | -0.008 | 0.026 | -0.006 | 0.016 |
| rs10876864 | 12 | 56401085 | G | A | -0.010 | 0.002 | 6.64E-06 | -0.013 | 0.002 | 1.00E-09 | TV | 0.004 | 0.006 | 0.008 | 0.008 | 0.040 | 0.018 | -0.011 | 0.015 | 0.008 | 0.026 | -0.024 | 0.015 |
| rs11173522 | 12 | 60953472 | A | C | 0.017 | 0.003 | 9.01E-10 | 0.004 | 0.003 | 1.20E-01 | BMI | -0.006 | 0.007 | -0.017 | 0.010 | 0.004 | 0.022 | -0.025 | 0.019 | 0.002 | 0.031 | 0.019 | 0.019 |
| rs8756 | 12 | 66359752 | C | A | 0.001 | 0.002 | 5.52E-01 | -0.013 | 0.002 | 5.30E-10 | TV | -0.003 | 0.006 | 0.004 | 0.008 | 0.009 | 0.018 | -0.007 | 0.015 | 0.019 | 0.025 | -0.023 | 0.015 |
| rs10746306 | 12 | 84477374 | T | G | 0.018 | 0.003 | 8.74E-09 | 0.003 | 0.003 | 2.70E-01 | BMI | 0.011 | 0.007 | 0.007 | 0.009 | 0.031 | 0.020 | 0.005 | 0.017 | 0.015 | 0.029 | 0.002 | 0.017 |
| rs2279574 | 12 | 89745477 | A | C | -0.017 | 0.002 | 1.96E-12 | -0.006 | 0.002 | 7.80E-03 | BMI | 0.003 | 0.007 | 0.009 | 0.009 | 0.006 | 0.019 | -0.007 | 0.017 | 0.035 | 0.028 | -0.018 | 0.017 |
| rs2712643 | 12 | 99638276 | A | C | -0.015 | 0.003 | 1.47E-09 | -0.004 | 0.003 | 1.40E-01 | BMI | 0.000 | 0.007 | -0.002 | 0.009 | 0.005 | 0.020 | 0.015 | 0.017 | -0.033 | 0.030 | -0.022 | 0.018 |
| rs17033633 | 12 | 1.04E+08 | T | C | -0.021 | 0.003 | 7.26E-12 | -0.003 | 0.003 | 2.30E-01 | BMI | 0.019 | 0.008 | 0.013 | 0.010 | -0.003 | 0.023 | 0.006 | 0.020 | -0.002 | 0.034 | 0.011 | 0.020 |
| rs2164744 | 12 | 1.1E+08 | T | C | -0.006 | 0.003 | 4.25E-02 | -0.013 | 0.002 | 1.10E-08 | TV | 0.019 | 0.006 | 0.009 | 0.008 | 0.024 | 0.018 | 0.042 | 0.016 | 0.072 | 0.026 | 0.011 | 0.016 |
| rs2173650 | 12 | 1.18E+08 | G | T | 0.003 | 0.004 | 4.77E-01 | 0.018 | 0.003 | 4.60E-09 | TV | 0.016 | 0.009 | 0.014 | 0.014 | 0.012 | 0.028 | 0.009 | 0.025 | 0.036 | 0.042 | 0.043 | 0.026 |
| rs9579775 | 13 | 20616557 | A | C | -0.025 | 0.004 | 3.66E-10 | -0.003 | 0.003 | 3.20E-01 | BMI | 0.023 | 0.011 | 0.044 | 0.015 | -0.028 | 0.031 | -0.007 | 0.027 | 0.003 | 0.046 | 0.067 | 0.028 |
| rs12872889 | 13 | 28674628 | T | C | -0.021 | 0.003 | 1.52E-11 | -0.002 | 0.003 | 5.50E-01 | BMI | 0.006 | 0.007 | 0.010 | 0.009 | 0.006 | 0.021 | 0.033 | 0.018 | 0.023 | 0.031 | -0.017 | 0.018 |
| rs9595630 | 13 | 33065443 | T | G | 0.015 | 0.002 | 2.04E-10 | 0.008 | 0.002 | 6.00E-04 | BMI | -0.006 | 0.006 | -0.008 | 0.008 | -0.036 | 0.018 | -0.006 | 0.015 | 0.008 | 0.026 | 0.005 | 0.016 |
| rs9563168 | 13 | 54247827 | G | A | 0.010 | 0.003 | 4.82E-04 | 0.018 | 0.003 | 4.30E-11 | TV | 0.003 | 0.008 | 0.011 | 0.010 | 0.010 | 0.022 | 0.003 | 0.019 | -0.004 | 0.032 | -0.013 | 0.019 |
| rs9569734 | 13 | 58319476 | A | G | 0.019 | 0.003 | 3.51E-09 | 0.019 | 0.003 | 3.40E-10 | TV | 0.002 | 0.008 | 0.010 | 0.011 | -0.034 | 0.024 | 0.014 | 0.021 | 0.002 | 0.036 | 0.023 | 0.021 |
| rs9571687 | 13 | 67472713 | A | C | -0.015 | 0.002 | 3.24E-09 | -0.005 | 0.002 | 3.40E-02 | BMI | -0.006 | 0.007 | -0.001 | 0.009 | -0.025 | 0.019 | -0.016 | 0.016 | -0.028 | 0.028 | 0.006 | 0.016 |
| rs1441264 | 13 | 79580919 | A | G | 0.017 | 0.002 | 5.19E-14 | -0.003 | 0.002 | 1.40E-01 | BMI | -0.008 | 0.007 | -0.024 | 0.009 | 0.006 | 0.019 | 0.000 | 0.016 | -0.033 | 0.027 | -0.009 | 0.017 |
| rs56858768 | 13 | 86511730 | G | A | -0.017 | 0.003 | 1.04E-08 | -0.015 | 0.002 | 3.40E-10 | TV | 0.012 | 0.007 | 0.008 | 0.009 | 0.012 | 0.020 | 0.017 | 0.017 | -0.002 | 0.029 | 0.004 | 0.017 |
| rs1927790 | 13 | 96922191 | T | C | -0.014 | 0.002 | 1.27E-09 | -0.005 | 0.002 | 3.20E-02 | BMI | 0.002 | 0.006 | 0.000 | 0.008 | -0.018 | 0.018 | 0.012 | 0.016 | 0.032 | 0.026 | -0.012 | 0.016 |
| rs7991062 | 13 | 1.01E+08 | C | G | -0.004 | 0.002 | 1.25E-01 | -0.018 | 0.002 | 8.80E-15 | TV | -0.004 | 0.006 | -0.012 | 0.008 | -0.001 | 0.019 | -0.001 | 0.016 | -0.036 | 0.027 | -0.005 | 0.016 |
| rs7335249 | 13 | 1.1E+08 | T | C | 0.014 | 0.002 | 4.91E-09 | 0.003 | 0.002 | 1.20E-01 | BMI | 0.002 | 0.006 | -0.002 | 0.008 | 0.006 | 0.018 | 0.009 | 0.016 | 0.005 | 0.026 | 0.001 | 0.016 |
| rs2479958 | 13 | 1.12E+08 | A | G | 0.014 | 0.002 | 1.38E-08 | 0.004 | 0.002 | 4.50E-02 | BMI | -0.004 | 0.006 | -0.001 | 0.008 | 0.028 | 0.018 | 0.008 | 0.016 | -0.033 | 0.027 | -0.013 | 0.016 |
| rs405797 | 14 | 26972834 | T | A | -0.005 | 0.003 | 4.22E-02 | -0.015 | 0.003 | 2.80E-09 | TV | 0.015 | 0.008 | 0.013 | 0.010 | 0.017 | 0.022 | 0.036 | 0.019 | 0.027 | 0.032 | 0.004 | 0.019 |
| rs1022785 | 14 | 29571653 | G | A | 0.010 | 0.003 | 1.48E-03 | 0.018 | 0.003 | 7.10E-09 | TV | 0.008 | 0.009 | 0.009 | 0.011 | 0.007 | 0.025 | -0.014 | 0.022 | -0.026 | 0.037 | 0.032 | 0.022 |
| rs35020117 | 14 | 73341655 | T | G | 0.017 | 0.003 | 1.23E-09 | 0.003 | 0.002 | 2.20E-01 | BMI | -0.005 | 0.006 | -0.003 | 0.009 | 0.019 | 0.019 | -0.025 | 0.016 | 0.005 | 0.028 | -0.007 | 0.016 |
| rs7141420 | 14 | 79899454 | T | C | 0.021 | 0.002 | 7.84E-22 | 0.006 | 0.002 | 6.50E-03 | BMI | 0.012 | 0.006 | 0.007 | 0.008 | 0.034 | 0.018 | -0.002 | 0.016 | 0.000 | 0.027 | 0.021 | 0.016 |
| rs10145592 | 14 | 94287215 | C | G | -0.004 | 0.002 | 6.48E-02 | -0.015 | 0.002 | 1.80E-11 | TV | 0.003 | 0.006 | -0.015 | 0.009 | -0.009 | 0.019 | 0.006 | 0.016 | 0.030 | 0.027 | 0.020 | 0.016 |
| rs7157001 | 14 | 99749484 | A | G | -0.003 | 0.003 | 3.52E-01 | -0.014 | 0.002 | 2.30E-08 | TV | -0.003 | 0.008 | -0.007 | 0.011 | -0.020 | 0.024 | 0.027 | 0.021 | 0.018 | 0.035 | 0.013 | 0.021 |
| rs3803286 | 14 | 1.03E+08 | A | G | 0.017 | 0.002 | 8.29E-13 | 0.000 | 0.002 | 9.90E-01 | BMI | -0.020 | 0.006 | -0.032 | 0.008 | -0.018 | 0.019 | 0.002 | 0.016 | 0.013 | 0.027 | 0.015 | 0.016 |
| rs12101625 | 15 | 26972431 | C | G | 0.014 | 0.002 | 5.54E-09 | 0.002 | 0.002 | 4.40E-01 | BMI | -0.018 | 0.006 | -0.021 | 0.008 | -0.021 | 0.018 | -0.018 | 0.016 | 0.020 | 0.027 | -0.024 | 0.016 |
| rs8043253 | 15 | 41503730 | C | T | -0.006 | 0.002 | 9.24E-03 | -0.012 | 0.002 | 3.40E-08 | TV | -0.004 | 0.006 | -0.018 | 0.008 | 0.016 | 0.018 | 0.012 | 0.015 | -0.004 | 0.026 | -0.008 | 0.015 |
| rs2460 | 15 | 53073084 | G | A | -0.011 | 0.003 | 1.94E-05 | -0.015 | 0.002 | 5.00E-10 | TV | 0.000 | 0.007 | -0.006 | 0.010 | -0.001 | 0.021 | -0.007 | 0.018 | 0.024 | 0.031 | 0.024 | 0.019 |
| rs8024806 | 15 | 53473990 | T | C | 0.027 | 0.005 | 9.76E-09 | 0.010 | 0.005 | 2.60E-02 | BMI | -0.010 | 0.013 | -0.024 | 0.017 | -0.025 | 0.036 | 0.012 | 0.032 | 0.031 | 0.055 | -0.015 | 0.032 |
| rs4775373 | 15 | 61476936 | T | C | 0.005 | 0.003 | 7.06E-02 | 0.013 | 0.002 | 4.70E-09 | TV | 0.002 | 0.006 | 0.000 | 0.008 | -0.001 | 0.019 | 0.004 | 0.016 | 0.034 | 0.027 | -0.026 | 0.016 |
| rs12914489 | 15 | 74187937 | A | G | 0.023 | 0.004 | 1.64E-10 | 0.010 | 0.003 | 3.50E-03 | BMI | 0.022 | 0.010 | 0.026 | 0.013 | 0.026 | 0.029 | 0.051 | 0.024 | 0.026 | 0.042 | -0.030 | 0.026 |
| rs61331678 | 15 | 77995949 | G | C | 0.008 | 0.003 | 2.59E-03 | 0.015 | 0.002 | 6.10E-12 | TV | 0.002 | 0.006 | 0.002 | 0.008 | 0.027 | 0.018 | -0.034 | 0.015 | -0.027 | 0.026 | 0.017 | 0.016 |
| rs7498044 | 15 | 92573639 | A | G | -0.018 | 0.003 | 2.73E-08 | -0.011 | 0.003 | 6.40E-05 | BMI | 0.005 | 0.008 | 0.002 | 0.010 | 0.027 | 0.022 | -0.015 | 0.019 | 0.000 | 0.033 | -0.003 | 0.019 |
| rs11073383 | 15 | 95274349 | A | G | -0.016 | 0.002 | 3.26E-12 | -0.003 | 0.002 | 2.30E-01 | BMI | 0.003 | 0.006 | 0.001 | 0.008 | 0.002 | 0.018 | -0.004 | 0.015 | 0.023 | 0.025 | -0.013 | 0.015 |
| rs57221746 | 15 | 99230035 | A | G | -0.018 | 0.003 | 2.08E-08 | -0.008 | 0.003 | 1.00E-03 | BMI | 0.004 | 0.007 | 0.015 | 0.010 | 0.001 | 0.021 | 0.010 | 0.018 | 0.032 | 0.031 | -0.015 | 0.019 |
| rs214249 | 16 | 348687 | T | G | 0.017 | 0.002 | 1.37E-12 | 0.007 | 0.002 | 1.30E-03 | BMI | 0.000 | 0.006 | 0.003 | 0.008 | -0.004 | 0.018 | 0.016 | 0.015 | 0.015 | 0.026 | 0.003 | 0.015 |
| rs12448257 | 16 | 3599655 | A | G | 0.015 | 0.003 | 3.13E-08 | -0.004 | 0.003 | 1.20E-01 | BMI | 0.002 | 0.008 | 0.001 | 0.010 | -0.012 | 0.022 | 0.027 | 0.019 | -0.043 | 0.033 | 0.009 | 0.019 |
| rs879620 | 16 | 4015729 | T | C | 0.025 | 0.002 | 5.45E-26 | 0.006 | 0.002 | 1.20E-02 | BMI | 0.009 | 0.006 | 0.014 | 0.008 | 0.020 | 0.018 | 0.011 | 0.016 | 0.025 | 0.027 | 0.013 | 0.016 |
| rs35574015 | 16 | 15146061 | T | C | 0.004 | 0.003 | 2.01E-01 | -0.013 | 0.002 | 3.00E-08 | TV | -0.007 | 0.007 | 0.000 | 0.009 | -0.023 | 0.020 | -0.008 | 0.017 | 0.026 | 0.030 | -0.015 | 0.018 |
| rs7189927 | 16 | 28913787 | T | C | 0.025 | 0.002 | 3.50E-26 | 0.015 | 0.002 | 3.40E-11 | TV | 0.001 | 0.006 | 0.006 | 0.008 | -0.010 | 0.019 | -0.001 | 0.016 | 0.014 | 0.027 | -0.026 | 0.016 |
| rs749671 | 16 | 31088347 | G | A | 0.026 | 0.002 | 1.78E-31 | 0.016 | 0.002 | 2.70E-12 | TV | -0.017 | 0.006 | -0.014 | 0.008 | -0.010 | 0.018 | -0.010 | 0.015 | -0.002 | 0.026 | -0.015 | 0.016 |
| rs7184800 | 16 | 53509131 | G | A | -0.006 | 0.003 | 4.26E-02 | 0.017 | 0.002 | 8.20E-13 | TV | -0.005 | 0.006 | -0.010 | 0.008 | -0.014 | 0.019 | 0.005 | 0.016 | 0.026 | 0.028 | -0.011 | 0.016 |
| rs1558902 | 16 | 53803574 | A | T | 0.070 | 0.002 | 1.10E-215 | 0.001 | 0.002 | 6.60E-01 | BMI | -0.059 | 0.006 | -0.063 | 0.008 | -0.072 | 0.018 | -0.053 | 0.015 | -0.079 | 0.026 | -0.088 | 0.015 |
| rs4783718 | 16 | 69547741 | T | C | 0.020 | 0.002 | 1.77E-19 | 0.003 | 0.002 | 1.20E-01 | BMI | 0.015 | 0.006 | 0.008 | 0.008 | 0.043 | 0.019 | -0.019 | 0.016 | 0.022 | 0.027 | 0.035 | 0.016 |
| rs811054 | 16 | 72251132 | T | C | 0.016 | 0.002 | 3.18E-13 | -0.006 | 0.002 | 8.00E-03 | BMI | 0.019 | 0.006 | 0.009 | 0.008 | 0.036 | 0.018 | 0.030 | 0.015 | -0.011 | 0.026 | 0.012 | 0.015 |
| rs2012502 | 16 | 81728081 | A | C | 0.015 | 0.002 | 2.97E-11 | 0.002 | 0.002 | 2.80E-01 | BMI | -0.008 | 0.006 | -0.001 | 0.008 | -0.009 | 0.019 | -0.017 | 0.016 | -0.034 | 0.027 | -0.007 | 0.016 |
| rs2447098 | 17 | 2277720 | C | A | -0.002 | 0.002 | 3.60E-01 | -0.015 | 0.002 | 6.80E-12 | TV | 0.008 | 0.006 | 0.009 | 0.009 | 0.012 | 0.019 | 0.036 | 0.016 | -0.005 | 0.027 | 0.020 | 0.016 |
| rs11654952 | 17 | 4813799 | T | G | -0.001 | 0.003 | 7.62E-01 | -0.017 | 0.003 | 4.30E-08 | TV | 0.007 | 0.009 | 0.009 | 0.012 | 0.031 | 0.026 | 0.037 | 0.022 | 0.017 | 0.038 | -0.027 | 0.022 |
| rs1075901 | 17 | 15943910 | T | C | -0.014 | 0.002 | 4.36E-11 | 0.001 | 0.002 | 6.60E-01 | BMI | -0.001 | 0.006 | -0.003 | 0.008 | -0.010 | 0.018 | -0.011 | 0.015 | 0.012 | 0.026 | -0.019 | 0.015 |
| rs4986044 | 17 | 21261560 | T | C | -0.017 | 0.002 | 2.20E-15 | -0.009 | 0.002 | 1.80E-05 | BMI | -0.002 | 0.006 | -0.002 | 0.008 | 0.027 | 0.018 | -0.001 | 0.015 | -0.015 | 0.026 | 0.014 | 0.015 |
| rs12150665 | 17 | 34914787 | T | C | 0.017 | 0.002 | 5.92E-14 | 0.006 | 0.002 | 4.50E-03 | BMI | 0.014 | 0.007 | 0.024 | 0.009 | 0.025 | 0.019 | 0.003 | 0.017 | -0.020 | 0.028 | 0.003 | 0.017 |
| rs56161855 | 17 | 46288649 | A | T | -0.026 | 0.004 | 1.48E-11 | -0.007 | 0.003 | 2.00E-02 | BMI | 0.003 | 0.009 | -0.008 | 0.011 | 0.062 | 0.025 | 0.009 | 0.021 | -0.008 | 0.036 | 0.005 | 0.022 |
| rs9906944 | 17 | 47091420 | T | C | -0.019 | 0.002 | 7.25E-15 | -0.010 | 0.002 | 2.40E-05 | BMI | -0.004 | 0.007 | -0.002 | 0.009 | -0.028 | 0.019 | -0.017 | 0.017 | -0.039 | 0.028 | -0.007 | 0.017 |
| rs7211966 | 17 | 54374611 | T | C | -0.047 | 0.008 | 4.89E-09 | -0.005 | 0.008 | 5.00E-01 | BMI | 0.001 | 0.026 | 0.007 | 0.034 | 0.033 | 0.074 | -0.178 | 0.071 | 0.019 | 0.108 | -0.052 | 0.067 |
| rs8075273 | 17 | 61728881 | A | C | -0.016 | 0.002 | 5.56E-11 | -0.014 | 0.002 | 2.80E-09 | BMI | 0.011 | 0.007 | 0.006 | 0.009 | 0.011 | 0.019 | 0.018 | 0.017 | 0.042 | 0.028 | -0.001 | 0.017 |
| rs9902312 | 17 | 65070304 | T | C | -0.001 | 0.002 | 5.61E-01 | 0.015 | 0.002 | 4.50E-11 | TV | -0.009 | 0.006 | -0.024 | 0.008 | -0.026 | 0.019 | 0.016 | 0.016 | -0.021 | 0.027 | 0.009 | 0.016 |
| rs4969387 | 17 | 79081724 | C | G | -0.015 | 0.003 | 6.90E-09 | 0.001 | 0.002 | 6.40E-01 | BMI | -0.013 | 0.007 | -0.010 | 0.009 | -0.011 | 0.021 | 0.023 | 0.018 | -0.024 | 0.030 | -0.036 | 0.018 |
| rs11657730 | 17 | 79372489 | C | T | 0.002 | 0.003 | 4.99E-01 | 0.013 | 0.002 | 1.10E-08 | TV | 0.021 | 0.007 | 0.021 | 0.009 | 0.007 | 0.019 | 0.024 | 0.017 | 0.062 | 0.029 | 0.023 | 0.017 |
| rs9955276 | 18 | 1839339 | T | C | 0.022 | 0.003 | 8.02E-12 | 0.007 | 0.003 | 1.90E-02 | BMI | -0.002 | 0.009 | -0.003 | 0.011 | 0.034 | 0.025 | -0.021 | 0.021 | -0.044 | 0.036 | 0.045 | 0.021 |
| rs303753 | 18 | 21074922 | G | A | -0.019 | 0.002 | 1.99E-15 | -0.014 | 0.002 | 2.70E-10 | TV | -0.004 | 0.006 | -0.010 | 0.009 | -0.033 | 0.019 | 0.005 | 0.016 | 0.026 | 0.028 | -0.051 | 0.016 |
| rs9964724 | 18 | 35159124 | C | T | 0.003 | 0.003 | 2.32E-01 | 0.018 | 0.002 | 3.30E-14 | TV | 0.007 | 0.007 | 0.002 | 0.009 | 0.029 | 0.019 | 0.007 | 0.017 | -0.043 | 0.029 | 0.009 | 0.017 |
| rs1156541 | 18 | 39952989 | C | T | 0.014 | 0.003 | 2.81E-07 | 0.014 | 0.003 | 2.80E-08 | TV | -0.018 | 0.007 | -0.012 | 0.009 | -0.043 | 0.021 | -0.029 | 0.018 | -0.010 | 0.030 | -0.044 | 0.018 |
| rs17512836 | 18 | 53194961 | T | C | 0.021 | 0.008 | 1.30E-02 | 0.042 | 0.007 | 7.00E-10 | TV | -0.008 | 0.019 | -0.034 | 0.025 | 0.048 | 0.056 | -0.022 | 0.047 | -0.007 | 0.079 | 0.035 | 0.049 |
| rs6567160 | 18 | 57829135 | T | C | -0.057 | 0.003 | 8.74E-108 | 0.006 | 0.003 | 2.50E-02 | BMI | 0.021 | 0.007 | 0.001 | 0.009 | 0.024 | 0.021 | 0.025 | 0.018 | -0.002 | 0.030 | 0.012 | 0.018 |
| rs895330 | 19 | 4060707 | C | G | 0.018 | 0.003 | 6.65E-10 | 0.006 | 0.003 | 1.80E-02 | BMI | 0.021 | 0.008 | 0.031 | 0.011 | 0.016 | 0.023 | 0.033 | 0.020 | -0.011 | 0.034 | 0.025 | 0.021 |
| rs7248205 | 19 | 10770305 | C | T | 0.010 | 0.003 | 3.27E-04 | 0.014 | 0.002 | 3.40E-10 | TV | 0.012 | 0.006 | 0.012 | 0.008 | 0.030 | 0.019 | 0.021 | 0.016 | -0.012 | 0.027 | -0.022 | 0.016 |
| rs273504 | 19 | 18215247 | A | G | -0.015 | 0.002 | 1.27E-10 | -0.009 | 0.002 | 9.50E-05 | BMI | -0.017 | 0.006 | -0.019 | 0.008 | 0.012 | 0.018 | -0.039 | 0.015 | -0.041 | 0.026 | -0.016 | 0.015 |
| rs17724992 | 19 | 18454825 | A | G | 0.017 | 0.003 | 6.65E-12 | 0.004 | 0.002 | 1.10E-01 | BMI | 0.002 | 0.007 | 0.010 | 0.009 | 0.006 | 0.020 | -0.008 | 0.017 | 0.025 | 0.029 | -0.007 | 0.017 |
| rs111901094 | 19 | 19513570 | G | T | -0.011 | 0.003 | 1.57E-03 | -0.017 | 0.003 | 2.00E-09 | TV | -0.032 | 0.009 | -0.038 | 0.012 | -0.011 | 0.027 | -0.024 | 0.023 | -0.092 | 0.039 | -0.064 | 0.023 |
| rs12462975 | 19 | 30272202 | A | G | 0.020 | 0.003 | 2.63E-16 | 0.003 | 0.002 | 2.40E-01 | BMI | 0.020 | 0.006 | 0.011 | 0.008 | 0.045 | 0.019 | 0.037 | 0.016 | 0.022 | 0.027 | 0.054 | 0.016 |
| rs11667280 | 19 | 30705747 | C | G | 0.016 | 0.003 | 3.14E-08 | 0.007 | 0.003 | 9.10E-03 | BMI | 0.010 | 0.008 | 0.021 | 0.011 | -0.005 | 0.023 | 0.005 | 0.020 | 0.034 | 0.034 | 0.020 | 0.020 |
| rs7250833 | 19 | 33937277 | T | C | 0.015 | 0.002 | 7.22E-10 | 0.001 | 0.002 | 7.40E-01 | BMI | -0.010 | 0.007 | -0.018 | 0.009 | 0.006 | 0.020 | -0.014 | 0.017 | -0.012 | 0.028 | -0.015 | 0.017 |
| rs185350 | 19 | 34306816 | T | C | 0.013 | 0.002 | 7.14E-09 | 0.004 | 0.002 | 4.40E-02 | BMI | -0.012 | 0.006 | -0.011 | 0.008 | -0.011 | 0.018 | -0.004 | 0.015 | -0.012 | 0.026 | -0.017 | 0.015 |
| rs429358 | 19 | 45411941 | T | C | 0.028 | 0.004 | 3.17E-14 | 0.014 | 0.003 | 3.80E-06 | BMI | 0.015 | 0.009 | 0.017 | 0.012 | 0.002 | 0.026 | 0.000 | 0.022 | 0.064 | 0.038 | 0.024 | 0.022 |
| rs11672660 | 19 | 46180184 | T | C | -0.032 | 0.003 | 5.93E-31 | -0.004 | 0.003 | 1.40E-01 | BMI | 0.045 | 0.008 | 0.050 | 0.010 | 0.038 | 0.022 | 0.060 | 0.019 | 0.051 | 0.032 | 0.021 | 0.019 |
| rs9304665 | 19 | 47602577 | A | T | 0.020 | 0.003 | 1.84E-14 | -0.006 | 0.003 | 1.90E-02 | BMI | -0.019 | 0.007 | -0.015 | 0.009 | -0.008 | 0.021 | -0.014 | 0.018 | 0.005 | 0.030 | -0.005 | 0.018 |
| rs1884389 | 20 | 1410582 | T | C | -0.014 | 0.002 | 1.20E-09 | -0.001 | 0.002 | 5.80E-01 | BMI | 0.010 | 0.006 | 0.015 | 0.008 | 0.020 | 0.018 | 0.007 | 0.015 | 0.011 | 0.026 | 0.002 | 0.015 |
| rs1884897 | 20 | 6612832 | A | G | -0.017 | 0.002 | 9.06E-14 | 0.000 | 0.002 | 8.80E-01 | BMI | 0.005 | 0.006 | -0.003 | 0.008 | 0.015 | 0.018 | -0.004 | 0.016 | 0.001 | 0.027 | 0.048 | 0.016 |
| rs6131281 | 20 | 11891724 | C | T | 0.009 | 0.003 | 6.80E-04 | 0.016 | 0.002 | 3.10E-13 | TV | 0.002 | 0.007 | -0.006 | 0.009 | -0.007 | 0.019 | 0.008 | 0.016 | 0.016 | 0.027 | 0.010 | 0.016 |
| rs6141814 | 20 | 31368960 | C | A | 0.001 | 0.003 | 7.05E-01 | -0.014 | 0.002 | 1.30E-09 | TV | 0.004 | 0.006 | 0.005 | 0.008 | 0.029 | 0.018 | 0.000 | 0.015 | 0.006 | 0.026 | -0.006 | 0.016 |
| rs6142096 | 20 | 32686658 | A | G | 0.013 | 0.002 | 4.17E-09 | 0.001 | 0.002 | 7.30E-01 | BMI | -0.017 | 0.006 | -0.008 | 0.008 | -0.004 | 0.018 | -0.018 | 0.015 | 0.010 | 0.026 | -0.026 | 0.015 |
| rs55700114 | 20 | 43717080 | G | A | -0.003 | 0.003 | 3.02E-01 | -0.014 | 0.002 | 2.10E-09 | TV | -0.002 | 0.006 | -0.007 | 0.009 | 0.035 | 0.019 | -0.019 | 0.016 | 0.018 | 0.027 | -0.008 | 0.016 |
| rs17806224 | 20 | 51065854 | A | G | -0.029 | 0.003 | 3.52E-22 | -0.009 | 0.003 | 7.70E-04 | BMI | -0.019 | 0.008 | -0.005 | 0.011 | -0.039 | 0.024 | -0.020 | 0.020 | -0.071 | 0.036 | -0.055 | 0.021 |
| rs56103247 | 20 | 62483184 | C | T | 0.020 | 0.006 | 7.71E-04 | 0.030 | 0.005 | 3.80E-10 | TV | -0.006 | 0.015 | -0.007 | 0.020 | 0.020 | 0.045 | -0.013 | 0.037 | -0.068 | 0.061 | 0.014 | 0.038 |
| rs2836754 | 21 | 40291740 | T | C | -0.015 | 0.002 | 3.73E-11 | -0.003 | 0.002 | 2.60E-01 | BMI | 0.002 | 0.006 | 0.003 | 0.008 | 0.009 | 0.018 | 0.006 | 0.015 | 0.000 | 0.026 | -0.001 | 0.016 |
| rs10427502 | 21 | 40654840 | G | A | 0.010 | 0.003 | 3.20E-04 | 0.014 | 0.002 | 8.60E-10 | TV | -0.012 | 0.006 | -0.013 | 0.008 | -0.003 | 0.018 | -0.021 | 0.016 | -0.067 | 0.027 | -0.014 | 0.016 |
| rs427943 | 21 | 46570896 | A | C | -0.018 | 0.002 | 1.37E-14 | -0.009 | 0.002 | 3.70E-05 | BMI | 0.002 | 0.006 | -0.001 | 0.008 | 0.010 | 0.018 | 0.031 | 0.015 | 0.029 | 0.026 | 0.020 | 0.015 |
| rs2092829 | 22 | 31780514 | G | A | -0.007 | 0.003 | 4.45E-03 | 0.014 | 0.002 | 1.30E-08 | TV | -0.002 | 0.007 | -0.010 | 0.009 | -0.005 | 0.019 | -0.014 | 0.017 | -0.001 | 0.028 | 0.010 | 0.017 |
| rs28489620 | 22 | 41804716 | A | G | -0.018 | 0.003 | 6.90E-10 | -0.012 | 0.002 | 1.30E-06 | BMI | 0.021 | 0.007 | 0.019 | 0.009 | 0.029 | 0.019 | 0.026 | 0.017 | 0.002 | 0.028 | 0.022 | 0.017 |
| rs13053342 | 22 | 48871624 | A | G | 0.015 | 0.002 | 1.32E-10 | 0.006 | 0.002 | 5.90E-03 | BMI | 0.000 | 0.006 | 0.000 | 0.009 | -0.047 | 0.019 | 0.004 | 0.016 | -0.012 | 0.027 | 0.012 | 0.016 |

| **Table S17. Beta estimates for the SNPs included in the multivariable MR of BMI, leisure television watching, and colorectal cancer risk** | | | | | | | | | | | | | | | | | |
| --- | --- | --- | --- | --- | --- | --- | --- | --- | --- | --- | --- | --- | --- | --- | --- | --- | --- |
| **1) Analysis of colorectal cancer in men and women combined** | | | | | | | | | | | | | | | | | |
| SNP | CHR | POS | EA | OA | beta_bmi | se_bmi | pval_bmi | beta_tv | se_tv | pval_tv | phenotype | beta_crc | se_crc | beta_co | se_co | beta_re | se_re |
| rs79113395 | 1 | 1590521 | A | G | -0.020 | 0.002 | 2.03E-20 | -0.007 | 0.002 | 4.50E-03 | BMI | 0.003 | 0.014 | -0.001 | 0.017 | -0.020 | 0.021 |
| rs10909880 | 1 | 2727804 | T | C | -0.014 | 0.002 | 2.21E-16 | 0.000 | 0.002 | 9.30E-01 | BMI | -0.024 | 0.010 | -0.019 | 0.012 | -0.032 | 0.015 |
| rs11121210 | 1 | 8708529 | T | C | -0.011 | 0.002 | 2.37E-10 | -0.003 | 0.002 | 2.40E-01 | BMI | -0.004 | 0.010 | -0.005 | 0.012 | -0.005 | 0.016 |
| rs10779751 | 1 | 11284336 | A | G | 0.013 | 0.002 | 2.66E-13 | 0.001 | 0.002 | 5.50E-01 | BMI | -0.002 | 0.011 | 0.006 | 0.013 | -0.019 | 0.017 |
| rs61740466 | 1 | 19934900 | A | G | -0.014 | 0.002 | 2.24E-10 | -0.003 | 0.003 | 2.10E-01 | BMI | -0.010 | 0.011 | -0.028 | 0.013 | 0.010 | 0.017 |
| rs4655141 | 1 | 23312025 | T | C | -0.017 | 0.002 | 6.20E-15 | 0.000 | 0.003 | 9.60E-01 | BMI | -0.027 | 0.013 | -0.035 | 0.015 | -0.043 | 0.020 |
| rs2228552 | 1 | 32165495 | T | G | 0.012 | 0.002 | 1.80E-11 | 0.006 | 0.002 | 4.00E-03 | BMI | -0.008 | 0.010 | -0.011 | 0.012 | -0.015 | 0.016 |
| rs4653017 | 1 | 33776728 | T | C | 0.012 | 0.002 | 1.11E-10 | -0.001 | 0.002 | 7.30E-01 | BMI | -0.003 | 0.010 | -0.002 | 0.012 | -0.009 | 0.016 |
| rs7512146 | 1 | 34283008 | T | G | -0.010 | 0.002 | 1.29E-08 | 0.001 | 0.002 | 6.80E-01 | BMI | -0.012 | 0.010 | -0.013 | 0.012 | -0.012 | 0.015 |
| rs11577094 | 1 | 38026600 | T | C | 0.019 | 0.003 | 3.28E-10 | 0.008 | 0.004 | 2.90E-02 | BMI | -0.038 | 0.019 | -0.021 | 0.022 | -0.051 | 0.029 |
| rs11810109 | 1 | 44186812 | A | T | 0.001 | 0.002 | 6.53E-01 | 0.016 | 0.002 | 5.40E-12 | TV | 0.011 | 0.011 | 0.004 | 0.013 | 0.016 | 0.017 |
| rs2984618 | 1 | 47690438 | T | G | 0.017 | 0.002 | 3.80E-24 | 0.001 | 0.002 | 6.40E-01 | BMI | -0.019 | 0.010 | -0.022 | 0.012 | -0.037 | 0.015 |
| rs657452 | 1 | 49589847 | A | G | 0.019 | 0.002 | 3.17E-30 | -0.004 | 0.002 | 7.00E-02 | BMI | 0.015 | 0.010 | 0.015 | 0.012 | 0.010 | 0.016 |
| rs587271 | 1 | 54743111 | T | C | 0.012 | 0.002 | 6.21E-11 | -0.004 | 0.002 | 1.00E-01 | BMI | -0.002 | 0.011 | 0.007 | 0.013 | -0.024 | 0.017 |
| rs12725114 | 1 | 62432641 | G | A | 0.012 | 0.002 | 3.32E-07 | 0.015 | 0.003 | 1.60E-08 | TV | -0.016 | 0.012 | 0.003 | 0.014 | -0.060 | 0.018 |
| rs984409 | 1 | 67020440 | G | A | -0.005 | 0.002 | 1.05E-02 | -0.015 | 0.002 | 4.10E-11 | TV | -0.019 | 0.010 | -0.011 | 0.012 | -0.030 | 0.016 |
| rs71658797 | 1 | 77967507 | T | A | -0.037 | 0.003 | 8.42E-36 | -0.020 | 0.003 | 6.30E-10 | TV | -0.005 | 0.016 | -0.009 | 0.019 | -0.009 | 0.025 |
| rs6696828 | 1 | 80812020 | C | G | 0.012 | 0.002 | 6.42E-11 | 0.002 | 0.002 | 3.60E-01 | BMI | 0.003 | 0.011 | 0.004 | 0.013 | -0.002 | 0.017 |
| rs114328297 | 1 | 91190854 | T | G | -0.001 | 0.002 | 6.96E-01 | 0.014 | 0.003 | 4.40E-08 | TV | 0.002 | 0.012 | 0.016 | 0.015 | -0.056 | 0.019 |
| rs17379561 | 1 | 98340139 | A | T | -0.018 | 0.002 | 5.04E-13 | -0.025 | 0.003 | 1.10E-16 | TV | 0.016 | 0.014 | 0.016 | 0.017 | 0.016 | 0.022 |
| rs55909997 | 1 | 107581039 | G | A | -0.012 | 0.002 | 1.55E-11 | -0.014 | 0.002 | 2.40E-09 | TV | -0.027 | 0.010 | -0.011 | 0.012 | -0.033 | 0.016 |
| rs1278847 | 1 | 110037483 | C | A | -0.011 | 0.002 | 9.45E-08 | 0.016 | 0.002 | 1.20E-11 | TV | -0.006 | 0.011 | -0.006 | 0.013 | -0.004 | 0.017 |
| rs74887628 | 1 | 147032779 | A | G | 0.031 | 0.005 | 1.76E-08 | 0.004 | 0.006 | 5.20E-01 | BMI | -0.003 | 0.030 | -0.004 | 0.036 | -0.014 | 0.047 |
| rs4970991 | 1 | 151004003 | T | C | 0.012 | 0.002 | 1.81E-08 | 0.008 | 0.003 | 2.80E-03 | BMI | 0.015 | 0.012 | 0.013 | 0.014 | 0.010 | 0.019 |
| rs4845364 | 1 | 154141908 | A | G | -0.001 | 0.002 | 6.38E-01 | -0.015 | 0.002 | 1.30E-12 | TV | -0.006 | 0.010 | -0.008 | 0.012 | -0.013 | 0.015 |
| rs61813324 | 1 | 156049877 | T | C | 0.029 | 0.003 | 3.20E-24 | 0.015 | 0.003 | 4.30E-06 | BMI | 0.047 | 0.017 | 0.045 | 0.020 | 0.047 | 0.027 |
| rs10733051 | 1 | 167280354 | A | G | 0.009 | 0.002 | 6.96E-09 | 0.003 | 0.002 | 2.40E-01 | BMI | 0.027 | 0.010 | 0.029 | 0.012 | 0.022 | 0.015 |
| rs61828641 | 1 | 174321997 | A | G | 0.022 | 0.003 | 2.49E-13 | -0.008 | 0.003 | 1.50E-02 | BMI | -0.006 | 0.015 | 0.005 | 0.018 | -0.011 | 0.024 |
| rs6673341 | 1 | 184655649 | T | G | -0.008 | 0.002 | 4.45E-06 | -0.015 | 0.002 | 2.20E-11 | TV | 0.000 | 0.010 | 0.002 | 0.012 | -0.014 | 0.015 |
| rs10737620 | 1 | 193057971 | T | A | 0.010 | 0.002 | 8.36E-08 | 0.014 | 0.002 | 2.60E-09 | TV | -0.010 | 0.011 | 0.000 | 0.013 | -0.024 | 0.017 |
| rs2400414 | 1 | 194965200 | T | C | -0.013 | 0.002 | 5.52E-13 | -0.003 | 0.002 | 2.50E-01 | BMI | -0.028 | 0.010 | -0.028 | 0.012 | -0.033 | 0.016 |
| rs2820295 | 1 | 201800868 | A | G | 0.024 | 0.002 | 5.56E-39 | 0.010 | 0.002 | 8.70E-06 | BMI | 0.027 | 0.010 | 0.034 | 0.012 | 0.005 | 0.016 |
| rs10920336 | 1 | 202115945 | A | G | -0.010 | 0.002 | 4.20E-09 | -0.002 | 0.002 | 3.10E-01 | BMI | -0.025 | 0.010 | -0.027 | 0.012 | -0.005 | 0.015 |
| rs1006317 | 1 | 209552636 | T | G | 0.016 | 0.003 | 3.75E-10 | 0.001 | 0.003 | 6.90E-01 | BMI | -0.034 | 0.014 | -0.021 | 0.017 | -0.039 | 0.023 |
| rs6661316 | 1 | 210095527 | T | C | 0.012 | 0.002 | 1.72E-13 | 0.006 | 0.002 | 7.20E-03 | BMI | 0.009 | 0.010 | 0.013 | 0.012 | 0.013 | 0.015 |
| rs10864728 | 1 | 230304914 | A | G | 0.011 | 0.002 | 1.20E-08 | 0.007 | 0.002 | 3.10E-03 | BMI | 0.001 | 0.010 | -0.009 | 0.012 | 0.008 | 0.015 |
| rs946824 | 1 | 243684019 | T | C | 0.020 | 0.003 | 4.82E-15 | 0.010 | 0.003 | 2.60E-03 | BMI | 0.004 | 0.014 | 0.005 | 0.017 | 0.022 | 0.022 |
| rs6710091 | 2 | 239597 | C | G | 0.010 | 0.002 | 5.78E-09 | 0.005 | 0.002 | 4.50E-02 | BMI | 0.000 | 0.010 | 0.006 | 0.012 | -0.002 | 0.016 |
| rs77165542 | 2 | 430975 | T | C | -0.094 | 0.005 | 1.51E-70 | 0.003 | 0.006 | 5.90E-01 | BMI | -0.058 | 0.030 | -0.060 | 0.036 | -0.026 | 0.047 |
| rs13021737 | 2 | 632348 | A | G | -0.058 | 0.002 | 2.89E-161 | -0.004 | 0.003 | 1.40E-01 | BMI | -0.018 | 0.013 | -0.024 | 0.015 | -0.024 | 0.020 |
| rs10929925 | 2 | 6155557 | A | C | -0.014 | 0.002 | 3.05E-18 | -0.004 | 0.002 | 7.70E-02 | BMI | -0.010 | 0.010 | -0.012 | 0.012 | 0.006 | 0.015 |
| rs11902450 | 2 | 12845368 | T | C | 0.017 | 0.003 | 1.06E-09 | 0.012 | 0.004 | 5.30E-04 | BMI | 0.000 | 0.016 | 0.004 | 0.019 | 0.007 | 0.025 |
| rs72781699 | 2 | 24277709 | G | A | 0.000 | 0.002 | 9.01E-01 | -0.019 | 0.003 | 3.00E-12 | TV | 0.009 | 0.012 | -0.012 | 0.014 | 0.042 | 0.019 |
| rs935166 | 2 | 26949366 | A | G | -0.015 | 0.002 | 8.69E-16 | -0.009 | 0.002 | 8.10E-05 | BMI | -0.023 | 0.010 | -0.026 | 0.012 | -0.027 | 0.015 |
| rs3770890 | 2 | 36657992 | T | G | -0.030 | 0.005 | 2.10E-08 | -0.002 | 0.007 | 8.00E-01 | BMI | 0.026 | 0.035 | -0.007 | 0.043 | 0.099 | 0.059 |
| rs10185199 | 2 | 40282202 | A | G | -0.014 | 0.002 | 3.18E-13 | -0.006 | 0.002 | 1.00E-02 | BMI | 0.009 | 0.011 | 0.008 | 0.013 | 0.006 | 0.017 |
| rs12105701 | 2 | 41737200 | C | T | -0.010 | 0.002 | 3.12E-08 | -0.013 | 0.002 | 5.40E-09 | TV | -0.009 | 0.010 | -0.007 | 0.012 | -0.017 | 0.015 |
| rs7561278 | 2 | 48954905 | T | C | 0.017 | 0.002 | 4.89E-16 | 0.003 | 0.003 | 1.90E-01 | BMI | -0.015 | 0.012 | -0.024 | 0.014 | -0.032 | 0.019 |
| rs930295 | 2 | 50233352 | A | C | 0.021 | 0.002 | 2.03E-19 | 0.006 | 0.003 | 5.90E-02 | BMI | 0.000 | 0.013 | 0.007 | 0.016 | -0.014 | 0.021 |
| rs7564130 | 2 | 50606642 | T | C | -0.002 | 0.002 | 2.62E-01 | -0.015 | 0.002 | 2.80E-11 | TV | -0.021 | 0.010 | -0.032 | 0.012 | -0.002 | 0.016 |
| rs805412 | 2 | 54120820 | A | G | -0.010 | 0.002 | 1.14E-08 | 0.008 | 0.002 | 3.60E-04 | BMI | 0.014 | 0.010 | 0.009 | 0.012 | 0.026 | 0.015 |
| rs4671328 | 2 | 58935282 | T | G | 0.021 | 0.002 | 3.42E-36 | 0.010 | 0.002 | 4.60E-06 | BMI | -0.007 | 0.010 | -0.007 | 0.012 | 0.013 | 0.015 |
| rs10189857 | 2 | 60713235 | A | G | -0.007 | 0.002 | 3.23E-05 | -0.020 | 0.002 | 6.20E-21 | TV | -0.022 | 0.010 | -0.020 | 0.012 | -0.019 | 0.015 |
| rs62641636 | 2 | 68425427 | A | G | 0.006 | 0.002 | 5.59E-03 | 0.014 | 0.002 | 7.00E-10 | TV | 0.010 | 0.010 | 0.022 | 0.012 | 0.015 | 0.016 |
| rs17020497 | 2 | 81826131 | A | G | 0.014 | 0.003 | 2.04E-08 | 0.008 | 0.003 | 7.90E-03 | BMI | 0.008 | 0.014 | 0.018 | 0.017 | 0.014 | 0.022 |
| rs12714199 | 2 | 86812549 | T | C | -0.014 | 0.002 | 3.22E-16 | -0.002 | 0.002 | 4.80E-01 | BMI | 0.000 | 0.010 | 0.009 | 0.012 | -0.003 | 0.016 |
| rs11689199 | 2 | 100820421 | A | G | 0.017 | 0.002 | 1.85E-22 | 0.018 | 0.002 | 5.50E-17 | TV | 0.025 | 0.010 | 0.020 | 0.012 | 0.034 | 0.016 |
| rs264941 | 2 | 104297420 | A | C | -0.012 | 0.002 | 1.84E-13 | -0.012 | 0.002 | 9.30E-09 | BMI | -0.004 | 0.010 | -0.010 | 0.012 | -0.018 | 0.015 |
| rs1451533 | 2 | 105466005 | G | A | -0.016 | 0.002 | 3.03E-17 | -0.016 | 0.002 | 1.10E-10 | TV | 0.009 | 0.011 | 0.002 | 0.013 | 0.013 | 0.017 |
| rs12476388 | 2 | 116330983 | C | T | 0.002 | 0.002 | 2.37E-01 | 0.013 | 0.002 | 2.10E-08 | TV | -0.002 | 0.010 | -0.012 | 0.013 | 0.001 | 0.017 |
| rs13033310 | 2 | 133523605 | A | G | 0.015 | 0.002 | 3.40E-11 | 0.004 | 0.002 | 1.50E-01 | BMI | 0.001 | 0.011 | 0.005 | 0.013 | -0.002 | 0.017 |
| rs4988235 | 2 | 136608646 | A | G | 0.012 | 0.002 | 7.09E-13 | 0.007 | 0.003 | 5.80E-03 | BMI | -0.043 | 0.011 | -0.049 | 0.013 | -0.054 | 0.017 |
| rs17814208 | 2 | 144037998 | A | G | -0.013 | 0.002 | 7.24E-11 | -0.007 | 0.003 | 5.90E-03 | BMI | -0.003 | 0.011 | 0.003 | 0.013 | -0.006 | 0.017 |
| rs374722 | 2 | 147839830 | G | A | 0.014 | 0.002 | 9.78E-10 | 0.024 | 0.003 | 5.50E-16 | TV | 0.026 | 0.013 | 0.038 | 0.016 | 0.017 | 0.021 |
| rs2119753 | 2 | 151224579 | A | G | 0.010 | 0.002 | 1.00E-08 | 0.005 | 0.002 | 3.80E-02 | BMI | 0.001 | 0.010 | 0.000 | 0.012 | 0.008 | 0.016 |
| rs11695013 | 2 | 157057487 | T | C | -0.010 | 0.002 | 2.43E-09 | 0.000 | 0.002 | 8.80E-01 | BMI | 0.007 | 0.010 | 0.007 | 0.012 | 0.003 | 0.016 |
| rs3764835 | 2 | 159519368 | A | G | -0.013 | 0.002 | 3.84E-08 | -0.006 | 0.003 | 5.00E-02 | BMI | -0.003 | 0.013 | 0.003 | 0.016 | 0.000 | 0.021 |
| rs3754970 | 2 | 162091836 | T | C | -0.005 | 0.002 | 1.37E-02 | -0.015 | 0.002 | 4.80E-12 | TV | 0.006 | 0.010 | 0.011 | 0.012 | -0.011 | 0.015 |
| rs2060199 | 2 | 166196681 | T | A | 0.005 | 0.002 | 4.69E-03 | 0.012 | 0.002 | 2.40E-08 | TV | 0.016 | 0.010 | 0.012 | 0.012 | 0.028 | 0.015 |
| rs34234296 | 2 | 175166636 | A | G | -0.015 | 0.002 | 2.01E-13 | -0.004 | 0.002 | 1.10E-01 | BMI | -0.011 | 0.010 | 0.003 | 0.012 | -0.032 | 0.016 |
| rs7588437 | 2 | 181575281 | A | G | -0.017 | 0.002 | 2.32E-22 | -0.006 | 0.002 | 1.40E-02 | BMI | -0.002 | 0.010 | -0.009 | 0.012 | 0.008 | 0.016 |
| rs4577309 | 2 | 191288833 | A | G | 0.002 | 0.002 | 2.74E-01 | 0.016 | 0.002 | 1.60E-13 | TV | 0.001 | 0.010 | 0.004 | 0.012 | 0.009 | 0.015 |
| rs34224261 | 2 | 193855452 | G | C | -0.010 | 0.002 | 4.43E-07 | -0.018 | 0.002 | 4.60E-16 | TV | 0.001 | 0.010 | -0.002 | 0.012 | 0.000 | 0.015 |
| rs4675246 | 2 | 202864487 | G | T | 0.002 | 0.002 | 4.43E-01 | -0.015 | 0.003 | 2.90E-08 | TV | 0.037 | 0.013 | 0.028 | 0.015 | 0.047 | 0.020 |
| rs13029509 | 2 | 215374209 | G | A | -0.007 | 0.002 | 7.53E-05 | -0.018 | 0.002 | 2.00E-17 | TV | -0.005 | 0.010 | -0.001 | 0.012 | -0.011 | 0.015 |
| rs10932837 | 2 | 221044785 | C | T | 0.002 | 0.002 | 2.98E-01 | -0.013 | 0.002 | 1.30E-09 | TV | -0.003 | 0.010 | -0.005 | 0.012 | -0.016 | 0.015 |
| rs6720868 | 2 | 230663576 | T | C | 0.015 | 0.002 | 1.82E-17 | 0.006 | 0.002 | 1.10E-02 | BMI | -0.003 | 0.010 | -0.005 | 0.012 | 0.003 | 0.016 |
| rs4973576 | 2 | 233806771 | C | A | 0.002 | 0.002 | 2.55E-01 | -0.015 | 0.002 | 1.00E-09 | TV | 0.013 | 0.011 | 0.013 | 0.013 | -0.001 | 0.017 |
| rs7568228 | 2 | 236848488 | C | G | -0.010 | 0.002 | 1.00E-09 | -0.006 | 0.002 | 7.00E-03 | BMI | -0.018 | 0.010 | -0.026 | 0.012 | 0.006 | 0.015 |
| rs59302296 | 3 | 9507314 | A | T | 0.022 | 0.003 | 9.12E-12 | 0.007 | 0.004 | 5.30E-02 | BMI | -0.006 | 0.016 | -0.010 | 0.019 | -0.003 | 0.025 |
| rs138256022 | 3 | 11603090 | C | T | 0.002 | 0.005 | 6.40E-01 | -0.031 | 0.006 | 2.90E-08 | TV | 0.043 | 0.031 | 0.031 | 0.037 | 0.046 | 0.049 |
| rs10510419 | 3 | 12426936 | T | G | -0.017 | 0.002 | 2.23E-13 | -0.006 | 0.003 | 3.70E-02 | BMI | 0.037 | 0.014 | 0.034 | 0.016 | 0.047 | 0.021 |
| rs1048637 | 3 | 13358171 | T | G | -0.009 | 0.002 | 2.94E-08 | 0.003 | 0.002 | 1.80E-01 | BMI | 0.018 | 0.010 | 0.014 | 0.012 | 0.023 | 0.015 |
| rs4858193 | 3 | 20441050 | T | C | 0.013 | 0.002 | 2.44E-12 | 0.001 | 0.002 | 5.90E-01 | BMI | 0.002 | 0.011 | -0.011 | 0.013 | -0.006 | 0.017 |
| rs6804842 | 3 | 25106437 | A | G | -0.014 | 0.002 | 7.57E-18 | -0.001 | 0.002 | 5.60E-01 | BMI | 0.008 | 0.010 | 0.011 | 0.012 | -0.007 | 0.015 |
| rs11921432 | 3 | 35117776 | T | C | -0.019 | 0.003 | 5.38E-12 | 0.000 | 0.003 | 9.60E-01 | BMI | -0.008 | 0.016 | -0.018 | 0.018 | 0.006 | 0.024 |
| rs9834970 | 3 | 36856030 | T | C | 0.007 | 0.002 | 1.23E-04 | 0.013 | 0.002 | 3.30E-09 | TV | 0.013 | 0.010 | 0.010 | 0.012 | 0.017 | 0.015 |
| rs4017425 | 3 | 44028764 | T | C | -0.012 | 0.002 | 2.91E-12 | -0.008 | 0.002 | 2.50E-04 | BMI | 0.015 | 0.010 | 0.021 | 0.012 | 0.014 | 0.015 |
| rs3796386 | 3 | 49899795 | G | A | -0.018 | 0.002 | 1.11E-25 | -0.026 | 0.002 | 3.20E-33 | TV | -0.015 | 0.010 | -0.012 | 0.012 | -0.040 | 0.015 |
| rs11130793 | 3 | 60884659 | C | T | 0.008 | 0.002 | 4.42E-05 | 0.013 | 0.002 | 5.00E-09 | TV | 0.008 | 0.010 | -0.004 | 0.012 | 0.011 | 0.016 |
| rs11714337 | 3 | 71582521 | G | A | 0.007 | 0.002 | 4.38E-04 | 0.014 | 0.002 | 4.70E-11 | TV | -0.021 | 0.010 | -0.029 | 0.012 | -0.011 | 0.015 |
| rs12636480 | 3 | 82719412 | T | G | 0.013 | 0.002 | 6.96E-13 | 0.001 | 0.002 | 6.80E-01 | BMI | 0.005 | 0.010 | 0.000 | 0.012 | 0.003 | 0.016 |
| rs9827823 | 3 | 84221774 | T | C | 0.018 | 0.002 | 3.39E-15 | 0.012 | 0.003 | 1.10E-04 | BMI | -0.004 | 0.014 | -0.016 | 0.016 | 0.003 | 0.021 |
| rs6797840 | 3 | 85656569 | A | C | -0.013 | 0.002 | 1.02E-11 | -0.016 | 0.002 | 1.70E-13 | TV | 0.009 | 0.010 | 0.016 | 0.012 | 0.015 | 0.015 |
| rs2034768 | 3 | 93986371 | A | G | 0.020 | 0.002 | 2.09E-25 | 0.015 | 0.002 | 8.60E-12 | TV | -0.013 | 0.010 | -0.014 | 0.012 | -0.007 | 0.015 |
| rs1436344 | 3 | 104606144 | C | G | 0.015 | 0.002 | 1.07E-17 | 0.004 | 0.002 | 1.00E-01 | BMI | 0.011 | 0.010 | 0.016 | 0.012 | -0.004 | 0.015 |
| rs7640424 | 3 | 107820063 | T | C | -0.014 | 0.002 | 1.23E-14 | -0.006 | 0.002 | 1.20E-02 | BMI | -0.017 | 0.011 | -0.019 | 0.013 | -0.010 | 0.017 |
| rs9867121 | 3 | 114631548 | C | A | 0.008 | 0.002 | 1.28E-03 | 0.019 | 0.003 | 3.90E-12 | TV | 0.020 | 0.012 | 0.018 | 0.015 | 0.014 | 0.019 |
| rs6808814 | 3 | 116852469 | T | C | 0.012 | 0.002 | 1.31E-09 | 0.006 | 0.002 | 2.00E-02 | BMI | -0.004 | 0.011 | -0.004 | 0.013 | 0.004 | 0.017 |
| rs7631156 | 3 | 131751628 | A | G | 0.022 | 0.002 | 3.33E-32 | 0.002 | 0.002 | 5.10E-01 | BMI | 0.001 | 0.010 | -0.010 | 0.012 | 0.032 | 0.016 |
| rs687339 | 3 | 135932359 | T | C | 0.019 | 0.002 | 4.32E-22 | 0.004 | 0.003 | 1.10E-01 | BMI | 0.014 | 0.011 | 0.015 | 0.014 | 0.007 | 0.018 |
| rs16851483 | 3 | 141275436 | T | G | 0.035 | 0.003 | 4.87E-25 | 0.013 | 0.004 | 2.90E-03 | BMI | -0.003 | 0.018 | 0.005 | 0.023 | -0.001 | 0.031 |
| rs355777 | 3 | 154034950 | C | G | 0.015 | 0.002 | 2.13E-18 | 0.003 | 0.002 | 1.70E-01 | BMI | 0.001 | 0.010 | -0.005 | 0.012 | 0.012 | 0.015 |
| rs12491503 | 3 | 165706855 | G | A | -0.004 | 0.002 | 3.48E-02 | -0.014 | 0.002 | 5.50E-10 | TV | -0.001 | 0.010 | -0.005 | 0.012 | -0.006 | 0.016 |
| rs12635553 | 3 | 171113714 | A | T | 0.010 | 0.002 | 4.10E-09 | 0.001 | 0.002 | 5.00E-01 | BMI | -0.005 | 0.010 | -0.012 | 0.012 | 0.016 | 0.015 |
| rs39654 | 3 | 173095123 | A | G | -0.016 | 0.002 | 1.75E-21 | -0.005 | 0.002 | 2.50E-02 | BMI | 0.001 | 0.010 | 0.002 | 0.012 | -0.012 | 0.015 |
| rs114600294 | 3 | 181419367 | G | C | -0.006 | 0.002 | 1.31E-02 | -0.016 | 0.003 | 7.90E-10 | TV | 0.013 | 0.014 | 0.021 | 0.017 | 0.007 | 0.022 |
| rs9816226 | 3 | 185834499 | A | T | -0.032 | 0.002 | 1.45E-50 | 0.007 | 0.003 | 8.70E-03 | BMI | 0.020 | 0.013 | 0.020 | 0.015 | 0.001 | 0.020 |
| rs7616009 | 3 | 194881756 | A | G | -0.016 | 0.002 | 4.33E-11 | -0.008 | 0.003 | 5.60E-03 | BMI | 0.000 | 0.013 | 0.003 | 0.017 | -0.019 | 0.022 |
| rs6803161 | 3 | 196205694 | T | C | 0.011 | 0.002 | 2.71E-08 | 0.006 | 0.002 | 4.20E-03 | BMI | 0.003 | 0.010 | 0.012 | 0.012 | -0.005 | 0.016 |
| rs66852340 | 4 | 3311070 | C | T | -0.009 | 0.002 | 9.47E-06 | -0.018 | 0.003 | 7.90E-12 | TV | -0.004 | 0.011 | 0.000 | 0.014 | -0.030 | 0.018 |
| rs7700107 | 4 | 17880416 | A | C | -0.010 | 0.003 | 2.46E-05 | -0.021 | 0.003 | 1.90E-11 | TV | -0.003 | 0.014 | 0.018 | 0.017 | -0.011 | 0.022 |
| rs994596 | 4 | 18459828 | T | C | 0.013 | 0.002 | 1.06E-12 | 0.001 | 0.002 | 5.30E-01 | BMI | 0.006 | 0.010 | 0.007 | 0.012 | 0.014 | 0.016 |
| rs73213484 | 4 | 28489339 | A | T | 0.021 | 0.003 | 2.04E-14 | 0.002 | 0.003 | 4.40E-01 | BMI | 0.008 | 0.013 | 0.014 | 0.016 | 0.036 | 0.021 |
| rs337637 | 4 | 38604470 | A | G | -0.014 | 0.002 | 6.24E-16 | -0.002 | 0.002 | 3.40E-01 | BMI | -0.008 | 0.010 | -0.009 | 0.012 | -0.013 | 0.016 |
| rs10938397 | 4 | 45182527 | A | G | -0.032 | 0.002 | 2.42E-86 | -0.011 | 0.002 | 9.30E-07 | BMI | 0.000 | 0.010 | -0.006 | 0.012 | 0.000 | 0.015 |
| rs28971796 | 4 | 49151982 | A | G | 0.011 | 0.002 | 1.02E-07 | 0.015 | 0.002 | 2.10E-09 | TV | 0.010 | 0.012 | 0.003 | 0.015 | 0.037 | 0.019 |
| rs1492767 | 4 | 55221467 | T | C | 0.010 | 0.002 | 3.55E-09 | 0.004 | 0.002 | 7.90E-02 | BMI | 0.000 | 0.010 | -0.011 | 0.012 | 0.022 | 0.015 |
| rs1346841 | 4 | 65651730 | A | G | -0.013 | 0.002 | 3.18E-13 | -0.001 | 0.002 | 5.70E-01 | BMI | -0.004 | 0.010 | 0.000 | 0.012 | 0.020 | 0.015 |
| rs10002111 | 4 | 67815504 | A | G | 0.013 | 0.002 | 1.26E-09 | 0.007 | 0.003 | 4.30E-03 | BMI | 0.000 | 0.012 | 0.006 | 0.014 | -0.007 | 0.018 |
| rs6850494 | 4 | 82291771 | A | C | 0.001 | 0.002 | 5.16E-01 | -0.014 | 0.002 | 1.10E-10 | TV | -0.011 | 0.010 | -0.029 | 0.012 | -0.008 | 0.016 |
| rs4148155 | 4 | 89054667 | A | G | 0.019 | 0.003 | 1.34E-13 | 0.009 | 0.003 | 6.60E-03 | BMI | 0.004 | 0.015 | 0.014 | 0.019 | 0.018 | 0.025 |
| rs4286488 | 4 | 94440026 | A | G | 0.012 | 0.002 | 1.70E-09 | 0.010 | 0.003 | 1.20E-04 | BMI | 0.018 | 0.011 | 0.022 | 0.014 | 0.036 | 0.018 |
| rs7678054 | 4 | 95093855 | A | G | -0.010 | 0.002 | 4.57E-09 | -0.002 | 0.002 | 2.50E-01 | BMI | -0.025 | 0.010 | -0.025 | 0.012 | -0.028 | 0.015 |
| rs3796432 | 4 | 96030402 | T | G | -0.011 | 0.002 | 2.21E-10 | 0.000 | 0.002 | 9.30E-01 | BMI | 0.000 | 0.010 | 0.002 | 0.012 | -0.011 | 0.016 |
| rs13107325 | 4 | 103188709 | T | C | 0.047 | 0.003 | 3.81E-47 | 0.029 | 0.004 | 1.50E-12 | both | -0.008 | 0.019 | -0.012 | 0.022 | 0.006 | 0.029 |
| rs7693703 | 4 | 118357405 | G | A | 0.005 | 0.003 | 9.29E-02 | 0.023 | 0.004 | 2.70E-09 | TV | 0.022 | 0.017 | 0.018 | 0.020 | 0.070 | 0.027 |
| rs4334769 | 4 | 130245233 | G | T | 0.002 | 0.002 | 2.03E-01 | 0.012 | 0.002 | 2.10E-08 | TV | -0.015 | 0.010 | -0.023 | 0.012 | -0.015 | 0.015 |
| rs1296328 | 4 | 137083193 | A | C | 0.017 | 0.002 | 3.49E-22 | 0.006 | 0.002 | 1.00E-02 | BMI | -0.012 | 0.010 | -0.001 | 0.012 | -0.025 | 0.015 |
| rs17367750 | 4 | 140782542 | T | C | -0.012 | 0.002 | 1.97E-11 | -0.009 | 0.002 | 4.00E-05 | BMI | -0.013 | 0.010 | -0.018 | 0.012 | -0.019 | 0.016 |
| rs9992189 | 4 | 144060728 | C | G | -0.010 | 0.002 | 2.04E-08 | -0.002 | 0.002 | 4.70E-01 | BMI | 0.010 | 0.010 | 0.004 | 0.012 | 0.042 | 0.015 |
| rs3914628 | 4 | 147438019 | T | C | 0.017 | 0.002 | 6.91E-13 | 0.006 | 0.003 | 3.90E-02 | BMI | -0.010 | 0.013 | -0.002 | 0.016 | -0.006 | 0.021 |
| rs6825241 | 4 | 152543783 | C | A | -0.004 | 0.002 | 3.23E-02 | -0.017 | 0.002 | 4.90E-15 | TV | -0.008 | 0.010 | -0.005 | 0.012 | -0.016 | 0.015 |
| rs7693082 | 4 | 159857819 | G | C | 0.008 | 0.002 | 3.20E-04 | 0.015 | 0.002 | 1.70E-10 | TV | 0.017 | 0.011 | 0.026 | 0.013 | 0.020 | 0.017 |
| rs13110266 | 4 | 162129844 | A | G | -0.012 | 0.002 | 3.96E-14 | -0.006 | 0.002 | 7.90E-03 | BMI | -0.018 | 0.010 | -0.022 | 0.012 | -0.013 | 0.016 |
| rs7685628 | 4 | 165310133 | A | T | 0.010 | 0.002 | 6.15E-09 | 0.006 | 0.002 | 1.10E-02 | BMI | 0.027 | 0.010 | 0.021 | 0.012 | 0.042 | 0.015 |
| rs1522569 | 4 | 171632637 | T | G | 0.014 | 0.002 | 1.60E-10 | 0.004 | 0.003 | 1.10E-01 | BMI | 0.000 | 0.012 | 0.000 | 0.015 | -0.001 | 0.019 |
| rs1437842 | 4 | 173597016 | A | G | -0.011 | 0.002 | 8.49E-10 | -0.007 | 0.002 | 5.40E-04 | BMI | -0.011 | 0.010 | -0.020 | 0.012 | -0.008 | 0.015 |
| rs6850421 | 4 | 180187034 | A | G | 0.011 | 0.002 | 3.66E-09 | 0.000 | 0.002 | 9.20E-01 | BMI | 0.002 | 0.010 | 0.002 | 0.012 | -0.020 | 0.015 |
| rs35408866 | 4 | 187743245 | A | G | 0.016 | 0.003 | 1.50E-08 | 0.008 | 0.003 | 1.10E-02 | BMI | 0.004 | 0.015 | 0.015 | 0.017 | -0.003 | 0.023 |
| rs698147 | 5 | 3513485 | A | G | 0.012 | 0.002 | 9.67E-12 | -0.003 | 0.002 | 1.60E-01 | BMI | -0.003 | 0.010 | -0.011 | 0.012 | 0.001 | 0.015 |
| rs6890310 | 5 | 27193573 | A | G | -0.012 | 0.002 | 3.29E-10 | -0.003 | 0.002 | 1.90E-01 | BMI | 0.019 | 0.011 | 0.027 | 0.013 | 0.012 | 0.017 |
| rs7730004 | 5 | 43191033 | T | C | 0.014 | 0.002 | 1.46E-14 | 0.005 | 0.002 | 3.50E-02 | BMI | 0.024 | 0.010 | 0.021 | 0.012 | 0.043 | 0.016 |
| rs116374395 | 5 | 50723410 | A | G | 0.032 | 0.005 | 7.05E-10 | -0.004 | 0.006 | 5.50E-01 | BMI | -0.014 | 0.024 | -0.013 | 0.029 | 0.027 | 0.037 |
| rs150215901 | 5 | 50935903 | A | T | -0.028 | 0.005 | 1.54E-08 | -0.006 | 0.005 | 2.80E-01 | BMI | 0.013 | 0.026 | 0.004 | 0.031 | 0.035 | 0.040 |
| rs13186194 | 5 | 60795485 | T | C | 0.010 | 0.002 | 2.20E-09 | -0.015 | 0.002 | 2.10E-11 | BMI | 0.006 | 0.010 | 0.011 | 0.012 | 0.007 | 0.016 |
| rs262890 | 5 | 62930015 | A | G | -0.014 | 0.002 | 2.95E-11 | -0.019 | 0.002 | 3.20E-15 | TV | 0.031 | 0.011 | 0.028 | 0.013 | 0.043 | 0.017 |
| rs1159692 | 5 | 63977815 | A | C | 0.014 | 0.002 | 5.54E-15 | -0.003 | 0.002 | 2.20E-01 | BMI | 0.005 | 0.010 | 0.012 | 0.012 | -0.021 | 0.015 |
| rs2112347 | 5 | 75015242 | T | G | 0.028 | 0.002 | 1.17E-61 | 0.007 | 0.002 | 2.10E-03 | BMI | 0.010 | 0.010 | 0.005 | 0.012 | -0.002 | 0.016 |
| rs7716447 | 5 | 88800331 | A | G | -0.015 | 0.002 | 8.41E-17 | -0.013 | 0.002 | 3.60E-09 | TV | -0.016 | 0.010 | -0.007 | 0.012 | -0.021 | 0.016 |
| rs1031423 | 5 | 93276883 | T | C | 0.004 | 0.002 | 9.21E-02 | -0.019 | 0.003 | 1.80E-12 | TV | 0.020 | 0.012 | 0.022 | 0.014 | 0.017 | 0.019 |
| rs57585211 | 5 | 107197725 | T | G | -0.005 | 0.003 | 7.14E-02 | -0.017 | 0.003 | 4.30E-09 | TV | -0.003 | 0.013 | -0.008 | 0.016 | -0.009 | 0.021 |
| rs40067 | 5 | 107439012 | A | G | -0.025 | 0.002 | 9.73E-29 | -0.002 | 0.003 | 5.40E-01 | BMI | -0.008 | 0.013 | -0.008 | 0.015 | -0.014 | 0.020 |
| rs9326862 | 5 | 112054547 | A | T | 0.004 | 0.002 | 2.09E-02 | 0.012 | 0.002 | 2.60E-08 | TV | 0.001 | 0.010 | 0.006 | 0.012 | -0.010 | 0.015 |
| rs6893539 | 5 | 122705737 | A | C | -0.012 | 0.002 | 6.17E-11 | 0.004 | 0.002 | 6.50E-02 | BMI | -0.005 | 0.011 | 0.006 | 0.013 | -0.007 | 0.017 |
| rs10041724 | 5 | 124273520 | T | C | 0.006 | 0.002 | 2.64E-03 | 0.018 | 0.003 | 3.90E-11 | TV | 0.015 | 0.012 | 0.016 | 0.014 | 0.012 | 0.019 |
| rs62379379 | 5 | 141082015 | G | T | -0.005 | 0.004 | 1.61E-01 | -0.026 | 0.004 | 7.80E-10 | TV | -0.032 | 0.019 | -0.029 | 0.023 | -0.022 | 0.031 |
| rs17405603 | 5 | 144608340 | A | T | -0.013 | 0.002 | 4.43E-11 | -0.001 | 0.002 | 5.40E-01 | BMI | 0.008 | 0.011 | 0.000 | 0.013 | 0.012 | 0.017 |
| rs10054327 | 5 | 147871137 | G | A | 0.003 | 0.002 | 1.74E-01 | 0.017 | 0.002 | 3.40E-15 | TV | -0.002 | 0.010 | -0.013 | 0.012 | -0.002 | 0.016 |
| rs2861089 | 5 | 164557954 | A | T | 0.011 | 0.002 | 1.55E-09 | -0.003 | 0.002 | 1.50E-01 | BMI | 0.015 | 0.010 | 0.011 | 0.012 | 0.019 | 0.015 |
| rs42210 | 5 | 166408788 | G | C | -0.003 | 0.002 | 1.02E-01 | -0.014 | 0.002 | 7.30E-09 | TV | -0.007 | 0.011 | 0.004 | 0.013 | -0.024 | 0.017 |
| rs72828890 | 5 | 167806836 | C | T | 0.011 | 0.003 | 4.31E-04 | 0.019 | 0.003 | 4.90E-09 | TV | 0.012 | 0.016 | 0.017 | 0.019 | 0.003 | 0.025 |
| rs2228213 | 6 | 12124855 | A | G | -0.014 | 0.002 | 5.50E-17 | -0.005 | 0.002 | 3.30E-02 | BMI | -0.007 | 0.010 | -0.010 | 0.012 | 0.001 | 0.016 |
| rs11757278 | 6 | 13180454 | T | C | 0.013 | 0.002 | 6.92E-13 | 0.003 | 0.002 | 1.40E-01 | BMI | 0.006 | 0.010 | 0.005 | 0.013 | 0.016 | 0.016 |
| rs3806114 | 6 | 20482335 | A | G | -0.012 | 0.002 | 1.46E-11 | -0.009 | 0.002 | 1.30E-04 | BMI | 0.008 | 0.010 | 0.004 | 0.013 | 0.010 | 0.016 |
| rs72834698 | 6 | 26176517 | G | A | 0.015 | 0.003 | 3.33E-08 | 0.023 | 0.003 | 2.70E-13 | TV | 0.044 | 0.014 | 0.059 | 0.017 | 0.069 | 0.022 |
| rs9471333 | 6 | 40362023 | C | T | 0.021 | 0.002 | 6.90E-29 | 0.013 | 0.002 | 1.50E-09 | TV | 0.010 | 0.010 | 0.007 | 0.012 | 0.018 | 0.015 |
| rs2206277 | 6 | 50798526 | T | C | 0.041 | 0.002 | 1.82E-83 | 0.001 | 0.003 | 6.00E-01 | BMI | -0.010 | 0.012 | -0.007 | 0.015 | -0.026 | 0.020 |
| rs1327259 | 6 | 51177811 | A | G | 0.016 | 0.002 | 1.47E-19 | 0.001 | 0.002 | 6.60E-01 | BMI | 0.012 | 0.010 | 0.013 | 0.012 | 0.004 | 0.016 |
| rs6915002 | 6 | 54028069 | T | C | 0.010 | 0.002 | 8.71E-09 | 0.001 | 0.002 | 5.90E-01 | BMI | 0.004 | 0.010 | 0.007 | 0.012 | 0.001 | 0.015 |
| rs9370410 | 6 | 55171842 | A | G | 0.011 | 0.002 | 2.58E-08 | 0.004 | 0.002 | 8.00E-02 | BMI | 0.007 | 0.011 | 0.017 | 0.013 | -0.008 | 0.017 |
| rs2622274 | 6 | 64240516 | T | G | -0.011 | 0.002 | 3.23E-10 | 0.000 | 0.002 | 9.60E-01 | BMI | -0.005 | 0.010 | -0.002 | 0.012 | 0.001 | 0.015 |
| rs4523073 | 6 | 67550288 | A | G | -0.003 | 0.002 | 1.75E-01 | -0.014 | 0.002 | 5.00E-10 | TV | 0.006 | 0.010 | 0.002 | 0.012 | 0.022 | 0.016 |
| rs6921533 | 6 | 73742334 | T | C | 0.010 | 0.002 | 1.37E-08 | 0.002 | 0.002 | 4.10E-01 | BMI | -0.001 | 0.011 | -0.007 | 0.013 | 0.008 | 0.017 |
| rs9294260 | 6 | 83433228 | A | G | 0.014 | 0.002 | 8.16E-18 | -0.002 | 0.002 | 3.50E-01 | BMI | -0.005 | 0.010 | -0.006 | 0.012 | 0.005 | 0.015 |
| rs6905544 | 6 | 98411631 | A | G | -0.017 | 0.002 | 2.59E-21 | -0.019 | 0.002 | 8.50E-18 | TV | -0.029 | 0.010 | -0.024 | 0.012 | -0.035 | 0.015 |
| rs17789218 | 6 | 100600097 | T | C | -0.011 | 0.002 | 4.78E-09 | 0.019 | 0.003 | 1.40E-13 | TV | 0.004 | 0.012 | -0.006 | 0.014 | 0.011 | 0.018 |
| rs156201 | 6 | 104847441 | C | G | 0.012 | 0.002 | 1.65E-10 | 0.002 | 0.003 | 4.50E-01 | BMI | 0.003 | 0.011 | 0.005 | 0.013 | 0.012 | 0.017 |
| rs768023 | 6 | 108876002 | A | G | 0.016 | 0.002 | 1.13E-22 | 0.005 | 0.002 | 2.00E-02 | BMI | 0.015 | 0.010 | 0.019 | 0.012 | 0.006 | 0.015 |
| rs2357760 | 6 | 120213880 | A | G | 0.014 | 0.002 | 2.11E-16 | 0.006 | 0.002 | 1.60E-02 | BMI | 0.001 | 0.010 | 0.000 | 0.012 | -0.001 | 0.016 |
| rs2875762 | 6 | 124925032 | C | G | 0.013 | 0.002 | 1.07E-10 | 0.008 | 0.003 | 1.80E-03 | BMI | -0.003 | 0.011 | 0.002 | 0.014 | -0.001 | 0.018 |
| rs1268065 | 6 | 126042783 | A | G | -0.010 | 0.002 | 7.12E-10 | 0.002 | 0.002 | 4.50E-01 | BMI | -0.001 | 0.010 | 0.006 | 0.012 | -0.004 | 0.015 |
| rs9375702 | 6 | 130384187 | T | C | -0.011 | 0.002 | 5.52E-09 | -0.001 | 0.002 | 7.80E-01 | BMI | 0.004 | 0.010 | 0.004 | 0.013 | 0.002 | 0.016 |
| rs2246012 | 6 | 131898208 | T | C | -0.016 | 0.002 | 1.15E-13 | 0.000 | 0.003 | 9.30E-01 | BMI | -0.020 | 0.013 | -0.035 | 0.016 | -0.019 | 0.021 |
| rs2184364 | 6 | 143183596 | A | G | 0.010 | 0.002 | 3.38E-06 | 0.016 | 0.003 | 3.00E-09 | TV | 0.023 | 0.012 | 0.024 | 0.015 | 0.017 | 0.019 |
| rs12527426 | 6 | 153392002 | A | G | 0.015 | 0.002 | 5.54E-16 | 0.009 | 0.002 | 2.00E-04 | BMI | 0.012 | 0.010 | -0.003 | 0.013 | 0.024 | 0.016 |
| rs9478496 | 6 | 154333183 | T | C | -0.016 | 0.002 | 8.17E-12 | -0.006 | 0.003 | 5.80E-02 | BMI | -0.018 | 0.013 | -0.012 | 0.016 | -0.007 | 0.020 |
| rs13191362 | 6 | 163033350 | A | G | 0.024 | 0.003 | 4.08E-21 | 0.001 | 0.003 | 7.40E-01 | BMI | 0.021 | 0.015 | 0.036 | 0.018 | -0.008 | 0.023 |
| rs9458814 | 6 | 163771305 | T | C | -0.011 | 0.002 | 2.33E-08 | -0.004 | 0.003 | 7.50E-02 | BMI | -0.021 | 0.011 | -0.027 | 0.014 | -0.020 | 0.018 |
| rs9718104 | 6 | 166170539 | T | G | 0.000 | 0.004 | 9.54E-01 | -0.041 | 0.005 | 9.30E-19 | TV | -0.002 | 0.022 | 0.009 | 0.027 | 0.005 | 0.036 |
| rs17568389 | 7 | 8091876 | T | A | 0.003 | 0.002 | 5.75E-02 | 0.015 | 0.002 | 8.60E-13 | TV | 0.001 | 0.010 | 0.000 | 0.012 | 0.012 | 0.015 |
| rs4307239 | 7 | 24354300 | A | G | -0.012 | 0.002 | 1.47E-11 | -0.001 | 0.002 | 8.00E-01 | BMI | -0.008 | 0.010 | -0.017 | 0.012 | 0.016 | 0.015 |
| rs213518 | 7 | 26941065 | T | C | -0.015 | 0.002 | 2.49E-10 | 0.005 | 0.003 | 8.10E-02 | BMI | -0.001 | 0.014 | -0.003 | 0.017 | 0.028 | 0.022 |
| rs2108719 | 7 | 39481056 | A | G | 0.011 | 0.002 | 2.28E-08 | 0.003 | 0.002 | 2.00E-01 | BMI | -0.003 | 0.011 | 0.003 | 0.013 | -0.007 | 0.017 |
| rs10234444 | 7 | 41828527 | G | A | 0.005 | 0.002 | 1.80E-02 | 0.016 | 0.003 | 2.90E-08 | TV | -0.017 | 0.013 | -0.023 | 0.016 | -0.004 | 0.021 |
| rs2289379 | 7 | 44804225 | T | C | -0.014 | 0.002 | 7.02E-15 | -0.004 | 0.002 | 4.50E-02 | BMI | -0.015 | 0.010 | -0.009 | 0.012 | 0.000 | 0.015 |
| rs11772246 | 7 | 71603692 | T | C | 0.015 | 0.002 | 3.69E-11 | 0.003 | 0.003 | 2.80E-01 | BMI | 0.036 | 0.012 | 0.039 | 0.015 | 0.020 | 0.019 |
| rs17207196 | 7 | 75101065 | T | C | -0.022 | 0.002 | 1.58E-36 | -0.009 | 0.002 | 2.30E-05 | BMI | -0.006 | 0.011 | 0.003 | 0.013 | -0.004 | 0.017 |
| rs17149254 | 7 | 76634463 | T | C | 0.024 | 0.002 | 2.99E-25 | -0.003 | 0.003 | 3.50E-01 | BMI | 0.012 | 0.012 | 0.025 | 0.015 | 0.004 | 0.020 |
| rs6973656 | 7 | 77422583 | A | G | -0.010 | 0.002 | 6.31E-09 | -0.013 | 0.002 | 1.00E-09 | both | 0.029 | 0.010 | 0.028 | 0.012 | 0.036 | 0.015 |
| rs1965529 | 7 | 77825707 | A | G | 0.016 | 0.002 | 7.66E-14 | 0.008 | 0.003 | 2.30E-03 | BMI | -0.009 | 0.012 | -0.003 | 0.014 | -0.007 | 0.019 |
| rs274628 | 7 | 86265855 | A | C | -0.010 | 0.002 | 1.36E-08 | -0.002 | 0.002 | 4.30E-01 | BMI | -0.013 | 0.010 | -0.010 | 0.012 | -0.013 | 0.016 |
| rs973734 | 7 | 89387578 | C | A | 0.004 | 0.002 | 1.16E-01 | 0.017 | 0.003 | 1.50E-08 | TV | 0.002 | 0.014 | 0.013 | 0.016 | -0.009 | 0.021 |
| rs2283006 | 7 | 93085722 | A | G | 0.013 | 0.002 | 8.06E-15 | -0.002 | 0.002 | 2.80E-01 | BMI | -0.013 | 0.010 | -0.021 | 0.012 | 0.003 | 0.015 |
| rs13240600 | 7 | 99064466 | A | G | 0.018 | 0.002 | 7.90E-16 | 0.017 | 0.003 | 3.00E-08 | BMI | 0.011 | 0.013 | 0.011 | 0.016 | -0.006 | 0.021 |
| rs62471080 | 7 | 109128060 | G | C | -0.009 | 0.002 | 4.18E-06 | -0.013 | 0.002 | 3.70E-09 | TV | -0.005 | 0.010 | 0.010 | 0.012 | -0.012 | 0.015 |
| rs10261050 | 7 | 114337652 | T | C | 0.011 | 0.002 | 4.53E-11 | 0.005 | 0.002 | 3.60E-02 | BMI | -0.005 | 0.010 | -0.010 | 0.012 | 0.011 | 0.015 |
| rs10246289 | 7 | 115544763 | A | G | 0.005 | 0.003 | 7.85E-02 | 0.019 | 0.003 | 1.70E-08 | TV | 0.041 | 0.015 | 0.033 | 0.019 | 0.033 | 0.024 |
| rs11763734 | 7 | 126400910 | A | C | -0.003 | 0.002 | 1.35E-01 | -0.012 | 0.002 | 8.20E-09 | TV | -0.001 | 0.010 | -0.002 | 0.012 | 0.005 | 0.015 |
| rs7802342 | 7 | 137435925 | T | G | -0.012 | 0.002 | 6.23E-11 | 0.001 | 0.002 | 5.50E-01 | BMI | -0.013 | 0.011 | -0.014 | 0.013 | -0.025 | 0.018 |
| rs11525873 | 7 | 138817193 | T | C | 0.023 | 0.003 | 2.98E-13 | -0.003 | 0.004 | 4.70E-01 | BMI | 0.017 | 0.016 | 0.015 | 0.020 | 0.043 | 0.027 |
| rs11773362 | 7 | 147668180 | T | C | -0.011 | 0.002 | 6.39E-09 | 0.000 | 0.002 | 9.50E-01 | BMI | -0.009 | 0.010 | -0.022 | 0.012 | 0.002 | 0.016 |
| rs2907948 | 7 | 150638484 | A | G | -0.015 | 0.002 | 1.95E-14 | -0.004 | 0.003 | 7.40E-02 | BMI | 0.002 | 0.011 | -0.002 | 0.014 | 0.014 | 0.018 |
| rs56211164 | 7 | 158016764 | A | G | -0.013 | 0.002 | 8.90E-09 | 0.002 | 0.003 | 5.20E-01 | BMI | -0.012 | 0.012 | -0.020 | 0.014 | -0.024 | 0.018 |
| rs7834121 | 8 | 10770255 | G | T | 0.017 | 0.002 | 8.18E-19 | -0.014 | 0.002 | 1.10E-10 | TV | 0.007 | 0.010 | -0.007 | 0.012 | 0.028 | 0.016 |
| rs13263601 | 8 | 14095900 | A | C | -0.015 | 0.002 | 4.87E-16 | 0.006 | 0.002 | 1.20E-02 | BMI | 0.009 | 0.010 | 0.018 | 0.012 | -0.005 | 0.016 |
| rs10110189 | 8 | 15393380 | T | C | -0.016 | 0.003 | 2.20E-08 | 0.004 | 0.004 | 2.70E-01 | BMI | 0.014 | 0.016 | 0.003 | 0.019 | -0.001 | 0.026 |
| rs73225274 | 8 | 21088909 | A | G | -0.016 | 0.003 | 2.33E-08 | -0.006 | 0.003 | 6.10E-02 | BMI | -0.009 | 0.014 | -0.023 | 0.017 | -0.005 | 0.022 |
| rs13249650 | 8 | 25664336 | A | T | 0.011 | 0.002 | 1.02E-08 | 0.005 | 0.002 | 2.10E-02 | BMI | 0.002 | 0.010 | 0.006 | 0.012 | -0.001 | 0.015 |
| rs1421334 | 8 | 30865733 | A | C | 0.014 | 0.002 | 3.11E-15 | 0.017 | 0.002 | 2.20E-15 | both | 0.017 | 0.010 | 0.022 | 0.012 | -0.005 | 0.015 |
| rs2466103 | 8 | 32412304 | T | G | -0.012 | 0.002 | 8.00E-12 | 0.001 | 0.002 | 8.10E-01 | BMI | -0.010 | 0.011 | -0.009 | 0.012 | -0.009 | 0.016 |
| rs6468266 | 8 | 34380276 | A | T | -0.011 | 0.002 | 7.71E-11 | -0.010 | 0.002 | 9.70E-06 | BMI | -0.011 | 0.010 | -0.011 | 0.012 | -0.005 | 0.016 |
| rs12681792 | 8 | 62054463 | A | C | 0.015 | 0.002 | 2.88E-12 | 0.007 | 0.003 | 1.50E-02 | BMI | 0.019 | 0.012 | 0.010 | 0.015 | 0.015 | 0.020 |
| rs6996198 | 8 | 65463442 | C | T | -0.007 | 0.002 | 5.33E-03 | -0.016 | 0.003 | 3.50E-08 | TV | -0.016 | 0.013 | -0.011 | 0.016 | -0.017 | 0.020 |
| rs12334877 | 8 | 67194171 | A | G | -0.014 | 0.002 | 2.17E-11 | -0.002 | 0.003 | 5.10E-01 | BMI | 0.009 | 0.012 | 0.010 | 0.014 | 0.012 | 0.019 |
| rs6472942 | 8 | 76827190 | T | C | 0.017 | 0.002 | 3.49E-22 | -0.013 | 0.002 | 1.80E-09 | TV | 0.000 | 0.010 | -0.001 | 0.012 | 0.015 | 0.015 |
| rs11985394 | 8 | 92667521 | T | A | 0.001 | 0.002 | 4.61E-01 | 0.012 | 0.002 | 1.30E-08 | TV | 0.010 | 0.010 | 0.020 | 0.012 | 0.000 | 0.015 |
| rs1383592 | 8 | 106430676 | A | G | 0.012 | 0.002 | 4.92E-09 | 0.005 | 0.003 | 6.40E-02 | BMI | -0.013 | 0.012 | -0.004 | 0.014 | -0.018 | 0.018 |
| rs3808477 | 8 | 116670347 | T | C | -0.018 | 0.002 | 8.73E-22 | -0.006 | 0.002 | 8.50E-03 | BMI | -0.007 | 0.011 | -0.014 | 0.013 | -0.009 | 0.017 |
| rs12541615 | 8 | 118870151 | T | C | -0.015 | 0.003 | 4.97E-09 | -0.018 | 0.003 | 3.90E-10 | TV | -0.003 | 0.012 | -0.013 | 0.015 | 0.006 | 0.019 |
| rs6470144 | 8 | 124152245 | T | G | 0.010 | 0.002 | 3.06E-08 | 0.003 | 0.002 | 1.40E-01 | BMI | -0.002 | 0.010 | -0.006 | 0.012 | -0.002 | 0.016 |
| rs7842934 | 8 | 132838921 | T | C | -0.018 | 0.003 | 1.55E-08 | -0.001 | 0.004 | 8.80E-01 | BMI | 0.016 | 0.018 | 0.026 | 0.023 | 0.050 | 0.030 |
| rs16906838 | 8 | 138213836 | T | C | -0.025 | 0.004 | 4.31E-10 | 0.000 | 0.005 | 9.40E-01 | BMI | -0.006 | 0.022 | 0.004 | 0.025 | -0.020 | 0.034 |
| rs10099330 | 8 | 143383694 | A | G | -0.012 | 0.002 | 3.22E-12 | -0.007 | 0.002 | 9.80E-04 | BMI | -0.001 | 0.010 | -0.006 | 0.012 | -0.001 | 0.015 |
| rs2717559 | 8 | 143882420 | A | G | 0.004 | 0.002 | 4.06E-02 | 0.012 | 0.002 | 1.50E-08 | TV | 0.000 | 0.010 | -0.006 | 0.012 | 0.003 | 0.015 |
| rs2616830 | 9 | 1721385 | G | A | 0.001 | 0.002 | 6.54E-01 | 0.016 | 0.002 | 2.90E-14 | TV | 0.013 | 0.010 | 0.016 | 0.012 | 0.031 | 0.015 |
| rs1948080 | 9 | 11852043 | T | G | 0.014 | 0.002 | 1.13E-14 | 0.010 | 0.002 | 1.10E-05 | BMI | -0.019 | 0.010 | -0.022 | 0.012 | -0.007 | 0.016 |
| rs34864022 | 9 | 22609110 | A | G | 0.000 | 0.004 | 9.91E-01 | -0.026 | 0.004 | 1.40E-09 | TV | 0.013 | 0.021 | 0.015 | 0.025 | -0.026 | 0.033 |
| rs12554512 | 9 | 23352293 | T | C | -0.010 | 0.002 | 1.67E-07 | 0.021 | 0.002 | 3.80E-21 | TV | -0.002 | 0.010 | 0.011 | 0.012 | -0.005 | 0.016 |
| rs2275003 | 9 | 34124860 | A | G | 0.011 | 0.002 | 9.44E-12 | -0.006 | 0.002 | 3.80E-03 | BMI | 0.008 | 0.010 | 0.010 | 0.012 | 0.019 | 0.015 |
| rs13296413 | 9 | 37258105 | T | C | -0.015 | 0.002 | 2.49E-17 | 0.006 | 0.002 | 5.10E-03 | BMI | 0.015 | 0.010 | 0.006 | 0.012 | 0.032 | 0.015 |
| rs2134858 | 9 | 73837155 | T | C | -0.012 | 0.002 | 5.87E-12 | -0.004 | 0.002 | 6.00E-02 | BMI | -0.005 | 0.010 | -0.003 | 0.012 | -0.033 | 0.015 |
| rs7861160 | 9 | 80799579 | T | C | 0.010 | 0.002 | 3.68E-08 | 0.002 | 0.002 | 3.10E-01 | BMI | -0.008 | 0.010 | -0.009 | 0.012 | 0.002 | 0.015 |
| rs1999433 | 9 | 81371441 | T | C | -0.011 | 0.002 | 2.90E-10 | 0.000 | 0.002 | 8.60E-01 | BMI | -0.004 | 0.010 | -0.005 | 0.012 | -0.011 | 0.015 |
| rs2791574 | 9 | 82212094 | T | G | -0.004 | 0.002 | 2.17E-02 | -0.013 | 0.002 | 3.00E-08 | TV | -0.020 | 0.010 | -0.021 | 0.012 | -0.014 | 0.016 |
| rs2777768 | 9 | 84186734 | A | G | 0.012 | 0.002 | 6.38E-10 | 0.002 | 0.002 | 3.10E-01 | BMI | 0.005 | 0.011 | 0.011 | 0.013 | -0.028 | 0.017 |
| rs870151 | 9 | 92230747 | T | A | -0.011 | 0.002 | 1.14E-09 | -0.016 | 0.002 | 8.80E-13 | TV | 0.010 | 0.010 | 0.011 | 0.012 | 0.005 | 0.015 |
| rs2787374 | 9 | 103054951 | T | C | 0.011 | 0.002 | 1.36E-10 | 0.012 | 0.002 | 1.20E-08 | TV | 0.012 | 0.010 | 0.005 | 0.012 | 0.028 | 0.015 |
| rs7024334 | 9 | 109072075 | T | G | 0.014 | 0.002 | 4.71E-12 | 0.000 | 0.003 | 8.90E-01 | BMI | 0.011 | 0.011 | 0.003 | 0.014 | 0.018 | 0.018 |
| rs1928295 | 9 | 120378483 | T | C | 0.013 | 0.002 | 2.23E-16 | 0.006 | 0.002 | 3.90E-03 | BMI | 0.020 | 0.010 | 0.036 | 0.012 | 0.025 | 0.015 |
| rs1877875 | 9 | 120664469 | T | C | -0.011 | 0.002 | 2.51E-10 | -0.002 | 0.002 | 3.10E-01 | BMI | 0.013 | 0.010 | 0.010 | 0.012 | 0.008 | 0.015 |
| rs7043521 | 9 | 124602779 | A | T | 0.009 | 0.002 | 1.09E-06 | 0.014 | 0.002 | 6.50E-11 | TV | -0.021 | 0.010 | -0.021 | 0.012 | -0.043 | 0.015 |
| rs4382592 | 9 | 134870755 | T | G | 0.009 | 0.002 | 1.81E-06 | 0.014 | 0.002 | 6.70E-09 | TV | 0.009 | 0.010 | 0.006 | 0.013 | 0.000 | 0.016 |
| rs2073869 | 9 | 135763816 | C | T | 0.007 | 0.002 | 1.80E-03 | 0.019 | 0.003 | 1.50E-10 | TV | -0.011 | 0.013 | -0.018 | 0.015 | -0.019 | 0.020 |
| rs10858334 | 9 | 137989785 | C | G | -0.015 | 0.003 | 4.91E-09 | 0.000 | 0.003 | 8.90E-01 | BMI | 0.014 | 0.014 | 0.016 | 0.017 | 0.038 | 0.023 |
| rs1623025 | 10 | 11164813 | G | C | -0.004 | 0.002 | 3.50E-02 | -0.012 | 0.002 | 1.40E-08 | TV | 0.018 | 0.010 | 0.019 | 0.012 | 0.006 | 0.015 |
| rs7893571 | 10 | 16750129 | T | G | 0.013 | 0.002 | 5.83E-12 | 0.004 | 0.002 | 8.50E-02 | BMI | -0.001 | 0.010 | -0.005 | 0.012 | 0.005 | 0.016 |
| rs1243182 | 10 | 21916728 | C | T | -0.017 | 0.002 | 4.11E-21 | -0.019 | 0.002 | 2.00E-15 | TV | -0.029 | 0.011 | -0.025 | 0.013 | -0.054 | 0.016 |
| rs10829164 | 10 | 27318370 | T | C | 0.015 | 0.002 | 2.37E-10 | -0.005 | 0.003 | 9.10E-02 | BMI | 0.006 | 0.013 | 0.001 | 0.016 | 0.066 | 0.021 |
| rs4097319 | 10 | 33860515 | T | G | 0.011 | 0.002 | 4.32E-10 | 0.007 | 0.002 | 6.40E-04 | BMI | -0.005 | 0.010 | -0.017 | 0.012 | 0.019 | 0.015 |
| rs12765914 | 10 | 34013507 | T | C | 0.023 | 0.003 | 1.96E-13 | 0.006 | 0.004 | 1.30E-01 | BMI | 0.012 | 0.017 | 0.023 | 0.021 | 0.039 | 0.027 |
| rs1624134 | 10 | 34834482 | C | G | 0.010 | 0.002 | 6.51E-09 | 0.005 | 0.002 | 4.10E-02 | BMI | 0.009 | 0.010 | 0.009 | 0.012 | 0.003 | 0.015 |
| rs2045147 | 10 | 56660437 | A | G | 0.005 | 0.002 | 1.82E-03 | 0.013 | 0.002 | 5.90E-09 | TV | 0.009 | 0.010 | 0.006 | 0.012 | 0.019 | 0.015 |
| rs10994943 | 10 | 63591413 | T | G | 0.003 | 0.002 | 7.68E-02 | 0.013 | 0.002 | 3.10E-09 | TV | 0.007 | 0.010 | 0.000 | 0.012 | 0.014 | 0.015 |
| rs12098284 | 10 | 76047464 | T | C | 0.018 | 0.003 | 9.87E-13 | 0.003 | 0.003 | 2.90E-01 | BMI | -0.010 | 0.015 | -0.025 | 0.018 | 0.035 | 0.023 |
| rs11001259 | 10 | 76807650 | A | T | -0.014 | 0.002 | 1.22E-08 | -0.008 | 0.003 | 4.10E-03 | BMI | -0.005 | 0.015 | -0.005 | 0.018 | 0.006 | 0.024 |
| rs11201422 | 10 | 86924483 | T | C | 0.008 | 0.002 | 5.59E-06 | 0.013 | 0.002 | 3.40E-08 | TV | 0.019 | 0.011 | 0.022 | 0.012 | 0.027 | 0.016 |
| rs17094222 | 10 | 102395440 | T | C | -0.017 | 0.002 | 4.04E-18 | 0.002 | 0.003 | 5.00E-01 | BMI | -0.005 | 0.012 | -0.011 | 0.014 | -0.003 | 0.018 |
| rs10786658 | 10 | 103737193 | A | T | 0.000 | 0.002 | 8.76E-01 | -0.014 | 0.002 | 4.60E-11 | TV | 0.003 | 0.010 | 0.002 | 0.012 | -0.001 | 0.015 |
| rs11594179 | 10 | 104392580 | T | C | -0.011 | 0.002 | 2.16E-08 | 0.001 | 0.003 | 6.80E-01 | BMI | -0.002 | 0.012 | -0.008 | 0.014 | 0.016 | 0.018 |
| rs7089973 | 10 | 116569565 | C | A | 0.000 | 0.002 | 8.68E-01 | -0.013 | 0.002 | 2.40E-09 | TV | -0.021 | 0.010 | -0.028 | 0.012 | -0.036 | 0.016 |
| rs11245482 | 10 | 126733546 | T | C | -0.010 | 0.002 | 5.11E-08 | -0.013 | 0.002 | 2.60E-09 | TV | -0.004 | 0.010 | -0.002 | 0.012 | -0.008 | 0.015 |
| rs17727474 | 10 | 127182959 | C | T | 0.000 | 0.002 | 9.42E-01 | 0.018 | 0.003 | 3.10E-09 | TV | 0.001 | 0.013 | -0.009 | 0.016 | 0.010 | 0.021 |
| rs11246136 | 11 | 371265 | A | C | -0.017 | 0.003 | 2.03E-08 | 0.003 | 0.004 | 4.60E-01 | BMI | 0.008 | 0.017 | 0.019 | 0.021 | -0.003 | 0.027 |
| rs12421848 | 11 | 891338 | A | G | -0.014 | 0.002 | 3.99E-13 | -0.001 | 0.002 | 7.30E-01 | BMI | 0.018 | 0.010 | 0.020 | 0.012 | 0.016 | 0.016 |
| rs4256980 | 11 | 8673939 | C | G | -0.019 | 0.002 | 8.63E-29 | -0.003 | 0.002 | 2.20E-01 | BMI | -0.001 | 0.010 | -0.004 | 0.012 | 0.001 | 0.016 |
| rs12289262 | 11 | 12894758 | C | T | -0.007 | 0.002 | 3.82E-04 | -0.014 | 0.002 | 2.00E-08 | TV | -0.028 | 0.011 | -0.030 | 0.014 | -0.010 | 0.018 |
| rs900144 | 11 | 13294268 | T | C | 0.015 | 0.002 | 1.53E-18 | 0.006 | 0.002 | 3.40E-03 | BMI | -0.016 | 0.010 | -0.012 | 0.012 | -0.037 | 0.015 |
| rs6265 | 11 | 27679916 | T | C | -0.041 | 0.002 | 7.40E-89 | 0.003 | 0.003 | 2.20E-01 | BMI | -0.008 | 0.012 | -0.015 | 0.015 | -0.010 | 0.019 |
| rs570463 | 11 | 28739318 | A | C | -0.012 | 0.002 | 4.33E-11 | -0.002 | 0.002 | 4.20E-01 | BMI | -0.017 | 0.011 | -0.005 | 0.012 | -0.007 | 0.016 |
| rs11030618 | 11 | 29243293 | T | C | 0.011 | 0.002 | 1.67E-10 | 0.002 | 0.002 | 3.40E-01 | BMI | -0.016 | 0.010 | -0.014 | 0.012 | -0.025 | 0.015 |
| rs2065418 | 11 | 30422068 | T | G | 0.014 | 0.002 | 6.07E-15 | 0.005 | 0.002 | 3.30E-02 | BMI | 0.016 | 0.010 | 0.005 | 0.012 | 0.008 | 0.016 |
| rs2862996 | 11 | 43653833 | T | G | -0.022 | 0.002 | 3.60E-35 | -0.004 | 0.002 | 9.40E-02 | BMI | -0.009 | 0.010 | -0.013 | 0.012 | 0.010 | 0.016 |
| rs10742752 | 11 | 45438374 | T | C | -0.012 | 0.002 | 1.20E-13 | 0.002 | 0.002 | 3.80E-01 | BMI | 0.015 | 0.010 | 0.012 | 0.012 | 0.025 | 0.015 |
| rs118081010 | 11 | 46174948 | T | C | 0.052 | 0.008 | 5.28E-12 | 0.019 | 0.009 | 3.00E-02 | BMI | 0.033 | 0.040 | 0.029 | 0.050 | 0.064 | 0.065 |
| rs7124681 | 11 | 47529947 | A | C | 0.026 | 0.002 | 3.96E-55 | 0.004 | 0.002 | 5.10E-02 | BMI | 0.007 | 0.010 | 0.004 | 0.012 | 0.003 | 0.015 |
| rs801733 | 11 | 65934549 | A | C | 0.011 | 0.002 | 1.49E-10 | 0.017 | 0.002 | 7.30E-14 | TV | 0.027 | 0.010 | 0.023 | 0.012 | 0.048 | 0.016 |
| rs592483 | 11 | 69445173 | T | C | -0.014 | 0.002 | 1.53E-16 | -0.002 | 0.002 | 2.60E-01 | BMI | -0.015 | 0.010 | -0.024 | 0.012 | 0.000 | 0.015 |
| rs116175338 | 11 | 72467875 | G | T | -0.012 | 0.002 | 2.92E-10 | -0.014 | 0.003 | 1.40E-08 | TV | -0.001 | 0.011 | -0.002 | 0.014 | 0.020 | 0.018 |
| rs12272012 | 11 | 84861286 | G | C | -0.009 | 0.005 | 6.78E-02 | -0.030 | 0.005 | 1.90E-08 | TV | 0.031 | 0.024 | 0.036 | 0.028 | 0.064 | 0.037 |
| rs10741329 | 11 | 89997796 | A | G | 0.012 | 0.002 | 3.91E-10 | 0.004 | 0.002 | 5.40E-02 | BMI | -0.006 | 0.010 | -0.006 | 0.012 | -0.013 | 0.016 |
| rs11020045 | 11 | 92487907 | A | C | -0.001 | 0.002 | 7.82E-01 | -0.013 | 0.002 | 1.60E-08 | TV | 0.007 | 0.010 | 0.018 | 0.012 | -0.003 | 0.016 |
| rs17207890 | 11 | 95490754 | G | A | 0.001 | 0.002 | 4.81E-01 | 0.016 | 0.002 | 6.70E-12 | TV | 0.000 | 0.010 | 0.016 | 0.012 | -0.021 | 0.016 |
| rs12286929 | 11 | 115022404 | A | G | -0.018 | 0.002 | 1.93E-27 | -0.006 | 0.002 | 5.50E-03 | BMI | 0.011 | 0.010 | 0.015 | 0.012 | 0.006 | 0.015 |
| rs76942203 | 11 | 116973247 | A | G | 0.026 | 0.004 | 9.08E-11 | 0.017 | 0.005 | 3.10E-04 | BMI | -0.005 | 0.020 | -0.002 | 0.024 | -0.031 | 0.032 |
| rs3825061 | 11 | 118944675 | T | C | 0.014 | 0.002 | 6.15E-16 | 0.003 | 0.002 | 2.10E-01 | BMI | 0.018 | 0.010 | 0.013 | 0.012 | 0.028 | 0.016 |
| rs11218575 | 11 | 122154576 | C | T | 0.003 | 0.002 | 9.06E-02 | 0.015 | 0.002 | 2.10E-12 | TV | 0.001 | 0.010 | 0.001 | 0.012 | -0.001 | 0.015 |
| rs4937842 | 11 | 133734542 | G | C | -0.008 | 0.002 | 9.11E-06 | -0.013 | 0.002 | 1.40E-08 | TV | -0.012 | 0.010 | -0.018 | 0.012 | -0.023 | 0.016 |
| rs11611246 | 12 | 939480 | T | G | 0.022 | 0.002 | 2.04E-28 | -0.002 | 0.003 | 4.20E-01 | BMI | 0.014 | 0.012 | 0.026 | 0.014 | -0.004 | 0.019 |
| rs765125 | 12 | 2156207 | T | C | -0.010 | 0.002 | 1.27E-08 | -0.006 | 0.002 | 3.40E-03 | BMI | -0.003 | 0.010 | -0.008 | 0.012 | -0.007 | 0.015 |
| rs10772643 | 12 | 13415288 | C | T | 0.010 | 0.003 | 1.35E-03 | 0.025 | 0.003 | 1.30E-12 | TV | 0.027 | 0.016 | 0.024 | 0.019 | 0.056 | 0.024 |
| rs11046972 | 12 | 23705969 | T | C | 0.018 | 0.003 | 3.72E-08 | 0.009 | 0.004 | 2.90E-02 | BMI | -0.013 | 0.017 | -0.009 | 0.021 | 0.005 | 0.027 |
| rs10842240 | 12 | 24060075 | C | G | 0.020 | 0.003 | 1.21E-14 | 0.006 | 0.003 | 6.20E-02 | BMI | -0.001 | 0.014 | -0.003 | 0.017 | -0.008 | 0.022 |
| rs10771746 | 12 | 30791864 | C | T | -0.002 | 0.002 | 3.44E-01 | -0.014 | 0.002 | 2.50E-09 | TV | 0.006 | 0.010 | 0.001 | 0.013 | 0.031 | 0.017 |
| rs11170468 | 12 | 39430048 | A | C | 0.013 | 0.002 | 1.12E-11 | 0.003 | 0.003 | 2.70E-01 | BMI | 0.009 | 0.011 | 0.006 | 0.014 | 0.005 | 0.018 |
| rs1350430 | 12 | 41819215 | T | C | -0.013 | 0.002 | 2.08E-13 | 0.001 | 0.002 | 6.80E-01 | BMI | 0.001 | 0.010 | 0.008 | 0.012 | 0.000 | 0.015 |
| rs7138803 | 12 | 50247468 | A | G | 0.030 | 0.002 | 3.10E-71 | 0.004 | 0.002 | 1.10E-01 | BMI | 0.011 | 0.010 | 0.004 | 0.012 | 0.005 | 0.016 |
| rs10876864 | 12 | 56401085 | G | A | -0.011 | 0.002 | 1.70E-10 | -0.013 | 0.002 | 1.00E-09 | TV | -0.010 | 0.010 | -0.029 | 0.012 | 0.021 | 0.015 |
| rs7975187 | 12 | 60964108 | A | G | -0.014 | 0.002 | 3.86E-11 | -0.004 | 0.003 | 9.90E-02 | BMI | -0.010 | 0.012 | -0.010 | 0.014 | -0.015 | 0.018 |
| rs8756 | 12 | 66359752 | C | A | 0.005 | 0.002 | 5.18E-03 | -0.013 | 0.002 | 5.30E-10 | TV | -0.009 | 0.010 | -0.005 | 0.012 | -0.016 | 0.015 |
| rs650198 | 12 | 69674595 | T | C | -0.014 | 0.002 | 6.43E-13 | 0.000 | 0.002 | 8.70E-01 | BMI | 0.020 | 0.011 | 0.023 | 0.013 | 0.008 | 0.017 |
| rs11115176 | 12 | 82465797 | T | C | 0.013 | 0.002 | 6.79E-12 | 0.003 | 0.003 | 3.00E-01 | BMI | 0.001 | 0.011 | 0.003 | 0.014 | -0.019 | 0.018 |
| rs10506971 | 12 | 89757937 | A | G | -0.014 | 0.002 | 4.69E-17 | -0.006 | 0.002 | 4.80E-03 | BMI | 0.004 | 0.010 | 0.006 | 0.012 | -0.020 | 0.015 |
| rs11105839 | 12 | 91237920 | A | T | -0.011 | 0.002 | 1.25E-11 | -0.002 | 0.002 | 3.70E-01 | BMI | 0.027 | 0.010 | 0.038 | 0.012 | -0.010 | 0.016 |
| rs2712665 | 12 | 99594947 | T | C | -0.011 | 0.002 | 5.40E-09 | -0.005 | 0.002 | 3.80E-02 | BMI | 0.003 | 0.011 | -0.008 | 0.013 | 0.039 | 0.017 |
| rs6539064 | 12 | 103706754 | C | G | 0.019 | 0.002 | 1.14E-23 | 0.001 | 0.002 | 7.80E-01 | BMI | 0.005 | 0.011 | 0.008 | 0.013 | -0.009 | 0.017 |
| rs2164744 | 12 | 109872039 | T | C | -0.005 | 0.002 | 1.05E-02 | -0.013 | 0.002 | 1.10E-08 | TV | 0.014 | 0.010 | 0.007 | 0.012 | 0.027 | 0.015 |
| rs2173650 | 12 | 117525235 | G | T | 0.004 | 0.003 | 1.44E-01 | 0.018 | 0.003 | 4.60E-09 | TV | -0.006 | 0.013 | -0.002 | 0.016 | 0.010 | 0.021 |
| rs11614340 | 12 | 133426483 | T | C | -0.012 | 0.002 | 1.87E-10 | 0.003 | 0.002 | 1.60E-01 | BMI | -0.016 | 0.011 | -0.009 | 0.013 | -0.032 | 0.017 |
| rs9512648 | 13 | 27933910 | A | G | 0.010 | 0.002 | 4.11E-08 | 0.005 | 0.002 | 3.60E-02 | BMI | 0.010 | 0.010 | 0.011 | 0.012 | 0.016 | 0.015 |
| rs7323 | 13 | 28009031 | C | G | -0.017 | 0.002 | 2.18E-18 | -0.002 | 0.002 | 4.90E-01 | BMI | 0.007 | 0.011 | 0.004 | 0.013 | 0.017 | 0.017 |
| rs4771218 | 13 | 28655311 | A | G | -0.014 | 0.002 | 3.18E-15 | 0.000 | 0.002 | 9.30E-01 | BMI | 0.009 | 0.010 | 0.012 | 0.012 | -0.006 | 0.016 |
| rs9595908 | 13 | 33184288 | T | C | 0.015 | 0.002 | 3.73E-20 | 0.008 | 0.002 | 2.20E-04 | BMI | -0.002 | 0.010 | 0.004 | 0.012 | -0.013 | 0.016 |
| rs9603697 | 13 | 40783323 | T | C | 0.013 | 0.002 | 1.69E-13 | 0.005 | 0.002 | 2.20E-02 | BMI | -0.011 | 0.010 | -0.018 | 0.012 | -0.015 | 0.016 |
| rs9563168 | 13 | 54247827 | G | A | 0.008 | 0.002 | 5.86E-05 | 0.018 | 0.003 | 4.30E-11 | TV | 0.006 | 0.012 | 0.010 | 0.014 | -0.013 | 0.018 |
| rs9569734 | 13 | 58319476 | A | G | 0.018 | 0.002 | 3.82E-14 | 0.019 | 0.003 | 3.40E-10 | TV | 0.010 | 0.013 | 0.012 | 0.016 | 0.037 | 0.021 |
| rs8181823 | 13 | 65477940 | A | C | -0.013 | 0.002 | 4.36E-10 | 0.000 | 0.003 | 9.30E-01 | BMI | 0.002 | 0.011 | 0.013 | 0.014 | -0.018 | 0.018 |
| rs9599161 | 13 | 67434016 | T | C | 0.010 | 0.002 | 2.32E-09 | 0.004 | 0.002 | 8.10E-02 | BMI | 0.012 | 0.010 | 0.005 | 0.012 | 0.000 | 0.015 |
| rs1441264 | 13 | 79580919 | A | G | 0.017 | 0.002 | 7.60E-25 | -0.003 | 0.002 | 1.40E-01 | BMI | 0.017 | 0.010 | 0.020 | 0.012 | 0.010 | 0.016 |
| rs56858768 | 13 | 86511730 | G | A | -0.017 | 0.002 | 5.13E-15 | -0.015 | 0.002 | 3.40E-10 | TV | -0.010 | 0.011 | -0.012 | 0.013 | -0.019 | 0.017 |
| rs1927790 | 13 | 96922191 | T | C | -0.014 | 0.002 | 1.57E-17 | -0.005 | 0.002 | 3.20E-02 | BMI | 0.002 | 0.010 | -0.016 | 0.012 | 0.025 | 0.015 |
| rs9168 | 13 | 99101583 | A | C | -0.014 | 0.002 | 1.08E-13 | -0.006 | 0.002 | 1.80E-02 | BMI | 0.019 | 0.011 | 0.034 | 0.013 | 0.012 | 0.017 |
| rs7991062 | 13 | 100713194 | C | G | -0.001 | 0.002 | 4.84E-01 | -0.018 | 0.002 | 8.80E-15 | TV | -0.013 | 0.010 | -0.012 | 0.012 | -0.017 | 0.016 |
| rs9888533 | 13 | 107854612 | T | C | 0.013 | 0.002 | 1.38E-10 | 0.002 | 0.002 | 4.80E-01 | BMI | -0.018 | 0.010 | -0.010 | 0.012 | -0.029 | 0.016 |
| rs9522183 | 13 | 111977280 | T | G | -0.014 | 0.002 | 2.10E-12 | -0.005 | 0.002 | 1.80E-02 | BMI | -0.017 | 0.010 | -0.014 | 0.012 | 0.009 | 0.015 |
| rs12868881 | 13 | 112218924 | A | T | 0.014 | 0.002 | 1.40E-15 | 0.006 | 0.002 | 5.50E-03 | BMI | -0.002 | 0.010 | -0.002 | 0.012 | 0.008 | 0.015 |
| rs405797 | 14 | 26972834 | T | A | -0.007 | 0.002 | 6.68E-04 | -0.015 | 0.003 | 2.80E-09 | TV | -0.014 | 0.013 | -0.017 | 0.013 | -0.006 | 0.017 |
| rs1022785 | 14 | 29571653 | G | A | 0.008 | 0.002 | 9.68E-04 | 0.018 | 0.003 | 7.10E-09 | TV | -0.003 | 0.016 | -0.005 | 0.016 | 0.035 | 0.021 |
| rs1958898 | 14 | 40886886 | C | G | -0.015 | 0.002 | 4.23E-12 | -0.004 | 0.003 | 1.60E-01 | BMI | -0.004 | 0.014 | -0.010 | 0.015 | 0.001 | 0.019 |
| rs61983990 | 14 | 41475003 | A | G | 0.020 | 0.004 | 1.91E-08 | 0.019 | 0.004 | 9.00E-07 | BMI | 0.048 | 0.023 | 0.038 | 0.023 | 0.087 | 0.029 |
| rs4900714 | 14 | 47302219 | T | G | 0.015 | 0.002 | 1.00E-18 | 0.002 | 0.002 | 3.20E-01 | BMI | 0.000 | 0.011 | -0.003 | 0.012 | -0.024 | 0.015 |
| rs217669 | 14 | 62360075 | T | C | -0.017 | 0.002 | 6.27E-16 | -0.010 | 0.002 | 6.00E-05 | BMI | -0.003 | 0.013 | -0.011 | 0.013 | -0.002 | 0.017 |
| rs4430672 | 14 | 63094407 | T | C | 0.012 | 0.002 | 5.00E-09 | 0.009 | 0.003 | 7.20E-04 | BMI | 0.004 | 0.014 | 0.012 | 0.014 | -0.007 | 0.019 |
| rs3902951 | 14 | 69789755 | T | G | -0.013 | 0.002 | 2.88E-12 | -0.008 | 0.003 | 1.30E-03 | BMI | 0.010 | 0.013 | 0.019 | 0.014 | 0.013 | 0.018 |
| rs17182027 | 14 | 73348130 | A | G | -0.011 | 0.002 | 1.46E-10 | -0.003 | 0.002 | 1.20E-01 | BMI | -0.018 | 0.012 | -0.041 | 0.012 | 0.017 | 0.015 |
| rs7144011 | 14 | 79940383 | T | G | 0.026 | 0.002 | 2.37E-40 | 0.000 | 0.003 | 9.40E-01 | BMI | 0.009 | 0.015 | 0.002 | 0.014 | -0.006 | 0.019 |
| rs4517716 | 14 | 83013876 | C | G | -0.012 | 0.002 | 4.27E-09 | -0.004 | 0.003 | 1.00E-01 | BMI | 0.013 | 0.014 | 0.018 | 0.014 | 0.004 | 0.019 |
| rs12888545 | 14 | 88308044 | A | G | -0.013 | 0.002 | 1.76E-11 | -0.004 | 0.002 | 9.10E-02 | BMI | 0.014 | 0.014 | 0.006 | 0.014 | -0.006 | 0.018 |
| rs10145592 | 14 | 94287215 | C | G | -0.006 | 0.002 | 3.14E-04 | -0.015 | 0.002 | 1.80E-11 | TV | -0.023 | 0.012 | -0.019 | 0.012 | -0.029 | 0.015 |
| rs7157001 | 14 | 99749484 | A | G | -0.007 | 0.002 | 2.72E-03 | -0.014 | 0.002 | 2.30E-08 | TV | -0.007 | 0.014 | -0.003 | 0.014 | -0.051 | 0.019 |
| rs4906263 | 14 | 103249127 | C | G | -0.018 | 0.002 | 8.11E-23 | 0.001 | 0.002 | 7.60E-01 | BMI | 0.012 | 0.012 | 0.005 | 0.012 | 0.012 | 0.016 |
| rs12594043 | 15 | 27034988 | C | G | 0.010 | 0.002 | 1.31E-09 | 0.002 | 0.002 | 2.90E-01 | BMI | 0.004 | 0.010 | 0.006 | 0.012 | 0.010 | 0.015 |
| rs7172627 | 15 | 31877690 | A | G | -0.011 | 0.002 | 1.81E-11 | -0.004 | 0.002 | 9.30E-02 | BMI | 0.018 | 0.010 | 0.033 | 0.012 | 0.021 | 0.015 |
| rs11636611 | 15 | 36391965 | T | C | 0.010 | 0.002 | 8.85E-10 | 0.008 | 0.002 | 3.10E-04 | BMI | 0.018 | 0.010 | 0.015 | 0.012 | 0.019 | 0.015 |
| rs8043253 | 15 | 41503730 | C | T | -0.005 | 0.002 | 8.17E-03 | -0.012 | 0.002 | 3.40E-08 | TV | 0.017 | 0.010 | 0.028 | 0.012 | -0.002 | 0.015 |
| rs12912198 | 15 | 47103953 | T | C | -0.010 | 0.002 | 3.44E-08 | 0.000 | 0.002 | 9.20E-01 | BMI | -0.002 | 0.011 | -0.006 | 0.013 | -0.002 | 0.017 |
| rs2460 | 15 | 53073084 | G | A | -0.011 | 0.002 | 4.05E-09 | -0.015 | 0.002 | 5.00E-10 | TV | -0.015 | 0.011 | -0.021 | 0.013 | 0.015 | 0.018 |
| rs339991 | 15 | 60913637 | A | G | -0.013 | 0.002 | 3.51E-13 | -0.006 | 0.002 | 9.10E-03 | BMI | -0.022 | 0.010 | -0.026 | 0.012 | -0.025 | 0.015 |
| rs4775373 | 15 | 61476936 | T | C | 0.005 | 0.002 | 1.10E-02 | 0.013 | 0.002 | 4.70E-09 | TV | 0.006 | 0.010 | 0.007 | 0.012 | 0.017 | 0.016 |
| rs2470893 | 15 | 75019449 | T | C | 0.011 | 0.002 | 9.43E-10 | 0.004 | 0.002 | 7.60E-02 | BMI | -0.011 | 0.011 | -0.018 | 0.013 | -0.006 | 0.017 |
| rs61331678 | 15 | 77995949 | G | C | 0.009 | 0.002 | 4.87E-06 | 0.015 | 0.002 | 6.10E-12 | TV | -0.011 | 0.010 | -0.015 | 0.012 | -0.011 | 0.015 |
| rs72757415 | 15 | 92572762 | T | G | -0.016 | 0.002 | 1.34E-11 | -0.011 | 0.003 | 3.40E-05 | BMI | -0.009 | 0.012 | -0.007 | 0.014 | -0.009 | 0.019 |
| rs11633626 | 15 | 95271378 | A | C | -0.016 | 0.002 | 7.27E-19 | -0.003 | 0.002 | 1.40E-01 | BMI | -0.001 | 0.010 | -0.002 | 0.012 | -0.002 | 0.016 |
| rs6496248 | 15 | 98262923 | A | T | 0.010 | 0.002 | 2.93E-09 | 0.004 | 0.002 | 5.70E-02 | BMI | -0.015 | 0.010 | -0.014 | 0.012 | -0.022 | 0.016 |
| rs12591120 | 15 | 99236869 | T | C | 0.012 | 0.002 | 3.84E-08 | 0.007 | 0.002 | 7.10E-03 | BMI | -0.010 | 0.011 | -0.007 | 0.013 | -0.025 | 0.017 |
| rs2715423 | 15 | 99511873 | A | G | -0.012 | 0.002 | 1.90E-09 | -0.003 | 0.002 | 2.50E-01 | BMI | -0.010 | 0.011 | -0.009 | 0.013 | -0.004 | 0.017 |
| rs214249 | 16 | 348687 | T | G | 0.014 | 0.002 | 2.72E-15 | 0.007 | 0.002 | 1.30E-03 | BMI | 0.010 | 0.010 | 0.004 | 0.012 | 0.016 | 0.016 |
| rs12448257 | 16 | 3599655 | A | G | 0.016 | 0.002 | 9.04E-16 | -0.004 | 0.003 | 1.20E-01 | BMI | 0.003 | 0.012 | 0.000 | 0.015 | -0.016 | 0.019 |
| rs879620 | 16 | 4015729 | T | C | 0.023 | 0.002 | 8.45E-39 | 0.006 | 0.002 | 1.20E-02 | BMI | 0.010 | 0.010 | 0.004 | 0.012 | 0.012 | 0.015 |
| rs2058527 | 16 | 6704749 | T | G | -0.012 | 0.002 | 1.80E-09 | 0.000 | 0.002 | 9.90E-01 | BMI | 0.029 | 0.011 | 0.037 | 0.013 | 0.046 | 0.017 |
| rs35574015 | 16 | 15146061 | T | C | 0.008 | 0.002 | 7.78E-05 | -0.013 | 0.002 | 3.00E-08 | TV | 0.020 | 0.010 | 0.018 | 0.013 | -0.001 | 0.016 |
| rs7189927 | 16 | 28913787 | T | C | 0.026 | 0.002 | 1.13E-51 | 0.015 | 0.002 | 3.40E-11 | TV | 0.009 | 0.010 | 0.016 | 0.012 | 0.009 | 0.016 |
| rs749671 | 16 | 31088347 | G | A | 0.021 | 0.002 | 2.03E-35 | 0.016 | 0.002 | 2.70E-12 | TV | 0.023 | 0.010 | 0.023 | 0.012 | 0.015 | 0.016 |
| rs7184800 | 16 | 53509131 | G | A | -0.006 | 0.002 | 3.56E-03 | 0.017 | 0.002 | 8.20E-13 | TV | 0.005 | 0.011 | 0.003 | 0.013 | 0.024 | 0.016 |
| rs889398 | 16 | 69556715 | T | C | -0.020 | 0.002 | 3.23E-32 | -0.002 | 0.002 | 4.30E-01 | BMI | -0.016 | 0.010 | -0.020 | 0.012 | 0.003 | 0.015 |
| rs35949039 | 16 | 70572605 | T | G | -0.022 | 0.003 | 6.30E-12 | -0.008 | 0.004 | 3.20E-02 | BMI | 0.024 | 0.016 | 0.023 | 0.020 | 0.007 | 0.026 |
| rs12926250 | 16 | 72213316 | T | G | 0.018 | 0.003 | 1.78E-10 | 0.006 | 0.004 | 9.80E-02 | BMI | 0.012 | 0.016 | 0.021 | 0.019 | 0.006 | 0.025 |
| rs756717 | 16 | 72996162 | A | G | -0.013 | 0.002 | 2.38E-15 | -0.004 | 0.002 | 8.70E-02 | BMI | -0.005 | 0.010 | -0.023 | 0.012 | 0.019 | 0.016 |
| rs4390583 | 16 | 81694835 | A | C | -0.010 | 0.002 | 1.63E-08 | -0.001 | 0.002 | 7.70E-01 | BMI | -0.010 | 0.010 | -0.008 | 0.012 | -0.015 | 0.015 |
| rs12922346 | 16 | 82438337 | C | G | 0.013 | 0.002 | 1.48E-11 | 0.000 | 0.002 | 8.60E-01 | BMI | 0.003 | 0.011 | 0.002 | 0.013 | -0.010 | 0.017 |
| rs7206608 | 16 | 82872628 | C | G | -0.013 | 0.002 | 1.20E-12 | -0.010 | 0.002 | 3.40E-05 | BMI | -0.028 | 0.011 | -0.029 | 0.013 | -0.032 | 0.016 |
| rs2447098 | 17 | 2277720 | C | A | -0.001 | 0.002 | 6.77E-01 | -0.015 | 0.002 | 6.80E-12 | TV | -0.011 | 0.010 | -0.010 | 0.012 | -0.003 | 0.015 |
| rs11654952 | 17 | 4813799 | T | G | -0.004 | 0.003 | 1.22E-01 | -0.017 | 0.003 | 4.30E-08 | TV | 0.025 | 0.014 | 0.018 | 0.017 | 0.001 | 0.022 |
| rs1075901 | 17 | 15943910 | T | C | -0.012 | 0.002 | 4.43E-13 | 0.001 | 0.002 | 6.60E-01 | BMI | 0.007 | 0.010 | 0.006 | 0.012 | 0.005 | 0.015 |
| rs4986044 | 17 | 21261560 | T | C | -0.018 | 0.002 | 1.27E-27 | -0.009 | 0.002 | 1.80E-05 | BMI | -0.018 | 0.010 | -0.021 | 0.012 | 0.002 | 0.015 |
| rs8065172 | 17 | 31456969 | A | G | -0.012 | 0.002 | 5.29E-10 | -0.006 | 0.003 | 3.10E-02 | BMI | 0.025 | 0.011 | 0.023 | 0.013 | 0.023 | 0.018 |
| rs12150665 | 17 | 34914787 | T | C | 0.017 | 0.002 | 1.74E-24 | 0.006 | 0.002 | 4.50E-03 | BMI | 0.000 | 0.010 | 0.002 | 0.012 | 0.017 | 0.015 |
| rs6607337 | 17 | 35057373 | T | C | -0.012 | 0.002 | 2.55E-11 | -0.007 | 0.002 | 2.00E-03 | BMI | -0.024 | 0.010 | -0.027 | 0.013 | -0.022 | 0.016 |
| rs56161855 | 17 | 46288649 | A | T | -0.023 | 0.003 | 1.41E-16 | -0.007 | 0.003 | 2.00E-02 | BMI | -0.021 | 0.013 | -0.029 | 0.016 | -0.011 | 0.021 |
| rs9299 | 17 | 46669430 | T | C | 0.012 | 0.002 | 1.87E-11 | 0.002 | 0.002 | 2.90E-01 | BMI | 0.003 | 0.010 | 0.006 | 0.012 | -0.009 | 0.016 |
| rs11655587 | 17 | 47140794 | T | C | -0.021 | 0.002 | 6.87E-26 | -0.009 | 0.002 | 1.00E-04 | BMI | 0.015 | 0.010 | 0.007 | 0.012 | 0.021 | 0.016 |
| rs9902312 | 17 | 65070304 | T | C | 0.001 | 0.002 | 6.21E-01 | 0.015 | 0.002 | 4.50E-11 | TV | -0.001 | 0.010 | 0.000 | 0.012 | 0.010 | 0.016 |
| rs12602912 | 17 | 65870073 | T | C | 0.017 | 0.002 | 2.90E-16 | 0.011 | 0.003 | 2.80E-05 | BMI | 0.008 | 0.012 | 0.010 | 0.014 | 0.012 | 0.019 |
| rs35867081 | 17 | 79047278 | A | G | 0.015 | 0.002 | 6.65E-15 | -0.004 | 0.002 | 1.00E-01 | BMI | -0.003 | 0.010 | 0.003 | 0.012 | -0.020 | 0.016 |
| rs11657730 | 17 | 79372489 | C | T | 0.003 | 0.002 | 2.17E-01 | 0.013 | 0.002 | 1.10E-08 | TV | 0.010 | 0.011 | 0.010 | 0.012 | 0.016 | 0.016 |
| rs8097672 | 18 | 1839601 | A | T | -0.021 | 0.002 | 3.26E-18 | -0.007 | 0.003 | 2.50E-02 | BMI | -0.010 | 0.014 | 0.001 | 0.016 | -0.020 | 0.021 |
| rs478707 | 18 | 7543207 | T | C | -0.015 | 0.002 | 1.98E-10 | 0.005 | 0.003 | 6.60E-02 | BMI | 0.006 | 0.012 | -0.006 | 0.014 | 0.008 | 0.019 |
| rs303753 | 18 | 21074922 | G | A | -0.020 | 0.002 | 1.84E-27 | -0.014 | 0.002 | 2.70E-10 | TV | -0.013 | 0.010 | -0.008 | 0.012 | -0.022 | 0.016 |
| rs1945160 | 18 | 22164216 | A | G | -0.010 | 0.002 | 5.65E-09 | -0.001 | 0.002 | 7.80E-01 | BMI | -0.003 | 0.010 | 0.004 | 0.012 | -0.009 | 0.016 |
| rs9964724 | 18 | 35159124 | C | T | 0.000 | 0.002 | 9.88E-01 | 0.018 | 0.002 | 3.30E-14 | TV | 0.009 | 0.010 | 0.011 | 0.012 | 0.012 | 0.016 |
| rs1156541 | 18 | 39952989 | C | T | 0.011 | 0.002 | 4.90E-08 | 0.014 | 0.003 | 2.80E-08 | TV | 0.017 | 0.011 | 0.015 | 0.014 | 0.043 | 0.018 |
| rs2612576 | 18 | 42967950 | A | T | -0.011 | 0.002 | 8.49E-09 | -0.004 | 0.002 | 1.10E-01 | BMI | -0.010 | 0.011 | -0.008 | 0.013 | 0.004 | 0.017 |
| rs17512836 | 18 | 53194961 | T | C | 0.016 | 0.006 | 8.62E-03 | 0.042 | 0.007 | 7.00E-10 | TV | -0.014 | 0.028 | 0.019 | 0.033 | -0.075 | 0.043 |
| rs6567160 | 18 | 57829135 | T | C | -0.055 | 0.002 | 7.82E-184 | 0.006 | 0.003 | 2.50E-02 | BMI | -0.019 | 0.011 | -0.011 | 0.014 | -0.023 | 0.018 |
| rs17783165 | 18 | 63461638 | T | C | -0.013 | 0.002 | 2.74E-13 | -0.003 | 0.002 | 2.50E-01 | BMI | -0.007 | 0.010 | -0.009 | 0.012 | 0.008 | 0.016 |
| rs11150911 | 18 | 73498528 | A | C | 0.012 | 0.002 | 3.72E-11 | 0.001 | 0.002 | 7.10E-01 | BMI | -0.005 | 0.010 | -0.013 | 0.013 | 0.008 | 0.016 |
| rs45486197 | 19 | 2244849 | A | G | 0.028 | 0.004 | 1.80E-12 | 0.001 | 0.004 | 9.00E-01 | BMI | -0.002 | 0.024 | -0.015 | 0.028 | -0.054 | 0.037 |
| rs895330 | 19 | 4060707 | C | G | 0.020 | 0.002 | 1.59E-19 | 0.006 | 0.003 | 1.80E-02 | BMI | -0.032 | 0.012 | -0.021 | 0.015 | -0.042 | 0.019 |
| rs7248205 | 19 | 10770305 | C | T | 0.008 | 0.002 | 3.95E-05 | 0.014 | 0.002 | 3.40E-10 | TV | -0.029 | 0.010 | -0.021 | 0.012 | -0.023 | 0.016 |
| rs273512 | 19 | 18224729 | T | C | 0.016 | 0.002 | 4.48E-19 | 0.010 | 0.002 | 4.10E-06 | BMI | -0.008 | 0.010 | -0.009 | 0.012 | 0.004 | 0.015 |
| rs17724992 | 19 | 18454825 | A | G | 0.017 | 0.002 | 5.23E-21 | 0.004 | 0.002 | 1.10E-01 | BMI | 0.006 | 0.011 | 0.000 | 0.013 | 0.007 | 0.017 |
| rs111901094 | 19 | 19513570 | G | T | -0.009 | 0.003 | 2.96E-04 | -0.017 | 0.003 | 2.00E-09 | TV | -0.011 | 0.013 | 0.003 | 0.016 | -0.022 | 0.020 |
| rs12462975 | 19 | 30272202 | A | G | 0.019 | 0.002 | 1.47E-25 | 0.003 | 0.002 | 2.40E-01 | BMI | 0.025 | 0.010 | 0.028 | 0.012 | 0.028 | 0.016 |
| rs7245985 | 19 | 30710410 | T | G | 0.012 | 0.002 | 1.88E-08 | 0.007 | 0.003 | 1.30E-02 | BMI | 0.015 | 0.012 | 0.021 | 0.014 | 0.002 | 0.019 |
| rs11882409 | 19 | 34019685 | A | C | 0.012 | 0.002 | 3.31E-10 | 0.001 | 0.002 | 6.80E-01 | BMI | -0.020 | 0.011 | -0.026 | 0.013 | -0.021 | 0.017 |
| rs185350 | 19 | 34306816 | T | C | 0.014 | 0.002 | 9.10E-17 | 0.004 | 0.002 | 4.40E-02 | BMI | 0.009 | 0.010 | 0.015 | 0.012 | 0.003 | 0.015 |
| rs429358 | 19 | 45411941 | T | C | 0.026 | 0.003 | 2.60E-22 | 0.014 | 0.003 | 3.80E-06 | BMI | 0.005 | 0.014 | 0.017 | 0.017 | 0.007 | 0.022 |
| rs11672660 | 19 | 46180184 | T | C | -0.034 | 0.002 | 6.83E-60 | -0.004 | 0.003 | 1.40E-01 | BMI | 0.005 | 0.012 | 0.002 | 0.014 | 0.007 | 0.019 |
| rs9304665 | 19 | 47602577 | A | T | 0.023 | 0.002 | 2.20E-31 | -0.006 | 0.003 | 1.90E-02 | BMI | -0.003 | 0.012 | -0.005 | 0.014 | 0.001 | 0.018 |
| rs3764625 | 19 | 49649051 | T | G | 0.010 | 0.002 | 2.42E-08 | -0.002 | 0.002 | 3.00E-01 | BMI | 0.005 | 0.010 | 0.001 | 0.012 | -0.015 | 0.015 |
| rs1884389 | 20 | 1410582 | T | C | -0.011 | 0.002 | 3.72E-10 | -0.001 | 0.002 | 5.80E-01 | BMI | -0.002 | 0.010 | 0.006 | 0.012 | -0.015 | 0.015 |
| rs1884897 | 20 | 6612832 | A | G | -0.018 | 0.002 | 2.70E-28 | 0.000 | 0.002 | 8.80E-01 | BMI | 0.053 | 0.010 | 0.037 | 0.012 | 0.063 | 0.016 |
| rs6131281 | 20 | 11891724 | C | T | 0.007 | 0.002 | 5.17E-04 | 0.016 | 0.002 | 3.10E-13 | TV | 0.017 | 0.010 | 0.020 | 0.012 | 0.014 | 0.015 |
| rs852056 | 20 | 17102860 | T | C | 0.012 | 0.002 | 2.15E-10 | 0.002 | 0.003 | 5.40E-01 | BMI | -0.002 | 0.011 | 0.015 | 0.014 | -0.009 | 0.018 |
| rs8122855 | 20 | 25192049 | A | G | 0.014 | 0.002 | 4.11E-14 | 0.000 | 0.002 | 8.80E-01 | BMI | -0.018 | 0.010 | -0.010 | 0.012 | -0.025 | 0.016 |
| rs6141814 | 20 | 31368960 | C | A | 0.002 | 0.002 | 3.74E-01 | -0.014 | 0.002 | 1.30E-09 | TV | 0.013 | 0.010 | 0.015 | 0.012 | 0.003 | 0.016 |
| rs55700114 | 20 | 43717080 | G | A | -0.005 | 0.002 | 1.00E-02 | -0.014 | 0.002 | 2.10E-09 | TV | -0.001 | 0.011 | -0.002 | 0.013 | -0.006 | 0.016 |
| rs17806224 | 20 | 51065854 | A | G | -0.026 | 0.002 | 7.91E-32 | -0.009 | 0.003 | 7.70E-04 | BMI | -0.023 | 0.013 | -0.026 | 0.015 | -0.022 | 0.020 |
| rs1304549 | 20 | 54378256 | A | G | -0.012 | 0.002 | 1.35E-08 | 0.004 | 0.003 | 1.90E-01 | BMI | 0.011 | 0.012 | 0.001 | 0.014 | 0.037 | 0.018 |
| rs56103247 | 20 | 62483184 | C | T | 0.023 | 0.004 | 1.18E-07 | 0.030 | 0.005 | 3.80E-10 | TV | -0.077 | 0.024 | -0.074 | 0.029 | -0.074 | 0.038 |
| rs2064044 | 21 | 22119890 | A | C | -0.012 | 0.002 | 1.02E-08 | 0.005 | 0.003 | 5.50E-02 | BMI | -0.017 | 0.012 | -0.022 | 0.014 | 0.005 | 0.019 |
| rs2832283 | 21 | 30690558 | A | G | 0.012 | 0.002 | 4.72E-09 | 0.005 | 0.003 | 4.70E-02 | BMI | -0.031 | 0.012 | -0.039 | 0.014 | -0.015 | 0.018 |
| rs13047416 | 21 | 40309436 | C | G | 0.015 | 0.002 | 6.93E-18 | 0.002 | 0.002 | 3.80E-01 | BMI | -0.017 | 0.010 | -0.003 | 0.012 | -0.038 | 0.016 |
| rs10427502 | 21 | 40654840 | G | A | 0.012 | 0.002 | 3.56E-09 | 0.014 | 0.002 | 8.60E-10 | TV | 0.001 | 0.010 | -0.006 | 0.012 | 0.000 | 0.016 |
| rs8126575 | 21 | 46435610 | T | G | 0.015 | 0.003 | 5.88E-10 | 0.002 | 0.003 | 5.10E-01 | BMI | -0.006 | 0.015 | -0.009 | 0.018 | -0.001 | 0.024 |
| rs427943 | 21 | 46570896 | A | C | -0.018 | 0.002 | 3.60E-25 | -0.009 | 0.002 | 3.70E-05 | BMI | 0.006 | 0.010 | 0.008 | 0.012 | 0.016 | 0.015 |
| rs11538 | 22 | 18220831 | A | G | -0.014 | 0.002 | 1.09E-09 | -0.004 | 0.003 | 1.20E-01 | BMI | 0.000 | 0.013 | -0.013 | 0.016 | -0.017 | 0.020 |
| rs2238799 | 22 | 20109325 | A | G | 0.010 | 0.002 | 8.65E-09 | 0.002 | 0.002 | 2.80E-01 | BMI | 0.003 | 0.010 | 0.007 | 0.012 | -0.017 | 0.016 |
| rs2092829 | 22 | 31780514 | G | A | -0.005 | 0.002 | 5.75E-03 | 0.014 | 0.002 | 1.30E-08 | TV | -0.023 | 0.011 | -0.026 | 0.013 | -0.029 | 0.017 |
| rs28489620 | 22 | 41804716 | A | G | -0.015 | 0.002 | 1.42E-12 | -0.012 | 0.002 | 1.30E-06 | BMI | 0.020 | 0.011 | 0.025 | 0.013 | 0.022 | 0.017 |
| rs9615337 | 22 | 48898559 | C | G | -0.011 | 0.002 | 2.05E-08 | -0.005 | 0.002 | 2.40E-02 | BMI | 0.000 | 0.010 | -0.005 | 0.012 | 0.019 | 0.016 |
| **2) Analysis of colorectal cancer in men only** | | | | | | | | | | | | | | | | | |
| SNP | CHR | POS | EA | OA | beta_bmi | se_bmi | pval_bmi | beta_tv | se_tv | pval_tv | Phenotype | beta_crc | se_crc |  |  |  |  |
| rs2803316 | 1 | 1865298 | A | G | -0.015 | 0.003 | 4.03E-09 | -0.006 | 0.002 | 6.20E-03 | BMI | 0.016 | 0.014 |  |  |  |  |
| rs3762444 | 1 | 2427712 | T | C | -0.019 | 0.003 | 1.47E-14 | -0.001 | 0.002 | 5.90E-01 | BMI | -0.021 | 0.014 |  |  |  |  |
| rs11121210 | 1 | 8708529 | T | C | -0.015 | 0.003 | 7.86E-09 | -0.003 | 0.002 | 2.40E-01 | BMI | -0.017 | 0.014 |  |  |  |  |
| rs11581010 | 1 | 11212458 | A | G | -0.018 | 0.003 | 1.01E-09 | 0.001 | 0.003 | 8.10E-01 | BMI | 0.003 | 0.016 |  |  |  |  |
| rs2235549 | 1 | 23391775 | T | G | -0.021 | 0.003 | 8.27E-11 | 0.000 | 0.003 | 9.80E-01 | BMI | -0.047 | 0.018 |  |  |  |  |
| rs2271928 | 1 | 32127953 | A | G | -0.014 | 0.002 | 9.92E-09 | 0.002 | 0.002 | 2.70E-01 | BMI | 0.010 | 0.014 |  |  |  |  |
| rs12061406 | 1 | 40073479 | T | C | -0.027 | 0.005 | 1.70E-09 | 0.000 | 0.004 | 9.20E-01 | BMI | 0.033 | 0.026 |  |  |  |  |
| rs11810109 | 1 | 44186812 | A | T | 0.007 | 0.003 | 3.11E-02 | 0.016 | 0.002 | 5.40E-12 | TV | 0.000 | 0.015 |  |  |  |  |
| rs977747 | 1 | 47684677 | T | G | 0.016 | 0.002 | 1.35E-11 | 0.001 | 0.002 | 5.60E-01 | BMI | -0.016 | 0.014 |  |  |  |  |
| rs12725114 | 1 | 62432641 | G | A | 0.015 | 0.004 | 4.97E-05 | 0.015 | 0.003 | 1.60E-08 | TV | -0.023 | 0.017 |  |  |  |  |
| rs984409 | 1 | 67020440 | G | A | 0.002 | 0.003 | 5.13E-01 | -0.015 | 0.002 | 4.10E-11 | TV | -0.010 | 0.015 |  |  |  |  |
| rs71658797 | 1 | 77967507 | T | A | -0.036 | 0.005 | 1.04E-15 | -0.020 | 0.003 | 6.30E-10 | TV | -0.002 | 0.023 |  |  |  |  |
| rs114328297 | 1 | 91190854 | T | G | -0.003 | 0.004 | 3.45E-01 | 0.014 | 0.003 | 4.40E-08 | TV | 0.018 | 0.017 |  |  |  |  |
| rs17379561 | 1 | 98340139 | A | T | -0.010 | 0.004 | 5.83E-03 | -0.025 | 0.003 | 1.10E-16 | TV | 0.004 | 0.020 |  |  |  |  |
| rs55909997 | 1 | 107581039 | G | A | -0.011 | 0.003 | 2.87E-05 | -0.014 | 0.002 | 2.40E-09 | TV | -0.037 | 0.014 |  |  |  |  |
| rs1278847 | 1 | 110037483 | C | A | -0.013 | 0.003 | 1.77E-05 | 0.016 | 0.002 | 1.20E-11 | TV | 0.008 | 0.015 |  |  |  |  |
| rs4845364 | 1 | 154141908 | A | G | 0.000 | 0.003 | 9.54E-01 | -0.015 | 0.002 | 1.30E-12 | TV | -0.014 | 0.014 |  |  |  |  |
| rs61813324 | 1 | 156049877 | T | C | 0.028 | 0.004 | 1.29E-10 | 0.015 | 0.003 | 4.30E-06 | BMI | 0.037 | 0.024 |  |  |  |  |
| rs76194251 | 1 | 173931908 | C | G | -0.060 | 0.010 | 4.04E-10 | -0.025 | 0.007 | 6.20E-04 | BMI | 0.024 | 0.046 |  |  |  |  |
| rs6673341 | 1 | 184655649 | T | G | -0.003 | 0.003 | 2.03E-01 | -0.015 | 0.002 | 2.20E-11 | TV | -0.012 | 0.014 |  |  |  |  |
| rs10737620 | 1 | 193057971 | T | A | 0.006 | 0.003 | 2.31E-02 | 0.014 | 0.002 | 2.60E-09 | TV | -0.008 | 0.015 |  |  |  |  |
| rs2172935 | 1 | 201826340 | T | C | 0.027 | 0.003 | 3.56E-24 | 0.010 | 0.002 | 7.20E-06 | BMI | 0.031 | 0.015 |  |  |  |  |
| rs12217091 | 1 | 204813050 | T | C | 0.027 | 0.005 | 4.44E-08 | 0.004 | 0.004 | 2.90E-01 | BMI | -0.015 | 0.023 |  |  |  |  |
| rs823094 | 1 | 205689807 | T | G | 0.015 | 0.002 | 3.77E-10 | 0.001 | 0.002 | 5.50E-01 | BMI | 0.041 | 0.014 |  |  |  |  |
| rs4653942 | 1 | 228505204 | A | G | -0.016 | 0.003 | 4.41E-08 | 0.005 | 0.003 | 6.70E-02 | BMI | 0.016 | 0.016 |  |  |  |  |
| rs2125231 | 1 | 243762299 | T | C | 0.017 | 0.003 | 5.50E-11 | -0.004 | 0.002 | 5.50E-02 | BMI | -0.005 | 0.014 |  |  |  |  |
| rs76111507 | 2 | 455477 | T | C | -0.081 | 0.008 | 1.13E-25 | 0.003 | 0.006 | 6.20E-01 | BMI | -0.038 | 0.042 |  |  |  |  |
| rs12714415 | 2 | 651430 | T | C | 0.060 | 0.003 | 1.09E-80 | 0.005 | 0.003 | 1.20E-01 | BMI | 0.031 | 0.018 |  |  |  |  |
| rs2693826 | 2 | 6160943 | A | G | -0.016 | 0.003 | 4.96E-11 | -0.003 | 0.002 | 1.10E-01 | BMI | -0.006 | 0.014 |  |  |  |  |
| rs72781699 | 2 | 24277709 | G | A | 0.002 | 0.004 | 5.00E-01 | -0.019 | 0.003 | 3.00E-12 | TV | 0.008 | 0.017 |  |  |  |  |
| rs2888172 | 2 | 35439812 | A | G | -0.014 | 0.003 | 1.42E-08 | -0.001 | 0.002 | 7.20E-01 | BMI | 0.008 | 0.014 |  |  |  |  |
| rs10185199 | 2 | 40282202 | A | G | -0.016 | 0.003 | 3.79E-08 | -0.006 | 0.002 | 1.00E-02 | BMI | 0.007 | 0.016 |  |  |  |  |
| rs12105701 | 2 | 41737200 | C | T | -0.010 | 0.003 | 8.44E-05 | -0.013 | 0.002 | 5.40E-09 | TV | 0.013 | 0.014 |  |  |  |  |
| rs7561278 | 2 | 48954905 | T | C | 0.018 | 0.003 | 1.06E-08 | 0.003 | 0.003 | 1.90E-01 | BMI | -0.004 | 0.017 |  |  |  |  |
| rs7564130 | 2 | 50606642 | T | C | -0.002 | 0.003 | 3.59E-01 | -0.015 | 0.002 | 2.80E-11 | TV | -0.019 | 0.014 |  |  |  |  |
| rs887912 | 2 | 59302877 | T | C | 0.023 | 0.003 | 2.75E-19 | 0.000 | 0.002 | 9.40E-01 | BMI | 0.003 | 0.015 |  |  |  |  |
| rs10189857 | 2 | 60713235 | A | G | -0.009 | 0.003 | 3.02E-04 | -0.020 | 0.002 | 6.20E-21 | TV | -0.022 | 0.014 |  |  |  |  |
| rs62641636 | 2 | 68425427 | A | G | 0.010 | 0.003 | 1.29E-03 | 0.014 | 0.002 | 7.00E-10 | TV | 0.000 | 0.015 |  |  |  |  |
| rs10209325 | 2 | 86806356 | T | C | -0.018 | 0.003 | 1.49E-09 | -0.002 | 0.002 | 3.90E-01 | BMI | -0.009 | 0.014 |  |  |  |  |
| rs11689199 | 2 | 100820421 | A | G | 0.014 | 0.003 | 7.23E-08 | 0.018 | 0.002 | 5.50E-17 | TV | 0.039 | 0.014 |  |  |  |  |
| rs1451533 | 2 | 105466005 | G | A | -0.017 | 0.003 | 1.15E-09 | -0.016 | 0.002 | 1.10E-10 | TV | 0.009 | 0.015 |  |  |  |  |
| rs13405033 | 2 | 113994595 | T | C | 0.026 | 0.004 | 4.37E-11 | 0.004 | 0.003 | 2.40E-01 | BMI | 0.046 | 0.020 |  |  |  |  |
| rs12476388 | 2 | 116330983 | C | T | -0.001 | 0.003 | 6.84E-01 | 0.013 | 0.002 | 2.10E-08 | TV | -0.005 | 0.015 |  |  |  |  |
| rs11687120 | 2 | 119579558 | A | G | 0.014 | 0.003 | 4.74E-08 | -0.006 | 0.002 | 8.00E-03 | BMI | -0.017 | 0.014 |  |  |  |  |
| rs961360 | 2 | 136393658 | A | G | 0.021 | 0.003 | 3.75E-10 | 0.015 | 0.003 | 2.40E-05 | BMI | -0.023 | 0.017 |  |  |  |  |
| rs374722 | 2 | 147839830 | G | A | 0.016 | 0.004 | 1.99E-06 | 0.024 | 0.003 | 5.50E-16 | TV | 0.002 | 0.019 |  |  |  |  |
| rs3754970 | 2 | 162091836 | T | C | -0.009 | 0.003 | 2.10E-03 | -0.015 | 0.002 | 4.80E-12 | TV | 0.003 | 0.014 |  |  |  |  |
| rs2060199 | 2 | 166196681 | T | A | 0.008 | 0.003 | 2.39E-03 | 0.012 | 0.002 | 2.40E-08 | TV | 0.039 | 0.014 |  |  |  |  |
| rs34234296 | 2 | 175166636 | A | G | -0.018 | 0.003 | 2.86E-09 | -0.004 | 0.002 | 1.10E-01 | BMI | -0.011 | 0.015 |  |  |  |  |
| rs1019612 | 2 | 181602526 | T | C | 0.018 | 0.003 | 1.40E-11 | 0.008 | 0.002 | 5.70E-04 | BMI | 0.004 | 0.014 |  |  |  |  |
| rs4577309 | 2 | 191288833 | A | G | 0.001 | 0.003 | 7.52E-01 | 0.016 | 0.002 | 1.60E-13 | TV | 0.015 | 0.014 |  |  |  |  |
| rs34224261 | 2 | 193855452 | G | C | -0.009 | 0.003 | 2.01E-03 | -0.018 | 0.002 | 4.60E-16 | TV | -0.002 | 0.014 |  |  |  |  |
| rs4675246 | 2 | 202864487 | G | T | 0.002 | 0.003 | 5.96E-01 | -0.015 | 0.003 | 2.90E-08 | TV | 0.043 | 0.018 |  |  |  |  |
| rs13029509 | 2 | 215374209 | G | A | -0.005 | 0.003 | 2.91E-02 | -0.018 | 0.002 | 2.00E-17 | TV | -0.007 | 0.014 |  |  |  |  |
| rs10932837 | 2 | 221044785 | C | T | 0.004 | 0.003 | 8.62E-02 | -0.013 | 0.002 | 1.30E-09 | TV | 0.013 | 0.014 |  |  |  |  |
| rs4973576 | 2 | 233806771 | C | A | 0.007 | 0.003 | 2.79E-02 | -0.015 | 0.002 | 1.00E-09 | TV | -0.005 | 0.015 |  |  |  |  |
| rs138256022 | 3 | 11603090 | C | T | 0.005 | 0.008 | 5.23E-01 | -0.031 | 0.006 | 2.90E-08 | TV | 0.025 | 0.045 |  |  |  |  |
| rs2596902 | 3 | 12955982 | A | G | -0.015 | 0.003 | 1.30E-08 | -0.003 | 0.002 | 2.50E-01 | BMI | 0.011 | 0.014 |  |  |  |  |
| rs6804842 | 3 | 25106437 | A | G | -0.018 | 0.002 | 1.29E-14 | -0.001 | 0.002 | 5.60E-01 | BMI | 0.012 | 0.014 |  |  |  |  |
| rs9834970 | 3 | 36856030 | T | C | 0.008 | 0.003 | 2.09E-03 | 0.013 | 0.002 | 3.30E-09 | TV | 0.002 | 0.014 |  |  |  |  |
| rs762318 | 3 | 38516369 | A | C | 0.015 | 0.003 | 2.13E-09 | 0.003 | 0.002 | 1.60E-01 | BMI | -0.001 | 0.014 |  |  |  |  |
| rs1524887 | 3 | 44016371 | T | C | -0.014 | 0.003 | 3.60E-08 | -0.008 | 0.002 | 5.00E-04 | BMI | 0.017 | 0.014 |  |  |  |  |
| rs3796386 | 3 | 49899795 | G | A | -0.019 | 0.003 | 1.15E-13 | -0.026 | 0.002 | 3.20E-33 | TV | -0.014 | 0.014 |  |  |  |  |
| rs11130793 | 3 | 60884659 | C | T | 0.005 | 0.003 | 9.55E-02 | 0.013 | 0.002 | 5.00E-09 | TV | 0.018 | 0.014 |  |  |  |  |
| rs11714337 | 3 | 71582521 | G | A | 0.006 | 0.003 | 4.17E-02 | 0.014 | 0.002 | 4.70E-11 | TV | -0.010 | 0.014 |  |  |  |  |
| rs3849570 | 3 | 81792112 | A | C | 0.015 | 0.003 | 2.56E-09 | 0.000 | 0.002 | 9.20E-01 | BMI | -0.007 | 0.014 |  |  |  |  |
| rs6797840 | 3 | 85656569 | A | C | -0.017 | 0.003 | 2.24E-09 | -0.016 | 0.002 | 1.70E-13 | TV | -0.004 | 0.014 |  |  |  |  |
| rs2034768 | 3 | 93986371 | A | G | 0.021 | 0.003 | 2.43E-13 | 0.015 | 0.002 | 8.60E-12 | TV | -0.017 | 0.014 |  |  |  |  |
| rs1436351 | 3 | 104617973 | T | G | 0.018 | 0.003 | 3.39E-11 | 0.005 | 0.002 | 3.20E-02 | BMI | -0.004 | 0.016 |  |  |  |  |
| rs9867121 | 3 | 114631548 | C | A | 0.005 | 0.004 | 1.63E-01 | 0.019 | 0.003 | 3.90E-12 | TV | 0.041 | 0.017 |  |  |  |  |
| rs2918217 | 3 | 115546380 | T | C | 0.022 | 0.004 | 7.18E-10 | 0.004 | 0.003 | 2.40E-01 | BMI | -0.023 | 0.020 |  |  |  |  |
| rs1320903 | 3 | 131758077 | A | G | 0.026 | 0.003 | 1.65E-21 | 0.001 | 0.002 | 5.40E-01 | BMI | 0.003 | 0.015 |  |  |  |  |
| rs645040 | 3 | 135926622 | T | G | 0.019 | 0.003 | 3.16E-11 | 0.004 | 0.003 | 9.70E-02 | BMI | -0.002 | 0.016 |  |  |  |  |
| rs2035935 | 3 | 141306013 | A | G | -0.033 | 0.005 | 3.99E-13 | -0.014 | 0.004 | 1.70E-03 | BMI | 0.031 | 0.026 |  |  |  |  |
| rs355777 | 3 | 154034950 | C | G | 0.017 | 0.003 | 1.81E-11 | 0.003 | 0.002 | 1.70E-01 | BMI | -0.008 | 0.014 |  |  |  |  |
| rs12491503 | 3 | 165706855 | G | A | -0.001 | 0.003 | 6.70E-01 | -0.014 | 0.002 | 5.50E-10 | TV | -0.011 | 0.014 |  |  |  |  |
| rs114600294 | 3 | 181419367 | G | C | -0.005 | 0.004 | 2.08E-01 | -0.016 | 0.003 | 7.90E-10 | TV | 0.022 | 0.020 |  |  |  |  |
| rs9816226 | 3 | 185834499 | A | T | -0.034 | 0.003 | 5.05E-29 | 0.007 | 0.003 | 8.70E-03 | BMI | 0.028 | 0.018 |  |  |  |  |
| rs66852340 | 4 | 3311070 | C | T | -0.010 | 0.003 | 5.26E-04 | -0.018 | 0.003 | 7.90E-12 | TV | -0.013 | 0.016 |  |  |  |  |
| rs7700107 | 4 | 17880416 | A | C | -0.003 | 0.004 | 3.59E-01 | -0.021 | 0.003 | 1.90E-11 | TV | -0.025 | 0.019 |  |  |  |  |
| rs2242189 | 4 | 38691024 | T | C | 0.017 | 0.003 | 5.55E-11 | 0.001 | 0.002 | 5.40E-01 | BMI | -0.009 | 0.015 |  |  |  |  |
| rs13130484 | 4 | 45175691 | T | C | 0.035 | 0.002 | 6.44E-50 | 0.011 | 0.002 | 3.70E-07 | BMI | -0.017 | 0.014 |  |  |  |  |
| rs28971796 | 4 | 49151982 | A | G | 0.010 | 0.003 | 1.39E-03 | 0.015 | 0.002 | 2.10E-09 | TV | 0.004 | 0.017 |  |  |  |  |
| rs6850494 | 4 | 82291771 | A | C | -0.002 | 0.003 | 4.67E-01 | -0.014 | 0.002 | 1.10E-10 | TV | -0.019 | 0.014 |  |  |  |  |
| rs13107325 | 4 | 103188709 | T | C | 0.050 | 0.005 | 3.22E-26 | 0.029 | 0.004 | 1.50E-12 | both | 0.009 | 0.026 |  |  |  |  |
| rs7693703 | 4 | 118357405 | G | A | 0.005 | 0.004 | 2.19E-01 | 0.023 | 0.004 | 2.70E-09 | TV | -0.004 | 0.024 |  |  |  |  |
| rs4334769 | 4 | 130245233 | G | T | 0.000 | 0.003 | 9.22E-01 | 0.012 | 0.002 | 2.10E-08 | TV | -0.010 | 0.014 |  |  |  |  |
| rs1296328 | 4 | 137083193 | A | C | 0.020 | 0.003 | 2.07E-15 | 0.006 | 0.002 | 1.00E-02 | BMI | -0.028 | 0.014 |  |  |  |  |
| rs6825241 | 4 | 152543783 | C | A | -0.004 | 0.003 | 1.02E-01 | -0.017 | 0.002 | 4.90E-15 | TV | -0.003 | 0.014 |  |  |  |  |
| rs7693082 | 4 | 159857819 | G | C | 0.003 | 0.003 | 3.51E-01 | 0.015 | 0.002 | 1.70E-10 | TV | 0.012 | 0.015 |  |  |  |  |
| rs828544 | 5 | 3520064 | T | G | 0.014 | 0.003 | 4.45E-08 | -0.003 | 0.002 | 1.60E-01 | BMI | 0.001 | 0.014 |  |  |  |  |
| rs10940659 | 5 | 59360341 | A | G | -0.008 | 0.003 | 2.66E-03 | -0.013 | 0.002 | 1.50E-08 | TV | -0.027 | 0.014 |  |  |  |  |
| rs262890 | 5 | 62930015 | A | G | -0.014 | 0.003 | 8.98E-06 | -0.019 | 0.002 | 3.20E-15 | TV | 0.036 | 0.015 |  |  |  |  |
| rs253414 | 5 | 74956517 | T | C | 0.026 | 0.003 | 1.10E-24 | 0.007 | 0.002 | 1.40E-03 | BMI | 0.008 | 0.014 |  |  |  |  |
| rs4532349 | 5 | 77473165 | A | G | 0.016 | 0.003 | 6.64E-09 | 0.012 | 0.003 | 4.60E-06 | BMI | -0.017 | 0.017 |  |  |  |  |
| rs7716447 | 5 | 88800331 | A | G | -0.014 | 0.003 | 1.40E-07 | -0.013 | 0.002 | 3.60E-09 | TV | -0.018 | 0.014 |  |  |  |  |
| rs1031423 | 5 | 93276883 | T | C | -0.001 | 0.003 | 7.28E-01 | -0.019 | 0.003 | 1.80E-12 | TV | 0.023 | 0.017 |  |  |  |  |
| rs57585211 | 5 | 107197725 | T | G | -0.005 | 0.004 | 1.72E-01 | -0.017 | 0.003 | 4.30E-09 | TV | -0.020 | 0.018 |  |  |  |  |
| rs289228 | 5 | 107405834 | C | G | 0.022 | 0.003 | 9.78E-12 | 0.001 | 0.003 | 8.10E-01 | BMI | 0.010 | 0.018 |  |  |  |  |
| rs9326862 | 5 | 112054547 | A | T | 0.004 | 0.003 | 9.99E-02 | 0.012 | 0.002 | 2.60E-08 | TV | -0.011 | 0.014 |  |  |  |  |
| rs7711753 | 5 | 122733317 | A | G | -0.013 | 0.002 | 2.64E-08 | 0.007 | 0.002 | 1.10E-03 | BMI | 0.013 | 0.014 |  |  |  |  |
| rs10041724 | 5 | 124273520 | T | C | 0.008 | 0.003 | 8.30E-03 | 0.018 | 0.003 | 3.90E-11 | TV | 0.021 | 0.017 |  |  |  |  |
| rs62379379 | 5 | 141082015 | G | T | 0.003 | 0.006 | 6.20E-01 | -0.026 | 0.004 | 7.80E-10 | TV | -0.025 | 0.027 |  |  |  |  |
| rs10054327 | 5 | 147871137 | G | A | 0.002 | 0.003 | 4.71E-01 | 0.017 | 0.002 | 3.40E-15 | TV | 0.003 | 0.014 |  |  |  |  |
| rs42210 | 5 | 166408788 | G | C | -0.003 | 0.003 | 2.94E-01 | -0.014 | 0.002 | 7.30E-09 | TV | -0.016 | 0.015 |  |  |  |  |
| rs72828890 | 5 | 167806836 | C | T | 0.005 | 0.005 | 2.90E-01 | 0.019 | 0.003 | 4.90E-09 | TV | 0.026 | 0.023 |  |  |  |  |
| rs7730898 | 5 | 170459675 | A | G | 0.017 | 0.003 | 9.94E-11 | 0.008 | 0.002 | 1.50E-03 | BMI | 0.001 | 0.015 |  |  |  |  |
| rs2228213 | 6 | 12124855 | A | G | -0.015 | 0.003 | 6.77E-10 | -0.005 | 0.002 | 3.30E-02 | BMI | -0.011 | 0.014 |  |  |  |  |
| rs72834698 | 6 | 26176517 | G | A | 0.020 | 0.004 | 2.97E-06 | 0.023 | 0.003 | 2.70E-13 | TV | 0.060 | 0.020 |  |  |  |  |
| rs9277992 | 6 | 33312455 | A | G | 0.028 | 0.004 | 8.69E-14 | 0.013 | 0.003 | 2.30E-06 | BMI | -0.011 | 0.018 |  |  |  |  |
| rs9471333 | 6 | 40362023 | C | T | 0.023 | 0.003 | 4.20E-15 | 0.013 | 0.002 | 1.50E-09 | TV | 0.020 | 0.014 |  |  |  |  |
| rs2206277 | 6 | 50798526 | T | C | 0.045 | 0.003 | 5.35E-50 | 0.001 | 0.003 | 6.00E-01 | BMI | -0.004 | 0.017 |  |  |  |  |
| rs1327259 | 6 | 51177811 | A | G | 0.016 | 0.003 | 5.96E-10 | 0.001 | 0.002 | 6.60E-01 | BMI | 0.015 | 0.014 |  |  |  |  |
| rs4523073 | 6 | 67550288 | A | G | -0.001 | 0.003 | 8.57E-01 | -0.014 | 0.002 | 5.00E-10 | TV | 0.002 | 0.014 |  |  |  |  |
| rs7773045 | 6 | 81443423 | T | G | 0.026 | 0.004 | 1.60E-09 | -0.002 | 0.004 | 6.20E-01 | BMI | -0.003 | 0.027 |  |  |  |  |
| rs12663742 | 6 | 97792735 | A | G | -0.018 | 0.003 | 1.69E-12 | -0.003 | 0.002 | 1.20E-01 | BMI | -0.006 | 0.014 |  |  |  |  |
| rs6905544 | 6 | 98411631 | A | G | -0.020 | 0.003 | 5.21E-15 | -0.019 | 0.002 | 8.50E-18 | TV | -0.037 | 0.014 |  |  |  |  |
| rs17789218 | 6 | 100600097 | T | C | -0.015 | 0.003 | 3.33E-08 | 0.019 | 0.003 | 1.40E-13 | both | 0.016 | 0.016 |  |  |  |  |
| rs768023 | 6 | 108876002 | A | G | 0.013 | 0.002 | 3.05E-08 | 0.005 | 0.002 | 2.00E-02 | BMI | 0.028 | 0.014 |  |  |  |  |
| rs1159974 | 6 | 126090277 | T | C | -0.014 | 0.003 | 2.03E-08 | 0.000 | 0.002 | 8.80E-01 | BMI | -0.018 | 0.014 |  |  |  |  |
| rs6569648 | 6 | 130349119 | T | C | -0.015 | 0.003 | 3.66E-08 | -0.002 | 0.003 | 3.40E-01 | BMI | 0.025 | 0.016 |  |  |  |  |
| rs2184364 | 6 | 143183596 | A | G | 0.007 | 0.003 | 3.28E-02 | 0.016 | 0.003 | 3.00E-09 | TV | 0.025 | 0.017 |  |  |  |  |
| rs13191362 | 6 | 163033350 | A | G | 0.027 | 0.004 | 2.34E-13 | 0.001 | 0.003 | 7.40E-01 | BMI | 0.023 | 0.021 |  |  |  |  |
| rs9718104 | 6 | 166170539 | T | G | -0.004 | 0.006 | 5.44E-01 | -0.041 | 0.005 | 9.30E-19 | TV | 0.002 | 0.032 |  |  |  |  |
| rs17568389 | 7 | 8091876 | T | A | 0.005 | 0.003 | 2.94E-02 | 0.015 | 0.002 | 8.60E-13 | TV | 0.012 | 0.014 |  |  |  |  |
| rs10234444 | 7 | 41828527 | G | A | 0.005 | 0.003 | 1.33E-01 | 0.016 | 0.003 | 2.90E-08 | TV | -0.018 | 0.018 |  |  |  |  |
| rs1167827 | 7 | 75163169 | A | G | -0.022 | 0.002 | 2.88E-19 | -0.008 | 0.002 | 2.10E-04 | BMI | -0.020 | 0.014 |  |  |  |  |
| rs2245368 | 7 | 76608143 | T | C | -0.033 | 0.003 | 4.39E-23 | 0.004 | 0.003 | 1.40E-01 | BMI | -0.010 | 0.018 |  |  |  |  |
| rs6973656 | 7 | 77422583 | A | G | -0.009 | 0.003 | 7.23E-04 | -0.013 | 0.002 | 1.00E-09 | TV | 0.025 | 0.014 |  |  |  |  |
| rs1965529 | 7 | 77825707 | A | G | 0.018 | 0.003 | 4.22E-08 | 0.008 | 0.003 | 2.30E-03 | BMI | -0.004 | 0.017 |  |  |  |  |
| rs973734 | 7 | 89387578 | C | A | 0.007 | 0.004 | 5.80E-02 | 0.017 | 0.003 | 1.50E-08 | TV | -0.022 | 0.020 |  |  |  |  |
| rs62471080 | 7 | 109128060 | G | C | -0.009 | 0.003 | 1.55E-03 | -0.013 | 0.002 | 3.70E-09 | TV | -0.007 | 0.014 |  |  |  |  |
| rs1701829 | 7 | 113033426 | A | G | 0.021 | 0.003 | 6.76E-16 | 0.012 | 0.002 | 1.70E-07 | BMI | 0.000 | 0.015 |  |  |  |  |
| rs10246289 | 7 | 115544763 | A | G | 0.009 | 0.004 | 4.56E-02 | 0.019 | 0.003 | 1.70E-08 | TV | 0.039 | 0.022 |  |  |  |  |
| rs11763734 | 7 | 126400910 | A | C | -0.005 | 0.003 | 4.36E-02 | -0.012 | 0.002 | 8.20E-09 | TV | 0.001 | 0.014 |  |  |  |  |
| rs13228237 | 7 | 138780222 | C | G | -0.016 | 0.003 | 4.59E-08 | -0.003 | 0.003 | 2.60E-01 | BMI | -0.018 | 0.017 |  |  |  |  |
| rs7834121 | 8 | 10770255 | G | T | 0.017 | 0.003 | 1.56E-09 | -0.014 | 0.002 | 1.10E-10 | TV | 0.015 | 0.015 |  |  |  |  |
| rs4123853 | 8 | 14091025 | T | C | 0.017 | 0.003 | 1.10E-10 | -0.006 | 0.002 | 1.30E-02 | BMI | 0.005 | 0.014 |  |  |  |  |
| rs354510 | 8 | 15534749 | T | C | 0.024 | 0.004 | 1.98E-08 | -0.003 | 0.003 | 4.30E-01 | BMI | -0.024 | 0.020 |  |  |  |  |
| rs1421334 | 8 | 30865733 | A | C | 0.016 | 0.003 | 1.03E-10 | 0.017 | 0.002 | 2.20E-15 | TV | 0.017 | 0.014 |  |  |  |  |
| rs6996198 | 8 | 65463442 | C | T | -0.014 | 0.003 | 7.41E-05 | -0.016 | 0.003 | 3.50E-08 | TV | -0.009 | 0.018 |  |  |  |  |
| rs6472942 | 8 | 76827190 | T | C | 0.016 | 0.003 | 1.76E-10 | -0.013 | 0.002 | 1.80E-09 | TV | -0.006 | 0.014 |  |  |  |  |
| rs11985394 | 8 | 92667521 | T | A | 0.000 | 0.003 | 8.98E-01 | 0.012 | 0.002 | 1.30E-08 | TV | 0.017 | 0.014 |  |  |  |  |
| rs2960151 | 8 | 116718620 | A | C | 0.023 | 0.003 | 3.22E-11 | 0.007 | 0.003 | 9.40E-03 | BMI | 0.029 | 0.016 |  |  |  |  |
| rs12541615 | 8 | 118870151 | T | C | -0.020 | 0.004 | 6.59E-08 | -0.018 | 0.003 | 3.90E-10 | TV | 0.001 | 0.017 |  |  |  |  |
| rs7826388 | 8 | 128109776 | T | C | -0.037 | 0.007 | 1.40E-08 | 0.006 | 0.006 | 3.40E-01 | BMI | -0.028 | 0.035 |  |  |  |  |
| rs2717559 | 8 | 143882420 | A | G | 0.005 | 0.003 | 1.27E-01 | 0.012 | 0.002 | 1.50E-08 | TV | -0.018 | 0.014 |  |  |  |  |
| rs2616830 | 9 | 1721385 | G | A | -0.003 | 0.003 | 2.90E-01 | 0.016 | 0.002 | 2.90E-14 | TV | 0.014 | 0.014 |  |  |  |  |
| rs34864022 | 9 | 22609110 | A | G | 0.001 | 0.006 | 8.62E-01 | -0.026 | 0.004 | 1.40E-09 | TV | 0.033 | 0.030 |  |  |  |  |
| rs12554512 | 9 | 23352293 | T | C | -0.007 | 0.003 | 1.07E-02 | 0.021 | 0.002 | 3.80E-21 | TV | -0.001 | 0.014 |  |  |  |  |
| rs2791574 | 9 | 82212094 | T | G | -0.003 | 0.003 | 2.65E-01 | -0.013 | 0.002 | 3.00E-08 | TV | -0.017 | 0.014 |  |  |  |  |
| rs6559921 | 9 | 88872039 | A | T | 0.018 | 0.003 | 1.19E-09 | 0.001 | 0.003 | 8.40E-01 | BMI | 0.006 | 0.017 |  |  |  |  |
| rs870151 | 9 | 92230747 | T | A | -0.007 | 0.003 | 6.68E-03 | -0.016 | 0.002 | 8.80E-13 | TV | 0.005 | 0.014 |  |  |  |  |
| rs2787374 | 9 | 103054951 | T | C | 0.009 | 0.003 | 6.60E-04 | 0.012 | 0.002 | 1.20E-08 | TV | -0.002 | 0.014 |  |  |  |  |
| rs7038943 | 9 | 120377178 | T | C | 0.015 | 0.003 | 6.54E-09 | 0.007 | 0.002 | 2.90E-03 | BMI | 0.038 | 0.014 |  |  |  |  |
| rs7043521 | 9 | 124602779 | A | T | 0.008 | 0.003 | 7.13E-03 | 0.014 | 0.002 | 6.50E-11 | TV | -0.014 | 0.014 |  |  |  |  |
| rs4382592 | 9 | 134870755 | T | G | 0.010 | 0.003 | 2.84E-04 | 0.014 | 0.002 | 6.70E-09 | TV | 0.013 | 0.015 |  |  |  |  |
| rs2073869 | 9 | 135763816 | C | T | 0.007 | 0.003 | 3.42E-02 | 0.019 | 0.003 | 1.50E-10 | TV | -0.002 | 0.018 |  |  |  |  |
| rs11251291 | 10 | 2561766 | A | C | -0.014 | 0.003 | 1.65E-08 | 0.000 | 0.002 | 9.70E-01 | BMI | -0.010 | 0.014 |  |  |  |  |
| rs1623025 | 10 | 11164813 | G | C | -0.005 | 0.003 | 8.37E-02 | -0.012 | 0.002 | 1.40E-08 | TV | 0.019 | 0.014 |  |  |  |  |
| rs1243182 | 10 | 21916728 | C | T | -0.015 | 0.003 | 6.12E-08 | -0.019 | 0.002 | 2.00E-15 | TV | -0.039 | 0.015 |  |  |  |  |
| rs2045147 | 10 | 56660437 | A | G | 0.008 | 0.003 | 1.69E-03 | 0.013 | 0.002 | 5.90E-09 | TV | 0.018 | 0.014 |  |  |  |  |
| rs10994943 | 10 | 63591413 | T | G | -0.001 | 0.003 | 8.58E-01 | 0.013 | 0.002 | 3.10E-09 | TV | 0.017 | 0.014 |  |  |  |  |
| rs7072873 | 10 | 77210191 | T | C | -0.015 | 0.003 | 5.45E-09 | -0.002 | 0.002 | 4.50E-01 | BMI | -0.013 | 0.014 |  |  |  |  |
| rs11201422 | 10 | 86924483 | T | C | 0.003 | 0.003 | 2.01E-01 | 0.013 | 0.002 | 3.40E-08 | TV | 0.005 | 0.015 |  |  |  |  |
| rs17094222 | 10 | 102395440 | T | C | -0.026 | 0.003 | 1.14E-18 | 0.002 | 0.003 | 5.00E-01 | BMI | -0.012 | 0.017 |  |  |  |  |
| rs10786658 | 10 | 103737193 | A | T | -0.003 | 0.003 | 3.99E-01 | -0.014 | 0.002 | 4.60E-11 | TV | 0.011 | 0.014 |  |  |  |  |
| rs7089973 | 10 | 116569565 | C | A | -0.001 | 0.003 | 8.12E-01 | -0.013 | 0.002 | 2.40E-09 | TV | -0.022 | 0.015 |  |  |  |  |
| rs11245482 | 10 | 126733546 | T | C | -0.007 | 0.003 | 9.03E-03 | -0.013 | 0.002 | 2.60E-09 | TV | 0.006 | 0.014 |  |  |  |  |
| rs17727474 | 10 | 127182959 | C | T | 0.006 | 0.003 | 9.72E-02 | 0.018 | 0.003 | 3.10E-09 | TV | -0.001 | 0.019 |  |  |  |  |
| rs7113874 | 11 | 8688016 | T | C | -0.014 | 0.002 | 3.30E-09 | -0.003 | 0.002 | 2.20E-01 | BMI | 0.000 | 0.014 |  |  |  |  |
| rs12289262 | 11 | 12894758 | C | T | -0.005 | 0.003 | 7.18E-02 | -0.014 | 0.002 | 2.00E-08 | TV | -0.029 | 0.016 |  |  |  |  |
| rs900144 | 11 | 13294268 | T | C | 0.015 | 0.003 | 5.54E-10 | 0.006 | 0.002 | 3.40E-03 | BMI | -0.017 | 0.014 |  |  |  |  |
| rs6265 | 11 | 27679916 | T | C | -0.048 | 0.003 | 6.25E-58 | 0.003 | 0.003 | 2.20E-01 | BMI | -0.017 | 0.017 |  |  |  |  |
| rs1765139 | 11 | 30353725 | T | C | 0.022 | 0.003 | 1.80E-13 | 0.008 | 0.003 | 1.40E-03 | BMI | 0.001 | 0.017 |  |  |  |  |
| rs2862996 | 11 | 43653833 | T | G | -0.020 | 0.003 | 2.49E-15 | -0.004 | 0.002 | 9.40E-02 | BMI | 0.001 | 0.015 |  |  |  |  |
| rs7124681 | 11 | 47529947 | A | C | 0.028 | 0.002 | 5.10E-31 | 0.004 | 0.002 | 5.10E-02 | BMI | 0.013 | 0.014 |  |  |  |  |
| rs801733 | 11 | 65934549 | A | C | 0.014 | 0.003 | 3.00E-08 | 0.017 | 0.002 | 7.30E-14 | TV | 0.028 | 0.014 |  |  |  |  |
| rs116175338 | 11 | 72467875 | G | T | -0.017 | 0.003 | 3.38E-10 | -0.014 | 0.003 | 1.40E-08 | TV | 0.011 | 0.016 |  |  |  |  |
| rs12272012 | 11 | 84861286 | G | C | -0.007 | 0.007 | 3.08E-01 | -0.030 | 0.005 | 1.90E-08 | TV | 0.000 | 0.034 |  |  |  |  |
| rs647248 | 11 | 89917699 | A | G | 0.016 | 0.003 | 2.59E-10 | 0.003 | 0.002 | 1.60E-01 | BMI | -0.023 | 0.014 |  |  |  |  |
| rs11020045 | 11 | 92487907 | A | C | -0.005 | 0.003 | 7.87E-02 | -0.013 | 0.002 | 1.60E-08 | TV | 0.021 | 0.015 |  |  |  |  |
| rs17207890 | 11 | 95490754 | G | A | 0.004 | 0.003 | 9.60E-02 | 0.016 | 0.002 | 6.70E-12 | TV | 0.012 | 0.015 |  |  |  |  |
| rs12286929 | 11 | 115022404 | A | G | -0.018 | 0.002 | 2.34E-14 | -0.006 | 0.002 | 5.50E-03 | BMI | 0.000 | 0.014 |  |  |  |  |
| rs11218575 | 11 | 122154576 | C | T | 0.002 | 0.003 | 5.43E-01 | 0.015 | 0.002 | 2.10E-12 | TV | 0.013 | 0.014 |  |  |  |  |
| rs4937842 | 11 | 133734542 | G | C | -0.007 | 0.003 | 7.97E-03 | -0.013 | 0.002 | 1.40E-08 | TV | -0.018 | 0.014 |  |  |  |  |
| rs11611246 | 12 | 939480 | T | G | 0.025 | 0.003 | 7.99E-17 | -0.002 | 0.003 | 4.20E-01 | BMI | 0.010 | 0.017 |  |  |  |  |
| rs76895963 | 12 | 4384844 | T | G | -0.077 | 0.011 | 1.07E-11 | 0.011 | 0.008 | 1.80E-01 | BMI | -0.232 | 0.056 |  |  |  |  |
| rs10772643 | 12 | 13415288 | C | T | 0.005 | 0.005 | 3.32E-01 | 0.025 | 0.003 | 1.30E-12 | TV | 0.041 | 0.022 |  |  |  |  |
| rs10842240 | 12 | 24060075 | C | G | 0.026 | 0.004 | 4.56E-12 | 0.006 | 0.003 | 6.20E-02 | BMI | 0.008 | 0.020 |  |  |  |  |
| rs10771746 | 12 | 30791864 | C | T | -0.002 | 0.003 | 6.07E-01 | -0.014 | 0.002 | 2.50E-09 | TV | -0.008 | 0.015 |  |  |  |  |
| rs11170468 | 12 | 39430048 | A | C | 0.016 | 0.003 | 7.84E-09 | 0.003 | 0.003 | 2.70E-01 | BMI | 0.012 | 0.016 |  |  |  |  |
| rs11181001 | 12 | 41948196 | A | G | 0.014 | 0.002 | 1.25E-09 | -0.002 | 0.002 | 3.50E-01 | BMI | 0.017 | 0.014 |  |  |  |  |
| rs7132908 | 12 | 50263148 | A | G | 0.035 | 0.003 | 1.35E-38 | 0.003 | 0.002 | 1.90E-01 | BMI | -0.001 | 0.014 |  |  |  |  |
| rs10876864 | 12 | 56401085 | G | A | -0.013 | 0.003 | 3.82E-07 | -0.013 | 0.002 | 1.00E-09 | TV | -0.005 | 0.014 |  |  |  |  |
| rs8756 | 12 | 66359752 | C | A | 0.009 | 0.002 | 9.38E-05 | -0.013 | 0.002 | 5.30E-10 | TV | -0.008 | 0.014 |  |  |  |  |
| rs1689437 | 12 | 89758046 | A | G | -0.030 | 0.005 | 2.04E-09 | -0.009 | 0.004 | 4.90E-02 | BMI | 0.034 | 0.028 |  |  |  |  |
| rs7488867 | 12 | 103699685 | T | C | -0.022 | 0.003 | 5.69E-14 | 0.000 | 0.002 | 8.70E-01 | BMI | -0.004 | 0.015 |  |  |  |  |
| rs2164744 | 12 | 109872039 | T | C | -0.003 | 0.003 | 4.07E-01 | -0.013 | 0.002 | 1.10E-08 | TV | 0.013 | 0.014 |  |  |  |  |
| rs2173650 | 12 | 117525235 | G | T | 0.005 | 0.004 | 2.16E-01 | 0.018 | 0.003 | 4.60E-09 | TV | 0.016 | 0.019 |  |  |  |  |
| rs10902480 | 12 | 132414769 | T | G | 0.022 | 0.004 | 5.60E-09 | 0.002 | 0.003 | 5.70E-01 | BMI | -0.002 | 0.018 |  |  |  |  |
| rs2012835 | 13 | 27987643 | T | G | -0.021 | 0.003 | 3.06E-10 | -0.001 | 0.002 | 7.00E-01 | BMI | 0.012 | 0.016 |  |  |  |  |
| rs2504236 | 13 | 28613298 | T | C | -0.015 | 0.003 | 1.94E-09 | 0.001 | 0.002 | 6.30E-01 | BMI | 0.007 | 0.014 |  |  |  |  |
| rs9595893 | 13 | 33175793 | T | G | -0.020 | 0.003 | 1.24E-14 | -0.008 | 0.002 | 2.40E-04 | BMI | -0.002 | 0.014 |  |  |  |  |
| rs9603697 | 13 | 40783323 | T | C | 0.020 | 0.003 | 1.34E-13 | 0.005 | 0.002 | 2.20E-02 | BMI | -0.002 | 0.015 |  |  |  |  |
| rs9563168 | 13 | 54247827 | G | A | 0.008 | 0.003 | 9.40E-03 | 0.018 | 0.003 | 4.30E-11 | TV | -0.004 | 0.017 |  |  |  |  |
| rs9569734 | 13 | 58319476 | A | G | 0.017 | 0.003 | 6.37E-07 | 0.019 | 0.003 | 3.40E-10 | TV | 0.024 | 0.019 |  |  |  |  |
| rs1441264 | 13 | 79580919 | A | G | 0.018 | 0.002 | 1.35E-13 | -0.003 | 0.002 | 1.40E-01 | BMI | 0.017 | 0.014 |  |  |  |  |
| rs56858768 | 13 | 86511730 | G | A | -0.016 | 0.003 | 8.41E-07 | -0.015 | 0.002 | 3.40E-10 | TV | -0.006 | 0.015 |  |  |  |  |
| rs9168 | 13 | 99101583 | A | C | -0.015 | 0.003 | 4.62E-08 | -0.006 | 0.002 | 1.80E-02 | BMI | 0.040 | 0.015 |  |  |  |  |
| rs7991062 | 13 | 100713194 | C | G | 0.002 | 0.003 | 3.93E-01 | -0.018 | 0.002 | 8.80E-15 | TV | -0.011 | 0.014 |  |  |  |  |
| rs9522279 | 13 | 112221296 | T | C | 0.022 | 0.003 | 1.65E-14 | 0.006 | 0.002 | 5.30E-03 | BMI | 0.019 | 0.014 |  |  |  |  |
| rs405797 | 14 | 26972834 | T | A | -0.009 | 0.003 | 2.78E-03 | -0.015 | 0.003 | 2.80E-09 | TV | -0.009 | 0.016 |  |  |  |  |
| rs1022785 | 14 | 29571653 | G | A | 0.004 | 0.004 | 2.36E-01 | 0.018 | 0.003 | 7.10E-09 | TV | 0.027 | 0.019 |  |  |  |  |
| rs1955540 | 14 | 40801751 | T | C | -0.020 | 0.003 | 2.89E-10 | -0.005 | 0.003 | 5.60E-02 | BMI | -0.008 | 0.017 |  |  |  |  |
| rs4900714 | 14 | 47302219 | T | G | 0.020 | 0.003 | 1.17E-15 | 0.002 | 0.002 | 3.20E-01 | BMI | -0.005 | 0.014 |  |  |  |  |
| rs217672 | 14 | 62361021 | A | C | -0.020 | 0.003 | 4.40E-10 | -0.010 | 0.002 | 8.50E-05 | BMI | 0.012 | 0.015 |  |  |  |  |
| rs3902951 | 14 | 69789755 | T | G | -0.015 | 0.003 | 4.40E-08 | -0.008 | 0.003 | 1.30E-03 | BMI | 0.003 | 0.016 |  |  |  |  |
| rs17109256 | 14 | 79939993 | A | G | 0.031 | 0.003 | 2.81E-27 | 0.000 | 0.003 | 9.60E-01 | BMI | -0.005 | 0.017 |  |  |  |  |
| rs34918753 | 14 | 88312341 | A | G | 0.020 | 0.003 | 1.77E-09 | 0.003 | 0.003 | 1.90E-01 | BMI | -0.004 | 0.017 |  |  |  |  |
| rs10145592 | 14 | 94287215 | C | G | -0.007 | 0.003 | 5.31E-03 | -0.015 | 0.002 | 1.80E-11 | TV | -0.017 | 0.014 |  |  |  |  |
| rs7157001 | 14 | 99749484 | A | G | -0.012 | 0.003 | 3.59E-04 | -0.014 | 0.002 | 2.30E-08 | TV | -0.005 | 0.017 |  |  |  |  |
| rs7143963 | 14 | 103304425 | T | C | 0.021 | 0.003 | 3.35E-12 | 0.000 | 0.003 | 9.40E-01 | BMI | -0.008 | 0.017 |  |  |  |  |
| rs11636536 | 15 | 36391772 | T | C | 0.015 | 0.003 | 2.80E-09 | 0.008 | 0.002 | 3.50E-04 | BMI | 0.014 | 0.014 |  |  |  |  |
| rs8043253 | 15 | 41503730 | C | T | -0.004 | 0.003 | 1.07E-01 | -0.012 | 0.002 | 3.40E-08 | TV | 0.013 | 0.014 |  |  |  |  |
| rs10851458 | 15 | 47939956 | A | G | 0.015 | 0.003 | 5.95E-09 | -0.008 | 0.002 | 2.10E-04 | BMI | 0.001 | 0.014 |  |  |  |  |
| rs2460 | 15 | 53073084 | G | A | -0.013 | 0.003 | 3.33E-06 | -0.015 | 0.002 | 5.00E-10 | TV | 0.004 | 0.016 |  |  |  |  |
| rs4775373 | 15 | 61476936 | T | C | 0.006 | 0.003 | 4.06E-02 | 0.013 | 0.002 | 4.70E-09 | TV | 0.002 | 0.014 |  |  |  |  |
| rs61331678 | 15 | 77995949 | G | C | 0.009 | 0.003 | 1.25E-03 | 0.015 | 0.002 | 6.10E-12 | TV | 0.004 | 0.014 |  |  |  |  |
| rs8041222 | 15 | 79465549 | A | G | -0.019 | 0.003 | 3.28E-11 | -0.007 | 0.002 | 8.30E-04 | BMI | -0.040 | 0.014 |  |  |  |  |
| rs8036171 | 15 | 95266810 | A | C | 0.015 | 0.003 | 4.26E-10 | 0.003 | 0.002 | 1.40E-01 | BMI | 0.000 | 0.014 |  |  |  |  |
| rs12448257 | 16 | 3599655 | A | G | 0.019 | 0.003 | 1.17E-10 | -0.004 | 0.003 | 1.20E-01 | BMI | 0.011 | 0.017 |  |  |  |  |
| rs879620 | 16 | 4015729 | T | C | 0.021 | 0.003 | 3.11E-16 | 0.006 | 0.002 | 1.20E-02 | BMI | 0.011 | 0.014 |  |  |  |  |
| rs35574015 | 16 | 15146061 | T | C | 0.013 | 0.003 | 3.97E-05 | -0.013 | 0.002 | 3.00E-08 | TV | 0.028 | 0.015 |  |  |  |  |
| rs7189927 | 16 | 28913787 | T | C | 0.029 | 0.003 | 1.16E-29 | 0.015 | 0.002 | 3.40E-11 | TV | 0.006 | 0.015 |  |  |  |  |
| rs749671 | 16 | 31088347 | G | A | 0.014 | 0.002 | 2.72E-09 | 0.016 | 0.002 | 2.70E-12 | TV | 0.018 | 0.014 |  |  |  |  |
| rs7184800 | 16 | 53509131 | G | A | -0.005 | 0.003 | 1.46E-01 | 0.017 | 0.002 | 8.20E-13 | TV | -0.003 | 0.015 |  |  |  |  |
| rs1558902 | 16 | 53803574 | A | T | 0.082 | 0.002 | 1.19E-257 | 0.001 | 0.002 | 6.60E-01 | BMI | 0.000 | 0.014 |  |  |  |  |
| rs889398 | 16 | 69556715 | T | C | -0.021 | 0.002 | 1.54E-18 | -0.002 | 0.002 | 4.30E-01 | BMI | -0.014 | 0.014 |  |  |  |  |
| rs75950518 | 16 | 70378917 | T | C | -0.027 | 0.005 | 3.51E-09 | -0.006 | 0.003 | 6.70E-02 | BMI | 0.012 | 0.023 |  |  |  |  |
| rs28483459 | 16 | 71998398 | T | C | -0.018 | 0.003 | 2.92E-09 | -0.003 | 0.002 | 1.90E-01 | BMI | -0.021 | 0.015 |  |  |  |  |
| rs7206608 | 16 | 82872628 | C | G | -0.015 | 0.003 | 1.85E-08 | -0.010 | 0.002 | 3.40E-05 | BMI | -0.016 | 0.015 |  |  |  |  |
| rs2447098 | 17 | 2277720 | C | A | 0.000 | 0.003 | 9.21E-01 | -0.015 | 0.002 | 6.80E-12 | TV | -0.034 | 0.014 |  |  |  |  |
| rs11654952 | 17 | 4813799 | T | G | -0.007 | 0.004 | 4.93E-02 | -0.017 | 0.003 | 4.30E-08 | TV | 0.005 | 0.019 |  |  |  |  |
| rs4986044 | 17 | 21261560 | T | C | -0.018 | 0.002 | 1.45E-14 | -0.009 | 0.002 | 1.80E-05 | BMI | -0.005 | 0.014 |  |  |  |  |
| rs8065172 | 17 | 31456969 | A | G | -0.017 | 0.003 | 1.23E-08 | -0.006 | 0.003 | 3.10E-02 | BMI | 0.024 | 0.016 |  |  |  |  |
| rs12150665 | 17 | 34914787 | T | C | 0.018 | 0.002 | 3.43E-14 | 0.006 | 0.002 | 4.50E-03 | BMI | 0.000 | 0.014 |  |  |  |  |
| rs676387 | 17 | 40706273 | A | C | 0.016 | 0.003 | 4.10E-09 | 0.010 | 0.002 | 2.10E-05 | BMI | 0.032 | 0.015 |  |  |  |  |
| rs208015 | 17 | 46252346 | T | C | 0.037 | 0.005 | 8.72E-14 | 0.008 | 0.004 | 7.90E-02 | BMI | 0.003 | 0.024 |  |  |  |  |
| rs78369934 | 17 | 61739101 | T | C | 0.037 | 0.007 | 1.47E-08 | 0.022 | 0.005 | 3.90E-06 | BMI | 0.020 | 0.032 |  |  |  |  |
| rs9902312 | 17 | 65070304 | T | C | 0.004 | 0.003 | 1.59E-01 | 0.015 | 0.002 | 4.50E-11 | TV | -0.005 | 0.015 |  |  |  |  |
| rs11657730 | 17 | 79372489 | C | T | 0.004 | 0.003 | 1.63E-01 | 0.013 | 0.002 | 1.10E-08 | TV | 0.007 | 0.015 |  |  |  |  |
| rs7226371 | 18 | 1850771 | A | G | -0.019 | 0.003 | 7.73E-09 | -0.006 | 0.003 | 4.20E-02 | BMI | 0.004 | 0.019 |  |  |  |  |
| rs303753 | 18 | 21074922 | G | A | -0.021 | 0.003 | 5.82E-15 | -0.014 | 0.002 | 2.70E-10 | TV | -0.029 | 0.015 |  |  |  |  |
| rs9964724 | 18 | 35159124 | C | T | -0.003 | 0.003 | 2.88E-01 | 0.018 | 0.002 | 3.30E-14 | TV | 0.014 | 0.015 |  |  |  |  |
| rs1156541 | 18 | 39952989 | C | T | 0.007 | 0.003 | 1.56E-02 | 0.014 | 0.003 | 2.80E-08 | TV | 0.016 | 0.016 |  |  |  |  |
| rs2612575 | 18 | 42967340 | A | G | -0.016 | 0.003 | 1.45E-08 | -0.004 | 0.002 | 6.50E-02 | BMI | -0.015 | 0.016 |  |  |  |  |
| rs17512836 | 18 | 53194961 | T | C | 0.013 | 0.009 | 1.69E-01 | 0.042 | 0.007 | 7.00E-10 | TV | -0.018 | 0.040 |  |  |  |  |
| rs6567160 | 18 | 57829135 | T | C | -0.053 | 0.003 | 2.11E-82 | 0.006 | 0.003 | 2.50E-02 | BMI | -0.019 | 0.016 |  |  |  |  |
| rs17710386 | 18 | 63461201 | T | C | -0.016 | 0.003 | 3.75E-11 | -0.003 | 0.002 | 1.60E-01 | BMI | 0.009 | 0.015 |  |  |  |  |
| rs1787267 | 18 | 76742544 | C | G | -0.033 | 0.005 | 2.49E-10 | 0.001 | 0.005 | 7.50E-01 | BMI | 0.028 | 0.030 |  |  |  |  |
| rs45486197 | 19 | 2244849 | A | G | 0.035 | 0.006 | 3.49E-09 | 0.001 | 0.004 | 9.00E-01 | BMI | -0.025 | 0.034 |  |  |  |  |
| rs7258309 | 19 | 4016005 | T | G | 0.023 | 0.003 | 6.54E-14 | 0.009 | 0.003 | 1.40E-03 | BMI | -0.025 | 0.017 |  |  |  |  |
| rs7248205 | 19 | 10770305 | C | T | 0.007 | 0.003 | 2.55E-02 | 0.014 | 0.002 | 3.40E-10 | TV | -0.046 | 0.014 |  |  |  |  |
| rs273496 | 19 | 18241857 | T | C | 0.020 | 0.003 | 2.50E-11 | 0.010 | 0.002 | 1.40E-05 | BMI | -0.012 | 0.014 |  |  |  |  |
| rs111901094 | 19 | 19513570 | G | T | -0.006 | 0.004 | 1.14E-01 | -0.017 | 0.003 | 2.00E-09 | TV | 0.022 | 0.018 |  |  |  |  |
| rs17513613 | 19 | 30286822 | T | C | -0.018 | 0.003 | 1.50E-12 | -0.004 | 0.002 | 8.50E-02 | BMI | -0.021 | 0.015 |  |  |  |  |
| rs11084553 | 19 | 31019780 | A | G | 0.024 | 0.004 | 9.41E-12 | 0.004 | 0.003 | 2.00E-01 | BMI | 0.031 | 0.020 |  |  |  |  |
| rs769449 | 19 | 45410002 | A | G | -0.024 | 0.004 | 1.58E-09 | -0.014 | 0.003 | 1.70E-05 | BMI | 0.017 | 0.022 |  |  |  |  |
| rs1800437 | 19 | 46181392 | C | G | -0.037 | 0.003 | 1.73E-33 | -0.004 | 0.003 | 1.30E-01 | BMI | -0.006 | 0.017 |  |  |  |  |
| rs9304665 | 19 | 47602577 | A | T | 0.027 | 0.003 | 4.78E-21 | -0.006 | 0.003 | 1.90E-02 | BMI | -0.012 | 0.017 |  |  |  |  |
| rs7249149 | 19 | 51774538 | A | G | 0.015 | 0.003 | 4.41E-08 | 0.001 | 0.002 | 5.80E-01 | BMI | 0.005 | 0.016 |  |  |  |  |
| rs6131281 | 20 | 11891724 | C | T | 0.005 | 0.003 | 1.13E-01 | 0.016 | 0.002 | 3.10E-13 | TV | 0.021 | 0.014 |  |  |  |  |
| rs8123881 | 20 | 15819495 | A | G | -0.019 | 0.003 | 2.53E-08 | -0.003 | 0.003 | 3.60E-01 | BMI | 0.040 | 0.020 |  |  |  |  |
| rs6141814 | 20 | 31368960 | C | A | 0.005 | 0.003 | 7.01E-02 | -0.014 | 0.002 | 1.30E-09 | TV | 0.020 | 0.014 |  |  |  |  |
| rs55700114 | 20 | 43717080 | G | A | -0.007 | 0.003 | 3.08E-02 | -0.014 | 0.002 | 2.10E-09 | TV | -0.006 | 0.015 |  |  |  |  |
| rs7272093 | 20 | 49810724 | A | G | -0.017 | 0.003 | 1.10E-09 | -0.006 | 0.002 | 2.00E-02 | BMI | 0.010 | 0.016 |  |  |  |  |
| rs35388084 | 20 | 50960490 | A | G | -0.028 | 0.004 | 1.12E-13 | -0.010 | 0.003 | 4.70E-04 | BMI | -0.031 | 0.018 |  |  |  |  |
| rs6092143 | 20 | 54181875 | T | C | 0.015 | 0.003 | 1.10E-08 | 0.002 | 0.002 | 4.80E-01 | BMI | -0.005 | 0.014 |  |  |  |  |
| rs56103247 | 20 | 62483184 | C | T | 0.027 | 0.007 | 3.07E-05 | 0.030 | 0.005 | 3.80E-10 | TV | -0.075 | 0.035 |  |  |  |  |
| rs13047416 | 21 | 40309436 | C | G | 0.016 | 0.003 | 9.84E-10 | 0.002 | 0.002 | 3.80E-01 | BMI | -0.021 | 0.014 |  |  |  |  |
| rs10427502 | 21 | 40654840 | G | A | 0.014 | 0.003 | 1.40E-06 | 0.014 | 0.002 | 8.60E-10 | TV | 0.024 | 0.014 |  |  |  |  |
| rs427943 | 21 | 46570896 | A | C | -0.018 | 0.003 | 2.19E-12 | -0.009 | 0.002 | 3.70E-05 | BMI | 0.003 | 0.014 |  |  |  |  |
| rs2092829 | 22 | 31780514 | G | A | -0.001 | 0.003 | 6.28E-01 | 0.014 | 0.002 | 1.30E-08 | TV | -0.027 | 0.015 |  |  |  |  |
| **3) Analysis of colorectal cancer in women only** | | | | | | | | | | | | | | | | | |
| SNP | CHR | POS | EA | OA | beta_bmi | se_bmi | pval_bmi | beta_tv | se_tv | pval_tv | phenotype | beta_crc | se_crc |  |  |  |  |
| rs79113395 | 1 | 1590521 | A | G | -0.021 | 0.003 | 1.45E-12 | -0.007 | 0.002 | 4.50E-03 | BMI | -0.003 | 0.019 |  |  |  |  |
| rs10909880 | 1 | 2727804 | T | C | -0.012 | 0.002 | 1.97E-08 | 0.000 | 0.002 | 9.30E-01 | BMI | -0.013 | 0.014 |  |  |  |  |
| rs78886584 | 1 | 16859325 | A | G | -0.015 | 0.003 | 1.30E-08 | -0.007 | 0.002 | 2.40E-03 | BMI | -0.024 | 0.016 |  |  |  |  |
| rs4295917 | 1 | 32173360 | A | G | 0.014 | 0.002 | 1.62E-08 | 0.006 | 0.002 | 8.50E-03 | BMI | -0.001 | 0.014 |  |  |  |  |
| rs11810109 | 1 | 44186812 | A | T | -0.002 | 0.003 | 5.76E-01 | 0.016 | 0.002 | 5.40E-12 | TV | 0.022 | 0.015 |  |  |  |  |
| rs2984618 | 1 | 47690438 | T | G | 0.017 | 0.002 | 2.21E-14 | 0.001 | 0.002 | 6.40E-01 | BMI | -0.017 | 0.014 |  |  |  |  |
| rs657452 | 1 | 49589847 | A | G | 0.020 | 0.002 | 1.14E-19 | -0.004 | 0.002 | 7.00E-02 | BMI | 0.001 | 0.014 |  |  |  |  |
| rs12725114 | 1 | 62432641 | G | A | 0.008 | 0.003 | 1.01E-02 | 0.015 | 0.003 | 1.60E-08 | TV | -0.007 | 0.017 |  |  |  |  |
| rs984409 | 1 | 67020440 | G | A | -0.010 | 0.003 | 2.25E-04 | -0.015 | 0.002 | 4.10E-11 | TV | -0.028 | 0.015 |  |  |  |  |
| rs71658797 | 1 | 77967507 | T | A | -0.038 | 0.004 | 1.24E-20 | -0.020 | 0.003 | 6.30E-10 | TV | -0.009 | 0.023 |  |  |  |  |
| rs114328297 | 1 | 91190854 | T | G | 0.003 | 0.003 | 4.34E-01 | 0.014 | 0.003 | 4.40E-08 | TV | -0.018 | 0.018 |  |  |  |  |
| rs17379561 | 1 | 98340139 | A | T | -0.022 | 0.003 | 1.48E-11 | -0.025 | 0.003 | 1.10E-16 | TV | 0.024 | 0.020 |  |  |  |  |
| rs55909997 | 1 | 107581039 | G | A | -0.013 | 0.002 | 1.72E-07 | -0.014 | 0.002 | 2.40E-09 | TV | -0.017 | 0.014 |  |  |  |  |
| rs1278847 | 1 | 110037483 | C | A | -0.010 | 0.003 | 4.05E-04 | 0.016 | 0.002 | 1.20E-11 | TV | -0.018 | 0.015 |  |  |  |  |
| rs4845364 | 1 | 154141908 | A | G | -0.002 | 0.002 | 5.13E-01 | -0.015 | 0.002 | 1.30E-12 | TV | 0.001 | 0.014 |  |  |  |  |
| rs61813324 | 1 | 156049877 | T | C | 0.030 | 0.004 | 3.60E-14 | 0.015 | 0.003 | 4.30E-06 | BMI | 0.063 | 0.024 |  |  |  |  |
| rs12564992 | 1 | 174478100 | A | G | -0.021 | 0.004 | 2.37E-09 | 0.008 | 0.003 | 2.00E-02 | BMI | -0.012 | 0.021 |  |  |  |  |
| rs6673341 | 1 | 184655649 | T | G | -0.011 | 0.002 | 1.15E-06 | -0.015 | 0.002 | 2.20E-11 | TV | 0.012 | 0.014 |  |  |  |  |
| rs10737620 | 1 | 193057971 | T | A | 0.013 | 0.003 | 1.16E-07 | 0.014 | 0.002 | 2.60E-09 | TV | -0.011 | 0.015 |  |  |  |  |
| rs4474229 | 1 | 195037001 | A | G | -0.017 | 0.003 | 1.64E-10 | -0.003 | 0.002 | 1.20E-01 | BMI | -0.009 | 0.014 |  |  |  |  |
| rs2820295 | 1 | 201800868 | A | G | 0.021 | 0.002 | 8.03E-19 | 0.010 | 0.002 | 8.70E-06 | BMI | 0.026 | 0.015 |  |  |  |  |
| rs17014375 | 1 | 209543560 | T | G | -0.019 | 0.003 | 1.46E-08 | -0.001 | 0.003 | 7.20E-01 | BMI | 0.049 | 0.020 |  |  |  |  |
| rs12042959 | 1 | 243533273 | A | G | 0.019 | 0.003 | 2.33E-09 | 0.004 | 0.003 | 2.20E-01 | BMI | -0.002 | 0.019 |  |  |  |  |
| rs62106258 | 2 | 417167 | T | C | 0.099 | 0.006 | 8.72E-58 | -0.007 | 0.005 | 1.90E-01 | BMI | 0.041 | 0.036 |  |  |  |  |
| rs6548237 | 2 | 621461 | A | C | -0.058 | 0.003 | 4.43E-89 | -0.005 | 0.003 | 1.00E-01 | BMI | -0.004 | 0.018 |  |  |  |  |
| rs7571496 | 2 | 6169351 | A | G | 0.015 | 0.003 | 1.45E-08 | 0.004 | 0.002 | 1.20E-01 | BMI | 0.008 | 0.015 |  |  |  |  |
| rs7601870 | 2 | 15384392 | A | G | -0.013 | 0.002 | 1.23E-08 | -0.006 | 0.002 | 9.10E-03 | BMI | -0.013 | 0.014 |  |  |  |  |
| rs72781699 | 2 | 24277709 | G | A | -0.002 | 0.003 | 4.85E-01 | -0.019 | 0.003 | 3.00E-12 | TV | 0.008 | 0.017 |  |  |  |  |
| rs10048652 | 2 | 35449306 | T | C | 0.014 | 0.002 | 3.47E-09 | 0.001 | 0.002 | 7.30E-01 | BMI | 0.010 | 0.014 |  |  |  |  |
| rs12105701 | 2 | 41737200 | C | T | -0.009 | 0.002 | 6.21E-05 | -0.013 | 0.002 | 5.40E-09 | TV | -0.030 | 0.014 |  |  |  |  |
| rs786420 | 2 | 44719893 | T | C | 0.016 | 0.003 | 8.84E-09 | -0.001 | 0.003 | 6.90E-01 | BMI | 0.009 | 0.016 |  |  |  |  |
| rs7564130 | 2 | 50606642 | T | C | -0.001 | 0.002 | 5.70E-01 | -0.015 | 0.002 | 2.80E-11 | TV | -0.022 | 0.014 |  |  |  |  |
| rs10189857 | 2 | 60713235 | A | G | -0.005 | 0.002 | 2.16E-02 | -0.020 | 0.002 | 6.20E-21 | TV | -0.021 | 0.014 |  |  |  |  |
| rs62641636 | 2 | 68425427 | A | G | 0.003 | 0.003 | 2.52E-01 | 0.014 | 0.002 | 7.00E-10 | TV | 0.018 | 0.015 |  |  |  |  |
| rs12714199 | 2 | 86812549 | T | C | -0.015 | 0.002 | 5.12E-10 | -0.002 | 0.002 | 4.80E-01 | BMI | 0.007 | 0.014 |  |  |  |  |
| rs11689199 | 2 | 100820421 | A | G | 0.019 | 0.002 | 1.45E-15 | 0.018 | 0.002 | 5.50E-17 | TV | 0.012 | 0.014 |  |  |  |  |
| rs1457457 | 2 | 104340148 | A | T | 0.014 | 0.002 | 3.79E-10 | 0.012 | 0.002 | 1.20E-08 | BMI | -0.002 | 0.014 |  |  |  |  |
| rs1451533 | 2 | 105466005 | G | A | -0.015 | 0.003 | 3.95E-09 | -0.016 | 0.002 | 1.10E-10 | TV | 0.011 | 0.015 |  |  |  |  |
| rs12476388 | 2 | 116330983 | C | T | 0.006 | 0.003 | 1.70E-02 | 0.013 | 0.002 | 2.10E-08 | TV | 0.000 | 0.015 |  |  |  |  |
| rs1984559 | 2 | 135678888 | A | G | 0.016 | 0.003 | 3.27E-08 | 0.007 | 0.003 | 9.40E-03 | BMI | -0.004 | 0.016 |  |  |  |  |
| rs13002158 | 2 | 144021712 | A | G | -0.021 | 0.003 | 1.15E-12 | -0.003 | 0.003 | 2.90E-01 | BMI | -0.001 | 0.017 |  |  |  |  |
| rs374722 | 2 | 147839830 | G | A | 0.013 | 0.003 | 5.05E-05 | 0.024 | 0.003 | 5.50E-16 | TV | 0.049 | 0.019 |  |  |  |  |
| rs3754970 | 2 | 162091836 | T | C | -0.003 | 0.003 | 2.07E-01 | -0.015 | 0.002 | 4.80E-12 | TV | 0.007 | 0.014 |  |  |  |  |
| rs2060199 | 2 | 166196681 | T | A | 0.003 | 0.002 | 1.88E-01 | 0.012 | 0.002 | 2.40E-08 | TV | -0.002 | 0.014 |  |  |  |  |
| rs7588437 | 2 | 181575281 | A | G | -0.018 | 0.002 | 8.38E-15 | -0.006 | 0.002 | 1.40E-02 | BMI | 0.010 | 0.014 |  |  |  |  |
| rs4577309 | 2 | 191288833 | A | G | 0.002 | 0.003 | 3.61E-01 | 0.016 | 0.002 | 1.60E-13 | TV | -0.012 | 0.014 |  |  |  |  |
| rs34224261 | 2 | 193855452 | G | C | -0.010 | 0.003 | 1.97E-04 | -0.018 | 0.002 | 4.60E-16 | TV | 0.005 | 0.014 |  |  |  |  |
| rs4675246 | 2 | 202864487 | G | T | 0.002 | 0.003 | 4.91E-01 | -0.015 | 0.003 | 2.90E-08 | TV | 0.031 | 0.018 |  |  |  |  |
| rs13029509 | 2 | 215374209 | G | A | -0.007 | 0.002 | 2.60E-03 | -0.018 | 0.002 | 2.00E-17 | TV | -0.004 | 0.014 |  |  |  |  |
| rs10932837 | 2 | 221044785 | C | T | 0.001 | 0.002 | 7.82E-01 | -0.013 | 0.002 | 1.30E-09 | TV | -0.020 | 0.014 |  |  |  |  |
| rs6720868 | 2 | 230663576 | T | C | 0.017 | 0.002 | 4.65E-12 | 0.006 | 0.002 | 1.10E-02 | BMI | -0.013 | 0.014 |  |  |  |  |
| rs4973576 | 2 | 233806771 | C | A | 0.000 | 0.003 | 8.92E-01 | -0.015 | 0.002 | 1.00E-09 | TV | 0.034 | 0.015 |  |  |  |  |
| rs138256022 | 3 | 11603090 | C | T | 0.000 | 0.007 | 9.75E-01 | -0.031 | 0.006 | 2.90E-08 | TV | 0.065 | 0.044 |  |  |  |  |
| rs13062093 | 3 | 35667057 | T | G | -0.014 | 0.002 | 8.00E-09 | 0.001 | 0.002 | 6.20E-01 | BMI | 0.003 | 0.014 |  |  |  |  |
| rs9834970 | 3 | 36856030 | T | C | 0.006 | 0.002 | 1.14E-02 | 0.013 | 0.002 | 3.30E-09 | TV | 0.024 | 0.014 |  |  |  |  |
| rs3796386 | 3 | 49899795 | G | A | -0.017 | 0.002 | 9.78E-14 | -0.026 | 0.002 | 3.20E-33 | TV | -0.018 | 0.014 |  |  |  |  |
| rs11130793 | 3 | 60884659 | C | T | 0.011 | 0.003 | 6.46E-05 | 0.013 | 0.002 | 5.00E-09 | TV | -0.003 | 0.014 |  |  |  |  |
| rs1452075 | 3 | 62481063 | T | C | 0.016 | 0.003 | 1.47E-10 | 0.002 | 0.002 | 3.70E-01 | BMI | -0.006 | 0.015 |  |  |  |  |
| rs11714337 | 3 | 71582521 | G | A | 0.009 | 0.003 | 1.28E-03 | 0.014 | 0.002 | 4.70E-11 | TV | -0.029 | 0.014 |  |  |  |  |
| rs7647242 | 3 | 82642734 | T | C | 0.015 | 0.002 | 3.92E-10 | 0.002 | 0.002 | 4.50E-01 | BMI | 0.005 | 0.014 |  |  |  |  |
| rs6797840 | 3 | 85656569 | A | C | -0.012 | 0.003 | 8.70E-06 | -0.016 | 0.002 | 1.70E-13 | TV | 0.018 | 0.014 |  |  |  |  |
| rs2034768 | 3 | 93986371 | A | G | 0.019 | 0.003 | 9.80E-14 | 0.015 | 0.002 | 8.60E-12 | TV | -0.007 | 0.014 |  |  |  |  |
| rs1436348 | 3 | 104612668 | A | G | -0.013 | 0.002 | 6.31E-09 | -0.004 | 0.002 | 9.70E-02 | BMI | -0.030 | 0.014 |  |  |  |  |
| rs1289736 | 3 | 107950892 | T | C | 0.014 | 0.002 | 1.43E-09 | 0.007 | 0.002 | 9.50E-04 | BMI | 0.008 | 0.014 |  |  |  |  |
| rs9867121 | 3 | 114631548 | C | A | 0.009 | 0.003 | 5.94E-03 | 0.019 | 0.003 | 3.90E-12 | TV | -0.004 | 0.017 |  |  |  |  |
| rs7428670 | 3 | 131579810 | C | G | 0.020 | 0.003 | 3.09E-15 | 0.003 | 0.002 | 2.20E-01 | BMI | 0.023 | 0.016 |  |  |  |  |
| rs7621025 | 3 | 136272246 | T | C | -0.020 | 0.003 | 1.62E-15 | -0.004 | 0.002 | 1.30E-01 | BMI | -0.029 | 0.016 |  |  |  |  |
| rs16851483 | 3 | 141275436 | T | G | 0.036 | 0.005 | 2.28E-15 | 0.013 | 0.004 | 2.90E-03 | BMI | 0.028 | 0.026 |  |  |  |  |
| rs355754 | 3 | 154024165 | T | C | -0.014 | 0.002 | 4.26E-10 | -0.003 | 0.002 | 1.60E-01 | BMI | -0.008 | 0.014 |  |  |  |  |
| rs12491503 | 3 | 165706855 | G | A | -0.005 | 0.002 | 3.29E-02 | -0.014 | 0.002 | 5.50E-10 | TV | 0.008 | 0.014 |  |  |  |  |
| rs247975 | 3 | 173107443 | T | C | -0.018 | 0.002 | 3.62E-15 | -0.005 | 0.002 | 1.80E-02 | BMI | 0.016 | 0.014 |  |  |  |  |
| rs114600294 | 3 | 181419367 | G | C | -0.007 | 0.003 | 3.65E-02 | -0.016 | 0.003 | 7.90E-10 | TV | 0.005 | 0.020 |  |  |  |  |
| rs10513801 | 3 | 185822353 | T | G | 0.035 | 0.003 | 1.26E-27 | -0.009 | 0.003 | 3.70E-03 | BMI | -0.019 | 0.021 |  |  |  |  |
| rs4677812 | 3 | 194863794 | A | C | -0.014 | 0.003 | 9.38E-09 | -0.009 | 0.002 | 1.10E-04 | BMI | -0.026 | 0.015 |  |  |  |  |
| rs66852340 | 4 | 3311070 | C | T | -0.009 | 0.003 | 1.10E-03 | -0.018 | 0.003 | 7.90E-12 | TV | 0.002 | 0.016 |  |  |  |  |
| rs7700107 | 4 | 17880416 | A | C | -0.016 | 0.003 | 3.40E-06 | -0.021 | 0.003 | 1.90E-11 | TV | 0.017 | 0.019 |  |  |  |  |
| rs994596 | 4 | 18459828 | T | C | 0.015 | 0.003 | 1.02E-09 | 0.001 | 0.002 | 5.30E-01 | BMI | 0.003 | 0.014 |  |  |  |  |
| rs73213484 | 4 | 28489339 | A | T | 0.025 | 0.004 | 2.28E-11 | 0.002 | 0.003 | 4.40E-01 | BMI | 0.014 | 0.019 |  |  |  |  |
| rs337637 | 4 | 38604470 | A | G | -0.013 | 0.002 | 3.04E-08 | -0.002 | 0.002 | 3.40E-01 | BMI | -0.031 | 0.014 |  |  |  |  |
| rs10938397 | 4 | 45182527 | A | G | -0.032 | 0.002 | 1.66E-45 | -0.011 | 0.002 | 9.30E-07 | BMI | -0.019 | 0.014 |  |  |  |  |
| rs28971796 | 4 | 49151982 | A | G | 0.012 | 0.003 | 3.93E-05 | 0.015 | 0.002 | 2.10E-09 | TV | 0.018 | 0.017 |  |  |  |  |
| rs11945861 | 4 | 65700865 | A | G | -0.015 | 0.003 | 3.13E-08 | 0.002 | 0.003 | 3.40E-01 | BMI | -0.005 | 0.016 |  |  |  |  |
| rs10002111 | 4 | 67815504 | A | G | 0.017 | 0.003 | 9.26E-10 | 0.007 | 0.003 | 4.30E-03 | BMI | 0.022 | 0.016 |  |  |  |  |
| rs6850494 | 4 | 82291771 | A | C | 0.003 | 0.003 | 2.38E-01 | -0.014 | 0.002 | 1.10E-10 | TV | -0.002 | 0.014 |  |  |  |  |
| rs4148155 | 4 | 89054667 | A | G | 0.020 | 0.004 | 6.20E-09 | 0.009 | 0.003 | 6.60E-03 | BMI | -0.015 | 0.022 |  |  |  |  |
| rs12645001 | 4 | 96018337 | A | G | -0.014 | 0.002 | 4.11E-09 | 0.001 | 0.002 | 7.30E-01 | BMI | 0.011 | 0.014 |  |  |  |  |
| rs13107325 | 4 | 103188709 | T | C | 0.045 | 0.004 | 9.33E-25 | 0.029 | 0.004 | 1.50E-12 | both | -0.028 | 0.027 |  |  |  |  |
| rs4834272 | 4 | 113313986 | T | C | -0.014 | 0.002 | 8.24E-10 | 0.002 | 0.002 | 4.40E-01 | BMI | -0.001 | 0.014 |  |  |  |  |
| rs7693703 | 4 | 118357405 | G | A | 0.004 | 0.004 | 3.03E-01 | 0.023 | 0.004 | 2.70E-09 | TV | 0.050 | 0.024 |  |  |  |  |
| rs1403846 | 4 | 119101723 | T | C | 0.016 | 0.003 | 1.62E-08 | 0.008 | 0.003 | 3.50E-03 | BMI | 0.028 | 0.018 |  |  |  |  |
| rs4334769 | 4 | 130245233 | G | T | 0.004 | 0.002 | 1.22E-01 | 0.012 | 0.002 | 2.10E-08 | TV | -0.016 | 0.014 |  |  |  |  |
| rs57800857 | 4 | 140863365 | A | C | 0.019 | 0.003 | 1.07E-11 | 0.004 | 0.002 | 5.20E-02 | BMI | 0.001 | 0.014 |  |  |  |  |
| rs35107973 | 4 | 143125948 | T | C | 0.023 | 0.004 | 7.66E-09 | 0.002 | 0.003 | 5.50E-01 | BMI | 0.026 | 0.022 |  |  |  |  |
| rs6825241 | 4 | 152543783 | C | A | -0.003 | 0.002 | 2.30E-01 | -0.017 | 0.002 | 4.90E-15 | TV | -0.012 | 0.014 |  |  |  |  |
| rs7693082 | 4 | 159857819 | G | C | 0.012 | 0.003 | 3.71E-05 | 0.015 | 0.002 | 1.70E-10 | TV | 0.020 | 0.015 |  |  |  |  |
| rs13110266 | 4 | 162129844 | A | G | -0.014 | 0.002 | 1.55E-10 | -0.006 | 0.002 | 7.90E-03 | BMI | -0.003 | 0.014 |  |  |  |  |
| rs1491333 | 4 | 172186288 | T | G | -0.015 | 0.003 | 9.85E-09 | -0.002 | 0.003 | 4.90E-01 | BMI | 0.000 | 0.015 |  |  |  |  |
| rs6890310 | 5 | 27193573 | A | G | -0.015 | 0.003 | 1.38E-09 | -0.003 | 0.002 | 1.90E-01 | BMI | 0.029 | 0.015 |  |  |  |  |
| rs782971 | 5 | 43124688 | A | G | -0.017 | 0.003 | 4.89E-12 | -0.001 | 0.002 | 5.90E-01 | BMI | -0.023 | 0.016 |  |  |  |  |
| rs10940659 | 5 | 59360341 | A | G | -0.007 | 0.002 | 4.25E-03 | -0.013 | 0.002 | 1.50E-08 | TV | 0.003 | 0.014 |  |  |  |  |
| rs262890 | 5 | 62930015 | A | G | -0.013 | 0.003 | 7.07E-06 | -0.019 | 0.002 | 3.20E-15 | TV | 0.028 | 0.015 |  |  |  |  |
| rs2112347 | 5 | 75015242 | T | G | 0.031 | 0.002 | 2.13E-42 | 0.007 | 0.002 | 2.10E-03 | BMI | 0.003 | 0.014 |  |  |  |  |
| rs7716447 | 5 | 88800331 | A | G | -0.015 | 0.002 | 1.46E-10 | -0.013 | 0.002 | 3.60E-09 | TV | -0.009 | 0.014 |  |  |  |  |
| rs1031423 | 5 | 93276883 | T | C | 0.007 | 0.003 | 1.55E-02 | -0.019 | 0.003 | 1.80E-12 | TV | 0.017 | 0.017 |  |  |  |  |
| rs2161097 | 5 | 103945178 | T | C | 0.013 | 0.002 | 2.31E-08 | 0.004 | 0.002 | 8.40E-02 | BMI | -0.019 | 0.014 |  |  |  |  |
| rs57585211 | 5 | 107197725 | T | G | -0.003 | 0.003 | 3.66E-01 | -0.017 | 0.003 | 4.30E-09 | TV | 0.012 | 0.018 |  |  |  |  |
| rs158186 | 5 | 107440856 | A | G | -0.029 | 0.003 | 3.23E-20 | -0.002 | 0.003 | 5.30E-01 | BMI | -0.011 | 0.018 |  |  |  |  |
| rs9326862 | 5 | 112054547 | A | T | 0.005 | 0.002 | 4.43E-02 | 0.012 | 0.002 | 2.60E-08 | TV | 0.014 | 0.014 |  |  |  |  |
| rs4895231 | 5 | 119388659 | C | G | 0.014 | 0.002 | 9.26E-10 | 0.008 | 0.002 | 4.60E-04 | BMI | 0.013 | 0.014 |  |  |  |  |
| rs10041724 | 5 | 124273520 | T | C | 0.005 | 0.003 | 1.17E-01 | 0.018 | 0.003 | 3.90E-11 | TV | 0.010 | 0.017 |  |  |  |  |
| rs62379379 | 5 | 141082015 | G | T | -0.011 | 0.005 | 3.92E-02 | -0.026 | 0.004 | 7.80E-10 | TV | -0.038 | 0.026 |  |  |  |  |
| rs10054327 | 5 | 147871137 | G | A | 0.004 | 0.003 | 1.83E-01 | 0.017 | 0.002 | 3.40E-15 | TV | -0.005 | 0.014 |  |  |  |  |
| rs42210 | 5 | 166408788 | G | C | -0.004 | 0.003 | 1.81E-01 | -0.014 | 0.002 | 7.30E-09 | TV | 0.004 | 0.015 |  |  |  |  |
| rs72828890 | 5 | 167806836 | C | T | 0.016 | 0.004 | 1.25E-04 | 0.019 | 0.003 | 4.90E-09 | TV | -0.002 | 0.023 |  |  |  |  |
| rs2228213 | 6 | 12124855 | A | G | -0.015 | 0.002 | 9.21E-11 | -0.005 | 0.002 | 3.30E-02 | BMI | -0.004 | 0.014 |  |  |  |  |
| rs9367369 | 6 | 13189941 | A | G | 0.017 | 0.003 | 2.32E-09 | 0.003 | 0.002 | 2.40E-01 | BMI | -0.005 | 0.015 |  |  |  |  |
| rs3806114 | 6 | 20482335 | A | G | -0.014 | 0.002 | 4.49E-09 | -0.009 | 0.002 | 1.30E-04 | BMI | 0.009 | 0.015 |  |  |  |  |
| rs72834698 | 6 | 26176517 | G | A | 0.013 | 0.004 | 9.74E-04 | 0.023 | 0.003 | 2.70E-13 | TV | 0.031 | 0.020 |  |  |  |  |
| rs9471333 | 6 | 40362023 | C | T | 0.021 | 0.003 | 4.04E-15 | 0.013 | 0.002 | 1.50E-09 | TV | 0.001 | 0.014 |  |  |  |  |
| rs943005 | 6 | 50865820 | T | C | 0.040 | 0.003 | 1.99E-43 | 0.003 | 0.003 | 3.10E-01 | BMI | -0.012 | 0.018 |  |  |  |  |
| rs2465043 | 6 | 51180765 | A | G | -0.014 | 0.002 | 3.22E-09 | -0.002 | 0.002 | 2.90E-01 | BMI | -0.012 | 0.014 |  |  |  |  |
| rs62412826 | 6 | 61881167 | T | C | 0.033 | 0.006 | 7.98E-09 | -0.003 | 0.005 | 5.10E-01 | BMI | 0.043 | 0.036 |  |  |  |  |
| rs4523073 | 6 | 67550288 | A | G | -0.004 | 0.003 | 1.07E-01 | -0.014 | 0.002 | 5.00E-10 | TV | 0.009 | 0.014 |  |  |  |  |
| rs209416 | 6 | 83534729 | A | T | 0.017 | 0.002 | 8.71E-12 | -0.003 | 0.002 | 2.50E-01 | BMI | 0.004 | 0.015 |  |  |  |  |
| rs6905544 | 6 | 98411631 | A | G | -0.015 | 0.002 | 1.28E-10 | -0.019 | 0.002 | 8.50E-18 | TV | -0.023 | 0.014 |  |  |  |  |
| rs17789218 | 6 | 100600097 | T | C | -0.007 | 0.003 | 1.07E-02 | 0.019 | 0.003 | 1.40E-13 | TV | -0.006 | 0.016 |  |  |  |  |
| rs768023 | 6 | 108876002 | A | G | 0.019 | 0.002 | 1.38E-16 | 0.005 | 0.002 | 2.00E-02 | BMI | 0.001 | 0.014 |  |  |  |  |
| rs2357760 | 6 | 120213880 | A | G | 0.016 | 0.002 | 1.09E-11 | 0.006 | 0.002 | 1.60E-02 | BMI | -0.005 | 0.014 |  |  |  |  |
| rs2184364 | 6 | 143183596 | A | G | 0.011 | 0.003 | 2.43E-04 | 0.016 | 0.003 | 3.00E-09 | TV | 0.022 | 0.017 |  |  |  |  |
| rs2185027 | 6 | 153381622 | A | C | -0.015 | 0.002 | 1.11E-10 | -0.009 | 0.002 | 2.10E-04 | BMI | -0.002 | 0.015 |  |  |  |  |
| rs12207125 | 6 | 154301575 | A | G | 0.018 | 0.003 | 7.67E-09 | -0.003 | 0.003 | 2.00E-01 | BMI | -0.039 | 0.016 |  |  |  |  |
| rs9718104 | 6 | 166170539 | T | G | 0.002 | 0.006 | 7.51E-01 | -0.041 | 0.005 | 9.30E-19 | TV | -0.013 | 0.031 |  |  |  |  |
| rs836525 | 7 | 6465909 | T | C | 0.018 | 0.003 | 9.75E-09 | 0.003 | 0.003 | 3.60E-01 | BMI | -0.024 | 0.019 |  |  |  |  |
| rs17568389 | 7 | 8091876 | T | A | 0.002 | 0.002 | 3.46E-01 | 0.015 | 0.002 | 8.60E-13 | TV | -0.012 | 0.014 |  |  |  |  |
| rs4307239 | 7 | 24354300 | A | G | -0.014 | 0.002 | 1.31E-09 | -0.001 | 0.002 | 8.00E-01 | BMI | 0.004 | 0.014 |  |  |  |  |
| rs11971098 | 7 | 26699061 | A | G | -0.024 | 0.004 | 6.49E-09 | 0.005 | 0.004 | 2.40E-01 | BMI | -0.019 | 0.024 |  |  |  |  |
| rs10234444 | 7 | 41828527 | G | A | 0.006 | 0.003 | 5.90E-02 | 0.016 | 0.003 | 2.90E-08 | TV | -0.018 | 0.018 |  |  |  |  |
| rs10237317 | 7 | 70045941 | A | G | -0.014 | 0.002 | 6.82E-10 | 0.000 | 0.002 | 9.20E-01 | BMI | -0.002 | 0.014 |  |  |  |  |
| rs1167800 | 7 | 75176196 | A | G | 0.020 | 0.002 | 3.66E-19 | 0.008 | 0.002 | 1.10E-04 | BMI | 0.004 | 0.014 |  |  |  |  |
| rs6973656 | 7 | 77422583 | A | G | -0.011 | 0.002 | 1.55E-06 | -0.013 | 0.002 | 1.00E-09 | TV | 0.031 | 0.014 |  |  |  |  |
| rs7805441 | 7 | 78121458 | T | C | 0.015 | 0.002 | 2.01E-10 | 0.007 | 0.002 | 1.60E-03 | BMI | 0.009 | 0.014 |  |  |  |  |
| rs973734 | 7 | 89387578 | C | A | 0.001 | 0.003 | 7.34E-01 | 0.017 | 0.003 | 1.50E-08 | TV | 0.031 | 0.020 |  |  |  |  |
| rs2528531 | 7 | 93236510 | A | C | -0.015 | 0.002 | 1.35E-09 | 0.004 | 0.002 | 1.00E-01 | BMI | -0.007 | 0.014 |  |  |  |  |
| rs10271582 | 7 | 99121950 | A | G | 0.019 | 0.003 | 8.41E-09 | 0.017 | 0.003 | 1.90E-08 | BMI | 0.033 | 0.018 |  |  |  |  |
| rs2299383 | 7 | 103418846 | T | C | 0.018 | 0.002 | 2.73E-16 | -0.002 | 0.002 | 2.90E-01 | BMI | -0.008 | 0.014 |  |  |  |  |
| rs62471080 | 7 | 109128060 | G | C | -0.009 | 0.003 | 9.05E-04 | -0.013 | 0.002 | 3.70E-09 | TV | -0.004 | 0.014 |  |  |  |  |
| rs10246289 | 7 | 115544763 | A | G | 0.004 | 0.004 | 3.87E-01 | 0.019 | 0.003 | 1.70E-08 | TV | 0.042 | 0.022 |  |  |  |  |
| rs11763734 | 7 | 126400910 | A | C | 0.000 | 0.002 | 9.14E-01 | -0.012 | 0.002 | 8.20E-09 | TV | -0.005 | 0.014 |  |  |  |  |
| rs2968864 | 7 | 150622162 | T | C | 0.016 | 0.003 | 9.35E-10 | 0.004 | 0.002 | 7.70E-02 | BMI | 0.001 | 0.016 |  |  |  |  |
| rs7834121 | 8 | 10770255 | G | T | 0.017 | 0.003 | 1.79E-10 | -0.014 | 0.002 | 1.10E-10 | TV | 0.000 | 0.015 |  |  |  |  |
| rs2959592 | 8 | 17002725 | T | C | 0.017 | 0.003 | 6.26E-10 | 0.006 | 0.003 | 3.30E-02 | BMI | 0.004 | 0.017 |  |  |  |  |
| rs1421334 | 8 | 30865733 | A | C | 0.013 | 0.002 | 2.39E-08 | 0.017 | 0.002 | 2.20E-15 | TV | 0.018 | 0.014 |  |  |  |  |
| rs4320543 | 8 | 34422967 | A | G | -0.013 | 0.002 | 2.95E-08 | -0.010 | 0.002 | 6.30E-06 | BMI | -0.010 | 0.014 |  |  |  |  |
| rs12681792 | 8 | 62054463 | A | C | 0.018 | 0.003 | 2.61E-10 | 0.007 | 0.003 | 1.50E-02 | BMI | 0.003 | 0.018 |  |  |  |  |
| rs7827848 | 8 | 64790774 | A | T | 0.015 | 0.002 | 1.88E-11 | 0.009 | 0.002 | 8.20E-05 | BMI | 0.016 | 0.014 |  |  |  |  |
| rs6996198 | 8 | 65463442 | C | T | -0.002 | 0.003 | 4.82E-01 | -0.016 | 0.003 | 3.50E-08 | TV | -0.025 | 0.018 |  |  |  |  |
| rs6472942 | 8 | 76827190 | T | C | 0.017 | 0.002 | 3.53E-13 | -0.013 | 0.002 | 1.80E-09 | TV | 0.004 | 0.014 |  |  |  |  |
| rs11985394 | 8 | 92667521 | T | A | 0.003 | 0.002 | 2.74E-01 | 0.012 | 0.002 | 1.30E-08 | TV | -0.001 | 0.014 |  |  |  |  |
| rs2721965 | 8 | 116662038 | A | C | 0.019 | 0.002 | 2.10E-14 | 0.006 | 0.002 | 6.60E-03 | BMI | -0.002 | 0.015 |  |  |  |  |
| rs12541615 | 8 | 118870151 | T | C | -0.010 | 0.003 | 3.99E-03 | -0.018 | 0.003 | 3.90E-10 | TV | -0.008 | 0.018 |  |  |  |  |
| rs10099330 | 8 | 143383694 | A | G | -0.013 | 0.002 | 9.94E-09 | -0.007 | 0.002 | 9.80E-04 | BMI | -0.010 | 0.014 |  |  |  |  |
| rs2717559 | 8 | 143882420 | A | G | 0.005 | 0.003 | 8.28E-02 | 0.012 | 0.002 | 1.50E-08 | TV | 0.018 | 0.014 |  |  |  |  |
| rs2616830 | 9 | 1721385 | G | A | 0.003 | 0.002 | 2.41E-01 | 0.016 | 0.002 | 2.90E-14 | TV | 0.008 | 0.014 |  |  |  |  |
| rs10124645 | 9 | 11321119 | A | G | 0.014 | 0.002 | 4.98E-09 | 0.007 | 0.002 | 2.70E-03 | BMI | -0.012 | 0.014 |  |  |  |  |
| rs34864022 | 9 | 22609110 | A | G | 0.001 | 0.005 | 9.19E-01 | -0.026 | 0.004 | 1.40E-09 | TV | -0.010 | 0.030 |  |  |  |  |
| rs12554512 | 9 | 23352293 | T | C | -0.012 | 0.003 | 4.61E-06 | 0.021 | 0.002 | 3.80E-21 | TV | -0.002 | 0.014 |  |  |  |  |
| rs16912921 | 9 | 28413461 | A | C | 0.025 | 0.002 | 1.56E-26 | 0.005 | 0.002 | 4.70E-02 | BMI | 0.027 | 0.015 |  |  |  |  |
| rs973345 | 9 | 29713169 | T | C | 0.014 | 0.002 | 1.09E-09 | 0.005 | 0.002 | 2.20E-02 | BMI | 0.006 | 0.014 |  |  |  |  |
| rs563132 | 9 | 37245476 | A | T | 0.018 | 0.002 | 1.96E-14 | -0.006 | 0.002 | 4.50E-03 | BMI | -0.006 | 0.014 |  |  |  |  |
| rs10116186 | 9 | 80532374 | A | G | -0.015 | 0.003 | 4.10E-08 | -0.003 | 0.002 | 1.70E-01 | BMI | 0.005 | 0.014 |  |  |  |  |
| rs2791574 | 9 | 82212094 | T | G | -0.004 | 0.002 | 8.01E-02 | -0.013 | 0.002 | 3.00E-08 | TV | -0.019 | 0.014 |  |  |  |  |
| rs870151 | 9 | 92230747 | T | A | -0.014 | 0.002 | 4.62E-09 | -0.016 | 0.002 | 8.80E-13 | TV | 0.018 | 0.014 |  |  |  |  |
| rs2787374 | 9 | 103054951 | T | C | 0.013 | 0.002 | 5.57E-08 | 0.012 | 0.002 | 1.20E-08 | TV | 0.025 | 0.014 |  |  |  |  |
| rs7024334 | 9 | 109072075 | T | G | 0.016 | 0.003 | 1.63E-09 | 0.000 | 0.003 | 8.90E-01 | BMI | 0.020 | 0.016 |  |  |  |  |
| rs1928295 | 9 | 120378483 | T | C | 0.016 | 0.002 | 2.85E-13 | 0.006 | 0.002 | 3.90E-03 | BMI | 0.003 | 0.014 |  |  |  |  |
| rs7043521 | 9 | 124602779 | A | T | 0.013 | 0.003 | 1.97E-06 | 0.014 | 0.002 | 6.50E-11 | TV | -0.027 | 0.014 |  |  |  |  |
| rs4382592 | 9 | 134870755 | T | G | 0.007 | 0.003 | 4.41E-03 | 0.014 | 0.002 | 6.70E-09 | TV | 0.005 | 0.015 |  |  |  |  |
| rs2073869 | 9 | 135763816 | C | T | 0.008 | 0.003 | 1.17E-02 | 0.019 | 0.003 | 1.50E-10 | TV | -0.018 | 0.018 |  |  |  |  |
| rs1623025 | 10 | 11164813 | G | C | -0.003 | 0.003 | 2.47E-01 | -0.012 | 0.002 | 1.40E-08 | TV | 0.017 | 0.014 |  |  |  |  |
| rs1243182 | 10 | 21916728 | C | T | -0.020 | 0.003 | 3.15E-15 | -0.019 | 0.002 | 2.00E-15 | TV | -0.020 | 0.015 |  |  |  |  |
| rs34930419 | 10 | 33987329 | T | C | -0.046 | 0.008 | 1.37E-09 | 0.000 | 0.006 | 9.70E-01 | BMI | -0.028 | 0.039 |  |  |  |  |
| rs2045147 | 10 | 56660437 | A | G | 0.003 | 0.002 | 1.72E-01 | 0.013 | 0.002 | 5.90E-09 | TV | -0.001 | 0.014 |  |  |  |  |
| rs10994943 | 10 | 63591413 | T | G | 0.007 | 0.003 | 1.48E-02 | 0.013 | 0.002 | 3.10E-09 | TV | -0.006 | 0.014 |  |  |  |  |
| rs10824347 | 10 | 77645229 | A | T | 0.018 | 0.003 | 5.47E-11 | 0.006 | 0.003 | 1.10E-02 | BMI | -0.007 | 0.016 |  |  |  |  |
| rs11201422 | 10 | 86924483 | T | C | 0.012 | 0.003 | 1.45E-06 | 0.013 | 0.002 | 3.40E-08 | TV | 0.036 | 0.015 |  |  |  |  |
| rs563296 | 10 | 99772404 | A | G | 0.018 | 0.002 | 8.85E-14 | 0.007 | 0.002 | 9.30E-04 | BMI | 0.001 | 0.014 |  |  |  |  |
| rs10786658 | 10 | 103737193 | A | T | 0.002 | 0.003 | 3.88E-01 | -0.014 | 0.002 | 4.60E-11 | TV | -0.007 | 0.014 |  |  |  |  |
| rs7089973 | 10 | 116569565 | C | A | 0.000 | 0.002 | 9.06E-01 | -0.013 | 0.002 | 2.40E-09 | TV | -0.020 | 0.014 |  |  |  |  |
| rs11245482 | 10 | 126733546 | T | C | -0.011 | 0.002 | 4.63E-06 | -0.013 | 0.002 | 2.60E-09 | TV | -0.016 | 0.014 |  |  |  |  |
| rs17727474 | 10 | 127182959 | C | T | -0.004 | 0.003 | 2.43E-01 | 0.018 | 0.003 | 3.10E-09 | TV | 0.005 | 0.019 |  |  |  |  |
| rs7395632 | 11 | 958789 | T | C | -0.013 | 0.002 | 3.87E-08 | 0.000 | 0.002 | 9.00E-01 | BMI | 0.025 | 0.014 |  |  |  |  |
| rs4929923 | 11 | 8639200 | T | C | -0.022 | 0.002 | 2.51E-22 | -0.003 | 0.002 | 1.30E-01 | BMI | -0.008 | 0.014 |  |  |  |  |
| rs12289262 | 11 | 12894758 | C | T | -0.009 | 0.003 | 4.88E-04 | -0.014 | 0.002 | 2.00E-08 | TV | -0.029 | 0.016 |  |  |  |  |
| rs11022762 | 11 | 13335926 | T | C | -0.015 | 0.002 | 5.56E-11 | -0.005 | 0.002 | 1.30E-02 | BMI | 0.018 | 0.014 |  |  |  |  |
| rs6265 | 11 | 27679916 | T | C | -0.037 | 0.003 | 7.95E-40 | 0.003 | 0.003 | 2.20E-01 | BMI | 0.001 | 0.017 |  |  |  |  |
| rs2862961 | 11 | 43656535 | A | G | -0.024 | 0.003 | 5.30E-22 | -0.004 | 0.002 | 1.10E-01 | BMI | -0.020 | 0.015 |  |  |  |  |
| rs10742752 | 11 | 45438374 | T | C | -0.013 | 0.002 | 3.98E-09 | 0.002 | 0.002 | 3.80E-01 | BMI | 0.023 | 0.014 |  |  |  |  |
| rs7124681 | 11 | 47529947 | A | C | 0.025 | 0.002 | 9.27E-29 | 0.004 | 0.002 | 5.10E-02 | BMI | 0.001 | 0.014 |  |  |  |  |
| rs801733 | 11 | 65934549 | A | C | 0.009 | 0.002 | 1.67E-04 | 0.017 | 0.002 | 7.30E-14 | TV | 0.026 | 0.014 |  |  |  |  |
| rs116175338 | 11 | 72467875 | G | T | -0.008 | 0.003 | 2.76E-03 | -0.014 | 0.003 | 1.40E-08 | TV | -0.011 | 0.016 |  |  |  |  |
| rs12272012 | 11 | 84861286 | G | C | -0.010 | 0.007 | 1.20E-01 | -0.030 | 0.005 | 1.90E-08 | TV | 0.062 | 0.033 |  |  |  |  |
| rs11020045 | 11 | 92487907 | A | C | 0.002 | 0.002 | 5.41E-01 | -0.013 | 0.002 | 1.60E-08 | TV | -0.006 | 0.015 |  |  |  |  |
| rs17207890 | 11 | 95490754 | G | A | -0.002 | 0.002 | 5.51E-01 | 0.016 | 0.002 | 6.70E-12 | TV | -0.010 | 0.015 |  |  |  |  |
| rs1003081 | 11 | 118913993 | T | C | 0.014 | 0.002 | 6.27E-11 | 0.003 | 0.002 | 2.40E-01 | BMI | 0.019 | 0.014 |  |  |  |  |
| rs11218575 | 11 | 122154576 | C | T | 0.005 | 0.002 | 4.42E-02 | 0.015 | 0.002 | 2.10E-12 | TV | -0.010 | 0.014 |  |  |  |  |
| rs4937842 | 11 | 133734542 | G | C | -0.009 | 0.002 | 2.80E-04 | -0.013 | 0.002 | 1.40E-08 | TV | -0.008 | 0.014 |  |  |  |  |
| rs11611246 | 12 | 939480 | T | G | 0.021 | 0.003 | 1.27E-14 | -0.002 | 0.003 | 4.20E-01 | BMI | 0.017 | 0.017 |  |  |  |  |
| rs10772643 | 12 | 13415288 | C | T | 0.013 | 0.004 | 1.86E-03 | 0.025 | 0.003 | 1.30E-12 | TV | 0.014 | 0.022 |  |  |  |  |
| rs7976757 | 12 | 19207948 | T | C | -0.019 | 0.003 | 2.94E-10 | -0.006 | 0.003 | 2.90E-02 | BMI | -0.001 | 0.018 |  |  |  |  |
| rs16926318 | 12 | 23678745 | A | G | -0.023 | 0.004 | 4.23E-08 | -0.011 | 0.004 | 5.50E-03 | BMI | -0.005 | 0.023 |  |  |  |  |
| rs16926778 | 12 | 24002125 | C | G | 0.026 | 0.004 | 1.41E-09 | 0.003 | 0.004 | 5.30E-01 | BMI | 0.008 | 0.024 |  |  |  |  |
| rs10771746 | 12 | 30791864 | C | T | -0.003 | 0.003 | 3.36E-01 | -0.014 | 0.002 | 2.50E-09 | TV | 0.017 | 0.015 |  |  |  |  |
| rs7138803 | 12 | 50247468 | A | G | 0.029 | 0.002 | 9.70E-38 | 0.004 | 0.002 | 1.10E-01 | BMI | 0.022 | 0.014 |  |  |  |  |
| rs10876864 | 12 | 56401085 | G | A | -0.010 | 0.002 | 6.64E-06 | -0.013 | 0.002 | 1.00E-09 | TV | -0.018 | 0.014 |  |  |  |  |
| rs11173522 | 12 | 60953472 | A | C | 0.017 | 0.003 | 9.01E-10 | 0.004 | 0.003 | 1.20E-01 | BMI | 0.017 | 0.016 |  |  |  |  |
| rs8756 | 12 | 66359752 | C | A | 0.001 | 0.002 | 5.52E-01 | -0.013 | 0.002 | 5.30E-10 | TV | -0.009 | 0.014 |  |  |  |  |
| rs10746306 | 12 | 84477374 | T | G | 0.018 | 0.003 | 8.74E-09 | 0.003 | 0.003 | 2.70E-01 | BMI | -0.014 | 0.016 |  |  |  |  |
| rs2279574 | 12 | 89745477 | A | C | -0.017 | 0.002 | 1.96E-12 | -0.006 | 0.002 | 7.80E-03 | BMI | 0.006 | 0.014 |  |  |  |  |
| rs2712643 | 12 | 99638276 | A | C | -0.015 | 0.003 | 1.47E-09 | -0.004 | 0.003 | 1.40E-01 | BMI | -0.004 | 0.016 |  |  |  |  |
| rs17033633 | 12 | 103656343 | T | C | -0.021 | 0.003 | 7.26E-12 | -0.003 | 0.003 | 2.30E-01 | BMI | -0.004 | 0.018 |  |  |  |  |
| rs2164744 | 12 | 109872039 | T | C | -0.006 | 0.003 | 4.25E-02 | -0.013 | 0.002 | 1.10E-08 | TV | 0.015 | 0.014 |  |  |  |  |
| rs2173650 | 12 | 117525235 | G | T | 0.003 | 0.004 | 4.77E-01 | 0.018 | 0.003 | 4.60E-09 | TV | -0.028 | 0.019 |  |  |  |  |
| rs9579775 | 13 | 20616557 | A | C | -0.025 | 0.004 | 3.66E-10 | -0.003 | 0.003 | 3.20E-01 | BMI | -0.039 | 0.023 |  |  |  |  |
| rs12872889 | 13 | 28674628 | T | C | -0.021 | 0.003 | 1.52E-11 | -0.002 | 0.003 | 5.50E-01 | BMI | -0.011 | 0.017 |  |  |  |  |
| rs9595630 | 13 | 33065443 | T | G | 0.015 | 0.002 | 2.04E-10 | 0.008 | 0.002 | 6.00E-04 | BMI | -0.009 | 0.014 |  |  |  |  |
| rs9563168 | 13 | 54247827 | G | A | 0.010 | 0.003 | 4.82E-04 | 0.018 | 0.003 | 4.30E-11 | TV | 0.016 | 0.017 |  |  |  |  |
| rs9569734 | 13 | 58319476 | A | G | 0.019 | 0.003 | 3.51E-09 | 0.019 | 0.003 | 3.40E-10 | TV | -0.011 | 0.019 |  |  |  |  |
| rs9571687 | 13 | 67472713 | A | C | -0.015 | 0.002 | 3.24E-09 | -0.005 | 0.002 | 3.40E-02 | BMI | -0.021 | 0.014 |  |  |  |  |
| rs1441264 | 13 | 79580919 | A | G | 0.017 | 0.002 | 5.19E-14 | -0.003 | 0.002 | 1.40E-01 | BMI | 0.017 | 0.014 |  |  |  |  |
| rs56858768 | 13 | 86511730 | G | A | -0.017 | 0.003 | 1.04E-08 | -0.015 | 0.002 | 3.40E-10 | TV | -0.015 | 0.015 |  |  |  |  |
| rs1927790 | 13 | 96922191 | T | C | -0.014 | 0.002 | 1.27E-09 | -0.005 | 0.002 | 3.20E-02 | BMI | 0.012 | 0.014 |  |  |  |  |
| rs7991062 | 13 | 100713194 | C | G | -0.004 | 0.002 | 1.25E-01 | -0.018 | 0.002 | 8.80E-15 | TV | -0.015 | 0.014 |  |  |  |  |
| rs7335249 | 13 | 109962579 | T | C | 0.014 | 0.002 | 4.91E-09 | 0.003 | 0.002 | 1.20E-01 | BMI | -0.008 | 0.014 |  |  |  |  |
| rs2479958 | 13 | 111984244 | A | G | 0.014 | 0.002 | 1.38E-08 | 0.004 | 0.002 | 4.50E-02 | BMI | 0.034 | 0.014 |  |  |  |  |
| rs405797 | 14 | 26972834 | T | A | -0.005 | 0.003 | 4.22E-02 | -0.015 | 0.003 | 2.80E-09 | TV | -0.023 | 0.016 |  |  |  |  |
| rs1022785 | 14 | 29571653 | G | A | 0.010 | 0.003 | 1.48E-03 | 0.018 | 0.003 | 7.10E-09 | TV | -0.024 | 0.019 |  |  |  |  |
| rs35020117 | 14 | 73341655 | T | G | 0.017 | 0.003 | 1.23E-09 | 0.003 | 0.002 | 2.20E-01 | BMI | 0.013 | 0.014 |  |  |  |  |
| rs7141420 | 14 | 79899454 | T | C | 0.021 | 0.002 | 7.84E-22 | 0.006 | 0.002 | 6.50E-03 | BMI | 0.002 | 0.014 |  |  |  |  |
| rs10145592 | 14 | 94287215 | C | G | -0.004 | 0.002 | 6.48E-02 | -0.015 | 0.002 | 1.80E-11 | TV | -0.014 | 0.014 |  |  |  |  |
| rs7157001 | 14 | 99749484 | A | G | -0.003 | 0.003 | 3.52E-01 | -0.014 | 0.002 | 2.30E-08 | TV | -0.013 | 0.017 |  |  |  |  |
| rs3803286 | 14 | 103246470 | A | G | 0.017 | 0.002 | 8.29E-13 | 0.000 | 0.002 | 9.90E-01 | BMI | -0.009 | 0.014 |  |  |  |  |
| rs12101625 | 15 | 26972431 | C | G | 0.014 | 0.002 | 5.54E-09 | 0.002 | 0.002 | 4.40E-01 | BMI | 0.006 | 0.014 |  |  |  |  |
| rs8043253 | 15 | 41503730 | C | T | -0.006 | 0.002 | 9.24E-03 | -0.012 | 0.002 | 3.40E-08 | TV | 0.019 | 0.014 |  |  |  |  |
| rs2460 | 15 | 53073084 | G | A | -0.011 | 0.003 | 1.94E-05 | -0.015 | 0.002 | 5.00E-10 | TV | -0.032 | 0.016 |  |  |  |  |
| rs8024806 | 15 | 53473990 | T | C | 0.027 | 0.005 | 9.76E-09 | 0.010 | 0.005 | 2.60E-02 | BMI | 0.041 | 0.028 |  |  |  |  |
| rs4775373 | 15 | 61476936 | T | C | 0.005 | 0.003 | 7.06E-02 | 0.013 | 0.002 | 4.70E-09 | TV | 0.009 | 0.014 |  |  |  |  |
| rs12914489 | 15 | 74187937 | A | G | 0.023 | 0.004 | 1.64E-10 | 0.010 | 0.003 | 3.50E-03 | BMI | 0.023 | 0.023 |  |  |  |  |
| rs61331678 | 15 | 77995949 | G | C | 0.008 | 0.003 | 2.59E-03 | 0.015 | 0.002 | 6.10E-12 | TV | -0.025 | 0.014 |  |  |  |  |
| rs7498044 | 15 | 92573639 | A | G | -0.018 | 0.003 | 2.73E-08 | -0.011 | 0.003 | 6.40E-05 | BMI | -0.006 | 0.017 |  |  |  |  |
| rs11073383 | 15 | 95274349 | A | G | -0.016 | 0.002 | 3.26E-12 | -0.003 | 0.002 | 2.30E-01 | BMI | -0.005 | 0.014 |  |  |  |  |
| rs57221746 | 15 | 99230035 | A | G | -0.018 | 0.003 | 2.08E-08 | -0.008 | 0.003 | 1.00E-03 | BMI | 0.013 | 0.016 |  |  |  |  |
| rs214249 | 16 | 348687 | T | G | 0.017 | 0.002 | 1.37E-12 | 0.007 | 0.002 | 1.30E-03 | BMI | 0.018 | 0.014 |  |  |  |  |
| rs12448257 | 16 | 3599655 | A | G | 0.015 | 0.003 | 3.13E-08 | -0.004 | 0.003 | 1.20E-01 | BMI | -0.003 | 0.017 |  |  |  |  |
| rs879620 | 16 | 4015729 | T | C | 0.025 | 0.002 | 5.45E-26 | 0.006 | 0.002 | 1.20E-02 | BMI | 0.011 | 0.014 |  |  |  |  |
| rs35574015 | 16 | 15146061 | T | C | 0.004 | 0.003 | 2.01E-01 | -0.013 | 0.002 | 3.00E-08 | TV | 0.011 | 0.015 |  |  |  |  |
| rs7189927 | 16 | 28913787 | T | C | 0.025 | 0.002 | 3.50E-26 | 0.015 | 0.002 | 3.40E-11 | TV | 0.012 | 0.015 |  |  |  |  |
| rs749671 | 16 | 31088347 | G | A | 0.026 | 0.002 | 1.78E-31 | 0.016 | 0.002 | 2.70E-12 | TV | 0.028 | 0.014 |  |  |  |  |
| rs7184800 | 16 | 53509131 | G | A | -0.006 | 0.003 | 4.26E-02 | 0.017 | 0.002 | 8.20E-13 | TV | 0.011 | 0.015 |  |  |  |  |
| rs1558902 | 16 | 53803574 | A | T | 0.070 | 0.002 | 1.10E-215 | 0.001 | 0.002 | 6.60E-01 | BMI | -0.005 | 0.014 |  |  |  |  |
| rs4783718 | 16 | 69547741 | T | C | 0.020 | 0.002 | 1.77E-19 | 0.003 | 0.002 | 1.20E-01 | BMI | 0.001 | 0.015 |  |  |  |  |
| rs811054 | 16 | 72251132 | T | C | 0.016 | 0.002 | 3.18E-13 | -0.006 | 0.002 | 8.00E-03 | BMI | 0.011 | 0.014 |  |  |  |  |
| rs2012502 | 16 | 81728081 | A | C | 0.015 | 0.002 | 2.97E-11 | 0.002 | 0.002 | 2.80E-01 | BMI | 0.008 | 0.014 |  |  |  |  |
| rs2447098 | 17 | 2277720 | C | A | -0.002 | 0.002 | 3.60E-01 | -0.015 | 0.002 | 6.80E-12 | TV | 0.009 | 0.014 |  |  |  |  |
| rs11654952 | 17 | 4813799 | T | G | -0.001 | 0.003 | 7.62E-01 | -0.017 | 0.003 | 4.30E-08 | TV | 0.047 | 0.020 |  |  |  |  |
| rs1075901 | 17 | 15943910 | T | C | -0.014 | 0.002 | 4.36E-11 | 0.001 | 0.002 | 6.60E-01 | BMI | -0.006 | 0.014 |  |  |  |  |
| rs4986044 | 17 | 21261560 | T | C | -0.017 | 0.002 | 2.20E-15 | -0.009 | 0.002 | 1.80E-05 | BMI | -0.030 | 0.014 |  |  |  |  |
| rs12150665 | 17 | 34914787 | T | C | 0.017 | 0.002 | 5.92E-14 | 0.006 | 0.002 | 4.50E-03 | BMI | 0.003 | 0.014 |  |  |  |  |
| rs56161855 | 17 | 46288649 | A | T | -0.026 | 0.004 | 1.48E-11 | -0.007 | 0.003 | 2.00E-02 | BMI | -0.039 | 0.019 |  |  |  |  |
| rs9906944 | 17 | 47091420 | T | C | -0.019 | 0.002 | 7.25E-15 | -0.010 | 0.002 | 2.40E-05 | BMI | 0.016 | 0.015 |  |  |  |  |
| rs7211966 | 17 | 54374611 | T | C | -0.047 | 0.008 | 4.89E-09 | -0.005 | 0.008 | 5.00E-01 | BMI | 0.013 | 0.056 |  |  |  |  |
| rs8075273 | 17 | 61728881 | A | C | -0.016 | 0.002 | 5.56E-11 | -0.014 | 0.002 | 2.80E-09 | BMI | 0.013 | 0.015 |  |  |  |  |
| rs9902312 | 17 | 65070304 | T | C | -0.001 | 0.002 | 5.61E-01 | 0.015 | 0.002 | 4.50E-11 | TV | 0.004 | 0.015 |  |  |  |  |
| rs4969387 | 17 | 79081724 | C | G | -0.015 | 0.003 | 6.90E-09 | 0.001 | 0.002 | 6.40E-01 | BMI | -0.005 | 0.016 |  |  |  |  |
| rs11657730 | 17 | 79372489 | C | T | 0.002 | 0.003 | 4.99E-01 | 0.013 | 0.002 | 1.10E-08 | TV | 0.012 | 0.015 |  |  |  |  |
| rs9955276 | 18 | 1839339 | T | C | 0.022 | 0.003 | 8.02E-12 | 0.007 | 0.003 | 1.90E-02 | BMI | 0.025 | 0.020 |  |  |  |  |
| rs303753 | 18 | 21074922 | G | A | -0.019 | 0.002 | 1.99E-15 | -0.014 | 0.002 | 2.70E-10 | TV | 0.001 | 0.015 |  |  |  |  |
| rs9964724 | 18 | 35159124 | C | T | 0.003 | 0.003 | 2.32E-01 | 0.018 | 0.002 | 3.30E-14 | TV | 0.004 | 0.015 |  |  |  |  |
| rs1156541 | 18 | 39952989 | C | T | 0.014 | 0.003 | 2.81E-07 | 0.014 | 0.003 | 2.80E-08 | TV | 0.019 | 0.016 |  |  |  |  |
| rs17512836 | 18 | 53194961 | T | C | 0.021 | 0.008 | 1.30E-02 | 0.042 | 0.007 | 7.00E-10 | TV | -0.009 | 0.040 |  |  |  |  |
| rs6567160 | 18 | 57829135 | T | C | -0.057 | 0.003 | 8.74E-108 | 0.006 | 0.003 | 2.50E-02 | BMI | -0.020 | 0.016 |  |  |  |  |
| rs895330 | 19 | 4060707 | C | G | 0.018 | 0.003 | 6.65E-10 | 0.006 | 0.003 | 1.80E-02 | BMI | -0.045 | 0.018 |  |  |  |  |
| rs7248205 | 19 | 10770305 | C | T | 0.010 | 0.003 | 3.27E-04 | 0.014 | 0.002 | 3.40E-10 | TV | -0.010 | 0.014 |  |  |  |  |
| rs273504 | 19 | 18215247 | A | G | -0.015 | 0.002 | 1.27E-10 | -0.009 | 0.002 | 9.50E-05 | BMI | -0.006 | 0.014 |  |  |  |  |
| rs17724992 | 19 | 18454825 | A | G | 0.017 | 0.003 | 6.65E-12 | 0.004 | 0.002 | 1.10E-01 | BMI | 0.029 | 0.015 |  |  |  |  |
| rs111901094 | 19 | 19513570 | G | T | -0.011 | 0.003 | 1.57E-03 | -0.017 | 0.003 | 2.00E-09 | TV | -0.044 | 0.018 |  |  |  |  |
| rs12462975 | 19 | 30272202 | A | G | 0.020 | 0.003 | 2.63E-16 | 0.003 | 0.002 | 2.40E-01 | BMI | 0.025 | 0.015 |  |  |  |  |
| rs11667280 | 19 | 30705747 | C | G | 0.016 | 0.003 | 3.14E-08 | 0.007 | 0.003 | 9.10E-03 | BMI | 0.017 | 0.017 |  |  |  |  |
| rs7250833 | 19 | 33937277 | T | C | 0.015 | 0.002 | 7.22E-10 | 0.001 | 0.002 | 7.40E-01 | BMI | -0.029 | 0.015 |  |  |  |  |
| rs185350 | 19 | 34306816 | T | C | 0.013 | 0.002 | 7.14E-09 | 0.004 | 0.002 | 4.40E-02 | BMI | 0.011 | 0.014 |  |  |  |  |
| rs429358 | 19 | 45411941 | T | C | 0.028 | 0.004 | 3.17E-14 | 0.014 | 0.003 | 3.80E-06 | BMI | 0.019 | 0.020 |  |  |  |  |
| rs11672660 | 19 | 46180184 | T | C | -0.032 | 0.003 | 5.93E-31 | -0.004 | 0.003 | 1.40E-01 | BMI | 0.011 | 0.017 |  |  |  |  |
| rs9304665 | 19 | 47602577 | A | T | 0.020 | 0.003 | 1.84E-14 | -0.006 | 0.003 | 1.90E-02 | BMI | 0.009 | 0.016 |  |  |  |  |
| rs1884389 | 20 | 1410582 | T | C | -0.014 | 0.002 | 1.20E-09 | -0.001 | 0.002 | 5.80E-01 | BMI | -0.007 | 0.014 |  |  |  |  |
| rs1884897 | 20 | 6612832 | A | G | -0.017 | 0.002 | 9.06E-14 | 0.000 | 0.002 | 8.80E-01 | BMI | 0.074 | 0.014 |  |  |  |  |
| rs6131281 | 20 | 11891724 | C | T | 0.009 | 0.003 | 6.80E-04 | 0.016 | 0.002 | 3.10E-13 | TV | 0.011 | 0.014 |  |  |  |  |
| rs6141814 | 20 | 31368960 | C | A | 0.001 | 0.003 | 7.05E-01 | -0.014 | 0.002 | 1.30E-09 | TV | 0.004 | 0.014 |  |  |  |  |
| rs6142096 | 20 | 32686658 | A | G | 0.013 | 0.002 | 4.17E-09 | 0.001 | 0.002 | 7.30E-01 | BMI | -0.004 | 0.014 |  |  |  |  |
| rs55700114 | 20 | 43717080 | G | A | -0.003 | 0.003 | 3.02E-01 | -0.014 | 0.002 | 2.10E-09 | TV | 0.006 | 0.015 |  |  |  |  |
| rs17806224 | 20 | 51065854 | A | G | -0.029 | 0.003 | 3.52E-22 | -0.009 | 0.003 | 7.70E-04 | BMI | -0.012 | 0.018 |  |  |  |  |
| rs56103247 | 20 | 62483184 | C | T | 0.020 | 0.006 | 7.71E-04 | 0.030 | 0.005 | 3.80E-10 | TV | -0.080 | 0.034 |  |  |  |  |
| rs2836754 | 21 | 40291740 | T | C | -0.015 | 0.002 | 3.73E-11 | -0.003 | 0.002 | 2.60E-01 | BMI | 0.003 | 0.014 |  |  |  |  |
| rs10427502 | 21 | 40654840 | G | A | 0.010 | 0.003 | 3.20E-04 | 0.014 | 0.002 | 8.60E-10 | TV | -0.017 | 0.014 |  |  |  |  |
| rs427943 | 21 | 46570896 | A | C | -0.018 | 0.002 | 1.37E-14 | -0.009 | 0.002 | 3.70E-05 | BMI | 0.008 | 0.014 |  |  |  |  |
| rs2092829 | 22 | 31780514 | G | A | -0.007 | 0.003 | 4.45E-03 | 0.014 | 0.002 | 1.30E-08 | TV | -0.016 | 0.015 |  |  |  |  |
| rs28489620 | 22 | 41804716 | A | G | -0.018 | 0.003 | 6.90E-10 | -0.012 | 0.002 | 1.30E-06 | BMI | 0.023 | 0.016 |  |  |  |  |
| rs13053342 | 22 | 48871624 | A | G | 0.015 | 0.002 | 1.32E-10 | 0.006 | 0.002 | 5.90E-03 | BMI | 0.008 | 0.014 |  |  |  |  |

| **Table S18. Beta estimates for the SNPs included in the multivariable MR of years of education, leisure computer use, and breast cancer risk** | | | | | | | | | | | | | | | | | | | | | | | |
| --- | --- | --- | --- | --- | --- | --- | --- | --- | --- | --- | --- | --- | --- | --- | --- | --- | --- | --- | --- | --- | --- | --- | --- |
| SNP | CHR | POS | EA | OA | beta_ed | se_ed | pval_ed | beta_pc | se_pc | pval | Phenotype | beta_br | se_br | Luminal_A  _beta | Luminal_A  _se | Luminal_B  _beta | Luminal_B  _se | Luminal_B  _HER2Neg_  beta | Luminal_B  _HER2Neg  _se | HER2_  Enriched_  beta | HER2_  Enriched_  se | Triple_Neg  _beta | Triple_Neg  _se |
| rs2748985 | 1 | 1853184 | T | C | -0.006 | 0.002 | 4.16E-04 | -0.015 | 0.002 | 4.10E-12 | PC | 0.003 | 0.007 | 0.007 | 0.009 | 0.017 | 0.019 | -0.002 | 0.017 | -0.011 | 0.028 | 0.014 | 0.017 |
| rs11121177 | 1 | 8445946 | A | G | 0.015 | 0.002 | 1.44E-16 | 0.007 | 0.003 | 1.20E-02 | Education | 0.013 | 0.008 | 0.030 | 0.011 | -0.031 | 0.024 | -0.017 | 0.020 | -0.034 | 0.034 | 0.013 | 0.020 |
| rs10799615 | 1 | 20541370 | A | G | -0.009 | 0.002 | 4.11E-08 | 0.001 | 0.003 | 7.30E-01 | Education | 0.000 | 0.007 | -0.003 | 0.010 | 0.032 | 0.021 | -0.017 | 0.018 | 0.033 | 0.031 | 0.012 | 0.018 |
| rs590013 | 1 | 29155738 | T | C | 0.010 | 0.002 | 2.47E-11 | 0.004 | 0.002 | 8.70E-02 | Education | -0.013 | 0.006 | -0.006 | 0.008 | 0.007 | 0.019 | -0.004 | 0.016 | -0.029 | 0.027 | -0.004 | 0.016 |
| rs10798888 | 1 | 32199099 | T | G | -0.014 | 0.002 | 5.15E-14 | -0.008 | 0.003 | 7.30E-03 | Education | 0.009 | 0.008 | -0.004 | 0.011 | 0.055 | 0.024 | 0.043 | 0.021 | -0.027 | 0.036 | 0.017 | 0.021 |
| rs2364544 | 1 | 41833162 | A | G | -0.011 | 0.001 | 2.91E-13 | -0.006 | 0.002 | 8.00E-03 | Education | -0.003 | 0.006 | -0.019 | 0.008 | 0.001 | 0.018 | 0.011 | 0.015 | 0.010 | 0.026 | -0.004 | 0.016 |
| rs12076635 | 1 | 44026656 | C | G | 0.021 | 0.002 | 3.33E-34 | -0.002 | 0.003 | 3.70E-01 | Education | 0.016 | 0.007 | 0.013 | 0.010 | 0.044 | 0.022 | 0.012 | 0.019 | 0.007 | 0.031 | 0.001 | 0.019 |
| rs1738050 | 1 | 44707295 | C | G | -0.009 | 0.001 | 1.42E-10 | 0.000 | 0.002 | 9.10E-01 | Education | -0.009 | 0.006 | -0.016 | 0.008 | -0.025 | 0.018 | 0.010 | 0.015 | -0.031 | 0.026 | -0.009 | 0.016 |
| rs11211123 | 1 | 45957609 | A | G | 0.010 | 0.002 | 7.62E-10 | 0.003 | 0.003 | 2.00E-01 | Education | -0.032 | 0.007 | -0.040 | 0.010 | -0.041 | 0.021 | -0.021 | 0.018 | -0.069 | 0.031 | -0.010 | 0.018 |
| rs2764684 | 1 | 58537919 | T | C | 0.014 | 0.002 | 9.99E-15 | 0.004 | 0.003 | 2.10E-01 | Education | 0.013 | 0.009 | 0.014 | 0.011 | -0.005 | 0.025 | 0.025 | 0.022 | 0.064 | 0.037 | -0.008 | 0.022 |
| rs2989476 | 1 | 61059259 | C | G | 0.008 | 0.001 | 3.73E-08 | -0.001 | 0.002 | 6.10E-01 | Education | -0.006 | 0.006 | 0.001 | 0.008 | 0.001 | 0.018 | -0.021 | 0.016 | 0.030 | 0.026 | -0.019 | 0.016 |
| rs4915735 | 1 | 61808444 | A | G | 0.012 | 0.002 | 9.56E-09 | 0.004 | 0.003 | 1.90E-01 | Education | -0.010 | 0.009 | -0.018 | 0.012 | 0.017 | 0.027 | 0.005 | 0.023 | 0.002 | 0.040 | 0.006 | 0.024 |
| rs34305371 | 1 | 72733610 | A | G | 0.031 | 0.002 | 1.10E-39 | 0.015 | 0.004 | 3.60E-05 | Education | -0.007 | 0.011 | -0.005 | 0.015 | 0.030 | 0.032 | -0.044 | 0.028 | -0.009 | 0.047 | -0.048 | 0.029 |
| rs79994730 | 1 | 75562222 | T | C | 0.024 | 0.004 | 5.88E-12 | 0.000 | 0.005 | 9.80E-01 | Education | 0.005 | 0.016 | -0.007 | 0.022 | 0.043 | 0.048 | -0.033 | 0.042 | 0.060 | 0.069 | 0.038 | 0.041 |
| rs1008078 | 1 | 91189731 | T | C | -0.018 | 0.001 | 4.50E-37 | -0.003 | 0.002 | 1.40E-01 | Education | 0.000 | 0.007 | 0.010 | 0.009 | -0.004 | 0.019 | -0.015 | 0.016 | 0.017 | 0.027 | -0.001 | 0.017 |
| rs6690195 | 1 | 96221653 | T | C | -0.011 | 0.001 | 7.89E-16 | -0.007 | 0.002 | 2.30E-03 | Education | 0.010 | 0.006 | 0.002 | 0.008 | 0.041 | 0.018 | 0.007 | 0.015 | 0.004 | 0.026 | 0.014 | 0.015 |
| rs10875121 | 1 | 98417446 | C | G | 0.017 | 0.002 | 4.46E-20 | 0.004 | 0.003 | 2.00E-01 | Education | 0.000 | 0.008 | -0.002 | 0.011 | 0.061 | 0.024 | -0.030 | 0.020 | 0.020 | 0.035 | -0.005 | 0.020 |
| rs12145677 | 1 | 110023610 | G | A | -0.012 | 0.002 | 1.32E-11 | -0.017 | 0.002 | 1.20E-12 | PC | -0.011 | 0.007 | -0.016 | 0.009 | -0.012 | 0.020 | -0.003 | 0.017 | -0.009 | 0.028 | 0.013 | 0.017 |
| rs4839155 | 1 | 112161489 | T | G | 0.012 | 0.002 | 4.66E-12 | 0.007 | 0.003 | 9.20E-03 | Education | -0.005 | 0.007 | -0.011 | 0.009 | -0.005 | 0.021 | -0.025 | 0.018 | 0.036 | 0.031 | -0.019 | 0.018 |
| rs3026996 | 1 | 159167290 | A | C | 0.014 | 0.002 | 5.42E-17 | 0.011 | 0.003 | 8.20E-06 | Education | 0.014 | 0.007 | 0.009 | 0.010 | -0.001 | 0.022 | 0.009 | 0.018 | 0.021 | 0.031 | 0.009 | 0.019 |
| rs34720381 | 1 | 171455322 | T | C | -0.018 | 0.002 | 1.90E-13 | 0.006 | 0.004 | 1.20E-01 | Education | 0.006 | 0.011 | 0.021 | 0.014 | -0.025 | 0.032 | 0.011 | 0.027 | -0.006 | 0.046 | 0.002 | 0.027 |
| rs6697584 | 1 | 181509718 | T | C | -0.013 | 0.002 | 1.43E-13 | -0.001 | 0.003 | 6.60E-01 | Education | 0.002 | 0.008 | 0.000 | 0.010 | 0.041 | 0.023 | -0.020 | 0.020 | -0.015 | 0.033 | 0.019 | 0.020 |
| rs7526456 | 1 | 197773734 | T | C | -0.003 | 0.002 | 8.28E-02 | -0.013 | 0.002 | 1.20E-08 | PC | 0.005 | 0.006 | 0.005 | 0.009 | 0.012 | 0.019 | 0.002 | 0.016 | -0.039 | 0.028 | 0.014 | 0.016 |
| rs1890132 | 1 | 199427321 | T | C | -0.009 | 0.002 | 6.56E-09 | 0.000 | 0.002 | 9.00E-01 | Education | 0.003 | 0.007 | 0.012 | 0.009 | -0.008 | 0.020 | -0.007 | 0.017 | 0.025 | 0.028 | 0.017 | 0.017 |
| rs8024 | 1 | 201845575 | A | C | -0.012 | 0.002 | 1.13E-14 | -0.006 | 0.002 | 1.40E-02 | Education | -0.006 | 0.006 | -0.006 | 0.008 | 0.007 | 0.019 | 0.025 | 0.016 | 0.034 | 0.027 | -0.017 | 0.016 |
| rs3111251 | 1 | 211409844 | T | C | -0.009 | 0.001 | 3.88E-10 | -0.002 | 0.002 | 3.60E-01 | Education | 0.003 | 0.006 | 0.001 | 0.008 | 0.039 | 0.018 | 0.001 | 0.015 | 0.007 | 0.026 | 0.009 | 0.015 |
| rs4846724 | 1 | 221967817 | A | G | 0.010 | 0.001 | 2.89E-12 | 0.005 | 0.002 | 3.20E-02 | Education | -0.001 | 0.006 | -0.001 | 0.009 | 0.014 | 0.019 | -0.005 | 0.016 | 0.023 | 0.027 | 0.003 | 0.016 |
| rs1329125 | 1 | 234740880 | T | C | -0.010 | 0.002 | 8.37E-11 | -0.003 | 0.002 | 2.10E-01 | Education | 0.016 | 0.007 | 0.014 | 0.009 | 0.035 | 0.019 | -0.010 | 0.017 | 0.014 | 0.028 | 0.029 | 0.017 |
| rs114593137 | 1 | 241876565 | A | T | -0.012 | 0.002 | 1.65E-12 | -0.011 | 0.003 | 6.00E-05 | Education | -0.013 | 0.007 | -0.012 | 0.010 | -0.003 | 0.022 | 0.016 | 0.019 | -0.044 | 0.031 | -0.039 | 0.019 |
| rs3897821 | 1 | 243420388 | A | G | 0.015 | 0.002 | 3.58E-25 | 0.009 | 0.002 | 1.20E-04 | Education | -0.015 | 0.006 | -0.022 | 0.008 | -0.002 | 0.019 | -0.027 | 0.016 | -0.003 | 0.027 | -0.024 | 0.016 |
| rs12614263 | 2 | 4123797 | A | G | 0.008 | 0.001 | 3.38E-09 | 0.002 | 0.002 | 2.60E-01 | Education | 0.010 | 0.006 | 0.012 | 0.008 | -0.016 | 0.018 | 0.016 | 0.016 | -0.012 | 0.027 | 0.011 | 0.016 |
| rs7603132 | 2 | 4951548 | A | G | 0.014 | 0.002 | 1.20E-14 | 0.005 | 0.003 | 9.20E-02 | Education | -0.003 | 0.008 | 0.007 | 0.011 | 0.023 | 0.024 | 0.003 | 0.021 | -0.050 | 0.036 | 0.003 | 0.021 |
| rs76076331 | 2 | 10977585 | T | C | 0.021 | 0.002 | 1.71E-23 | 0.003 | 0.003 | 4.00E-01 | Education | 0.002 | 0.009 | 0.010 | 0.012 | 0.002 | 0.027 | 0.011 | 0.023 | 0.033 | 0.039 | -0.016 | 0.024 |
| rs1991585 | 2 | 16647170 | T | C | -0.010 | 0.002 | 6.26E-11 | -0.002 | 0.002 | 3.40E-01 | Education | -0.014 | 0.007 | -0.010 | 0.009 | -0.023 | 0.019 | -0.028 | 0.016 | 0.016 | 0.028 | -0.015 | 0.017 |
| rs4358081 | 2 | 29100642 | A | C | -0.010 | 0.001 | 1.28E-11 | -0.004 | 0.002 | 1.10E-01 | Education | -0.011 | 0.006 | -0.020 | 0.008 | -0.032 | 0.017 | -0.010 | 0.015 | 0.017 | 0.025 | 0.018 | 0.015 |
| rs12468040 | 2 | 44854981 | T | G | 0.014 | 0.001 | 3.12E-21 | -0.001 | 0.002 | 7.60E-01 | Education | 0.006 | 0.006 | 0.006 | 0.008 | -0.001 | 0.018 | 0.004 | 0.016 | -0.008 | 0.027 | -0.023 | 0.016 |
| rs17502934 | 2 | 50994255 | T | G | -0.017 | 0.002 | 2.12E-17 | -0.007 | 0.003 | 3.20E-02 | Education | -0.006 | 0.009 | 0.001 | 0.011 | -0.021 | 0.025 | 0.009 | 0.021 | -0.004 | 0.037 | 0.010 | 0.022 |
| rs3948495 | 2 | 57383133 | T | G | -0.009 | 0.001 | 3.95E-10 | -0.003 | 0.002 | 1.50E-01 | Education | 0.002 | 0.006 | 0.008 | 0.008 | 0.008 | 0.018 | -0.024 | 0.015 | 0.016 | 0.026 | -0.020 | 0.016 |
| rs10189857 | 2 | 60713235 | A | G | 0.016 | 0.001 | 4.69E-29 | 0.005 | 0.002 | 3.20E-02 | Education | -0.017 | 0.006 | -0.019 | 0.008 | -0.011 | 0.018 | -0.022 | 0.015 | -0.008 | 0.026 | -0.015 | 0.015 |
| rs6731373 | 2 | 68503044 | A | G | -0.012 | 0.002 | 1.39E-14 | -0.009 | 0.002 | 7.90E-05 | Education | -0.003 | 0.007 | 0.001 | 0.009 | -0.007 | 0.020 | 0.007 | 0.017 | -0.029 | 0.029 | -0.001 | 0.017 |
| rs112806496 | 2 | 80421805 | C | G | -0.016 | 0.003 | 1.72E-10 | -0.006 | 0.004 | 1.10E-01 | Education | 0.006 | 0.012 | 0.012 | 0.016 | -0.076 | 0.033 | 0.006 | 0.030 | 0.075 | 0.052 | -0.030 | 0.029 |
| rs11894424 | 2 | 80588091 | A | C | 0.021 | 0.003 | 2.14E-10 | -0.001 | 0.005 | 8.90E-01 | Education | -0.006 | 0.014 | -0.008 | 0.019 | -0.038 | 0.040 | 0.063 | 0.036 | -0.039 | 0.058 | 0.004 | 0.036 |
| rs62155350 | 2 | 98932980 | A | G | 0.034 | 0.005 | 1.69E-10 | 0.019 | 0.008 | 1.70E-02 | Education | 0.010 | 0.029 | -0.002 | 0.039 | 0.004 | 0.088 | 0.063 | 0.072 | 0.187 | 0.120 | 0.000 | 0.075 |
| rs13018640 | 2 | 100821545 | T | C | -0.022 | 0.001 | 1.05E-50 | -0.006 | 0.002 | 4.50E-03 | Education | -0.010 | 0.006 | -0.008 | 0.008 | -0.016 | 0.018 | -0.007 | 0.016 | -0.014 | 0.027 | -0.017 | 0.016 |
| rs2570497 | 2 | 104441546 | T | C | -0.012 | 0.002 | 1.27E-16 | -0.007 | 0.002 | 2.30E-03 | Education | 0.006 | 0.006 | 0.008 | 0.008 | -0.018 | 0.018 | -0.009 | 0.015 | 0.027 | 0.026 | 0.006 | 0.016 |
| rs62155873 | 2 | 105969362 | T | C | -0.015 | 0.002 | 1.65E-12 | -0.002 | 0.003 | 5.70E-01 | Education | -0.003 | 0.009 | -0.002 | 0.012 | -0.017 | 0.027 | 0.005 | 0.023 | -0.009 | 0.040 | -0.016 | 0.024 |
| rs13402497 | 2 | 115826318 | A | T | -0.008 | 0.001 | 1.33E-08 | -0.001 | 0.002 | 7.80E-01 | Education | 0.004 | 0.006 | 0.006 | 0.008 | 0.002 | 0.018 | 0.008 | 0.015 | 0.022 | 0.026 | -0.004 | 0.015 |
| rs4848924 | 2 | 124991887 | A | C | -0.012 | 0.002 | 3.56E-14 | -0.006 | 0.002 | 2.40E-02 | Education | -0.001 | 0.007 | 0.009 | 0.009 | -0.010 | 0.019 | -0.010 | 0.016 | 0.025 | 0.028 | -0.004 | 0.017 |
| rs77609760 | 2 | 142358950 | A | G | 0.024 | 0.003 | 7.71E-17 | 0.009 | 0.004 | 3.40E-02 | Education | 0.007 | 0.012 | -0.002 | 0.016 | 0.039 | 0.037 | 0.004 | 0.031 | 0.028 | 0.053 | -0.004 | 0.031 |
| rs1320139 | 2 | 144158177 | C | G | -0.015 | 0.001 | 7.62E-26 | 0.000 | 0.002 | 8.70E-01 | Education | -0.010 | 0.006 | -0.011 | 0.008 | -0.007 | 0.018 | -0.001 | 0.015 | 0.016 | 0.026 | 0.002 | 0.015 |
| rs7575637 | 2 | 145796251 | A | G | 0.011 | 0.001 | 3.43E-14 | 0.007 | 0.002 | 3.10E-03 | Education | -0.016 | 0.006 | -0.021 | 0.008 | 0.009 | 0.018 | -0.021 | 0.015 | 0.024 | 0.026 | -0.020 | 0.015 |
| rs10754920 | 2 | 146456888 | C | G | -0.002 | 0.003 | 3.57E-01 | 0.020 | 0.003 | 6.70E-09 | PC | 0.004 | 0.010 | 0.016 | 0.013 | 0.005 | 0.028 | 0.015 | 0.024 | 0.014 | 0.040 | -0.064 | 0.025 |
| rs6744254 | 2 | 155986195 | C | T | -0.001 | 0.002 | 5.06E-01 | -0.016 | 0.002 | 5.40E-13 | PC | 0.007 | 0.006 | 0.002 | 0.008 | -0.014 | 0.018 | 0.005 | 0.015 | 0.024 | 0.026 | 0.004 | 0.015 |
| rs17428076 | 2 | 172851936 | C | G | 0.014 | 0.002 | 6.41E-17 | 0.002 | 0.003 | 4.90E-01 | Education | 0.003 | 0.007 | 0.011 | 0.009 | -0.037 | 0.020 | 0.012 | 0.018 | 0.051 | 0.030 | -0.012 | 0.018 |
| rs10193498 | 2 | 174094345 | A | T | 0.010 | 0.002 | 3.10E-09 | 0.005 | 0.003 | 7.80E-02 | Education | -0.009 | 0.007 | -0.012 | 0.009 | 0.003 | 0.020 | 0.000 | 0.017 | 0.000 | 0.029 | -0.019 | 0.017 |
| rs62174974 | 2 | 185053385 | A | G | -0.011 | 0.002 | 2.76E-09 | -0.008 | 0.003 | 5.80E-03 | Education | 0.006 | 0.008 | 0.018 | 0.010 | 0.014 | 0.022 | -0.022 | 0.019 | 0.045 | 0.031 | -0.017 | 0.019 |
| rs62179650 | 2 | 189135884 | A | G | 0.011 | 0.002 | 5.66E-13 | 0.006 | 0.002 | 1.40E-02 | Education | -0.010 | 0.007 | -0.015 | 0.009 | -0.014 | 0.020 | -0.015 | 0.017 | 0.010 | 0.029 | 0.000 | 0.017 |
| rs66721975 | 2 | 191701185 | A | G | -0.009 | 0.002 | 1.51E-08 | -0.009 | 0.002 | 4.40E-04 | Education | -0.010 | 0.007 | -0.003 | 0.009 | -0.032 | 0.020 | -0.006 | 0.017 | 0.025 | 0.029 | 0.012 | 0.017 |
| rs10931821 | 2 | 199495830 | A | T | -0.014 | 0.001 | 8.65E-24 | -0.011 | 0.002 | 1.40E-06 | Education | -0.002 | 0.006 | -0.002 | 0.008 | -0.025 | 0.018 | 0.010 | 0.016 | -0.027 | 0.027 | -0.004 | 0.016 |
| rs4675248 | 2 | 202880230 | A | G | -0.009 | 0.001 | 4.50E-11 | -0.007 | 0.002 | 1.40E-03 | Education | -0.010 | 0.006 | -0.003 | 0.008 | -0.009 | 0.018 | -0.035 | 0.016 | 0.061 | 0.026 | 0.015 | 0.016 |
| rs11274218 | 2 | 215035001 | C | T | -0.010 | 0.002 | 2.29E-08 | -0.014 | 0.002 | 2.50E-09 | PC | 0.014 | 0.006 | 0.008 | 0.008 | 0.030 | 0.018 | 0.003 | 0.016 | 0.006 | 0.027 | 0.021 | 0.016 |
| rs4673840 | 2 | 215363241 | T | C | -0.014 | 0.002 | 5.86E-13 | -0.015 | 0.003 | 7.20E-07 | Education | 0.008 | 0.008 | 0.006 | 0.011 | -0.030 | 0.024 | -0.007 | 0.021 | 0.019 | 0.035 | 0.013 | 0.021 |
| rs10208088 | 2 | 221055873 | C | T | 0.003 | 0.002 | 1.13E-01 | 0.013 | 0.002 | 4.40E-09 | PC | 0.001 | 0.006 | 0.002 | 0.008 | -0.021 | 0.018 | 0.012 | 0.015 | 0.000 | 0.026 | 0.006 | 0.015 |
| rs4500930 | 2 | 228985505 | T | C | -0.011 | 0.002 | 1.67E-13 | -0.003 | 0.002 | 2.60E-01 | Education | 0.000 | 0.007 | -0.001 | 0.009 | 0.031 | 0.019 | -0.007 | 0.017 | 0.069 | 0.028 | -0.012 | 0.017 |
| rs6704768 | 2 | 233592501 | A | G | -0.012 | 0.001 | 1.84E-16 | -0.006 | 0.002 | 9.50E-03 | Education | -0.005 | 0.006 | -0.015 | 0.008 | -0.007 | 0.018 | 0.008 | 0.015 | -0.018 | 0.026 | -0.016 | 0.015 |
| rs4663617 | 2 | 236744626 | A | T | 0.010 | 0.002 | 4.12E-10 | 0.001 | 0.003 | 7.10E-01 | Education | 0.007 | 0.008 | 0.006 | 0.010 | 0.017 | 0.022 | -0.008 | 0.019 | 0.048 | 0.032 | -0.002 | 0.019 |
| rs72993796 | 2 | 240321051 | T | C | 0.013 | 0.002 | 1.12E-08 | -0.001 | 0.003 | 6.90E-01 | Education | 0.002 | 0.010 | -0.003 | 0.014 | 0.032 | 0.030 | 0.005 | 0.026 | -0.028 | 0.043 | -0.015 | 0.026 |
| rs17048855 | 3 | 8258174 | A | G | 0.011 | 0.002 | 3.10E-13 | 0.010 | 0.002 | 6.50E-06 | Education | -0.008 | 0.006 | -0.006 | 0.008 | -0.029 | 0.019 | -0.014 | 0.016 | -0.010 | 0.027 | -0.014 | 0.016 |
| rs382196 | 3 | 9145554 | T | G | -0.008 | 0.001 | 1.47E-08 | -0.008 | 0.002 | 5.70E-04 | Education | -0.007 | 0.006 | -0.001 | 0.008 | 0.003 | 0.018 | 0.010 | 0.016 | -0.021 | 0.027 | -0.012 | 0.016 |
| rs9882532 | 3 | 16865845 | T | C | 0.012 | 0.002 | 2.15E-17 | -0.003 | 0.002 | 1.80E-01 | Education | 0.003 | 0.006 | 0.001 | 0.008 | 0.019 | 0.018 | -0.029 | 0.016 | 0.033 | 0.027 | 0.007 | 0.016 |
| rs13085461 | 3 | 24950387 | C | G | 0.010 | 0.001 | 2.40E-13 | 0.002 | 0.002 | 4.30E-01 | Education | -0.005 | 0.006 | -0.007 | 0.008 | 0.034 | 0.018 | 0.012 | 0.015 | -0.009 | 0.026 | -0.012 | 0.015 |
| rs73055556 | 3 | 28027538 | A | G | 0.011 | 0.002 | 4.95E-08 | 0.006 | 0.003 | 6.80E-02 | Education | -0.016 | 0.009 | -0.008 | 0.012 | -0.039 | 0.027 | -0.012 | 0.023 | 0.006 | 0.039 | 0.021 | 0.023 |
| rs4328757 | 3 | 36938180 | T | C | 0.010 | 0.001 | 2.95E-11 | 0.001 | 0.002 | 5.90E-01 | Education | 0.004 | 0.007 | 0.004 | 0.009 | 0.003 | 0.019 | 0.008 | 0.016 | -0.004 | 0.028 | -0.004 | 0.017 |
| rs11708955 | 3 | 49540114 | T | C | -0.028 | 0.002 | 3.18E-53 | -0.016 | 0.002 | 4.20E-11 | PC | 0.023 | 0.007 | 0.018 | 0.009 | 0.016 | 0.019 | 0.025 | 0.016 | 0.062 | 0.028 | 0.053 | 0.016 |
| rs77719387 | 3 | 49917021 | A | T | -0.040 | 0.006 | 3.78E-12 | -0.008 | 0.009 | 3.90E-01 | Education | 0.042 | 0.029 | 0.034 | 0.038 | 0.047 | 0.085 | 0.127 | 0.069 | 0.237 | 0.113 | 0.070 | 0.072 |
| rs6774533 | 3 | 62471086 | C | T | -0.013 | 0.002 | 1.73E-11 | -0.015 | 0.002 | 1.20E-09 | PC | -0.012 | 0.007 | -0.022 | 0.009 | -0.004 | 0.021 | -0.004 | 0.018 | -0.047 | 0.031 | 0.018 | 0.018 |
| rs12638072 | 3 | 65654480 | A | G | -0.011 | 0.002 | 3.13E-12 | -0.004 | 0.002 | 8.60E-02 | Education | -0.013 | 0.007 | -0.012 | 0.009 | 0.003 | 0.019 | -0.030 | 0.017 | 0.010 | 0.028 | 0.016 | 0.017 |
| rs55736314 | 3 | 71586293 | C | G | -0.015 | 0.001 | 4.59E-27 | 0.001 | 0.002 | 6.90E-01 | Education | 0.029 | 0.006 | 0.040 | 0.008 | 0.032 | 0.018 | 0.027 | 0.015 | -0.012 | 0.026 | 0.023 | 0.016 |
| rs66568921 | 3 | 85672018 | T | G | -0.018 | 0.002 | 7.60E-34 | -0.004 | 0.002 | 1.10E-01 | Education | 0.001 | 0.006 | 0.004 | 0.008 | 0.024 | 0.018 | 0.017 | 0.016 | -0.047 | 0.027 | -0.019 | 0.016 |
| rs9853928 | 3 | 103295384 | T | C | -0.013 | 0.002 | 1.59E-13 | -0.003 | 0.003 | 3.00E-01 | Education | -0.016 | 0.007 | -0.007 | 0.009 | -0.038 | 0.021 | -0.025 | 0.018 | -0.025 | 0.031 | -0.017 | 0.018 |
| rs28513882 | 3 | 107296628 | A | G | -0.011 | 0.002 | 2.03E-09 | -0.005 | 0.003 | 5.50E-02 | Education | -0.006 | 0.008 | -0.015 | 0.011 | 0.006 | 0.023 | 0.017 | 0.020 | 0.025 | 0.034 | -0.009 | 0.020 |
| rs2290601 | 3 | 108112760 | T | C | -0.015 | 0.002 | 4.49E-19 | -0.003 | 0.003 | 2.40E-01 | Education | 0.001 | 0.007 | -0.004 | 0.010 | 0.022 | 0.022 | 0.013 | 0.018 | 0.011 | 0.031 | -0.010 | 0.018 |
| rs78082503 | 3 | 116556913 | G | C | -0.006 | 0.002 | 4.59E-03 | -0.015 | 0.003 | 1.10E-08 | PC | 0.004 | 0.008 | -0.001 | 0.010 | 0.022 | 0.022 | 0.013 | 0.019 | 0.054 | 0.032 | 0.012 | 0.019 |
| rs1717204 | 3 | 118461145 | A | C | -0.012 | 0.002 | 1.20E-10 | -0.003 | 0.003 | 2.20E-01 | Education | -0.009 | 0.008 | -0.014 | 0.010 | -0.017 | 0.023 | 0.018 | 0.019 | 0.026 | 0.033 | 0.013 | 0.020 |
| rs34067381 | 3 | 123594419 | T | G | -0.010 | 0.002 | 9.77E-13 | -0.004 | 0.002 | 1.30E-01 | Education | 0.007 | 0.006 | 0.002 | 0.008 | -0.016 | 0.019 | 0.000 | 0.016 | 0.024 | 0.027 | 0.042 | 0.016 |
| rs9289300 | 3 | 127144988 | T | C | -0.016 | 0.002 | 1.00E-17 | -0.005 | 0.003 | 1.00E-01 | Education | 0.005 | 0.008 | -0.004 | 0.011 | 0.060 | 0.025 | 0.000 | 0.021 | 0.000 | 0.036 | -0.001 | 0.021 |
| rs7650602 | 3 | 141147414 | T | C | -0.009 | 0.001 | 2.77E-10 | -0.010 | 0.002 | 2.90E-06 | Education | -0.048 | 0.006 | -0.058 | 0.008 | -0.052 | 0.018 | -0.078 | 0.015 | -0.079 | 0.026 | -0.011 | 0.015 |
| rs2885198 | 3 | 143636421 | A | G | 0.009 | 0.001 | 4.63E-10 | 0.001 | 0.002 | 6.30E-01 | Education | -0.009 | 0.006 | -0.012 | 0.008 | 0.014 | 0.018 | -0.015 | 0.016 | -0.076 | 0.027 | -0.017 | 0.016 |
| rs12054166 | 3 | 150093445 | C | G | 0.010 | 0.002 | 2.30E-09 | -0.001 | 0.003 | 7.70E-01 | Education | 0.010 | 0.007 | 0.012 | 0.010 | 0.016 | 0.021 | 0.037 | 0.019 | 0.024 | 0.031 | -0.016 | 0.019 |
| rs7617204 | 3 | 160821969 | A | G | -0.010 | 0.001 | 4.33E-12 | -0.001 | 0.002 | 6.00E-01 | Education | 0.005 | 0.006 | 0.000 | 0.008 | 0.013 | 0.018 | 0.023 | 0.015 | 0.015 | 0.026 | -0.016 | 0.015 |
| rs72622559 | 3 | 175672897 | T | C | -0.012 | 0.002 | 5.50E-13 | 0.000 | 0.003 | 9.70E-01 | Education | 0.016 | 0.007 | 0.015 | 0.009 | 0.008 | 0.021 | 0.016 | 0.017 | 0.018 | 0.030 | 0.013 | 0.018 |
| rs77025239 | 3 | 180734185 | A | G | -0.014 | 0.002 | 4.65E-13 | -0.011 | 0.003 | 2.40E-04 | Education | 0.010 | 0.008 | 0.027 | 0.011 | -0.008 | 0.024 | -0.007 | 0.021 | 0.021 | 0.035 | -0.013 | 0.021 |
| rs13327482 | 3 | 196788363 | A | G | -0.012 | 0.002 | 4.47E-11 | -0.003 | 0.003 | 2.60E-01 | Education | 0.011 | 0.008 | 0.011 | 0.010 | 0.034 | 0.023 | -0.024 | 0.019 | -0.045 | 0.032 | 0.034 | 0.020 |
| rs150537577 | 4 | 705629 | A | G | -0.015 | 0.003 | 9.50E-09 | -0.013 | 0.004 | 2.60E-03 | Education | 0.016 | 0.011 | 0.011 | 0.015 | 0.052 | 0.034 | 0.015 | 0.028 | -0.038 | 0.047 | 0.057 | 0.029 |
| rs363096 | 4 | 3180021 | T | C | -0.014 | 0.001 | 5.77E-24 | -0.008 | 0.002 | 1.70E-04 | Education | 0.016 | 0.006 | 0.013 | 0.008 | 0.008 | 0.018 | 0.032 | 0.015 | -0.011 | 0.026 | 0.024 | 0.015 |
| rs13133213 | 4 | 15646794 | A | G | 0.010 | 0.001 | 7.57E-12 | -0.002 | 0.002 | 4.10E-01 | Education | -0.010 | 0.006 | -0.007 | 0.008 | -0.015 | 0.017 | 0.000 | 0.015 | -0.030 | 0.025 | -0.002 | 0.015 |
| rs2011603 | 4 | 18025484 | A | G | -0.009 | 0.002 | 1.12E-08 | -0.004 | 0.002 | 8.30E-02 | Education | -0.010 | 0.007 | -0.018 | 0.009 | -0.032 | 0.020 | -0.015 | 0.017 | -0.076 | 0.029 | -0.005 | 0.018 |
| rs4467547 | 4 | 21945933 | T | G | 0.012 | 0.001 | 4.88E-17 | 0.003 | 0.002 | 2.40E-01 | Education | -0.001 | 0.006 | -0.004 | 0.008 | 0.011 | 0.018 | -0.011 | 0.016 | -0.017 | 0.027 | 0.008 | 0.016 |
| rs10021733 | 4 | 28609476 | T | C | -0.016 | 0.003 | 9.45E-10 | 0.005 | 0.003 | 7.20E-02 | Education | -0.007 | 0.009 | -0.021 | 0.011 | 0.016 | 0.025 | -0.016 | 0.022 | 0.018 | 0.036 | 0.000 | 0.022 |
| rs6857629 | 4 | 39684977 | G | A | 0.011 | 0.002 | 6.04E-08 | 0.015 | 0.003 | 1.90E-09 | PC | 0.009 | 0.007 | 0.014 | 0.009 | 0.003 | 0.021 | 0.023 | 0.018 | 0.005 | 0.030 | 0.015 | 0.018 |
| rs2055940 | 4 | 46997913 | A | G | 0.008 | 0.002 | 4.95E-08 | 0.003 | 0.002 | 2.30E-01 | Education | 0.006 | 0.007 | -0.003 | 0.009 | 0.001 | 0.019 | 0.003 | 0.017 | 0.047 | 0.028 | 0.010 | 0.017 |
| rs1505676 | 4 | 62127291 | C | G | 0.008 | 0.002 | 6.67E-09 | 0.001 | 0.002 | 7.10E-01 | Education | -0.002 | 0.007 | -0.002 | 0.009 | 0.005 | 0.019 | -0.025 | 0.016 | -0.011 | 0.028 | -0.039 | 0.017 |
| rs12506221 | 4 | 67899235 | T | G | -0.013 | 0.001 | 4.23E-20 | -0.007 | 0.002 | 2.70E-03 | Education | -0.010 | 0.006 | -0.004 | 0.008 | 0.016 | 0.018 | -0.003 | 0.015 | 0.018 | 0.026 | -0.019 | 0.015 |
| rs2034631 | 4 | 82225529 | T | C | 0.009 | 0.002 | 2.64E-09 | 0.005 | 0.002 | 2.30E-02 | Education | 0.008 | 0.006 | 0.003 | 0.008 | 0.030 | 0.018 | 0.010 | 0.016 | -0.019 | 0.026 | 0.018 | 0.016 |
| rs7692359 | 4 | 83209346 | T | C | -0.011 | 0.002 | 1.40E-10 | -0.002 | 0.003 | 3.70E-01 | Education | -0.003 | 0.008 | 0.013 | 0.010 | -0.014 | 0.022 | 0.016 | 0.020 | 0.003 | 0.033 | -0.025 | 0.020 |
| rs12503522 | 4 | 94543233 | T | C | -0.011 | 0.002 | 3.93E-13 | -0.004 | 0.002 | 1.10E-01 | Education | 0.008 | 0.007 | 0.003 | 0.009 | 0.029 | 0.020 | 0.021 | 0.017 | 0.024 | 0.029 | 0.008 | 0.017 |
| rs7683416 | 4 | 106152984 | T | C | 0.014 | 0.001 | 6.05E-23 | 0.005 | 0.002 | 2.40E-02 | Education | 0.025 | 0.006 | 0.025 | 0.008 | 0.019 | 0.018 | 0.017 | 0.015 | -0.025 | 0.025 | 0.004 | 0.015 |
| rs1955250 | 4 | 118514236 | A | C | -0.014 | 0.003 | 2.26E-08 | -0.001 | 0.004 | 7.70E-01 | Education | -0.002 | 0.011 | 0.001 | 0.014 | -0.032 | 0.031 | 0.012 | 0.027 | 0.062 | 0.046 | 0.033 | 0.027 |
| rs13145650 | 4 | 122963344 | T | C | -0.017 | 0.003 | 8.99E-12 | -0.010 | 0.004 | 9.10E-03 | Education | -0.001 | 0.011 | -0.004 | 0.014 | 0.023 | 0.033 | 0.028 | 0.028 | 0.054 | 0.049 | 0.020 | 0.028 |
| rs10006235 | 4 | 130670108 | T | C | -0.009 | 0.002 | 4.48E-09 | -0.003 | 0.002 | 1.60E-01 | Education | 0.006 | 0.007 | 0.002 | 0.009 | 0.009 | 0.020 | 0.017 | 0.017 | 0.078 | 0.028 | 0.018 | 0.017 |
| rs12643771 | 4 | 140753103 | T | C | 0.013 | 0.002 | 1.32E-17 | -0.006 | 0.002 | 1.70E-02 | Education | -0.008 | 0.007 | 0.003 | 0.009 | -0.007 | 0.020 | -0.024 | 0.018 | 0.007 | 0.030 | -0.043 | 0.018 |
| rs969512 | 4 | 147872742 | A | T | -0.011 | 0.002 | 9.84E-13 | -0.004 | 0.002 | 1.00E-01 | Education | -0.001 | 0.006 | -0.003 | 0.008 | -0.027 | 0.018 | -0.006 | 0.016 | 0.008 | 0.027 | -0.033 | 0.016 |
| rs7672622 | 4 | 157705551 | A | G | -0.009 | 0.002 | 4.72E-08 | -0.001 | 0.003 | 8.40E-01 | Education | -0.006 | 0.007 | -0.010 | 0.009 | -0.019 | 0.020 | 0.009 | 0.018 | -0.045 | 0.029 | 0.001 | 0.018 |
| rs2068625 | 4 | 159856739 | T | C | -0.014 | 0.002 | 3.08E-13 | -0.016 | 0.002 | 4.00E-11 | PC | -0.005 | 0.007 | -0.013 | 0.009 | -0.022 | 0.020 | -0.002 | 0.017 | 0.041 | 0.028 | -0.014 | 0.017 |
| rs12646523 | 4 | 170944698 | T | C | -0.013 | 0.002 | 3.79E-16 | -0.004 | 0.003 | 8.70E-02 | Education | -0.002 | 0.007 | -0.004 | 0.009 | 0.002 | 0.021 | 0.004 | 0.018 | 0.035 | 0.030 | 0.008 | 0.018 |
| rs17598675 | 4 | 176647637 | T | C | -0.012 | 0.001 | 1.37E-16 | -0.006 | 0.002 | 9.80E-03 | Education | -0.003 | 0.007 | 0.003 | 0.009 | -0.002 | 0.019 | -0.002 | 0.016 | 0.015 | 0.028 | -0.005 | 0.017 |
| rs72709560 | 4 | 178540163 | A | G | 0.009 | 0.002 | 2.03E-08 | 0.002 | 0.002 | 4.20E-01 | Education | 0.001 | 0.007 | 0.010 | 0.009 | 0.007 | 0.020 | -0.010 | 0.017 | -0.054 | 0.028 | 0.015 | 0.017 |
| rs1128956 | 4 | 183724005 | T | G | -0.014 | 0.002 | 5.34E-14 | -0.007 | 0.003 | 2.20E-02 | Education | 0.004 | 0.009 | 0.008 | 0.012 | -0.003 | 0.025 | -0.010 | 0.022 | -0.026 | 0.036 | 0.019 | 0.022 |
| rs11724690 | 4 | 186764747 | T | G | 0.009 | 0.002 | 2.08E-09 | 0.006 | 0.002 | 1.30E-02 | Education | 0.001 | 0.007 | 0.010 | 0.009 | -0.023 | 0.021 | 0.003 | 0.018 | 0.012 | 0.030 | 0.012 | 0.018 |
| rs2220599 | 5 | 7378854 | C | G | 0.001 | 0.002 | 5.35E-01 | -0.016 | 0.002 | 2.40E-12 | PC | 0.008 | 0.007 | 0.014 | 0.009 | 0.018 | 0.019 | 0.005 | 0.016 | -0.005 | 0.027 | -0.015 | 0.016 |
| rs2434672 | 5 | 11471879 | A | C | 0.009 | 0.001 | 1.06E-10 | 0.003 | 0.002 | 2.10E-01 | Education | 0.003 | 0.006 | 0.003 | 0.008 | -0.005 | 0.018 | -0.015 | 0.015 | 0.017 | 0.026 | -0.003 | 0.015 |
| rs17563464 | 5 | 26913774 | A | C | -0.015 | 0.002 | 1.74E-17 | -0.008 | 0.003 | 5.00E-03 | Education | 0.002 | 0.008 | -0.005 | 0.011 | -0.048 | 0.024 | 0.045 | 0.020 | -0.032 | 0.035 | 0.031 | 0.021 |
| rs10940921 | 5 | 30808645 | T | G | 0.011 | 0.001 | 1.31E-14 | 0.003 | 0.002 | 1.60E-01 | Education | 0.006 | 0.006 | 0.005 | 0.008 | 0.004 | 0.018 | 0.027 | 0.015 | -0.030 | 0.026 | 0.011 | 0.016 |
| rs2964199 | 5 | 57532775 | T | C | -0.010 | 0.002 | 4.61E-11 | -0.003 | 0.002 | 2.80E-01 | Education | 0.005 | 0.006 | -0.014 | 0.009 | 0.032 | 0.019 | -0.006 | 0.016 | 0.122 | 0.027 | 0.025 | 0.016 |
| rs6449503 | 5 | 60095272 | A | G | 0.019 | 0.001 | 1.84E-39 | 0.009 | 0.002 | 6.20E-05 | Education | -0.004 | 0.006 | -0.006 | 0.008 | -0.001 | 0.018 | -0.019 | 0.015 | -0.010 | 0.026 | 0.004 | 0.016 |
| rs10805383 | 5 | 63034606 | A | G | -0.010 | 0.001 | 2.22E-13 | 0.002 | 0.002 | 3.90E-01 | Education | 0.008 | 0.006 | 0.015 | 0.008 | 0.005 | 0.017 | 0.000 | 0.015 | 0.043 | 0.025 | 0.003 | 0.015 |
| rs42302 | 5 | 74965554 | A | G | 0.009 | 0.002 | 5.96E-09 | 0.006 | 0.002 | 1.10E-02 | Education | 0.017 | 0.006 | 0.013 | 0.008 | -0.017 | 0.019 | 0.021 | 0.016 | 0.048 | 0.027 | 0.014 | 0.016 |
| rs11749912 | 5 | 88065628 | A | G | 0.018 | 0.002 | 1.84E-25 | 0.014 | 0.002 | 4.90E-10 | PC | 0.002 | 0.006 | -0.001 | 0.008 | 0.010 | 0.018 | 0.019 | 0.015 | -0.015 | 0.026 | 0.006 | 0.015 |
| rs12332731 | 5 | 93033082 | A | T | 0.014 | 0.002 | 5.96E-14 | 0.003 | 0.003 | 3.60E-01 | Education | 0.003 | 0.008 | -0.003 | 0.010 | 0.008 | 0.023 | 0.021 | 0.019 | -0.008 | 0.033 | 0.043 | 0.019 |
| rs252991 | 5 | 106767346 | A | G | 0.010 | 0.002 | 6.30E-11 | 0.004 | 0.002 | 5.90E-02 | Education | 0.008 | 0.006 | 0.008 | 0.008 | 0.008 | 0.018 | 0.020 | 0.016 | 0.015 | 0.026 | -0.004 | 0.016 |
| rs1469249 | 5 | 113837198 | G | A | 0.017 | 0.002 | 1.55E-15 | 0.016 | 0.003 | 9.50E-09 | PC | -0.011 | 0.007 | -0.008 | 0.010 | -0.023 | 0.021 | -0.006 | 0.018 | -0.013 | 0.031 | -0.019 | 0.019 |
| rs62379838 | 5 | 120102028 | T | C | 0.012 | 0.002 | 1.77E-15 | -0.002 | 0.002 | 3.40E-01 | Education | 0.000 | 0.007 | 0.006 | 0.009 | -0.006 | 0.019 | 0.010 | 0.016 | -0.026 | 0.028 | -0.005 | 0.016 |
| rs162894 | 5 | 131611872 | T | G | 0.005 | 0.002 | 3.26E-03 | 0.013 | 0.002 | 1.10E-08 | PC | -0.024 | 0.006 | -0.034 | 0.008 | -0.048 | 0.019 | -0.031 | 0.016 | 0.013 | 0.027 | -0.030 | 0.016 |
| rs56158102 | 5 | 144027803 | C | T | 0.005 | 0.002 | 4.09E-03 | 0.013 | 0.002 | 3.10E-08 | PC | -0.015 | 0.006 | -0.012 | 0.008 | -0.042 | 0.019 | -0.016 | 0.016 | -0.054 | 0.027 | -0.015 | 0.016 |
| rs2964255 | 5 | 152052332 | A | G | 0.009 | 0.002 | 6.34E-09 | -0.006 | 0.002 | 1.60E-02 | Education | -0.002 | 0.007 | 0.001 | 0.009 | 0.028 | 0.019 | -0.014 | 0.017 | -0.031 | 0.028 | -0.025 | 0.017 |
| rs42210 | 5 | 166408788 | C | G | -0.010 | 0.002 | 2.74E-10 | 0.002 | 0.002 | 3.50E-01 | Education | 0.020 | 0.007 | 0.008 | 0.010 | 0.046 | 0.021 | 0.046 | 0.018 | 0.083 | 0.031 | 0.019 | 0.018 |
| rs2545795 | 5 | 176887106 | A | C | 0.013 | 0.001 | 1.03E-18 | 0.008 | 0.002 | 3.30E-04 | Education | 0.009 | 0.006 | 0.005 | 0.008 | 0.031 | 0.018 | 0.007 | 0.015 | 0.006 | 0.026 | 0.004 | 0.015 |
| rs12524795 | 6 | 3464074 | T | C | 0.010 | 0.001 | 5.28E-13 | 0.006 | 0.002 | 1.20E-02 | Education | -0.003 | 0.007 | -0.010 | 0.009 | 0.002 | 0.019 | -0.001 | 0.016 | -0.002 | 0.027 | -0.029 | 0.017 |
| rs112603734 | 6 | 6069588 | A | C | -0.013 | 0.002 | 2.72E-09 | -0.004 | 0.003 | 1.30E-01 | Education | 0.005 | 0.007 | 0.003 | 0.010 | -0.003 | 0.021 | -0.010 | 0.018 | 0.001 | 0.031 | 0.042 | 0.019 |
| rs72829857 | 6 | 16966052 | A | G | -0.015 | 0.002 | 1.52E-19 | -0.002 | 0.003 | 4.80E-01 | Education | -0.022 | 0.007 | -0.020 | 0.009 | -0.034 | 0.020 | -0.028 | 0.017 | -0.029 | 0.029 | -0.022 | 0.018 |
| rs9477970 | 6 | 19082663 | T | A | -0.017 | 0.002 | 3.16E-14 | -0.016 | 0.003 | 1.00E-08 | PC | -0.002 | 0.008 | 0.001 | 0.010 | -0.001 | 0.023 | 0.017 | 0.020 | 0.007 | 0.033 | -0.002 | 0.020 |
| rs2179152 | 6 | 26325888 | T | C | -0.013 | 0.002 | 1.68E-19 | -0.004 | 0.002 | 5.20E-02 | Education | 0.002 | 0.006 | 0.013 | 0.008 | 0.011 | 0.018 | -0.005 | 0.016 | -0.026 | 0.027 | -0.006 | 0.016 |
| rs2256965 | 6 | 31555130 | A | G | 0.011 | 0.001 | 2.40E-13 | 0.005 | 0.002 | 2.70E-02 | Education | 0.011 | 0.006 | 0.018 | 0.008 | 0.011 | 0.018 | -0.005 | 0.015 | 0.037 | 0.026 | -0.011 | 0.015 |
| rs57349798 | 6 | 37486052 | A | G | 0.009 | 0.001 | 8.63E-11 | -0.003 | 0.002 | 2.60E-01 | Education | -0.005 | 0.006 | -0.005 | 0.008 | 0.017 | 0.018 | -0.005 | 0.016 | -0.011 | 0.027 | -0.011 | 0.016 |
| rs2436760 | 6 | 40374288 | T | C | 0.023 | 0.004 | 4.52E-08 | 0.007 | 0.006 | 3.10E-01 | Education | -0.005 | 0.020 | -0.019 | 0.026 | 0.095 | 0.060 | -0.032 | 0.048 | 0.021 | 0.085 | 0.041 | 0.051 |
| rs912883 | 6 | 41552040 | T | C | 0.009 | 0.002 | 6.20E-09 | 0.001 | 0.002 | 7.90E-01 | Education | 0.000 | 0.006 | 0.004 | 0.008 | -0.015 | 0.019 | -0.007 | 0.016 | -0.010 | 0.027 | 0.060 | 0.016 |
| rs78648104 | 6 | 50683009 | T | C | -0.014 | 0.003 | 1.70E-08 | 0.008 | 0.004 | 3.60E-02 | Education | -0.024 | 0.011 | -0.021 | 0.014 | -0.014 | 0.031 | 0.010 | 0.027 | -0.080 | 0.044 | -0.032 | 0.027 |
| rs7449561 | 6 | 66215549 | A | G | 0.010 | 0.002 | 9.73E-10 | 0.010 | 0.003 | 2.40E-04 | Education | 0.005 | 0.007 | 0.006 | 0.010 | -0.002 | 0.022 | 0.006 | 0.019 | 0.021 | 0.031 | 0.003 | 0.019 |
| rs13212041 | 6 | 78171124 | T | C | 0.010 | 0.002 | 7.42E-09 | 0.005 | 0.003 | 4.60E-02 | Education | 0.015 | 0.008 | 0.019 | 0.010 | 0.001 | 0.022 | -0.011 | 0.019 | 0.025 | 0.033 | -0.013 | 0.019 |
| rs4352658 | 6 | 88279872 | T | C | -0.019 | 0.003 | 1.71E-13 | -0.007 | 0.004 | 9.10E-02 | Education | 0.006 | 0.011 | 0.003 | 0.015 | -0.019 | 0.033 | 0.001 | 0.028 | 0.121 | 0.045 | 0.008 | 0.028 |
| rs9372625 | 6 | 98344031 | G | A | -0.024 | 0.002 | 6.76E-42 | -0.018 | 0.002 | 5.70E-16 | PC | 0.005 | 0.006 | 0.009 | 0.008 | 0.003 | 0.019 | 0.003 | 0.016 | -0.008 | 0.027 | 0.006 | 0.016 |
| rs10080647 | 6 | 114742335 | A | C | 0.012 | 0.002 | 7.05E-10 | 0.001 | 0.003 | 7.40E-01 | Education | -0.007 | 0.009 | 0.000 | 0.011 | 0.041 | 0.025 | 0.019 | 0.021 | -0.003 | 0.037 | -0.018 | 0.022 |
| rs11542663 | 6 | 119215402 | A | C | 0.011 | 0.002 | 2.44E-13 | -0.001 | 0.002 | 7.60E-01 | Education | 0.004 | 0.007 | -0.008 | 0.009 | 0.049 | 0.020 | -0.001 | 0.017 | 0.006 | 0.028 | -0.010 | 0.017 |
| rs13197257 | 6 | 128333682 | T | G | 0.011 | 0.002 | 2.83E-12 | 0.003 | 0.002 | 2.10E-01 | Education | -0.014 | 0.007 | -0.014 | 0.010 | -0.043 | 0.021 | -0.056 | 0.018 | -0.034 | 0.031 | -0.007 | 0.018 |
| rs6935828 | 6 | 140811367 | C | T | -0.005 | 0.002 | 6.57E-03 | -0.012 | 0.002 | 4.30E-08 | PC | -0.004 | 0.006 | -0.003 | 0.008 | 0.018 | 0.018 | 0.007 | 0.015 | -0.028 | 0.026 | 0.001 | 0.015 |
| rs4895650 | 6 | 145024122 | T | C | 0.009 | 0.001 | 5.31E-11 | 0.001 | 0.002 | 8.10E-01 | Education | 0.003 | 0.006 | -0.008 | 0.008 | 0.036 | 0.018 | -0.006 | 0.015 | 0.018 | 0.026 | 0.008 | 0.016 |
| rs6557171 | 6 | 152234593 | T | C | -0.016 | 0.002 | 5.48E-25 | -0.006 | 0.002 | 6.10E-03 | Education | -0.016 | 0.006 | -0.007 | 0.008 | -0.007 | 0.019 | -0.030 | 0.016 | -0.016 | 0.027 | -0.045 | 0.016 |
| rs1933264 | 6 | 153391618 | T | C | 0.010 | 0.002 | 5.85E-10 | -0.002 | 0.003 | 3.40E-01 | Education | 0.001 | 0.007 | 0.003 | 0.009 | 0.005 | 0.020 | -0.029 | 0.017 | -0.012 | 0.029 | 0.012 | 0.018 |
| rs6917154 | 6 | 167135156 | T | C | 0.013 | 0.002 | 2.55E-09 | -0.004 | 0.003 | 2.80E-01 | Education | -0.002 | 0.010 | -0.010 | 0.014 | -0.026 | 0.030 | 0.002 | 0.025 | -0.051 | 0.044 | 0.002 | 0.026 |
| rs6924023 | 6 | 170076041 | A | G | 0.011 | 0.002 | 5.17E-11 | 0.006 | 0.003 | 1.60E-02 | Education | 0.005 | 0.007 | -0.001 | 0.009 | 0.033 | 0.021 | -0.008 | 0.018 | 0.041 | 0.031 | 0.017 | 0.018 |
| rs13240401 | 7 | 2194396 | T | C | 0.018 | 0.002 | 2.36E-24 | 0.008 | 0.003 | 2.90E-03 | Education | -0.007 | 0.007 | -0.006 | 0.010 | 0.061 | 0.022 | 0.014 | 0.019 | -0.034 | 0.032 | -0.023 | 0.019 |
| rs4719944 | 7 | 3496032 | T | C | -0.012 | 0.001 | 9.25E-18 | -0.003 | 0.002 | 1.20E-01 | Education | -0.014 | 0.006 | -0.003 | 0.008 | -0.029 | 0.018 | -0.018 | 0.015 | -0.066 | 0.026 | 0.009 | 0.015 |
| rs929511 | 7 | 8003017 | T | C | -0.016 | 0.002 | 3.91E-14 | -0.011 | 0.003 | 1.30E-03 | Education | 0.000 | 0.010 | 0.009 | 0.013 | -0.028 | 0.029 | 0.006 | 0.024 | -0.010 | 0.041 | -0.023 | 0.025 |
| rs118134876 | 7 | 11500372 | T | C | -0.022 | 0.003 | 1.50E-13 | -0.003 | 0.005 | 5.90E-01 | Education | 0.009 | 0.013 | 0.020 | 0.018 | 0.009 | 0.039 | -0.056 | 0.034 | 0.064 | 0.055 | 0.076 | 0.033 |
| rs6977237 | 7 | 14027991 | T | C | 0.008 | 0.001 | 5.59E-09 | -0.001 | 0.002 | 6.30E-01 | Education | -0.003 | 0.006 | -0.010 | 0.008 | 0.022 | 0.018 | -0.019 | 0.015 | -0.032 | 0.026 | 0.020 | 0.016 |
| rs35929923 | 7 | 21400525 | A | G | -0.011 | 0.002 | 1.77E-12 | 0.000 | 0.003 | 9.40E-01 | Education | 0.004 | 0.007 | 0.001 | 0.009 | 0.014 | 0.020 | 0.004 | 0.017 | -0.022 | 0.030 | -0.001 | 0.018 |
| rs79265434 | 7 | 24621381 | A | G | -0.020 | 0.002 | 1.98E-19 | -0.011 | 0.003 | 1.70E-03 | Education | -0.006 | 0.010 | -0.012 | 0.013 | -0.014 | 0.029 | 0.017 | 0.025 | 0.019 | 0.042 | -0.053 | 0.024 |
| rs10951590 | 7 | 39091043 | T | C | -0.011 | 0.002 | 3.62E-13 | -0.005 | 0.002 | 3.80E-02 | Education | 0.011 | 0.006 | 0.018 | 0.008 | 0.031 | 0.019 | 0.007 | 0.016 | 0.042 | 0.027 | 0.001 | 0.016 |
| rs12670376 | 7 | 48823154 | A | G | 0.009 | 0.001 | 5.99E-11 | 0.002 | 0.002 | 4.70E-01 | Education | 0.001 | 0.006 | -0.012 | 0.008 | -0.007 | 0.018 | 0.014 | 0.015 | -0.002 | 0.026 | 0.021 | 0.015 |
| rs17551064 | 7 | 49869771 | A | G | 0.014 | 0.002 | 1.53E-13 | -0.001 | 0.003 | 6.50E-01 | Education | 0.001 | 0.009 | -0.014 | 0.012 | 0.038 | 0.026 | 0.013 | 0.022 | -0.071 | 0.036 | -0.036 | 0.022 |
| rs1220779 | 7 | 54734673 | A | G | -0.008 | 0.001 | 1.58E-08 | -0.004 | 0.002 | 1.00E-01 | Education | 0.005 | 0.006 | 0.006 | 0.008 | 0.002 | 0.018 | 0.003 | 0.015 | -0.037 | 0.026 | 0.017 | 0.015 |
| rs55772938 | 7 | 70025113 | A | G | -0.007 | 0.002 | 6.27E-05 | -0.015 | 0.002 | 3.90E-10 | PC | 0.003 | 0.007 | -0.003 | 0.009 | 0.020 | 0.019 | -0.024 | 0.016 | 0.042 | 0.028 | 0.015 | 0.017 |
| rs10215082 | 7 | 92657985 | A | G | -0.015 | 0.001 | 7.03E-26 | -0.008 | 0.002 | 1.40E-04 | Education | -0.001 | 0.006 | -0.004 | 0.008 | -0.016 | 0.018 | 0.016 | 0.015 | -0.013 | 0.026 | -0.001 | 0.016 |
| rs401966 | 7 | 101695817 | C | G | -0.010 | 0.001 | 1.72E-11 | 0.006 | 0.002 | 5.30E-03 | Education | 0.012 | 0.006 | 0.019 | 0.008 | 0.002 | 0.018 | 0.011 | 0.015 | -0.028 | 0.026 | 0.000 | 0.016 |
| rs9655780 | 7 | 104667334 | A | G | -0.015 | 0.002 | 3.70E-16 | -0.002 | 0.003 | 5.10E-01 | Education | -0.008 | 0.008 | -0.006 | 0.011 | 0.004 | 0.025 | -0.009 | 0.021 | 0.087 | 0.037 | -0.044 | 0.021 |
| rs12113634 | 7 | 111996952 | T | C | 0.009 | 0.002 | 2.50E-09 | 0.003 | 0.002 | 1.50E-01 | Education | 0.001 | 0.006 | -0.002 | 0.008 | 0.011 | 0.019 | -0.004 | 0.016 | 0.000 | 0.027 | 0.007 | 0.016 |
| rs113615161 | 7 | 114519339 | T | C | -0.012 | 0.002 | 2.98E-09 | -0.009 | 0.003 | 5.20E-03 | Education | -0.001 | 0.009 | 0.002 | 0.013 | -0.033 | 0.028 | 0.025 | 0.023 | 0.041 | 0.040 | 0.003 | 0.024 |
| rs6969783 | 7 | 117593308 | A | T | -0.010 | 0.001 | 2.87E-12 | 0.001 | 0.002 | 6.20E-01 | Education | 0.001 | 0.006 | 0.020 | 0.008 | -0.017 | 0.018 | 0.001 | 0.015 | -0.074 | 0.026 | 0.003 | 0.015 |
| rs12706626 | 7 | 124531370 | G | A | -0.003 | 0.002 | 8.52E-02 | -0.013 | 0.002 | 1.40E-08 | PC | -0.009 | 0.006 | -0.016 | 0.008 | 0.012 | 0.018 | -0.015 | 0.015 | 0.006 | 0.026 | 0.004 | 0.016 |
| rs3944151 | 7 | 126970005 | A | G | 0.004 | 0.002 | 2.25E-02 | 0.014 | 0.002 | 3.10E-08 | PC | 0.016 | 0.007 | 0.013 | 0.009 | 0.003 | 0.020 | 0.015 | 0.017 | 0.037 | 0.028 | 0.010 | 0.017 |
| rs113520408 | 7 | 128402782 | A | G | 0.016 | 0.002 | 1.03E-22 | 0.006 | 0.002 | 2.00E-02 | Education | -0.002 | 0.007 | -0.001 | 0.009 | 0.042 | 0.020 | -0.029 | 0.018 | -0.057 | 0.031 | -0.003 | 0.018 |
| rs2345941 | 7 | 133337635 | A | G | 0.010 | 0.002 | 8.41E-09 | 0.015 | 0.002 | 4.00E-11 | PC | 0.007 | 0.006 | 0.002 | 0.008 | 0.036 | 0.018 | 0.007 | 0.015 | 0.019 | 0.026 | -0.002 | 0.015 |
| rs4726070 | 7 | 151328218 | A | G | 0.012 | 0.001 | 1.36E-17 | 0.004 | 0.002 | 1.10E-01 | Education | -0.003 | 0.006 | -0.002 | 0.008 | -0.023 | 0.018 | 0.026 | 0.016 | -0.034 | 0.027 | 0.005 | 0.016 |
| rs73191311 | 8 | 4796094 | A | G | -0.009 | 0.002 | 2.03E-10 | -0.002 | 0.002 | 3.80E-01 | Education | 0.001 | 0.007 | 0.009 | 0.009 | 0.009 | 0.019 | -0.021 | 0.016 | -0.038 | 0.028 | 0.020 | 0.016 |
| rs6994287 | 8 | 9340932 | A | G | 0.010 | 0.001 | 1.11E-12 | 0.004 | 0.002 | 7.30E-02 | Education | 0.000 | 0.006 | 0.005 | 0.008 | -0.020 | 0.018 | 0.000 | 0.015 | -0.013 | 0.026 | -0.013 | 0.016 |
| rs2517086 | 8 | 17042416 | C | G | -0.009 | 0.001 | 8.00E-10 | 0.001 | 0.002 | 8.00E-01 | Education | 0.002 | 0.006 | -0.004 | 0.008 | 0.011 | 0.018 | 0.006 | 0.015 | -0.030 | 0.025 | 0.005 | 0.015 |
| rs13266287 | 8 | 17871598 | A | T | -0.009 | 0.002 | 1.75E-08 | 0.001 | 0.002 | 7.60E-01 | Education | -0.022 | 0.006 | -0.017 | 0.008 | -0.038 | 0.019 | -0.040 | 0.016 | 0.002 | 0.027 | -0.021 | 0.016 |
| rs34807077 | 8 | 28680769 | A | C | 0.013 | 0.002 | 3.29E-11 | -0.002 | 0.003 | 4.10E-01 | Education | -0.008 | 0.008 | -0.020 | 0.011 | 0.035 | 0.025 | -0.024 | 0.021 | 0.006 | 0.036 | -0.023 | 0.021 |
| rs2725370 | 8 | 30852826 | T | C | -0.014 | 0.002 | 4.96E-20 | -0.004 | 0.002 | 9.20E-02 | Education | 0.002 | 0.007 | -0.003 | 0.009 | 0.029 | 0.019 | -0.011 | 0.017 | -0.014 | 0.028 | 0.002 | 0.017 |
| rs2923424 | 8 | 42382222 | A | G | 0.012 | 0.001 | 6.31E-17 | 0.001 | 0.002 | 5.40E-01 | Education | 0.011 | 0.007 | 0.026 | 0.009 | 0.014 | 0.019 | -0.022 | 0.016 | 0.022 | 0.028 | 0.012 | 0.017 |
| rs2929032 | 8 | 56371745 | A | G | -0.008 | 0.001 | 8.40E-09 | -0.004 | 0.002 | 8.60E-02 | Education | 0.007 | 0.006 | 0.005 | 0.008 | 0.008 | 0.018 | 0.009 | 0.015 | 0.014 | 0.026 | -0.002 | 0.015 |
| rs1866823 | 8 | 57436577 | A | G | 0.011 | 0.001 | 3.09E-14 | 0.001 | 0.002 | 5.40E-01 | Education | -0.016 | 0.007 | -0.005 | 0.009 | 0.014 | 0.019 | -0.015 | 0.016 | -0.011 | 0.028 | -0.019 | 0.017 |
| rs56099375 | 8 | 77372988 | T | C | 0.012 | 0.002 | 3.10E-13 | 0.001 | 0.003 | 6.10E-01 | Education | -0.003 | 0.008 | 0.007 | 0.010 | -0.035 | 0.022 | -0.006 | 0.019 | 0.004 | 0.032 | -0.001 | 0.019 |
| rs4320563 | 8 | 87769961 | A | G | 0.011 | 0.001 | 2.24E-14 | 0.001 | 0.002 | 5.40E-01 | Education | -0.003 | 0.006 | -0.002 | 0.008 | 0.004 | 0.017 | -0.021 | 0.015 | -0.030 | 0.025 | -0.011 | 0.015 |
| rs35606437 | 8 | 93327532 | A | G | 0.011 | 0.002 | 3.98E-11 | 0.008 | 0.002 | 1.10E-03 | Education | 0.002 | 0.007 | -0.010 | 0.009 | 0.038 | 0.020 | 0.018 | 0.017 | 0.011 | 0.029 | -0.018 | 0.018 |
| rs7012546 | 8 | 105067737 | T | C | 0.009 | 0.001 | 1.25E-10 | 0.002 | 0.002 | 4.10E-01 | Education | 0.004 | 0.006 | 0.003 | 0.008 | 0.013 | 0.018 | -0.001 | 0.015 | -0.010 | 0.026 | 0.003 | 0.016 |
| rs72672052 | 8 | 115798424 | A | T | -0.010 | 0.002 | 4.87E-08 | 0.000 | 0.003 | 9.10E-01 | Education | 0.005 | 0.008 | 0.004 | 0.010 | 0.015 | 0.023 | -0.023 | 0.020 | 0.044 | 0.033 | 0.019 | 0.020 |
| rs4298514 | 8 | 118932887 | T | C | 0.010 | 0.002 | 1.07E-11 | 0.008 | 0.002 | 6.90E-04 | Education | 0.026 | 0.007 | 0.016 | 0.009 | 0.030 | 0.019 | 0.028 | 0.017 | 0.076 | 0.029 | 0.001 | 0.017 |
| rs13261773 | 8 | 120187090 | C | G | 0.012 | 0.002 | 3.12E-10 | 0.008 | 0.003 | 1.20E-02 | Education | 0.007 | 0.008 | 0.000 | 0.011 | 0.018 | 0.024 | 0.028 | 0.020 | -0.027 | 0.035 | -0.009 | 0.021 |
| rs401526 | 8 | 130929166 | T | C | -0.010 | 0.001 | 1.15E-13 | -0.005 | 0.002 | 3.00E-02 | Education | -0.002 | 0.006 | -0.002 | 0.008 | -0.011 | 0.018 | 0.004 | 0.015 | 0.036 | 0.026 | 0.021 | 0.015 |
| rs746839 | 8 | 142617261 | C | G | 0.014 | 0.002 | 1.19E-20 | 0.006 | 0.002 | 8.20E-03 | Education | 0.023 | 0.006 | 0.023 | 0.008 | 0.008 | 0.018 | 0.037 | 0.016 | 0.027 | 0.027 | 0.002 | 0.016 |
| rs13262595 | 8 | 143316970 | A | G | -0.012 | 0.002 | 1.85E-11 | -0.016 | 0.002 | 1.20E-12 | PC | -0.001 | 0.006 | 0.005 | 0.008 | -0.016 | 0.018 | 0.013 | 0.015 | 0.043 | 0.026 | -0.005 | 0.015 |
| rs7849487 | 9 | 1757274 | T | G | -0.017 | 0.002 | 1.25E-29 | -0.003 | 0.002 | 2.10E-01 | Education | -0.001 | 0.006 | -0.005 | 0.008 | -0.002 | 0.018 | 0.000 | 0.016 | -0.042 | 0.027 | 0.006 | 0.016 |
| rs73648455 | 9 | 14077378 | T | C | -0.018 | 0.003 | 1.99E-12 | -0.006 | 0.004 | 1.30E-01 | Education | -0.003 | 0.013 | -0.002 | 0.017 | 0.033 | 0.036 | 0.014 | 0.032 | -0.064 | 0.055 | -0.007 | 0.032 |
| rs7875078 | 9 | 14494845 | A | C | -0.008 | 0.001 | 2.72E-08 | 0.002 | 0.002 | 4.20E-01 | Education | 0.001 | 0.006 | 0.003 | 0.008 | 0.018 | 0.018 | -0.018 | 0.015 | -0.009 | 0.026 | 0.011 | 0.015 |
| rs4977839 | 9 | 23355310 | G | A | -0.024 | 0.002 | 2.96E-44 | -0.020 | 0.002 | 4.70E-19 | PC | -0.006 | 0.006 | -0.001 | 0.008 | -0.037 | 0.019 | -0.006 | 0.016 | 0.019 | 0.027 | -0.022 | 0.016 |
| rs4977885 | 9 | 23687983 | A | G | -0.008 | 0.001 | 6.78E-09 | -0.004 | 0.002 | 6.90E-02 | Education | 0.009 | 0.007 | -0.001 | 0.009 | -0.002 | 0.019 | 0.014 | 0.017 | 0.029 | 0.028 | 0.012 | 0.017 |
| rs7041702 | 9 | 33053430 | A | G | -0.011 | 0.002 | 4.53E-12 | -0.004 | 0.003 | 1.20E-01 | Education | 0.004 | 0.007 | 0.009 | 0.009 | 0.035 | 0.020 | 0.007 | 0.017 | -0.017 | 0.029 | 0.003 | 0.017 |
| rs11138947 | 9 | 72110562 | T | C | 0.011 | 0.002 | 2.36E-11 | 0.006 | 0.002 | 1.10E-02 | Education | -0.005 | 0.007 | 0.005 | 0.009 | 0.006 | 0.020 | -0.008 | 0.017 | -0.031 | 0.029 | 0.013 | 0.017 |
| rs9886703 | 9 | 82246351 | A | T | -0.014 | 0.002 | 4.46E-13 | 0.001 | 0.003 | 8.30E-01 | Education | -0.010 | 0.008 | -0.024 | 0.011 | -0.005 | 0.023 | -0.013 | 0.020 | -0.007 | 0.034 | 0.014 | 0.020 |
| rs10761251 | 9 | 96421546 | A | T | 0.011 | 0.002 | 2.52E-14 | 0.001 | 0.002 | 7.20E-01 | Education | -0.001 | 0.006 | -0.008 | 0.008 | 0.001 | 0.019 | 0.020 | 0.016 | -0.029 | 0.027 | 0.013 | 0.016 |
| rs113851275 | 9 | 98297220 | G | A | -0.003 | 0.003 | 2.96E-01 | -0.021 | 0.004 | 3.20E-09 | PC | -0.028 | 0.010 | -0.044 | 0.012 | -0.025 | 0.028 | -0.009 | 0.024 | 0.010 | 0.041 | 0.008 | 0.024 |
| rs7020477 | 9 | 116827760 | A | G | 0.005 | 0.002 | 2.23E-02 | 0.014 | 0.002 | 1.20E-08 | PC | -0.005 | 0.008 | -0.011 | 0.010 | -0.012 | 0.022 | 0.007 | 0.019 | 0.007 | 0.033 | 0.037 | 0.020 |
| rs73578186 | 9 | 126334485 | C | T | 0.010 | 0.002 | 1.02E-07 | 0.015 | 0.002 | 2.30E-10 | PC | -0.003 | 0.006 | -0.011 | 0.008 | 0.011 | 0.019 | -0.021 | 0.016 | -0.002 | 0.027 | -0.028 | 0.016 |
| rs2039204 | 9 | 130371107 | A | T | 0.008 | 0.001 | 5.23E-09 | 0.002 | 0.002 | 4.50E-01 | Education | 0.001 | 0.006 | 0.002 | 0.008 | -0.006 | 0.018 | 0.008 | 0.015 | -0.001 | 0.026 | -0.018 | 0.015 |
| rs73581580 | 9 | 140251458 | A | G | -0.018 | 0.002 | 5.78E-16 | 0.008 | 0.003 | 1.10E-02 | Education | -0.001 | 0.012 | 0.016 | 0.016 | -0.044 | 0.036 | 0.042 | 0.030 | 0.000 | 0.051 | -0.049 | 0.031 |
| rs7894722 | 10 | 9972046 | T | C | 0.009 | 0.001 | 5.41E-09 | 0.002 | 0.002 | 4.70E-01 | Education | -0.001 | 0.006 | -0.009 | 0.008 | 0.016 | 0.018 | 0.005 | 0.016 | 0.015 | 0.026 | 0.015 | 0.016 |
| rs17148998 | 10 | 10909401 | A | G | 0.010 | 0.002 | 6.98E-09 | 0.010 | 0.003 | 4.20E-04 | Education | -0.020 | 0.008 | -0.024 | 0.010 | -0.011 | 0.023 | 0.006 | 0.020 | 0.013 | 0.033 | -0.022 | 0.020 |
| rs1747714 | 10 | 11161316 | T | C | 0.011 | 0.001 | 3.82E-14 | 0.004 | 0.002 | 7.60E-02 | Education | -0.010 | 0.006 | -0.013 | 0.008 | -0.012 | 0.018 | -0.012 | 0.015 | -0.041 | 0.026 | 0.000 | 0.015 |
| rs2183271 | 10 | 21957229 | T | C | 0.008 | 0.002 | 1.91E-08 | -0.008 | 0.002 | 4.30E-04 | Education | -0.054 | 0.006 | -0.076 | 0.008 | -0.055 | 0.018 | -0.051 | 0.015 | -0.013 | 0.026 | 0.035 | 0.016 |
| rs11598765 | 10 | 23953725 | A | G | -0.011 | 0.002 | 1.73E-09 | -0.002 | 0.003 | 5.70E-01 | Education | -0.006 | 0.008 | 0.009 | 0.010 | -0.051 | 0.022 | 0.008 | 0.019 | -0.006 | 0.032 | -0.003 | 0.019 |
| rs806816 | 10 | 32285942 | A | T | 0.010 | 0.002 | 4.80E-10 | -0.001 | 0.003 | 5.80E-01 | Education | 0.020 | 0.007 | 0.022 | 0.009 | 0.056 | 0.021 | -0.007 | 0.017 | 0.034 | 0.030 | 0.033 | 0.018 |
| rs2007655 | 10 | 34586689 | T | G | 0.009 | 0.001 | 8.64E-10 | -0.001 | 0.002 | 7.00E-01 | Education | -0.011 | 0.006 | -0.011 | 0.008 | 0.011 | 0.018 | -0.028 | 0.015 | -0.044 | 0.026 | 0.008 | 0.015 |
| rs11003463 | 10 | 51801793 | T | G | 0.009 | 0.002 | 5.58E-10 | 0.005 | 0.002 | 4.10E-02 | Education | 0.013 | 0.007 | 0.006 | 0.009 | 0.015 | 0.020 | 0.010 | 0.017 | 0.046 | 0.029 | 0.014 | 0.017 |
| rs7924036 | 10 | 65191645 | T | G | 0.013 | 0.001 | 2.55E-21 | 0.002 | 0.002 | 3.60E-01 | Education | -0.026 | 0.006 | -0.029 | 0.008 | -0.034 | 0.018 | -0.022 | 0.015 | 0.011 | 0.026 | -0.011 | 0.015 |
| rs10996167 | 10 | 66813928 | C | G | 0.011 | 0.002 | 7.47E-09 | 0.001 | 0.002 | 7.20E-01 | Education | 0.005 | 0.006 | 0.011 | 0.008 | 0.007 | 0.019 | 0.003 | 0.016 | -0.007 | 0.027 | -0.004 | 0.016 |
| rs7920624 | 10 | 67963186 | A | T | 0.012 | 0.001 | 1.06E-17 | 0.009 | 0.002 | 7.00E-05 | Education | 0.005 | 0.006 | 0.003 | 0.008 | 0.019 | 0.018 | -0.012 | 0.015 | -0.009 | 0.026 | -0.018 | 0.015 |
| rs7910403 | 10 | 87039251 | T | G | 0.012 | 0.002 | 5.08E-12 | 0.001 | 0.003 | 7.10E-01 | Education | 0.015 | 0.008 | 0.002 | 0.010 | 0.050 | 0.022 | 0.020 | 0.019 | 0.043 | 0.032 | 0.008 | 0.020 |
| rs1426619 | 10 | 90091540 | T | C | 0.010 | 0.001 | 1.40E-11 | 0.000 | 0.002 | 8.50E-01 | Education | 0.012 | 0.006 | 0.010 | 0.008 | 0.006 | 0.018 | 0.035 | 0.015 | 0.050 | 0.026 | 0.012 | 0.015 |
| rs17882802 | 10 | 101983413 | A | G | 0.008 | 0.001 | 3.00E-09 | -0.005 | 0.002 | 2.80E-02 | Education | 0.003 | 0.006 | -0.004 | 0.008 | 0.014 | 0.018 | 0.009 | 0.015 | 0.007 | 0.026 | 0.012 | 0.015 |
| rs73344830 | 10 | 103816828 | A | G | 0.017 | 0.001 | 1.00E-32 | 0.005 | 0.002 | 1.40E-02 | Education | -0.001 | 0.006 | 0.001 | 0.008 | -0.032 | 0.018 | 0.018 | 0.016 | -0.018 | 0.026 | 0.012 | 0.016 |
| rs790647 | 10 | 106776484 | A | C | -0.015 | 0.002 | 3.81E-18 | 0.001 | 0.003 | 8.00E-01 | Education | 0.006 | 0.007 | -0.004 | 0.009 | 0.028 | 0.021 | 0.021 | 0.018 | 0.012 | 0.030 | 0.007 | 0.018 |
| rs12571549 | 10 | 111770013 | A | G | 0.015 | 0.002 | 1.45E-13 | 0.005 | 0.003 | 1.50E-01 | Education | -0.011 | 0.009 | -0.003 | 0.011 | -0.045 | 0.026 | 0.001 | 0.022 | -0.123 | 0.038 | -0.001 | 0.022 |
| rs1865955 | 10 | 118837990 | T | C | 0.012 | 0.002 | 4.06E-11 | -0.003 | 0.003 | 2.60E-01 | Education | -0.008 | 0.008 | -0.010 | 0.010 | 0.015 | 0.023 | -0.027 | 0.019 | -0.102 | 0.032 | -0.012 | 0.020 |
| rs4384309 | 10 | 133110596 | A | G | 0.010 | 0.001 | 4.89E-13 | 0.004 | 0.002 | 5.90E-02 | Education | -0.003 | 0.006 | 0.006 | 0.008 | -0.006 | 0.018 | -0.016 | 0.016 | -0.030 | 0.027 | -0.005 | 0.016 |
| rs12761761 | 10 | 133775375 | T | C | 0.015 | 0.002 | 4.79E-20 | -0.001 | 0.003 | 7.70E-01 | Education | -0.010 | 0.008 | -0.011 | 0.010 | -0.048 | 0.022 | 0.000 | 0.019 | 0.004 | 0.032 | 0.045 | 0.019 |
| rs12765185 | 10 | 134977077 | A | T | -0.009 | 0.002 | 2.96E-08 | -0.003 | 0.002 | 2.40E-01 | Education | 0.007 | 0.007 | 0.005 | 0.009 | 0.042 | 0.020 | 0.023 | 0.017 | 0.014 | 0.029 | 0.020 | 0.018 |
| rs4757957 | 11 | 12881398 | C | G | 0.013 | 0.002 | 1.79E-18 | 0.005 | 0.002 | 4.60E-02 | Education | 0.007 | 0.007 | 0.005 | 0.009 | -0.022 | 0.019 | -0.003 | 0.016 | -0.011 | 0.027 | 0.024 | 0.017 |
| rs11023764 | 11 | 15910939 | A | G | 0.009 | 0.002 | 5.32E-10 | 0.007 | 0.002 | 2.30E-03 | Education | -0.009 | 0.006 | -0.001 | 0.008 | -0.036 | 0.019 | -0.015 | 0.016 | -0.001 | 0.027 | -0.008 | 0.016 |
| rs12789313 | 11 | 25058937 | T | C | 0.009 | 0.001 | 6.79E-11 | 0.004 | 0.002 | 5.50E-02 | Education | -0.004 | 0.006 | 0.002 | 0.008 | -0.033 | 0.018 | 0.012 | 0.016 | -0.028 | 0.027 | -0.015 | 0.016 |
| rs11030102 | 11 | 27681596 | C | G | 0.010 | 0.002 | 4.60E-10 | -0.006 | 0.002 | 1.00E-02 | Education | 0.006 | 0.007 | 0.010 | 0.009 | -0.009 | 0.020 | 0.024 | 0.018 | -0.023 | 0.030 | -0.010 | 0.018 |
| rs2199409 | 11 | 39915234 | T | C | 0.010 | 0.002 | 3.14E-09 | 0.003 | 0.003 | 2.60E-01 | Education | -0.006 | 0.007 | -0.011 | 0.010 | 0.028 | 0.022 | -0.035 | 0.018 | -0.032 | 0.031 | -0.004 | 0.019 |
| rs79728014 | 11 | 46062245 | A | G | -0.013 | 0.002 | 2.06E-11 | 0.001 | 0.003 | 7.10E-01 | Education | 0.018 | 0.009 | 0.022 | 0.012 | 0.005 | 0.027 | 0.020 | 0.023 | 0.065 | 0.040 | 0.015 | 0.023 |
| rs75033012 | 11 | 61347033 | C | G | -0.026 | 0.004 | 1.38E-11 | -0.018 | 0.006 | 1.20E-03 | Education | 0.023 | 0.019 | 0.048 | 0.024 | 0.068 | 0.053 | 0.022 | 0.046 | -0.019 | 0.080 | 0.006 | 0.047 |
| rs628993 | 11 | 61539691 | A | G | 0.013 | 0.002 | 3.83E-08 | 0.001 | 0.004 | 8.00E-01 | Education | 0.017 | 0.010 | 0.020 | 0.014 | 0.028 | 0.030 | 0.024 | 0.026 | 0.037 | 0.044 | 0.018 | 0.026 |
| rs76878669 | 11 | 66092567 | C | G | 0.014 | 0.002 | 3.10E-17 | 0.000 | 0.003 | 9.30E-01 | Education | -0.024 | 0.007 | -0.020 | 0.009 | -0.004 | 0.021 | -0.022 | 0.018 | -0.042 | 0.030 | -0.045 | 0.018 |
| rs72962169 | 11 | 72365669 | T | C | -0.017 | 0.002 | 1.26E-19 | -0.003 | 0.003 | 3.00E-01 | Education | 0.022 | 0.008 | 0.028 | 0.011 | 0.023 | 0.025 | 0.022 | 0.021 | -0.026 | 0.037 | 0.035 | 0.021 |
| rs580652 | 11 | 84904742 | T | C | -0.015 | 0.002 | 4.65E-10 | 0.007 | 0.004 | 6.00E-02 | Education | -0.008 | 0.010 | -0.004 | 0.014 | -0.049 | 0.030 | 0.019 | 0.026 | 0.064 | 0.045 | 0.012 | 0.026 |
| rs10830858 | 11 | 91896638 | T | C | 0.009 | 0.001 | 5.31E-11 | 0.006 | 0.002 | 1.00E-02 | Education | -0.001 | 0.006 | -0.006 | 0.008 | 0.000 | 0.018 | 0.003 | 0.015 | 0.022 | 0.026 | -0.013 | 0.015 |
| rs488476 | 11 | 95543715 | C | G | -0.011 | 0.002 | 8.29E-15 | -0.002 | 0.002 | 4.80E-01 | Education | 0.019 | 0.006 | 0.035 | 0.008 | 0.006 | 0.018 | 0.031 | 0.015 | 0.001 | 0.026 | 0.023 | 0.016 |
| rs1301838 | 11 | 99670514 | T | C | -0.010 | 0.002 | 1.29E-10 | -0.001 | 0.002 | 7.40E-01 | Education | -0.007 | 0.007 | -0.009 | 0.009 | 0.023 | 0.019 | 0.012 | 0.016 | 0.021 | 0.028 | -0.016 | 0.017 |
| rs72486027 | 11 | 107167486 | T | C | -0.010 | 0.002 | 1.61E-10 | -0.005 | 0.003 | 4.20E-02 | Education | -0.007 | 0.007 | -0.004 | 0.009 | -0.020 | 0.020 | 0.008 | 0.017 | -0.015 | 0.030 | 0.007 | 0.018 |
| rs2734849 | 11 | 113270160 | A | G | -0.006 | 0.002 | 2.37E-04 | -0.014 | 0.002 | 8.20E-10 | PC | 0.003 | 0.006 | 0.001 | 0.008 | 0.023 | 0.018 | -0.002 | 0.015 | 0.004 | 0.025 | -0.005 | 0.015 |
| rs12574281 | 11 | 131205421 | A | C | -0.008 | 0.002 | 3.04E-08 | -0.001 | 0.002 | 6.80E-01 | Education | 0.006 | 0.007 | 0.009 | 0.009 | 0.002 | 0.019 | -0.001 | 0.016 | -0.009 | 0.028 | 0.004 | 0.017 |
| rs1448355 | 11 | 131286685 | C | T | -0.010 | 0.002 | 2.47E-09 | -0.015 | 0.002 | 1.30E-10 | PC | -0.001 | 0.006 | -0.001 | 0.008 | 0.011 | 0.018 | -0.002 | 0.015 | 0.007 | 0.026 | -0.006 | 0.016 |
| rs9633970 | 11 | 132502212 | T | C | 0.009 | 0.002 | 2.81E-08 | 0.002 | 0.003 | 3.70E-01 | Education | -0.003 | 0.008 | 0.015 | 0.010 | 0.018 | 0.022 | -0.050 | 0.019 | -0.017 | 0.032 | 0.004 | 0.019 |
| rs4766424 | 12 | 1954096 | C | G | 0.014 | 0.002 | 2.47E-11 | 0.002 | 0.003 | 4.60E-01 | Education | -0.016 | 0.009 | -0.027 | 0.012 | 0.035 | 0.026 | -0.023 | 0.023 | -0.006 | 0.039 | 0.004 | 0.023 |
| rs10772644 | 12 | 13417617 | C | G | 0.014 | 0.002 | 1.69E-10 | -0.001 | 0.004 | 7.50E-01 | Education | -0.009 | 0.010 | -0.022 | 0.013 | 0.013 | 0.030 | 0.004 | 0.025 | 0.046 | 0.044 | 0.015 | 0.026 |
| rs1861786 | 12 | 14000467 | A | G | -0.008 | 0.001 | 2.39E-08 | -0.003 | 0.002 | 2.00E-01 | Education | 0.007 | 0.006 | 0.012 | 0.008 | 0.026 | 0.018 | -0.002 | 0.016 | 0.009 | 0.027 | -0.006 | 0.016 |
| rs7974852 | 12 | 14511679 | A | C | 0.012 | 0.001 | 1.14E-16 | 0.004 | 0.002 | 6.70E-02 | Education | 0.009 | 0.006 | 0.008 | 0.008 | 0.010 | 0.018 | 0.008 | 0.015 | 0.013 | 0.026 | 0.009 | 0.015 |
| rs1603460 | 12 | 23092626 | T | G | 0.009 | 0.001 | 2.20E-10 | 0.002 | 0.002 | 4.20E-01 | Education | 0.001 | 0.006 | 0.002 | 0.008 | 0.008 | 0.018 | -0.012 | 0.015 | 0.018 | 0.026 | -0.008 | 0.015 |
| rs117398064 | 12 | 26523628 | C | G | -0.014 | 0.003 | 1.16E-08 | -0.001 | 0.004 | 7.20E-01 | Education | 0.004 | 0.011 | 0.011 | 0.015 | -0.006 | 0.034 | -0.062 | 0.029 | 0.002 | 0.048 | 0.011 | 0.028 |
| rs7972246 | 12 | 27209328 | T | C | 0.011 | 0.002 | 1.01E-13 | -0.003 | 0.002 | 2.60E-01 | Education | -0.003 | 0.006 | -0.004 | 0.008 | 0.025 | 0.018 | -0.001 | 0.016 | 0.016 | 0.027 | -0.010 | 0.016 |
| rs35933007 | 12 | 38929341 | G | A | -0.007 | 0.002 | 1.89E-03 | -0.015 | 0.003 | 9.20E-09 | PC | 0.003 | 0.008 | 0.017 | 0.011 | 0.000 | 0.024 | 0.018 | 0.020 | -0.017 | 0.034 | -0.030 | 0.021 |
| rs80257979 | 12 | 49402562 | T | G | -0.025 | 0.004 | 3.31E-10 | -0.010 | 0.007 | 1.40E-01 | Education | -0.014 | 0.017 | -0.014 | 0.022 | 0.011 | 0.049 | 0.021 | 0.042 | 0.011 | 0.071 | -0.034 | 0.041 |
| rs17110109 | 12 | 54668908 | T | C | -0.009 | 0.001 | 1.47E-10 | -0.005 | 0.002 | 4.60E-02 | Education | 0.013 | 0.006 | 0.016 | 0.008 | 0.011 | 0.018 | 0.015 | 0.016 | -0.059 | 0.026 | -0.013 | 0.016 |
| rs1689510 | 12 | 56396768 | C | G | 0.019 | 0.002 | 1.35E-38 | 0.007 | 0.002 | 1.70E-03 | Education | -0.005 | 0.006 | -0.003 | 0.008 | 0.005 | 0.019 | -0.002 | 0.016 | 0.011 | 0.027 | -0.032 | 0.016 |
| rs3751331 | 12 | 58290278 | A | G | -0.010 | 0.001 | 1.75E-11 | -0.004 | 0.002 | 1.20E-01 | Education | 0.006 | 0.006 | 0.016 | 0.008 | -0.022 | 0.018 | 0.014 | 0.015 | -0.049 | 0.027 | 0.019 | 0.016 |
| rs57661533 | 12 | 78629917 | T | C | -0.011 | 0.002 | 3.24E-08 | -0.010 | 0.003 | 1.60E-03 | Education | 0.015 | 0.009 | 0.022 | 0.012 | 0.002 | 0.027 | -0.007 | 0.023 | 0.068 | 0.038 | 0.002 | 0.023 |
| rs1245829 | 12 | 79648170 | A | T | -0.010 | 0.001 | 3.29E-12 | -0.001 | 0.002 | 5.80E-01 | Education | -0.003 | 0.007 | -0.002 | 0.009 | -0.006 | 0.019 | 0.006 | 0.016 | 0.000 | 0.028 | -0.009 | 0.017 |
| rs717996 | 12 | 84133820 | T | C | -0.012 | 0.001 | 1.89E-17 | -0.002 | 0.002 | 4.80E-01 | Education | 0.000 | 0.006 | -0.011 | 0.008 | 0.038 | 0.018 | -0.004 | 0.015 | -0.006 | 0.026 | 0.023 | 0.016 |
| rs2216144 | 12 | 97666478 | T | C | 0.010 | 0.001 | 2.58E-12 | 0.006 | 0.002 | 4.00E-03 | Education | 0.014 | 0.006 | 0.014 | 0.008 | 0.007 | 0.018 | 0.022 | 0.015 | 0.053 | 0.026 | -0.008 | 0.015 |
| rs2131167 | 12 | 99633189 | A | G | 0.008 | 0.001 | 1.52E-08 | 0.005 | 0.002 | 3.60E-02 | Education | -0.009 | 0.006 | -0.011 | 0.008 | -0.014 | 0.018 | 0.024 | 0.015 | -0.035 | 0.026 | -0.022 | 0.016 |
| rs7977614 | 12 | 110115286 | A | G | -0.013 | 0.002 | 6.84E-17 | -0.002 | 0.002 | 3.90E-01 | Education | -0.015 | 0.008 | -0.004 | 0.011 | 0.030 | 0.025 | -0.028 | 0.021 | -0.039 | 0.036 | 0.002 | 0.022 |
| rs206965 | 12 | 120856332 | T | C | 0.008 | 0.002 | 9.36E-05 | 0.016 | 0.003 | 9.10E-09 | PC | 0.010 | 0.007 | 0.008 | 0.010 | 0.014 | 0.022 | 0.032 | 0.019 | -0.008 | 0.032 | 0.014 | 0.019 |
| rs10773208 | 12 | 122970156 | T | C | -0.011 | 0.002 | 8.09E-12 | 0.002 | 0.003 | 5.00E-01 | Education | -0.013 | 0.007 | -0.007 | 0.009 | -0.025 | 0.020 | -0.036 | 0.017 | -0.066 | 0.030 | 0.007 | 0.018 |
| rs10773002 | 12 | 123746961 | A | T | 0.019 | 0.002 | 2.50E-30 | 0.000 | 0.003 | 9.80E-01 | Education | -0.005 | 0.007 | -0.002 | 0.009 | -0.008 | 0.020 | -0.027 | 0.017 | -0.029 | 0.029 | 0.004 | 0.017 |
| rs6490618 | 13 | 21338343 | T | C | 0.009 | 0.002 | 3.48E-09 | 0.008 | 0.002 | 8.20E-04 | Education | -0.010 | 0.006 | -0.007 | 0.008 | -0.005 | 0.019 | -0.011 | 0.016 | -0.030 | 0.027 | -0.032 | 0.016 |
| rs9529146 | 13 | 31785404 | T | C | 0.013 | 0.002 | 3.48E-14 | 0.007 | 0.003 | 4.50E-03 | Education | -0.011 | 0.007 | -0.014 | 0.010 | 0.001 | 0.021 | 0.003 | 0.018 | -0.044 | 0.031 | -0.046 | 0.019 |
| rs936496 | 13 | 36100541 | A | G | -0.008 | 0.001 | 4.38E-08 | -0.005 | 0.002 | 4.00E-02 | Education | -0.009 | 0.006 | -0.012 | 0.008 | 0.016 | 0.018 | -0.031 | 0.016 | 0.036 | 0.027 | 0.014 | 0.016 |
| rs12874776 | 13 | 57794345 | C | A | -0.015 | 0.003 | 1.13E-07 | -0.023 | 0.004 | 8.00E-11 | PC | 0.001 | 0.010 | 0.007 | 0.013 | 0.036 | 0.030 | 0.007 | 0.026 | -0.005 | 0.044 | 0.018 | 0.026 |
| rs7321274 | 13 | 69146186 | A | G | 0.011 | 0.002 | 8.21E-11 | 0.008 | 0.003 | 5.40E-03 | Education | -0.011 | 0.008 | -0.017 | 0.010 | -0.023 | 0.023 | -0.023 | 0.019 | -0.007 | 0.033 | 0.002 | 0.020 |
| rs2478208 | 13 | 81471113 | C | G | -0.010 | 0.001 | 2.89E-13 | -0.003 | 0.002 | 2.20E-01 | Education | 0.006 | 0.006 | 0.012 | 0.008 | 0.023 | 0.018 | 0.023 | 0.015 | 0.003 | 0.026 | -0.009 | 0.015 |
| rs7326331 | 13 | 91615838 | A | G | -0.013 | 0.002 | 3.42E-16 | -0.007 | 0.002 | 3.20E-03 | Education | 0.009 | 0.007 | 0.009 | 0.009 | 0.009 | 0.020 | 0.005 | 0.017 | 0.050 | 0.028 | 0.007 | 0.017 |
| rs11620355 | 13 | 92044587 | A | G | 0.018 | 0.003 | 1.09E-13 | 0.005 | 0.004 | 2.10E-01 | Education | -0.015 | 0.011 | -0.016 | 0.014 | -0.009 | 0.031 | -0.018 | 0.026 | -0.035 | 0.045 | -0.017 | 0.026 |
| rs9556958 | 13 | 99100046 | T | C | -0.011 | 0.001 | 5.96E-15 | -0.007 | 0.002 | 9.80E-04 | Education | -0.014 | 0.006 | -0.013 | 0.008 | -0.028 | 0.018 | 0.011 | 0.015 | -0.026 | 0.026 | 0.004 | 0.015 |
| rs11157931 | 14 | 23403193 | A | C | -0.014 | 0.001 | 3.86E-22 | -0.005 | 0.002 | 3.50E-02 | Education | 0.010 | 0.006 | 0.006 | 0.008 | 0.024 | 0.018 | 0.009 | 0.015 | 0.039 | 0.026 | 0.009 | 0.016 |
| rs178183 | 14 | 26979412 | T | C | 0.015 | 0.002 | 4.81E-19 | 0.007 | 0.003 | 3.70E-03 | Education | 0.014 | 0.008 | 0.011 | 0.010 | 0.017 | 0.022 | 0.034 | 0.019 | 0.029 | 0.032 | 0.003 | 0.019 |
| rs176218 | 14 | 29600506 | T | G | 0.020 | 0.002 | 1.10E-29 | 0.003 | 0.003 | 3.10E-01 | Education | 0.003 | 0.008 | 0.001 | 0.010 | 0.017 | 0.022 | 0.005 | 0.019 | 0.011 | 0.032 | 0.012 | 0.019 |
| rs198262 | 14 | 57283283 | C | T | 0.020 | 0.004 | 1.49E-06 | 0.030 | 0.005 | 3.90E-08 | PC | 0.002 | 0.015 | 0.011 | 0.020 | 0.041 | 0.044 | -0.020 | 0.038 | -0.019 | 0.065 | -0.010 | 0.039 |
| rs4899012 | 14 | 61003889 | C | G | -0.010 | 0.001 | 8.06E-13 | -0.007 | 0.002 | 1.50E-03 | Education | 0.002 | 0.006 | 0.007 | 0.008 | 0.007 | 0.018 | 0.018 | 0.015 | 0.007 | 0.026 | -0.010 | 0.015 |
| rs12888615 | 14 | 72435705 | T | C | 0.012 | 0.002 | 9.42E-12 | 0.000 | 0.003 | 9.00E-01 | Education | 0.012 | 0.008 | 0.014 | 0.010 | -0.026 | 0.022 | 0.015 | 0.019 | -0.006 | 0.033 | 0.010 | 0.019 |
| rs730384 | 14 | 74889870 | A | G | 0.010 | 0.001 | 9.38E-13 | -0.003 | 0.002 | 1.70E-01 | Education | 0.003 | 0.006 | -0.002 | 0.008 | 0.020 | 0.018 | 0.018 | 0.015 | 0.026 | 0.026 | 0.015 | 0.015 |
| rs2998299 | 14 | 84997363 | T | G | -0.014 | 0.002 | 1.51E-15 | -0.005 | 0.003 | 4.40E-02 | Education | 0.008 | 0.008 | 0.005 | 0.010 | 0.017 | 0.022 | -0.011 | 0.019 | 0.071 | 0.031 | 0.026 | 0.019 |
| rs4904523 | 14 | 89723630 | A | G | -0.008 | 0.001 | 6.27E-09 | -0.002 | 0.002 | 3.20E-01 | Education | 0.006 | 0.006 | -0.007 | 0.008 | 0.024 | 0.018 | 0.056 | 0.015 | -0.017 | 0.025 | 0.019 | 0.015 |
| rs736281 | 14 | 94287830 | T | C | 0.009 | 0.001 | 2.49E-09 | 0.008 | 0.002 | 8.20E-04 | Education | 0.005 | 0.006 | -0.013 | 0.009 | -0.007 | 0.019 | 0.003 | 0.016 | 0.028 | 0.027 | 0.023 | 0.016 |
| rs2496482 | 14 | 99750164 | T | C | 0.012 | 0.002 | 9.20E-17 | 0.009 | 0.002 | 1.10E-04 | Education | -0.003 | 0.007 | -0.008 | 0.009 | 0.006 | 0.019 | 0.009 | 0.017 | -0.011 | 0.028 | 0.026 | 0.017 |
| rs8008382 | 14 | 104054425 | T | C | -0.011 | 0.002 | 4.07E-12 | -0.005 | 0.002 | 4.80E-02 | Education | -0.013 | 0.007 | -0.011 | 0.009 | -0.001 | 0.019 | 0.000 | 0.016 | -0.034 | 0.028 | 0.001 | 0.017 |
| rs7167688 | 15 | 26813137 | T | C | -0.009 | 0.001 | 1.71E-11 | -0.007 | 0.002 | 1.70E-03 | Education | 0.010 | 0.006 | 0.014 | 0.008 | -0.022 | 0.018 | 0.005 | 0.015 | 0.060 | 0.025 | 0.016 | 0.015 |
| rs891793 | 15 | 27256021 | C | G | -0.010 | 0.001 | 1.87E-12 | -0.003 | 0.002 | 1.90E-01 | Education | 0.002 | 0.006 | 0.000 | 0.008 | -0.005 | 0.018 | -0.010 | 0.015 | 0.002 | 0.026 | 0.026 | 0.016 |
| rs166835 | 15 | 47716037 | C | T | 0.005 | 0.002 | 6.84E-03 | 0.013 | 0.002 | 3.40E-09 | PC | -0.013 | 0.006 | -0.023 | 0.008 | -0.038 | 0.018 | 0.002 | 0.015 | 0.001 | 0.026 | -0.015 | 0.015 |
| rs7171405 | 15 | 61466893 | A | G | -0.010 | 0.002 | 7.53E-10 | 0.000 | 0.003 | 9.70E-01 | Education | 0.001 | 0.007 | 0.001 | 0.009 | -0.012 | 0.021 | 0.015 | 0.018 | 0.029 | 0.030 | -0.032 | 0.018 |
| rs12591647 | 15 | 65959729 | T | C | -0.015 | 0.002 | 7.78E-17 | -0.009 | 0.003 | 1.70E-03 | Education | 0.016 | 0.008 | 0.004 | 0.010 | 0.071 | 0.023 | 0.034 | 0.020 | 0.007 | 0.033 | 0.014 | 0.020 |
| rs1564347 | 15 | 73438848 | T | G | 0.009 | 0.002 | 2.39E-09 | 0.004 | 0.002 | 8.20E-02 | Education | 0.003 | 0.006 | 0.001 | 0.008 | 0.015 | 0.019 | 0.022 | 0.016 | -0.003 | 0.027 | -0.012 | 0.016 |
| rs2469226 | 15 | 77246990 | A | T | 0.010 | 0.002 | 1.28E-08 | 0.006 | 0.003 | 3.50E-02 | Education | -0.008 | 0.007 | 0.001 | 0.010 | -0.047 | 0.022 | 0.012 | 0.018 | -0.015 | 0.031 | -0.003 | 0.019 |
| rs56391344 | 15 | 78006899 | A | G | 0.015 | 0.002 | 7.49E-20 | 0.002 | 0.003 | 3.40E-01 | Education | -0.004 | 0.007 | -0.008 | 0.009 | -0.011 | 0.021 | 0.037 | 0.017 | 0.043 | 0.030 | -0.027 | 0.018 |
| rs4702 | 15 | 91426560 | G | A | 0.000 | 0.002 | 9.61E-01 | 0.012 | 0.002 | 3.80E-08 | PC | 0.029 | 0.006 | 0.016 | 0.008 | 0.057 | 0.018 | 0.071 | 0.015 | -0.002 | 0.026 | 0.007 | 0.015 |
| rs9933256 | 16 | 1246748 | A | G | 0.012 | 0.001 | 6.06E-17 | 0.006 | 0.002 | 5.50E-03 | Education | 0.003 | 0.006 | -0.006 | 0.008 | 0.023 | 0.018 | 0.011 | 0.015 | 0.014 | 0.026 | 0.011 | 0.015 |
| rs11076962 | 16 | 5811367 | T | C | 0.009 | 0.002 | 4.29E-08 | 0.004 | 0.002 | 9.20E-02 | Education | 0.006 | 0.007 | 0.012 | 0.010 | -0.003 | 0.021 | -0.006 | 0.018 | -0.012 | 0.030 | -0.017 | 0.018 |
| rs34098770 | 16 | 12237812 | A | G | -0.016 | 0.002 | 2.95E-16 | -0.005 | 0.003 | 7.90E-02 | Education | -0.006 | 0.008 | 0.007 | 0.011 | 0.007 | 0.024 | -0.024 | 0.021 | -0.035 | 0.036 | -0.030 | 0.021 |
| rs9927842 | 16 | 15153717 | T | C | -0.013 | 0.002 | 6.22E-11 | 0.004 | 0.003 | 2.20E-01 | Education | -0.004 | 0.009 | 0.008 | 0.012 | -0.027 | 0.026 | -0.015 | 0.022 | 0.007 | 0.038 | -0.009 | 0.023 |
| rs6498759 | 16 | 18020295 | T | C | 0.000 | 0.002 | 8.45E-01 | 0.013 | 0.002 | 2.70E-08 | PC | 0.012 | 0.007 | 0.021 | 0.009 | 0.034 | 0.020 | -0.028 | 0.017 | 0.021 | 0.029 | 0.017 | 0.018 |
| rs4787457 | 16 | 28555400 | A | G | 0.016 | 0.002 | 1.23E-28 | 0.004 | 0.002 | 4.80E-02 | Education | -0.003 | 0.006 | 0.000 | 0.008 | -0.005 | 0.019 | -0.026 | 0.016 | 0.021 | 0.027 | 0.030 | 0.016 |
| rs9927137 | 16 | 30634300 | A | G | 0.008 | 0.001 | 5.11E-09 | 0.004 | 0.002 | 5.70E-02 | Education | 0.006 | 0.006 | -0.006 | 0.008 | -0.001 | 0.018 | 0.012 | 0.015 | 0.020 | 0.026 | 0.021 | 0.016 |
| rs2052285 | 16 | 51188432 | A | G | 0.011 | 0.001 | 7.25E-15 | 0.006 | 0.002 | 6.50E-03 | Education | -0.011 | 0.006 | -0.010 | 0.008 | -0.005 | 0.018 | -0.015 | 0.015 | 0.021 | 0.026 | -0.020 | 0.015 |
| rs3809634 | 16 | 53538157 | A | G | -0.011 | 0.002 | 6.82E-13 | -0.009 | 0.002 | 1.00E-04 | Education | -0.005 | 0.006 | -0.010 | 0.008 | -0.012 | 0.019 | 0.002 | 0.016 | 0.024 | 0.027 | -0.011 | 0.016 |
| rs17248751 | 16 | 61579618 | A | G | -0.014 | 0.002 | 1.28E-15 | -0.008 | 0.003 | 1.70E-03 | Education | 0.000 | 0.008 | 0.007 | 0.010 | 0.045 | 0.022 | -0.017 | 0.019 | -0.011 | 0.032 | -0.010 | 0.019 |
| rs3730399 | 16 | 67229019 | A | G | 0.004 | 0.003 | 2.51E-01 | 0.025 | 0.004 | 3.30E-08 | PC | -0.015 | 0.012 | 0.005 | 0.016 | -0.038 | 0.035 | 0.021 | 0.030 | -0.032 | 0.051 | -0.015 | 0.030 |
| rs9929762 | 16 | 78169675 | A | G | 0.010 | 0.001 | 1.03E-11 | 0.000 | 0.002 | 8.80E-01 | Education | -0.011 | 0.006 | -0.014 | 0.008 | -0.017 | 0.018 | 0.006 | 0.015 | -0.005 | 0.026 | -0.014 | 0.015 |
| rs61527214 | 16 | 83608599 | A | G | 0.011 | 0.001 | 1.13E-14 | 0.005 | 0.002 | 2.50E-02 | Education | -0.001 | 0.006 | -0.002 | 0.009 | -0.024 | 0.019 | -0.013 | 0.016 | -0.028 | 0.027 | -0.006 | 0.016 |
| rs74415461 | 16 | 89990843 | T | C | 0.018 | 0.003 | 6.25E-12 | 0.002 | 0.004 | 6.60E-01 | Education | 0.017 | 0.011 | 0.005 | 0.015 | 0.009 | 0.034 | 0.014 | 0.028 | 0.048 | 0.048 | 0.018 | 0.029 |
| rs2447097 | 17 | 2278064 | T | G | 0.010 | 0.001 | 1.10E-11 | 0.005 | 0.002 | 4.20E-02 | Education | 0.008 | 0.006 | 0.010 | 0.009 | 0.011 | 0.018 | 0.036 | 0.016 | 0.000 | 0.027 | 0.018 | 0.016 |
| rs17732878 | 17 | 7362359 | T | C | -0.010 | 0.002 | 4.06E-08 | -0.004 | 0.003 | 1.10E-01 | Education | 0.017 | 0.007 | 0.019 | 0.010 | 0.045 | 0.022 | 0.004 | 0.018 | 0.042 | 0.032 | -0.022 | 0.018 |
| rs7209653 | 17 | 19882084 | T | C | 0.008 | 0.002 | 4.49E-05 | 0.016 | 0.002 | 9.10E-11 | PC | -0.007 | 0.007 | 0.000 | 0.009 | -0.035 | 0.020 | -0.012 | 0.017 | 0.012 | 0.029 | -0.023 | 0.017 |
| rs11657979 | 17 | 32900837 | A | G | -0.009 | 0.002 | 4.57E-08 | -0.002 | 0.003 | 5.40E-01 | Education | 0.013 | 0.008 | 0.011 | 0.010 | 0.005 | 0.023 | 0.051 | 0.019 | 0.016 | 0.033 | 0.016 | 0.020 |
| rs1842713 | 17 | 33200385 | A | G | -0.012 | 0.002 | 9.67E-12 | -0.009 | 0.003 | 6.90E-04 | Education | -0.009 | 0.008 | -0.009 | 0.010 | -0.016 | 0.022 | -0.012 | 0.019 | -0.028 | 0.032 | 0.007 | 0.019 |
| rs12603813 | 17 | 43196584 | T | C | 0.006 | 0.002 | 4.02E-03 | 0.014 | 0.003 | 1.30E-08 | PC | 0.005 | 0.007 | 0.017 | 0.009 | 0.036 | 0.020 | -0.003 | 0.017 | 0.033 | 0.030 | -0.032 | 0.017 |
| rs68145588 | 17 | 75864805 | T | G | -0.013 | 0.002 | 7.53E-10 | -0.001 | 0.003 | 7.50E-01 | Education | 0.013 | 0.009 | 0.014 | 0.012 | -0.010 | 0.027 | 0.039 | 0.023 | -0.024 | 0.040 | -0.022 | 0.024 |
| rs66643547 | 17 | 79375315 | C | T | -0.013 | 0.002 | 2.85E-11 | -0.015 | 0.002 | 3.20E-11 | PC | 0.020 | 0.007 | 0.021 | 0.009 | 0.005 | 0.019 | 0.024 | 0.017 | 0.061 | 0.029 | 0.019 | 0.017 |
| rs12967010 | 18 | 9645035 | T | C | 0.011 | 0.002 | 1.33E-11 | 0.001 | 0.003 | 6.40E-01 | Education | -0.016 | 0.008 | -0.017 | 0.010 | -0.012 | 0.022 | -0.008 | 0.019 | -0.037 | 0.031 | -0.001 | 0.019 |
| rs8097125 | 18 | 13005473 | T | C | 0.009 | 0.001 | 2.31E-09 | 0.005 | 0.002 | 2.20E-02 | Education | -0.004 | 0.006 | 0.002 | 0.009 | 0.009 | 0.019 | -0.009 | 0.016 | 0.021 | 0.027 | 0.003 | 0.016 |
| rs303752 | 18 | 21074255 | A | G | -0.011 | 0.001 | 1.47E-14 | -0.010 | 0.002 | 4.50E-06 | Education | -0.003 | 0.006 | -0.002 | 0.008 | 0.010 | 0.019 | -0.008 | 0.016 | -0.028 | 0.027 | 0.044 | 0.016 |
| rs10460095 | 18 | 22624708 | A | G | -0.012 | 0.001 | 2.61E-18 | -0.002 | 0.002 | 4.10E-01 | Education | 0.005 | 0.006 | -0.001 | 0.008 | -0.031 | 0.019 | 0.016 | 0.016 | -0.066 | 0.027 | 0.005 | 0.016 |
| rs111852224 | 18 | 25596884 | T | C | 0.016 | 0.002 | 4.85E-13 | 0.013 | 0.003 | 1.40E-04 | Education | -0.010 | 0.009 | -0.015 | 0.012 | 0.009 | 0.028 | 0.006 | 0.024 | -0.011 | 0.041 | -0.031 | 0.024 |
| rs7226824 | 18 | 27676827 | T | C | -0.008 | 0.001 | 2.72E-08 | 0.001 | 0.002 | 8.10E-01 | Education | 0.006 | 0.006 | 0.007 | 0.008 | 0.005 | 0.017 | 0.008 | 0.015 | -0.011 | 0.025 | 0.011 | 0.015 |
| rs11082011 | 18 | 35145122 | T | C | 0.019 | 0.002 | 2.06E-38 | 0.005 | 0.002 | 4.40E-02 | Education | -0.007 | 0.007 | -0.007 | 0.009 | -0.027 | 0.019 | 0.004 | 0.017 | 0.043 | 0.028 | -0.011 | 0.017 |
| rs12957463 | 18 | 37412228 | A | G | 0.015 | 0.002 | 9.20E-17 | 0.014 | 0.003 | 7.70E-07 | Education | 0.000 | 0.008 | 0.010 | 0.010 | 0.020 | 0.022 | -0.022 | 0.019 | 0.010 | 0.032 | -0.021 | 0.019 |
| rs784256 | 18 | 53398626 | G | A | 0.015 | 0.002 | 9.15E-12 | 0.019 | 0.003 | 6.20E-11 | PC | -0.005 | 0.008 | 0.006 | 0.010 | -0.020 | 0.023 | 0.009 | 0.020 | -0.015 | 0.033 | -0.034 | 0.020 |
| rs72944064 | 18 | 63516255 | T | C | -0.010 | 0.002 | 9.39E-10 | -0.007 | 0.003 | 3.30E-03 | Education | -0.006 | 0.007 | -0.008 | 0.010 | -0.014 | 0.021 | -0.011 | 0.019 | -0.027 | 0.031 | 0.000 | 0.019 |
| rs2554835 | 18 | 74141190 | A | G | 0.009 | 0.001 | 8.95E-10 | 0.005 | 0.002 | 1.50E-02 | Education | 0.009 | 0.007 | 0.015 | 0.009 | 0.019 | 0.019 | 0.035 | 0.017 | 0.017 | 0.028 | -0.031 | 0.017 |
| rs11081529 | 18 | 75902735 | T | C | 0.013 | 0.002 | 5.22E-16 | 0.010 | 0.002 | 3.60E-05 | Education | 0.026 | 0.007 | 0.019 | 0.010 | 0.062 | 0.021 | 0.026 | 0.018 | 0.028 | 0.030 | 0.025 | 0.018 |
| rs11663602 | 18 | 77578191 | A | C | -0.013 | 0.002 | 9.27E-17 | 0.002 | 0.002 | 4.60E-01 | Education | -0.001 | 0.007 | -0.017 | 0.010 | 0.055 | 0.021 | 0.012 | 0.018 | -0.013 | 0.031 | -0.005 | 0.019 |
| rs75177132 | 19 | 4386911 | T | C | 0.030 | 0.004 | 1.01E-16 | -0.004 | 0.005 | 4.80E-01 | Education | -0.031 | 0.019 | -0.016 | 0.024 | 0.074 | 0.051 | -0.046 | 0.047 | -0.038 | 0.078 | 0.028 | 0.046 |
| rs2287838 | 19 | 9959014 | A | G | -0.010 | 0.001 | 1.27E-13 | -0.005 | 0.002 | 2.50E-02 | Education | -0.012 | 0.006 | -0.010 | 0.008 | -0.032 | 0.018 | -0.004 | 0.015 | -0.008 | 0.025 | -0.008 | 0.015 |
| rs12151248 | 19 | 13212025 | T | C | -0.015 | 0.002 | 3.81E-11 | -0.015 | 0.003 | 2.20E-05 | Education | 0.035 | 0.010 | 0.054 | 0.013 | -0.021 | 0.029 | 0.029 | 0.025 | -0.019 | 0.043 | 0.015 | 0.025 |
| rs12981405 | 19 | 19651577 | T | C | -0.011 | 0.002 | 2.75E-09 | 0.003 | 0.003 | 3.30E-01 | Education | 0.032 | 0.008 | 0.033 | 0.011 | 0.019 | 0.024 | 0.026 | 0.020 | 0.080 | 0.034 | 0.040 | 0.020 |
| rs7254263 | 19 | 30741257 | T | C | -0.012 | 0.002 | 1.84E-14 | -0.005 | 0.002 | 3.70E-02 | Education | 0.003 | 0.007 | 0.004 | 0.010 | -0.016 | 0.021 | -0.020 | 0.018 | 0.031 | 0.030 | -0.016 | 0.018 |
| rs3859523 | 19 | 32972296 | T | C | 0.012 | 0.002 | 9.92E-12 | 0.008 | 0.003 | 6.50E-03 | Education | -0.005 | 0.008 | -0.007 | 0.011 | -0.040 | 0.024 | -0.022 | 0.021 | 0.013 | 0.036 | 0.022 | 0.021 |
| rs173003 | 19 | 36240960 | A | C | -0.008 | 0.001 | 5.50E-09 | -0.009 | 0.002 | 1.80E-05 | Education | 0.011 | 0.006 | 0.020 | 0.008 | 0.007 | 0.018 | 0.000 | 0.015 | 0.008 | 0.026 | -0.013 | 0.015 |
| rs76246107 | 19 | 50121274 | A | G | -0.016 | 0.003 | 6.52E-10 | 0.004 | 0.004 | 3.80E-01 | Education | 0.000 | 0.012 | 0.010 | 0.016 | -0.022 | 0.036 | -0.046 | 0.031 | -0.010 | 0.053 | 0.004 | 0.031 |
| rs192436652 | 19 | 54960747 | T | C | -0.032 | 0.005 | 2.06E-12 | 0.004 | 0.007 | 5.10E-01 | Education | 0.017 | 0.021 | 0.014 | 0.027 | -0.022 | 0.060 | -0.031 | 0.052 | 0.043 | 0.085 | 0.072 | 0.051 |
| rs6043521 | 20 | 15739745 | T | C | -0.008 | 0.001 | 9.72E-09 | 0.009 | 0.002 | 3.80E-05 | Education | 0.008 | 0.006 | 0.015 | 0.009 | 0.008 | 0.019 | 0.017 | 0.016 | 0.029 | 0.028 | -0.010 | 0.016 |
| rs175325 | 20 | 22317310 | A | T | -0.010 | 0.001 | 2.24E-12 | -0.006 | 0.002 | 1.30E-02 | Education | -0.012 | 0.006 | -0.012 | 0.008 | -0.022 | 0.018 | -0.011 | 0.015 | -0.045 | 0.026 | -0.013 | 0.016 |
| rs4369924 | 20 | 41954383 | A | G | 0.013 | 0.002 | 5.58E-11 | 0.005 | 0.003 | 1.00E-01 | Education | 0.009 | 0.008 | 0.006 | 0.011 | 0.045 | 0.024 | -0.007 | 0.021 | 0.007 | 0.035 | 0.019 | 0.021 |
| rs6065784 | 20 | 43717435 | C | G | 0.011 | 0.002 | 5.78E-13 | 0.001 | 0.002 | 7.60E-01 | Education | -0.002 | 0.006 | -0.007 | 0.009 | 0.034 | 0.019 | -0.020 | 0.016 | 0.017 | 0.027 | -0.008 | 0.016 |
| rs4810894 | 20 | 47532999 | A | G | -0.009 | 0.002 | 4.38E-09 | 0.001 | 0.002 | 7.60E-01 | Education | 0.002 | 0.006 | -0.002 | 0.008 | 0.023 | 0.019 | -0.045 | 0.016 | -0.021 | 0.027 | 0.018 | 0.016 |
| rs6123924 | 20 | 58219764 | A | G | 0.013 | 0.002 | 3.20E-11 | 0.010 | 0.003 | 1.10E-03 | Education | 0.025 | 0.009 | 0.012 | 0.011 | 0.032 | 0.025 | 0.003 | 0.021 | 0.033 | 0.037 | 0.023 | 0.022 |
| rs6129084 | 20 | 59828857 | A | T | -0.012 | 0.002 | 3.05E-12 | -0.014 | 0.002 | 9.30E-10 | PC | -0.001 | 0.006 | 0.005 | 0.008 | -0.004 | 0.019 | -0.010 | 0.016 | 0.015 | 0.027 | -0.001 | 0.016 |
| rs13050131 | 21 | 20018118 | A | C | -0.008 | 0.002 | 4.40E-08 | -0.007 | 0.002 | 3.10E-03 | Education | -0.006 | 0.006 | -0.001 | 0.008 | -0.012 | 0.019 | -0.019 | 0.016 | -0.018 | 0.027 | -0.005 | 0.016 |
| rs232496 | 21 | 22734409 | T | C | 0.009 | 0.002 | 7.14E-10 | -0.001 | 0.002 | 6.00E-01 | Education | -0.010 | 0.006 | -0.002 | 0.008 | -0.033 | 0.018 | 0.002 | 0.015 | 0.005 | 0.026 | -0.018 | 0.016 |
| rs1544 | 21 | 33085585 | A | G | -0.010 | 0.002 | 1.76E-09 | 0.005 | 0.002 | 5.00E-02 | Education | -0.007 | 0.007 | -0.009 | 0.009 | 0.007 | 0.021 | -0.029 | 0.018 | -0.059 | 0.030 | 0.037 | 0.018 |
| rs743316 | 21 | 35252696 | T | C | 0.011 | 0.002 | 8.21E-11 | 0.000 | 0.003 | 9.00E-01 | Education | 0.003 | 0.008 | -0.002 | 0.010 | -0.003 | 0.022 | 0.011 | 0.019 | 0.021 | 0.032 | 0.004 | 0.019 |
| rs2838006 | 21 | 42653567 | T | C | 0.013 | 0.002 | 1.38E-19 | 0.001 | 0.002 | 6.10E-01 | Education | -0.001 | 0.006 | -0.003 | 0.008 | -0.013 | 0.018 | 0.016 | 0.016 | 0.034 | 0.026 | 0.006 | 0.016 |
| rs2297293 | 21 | 47125046 | C | G | 0.010 | 0.002 | 8.80E-11 | 0.002 | 0.002 | 3.20E-01 | Education | 0.006 | 0.007 | 0.007 | 0.009 | 0.022 | 0.019 | -0.003 | 0.016 | 0.017 | 0.028 | -0.012 | 0.017 |
| rs136553 | 22 | 27255675 | C | T | -0.005 | 0.002 | 2.43E-03 | -0.015 | 0.002 | 2.30E-11 | PC | -0.004 | 0.006 | -0.001 | 0.008 | 0.000 | 0.018 | -0.005 | 0.015 | 0.014 | 0.027 | 0.002 | 0.016 |
| rs143743568 | 22 | 39289014 | A | G | 0.012 | 0.002 | 4.99E-09 | -0.004 | 0.003 | 2.00E-01 | Education | 0.031 | 0.009 | 0.044 | 0.011 | 0.023 | 0.025 | 0.004 | 0.022 | -0.042 | 0.037 | 0.048 | 0.022 |
| rs7288455 | 22 | 39966547 | A | G | 0.010 | 0.002 | 1.85E-09 | 0.013 | 0.002 | 4.00E-09 | PC | 0.001 | 0.006 | 0.005 | 0.008 | -0.018 | 0.019 | 0.004 | 0.016 | -0.025 | 0.027 | -0.007 | 0.016 |
| rs137079 | 22 | 42977907 | T | C | 0.013 | 0.002 | 3.06E-10 | 0.001 | 0.003 | 7.00E-01 | Education | -0.007 | 0.009 | -0.015 | 0.012 | -0.006 | 0.026 | 0.028 | 0.022 | -0.016 | 0.038 | -0.005 | 0.023 |
| rs9616947 | 22 | 51151631 | T | C | -0.013 | 0.002 | 3.58E-20 | -0.003 | 0.002 | 1.70E-01 | Education | -0.004 | 0.007 | -0.005 | 0.009 | 0.033 | 0.020 | -0.024 | 0.017 | -0.009 | 0.029 | -0.040 | 0.017 |

| **Table S19. Beta estimates for the SNPs included in the multivariable MR of years of education, leisure computer use, and colorectal cancer risk** | | | | | | | | | | | | | | | | | | | | | |
| --- | --- | --- | --- | --- | --- | --- | --- | --- | --- | --- | --- | --- | --- | --- | --- | --- | --- | --- | --- | --- | --- |
| SNP | CHR | POS | EA | OA | beta_ed | se_ed | pval_ed | beta_pc | se_pc | pval | Phenotype | beta_crc | se_crc | beta_crc  _men | se_crc  _men | beta_crc  _women | se_crc  _women | beta_co | se_co | beta_re | se_re |
| rs2748985 | 1 | 1853184 | T | C | -0.006 | 0.002 | 4.16E-04 | -0.015 | 0.002 | 4.10E-12 | PC | 0.006 | 0.010 | 0.017 | 0.014 | -0.003 | 0.014 | 0.016 | 0.012 | -0.003 | 0.015 |
| rs11121177 | 1 | 8445946 | A | G | 0.015 | 0.002 | 1.44E-16 | 0.007 | 0.003 | 1.20E-02 | Education | 0.010 | 0.012 | 0.001 | 0.018 | 0.016 | 0.018 | -0.005 | 0.015 | 0.015 | 0.019 |
| rs10799615 | 1 | 20541370 | A | G | -0.009 | 0.002 | 4.11E-08 | 0.001 | 0.003 | 7.30E-01 | Education | 0.001 | 0.011 | 0.006 | 0.016 | -0.002 | 0.016 | 0.008 | 0.013 | -0.001 | 0.017 |
| rs590013 | 1 | 29155738 | T | C | 0.010 | 0.002 | 2.47E-11 | 0.004 | 0.002 | 8.70E-02 | Education | 0.008 | 0.010 | 0.010 | 0.015 | 0.004 | 0.014 | 0.007 | 0.012 | 0.003 | 0.016 |
| rs10798888 | 1 | 32199099 | T | G | -0.014 | 0.002 | 5.15E-14 | -0.008 | 0.003 | 7.30E-03 | Education | -0.010 | 0.013 | -0.022 | 0.018 | 0.001 | 0.018 | -0.014 | 0.015 | -0.020 | 0.020 |
| rs2364544 | 1 | 41833162 | A | G | -0.011 | 0.001 | 2.91E-13 | -0.006 | 0.002 | 8.00E-03 | Education | 0.009 | 0.010 | 0.027 | 0.014 | -0.007 | 0.014 | 0.007 | 0.012 | 0.023 | 0.016 |
| rs12076635 | 1 | 44026656 | C | G | 0.021 | 0.002 | 3.33E-34 | -0.002 | 0.003 | 3.70E-01 | Education | -0.007 | 0.011 | -0.007 | 0.016 | -0.006 | 0.016 | -0.001 | 0.014 | -0.010 | 0.018 |
| rs1738050 | 1 | 44707295 | C | G | -0.009 | 0.001 | 1.42E-10 | 0.000 | 0.002 | 9.10E-01 | Education | 0.026 | 0.010 | 0.038 | 0.014 | 0.016 | 0.014 | 0.023 | 0.012 | 0.017 | 0.016 |
| rs11211123 | 1 | 45957609 | A | G | 0.010 | 0.002 | 7.62E-10 | 0.003 | 0.003 | 2.00E-01 | Education | 0.008 | 0.011 | 0.012 | 0.016 | 0.000 | 0.016 | 0.008 | 0.014 | -0.003 | 0.018 |
| rs2764684 | 1 | 58537919 | T | C | 0.014 | 0.002 | 9.99E-15 | 0.004 | 0.003 | 2.10E-01 | Education | -0.008 | 0.013 | -0.008 | 0.019 | -0.009 | 0.019 | -0.010 | 0.016 | -0.002 | 0.021 |
| rs2989476 | 1 | 61059259 | C | G | 0.008 | 0.001 | 3.73E-08 | -0.001 | 0.002 | 6.10E-01 | Education | -0.005 | 0.010 | -0.019 | 0.014 | 0.006 | 0.014 | -0.005 | 0.012 | -0.010 | 0.015 |
| rs4915735 | 1 | 61808444 | A | G | 0.012 | 0.002 | 9.56E-09 | 0.004 | 0.003 | 1.90E-01 | Education | -0.041 | 0.014 | -0.025 | 0.020 | -0.060 | 0.020 | -0.042 | 0.017 | -0.069 | 0.022 |
| rs34305371 | 1 | 72733610 | A | G | 0.031 | 0.002 | 1.10E-39 | 0.015 | 0.004 | 3.60E-05 | Education | -0.008 | 0.019 | 0.014 | 0.026 | -0.028 | 0.026 | -0.011 | 0.022 | 0.002 | 0.029 |
| rs79994730 | 1 | 75562222 | T | C | 0.024 | 0.004 | 5.88E-12 | 0.000 | 0.005 | 9.80E-01 | Education | -0.019 | 0.028 | -0.024 | 0.040 | -0.010 | 0.039 | -0.025 | 0.032 | 0.013 | 0.042 |
| rs1008078 | 1 | 91189731 | T | C | -0.018 | 0.001 | 4.50E-37 | -0.003 | 0.002 | 1.40E-01 | Education | 0.004 | 0.010 | 0.010 | 0.014 | -0.003 | 0.014 | 0.003 | 0.012 | -0.019 | 0.015 |
| rs6690195 | 1 | 96221653 | T | C | -0.011 | 0.001 | 7.89E-16 | -0.007 | 0.002 | 2.30E-03 | Education | -0.017 | 0.010 | -0.006 | 0.014 | -0.025 | 0.014 | -0.024 | 0.012 | -0.006 | 0.016 |
| rs10875121 | 1 | 98417446 | C | G | 0.017 | 0.002 | 4.46E-20 | 0.004 | 0.003 | 2.00E-01 | Education | 0.016 | 0.013 | 0.009 | 0.019 | 0.020 | 0.019 | 0.012 | 0.016 | 0.019 | 0.021 |
| rs12145677 | 1 | 110023610 | G | A | -0.012 | 0.002 | 1.32E-11 | -0.017 | 0.002 | 1.20E-12 | PC | -0.006 | 0.011 | 0.012 | 0.015 | -0.023 | 0.015 | -0.007 | 0.013 | -0.004 | 0.017 |
| rs4839155 | 1 | 112161489 | T | G | 0.012 | 0.002 | 4.66E-12 | 0.007 | 0.003 | 9.20E-03 | Education | -0.021 | 0.011 | -0.023 | 0.016 | -0.021 | 0.016 | -0.016 | 0.014 | -0.033 | 0.018 |
| rs3026996 | 1 | 159167290 | A | C | 0.014 | 0.002 | 5.42E-17 | 0.011 | 0.003 | 8.20E-06 | Education | 0.004 | 0.012 | -0.007 | 0.017 | 0.013 | 0.017 | -0.011 | 0.014 | 0.012 | 0.018 |
| rs34720381 | 1 | 171455322 | T | C | -0.018 | 0.002 | 1.90E-13 | 0.006 | 0.004 | 1.20E-01 | Education | 0.036 | 0.017 | 0.008 | 0.024 | 0.063 | 0.025 | 0.035 | 0.021 | 0.044 | 0.027 |
| rs6697584 | 1 | 181509718 | T | C | -0.013 | 0.002 | 1.43E-13 | -0.001 | 0.003 | 6.60E-01 | Education | 0.012 | 0.012 | 0.023 | 0.017 | -0.001 | 0.017 | 0.012 | 0.015 | 0.004 | 0.019 |
| rs7526456 | 1 | 197773734 | T | C | -0.003 | 0.002 | 8.28E-02 | -0.013 | 0.002 | 1.20E-08 | PC | -0.005 | 0.010 | -0.001 | 0.014 | -0.009 | 0.014 | -0.004 | 0.012 | -0.012 | 0.015 |
| rs1890132 | 1 | 199427321 | T | C | -0.009 | 0.002 | 6.56E-09 | 0.000 | 0.002 | 9.00E-01 | Education | 0.002 | 0.011 | 0.011 | 0.015 | -0.008 | 0.015 | -0.007 | 0.013 | 0.014 | 0.017 |
| rs8024 | 1 | 201845575 | A | C | -0.012 | 0.002 | 1.13E-14 | -0.006 | 0.002 | 1.40E-02 | Education | 0.026 | 0.010 | 0.029 | 0.015 | 0.025 | 0.015 | 0.033 | 0.013 | 0.005 | 0.016 |
| rs3111251 | 1 | 211409844 | T | C | -0.009 | 0.001 | 3.88E-10 | -0.002 | 0.002 | 3.60E-01 | Education | -0.002 | 0.010 | 0.004 | 0.014 | -0.007 | 0.014 | 0.003 | 0.012 | 0.008 | 0.015 |
| rs4846724 | 1 | 221967817 | A | G | 0.010 | 0.001 | 2.89E-12 | 0.005 | 0.002 | 3.20E-02 | Education | 0.007 | 0.010 | 0.002 | 0.014 | 0.013 | 0.014 | 0.000 | 0.012 | 0.005 | 0.015 |
| rs1329125 | 1 | 234740880 | T | C | -0.010 | 0.002 | 8.37E-11 | -0.003 | 0.002 | 2.10E-01 | Education | 0.028 | 0.011 | 0.035 | 0.015 | 0.019 | 0.015 | 0.028 | 0.013 | 0.042 | 0.016 |
| rs114593137 | 1 | 241876565 | A | T | -0.012 | 0.002 | 1.65E-12 | -0.011 | 0.003 | 6.00E-05 | Education | 0.004 | 0.012 | 0.001 | 0.017 | 0.011 | 0.017 | 0.007 | 0.014 | -0.006 | 0.019 |
| rs3897821 | 1 | 243420388 | A | G | 0.015 | 0.002 | 3.58E-25 | 0.009 | 0.002 | 1.20E-04 | Education | -0.011 | 0.010 | -0.026 | 0.014 | 0.005 | 0.014 | -0.009 | 0.012 | -0.018 | 0.016 |
| rs12614263 | 2 | 4123797 | A | G | 0.008 | 0.001 | 3.38E-09 | 0.002 | 0.002 | 2.60E-01 | Education | -0.002 | 0.010 | -0.010 | 0.014 | 0.005 | 0.014 | -0.005 | 0.012 | -0.010 | 0.015 |
| rs7603132 | 2 | 4951548 | A | G | 0.014 | 0.002 | 1.20E-14 | 0.005 | 0.003 | 9.20E-02 | Education | -0.019 | 0.012 | -0.024 | 0.017 | -0.013 | 0.017 | -0.026 | 0.015 | -0.017 | 0.019 |
| rs76076331 | 2 | 10977585 | T | C | 0.021 | 0.002 | 1.71E-23 | 0.003 | 0.003 | 4.00E-01 | Education | -0.005 | 0.015 | -0.022 | 0.021 | 0.010 | 0.021 | -0.006 | 0.018 | 0.003 | 0.023 |
| rs1991585 | 2 | 16647170 | T | C | -0.010 | 0.002 | 6.26E-11 | -0.002 | 0.002 | 3.40E-01 | Education | 0.011 | 0.010 | -0.013 | 0.015 | 0.033 | 0.015 | 0.014 | 0.013 | 0.018 | 0.017 |
| rs4358081 | 2 | 29100642 | A | C | -0.010 | 0.001 | 1.28E-11 | -0.004 | 0.002 | 1.10E-01 | Education | 0.020 | 0.010 | 0.024 | 0.014 | 0.015 | 0.014 | 0.044 | 0.012 | 0.003 | 0.015 |
| rs12468040 | 2 | 44854981 | T | G | 0.014 | 0.001 | 3.12E-21 | -0.001 | 0.002 | 7.60E-01 | Education | -0.011 | 0.010 | -0.001 | 0.014 | -0.019 | 0.014 | -0.002 | 0.012 | -0.029 | 0.016 |
| rs17502934 | 2 | 50994255 | T | G | -0.017 | 0.002 | 2.12E-17 | -0.007 | 0.003 | 3.20E-02 | Education | 0.013 | 0.013 | 0.027 | 0.019 | 0.003 | 0.019 | 0.025 | 0.016 | 0.009 | 0.021 |
| rs3948495 | 2 | 57383133 | T | G | -0.009 | 0.001 | 3.95E-10 | -0.003 | 0.002 | 1.50E-01 | Education | -0.018 | 0.010 | -0.014 | 0.014 | -0.024 | 0.014 | -0.013 | 0.012 | -0.036 | 0.015 |
| rs10189857 | 2 | 60713235 | A | G | 0.016 | 0.001 | 4.69E-29 | 0.005 | 0.002 | 3.20E-02 | Education | -0.022 | 0.010 | -0.022 | 0.014 | -0.021 | 0.014 | -0.020 | 0.012 | -0.019 | 0.015 |
| rs6731373 | 2 | 68503044 | A | G | -0.012 | 0.002 | 1.39E-14 | -0.009 | 0.002 | 7.90E-05 | Education | 0.032 | 0.011 | 0.040 | 0.015 | 0.027 | 0.015 | 0.036 | 0.013 | 0.040 | 0.016 |
| rs112806496 | 2 | 80421805 | C | G | -0.016 | 0.003 | 1.72E-10 | -0.006 | 0.004 | 1.10E-01 | Education | -0.017 | 0.018 | -0.023 | 0.025 | -0.014 | 0.025 | -0.026 | 0.021 | 0.005 | 0.028 |
| rs11894424 | 2 | 80588091 | A | C | 0.021 | 0.003 | 2.14E-10 | -0.001 | 0.005 | 8.90E-01 | Education | -0.005 | 0.020 | -0.007 | 0.029 | -0.003 | 0.029 | 0.021 | 0.026 | -0.028 | 0.033 |
| rs62155350 | 2 | 98932980 | A | G | 0.034 | 0.005 | 1.69E-10 | 0.019 | 0.008 | 1.70E-02 | Education | -0.043 | 0.043 | -0.047 | 0.063 | -0.041 | 0.060 | -0.068 | 0.051 | -0.039 | 0.068 |
| rs13018640 | 2 | 100821545 | T | C | -0.022 | 0.001 | 1.05E-50 | -0.006 | 0.002 | 4.50E-03 | Education | 0.025 | 0.010 | 0.040 | 0.014 | 0.011 | 0.014 | 0.020 | 0.012 | 0.034 | 0.016 |
| rs2570497 | 2 | 104441546 | T | C | -0.012 | 0.002 | 1.27E-16 | -0.007 | 0.002 | 2.30E-03 | Education | 0.000 | 0.010 | 0.012 | 0.014 | -0.012 | 0.014 | 0.010 | 0.012 | 0.008 | 0.016 |
| rs62155873 | 2 | 105969362 | T | C | -0.015 | 0.002 | 1.65E-12 | -0.002 | 0.003 | 5.70E-01 | Education | 0.011 | 0.015 | 0.014 | 0.021 | 0.003 | 0.021 | 0.002 | 0.018 | 0.000 | 0.023 |
| rs13402497 | 2 | 115826318 | A | T | -0.008 | 0.001 | 1.33E-08 | -0.001 | 0.002 | 7.80E-01 | Education | 0.008 | 0.010 | -0.002 | 0.014 | 0.017 | 0.014 | -0.003 | 0.012 | 0.024 | 0.015 |
| rs4848924 | 2 | 124991887 | A | C | -0.012 | 0.002 | 3.56E-14 | -0.006 | 0.002 | 2.40E-02 | Education | 0.015 | 0.010 | 0.018 | 0.015 | 0.013 | 0.015 | 0.032 | 0.013 | 0.011 | 0.016 |
| rs77609760 | 2 | 142358950 | A | G | 0.024 | 0.003 | 7.71E-17 | 0.009 | 0.004 | 3.40E-02 | Education | -0.017 | 0.020 | -0.007 | 0.029 | -0.027 | 0.029 | -0.003 | 0.024 | -0.026 | 0.031 |
| rs1320139 | 2 | 144158177 | C | G | -0.015 | 0.001 | 7.62E-26 | 0.000 | 0.002 | 8.70E-01 | Education | 0.005 | 0.010 | 0.005 | 0.014 | 0.005 | 0.014 | 0.005 | 0.012 | 0.007 | 0.015 |
| rs7575637 | 2 | 145796251 | A | G | 0.011 | 0.001 | 3.43E-14 | 0.007 | 0.002 | 3.10E-03 | Education | -0.013 | 0.010 | -0.019 | 0.014 | -0.005 | 0.014 | -0.012 | 0.012 | -0.022 | 0.015 |
| rs10754920 | 2 | 146456888 | C | G | -0.002 | 0.003 | 3.57E-01 | 0.020 | 0.003 | 6.70E-09 | PC | 0.002 | 0.014 | -0.012 | 0.020 | 0.011 | 0.020 | 0.007 | 0.018 | 0.011 | 0.023 |
| rs6744254 | 2 | 155986195 | C | T | -0.001 | 0.002 | 5.06E-01 | -0.016 | 0.002 | 5.40E-13 | PC | 0.014 | 0.010 | 0.041 | 0.014 | -0.012 | 0.014 | 0.020 | 0.012 | 0.021 | 0.015 |
| rs17428076 | 2 | 172851936 | C | G | 0.014 | 0.002 | 6.41E-17 | 0.002 | 0.003 | 4.90E-01 | Education | 0.019 | 0.011 | 0.018 | 0.016 | 0.020 | 0.016 | 0.018 | 0.013 | 0.014 | 0.018 |
| rs10193498 | 2 | 174094345 | A | T | 0.010 | 0.002 | 3.10E-09 | 0.005 | 0.003 | 7.80E-02 | Education | -0.009 | 0.011 | -0.023 | 0.015 | 0.000 | 0.015 | -0.001 | 0.013 | -0.029 | 0.017 |
| rs62174974 | 2 | 185053385 | A | G | -0.011 | 0.002 | 2.76E-09 | -0.008 | 0.003 | 5.80E-03 | Education | 0.006 | 0.012 | 0.032 | 0.017 | -0.020 | 0.017 | 0.013 | 0.014 | 0.009 | 0.019 |
| rs62179650 | 2 | 189135884 | A | G | 0.011 | 0.002 | 5.66E-13 | 0.006 | 0.002 | 1.40E-02 | Education | -0.001 | 0.011 | 0.004 | 0.015 | -0.007 | 0.015 | -0.010 | 0.013 | -0.007 | 0.017 |
| rs66721975 | 2 | 191701185 | A | G | -0.009 | 0.002 | 1.51E-08 | -0.009 | 0.002 | 4.40E-04 | Education | 0.000 | 0.011 | 0.020 | 0.015 | -0.016 | 0.015 | 0.012 | 0.013 | -0.020 | 0.017 |
| rs10931821 | 2 | 199495830 | A | T | -0.014 | 0.001 | 8.65E-24 | -0.011 | 0.002 | 1.40E-06 | Education | 0.030 | 0.010 | 0.025 | 0.014 | 0.031 | 0.014 | 0.024 | 0.012 | 0.038 | 0.015 |
| rs4675248 | 2 | 202880230 | A | G | -0.009 | 0.001 | 4.50E-11 | -0.007 | 0.002 | 1.40E-03 | Education | -0.023 | 0.010 | -0.019 | 0.014 | -0.026 | 0.014 | -0.026 | 0.012 | -0.016 | 0.016 |
| rs11274218 | 2 | 215035001 | C | T | -0.010 | 0.002 | 2.29E-08 | -0.014 | 0.002 | 2.50E-09 | PC | -0.007 | 0.010 | 0.005 | 0.014 | -0.016 | 0.014 | -0.009 | 0.012 | 0.001 | 0.015 |
| rs4673840 | 2 | 215363241 | T | C | -0.014 | 0.002 | 5.86E-13 | -0.015 | 0.003 | 7.20E-07 | Education | 0.008 | 0.013 | 0.011 | 0.019 | 0.004 | 0.018 | 0.007 | 0.016 | -0.005 | 0.021 |
| rs10208088 | 2 | 221055873 | C | T | 0.003 | 0.002 | 1.13E-01 | 0.013 | 0.002 | 4.40E-09 | PC | 0.002 | 0.010 | 0.007 | 0.014 | -0.006 | 0.014 | 0.003 | 0.012 | -0.019 | 0.015 |
| rs4500930 | 2 | 228985505 | T | C | -0.011 | 0.002 | 1.67E-13 | -0.003 | 0.002 | 2.60E-01 | Education | 0.006 | 0.010 | 0.008 | 0.014 | 0.004 | 0.014 | 0.002 | 0.012 | 0.013 | 0.016 |
| rs6704768 | 2 | 233592501 | A | G | -0.012 | 0.001 | 1.84E-16 | -0.006 | 0.002 | 9.50E-03 | Education | 0.002 | 0.010 | 0.013 | 0.014 | -0.011 | 0.014 | 0.000 | 0.012 | 0.034 | 0.016 |
| rs4663617 | 2 | 236744626 | A | T | 0.010 | 0.002 | 4.12E-10 | 0.001 | 0.003 | 7.10E-01 | Education | -0.019 | 0.012 | -0.011 | 0.016 | -0.026 | 0.016 | -0.021 | 0.014 | -0.010 | 0.018 |
| rs72993796 | 2 | 240321051 | T | C | 0.013 | 0.002 | 1.12E-08 | -0.001 | 0.003 | 6.90E-01 | Education | -0.011 | 0.017 | -0.023 | 0.024 | 0.006 | 0.024 | -0.017 | 0.020 | -0.014 | 0.026 |
| rs17048855 | 3 | 8258174 | A | G | 0.011 | 0.002 | 3.10E-13 | 0.010 | 0.002 | 6.50E-06 | Education | -0.009 | 0.010 | -0.017 | 0.014 | -0.003 | 0.014 | 0.000 | 0.012 | -0.008 | 0.016 |
| rs382196 | 3 | 9145554 | T | G | -0.008 | 0.001 | 1.47E-08 | -0.008 | 0.002 | 5.70E-04 | Education | 0.001 | 0.010 | 0.008 | 0.014 | -0.007 | 0.014 | -0.005 | 0.012 | 0.010 | 0.016 |
| rs9882532 | 3 | 16865845 | T | C | 0.012 | 0.002 | 2.15E-17 | -0.003 | 0.002 | 1.80E-01 | Education | 0.000 | 0.010 | 0.002 | 0.014 | 0.002 | 0.014 | -0.005 | 0.012 | -0.023 | 0.016 |
| rs13085461 | 3 | 24950387 | C | G | 0.010 | 0.001 | 2.40E-13 | 0.002 | 0.002 | 4.30E-01 | Education | -0.004 | 0.010 | 0.008 | 0.014 | -0.011 | 0.014 | -0.004 | 0.012 | 0.000 | 0.015 |
| rs73055556 | 3 | 28027538 | A | G | 0.011 | 0.002 | 4.95E-08 | 0.006 | 0.003 | 6.80E-02 | Education | -0.029 | 0.014 | -0.036 | 0.020 | -0.021 | 0.019 | -0.025 | 0.017 | -0.028 | 0.022 |
| rs4328757 | 3 | 36938180 | T | C | 0.010 | 0.001 | 2.95E-11 | 0.001 | 0.002 | 5.90E-01 | Education | -0.013 | 0.010 | -0.014 | 0.014 | -0.011 | 0.014 | -0.011 | 0.012 | 0.003 | 0.016 |
| rs11708955 | 3 | 49540114 | T | C | -0.028 | 0.002 | 3.18E-53 | -0.016 | 0.002 | 4.20E-11 | PC | -0.001 | 0.010 | 0.007 | 0.015 | -0.006 | 0.015 | -0.007 | 0.012 | 0.029 | 0.016 |
| rs77719387 | 3 | 49917021 | A | T | -0.040 | 0.006 | 3.78E-12 | -0.008 | 0.009 | 3.90E-01 | Education | 0.015 | 0.049 | -0.013 | 0.070 | 0.028 | 0.069 | 0.032 | 0.058 | 0.055 | 0.076 |
| rs6774533 | 3 | 62471086 | C | T | -0.013 | 0.002 | 1.73E-11 | -0.015 | 0.002 | 1.20E-09 | PC | -0.004 | 0.011 | -0.008 | 0.015 | -0.001 | 0.015 | -0.009 | 0.013 | 0.009 | 0.017 |
| rs12638072 | 3 | 65654480 | A | G | -0.011 | 0.002 | 3.13E-12 | -0.004 | 0.002 | 8.60E-02 | Education | 0.012 | 0.011 | 0.019 | 0.015 | 0.008 | 0.015 | 0.020 | 0.013 | 0.001 | 0.016 |
| rs55736314 | 3 | 71586293 | C | G | -0.015 | 0.001 | 4.59E-27 | 0.001 | 0.002 | 6.90E-01 | Education | -0.021 | 0.010 | -0.015 | 0.014 | -0.026 | 0.014 | -0.031 | 0.012 | -0.014 | 0.016 |
| rs66568921 | 3 | 85672018 | T | G | -0.018 | 0.002 | 7.60E-34 | -0.004 | 0.002 | 1.10E-01 | Education | -0.017 | 0.010 | -0.014 | 0.014 | -0.018 | 0.014 | -0.025 | 0.012 | -0.025 | 0.016 |
| rs9853928 | 3 | 103295384 | T | C | -0.013 | 0.002 | 1.59E-13 | -0.003 | 0.003 | 3.00E-01 | Education | -0.026 | 0.011 | -0.034 | 0.016 | -0.018 | 0.016 | -0.030 | 0.014 | -0.003 | 0.018 |
| rs28513882 | 3 | 107296628 | A | G | -0.011 | 0.002 | 2.03E-09 | -0.005 | 0.003 | 5.50E-02 | Education | -0.002 | 0.013 | 0.022 | 0.018 | -0.021 | 0.019 | -0.008 | 0.015 | 0.006 | 0.020 |
| rs2290601 | 3 | 108112760 | T | C | -0.015 | 0.002 | 4.49E-19 | -0.003 | 0.003 | 2.40E-01 | Education | -0.002 | 0.011 | 0.001 | 0.016 | -0.006 | 0.016 | -0.004 | 0.014 | 0.013 | 0.018 |
| rs78082503 | 3 | 116556913 | G | C | -0.006 | 0.002 | 4.59E-03 | -0.015 | 0.003 | 1.10E-08 | PC | -0.007 | 0.011 | -0.020 | 0.016 | 0.004 | 0.016 | -0.002 | 0.014 | 0.033 | 0.018 |
| rs1717204 | 3 | 118461145 | A | C | -0.012 | 0.002 | 1.20E-10 | -0.003 | 0.003 | 2.20E-01 | Education | 0.003 | 0.012 | -0.008 | 0.018 | 0.015 | 0.018 | 0.006 | 0.015 | -0.003 | 0.020 |
| rs34067381 | 3 | 123594419 | T | G | -0.010 | 0.002 | 9.77E-13 | -0.004 | 0.002 | 1.30E-01 | Education | -0.019 | 0.010 | -0.024 | 0.014 | -0.014 | 0.014 | -0.016 | 0.012 | -0.015 | 0.016 |
| rs9289300 | 3 | 127144988 | T | C | -0.016 | 0.002 | 1.00E-17 | -0.005 | 0.003 | 1.00E-01 | Education | 0.005 | 0.013 | 0.026 | 0.019 | -0.016 | 0.019 | 0.004 | 0.016 | 0.013 | 0.020 |
| rs7650602 | 3 | 141147414 | T | C | -0.009 | 0.001 | 2.77E-10 | -0.010 | 0.002 | 2.90E-06 | Education | -0.004 | 0.010 | -0.011 | 0.014 | 0.002 | 0.014 | -0.004 | 0.012 | -0.005 | 0.015 |
| rs2885198 | 3 | 143636421 | A | G | 0.009 | 0.001 | 4.63E-10 | 0.001 | 0.002 | 6.30E-01 | Education | -0.020 | 0.010 | 0.001 | 0.014 | -0.037 | 0.014 | -0.019 | 0.012 | -0.026 | 0.016 |
| rs12054166 | 3 | 150093445 | C | G | 0.010 | 0.002 | 2.30E-09 | -0.001 | 0.003 | 7.70E-01 | Education | 0.013 | 0.011 | 0.021 | 0.016 | 0.007 | 0.016 | 0.005 | 0.013 | 0.031 | 0.017 |
| rs7617204 | 3 | 160821969 | A | G | -0.010 | 0.001 | 4.33E-12 | -0.001 | 0.002 | 6.00E-01 | Education | 0.002 | 0.010 | 0.005 | 0.014 | -0.001 | 0.014 | -0.006 | 0.012 | 0.021 | 0.015 |
| rs72622559 | 3 | 175672897 | T | C | -0.012 | 0.002 | 5.50E-13 | 0.000 | 0.003 | 9.70E-01 | Education | 0.002 | 0.013 | -0.006 | 0.018 | 0.010 | 0.018 | 0.002 | 0.015 | -0.006 | 0.020 |
| rs77025239 | 3 | 180734185 | A | G | -0.014 | 0.002 | 4.65E-13 | -0.011 | 0.003 | 2.40E-04 | Education | 0.012 | 0.013 | 0.018 | 0.019 | 0.007 | 0.019 | 0.019 | 0.016 | 0.010 | 0.021 |
| rs13327482 | 3 | 196788363 | A | G | -0.012 | 0.002 | 4.47E-11 | -0.003 | 0.003 | 2.60E-01 | Education | 0.013 | 0.013 | 0.008 | 0.018 | 0.007 | 0.018 | 0.012 | 0.015 | 0.015 | 0.020 |
| rs150537577 | 4 | 705629 | A | G | -0.015 | 0.003 | 9.50E-09 | -0.013 | 0.004 | 2.60E-03 | Education | -0.017 | 0.018 | -0.017 | 0.025 | -0.020 | 0.025 | -0.001 | 0.022 | -0.038 | 0.028 |
| rs363096 | 4 | 3180021 | T | C | -0.014 | 0.001 | 5.77E-24 | -0.008 | 0.002 | 1.70E-04 | Education | 0.010 | 0.010 | 0.017 | 0.014 | 0.004 | 0.014 | 0.009 | 0.012 | 0.035 | 0.015 |
| rs13133213 | 4 | 15646794 | A | G | 0.010 | 0.001 | 7.57E-12 | -0.002 | 0.002 | 4.10E-01 | Education | 0.003 | 0.010 | 0.004 | 0.013 | 0.002 | 0.014 | -0.003 | 0.011 | -0.010 | 0.015 |
| rs2011603 | 4 | 18025484 | A | G | -0.009 | 0.002 | 1.12E-08 | -0.004 | 0.002 | 8.30E-02 | Education | -0.009 | 0.011 | -0.010 | 0.016 | -0.008 | 0.016 | -0.012 | 0.013 | 0.009 | 0.018 |
| rs4467547 | 4 | 21945933 | T | G | 0.012 | 0.001 | 4.88E-17 | 0.003 | 0.002 | 2.40E-01 | Education | -0.009 | 0.010 | -0.010 | 0.014 | -0.006 | 0.014 | -0.006 | 0.012 | -0.007 | 0.016 |
| rs10021733 | 4 | 28609476 | T | C | -0.016 | 0.003 | 9.45E-10 | 0.005 | 0.003 | 7.20E-02 | Education | 0.020 | 0.014 | 0.021 | 0.019 | 0.021 | 0.019 | 0.006 | 0.016 | 0.047 | 0.021 |
| rs6857629 | 4 | 39684977 | G | A | 0.011 | 0.002 | 6.04E-08 | 0.015 | 0.003 | 1.90E-09 | PC | 0.021 | 0.011 | 0.034 | 0.016 | 0.012 | 0.016 | 0.014 | 0.013 | 0.026 | 0.017 |
| rs2055940 | 4 | 46997913 | A | G | 0.008 | 0.002 | 4.95E-08 | 0.003 | 0.002 | 2.30E-01 | Education | -0.012 | 0.010 | 0.001 | 0.015 | -0.025 | 0.015 | -0.021 | 0.012 | -0.017 | 0.016 |
| rs1505676 | 4 | 62127291 | C | G | 0.008 | 0.002 | 6.67E-09 | 0.001 | 0.002 | 7.10E-01 | Education | 0.009 | 0.010 | 0.016 | 0.014 | -0.001 | 0.014 | 0.009 | 0.012 | 0.011 | 0.016 |
| rs12506221 | 4 | 67899235 | T | G | -0.013 | 0.001 | 4.23E-20 | -0.007 | 0.002 | 2.70E-03 | Education | -0.006 | 0.010 | -0.030 | 0.014 | 0.019 | 0.014 | -0.008 | 0.012 | -0.005 | 0.015 |
| rs2034631 | 4 | 82225529 | T | C | 0.009 | 0.002 | 2.64E-09 | 0.005 | 0.002 | 2.30E-02 | Education | -0.015 | 0.010 | -0.027 | 0.014 | -0.002 | 0.014 | -0.029 | 0.012 | -0.007 | 0.016 |
| rs7692359 | 4 | 83209346 | T | C | -0.011 | 0.002 | 1.40E-10 | -0.002 | 0.003 | 3.70E-01 | Education | 0.016 | 0.012 | 0.007 | 0.017 | 0.022 | 0.017 | 0.026 | 0.014 | 0.024 | 0.018 |
| rs12503522 | 4 | 94543233 | T | C | -0.011 | 0.002 | 3.93E-13 | -0.004 | 0.002 | 1.10E-01 | Education | 0.009 | 0.011 | 0.007 | 0.015 | 0.009 | 0.015 | 0.006 | 0.013 | 0.002 | 0.017 |
| rs7683416 | 4 | 106152984 | T | C | 0.014 | 0.001 | 6.05E-23 | 0.005 | 0.002 | 2.40E-02 | Education | -0.039 | 0.010 | -0.047 | 0.014 | -0.032 | 0.014 | -0.037 | 0.012 | -0.052 | 0.015 |
| rs1955250 | 4 | 118514236 | A | C | -0.014 | 0.003 | 2.26E-08 | -0.001 | 0.004 | 7.70E-01 | Education | 0.014 | 0.017 | 0.002 | 0.024 | 0.027 | 0.023 | 0.015 | 0.020 | 0.030 | 0.026 |
| rs13145650 | 4 | 122963344 | T | C | -0.017 | 0.003 | 8.99E-12 | -0.010 | 0.004 | 9.10E-03 | Education | -0.015 | 0.018 | -0.031 | 0.025 | 0.000 | 0.025 | -0.033 | 0.022 | -0.039 | 0.028 |
| rs10006235 | 4 | 130670108 | T | C | -0.009 | 0.002 | 4.48E-09 | -0.003 | 0.002 | 1.60E-01 | Education | 0.000 | 0.011 | 0.003 | 0.015 | -0.004 | 0.015 | 0.011 | 0.013 | 0.006 | 0.017 |
| rs12643771 | 4 | 140753103 | T | C | 0.013 | 0.002 | 1.32E-17 | -0.006 | 0.002 | 1.70E-02 | Education | -0.019 | 0.011 | -0.008 | 0.015 | -0.030 | 0.015 | -0.023 | 0.013 | -0.032 | 0.017 |
| rs969512 | 4 | 147872742 | A | T | -0.011 | 0.002 | 9.84E-13 | -0.004 | 0.002 | 1.00E-01 | Education | 0.007 | 0.010 | 0.011 | 0.014 | 0.005 | 0.014 | 0.009 | 0.012 | 0.009 | 0.016 |
| rs7672622 | 4 | 157705551 | A | G | -0.009 | 0.002 | 4.72E-08 | -0.001 | 0.003 | 8.40E-01 | Education | 0.030 | 0.011 | 0.028 | 0.016 | 0.031 | 0.016 | 0.029 | 0.013 | 0.055 | 0.018 |
| rs2068625 | 4 | 159856739 | T | C | -0.014 | 0.002 | 3.08E-13 | -0.016 | 0.002 | 4.00E-11 | PC | 0.017 | 0.011 | 0.011 | 0.015 | 0.020 | 0.015 | 0.025 | 0.013 | 0.020 | 0.017 |
| rs12646523 | 4 | 170944698 | T | C | -0.013 | 0.002 | 3.79E-16 | -0.004 | 0.003 | 8.70E-02 | Education | -0.008 | 0.011 | 0.000 | 0.016 | -0.017 | 0.016 | 0.001 | 0.013 | -0.033 | 0.018 |
| rs17598675 | 4 | 176647637 | T | C | -0.012 | 0.001 | 1.37E-16 | -0.006 | 0.002 | 9.80E-03 | Education | 0.011 | 0.010 | 0.011 | 0.014 | 0.012 | 0.014 | 0.007 | 0.012 | 0.027 | 0.015 |
| rs72709560 | 4 | 178540163 | A | G | 0.009 | 0.002 | 2.03E-08 | 0.002 | 0.002 | 4.20E-01 | Education | -0.016 | 0.010 | 0.008 | 0.014 | -0.038 | 0.014 | -0.013 | 0.012 | 0.002 | 0.016 |
| rs1128956 | 4 | 183724005 | T | G | -0.014 | 0.002 | 5.34E-14 | -0.007 | 0.003 | 2.20E-02 | Education | 0.003 | 0.013 | -0.005 | 0.018 | 0.013 | 0.018 | -0.008 | 0.015 | 0.009 | 0.020 |
| rs11724690 | 4 | 186764747 | T | G | 0.009 | 0.002 | 2.08E-09 | 0.006 | 0.002 | 1.30E-02 | Education | 0.017 | 0.011 | 0.024 | 0.015 | 0.007 | 0.015 | 0.019 | 0.013 | -0.001 | 0.017 |
| rs2220599 | 5 | 7378854 | C | G | 0.001 | 0.002 | 5.35E-01 | -0.016 | 0.002 | 2.40E-12 | PC | -0.003 | 0.010 | -0.010 | 0.014 | 0.000 | 0.014 | 0.001 | 0.012 | -0.002 | 0.016 |
| rs2434672 | 5 | 11471879 | A | C | 0.009 | 0.001 | 1.06E-10 | 0.003 | 0.002 | 2.10E-01 | Education | -0.008 | 0.010 | 0.009 | 0.014 | -0.022 | 0.014 | -0.008 | 0.012 | -0.007 | 0.015 |
| rs17563464 | 5 | 26913774 | A | C | -0.015 | 0.002 | 1.74E-17 | -0.008 | 0.003 | 5.00E-03 | Education | -0.003 | 0.013 | 0.008 | 0.018 | -0.015 | 0.018 | -0.009 | 0.015 | 0.003 | 0.020 |
| rs10940921 | 5 | 30808645 | T | G | 0.011 | 0.001 | 1.31E-14 | 0.003 | 0.002 | 1.60E-01 | Education | 0.002 | 0.010 | -0.013 | 0.014 | 0.015 | 0.014 | -0.001 | 0.012 | 0.022 | 0.015 |
| rs2964199 | 5 | 57532775 | T | C | -0.010 | 0.002 | 4.61E-11 | -0.003 | 0.002 | 2.80E-01 | Education | -0.008 | 0.011 | 0.005 | 0.015 | -0.020 | 0.015 | -0.010 | 0.013 | 0.005 | 0.017 |
| rs6449503 | 5 | 60095272 | A | G | 0.019 | 0.001 | 1.84E-39 | 0.009 | 0.002 | 6.20E-05 | Education | -0.001 | 0.010 | 0.002 | 0.014 | -0.004 | 0.014 | -0.002 | 0.012 | 0.000 | 0.015 |
| rs10805383 | 5 | 63034606 | A | G | -0.010 | 0.001 | 2.22E-13 | 0.002 | 0.002 | 3.90E-01 | Education | -0.025 | 0.010 | -0.018 | 0.014 | -0.033 | 0.014 | -0.022 | 0.012 | -0.018 | 0.015 |
| rs42302 | 5 | 74965554 | A | G | 0.009 | 0.002 | 5.96E-09 | 0.006 | 0.002 | 1.10E-02 | Education | -0.005 | 0.010 | -0.013 | 0.014 | 0.006 | 0.014 | -0.002 | 0.012 | 0.011 | 0.016 |
| rs11749912 | 5 | 88065628 | A | G | 0.018 | 0.002 | 1.84E-25 | 0.014 | 0.002 | 4.90E-10 | PC | 0.015 | 0.010 | 0.010 | 0.014 | 0.023 | 0.014 | 0.019 | 0.012 | -0.001 | 0.015 |
| rs12332731 | 5 | 93033082 | A | T | 0.014 | 0.002 | 5.96E-14 | 0.003 | 0.003 | 3.60E-01 | Education | 0.022 | 0.012 | 0.040 | 0.018 | 0.006 | 0.018 | 0.025 | 0.015 | 0.016 | 0.019 |
| rs252991 | 5 | 106767346 | A | G | 0.010 | 0.002 | 6.30E-11 | 0.004 | 0.002 | 5.90E-02 | Education | -0.009 | 0.010 | -0.016 | 0.014 | -0.003 | 0.014 | -0.020 | 0.012 | 0.001 | 0.016 |
| rs1469249 | 5 | 113837198 | G | A | 0.017 | 0.002 | 1.55E-15 | 0.016 | 0.003 | 9.50E-09 | PC | 0.009 | 0.012 | 0.017 | 0.017 | 0.005 | 0.017 | -0.001 | 0.014 | 0.038 | 0.018 |
| rs62379838 | 5 | 120102028 | T | C | 0.012 | 0.002 | 1.77E-15 | -0.002 | 0.002 | 3.40E-01 | Education | -0.007 | 0.011 | -0.002 | 0.015 | -0.013 | 0.015 | -0.005 | 0.013 | -0.008 | 0.017 |
| rs162894 | 5 | 131611872 | T | G | 0.005 | 0.002 | 3.26E-03 | 0.013 | 0.002 | 1.10E-08 | PC | -0.012 | 0.010 | -0.017 | 0.014 | -0.007 | 0.014 | -0.018 | 0.012 | -0.012 | 0.016 |
| rs56158102 | 5 | 144027803 | C | T | 0.005 | 0.002 | 4.09E-03 | 0.013 | 0.002 | 3.10E-08 | PC | -0.006 | 0.010 | 0.003 | 0.014 | -0.014 | 0.014 | -0.001 | 0.012 | -0.001 | 0.016 |
| rs2964255 | 5 | 152052332 | A | G | 0.009 | 0.002 | 6.34E-09 | -0.006 | 0.002 | 1.60E-02 | Education | -0.010 | 0.010 | -0.012 | 0.015 | -0.006 | 0.015 | 0.002 | 0.013 | -0.047 | 0.016 |
| rs42210 | 5 | 166408788 | C | G | -0.010 | 0.002 | 2.74E-10 | 0.002 | 0.002 | 3.50E-01 | Education | 0.007 | 0.011 | 0.016 | 0.015 | -0.004 | 0.015 | -0.004 | 0.013 | 0.024 | 0.017 |
| rs2545795 | 5 | 176887106 | A | C | 0.013 | 0.001 | 1.03E-18 | 0.008 | 0.002 | 3.30E-04 | Education | -0.016 | 0.010 | -0.013 | 0.014 | -0.015 | 0.014 | -0.007 | 0.012 | -0.014 | 0.015 |
| rs12524795 | 6 | 3464074 | T | C | 0.010 | 0.001 | 5.28E-13 | 0.006 | 0.002 | 1.20E-02 | Education | -0.001 | 0.010 | -0.008 | 0.014 | 0.007 | 0.014 | 0.007 | 0.012 | -0.018 | 0.015 |
| rs112603734 | 6 | 6069588 | A | C | -0.013 | 0.002 | 2.72E-09 | -0.004 | 0.003 | 1.30E-01 | Education | -0.025 | 0.012 | -0.041 | 0.016 | -0.013 | 0.016 | -0.015 | 0.014 | -0.038 | 0.018 |
| rs72829857 | 6 | 16966052 | A | G | -0.015 | 0.002 | 1.52E-19 | -0.002 | 0.003 | 4.80E-01 | Education | -0.013 | 0.011 | -0.017 | 0.016 | -0.006 | 0.016 | -0.010 | 0.013 | -0.012 | 0.017 |
| rs9477970 | 6 | 19082663 | T | A | -0.017 | 0.002 | 3.16E-14 | -0.016 | 0.003 | 1.00E-08 | PC | -0.017 | 0.012 | -0.032 | 0.018 | -0.004 | 0.018 | -0.016 | 0.015 | -0.005 | 0.020 |
| rs2179152 | 6 | 26325888 | T | C | -0.013 | 0.002 | 1.68E-19 | -0.004 | 0.002 | 5.20E-02 | Education | 0.005 | 0.010 | 0.013 | 0.014 | -0.006 | 0.014 | 0.011 | 0.012 | 0.001 | 0.016 |
| rs2256965 | 6 | 31555130 | A | G | 0.011 | 0.001 | 2.40E-13 | 0.005 | 0.002 | 2.70E-02 | Education | -0.043 | 0.010 | -0.057 | 0.014 | -0.029 | 0.014 | -0.049 | 0.012 | -0.051 | 0.015 |
| rs57349798 | 6 | 37486052 | A | G | 0.009 | 0.001 | 8.63E-11 | -0.003 | 0.002 | 2.60E-01 | Education | -0.002 | 0.010 | 0.013 | 0.014 | -0.016 | 0.014 | -0.011 | 0.012 | -0.007 | 0.016 |
| rs2436760 | 6 | 40374288 | T | C | 0.023 | 0.004 | 4.52E-08 | 0.007 | 0.006 | 3.10E-01 | Education | -0.008 | 0.030 | -0.064 | 0.044 | 0.038 | 0.043 | 0.005 | 0.037 | -0.029 | 0.049 |
| rs912883 | 6 | 41552040 | T | C | 0.009 | 0.002 | 6.20E-09 | 0.001 | 0.002 | 7.90E-01 | Education | 0.015 | 0.010 | 0.022 | 0.014 | 0.007 | 0.014 | 0.011 | 0.012 | 0.000 | 0.016 |
| rs78648104 | 6 | 50683009 | T | C | -0.014 | 0.003 | 1.70E-08 | 0.008 | 0.004 | 3.60E-02 | Education | 0.000 | 0.017 | 0.004 | 0.023 | 0.000 | 0.024 | 0.011 | 0.020 | -0.008 | 0.027 |
| rs7449561 | 6 | 66215549 | A | G | 0.010 | 0.002 | 9.73E-10 | 0.010 | 0.003 | 2.40E-04 | Education | 0.007 | 0.011 | 0.013 | 0.016 | 0.002 | 0.016 | 0.000 | 0.014 | 0.022 | 0.018 |
| rs13212041 | 6 | 78171124 | T | C | 0.010 | 0.002 | 7.42E-09 | 0.005 | 0.003 | 4.60E-02 | Education | 0.022 | 0.012 | 0.012 | 0.017 | 0.032 | 0.017 | 0.025 | 0.015 | 0.013 | 0.019 |
| rs4352658 | 6 | 88279872 | T | C | -0.019 | 0.003 | 1.71E-13 | -0.007 | 0.004 | 9.10E-02 | Education | 0.024 | 0.018 | -0.013 | 0.025 | 0.056 | 0.025 | 0.015 | 0.022 | 0.034 | 0.028 |
| rs9372625 | 6 | 98344031 | G | A | -0.024 | 0.002 | 6.76E-42 | -0.018 | 0.002 | 5.70E-16 | PC | 0.031 | 0.010 | 0.033 | 0.014 | 0.030 | 0.014 | 0.026 | 0.012 | 0.041 | 0.016 |
| rs10080647 | 6 | 114742335 | A | C | 0.012 | 0.002 | 7.05E-10 | 0.001 | 0.003 | 7.40E-01 | Education | -0.011 | 0.014 | 0.008 | 0.019 | -0.028 | 0.019 | -0.016 | 0.016 | -0.031 | 0.022 |
| rs11542663 | 6 | 119215402 | A | C | 0.011 | 0.002 | 2.44E-13 | -0.001 | 0.002 | 7.60E-01 | Education | -0.006 | 0.010 | 0.007 | 0.015 | -0.017 | 0.015 | -0.005 | 0.013 | 0.006 | 0.016 |
| rs13197257 | 6 | 128333682 | T | G | 0.011 | 0.002 | 2.83E-12 | 0.003 | 0.002 | 2.10E-01 | Education | 0.000 | 0.011 | -0.014 | 0.016 | 0.013 | 0.016 | -0.005 | 0.013 | 0.016 | 0.017 |
| rs6935828 | 6 | 140811367 | C | T | -0.005 | 0.002 | 6.57E-03 | -0.012 | 0.002 | 4.30E-08 | PC | -0.002 | 0.010 | 0.005 | 0.014 | -0.004 | 0.014 | 0.002 | 0.012 | 0.022 | 0.015 |
| rs4895650 | 6 | 145024122 | T | C | 0.009 | 0.001 | 5.31E-11 | 0.001 | 0.002 | 8.10E-01 | Education | -0.013 | 0.010 | -0.011 | 0.014 | -0.013 | 0.014 | -0.020 | 0.012 | -0.015 | 0.016 |
| rs6557171 | 6 | 152234593 | T | C | -0.016 | 0.002 | 5.48E-25 | -0.006 | 0.002 | 6.10E-03 | Education | 0.004 | 0.010 | 0.012 | 0.015 | -0.006 | 0.015 | 0.005 | 0.013 | 0.002 | 0.016 |
| rs1933264 | 6 | 153391618 | T | C | 0.010 | 0.002 | 5.85E-10 | -0.002 | 0.003 | 3.40E-01 | Education | -0.017 | 0.011 | -0.021 | 0.015 | -0.009 | 0.016 | -0.004 | 0.013 | -0.023 | 0.017 |
| rs6917154 | 6 | 167135156 | T | C | 0.013 | 0.002 | 2.55E-09 | -0.004 | 0.003 | 2.80E-01 | Education | -0.022 | 0.015 | -0.041 | 0.022 | 0.002 | 0.022 | -0.002 | 0.018 | -0.037 | 0.024 |
| rs6924023 | 6 | 170076041 | A | G | 0.011 | 0.002 | 5.17E-11 | 0.006 | 0.003 | 1.60E-02 | Education | 0.004 | 0.011 | 0.004 | 0.016 | 0.000 | 0.016 | 0.013 | 0.013 | -0.015 | 0.017 |
| rs13240401 | 7 | 2194396 | T | C | 0.018 | 0.002 | 2.36E-24 | 0.008 | 0.003 | 2.90E-03 | Education | -0.026 | 0.012 | -0.033 | 0.017 | -0.018 | 0.017 | -0.030 | 0.014 | -0.024 | 0.018 |
| rs4719944 | 7 | 3496032 | T | C | -0.012 | 0.001 | 9.25E-18 | -0.003 | 0.002 | 1.20E-01 | Education | -0.012 | 0.010 | -0.019 | 0.014 | -0.006 | 0.014 | -0.015 | 0.012 | -0.013 | 0.015 |
| rs929511 | 7 | 8003017 | T | C | -0.016 | 0.002 | 3.91E-14 | -0.011 | 0.003 | 1.30E-03 | Education | 0.007 | 0.015 | -0.009 | 0.022 | 0.021 | 0.022 | -0.007 | 0.019 | 0.027 | 0.025 |
| rs118134876 | 7 | 11500372 | T | C | -0.022 | 0.003 | 1.50E-13 | -0.003 | 0.005 | 5.90E-01 | Education | 0.003 | 0.021 | 0.023 | 0.029 | -0.015 | 0.029 | 0.008 | 0.024 | 0.001 | 0.032 |
| rs6977237 | 7 | 14027991 | T | C | 0.008 | 0.001 | 5.59E-09 | -0.001 | 0.002 | 6.30E-01 | Education | 0.013 | 0.010 | 0.006 | 0.014 | 0.021 | 0.014 | 0.009 | 0.012 | 0.023 | 0.015 |
| rs35929923 | 7 | 21400525 | A | G | -0.011 | 0.002 | 1.77E-12 | 0.000 | 0.003 | 9.40E-01 | Education | 0.000 | 0.011 | -0.016 | 0.016 | 0.014 | 0.016 | -0.004 | 0.013 | 0.008 | 0.017 |
| rs79265434 | 7 | 24621381 | A | G | -0.020 | 0.002 | 1.98E-19 | -0.011 | 0.003 | 1.70E-03 | Education | 0.024 | 0.016 | 0.029 | 0.023 | 0.014 | 0.023 | 0.029 | 0.019 | 0.059 | 0.026 |
| rs10951590 | 7 | 39091043 | T | C | -0.011 | 0.002 | 3.62E-13 | -0.005 | 0.002 | 3.80E-02 | Education | 0.008 | 0.010 | -0.001 | 0.015 | 0.019 | 0.015 | 0.018 | 0.012 | 0.009 | 0.016 |
| rs12670376 | 7 | 48823154 | A | G | 0.009 | 0.001 | 5.99E-11 | 0.002 | 0.002 | 4.70E-01 | Education | 0.000 | 0.010 | 0.004 | 0.014 | -0.001 | 0.014 | 0.008 | 0.012 | -0.011 | 0.015 |
| rs17551064 | 7 | 49869771 | A | G | 0.014 | 0.002 | 1.53E-13 | -0.001 | 0.003 | 6.50E-01 | Education | -0.006 | 0.013 | -0.011 | 0.019 | -0.002 | 0.019 | 0.003 | 0.016 | 0.003 | 0.021 |
| rs1220779 | 7 | 54734673 | A | G | -0.008 | 0.001 | 1.58E-08 | -0.004 | 0.002 | 1.00E-01 | Education | 0.024 | 0.010 | 0.029 | 0.014 | 0.018 | 0.014 | 0.021 | 0.012 | 0.027 | 0.015 |
| rs55772938 | 7 | 70025113 | A | G | -0.007 | 0.002 | 6.27E-05 | -0.015 | 0.002 | 3.90E-10 | PC | -0.008 | 0.011 | 0.001 | 0.015 | -0.017 | 0.015 | -0.001 | 0.013 | -0.006 | 0.016 |
| rs10215082 | 7 | 92657985 | A | G | -0.015 | 0.001 | 7.03E-26 | -0.008 | 0.002 | 1.40E-04 | Education | -0.026 | 0.010 | -0.028 | 0.014 | -0.025 | 0.014 | -0.038 | 0.012 | -0.028 | 0.015 |
| rs401966 | 7 | 101695817 | C | G | -0.010 | 0.001 | 1.72E-11 | 0.006 | 0.002 | 5.30E-03 | Education | -0.010 | 0.010 | -0.012 | 0.014 | -0.007 | 0.014 | -0.004 | 0.012 | -0.012 | 0.016 |
| rs9655780 | 7 | 104667334 | A | G | -0.015 | 0.002 | 3.70E-16 | -0.002 | 0.003 | 5.10E-01 | Education | 0.018 | 0.014 | 0.015 | 0.019 | 0.021 | 0.019 | 0.004 | 0.016 | 0.043 | 0.022 |
| rs12113634 | 7 | 111996952 | T | C | 0.009 | 0.002 | 2.50E-09 | 0.003 | 0.002 | 1.50E-01 | Education | -0.006 | 0.010 | 0.008 | 0.014 | -0.018 | 0.014 | -0.007 | 0.012 | 0.006 | 0.016 |
| rs113615161 | 7 | 114519339 | T | C | -0.012 | 0.002 | 2.98E-09 | -0.009 | 0.003 | 5.20E-03 | Education | -0.001 | 0.014 | -0.002 | 0.020 | -0.002 | 0.020 | 0.000 | 0.017 | -0.030 | 0.023 |
| rs6969783 | 7 | 117593308 | A | T | -0.010 | 0.001 | 2.87E-12 | 0.001 | 0.002 | 6.20E-01 | Education | -0.001 | 0.010 | 0.004 | 0.014 | -0.004 | 0.014 | 0.006 | 0.012 | -0.005 | 0.015 |
| rs12706626 | 7 | 124531370 | G | A | -0.003 | 0.002 | 8.52E-02 | -0.013 | 0.002 | 1.40E-08 | PC | -0.007 | 0.010 | -0.029 | 0.014 | 0.016 | 0.014 | -0.007 | 0.012 | 0.003 | 0.016 |
| rs3944151 | 7 | 126970005 | A | G | 0.004 | 0.002 | 2.25E-02 | 0.014 | 0.002 | 3.10E-08 | PC | 0.021 | 0.011 | 0.020 | 0.015 | 0.019 | 0.015 | 0.029 | 0.013 | 0.016 | 0.017 |
| rs113520408 | 7 | 128402782 | A | G | 0.016 | 0.002 | 1.03E-22 | 0.006 | 0.002 | 2.00E-02 | Education | -0.012 | 0.011 | -0.014 | 0.015 | -0.012 | 0.015 | -0.024 | 0.013 | 0.005 | 0.017 |
| rs2345941 | 7 | 133337635 | A | G | 0.010 | 0.002 | 8.41E-09 | 0.015 | 0.002 | 4.00E-11 | PC | -0.023 | 0.010 | -0.013 | 0.014 | -0.033 | 0.014 | -0.025 | 0.012 | -0.012 | 0.015 |
| rs4726070 | 7 | 151328218 | A | G | 0.012 | 0.001 | 1.36E-17 | 0.004 | 0.002 | 1.10E-01 | Education | 0.000 | 0.010 | -0.014 | 0.014 | 0.013 | 0.014 | 0.007 | 0.012 | -0.004 | 0.015 |
| rs73191311 | 8 | 4796094 | A | G | -0.009 | 0.002 | 2.03E-10 | -0.002 | 0.002 | 3.80E-01 | Education | 0.002 | 0.010 | 0.013 | 0.014 | -0.008 | 0.014 | 0.008 | 0.012 | -0.009 | 0.016 |
| rs6994287 | 8 | 9340932 | A | G | 0.010 | 0.001 | 1.11E-12 | 0.004 | 0.002 | 7.30E-02 | Education | -0.007 | 0.010 | -0.009 | 0.014 | -0.006 | 0.014 | -0.021 | 0.012 | -0.012 | 0.015 |
| rs2517086 | 8 | 17042416 | C | G | -0.009 | 0.001 | 8.00E-10 | 0.001 | 0.002 | 8.00E-01 | Education | 0.003 | 0.010 | -0.015 | 0.014 | 0.021 | 0.014 | 0.009 | 0.012 | 0.006 | 0.015 |
| rs13266287 | 8 | 17871598 | A | T | -0.009 | 0.002 | 1.75E-08 | 0.001 | 0.002 | 7.60E-01 | Education | 0.006 | 0.010 | 0.010 | 0.014 | 0.003 | 0.014 | 0.013 | 0.012 | -0.006 | 0.016 |
| rs34807077 | 8 | 28680769 | A | C | 0.013 | 0.002 | 3.29E-11 | -0.002 | 0.003 | 4.10E-01 | Education | -0.023 | 0.013 | -0.019 | 0.019 | -0.020 | 0.019 | -0.019 | 0.016 | 0.007 | 0.021 |
| rs2725370 | 8 | 30852826 | T | C | -0.014 | 0.002 | 4.96E-20 | -0.004 | 0.002 | 9.20E-02 | Education | 0.011 | 0.011 | 0.025 | 0.015 | 0.001 | 0.015 | 0.018 | 0.013 | -0.005 | 0.017 |
| rs2923424 | 8 | 42382222 | A | G | 0.012 | 0.001 | 6.31E-17 | 0.001 | 0.002 | 5.40E-01 | Education | -0.009 | 0.010 | -0.007 | 0.014 | -0.014 | 0.014 | -0.006 | 0.012 | 0.007 | 0.016 |
| rs2929032 | 8 | 56371745 | A | G | -0.008 | 0.001 | 8.40E-09 | -0.004 | 0.002 | 8.60E-02 | Education | -0.001 | 0.010 | 0.006 | 0.014 | -0.005 | 0.014 | -0.007 | 0.012 | -0.012 | 0.015 |
| rs1866823 | 8 | 57436577 | A | G | 0.011 | 0.001 | 3.09E-14 | 0.001 | 0.002 | 5.40E-01 | Education | -0.015 | 0.010 | -0.032 | 0.014 | 0.003 | 0.014 | -0.013 | 0.012 | -0.019 | 0.016 |
| rs56099375 | 8 | 77372988 | T | C | 0.012 | 0.002 | 3.10E-13 | 0.001 | 0.003 | 6.10E-01 | Education | -0.019 | 0.012 | -0.039 | 0.017 | 0.002 | 0.017 | -0.004 | 0.014 | -0.058 | 0.019 |
| rs4320563 | 8 | 87769961 | A | G | 0.011 | 0.001 | 2.24E-14 | 0.001 | 0.002 | 5.40E-01 | Education | 0.002 | 0.010 | -0.002 | 0.014 | 0.005 | 0.014 | -0.009 | 0.012 | 0.023 | 0.015 |
| rs35606437 | 8 | 93327532 | A | G | 0.011 | 0.002 | 3.98E-11 | 0.008 | 0.002 | 1.10E-03 | Education | 0.002 | 0.011 | -0.015 | 0.015 | 0.015 | 0.015 | -0.003 | 0.013 | -0.006 | 0.017 |
| rs7012546 | 8 | 105067737 | T | C | 0.009 | 0.001 | 1.25E-10 | 0.002 | 0.002 | 4.10E-01 | Education | -0.023 | 0.010 | -0.031 | 0.014 | -0.014 | 0.014 | -0.029 | 0.012 | -0.023 | 0.015 |
| rs72672052 | 8 | 115798424 | A | T | -0.010 | 0.002 | 4.87E-08 | 0.000 | 0.003 | 9.10E-01 | Education | 0.009 | 0.013 | 0.021 | 0.018 | -0.003 | 0.018 | 0.005 | 0.015 | -0.013 | 0.020 |
| rs4298514 | 8 | 118932887 | T | C | 0.010 | 0.002 | 1.07E-11 | 0.008 | 0.002 | 6.90E-04 | Education | -0.009 | 0.011 | -0.018 | 0.015 | 0.002 | 0.015 | -0.018 | 0.013 | -0.038 | 0.017 |
| rs13261773 | 8 | 120187090 | C | G | 0.012 | 0.002 | 3.12E-10 | 0.008 | 0.003 | 1.20E-02 | Education | -0.019 | 0.013 | -0.025 | 0.018 | -0.018 | 0.018 | -0.018 | 0.015 | -0.023 | 0.020 |
| rs401526 | 8 | 130929166 | T | C | -0.010 | 0.001 | 1.15E-13 | -0.005 | 0.002 | 3.00E-02 | Education | 0.034 | 0.010 | 0.041 | 0.014 | 0.029 | 0.014 | 0.042 | 0.012 | 0.045 | 0.015 |
| rs746839 | 8 | 142617261 | C | G | 0.014 | 0.002 | 1.19E-20 | 0.006 | 0.002 | 8.20E-03 | Education | -0.006 | 0.010 | -0.006 | 0.015 | -0.005 | 0.015 | 0.003 | 0.012 | 0.004 | 0.016 |
| rs13262595 | 8 | 143316970 | A | G | -0.012 | 0.002 | 1.85E-11 | -0.016 | 0.002 | 1.20E-12 | PC | -0.001 | 0.010 | 0.004 | 0.014 | -0.008 | 0.014 | -0.007 | 0.012 | 0.012 | 0.015 |
| rs7849487 | 9 | 1757274 | T | G | -0.017 | 0.002 | 1.25E-29 | -0.003 | 0.002 | 2.10E-01 | Education | -0.001 | 0.010 | 0.008 | 0.014 | -0.015 | 0.014 | 0.005 | 0.012 | 0.017 | 0.016 |
| rs73648455 | 9 | 14077378 | T | C | -0.018 | 0.003 | 1.99E-12 | -0.006 | 0.004 | 1.30E-01 | Education | 0.029 | 0.018 | 0.052 | 0.025 | 0.004 | 0.025 | 0.028 | 0.021 | 0.045 | 0.028 |
| rs7875078 | 9 | 14494845 | A | C | -0.008 | 0.001 | 2.72E-08 | 0.002 | 0.002 | 4.20E-01 | Education | -0.001 | 0.010 | -0.008 | 0.014 | 0.006 | 0.014 | 0.007 | 0.012 | -0.004 | 0.015 |
| rs4977839 | 9 | 23355310 | G | A | -0.024 | 0.002 | 2.96E-44 | -0.020 | 0.002 | 4.70E-19 | PC | -0.004 | 0.010 | -0.002 | 0.014 | -0.005 | 0.014 | 0.010 | 0.012 | -0.006 | 0.016 |
| rs4977885 | 9 | 23687983 | A | G | -0.008 | 0.001 | 6.78E-09 | -0.004 | 0.002 | 6.90E-02 | Education | -0.004 | 0.010 | 0.003 | 0.014 | -0.008 | 0.014 | -0.003 | 0.012 | -0.019 | 0.016 |
| rs7041702 | 9 | 33053430 | A | G | -0.011 | 0.002 | 4.53E-12 | -0.004 | 0.003 | 1.20E-01 | Education | 0.021 | 0.011 | 0.034 | 0.016 | 0.004 | 0.016 | 0.030 | 0.013 | 0.016 | 0.017 |
| rs11138947 | 9 | 72110562 | T | C | 0.011 | 0.002 | 2.36E-11 | 0.006 | 0.002 | 1.10E-02 | Education | -0.003 | 0.011 | 0.003 | 0.015 | -0.007 | 0.015 | -0.006 | 0.013 | 0.007 | 0.017 |
| rs9886703 | 9 | 82246351 | A | T | -0.014 | 0.002 | 4.46E-13 | 0.001 | 0.003 | 8.30E-01 | Education | 0.025 | 0.013 | 0.023 | 0.018 | 0.021 | 0.018 | 0.021 | 0.015 | 0.017 | 0.020 |
| rs10761251 | 9 | 96421546 | A | T | 0.011 | 0.002 | 2.52E-14 | 0.001 | 0.002 | 7.20E-01 | Education | -0.004 | 0.010 | -0.010 | 0.014 | 0.004 | 0.014 | -0.003 | 0.012 | -0.001 | 0.016 |
| rs113851275 | 9 | 98297220 | G | A | -0.003 | 0.003 | 2.96E-01 | -0.021 | 0.004 | 3.20E-09 | PC | 0.022 | 0.015 | 0.001 | 0.022 | 0.043 | 0.022 | 0.010 | 0.019 | -0.001 | 0.024 |
| rs7020477 | 9 | 116827760 | A | G | 0.005 | 0.002 | 2.23E-02 | 0.014 | 0.002 | 1.20E-08 | PC | 0.016 | 0.012 | -0.004 | 0.017 | 0.033 | 0.017 | 0.020 | 0.014 | 0.011 | 0.018 |
| rs73578186 | 9 | 126334485 | C | T | 0.010 | 0.002 | 1.02E-07 | 0.015 | 0.002 | 2.30E-10 | PC | -0.015 | 0.010 | -0.022 | 0.014 | -0.006 | 0.015 | -0.018 | 0.012 | -0.003 | 0.016 |
| rs2039204 | 9 | 130371107 | A | T | 0.008 | 0.001 | 5.23E-09 | 0.002 | 0.002 | 4.50E-01 | Education | -0.008 | 0.010 | -0.001 | 0.014 | -0.015 | 0.014 | -0.015 | 0.012 | 0.004 | 0.015 |
| rs73581580 | 9 | 140251458 | A | G | -0.018 | 0.002 | 5.78E-16 | 0.008 | 0.003 | 1.10E-02 | Education | -0.023 | 0.019 | -0.038 | 0.027 | -0.008 | 0.027 | -0.013 | 0.023 | -0.037 | 0.029 |
| rs7894722 | 10 | 9972046 | T | C | 0.009 | 0.001 | 5.41E-09 | 0.002 | 0.002 | 4.70E-01 | Education | 0.014 | 0.010 | -0.020 | 0.014 | 0.045 | 0.014 | 0.001 | 0.012 | 0.001 | 0.016 |
| rs17148998 | 10 | 10909401 | A | G | 0.010 | 0.002 | 6.98E-09 | 0.010 | 0.003 | 4.20E-04 | Education | -0.003 | 0.012 | -0.020 | 0.017 | 0.018 | 0.017 | -0.016 | 0.015 | 0.027 | 0.019 |
| rs1747714 | 10 | 11161316 | T | C | 0.011 | 0.001 | 3.82E-14 | 0.004 | 0.002 | 7.60E-02 | Education | 0.018 | 0.010 | 0.018 | 0.014 | 0.018 | 0.014 | 0.019 | 0.012 | 0.007 | 0.015 |
| rs2183271 | 10 | 21957229 | T | C | 0.008 | 0.002 | 1.91E-08 | -0.008 | 0.002 | 4.30E-04 | Education | -0.016 | 0.010 | -0.017 | 0.014 | -0.015 | 0.015 | -0.011 | 0.012 | -0.042 | 0.016 |
| rs11598765 | 10 | 23953725 | A | G | -0.011 | 0.002 | 1.73E-09 | -0.002 | 0.003 | 5.70E-01 | Education | -0.014 | 0.012 | -0.010 | 0.017 | -0.018 | 0.017 | -0.013 | 0.014 | -0.018 | 0.019 |
| rs806816 | 10 | 32285942 | A | T | 0.010 | 0.002 | 4.80E-10 | -0.001 | 0.003 | 5.80E-01 | Education | 0.000 | 0.011 | -0.007 | 0.016 | 0.006 | 0.016 | 0.008 | 0.013 | -0.014 | 0.017 |
| rs2007655 | 10 | 34586689 | T | G | 0.009 | 0.001 | 8.64E-10 | -0.001 | 0.002 | 7.00E-01 | Education | -0.015 | 0.010 | -0.008 | 0.014 | -0.019 | 0.013 | -0.007 | 0.011 | -0.038 | 0.015 |
| rs11003463 | 10 | 51801793 | T | G | 0.009 | 0.002 | 5.58E-10 | 0.005 | 0.002 | 4.10E-02 | Education | -0.029 | 0.010 | -0.037 | 0.015 | -0.023 | 0.015 | -0.025 | 0.012 | -0.039 | 0.016 |
| rs7924036 | 10 | 65191645 | T | G | 0.013 | 0.001 | 2.55E-21 | 0.002 | 0.002 | 3.60E-01 | Education | 0.008 | 0.010 | -0.006 | 0.014 | 0.017 | 0.014 | 0.012 | 0.012 | -0.002 | 0.015 |
| rs10996167 | 10 | 66813928 | C | G | 0.011 | 0.002 | 7.47E-09 | 0.001 | 0.002 | 7.20E-01 | Education | -0.020 | 0.010 | -0.020 | 0.014 | -0.017 | 0.014 | -0.018 | 0.012 | -0.006 | 0.016 |
| rs7920624 | 10 | 67963186 | A | T | 0.012 | 0.001 | 1.06E-17 | 0.009 | 0.002 | 7.00E-05 | Education | 0.000 | 0.010 | 0.011 | 0.014 | -0.015 | 0.014 | 0.005 | 0.012 | -0.008 | 0.015 |
| rs7910403 | 10 | 87039251 | T | G | 0.012 | 0.002 | 5.08E-12 | 0.001 | 0.003 | 7.10E-01 | Education | -0.015 | 0.013 | 0.013 | 0.018 | -0.046 | 0.018 | -0.024 | 0.015 | -0.006 | 0.019 |
| rs1426619 | 10 | 90091540 | T | C | 0.010 | 0.001 | 1.40E-11 | 0.000 | 0.002 | 8.50E-01 | Education | 0.000 | 0.010 | -0.009 | 0.014 | 0.011 | 0.014 | 0.008 | 0.012 | -0.013 | 0.015 |
| rs17882802 | 10 | 101983413 | A | G | 0.008 | 0.001 | 3.00E-09 | -0.005 | 0.002 | 2.80E-02 | Education | 0.005 | 0.010 | 0.010 | 0.014 | 0.002 | 0.014 | 0.020 | 0.012 | -0.002 | 0.015 |
| rs73344830 | 10 | 103816828 | A | G | 0.017 | 0.001 | 1.00E-32 | 0.005 | 0.002 | 1.40E-02 | Education | 0.002 | 0.010 | 0.009 | 0.014 | -0.008 | 0.014 | 0.000 | 0.012 | -0.004 | 0.015 |
| rs790647 | 10 | 106776484 | A | C | -0.015 | 0.002 | 3.81E-18 | 0.001 | 0.003 | 8.00E-01 | Education | -0.007 | 0.012 | -0.002 | 0.016 | -0.011 | 0.016 | -0.020 | 0.014 | -0.021 | 0.018 |
| rs12571549 | 10 | 111770013 | A | G | 0.015 | 0.002 | 1.45E-13 | 0.005 | 0.003 | 1.50E-01 | Education | -0.014 | 0.013 | -0.018 | 0.019 | -0.009 | 0.019 | -0.006 | 0.017 | -0.006 | 0.022 |
| rs1865955 | 10 | 118837990 | T | C | 0.012 | 0.002 | 4.06E-11 | -0.003 | 0.003 | 2.60E-01 | Education | 0.019 | 0.012 | 0.022 | 0.018 | 0.016 | 0.018 | 0.023 | 0.015 | -0.012 | 0.020 |
| rs4384309 | 10 | 133110596 | A | G | 0.010 | 0.001 | 4.89E-13 | 0.004 | 0.002 | 5.90E-02 | Education | -0.011 | 0.010 | -0.003 | 0.014 | -0.017 | 0.014 | -0.007 | 0.012 | -0.031 | 0.015 |
| rs12761761 | 10 | 133775375 | T | C | 0.015 | 0.002 | 4.79E-20 | -0.001 | 0.003 | 7.70E-01 | Education | 0.013 | 0.012 | 0.001 | 0.016 | 0.024 | 0.016 | 0.014 | 0.014 | 0.015 | 0.018 |
| rs12765185 | 10 | 134977077 | A | T | -0.009 | 0.002 | 2.96E-08 | -0.003 | 0.002 | 2.40E-01 | Education | 0.007 | 0.011 | 0.017 | 0.015 | -0.005 | 0.015 | 0.006 | 0.013 | 0.026 | 0.017 |
| rs4757957 | 11 | 12881398 | C | G | 0.013 | 0.002 | 1.79E-18 | 0.005 | 0.002 | 4.60E-02 | Education | -0.013 | 0.010 | -0.019 | 0.015 | -0.010 | 0.015 | -0.016 | 0.013 | 0.008 | 0.016 |
| rs11023764 | 11 | 15910939 | A | G | 0.009 | 0.002 | 5.32E-10 | 0.007 | 0.002 | 2.30E-03 | Education | -0.012 | 0.010 | -0.022 | 0.015 | 0.002 | 0.014 | -0.024 | 0.012 | 0.008 | 0.016 |
| rs12789313 | 11 | 25058937 | T | C | 0.009 | 0.001 | 6.79E-11 | 0.004 | 0.002 | 5.50E-02 | Education | -0.007 | 0.010 | -0.012 | 0.014 | -0.004 | 0.014 | -0.007 | 0.012 | -0.016 | 0.015 |
| rs11030102 | 11 | 27681596 | C | G | 0.010 | 0.002 | 4.60E-10 | -0.006 | 0.002 | 1.00E-02 | Education | 0.023 | 0.012 | 0.022 | 0.016 | 0.020 | 0.016 | 0.021 | 0.014 | 0.032 | 0.018 |
| rs2199409 | 11 | 39915234 | T | C | 0.010 | 0.002 | 3.14E-09 | 0.003 | 0.003 | 2.60E-01 | Education | 0.016 | 0.012 | -0.005 | 0.017 | 0.035 | 0.017 | 0.017 | 0.014 | 0.007 | 0.018 |
| rs79728014 | 11 | 46062245 | A | G | -0.013 | 0.002 | 2.06E-11 | 0.001 | 0.003 | 7.10E-01 | Education | -0.015 | 0.014 | -0.029 | 0.021 | -0.003 | 0.020 | -0.013 | 0.017 | -0.001 | 0.023 |
| rs75033012 | 11 | 61347033 | C | G | -0.026 | 0.004 | 1.38E-11 | -0.018 | 0.006 | 1.20E-03 | Education | 0.007 | 0.030 | 0.008 | 0.043 | 0.009 | 0.041 | -0.024 | 0.036 | 0.076 | 0.046 |
| rs628993 | 11 | 61539691 | A | G | 0.013 | 0.002 | 3.83E-08 | 0.001 | 0.004 | 8.00E-01 | Education | 0.024 | 0.016 | 0.026 | 0.022 | 0.020 | 0.022 | 0.016 | 0.020 | 0.027 | 0.025 |
| rs76878669 | 11 | 66092567 | C | G | 0.014 | 0.002 | 3.10E-17 | 0.000 | 0.003 | 9.30E-01 | Education | -0.028 | 0.011 | -0.046 | 0.016 | -0.008 | 0.016 | -0.042 | 0.013 | -0.034 | 0.018 |
| rs72962169 | 11 | 72365669 | T | C | -0.017 | 0.002 | 1.26E-19 | -0.003 | 0.003 | 3.00E-01 | Education | -0.019 | 0.014 | -0.038 | 0.019 | -0.001 | 0.019 | -0.024 | 0.016 | -0.006 | 0.021 |
| rs580652 | 11 | 84904742 | T | C | -0.015 | 0.002 | 4.65E-10 | 0.007 | 0.004 | 6.00E-02 | Education | 0.013 | 0.016 | -0.012 | 0.023 | 0.042 | 0.023 | 0.013 | 0.020 | 0.004 | 0.026 |
| rs10830858 | 11 | 91896638 | T | C | 0.009 | 0.001 | 5.31E-11 | 0.006 | 0.002 | 1.00E-02 | Education | -0.003 | 0.010 | 0.001 | 0.014 | -0.005 | 0.014 | -0.005 | 0.012 | 0.005 | 0.015 |
| rs488476 | 11 | 95543715 | C | G | -0.011 | 0.002 | 8.29E-15 | -0.002 | 0.002 | 4.80E-01 | Education | 0.003 | 0.010 | 0.018 | 0.014 | -0.013 | 0.014 | 0.016 | 0.012 | -0.016 | 0.016 |
| rs1301838 | 11 | 99670514 | T | C | -0.010 | 0.002 | 1.29E-10 | -0.001 | 0.002 | 7.40E-01 | Education | 0.005 | 0.010 | -0.002 | 0.015 | 0.011 | 0.015 | 0.001 | 0.012 | 0.013 | 0.016 |
| rs72486027 | 11 | 107167486 | T | C | -0.010 | 0.002 | 1.61E-10 | -0.005 | 0.003 | 4.20E-02 | Education | 0.000 | 0.011 | -0.027 | 0.016 | 0.027 | 0.016 | -0.009 | 0.013 | 0.010 | 0.017 |
| rs2734849 | 11 | 113270160 | A | G | -0.006 | 0.002 | 2.37E-04 | -0.014 | 0.002 | 8.20E-10 | PC | -0.003 | 0.010 | 0.004 | 0.014 | -0.010 | 0.014 | -0.009 | 0.012 | 0.012 | 0.015 |
| rs12574281 | 11 | 131205421 | A | C | -0.008 | 0.002 | 3.04E-08 | -0.001 | 0.002 | 6.80E-01 | Education | 0.006 | 0.010 | 0.018 | 0.014 | -0.005 | 0.014 | 0.018 | 0.012 | 0.001 | 0.016 |
| rs1448355 | 11 | 131286685 | C | T | -0.010 | 0.002 | 2.47E-09 | -0.015 | 0.002 | 1.30E-10 | PC | 0.012 | 0.010 | -0.008 | 0.014 | 0.030 | 0.014 | 0.004 | 0.012 | 0.013 | 0.016 |
| rs9633970 | 11 | 132502212 | T | C | 0.009 | 0.002 | 2.81E-08 | 0.002 | 0.003 | 3.70E-01 | Education | 0.009 | 0.011 | -0.003 | 0.016 | 0.018 | 0.016 | 0.006 | 0.014 | 0.029 | 0.018 |
| rs4766424 | 12 | 1954096 | C | G | 0.014 | 0.002 | 2.47E-11 | 0.002 | 0.003 | 4.60E-01 | Education | 0.006 | 0.015 | 0.009 | 0.021 | 0.010 | 0.021 | 0.001 | 0.017 | 0.002 | 0.023 |
| rs10772644 | 12 | 13417617 | C | G | 0.014 | 0.002 | 1.69E-10 | -0.001 | 0.004 | 7.50E-01 | Education | -0.028 | 0.016 | -0.042 | 0.022 | -0.016 | 0.022 | -0.024 | 0.019 | -0.056 | 0.024 |
| rs1861786 | 12 | 14000467 | A | G | -0.008 | 0.001 | 2.39E-08 | -0.003 | 0.002 | 2.00E-01 | Education | 0.005 | 0.010 | 0.013 | 0.014 | -0.003 | 0.014 | 0.004 | 0.012 | 0.019 | 0.016 |
| rs7974852 | 12 | 14511679 | A | C | 0.012 | 0.001 | 1.14E-16 | 0.004 | 0.002 | 6.70E-02 | Education | -0.006 | 0.010 | -0.010 | 0.014 | -0.003 | 0.014 | -0.011 | 0.012 | -0.018 | 0.015 |
| rs1603460 | 12 | 23092626 | T | G | 0.009 | 0.001 | 2.20E-10 | 0.002 | 0.002 | 4.20E-01 | Education | -0.009 | 0.010 | 0.004 | 0.014 | -0.022 | 0.014 | -0.010 | 0.012 | -0.007 | 0.015 |
| rs117398064 | 12 | 26523628 | C | G | -0.014 | 0.003 | 1.16E-08 | -0.001 | 0.004 | 7.20E-01 | Education | -0.011 | 0.018 | -0.029 | 0.025 | 0.005 | 0.025 | -0.017 | 0.021 | 0.001 | 0.027 |
| rs7972246 | 12 | 27209328 | T | C | 0.011 | 0.002 | 1.01E-13 | -0.003 | 0.002 | 2.60E-01 | Education | -0.012 | 0.010 | 0.006 | 0.014 | -0.031 | 0.014 | -0.018 | 0.012 | -0.002 | 0.016 |
| rs35933007 | 12 | 38929341 | G | A | -0.007 | 0.002 | 1.89E-03 | -0.015 | 0.003 | 9.20E-09 | PC | -0.009 | 0.013 | -0.005 | 0.019 | -0.017 | 0.019 | -0.011 | 0.016 | -0.002 | 0.021 |
| rs80257979 | 12 | 49402562 | T | G | -0.025 | 0.004 | 3.31E-10 | -0.010 | 0.007 | 1.40E-01 | Education | 0.017 | 0.028 | 0.013 | 0.039 | 0.028 | 0.039 | 0.022 | 0.033 | 0.008 | 0.043 |
| rs17110109 | 12 | 54668908 | T | C | -0.009 | 0.001 | 1.47E-10 | -0.005 | 0.002 | 4.60E-02 | Education | -0.008 | 0.010 | -0.004 | 0.014 | -0.011 | 0.015 | -0.009 | 0.012 | -0.012 | 0.016 |
| rs1689510 | 12 | 56396768 | C | G | 0.019 | 0.002 | 1.35E-38 | 0.007 | 0.002 | 1.70E-03 | Education | -0.004 | 0.010 | 0.001 | 0.015 | -0.012 | 0.015 | -0.020 | 0.012 | 0.006 | 0.016 |
| rs3751331 | 12 | 58290278 | A | G | -0.010 | 0.001 | 1.75E-11 | -0.004 | 0.002 | 1.20E-01 | Education | 0.012 | 0.010 | 0.015 | 0.014 | 0.011 | 0.014 | 0.001 | 0.012 | 0.022 | 0.016 |
| rs57661533 | 12 | 78629917 | T | C | -0.011 | 0.002 | 3.24E-08 | -0.010 | 0.003 | 1.60E-03 | Education | -0.004 | 0.014 | -0.008 | 0.019 | 0.000 | 0.019 | -0.011 | 0.017 | -0.020 | 0.022 |
| rs1245829 | 12 | 79648170 | A | T | -0.010 | 0.001 | 3.29E-12 | -0.001 | 0.002 | 5.80E-01 | Education | 0.003 | 0.010 | 0.003 | 0.014 | 0.001 | 0.014 | 0.008 | 0.012 | 0.002 | 0.016 |
| rs717996 | 12 | 84133820 | T | C | -0.012 | 0.001 | 1.89E-17 | -0.002 | 0.002 | 4.80E-01 | Education | -0.005 | 0.010 | -0.012 | 0.014 | 0.003 | 0.014 | -0.017 | 0.012 | 0.019 | 0.016 |
| rs2216144 | 12 | 97666478 | T | C | 0.010 | 0.001 | 2.58E-12 | 0.006 | 0.002 | 4.00E-03 | Education | -0.023 | 0.010 | -0.035 | 0.014 | -0.011 | 0.014 | -0.016 | 0.012 | -0.046 | 0.015 |
| rs2131167 | 12 | 99633189 | A | G | 0.008 | 0.001 | 1.52E-08 | 0.005 | 0.002 | 3.60E-02 | Education | -0.011 | 0.010 | -0.021 | 0.014 | 0.000 | 0.014 | -0.014 | 0.012 | -0.007 | 0.015 |
| rs7977614 | 12 | 110115286 | A | G | -0.013 | 0.002 | 6.84E-17 | -0.002 | 0.002 | 3.90E-01 | Education | 0.007 | 0.011 | 0.015 | 0.016 | 0.001 | 0.016 | 0.002 | 0.014 | 0.023 | 0.018 |
| rs206965 | 12 | 120856332 | T | C | 0.008 | 0.002 | 9.36E-05 | 0.016 | 0.003 | 9.10E-09 | PC | -0.008 | 0.012 | -0.014 | 0.017 | 0.001 | 0.017 | -0.001 | 0.014 | -0.029 | 0.019 |
| rs10773208 | 12 | 122970156 | T | C | -0.011 | 0.002 | 8.09E-12 | 0.002 | 0.003 | 5.00E-01 | Education | -0.008 | 0.011 | 0.003 | 0.016 | -0.017 | 0.016 | -0.007 | 0.013 | -0.009 | 0.017 |
| rs10773002 | 12 | 123746961 | A | T | 0.019 | 0.002 | 2.50E-30 | 0.000 | 0.003 | 9.80E-01 | Education | -0.025 | 0.011 | -0.016 | 0.015 | -0.035 | 0.015 | -0.031 | 0.013 | -0.036 | 0.017 |
| rs6490618 | 13 | 21338343 | T | C | 0.009 | 0.002 | 3.48E-09 | 0.008 | 0.002 | 8.20E-04 | Education | 0.017 | 0.010 | 0.003 | 0.015 | 0.036 | 0.015 | 0.002 | 0.012 | 0.038 | 0.016 |
| rs9529146 | 13 | 31785404 | T | C | 0.013 | 0.002 | 3.48E-14 | 0.007 | 0.003 | 4.50E-03 | Education | -0.004 | 0.011 | 0.014 | 0.016 | -0.021 | 0.016 | 0.002 | 0.014 | -0.009 | 0.018 |
| rs936496 | 13 | 36100541 | A | G | -0.008 | 0.001 | 4.38E-08 | -0.005 | 0.002 | 4.00E-02 | Education | 0.002 | 0.010 | -0.002 | 0.014 | 0.007 | 0.014 | -0.001 | 0.012 | -0.001 | 0.016 |
| rs12874776 | 13 | 57794345 | C | A | -0.015 | 0.003 | 1.13E-07 | -0.023 | 0.004 | 8.00E-11 | PC | -0.010 | 0.016 | -0.008 | 0.023 | -0.009 | 0.022 | -0.008 | 0.019 | -0.012 | 0.025 |
| rs7321274 | 13 | 69146186 | A | G | 0.011 | 0.002 | 8.21E-11 | 0.008 | 0.003 | 5.40E-03 | Education | 0.018 | 0.012 | 0.024 | 0.017 | 0.010 | 0.017 | 0.005 | 0.015 | 0.023 | 0.019 |
| rs2478208 | 13 | 81471113 | C | G | -0.010 | 0.001 | 2.89E-13 | -0.003 | 0.002 | 2.20E-01 | Education | -0.002 | 0.010 | -0.005 | 0.014 | 0.000 | 0.014 | -0.004 | 0.012 | -0.020 | 0.015 |
| rs7326331 | 13 | 91615838 | A | G | -0.013 | 0.002 | 3.42E-16 | -0.007 | 0.002 | 3.20E-03 | Education | 0.014 | 0.011 | 0.011 | 0.015 | 0.020 | 0.015 | 0.004 | 0.013 | 0.021 | 0.017 |
| rs11620355 | 13 | 92044587 | A | G | 0.018 | 0.003 | 1.09E-13 | 0.005 | 0.004 | 2.10E-01 | Education | -0.026 | 0.017 | -0.017 | 0.023 | -0.030 | 0.024 | -0.036 | 0.020 | -0.006 | 0.026 |
| rs9556958 | 13 | 99100046 | T | C | -0.011 | 0.001 | 5.96E-15 | -0.007 | 0.002 | 9.80E-04 | Education | -0.004 | 0.010 | -0.008 | 0.014 | 0.002 | 0.014 | -0.012 | 0.012 | 0.002 | 0.015 |
| rs11157931 | 14 | 23403193 | A | C | -0.014 | 0.001 | 3.86E-22 | -0.005 | 0.002 | 3.50E-02 | Education | 0.018 | 0.012 | 0.029 | 0.014 | 0.028 | 0.014 | 0.031 | 0.012 | 0.020 | 0.015 |
| rs178183 | 14 | 26979412 | T | C | 0.015 | 0.002 | 4.81E-19 | 0.007 | 0.003 | 3.70E-03 | Education | -0.015 | 0.013 | -0.009 | 0.016 | -0.023 | 0.016 | -0.017 | 0.013 | -0.007 | 0.017 |
| rs176218 | 14 | 29600506 | T | G | 0.020 | 0.002 | 1.10E-29 | 0.003 | 0.003 | 3.10E-01 | Education | 0.002 | 0.014 | 0.008 | 0.017 | -0.007 | 0.017 | -0.010 | 0.015 | 0.010 | 0.019 |
| rs198262 | 14 | 57283283 | C | T | 0.020 | 0.004 | 1.49E-06 | 0.030 | 0.005 | 3.90E-08 | PC | -0.023 | 0.030 | 0.005 | 0.035 | -0.003 | 0.034 | 0.015 | 0.029 | -0.038 | 0.038 |
| rs4899012 | 14 | 61003889 | C | G | -0.010 | 0.001 | 8.06E-13 | -0.007 | 0.002 | 1.50E-03 | Education | 0.005 | 0.012 | 0.023 | 0.014 | -0.002 | 0.014 | 0.011 | 0.012 | 0.030 | 0.015 |
| rs12888615 | 14 | 72435705 | T | C | 0.012 | 0.002 | 9.42E-12 | 0.000 | 0.003 | 9.00E-01 | Education | 0.001 | 0.015 | 0.012 | 0.018 | -0.035 | 0.018 | -0.003 | 0.015 | -0.001 | 0.020 |
| rs730384 | 14 | 74889870 | A | G | 0.010 | 0.001 | 9.38E-13 | -0.003 | 0.002 | 1.70E-01 | Education | 0.003 | 0.012 | 0.003 | 0.014 | 0.013 | 0.014 | 0.007 | 0.012 | -0.011 | 0.015 |
| rs2998299 | 14 | 84997363 | T | G | -0.014 | 0.002 | 1.51E-15 | -0.005 | 0.003 | 4.40E-02 | Education | -0.007 | 0.014 | 0.000 | 0.016 | -0.003 | 0.016 | -0.008 | 0.014 | 0.003 | 0.018 |
| rs4904523 | 14 | 89723630 | A | G | -0.008 | 0.001 | 6.27E-09 | -0.002 | 0.002 | 3.20E-01 | Education | 0.001 | 0.011 | 0.028 | 0.014 | -0.031 | 0.014 | 0.004 | 0.012 | 0.023 | 0.015 |
| rs736281 | 14 | 94287830 | T | C | 0.009 | 0.001 | 2.49E-09 | 0.008 | 0.002 | 8.20E-04 | Education | -0.026 | 0.012 | -0.021 | 0.014 | -0.014 | 0.014 | -0.021 | 0.012 | -0.030 | 0.015 |
| rs2496482 | 14 | 99750164 | T | C | 0.012 | 0.002 | 9.20E-17 | 0.009 | 0.002 | 1.10E-04 | Education | -0.018 | 0.012 | -0.028 | 0.014 | -0.006 | 0.014 | -0.016 | 0.012 | -0.043 | 0.016 |
| rs8008382 | 14 | 104054425 | T | C | -0.011 | 0.002 | 4.07E-12 | -0.005 | 0.002 | 4.80E-02 | Education | 0.001 | 0.012 | -0.014 | 0.015 | 0.014 | 0.015 | -0.003 | 0.013 | -0.001 | 0.016 |
| rs7167688 | 15 | 26813137 | T | C | -0.009 | 0.001 | 1.71E-11 | -0.007 | 0.002 | 1.70E-03 | Education | -0.004 | 0.010 | 0.012 | 0.014 | -0.020 | 0.014 | 0.002 | 0.012 | -0.013 | 0.015 |
| rs891793 | 15 | 27256021 | C | G | -0.010 | 0.001 | 1.87E-12 | -0.003 | 0.002 | 1.90E-01 | Education | 0.017 | 0.010 | 0.025 | 0.014 | 0.017 | 0.014 | 0.012 | 0.012 | 0.036 | 0.015 |
| rs166835 | 15 | 47716037 | C | T | 0.005 | 0.002 | 6.84E-03 | 0.013 | 0.002 | 3.40E-09 | PC | -0.007 | 0.010 | -0.013 | 0.014 | 0.002 | 0.014 | -0.001 | 0.012 | -0.012 | 0.015 |
| rs7171405 | 15 | 61466893 | A | G | -0.010 | 0.002 | 7.53E-10 | 0.000 | 0.003 | 9.70E-01 | Education | 0.026 | 0.011 | 0.015 | 0.016 | 0.037 | 0.016 | 0.026 | 0.013 | 0.023 | 0.017 |
| rs12591647 | 15 | 65959729 | T | C | -0.015 | 0.002 | 7.78E-17 | -0.009 | 0.003 | 1.70E-03 | Education | 0.005 | 0.012 | 0.005 | 0.018 | 0.006 | 0.017 | -0.010 | 0.015 | 0.035 | 0.020 |
| rs1564347 | 15 | 73438848 | T | G | 0.009 | 0.002 | 2.39E-09 | 0.004 | 0.002 | 8.20E-02 | Education | -0.013 | 0.010 | -0.013 | 0.014 | -0.011 | 0.014 | -0.020 | 0.012 | -0.012 | 0.016 |
| rs2469226 | 15 | 77246990 | A | T | 0.010 | 0.002 | 1.28E-08 | 0.006 | 0.003 | 3.50E-02 | Education | 0.009 | 0.012 | 0.003 | 0.017 | 0.015 | 0.017 | -0.004 | 0.014 | 0.027 | 0.018 |
| rs56391344 | 15 | 78006899 | A | G | 0.015 | 0.002 | 7.49E-20 | 0.002 | 0.003 | 3.40E-01 | Education | 0.013 | 0.011 | 0.020 | 0.016 | 0.006 | 0.016 | 0.021 | 0.013 | 0.011 | 0.018 |
| rs4702 | 15 | 91426560 | G | A | 0.000 | 0.002 | 9.61E-01 | 0.012 | 0.002 | 3.80E-08 | PC | 0.018 | 0.010 | 0.022 | 0.014 | 0.014 | 0.014 | 0.020 | 0.012 | 0.013 | 0.015 |
| rs9933256 | 16 | 1246748 | A | G | 0.012 | 0.001 | 6.06E-17 | 0.006 | 0.002 | 5.50E-03 | Education | 0.013 | 0.010 | -0.008 | 0.015 | 0.033 | 0.014 | 0.020 | 0.012 | 0.005 | 0.016 |
| rs11076962 | 16 | 5811367 | T | C | 0.009 | 0.002 | 4.29E-08 | 0.004 | 0.002 | 9.20E-02 | Education | -0.009 | 0.011 | -0.010 | 0.015 | -0.008 | 0.015 | -0.003 | 0.013 | -0.006 | 0.017 |
| rs34098770 | 16 | 12237812 | A | G | -0.016 | 0.002 | 2.95E-16 | -0.005 | 0.003 | 7.90E-02 | Education | 0.010 | 0.013 | 0.005 | 0.019 | 0.013 | 0.018 | 0.012 | 0.016 | 0.012 | 0.021 |
| rs9927842 | 16 | 15153717 | T | C | -0.013 | 0.002 | 6.22E-11 | 0.004 | 0.003 | 2.20E-01 | Education | 0.018 | 0.014 | 0.025 | 0.019 | 0.009 | 0.019 | 0.020 | 0.016 | -0.007 | 0.021 |
| rs6498759 | 16 | 18020295 | T | C | 0.000 | 0.002 | 8.45E-01 | 0.013 | 0.002 | 2.70E-08 | PC | -0.003 | 0.010 | 0.001 | 0.014 | -0.009 | 0.014 | -0.004 | 0.012 | 0.000 | 0.016 |
| rs4787457 | 16 | 28555400 | A | G | 0.016 | 0.002 | 1.23E-28 | 0.004 | 0.002 | 4.80E-02 | Education | -0.013 | 0.010 | -0.015 | 0.014 | -0.011 | 0.014 | -0.024 | 0.012 | 0.013 | 0.016 |
| rs9927137 | 16 | 30634300 | A | G | 0.008 | 0.001 | 5.11E-09 | 0.004 | 0.002 | 5.70E-02 | Education | -0.024 | 0.010 | -0.008 | 0.014 | -0.041 | 0.014 | -0.024 | 0.012 | -0.010 | 0.016 |
| rs2052285 | 16 | 51188432 | A | G | 0.011 | 0.001 | 7.25E-15 | 0.006 | 0.002 | 6.50E-03 | Education | -0.017 | 0.010 | -0.005 | 0.014 | -0.030 | 0.014 | -0.020 | 0.012 | -0.012 | 0.015 |
| rs3809634 | 16 | 53538157 | A | G | -0.011 | 0.002 | 6.82E-13 | -0.009 | 0.002 | 1.00E-04 | Education | 0.011 | 0.011 | 0.004 | 0.015 | 0.018 | 0.015 | 0.009 | 0.013 | 0.031 | 0.016 |
| rs17248751 | 16 | 61579618 | A | G | -0.014 | 0.002 | 1.28E-15 | -0.008 | 0.003 | 1.70E-03 | Education | 0.012 | 0.012 | 0.035 | 0.017 | -0.011 | 0.017 | -0.002 | 0.014 | 0.028 | 0.019 |
| rs3730399 | 16 | 67229019 | A | G | 0.004 | 0.003 | 2.51E-01 | 0.025 | 0.004 | 3.30E-08 | PC | -0.054 | 0.020 | -0.003 | 0.028 | -0.104 | 0.029 | -0.075 | 0.024 | -0.048 | 0.031 |
| rs9929762 | 16 | 78169675 | A | G | 0.010 | 0.001 | 1.03E-11 | 0.000 | 0.002 | 8.80E-01 | Education | 0.007 | 0.010 | 0.037 | 0.014 | -0.020 | 0.014 | 0.011 | 0.012 | 0.012 | 0.015 |
| rs61527214 | 16 | 83608599 | A | G | 0.011 | 0.001 | 1.13E-14 | 0.005 | 0.002 | 2.50E-02 | Education | -0.018 | 0.010 | -0.019 | 0.014 | -0.015 | 0.014 | -0.027 | 0.012 | 0.006 | 0.016 |
| rs74415461 | 16 | 89990843 | T | C | 0.018 | 0.003 | 6.25E-12 | 0.002 | 0.004 | 6.60E-01 | Education | -0.010 | 0.019 | 0.010 | 0.027 | -0.027 | 0.027 | -0.017 | 0.023 | 0.047 | 0.029 |
| rs2447097 | 17 | 2278064 | T | G | 0.010 | 0.001 | 1.10E-11 | 0.005 | 0.002 | 4.20E-02 | Education | -0.013 | 0.010 | -0.033 | 0.014 | 0.007 | 0.014 | -0.013 | 0.012 | 0.000 | 0.015 |
| rs17732878 | 17 | 7362359 | T | C | -0.010 | 0.002 | 4.06E-08 | -0.004 | 0.003 | 1.10E-01 | Education | -0.016 | 0.012 | -0.006 | 0.017 | -0.025 | 0.017 | -0.008 | 0.014 | -0.020 | 0.019 |
| rs7209653 | 17 | 19882084 | T | C | 0.008 | 0.002 | 4.49E-05 | 0.016 | 0.002 | 9.10E-11 | PC | -0.006 | 0.011 | -0.010 | 0.015 | 0.001 | 0.015 | -0.016 | 0.013 | 0.011 | 0.017 |
| rs11657979 | 17 | 32900837 | A | G | -0.009 | 0.002 | 4.57E-08 | -0.002 | 0.003 | 5.40E-01 | Education | 0.002 | 0.012 | -0.010 | 0.017 | 0.013 | 0.017 | 0.008 | 0.014 | -0.027 | 0.019 |
| rs1842713 | 17 | 33200385 | A | G | -0.012 | 0.002 | 9.67E-12 | -0.009 | 0.003 | 6.90E-04 | Education | 0.017 | 0.012 | 0.028 | 0.016 | 0.010 | 0.017 | 0.014 | 0.014 | 0.029 | 0.019 |
| rs12603813 | 17 | 43196584 | T | C | 0.006 | 0.002 | 4.02E-03 | 0.014 | 0.003 | 1.30E-08 | PC | -0.003 | 0.011 | -0.024 | 0.016 | 0.018 | 0.016 | 0.012 | 0.013 | -0.019 | 0.017 |
| rs68145588 | 17 | 75864805 | T | G | -0.013 | 0.002 | 7.53E-10 | -0.001 | 0.003 | 7.50E-01 | Education | -0.026 | 0.014 | -0.018 | 0.020 | -0.037 | 0.020 | -0.039 | 0.017 | -0.022 | 0.023 |
| rs66643547 | 17 | 79375315 | C | T | -0.013 | 0.002 | 2.85E-11 | -0.015 | 0.002 | 3.20E-11 | PC | 0.011 | 0.010 | 0.010 | 0.015 | 0.011 | 0.015 | 0.009 | 0.012 | 0.016 | 0.016 |
| rs12967010 | 18 | 9645035 | T | C | 0.011 | 0.002 | 1.33E-11 | 0.001 | 0.003 | 6.40E-01 | Education | -0.001 | 0.012 | 0.011 | 0.017 | -0.015 | 0.017 | -0.002 | 0.014 | -0.019 | 0.018 |
| rs8097125 | 18 | 13005473 | T | C | 0.009 | 0.001 | 2.31E-09 | 0.005 | 0.002 | 2.20E-02 | Education | 0.002 | 0.010 | -0.008 | 0.014 | 0.009 | 0.014 | 0.009 | 0.012 | 0.003 | 0.016 |
| rs303752 | 18 | 21074255 | A | G | -0.011 | 0.001 | 1.47E-14 | -0.010 | 0.002 | 4.50E-06 | Education | 0.012 | 0.010 | 0.031 | 0.014 | -0.005 | 0.014 | 0.008 | 0.012 | 0.022 | 0.016 |
| rs10460095 | 18 | 22624708 | A | G | -0.012 | 0.001 | 2.61E-18 | -0.002 | 0.002 | 4.10E-01 | Education | 0.027 | 0.010 | 0.027 | 0.014 | 0.024 | 0.014 | 0.035 | 0.012 | 0.011 | 0.015 |
| rs111852224 | 18 | 25596884 | T | C | 0.016 | 0.002 | 4.85E-13 | 0.013 | 0.003 | 1.40E-04 | Education | 0.038 | 0.016 | 0.044 | 0.022 | 0.038 | 0.022 | 0.037 | 0.018 | 0.010 | 0.024 |
| rs7226824 | 18 | 27676827 | T | C | -0.008 | 0.001 | 2.72E-08 | 0.001 | 0.002 | 8.10E-01 | Education | -0.011 | 0.010 | -0.009 | 0.014 | -0.013 | 0.014 | -0.012 | 0.012 | -0.008 | 0.015 |
| rs11082011 | 18 | 35145122 | T | C | 0.019 | 0.002 | 2.06E-38 | 0.005 | 0.002 | 4.40E-02 | Education | -0.001 | 0.010 | -0.008 | 0.014 | 0.005 | 0.015 | -0.002 | 0.012 | -0.005 | 0.016 |
| rs12957463 | 18 | 37412228 | A | G | 0.015 | 0.002 | 9.20E-17 | 0.014 | 0.003 | 7.70E-07 | Education | -0.008 | 0.012 | 0.010 | 0.017 | -0.027 | 0.017 | -0.007 | 0.014 | -0.016 | 0.019 |
| rs784256 | 18 | 53398626 | G | A | 0.015 | 0.002 | 9.15E-12 | 0.019 | 0.003 | 6.20E-11 | PC | 0.011 | 0.013 | 0.010 | 0.018 | 0.013 | 0.018 | 0.006 | 0.015 | 0.006 | 0.020 |
| rs72944064 | 18 | 63516255 | T | C | -0.010 | 0.002 | 9.39E-10 | -0.007 | 0.003 | 3.30E-03 | Education | 0.024 | 0.011 | 0.033 | 0.016 | 0.017 | 0.016 | 0.024 | 0.014 | 0.020 | 0.018 |
| rs2554835 | 18 | 74141190 | A | G | 0.009 | 0.001 | 8.95E-10 | 0.005 | 0.002 | 1.50E-02 | Education | 0.005 | 0.010 | 0.009 | 0.015 | 0.002 | 0.015 | 0.001 | 0.012 | -0.011 | 0.016 |
| rs11081529 | 18 | 75902735 | T | C | 0.013 | 0.002 | 5.22E-16 | 0.010 | 0.002 | 3.60E-05 | Education | 0.004 | 0.011 | 0.017 | 0.016 | -0.008 | 0.016 | 0.009 | 0.013 | 0.001 | 0.017 |
| rs11663602 | 18 | 77578191 | A | C | -0.013 | 0.002 | 9.27E-17 | 0.002 | 0.002 | 4.60E-01 | Education | 0.012 | 0.011 | 0.003 | 0.015 | 0.019 | 0.015 | 0.015 | 0.013 | 0.030 | 0.017 |
| rs75177132 | 19 | 4386911 | T | C | 0.030 | 0.004 | 1.01E-16 | -0.004 | 0.005 | 4.80E-01 | Education | -0.016 | 0.030 | 0.001 | 0.042 | -0.035 | 0.041 | -0.059 | 0.035 | 0.065 | 0.045 |
| rs2287838 | 19 | 9959014 | A | G | -0.010 | 0.001 | 1.27E-13 | -0.005 | 0.002 | 2.50E-02 | Education | -0.001 | 0.010 | 0.007 | 0.014 | -0.008 | 0.014 | -0.006 | 0.012 | 0.008 | 0.015 |
| rs12151248 | 19 | 13212025 | T | C | -0.015 | 0.002 | 3.81E-11 | -0.015 | 0.003 | 2.20E-05 | Education | 0.005 | 0.016 | -0.005 | 0.023 | 0.007 | 0.023 | 0.025 | 0.019 | 0.005 | 0.025 |
| rs12981405 | 19 | 19651577 | T | C | -0.011 | 0.002 | 2.75E-09 | 0.003 | 0.003 | 3.30E-01 | Education | 0.005 | 0.013 | -0.023 | 0.018 | 0.032 | 0.018 | -0.010 | 0.016 | 0.015 | 0.020 |
| rs7254263 | 19 | 30741257 | T | C | -0.012 | 0.002 | 1.84E-14 | -0.005 | 0.002 | 3.70E-02 | Education | -0.003 | 0.011 | -0.011 | 0.015 | 0.005 | 0.015 | -0.010 | 0.013 | 0.017 | 0.017 |
| rs3859523 | 19 | 32972296 | T | C | 0.012 | 0.002 | 9.92E-12 | 0.008 | 0.003 | 6.50E-03 | Education | 0.020 | 0.013 | 0.015 | 0.018 | 0.026 | 0.018 | 0.019 | 0.015 | 0.058 | 0.020 |
| rs173003 | 19 | 36240960 | A | C | -0.008 | 0.001 | 5.50E-09 | -0.009 | 0.002 | 1.80E-05 | Education | -0.002 | 0.010 | 0.001 | 0.014 | -0.002 | 0.014 | -0.007 | 0.012 | 0.004 | 0.015 |
| rs76246107 | 19 | 50121274 | A | G | -0.016 | 0.003 | 6.52E-10 | 0.004 | 0.004 | 3.80E-01 | Education | -0.028 | 0.019 | -0.001 | 0.026 | -0.056 | 0.027 | -0.046 | 0.023 | -0.035 | 0.029 |
| rs192436652 | 19 | 54960747 | T | C | -0.032 | 0.005 | 2.06E-12 | 0.004 | 0.007 | 5.10E-01 | Education | 0.027 | 0.031 | 0.067 | 0.044 | -0.018 | 0.044 | 0.023 | 0.038 | 0.048 | 0.049 |
| rs6043521 | 20 | 15739745 | T | C | -0.008 | 0.001 | 9.72E-09 | 0.009 | 0.002 | 3.80E-05 | Education | -0.002 | 0.010 | 0.010 | 0.014 | -0.015 | 0.014 | 0.004 | 0.012 | 0.003 | 0.016 |
| rs175325 | 20 | 22317310 | A | T | -0.010 | 0.001 | 2.24E-12 | -0.006 | 0.002 | 1.30E-02 | Education | -0.014 | 0.010 | -0.009 | 0.014 | -0.019 | 0.014 | -0.009 | 0.012 | -0.025 | 0.015 |
| rs4369924 | 20 | 41954383 | A | G | 0.013 | 0.002 | 5.58E-11 | 0.005 | 0.003 | 1.00E-01 | Education | -0.018 | 0.013 | -0.009 | 0.018 | -0.030 | 0.018 | -0.023 | 0.016 | -0.041 | 0.021 |
| rs6065784 | 20 | 43717435 | C | G | 0.011 | 0.002 | 5.78E-13 | 0.001 | 0.002 | 7.60E-01 | Education | -0.001 | 0.011 | -0.007 | 0.015 | 0.006 | 0.015 | -0.002 | 0.013 | -0.006 | 0.016 |
| rs4810894 | 20 | 47532999 | A | G | -0.009 | 0.002 | 4.38E-09 | 0.001 | 0.002 | 7.60E-01 | Education | -0.007 | 0.010 | -0.010 | 0.014 | -0.005 | 0.014 | -0.014 | 0.012 | 0.014 | 0.016 |
| rs6123924 | 20 | 58219764 | A | G | 0.013 | 0.002 | 3.20E-11 | 0.010 | 0.003 | 1.10E-03 | Education | 0.004 | 0.013 | 0.014 | 0.018 | -0.004 | 0.018 | 0.003 | 0.016 | 0.022 | 0.021 |
| rs6129084 | 20 | 59828857 | A | T | -0.012 | 0.002 | 3.05E-12 | -0.014 | 0.002 | 9.30E-10 | PC | 0.015 | 0.010 | 0.019 | 0.014 | 0.009 | 0.014 | 0.003 | 0.012 | 0.051 | 0.016 |
| rs13050131 | 21 | 20018118 | A | C | -0.008 | 0.002 | 4.40E-08 | -0.007 | 0.002 | 3.10E-03 | Education | -0.012 | 0.010 | -0.018 | 0.015 | -0.005 | 0.015 | -0.017 | 0.012 | -0.009 | 0.016 |
| rs232496 | 21 | 22734409 | T | C | 0.009 | 0.002 | 7.14E-10 | -0.001 | 0.002 | 6.00E-01 | Education | 0.005 | 0.010 | -0.001 | 0.014 | 0.006 | 0.014 | -0.001 | 0.012 | 0.006 | 0.016 |
| rs1544 | 21 | 33085585 | A | G | -0.010 | 0.002 | 1.76E-09 | 0.005 | 0.002 | 5.00E-02 | Education | -0.002 | 0.011 | 0.023 | 0.016 | -0.021 | 0.016 | 0.000 | 0.014 | 0.017 | 0.018 |
| rs743316 | 21 | 35252696 | T | C | 0.011 | 0.002 | 8.21E-11 | 0.000 | 0.003 | 9.00E-01 | Education | -0.014 | 0.012 | -0.006 | 0.017 | -0.022 | 0.017 | -0.012 | 0.014 | -0.007 | 0.019 |
| rs2838006 | 21 | 42653567 | T | C | 0.013 | 0.002 | 1.38E-19 | 0.001 | 0.002 | 6.10E-01 | Education | -0.006 | 0.010 | -0.001 | 0.014 | -0.011 | 0.014 | -0.003 | 0.012 | -0.014 | 0.016 |
| rs2297293 | 21 | 47125046 | C | G | 0.010 | 0.002 | 8.80E-11 | 0.002 | 0.002 | 3.20E-01 | Education | -0.021 | 0.010 | -0.012 | 0.015 | -0.030 | 0.015 | -0.012 | 0.013 | -0.015 | 0.017 |
| rs136553 | 22 | 27255675 | C | T | -0.005 | 0.002 | 2.43E-03 | -0.015 | 0.002 | 2.30E-11 | PC | 0.014 | 0.010 | 0.024 | 0.014 | 0.005 | 0.014 | 0.007 | 0.012 | 0.011 | 0.016 |
| rs143743568 | 22 | 39289014 | A | G | 0.012 | 0.002 | 4.99E-09 | -0.004 | 0.003 | 2.00E-01 | Education | 0.000 | 0.013 | -0.017 | 0.019 | 0.014 | 0.019 | 0.000 | 0.017 | 0.008 | 0.022 |
| rs7288455 | 22 | 39966547 | A | G | 0.010 | 0.002 | 1.85E-09 | 0.013 | 0.002 | 4.00E-09 | PC | -0.002 | 0.010 | -0.004 | 0.014 | -0.004 | 0.014 | 0.006 | 0.012 | -0.010 | 0.016 |
| rs137079 | 22 | 42977907 | T | C | 0.013 | 0.002 | 3.06E-10 | 0.001 | 0.003 | 7.00E-01 | Education | -0.012 | 0.014 | -0.011 | 0.020 | -0.017 | 0.020 | -0.015 | 0.017 | -0.019 | 0.022 |
| rs9616947 | 22 | 51151631 | T | C | -0.013 | 0.002 | 3.58E-20 | -0.003 | 0.002 | 1.70E-01 | Education | 0.005 | 0.011 | 0.009 | 0.016 | 0.002 | 0.016 | 0.013 | 0.013 | -0.032 | 0.017 |

| **Table S20. Beta estimates for the SNPs included in the multivariable MR of years of education, leisure television watching, and breast cancer risk** | | | | | | | | | | | | | | | | | | | | | | | | |
| --- | --- | --- | --- | --- | --- | --- | --- | --- | --- | --- | --- | --- | --- | --- | --- | --- | --- | --- | --- | --- | --- | --- | --- | --- |
| SNP | CHR | POS | EA | OA | EAF | beta_ed | se_ed | pval_ed | beta_tv | se_tv | pval_tv | Phenotype | beta_br | se_br | Luminal_A  _beta | Luminal_A  _se | Luminal_B  _beta | Luminal_B  _se | Luminal_B  _HER2Neg  _beta | Luminal_B  _HER2Neg  _se | HER2  _Enriched  _beta | HER2_  Enriched  _se | Triple  _Neg_  beta | Triple_  Neg_  se |
| rs34394051 | 1 | 6853091 | A | G | 0.8429 | -0.012 | 0.002 | 2.30E-09 | 0.009 | 0.003 | 2.50E-03 | education | 0.001 | 0.009 | 0.001 | 0.012 | 0.004 | 0.025 | 0.041 | 0.022 | -0.014 | 0.037 | -0.022 | 0.022 |
| rs11121177 | 1 | 8445946 | A | G | 0.1785 | 0.015 | 0.002 | 1.44E-16 | -0.009 | 0.003 | 2.00E-03 | education | 0.013 | 0.008 | 0.030 | 0.011 | -0.031 | 0.024 | -0.017 | 0.020 | -0.034 | 0.034 | 0.013 | 0.020 |
| rs10799615 | 1 | 20541370 | A | G | 0.2525 | -0.009 | 0.002 | 4.11E-08 | 0.008 | 0.002 | 2.20E-03 | education | 0.000 | 0.007 | -0.003 | 0.010 | 0.032 | 0.021 | -0.017 | 0.018 | 0.033 | 0.031 | 0.012 | 0.018 |
| rs590013 | 1 | 29155738 | T | C | 0.6772 | 0.010 | 0.002 | 2.47E-11 | -0.004 | 0.002 | 1.10E-01 | education | -0.013 | 0.006 | -0.006 | 0.008 | 0.007 | 0.019 | -0.004 | 0.016 | -0.029 | 0.027 | -0.004 | 0.016 |
| rs10798888 | 1 | 32199099 | T | G | 0.1725 | -0.014 | 0.002 | 5.15E-14 | 0.013 | 0.003 | 4.30E-06 | education | 0.009 | 0.009 | -0.004 | 0.011 | 0.055 | 0.024 | 0.043 | 0.021 | -0.027 | 0.036 | 0.017 | 0.021 |
| rs11810109 | 1 | 44186812 | A | T | 0.701 | -0.019 | 0.002 | 1.16E-23 | 0.016 | 0.002 | 5.40E-12 | TV | 0.005 | 0.007 | -0.001 | 0.009 | 0.020 | 0.019 | 0.016 | 0.016 | 0.000 | 0.028 | 0.026 | 0.017 |
| rs1738050 | 1 | 44707295 | C | G | 0.6197 | -0.009 | 0.001 | 1.42E-10 | 0.003 | 0.002 | 1.90E-01 | education | -0.009 | 0.006 | -0.016 | 0.008 | -0.025 | 0.018 | 0.010 | 0.016 | -0.031 | 0.026 | -0.009 | 0.016 |
| rs11211123 | 1 | 45957609 | A | G | 0.2228 | 0.010 | 0.002 | 7.62E-10 | 0.000 | 0.003 | 9.70E-01 | education | -0.032 | 0.007 | -0.040 | 0.010 | -0.041 | 0.021 | -0.021 | 0.018 | -0.069 | 0.031 | -0.010 | 0.018 |
| rs2989476 | 1 | 61059259 | C | G | 0.4193 | 0.008 | 0.001 | 3.73E-08 | -0.012 | 0.002 | 1.00E-07 | education | -0.006 | 0.006 | 0.001 | 0.008 | 0.001 | 0.018 | -0.021 | 0.016 | 0.030 | 0.026 | -0.019 | 0.016 |
| rs12725114 | 1 | 62432641 | G | A | 0.802 | -0.004 | 0.002 | 6.55E-02 | 0.015 | 0.003 | 1.60E-08 | TV | 0.007 | 0.007 | 0.016 | 0.010 | 0.029 | 0.022 | 0.002 | 0.018 | 0.040 | 0.032 | -0.004 | 0.019 |
| rs984409 | 1 | 67020440 | G | A | 0.363 | 0.005 | 0.002 | 3.60E-03 | -0.015 | 0.002 | 4.10E-11 | TV | -0.015 | 0.006 | -0.019 | 0.008 | -0.006 | 0.019 | -0.032 | 0.016 | 0.022 | 0.027 | -0.025 | 0.016 |
| rs71658797 | 1 | 77967507 | T | A | 0.877 | 0.010 | 0.003 | 1.02E-04 | -0.020 | 0.003 | 6.30E-10 | TV | -0.004 | 0.010 | -0.003 | 0.013 | -0.034 | 0.029 | 0.059 | 0.025 | -0.038 | 0.042 | 0.002 | 0.025 |
| rs114328297 | 1 | 91190854 | T | G | 0.778 | -0.011 | 0.002 | 3.52E-07 | 0.014 | 0.003 | 4.40E-08 | TV | -0.006 | 0.008 | 0.001 | 0.011 | -0.021 | 0.023 | -0.019 | 0.020 | 0.053 | 0.034 | -0.033 | 0.020 |
| rs6690195 | 1 | 96221653 | T | C | 0.4907 | -0.011 | 0.001 | 7.89E-16 | 0.009 | 0.002 | 5.50E-05 | education | 0.010 | 0.006 | 0.002 | 0.008 | 0.042 | 0.018 | 0.007 | 0.015 | 0.004 | 0.026 | 0.014 | 0.015 |
| rs17379561 | 1 | 98340139 | A | T | 0.856 | 0.019 | 0.002 | 1.73E-15 | -0.025 | 0.003 | 1.10E-16 | TV | -0.002 | 0.009 | -0.001 | 0.011 | 0.052 | 0.025 | -0.029 | 0.021 | 0.010 | 0.036 | -0.008 | 0.021 |
| rs55909997 | 1 | 107581039 | G | A | 0.65 | 0.006 | 0.002 | 6.29E-04 | -0.014 | 0.002 | 2.40E-09 | TV | -0.002 | 0.006 | 0.002 | 0.008 | 0.016 | 0.019 | -0.024 | 0.016 | -0.005 | 0.027 | -0.024 | 0.016 |
| rs1278847 | 1 | 110037483 | C | A | 0.694 | -0.012 | 0.002 | 7.79E-11 | 0.016 | 0.002 | 1.20E-11 | TV | -0.012 | 0.007 | -0.016 | 0.009 | -0.008 | 0.020 | -0.004 | 0.017 | -0.017 | 0.028 | 0.011 | 0.017 |
| rs4839155 | 1 | 112161489 | T | G | 0.7671 | 0.012 | 0.002 | 4.66E-12 | -0.008 | 0.003 | 1.10E-03 | education | -0.005 | 0.007 | -0.011 | 0.009 | -0.005 | 0.021 | -0.025 | 0.018 | 0.037 | 0.031 | -0.019 | 0.018 |
| rs4845364 | 1 | 154141908 | A | G | 0.495 | 0.002 | 0.002 | 2.84E-01 | -0.015 | 0.002 | 1.30E-12 | TV | 0.005 | 0.006 | 0.007 | 0.008 | 0.002 | 0.018 | -0.002 | 0.015 | 0.018 | 0.026 | 0.020 | 0.015 |
| rs34720381 | 1 | 171455322 | T | C | 0.0907 | -0.018 | 0.002 | 1.90E-13 | 0.016 | 0.004 | 2.70E-05 | education | 0.006 | 0.011 | 0.021 | 0.014 | -0.025 | 0.032 | 0.011 | 0.027 | -0.006 | 0.046 | 0.002 | 0.027 |
| rs6673341 | 1 | 184655649 | T | G | 0.465 | 0.007 | 0.002 | 1.51E-05 | -0.015 | 0.002 | 2.20E-11 | TV | 0.000 | 0.006 | -0.006 | 0.008 | -0.004 | 0.018 | 0.007 | 0.015 | -0.005 | 0.026 | -0.026 | 0.015 |
| rs10737620 | 1 | 193057971 | T | A | 0.274 | -0.007 | 0.002 | 3.12E-04 | 0.014 | 0.002 | 2.60E-09 | TV | 0.011 | 0.007 | 0.023 | 0.009 | 0.017 | 0.020 | 0.012 | 0.017 | 0.012 | 0.029 | -0.017 | 0.017 |
| rs1890132 | 1 | 199427321 | T | C | 0.7056 | -0.009 | 0.002 | 6.56E-09 | 0.003 | 0.002 | 2.60E-01 | education | 0.003 | 0.007 | 0.012 | 0.009 | -0.009 | 0.020 | -0.007 | 0.017 | 0.025 | 0.029 | 0.017 | 0.017 |
| rs8024 | 1 | 201845575 | A | C | 0.3294 | -0.012 | 0.002 | 1.13E-14 | 0.010 | 0.002 | 7.30E-06 | education | -0.006 | 0.006 | -0.006 | 0.009 | 0.007 | 0.019 | 0.025 | 0.016 | 0.034 | 0.027 | -0.018 | 0.016 |
| rs11588857 | 1 | 204587047 | A | G | 0.2131 | 0.020 | 0.002 | 4.45E-31 | -0.012 | 0.003 | 1.30E-05 | education | -0.011 | 0.007 | -0.002 | 0.010 | 0.003 | 0.021 | -0.012 | 0.018 | -0.061 | 0.031 | -0.061 | 0.019 |
| rs3111251 | 1 | 211409844 | T | C | 0.4325 | -0.009 | 0.001 | 3.88E-10 | 0.003 | 0.002 | 2.40E-01 | education | 0.003 | 0.006 | 0.001 | 0.008 | 0.039 | 0.018 | 0.001 | 0.015 | 0.007 | 0.026 | 0.009 | 0.016 |
| rs4846724 | 1 | 221967817 | A | G | 0.5347 | 0.010 | 0.001 | 2.89E-12 | -0.006 | 0.002 | 8.30E-03 | education | -0.001 | 0.007 | -0.001 | 0.009 | 0.014 | 0.019 | -0.005 | 0.016 | 0.023 | 0.027 | 0.003 | 0.016 |
| rs1329125 | 1 | 234740880 | T | C | 0.3273 | -0.010 | 0.002 | 8.37E-11 | 0.008 | 0.002 | 6.20E-04 | education | 0.016 | 0.007 | 0.014 | 0.009 | 0.035 | 0.019 | -0.010 | 0.017 | 0.014 | 0.028 | 0.029 | 0.017 |
| rs114593137 | 1 | 241876565 | A | T | 0.793 | -0.012 | 0.002 | 1.65E-12 | 0.014 | 0.003 | 3.20E-07 | education | -0.013 | 0.008 | -0.012 | 0.010 | -0.003 | 0.022 | 0.016 | 0.019 | -0.044 | 0.031 | -0.039 | 0.019 |
| rs3897821 | 1 | 243420388 | A | G | 0.6654 | 0.015 | 0.002 | 3.58E-25 | -0.007 | 0.002 | 2.00E-03 | education | -0.015 | 0.006 | -0.022 | 0.008 | -0.002 | 0.019 | -0.027 | 0.016 | -0.003 | 0.027 | -0.024 | 0.016 |
| rs12614263 | 2 | 4123797 | A | G | 0.4959 | 0.008 | 0.001 | 3.38E-09 | -0.004 | 0.002 | 8.60E-02 | education | 0.010 | 0.006 | 0.012 | 0.008 | -0.016 | 0.018 | 0.016 | 0.016 | -0.012 | 0.027 | 0.011 | 0.016 |
| rs7603132 | 2 | 4951548 | A | G | 0.1938 | 0.014 | 0.002 | 1.20E-14 | -0.009 | 0.003 | 1.00E-03 | education | -0.003 | 0.008 | 0.007 | 0.011 | 0.023 | 0.024 | 0.003 | 0.021 | -0.050 | 0.036 | 0.003 | 0.021 |
| rs76076331 | 2 | 10977585 | T | C | 0.1346 | 0.021 | 0.002 | 1.71E-23 | -0.015 | 0.003 | 6.60E-06 | education | 0.002 | 0.009 | 0.010 | 0.012 | 0.002 | 0.027 | 0.011 | 0.023 | 0.033 | 0.039 | -0.016 | 0.024 |
| rs1991585 | 2 | 16647170 | T | C | 0.706 | -0.010 | 0.002 | 6.26E-11 | 0.009 | 0.002 | 1.20E-04 | education | -0.014 | 0.007 | -0.010 | 0.009 | -0.023 | 0.019 | -0.028 | 0.016 | 0.016 | 0.028 | -0.015 | 0.017 |
| rs72781699 | 2 | 24277709 | G | A | 0.797 | 0.006 | 0.002 | 2.99E-03 | -0.019 | 0.003 | 3.00E-12 | TV | 0.011 | 0.008 | 0.007 | 0.010 | 0.017 | 0.022 | -0.015 | 0.019 | -0.007 | 0.032 | 0.043 | 0.019 |
| rs12105701 | 2 | 41737200 | C | T | 0.396 | 0.005 | 0.002 | 2.45E-03 | -0.013 | 0.002 | 5.40E-09 | TV | -0.003 | 0.006 | -0.004 | 0.008 | -0.020 | 0.018 | 0.043 | 0.016 | 0.026 | 0.027 | -0.027 | 0.016 |
| rs7564130 | 2 | 50606642 | T | C | 0.641 | 0.009 | 0.002 | 1.49E-06 | -0.015 | 0.002 | 2.80E-11 | TV | -0.003 | 0.006 | -0.010 | 0.008 | -0.016 | 0.018 | -0.005 | 0.016 | 0.005 | 0.027 | 0.006 | 0.016 |
| rs10189857 | 2 | 60713235 | A | G | 0.5654 | 0.016 | 0.001 | 4.69E-29 | -0.020 | 0.002 | 6.20E-21 | both | -0.017 | 0.006 | -0.020 | 0.008 | -0.011 | 0.018 | -0.022 | 0.015 | -0.009 | 0.026 | -0.015 | 0.015 |
| rs62641636 | 2 | 68425427 | A | G | 0.692 | -0.011 | 0.002 | 1.06E-09 | 0.014 | 0.002 | 7.00E-10 | TV | 0.001 | 0.007 | -0.003 | 0.009 | 0.039 | 0.019 | 0.009 | 0.017 | -0.042 | 0.028 | 0.016 | 0.017 |
| rs112806496 | 2 | 80421805 | C | G | 0.9129 | -0.016 | 0.003 | 1.72E-10 | 0.014 | 0.004 | 1.50E-04 | education | 0.006 | 0.012 | 0.012 | 0.016 | -0.077 | 0.034 | 0.006 | 0.030 | 0.075 | 0.052 | -0.030 | 0.029 |
| rs11894424 | 2 | 80588091 | A | C | 0.9524 | 0.021 | 0.003 | 2.14E-10 | -0.002 | 0.005 | 7.50E-01 | education | -0.006 | 0.014 | -0.008 | 0.019 | -0.039 | 0.040 | 0.063 | 0.036 | -0.039 | 0.058 | 0.004 | 0.036 |
| rs11689199 | 2 | 100820421 | A | G | 0.598 | -0.020 | 0.002 | 1.17E-29 | 0.018 | 0.002 | 5.50E-17 | TV | -0.010 | 0.006 | -0.008 | 0.008 | -0.017 | 0.018 | -0.007 | 0.016 | -0.014 | 0.027 | -0.017 | 0.016 |
| rs1451533 | 2 | 105466005 | G | A | 0.725 | 0.000 | 0.002 | 8.43E-01 | -0.016 | 0.002 | 1.10E-10 | TV | -0.004 | 0.007 | -0.015 | 0.010 | 0.032 | 0.021 | -0.001 | 0.018 | 0.018 | 0.031 | -0.005 | 0.018 |
| rs13402497 | 2 | 115826318 | A | T | 0.4902 | -0.008 | 0.001 | 1.33E-08 | -0.002 | 0.002 | 2.80E-01 | education | 0.004 | 0.006 | 0.006 | 0.008 | 0.002 | 0.018 | 0.008 | 0.015 | 0.022 | 0.026 | -0.004 | 0.015 |
| rs12476388 | 2 | 116330983 | C | T | 0.709 | 0.000 | 0.002 | 8.32E-01 | 0.013 | 0.002 | 2.10E-08 | TV | -0.002 | 0.007 | 0.002 | 0.009 | 0.009 | 0.019 | -0.004 | 0.017 | -0.035 | 0.028 | -0.014 | 0.017 |
| rs7575637 | 2 | 145796251 | A | G | 0.4538 | 0.011 | 0.001 | 3.43E-14 | -0.003 | 0.002 | 1.20E-01 | education | -0.016 | 0.006 | -0.021 | 0.008 | 0.009 | 0.018 | -0.021 | 0.015 | 0.024 | 0.026 | -0.020 | 0.015 |
| rs374722 | 2 | 147839830 | G | A | 0.15 | -0.006 | 0.002 | 8.51E-03 | 0.024 | 0.003 | 5.50E-16 | TV | 0.011 | 0.009 | 0.018 | 0.012 | 0.037 | 0.025 | -0.012 | 0.022 | -0.080 | 0.039 | 0.001 | 0.023 |
| rs3754970 | 2 | 162091836 | T | C | 0.496 | 0.016 | 0.002 | 3.53E-20 | -0.015 | 0.002 | 4.80E-12 | TV | 0.007 | 0.006 | 0.000 | 0.008 | -0.003 | 0.018 | 0.006 | 0.015 | -0.017 | 0.026 | 0.023 | 0.016 |
| rs2060199 | 2 | 166196681 | T | A | 0.446 | -0.003 | 0.002 | 6.82E-02 | 0.012 | 0.002 | 2.40E-08 | TV | -0.008 | 0.006 | -0.007 | 0.008 | 0.010 | 0.018 | 0.019 | 0.016 | 0.011 | 0.026 | -0.021 | 0.016 |
| rs17428076 | 2 | 172851936 | C | G | 0.7598 | 0.014 | 0.002 | 6.41E-17 | -0.007 | 0.003 | 5.40E-03 | education | 0.004 | 0.007 | 0.011 | 0.009 | -0.037 | 0.020 | 0.012 | 0.018 | 0.051 | 0.030 | -0.012 | 0.018 |
| rs10193498 | 2 | 174094345 | A | T | 0.755 | 0.010 | 0.002 | 3.10E-09 | -0.007 | 0.003 | 5.00E-03 | education | -0.009 | 0.007 | -0.012 | 0.009 | 0.004 | 0.020 | 0.000 | 0.017 | 0.000 | 0.029 | -0.019 | 0.017 |
| rs72917504 | 2 | 175225018 | T | C | 0.0583 | -0.018 | 0.003 | 2.91E-09 | 0.007 | 0.005 | 1.10E-01 | education | 0.012 | 0.015 | 0.013 | 0.020 | 0.044 | 0.041 | 0.055 | 0.036 | 0.038 | 0.061 | 0.039 | 0.037 |
| rs4577309 | 2 | 191288833 | A | G | 0.469 | -0.005 | 0.002 | 3.33E-03 | 0.016 | 0.002 | 1.60E-13 | TV | -0.001 | 0.006 | 0.004 | 0.008 | -0.014 | 0.018 | 0.008 | 0.015 | -0.006 | 0.026 | -0.015 | 0.015 |
| rs34224261 | 2 | 193855452 | G | C | 0.469 | 0.011 | 0.002 | 3.79E-11 | -0.018 | 0.002 | 4.60E-16 | TV | 0.000 | 0.006 | -0.006 | 0.008 | 0.004 | 0.018 | -0.026 | 0.015 | -0.060 | 0.026 | -0.002 | 0.015 |
| rs4675246 | 2 | 202864487 | G | T | 0.797 | 0.009 | 0.002 | 5.48E-05 | -0.015 | 0.003 | 2.90E-08 | TV | 0.006 | 0.008 | 0.013 | 0.010 | -0.005 | 0.022 | 0.038 | 0.019 | -0.077 | 0.032 | -0.040 | 0.019 |
| rs13029509 | 2 | 215374209 | G | A | 0.52 | 0.010 | 0.002 | 7.17E-10 | -0.018 | 0.002 | 2.00E-17 | TV | -0.009 | 0.006 | -0.008 | 0.008 | 0.008 | 0.018 | -0.019 | 0.015 | 0.018 | 0.026 | -0.017 | 0.015 |
| rs10932837 | 2 | 221044785 | C | T | 0.494 | 0.002 | 0.002 | 1.52E-01 | -0.013 | 0.002 | 1.30E-09 | TV | 0.001 | 0.006 | 0.003 | 0.008 | -0.011 | 0.018 | 0.009 | 0.015 | -0.023 | 0.026 | 0.000 | 0.015 |
| rs1542354 | 2 | 221814085 | A | G | 0.4764 | -0.008 | 0.001 | 2.72E-09 | 0.005 | 0.002 | 1.20E-02 | education | -0.007 | 0.006 | -0.006 | 0.008 | 0.017 | 0.018 | 0.014 | 0.016 | 0.012 | 0.026 | -0.041 | 0.016 |
| rs4973576 | 2 | 233806771 | C | A | 0.298 | 0.008 | 0.002 | 7.60E-06 | -0.015 | 0.002 | 1.00E-09 | TV | 0.012 | 0.007 | 0.022 | 0.009 | 0.031 | 0.020 | -0.015 | 0.017 | 0.018 | 0.029 | 0.026 | 0.017 |
| rs4663617 | 2 | 236744626 | A | T | 0.235 | 0.010 | 0.002 | 4.12E-10 | -0.007 | 0.003 | 4.30E-03 | education | 0.007 | 0.008 | 0.007 | 0.010 | 0.017 | 0.022 | -0.008 | 0.019 | 0.048 | 0.032 | -0.002 | 0.019 |
| rs72993796 | 2 | 240321051 | T | C | 0.8836 | 0.013 | 0.002 | 1.12E-08 | -0.005 | 0.003 | 1.10E-01 | education | 0.002 | 0.010 | -0.003 | 0.014 | 0.032 | 0.031 | 0.005 | 0.026 | -0.028 | 0.043 | -0.015 | 0.026 |
| rs17048855 | 3 | 8258174 | A | G | 0.3426 | 0.011 | 0.002 | 3.10E-13 | -0.004 | 0.002 | 6.20E-02 | education | -0.008 | 0.006 | -0.006 | 0.008 | -0.029 | 0.019 | -0.014 | 0.016 | -0.010 | 0.028 | -0.014 | 0.016 |
| rs138256022 | 3 | 11603090 | C | T | 0.959 | 0.011 | 0.004 | 1.06E-02 | -0.031 | 0.006 | 2.90E-08 | TV | -0.016 | 0.017 | -0.025 | 0.023 | -0.024 | 0.050 | -0.043 | 0.043 | -0.062 | 0.072 | -0.005 | 0.044 |
| rs13085461 | 3 | 24950387 | C | G | 0.474 | 0.010 | 0.001 | 2.40E-13 | -0.003 | 0.002 | 2.00E-01 | education | -0.005 | 0.006 | -0.007 | 0.008 | 0.034 | 0.018 | 0.012 | 0.015 | -0.009 | 0.026 | -0.012 | 0.015 |
| rs73055556 | 3 | 28027538 | A | G | 0.1385 | 0.011 | 0.002 | 4.95E-08 | -0.005 | 0.003 | 1.10E-01 | education | -0.016 | 0.009 | -0.008 | 0.012 | -0.039 | 0.027 | -0.012 | 0.023 | 0.006 | 0.039 | 0.021 | 0.023 |
| rs9834970 | 3 | 36856030 | T | C | 0.501 | -0.008 | 0.002 | 7.65E-07 | 0.013 | 0.002 | 3.30E-09 | TV | 0.001 | 0.006 | 0.001 | 0.008 | 0.005 | 0.018 | 0.004 | 0.016 | -0.035 | 0.027 | 0.011 | 0.016 |
| rs3796386 | 3 | 49899795 | G | A | 0.572 | 0.026 | 0.002 | 7.19E-51 | -0.026 | 0.002 | 3.20E-33 | TV | -0.018 | 0.006 | -0.014 | 0.008 | -0.038 | 0.018 | -0.013 | 0.015 | -0.063 | 0.026 | -0.043 | 0.015 |
| rs11130793 | 3 | 60884659 | C | T | 0.602 | -0.005 | 0.002 | 7.56E-03 | 0.013 | 0.002 | 5.00E-09 | TV | 0.001 | 0.006 | 0.006 | 0.008 | 0.012 | 0.018 | -0.021 | 0.016 | 0.004 | 0.027 | 0.002 | 0.016 |
| rs11714337 | 3 | 71582521 | G | A | 0.569 | -0.013 | 0.002 | 7.66E-15 | 0.014 | 0.002 | 4.70E-11 | TV | 0.032 | 0.006 | 0.046 | 0.008 | 0.036 | 0.018 | 0.029 | 0.015 | 0.000 | 0.026 | 0.014 | 0.015 |
| rs6797840 | 3 | 85656569 | A | C | 0.456 | 0.010 | 0.002 | 1.29E-08 | -0.016 | 0.002 | 1.70E-13 | TV | -0.005 | 0.006 | -0.007 | 0.008 | -0.015 | 0.018 | -0.022 | 0.015 | 0.024 | 0.026 | 0.006 | 0.015 |
| rs2034768 | 3 | 93986371 | A | G | 0.487 | -0.006 | 0.002 | 2.08E-04 | 0.015 | 0.002 | 8.60E-12 | TV | -0.003 | 0.006 | -0.006 | 0.008 | 0.010 | 0.018 | 0.016 | 0.015 | 0.016 | 0.025 | -0.025 | 0.015 |
| rs9867121 | 3 | 114631548 | C | A | 0.818 | -0.004 | 0.002 | 9.97E-02 | 0.019 | 0.003 | 3.90E-12 | TV | 0.013 | 0.008 | 0.015 | 0.010 | 0.020 | 0.023 | 0.001 | 0.020 | 0.052 | 0.034 | 0.009 | 0.020 |
| rs34067381 | 3 | 123594419 | T | G | 0.3617 | -0.010 | 0.002 | 9.77E-13 | 0.011 | 0.002 | 2.30E-06 | education | 0.007 | 0.006 | 0.002 | 0.008 | -0.016 | 0.019 | 0.000 | 0.016 | 0.024 | 0.027 | 0.042 | 0.016 |
| rs9289300 | 3 | 127144988 | T | C | 0.8412 | -0.016 | 0.002 | 1.00E-17 | 0.006 | 0.003 | 3.40E-02 | education | 0.005 | 0.008 | -0.004 | 0.011 | 0.060 | 0.025 | -0.001 | 0.021 | 0.000 | 0.036 | -0.001 | 0.021 |
| rs7650602 | 3 | 141147414 | T | C | 0.5599 | -0.009 | 0.001 | 2.77E-10 | 0.006 | 0.002 | 7.40E-03 | education | -0.048 | 0.006 | -0.058 | 0.008 | -0.052 | 0.018 | -0.079 | 0.015 | -0.079 | 0.026 | -0.011 | 0.015 |
| rs2885198 | 3 | 143636421 | A | G | 0.5318 | 0.009 | 0.001 | 4.63E-10 | -0.009 | 0.002 | 1.40E-05 | education | -0.009 | 0.006 | -0.012 | 0.008 | 0.014 | 0.019 | -0.016 | 0.016 | -0.076 | 0.027 | -0.017 | 0.016 |
| rs12054166 | 3 | 150093445 | C | G | 0.7399 | 0.010 | 0.002 | 2.30E-09 | 0.004 | 0.002 | 1.30E-01 | education | 0.010 | 0.007 | 0.012 | 0.010 | 0.016 | 0.021 | 0.037 | 0.019 | 0.024 | 0.031 | -0.016 | 0.019 |
| rs12491503 | 3 | 165706855 | G | A | 0.67 | 0.007 | 0.002 | 1.41E-04 | -0.014 | 0.002 | 5.50E-10 | TV | -0.001 | 0.007 | 0.008 | 0.009 | -0.018 | 0.019 | -0.029 | 0.016 | -0.025 | 0.028 | 0.009 | 0.017 |
| rs77025239 | 3 | 180734185 | A | G | 0.1559 | -0.014 | 0.002 | 4.65E-13 | 0.004 | 0.003 | 1.60E-01 | education | 0.010 | 0.008 | 0.027 | 0.011 | -0.008 | 0.025 | -0.007 | 0.021 | 0.022 | 0.035 | -0.014 | 0.021 |
| rs114600294 | 3 | 181419367 | G | C | 0.789 | 0.011 | 0.002 | 3.21E-06 | -0.016 | 0.003 | 7.90E-10 | TV | 0.018 | 0.010 | 0.002 | 0.013 | 0.029 | 0.028 | -0.015 | 0.024 | 0.035 | 0.042 | 0.007 | 0.025 |
| rs13327482 | 3 | 196788363 | A | G | 0.8221 | -0.012 | 0.002 | 4.47E-11 | 0.005 | 0.003 | 9.60E-02 | education | 0.011 | 0.008 | 0.011 | 0.010 | 0.034 | 0.023 | -0.024 | 0.019 | -0.045 | 0.032 | 0.034 | 0.020 |
| rs66852340 | 4 | 3311070 | C | T | 0.776 | 0.013 | 0.002 | 1.94E-10 | -0.018 | 0.003 | 7.90E-12 | TV | -0.016 | 0.007 | -0.019 | 0.010 | 0.027 | 0.021 | -0.046 | 0.018 | -0.011 | 0.031 | -0.022 | 0.018 |
| rs13133213 | 4 | 15646794 | A | G | 0.498 | 0.010 | 0.001 | 7.57E-12 | 0.005 | 0.002 | 1.20E-02 | education | -0.010 | 0.006 | -0.007 | 0.008 | -0.015 | 0.018 | 0.000 | 0.015 | -0.030 | 0.025 | -0.002 | 0.015 |
| rs7700107 | 4 | 17880416 | A | C | 0.863 | 0.007 | 0.003 | 5.27E-03 | -0.021 | 0.003 | 1.90E-11 | TV | 0.005 | 0.009 | 0.033 | 0.012 | -0.019 | 0.025 | -0.026 | 0.022 | -0.067 | 0.036 | -0.009 | 0.022 |
| rs10021733 | 4 | 28609476 | T | C | 0.1475 | -0.016 | 0.003 | 9.45E-10 | -0.001 | 0.003 | 6.10E-01 | education | -0.007 | 0.009 | -0.021 | 0.011 | 0.016 | 0.025 | -0.016 | 0.022 | 0.018 | 0.036 | 0.000 | 0.022 |
| rs77215114 | 4 | 45187658 | A | T | 0.93 | -0.008 | 0.003 | 1.74E-02 | 0.024 | 0.004 | 1.50E-08 | TV | -0.016 | 0.013 | -0.022 | 0.018 | -0.019 | 0.039 | 0.021 | 0.034 | 0.022 | 0.057 | -0.051 | 0.033 |
| rs1505676 | 4 | 62127291 | C | G | 0.6293 | 0.008 | 0.002 | 6.67E-09 | -0.008 | 0.002 | 4.20E-04 | education | -0.002 | 0.007 | -0.002 | 0.009 | 0.005 | 0.019 | -0.025 | 0.016 | -0.011 | 0.028 | -0.039 | 0.017 |
| rs12506221 | 4 | 67899235 | T | G | 0.436 | -0.013 | 0.001 | 4.23E-20 | 0.004 | 0.002 | 8.40E-02 | education | -0.011 | 0.006 | -0.004 | 0.008 | 0.016 | 0.018 | -0.003 | 0.015 | 0.018 | 0.026 | -0.019 | 0.015 |
| rs6850494 | 4 | 82291771 | A | C | 0.616 | 0.005 | 0.002 | 8.27E-03 | -0.014 | 0.002 | 1.10E-10 | TV | 0.009 | 0.006 | 0.004 | 0.008 | 0.030 | 0.018 | 0.002 | 0.016 | -0.045 | 0.026 | 0.021 | 0.016 |
| rs13107325 | 4 | 103188709 | C | T | 0.926 | 0.019 | 0.003 | 4.45E-08 | -0.029 | 0.004 | 1.50E-12 | TV | -0.019 | 0.012 | -0.021 | 0.016 | -0.051 | 0.034 | -0.007 | 0.030 | 0.051 | 0.051 | -0.025 | 0.030 |
| rs7693703 | 4 | 118357405 | G | A | 0.91 | -0.008 | 0.003 | 5.22E-03 | 0.023 | 0.004 | 2.70E-09 | TV | -0.007 | 0.010 | -0.016 | 0.014 | -0.012 | 0.030 | 0.009 | 0.026 | 0.052 | 0.046 | 0.005 | 0.027 |
| rs4334769 | 4 | 130245233 | G | T | 0.474 | 0.000 | 0.002 | 8.58E-01 | 0.012 | 0.002 | 2.10E-08 | TV | -0.009 | 0.006 | -0.007 | 0.008 | -0.016 | 0.018 | -0.032 | 0.015 | 0.012 | 0.026 | 0.015 | 0.015 |
| rs10006235 | 4 | 130670108 | T | C | 0.2689 | -0.009 | 0.002 | 4.48E-09 | 0.004 | 0.002 | 7.20E-02 | education | 0.006 | 0.007 | 0.002 | 0.009 | 0.009 | 0.020 | 0.017 | 0.017 | 0.078 | 0.028 | 0.018 | 0.017 |
| rs12643771 | 4 | 140753103 | T | C | 0.3094 | 0.013 | 0.002 | 1.32E-17 | -0.009 | 0.002 | 1.30E-04 | education | -0.009 | 0.007 | 0.003 | 0.009 | -0.007 | 0.020 | -0.024 | 0.018 | 0.007 | 0.030 | -0.043 | 0.018 |
| rs6825241 | 4 | 152543783 | C | A | 0.536 | 0.009 | 0.002 | 2.82E-07 | -0.017 | 0.002 | 4.90E-15 | TV | 0.002 | 0.006 | 0.009 | 0.008 | -0.008 | 0.018 | -0.018 | 0.016 | 0.008 | 0.027 | 0.006 | 0.016 |
| rs7693082 | 4 | 159857819 | G | C | 0.3 | -0.013 | 0.002 | 4.05E-13 | 0.015 | 0.002 | 1.70E-10 | TV | -0.004 | 0.007 | -0.014 | 0.009 | -0.023 | 0.020 | -0.001 | 0.017 | 0.044 | 0.028 | -0.013 | 0.017 |
| rs12646523 | 4 | 170944698 | T | C | 0.2483 | -0.013 | 0.002 | 3.79E-16 | 0.005 | 0.002 | 3.10E-02 | education | -0.002 | 0.007 | -0.004 | 0.009 | 0.002 | 0.021 | 0.004 | 0.018 | 0.035 | 0.030 | 0.008 | 0.018 |
| rs17598675 | 4 | 176647637 | T | C | 0.5132 | -0.012 | 0.001 | 1.37E-16 | 0.002 | 0.002 | 4.90E-01 | education | -0.003 | 0.007 | 0.003 | 0.009 | -0.002 | 0.019 | -0.002 | 0.016 | 0.015 | 0.028 | -0.005 | 0.017 |
| rs72709560 | 4 | 178540163 | A | G | 0.6865 | 0.009 | 0.002 | 2.03E-08 | -0.005 | 0.002 | 1.70E-02 | education | 0.001 | 0.007 | 0.010 | 0.009 | 0.007 | 0.020 | -0.011 | 0.017 | -0.054 | 0.028 | 0.015 | 0.017 |
| rs1128956 | 4 | 183724005 | T | G | 0.8251 | -0.014 | 0.002 | 5.34E-14 | 0.010 | 0.003 | 3.00E-04 | education | 0.004 | 0.009 | 0.008 | 0.012 | -0.003 | 0.025 | -0.010 | 0.022 | -0.026 | 0.036 | 0.019 | 0.022 |
| rs11724690 | 4 | 186764747 | T | G | 0.2887 | 0.009 | 0.002 | 2.08E-09 | -0.006 | 0.002 | 2.10E-02 | education | 0.001 | 0.007 | 0.010 | 0.010 | -0.023 | 0.021 | 0.003 | 0.018 | 0.012 | 0.030 | 0.012 | 0.018 |
| rs13163845 | 5 | 3264389 | T | C | 0.8488 | -0.015 | 0.002 | 4.66E-14 | 0.007 | 0.003 | 2.30E-02 | education | -0.012 | 0.009 | -0.014 | 0.012 | 0.000 | 0.026 | 0.008 | 0.022 | -0.131 | 0.036 | -0.027 | 0.022 |
| rs162445 | 5 | 7943246 | A | G | 0.0788 | 0.015 | 0.003 | 5.72E-09 | -0.007 | 0.004 | 1.00E-01 | education | 0.008 | 0.011 | 0.007 | 0.015 | 0.031 | 0.033 | 0.009 | 0.028 | 0.016 | 0.048 | 0.017 | 0.028 |
| rs2434672 | 5 | 11471879 | A | C | 0.5321 | 0.009 | 0.001 | 1.06E-10 | -0.004 | 0.002 | 4.40E-02 | education | 0.003 | 0.006 | 0.003 | 0.008 | -0.005 | 0.018 | -0.015 | 0.015 | 0.017 | 0.026 | -0.003 | 0.016 |
| rs17563464 | 5 | 26913774 | A | C | 0.2173 | -0.015 | 0.002 | 1.74E-17 | 0.006 | 0.003 | 2.20E-02 | education | 0.002 | 0.008 | -0.005 | 0.011 | -0.048 | 0.024 | 0.045 | 0.020 | -0.032 | 0.035 | 0.031 | 0.021 |
| rs10940921 | 5 | 30808645 | T | G | 0.4194 | 0.011 | 0.001 | 1.31E-14 | -0.009 | 0.002 | 3.40E-05 | education | 0.006 | 0.006 | 0.005 | 0.008 | 0.004 | 0.018 | 0.027 | 0.015 | -0.030 | 0.026 | 0.011 | 0.016 |
| rs2964199 | 5 | 57532775 | T | C | 0.3149 | -0.010 | 0.002 | 4.61E-11 | 0.008 | 0.002 | 3.40E-04 | education | 0.005 | 0.007 | -0.014 | 0.009 | 0.032 | 0.019 | -0.006 | 0.016 | 0.122 | 0.027 | 0.025 | 0.016 |
| rs6449503 | 5 | 60095272 | A | G | 0.5038 | 0.019 | 0.001 | 1.84E-39 | -0.015 | 0.002 | 1.40E-11 | education | -0.004 | 0.006 | -0.006 | 0.008 | -0.001 | 0.018 | -0.019 | 0.015 | -0.010 | 0.026 | 0.004 | 0.016 |
| rs262890 | 5 | 62930015 | A | G | 0.699 | 0.010 | 0.002 | 8.30E-08 | -0.019 | 0.002 | 3.20E-15 | TV | -0.003 | 0.007 | -0.002 | 0.009 | 0.032 | 0.019 | -0.006 | 0.016 | -0.021 | 0.028 | -0.008 | 0.017 |
| rs42302 | 5 | 74965554 | A | G | 0.3441 | 0.009 | 0.002 | 5.96E-09 | -0.008 | 0.002 | 4.00E-04 | education | 0.017 | 0.006 | 0.013 | 0.008 | -0.017 | 0.019 | 0.021 | 0.016 | 0.048 | 0.027 | 0.014 | 0.016 |
| rs7716447 | 5 | 88800331 | A | G | 0.639 | 0.006 | 0.002 | 2.78E-04 | -0.013 | 0.002 | 3.60E-09 | TV | 0.007 | 0.007 | 0.009 | 0.009 | -0.013 | 0.019 | 0.011 | 0.016 | 0.034 | 0.028 | -0.011 | 0.017 |
| rs10046069 | 5 | 89115576 | A | G | 0.1203 | 0.013 | 0.002 | 4.40E-09 | -0.009 | 0.003 | 1.10E-02 | education | 0.006 | 0.009 | 0.005 | 0.012 | -0.012 | 0.027 | -0.005 | 0.023 | 0.053 | 0.038 | -0.013 | 0.024 |
| rs1031423 | 5 | 93276883 | T | C | 0.215 | 0.012 | 0.002 | 4.11E-09 | -0.019 | 0.003 | 1.80E-12 | TV | -0.002 | 0.008 | -0.007 | 0.010 | 0.013 | 0.022 | 0.013 | 0.019 | -0.010 | 0.032 | 0.021 | 0.019 |
| rs252991 | 5 | 106767346 | A | G | 0.3695 | 0.010 | 0.002 | 6.30E-11 | -0.010 | 0.002 | 1.40E-05 | education | 0.008 | 0.006 | 0.008 | 0.008 | 0.008 | 0.018 | 0.021 | 0.016 | 0.015 | 0.027 | -0.004 | 0.016 |
| rs57585211 | 5 | 107197725 | T | G | 0.827 | 0.010 | 0.002 | 7.87E-06 | -0.017 | 0.003 | 4.30E-09 | TV | 0.001 | 0.008 | 0.000 | 0.011 | 0.021 | 0.024 | -0.036 | 0.020 | 0.044 | 0.035 | -0.026 | 0.020 |
| rs17489649 | 5 | 109156184 | A | G | 0.6788 | 0.013 | 0.002 | 2.77E-17 | 0.000 | 0.002 | 9.90E-01 | education | 0.007 | 0.007 | -0.001 | 0.009 | 0.009 | 0.019 | 0.018 | 0.017 | 0.034 | 0.028 | 0.023 | 0.017 |
| rs9326862 | 5 | 112054547 | A | T | 0.468 | -0.003 | 0.002 | 1.36E-01 | 0.012 | 0.002 | 2.60E-08 | TV | 0.005 | 0.006 | 0.008 | 0.008 | 0.008 | 0.018 | -0.002 | 0.015 | 0.025 | 0.026 | 0.005 | 0.015 |
| rs10041724 | 5 | 124273520 | T | C | 0.808 | -0.013 | 0.002 | 1.58E-09 | 0.018 | 0.003 | 3.90E-11 | TV | 0.008 | 0.008 | 0.006 | 0.010 | 0.020 | 0.022 | -0.011 | 0.019 | 0.065 | 0.033 | -0.021 | 0.019 |
| rs12519073 | 5 | 136776762 | T | C | 0.2317 | -0.011 | 0.002 | 1.16E-10 | 0.006 | 0.003 | 3.20E-02 | education | 0.008 | 0.007 | 0.019 | 0.010 | 0.010 | 0.021 | 0.026 | 0.018 | 0.061 | 0.030 | 0.001 | 0.018 |
| rs62379379 | 5 | 141082015 | G | T | 0.929 | 0.015 | 0.003 | 1.86E-06 | -0.026 | 0.004 | 7.80E-10 | TV | -0.001 | 0.012 | -0.014 | 0.016 | 0.074 | 0.037 | 0.019 | 0.031 | 0.122 | 0.055 | -0.031 | 0.030 |
| rs10054327 | 5 | 147871137 | G | A | 0.576 | -0.004 | 0.002 | 3.91E-02 | 0.017 | 0.002 | 3.40E-15 | TV | -0.010 | 0.006 | -0.013 | 0.008 | 0.000 | 0.018 | 0.004 | 0.015 | 0.003 | 0.026 | -0.022 | 0.016 |
| rs2964255 | 5 | 152052332 | A | G | 0.3066 | 0.009 | 0.002 | 6.34E-09 | -0.012 | 0.002 | 1.40E-07 | education | -0.002 | 0.007 | 0.001 | 0.009 | 0.028 | 0.019 | -0.014 | 0.017 | -0.031 | 0.028 | -0.026 | 0.017 |
| rs182902112 | 5 | 153304758 | A | C | 0.0186 | -0.030 | 0.005 | 1.17E-08 | 0.025 | 0.008 | 1.90E-03 | education | -0.008 | 0.028 | 0.010 | 0.036 | -0.039 | 0.082 | -0.027 | 0.069 | -0.197 | 0.126 | 0.082 | 0.067 |
| rs42210 | 5 | 166408788 | C | G | 0.7122 | -0.010 | 0.002 | 2.74E-10 | 0.014 | 0.002 | 7.30E-09 | both | 0.020 | 0.007 | 0.008 | 0.010 | 0.046 | 0.021 | 0.046 | 0.018 | 0.083 | 0.031 | 0.019 | 0.018 |
| rs72828890 | 5 | 167806836 | C | T | 0.869 | -0.006 | 0.003 | 1.73E-02 | 0.019 | 0.003 | 4.90E-09 | TV | -0.005 | 0.011 | 0.006 | 0.014 | -0.010 | 0.032 | 0.027 | 0.028 | 0.002 | 0.047 | 0.012 | 0.028 |
| rs12524795 | 6 | 3464074 | T | C | 0.4374 | 0.010 | 0.001 | 5.28E-13 | -0.006 | 0.002 | 4.50E-03 | education | -0.003 | 0.007 | -0.010 | 0.009 | 0.002 | 0.019 | -0.001 | 0.016 | -0.003 | 0.027 | -0.029 | 0.017 |
| rs112603734 | 6 | 6069588 | A | C | 0.7699 | -0.013 | 0.002 | 2.72E-09 | 0.000 | 0.003 | 8.60E-01 | education | 0.005 | 0.007 | 0.003 | 0.010 | -0.004 | 0.021 | -0.010 | 0.018 | 0.001 | 0.031 | 0.042 | 0.019 |
| rs72829857 | 6 | 16966052 | A | G | 0.761 | -0.015 | 0.002 | 1.52E-19 | 0.011 | 0.003 | 1.40E-05 | education | -0.022 | 0.007 | -0.020 | 0.009 | -0.034 | 0.021 | -0.028 | 0.018 | -0.029 | 0.030 | -0.022 | 0.018 |
| rs72834698 | 6 | 26176517 | G | A | 0.858 | -0.014 | 0.002 | 2.15E-08 | 0.023 | 0.003 | 2.70E-13 | TV | 0.007 | 0.009 | 0.019 | 0.012 | -0.001 | 0.026 | -0.022 | 0.022 | 0.030 | 0.038 | 0.019 | 0.023 |
| rs57349798 | 6 | 37486052 | A | G | 0.4094 | 0.009 | 0.001 | 8.63E-11 | -0.006 | 0.002 | 4.20E-03 | education | -0.005 | 0.006 | -0.005 | 0.008 | 0.018 | 0.018 | -0.005 | 0.016 | -0.012 | 0.027 | -0.011 | 0.016 |
| rs9471333 | 6 | 40362023 | C | T | 0.45 | -0.009 | 0.002 | 3.40E-07 | 0.013 | 0.002 | 1.50E-09 | TV | -0.001 | 0.006 | 0.012 | 0.009 | -0.008 | 0.019 | -0.039 | 0.016 | -0.044 | 0.027 | 0.011 | 0.016 |
| rs78648104 | 6 | 50683009 | T | C | 0.9137 | -0.014 | 0.003 | 1.70E-08 | 0.005 | 0.004 | 2.30E-01 | education | -0.024 | 0.011 | -0.021 | 0.014 | -0.014 | 0.031 | 0.010 | 0.027 | -0.080 | 0.044 | -0.032 | 0.027 |
| rs7449561 | 6 | 66215549 | A | G | 0.226 | 0.010 | 0.002 | 9.73E-10 | -0.006 | 0.003 | 1.40E-02 | education | 0.006 | 0.008 | 0.006 | 0.010 | -0.002 | 0.022 | 0.006 | 0.019 | 0.021 | 0.031 | 0.003 | 0.019 |
| rs4523073 | 6 | 67550288 | A | G | 0.591 | 0.002 | 0.002 | 1.82E-01 | -0.014 | 0.002 | 5.00E-10 | TV | -0.002 | 0.006 | -0.004 | 0.008 | 0.025 | 0.018 | -0.006 | 0.016 | -0.003 | 0.027 | 0.008 | 0.016 |
| rs13212041 | 6 | 78171124 | T | C | 0.7978 | 0.010 | 0.002 | 7.42E-09 | -0.002 | 0.003 | 5.10E-01 | education | 0.015 | 0.008 | 0.019 | 0.010 | 0.001 | 0.022 | -0.011 | 0.019 | 0.025 | 0.033 | -0.013 | 0.019 |
| rs4352658 | 6 | 88279872 | T | C | 0.081 | -0.019 | 0.003 | 1.71E-13 | 0.015 | 0.004 | 2.20E-04 | education | 0.006 | 0.011 | 0.003 | 0.015 | -0.019 | 0.033 | 0.001 | 0.028 | 0.121 | 0.045 | 0.008 | 0.028 |
| rs6905544 | 6 | 98411631 | A | G | 0.399 | 0.023 | 0.002 | 7.33E-41 | -0.019 | 0.002 | 8.50E-18 | TV | -0.011 | 0.006 | -0.016 | 0.008 | -0.007 | 0.018 | -0.012 | 0.015 | 0.005 | 0.026 | -0.012 | 0.016 |
| rs17789218 | 6 | 100600097 | T | C | 0.756 | -0.001 | 0.002 | 6.08E-01 | 0.019 | 0.003 | 1.40E-13 | TV | -0.013 | 0.008 | -0.025 | 0.011 | -0.002 | 0.023 | 0.030 | 0.020 | 0.068 | 0.035 | 0.014 | 0.021 |
| rs10080647 | 6 | 114742335 | A | C | 0.1405 | 0.012 | 0.002 | 7.05E-10 | -0.006 | 0.003 | 4.10E-02 | education | -0.007 | 0.009 | -0.001 | 0.011 | 0.041 | 0.025 | 0.019 | 0.021 | -0.003 | 0.037 | -0.018 | 0.022 |
| rs11542663 | 6 | 119215402 | A | C | 0.6947 | 0.011 | 0.002 | 2.44E-13 | -0.005 | 0.002 | 2.80E-02 | education | 0.004 | 0.007 | -0.008 | 0.009 | 0.049 | 0.020 | -0.001 | 0.017 | 0.006 | 0.028 | -0.010 | 0.017 |
| rs13197257 | 6 | 128333682 | T | G | 0.279 | 0.011 | 0.002 | 2.83E-12 | 0.002 | 0.002 | 4.70E-01 | education | -0.014 | 0.007 | -0.014 | 0.010 | -0.043 | 0.021 | -0.056 | 0.018 | -0.034 | 0.031 | -0.007 | 0.018 |
| rs9492774 | 6 | 131302489 | C | G | 0.3692 | 0.008 | 0.002 | 4.33E-08 | -0.004 | 0.002 | 8.10E-02 | education | -0.007 | 0.006 | -0.002 | 0.008 | -0.022 | 0.018 | -0.007 | 0.016 | -0.051 | 0.027 | -0.020 | 0.016 |
| rs2184364 | 6 | 143183596 | A | G | 0.782 | -0.009 | 0.002 | 2.05E-05 | 0.016 | 0.003 | 3.00E-09 | TV | 0.007 | 0.007 | 0.009 | 0.010 | 0.009 | 0.022 | -0.004 | 0.018 | -0.035 | 0.031 | 0.023 | 0.019 |
| rs4895650 | 6 | 145024122 | T | C | 0.5472 | 0.009 | 0.001 | 5.31E-11 | -0.002 | 0.002 | 4.20E-01 | education | 0.003 | 0.006 | -0.008 | 0.008 | 0.036 | 0.018 | -0.006 | 0.015 | 0.018 | 0.026 | 0.008 | 0.016 |
| rs1933264 | 6 | 153391618 | T | C | 0.7491 | 0.010 | 0.002 | 5.85E-10 | -0.010 | 0.003 | 5.60E-05 | education | 0.001 | 0.007 | 0.003 | 0.009 | 0.005 | 0.020 | -0.029 | 0.017 | -0.012 | 0.029 | 0.012 | 0.018 |
| rs9718104 | 6 | 166170539 | T | G | 0.942 | -0.003 | 0.004 | 4.30E-01 | -0.041 | 0.005 | 9.30E-19 | TV | -0.010 | 0.014 | -0.009 | 0.018 | 0.009 | 0.041 | 0.004 | 0.034 | 0.041 | 0.060 | -0.054 | 0.034 |
| rs6917154 | 6 | 167135156 | T | C | 0.1236 | 0.013 | 0.002 | 2.55E-09 | -0.017 | 0.003 | 1.90E-07 | education | -0.002 | 0.010 | -0.010 | 0.014 | -0.026 | 0.030 | 0.002 | 0.026 | -0.051 | 0.044 | 0.002 | 0.026 |
| rs6924023 | 6 | 170076041 | A | G | 0.7548 | 0.011 | 0.002 | 5.17E-11 | -0.009 | 0.003 | 6.60E-04 | education | 0.005 | 0.007 | -0.001 | 0.010 | 0.033 | 0.021 | -0.008 | 0.018 | 0.041 | 0.031 | 0.018 | 0.018 |
| rs17568389 | 7 | 8091876 | T | A | 0.487 | -0.012 | 0.002 | 1.53E-12 | 0.015 | 0.002 | 8.60E-13 | TV | -0.003 | 0.006 | -0.008 | 0.008 | -0.012 | 0.018 | -0.014 | 0.015 | 0.038 | 0.026 | 0.011 | 0.016 |
| rs118134876 | 7 | 11500372 | T | C | 0.0578 | -0.022 | 0.003 | 1.50E-13 | 0.010 | 0.005 | 3.60E-02 | education | 0.009 | 0.013 | 0.020 | 0.018 | 0.009 | 0.039 | -0.056 | 0.034 | 0.064 | 0.055 | 0.076 | 0.033 |
| rs35929923 | 7 | 21400525 | A | G | 0.2495 | -0.011 | 0.002 | 1.77E-12 | 0.008 | 0.002 | 1.00E-03 | education | 0.004 | 0.007 | 0.001 | 0.009 | 0.015 | 0.020 | 0.004 | 0.017 | -0.022 | 0.030 | -0.001 | 0.018 |
| rs79265434 | 7 | 24621381 | A | G | 0.8834 | -0.020 | 0.002 | 1.98E-19 | 0.010 | 0.003 | 2.80E-03 | education | -0.006 | 0.010 | -0.012 | 0.013 | -0.014 | 0.029 | 0.017 | 0.025 | 0.019 | 0.042 | -0.053 | 0.024 |
| rs10951590 | 7 | 39091043 | T | C | 0.3275 | -0.011 | 0.002 | 3.62E-13 | 0.004 | 0.002 | 6.40E-02 | education | 0.011 | 0.006 | 0.018 | 0.009 | 0.031 | 0.019 | 0.007 | 0.016 | 0.042 | 0.027 | 0.002 | 0.016 |
| rs10234444 | 7 | 41828527 | G | A | 0.823 | -0.008 | 0.002 | 8.80E-04 | 0.016 | 0.003 | 2.90E-08 | TV | 0.012 | 0.009 | 0.001 | 0.011 | 0.031 | 0.025 | 0.005 | 0.021 | -0.069 | 0.035 | 0.021 | 0.022 |
| rs1220779 | 7 | 54734673 | A | G | 0.4679 | -0.008 | 0.001 | 1.58E-08 | 0.003 | 0.002 | 1.20E-01 | education | 0.005 | 0.006 | 0.006 | 0.008 | 0.002 | 0.018 | 0.003 | 0.015 | -0.037 | 0.026 | 0.017 | 0.015 |
| rs1167827 | 7 | 75163169 | A | G | 0.4347 | 0.010 | 0.001 | 2.93E-13 | -0.008 | 0.002 | 2.10E-04 | education | -0.009 | 0.006 | 0.000 | 0.008 | -0.021 | 0.018 | -0.030 | 0.015 | -0.037 | 0.026 | -0.004 | 0.015 |
| rs6973656 | 7 | 77422583 | A | G | 0.603 | 0.004 | 0.002 | 3.09E-02 | -0.013 | 0.002 | 1.00E-09 | TV | 0.000 | 0.007 | 0.006 | 0.009 | -0.013 | 0.019 | -0.019 | 0.016 | -0.013 | 0.027 | 0.003 | 0.017 |
| rs973734 | 7 | 89387578 | C | A | 0.152 | -0.007 | 0.002 | 1.58E-03 | 0.017 | 0.003 | 1.50E-08 | TV | 0.013 | 0.009 | 0.015 | 0.011 | 0.032 | 0.025 | 0.015 | 0.021 | 0.017 | 0.036 | 0.023 | 0.022 |
| rs9655780 | 7 | 104667334 | A | G | 0.8287 | -0.015 | 0.002 | 3.70E-16 | 0.010 | 0.003 | 7.80E-04 | education | -0.008 | 0.008 | -0.006 | 0.011 | 0.004 | 0.025 | -0.009 | 0.021 | 0.087 | 0.037 | -0.044 | 0.021 |
| rs62471080 | 7 | 109128060 | G | C | 0.541 | 0.005 | 0.002 | 1.48E-03 | -0.013 | 0.002 | 3.70E-09 | TV | 0.005 | 0.006 | 0.006 | 0.008 | 0.027 | 0.018 | 0.009 | 0.015 | 0.005 | 0.026 | -0.004 | 0.015 |
| rs10246289 | 7 | 115544763 | A | G | 0.113 | -0.007 | 0.003 | 9.30E-03 | 0.019 | 0.003 | 1.70E-08 | TV | 0.021 | 0.010 | 0.018 | 0.013 | 0.021 | 0.027 | 0.014 | 0.024 | 0.032 | 0.040 | 0.048 | 0.024 |
| rs11763734 | 7 | 126400910 | A | C | 0.51 | 0.007 | 0.002 | 3.27E-05 | -0.012 | 0.002 | 8.20E-09 | TV | 0.003 | 0.006 | -0.003 | 0.008 | 0.011 | 0.018 | 0.009 | 0.015 | -0.006 | 0.026 | 0.024 | 0.015 |
| rs113520408 | 7 | 128402782 | A | G | 0.2756 | 0.016 | 0.002 | 1.03E-22 | -0.006 | 0.002 | 1.90E-02 | education | -0.002 | 0.007 | -0.001 | 0.009 | 0.042 | 0.020 | -0.029 | 0.018 | -0.058 | 0.031 | -0.003 | 0.018 |
| rs57352738 | 7 | 133304694 | A | T | 0.204 | -0.015 | 0.002 | 2.51E-18 | 0.002 | 0.003 | 5.60E-01 | education | -0.022 | 0.008 | -0.014 | 0.010 | -0.044 | 0.022 | -0.009 | 0.019 | -0.078 | 0.033 | -0.017 | 0.019 |
| rs4726070 | 7 | 151328218 | A | G | 0.6021 | 0.012 | 0.001 | 1.36E-17 | -0.004 | 0.002 | 6.60E-02 | education | -0.003 | 0.006 | -0.002 | 0.009 | -0.023 | 0.019 | 0.026 | 0.016 | -0.034 | 0.027 | 0.005 | 0.016 |
| rs6994287 | 8 | 9340932 | A | G | 0.4055 | 0.010 | 0.001 | 1.11E-12 | -0.001 | 0.002 | 6.30E-01 | education | 0.000 | 0.006 | 0.005 | 0.008 | -0.020 | 0.018 | -0.001 | 0.016 | -0.013 | 0.026 | -0.013 | 0.016 |
| rs7834121 | 8 | 10770255 | G | T | 0.504 | -0.002 | 0.002 | 2.03E-01 | -0.014 | 0.002 | 1.10E-10 | TV | -0.008 | 0.007 | -0.009 | 0.009 | 0.008 | 0.021 | 0.005 | 0.017 | -0.014 | 0.028 | -0.008 | 0.018 |
| rs34807077 | 8 | 28680769 | A | C | 0.1569 | 0.013 | 0.002 | 3.29E-11 | -0.006 | 0.003 | 3.10E-02 | education | -0.008 | 0.008 | -0.020 | 0.011 | 0.035 | 0.025 | -0.024 | 0.021 | 0.006 | 0.036 | -0.023 | 0.021 |
| rs1421334 | 8 | 30865733 | A | C | 0.45 | -0.011 | 0.002 | 5.46E-11 | 0.017 | 0.002 | 2.20E-15 | TV | 0.005 | 0.006 | 0.003 | 0.008 | 0.031 | 0.018 | -0.006 | 0.016 | 0.018 | 0.026 | 0.009 | 0.016 |
| rs2923424 | 8 | 42382222 | A | G | 0.6069 | 0.012 | 0.001 | 6.31E-17 | -0.004 | 0.002 | 1.10E-01 | education | 0.011 | 0.007 | 0.026 | 0.009 | 0.014 | 0.019 | -0.022 | 0.017 | 0.022 | 0.028 | 0.012 | 0.017 |
| rs1866823 | 8 | 57436577 | A | G | 0.5444 | 0.011 | 0.001 | 3.09E-14 | -0.003 | 0.002 | 1.70E-01 | education | -0.016 | 0.007 | -0.005 | 0.009 | 0.014 | 0.019 | -0.015 | 0.016 | -0.011 | 0.028 | -0.019 | 0.017 |
| rs6996198 | 8 | 65463442 | C | T | 0.843 | 0.007 | 0.002 | 1.73E-03 | -0.016 | 0.003 | 3.50E-08 | TV | -0.001 | 0.008 | -0.001 | 0.011 | -0.003 | 0.024 | -0.003 | 0.021 | 0.065 | 0.036 | -0.036 | 0.021 |
| rs6472942 | 8 | 76827190 | T | C | 0.568 | 0.000 | 0.002 | 9.62E-01 | -0.013 | 0.002 | 1.80E-09 | TV | -0.012 | 0.006 | -0.020 | 0.008 | 0.023 | 0.018 | -0.013 | 0.015 | -0.031 | 0.026 | -0.006 | 0.015 |
| rs11985394 | 8 | 92667521 | T | A | 0.545 | -0.002 | 0.002 | 2.66E-01 | 0.012 | 0.002 | 1.30E-08 | TV | 0.005 | 0.006 | 0.010 | 0.008 | 0.002 | 0.018 | 0.005 | 0.016 | -0.012 | 0.026 | 0.001 | 0.016 |
| rs7012546 | 8 | 105067737 | T | C | 0.4166 | 0.009 | 0.001 | 1.25E-10 | -0.004 | 0.002 | 9.30E-02 | education | 0.004 | 0.006 | 0.003 | 0.008 | 0.013 | 0.018 | -0.001 | 0.015 | -0.010 | 0.026 | 0.003 | 0.016 |
| rs12541615 | 8 | 118870151 | T | C | 0.817 | 0.008 | 0.002 | 1.03E-04 | -0.018 | 0.003 | 3.90E-10 | TV | 0.029 | 0.008 | 0.037 | 0.011 | 0.035 | 0.023 | 0.048 | 0.020 | 0.039 | 0.034 | 0.004 | 0.020 |
| rs13261773 | 8 | 120187090 | C | G | 0.1602 | 0.012 | 0.002 | 3.12E-10 | -0.012 | 0.003 | 1.10E-04 | education | 0.007 | 0.008 | 0.000 | 0.011 | 0.018 | 0.024 | 0.028 | 0.020 | -0.027 | 0.035 | -0.009 | 0.021 |
| rs401526 | 8 | 130929166 | T | C | 0.5258 | -0.010 | 0.001 | 1.15E-13 | 0.008 | 0.002 | 4.70E-04 | education | -0.002 | 0.006 | -0.002 | 0.008 | -0.012 | 0.018 | 0.004 | 0.015 | 0.036 | 0.026 | 0.021 | 0.015 |
| rs7460106 | 8 | 143534777 | T | C | 0.762 | -0.012 | 0.002 | 2.06E-13 | 0.004 | 0.003 | 1.00E-01 | education | 0.001 | 0.008 | 0.005 | 0.010 | -0.011 | 0.022 | 0.008 | 0.019 | 0.015 | 0.032 | -0.033 | 0.019 |
| rs2717559 | 8 | 143882420 | A | G | 0.559 | -0.009 | 0.002 | 5.93E-07 | 0.012 | 0.002 | 1.50E-08 | TV | 0.000 | 0.007 | -0.009 | 0.009 | 0.019 | 0.019 | -0.022 | 0.016 | 0.030 | 0.028 | -0.003 | 0.017 |
| rs2616830 | 9 | 1721385 | G | A | 0.462 | -0.015 | 0.002 | 1.23E-17 | 0.016 | 0.002 | 2.90E-14 | TV | 0.003 | 0.006 | 0.006 | 0.008 | -0.001 | 0.018 | 0.012 | 0.015 | -0.052 | 0.026 | 0.001 | 0.015 |
| rs7875078 | 9 | 14494845 | A | C | 0.4564 | -0.008 | 0.001 | 2.72E-08 | 0.010 | 0.002 | 2.70E-06 | education | 0.001 | 0.006 | 0.003 | 0.008 | 0.018 | 0.018 | -0.018 | 0.015 | -0.009 | 0.026 | 0.011 | 0.015 |
| rs34864022 | 9 | 22609110 | A | G | 0.934 | 0.009 | 0.003 | 8.92E-03 | -0.026 | 0.004 | 1.40E-09 | TV | 0.008 | 0.013 | 0.002 | 0.017 | 0.001 | 0.038 | 0.043 | 0.032 | -0.046 | 0.054 | 0.011 | 0.033 |
| rs12554512 | 9 | 23352293 | T | C | 0.584 | -0.024 | 0.002 | 1.69E-43 | 0.021 | 0.002 | 3.80E-21 | TV | -0.006 | 0.006 | -0.001 | 0.009 | -0.038 | 0.019 | -0.006 | 0.016 | 0.017 | 0.027 | -0.018 | 0.016 |
| rs4977885 | 9 | 23687983 | A | G | 0.3971 | -0.008 | 0.001 | 6.78E-09 | 0.005 | 0.002 | 3.50E-02 | education | 0.009 | 0.007 | -0.001 | 0.009 | -0.002 | 0.019 | 0.014 | 0.017 | 0.029 | 0.028 | 0.012 | 0.017 |
| rs7041702 | 9 | 33053430 | A | G | 0.7406 | -0.011 | 0.002 | 4.53E-12 | -0.004 | 0.002 | 1.10E-01 | education | 0.004 | 0.007 | 0.009 | 0.009 | 0.035 | 0.020 | 0.007 | 0.017 | -0.017 | 0.029 | 0.003 | 0.017 |
| rs11138947 | 9 | 72110562 | T | C | 0.7236 | 0.011 | 0.002 | 2.36E-11 | -0.002 | 0.002 | 5.10E-01 | education | -0.005 | 0.007 | 0.005 | 0.009 | 0.006 | 0.020 | -0.008 | 0.017 | -0.031 | 0.029 | 0.013 | 0.017 |
| rs2791574 | 9 | 82212094 | T | G | 0.657 | 0.008 | 0.002 | 2.22E-06 | -0.013 | 0.002 | 3.00E-08 | TV | 0.003 | 0.006 | 0.011 | 0.008 | 0.009 | 0.019 | -0.004 | 0.016 | 0.026 | 0.027 | -0.029 | 0.016 |
| rs870151 | 9 | 92230747 | T | A | 0.528 | 0.009 | 0.002 | 1.28E-07 | -0.016 | 0.002 | 8.80E-13 | TV | 0.005 | 0.006 | -0.008 | 0.008 | 0.014 | 0.018 | -0.005 | 0.015 | 0.043 | 0.026 | 0.021 | 0.015 |
| rs10761251 | 9 | 96421546 | A | T | 0.6633 | 0.011 | 0.002 | 2.52E-14 | -0.006 | 0.002 | 1.60E-02 | education | -0.001 | 0.006 | -0.008 | 0.008 | 0.001 | 0.019 | 0.020 | 0.016 | -0.029 | 0.027 | 0.013 | 0.016 |
| rs2787374 | 9 | 103054951 | T | C | 0.408 | -0.004 | 0.002 | 1.24E-02 | 0.012 | 0.002 | 1.20E-08 | TV | -0.004 | 0.006 | -0.010 | 0.008 | 0.001 | 0.018 | 0.042 | 0.015 | -0.015 | 0.026 | 0.000 | 0.015 |
| rs76235882 | 9 | 121096681 | A | G | 0.966 | 0.024 | 0.004 | 4.52E-10 | -0.012 | 0.006 | 5.20E-02 | education | 0.018 | 0.017 | 0.002 | 0.023 | 0.153 | 0.054 | 0.011 | 0.043 | -0.014 | 0.073 | 0.048 | 0.045 |
| rs7043521 | 9 | 124602779 | A | T | 0.431 | -0.014 | 0.002 | 5.88E-16 | 0.014 | 0.002 | 6.50E-11 | TV | 0.003 | 0.006 | 0.003 | 0.008 | -0.019 | 0.018 | 0.016 | 0.015 | 0.037 | 0.026 | -0.019 | 0.015 |
| rs2039204 | 9 | 130371107 | A | T | 0.5296 | 0.008 | 0.001 | 5.23E-09 | 0.002 | 0.002 | 4.10E-01 | education | 0.001 | 0.006 | 0.002 | 0.008 | -0.006 | 0.018 | 0.008 | 0.015 | -0.001 | 0.026 | -0.018 | 0.015 |
| rs4382592 | 9 | 134870755 | T | G | 0.301 | -0.016 | 0.002 | 1.01E-18 | 0.014 | 0.002 | 6.70E-09 | TV | 0.011 | 0.007 | 0.004 | 0.009 | 0.030 | 0.019 | 0.002 | 0.017 | 0.045 | 0.028 | 0.006 | 0.017 |
| rs2073869 | 9 | 135763816 | C | T | 0.834 | -0.010 | 0.002 | 1.04E-05 | 0.019 | 0.003 | 1.50E-10 | TV | 0.011 | 0.008 | 0.001 | 0.011 | 0.012 | 0.024 | 0.029 | 0.020 | 0.019 | 0.034 | -0.013 | 0.020 |
| rs7894722 | 10 | 9972046 | T | C | 0.3748 | 0.009 | 0.001 | 5.41E-09 | 0.004 | 0.002 | 5.90E-02 | education | -0.001 | 0.006 | -0.009 | 0.008 | 0.016 | 0.018 | 0.005 | 0.016 | 0.015 | 0.026 | 0.015 | 0.016 |
| rs17148998 | 10 | 10909401 | A | G | 0.2042 | 0.010 | 0.002 | 6.98E-09 | -0.012 | 0.003 | 1.20E-05 | education | -0.020 | 0.008 | -0.025 | 0.011 | -0.011 | 0.023 | 0.006 | 0.020 | 0.013 | 0.033 | -0.022 | 0.020 |
| rs1623025 | 10 | 11164813 | G | C | 0.529 | 0.010 | 0.002 | 4.50E-09 | -0.012 | 0.002 | 1.40E-08 | TV | -0.009 | 0.006 | -0.012 | 0.008 | -0.011 | 0.018 | -0.013 | 0.015 | -0.044 | 0.026 | 0.000 | 0.015 |
| rs1243182 | 10 | 21916728 | C | T | 0.69 | 0.008 | 0.002 | 4.66E-05 | -0.019 | 0.002 | 2.00E-15 | TV | -0.056 | 0.006 | -0.081 | 0.008 | -0.059 | 0.019 | -0.051 | 0.016 | -0.016 | 0.027 | 0.042 | 0.016 |
| rs11598765 | 10 | 23953725 | A | G | 0.1966 | -0.011 | 0.002 | 1.73E-09 | 0.011 | 0.003 | 1.30E-04 | education | -0.006 | 0.008 | 0.009 | 0.010 | -0.051 | 0.022 | 0.009 | 0.019 | -0.006 | 0.032 | -0.003 | 0.019 |
| rs806816 | 10 | 32285942 | A | T | 0.7498 | 0.010 | 0.002 | 4.80E-10 | -0.004 | 0.003 | 1.50E-01 | education | 0.020 | 0.007 | 0.022 | 0.009 | 0.056 | 0.021 | -0.007 | 0.017 | 0.034 | 0.030 | 0.033 | 0.018 |
| rs2007655 | 10 | 34586689 | T | G | 0.4976 | 0.009 | 0.001 | 8.64E-10 | -0.005 | 0.002 | 2.10E-02 | education | -0.011 | 0.006 | -0.011 | 0.008 | 0.011 | 0.018 | -0.028 | 0.015 | -0.044 | 0.026 | 0.008 | 0.015 |
| rs2045147 | 10 | 56660437 | A | G | 0.449 | -0.002 | 0.002 | 1.58E-01 | 0.013 | 0.002 | 5.90E-09 | TV | 0.002 | 0.006 | -0.002 | 0.008 | 0.023 | 0.018 | 0.032 | 0.015 | 0.045 | 0.026 | 0.001 | 0.015 |
| rs10994943 | 10 | 63591413 | T | G | 0.581 | -0.008 | 0.002 | 5.94E-06 | 0.013 | 0.002 | 3.10E-09 | TV | 0.011 | 0.006 | 0.010 | 0.008 | -0.009 | 0.018 | 0.015 | 0.015 | 0.028 | 0.026 | 0.032 | 0.016 |
| rs10996167 | 10 | 66813928 | C | G | 0.6512 | 0.011 | 0.002 | 7.47E-09 | -0.003 | 0.002 | 1.30E-01 | education | 0.005 | 0.006 | 0.011 | 0.008 | 0.007 | 0.019 | 0.003 | 0.016 | -0.007 | 0.027 | -0.004 | 0.016 |
| rs11201422 | 10 | 86924483 | T | C | 0.669 | -0.006 | 0.002 | 9.87E-04 | 0.013 | 0.002 | 3.40E-08 | TV | -0.015 | 0.007 | -0.007 | 0.009 | -0.025 | 0.019 | -0.027 | 0.016 | -0.060 | 0.028 | 0.007 | 0.017 |
| rs1426619 | 10 | 90091540 | T | C | 0.4346 | 0.010 | 0.001 | 1.40E-11 | -0.006 | 0.002 | 3.80E-03 | education | 0.012 | 0.006 | 0.010 | 0.008 | 0.006 | 0.018 | 0.035 | 0.015 | 0.050 | 0.026 | 0.012 | 0.015 |
| rs17882802 | 10 | 101983413 | A | G | 0.4295 | 0.008 | 0.001 | 3.00E-09 | 0.000 | 0.002 | 9.90E-01 | education | 0.003 | 0.006 | -0.004 | 0.008 | 0.014 | 0.018 | 0.009 | 0.015 | 0.007 | 0.026 | 0.012 | 0.015 |
| rs10786658 | 10 | 103737193 | A | T | 0.414 | 0.016 | 0.002 | 1.07E-20 | -0.014 | 0.002 | 4.60E-11 | TV | 0.001 | 0.006 | 0.002 | 0.008 | -0.032 | 0.018 | 0.022 | 0.016 | -0.012 | 0.026 | 0.014 | 0.016 |
| rs790647 | 10 | 106776484 | A | C | 0.2278 | -0.015 | 0.002 | 3.81E-18 | 0.004 | 0.003 | 1.50E-01 | education | 0.006 | 0.007 | -0.004 | 0.009 | 0.028 | 0.021 | 0.021 | 0.018 | 0.012 | 0.030 | 0.007 | 0.018 |
| rs12571549 | 10 | 111770013 | A | G | 0.1368 | 0.015 | 0.002 | 1.45E-13 | -0.008 | 0.003 | 8.70E-03 | education | -0.011 | 0.009 | -0.003 | 0.011 | -0.045 | 0.026 | 0.001 | 0.022 | -0.123 | 0.038 | -0.001 | 0.022 |
| rs7089973 | 10 | 116569565 | C | A | 0.619 | 0.003 | 0.002 | 1.01E-01 | -0.013 | 0.002 | 2.40E-09 | TV | -0.005 | 0.007 | -0.008 | 0.009 | 0.025 | 0.019 | -0.007 | 0.016 | -0.045 | 0.027 | 0.003 | 0.016 |
| rs11245482 | 10 | 126733546 | T | C | 0.613 | 0.006 | 0.002 | 5.32E-04 | -0.013 | 0.002 | 2.60E-09 | TV | -0.005 | 0.006 | -0.003 | 0.008 | -0.037 | 0.018 | 0.004 | 0.015 | -0.016 | 0.026 | 0.022 | 0.016 |
| rs17727474 | 10 | 127182959 | C | T | 0.832 | -0.003 | 0.002 | 1.30E-01 | 0.018 | 0.003 | 3.10E-09 | TV | -0.013 | 0.009 | -0.020 | 0.011 | 0.021 | 0.025 | -0.048 | 0.021 | -0.078 | 0.035 | 0.025 | 0.022 |
| rs12289262 | 11 | 12894758 | C | T | 0.731 | 0.014 | 0.002 | 9.54E-13 | -0.014 | 0.002 | 2.00E-08 | TV | 0.002 | 0.007 | 0.008 | 0.010 | -0.065 | 0.021 | -0.013 | 0.018 | -0.001 | 0.030 | 0.012 | 0.018 |
| rs11023764 | 11 | 15910939 | A | G | 0.6418 | 0.009 | 0.002 | 5.32E-10 | -0.004 | 0.002 | 1.00E-01 | education | -0.009 | 0.006 | -0.001 | 0.009 | -0.036 | 0.019 | -0.015 | 0.016 | -0.001 | 0.027 | -0.008 | 0.016 |
| rs12789313 | 11 | 25058937 | T | C | 0.4907 | 0.009 | 0.001 | 6.79E-11 | -0.002 | 0.002 | 3.00E-01 | education | -0.004 | 0.006 | 0.002 | 0.008 | -0.033 | 0.018 | 0.012 | 0.016 | -0.028 | 0.027 | -0.015 | 0.016 |
| rs11030102 | 11 | 27681596 | C | G | 0.7479 | 0.010 | 0.002 | 4.60E-10 | -0.004 | 0.002 | 1.20E-01 | education | 0.006 | 0.007 | 0.010 | 0.009 | -0.009 | 0.021 | 0.024 | 0.018 | -0.023 | 0.030 | -0.010 | 0.018 |
| rs2199409 | 11 | 39915234 | T | C | 0.7962 | 0.010 | 0.002 | 3.14E-09 | -0.009 | 0.003 | 5.10E-04 | education | -0.006 | 0.007 | -0.011 | 0.010 | 0.028 | 0.022 | -0.035 | 0.018 | -0.032 | 0.031 | -0.004 | 0.019 |
| rs79728014 | 11 | 46062245 | A | G | 0.8553 | -0.013 | 0.002 | 2.06E-11 | 0.005 | 0.003 | 9.30E-02 | education | 0.018 | 0.009 | 0.022 | 0.012 | 0.005 | 0.027 | 0.021 | 0.023 | 0.065 | 0.040 | 0.015 | 0.023 |
| rs75033012 | 11 | 61347033 | C | G | 0.0391 | -0.026 | 0.004 | 1.38E-11 | 0.004 | 0.006 | 4.70E-01 | education | 0.023 | 0.019 | 0.048 | 0.024 | 0.068 | 0.053 | 0.023 | 0.046 | -0.019 | 0.080 | 0.006 | 0.047 |
| rs801733 | 11 | 65934549 | A | C | 0.641 | -0.010 | 0.002 | 1.65E-07 | 0.017 | 0.002 | 7.30E-14 | TV | 0.023 | 0.006 | 0.031 | 0.008 | 0.016 | 0.019 | 0.001 | 0.016 | 0.057 | 0.027 | -0.002 | 0.016 |
| rs116175338 | 11 | 72467875 | G | T | 0.754 | 0.016 | 0.002 | 1.36E-16 | -0.014 | 0.003 | 1.40E-08 | TV | -0.017 | 0.007 | -0.028 | 0.009 | 0.007 | 0.021 | -0.004 | 0.018 | -0.016 | 0.030 | -0.025 | 0.018 |
| rs12272012 | 11 | 84861286 | G | C | 0.956 | 0.009 | 0.004 | 3.62E-02 | -0.030 | 0.005 | 1.90E-08 | TV | -0.023 | 0.015 | -0.017 | 0.019 | 0.098 | 0.046 | -0.003 | 0.037 | -0.045 | 0.063 | -0.040 | 0.037 |
| rs11020045 | 11 | 92487907 | A | C | 0.669 | 0.007 | 0.002 | 1.88E-04 | -0.013 | 0.002 | 1.60E-08 | TV | -0.009 | 0.006 | -0.003 | 0.008 | 0.000 | 0.019 | -0.017 | 0.016 | -0.020 | 0.027 | -0.022 | 0.016 |
| rs17207890 | 11 | 95490754 | G | A | 0.657 | -0.014 | 0.002 | 3.58E-14 | 0.016 | 0.002 | 6.70E-12 | TV | 0.017 | 0.007 | 0.033 | 0.009 | 0.000 | 0.019 | 0.039 | 0.016 | -0.011 | 0.027 | 0.019 | 0.016 |
| rs1301838 | 11 | 99670514 | T | C | 0.3227 | -0.010 | 0.002 | 1.29E-10 | 0.005 | 0.002 | 3.70E-02 | education | -0.007 | 0.007 | -0.009 | 0.009 | 0.023 | 0.019 | 0.012 | 0.016 | 0.022 | 0.028 | -0.016 | 0.017 |
| rs72486027 | 11 | 107167486 | T | C | 0.2459 | -0.010 | 0.002 | 1.61E-10 | 0.002 | 0.003 | 5.00E-01 | education | -0.007 | 0.007 | -0.004 | 0.009 | -0.020 | 0.020 | 0.008 | 0.017 | -0.015 | 0.030 | 0.007 | 0.018 |
| rs2212430 | 11 | 109289538 | T | C | 0.3038 | -0.010 | 0.002 | 1.32E-10 | 0.007 | 0.002 | 1.60E-03 | education | 0.001 | 0.007 | 0.011 | 0.009 | 0.004 | 0.019 | -0.009 | 0.016 | 0.000 | 0.028 | -0.034 | 0.017 |
| rs11213482 | 11 | 110435636 | A | G | 0.836 | -0.011 | 0.002 | 2.68E-08 | 0.010 | 0.003 | 4.20E-04 | education | 0.014 | 0.009 | 0.014 | 0.012 | -0.004 | 0.027 | 0.024 | 0.023 | -0.006 | 0.039 | 0.022 | 0.024 |
| rs17565975 | 11 | 111586950 | A | G | 0.5563 | -0.011 | 0.001 | 5.54E-14 | 0.005 | 0.002 | 2.20E-02 | education | 0.010 | 0.006 | 0.020 | 0.008 | -0.009 | 0.018 | 0.006 | 0.015 | 0.008 | 0.026 | 0.018 | 0.015 |
| rs11218575 | 11 | 122154576 | C | T | 0.566 | -0.006 | 0.002 | 5.66E-04 | 0.015 | 0.002 | 2.10E-12 | TV | 0.009 | 0.007 | 0.000 | 0.009 | 0.024 | 0.019 | 0.001 | 0.016 | 0.063 | 0.028 | 0.008 | 0.016 |
| rs12574281 | 11 | 131205421 | A | C | 0.631 | -0.008 | 0.002 | 3.04E-08 | 0.006 | 0.002 | 4.40E-03 | education | 0.006 | 0.007 | 0.009 | 0.009 | 0.002 | 0.019 | -0.001 | 0.016 | -0.009 | 0.028 | 0.004 | 0.017 |
| rs4937842 | 11 | 133734542 | G | C | 0.634 | 0.002 | 0.002 | 1.87E-01 | -0.013 | 0.002 | 1.40E-08 | TV | 0.009 | 0.006 | 0.012 | 0.008 | -0.005 | 0.018 | 0.017 | 0.016 | 0.020 | 0.027 | 0.010 | 0.016 |
| rs4766424 | 12 | 1954096 | C | G | 0.1237 | 0.014 | 0.002 | 2.47E-11 | -0.001 | 0.003 | 6.50E-01 | education | -0.016 | 0.009 | -0.027 | 0.012 | 0.035 | 0.027 | -0.023 | 0.023 | -0.006 | 0.039 | 0.004 | 0.023 |
| rs10772643 | 12 | 13415288 | C | T | 0.108 | -0.016 | 0.003 | 3.54E-09 | 0.025 | 0.003 | 1.30E-12 | TV | 0.008 | 0.010 | 0.022 | 0.013 | -0.012 | 0.030 | -0.005 | 0.025 | -0.044 | 0.044 | -0.017 | 0.026 |
| rs1861786 | 12 | 14000467 | A | G | 0.3813 | -0.008 | 0.001 | 2.39E-08 | 0.005 | 0.002 | 1.90E-02 | education | 0.007 | 0.006 | 0.012 | 0.008 | 0.026 | 0.018 | -0.002 | 0.016 | 0.009 | 0.027 | -0.006 | 0.016 |
| rs7974852 | 12 | 14511679 | A | C | 0.5235 | 0.012 | 0.001 | 1.14E-16 | -0.003 | 0.002 | 1.90E-01 | education | 0.009 | 0.006 | 0.008 | 0.008 | 0.010 | 0.018 | 0.008 | 0.015 | 0.013 | 0.026 | 0.009 | 0.015 |
| rs117398064 | 12 | 26523628 | C | G | 0.0912 | -0.014 | 0.003 | 1.16E-08 | 0.007 | 0.004 | 6.20E-02 | education | 0.004 | 0.011 | 0.011 | 0.015 | -0.006 | 0.034 | -0.062 | 0.029 | 0.002 | 0.048 | 0.011 | 0.029 |
| rs7972246 | 12 | 27209328 | T | C | 0.3451 | 0.011 | 0.002 | 1.01E-13 | -0.006 | 0.002 | 5.20E-03 | education | -0.003 | 0.006 | -0.004 | 0.008 | 0.025 | 0.018 | -0.001 | 0.016 | 0.016 | 0.027 | -0.010 | 0.016 |
| rs10771746 | 12 | 30791864 | C | T | 0.717 | 0.005 | 0.002 | 4.35E-03 | -0.014 | 0.002 | 2.50E-09 | TV | -0.024 | 0.007 | -0.015 | 0.009 | -0.037 | 0.019 | -0.053 | 0.016 | -0.018 | 0.028 | 0.013 | 0.017 |
| rs17110109 | 12 | 54668908 | T | C | 0.6163 | -0.009 | 0.001 | 1.47E-10 | 0.002 | 0.002 | 2.70E-01 | education | 0.013 | 0.006 | 0.016 | 0.008 | 0.011 | 0.018 | 0.015 | 0.016 | -0.059 | 0.026 | -0.013 | 0.016 |
| rs10876864 | 12 | 56401085 | G | A | 0.427 | 0.016 | 0.002 | 1.22E-19 | -0.013 | 0.002 | 1.00E-09 | TV | 0.004 | 0.006 | 0.008 | 0.008 | 0.040 | 0.018 | -0.011 | 0.015 | 0.008 | 0.026 | -0.024 | 0.016 |
| rs8756 | 12 | 66359752 | C | A | 0.485 | 0.008 | 0.002 | 1.41E-06 | -0.013 | 0.002 | 5.30E-10 | TV | -0.003 | 0.006 | 0.004 | 0.008 | 0.009 | 0.018 | -0.007 | 0.015 | 0.019 | 0.025 | -0.023 | 0.015 |
| rs57661533 | 12 | 78629917 | T | C | 0.1341 | -0.011 | 0.002 | 3.24E-08 | 0.003 | 0.003 | 2.90E-01 | education | 0.015 | 0.009 | 0.022 | 0.012 | 0.002 | 0.027 | -0.007 | 0.023 | 0.068 | 0.038 | 0.002 | 0.023 |
| rs1245829 | 12 | 79648170 | A | T | 0.5814 | -0.010 | 0.001 | 3.29E-12 | 0.011 | 0.002 | 7.50E-07 | education | -0.003 | 0.007 | -0.002 | 0.009 | -0.006 | 0.019 | 0.006 | 0.017 | 0.000 | 0.028 | -0.009 | 0.017 |
| rs717996 | 12 | 84133820 | T | C | 0.5986 | -0.012 | 0.001 | 1.89E-17 | 0.007 | 0.002 | 8.60E-04 | education | 0.000 | 0.006 | -0.011 | 0.008 | 0.038 | 0.018 | -0.004 | 0.016 | -0.006 | 0.026 | 0.023 | 0.016 |
| rs2216144 | 12 | 97666478 | T | C | 0.5306 | 0.010 | 0.001 | 2.58E-12 | -0.004 | 0.002 | 7.70E-02 | education | 0.014 | 0.006 | 0.014 | 0.008 | 0.007 | 0.018 | 0.022 | 0.015 | 0.053 | 0.026 | -0.008 | 0.015 |
| rs2131167 | 12 | 99633189 | A | G | 0.4458 | 0.008 | 0.001 | 1.52E-08 | -0.003 | 0.002 | 1.20E-01 | education | -0.009 | 0.006 | -0.012 | 0.008 | -0.014 | 0.018 | 0.024 | 0.015 | -0.035 | 0.026 | -0.022 | 0.016 |
| rs2164744 | 12 | 109872039 | T | C | 0.635 | 0.004 | 0.002 | 1.28E-02 | -0.013 | 0.002 | 1.10E-08 | TV | 0.019 | 0.006 | 0.009 | 0.008 | 0.024 | 0.018 | 0.042 | 0.016 | 0.072 | 0.027 | 0.011 | 0.016 |
| rs2173650 | 12 | 117525235 | G | T | 0.852 | -0.012 | 0.002 | 3.06E-07 | 0.018 | 0.003 | 4.60E-09 | TV | 0.016 | 0.009 | 0.014 | 0.014 | 0.012 | 0.028 | 0.009 | 0.025 | 0.036 | 0.042 | 0.043 | 0.026 |
| rs6490618 | 13 | 21338343 | T | C | 0.327 | 0.009 | 0.002 | 3.48E-09 | -0.005 | 0.002 | 3.40E-02 | education | -0.010 | 0.006 | -0.007 | 0.008 | -0.005 | 0.019 | -0.011 | 0.016 | -0.030 | 0.027 | -0.032 | 0.016 |
| rs9529146 | 13 | 31785404 | T | C | 0.2282 | 0.013 | 0.002 | 3.48E-14 | -0.006 | 0.003 | 2.00E-02 | education | -0.011 | 0.007 | -0.014 | 0.010 | 0.001 | 0.021 | 0.003 | 0.018 | -0.044 | 0.031 | -0.046 | 0.019 |
| rs936496 | 13 | 36100541 | A | G | 0.6229 | -0.008 | 0.001 | 4.38E-08 | 0.006 | 0.002 | 3.90E-03 | education | -0.009 | 0.006 | -0.012 | 0.008 | 0.016 | 0.018 | -0.031 | 0.016 | 0.036 | 0.027 | 0.014 | 0.016 |
| rs9563168 | 13 | 54247827 | G | A | 0.791 | -0.010 | 0.002 | 5.93E-07 | 0.018 | 0.003 | 4.30E-11 | TV | 0.003 | 0.008 | 0.011 | 0.010 | 0.010 | 0.022 | 0.003 | 0.019 | -0.004 | 0.032 | -0.013 | 0.019 |
| rs9569734 | 13 | 58319476 | A | G | 0.844 | -0.009 | 0.002 | 2.77E-04 | 0.019 | 0.003 | 3.40E-10 | TV | 0.002 | 0.008 | 0.010 | 0.011 | -0.034 | 0.024 | 0.014 | 0.021 | 0.002 | 0.036 | 0.023 | 0.021 |
| rs7321274 | 13 | 69146186 | A | G | 0.7999 | 0.011 | 0.002 | 8.21E-11 | -0.005 | 0.003 | 6.20E-02 | education | -0.011 | 0.008 | -0.017 | 0.010 | -0.023 | 0.023 | -0.023 | 0.019 | -0.007 | 0.033 | 0.002 | 0.020 |
| rs2478208 | 13 | 81471113 | C | G | 0.5204 | -0.010 | 0.001 | 2.89E-13 | 0.009 | 0.002 | 6.10E-05 | education | 0.006 | 0.006 | 0.012 | 0.008 | 0.023 | 0.018 | 0.023 | 0.015 | 0.003 | 0.026 | -0.009 | 0.015 |
| rs56858768 | 13 | 86511730 | G | A | 0.701 | 0.006 | 0.002 | 1.18E-03 | -0.015 | 0.002 | 3.40E-10 | TV | 0.012 | 0.007 | 0.008 | 0.009 | 0.012 | 0.020 | 0.017 | 0.017 | -0.002 | 0.029 | 0.004 | 0.017 |
| rs7326331 | 13 | 91615838 | A | G | 0.2761 | -0.013 | 0.002 | 3.42E-16 | 0.009 | 0.002 | 1.00E-04 | education | 0.009 | 0.007 | 0.009 | 0.009 | 0.009 | 0.020 | 0.005 | 0.017 | 0.050 | 0.028 | 0.007 | 0.017 |
| rs9556958 | 13 | 99100046 | T | C | 0.5233 | -0.011 | 0.001 | 5.96E-15 | 0.004 | 0.002 | 6.50E-02 | education | -0.014 | 0.006 | -0.013 | 0.008 | -0.028 | 0.018 | 0.011 | 0.015 | -0.026 | 0.026 | 0.004 | 0.015 |
| rs7991062 | 13 | 100713194 | C | G | 0.659 | 0.008 | 0.002 | 3.09E-06 | -0.018 | 0.002 | 8.80E-15 | TV | -0.004 | 0.006 | -0.012 | 0.008 | -0.001 | 0.019 | -0.001 | 0.016 | -0.036 | 0.027 | -0.005 | 0.016 |
| rs11157931 | 14 | 23403193 | A | C | 0.3924 | -0.014 | 0.001 | 3.86E-22 | 0.010 | 0.002 | 1.10E-05 | education | 0.010 | 0.006 | 0.006 | 0.008 | 0.024 | 0.018 | 0.009 | 0.015 | 0.039 | 0.026 | 0.009 | 0.016 |
| rs405797 | 14 | 26972834 | T | A | 0.247 | 0.015 | 0.002 | 2.12E-14 | -0.015 | 0.003 | 2.80E-09 | TV | 0.015 | 0.008 | 0.013 | 0.010 | 0.017 | 0.022 | 0.036 | 0.019 | 0.027 | 0.032 | 0.004 | 0.019 |
| rs1022785 | 14 | 29571653 | G | A | 0.142 | -0.018 | 0.002 | 2.05E-13 | 0.018 | 0.003 | 7.10E-09 | TV | 0.008 | 0.009 | 0.009 | 0.011 | 0.007 | 0.025 | -0.014 | 0.022 | -0.026 | 0.037 | 0.032 | 0.022 |
| rs61997667 | 14 | 57280046 | T | C | 0.1511 | -0.014 | 0.002 | 2.24E-13 | 0.014 | 0.003 | 1.60E-06 | education | 0.014 | 0.009 | 0.013 | 0.011 | -0.017 | 0.025 | -0.002 | 0.021 | 0.045 | 0.036 | 0.012 | 0.022 |
| rs4899012 | 14 | 61003889 | C | G | 0.6019 | -0.010 | 0.001 | 8.06E-13 | 0.004 | 0.002 | 9.60E-02 | education | 0.002 | 0.006 | 0.007 | 0.008 | 0.007 | 0.018 | 0.018 | 0.015 | 0.007 | 0.026 | -0.010 | 0.016 |
| rs12888615 | 14 | 72435705 | T | C | 0.1991 | 0.012 | 0.002 | 9.42E-12 | -0.012 | 0.003 | 8.00E-06 | education | 0.012 | 0.008 | 0.014 | 0.010 | -0.026 | 0.022 | 0.015 | 0.019 | -0.006 | 0.033 | 0.010 | 0.019 |
| rs730384 | 14 | 74889870 | A | G | 0.4388 | 0.010 | 0.001 | 9.38E-13 | -0.005 | 0.002 | 1.20E-02 | education | 0.003 | 0.006 | -0.002 | 0.008 | 0.020 | 0.018 | 0.018 | 0.015 | 0.026 | 0.026 | 0.015 | 0.015 |
| rs2998299 | 14 | 84997363 | T | G | 0.2137 | -0.014 | 0.002 | 1.51E-15 | 0.007 | 0.003 | 1.10E-02 | education | 0.008 | 0.008 | 0.005 | 0.010 | 0.017 | 0.022 | -0.011 | 0.019 | 0.071 | 0.032 | 0.026 | 0.019 |
| rs10145592 | 14 | 94287215 | C | G | 0.409 | 0.011 | 0.002 | 1.02E-09 | -0.015 | 0.002 | 1.80E-11 | TV | 0.003 | 0.007 | -0.015 | 0.009 | -0.009 | 0.019 | 0.006 | 0.016 | 0.030 | 0.027 | 0.020 | 0.016 |
| rs7157001 | 14 | 99749484 | A | G | 0.744 | 0.009 | 0.002 | 1.29E-05 | -0.014 | 0.002 | 2.30E-08 | TV | -0.003 | 0.008 | -0.008 | 0.011 | -0.020 | 0.024 | 0.027 | 0.021 | 0.018 | 0.035 | 0.013 | 0.021 |
| rs7167688 | 15 | 26813137 | T | C | 0.4917 | -0.009 | 0.001 | 1.71E-11 | 0.005 | 0.002 | 2.10E-02 | education | 0.010 | 0.006 | 0.014 | 0.008 | -0.022 | 0.018 | 0.005 | 0.015 | 0.060 | 0.025 | 0.016 | 0.015 |
| rs891793 | 15 | 27256021 | C | G | 0.5403 | -0.010 | 0.001 | 1.87E-12 | 0.005 | 0.002 | 1.90E-02 | education | 0.002 | 0.006 | 0.000 | 0.008 | -0.005 | 0.018 | -0.010 | 0.015 | 0.002 | 0.026 | 0.026 | 0.016 |
| rs8043253 | 15 | 41503730 | C | T | 0.573 | 0.001 | 0.002 | 5.79E-01 | -0.012 | 0.002 | 3.40E-08 | TV | -0.004 | 0.006 | -0.018 | 0.008 | 0.016 | 0.018 | 0.012 | 0.015 | -0.004 | 0.026 | -0.008 | 0.015 |
| rs6493265 | 15 | 47513253 | T | C | 0.3915 | -0.012 | 0.001 | 2.26E-17 | 0.001 | 0.002 | 5.20E-01 | education | 0.011 | 0.007 | 0.007 | 0.009 | 0.018 | 0.019 | 0.043 | 0.016 | -0.012 | 0.028 | 0.010 | 0.016 |
| rs2460 | 15 | 53073084 | G | A | 0.736 | 0.009 | 0.002 | 1.04E-05 | -0.015 | 0.002 | 5.00E-10 | TV | 0.000 | 0.007 | -0.007 | 0.010 | -0.001 | 0.021 | -0.007 | 0.018 | 0.024 | 0.031 | 0.025 | 0.019 |
| rs4775373 | 15 | 61476936 | T | C | 0.361 | -0.007 | 0.002 | 2.40E-04 | 0.013 | 0.002 | 4.70E-09 | TV | 0.002 | 0.006 | 0.000 | 0.009 | -0.002 | 0.019 | 0.004 | 0.016 | 0.034 | 0.027 | -0.026 | 0.016 |
| rs12591647 | 15 | 65959729 | T | C | 0.8171 | -0.015 | 0.002 | 7.78E-17 | 0.003 | 0.003 | 3.40E-01 | education | 0.016 | 0.008 | 0.004 | 0.010 | 0.071 | 0.023 | 0.034 | 0.020 | 0.007 | 0.033 | 0.014 | 0.020 |
| rs1564347 | 15 | 73438848 | T | G | 0.3433 | 0.009 | 0.002 | 2.39E-09 | -0.010 | 0.002 | 7.10E-06 | education | 0.003 | 0.006 | 0.001 | 0.008 | 0.015 | 0.019 | 0.022 | 0.016 | -0.003 | 0.027 | -0.012 | 0.016 |
| rs61331678 | 15 | 77995949 | G | C | 0.568 | -0.013 | 0.002 | 2.63E-13 | 0.015 | 0.002 | 6.10E-12 | TV | 0.002 | 0.006 | 0.002 | 0.008 | 0.027 | 0.018 | -0.034 | 0.015 | -0.027 | 0.026 | 0.017 | 0.016 |
| rs4778058 | 15 | 93456069 | T | C | 0.4964 | -0.009 | 0.001 | 4.92E-11 | 0.010 | 0.002 | 2.00E-06 | education | 0.003 | 0.007 | -0.005 | 0.009 | -0.007 | 0.019 | 0.001 | 0.016 | -0.002 | 0.027 | 0.010 | 0.016 |
| rs4984541 | 15 | 96911139 | A | G | 0.7666 | -0.014 | 0.002 | 3.40E-16 | 0.006 | 0.003 | 3.00E-02 | education | 0.003 | 0.008 | 0.016 | 0.011 | -0.035 | 0.023 | -0.002 | 0.020 | 0.003 | 0.034 | 0.018 | 0.020 |
| rs8030487 | 15 | 97119595 | A | G | 0.6907 | 0.009 | 0.002 | 2.52E-08 | -0.007 | 0.002 | 5.30E-03 | education | -0.009 | 0.007 | -0.004 | 0.009 | 0.013 | 0.019 | -0.013 | 0.016 | 0.002 | 0.028 | 0.013 | 0.016 |
| rs9933256 | 16 | 1246748 | A | G | 0.5558 | 0.012 | 0.001 | 6.06E-17 | -0.006 | 0.002 | 2.90E-03 | education | 0.003 | 0.006 | -0.006 | 0.008 | 0.023 | 0.018 | 0.011 | 0.015 | 0.014 | 0.026 | 0.011 | 0.016 |
| rs34098770 | 16 | 12237812 | A | G | 0.152 | -0.016 | 0.002 | 2.95E-16 | 0.007 | 0.003 | 1.40E-02 | education | -0.006 | 0.008 | 0.007 | 0.011 | 0.007 | 0.024 | -0.024 | 0.021 | -0.035 | 0.036 | -0.030 | 0.021 |
| rs35574015 | 16 | 15146061 | T | C | 0.706 | 0.002 | 0.002 | 2.21E-01 | -0.013 | 0.002 | 3.00E-08 | TV | -0.008 | 0.007 | 0.000 | 0.009 | -0.023 | 0.020 | -0.008 | 0.018 | 0.026 | 0.030 | -0.015 | 0.018 |
| rs7189927 | 16 | 28913787 | T | C | 0.356 | -0.015 | 0.002 | 7.31E-18 | 0.015 | 0.002 | 3.40E-11 | TV | 0.001 | 0.006 | 0.006 | 0.008 | -0.010 | 0.019 | -0.001 | 0.016 | 0.014 | 0.027 | -0.026 | 0.016 |
| rs749671 | 16 | 31088347 | G | A | 0.629 | -0.006 | 0.002 | 3.15E-04 | 0.016 | 0.002 | 2.70E-12 | TV | -0.017 | 0.006 | -0.014 | 0.008 | -0.010 | 0.018 | -0.010 | 0.015 | -0.003 | 0.026 | -0.015 | 0.016 |
| rs2052285 | 16 | 51188432 | A | G | 0.5959 | 0.011 | 0.001 | 7.25E-15 | -0.008 | 0.002 | 3.50E-04 | education | -0.011 | 0.006 | -0.010 | 0.008 | -0.005 | 0.018 | -0.015 | 0.015 | 0.021 | 0.026 | -0.020 | 0.016 |
| rs7184800 | 16 | 53509131 | G | A | 0.697 | -0.010 | 0.002 | 2.38E-08 | 0.017 | 0.002 | 8.20E-13 | TV | -0.005 | 0.007 | -0.010 | 0.009 | -0.014 | 0.019 | 0.005 | 0.016 | 0.026 | 0.028 | -0.011 | 0.016 |
| rs17248751 | 16 | 61579618 | A | G | 0.7827 | -0.014 | 0.002 | 1.28E-15 | 0.006 | 0.003 | 1.30E-02 | education | 0.000 | 0.008 | 0.007 | 0.010 | 0.045 | 0.022 | -0.017 | 0.019 | -0.011 | 0.032 | -0.010 | 0.019 |
| rs9929556 | 16 | 62193640 | T | G | 0.5688 | -0.010 | 0.001 | 2.31E-12 | 0.004 | 0.002 | 6.90E-02 | education | -0.001 | 0.006 | 0.013 | 0.008 | -0.009 | 0.018 | -0.006 | 0.015 | -0.016 | 0.026 | -0.001 | 0.016 |
| rs17604349 | 16 | 72210865 | A | G | 0.1852 | 0.013 | 0.002 | 2.36E-12 | -0.013 | 0.003 | 5.10E-06 | education | 0.008 | 0.008 | 0.000 | 0.010 | -0.023 | 0.023 | 0.016 | 0.019 | -0.007 | 0.033 | 0.000 | 0.019 |
| rs9929762 | 16 | 78169675 | A | G | 0.5595 | 0.010 | 0.001 | 1.03E-11 | -0.008 | 0.002 | 5.50E-04 | education | -0.011 | 0.006 | -0.014 | 0.008 | -0.017 | 0.018 | 0.006 | 0.015 | -0.005 | 0.026 | -0.014 | 0.015 |
| rs61527214 | 16 | 83608599 | A | G | 0.4113 | 0.011 | 0.001 | 1.13E-14 | -0.007 | 0.002 | 3.10E-03 | education | -0.001 | 0.007 | -0.002 | 0.009 | -0.024 | 0.019 | -0.013 | 0.016 | -0.028 | 0.027 | -0.006 | 0.016 |
| rs74415461 | 16 | 89990843 | T | C | 0.0843 | 0.018 | 0.003 | 6.25E-12 | -0.015 | 0.004 | 1.20E-04 | education | 0.017 | 0.011 | 0.005 | 0.015 | 0.009 | 0.034 | 0.014 | 0.028 | 0.048 | 0.049 | 0.018 | 0.029 |
| rs2447098 | 17 | 2277720 | C | A | 0.475 | 0.008 | 0.002 | 7.73E-07 | -0.015 | 0.002 | 6.80E-12 | TV | 0.008 | 0.007 | 0.009 | 0.009 | 0.012 | 0.019 | 0.036 | 0.016 | -0.005 | 0.027 | 0.020 | 0.016 |
| rs11654952 | 17 | 4813799 | T | G | 0.861 | 0.003 | 0.003 | 3.08E-01 | -0.017 | 0.003 | 4.30E-08 | TV | 0.007 | 0.009 | 0.009 | 0.012 | 0.031 | 0.026 | 0.037 | 0.022 | 0.017 | 0.038 | -0.027 | 0.022 |
| rs12602286 | 17 | 19236954 | T | G | 0.8731 | 0.016 | 0.002 | 4.62E-14 | -0.001 | 0.003 | 8.60E-01 | education | -0.008 | 0.010 | -0.002 | 0.013 | -0.008 | 0.027 | -0.036 | 0.023 | -0.037 | 0.040 | -0.014 | 0.024 |
| rs11657979 | 17 | 32900837 | A | G | 0.2449 | -0.009 | 0.002 | 4.57E-08 | 0.009 | 0.003 | 6.90E-04 | education | 0.013 | 0.008 | 0.011 | 0.010 | 0.005 | 0.023 | 0.051 | 0.019 | 0.016 | 0.033 | 0.016 | 0.020 |
| rs1842713 | 17 | 33200385 | A | G | 0.7882 | -0.012 | 0.002 | 9.67E-12 | 0.010 | 0.003 | 3.00E-04 | education | -0.009 | 0.008 | -0.009 | 0.010 | -0.016 | 0.022 | -0.012 | 0.019 | -0.028 | 0.032 | 0.007 | 0.019 |
| rs4793090 | 17 | 40686342 | A | G | 0.6635 | 0.008 | 0.002 | 1.67E-08 | -0.010 | 0.002 | 6.00E-06 | education | -0.031 | 0.006 | -0.044 | 0.008 | -0.029 | 0.019 | -0.044 | 0.016 | -0.044 | 0.027 | -0.023 | 0.016 |
| rs11871429 | 17 | 42920929 | A | G | 0.7727 | 0.013 | 0.002 | 2.52E-14 | -0.006 | 0.003 | 1.30E-02 | education | -0.011 | 0.007 | -0.014 | 0.009 | -0.011 | 0.021 | 0.003 | 0.018 | -0.012 | 0.030 | -0.004 | 0.018 |
| rs56319902 | 17 | 43871982 | T | C | 0.2155 | -0.021 | 0.002 | 5.81E-33 | 0.005 | 0.003 | 7.50E-02 | education | -0.045 | 0.007 | -0.042 | 0.010 | -0.067 | 0.022 | -0.006 | 0.018 | -0.060 | 0.032 | -0.016 | 0.019 |
| rs56794817 | 17 | 60001978 | A | G | 0.1615 | 0.012 | 0.002 | 1.69E-10 | -0.005 | 0.003 | 9.90E-02 | education | 0.031 | 0.008 | 0.039 | 0.011 | 0.009 | 0.024 | 0.006 | 0.020 | 0.042 | 0.034 | 0.020 | 0.020 |
| rs9902312 | 17 | 65070304 | T | C | 0.683 | 0.000 | 0.002 | 8.57E-01 | 0.015 | 0.002 | 4.50E-11 | TV | -0.009 | 0.006 | -0.024 | 0.008 | -0.026 | 0.019 | 0.016 | 0.016 | -0.021 | 0.027 | 0.009 | 0.016 |
| rs68145588 | 17 | 75864805 | T | G | 0.1395 | -0.013 | 0.002 | 7.53E-10 | -0.001 | 0.003 | 8.10E-01 | education | 0.013 | 0.009 | 0.014 | 0.012 | -0.010 | 0.027 | 0.039 | 0.023 | -0.024 | 0.040 | -0.022 | 0.024 |
| rs11657730 | 17 | 79372489 | C | T | 0.637 | -0.013 | 0.002 | 4.31E-11 | 0.013 | 0.002 | 1.10E-08 | TV | 0.021 | 0.007 | 0.021 | 0.009 | 0.007 | 0.020 | 0.024 | 0.017 | 0.062 | 0.029 | 0.023 | 0.017 |
| rs12967010 | 18 | 9645035 | T | C | 0.7746 | 0.011 | 0.002 | 1.33E-11 | -0.005 | 0.003 | 6.00E-02 | education | -0.016 | 0.008 | -0.017 | 0.010 | -0.012 | 0.022 | -0.008 | 0.019 | -0.037 | 0.031 | -0.001 | 0.019 |
| rs303753 | 18 | 21074922 | G | A | 0.653 | 0.015 | 0.002 | 2.60E-16 | -0.014 | 0.002 | 2.70E-10 | TV | -0.004 | 0.007 | -0.010 | 0.009 | -0.033 | 0.019 | 0.005 | 0.016 | 0.026 | 0.028 | -0.051 | 0.016 |
| rs9964724 | 18 | 35159124 | C | T | 0.318 | -0.020 | 0.002 | 2.66E-27 | 0.018 | 0.002 | 3.30E-14 | TV | 0.007 | 0.007 | 0.002 | 0.009 | 0.029 | 0.019 | 0.007 | 0.017 | -0.043 | 0.029 | 0.009 | 0.017 |
| rs1156541 | 18 | 39952989 | C | T | 0.223 | -0.005 | 0.002 | 1.23E-02 | 0.014 | 0.003 | 2.80E-08 | TV | -0.018 | 0.007 | -0.012 | 0.009 | -0.043 | 0.021 | -0.029 | 0.018 | -0.010 | 0.030 | -0.044 | 0.018 |
| rs17411339 | 18 | 50807359 | A | G | 0.5612 | 0.014 | 0.001 | 5.10E-23 | -0.006 | 0.002 | 7.90E-03 | education | -0.003 | 0.006 | -0.007 | 0.008 | -0.021 | 0.018 | 0.010 | 0.015 | -0.021 | 0.026 | 0.006 | 0.015 |
| rs17512836 | 18 | 53194961 | T | C | 0.974 | -0.028 | 0.005 | 4.70E-07 | 0.042 | 0.007 | 7.00E-10 | TV | -0.008 | 0.020 | -0.034 | 0.025 | 0.048 | 0.056 | -0.022 | 0.047 | -0.008 | 0.079 | 0.035 | 0.049 |
| rs72944064 | 18 | 63516255 | T | C | 0.7437 | -0.010 | 0.002 | 9.39E-10 | 0.010 | 0.002 | 8.70E-05 | education | -0.006 | 0.007 | -0.008 | 0.010 | -0.014 | 0.021 | -0.011 | 0.019 | -0.027 | 0.031 | 0.000 | 0.019 |
| rs2554835 | 18 | 74141190 | A | G | 0.3998 | 0.009 | 0.001 | 8.95E-10 | -0.003 | 0.002 | 2.40E-01 | education | 0.009 | 0.007 | 0.015 | 0.009 | 0.019 | 0.019 | 0.035 | 0.017 | 0.017 | 0.028 | -0.031 | 0.017 |
| rs11081529 | 18 | 75902735 | T | C | 0.7112 | 0.013 | 0.002 | 5.22E-16 | -0.004 | 0.002 | 1.10E-01 | education | 0.026 | 0.007 | 0.019 | 0.010 | 0.062 | 0.021 | 0.026 | 0.018 | 0.028 | 0.031 | 0.025 | 0.018 |
| rs11663602 | 18 | 77578191 | A | C | 0.2761 | -0.013 | 0.002 | 9.27E-17 | 0.007 | 0.002 | 2.70E-03 | education | -0.002 | 0.007 | -0.017 | 0.010 | 0.055 | 0.021 | 0.012 | 0.018 | -0.013 | 0.031 | -0.005 | 0.019 |
| rs7248205 | 19 | 10770305 | C | T | 0.398 | -0.005 | 0.002 | 6.35E-03 | 0.014 | 0.002 | 3.40E-10 | TV | 0.012 | 0.006 | 0.012 | 0.009 | 0.030 | 0.019 | 0.021 | 0.016 | -0.012 | 0.027 | -0.022 | 0.016 |
| rs12151248 | 19 | 13212025 | T | C | 0.1141 | -0.015 | 0.002 | 3.81E-11 | 0.003 | 0.003 | 3.20E-01 | education | 0.035 | 0.010 | 0.054 | 0.013 | -0.021 | 0.029 | 0.029 | 0.025 | -0.019 | 0.043 | 0.015 | 0.025 |
| rs111901094 | 19 | 19513570 | G | T | 0.817 | 0.011 | 0.003 | 5.48E-05 | -0.017 | 0.003 | 2.00E-09 | TV | -0.032 | 0.009 | -0.038 | 0.012 | -0.012 | 0.028 | -0.024 | 0.023 | -0.092 | 0.039 | -0.064 | 0.023 |
| rs7254263 | 19 | 30741257 | T | C | 0.285 | -0.012 | 0.002 | 1.84E-14 | 0.004 | 0.002 | 6.80E-02 | education | 0.003 | 0.007 | 0.004 | 0.010 | -0.016 | 0.021 | -0.020 | 0.018 | 0.031 | 0.030 | -0.017 | 0.018 |
| rs3859523 | 19 | 32972296 | T | C | 0.8208 | 0.012 | 0.002 | 9.92E-12 | -0.006 | 0.003 | 2.40E-02 | education | -0.005 | 0.008 | -0.007 | 0.011 | -0.040 | 0.024 | -0.022 | 0.021 | 0.013 | 0.036 | 0.022 | 0.021 |
| rs173003 | 19 | 36240960 | A | C | 0.4934 | -0.008 | 0.001 | 5.50E-09 | 0.007 | 0.002 | 2.70E-03 | education | 0.011 | 0.006 | 0.020 | 0.008 | 0.007 | 0.018 | 0.000 | 0.015 | 0.009 | 0.026 | -0.013 | 0.015 |
| rs76246107 | 19 | 50121274 | A | G | 0.0941 | -0.016 | 0.003 | 6.52E-10 | 0.022 | 0.004 | 7.00E-08 | education | 0.000 | 0.012 | 0.010 | 0.016 | -0.022 | 0.036 | -0.046 | 0.031 | -0.010 | 0.053 | 0.004 | 0.031 |
| rs192436652 | 19 | 54960747 | T | C | 0.0268 | -0.032 | 0.005 | 2.06E-12 | 0.026 | 0.007 | 7.50E-05 | education | 0.017 | 0.021 | 0.014 | 0.027 | -0.022 | 0.060 | -0.032 | 0.052 | 0.043 | 0.085 | 0.072 | 0.051 |
| rs6131281 | 20 | 11891724 | C | T | 0.597 | -0.006 | 0.002 | 2.46E-04 | 0.016 | 0.002 | 3.10E-13 | TV | 0.002 | 0.007 | -0.006 | 0.009 | -0.007 | 0.019 | 0.008 | 0.016 | 0.016 | 0.027 | 0.010 | 0.016 |
| rs6141814 | 20 | 31368960 | C | A | 0.613 | 0.003 | 0.002 | 5.19E-02 | -0.014 | 0.002 | 1.30E-09 | TV | 0.004 | 0.006 | 0.005 | 0.008 | 0.029 | 0.018 | 0.000 | 0.015 | 0.006 | 0.026 | -0.006 | 0.016 |
| rs4369924 | 20 | 41954383 | A | G | 0.1615 | 0.013 | 0.002 | 5.58E-11 | -0.012 | 0.003 | 8.60E-05 | education | 0.009 | 0.008 | 0.006 | 0.011 | 0.045 | 0.024 | -0.007 | 0.021 | 0.007 | 0.035 | 0.019 | 0.021 |
| rs55700114 | 20 | 43717080 | G | A | 0.713 | 0.012 | 0.002 | 3.34E-10 | -0.014 | 0.002 | 2.10E-09 | TV | -0.002 | 0.007 | -0.007 | 0.009 | 0.035 | 0.019 | -0.019 | 0.016 | 0.018 | 0.027 | -0.008 | 0.016 |
| rs4810894 | 20 | 47532999 | A | G | 0.3706 | -0.009 | 0.002 | 4.38E-09 | 0.008 | 0.002 | 5.70E-04 | education | 0.002 | 0.006 | -0.002 | 0.009 | 0.023 | 0.019 | -0.045 | 0.016 | -0.021 | 0.027 | 0.018 | 0.016 |
| rs6123924 | 20 | 58219764 | A | G | 0.8445 | 0.013 | 0.002 | 3.20E-11 | -0.010 | 0.003 | 8.20E-04 | education | 0.025 | 0.009 | 0.012 | 0.011 | 0.032 | 0.025 | 0.003 | 0.021 | 0.033 | 0.037 | 0.023 | 0.022 |
| rs6065080 | 20 | 59832791 | T | C | 0.3589 | -0.013 | 0.002 | 7.49E-19 | 0.004 | 0.002 | 5.80E-02 | education | -0.001 | 0.006 | 0.005 | 0.008 | -0.003 | 0.019 | -0.012 | 0.016 | 0.015 | 0.027 | -0.002 | 0.016 |
| rs56103247 | 20 | 62483184 | C | T | 0.944 | -0.016 | 0.004 | 3.14E-05 | 0.030 | 0.005 | 3.80E-10 | TV | -0.006 | 0.015 | -0.007 | 0.020 | 0.020 | 0.045 | -0.013 | 0.037 | -0.068 | 0.061 | 0.014 | 0.038 |
| rs13050131 | 21 | 20018118 | A | C | 0.6647 | -0.008 | 0.002 | 4.40E-08 | 0.006 | 0.002 | 1.40E-02 | education | -0.006 | 0.006 | -0.001 | 0.008 | -0.012 | 0.019 | -0.019 | 0.016 | -0.018 | 0.027 | -0.005 | 0.016 |
| rs232496 | 21 | 22734409 | T | C | 0.3657 | 0.009 | 0.002 | 7.14E-10 | -0.003 | 0.002 | 2.00E-01 | education | -0.010 | 0.006 | -0.002 | 0.008 | -0.033 | 0.018 | 0.002 | 0.015 | 0.005 | 0.026 | -0.018 | 0.016 |
| rs1544 | 21 | 33085585 | A | G | 0.2636 | -0.010 | 0.002 | 1.76E-09 | 0.007 | 0.002 | 4.90E-03 | education | -0.007 | 0.007 | -0.009 | 0.009 | 0.007 | 0.021 | -0.029 | 0.018 | -0.059 | 0.030 | 0.037 | 0.018 |
| rs10427502 | 21 | 40654840 | G | A | 0.622 | -0.004 | 0.002 | 1.36E-02 | 0.014 | 0.002 | 8.60E-10 | TV | -0.012 | 0.006 | -0.013 | 0.008 | -0.003 | 0.019 | -0.021 | 0.016 | -0.067 | 0.027 | -0.014 | 0.016 |
| rs2838006 | 21 | 42653567 | T | C | 0.3597 | 0.013 | 0.002 | 1.38E-19 | -0.006 | 0.002 | 5.10E-03 | education | -0.001 | 0.006 | -0.003 | 0.008 | -0.013 | 0.018 | 0.016 | 0.016 | 0.034 | 0.027 | 0.006 | 0.016 |
| rs2092829 | 22 | 31780514 | G | A | 0.706 | -0.002 | 0.002 | 3.89E-01 | 0.014 | 0.002 | 1.30E-08 | TV | -0.002 | 0.007 | -0.010 | 0.009 | -0.006 | 0.020 | -0.014 | 0.017 | -0.001 | 0.028 | 0.010 | 0.017 |
| rs5754753 | 22 | 34296751 | T | C | 0.7178 | -0.013 | 0.002 | 6.15E-16 | 0.009 | 0.002 | 1.40E-04 | education | 0.006 | 0.007 | 0.006 | 0.010 | 0.021 | 0.021 | 0.011 | 0.018 | -0.033 | 0.030 | 0.014 | 0.018 |
| rs143743568 | 22 | 39289014 | A | G | 0.154 | 0.012 | 0.002 | 4.99E-09 | -0.008 | 0.003 | 9.40E-03 | education | 0.031 | 0.009 | 0.044 | 0.011 | 0.023 | 0.025 | 0.004 | 0.022 | -0.042 | 0.037 | 0.048 | 0.022 |
| rs137079 | 22 | 42977907 | T | C | 0.1375 | 0.013 | 0.002 | 3.06E-10 | -0.016 | 0.003 | 1.90E-07 | education | -0.007 | 0.009 | -0.016 | 0.012 | -0.006 | 0.026 | 0.028 | 0.022 | -0.016 | 0.038 | -0.005 | 0.023 |
| rs9616947 | 22 | 51151631 | T | C | 0.3818 | -0.013 | 0.002 | 3.58E-20 | 0.004 | 0.002 | 6.60E-02 | education | -0.004 | 0.007 | -0.005 | 0.009 | 0.033 | 0.020 | -0.024 | 0.017 | -0.009 | 0.029 | -0.040 | 0.017 |

| **Table S21. Beta estimates for the SNPs included in the multivariable MR of years of education, leisure television watching, and colorectal cancer risk** | | | | | | | | | | | | | | | | | | | | | |
| --- | --- | --- | --- | --- | --- | --- | --- | --- | --- | --- | --- | --- | --- | --- | --- | --- | --- | --- | --- | --- | --- |
| SNP | CHR | POS | EA | OA | beta_ed | se_ed | pval_ed | beta_tv | se_tv | pval_tv | Phenotype | beta_crc | se_crc | beta_crc_men | se_crc_men | beta_crc_women | se_crc_women | beta_co | se_co | beta_re | se_re |
| rs34394051 | 1 | 6853091 | A | G | -0.012 | 0.002 | 2.30E-09 | 0.009 | 0.003 | 2.50E-03 | education | 0.018 | 0.013 | 0.005 | 0.019 | 0.031 | 0.019 | 0.021 | 0.016 | -0.005 | 0.021 |
| rs11121177 | 1 | 8445946 | A | G | 0.015 | 0.002 | 1.44E-16 | -0.009 | 0.003 | 2.00E-03 | education | 0.010 | 0.012 | 0.001 | 0.018 | 0.016 | 0.018 | -0.005 | 0.015 | 0.015 | 0.019 |
| rs10799615 | 1 | 20541370 | A | G | -0.009 | 0.002 | 4.11E-08 | 0.008 | 0.002 | 2.20E-03 | education | 0.001 | 0.011 | 0.006 | 0.016 | -0.002 | 0.016 | 0.008 | 0.013 | -0.001 | 0.017 |
| rs590013 | 1 | 29155738 | T | C | 0.010 | 0.002 | 2.47E-11 | -0.004 | 0.002 | 1.10E-01 | education | 0.008 | 0.010 | 0.010 | 0.015 | 0.004 | 0.014 | 0.007 | 0.012 | 0.003 | 0.016 |
| rs10798888 | 1 | 32199099 | T | G | -0.014 | 0.002 | 5.15E-14 | 0.013 | 0.003 | 4.30E-06 | education | -0.010 | 0.013 | -0.022 | 0.018 | 0.001 | 0.018 | -0.014 | 0.015 | -0.020 | 0.020 |
| rs11810109 | 1 | 44186812 | A | T | -0.019 | 0.002 | 1.16E-23 | 0.016 | 0.002 | 5.40E-12 | TV | 0.011 | 0.011 | 0.000 | 0.015 | 0.022 | 0.015 | 0.004 | 0.013 | 0.016 | 0.017 |
| rs1738050 | 1 | 44707295 | C | G | -0.009 | 0.001 | 1.42E-10 | 0.003 | 0.002 | 1.90E-01 | education | 0.026 | 0.010 | 0.038 | 0.014 | 0.016 | 0.014 | 0.023 | 0.012 | 0.017 | 0.016 |
| rs11211123 | 1 | 45957609 | A | G | 0.010 | 0.002 | 7.62E-10 | 0.000 | 0.003 | 9.70E-01 | education | 0.008 | 0.011 | 0.012 | 0.016 | 0.000 | 0.016 | 0.008 | 0.014 | -0.003 | 0.018 |
| rs2989476 | 1 | 61059259 | C | G | 0.008 | 0.001 | 3.73E-08 | -0.012 | 0.002 | 1.00E-07 | education | -0.005 | 0.010 | -0.019 | 0.014 | 0.006 | 0.014 | -0.005 | 0.012 | -0.010 | 0.015 |
| rs12725114 | 1 | 62432641 | G | A | -0.004 | 0.002 | 6.55E-02 | 0.015 | 0.003 | 1.60E-08 | TV | -0.016 | 0.012 | -0.023 | 0.017 | -0.007 | 0.017 | 0.003 | 0.014 | -0.060 | 0.018 |
| rs984409 | 1 | 67020440 | G | A | 0.005 | 0.002 | 3.60E-03 | -0.015 | 0.002 | 4.10E-11 | TV | -0.019 | 0.010 | -0.010 | 0.015 | -0.028 | 0.015 | -0.011 | 0.012 | -0.030 | 0.016 |
| rs71658797 | 1 | 77967507 | T | A | 0.010 | 0.003 | 1.02E-04 | -0.020 | 0.003 | 6.30E-10 | TV | -0.005 | 0.016 | -0.002 | 0.023 | -0.009 | 0.023 | -0.009 | 0.019 | -0.009 | 0.025 |
| rs114328297 | 1 | 91190854 | T | G | -0.011 | 0.002 | 3.52E-07 | 0.014 | 0.003 | 4.40E-08 | TV | 0.002 | 0.012 | 0.018 | 0.017 | -0.018 | 0.018 | 0.016 | 0.015 | -0.056 | 0.019 |
| rs6690195 | 1 | 96221653 | T | C | -0.011 | 0.001 | 7.89E-16 | 0.009 | 0.002 | 5.50E-05 | education | -0.017 | 0.010 | -0.006 | 0.014 | -0.025 | 0.014 | -0.024 | 0.012 | -0.006 | 0.016 |
| rs17379561 | 1 | 98340139 | A | T | 0.019 | 0.002 | 1.73E-15 | -0.025 | 0.003 | 1.10E-16 | TV | 0.016 | 0.014 | 0.004 | 0.020 | 0.024 | 0.020 | 0.016 | 0.017 | 0.016 | 0.022 |
| rs55909997 | 1 | 107581039 | G | A | 0.006 | 0.002 | 6.29E-04 | -0.014 | 0.002 | 2.40E-09 | TV | -0.027 | 0.010 | -0.037 | 0.014 | -0.017 | 0.014 | -0.011 | 0.012 | -0.033 | 0.016 |
| rs1278847 | 1 | 110037483 | C | A | -0.012 | 0.002 | 7.79E-11 | 0.016 | 0.002 | 1.20E-11 | TV | -0.006 | 0.011 | 0.008 | 0.015 | -0.018 | 0.015 | -0.006 | 0.013 | -0.004 | 0.017 |
| rs4839155 | 1 | 112161489 | T | G | 0.012 | 0.002 | 4.66E-12 | -0.008 | 0.003 | 1.10E-03 | education | -0.021 | 0.011 | -0.023 | 0.016 | -0.021 | 0.016 | -0.016 | 0.014 | -0.033 | 0.018 |
| rs4845364 | 1 | 154141908 | A | G | 0.002 | 0.002 | 2.84E-01 | -0.015 | 0.002 | 1.30E-12 | TV | -0.006 | 0.010 | -0.014 | 0.014 | 0.001 | 0.014 | -0.008 | 0.012 | -0.013 | 0.015 |
| rs34720381 | 1 | 171455322 | T | C | -0.018 | 0.002 | 1.90E-13 | 0.016 | 0.004 | 2.70E-05 | education | 0.036 | 0.017 | 0.008 | 0.024 | 0.063 | 0.025 | 0.035 | 0.021 | 0.044 | 0.027 |
| rs6673341 | 1 | 184655649 | T | G | 0.007 | 0.002 | 1.51E-05 | -0.015 | 0.002 | 2.20E-11 | TV | 0.000 | 0.010 | -0.012 | 0.014 | 0.012 | 0.014 | 0.002 | 0.012 | -0.014 | 0.015 |
| rs10737620 | 1 | 193057971 | T | A | -0.007 | 0.002 | 3.12E-04 | 0.014 | 0.002 | 2.60E-09 | TV | -0.010 | 0.011 | -0.008 | 0.015 | -0.011 | 0.015 | 0.000 | 0.013 | -0.024 | 0.017 |
| rs1890132 | 1 | 199427321 | T | C | -0.009 | 0.002 | 6.56E-09 | 0.003 | 0.002 | 2.60E-01 | education | 0.002 | 0.011 | 0.011 | 0.015 | -0.008 | 0.015 | -0.007 | 0.013 | 0.014 | 0.017 |
| rs8024 | 1 | 201845575 | A | C | -0.012 | 0.002 | 1.13E-14 | 0.010 | 0.002 | 7.30E-06 | education | 0.026 | 0.010 | 0.029 | 0.015 | 0.025 | 0.015 | 0.033 | 0.013 | 0.005 | 0.016 |
| rs11588857 | 1 | 204587047 | A | G | 0.020 | 0.002 | 4.45E-31 | -0.012 | 0.003 | 1.30E-05 | education | -0.019 | 0.012 | -0.018 | 0.016 | -0.020 | 0.016 | -0.039 | 0.014 | -0.014 | 0.018 |
| rs3111251 | 1 | 211409844 | T | C | -0.009 | 0.001 | 3.88E-10 | 0.003 | 0.002 | 2.40E-01 | education | -0.002 | 0.010 | 0.004 | 0.014 | -0.007 | 0.014 | 0.003 | 0.012 | 0.008 | 0.015 |
| rs4846724 | 1 | 221967817 | A | G | 0.010 | 0.001 | 2.89E-12 | -0.006 | 0.002 | 8.30E-03 | education | 0.007 | 0.010 | 0.002 | 0.014 | 0.013 | 0.014 | 0.000 | 0.012 | 0.005 | 0.015 |
| rs1329125 | 1 | 234740880 | T | C | -0.010 | 0.002 | 8.37E-11 | 0.008 | 0.002 | 6.20E-04 | education | 0.028 | 0.011 | 0.035 | 0.015 | 0.019 | 0.015 | 0.028 | 0.013 | 0.042 | 0.016 |
| rs114593137 | 1 | 241876565 | A | T | -0.012 | 0.002 | 1.65E-12 | 0.014 | 0.003 | 3.20E-07 | education | 0.004 | 0.012 | 0.001 | 0.017 | 0.011 | 0.017 | 0.007 | 0.014 | -0.006 | 0.019 |
| rs3897821 | 1 | 243420388 | A | G | 0.015 | 0.002 | 3.58E-25 | -0.007 | 0.002 | 2.00E-03 | education | -0.011 | 0.010 | -0.026 | 0.014 | 0.005 | 0.014 | -0.009 | 0.012 | -0.018 | 0.016 |
| rs12614263 | 2 | 4123797 | A | G | 0.008 | 0.001 | 3.38E-09 | -0.004 | 0.002 | 8.60E-02 | education | -0.002 | 0.010 | -0.010 | 0.014 | 0.005 | 0.014 | -0.005 | 0.012 | -0.010 | 0.015 |
| rs7603132 | 2 | 4951548 | A | G | 0.014 | 0.002 | 1.20E-14 | -0.009 | 0.003 | 1.00E-03 | education | -0.019 | 0.012 | -0.024 | 0.017 | -0.013 | 0.017 | -0.026 | 0.015 | -0.017 | 0.019 |
| rs76076331 | 2 | 10977585 | T | C | 0.021 | 0.002 | 1.71E-23 | -0.015 | 0.003 | 6.60E-06 | education | -0.005 | 0.015 | -0.022 | 0.021 | 0.010 | 0.021 | -0.006 | 0.018 | 0.003 | 0.023 |
| rs1991585 | 2 | 16647170 | T | C | -0.010 | 0.002 | 6.26E-11 | 0.009 | 0.002 | 1.20E-04 | education | 0.011 | 0.010 | -0.013 | 0.015 | 0.033 | 0.015 | 0.014 | 0.013 | 0.018 | 0.017 |
| rs72781699 | 2 | 24277709 | G | A | 0.006 | 0.002 | 2.99E-03 | -0.019 | 0.003 | 3.00E-12 | TV | 0.009 | 0.012 | 0.008 | 0.017 | 0.008 | 0.017 | -0.012 | 0.014 | 0.042 | 0.019 |
| rs12105701 | 2 | 41737200 | C | T | 0.005 | 0.002 | 2.45E-03 | -0.013 | 0.002 | 5.40E-09 | TV | -0.009 | 0.010 | 0.013 | 0.014 | -0.030 | 0.014 | -0.007 | 0.012 | -0.017 | 0.015 |
| rs7564130 | 2 | 50606642 | T | C | 0.009 | 0.002 | 1.49E-06 | -0.015 | 0.002 | 2.80E-11 | TV | -0.021 | 0.010 | -0.019 | 0.014 | -0.022 | 0.014 | -0.032 | 0.012 | -0.002 | 0.016 |
| rs10189857 | 2 | 60713235 | A | G | 0.016 | 0.001 | 4.69E-29 | -0.020 | 0.002 | 6.20E-21 | both | -0.022 | 0.010 | -0.022 | 0.014 | -0.021 | 0.014 | -0.020 | 0.012 | -0.019 | 0.015 |
| rs62641636 | 2 | 68425427 | A | G | -0.011 | 0.002 | 1.06E-09 | 0.014 | 0.002 | 7.00E-10 | TV | 0.010 | 0.010 | 0.000 | 0.015 | 0.018 | 0.015 | 0.022 | 0.012 | 0.015 | 0.016 |
| rs112806496 | 2 | 80421805 | C | G | -0.016 | 0.003 | 1.72E-10 | 0.014 | 0.004 | 1.50E-04 | education | -0.017 | 0.018 | -0.023 | 0.025 | -0.014 | 0.025 | -0.026 | 0.021 | 0.005 | 0.028 |
| rs11894424 | 2 | 80588091 | A | C | 0.021 | 0.003 | 2.14E-10 | -0.002 | 0.005 | 7.50E-01 | education | -0.005 | 0.020 | -0.007 | 0.029 | -0.003 | 0.029 | 0.021 | 0.026 | -0.028 | 0.033 |
| rs11689199 | 2 | 100820421 | A | G | -0.020 | 0.002 | 1.17E-29 | 0.018 | 0.002 | 5.50E-17 | TV | 0.025 | 0.010 | 0.039 | 0.014 | 0.012 | 0.014 | 0.020 | 0.012 | 0.034 | 0.016 |
| rs1451533 | 2 | 105466005 | G | A | 0.000 | 0.002 | 8.43E-01 | -0.016 | 0.002 | 1.10E-10 | TV | 0.009 | 0.011 | 0.009 | 0.015 | 0.011 | 0.015 | 0.002 | 0.013 | 0.013 | 0.017 |
| rs13402497 | 2 | 115826318 | A | T | -0.008 | 0.001 | 1.33E-08 | -0.002 | 0.002 | 2.80E-01 | education | 0.008 | 0.010 | -0.002 | 0.014 | 0.017 | 0.014 | -0.003 | 0.012 | 0.024 | 0.015 |
| rs12476388 | 2 | 116330983 | C | T | 0.000 | 0.002 | 8.32E-01 | 0.013 | 0.002 | 2.10E-08 | TV | -0.002 | 0.010 | -0.005 | 0.015 | 0.000 | 0.015 | -0.012 | 0.013 | 0.001 | 0.017 |
| rs7575637 | 2 | 145796251 | A | G | 0.011 | 0.001 | 3.43E-14 | -0.003 | 0.002 | 1.20E-01 | education | -0.013 | 0.010 | -0.019 | 0.014 | -0.005 | 0.014 | -0.012 | 0.012 | -0.022 | 0.015 |
| rs374722 | 2 | 147839830 | G | A | -0.006 | 0.002 | 8.51E-03 | 0.024 | 0.003 | 5.50E-16 | TV | 0.026 | 0.013 | 0.002 | 0.019 | 0.049 | 0.019 | 0.038 | 0.016 | 0.017 | 0.021 |
| rs3754970 | 2 | 162091836 | T | C | 0.016 | 0.002 | 3.53E-20 | -0.015 | 0.002 | 4.80E-12 | TV | 0.006 | 0.010 | 0.003 | 0.014 | 0.007 | 0.014 | 0.011 | 0.012 | -0.011 | 0.015 |
| rs2060199 | 2 | 166196681 | T | A | -0.003 | 0.002 | 6.82E-02 | 0.012 | 0.002 | 2.40E-08 | TV | 0.016 | 0.010 | 0.039 | 0.014 | -0.002 | 0.014 | 0.012 | 0.012 | 0.028 | 0.015 |
| rs17428076 | 2 | 172851936 | C | G | 0.014 | 0.002 | 6.41E-17 | -0.007 | 0.003 | 5.40E-03 | education | 0.019 | 0.011 | 0.018 | 0.016 | 0.020 | 0.016 | 0.018 | 0.013 | 0.014 | 0.018 |
| rs10193498 | 2 | 174094345 | A | T | 0.010 | 0.002 | 3.10E-09 | -0.007 | 0.003 | 5.00E-03 | education | -0.009 | 0.011 | -0.023 | 0.015 | 0.000 | 0.015 | -0.001 | 0.013 | -0.029 | 0.017 |
| rs72917504 | 2 | 175225018 | T | C | -0.018 | 0.003 | 2.91E-09 | 0.007 | 0.005 | 1.10E-01 | education | 0.002 | 0.021 | 0.028 | 0.030 | -0.024 | 0.029 | 0.003 | 0.025 | -0.015 | 0.032 |
| rs4577309 | 2 | 191288833 | A | G | -0.005 | 0.002 | 3.33E-03 | 0.016 | 0.002 | 1.60E-13 | TV | 0.001 | 0.010 | 0.015 | 0.014 | -0.012 | 0.014 | 0.004 | 0.012 | 0.009 | 0.015 |
| rs34224261 | 2 | 193855452 | G | C | 0.011 | 0.002 | 3.79E-11 | -0.018 | 0.002 | 4.60E-16 | TV | 0.001 | 0.010 | -0.002 | 0.014 | 0.005 | 0.014 | -0.002 | 0.012 | 0.000 | 0.015 |
| rs4675246 | 2 | 202864487 | G | T | 0.009 | 0.002 | 5.48E-05 | -0.015 | 0.003 | 2.90E-08 | TV | 0.037 | 0.013 | 0.043 | 0.018 | 0.031 | 0.018 | 0.028 | 0.015 | 0.047 | 0.020 |
| rs13029509 | 2 | 215374209 | G | A | 0.010 | 0.002 | 7.17E-10 | -0.018 | 0.002 | 2.00E-17 | TV | -0.005 | 0.010 | -0.007 | 0.014 | -0.004 | 0.014 | -0.001 | 0.012 | -0.011 | 0.015 |
| rs10932837 | 2 | 221044785 | C | T | 0.002 | 0.002 | 1.52E-01 | -0.013 | 0.002 | 1.30E-09 | TV | -0.003 | 0.010 | 0.013 | 0.014 | -0.020 | 0.014 | -0.005 | 0.012 | -0.016 | 0.015 |
| rs1542354 | 2 | 221814085 | A | G | -0.008 | 0.001 | 2.72E-09 | 0.005 | 0.002 | 1.20E-02 | education | 0.002 | 0.010 | 0.007 | 0.014 | -0.005 | 0.014 | -0.008 | 0.012 | 0.021 | 0.016 |
| rs4973576 | 2 | 233806771 | C | A | 0.008 | 0.002 | 7.60E-06 | -0.015 | 0.002 | 1.00E-09 | TV | 0.013 | 0.011 | -0.005 | 0.015 | 0.034 | 0.015 | 0.013 | 0.013 | -0.001 | 0.017 |
| rs4663617 | 2 | 236744626 | A | T | 0.010 | 0.002 | 4.12E-10 | -0.007 | 0.003 | 4.30E-03 | education | -0.019 | 0.012 | -0.011 | 0.016 | -0.026 | 0.016 | -0.021 | 0.014 | -0.010 | 0.018 |
| rs72993796 | 2 | 240321051 | T | C | 0.013 | 0.002 | 1.12E-08 | -0.005 | 0.003 | 1.10E-01 | education | -0.011 | 0.017 | -0.023 | 0.024 | 0.006 | 0.024 | -0.017 | 0.020 | -0.014 | 0.026 |
| rs17048855 | 3 | 8258174 | A | G | 0.011 | 0.002 | 3.10E-13 | -0.004 | 0.002 | 6.20E-02 | education | -0.009 | 0.010 | -0.017 | 0.014 | -0.003 | 0.014 | 0.000 | 0.012 | -0.008 | 0.016 |
| rs138256022 | 3 | 11603090 | C | T | 0.011 | 0.004 | 1.06E-02 | -0.031 | 0.006 | 2.90E-08 | TV | 0.043 | 0.031 | 0.025 | 0.045 | 0.065 | 0.044 | 0.031 | 0.037 | 0.046 | 0.049 |
| rs13085461 | 3 | 24950387 | C | G | 0.010 | 0.001 | 2.40E-13 | -0.003 | 0.002 | 2.00E-01 | education | -0.004 | 0.010 | 0.008 | 0.014 | -0.011 | 0.014 | -0.004 | 0.012 | 0.000 | 0.015 |
| rs73055556 | 3 | 28027538 | A | G | 0.011 | 0.002 | 4.95E-08 | -0.005 | 0.003 | 1.10E-01 | education | -0.029 | 0.014 | -0.036 | 0.020 | -0.021 | 0.019 | -0.025 | 0.017 | -0.028 | 0.022 |
| rs9834970 | 3 | 36856030 | T | C | -0.008 | 0.002 | 7.65E-07 | 0.013 | 0.002 | 3.30E-09 | TV | 0.013 | 0.010 | 0.002 | 0.014 | 0.024 | 0.014 | 0.010 | 0.012 | 0.017 | 0.015 |
| rs3796386 | 3 | 49899795 | G | A | 0.026 | 0.002 | 7.19E-51 | -0.026 | 0.002 | 3.20E-33 | TV | -0.015 | 0.010 | -0.014 | 0.014 | -0.018 | 0.014 | -0.012 | 0.012 | -0.040 | 0.015 |
| rs11130793 | 3 | 60884659 | C | T | -0.005 | 0.002 | 7.56E-03 | 0.013 | 0.002 | 5.00E-09 | TV | 0.008 | 0.010 | 0.018 | 0.014 | -0.003 | 0.014 | -0.004 | 0.012 | 0.011 | 0.016 |
| rs11714337 | 3 | 71582521 | G | A | -0.013 | 0.002 | 7.66E-15 | 0.014 | 0.002 | 4.70E-11 | TV | -0.021 | 0.010 | -0.010 | 0.014 | -0.029 | 0.014 | -0.029 | 0.012 | -0.011 | 0.015 |
| rs6797840 | 3 | 85656569 | A | C | 0.010 | 0.002 | 1.29E-08 | -0.016 | 0.002 | 1.70E-13 | TV | 0.009 | 0.010 | -0.004 | 0.014 | 0.018 | 0.014 | 0.016 | 0.012 | 0.015 | 0.015 |
| rs2034768 | 3 | 93986371 | A | G | -0.006 | 0.002 | 2.08E-04 | 0.015 | 0.002 | 8.60E-12 | TV | -0.013 | 0.010 | -0.017 | 0.014 | -0.007 | 0.014 | -0.014 | 0.012 | -0.007 | 0.015 |
| rs9867121 | 3 | 114631548 | C | A | -0.004 | 0.002 | 9.97E-02 | 0.019 | 0.003 | 3.90E-12 | TV | 0.020 | 0.012 | 0.041 | 0.017 | -0.004 | 0.017 | 0.018 | 0.015 | 0.014 | 0.019 |
| rs34067381 | 3 | 123594419 | T | G | -0.010 | 0.002 | 9.77E-13 | 0.011 | 0.002 | 2.30E-06 | education | -0.019 | 0.010 | -0.024 | 0.014 | -0.014 | 0.014 | -0.016 | 0.012 | -0.015 | 0.016 |
| rs9289300 | 3 | 127144988 | T | C | -0.016 | 0.002 | 1.00E-17 | 0.006 | 0.003 | 3.40E-02 | education | 0.005 | 0.013 | 0.026 | 0.019 | -0.016 | 0.019 | 0.004 | 0.016 | 0.013 | 0.020 |
| rs7650602 | 3 | 141147414 | T | C | -0.009 | 0.001 | 2.77E-10 | 0.006 | 0.002 | 7.40E-03 | education | -0.004 | 0.010 | -0.011 | 0.014 | 0.002 | 0.014 | -0.004 | 0.012 | -0.005 | 0.015 |
| rs2885198 | 3 | 143636421 | A | G | 0.009 | 0.001 | 4.63E-10 | -0.009 | 0.002 | 1.40E-05 | education | -0.020 | 0.010 | 0.001 | 0.014 | -0.037 | 0.014 | -0.019 | 0.012 | -0.026 | 0.016 |
| rs12054166 | 3 | 150093445 | C | G | 0.010 | 0.002 | 2.30E-09 | 0.004 | 0.002 | 1.30E-01 | education | 0.013 | 0.011 | 0.021 | 0.016 | 0.007 | 0.016 | 0.005 | 0.013 | 0.031 | 0.017 |
| rs12491503 | 3 | 165706855 | G | A | 0.007 | 0.002 | 1.41E-04 | -0.014 | 0.002 | 5.50E-10 | TV | -0.001 | 0.010 | -0.011 | 0.014 | 0.008 | 0.014 | -0.005 | 0.012 | -0.006 | 0.016 |
| rs77025239 | 3 | 180734185 | A | G | -0.014 | 0.002 | 4.65E-13 | 0.004 | 0.003 | 1.60E-01 | education | 0.012 | 0.013 | 0.018 | 0.019 | 0.007 | 0.019 | 0.019 | 0.016 | 0.010 | 0.021 |
| rs114600294 | 3 | 181419367 | G | C | 0.011 | 0.002 | 3.21E-06 | -0.016 | 0.003 | 7.90E-10 | TV | 0.013 | 0.014 | 0.022 | 0.020 | 0.005 | 0.020 | 0.021 | 0.017 | 0.007 | 0.022 |
| rs13327482 | 3 | 196788363 | A | G | -0.012 | 0.002 | 4.47E-11 | 0.005 | 0.003 | 9.60E-02 | education | 0.013 | 0.013 | 0.008 | 0.018 | 0.007 | 0.018 | 0.012 | 0.015 | 0.015 | 0.020 |
| rs66852340 | 4 | 3311070 | C | T | 0.013 | 0.002 | 1.94E-10 | -0.018 | 0.003 | 7.90E-12 | TV | -0.004 | 0.011 | -0.013 | 0.016 | 0.002 | 0.016 | 0.000 | 0.014 | -0.030 | 0.018 |
| rs13133213 | 4 | 15646794 | A | G | 0.010 | 0.001 | 7.57E-12 | 0.005 | 0.002 | 1.20E-02 | education | 0.003 | 0.010 | 0.004 | 0.013 | 0.002 | 0.014 | -0.003 | 0.011 | -0.010 | 0.015 |
| rs7700107 | 4 | 17880416 | A | C | 0.007 | 0.003 | 5.27E-03 | -0.021 | 0.003 | 1.90E-11 | TV | -0.003 | 0.014 | -0.025 | 0.019 | 0.017 | 0.019 | 0.018 | 0.017 | -0.011 | 0.022 |
| rs10021733 | 4 | 28609476 | T | C | -0.016 | 0.003 | 9.45E-10 | -0.001 | 0.003 | 6.10E-01 | education | 0.020 | 0.014 | 0.021 | 0.019 | 0.021 | 0.019 | 0.006 | 0.016 | 0.047 | 0.021 |
| rs77215114 | 4 | 45187658 | A | T | -0.008 | 0.003 | 1.74E-02 | 0.024 | 0.004 | 1.50E-08 | TV | 0.011 | 0.019 | -0.017 | 0.027 | 0.036 | 0.027 | 0.031 | 0.024 | 0.017 | 0.032 |
| rs1505676 | 4 | 62127291 | C | G | 0.008 | 0.002 | 6.67E-09 | -0.008 | 0.002 | 4.20E-04 | education | 0.009 | 0.010 | 0.016 | 0.014 | -0.001 | 0.014 | 0.009 | 0.012 | 0.011 | 0.016 |
| rs12506221 | 4 | 67899235 | T | G | -0.013 | 0.001 | 4.23E-20 | 0.004 | 0.002 | 8.40E-02 | education | -0.006 | 0.010 | -0.030 | 0.014 | 0.019 | 0.014 | -0.008 | 0.012 | -0.005 | 0.015 |
| rs6850494 | 4 | 82291771 | A | C | 0.005 | 0.002 | 8.27E-03 | -0.014 | 0.002 | 1.10E-10 | TV | -0.011 | 0.010 | -0.019 | 0.014 | -0.002 | 0.014 | -0.029 | 0.012 | -0.008 | 0.016 |
| rs13107325 | 4 | 103188709 | C | T | 0.019 | 0.003 | 4.45E-08 | -0.029 | 0.004 | 1.50E-12 | TV | 0.008 | 0.019 | -0.009 | 0.026 | 0.028 | 0.027 | 0.012 | 0.022 | -0.006 | 0.029 |
| rs7693703 | 4 | 118357405 | G | A | -0.008 | 0.003 | 5.22E-03 | 0.023 | 0.004 | 2.70E-09 | TV | 0.022 | 0.017 | -0.004 | 0.024 | 0.050 | 0.024 | 0.018 | 0.020 | 0.070 | 0.027 |
| rs4334769 | 4 | 130245233 | G | T | 0.000 | 0.002 | 8.58E-01 | 0.012 | 0.002 | 2.10E-08 | TV | -0.015 | 0.010 | -0.010 | 0.014 | -0.016 | 0.014 | -0.023 | 0.012 | -0.015 | 0.015 |
| rs10006235 | 4 | 130670108 | T | C | -0.009 | 0.002 | 4.48E-09 | 0.004 | 0.002 | 7.20E-02 | education | 0.000 | 0.011 | 0.003 | 0.015 | -0.004 | 0.015 | 0.011 | 0.013 | 0.006 | 0.017 |
| rs12643771 | 4 | 140753103 | T | C | 0.013 | 0.002 | 1.32E-17 | -0.009 | 0.002 | 1.30E-04 | education | -0.019 | 0.011 | -0.008 | 0.015 | -0.030 | 0.015 | -0.023 | 0.013 | -0.032 | 0.017 |
| rs6825241 | 4 | 152543783 | C | A | 0.009 | 0.002 | 2.82E-07 | -0.017 | 0.002 | 4.90E-15 | TV | -0.008 | 0.010 | -0.003 | 0.014 | -0.012 | 0.014 | -0.005 | 0.012 | -0.016 | 0.015 |
| rs7693082 | 4 | 159857819 | G | C | -0.013 | 0.002 | 4.05E-13 | 0.015 | 0.002 | 1.70E-10 | TV | 0.017 | 0.011 | 0.012 | 0.015 | 0.020 | 0.015 | 0.026 | 0.013 | 0.020 | 0.017 |
| rs12646523 | 4 | 170944698 | T | C | -0.013 | 0.002 | 3.79E-16 | 0.005 | 0.002 | 3.10E-02 | education | -0.008 | 0.011 | 0.000 | 0.016 | -0.017 | 0.016 | 0.001 | 0.013 | -0.033 | 0.018 |
| rs17598675 | 4 | 176647637 | T | C | -0.012 | 0.001 | 1.37E-16 | 0.002 | 0.002 | 4.90E-01 | education | 0.011 | 0.010 | 0.011 | 0.014 | 0.012 | 0.014 | 0.007 | 0.012 | 0.027 | 0.015 |
| rs72709560 | 4 | 178540163 | A | G | 0.009 | 0.002 | 2.03E-08 | -0.005 | 0.002 | 1.70E-02 | education | -0.016 | 0.010 | 0.008 | 0.014 | -0.038 | 0.014 | -0.013 | 0.012 | 0.002 | 0.016 |
| rs1128956 | 4 | 183724005 | T | G | -0.014 | 0.002 | 5.34E-14 | 0.010 | 0.003 | 3.00E-04 | education | 0.003 | 0.013 | -0.005 | 0.018 | 0.013 | 0.018 | -0.008 | 0.015 | 0.009 | 0.020 |
| rs11724690 | 4 | 186764747 | T | G | 0.009 | 0.002 | 2.08E-09 | -0.006 | 0.002 | 2.10E-02 | education | 0.017 | 0.011 | 0.024 | 0.015 | 0.007 | 0.015 | 0.019 | 0.013 | -0.001 | 0.017 |
| rs13163845 | 5 | 3264389 | T | C | -0.015 | 0.002 | 4.66E-14 | 0.007 | 0.003 | 2.30E-02 | education | -0.008 | 0.014 | -0.002 | 0.019 | -0.017 | 0.020 | 0.005 | 0.017 | -0.040 | 0.022 |
| rs162445 | 5 | 7943246 | A | G | 0.015 | 0.003 | 5.72E-09 | -0.007 | 0.004 | 1.00E-01 | education | -0.031 | 0.017 | -0.028 | 0.024 | -0.038 | 0.025 | -0.045 | 0.021 | -0.072 | 0.028 |
| rs2434672 | 5 | 11471879 | A | C | 0.009 | 0.001 | 1.06E-10 | -0.004 | 0.002 | 4.40E-02 | education | -0.008 | 0.010 | 0.009 | 0.014 | -0.022 | 0.014 | -0.008 | 0.012 | -0.007 | 0.015 |
| rs17563464 | 5 | 26913774 | A | C | -0.015 | 0.002 | 1.74E-17 | 0.006 | 0.003 | 2.20E-02 | education | -0.003 | 0.013 | 0.008 | 0.018 | -0.015 | 0.018 | -0.009 | 0.015 | 0.003 | 0.020 |
| rs10940921 | 5 | 30808645 | T | G | 0.011 | 0.001 | 1.31E-14 | -0.009 | 0.002 | 3.40E-05 | education | 0.002 | 0.010 | -0.013 | 0.014 | 0.015 | 0.014 | -0.001 | 0.012 | 0.022 | 0.015 |
| rs2964199 | 5 | 57532775 | T | C | -0.010 | 0.002 | 4.61E-11 | 0.008 | 0.002 | 3.40E-04 | education | -0.008 | 0.011 | 0.005 | 0.015 | -0.020 | 0.015 | -0.010 | 0.013 | 0.005 | 0.017 |
| rs6449503 | 5 | 60095272 | A | G | 0.019 | 0.001 | 1.84E-39 | -0.015 | 0.002 | 1.40E-11 | education | -0.001 | 0.010 | 0.002 | 0.014 | -0.004 | 0.014 | -0.002 | 0.012 | 0.000 | 0.015 |
| rs262890 | 5 | 62930015 | A | G | 0.010 | 0.002 | 8.30E-08 | -0.019 | 0.002 | 3.20E-15 | TV | 0.031 | 0.011 | 0.036 | 0.015 | 0.028 | 0.015 | 0.028 | 0.013 | 0.043 | 0.017 |
| rs42302 | 5 | 74965554 | A | G | 0.009 | 0.002 | 5.96E-09 | -0.008 | 0.002 | 4.00E-04 | education | -0.005 | 0.010 | -0.013 | 0.014 | 0.006 | 0.014 | -0.002 | 0.012 | 0.011 | 0.016 |
| rs7716447 | 5 | 88800331 | A | G | 0.006 | 0.002 | 2.78E-04 | -0.013 | 0.002 | 3.60E-09 | TV | -0.016 | 0.010 | -0.018 | 0.014 | -0.009 | 0.014 | -0.007 | 0.012 | -0.021 | 0.016 |
| rs10046069 | 5 | 89115576 | A | G | 0.013 | 0.002 | 4.40E-09 | -0.009 | 0.003 | 1.10E-02 | education | 0.010 | 0.014 | 0.001 | 0.020 | 0.018 | 0.020 | 0.013 | 0.017 | 0.009 | 0.022 |
| rs1031423 | 5 | 93276883 | T | C | 0.012 | 0.002 | 4.11E-09 | -0.019 | 0.003 | 1.80E-12 | TV | 0.020 | 0.012 | 0.023 | 0.017 | 0.017 | 0.017 | 0.022 | 0.014 | 0.017 | 0.019 |
| rs252991 | 5 | 106767346 | A | G | 0.010 | 0.002 | 6.30E-11 | -0.010 | 0.002 | 1.40E-05 | education | -0.009 | 0.010 | -0.016 | 0.014 | -0.003 | 0.014 | -0.020 | 0.012 | 0.001 | 0.016 |
| rs57585211 | 5 | 107197725 | T | G | 0.010 | 0.002 | 7.87E-06 | -0.017 | 0.003 | 4.30E-09 | TV | -0.003 | 0.013 | -0.020 | 0.018 | 0.012 | 0.018 | -0.008 | 0.016 | -0.009 | 0.021 |
| rs17489649 | 5 | 109156184 | A | G | 0.013 | 0.002 | 2.77E-17 | 0.000 | 0.002 | 9.90E-01 | education | -0.016 | 0.011 | -0.029 | 0.015 | -0.005 | 0.015 | -0.002 | 0.012 | -0.038 | 0.016 |
| rs9326862 | 5 | 112054547 | A | T | -0.003 | 0.002 | 1.36E-01 | 0.012 | 0.002 | 2.60E-08 | TV | 0.001 | 0.010 | -0.011 | 0.014 | 0.014 | 0.014 | 0.006 | 0.012 | -0.010 | 0.015 |
| rs10041724 | 5 | 124273520 | T | C | -0.013 | 0.002 | 1.58E-09 | 0.018 | 0.003 | 3.90E-11 | TV | 0.015 | 0.012 | 0.021 | 0.017 | 0.010 | 0.017 | 0.016 | 0.014 | 0.012 | 0.019 |
| rs12519073 | 5 | 136776762 | T | C | -0.011 | 0.002 | 1.16E-10 | 0.006 | 0.003 | 3.20E-02 | education | 0.006 | 0.011 | 0.014 | 0.016 | -0.004 | 0.016 | -0.011 | 0.014 | 0.031 | 0.018 |
| rs62379379 | 5 | 141082015 | G | T | 0.015 | 0.003 | 1.86E-06 | -0.026 | 0.004 | 7.80E-10 | TV | -0.032 | 0.019 | -0.025 | 0.027 | -0.038 | 0.026 | -0.029 | 0.023 | -0.022 | 0.031 |
| rs10054327 | 5 | 147871137 | G | A | -0.004 | 0.002 | 3.91E-02 | 0.017 | 0.002 | 3.40E-15 | TV | -0.002 | 0.010 | 0.003 | 0.014 | -0.005 | 0.014 | -0.013 | 0.012 | -0.002 | 0.016 |
| rs2964255 | 5 | 152052332 | A | G | 0.009 | 0.002 | 6.34E-09 | -0.012 | 0.002 | 1.40E-07 | education | -0.010 | 0.010 | -0.012 | 0.015 | -0.006 | 0.015 | 0.002 | 0.013 | -0.047 | 0.016 |
| rs182902112 | 5 | 153304758 | A | C | -0.030 | 0.005 | 1.17E-08 | 0.025 | 0.008 | 1.90E-03 | education | 0.056 | 0.045 | 0.077 | 0.065 | 0.024 | 0.065 | 0.048 | 0.054 | 0.009 | 0.071 |
| rs42210 | 5 | 166408788 | C | G | -0.010 | 0.002 | 2.74E-10 | 0.014 | 0.002 | 7.30E-09 | both | 0.007 | 0.011 | 0.016 | 0.015 | -0.004 | 0.015 | -0.004 | 0.013 | 0.024 | 0.017 |
| rs72828890 | 5 | 167806836 | C | T | -0.006 | 0.003 | 1.73E-02 | 0.019 | 0.003 | 4.90E-09 | TV | 0.012 | 0.016 | 0.026 | 0.023 | -0.002 | 0.023 | 0.017 | 0.019 | 0.003 | 0.025 |
| rs12524795 | 6 | 3464074 | T | C | 0.010 | 0.001 | 5.28E-13 | -0.006 | 0.002 | 4.50E-03 | education | -0.001 | 0.010 | -0.008 | 0.014 | 0.007 | 0.014 | 0.007 | 0.012 | -0.018 | 0.015 |
| rs112603734 | 6 | 6069588 | A | C | -0.013 | 0.002 | 2.72E-09 | 0.000 | 0.003 | 8.60E-01 | education | -0.025 | 0.012 | -0.041 | 0.016 | -0.013 | 0.016 | -0.015 | 0.014 | -0.038 | 0.018 |
| rs72829857 | 6 | 16966052 | A | G | -0.015 | 0.002 | 1.52E-19 | 0.011 | 0.003 | 1.40E-05 | education | -0.013 | 0.011 | -0.017 | 0.016 | -0.006 | 0.016 | -0.010 | 0.013 | -0.012 | 0.017 |
| rs72834698 | 6 | 26176517 | G | A | -0.014 | 0.002 | 2.15E-08 | 0.023 | 0.003 | 2.70E-13 | TV | 0.044 | 0.014 | 0.060 | 0.020 | 0.031 | 0.020 | 0.059 | 0.017 | 0.069 | 0.022 |
| rs57349798 | 6 | 37486052 | A | G | 0.009 | 0.001 | 8.63E-11 | -0.006 | 0.002 | 4.20E-03 | education | -0.002 | 0.010 | 0.013 | 0.014 | -0.016 | 0.014 | -0.011 | 0.012 | -0.007 | 0.016 |
| rs9471333 | 6 | 40362023 | C | T | -0.009 | 0.002 | 3.40E-07 | 0.013 | 0.002 | 1.50E-09 | TV | 0.010 | 0.010 | 0.020 | 0.014 | 0.001 | 0.014 | 0.007 | 0.012 | 0.018 | 0.015 |
| rs78648104 | 6 | 50683009 | T | C | -0.014 | 0.003 | 1.70E-08 | 0.005 | 0.004 | 2.30E-01 | education | 0.000 | 0.017 | 0.004 | 0.023 | 0.000 | 0.024 | 0.011 | 0.020 | -0.008 | 0.027 |
| rs7449561 | 6 | 66215549 | A | G | 0.010 | 0.002 | 9.73E-10 | -0.006 | 0.003 | 1.40E-02 | education | 0.007 | 0.011 | 0.013 | 0.016 | 0.002 | 0.016 | 0.000 | 0.014 | 0.022 | 0.018 |
| rs4523073 | 6 | 67550288 | A | G | 0.002 | 0.002 | 1.82E-01 | -0.014 | 0.002 | 5.00E-10 | TV | 0.006 | 0.010 | 0.002 | 0.014 | 0.009 | 0.014 | 0.002 | 0.012 | 0.022 | 0.016 |
| rs13212041 | 6 | 78171124 | T | C | 0.010 | 0.002 | 7.42E-09 | -0.002 | 0.003 | 5.10E-01 | education | 0.022 | 0.012 | 0.012 | 0.017 | 0.032 | 0.017 | 0.025 | 0.015 | 0.013 | 0.019 |
| rs4352658 | 6 | 88279872 | T | C | -0.019 | 0.003 | 1.71E-13 | 0.015 | 0.004 | 2.20E-04 | education | 0.024 | 0.018 | -0.013 | 0.025 | 0.056 | 0.025 | 0.015 | 0.022 | 0.034 | 0.028 |
| rs6905544 | 6 | 98411631 | A | G | 0.023 | 0.002 | 7.33E-41 | -0.019 | 0.002 | 8.50E-18 | TV | -0.029 | 0.010 | -0.037 | 0.014 | -0.023 | 0.014 | -0.024 | 0.012 | -0.035 | 0.015 |
| rs17789218 | 6 | 100600097 | T | C | -0.001 | 0.002 | 6.08E-01 | 0.019 | 0.003 | 1.40E-13 | TV | 0.004 | 0.012 | 0.016 | 0.016 | -0.006 | 0.016 | -0.006 | 0.014 | 0.011 | 0.018 |
| rs10080647 | 6 | 114742335 | A | C | 0.012 | 0.002 | 7.05E-10 | -0.006 | 0.003 | 4.10E-02 | education | -0.011 | 0.014 | 0.008 | 0.019 | -0.028 | 0.019 | -0.016 | 0.016 | -0.031 | 0.022 |
| rs11542663 | 6 | 119215402 | A | C | 0.011 | 0.002 | 2.44E-13 | -0.005 | 0.002 | 2.80E-02 | education | -0.006 | 0.010 | 0.007 | 0.015 | -0.017 | 0.015 | -0.005 | 0.013 | 0.006 | 0.016 |
| rs13197257 | 6 | 128333682 | T | G | 0.011 | 0.002 | 2.83E-12 | 0.002 | 0.002 | 4.70E-01 | education | 0.000 | 0.011 | -0.014 | 0.016 | 0.013 | 0.016 | -0.005 | 0.013 | 0.016 | 0.017 |
| rs9492774 | 6 | 131302489 | C | G | 0.008 | 0.002 | 4.33E-08 | -0.004 | 0.002 | 8.10E-02 | education | 0.024 | 0.010 | 0.015 | 0.014 | 0.033 | 0.014 | 0.012 | 0.012 | 0.031 | 0.016 |
| rs2184364 | 6 | 143183596 | A | G | -0.009 | 0.002 | 2.05E-05 | 0.016 | 0.003 | 3.00E-09 | TV | 0.023 | 0.012 | 0.025 | 0.017 | 0.022 | 0.017 | 0.024 | 0.015 | 0.017 | 0.019 |
| rs4895650 | 6 | 145024122 | T | C | 0.009 | 0.001 | 5.31E-11 | -0.002 | 0.002 | 4.20E-01 | education | -0.013 | 0.010 | -0.011 | 0.014 | -0.013 | 0.014 | -0.020 | 0.012 | -0.015 | 0.016 |
| rs1933264 | 6 | 153391618 | T | C | 0.010 | 0.002 | 5.85E-10 | -0.010 | 0.003 | 5.60E-05 | education | -0.017 | 0.011 | -0.021 | 0.015 | -0.009 | 0.016 | -0.004 | 0.013 | -0.023 | 0.017 |
| rs9718104 | 6 | 166170539 | T | G | -0.003 | 0.004 | 4.30E-01 | -0.041 | 0.005 | 9.30E-19 | TV | -0.002 | 0.022 | 0.002 | 0.032 | -0.013 | 0.031 | 0.009 | 0.027 | 0.005 | 0.036 |
| rs6917154 | 6 | 167135156 | T | C | 0.013 | 0.002 | 2.55E-09 | -0.017 | 0.003 | 1.90E-07 | education | -0.022 | 0.015 | -0.041 | 0.022 | 0.002 | 0.022 | -0.002 | 0.018 | -0.037 | 0.024 |
| rs6924023 | 6 | 170076041 | A | G | 0.011 | 0.002 | 5.17E-11 | -0.009 | 0.003 | 6.60E-04 | education | 0.004 | 0.011 | 0.004 | 0.016 | 0.000 | 0.016 | 0.013 | 0.013 | -0.015 | 0.017 |
| rs17568389 | 7 | 8091876 | T | A | -0.012 | 0.002 | 1.53E-12 | 0.015 | 0.002 | 8.60E-13 | TV | 0.001 | 0.010 | 0.012 | 0.014 | -0.012 | 0.014 | 0.000 | 0.012 | 0.012 | 0.015 |
| rs118134876 | 7 | 11500372 | T | C | -0.022 | 0.003 | 1.50E-13 | 0.010 | 0.005 | 3.60E-02 | education | 0.003 | 0.021 | 0.023 | 0.029 | -0.015 | 0.029 | 0.008 | 0.024 | 0.001 | 0.032 |
| rs35929923 | 7 | 21400525 | A | G | -0.011 | 0.002 | 1.77E-12 | 0.008 | 0.002 | 1.00E-03 | education | 0.000 | 0.011 | -0.016 | 0.016 | 0.014 | 0.016 | -0.004 | 0.013 | 0.008 | 0.017 |
| rs79265434 | 7 | 24621381 | A | G | -0.020 | 0.002 | 1.98E-19 | 0.010 | 0.003 | 2.80E-03 | education | 0.024 | 0.016 | 0.029 | 0.023 | 0.014 | 0.023 | 0.029 | 0.019 | 0.059 | 0.026 |
| rs10951590 | 7 | 39091043 | T | C | -0.011 | 0.002 | 3.62E-13 | 0.004 | 0.002 | 6.40E-02 | education | 0.008 | 0.010 | -0.001 | 0.015 | 0.019 | 0.015 | 0.018 | 0.012 | 0.009 | 0.016 |
| rs10234444 | 7 | 41828527 | G | A | -0.008 | 0.002 | 8.80E-04 | 0.016 | 0.003 | 2.90E-08 | TV | -0.017 | 0.013 | -0.018 | 0.018 | -0.018 | 0.018 | -0.023 | 0.016 | -0.004 | 0.021 |
| rs1220779 | 7 | 54734673 | A | G | -0.008 | 0.001 | 1.58E-08 | 0.003 | 0.002 | 1.20E-01 | education | 0.024 | 0.010 | 0.029 | 0.014 | 0.018 | 0.014 | 0.021 | 0.012 | 0.027 | 0.015 |
| rs1167827 | 7 | 75163169 | A | G | 0.010 | 0.001 | 2.93E-13 | -0.008 | 0.002 | 2.10E-04 | education | -0.009 | 0.010 | -0.020 | 0.014 | 0.003 | 0.014 | -0.004 | 0.012 | -0.005 | 0.015 |
| rs6973656 | 7 | 77422583 | A | G | 0.004 | 0.002 | 3.09E-02 | -0.013 | 0.002 | 1.00E-09 | TV | 0.029 | 0.010 | 0.025 | 0.014 | 0.031 | 0.014 | 0.028 | 0.012 | 0.036 | 0.015 |
| rs973734 | 7 | 89387578 | C | A | -0.007 | 0.002 | 1.58E-03 | 0.017 | 0.003 | 1.50E-08 | TV | 0.002 | 0.014 | -0.022 | 0.020 | 0.031 | 0.020 | 0.013 | 0.016 | -0.009 | 0.021 |
| rs9655780 | 7 | 104667334 | A | G | -0.015 | 0.002 | 3.70E-16 | 0.010 | 0.003 | 7.80E-04 | education | 0.018 | 0.014 | 0.015 | 0.019 | 0.021 | 0.019 | 0.004 | 0.016 | 0.043 | 0.022 |
| rs62471080 | 7 | 109128060 | G | C | 0.005 | 0.002 | 1.48E-03 | -0.013 | 0.002 | 3.70E-09 | TV | -0.005 | 0.010 | -0.007 | 0.014 | -0.004 | 0.014 | 0.010 | 0.012 | -0.012 | 0.015 |
| rs10246289 | 7 | 115544763 | A | G | -0.007 | 0.003 | 9.30E-03 | 0.019 | 0.003 | 1.70E-08 | TV | 0.041 | 0.015 | 0.039 | 0.022 | 0.042 | 0.022 | 0.033 | 0.019 | 0.033 | 0.024 |
| rs11763734 | 7 | 126400910 | A | C | 0.007 | 0.002 | 3.27E-05 | -0.012 | 0.002 | 8.20E-09 | TV | -0.001 | 0.010 | 0.001 | 0.014 | -0.005 | 0.014 | -0.002 | 0.012 | 0.005 | 0.015 |
| rs113520408 | 7 | 128402782 | A | G | 0.016 | 0.002 | 1.03E-22 | -0.006 | 0.002 | 1.90E-02 | education | -0.012 | 0.011 | -0.014 | 0.015 | -0.012 | 0.015 | -0.024 | 0.013 | 0.005 | 0.017 |
| rs57352738 | 7 | 133304694 | A | T | -0.015 | 0.002 | 2.51E-18 | 0.002 | 0.003 | 5.60E-01 | education | 0.017 | 0.012 | 0.026 | 0.017 | 0.007 | 0.017 | 0.024 | 0.014 | 0.019 | 0.019 |
| rs4726070 | 7 | 151328218 | A | G | 0.012 | 0.001 | 1.36E-17 | -0.004 | 0.002 | 6.60E-02 | education | 0.000 | 0.010 | -0.014 | 0.014 | 0.013 | 0.014 | 0.007 | 0.012 | -0.004 | 0.015 |
| rs6994287 | 8 | 9340932 | A | G | 0.010 | 0.001 | 1.11E-12 | -0.001 | 0.002 | 6.30E-01 | education | -0.007 | 0.010 | -0.009 | 0.014 | -0.006 | 0.014 | -0.021 | 0.012 | -0.012 | 0.015 |
| rs7834121 | 8 | 10770255 | G | T | -0.002 | 0.002 | 2.03E-01 | -0.014 | 0.002 | 1.10E-10 | TV | 0.007 | 0.010 | 0.015 | 0.015 | 0.000 | 0.015 | -0.007 | 0.012 | 0.028 | 0.016 |
| rs34807077 | 8 | 28680769 | A | C | 0.013 | 0.002 | 3.29E-11 | -0.006 | 0.003 | 3.10E-02 | education | -0.023 | 0.013 | -0.019 | 0.019 | -0.020 | 0.019 | -0.019 | 0.016 | 0.007 | 0.021 |
| rs1421334 | 8 | 30865733 | A | C | -0.011 | 0.002 | 5.46E-11 | 0.017 | 0.002 | 2.20E-15 | TV | 0.017 | 0.010 | 0.017 | 0.014 | 0.018 | 0.014 | 0.022 | 0.012 | -0.005 | 0.015 |
| rs2923424 | 8 | 42382222 | A | G | 0.012 | 0.001 | 6.31E-17 | -0.004 | 0.002 | 1.10E-01 | education | -0.009 | 0.010 | -0.007 | 0.014 | -0.014 | 0.014 | -0.006 | 0.012 | 0.007 | 0.016 |
| rs1866823 | 8 | 57436577 | A | G | 0.011 | 0.001 | 3.09E-14 | -0.003 | 0.002 | 1.70E-01 | education | -0.015 | 0.010 | -0.032 | 0.014 | 0.003 | 0.014 | -0.013 | 0.012 | -0.019 | 0.016 |
| rs6996198 | 8 | 65463442 | C | T | 0.007 | 0.002 | 1.73E-03 | -0.016 | 0.003 | 3.50E-08 | TV | -0.016 | 0.013 | -0.009 | 0.018 | -0.025 | 0.018 | -0.011 | 0.016 | -0.017 | 0.020 |
| rs6472942 | 8 | 76827190 | T | C | 0.000 | 0.002 | 9.62E-01 | -0.013 | 0.002 | 1.80E-09 | TV | 0.000 | 0.010 | -0.006 | 0.014 | 0.004 | 0.014 | -0.001 | 0.012 | 0.015 | 0.015 |
| rs11985394 | 8 | 92667521 | T | A | -0.002 | 0.002 | 2.66E-01 | 0.012 | 0.002 | 1.30E-08 | TV | 0.010 | 0.010 | 0.017 | 0.014 | -0.001 | 0.014 | 0.020 | 0.012 | 0.000 | 0.015 |
| rs7012546 | 8 | 105067737 | T | C | 0.009 | 0.001 | 1.25E-10 | -0.004 | 0.002 | 9.30E-02 | education | -0.023 | 0.010 | -0.031 | 0.014 | -0.014 | 0.014 | -0.029 | 0.012 | -0.023 | 0.015 |
| rs12541615 | 8 | 118870151 | T | C | 0.008 | 0.002 | 1.03E-04 | -0.018 | 0.003 | 3.90E-10 | TV | -0.003 | 0.012 | 0.001 | 0.017 | -0.008 | 0.018 | -0.013 | 0.015 | 0.006 | 0.019 |
| rs13261773 | 8 | 120187090 | C | G | 0.012 | 0.002 | 3.12E-10 | -0.012 | 0.003 | 1.10E-04 | education | -0.019 | 0.013 | -0.025 | 0.018 | -0.018 | 0.018 | -0.018 | 0.015 | -0.023 | 0.020 |
| rs401526 | 8 | 130929166 | T | C | -0.010 | 0.001 | 1.15E-13 | 0.008 | 0.002 | 4.70E-04 | education | 0.034 | 0.010 | 0.041 | 0.014 | 0.029 | 0.014 | 0.042 | 0.012 | 0.045 | 0.015 |
| rs7460106 | 8 | 143534777 | T | C | -0.012 | 0.002 | 2.06E-13 | 0.004 | 0.003 | 1.00E-01 | education | 0.021 | 0.012 | 0.037 | 0.017 | 0.004 | 0.017 | 0.031 | 0.014 | 0.034 | 0.019 |
| rs2717559 | 8 | 143882420 | A | G | -0.009 | 0.002 | 5.93E-07 | 0.012 | 0.002 | 1.50E-08 | TV | 0.000 | 0.010 | -0.018 | 0.014 | 0.018 | 0.014 | -0.006 | 0.012 | 0.003 | 0.015 |
| rs2616830 | 9 | 1721385 | G | A | -0.015 | 0.002 | 1.23E-17 | 0.016 | 0.002 | 2.90E-14 | TV | 0.013 | 0.010 | 0.014 | 0.014 | 0.008 | 0.014 | 0.016 | 0.012 | 0.031 | 0.015 |
| rs7875078 | 9 | 14494845 | A | C | -0.008 | 0.001 | 2.72E-08 | 0.010 | 0.002 | 2.70E-06 | education | -0.001 | 0.010 | -0.008 | 0.014 | 0.006 | 0.014 | 0.007 | 0.012 | -0.004 | 0.015 |
| rs34864022 | 9 | 22609110 | A | G | 0.009 | 0.003 | 8.92E-03 | -0.026 | 0.004 | 1.40E-09 | TV | 0.013 | 0.021 | 0.033 | 0.030 | -0.010 | 0.030 | 0.015 | 0.025 | -0.026 | 0.033 |
| rs12554512 | 9 | 23352293 | T | C | -0.024 | 0.002 | 1.69E-43 | 0.021 | 0.002 | 3.80E-21 | TV | -0.002 | 0.010 | -0.001 | 0.014 | -0.002 | 0.014 | 0.011 | 0.012 | -0.005 | 0.016 |
| rs4977885 | 9 | 23687983 | A | G | -0.008 | 0.001 | 6.78E-09 | 0.005 | 0.002 | 3.50E-02 | education | -0.004 | 0.010 | 0.003 | 0.014 | -0.008 | 0.014 | -0.003 | 0.012 | -0.019 | 0.016 |
| rs7041702 | 9 | 33053430 | A | G | -0.011 | 0.002 | 4.53E-12 | -0.004 | 0.002 | 1.10E-01 | education | 0.021 | 0.011 | 0.034 | 0.016 | 0.004 | 0.016 | 0.030 | 0.013 | 0.016 | 0.017 |
| rs11138947 | 9 | 72110562 | T | C | 0.011 | 0.002 | 2.36E-11 | -0.002 | 0.002 | 5.10E-01 | education | -0.003 | 0.011 | 0.003 | 0.015 | -0.007 | 0.015 | -0.006 | 0.013 | 0.007 | 0.017 |
| rs2791574 | 9 | 82212094 | T | G | 0.008 | 0.002 | 2.22E-06 | -0.013 | 0.002 | 3.00E-08 | TV | -0.020 | 0.010 | -0.017 | 0.014 | -0.019 | 0.014 | -0.021 | 0.012 | -0.014 | 0.016 |
| rs870151 | 9 | 92230747 | T | A | 0.009 | 0.002 | 1.28E-07 | -0.016 | 0.002 | 8.80E-13 | TV | 0.010 | 0.010 | 0.005 | 0.014 | 0.018 | 0.014 | 0.011 | 0.012 | 0.005 | 0.015 |
| rs10761251 | 9 | 96421546 | A | T | 0.011 | 0.002 | 2.52E-14 | -0.006 | 0.002 | 1.60E-02 | education | -0.004 | 0.010 | -0.010 | 0.014 | 0.004 | 0.014 | -0.003 | 0.012 | -0.001 | 0.016 |
| rs2787374 | 9 | 103054951 | T | C | -0.004 | 0.002 | 1.24E-02 | 0.012 | 0.002 | 1.20E-08 | TV | 0.012 | 0.010 | -0.002 | 0.014 | 0.025 | 0.014 | 0.005 | 0.012 | 0.028 | 0.015 |
| rs76235882 | 9 | 121096681 | A | G | 0.024 | 0.004 | 4.52E-10 | -0.012 | 0.006 | 5.20E-02 | education | -0.002 | 0.029 | 0.053 | 0.042 | -0.053 | 0.041 | -0.003 | 0.035 | 0.010 | 0.046 |
| rs7043521 | 9 | 124602779 | A | T | -0.014 | 0.002 | 5.88E-16 | 0.014 | 0.002 | 6.50E-11 | TV | -0.021 | 0.010 | -0.014 | 0.014 | -0.027 | 0.014 | -0.021 | 0.012 | -0.043 | 0.015 |
| rs2039204 | 9 | 130371107 | A | T | 0.008 | 0.001 | 5.23E-09 | 0.002 | 0.002 | 4.10E-01 | education | -0.008 | 0.010 | -0.001 | 0.014 | -0.015 | 0.014 | -0.015 | 0.012 | 0.004 | 0.015 |
| rs4382592 | 9 | 134870755 | T | G | -0.016 | 0.002 | 1.01E-18 | 0.014 | 0.002 | 6.70E-09 | TV | 0.009 | 0.010 | 0.013 | 0.015 | 0.005 | 0.015 | 0.006 | 0.013 | 0.000 | 0.016 |
| rs2073869 | 9 | 135763816 | C | T | -0.010 | 0.002 | 1.04E-05 | 0.019 | 0.003 | 1.50E-10 | TV | -0.011 | 0.013 | -0.002 | 0.018 | -0.018 | 0.018 | -0.018 | 0.015 | -0.019 | 0.020 |
| rs7894722 | 10 | 9972046 | T | C | 0.009 | 0.001 | 5.41E-09 | 0.004 | 0.002 | 5.90E-02 | education | 0.014 | 0.010 | -0.020 | 0.014 | 0.045 | 0.014 | 0.001 | 0.012 | 0.001 | 0.016 |
| rs17148998 | 10 | 10909401 | A | G | 0.010 | 0.002 | 6.98E-09 | -0.012 | 0.003 | 1.20E-05 | education | -0.003 | 0.012 | -0.020 | 0.017 | 0.018 | 0.017 | -0.016 | 0.015 | 0.027 | 0.019 |
| rs1623025 | 10 | 11164813 | G | C | 0.010 | 0.002 | 4.50E-09 | -0.012 | 0.002 | 1.40E-08 | TV | 0.018 | 0.010 | 0.019 | 0.014 | 0.017 | 0.014 | 0.019 | 0.012 | 0.006 | 0.015 |
| rs1243182 | 10 | 21916728 | C | T | 0.008 | 0.002 | 4.66E-05 | -0.019 | 0.002 | 2.00E-15 | TV | -0.029 | 0.011 | -0.039 | 0.015 | -0.020 | 0.015 | -0.025 | 0.013 | -0.054 | 0.016 |
| rs11598765 | 10 | 23953725 | A | G | -0.011 | 0.002 | 1.73E-09 | 0.011 | 0.003 | 1.30E-04 | education | -0.014 | 0.012 | -0.010 | 0.017 | -0.018 | 0.017 | -0.013 | 0.014 | -0.018 | 0.019 |
| rs806816 | 10 | 32285942 | A | T | 0.010 | 0.002 | 4.80E-10 | -0.004 | 0.003 | 1.50E-01 | education | 0.000 | 0.011 | -0.007 | 0.016 | 0.006 | 0.016 | 0.008 | 0.013 | -0.014 | 0.017 |
| rs2007655 | 10 | 34586689 | T | G | 0.009 | 0.001 | 8.64E-10 | -0.005 | 0.002 | 2.10E-02 | education | -0.015 | 0.010 | -0.008 | 0.014 | -0.019 | 0.013 | -0.007 | 0.011 | -0.038 | 0.015 |
| rs2045147 | 10 | 56660437 | A | G | -0.002 | 0.002 | 1.58E-01 | 0.013 | 0.002 | 5.90E-09 | TV | 0.009 | 0.010 | 0.018 | 0.014 | -0.001 | 0.014 | 0.006 | 0.012 | 0.019 | 0.015 |
| rs10994943 | 10 | 63591413 | T | G | -0.008 | 0.002 | 5.94E-06 | 0.013 | 0.002 | 3.10E-09 | TV | 0.007 | 0.010 | 0.017 | 0.014 | -0.006 | 0.014 | 0.000 | 0.012 | 0.014 | 0.015 |
| rs10996167 | 10 | 66813928 | C | G | 0.011 | 0.002 | 7.47E-09 | -0.003 | 0.002 | 1.30E-01 | education | -0.020 | 0.010 | -0.020 | 0.014 | -0.017 | 0.014 | -0.018 | 0.012 | -0.006 | 0.016 |
| rs11201422 | 10 | 86924483 | T | C | -0.006 | 0.002 | 9.87E-04 | 0.013 | 0.002 | 3.40E-08 | TV | 0.019 | 0.011 | 0.005 | 0.015 | 0.036 | 0.015 | 0.022 | 0.012 | 0.027 | 0.016 |
| rs1426619 | 10 | 90091540 | T | C | 0.010 | 0.001 | 1.40E-11 | -0.006 | 0.002 | 3.80E-03 | education | 0.000 | 0.010 | -0.009 | 0.014 | 0.011 | 0.014 | 0.008 | 0.012 | -0.013 | 0.015 |
| rs17882802 | 10 | 101983413 | A | G | 0.008 | 0.001 | 3.00E-09 | 0.000 | 0.002 | 9.90E-01 | education | 0.005 | 0.010 | 0.010 | 0.014 | 0.002 | 0.014 | 0.020 | 0.012 | -0.002 | 0.015 |
| rs10786658 | 10 | 103737193 | A | T | 0.016 | 0.002 | 1.07E-20 | -0.014 | 0.002 | 4.60E-11 | TV | 0.003 | 0.010 | 0.011 | 0.014 | -0.007 | 0.014 | 0.002 | 0.012 | -0.001 | 0.015 |
| rs790647 | 10 | 106776484 | A | C | -0.015 | 0.002 | 3.81E-18 | 0.004 | 0.003 | 1.50E-01 | education | -0.007 | 0.012 | -0.002 | 0.016 | -0.011 | 0.016 | -0.020 | 0.014 | -0.021 | 0.018 |
| rs12571549 | 10 | 111770013 | A | G | 0.015 | 0.002 | 1.45E-13 | -0.008 | 0.003 | 8.70E-03 | education | -0.014 | 0.013 | -0.018 | 0.019 | -0.009 | 0.019 | -0.006 | 0.017 | -0.006 | 0.022 |
| rs7089973 | 10 | 116569565 | C | A | 0.003 | 0.002 | 1.01E-01 | -0.013 | 0.002 | 2.40E-09 | TV | -0.021 | 0.010 | -0.022 | 0.015 | -0.020 | 0.014 | -0.028 | 0.012 | -0.036 | 0.016 |
| rs11245482 | 10 | 126733546 | T | C | 0.006 | 0.002 | 5.32E-04 | -0.013 | 0.002 | 2.60E-09 | TV | -0.004 | 0.010 | 0.006 | 0.014 | -0.016 | 0.014 | -0.002 | 0.012 | -0.008 | 0.015 |
| rs17727474 | 10 | 127182959 | C | T | -0.003 | 0.002 | 1.30E-01 | 0.018 | 0.003 | 3.10E-09 | TV | 0.001 | 0.013 | -0.001 | 0.019 | 0.005 | 0.019 | -0.009 | 0.016 | 0.010 | 0.021 |
| rs12289262 | 11 | 12894758 | C | T | 0.014 | 0.002 | 9.54E-13 | -0.014 | 0.002 | 2.00E-08 | TV | -0.028 | 0.011 | -0.029 | 0.016 | -0.029 | 0.016 | -0.030 | 0.014 | -0.010 | 0.018 |
| rs11023764 | 11 | 15910939 | A | G | 0.009 | 0.002 | 5.32E-10 | -0.004 | 0.002 | 1.00E-01 | education | -0.012 | 0.010 | -0.022 | 0.015 | 0.002 | 0.014 | -0.024 | 0.012 | 0.008 | 0.016 |
| rs12789313 | 11 | 25058937 | T | C | 0.009 | 0.001 | 6.79E-11 | -0.002 | 0.002 | 3.00E-01 | education | -0.007 | 0.010 | -0.012 | 0.014 | -0.004 | 0.014 | -0.007 | 0.012 | -0.016 | 0.015 |
| rs11030102 | 11 | 27681596 | C | G | 0.010 | 0.002 | 4.60E-10 | -0.004 | 0.002 | 1.20E-01 | education | 0.023 | 0.012 | 0.022 | 0.016 | 0.020 | 0.016 | 0.021 | 0.014 | 0.032 | 0.018 |
| rs2199409 | 11 | 39915234 | T | C | 0.010 | 0.002 | 3.14E-09 | -0.009 | 0.003 | 5.10E-04 | education | 0.016 | 0.012 | -0.005 | 0.017 | 0.035 | 0.017 | 0.017 | 0.014 | 0.007 | 0.018 |
| rs79728014 | 11 | 46062245 | A | G | -0.013 | 0.002 | 2.06E-11 | 0.005 | 0.003 | 9.30E-02 | education | -0.015 | 0.014 | -0.029 | 0.021 | -0.003 | 0.020 | -0.013 | 0.017 | -0.001 | 0.023 |
| rs75033012 | 11 | 61347033 | C | G | -0.026 | 0.004 | 1.38E-11 | 0.004 | 0.006 | 4.70E-01 | education | 0.007 | 0.030 | 0.008 | 0.043 | 0.009 | 0.041 | -0.024 | 0.036 | 0.076 | 0.046 |
| rs801733 | 11 | 65934549 | A | C | -0.010 | 0.002 | 1.65E-07 | 0.017 | 0.002 | 7.30E-14 | TV | 0.027 | 0.010 | 0.028 | 0.014 | 0.026 | 0.014 | 0.023 | 0.012 | 0.048 | 0.016 |
| rs116175338 | 11 | 72467875 | G | T | 0.016 | 0.002 | 1.36E-16 | -0.014 | 0.003 | 1.40E-08 | TV | -0.001 | 0.011 | 0.011 | 0.016 | -0.011 | 0.016 | -0.002 | 0.014 | 0.020 | 0.018 |
| rs12272012 | 11 | 84861286 | G | C | 0.009 | 0.004 | 3.62E-02 | -0.030 | 0.005 | 1.90E-08 | TV | 0.031 | 0.024 | 0.000 | 0.034 | 0.062 | 0.033 | 0.036 | 0.028 | 0.064 | 0.037 |
| rs11020045 | 11 | 92487907 | A | C | 0.007 | 0.002 | 1.88E-04 | -0.013 | 0.002 | 1.60E-08 | TV | 0.007 | 0.010 | 0.021 | 0.015 | -0.006 | 0.015 | 0.018 | 0.012 | -0.003 | 0.016 |
| rs17207890 | 11 | 95490754 | G | A | -0.014 | 0.002 | 3.58E-14 | 0.016 | 0.002 | 6.70E-12 | TV | 0.000 | 0.010 | 0.012 | 0.015 | -0.010 | 0.015 | 0.016 | 0.012 | -0.021 | 0.016 |
| rs1301838 | 11 | 99670514 | T | C | -0.010 | 0.002 | 1.29E-10 | 0.005 | 0.002 | 3.70E-02 | education | 0.005 | 0.010 | -0.002 | 0.015 | 0.011 | 0.015 | 0.001 | 0.012 | 0.013 | 0.016 |
| rs72486027 | 11 | 107167486 | T | C | -0.010 | 0.002 | 1.61E-10 | 0.002 | 0.003 | 5.00E-01 | education | 0.000 | 0.011 | -0.027 | 0.016 | 0.027 | 0.016 | -0.009 | 0.013 | 0.010 | 0.017 |
| rs2212430 | 11 | 109289538 | T | C | -0.010 | 0.002 | 1.32E-10 | 0.007 | 0.002 | 1.60E-03 | education | -0.015 | 0.010 | -0.027 | 0.015 | -0.006 | 0.015 | -0.002 | 0.013 | -0.030 | 0.017 |
| rs11213482 | 11 | 110435636 | A | G | -0.011 | 0.002 | 2.68E-08 | 0.010 | 0.003 | 4.20E-04 | education | 0.008 | 0.015 | 0.025 | 0.021 | -0.008 | 0.021 | 0.020 | 0.017 | 0.013 | 0.023 |
| rs17565975 | 11 | 111586950 | A | G | -0.011 | 0.001 | 5.54E-14 | 0.005 | 0.002 | 2.20E-02 | education | -0.019 | 0.010 | -0.015 | 0.014 | -0.025 | 0.014 | -0.021 | 0.012 | -0.016 | 0.015 |
| rs11218575 | 11 | 122154576 | C | T | -0.006 | 0.002 | 5.66E-04 | 0.015 | 0.002 | 2.10E-12 | TV | 0.001 | 0.010 | 0.013 | 0.014 | -0.010 | 0.014 | 0.001 | 0.012 | -0.001 | 0.015 |
| rs12574281 | 11 | 131205421 | A | C | -0.008 | 0.002 | 3.04E-08 | 0.006 | 0.002 | 4.40E-03 | education | 0.006 | 0.010 | 0.018 | 0.014 | -0.005 | 0.014 | 0.018 | 0.012 | 0.001 | 0.016 |
| rs4937842 | 11 | 133734542 | G | C | 0.002 | 0.002 | 1.87E-01 | -0.013 | 0.002 | 1.40E-08 | TV | -0.012 | 0.010 | -0.018 | 0.014 | -0.008 | 0.014 | -0.018 | 0.012 | -0.023 | 0.016 |
| rs4766424 | 12 | 1954096 | C | G | 0.014 | 0.002 | 2.47E-11 | -0.001 | 0.003 | 6.50E-01 | education | 0.006 | 0.015 | 0.009 | 0.021 | 0.010 | 0.021 | 0.001 | 0.017 | 0.002 | 0.023 |
| rs10772643 | 12 | 13415288 | C | T | -0.016 | 0.003 | 3.54E-09 | 0.025 | 0.003 | 1.30E-12 | TV | 0.027 | 0.016 | 0.041 | 0.022 | 0.014 | 0.022 | 0.024 | 0.019 | 0.056 | 0.024 |
| rs1861786 | 12 | 14000467 | A | G | -0.008 | 0.001 | 2.39E-08 | 0.005 | 0.002 | 1.90E-02 | education | 0.005 | 0.010 | 0.013 | 0.014 | -0.003 | 0.014 | 0.004 | 0.012 | 0.019 | 0.016 |
| rs7974852 | 12 | 14511679 | A | C | 0.012 | 0.001 | 1.14E-16 | -0.003 | 0.002 | 1.90E-01 | education | -0.006 | 0.010 | -0.010 | 0.014 | -0.003 | 0.014 | -0.011 | 0.012 | -0.018 | 0.015 |
| rs117398064 | 12 | 26523628 | C | G | -0.014 | 0.003 | 1.16E-08 | 0.007 | 0.004 | 6.20E-02 | education | -0.011 | 0.018 | -0.029 | 0.025 | 0.005 | 0.025 | -0.017 | 0.021 | 0.001 | 0.027 |
| rs7972246 | 12 | 27209328 | T | C | 0.011 | 0.002 | 1.01E-13 | -0.006 | 0.002 | 5.20E-03 | education | -0.012 | 0.010 | 0.006 | 0.014 | -0.031 | 0.014 | -0.018 | 0.012 | -0.002 | 0.016 |
| rs10771746 | 12 | 30791864 | C | T | 0.005 | 0.002 | 4.35E-03 | -0.014 | 0.002 | 2.50E-09 | TV | 0.006 | 0.010 | -0.008 | 0.015 | 0.017 | 0.015 | 0.001 | 0.013 | 0.031 | 0.017 |
| rs17110109 | 12 | 54668908 | T | C | -0.009 | 0.001 | 1.47E-10 | 0.002 | 0.002 | 2.70E-01 | education | -0.008 | 0.010 | -0.004 | 0.014 | -0.011 | 0.015 | -0.009 | 0.012 | -0.012 | 0.016 |
| rs10876864 | 12 | 56401085 | G | A | 0.016 | 0.002 | 1.22E-19 | -0.013 | 0.002 | 1.00E-09 | TV | -0.010 | 0.010 | -0.005 | 0.014 | -0.018 | 0.014 | -0.029 | 0.012 | 0.021 | 0.015 |
| rs8756 | 12 | 66359752 | C | A | 0.008 | 0.002 | 1.41E-06 | -0.013 | 0.002 | 5.30E-10 | TV | -0.009 | 0.010 | -0.008 | 0.014 | -0.009 | 0.014 | -0.005 | 0.012 | -0.016 | 0.015 |
| rs57661533 | 12 | 78629917 | T | C | -0.011 | 0.002 | 3.24E-08 | 0.003 | 0.003 | 2.90E-01 | education | -0.004 | 0.014 | -0.008 | 0.019 | 0.000 | 0.019 | -0.011 | 0.017 | -0.020 | 0.022 |
| rs1245829 | 12 | 79648170 | A | T | -0.010 | 0.001 | 3.29E-12 | 0.011 | 0.002 | 7.50E-07 | education | 0.003 | 0.010 | 0.003 | 0.014 | 0.001 | 0.014 | 0.008 | 0.012 | 0.002 | 0.016 |
| rs717996 | 12 | 84133820 | T | C | -0.012 | 0.001 | 1.89E-17 | 0.007 | 0.002 | 8.60E-04 | education | -0.005 | 0.010 | -0.012 | 0.014 | 0.003 | 0.014 | -0.017 | 0.012 | 0.019 | 0.016 |
| rs2216144 | 12 | 97666478 | T | C | 0.010 | 0.001 | 2.58E-12 | -0.004 | 0.002 | 7.70E-02 | education | -0.023 | 0.010 | -0.035 | 0.014 | -0.011 | 0.014 | -0.016 | 0.012 | -0.046 | 0.015 |
| rs2131167 | 12 | 99633189 | A | G | 0.008 | 0.001 | 1.52E-08 | -0.003 | 0.002 | 1.20E-01 | education | -0.011 | 0.010 | -0.021 | 0.014 | 0.000 | 0.014 | -0.014 | 0.012 | -0.007 | 0.015 |
| rs2164744 | 12 | 109872039 | T | C | 0.004 | 0.002 | 1.28E-02 | -0.013 | 0.002 | 1.10E-08 | TV | 0.014 | 0.010 | 0.013 | 0.014 | 0.015 | 0.014 | 0.007 | 0.012 | 0.027 | 0.015 |
| rs2173650 | 12 | 117525235 | G | T | -0.012 | 0.002 | 3.06E-07 | 0.018 | 0.003 | 4.60E-09 | TV | -0.006 | 0.013 | 0.016 | 0.019 | -0.028 | 0.019 | -0.002 | 0.016 | 0.010 | 0.021 |
| rs6490618 | 13 | 21338343 | T | C | 0.009 | 0.002 | 3.48E-09 | -0.005 | 0.002 | 3.40E-02 | education | 0.017 | 0.010 | 0.003 | 0.015 | 0.036 | 0.015 | 0.002 | 0.012 | 0.038 | 0.016 |
| rs9529146 | 13 | 31785404 | T | C | 0.013 | 0.002 | 3.48E-14 | -0.006 | 0.003 | 2.00E-02 | education | -0.004 | 0.011 | 0.014 | 0.016 | -0.021 | 0.016 | 0.002 | 0.014 | -0.009 | 0.018 |
| rs936496 | 13 | 36100541 | A | G | -0.008 | 0.001 | 4.38E-08 | 0.006 | 0.002 | 3.90E-03 | education | 0.002 | 0.010 | -0.002 | 0.014 | 0.007 | 0.014 | -0.001 | 0.012 | -0.001 | 0.016 |
| rs9563168 | 13 | 54247827 | G | A | -0.010 | 0.002 | 5.93E-07 | 0.018 | 0.003 | 4.30E-11 | TV | 0.006 | 0.012 | -0.004 | 0.017 | 0.016 | 0.017 | 0.010 | 0.014 | -0.013 | 0.018 |
| rs9569734 | 13 | 58319476 | A | G | -0.009 | 0.002 | 2.77E-04 | 0.019 | 0.003 | 3.40E-10 | TV | 0.010 | 0.013 | 0.024 | 0.019 | -0.011 | 0.019 | 0.012 | 0.016 | 0.037 | 0.021 |
| rs7321274 | 13 | 69146186 | A | G | 0.011 | 0.002 | 8.21E-11 | -0.005 | 0.003 | 6.20E-02 | education | 0.018 | 0.012 | 0.024 | 0.017 | 0.010 | 0.017 | 0.005 | 0.015 | 0.023 | 0.019 |
| rs2478208 | 13 | 81471113 | C | G | -0.010 | 0.001 | 2.89E-13 | 0.009 | 0.002 | 6.10E-05 | education | -0.002 | 0.010 | -0.005 | 0.014 | 0.000 | 0.014 | -0.004 | 0.012 | -0.020 | 0.015 |
| rs56858768 | 13 | 86511730 | G | A | 0.006 | 0.002 | 1.18E-03 | -0.015 | 0.002 | 3.40E-10 | TV | -0.010 | 0.011 | -0.006 | 0.015 | -0.015 | 0.015 | -0.012 | 0.013 | -0.019 | 0.017 |
| rs7326331 | 13 | 91615838 | A | G | -0.013 | 0.002 | 3.42E-16 | 0.009 | 0.002 | 1.00E-04 | education | 0.014 | 0.011 | 0.011 | 0.015 | 0.020 | 0.015 | 0.004 | 0.013 | 0.021 | 0.017 |
| rs9556958 | 13 | 99100046 | T | C | -0.011 | 0.001 | 5.96E-15 | 0.004 | 0.002 | 6.50E-02 | education | -0.004 | 0.010 | -0.008 | 0.014 | 0.002 | 0.014 | -0.012 | 0.012 | 0.002 | 0.015 |
| rs7991062 | 13 | 100713194 | C | G | 0.008 | 0.002 | 3.09E-06 | -0.018 | 0.002 | 8.80E-15 | TV | -0.013 | 0.010 | -0.011 | 0.014 | -0.015 | 0.014 | -0.012 | 0.012 | -0.017 | 0.016 |
| rs11157931 | 14 | 23403193 | A | C | -0.014 | 0.001 | 3.86E-22 | 0.010 | 0.002 | 1.10E-05 | education | 0.018 | 0.012 | 0.029 | 0.014 | 0.028 | 0.014 | 0.031 | 0.012 | 0.020 | 0.015 |
| rs405797 | 14 | 26972834 | T | A | 0.015 | 0.002 | 2.12E-14 | -0.015 | 0.003 | 2.80E-09 | TV | -0.014 | 0.013 | -0.009 | 0.016 | -0.023 | 0.016 | -0.017 | 0.013 | -0.006 | 0.017 |
| rs1022785 | 14 | 29571653 | G | A | -0.018 | 0.002 | 2.05E-13 | 0.018 | 0.003 | 7.10E-09 | TV | -0.003 | 0.016 | 0.027 | 0.019 | -0.024 | 0.019 | -0.005 | 0.016 | 0.035 | 0.021 |
| rs61997667 | 14 | 57280046 | T | C | -0.014 | 0.002 | 2.24E-13 | 0.014 | 0.003 | 1.60E-06 | education | -0.003 | 0.016 | -0.003 | 0.019 | 0.008 | 0.020 | 0.016 | 0.016 | -0.018 | 0.021 |
| rs4899012 | 14 | 61003889 | C | G | -0.010 | 0.001 | 8.06E-13 | 0.004 | 0.002 | 9.60E-02 | education | 0.005 | 0.012 | 0.023 | 0.014 | -0.002 | 0.014 | 0.011 | 0.012 | 0.030 | 0.015 |
| rs12888615 | 14 | 72435705 | T | C | 0.012 | 0.002 | 9.42E-12 | -0.012 | 0.003 | 8.00E-06 | education | 0.001 | 0.015 | 0.012 | 0.018 | -0.035 | 0.018 | -0.003 | 0.015 | -0.001 | 0.020 |
| rs730384 | 14 | 74889870 | A | G | 0.010 | 0.001 | 9.38E-13 | -0.005 | 0.002 | 1.20E-02 | education | 0.003 | 0.012 | 0.003 | 0.014 | 0.013 | 0.014 | 0.007 | 0.012 | -0.011 | 0.015 |
| rs2998299 | 14 | 84997363 | T | G | -0.014 | 0.002 | 1.51E-15 | 0.007 | 0.003 | 1.10E-02 | education | -0.007 | 0.014 | 0.000 | 0.016 | -0.003 | 0.016 | -0.008 | 0.014 | 0.003 | 0.018 |
| rs10145592 | 14 | 94287215 | C | G | 0.011 | 0.002 | 1.02E-09 | -0.015 | 0.002 | 1.80E-11 | TV | -0.023 | 0.012 | -0.017 | 0.014 | -0.014 | 0.014 | -0.019 | 0.012 | -0.029 | 0.015 |
| rs7157001 | 14 | 99749484 | A | G | 0.009 | 0.002 | 1.29E-05 | -0.014 | 0.002 | 2.30E-08 | TV | -0.007 | 0.014 | -0.005 | 0.017 | -0.013 | 0.017 | -0.003 | 0.014 | -0.051 | 0.019 |
| rs7167688 | 15 | 26813137 | T | C | -0.009 | 0.001 | 1.71E-11 | 0.005 | 0.002 | 2.10E-02 | education | -0.004 | 0.010 | 0.012 | 0.014 | -0.020 | 0.014 | 0.002 | 0.012 | -0.013 | 0.015 |
| rs891793 | 15 | 27256021 | C | G | -0.010 | 0.001 | 1.87E-12 | 0.005 | 0.002 | 1.90E-02 | education | 0.017 | 0.010 | 0.025 | 0.014 | 0.017 | 0.014 | 0.012 | 0.012 | 0.036 | 0.015 |
| rs8043253 | 15 | 41503730 | C | T | 0.001 | 0.002 | 5.79E-01 | -0.012 | 0.002 | 3.40E-08 | TV | 0.017 | 0.010 | 0.013 | 0.014 | 0.019 | 0.014 | 0.028 | 0.012 | -0.002 | 0.015 |
| rs6493265 | 15 | 47513253 | T | C | -0.012 | 0.001 | 2.26E-17 | 0.001 | 0.002 | 5.20E-01 | education | 0.001 | 0.010 | -0.010 | 0.014 | 0.015 | 0.014 | 0.004 | 0.012 | 0.028 | 0.015 |
| rs2460 | 15 | 53073084 | G | A | 0.009 | 0.002 | 1.04E-05 | -0.015 | 0.002 | 5.00E-10 | TV | -0.015 | 0.011 | 0.004 | 0.016 | -0.032 | 0.016 | -0.021 | 0.013 | 0.015 | 0.018 |
| rs4775373 | 15 | 61476936 | T | C | -0.007 | 0.002 | 2.40E-04 | 0.013 | 0.002 | 4.70E-09 | TV | 0.006 | 0.010 | 0.002 | 0.014 | 0.009 | 0.014 | 0.007 | 0.012 | 0.017 | 0.016 |
| rs12591647 | 15 | 65959729 | T | C | -0.015 | 0.002 | 7.78E-17 | 0.003 | 0.003 | 3.40E-01 | education | 0.005 | 0.012 | 0.005 | 0.018 | 0.006 | 0.017 | -0.010 | 0.015 | 0.035 | 0.020 |
| rs1564347 | 15 | 73438848 | T | G | 0.009 | 0.002 | 2.39E-09 | -0.010 | 0.002 | 7.10E-06 | education | -0.013 | 0.010 | -0.013 | 0.014 | -0.011 | 0.014 | -0.020 | 0.012 | -0.012 | 0.016 |
| rs61331678 | 15 | 77995949 | G | C | -0.013 | 0.002 | 2.63E-13 | 0.015 | 0.002 | 6.10E-12 | TV | -0.011 | 0.010 | 0.004 | 0.014 | -0.025 | 0.014 | -0.015 | 0.012 | -0.011 | 0.015 |
| rs4778058 | 15 | 93456069 | T | C | -0.009 | 0.001 | 4.92E-11 | 0.010 | 0.002 | 2.00E-06 | education | 0.011 | 0.010 | 0.015 | 0.014 | 0.007 | 0.014 | 0.008 | 0.012 | 0.022 | 0.015 |
| rs4984541 | 15 | 96911139 | A | G | -0.014 | 0.002 | 3.40E-16 | 0.006 | 0.003 | 3.00E-02 | education | 0.010 | 0.012 | -0.023 | 0.017 | 0.041 | 0.017 | 0.018 | 0.015 | -0.001 | 0.019 |
| rs8030487 | 15 | 97119595 | A | G | 0.009 | 0.002 | 2.52E-08 | -0.007 | 0.002 | 5.30E-03 | education | -0.012 | 0.011 | 0.003 | 0.015 | -0.025 | 0.015 | -0.017 | 0.012 | 0.003 | 0.016 |
| rs9933256 | 16 | 1246748 | A | G | 0.012 | 0.001 | 6.06E-17 | -0.006 | 0.002 | 2.90E-03 | education | 0.013 | 0.010 | -0.008 | 0.015 | 0.033 | 0.014 | 0.020 | 0.012 | 0.005 | 0.016 |
| rs34098770 | 16 | 12237812 | A | G | -0.016 | 0.002 | 2.95E-16 | 0.007 | 0.003 | 1.40E-02 | education | 0.010 | 0.013 | 0.005 | 0.019 | 0.013 | 0.018 | 0.012 | 0.016 | 0.012 | 0.021 |
| rs35574015 | 16 | 15146061 | T | C | 0.002 | 0.002 | 2.21E-01 | -0.013 | 0.002 | 3.00E-08 | TV | 0.020 | 0.010 | 0.028 | 0.015 | 0.011 | 0.015 | 0.018 | 0.013 | -0.001 | 0.016 |
| rs7189927 | 16 | 28913787 | T | C | -0.015 | 0.002 | 7.31E-18 | 0.015 | 0.002 | 3.40E-11 | TV | 0.009 | 0.010 | 0.006 | 0.015 | 0.012 | 0.015 | 0.016 | 0.012 | 0.009 | 0.016 |
| rs749671 | 16 | 31088347 | G | A | -0.006 | 0.002 | 3.15E-04 | 0.016 | 0.002 | 2.70E-12 | TV | 0.023 | 0.010 | 0.018 | 0.014 | 0.028 | 0.014 | 0.023 | 0.012 | 0.015 | 0.016 |
| rs2052285 | 16 | 51188432 | A | G | 0.011 | 0.001 | 7.25E-15 | -0.008 | 0.002 | 3.50E-04 | education | -0.017 | 0.010 | -0.005 | 0.014 | -0.030 | 0.014 | -0.020 | 0.012 | -0.012 | 0.015 |
| rs7184800 | 16 | 53509131 | G | A | -0.010 | 0.002 | 2.38E-08 | 0.017 | 0.002 | 8.20E-13 | TV | 0.005 | 0.011 | -0.003 | 0.015 | 0.011 | 0.015 | 0.003 | 0.013 | 0.024 | 0.016 |
| rs17248751 | 16 | 61579618 | A | G | -0.014 | 0.002 | 1.28E-15 | 0.006 | 0.003 | 1.30E-02 | education | 0.012 | 0.012 | 0.035 | 0.017 | -0.011 | 0.017 | -0.002 | 0.014 | 0.028 | 0.019 |
| rs9929556 | 16 | 62193640 | T | G | -0.010 | 0.001 | 2.31E-12 | 0.004 | 0.002 | 6.90E-02 | education | 0.007 | 0.010 | 0.010 | 0.014 | 0.001 | 0.014 | 0.023 | 0.012 | -0.010 | 0.015 |
| rs17604349 | 16 | 72210865 | A | G | 0.013 | 0.002 | 2.36E-12 | -0.013 | 0.003 | 5.10E-06 | education | -0.003 | 0.012 | -0.023 | 0.018 | 0.016 | 0.018 | 0.001 | 0.015 | -0.025 | 0.019 |
| rs9929762 | 16 | 78169675 | A | G | 0.010 | 0.001 | 1.03E-11 | -0.008 | 0.002 | 5.50E-04 | education | 0.007 | 0.010 | 0.037 | 0.014 | -0.020 | 0.014 | 0.011 | 0.012 | 0.012 | 0.015 |
| rs61527214 | 16 | 83608599 | A | G | 0.011 | 0.001 | 1.13E-14 | -0.007 | 0.002 | 3.10E-03 | education | -0.018 | 0.010 | -0.019 | 0.014 | -0.015 | 0.014 | -0.027 | 0.012 | 0.006 | 0.016 |
| rs74415461 | 16 | 89990843 | T | C | 0.018 | 0.003 | 6.25E-12 | -0.015 | 0.004 | 1.20E-04 | education | -0.010 | 0.019 | 0.010 | 0.027 | -0.027 | 0.027 | -0.017 | 0.023 | 0.047 | 0.029 |
| rs2447098 | 17 | 2277720 | C | A | 0.008 | 0.002 | 7.73E-07 | -0.015 | 0.002 | 6.80E-12 | TV | -0.011 | 0.010 | -0.034 | 0.014 | 0.009 | 0.014 | -0.010 | 0.012 | -0.003 | 0.015 |
| rs11654952 | 17 | 4813799 | T | G | 0.003 | 0.003 | 3.08E-01 | -0.017 | 0.003 | 4.30E-08 | TV | 0.025 | 0.014 | 0.005 | 0.019 | 0.047 | 0.020 | 0.018 | 0.017 | 0.001 | 0.022 |
| rs12602286 | 17 | 19236954 | T | G | 0.016 | 0.002 | 4.62E-14 | -0.001 | 0.003 | 8.60E-01 | education | 0.006 | 0.015 | 0.010 | 0.021 | -0.001 | 0.021 | -0.005 | 0.018 | 0.007 | 0.023 |
| rs11657979 | 17 | 32900837 | A | G | -0.009 | 0.002 | 4.57E-08 | 0.009 | 0.003 | 6.90E-04 | education | 0.002 | 0.012 | -0.010 | 0.017 | 0.013 | 0.017 | 0.008 | 0.014 | -0.027 | 0.019 |
| rs1842713 | 17 | 33200385 | A | G | -0.012 | 0.002 | 9.67E-12 | 0.010 | 0.003 | 3.00E-04 | education | 0.017 | 0.012 | 0.028 | 0.016 | 0.010 | 0.017 | 0.014 | 0.014 | 0.029 | 0.019 |
| rs4793090 | 17 | 40686342 | A | G | 0.008 | 0.002 | 1.67E-08 | -0.010 | 0.002 | 6.00E-06 | education | -0.012 | 0.010 | -0.024 | 0.015 | 0.001 | 0.014 | -0.016 | 0.012 | -0.020 | 0.016 |
| rs11871429 | 17 | 42920929 | A | G | 0.013 | 0.002 | 2.52E-14 | -0.006 | 0.003 | 1.30E-02 | education | 0.000 | 0.011 | -0.009 | 0.016 | 0.009 | 0.016 | 0.006 | 0.014 | -0.003 | 0.018 |
| rs56319902 | 17 | 43871982 | T | C | -0.021 | 0.002 | 5.81E-33 | 0.005 | 0.003 | 7.50E-02 | education | -0.019 | 0.012 | 0.017 | 0.017 | -0.052 | 0.017 | -0.016 | 0.014 | 0.001 | 0.018 |
| rs56794817 | 17 | 60001978 | A | G | 0.012 | 0.002 | 1.69E-10 | -0.005 | 0.003 | 9.90E-02 | education | -0.002 | 0.013 | 0.006 | 0.018 | -0.008 | 0.018 | 0.001 | 0.016 | -0.030 | 0.020 |
| rs9902312 | 17 | 65070304 | T | C | 0.000 | 0.002 | 8.57E-01 | 0.015 | 0.002 | 4.50E-11 | TV | -0.001 | 0.010 | -0.005 | 0.015 | 0.004 | 0.015 | 0.000 | 0.012 | 0.010 | 0.016 |
| rs68145588 | 17 | 75864805 | T | G | -0.013 | 0.002 | 7.53E-10 | -0.001 | 0.003 | 8.10E-01 | education | -0.026 | 0.014 | -0.018 | 0.020 | -0.037 | 0.020 | -0.039 | 0.017 | -0.022 | 0.023 |
| rs11657730 | 17 | 79372489 | C | T | -0.013 | 0.002 | 4.31E-11 | 0.013 | 0.002 | 1.10E-08 | TV | 0.010 | 0.011 | 0.007 | 0.015 | 0.012 | 0.015 | 0.010 | 0.012 | 0.016 | 0.016 |
| rs12967010 | 18 | 9645035 | T | C | 0.011 | 0.002 | 1.33E-11 | -0.005 | 0.003 | 6.00E-02 | education | -0.001 | 0.012 | 0.011 | 0.017 | -0.015 | 0.017 | -0.002 | 0.014 | -0.019 | 0.018 |
| rs303753 | 18 | 21074922 | G | A | 0.015 | 0.002 | 2.60E-16 | -0.014 | 0.002 | 2.70E-10 | TV | -0.013 | 0.010 | -0.029 | 0.015 | 0.001 | 0.015 | -0.008 | 0.012 | -0.022 | 0.016 |
| rs9964724 | 18 | 35159124 | C | T | -0.020 | 0.002 | 2.66E-27 | 0.018 | 0.002 | 3.30E-14 | TV | 0.009 | 0.010 | 0.014 | 0.015 | 0.004 | 0.015 | 0.011 | 0.012 | 0.012 | 0.016 |
| rs1156541 | 18 | 39952989 | C | T | -0.005 | 0.002 | 1.23E-02 | 0.014 | 0.003 | 2.80E-08 | TV | 0.017 | 0.011 | 0.016 | 0.016 | 0.019 | 0.016 | 0.015 | 0.014 | 0.043 | 0.018 |
| rs17411339 | 18 | 50807359 | A | G | 0.014 | 0.001 | 5.10E-23 | -0.006 | 0.002 | 7.90E-03 | education | -0.011 | 0.010 | -0.009 | 0.014 | -0.014 | 0.014 | -0.008 | 0.012 | -0.009 | 0.015 |
| rs17512836 | 18 | 53194961 | T | C | -0.028 | 0.005 | 4.70E-07 | 0.042 | 0.007 | 7.00E-10 | TV | -0.014 | 0.028 | -0.018 | 0.040 | -0.009 | 0.040 | 0.019 | 0.033 | -0.075 | 0.043 |
| rs72944064 | 18 | 63516255 | T | C | -0.010 | 0.002 | 9.39E-10 | 0.010 | 0.002 | 8.70E-05 | education | 0.024 | 0.011 | 0.033 | 0.016 | 0.017 | 0.016 | 0.024 | 0.014 | 0.020 | 0.018 |
| rs2554835 | 18 | 74141190 | A | G | 0.009 | 0.001 | 8.95E-10 | -0.003 | 0.002 | 2.40E-01 | education | 0.005 | 0.010 | 0.009 | 0.015 | 0.002 | 0.015 | 0.001 | 0.012 | -0.011 | 0.016 |
| rs11081529 | 18 | 75902735 | T | C | 0.013 | 0.002 | 5.22E-16 | -0.004 | 0.002 | 1.10E-01 | education | 0.004 | 0.011 | 0.017 | 0.016 | -0.008 | 0.016 | 0.009 | 0.013 | 0.001 | 0.017 |
| rs11663602 | 18 | 77578191 | A | C | -0.013 | 0.002 | 9.27E-17 | 0.007 | 0.002 | 2.70E-03 | education | 0.012 | 0.011 | 0.003 | 0.015 | 0.019 | 0.015 | 0.015 | 0.013 | 0.030 | 0.017 |
| rs7248205 | 19 | 10770305 | C | T | -0.005 | 0.002 | 6.35E-03 | 0.014 | 0.002 | 3.40E-10 | TV | -0.029 | 0.010 | -0.046 | 0.014 | -0.010 | 0.014 | -0.021 | 0.012 | -0.023 | 0.016 |
| rs12151248 | 19 | 13212025 | T | C | -0.015 | 0.002 | 3.81E-11 | 0.003 | 0.003 | 3.20E-01 | education | 0.005 | 0.016 | -0.005 | 0.023 | 0.007 | 0.023 | 0.025 | 0.019 | 0.005 | 0.025 |
| rs111901094 | 19 | 19513570 | G | T | 0.011 | 0.003 | 5.48E-05 | -0.017 | 0.003 | 2.00E-09 | TV | -0.011 | 0.013 | 0.022 | 0.018 | -0.044 | 0.018 | 0.003 | 0.016 | -0.022 | 0.020 |
| rs7254263 | 19 | 30741257 | T | C | -0.012 | 0.002 | 1.84E-14 | 0.004 | 0.002 | 6.80E-02 | education | -0.003 | 0.011 | -0.011 | 0.015 | 0.005 | 0.015 | -0.010 | 0.013 | 0.017 | 0.017 |
| rs3859523 | 19 | 32972296 | T | C | 0.012 | 0.002 | 9.92E-12 | -0.006 | 0.003 | 2.40E-02 | education | 0.020 | 0.013 | 0.015 | 0.018 | 0.026 | 0.018 | 0.019 | 0.015 | 0.058 | 0.020 |
| rs173003 | 19 | 36240960 | A | C | -0.008 | 0.001 | 5.50E-09 | 0.007 | 0.002 | 2.70E-03 | education | -0.002 | 0.010 | 0.001 | 0.014 | -0.002 | 0.014 | -0.007 | 0.012 | 0.004 | 0.015 |
| rs76246107 | 19 | 50121274 | A | G | -0.016 | 0.003 | 6.52E-10 | 0.022 | 0.004 | 7.00E-08 | education | -0.028 | 0.019 | -0.001 | 0.026 | -0.056 | 0.027 | -0.046 | 0.023 | -0.035 | 0.029 |
| rs192436652 | 19 | 54960747 | T | C | -0.032 | 0.005 | 2.06E-12 | 0.026 | 0.007 | 7.50E-05 | education | 0.027 | 0.031 | 0.067 | 0.044 | -0.018 | 0.044 | 0.023 | 0.038 | 0.048 | 0.049 |
| rs6131281 | 20 | 11891724 | C | T | -0.006 | 0.002 | 2.46E-04 | 0.016 | 0.002 | 3.10E-13 | TV | 0.017 | 0.010 | 0.021 | 0.014 | 0.011 | 0.014 | 0.020 | 0.012 | 0.014 | 0.015 |
| rs6141814 | 20 | 31368960 | C | A | 0.003 | 0.002 | 5.19E-02 | -0.014 | 0.002 | 1.30E-09 | TV | 0.013 | 0.010 | 0.020 | 0.014 | 0.004 | 0.014 | 0.015 | 0.012 | 0.003 | 0.016 |
| rs4369924 | 20 | 41954383 | A | G | 0.013 | 0.002 | 5.58E-11 | -0.012 | 0.003 | 8.60E-05 | education | -0.018 | 0.013 | -0.009 | 0.018 | -0.030 | 0.018 | -0.023 | 0.016 | -0.041 | 0.021 |
| rs55700114 | 20 | 43717080 | G | A | 0.012 | 0.002 | 3.34E-10 | -0.014 | 0.002 | 2.10E-09 | TV | -0.001 | 0.011 | -0.006 | 0.015 | 0.006 | 0.015 | -0.002 | 0.013 | -0.006 | 0.016 |
| rs4810894 | 20 | 47532999 | A | G | -0.009 | 0.002 | 4.38E-09 | 0.008 | 0.002 | 5.70E-04 | education | -0.007 | 0.010 | -0.010 | 0.014 | -0.005 | 0.014 | -0.014 | 0.012 | 0.014 | 0.016 |
| rs6123924 | 20 | 58219764 | A | G | 0.013 | 0.002 | 3.20E-11 | -0.010 | 0.003 | 8.20E-04 | education | 0.004 | 0.013 | 0.014 | 0.018 | -0.004 | 0.018 | 0.003 | 0.016 | 0.022 | 0.021 |
| rs6065080 | 20 | 59832791 | T | C | -0.013 | 0.002 | 7.49E-19 | 0.004 | 0.002 | 5.80E-02 | education | 0.014 | 0.010 | 0.020 | 0.014 | 0.008 | 0.014 | 0.002 | 0.012 | 0.052 | 0.016 |
| rs56103247 | 20 | 62483184 | C | T | -0.016 | 0.004 | 3.14E-05 | 0.030 | 0.005 | 3.80E-10 | TV | -0.077 | 0.024 | -0.075 | 0.035 | -0.080 | 0.034 | -0.074 | 0.029 | -0.074 | 0.038 |
| rs13050131 | 21 | 20018118 | A | C | -0.008 | 0.002 | 4.40E-08 | 0.006 | 0.002 | 1.40E-02 | education | -0.012 | 0.010 | -0.018 | 0.015 | -0.005 | 0.015 | -0.017 | 0.012 | -0.009 | 0.016 |
| rs232496 | 21 | 22734409 | T | C | 0.009 | 0.002 | 7.14E-10 | -0.003 | 0.002 | 2.00E-01 | education | 0.005 | 0.010 | -0.001 | 0.014 | 0.006 | 0.014 | -0.001 | 0.012 | 0.006 | 0.016 |
| rs1544 | 21 | 33085585 | A | G | -0.010 | 0.002 | 1.76E-09 | 0.007 | 0.002 | 4.90E-03 | education | -0.002 | 0.011 | 0.023 | 0.016 | -0.021 | 0.016 | 0.000 | 0.014 | 0.017 | 0.018 |
| rs10427502 | 21 | 40654840 | G | A | -0.004 | 0.002 | 1.36E-02 | 0.014 | 0.002 | 8.60E-10 | TV | 0.001 | 0.010 | 0.024 | 0.014 | -0.017 | 0.014 | -0.006 | 0.012 | 0.000 | 0.016 |
| rs2838006 | 21 | 42653567 | T | C | 0.013 | 0.002 | 1.38E-19 | -0.006 | 0.002 | 5.10E-03 | education | -0.006 | 0.010 | -0.001 | 0.014 | -0.011 | 0.014 | -0.003 | 0.012 | -0.014 | 0.016 |
| rs2092829 | 22 | 31780514 | G | A | -0.002 | 0.002 | 3.89E-01 | 0.014 | 0.002 | 1.30E-08 | TV | -0.023 | 0.011 | -0.027 | 0.015 | -0.016 | 0.015 | -0.026 | 0.013 | -0.029 | 0.017 |
| rs5754753 | 22 | 34296751 | T | C | -0.013 | 0.002 | 6.15E-16 | 0.009 | 0.002 | 1.40E-04 | education | -0.006 | 0.012 | -0.003 | 0.017 | -0.009 | 0.016 | -0.011 | 0.014 | -0.003 | 0.019 |
| rs143743568 | 22 | 39289014 | A | G | 0.012 | 0.002 | 4.99E-09 | -0.008 | 0.003 | 9.40E-03 | education | 0.000 | 0.013 | -0.017 | 0.019 | 0.014 | 0.019 | 0.000 | 0.017 | 0.008 | 0.022 |
| rs137079 | 22 | 42977907 | T | C | 0.013 | 0.002 | 3.06E-10 | -0.016 | 0.003 | 1.90E-07 | education | -0.012 | 0.014 | -0.011 | 0.020 | -0.017 | 0.020 | -0.015 | 0.017 | -0.019 | 0.022 |
| rs9616947 | 22 | 51151631 | T | C | -0.013 | 0.002 | 3.58E-20 | 0.004 | 0.002 | 6.60E-02 | education | 0.005 | 0.011 | 0.009 | 0.016 | 0.002 | 0.016 | 0.013 | 0.013 | -0.032 | 0.017 |

| **Table S22. Beta estimates for the SNPs included in the multivariable MR of smoking, leisure computer use, and breast cancer risk** | | | | | | | | | | | | | | | | | | | | | | | | |
| --- | --- | --- | --- | --- | --- | --- | --- | --- | --- | --- | --- | --- | --- | --- | --- | --- | --- | --- | --- | --- | --- | --- | --- | --- |
| SNP | CHR | POS | EA | OA | beta_smk | se_smk | pval_smk | beta_pc | se_pc | pval_pc | Phenotype | beta_br | se_br | Luminal_A  _beta | Luminal_A  _se | Luminal_B  _beta | Luminal_B  _se | Luminal_B  _HER2Neg  _beta | Luminal_B  _HER2Neg  _se | HER2_  Enriched_  beta | HER2_  Enriched  _se | Triple_Neg  _beta | Triple_Neg  _se |  |
| rs2748985 | 1 | 1853184 | T | C | -0.001 | 0.001 | 4.40E-01 | -0.015 | 0.002 | 4.10E-12 | PC | 0.003 | 0.007 | 0.007 | 0.009 | 0.017 | 0.019 | -0.002 | 0.017 | -0.011 | 0.028 | 0.014 | 0.017 |  |
| rs1193237 | 1 | 7526486 | G | C | -0.008 | 0.001 | 2.80E-08 | -0.001 | 0.002 | 6.90E-01 | Smoking | 0.005 | 0.007 | 0.010 | 0.009 | -0.012 | 0.019 | -0.003 | 0.016 | 0.016 | 0.027 | -0.007 | 0.017 |  |
| rs4949465 | 1 | 32178489 | T | C | -0.012 | 0.002 | 1.70E-08 | 0.007 | 0.003 | 4.40E-02 | Smoking | 0.001 | 0.009 | 0.014 | 0.012 | -0.050 | 0.026 | -0.011 | 0.023 | 0.030 | 0.039 | -0.009 | 0.023 |  |
| rs549845 | 1 | 44076469 | G | A | 0.011 | 0.002 | 8.30E-14 | 0.002 | 0.002 | 3.60E-01 | Smoking | -0.015 | 0.007 | -0.014 | 0.009 | -0.042 | 0.020 | -0.002 | 0.017 | 0.021 | 0.028 | -0.009 | 0.017 |  |
| rs1933270 | 1 | 49977965 | T | G | 0.009 | 0.001 | 1.50E-10 | -0.005 | 0.002 | 3.50E-02 | Smoking | -0.007 | 0.006 | -0.011 | 0.008 | 0.018 | 0.018 | -0.016 | 0.015 | -0.015 | 0.026 | 0.002 | 0.016 |  |
| rs7528604 | 1 | 66407352 | G | A | 0.010 | 0.001 | 5.70E-12 | -0.001 | 0.002 | 5.20E-01 | Smoking | -0.017 | 0.006 | -0.027 | 0.008 | -0.010 | 0.018 | -0.022 | 0.016 | 0.018 | 0.027 | -0.027 | 0.016 |  |
| rs11210229 | 1 | 73860028 | A | G | 0.012 | 0.001 | 2.00E-16 | -0.002 | 0.002 | 4.70E-01 | Smoking | 0.002 | 0.006 | -0.001 | 0.008 | -0.013 | 0.018 | 0.009 | 0.015 | 0.014 | 0.026 | 0.006 | 0.015 |  |
| rs7553348 | 1 | 75005067 | G | A | 0.010 | 0.001 | 5.20E-12 | -0.004 | 0.002 | 6.20E-02 | Smoking | -0.007 | 0.006 | -0.006 | 0.008 | -0.030 | 0.018 | 0.005 | 0.015 | -0.007 | 0.026 | -0.008 | 0.015 |  |
| rs10922907 | 1 | 91193049 | A | T | 0.010 | 0.001 | 3.00E-13 | -0.003 | 0.002 | 2.20E-01 | Smoking | -0.008 | 0.006 | 0.001 | 0.009 | -0.024 | 0.019 | -0.016 | 0.016 | 0.016 | 0.027 | -0.006 | 0.016 |  |
| rs1931263 | 1 | 96175101 | G | T | -0.008 | 0.001 | 4.00E-08 | 0.007 | 0.002 | 1.90E-03 | Smoking | -0.009 | 0.006 | 0.001 | 0.008 | -0.041 | 0.018 | -0.005 | 0.015 | 0.001 | 0.025 | -0.015 | 0.015 |  |
| rs7519626 | 1 | 99514554 | C | T | 0.008 | 0.001 | 1.20E-08 | -0.001 | 0.002 | 5.60E-01 | Smoking | 0.004 | 0.006 | -0.002 | 0.009 | -0.001 | 0.019 | 0.022 | 0.016 | 0.002 | 0.028 | 0.007 | 0.016 |  |
| rs9435340 | 1 | 107593201 | T | A | 0.008 | 0.001 | 1.20E-08 | 0.000 | 0.002 | 9.20E-01 | Smoking | 0.002 | 0.006 | -0.002 | 0.008 | -0.014 | 0.019 | 0.022 | 0.016 | 0.008 | 0.027 | 0.025 | 0.016 |  |
| rs12145677 | 1 | 110023610 | G | A | 0.002 | 0.002 | 2.20E-01 | -0.017 | 0.002 | 1.20E-12 | PC | -0.011 | 0.007 | -0.016 | 0.009 | -0.012 | 0.020 | -0.003 | 0.017 | -0.009 | 0.028 | 0.013 | 0.017 |  |
| rs10918701 | 1 | 162090536 | G | A | 0.008 | 0.001 | 2.10E-08 | -0.007 | 0.002 | 2.40E-03 | Smoking | 0.004 | 0.006 | 0.005 | 0.008 | 0.014 | 0.018 | -0.008 | 0.016 | 0.023 | 0.026 | 0.024 | 0.016 |  |
| rs7526456 | 1 | 197773734 | T | C | -0.001 | 0.001 | 3.00E-01 | -0.013 | 0.002 | 1.20E-08 | PC | 0.005 | 0.006 | 0.005 | 0.009 | 0.012 | 0.019 | 0.002 | 0.016 | -0.039 | 0.028 | 0.014 | 0.016 |  |
| rs2867112 | 2 | 651349 | T | G | 0.015 | 0.002 | 4.80E-15 | 0.009 | 0.003 | 1.80E-03 | Smoking | -0.030 | 0.008 | -0.017 | 0.010 | -0.013 | 0.023 | -0.022 | 0.020 | -0.099 | 0.032 | -0.034 | 0.020 |  |
| rs6741228 | 2 | 22548774 | T | C | 0.008 | 0.001 | 1.60E-08 | -0.002 | 0.002 | 3.70E-01 | Smoking | -0.004 | 0.006 | -0.006 | 0.008 | 0.011 | 0.018 | -0.005 | 0.015 | 0.027 | 0.026 | 0.005 | 0.015 |  |
| rs62135536 | 2 | 44326028 | C | T | 0.024 | 0.004 | 8.00E-10 | 0.005 | 0.006 | 4.10E-01 | Smoking | 0.021 | 0.019 | 0.016 | 0.025 | -0.026 | 0.056 | 0.032 | 0.049 | 0.112 | 0.088 | -0.075 | 0.048 |  |
| rs7569203 | 2 | 45154418 | A | C | -0.011 | 0.002 | 7.40E-13 | 0.000 | 0.002 | 8.80E-01 | Smoking | 0.001 | 0.006 | 0.009 | 0.008 | -0.025 | 0.019 | 0.017 | 0.016 | -0.059 | 0.027 | 0.011 | 0.016 |  |
| rs13016665 | 2 | 57995348 | C | A | -0.008 | 0.001 | 1.80E-09 | 0.010 | 0.002 | 2.20E-05 | Smoking | 0.012 | 0.006 | 0.012 | 0.008 | 0.014 | 0.018 | 0.019 | 0.015 | 0.014 | 0.026 | 0.022 | 0.016 |  |
| rs4671357 | 2 | 60136176 | T | C | -0.009 | 0.001 | 1.10E-11 | -0.001 | 0.002 | 5.10E-01 | Smoking | -0.006 | 0.006 | -0.007 | 0.008 | 0.005 | 0.018 | -0.010 | 0.015 | -0.006 | 0.026 | -0.015 | 0.016 |  |
| rs359243 | 2 | 60475509 | T | C | -0.009 | 0.001 | 9.50E-10 | -0.010 | 0.002 | 2.50E-05 | Smoking | -0.009 | 0.006 | -0.015 | 0.008 | -0.028 | 0.018 | -0.019 | 0.015 | 0.030 | 0.026 | 0.017 | 0.016 |  |
| rs2678670 | 2 | 104469564 | A | T | 0.009 | 0.001 | 3.10E-10 | -0.002 | 0.002 | 4.50E-01 | Smoking | 0.015 | 0.006 | 0.012 | 0.008 | -0.004 | 0.018 | 0.023 | 0.015 | -0.001 | 0.026 | 0.032 | 0.015 |  |
| rs62155874 | 2 | 105973094 | A | G | -0.017 | 0.002 | 5.20E-16 | 0.002 | 0.003 | 5.80E-01 | Smoking | 0.003 | 0.009 | 0.001 | 0.012 | 0.018 | 0.027 | -0.004 | 0.023 | 0.013 | 0.040 | 0.011 | 0.024 |  |
| rs3811038 | 2 | 113240183 | T | C | -0.010 | 0.002 | 8.90E-10 | -0.004 | 0.002 | 1.40E-01 | Smoking | -0.006 | 0.007 | -0.016 | 0.009 | 0.014 | 0.019 | -0.003 | 0.017 | 0.031 | 0.029 | -0.008 | 0.017 |  |
| rs10754920 | 2 | 146456888 | C | G | 0.003 | 0.002 | 2.10E-01 | 0.020 | 0.003 | 6.70E-09 | PC | 0.004 | 0.010 | 0.016 | 0.013 | 0.005 | 0.028 | 0.015 | 0.024 | 0.014 | 0.040 | -0.064 | 0.025 |  |
| rs6744254 | 2 | 155986195 | C | T | -0.006 | 0.001 | 9.40E-06 | -0.016 | 0.002 | 5.40E-13 | PC | 0.007 | 0.006 | 0.002 | 0.008 | -0.014 | 0.018 | 0.005 | 0.015 | 0.024 | 0.026 | 0.004 | 0.015 |  |
| rs62175972 | 2 | 161362830 | T | C | 0.022 | 0.004 | 1.70E-08 | -0.005 | 0.006 | 4.10E-01 | Smoking | 0.019 | 0.016 | 0.005 | 0.021 | 0.035 | 0.048 | 0.022 | 0.040 | 0.027 | 0.069 | 0.002 | 0.041 |  |
| rs3769949 | 2 | 166199284 | T | A | -0.008 | 0.001 | 2.50E-09 | 0.005 | 0.002 | 2.70E-02 | Smoking | -0.007 | 0.006 | -0.001 | 0.008 | 0.000 | 0.018 | -0.013 | 0.015 | -0.010 | 0.026 | -0.037 | 0.015 |  |
| rs13009008 | 2 | 174043233 | A | G | 0.009 | 0.001 | 4.60E-09 | 0.000 | 0.002 | 8.70E-01 | Smoking | 0.007 | 0.006 | 0.006 | 0.008 | 0.005 | 0.019 | 0.006 | 0.016 | -0.037 | 0.027 | 0.025 | 0.016 |  |
| rs4473348 | 2 | 182073742 | A | T | -0.010 | 0.002 | 6.40E-11 | 0.003 | 0.003 | 3.10E-01 | Smoking | 0.003 | 0.007 | 0.000 | 0.009 | -0.023 | 0.021 | -0.008 | 0.018 | -0.040 | 0.030 | 0.024 | 0.018 |  |
| rs12623702 | 2 | 202885506 | A | G | -0.010 | 0.001 | 7.70E-12 | 0.007 | 0.002 | 3.60E-03 | Smoking | 0.010 | 0.006 | 0.005 | 0.008 | 0.007 | 0.018 | 0.037 | 0.016 | -0.065 | 0.027 | -0.017 | 0.016 |  |
| rs11274218 | 2 | 215035001 | C | T | -0.004 | 0.001 | 7.80E-03 | -0.014 | 0.002 | 2.50E-09 | PC | 0.014 | 0.006 | 0.008 | 0.008 | 0.030 | 0.018 | 0.003 | 0.016 | 0.006 | 0.027 | 0.021 | 0.016 |  |
| rs10208088 | 2 | 221055873 | C | T | 0.001 | 0.001 | 6.90E-01 | 0.013 | 0.002 | 4.40E-09 | PC | 0.001 | 0.006 | 0.002 | 0.008 | -0.021 | 0.018 | 0.012 | 0.015 | 0.000 | 0.026 | 0.006 | 0.015 |  |
| rs6779302 | 3 | 16859710 | G | T | -0.009 | 0.001 | 1.20E-09 | -0.004 | 0.002 | 7.60E-02 | Smoking | 0.003 | 0.006 | 0.000 | 0.008 | 0.017 | 0.018 | -0.029 | 0.015 | 0.024 | 0.027 | 0.012 | 0.016 |  |
| rs11708955 | 3 | 49540114 | T | C | 0.007 | 0.002 | 1.20E-05 | -0.016 | 0.002 | 4.20E-11 | PC | 0.023 | 0.007 | 0.018 | 0.009 | 0.016 | 0.019 | 0.025 | 0.016 | 0.062 | 0.028 | 0.053 | 0.016 |  |
| rs6774533 | 3 | 62471086 | C | T | 0.001 | 0.002 | 4.50E-01 | -0.015 | 0.002 | 1.20E-09 | PC | -0.012 | 0.007 | -0.022 | 0.009 | -0.004 | 0.021 | -0.004 | 0.018 | -0.047 | 0.031 | 0.018 | 0.018 |  |
| rs775758 | 3 | 77582005 | A | T | 0.008 | 0.001 | 1.10E-08 | 0.003 | 0.002 | 1.80E-01 | Smoking | 0.002 | 0.006 | 0.008 | 0.009 | 0.010 | 0.019 | 0.019 | 0.016 | -0.002 | 0.027 | 0.007 | 0.016 |  |
| rs421983 | 3 | 84892866 | T | C | 0.009 | 0.001 | 3.30E-10 | 0.004 | 0.002 | 6.90E-02 | Smoking | 0.002 | 0.006 | 0.001 | 0.008 | -0.011 | 0.018 | 0.010 | 0.015 | 0.005 | 0.026 | 0.010 | 0.016 |  |
| rs78082503 | 3 | 116556913 | G | C | 0.004 | 0.002 | 1.30E-02 | -0.015 | 0.003 | 1.10E-08 | PC | 0.004 | 0.008 | -0.001 | 0.010 | 0.022 | 0.022 | 0.013 | 0.019 | 0.054 | 0.032 | 0.012 | 0.019 |  |
| rs73220544 | 3 | 131074511 | A | C | -0.011 | 0.002 | 1.50E-08 | 0.001 | 0.003 | 6.90E-01 | Smoking | -0.002 | 0.009 | -0.002 | 0.011 | -0.013 | 0.024 | 0.001 | 0.021 | 0.056 | 0.036 | 0.011 | 0.021 |  |
| rs9842947 | 3 | 157412246 | C | T | -0.009 | 0.001 | 3.10E-09 | -0.001 | 0.002 | 5.40E-01 | Smoking | 0.006 | 0.007 | 0.012 | 0.009 | -0.006 | 0.019 | 0.023 | 0.017 | 0.040 | 0.028 | -0.009 | 0.017 |  |
| rs624833 | 4 | 2881256 | T | G | 0.009 | 0.002 | 6.60E-10 | -0.007 | 0.002 | 5.70E-03 | Smoking | 0.001 | 0.006 | -0.003 | 0.009 | -0.028 | 0.019 | 0.019 | 0.016 | -0.004 | 0.027 | 0.012 | 0.016 |  |
| rs61796681 | 4 | 23678196 | A | T | -0.013 | 0.002 | 4.20E-08 | 0.009 | 0.004 | 1.70E-02 | Smoking | -0.010 | 0.011 | -0.010 | 0.015 | -0.029 | 0.032 | -0.031 | 0.027 | -0.017 | 0.048 | 0.008 | 0.028 |  |
| rs317021 | 4 | 35418368 | T | A | -0.012 | 0.002 | 1.10E-10 | 0.006 | 0.003 | 2.90E-02 | Smoking | -0.008 | 0.009 | -0.009 | 0.011 | 0.035 | 0.025 | -0.053 | 0.021 | -0.012 | 0.036 | -0.015 | 0.022 |  |
| rs6857629 | 4 | 39684977 | G | A | -0.005 | 0.002 | 1.70E-03 | 0.015 | 0.003 | 1.90E-09 | PC | 0.009 | 0.007 | 0.014 | 0.009 | 0.003 | 0.021 | 0.023 | 0.018 | 0.005 | 0.030 | 0.015 | 0.018 |  |
| rs72678864 | 4 | 112422145 | G | A | 0.012 | 0.002 | 1.60E-11 | -0.004 | 0.003 | 1.50E-01 | Smoking | -0.008 | 0.009 | 0.003 | 0.011 | -0.029 | 0.025 | -0.016 | 0.021 | 0.041 | 0.037 | -0.059 | 0.021 |  |
| rs17576594 | 4 | 147952241 | G | A | 0.011 | 0.002 | 1.70E-12 | -0.002 | 0.002 | 5.10E-01 | Smoking | -0.001 | 0.007 | -0.005 | 0.009 | -0.013 | 0.020 | -0.020 | 0.017 | -0.002 | 0.029 | -0.006 | 0.017 |  |
| rs2068625 | 4 | 159856739 | T | C | 0.002 | 0.002 | 2.90E-01 | -0.016 | 0.002 | 4.00E-11 | PC | -0.005 | 0.007 | -0.013 | 0.009 | -0.022 | 0.020 | -0.002 | 0.017 | 0.041 | 0.028 | -0.014 | 0.017 |  |
| rs2220599 | 5 | 7378854 | C | G | 0.001 | 0.001 | 4.50E-01 | -0.016 | 0.002 | 2.40E-12 | PC | 0.008 | 0.007 | 0.014 | 0.009 | 0.018 | 0.019 | 0.005 | 0.016 | -0.005 | 0.027 | -0.015 | 0.016 |  |
| rs71627581 | 5 | 43161351 | G | A | 0.013 | 0.002 | 1.60E-09 | -0.005 | 0.003 | 1.90E-01 | Smoking | 0.014 | 0.010 | 0.016 | 0.013 | 0.002 | 0.029 | 0.020 | 0.025 | 0.007 | 0.042 | -0.018 | 0.025 |  |
| rs2080870 | 5 | 60388313 | A | T | 0.009 | 0.002 | 4.90E-08 | -0.010 | 0.003 | 4.20E-05 | Smoking | -0.016 | 0.007 | -0.020 | 0.009 | -0.016 | 0.020 | -0.010 | 0.017 | -0.010 | 0.029 | -0.014 | 0.017 |  |
| rs11749912 | 5 | 88065628 | A | G | -0.004 | 0.001 | 6.00E-03 | 0.014 | 0.002 | 4.90E-10 | PC | 0.002 | 0.006 | -0.001 | 0.008 | 0.010 | 0.018 | 0.019 | 0.015 | -0.015 | 0.026 | 0.006 | 0.015 |  |
| rs4957528 | 5 | 106420589 | A | C | -0.010 | 0.002 | 4.20E-09 | 0.002 | 0.003 | 5.00E-01 | Smoking | -0.025 | 0.008 | -0.023 | 0.010 | -0.035 | 0.023 | -0.017 | 0.020 | -0.028 | 0.033 | 0.004 | 0.020 |  |
| rs1469249 | 5 | 113837198 | G | A | -0.001 | 0.002 | 4.00E-01 | 0.016 | 0.003 | 9.50E-09 | PC | -0.011 | 0.007 | -0.008 | 0.010 | -0.023 | 0.021 | -0.006 | 0.018 | -0.013 | 0.031 | -0.019 | 0.019 |  |
| rs162894 | 5 | 131611872 | T | G | -0.001 | 0.001 | 3.60E-01 | 0.013 | 0.002 | 1.10E-08 | PC | -0.024 | 0.006 | -0.034 | 0.008 | -0.048 | 0.019 | -0.031 | 0.016 | 0.013 | 0.027 | -0.030 | 0.016 |  |
| rs329120 | 5 | 133861756 | C | T | 0.010 | 0.001 | 6.30E-12 | -0.005 | 0.002 | 1.70E-02 | Smoking | 0.004 | 0.006 | 0.007 | 0.008 | 0.028 | 0.018 | 0.017 | 0.015 | -0.018 | 0.026 | 0.027 | 0.015 |  |
| rs56158102 | 5 | 144027803 | C | T | 0.002 | 0.001 | 1.80E-01 | 0.013 | 0.002 | 3.10E-08 | PC | -0.015 | 0.006 | -0.012 | 0.008 | -0.042 | 0.019 | -0.016 | 0.016 | -0.054 | 0.027 | -0.015 | 0.016 |  |
| rs986391 | 5 | 166993972 | G | A | 0.011 | 0.001 | 9.40E-15 | -0.006 | 0.002 | 1.60E-02 | Smoking | 0.011 | 0.007 | 0.018 | 0.009 | -0.001 | 0.019 | 0.019 | 0.017 | -0.018 | 0.028 | 0.004 | 0.017 |  |
| rs13153393 | 5 | 167604213 | A | G | -0.014 | 0.002 | 2.50E-10 | -0.002 | 0.003 | 6.60E-01 | Smoking | -0.010 | 0.010 | -0.029 | 0.013 | 0.010 | 0.030 | -0.019 | 0.025 | 0.042 | 0.044 | 0.002 | 0.026 |  |
| rs245774 | 5 | 170530930 | A | G | -0.009 | 0.002 | 7.40E-09 | -0.002 | 0.002 | 5.00E-01 | Smoking | 0.000 | 0.007 | -0.006 | 0.009 | 0.020 | 0.020 | -0.003 | 0.017 | -0.010 | 0.029 | 0.020 | 0.017 |  |
| rs9477970 | 6 | 19082663 | T | A | -0.002 | 0.002 | 3.30E-01 | -0.016 | 0.003 | 1.00E-08 | PC | -0.002 | 0.008 | 0.001 | 0.010 | -0.001 | 0.023 | 0.017 | 0.020 | 0.007 | 0.033 | -0.002 | 0.020 |  |
| rs6935954 | 6 | 26255451 | A | G | 0.010 | 0.001 | 8.20E-12 | -0.008 | 0.002 | 4.40E-04 | Smoking | -0.002 | 0.006 | 0.007 | 0.008 | 0.007 | 0.018 | 0.002 | 0.015 | -0.026 | 0.026 | -0.015 | 0.016 |  |
| rs2254710 | 6 | 37477000 | C | A | 0.009 | 0.002 | 3.50E-08 | 0.002 | 0.003 | 5.20E-01 | Smoking | 0.000 | 0.007 | 0.008 | 0.009 | -0.016 | 0.021 | -0.006 | 0.018 | 0.021 | 0.030 | -0.006 | 0.018 |  |
| rs2894808 | 6 | 52861990 | T | A | -0.015 | 0.003 | 3.50E-09 | 0.001 | 0.004 | 7.60E-01 | Smoking | -0.021 | 0.011 | -0.021 | 0.015 | 0.027 | 0.033 | -0.009 | 0.028 | -0.040 | 0.047 | -0.013 | 0.028 |  |
| rs12202536 | 6 | 67475273 | A | G | -0.008 | 0.001 | 2.80E-09 | 0.002 | 0.002 | 2.90E-01 | Smoking | 0.000 | 0.006 | -0.001 | 0.008 | 0.016 | 0.017 | -0.003 | 0.015 | 0.025 | 0.025 | -0.008 | 0.015 |  |
| rs9372625 | 6 | 98344031 | G | A | 0.002 | 0.001 | 1.00E-01 | -0.018 | 0.002 | 5.70E-16 | PC | 0.005 | 0.006 | 0.009 | 0.008 | 0.003 | 0.019 | 0.003 | 0.016 | -0.008 | 0.027 | 0.006 | 0.016 |  |
| rs7766610 | 6 | 111707821 | C | A | 0.013 | 0.002 | 2.20E-12 | 0.000 | 0.003 | 9.20E-01 | Smoking | -0.004 | 0.008 | -0.004 | 0.010 | -0.011 | 0.022 | -0.030 | 0.019 | -0.003 | 0.032 | -0.015 | 0.019 |  |
| rs6935828 | 6 | 140811367 | C | T | 0.000 | 0.001 | 9.90E-01 | -0.012 | 0.002 | 4.30E-08 | PC | -0.004 | 0.006 | -0.003 | 0.008 | 0.018 | 0.018 | 0.007 | 0.015 | -0.028 | 0.026 | 0.001 | 0.015 |  |
| rs1922018 | 7 | 3560401 | C | T | 0.010 | 0.001 | 3.00E-12 | 0.000 | 0.002 | 8.80E-01 | Smoking | 0.001 | 0.006 | 0.002 | 0.008 | -0.015 | 0.018 | 0.006 | 0.016 | 0.000 | 0.027 | 0.034 | 0.016 |  |
| rs10226228 | 7 | 32315613 | A | G | -0.011 | 0.001 | 2.00E-15 | 0.002 | 0.002 | 4.40E-01 | Smoking | 0.013 | 0.006 | 0.014 | 0.008 | 0.034 | 0.018 | 0.007 | 0.016 | -0.016 | 0.027 | 0.005 | 0.016 |  |
| rs55772938 | 7 | 70025113 | A | G | -0.002 | 0.002 | 1.40E-01 | -0.015 | 0.002 | 3.90E-10 | PC | 0.003 | 0.007 | -0.003 | 0.009 | 0.020 | 0.019 | -0.024 | 0.016 | 0.042 | 0.028 | 0.015 | 0.017 |  |
| rs11768481 | 7 | 96629103 | C | A | 0.009 | 0.001 | 9.90E-10 | 0.001 | 0.002 | 6.80E-01 | Smoking | 0.005 | 0.007 | 0.000 | 0.010 | 0.026 | 0.021 | -0.006 | 0.018 | -0.010 | 0.030 | 0.030 | 0.018 |  |
| rs6962772 | 7 | 99081730 | A | G | 0.011 | 0.002 | 7.80E-09 | 0.012 | 0.003 | 5.30E-05 | Smoking | 0.010 | 0.008 | 0.002 | 0.011 | 0.019 | 0.024 | 0.003 | 0.021 | 0.037 | 0.035 | 0.020 | 0.021 |  |
| rs10282292 | 7 | 111092478 | C | T | 0.009 | 0.001 | 5.90E-10 | 0.006 | 0.002 | 4.80E-03 | Smoking | -0.008 | 0.006 | -0.006 | 0.008 | -0.001 | 0.019 | -0.015 | 0.016 | -0.030 | 0.027 | 0.019 | 0.016 |  |
| rs2401924 | 7 | 115057862 | G | C | 0.011 | 0.001 | 2.70E-14 | 0.008 | 0.002 | 6.60E-04 | Smoking | 0.002 | 0.006 | -0.003 | 0.008 | -0.011 | 0.018 | 0.004 | 0.016 | 0.012 | 0.027 | -0.015 | 0.016 |  |
| rs7807019 | 7 | 117543063 | A | G | -0.010 | 0.001 | 6.70E-14 | -0.001 | 0.002 | 6.40E-01 | Smoking | -0.001 | 0.006 | -0.020 | 0.008 | 0.017 | 0.018 | -0.001 | 0.015 | 0.072 | 0.026 | -0.002 | 0.015 |  |
| rs12706626 | 7 | 124531370 | G | A | -0.001 | 0.001 | 3.20E-01 | -0.013 | 0.002 | 1.40E-08 | PC | -0.009 | 0.006 | -0.016 | 0.008 | 0.012 | 0.018 | -0.015 | 0.015 | 0.006 | 0.026 | 0.004 | 0.016 |  |
| rs3944151 | 7 | 126970005 | A | G | -0.002 | 0.002 | 2.00E-01 | 0.014 | 0.002 | 3.10E-08 | PC | 0.016 | 0.007 | 0.013 | 0.009 | 0.003 | 0.020 | 0.015 | 0.017 | 0.037 | 0.028 | 0.010 | 0.017 |  |
| rs2345941 | 7 | 133337635 | A | G | -0.005 | 0.001 | 4.00E-04 | 0.015 | 0.002 | 4.00E-11 | PC | 0.007 | 0.006 | 0.002 | 0.008 | 0.036 | 0.018 | 0.007 | 0.015 | 0.019 | 0.026 | -0.002 | 0.015 |  |
| rs35169606 | 8 | 9604066 | T | G | 0.009 | 0.001 | 1.20E-09 | 0.002 | 0.002 | 4.80E-01 | Smoking | -0.007 | 0.007 | 0.000 | 0.010 | -0.001 | 0.021 | 0.004 | 0.017 | -0.040 | 0.029 | 0.003 | 0.018 |  |
| rs11783093 | 8 | 27425349 | C | T | 0.016 | 0.002 | 1.20E-16 | -0.001 | 0.003 | 6.30E-01 | Smoking | 0.032 | 0.009 | 0.044 | 0.011 | 0.038 | 0.025 | 0.034 | 0.021 | 0.003 | 0.036 | 0.008 | 0.021 |  |
| rs2062882 | 8 | 91839576 | G | A | -0.008 | 0.001 | 1.10E-08 | 0.008 | 0.002 | 8.60E-04 | Smoking | -0.008 | 0.006 | -0.004 | 0.008 | -0.025 | 0.018 | -0.006 | 0.016 | -0.018 | 0.026 | -0.034 | 0.016 |  |
| rs72674867 | 8 | 95578201 | A | T | 0.009 | 0.002 | 3.80E-08 | 0.001 | 0.003 | 6.00E-01 | Smoking | -0.003 | 0.007 | -0.006 | 0.009 | 0.019 | 0.020 | 0.000 | 0.017 | 0.010 | 0.029 | -0.007 | 0.017 |  |
| rs13262595 | 8 | 143316970 | A | G | 0.000 | 0.001 | 8.00E-01 | -0.016 | 0.002 | 1.20E-12 | PC | -0.001 | 0.006 | 0.005 | 0.008 | -0.016 | 0.018 | 0.013 | 0.015 | 0.043 | 0.026 | -0.005 | 0.015 |  |
| rs4543592 | 9 | 3014254 | T | C | -0.009 | 0.001 | 4.50E-10 | -0.001 | 0.002 | 8.00E-01 | Smoking | 0.006 | 0.006 | 0.004 | 0.008 | -0.006 | 0.018 | 0.007 | 0.015 | 0.001 | 0.026 | -0.005 | 0.015 |  |
| rs4977839 | 9 | 23355310 | G | A | 0.004 | 0.001 | 1.10E-02 | -0.020 | 0.002 | 4.70E-19 | PC | -0.006 | 0.006 | -0.001 | 0.008 | -0.037 | 0.019 | -0.006 | 0.016 | 0.019 | 0.027 | -0.022 | 0.016 |  |
| rs7039819 | 9 | 82430418 | G | A | 0.009 | 0.001 | 5.10E-10 | -0.001 | 0.002 | 5.20E-01 | Smoking | 0.014 | 0.006 | 0.015 | 0.008 | 0.006 | 0.019 | 0.016 | 0.016 | 0.002 | 0.027 | 0.028 | 0.016 |  |
| rs1246265 | 9 | 86761745 | T | C | -0.009 | 0.002 | 4.20E-09 | 0.002 | 0.002 | 4.40E-01 | Smoking | 0.001 | 0.007 | 0.006 | 0.009 | -0.004 | 0.019 | 0.030 | 0.016 | -0.013 | 0.028 | -0.010 | 0.017 |  |
| rs113851275 | 9 | 98297220 | G | A | -0.003 | 0.002 | 2.60E-01 | -0.021 | 0.004 | 3.20E-09 | PC | -0.028 | 0.010 | -0.044 | 0.012 | -0.025 | 0.028 | -0.009 | 0.024 | 0.010 | 0.041 | 0.008 | 0.024 |  |
| rs7020477 | 9 | 116827760 | A | G | 0.002 | 0.002 | 1.10E-01 | 0.014 | 0.002 | 1.20E-08 | PC | -0.005 | 0.008 | -0.011 | 0.010 | -0.012 | 0.022 | 0.007 | 0.019 | 0.007 | 0.033 | 0.037 | 0.020 |  |
| rs73578186 | 9 | 126334485 | C | T | 0.000 | 0.001 | 7.60E-01 | 0.015 | 0.002 | 2.30E-10 | PC | -0.003 | 0.006 | -0.011 | 0.008 | 0.011 | 0.019 | -0.021 | 0.016 | -0.002 | 0.027 | -0.028 | 0.016 |  |
| rs113382419 | 9 | 136463019 | C | A | -0.028 | 0.002 | 3.00E-37 | 0.000 | 0.003 | 9.00E-01 | Smoking | -0.014 | 0.010 | -0.020 | 0.013 | -0.011 | 0.029 | 0.028 | 0.025 | 0.040 | 0.044 | -0.033 | 0.025 |  |
| rs11255908 | 10 | 8802912 | T | G | -0.010 | 0.002 | 2.30E-10 | 0.003 | 0.003 | 1.80E-01 | Smoking | -0.026 | 0.007 | -0.039 | 0.009 | -0.010 | 0.021 | -0.010 | 0.018 | 0.016 | 0.030 | -0.022 | 0.018 |  |
| rs2675638 | 10 | 63576286 | G | A | 0.008 | 0.001 | 1.30E-09 | -0.001 | 0.002 | 5.40E-01 | Smoking | 0.006 | 0.006 | 0.002 | 0.008 | 0.013 | 0.018 | -0.001 | 0.015 | 0.014 | 0.026 | 0.019 | 0.016 |  |
| rs10823968 | 10 | 74738269 | A | T | 0.008 | 0.001 | 2.10E-08 | -0.005 | 0.002 | 5.00E-02 | Smoking | 0.003 | 0.006 | -0.009 | 0.008 | 0.004 | 0.018 | 0.012 | 0.015 | 0.000 | 0.026 | 0.000 | 0.016 |  |
| rs17553262 | 10 | 92912773 | A | C | -0.013 | 0.002 | 5.30E-09 | -0.001 | 0.003 | 7.80E-01 | Smoking | -0.016 | 0.010 | -0.011 | 0.013 | 0.023 | 0.029 | -0.041 | 0.024 | 0.014 | 0.042 | -0.050 | 0.024 |  |
| rs7077678 | 10 | 104438565 | C | T | 0.009 | 0.001 | 2.60E-09 | 0.005 | 0.002 | 4.10E-02 | Smoking | 0.007 | 0.006 | -0.003 | 0.008 | 0.011 | 0.018 | 0.008 | 0.015 | -0.024 | 0.026 | -0.013 | 0.016 |  |
| rs12244388 | 10 | 104640052 | G | A | -0.013 | 0.001 | 1.40E-19 | 0.000 | 0.002 | 8.90E-01 | Smoking | -0.015 | 0.006 | -0.019 | 0.008 | 0.007 | 0.018 | -0.046 | 0.016 | -0.023 | 0.026 | -0.006 | 0.016 |  |
| rs3896224 | 10 | 106467853 | A | G | 0.010 | 0.001 | 1.10E-11 | 0.006 | 0.002 | 1.10E-02 | Smoking | 0.015 | 0.006 | 0.018 | 0.008 | 0.020 | 0.018 | 0.013 | 0.015 | 0.037 | 0.026 | 0.019 | 0.015 |  |
| rs34866095 | 11 | 16377356 | A | G | -0.009 | 0.002 | 1.20E-08 | -0.002 | 0.002 | 4.40E-01 | Smoking | 0.001 | 0.006 | 0.001 | 0.009 | 0.024 | 0.019 | -0.003 | 0.016 | 0.000 | 0.028 | 0.025 | 0.016 |  |
| rs75742406 | 11 | 17070365 | G | A | 0.010 | 0.002 | 1.30E-09 | -0.003 | 0.003 | 2.70E-01 | Smoking | -0.004 | 0.007 | -0.010 | 0.009 | -0.013 | 0.021 | -0.017 | 0.018 | -0.001 | 0.030 | -0.008 | 0.018 |  |
| rs17309874 | 11 | 27667236 | G | A | -0.011 | 0.002 | 9.70E-13 | -0.006 | 0.003 | 1.20E-02 | Smoking | 0.009 | 0.007 | 0.014 | 0.009 | -0.016 | 0.021 | 0.028 | 0.018 | -0.016 | 0.030 | -0.005 | 0.018 |  |
| rs4391802 | 11 | 28674592 | A | G | 0.010 | 0.002 | 1.40E-11 | -0.004 | 0.002 | 1.30E-01 | Smoking | -0.004 | 0.007 | -0.006 | 0.009 | -0.009 | 0.020 | 0.009 | 0.017 | 0.022 | 0.029 | -0.005 | 0.017 |  |
| rs112282219 | 11 | 46632809 | G | A | -0.023 | 0.004 | 3.80E-11 | 0.004 | 0.006 | 4.50E-01 | Smoking | -0.034 | 0.016 | -0.053 | 0.021 | -0.038 | 0.046 | 0.017 | 0.041 | -0.010 | 0.069 | -0.030 | 0.041 |  |
| rs2734849 | 11 | 113270160 | A | G | 0.003 | 0.001 | 1.90E-02 | -0.014 | 0.002 | 8.20E-10 | PC | 0.003 | 0.006 | 0.001 | 0.008 | 0.023 | 0.018 | -0.002 | 0.015 | 0.004 | 0.025 | -0.005 | 0.015 |  |
| rs1448355 | 11 | 131286685 | C | T | 0.004 | 0.001 | 1.40E-02 | -0.015 | 0.002 | 1.30E-10 | PC | -0.001 | 0.006 | -0.001 | 0.008 | 0.011 | 0.018 | -0.002 | 0.015 | 0.007 | 0.026 | -0.006 | 0.016 |  |
| rs35933007 | 12 | 38929341 | G | A | 0.007 | 0.002 | 1.50E-05 | -0.015 | 0.003 | 9.20E-09 | PC | 0.003 | 0.008 | 0.017 | 0.011 | 0.000 | 0.024 | 0.018 | 0.020 | -0.017 | 0.034 | -0.030 | 0.021 |  |
| rs74086911 | 12 | 50015942 | G | A | 0.015 | 0.003 | 2.10E-08 | -0.003 | 0.004 | 4.10E-01 | Smoking | 0.012 | 0.012 | 0.004 | 0.015 | 0.024 | 0.034 | 0.024 | 0.029 | 0.026 | 0.049 | 0.046 | 0.030 |  |
| rs7297175 | 12 | 56473808 | T | C | -0.008 | 0.001 | 6.60E-09 | 0.003 | 0.002 | 1.70E-01 | Smoking | 0.004 | 0.006 | 0.006 | 0.008 | 0.038 | 0.018 | -0.010 | 0.015 | 0.017 | 0.026 | -0.028 | 0.016 |  |
| rs10879871 | 12 | 75380511 | T | G | -0.010 | 0.001 | 5.00E-11 | 0.005 | 0.002 | 2.70E-02 | Smoking | 0.005 | 0.006 | 0.006 | 0.008 | -0.004 | 0.019 | 0.002 | 0.016 | -0.038 | 0.027 | 0.025 | 0.016 |  |
| rs12831617 | 12 | 84758368 | C | T | -0.009 | 0.002 | 1.90E-08 | 0.004 | 0.003 | 9.30E-02 | Smoking | -0.028 | 0.007 | -0.020 | 0.009 | -0.040 | 0.021 | -0.030 | 0.018 | -0.002 | 0.030 | -0.041 | 0.018 |  |
| rs206965 | 12 | 120856332 | T | C | 0.000 | 0.002 | 7.90E-01 | 0.016 | 0.003 | 9.10E-09 | PC | 0.010 | 0.007 | 0.008 | 0.010 | 0.014 | 0.022 | 0.032 | 0.019 | -0.008 | 0.032 | 0.014 | 0.019 |  |
| rs12874776 | 13 | 57794345 | C | A | 0.005 | 0.002 | 2.40E-02 | -0.023 | 0.004 | 8.00E-11 | PC | 0.001 | 0.010 | 0.007 | 0.013 | 0.036 | 0.030 | 0.007 | 0.026 | -0.005 | 0.044 | 0.018 | 0.026 |  |
| rs6562474 | 13 | 67332812 | C | G | 0.008 | 0.001 | 1.00E-08 | -0.005 | 0.002 | 4.00E-02 | Smoking | -0.012 | 0.006 | -0.005 | 0.008 | -0.033 | 0.018 | -0.034 | 0.016 | -0.069 | 0.027 | 0.007 | 0.016 |  |
| rs7333559 | 13 | 100546450 | G | A | 0.011 | 0.002 | 3.20E-10 | 0.002 | 0.003 | 4.60E-01 | Smoking | 0.016 | 0.008 | 0.015 | 0.010 | 0.001 | 0.023 | 0.000 | 0.020 | 0.002 | 0.033 | 0.030 | 0.020 |  |
| rs198262 | 14 | 57283283 | C | T | -0.001 | 0.003 | 8.40E-01 | 0.030 | 0.005 | 3.90E-08 | PC | 0.002 | 0.015 | 0.011 | 0.020 | 0.041 | 0.044 | -0.020 | 0.038 | -0.019 | 0.065 | -0.010 | 0.039 |  |
| rs7155595 | 14 | 77502546 | A | C | -0.009 | 0.001 | 2.50E-09 | 0.006 | 0.002 | 1.50E-02 | Smoking | -0.012 | 0.006 | -0.002 | 0.008 | 0.001 | 0.019 | -0.029 | 0.016 | -0.035 | 0.027 | 0.022 | 0.016 |  |
| rs3742365 | 14 | 104198251 | T | C | -0.011 | 0.001 | 2.50E-14 | -0.004 | 0.002 | 4.70E-02 | Smoking | 0.009 | 0.006 | 0.007 | 0.008 | 0.015 | 0.018 | 0.023 | 0.015 | 0.037 | 0.026 | 0.005 | 0.016 |  |
| rs166835 | 15 | 47716037 | C | T | -0.002 | 0.001 | 2.40E-01 | 0.013 | 0.002 | 3.40E-09 | PC | -0.013 | 0.006 | -0.023 | 0.008 | -0.038 | 0.018 | 0.002 | 0.015 | 0.001 | 0.026 | -0.015 | 0.015 |  |
| rs28485305 | 15 | 74044197 | C | T | 0.008 | 0.001 | 2.60E-08 | 0.003 | 0.002 | 1.50E-01 | Smoking | 0.005 | 0.006 | -0.002 | 0.008 | -0.002 | 0.018 | 0.017 | 0.015 | -0.021 | 0.026 | 0.027 | 0.016 |  |
| rs8042849 | 15 | 78817929 | C | T | 0.019 | 0.001 | 1.80E-39 | -0.002 | 0.002 | 3.70E-01 | Smoking | 0.007 | 0.006 | 0.009 | 0.008 | -0.002 | 0.018 | -0.004 | 0.016 | -0.020 | 0.027 | 0.002 | 0.016 |  |
| rs4702 | 15 | 91426560 | G | A | 0.005 | 0.001 | 1.10E-04 | 0.012 | 0.002 | 3.80E-08 | PC | 0.029 | 0.006 | 0.016 | 0.008 | 0.057 | 0.018 | 0.071 | 0.015 | -0.002 | 0.026 | 0.007 | 0.015 |  |
| rs11861214 | 16 | 746611 | G | T | 0.009 | 0.002 | 2.00E-08 | -0.002 | 0.003 | 3.90E-01 | Smoking | -0.003 | 0.007 | 0.001 | 0.009 | -0.007 | 0.021 | 0.009 | 0.018 | -0.051 | 0.030 | 0.010 | 0.018 |  |
| rs6498759 | 16 | 18020295 | T | C | 0.000 | 0.002 | 7.40E-01 | 0.013 | 0.002 | 2.70E-08 | PC | 0.012 | 0.007 | 0.021 | 0.009 | 0.034 | 0.020 | -0.028 | 0.017 | 0.021 | 0.029 | 0.017 | 0.018 |  |
| rs57611503 | 16 | 31165795 | G | A | 0.008 | 0.001 | 4.00E-08 | 0.001 | 0.002 | 7.00E-01 | Smoking | -0.009 | 0.006 | 0.001 | 0.008 | -0.021 | 0.018 | -0.014 | 0.016 | -0.035 | 0.027 | -0.013 | 0.016 |  |
| rs3730399 | 16 | 67229019 | A | G | -0.002 | 0.003 | 4.90E-01 | 0.025 | 0.004 | 3.30E-08 | PC | -0.015 | 0.012 | 0.005 | 0.016 | -0.038 | 0.035 | 0.021 | 0.030 | -0.032 | 0.051 | -0.015 | 0.030 |  |
| rs889398 | 16 | 69556715 | C | T | 0.009 | 0.001 | 6.30E-11 | 0.002 | 0.002 | 4.90E-01 | Smoking | 0.015 | 0.006 | 0.010 | 0.008 | 0.028 | 0.018 | -0.006 | 0.015 | 0.017 | 0.026 | 0.031 | 0.015 |  |
| rs60952428 | 16 | 75640521 | T | C | 0.013 | 0.002 | 3.00E-08 | 0.002 | 0.004 | 5.70E-01 | Smoking | 0.001 | 0.011 | -0.002 | 0.014 | -0.024 | 0.031 | 0.033 | 0.027 | 0.026 | 0.046 | 0.008 | 0.027 |  |
| rs1050847 | 16 | 87443734 | C | T | 0.008 | 0.001 | 1.40E-08 | 0.000 | 0.002 | 8.50E-01 | Smoking | -0.007 | 0.006 | -0.005 | 0.008 | 0.017 | 0.018 | -0.011 | 0.016 | 0.012 | 0.026 | -0.025 | 0.016 |  |
| rs369230 | 16 | 89645437 | G | T | -0.009 | 0.002 | 1.80E-09 | -0.001 | 0.002 | 6.30E-01 | Smoking | -0.021 | 0.007 | -0.018 | 0.009 | -0.040 | 0.020 | -0.017 | 0.017 | -0.034 | 0.029 | -0.028 | 0.017 |  |
| rs7209653 | 17 | 19882084 | T | C | 0.000 | 0.002 | 8.40E-01 | 0.016 | 0.002 | 9.10E-11 | PC | -0.007 | 0.007 | 0.000 | 0.009 | -0.035 | 0.020 | -0.012 | 0.017 | 0.012 | 0.029 | -0.023 | 0.017 |  |
| rs8614 | 17 | 27588806 | C | A | -0.011 | 0.002 | 1.80E-10 | -0.004 | 0.003 | 1.80E-01 | Smoking | -0.016 | 0.008 | -0.016 | 0.011 | -0.067 | 0.024 | -0.013 | 0.021 | 0.013 | 0.035 | 0.003 | 0.021 |  |
| rs12603813 | 17 | 43196584 | T | C | -0.001 | 0.002 | 3.90E-01 | 0.014 | 0.003 | 1.30E-08 | PC | 0.005 | 0.007 | 0.017 | 0.009 | 0.036 | 0.020 | -0.003 | 0.017 | 0.033 | 0.030 | -0.032 | 0.017 |  |
| rs9904288 | 17 | 47031973 | T | C | 0.008 | 0.002 | 3.10E-08 | -0.004 | 0.002 | 9.30E-02 | Smoking | 0.005 | 0.007 | 0.004 | 0.009 | -0.006 | 0.019 | 0.009 | 0.017 | 0.029 | 0.029 | 0.006 | 0.017 |  |
| rs66643547 | 17 | 79375315 | C | T | 0.004 | 0.001 | 1.60E-02 | -0.015 | 0.002 | 3.20E-11 | PC | 0.020 | 0.007 | 0.021 | 0.009 | 0.005 | 0.019 | 0.024 | 0.017 | 0.061 | 0.029 | 0.019 | 0.017 |  |
| rs12967855 | 18 | 35138245 | A | G | 0.008 | 0.001 | 3.10E-08 | -0.005 | 0.002 | 4.50E-02 | Smoking | 0.009 | 0.007 | 0.010 | 0.009 | 0.029 | 0.019 | 0.000 | 0.017 | -0.040 | 0.028 | 0.011 | 0.017 |  |
| rs62098013 | 18 | 50863861 | G | A | -0.009 | 0.001 | 4.10E-09 | 0.003 | 0.002 | 2.40E-01 | Smoking | -0.009 | 0.006 | -0.008 | 0.008 | -0.023 | 0.018 | -0.002 | 0.016 | -0.056 | 0.027 | 0.007 | 0.016 |  |
| rs784256 | 18 | 53398626 | G | A | -0.005 | 0.002 | 5.30E-03 | 0.019 | 0.003 | 6.20E-11 | PC | -0.005 | 0.008 | 0.006 | 0.010 | -0.020 | 0.023 | 0.009 | 0.020 | -0.015 | 0.033 | -0.034 | 0.020 |  |
| rs71367545 | 18 | 77576337 | G | A | -0.010 | 0.002 | 1.40E-09 | -0.004 | 0.003 | 1.80E-01 | Smoking | 0.006 | 0.008 | 0.022 | 0.011 | -0.038 | 0.024 | 0.001 | 0.021 | 0.005 | 0.035 | 0.011 | 0.021 |  |
| rs76608582 | 19 | 4474725 | C | A | 0.022 | 0.003 | 3.20E-10 | 0.001 | 0.005 | 8.40E-01 | Smoking | 0.031 | 0.018 | 0.027 | 0.023 | -0.088 | 0.049 | 0.035 | 0.045 | 0.082 | 0.076 | -0.018 | 0.044 |  |
| rs35343344 | 19 | 18471610 | C | A | 0.009 | 0.002 | 8.80E-09 | -0.005 | 0.003 | 6.20E-02 | Smoking | -0.004 | 0.007 | 0.005 | 0.009 | 0.007 | 0.021 | -0.016 | 0.018 | 0.021 | 0.030 | -0.012 | 0.018 |  |
| rs4814873 | 20 | 19616429 | C | T | 0.010 | 0.002 | 2.90E-09 | -0.008 | 0.003 | 2.80E-03 | Smoking | 0.007 | 0.007 | -0.006 | 0.009 | 0.023 | 0.021 | 0.007 | 0.018 | -0.021 | 0.030 | 0.027 | 0.018 |  |
| rs6119897 | 20 | 31145415 | G | A | -0.013 | 0.002 | 3.60E-15 | 0.004 | 0.003 | 1.20E-01 | Smoking | -0.020 | 0.007 | -0.019 | 0.009 | 0.010 | 0.021 | -0.006 | 0.018 | 0.007 | 0.030 | -0.050 | 0.018 |  |
| rs12481282 | 20 | 44761377 | G | C | -0.009 | 0.002 | 7.80E-09 | -0.002 | 0.002 | 3.30E-01 | Smoking | 0.004 | 0.007 | 0.011 | 0.009 | 0.003 | 0.020 | 0.005 | 0.017 | -0.020 | 0.029 | -0.003 | 0.017 |  |
| rs348809 | 20 | 59032097 | A | G | -0.008 | 0.001 | 1.30E-08 | -0.001 | 0.002 | 7.50E-01 | Smoking | -0.022 | 0.007 | -0.026 | 0.009 | -0.016 | 0.019 | 0.000 | 0.017 | -0.034 | 0.028 | -0.030 | 0.017 |  |
| rs6129084 | 20 | 59828857 | A | T | -0.001 | 0.001 | 5.10E-01 | -0.014 | 0.002 | 9.30E-10 | PC | -0.001 | 0.006 | 0.005 | 0.008 | -0.004 | 0.019 | -0.010 | 0.016 | 0.015 | 0.027 | -0.001 | 0.016 |  |
| rs6011779 | 20 | 61984317 | C | T | 0.019 | 0.002 | 2.30E-27 | -0.007 | 0.003 | 1.10E-02 | Smoking | -0.020 | 0.008 | -0.010 | 0.010 | 0.005 | 0.022 | -0.033 | 0.019 | -0.056 | 0.033 | -0.040 | 0.020 |  |
| rs147412694 | 21 | 40702786 | G | A | -0.012 | 0.002 | 2.90E-09 | -0.008 | 0.003 | 1.30E-02 | Smoking | -0.022 | 0.009 | -0.019 | 0.012 | -0.035 | 0.026 | -0.024 | 0.022 | -0.100 | 0.037 | 0.018 | 0.023 |  |
| rs2838834 | 21 | 46665208 | C | T | -0.009 | 0.002 | 6.30E-10 | 0.000 | 0.002 | 9.80E-01 | Smoking | -0.003 | 0.007 | 0.002 | 0.009 | -0.051 | 0.019 | 0.018 | 0.017 | 0.011 | 0.028 | 0.014 | 0.017 |  |
| rs136553 | 22 | 27255675 | C | T | -0.002 | 0.001 | 2.10E-01 | -0.015 | 0.002 | 2.30E-11 | PC | -0.004 | 0.006 | -0.001 | 0.008 | 0.000 | 0.018 | -0.005 | 0.015 | 0.014 | 0.027 | 0.002 | 0.016 |  |
| rs136233 | 22 | 31212410 | A | G | -0.010 | 0.002 | 1.80E-08 | 0.001 | 0.003 | 6.70E-01 | Smoking | 0.002 | 0.008 | -0.007 | 0.011 | 0.016 | 0.024 | 0.018 | 0.020 | 0.037 | 0.035 | 0.003 | 0.021 |  |
| rs7288455 | 22 | 39966547 | A | G | -0.002 | 0.001 | 1.30E-01 | 0.013 | 0.002 | 4.00E-09 | PC | 0.001 | 0.006 | 0.005 | 0.008 | -0.018 | 0.019 | 0.004 | 0.016 | -0.025 | 0.027 | -0.007 | 0.016 |  |
| rs202645 | 22 | 41798520 | A | G | -0.010 | 0.002 | 3.90E-09 | 0.005 | 0.003 | 7.60E-02 | Smoking | -0.002 | 0.007 | 0.014 | 0.010 | 0.031 | 0.022 | -0.039 | 0.019 | -0.006 | 0.032 | -0.030 | 0.019 |  |

| **Table S23. Beta estimates for the SNPs included in the multivariable MR of smoking, leisure computer use, and colorectal cancer risk** | | | | | | | | | | | | | | | | | | | | | | |
| --- | --- | --- | --- | --- | --- | --- | --- | --- | --- | --- | --- | --- | --- | --- | --- | --- | --- | --- | --- | --- | --- | --- |
| SNP | CHR | POS | EA | OA | beta_smk | se_smk | pval_smk | beta_pc | se_pc | pval_pc | Phenotype | beta_crc | se_crc | beta_crc_men | se_crc_men | beta_crc_women | se_crc_women | beta_co | se_co | beta_re | se_re |  |
| rs2748985 | 1 | 1853184 | T | C | -0.001 | 0.001 | 4.40E-01 | -0.015 | 0.002 | 4.10E-12 | PC | 0.006 | 0.010 | 0.017 | 0.014 | -0.003 | 0.014 | 0.016 | 0.012 | -0.003 | 0.015 |  |
| rs1193237 | 1 | 7526486 | G | C | -0.008 | 0.001 | 2.80E-08 | -0.001 | 0.002 | 6.90E-01 | Smoking | 0.004 | 0.010 | -0.004 | 0.014 | 0.009 | 0.014 | 0.007 | 0.012 | 0.015 | 0.015 |  |
| rs4949465 | 1 | 32178489 | T | C | -0.012 | 0.002 | 1.70E-08 | 0.007 | 0.003 | 4.40E-02 | Smoking | 0.015 | 0.014 | 0.044 | 0.020 | -0.011 | 0.020 | 0.021 | 0.017 | 0.032 | 0.022 |  |
| rs549845 | 1 | 44076469 | G | A | 0.011 | 0.002 | 8.30E-14 | 0.002 | 0.002 | 3.60E-01 | Smoking | 0.010 | 0.010 | 0.014 | 0.015 | 0.003 | 0.015 | 0.005 | 0.012 | 0.016 | 0.016 |  |
| rs1933270 | 1 | 49977965 | T | G | 0.009 | 0.001 | 1.50E-10 | -0.005 | 0.002 | 3.50E-02 | Smoking | 0.008 | 0.010 | 0.016 | 0.014 | -0.001 | 0.014 | 0.007 | 0.012 | 0.010 | 0.016 |  |
| rs7528604 | 1 | 66407352 | G | A | 0.010 | 0.001 | 5.70E-12 | -0.001 | 0.002 | 5.20E-01 | Smoking | -0.018 | 0.010 | -0.014 | 0.014 | -0.023 | 0.014 | -0.009 | 0.012 | -0.020 | 0.015 |  |
| rs11210229 | 1 | 73860028 | A | G | 0.012 | 0.001 | 2.00E-16 | -0.002 | 0.002 | 4.70E-01 | Smoking | -0.008 | 0.010 | -0.002 | 0.014 | -0.011 | 0.014 | -0.014 | 0.012 | 0.022 | 0.015 |  |
| rs7553348 | 1 | 75005067 | G | A | 0.010 | 0.001 | 5.20E-12 | -0.004 | 0.002 | 6.20E-02 | Smoking | 0.001 | 0.010 | 0.012 | 0.014 | -0.011 | 0.014 | 0.011 | 0.012 | 0.006 | 0.015 |  |
| rs10922907 | 1 | 91193049 | A | T | 0.010 | 0.001 | 3.00E-13 | -0.003 | 0.002 | 2.20E-01 | Smoking | 0.004 | 0.010 | 0.020 | 0.014 | -0.015 | 0.014 | 0.004 | 0.012 | -0.022 | 0.015 |  |
| rs1931263 | 1 | 96175101 | G | T | -0.008 | 0.001 | 4.00E-08 | 0.007 | 0.002 | 1.90E-03 | Smoking | 0.018 | 0.010 | 0.005 | 0.014 | 0.028 | 0.014 | 0.025 | 0.012 | 0.008 | 0.015 |  |
| rs7519626 | 1 | 99514554 | C | T | 0.008 | 0.001 | 1.20E-08 | -0.001 | 0.002 | 5.60E-01 | Smoking | 0.003 | 0.010 | -0.006 | 0.015 | 0.009 | 0.015 | 0.007 | 0.012 | 0.010 | 0.016 |  |
| rs9435340 | 1 | 107593201 | T | A | 0.008 | 0.001 | 1.20E-08 | 0.000 | 0.002 | 9.20E-01 | Smoking | 0.025 | 0.010 | 0.037 | 0.014 | 0.014 | 0.015 | 0.011 | 0.012 | 0.030 | 0.016 |  |
| rs12145677 | 1 | 110023610 | G | A | 0.002 | 0.002 | 2.20E-01 | -0.017 | 0.002 | 1.20E-12 | PC | -0.006 | 0.011 | 0.012 | 0.015 | -0.023 | 0.015 | -0.007 | 0.013 | -0.004 | 0.017 |  |
| rs10918701 | 1 | 162090536 | G | A | 0.008 | 0.001 | 2.10E-08 | -0.007 | 0.002 | 2.40E-03 | Smoking | 0.008 | 0.010 | -0.004 | 0.014 | 0.023 | 0.014 | 0.014 | 0.012 | 0.023 | 0.016 |  |
| rs7526456 | 1 | 197773734 | T | C | -0.001 | 0.001 | 3.00E-01 | -0.013 | 0.002 | 1.20E-08 | PC | -0.005 | 0.010 | -0.001 | 0.014 | -0.009 | 0.014 | -0.004 | 0.012 | -0.012 | 0.015 |  |
| rs2867112 | 2 | 651349 | T | G | 0.015 | 0.002 | 4.80E-15 | 0.009 | 0.003 | 1.80E-03 | Smoking | 0.012 | 0.013 | 0.029 | 0.018 | -0.002 | 0.018 | 0.018 | 0.016 | 0.019 | 0.020 |  |
| rs6741228 | 2 | 22548774 | T | C | 0.008 | 0.001 | 1.60E-08 | -0.002 | 0.002 | 3.70E-01 | Smoking | 0.002 | 0.010 | 0.008 | 0.014 | -0.006 | 0.014 | 0.003 | 0.012 | 0.005 | 0.015 |  |
| rs62135536 | 2 | 44326028 | C | T | 0.024 | 0.004 | 8.00E-10 | 0.005 | 0.006 | 4.10E-01 | Smoking | 0.011 | 0.031 | -0.027 | 0.044 | 0.059 | 0.045 | 0.012 | 0.037 | -0.043 | 0.048 |  |
| rs7569203 | 2 | 45154418 | A | C | -0.011 | 0.002 | 7.40E-13 | 0.000 | 0.002 | 8.80E-01 | Smoking | 0.015 | 0.010 | 0.011 | 0.014 | 0.017 | 0.014 | 0.007 | 0.012 | 0.031 | 0.016 |  |
| rs13016665 | 2 | 57995348 | C | A | -0.008 | 0.001 | 1.80E-09 | 0.010 | 0.002 | 2.20E-05 | Smoking | -0.010 | 0.010 | -0.033 | 0.014 | 0.014 | 0.014 | -0.012 | 0.012 | -0.023 | 0.015 |  |
| rs4671357 | 2 | 60136176 | T | C | -0.009 | 0.001 | 1.10E-11 | -0.001 | 0.002 | 5.10E-01 | Smoking | -0.011 | 0.010 | -0.003 | 0.014 | -0.017 | 0.014 | -0.010 | 0.012 | -0.010 | 0.015 |  |
| rs359243 | 2 | 60475509 | T | C | -0.009 | 0.001 | 9.50E-10 | -0.010 | 0.002 | 2.50E-05 | Smoking | 0.002 | 0.010 | -0.005 | 0.014 | 0.007 | 0.014 | -0.005 | 0.012 | 0.014 | 0.015 |  |
| rs2678670 | 2 | 104469564 | A | T | 0.009 | 0.001 | 3.10E-10 | -0.002 | 0.002 | 4.50E-01 | Smoking | 0.004 | 0.010 | 0.012 | 0.014 | -0.005 | 0.014 | 0.011 | 0.012 | 0.005 | 0.015 |  |
| rs62155874 | 2 | 105973094 | A | G | -0.017 | 0.002 | 5.20E-16 | 0.002 | 0.003 | 5.80E-01 | Smoking | -0.014 | 0.015 | -0.019 | 0.021 | -0.004 | 0.021 | -0.004 | 0.018 | -0.005 | 0.023 |  |
| rs3811038 | 2 | 113240183 | T | C | -0.010 | 0.002 | 8.90E-10 | -0.004 | 0.002 | 1.40E-01 | Smoking | -0.013 | 0.011 | -0.009 | 0.015 | -0.018 | 0.015 | -0.005 | 0.013 | -0.049 | 0.017 |  |
| rs10754920 | 2 | 146456888 | C | G | 0.003 | 0.002 | 2.10E-01 | 0.020 | 0.003 | 6.70E-09 | PC | 0.002 | 0.014 | -0.012 | 0.020 | 0.011 | 0.020 | 0.007 | 0.018 | 0.011 | 0.023 |  |
| rs6744254 | 2 | 155986195 | C | T | -0.006 | 0.001 | 9.40E-06 | -0.016 | 0.002 | 5.40E-13 | PC | 0.014 | 0.010 | 0.041 | 0.014 | -0.012 | 0.014 | 0.020 | 0.012 | 0.021 | 0.015 |  |
| rs62175972 | 2 | 161362830 | T | C | 0.022 | 0.004 | 1.70E-08 | -0.005 | 0.006 | 4.10E-01 | Smoking | 0.093 | 0.026 | 0.034 | 0.036 | 0.144 | 0.037 | 0.077 | 0.030 | 0.105 | 0.040 |  |
| rs3769949 | 2 | 166199284 | T | A | -0.008 | 0.001 | 2.50E-09 | 0.005 | 0.002 | 2.70E-02 | Smoking | 0.000 | 0.010 | 0.000 | 0.014 | -0.001 | 0.014 | -0.003 | 0.012 | -0.002 | 0.015 |  |
| rs13009008 | 2 | 174043233 | A | G | 0.009 | 0.001 | 4.60E-09 | 0.000 | 0.002 | 8.70E-01 | Smoking | 0.012 | 0.010 | 0.026 | 0.014 | 0.005 | 0.014 | 0.015 | 0.012 | 0.013 | 0.016 |  |
| rs4473348 | 2 | 182073742 | A | T | -0.010 | 0.002 | 6.40E-11 | 0.003 | 0.003 | 3.10E-01 | Smoking | -0.021 | 0.011 | -0.003 | 0.016 | -0.038 | 0.016 | -0.038 | 0.014 | 0.009 | 0.018 |  |
| rs12623702 | 2 | 202885506 | A | G | -0.010 | 0.001 | 7.70E-12 | 0.007 | 0.002 | 3.60E-03 | Smoking | 0.027 | 0.010 | 0.021 | 0.014 | 0.032 | 0.014 | 0.031 | 0.012 | 0.016 | 0.016 |  |
| rs11274218 | 2 | 215035001 | C | T | -0.004 | 0.001 | 7.80E-03 | -0.014 | 0.002 | 2.50E-09 | PC | -0.007 | 0.010 | 0.005 | 0.014 | -0.016 | 0.014 | -0.009 | 0.012 | 0.001 | 0.015 |  |
| rs10208088 | 2 | 221055873 | C | T | 0.001 | 0.001 | 6.90E-01 | 0.013 | 0.002 | 4.40E-09 | PC | 0.002 | 0.010 | 0.007 | 0.014 | -0.006 | 0.014 | 0.003 | 0.012 | -0.019 | 0.015 |  |
| rs6779302 | 3 | 16859710 | G | T | -0.009 | 0.001 | 1.20E-09 | -0.004 | 0.002 | 7.60E-02 | Smoking | -0.002 | 0.010 | -0.002 | 0.014 | 0.003 | 0.014 | -0.006 | 0.012 | -0.028 | 0.016 |  |
| rs11708955 | 3 | 49540114 | T | C | 0.007 | 0.002 | 1.20E-05 | -0.016 | 0.002 | 4.20E-11 | PC | -0.001 | 0.010 | 0.007 | 0.015 | -0.006 | 0.015 | -0.007 | 0.012 | 0.029 | 0.016 |  |
| rs6774533 | 3 | 62471086 | C | T | 0.001 | 0.002 | 4.50E-01 | -0.015 | 0.002 | 1.20E-09 | PC | -0.004 | 0.011 | -0.008 | 0.015 | -0.001 | 0.015 | -0.009 | 0.013 | 0.009 | 0.017 |  |
| rs775758 | 3 | 77582005 | A | T | 0.008 | 0.001 | 1.10E-08 | 0.003 | 0.002 | 1.80E-01 | Smoking | -0.010 | 0.010 | -0.014 | 0.014 | -0.006 | 0.014 | -0.011 | 0.012 | 0.003 | 0.015 |  |
| rs421983 | 3 | 84892866 | T | C | 0.009 | 0.001 | 3.30E-10 | 0.004 | 0.002 | 6.90E-02 | Smoking | -0.010 | 0.010 | -0.017 | 0.014 | -0.005 | 0.014 | -0.023 | 0.012 | -0.016 | 0.015 |  |
| rs78082503 | 3 | 116556913 | G | C | 0.004 | 0.002 | 1.30E-02 | -0.015 | 0.003 | 1.10E-08 | PC | -0.007 | 0.011 | -0.020 | 0.016 | 0.004 | 0.016 | -0.002 | 0.014 | 0.033 | 0.018 |  |
| rs73220544 | 3 | 131074511 | A | C | -0.011 | 0.002 | 1.50E-08 | 0.001 | 0.003 | 6.90E-01 | Smoking | -0.010 | 0.013 | -0.028 | 0.019 | 0.008 | 0.019 | -0.006 | 0.016 | -0.017 | 0.021 |  |
| rs9842947 | 3 | 157412246 | C | T | -0.009 | 0.001 | 3.10E-09 | -0.001 | 0.002 | 5.40E-01 | Smoking | 0.003 | 0.011 | -0.014 | 0.015 | 0.020 | 0.015 | 0.001 | 0.013 | 0.002 | 0.017 |  |
| rs624833 | 4 | 2881256 | T | G | 0.009 | 0.002 | 6.60E-10 | -0.007 | 0.002 | 5.70E-03 | Smoking | 0.001 | 0.011 | 0.014 | 0.015 | -0.015 | 0.015 | -0.002 | 0.012 | 0.019 | 0.016 |  |
| rs61796681 | 4 | 23678196 | A | T | -0.013 | 0.002 | 4.20E-08 | 0.009 | 0.004 | 1.70E-02 | Smoking | -0.013 | 0.018 | -0.016 | 0.026 | -0.011 | 0.026 | -0.016 | 0.022 | 0.011 | 0.028 |  |
| rs317021 | 4 | 35418368 | T | A | -0.012 | 0.002 | 1.10E-10 | 0.006 | 0.003 | 2.90E-02 | Smoking | -0.004 | 0.013 | 0.014 | 0.019 | -0.022 | 0.019 | -0.008 | 0.016 | 0.000 | 0.021 |  |
| rs6857629 | 4 | 39684977 | G | A | -0.005 | 0.002 | 1.70E-03 | 0.015 | 0.003 | 1.90E-09 | PC | 0.021 | 0.011 | 0.034 | 0.016 | 0.012 | 0.016 | 0.014 | 0.013 | 0.026 | 0.017 |  |
| rs72678864 | 4 | 112422145 | G | A | 0.012 | 0.002 | 1.60E-11 | -0.004 | 0.003 | 1.50E-01 | Smoking | 0.003 | 0.014 | -0.008 | 0.019 | 0.013 | 0.019 | 0.017 | 0.016 | -0.012 | 0.021 |  |
| rs17576594 | 4 | 147952241 | G | A | 0.011 | 0.002 | 1.70E-12 | -0.002 | 0.002 | 5.10E-01 | Smoking | 0.010 | 0.011 | 0.011 | 0.015 | 0.012 | 0.015 | 0.009 | 0.013 | 0.012 | 0.017 |  |
| rs2068625 | 4 | 159856739 | T | C | 0.002 | 0.002 | 2.90E-01 | -0.016 | 0.002 | 4.00E-11 | PC | 0.017 | 0.011 | 0.011 | 0.015 | 0.020 | 0.015 | 0.025 | 0.013 | 0.020 | 0.017 |  |
| rs2220599 | 5 | 7378854 | C | G | 0.001 | 0.001 | 4.50E-01 | -0.016 | 0.002 | 2.40E-12 | PC | -0.003 | 0.010 | -0.010 | 0.014 | 0.000 | 0.014 | 0.001 | 0.012 | -0.002 | 0.016 |  |
| rs71627581 | 5 | 43161351 | G | A | 0.013 | 0.002 | 1.60E-09 | -0.005 | 0.003 | 1.90E-01 | Smoking | 0.029 | 0.017 | 0.042 | 0.023 | 0.021 | 0.024 | 0.048 | 0.020 | 0.041 | 0.025 |  |
| rs2080870 | 5 | 60388313 | A | T | 0.009 | 0.002 | 4.90E-08 | -0.010 | 0.003 | 4.20E-05 | Smoking | 0.001 | 0.011 | -0.003 | 0.016 | 0.004 | 0.016 | 0.004 | 0.013 | -0.009 | 0.018 |  |
| rs11749912 | 5 | 88065628 | A | G | -0.004 | 0.001 | 6.00E-03 | 0.014 | 0.002 | 4.90E-10 | PC | 0.015 | 0.010 | 0.010 | 0.014 | 0.023 | 0.014 | 0.019 | 0.012 | -0.001 | 0.015 |  |
| rs4957528 | 5 | 106420589 | A | C | -0.010 | 0.002 | 4.20E-09 | 0.002 | 0.003 | 5.00E-01 | Smoking | -0.002 | 0.012 | -0.016 | 0.017 | 0.012 | 0.017 | -0.007 | 0.015 | 0.027 | 0.019 |  |
| rs1469249 | 5 | 113837198 | G | A | -0.001 | 0.002 | 4.00E-01 | 0.016 | 0.003 | 9.50E-09 | PC | 0.009 | 0.012 | 0.017 | 0.017 | 0.005 | 0.017 | -0.001 | 0.014 | 0.038 | 0.018 |  |
| rs162894 | 5 | 131611872 | T | G | -0.001 | 0.001 | 3.60E-01 | 0.013 | 0.002 | 1.10E-08 | PC | -0.012 | 0.010 | -0.017 | 0.014 | -0.007 | 0.014 | -0.018 | 0.012 | -0.012 | 0.016 |  |
| rs329120 | 5 | 133861756 | C | T | 0.010 | 0.001 | 6.30E-12 | -0.005 | 0.002 | 1.70E-02 | Smoking | 0.005 | 0.010 | 0.003 | 0.014 | 0.006 | 0.014 | 0.008 | 0.012 | 0.024 | 0.015 |  |
| rs56158102 | 5 | 144027803 | C | T | 0.002 | 0.001 | 1.80E-01 | 0.013 | 0.002 | 3.10E-08 | PC | -0.006 | 0.010 | 0.003 | 0.014 | -0.014 | 0.014 | -0.001 | 0.012 | -0.001 | 0.016 |  |
| rs986391 | 5 | 166993972 | G | A | 0.011 | 0.001 | 9.40E-15 | -0.006 | 0.002 | 1.60E-02 | Smoking | 0.010 | 0.010 | 0.017 | 0.014 | 0.003 | 0.014 | 0.018 | 0.012 | 0.028 | 0.016 |  |
| rs13153393 | 5 | 167604213 | A | G | -0.014 | 0.002 | 2.50E-10 | -0.002 | 0.003 | 6.60E-01 | Smoking | -0.006 | 0.015 | -0.030 | 0.022 | 0.015 | 0.022 | 0.008 | 0.018 | 0.025 | 0.024 |  |
| rs245774 | 5 | 170530930 | A | G | -0.009 | 0.002 | 7.40E-09 | -0.002 | 0.002 | 5.00E-01 | Smoking | 0.003 | 0.011 | -0.004 | 0.015 | 0.008 | 0.016 | 0.003 | 0.013 | -0.008 | 0.017 |  |
| rs9477970 | 6 | 19082663 | T | A | -0.002 | 0.002 | 3.30E-01 | -0.016 | 0.003 | 1.00E-08 | PC | -0.017 | 0.012 | -0.032 | 0.018 | -0.004 | 0.018 | -0.016 | 0.015 | -0.005 | 0.020 |  |
| rs6935954 | 6 | 26255451 | A | G | 0.010 | 0.001 | 8.20E-12 | -0.008 | 0.002 | 4.40E-04 | Smoking | 0.006 | 0.010 | 0.016 | 0.014 | -0.007 | 0.014 | 0.014 | 0.012 | -0.005 | 0.016 |  |
| rs2254710 | 6 | 37477000 | C | A | 0.009 | 0.002 | 3.50E-08 | 0.002 | 0.003 | 5.20E-01 | Smoking | -0.013 | 0.011 | -0.006 | 0.016 | -0.022 | 0.016 | -0.011 | 0.014 | -0.023 | 0.018 |  |
| rs2894808 | 6 | 52861990 | T | A | -0.015 | 0.003 | 3.50E-09 | 0.001 | 0.004 | 7.60E-01 | Smoking | 0.003 | 0.017 | 0.000 | 0.024 | 0.006 | 0.024 | 0.009 | 0.021 | -0.025 | 0.027 |  |
| rs12202536 | 6 | 67475273 | A | G | -0.008 | 0.001 | 2.80E-09 | 0.002 | 0.002 | 2.90E-01 | Smoking | 0.008 | 0.010 | -0.005 | 0.014 | 0.021 | 0.014 | 0.000 | 0.012 | 0.024 | 0.015 |  |
| rs9372625 | 6 | 98344031 | G | A | 0.002 | 0.001 | 1.00E-01 | -0.018 | 0.002 | 5.70E-16 | PC | 0.031 | 0.010 | 0.033 | 0.014 | 0.030 | 0.014 | 0.026 | 0.012 | 0.041 | 0.016 |  |
| rs7766610 | 6 | 111707821 | C | A | 0.013 | 0.002 | 2.20E-12 | 0.000 | 0.003 | 9.20E-01 | Smoking | 0.016 | 0.012 | 0.038 | 0.017 | -0.006 | 0.017 | 0.020 | 0.015 | 0.007 | 0.019 |  |
| rs6935828 | 6 | 140811367 | C | T | 0.000 | 0.001 | 9.90E-01 | -0.012 | 0.002 | 4.30E-08 | PC | -0.002 | 0.010 | 0.005 | 0.014 | -0.004 | 0.014 | 0.002 | 0.012 | 0.022 | 0.015 |  |
| rs1922018 | 7 | 3560401 | C | T | 0.010 | 0.001 | 3.00E-12 | 0.000 | 0.002 | 8.80E-01 | Smoking | -0.014 | 0.010 | -0.013 | 0.014 | -0.015 | 0.014 | -0.011 | 0.012 | -0.007 | 0.016 |  |
| rs10226228 | 7 | 32315613 | A | G | -0.011 | 0.001 | 2.00E-15 | 0.002 | 0.002 | 4.40E-01 | Smoking | 0.003 | 0.010 | 0.004 | 0.014 | 0.001 | 0.014 | 0.008 | 0.012 | 0.014 | 0.016 |  |
| rs55772938 | 7 | 70025113 | A | G | -0.002 | 0.002 | 1.40E-01 | -0.015 | 0.002 | 3.90E-10 | PC | -0.008 | 0.011 | 0.001 | 0.015 | -0.017 | 0.015 | -0.001 | 0.013 | -0.006 | 0.016 |  |
| rs11768481 | 7 | 96629103 | C | A | 0.009 | 0.001 | 9.90E-10 | 0.001 | 0.002 | 6.80E-01 | Smoking | 0.000 | 0.011 | 0.023 | 0.015 | -0.018 | 0.015 | 0.012 | 0.013 | -0.001 | 0.017 |  |
| rs6962772 | 7 | 99081730 | A | G | 0.011 | 0.002 | 7.80E-09 | 0.012 | 0.003 | 5.30E-05 | Smoking | 0.011 | 0.013 | -0.007 | 0.018 | 0.034 | 0.018 | 0.011 | 0.016 | -0.005 | 0.021 |  |
| rs10282292 | 7 | 111092478 | C | T | 0.009 | 0.001 | 5.90E-10 | 0.006 | 0.002 | 4.80E-03 | Smoking | 0.008 | 0.010 | 0.017 | 0.014 | 0.001 | 0.014 | 0.013 | 0.012 | -0.005 | 0.016 |  |
| rs2401924 | 7 | 115057862 | G | C | 0.011 | 0.001 | 2.70E-14 | 0.008 | 0.002 | 6.60E-04 | Smoking | -0.004 | 0.010 | -0.020 | 0.014 | 0.014 | 0.014 | 0.001 | 0.012 | -0.017 | 0.015 |  |
| rs7807019 | 7 | 117543063 | A | G | -0.010 | 0.001 | 6.70E-14 | -0.001 | 0.002 | 6.40E-01 | Smoking | 0.001 | 0.010 | -0.003 | 0.014 | 0.003 | 0.014 | -0.005 | 0.012 | 0.004 | 0.015 |  |
| rs12706626 | 7 | 124531370 | G | A | -0.001 | 0.001 | 3.20E-01 | -0.013 | 0.002 | 1.40E-08 | PC | -0.007 | 0.010 | -0.029 | 0.014 | 0.016 | 0.014 | -0.007 | 0.012 | 0.003 | 0.016 |  |
| rs3944151 | 7 | 126970005 | A | G | -0.002 | 0.002 | 2.00E-01 | 0.014 | 0.002 | 3.10E-08 | PC | 0.021 | 0.011 | 0.020 | 0.015 | 0.019 | 0.015 | 0.029 | 0.013 | 0.016 | 0.017 |  |
| rs2345941 | 7 | 133337635 | A | G | -0.005 | 0.001 | 4.00E-04 | 0.015 | 0.002 | 4.00E-11 | PC | -0.023 | 0.010 | -0.013 | 0.014 | -0.033 | 0.014 | -0.025 | 0.012 | -0.012 | 0.015 |  |
| rs35169606 | 8 | 9604066 | T | G | 0.009 | 0.001 | 1.20E-09 | 0.002 | 0.002 | 4.80E-01 | Smoking | -0.003 | 0.011 | -0.018 | 0.015 | 0.017 | 0.015 | -0.024 | 0.013 | 0.024 | 0.017 |  |
| rs11783093 | 8 | 27425349 | C | T | 0.016 | 0.002 | 1.20E-16 | -0.001 | 0.003 | 6.30E-01 | Smoking | 0.012 | 0.013 | 0.002 | 0.019 | 0.020 | 0.019 | 0.017 | 0.016 | 0.009 | 0.021 |  |
| rs2062882 | 8 | 91839576 | G | A | -0.008 | 0.001 | 1.10E-08 | 0.008 | 0.002 | 8.60E-04 | Smoking | -0.019 | 0.010 | -0.009 | 0.014 | -0.030 | 0.014 | -0.024 | 0.012 | -0.036 | 0.015 |  |
| rs72674867 | 8 | 95578201 | A | T | 0.009 | 0.002 | 3.80E-08 | 0.001 | 0.003 | 6.00E-01 | Smoking | 0.013 | 0.011 | 0.016 | 0.015 | 0.006 | 0.016 | 0.007 | 0.013 | 0.004 | 0.017 |  |
| rs13262595 | 8 | 143316970 | A | G | 0.000 | 0.001 | 8.00E-01 | -0.016 | 0.002 | 1.20E-12 | PC | -0.001 | 0.010 | 0.004 | 0.014 | -0.008 | 0.014 | -0.007 | 0.012 | 0.012 | 0.015 |  |
| rs4543592 | 9 | 3014254 | T | C | -0.009 | 0.001 | 4.50E-10 | -0.001 | 0.002 | 8.00E-01 | Smoking | -0.007 | 0.010 | -0.019 | 0.014 | 0.004 | 0.014 | -0.011 | 0.012 | -0.006 | 0.015 |  |
| rs4977839 | 9 | 23355310 | G | A | 0.004 | 0.001 | 1.10E-02 | -0.020 | 0.002 | 4.70E-19 | PC | -0.004 | 0.010 | -0.002 | 0.014 | -0.005 | 0.014 | 0.010 | 0.012 | -0.006 | 0.016 |  |
| rs7039819 | 9 | 82430418 | G | A | 0.009 | 0.001 | 5.10E-10 | -0.001 | 0.002 | 5.20E-01 | Smoking | 0.008 | 0.010 | 0.009 | 0.014 | 0.003 | 0.014 | 0.005 | 0.012 | 0.011 | 0.015 |  |
| rs1246265 | 9 | 86761745 | T | C | -0.009 | 0.002 | 4.20E-09 | 0.002 | 0.002 | 4.40E-01 | Smoking | -0.005 | 0.010 | 0.001 | 0.015 | -0.011 | 0.015 | -0.011 | 0.013 | 0.000 | 0.016 |  |
| rs113851275 | 9 | 98297220 | G | A | -0.003 | 0.002 | 2.60E-01 | -0.021 | 0.004 | 3.20E-09 | PC | 0.022 | 0.015 | 0.001 | 0.022 | 0.043 | 0.022 | 0.010 | 0.019 | -0.001 | 0.024 |  |
| rs7020477 | 9 | 116827760 | A | G | 0.002 | 0.002 | 1.10E-01 | 0.014 | 0.002 | 1.20E-08 | PC | 0.016 | 0.012 | -0.004 | 0.017 | 0.033 | 0.017 | 0.020 | 0.014 | 0.011 | 0.018 |  |
| rs73578186 | 9 | 126334485 | C | T | 0.000 | 0.001 | 7.60E-01 | 0.015 | 0.002 | 2.30E-10 | PC | -0.015 | 0.010 | -0.022 | 0.014 | -0.006 | 0.015 | -0.018 | 0.012 | -0.003 | 0.016 |  |
| rs113382419 | 9 | 136463019 | C | A | -0.028 | 0.002 | 3.00E-37 | 0.000 | 0.003 | 9.00E-01 | Smoking | 0.023 | 0.017 | 0.005 | 0.024 | 0.049 | 0.024 | 0.006 | 0.020 | 0.069 | 0.026 |  |
| rs11255908 | 10 | 8802912 | T | G | -0.010 | 0.002 | 2.30E-10 | 0.003 | 0.003 | 1.80E-01 | Smoking | 0.015 | 0.011 | 0.033 | 0.016 | -0.006 | 0.016 | 0.008 | 0.013 | 0.018 | 0.017 |  |
| rs2675638 | 10 | 63576286 | G | A | 0.008 | 0.001 | 1.30E-09 | -0.001 | 0.002 | 5.40E-01 | Smoking | -0.001 | 0.010 | 0.008 | 0.014 | -0.011 | 0.014 | 0.002 | 0.012 | 0.016 | 0.015 |  |
| rs10823968 | 10 | 74738269 | A | T | 0.008 | 0.001 | 2.10E-08 | -0.005 | 0.002 | 5.00E-02 | Smoking | -0.009 | 0.010 | -0.015 | 0.014 | 0.001 | 0.014 | -0.014 | 0.012 | 0.016 | 0.016 |  |
| rs17553262 | 10 | 92912773 | A | C | -0.013 | 0.002 | 5.30E-09 | -0.001 | 0.003 | 7.80E-01 | Smoking | -0.015 | 0.016 | -0.005 | 0.023 | -0.028 | 0.022 | -0.013 | 0.019 | -0.032 | 0.025 |  |
| rs7077678 | 10 | 104438565 | C | T | 0.009 | 0.001 | 2.60E-09 | 0.005 | 0.002 | 4.10E-02 | Smoking | -0.019 | 0.010 | -0.016 | 0.014 | -0.022 | 0.014 | -0.028 | 0.012 | 0.010 | 0.016 |  |
| rs12244388 | 10 | 104640052 | G | A | -0.013 | 0.001 | 1.40E-19 | 0.000 | 0.002 | 8.90E-01 | Smoking | -0.007 | 0.010 | -0.001 | 0.014 | -0.011 | 0.014 | -0.017 | 0.012 | 0.015 | 0.016 |  |
| rs3896224 | 10 | 106467853 | A | G | 0.010 | 0.001 | 1.10E-11 | 0.006 | 0.002 | 1.10E-02 | Smoking | -0.002 | 0.010 | -0.011 | 0.014 | 0.004 | 0.014 | 0.002 | 0.012 | -0.017 | 0.015 |  |
| rs34866095 | 11 | 16377356 | A | G | -0.009 | 0.002 | 1.20E-08 | -0.002 | 0.002 | 4.40E-01 | Smoking | -0.002 | 0.011 | -0.013 | 0.015 | 0.008 | 0.015 | 0.008 | 0.013 | -0.005 | 0.016 |  |
| rs75742406 | 11 | 17070365 | G | A | 0.010 | 0.002 | 1.30E-09 | -0.003 | 0.003 | 2.70E-01 | Smoking | 0.002 | 0.011 | -0.011 | 0.016 | 0.012 | 0.016 | 0.004 | 0.014 | -0.006 | 0.018 |  |
| rs17309874 | 11 | 27667236 | G | A | -0.011 | 0.002 | 9.70E-13 | -0.006 | 0.003 | 1.20E-02 | Smoking | 0.018 | 0.012 | 0.016 | 0.017 | 0.016 | 0.017 | 0.015 | 0.014 | 0.030 | 0.018 |  |
| rs4391802 | 11 | 28674592 | A | G | 0.010 | 0.002 | 1.40E-11 | -0.004 | 0.002 | 1.30E-01 | Smoking | 0.025 | 0.011 | 0.013 | 0.015 | 0.038 | 0.015 | 0.016 | 0.013 | 0.047 | 0.017 |  |
| rs112282219 | 11 | 46632809 | G | A | -0.023 | 0.004 | 3.80E-11 | 0.004 | 0.006 | 4.50E-01 | Smoking | 0.003 | 0.026 | 0.024 | 0.038 | -0.017 | 0.037 | -0.017 | 0.032 | -0.001 | 0.041 |  |
| rs2734849 | 11 | 113270160 | A | G | 0.003 | 0.001 | 1.90E-02 | -0.014 | 0.002 | 8.20E-10 | PC | -0.003 | 0.010 | 0.004 | 0.014 | -0.010 | 0.014 | -0.009 | 0.012 | 0.012 | 0.015 |  |
| rs1448355 | 11 | 131286685 | C | T | 0.004 | 0.001 | 1.40E-02 | -0.015 | 0.002 | 1.30E-10 | PC | 0.012 | 0.010 | -0.008 | 0.014 | 0.030 | 0.014 | 0.004 | 0.012 | 0.013 | 0.016 |  |
| rs35933007 | 12 | 38929341 | G | A | 0.007 | 0.002 | 1.50E-05 | -0.015 | 0.003 | 9.20E-09 | PC | -0.009 | 0.013 | -0.005 | 0.019 | -0.017 | 0.019 | -0.011 | 0.016 | -0.002 | 0.021 |  |
| rs74086911 | 12 | 50015942 | G | A | 0.015 | 0.003 | 2.10E-08 | -0.003 | 0.004 | 4.10E-01 | Smoking | -0.006 | 0.019 | 0.005 | 0.027 | -0.016 | 0.027 | -0.013 | 0.022 | 0.036 | 0.030 |  |
| rs7297175 | 12 | 56473808 | T | C | -0.008 | 0.001 | 6.60E-09 | 0.003 | 0.002 | 1.70E-01 | Smoking | -0.007 | 0.010 | 0.002 | 0.014 | -0.017 | 0.014 | -0.027 | 0.012 | 0.024 | 0.016 |  |
| rs10879871 | 12 | 75380511 | T | G | -0.010 | 0.001 | 5.00E-11 | 0.005 | 0.002 | 2.70E-02 | Smoking | 0.001 | 0.010 | -0.004 | 0.014 | 0.004 | 0.014 | -0.003 | 0.012 | -0.001 | 0.016 |  |
| rs12831617 | 12 | 84758368 | C | T | -0.009 | 0.002 | 1.90E-08 | 0.004 | 0.003 | 9.30E-02 | Smoking | 0.007 | 0.011 | -0.002 | 0.016 | 0.015 | 0.016 | 0.002 | 0.013 | 0.003 | 0.018 |  |
| rs206965 | 12 | 120856332 | T | C | 0.000 | 0.002 | 7.90E-01 | 0.016 | 0.003 | 9.10E-09 | PC | -0.008 | 0.012 | -0.014 | 0.017 | 0.001 | 0.017 | -0.001 | 0.014 | -0.029 | 0.019 |  |
| rs12874776 | 13 | 57794345 | C | A | 0.005 | 0.002 | 2.40E-02 | -0.023 | 0.004 | 8.00E-11 | PC | -0.010 | 0.016 | -0.008 | 0.023 | -0.009 | 0.022 | -0.008 | 0.019 | -0.012 | 0.025 |  |
| rs6562474 | 13 | 67332812 | C | G | 0.008 | 0.001 | 1.00E-08 | -0.005 | 0.002 | 4.00E-02 | Smoking | 0.010 | 0.010 | 0.010 | 0.014 | 0.008 | 0.014 | 0.016 | 0.012 | -0.004 | 0.016 |  |
| rs7333559 | 13 | 1.01E+08 | G | A | 0.011 | 0.002 | 3.20E-10 | 0.002 | 0.003 | 4.60E-01 | Smoking | -0.012 | 0.012 | -0.024 | 0.017 | 0.003 | 0.017 | 0.002 | 0.014 | -0.038 | 0.019 |  |
| rs198262 | 14 | 57283283 | C | T | -0.001 | 0.003 | 8.40E-01 | 0.030 | 0.005 | 3.90E-08 | PC | -0.023 | 0.030 | 0.005 | 0.035 | -0.003 | 0.034 | 0.015 | 0.029 | -0.038 | 0.038 |  |
| rs7155595 | 14 | 77502546 | A | C | -0.009 | 0.001 | 2.50E-09 | 0.006 | 0.002 | 1.50E-02 | Smoking | -0.021 | 0.012 | -0.026 | 0.015 | -0.024 | 0.015 | -0.020 | 0.012 | -0.026 | 0.016 |  |
| rs3742365 | 14 | 104198251 | T | C | -0.011 | 0.001 | 2.50E-14 | -0.004 | 0.002 | 4.70E-02 | Smoking | -0.021 | 0.012 | -0.036 | 0.014 | 0.014 | 0.014 | -0.013 | 0.012 | -0.034 | 0.016 |  |
| rs166835 | 15 | 47716037 | C | T | -0.002 | 0.001 | 2.40E-01 | 0.013 | 0.002 | 3.40E-09 | PC | -0.007 | 0.010 | -0.013 | 0.014 | 0.002 | 0.014 | -0.001 | 0.012 | -0.012 | 0.015 |  |
| rs28485305 | 15 | 74044197 | C | T | 0.008 | 0.001 | 2.60E-08 | 0.003 | 0.002 | 1.50E-01 | Smoking | -0.014 | 0.010 | -0.001 | 0.014 | -0.029 | 0.014 | -0.009 | 0.012 | -0.032 | 0.015 |  |
| rs8042849 | 15 | 78817929 | C | T | 0.019 | 0.001 | 1.80E-39 | -0.002 | 0.002 | 3.70E-01 | Smoking | 0.002 | 0.010 | 0.004 | 0.014 | -0.002 | 0.014 | -0.001 | 0.012 | 0.009 | 0.016 |  |
| rs4702 | 15 | 91426560 | G | A | 0.005 | 0.001 | 1.10E-04 | 0.012 | 0.002 | 3.80E-08 | PC | 0.018 | 0.010 | 0.022 | 0.014 | 0.014 | 0.014 | 0.020 | 0.012 | 0.013 | 0.015 |  |
| rs11861214 | 16 | 746611 | G | T | 0.009 | 0.002 | 2.00E-08 | -0.002 | 0.003 | 3.90E-01 | Smoking | 0.010 | 0.012 | 0.008 | 0.017 | 0.009 | 0.016 | 0.015 | 0.014 | 0.010 | 0.019 |  |
| rs6498759 | 16 | 18020295 | T | C | 0.000 | 0.002 | 7.40E-01 | 0.013 | 0.002 | 2.70E-08 | PC | -0.003 | 0.010 | 0.001 | 0.014 | -0.009 | 0.014 | -0.004 | 0.012 | 0.000 | 0.016 |  |
| rs57611503 | 16 | 31165795 | G | A | 0.008 | 0.001 | 4.00E-08 | 0.001 | 0.002 | 7.00E-01 | Smoking | 0.012 | 0.010 | 0.005 | 0.015 | 0.016 | 0.015 | 0.005 | 0.012 | 0.016 | 0.016 |  |
| rs3730399 | 16 | 67229019 | A | G | -0.002 | 0.003 | 4.90E-01 | 0.025 | 0.004 | 3.30E-08 | PC | -0.054 | 0.020 | -0.003 | 0.028 | -0.104 | 0.029 | -0.075 | 0.024 | -0.048 | 0.031 |  |
| rs889398 | 16 | 69556715 | C | T | 0.009 | 0.001 | 6.30E-11 | 0.002 | 0.002 | 4.90E-01 | Smoking | 0.016 | 0.010 | 0.014 | 0.014 | 0.016 | 0.014 | 0.020 | 0.012 | -0.003 | 0.015 |  |
| rs60952428 | 16 | 75640521 | T | C | 0.013 | 0.002 | 3.00E-08 | 0.002 | 0.004 | 5.70E-01 | Smoking | 0.022 | 0.017 | 0.011 | 0.024 | 0.032 | 0.024 | 0.028 | 0.021 | 0.001 | 0.027 |  |
| rs1050847 | 16 | 87443734 | C | T | 0.008 | 0.001 | 1.40E-08 | 0.000 | 0.002 | 8.50E-01 | Smoking | -0.017 | 0.010 | -0.024 | 0.014 | -0.010 | 0.014 | -0.021 | 0.012 | 0.008 | 0.016 |  |
| rs369230 | 16 | 89645437 | G | T | -0.009 | 0.002 | 1.80E-09 | -0.001 | 0.002 | 6.30E-01 | Smoking | -0.023 | 0.011 | -0.010 | 0.016 | -0.036 | 0.016 | -0.037 | 0.013 | -0.003 | 0.018 |  |
| rs7209653 | 17 | 19882084 | T | C | 0.000 | 0.002 | 8.40E-01 | 0.016 | 0.002 | 9.10E-11 | PC | -0.006 | 0.011 | -0.010 | 0.015 | 0.001 | 0.015 | -0.016 | 0.013 | 0.011 | 0.017 |  |
| rs8614 | 17 | 27588806 | C | A | -0.011 | 0.002 | 1.80E-10 | -0.004 | 0.003 | 1.80E-01 | Smoking | -0.025 | 0.013 | -0.030 | 0.018 | -0.025 | 0.018 | -0.016 | 0.015 | -0.060 | 0.020 |  |
| rs12603813 | 17 | 43196584 | T | C | -0.001 | 0.002 | 3.90E-01 | 0.014 | 0.003 | 1.30E-08 | PC | -0.003 | 0.011 | -0.024 | 0.016 | 0.018 | 0.016 | 0.012 | 0.013 | -0.019 | 0.017 |  |
| rs9904288 | 17 | 47031973 | T | C | 0.008 | 0.002 | 3.10E-08 | -0.004 | 0.002 | 9.30E-02 | Smoking | -0.020 | 0.011 | -0.012 | 0.015 | -0.032 | 0.015 | -0.023 | 0.013 | -0.024 | 0.017 |  |
| rs66643547 | 17 | 79375315 | C | T | 0.004 | 0.001 | 1.60E-02 | -0.015 | 0.002 | 3.20E-11 | PC | 0.011 | 0.010 | 0.010 | 0.015 | 0.011 | 0.015 | 0.009 | 0.012 | 0.016 | 0.016 |  |
| rs12967855 | 18 | 35138245 | A | G | 0.008 | 0.001 | 3.10E-08 | -0.005 | 0.002 | 4.50E-02 | Smoking | 0.000 | 0.010 | 0.008 | 0.014 | -0.006 | 0.015 | 0.002 | 0.012 | 0.005 | 0.016 |  |
| rs62098013 | 18 | 50863861 | G | A | -0.009 | 0.001 | 4.10E-09 | 0.003 | 0.002 | 2.40E-01 | Smoking | -0.016 | 0.010 | -0.015 | 0.015 | -0.016 | 0.015 | -0.010 | 0.012 | -0.014 | 0.016 |  |
| rs784256 | 18 | 53398626 | G | A | -0.005 | 0.002 | 5.30E-03 | 0.019 | 0.003 | 6.20E-11 | PC | 0.011 | 0.013 | 0.010 | 0.018 | 0.013 | 0.018 | 0.006 | 0.015 | 0.006 | 0.020 |  |
| rs71367545 | 18 | 77576337 | G | A | -0.010 | 0.002 | 1.40E-09 | -0.004 | 0.003 | 1.80E-01 | Smoking | -0.024 | 0.012 | -0.005 | 0.018 | -0.038 | 0.018 | -0.016 | 0.015 | -0.052 | 0.019 |  |
| rs76608582 | 19 | 4474725 | C | A | 0.022 | 0.003 | 3.20E-10 | 0.001 | 0.005 | 8.40E-01 | Smoking | 0.048 | 0.030 | 0.024 | 0.043 | 0.075 | 0.042 | 0.106 | 0.036 | -0.040 | 0.046 |  |
| rs35343344 | 19 | 18471610 | C | A | 0.009 | 0.002 | 8.80E-09 | -0.005 | 0.003 | 6.20E-02 | Smoking | 0.000 | 0.011 | -0.022 | 0.016 | 0.023 | 0.016 | -0.004 | 0.013 | -0.002 | 0.018 |  |
| rs4814873 | 20 | 19616429 | C | T | 0.010 | 0.002 | 2.90E-09 | -0.008 | 0.003 | 2.80E-03 | Smoking | -0.006 | 0.012 | -0.005 | 0.016 | -0.009 | 0.016 | 0.000 | 0.014 | -0.018 | 0.018 |  |
| rs6119897 | 20 | 31145415 | G | A | -0.013 | 0.002 | 3.60E-15 | 0.004 | 0.003 | 1.20E-01 | Smoking | -0.010 | 0.012 | -0.002 | 0.016 | -0.015 | 0.016 | -0.015 | 0.014 | -0.004 | 0.018 |  |
| rs12481282 | 20 | 44761377 | G | C | -0.009 | 0.002 | 7.80E-09 | -0.002 | 0.002 | 3.30E-01 | Smoking | -0.011 | 0.011 | 0.014 | 0.015 | -0.033 | 0.015 | -0.023 | 0.013 | 0.003 | 0.017 |  |
| rs348809 | 20 | 59032097 | A | G | -0.008 | 0.001 | 1.30E-08 | -0.001 | 0.002 | 7.50E-01 | Smoking | 0.003 | 0.010 | -0.009 | 0.014 | 0.013 | 0.014 | -0.003 | 0.012 | -0.003 | 0.016 |  |
| rs6129084 | 20 | 59828857 | A | T | -0.001 | 0.001 | 5.10E-01 | -0.014 | 0.002 | 9.30E-10 | PC | 0.015 | 0.010 | 0.019 | 0.014 | 0.009 | 0.014 | 0.003 | 0.012 | 0.051 | 0.016 |  |
| rs6011779 | 20 | 61984317 | C | T | 0.019 | 0.002 | 2.30E-27 | -0.007 | 0.003 | 1.10E-02 | Smoking | 0.019 | 0.012 | 0.034 | 0.017 | 0.007 | 0.017 | 0.008 | 0.015 | 0.056 | 0.019 |  |
| rs147412694 | 21 | 40702786 | G | A | -0.012 | 0.002 | 2.90E-09 | -0.008 | 0.003 | 1.30E-02 | Smoking | 0.030 | 0.014 | 0.015 | 0.020 | 0.043 | 0.020 | 0.031 | 0.017 | 0.003 | 0.022 |  |
| rs2838834 | 21 | 46665208 | C | T | -0.009 | 0.002 | 6.30E-10 | 0.000 | 0.002 | 9.80E-01 | Smoking | -0.002 | 0.010 | -0.005 | 0.015 | -0.002 | 0.015 | -0.001 | 0.013 | -0.014 | 0.017 |  |
| rs136553 | 22 | 27255675 | C | T | -0.002 | 0.001 | 2.10E-01 | -0.015 | 0.002 | 2.30E-11 | PC | 0.014 | 0.010 | 0.024 | 0.014 | 0.005 | 0.014 | 0.007 | 0.012 | 0.011 | 0.016 |  |
| rs136233 | 22 | 31212410 | A | G | -0.010 | 0.002 | 1.80E-08 | 0.001 | 0.003 | 6.70E-01 | Smoking | 0.000 | 0.012 | -0.005 | 0.017 | 0.007 | 0.017 | -0.001 | 0.015 | -0.014 | 0.019 |  |
| rs7288455 | 22 | 39966547 | A | G | -0.002 | 0.001 | 1.30E-01 | 0.013 | 0.002 | 4.00E-09 | PC | -0.002 | 0.010 | -0.004 | 0.014 | -0.004 | 0.014 | 0.006 | 0.012 | -0.010 | 0.016 |  |
| rs202645 | 22 | 41798520 | A | G | -0.010 | 0.002 | 3.90E-09 | 0.005 | 0.003 | 7.60E-02 | Smoking | 0.001 | 0.012 | -0.012 | 0.017 | 0.018 | 0.017 | 0.002 | 0.014 | 0.003 | 0.019 |  |

| **Table S24. Beta estimates for the SNPs included in the multivariable MR of smoking, leisure television use, and breast cancer risk** | | | | | | | | | | | | | | | | | | | | | | | |
| --- | --- | --- | --- | --- | --- | --- | --- | --- | --- | --- | --- | --- | --- | --- | --- | --- | --- | --- | --- | --- | --- | --- | --- |
| SNP | CHR | POS | EA | OA | beta_smk | se_smk | pval_smk | beta_tv | se_tv | pval_tv | Phenotype | beta_br | se_br | Luminal_A  _beta | Luminal_A  _se | Luminal_B  _beta | Luminal_B  _se | Luminal_B  _HER2Neg  _beta | Luminal_B  _HER2Neg  _se | HER2  _Enriched  _beta | HER2_  Enriched  _se | Triple_Neg  _beta | Triple_Neg  _se |
| rs1193237 | 1 | 7526486 | G | C | -0.008 | 0.001 | 2.80E-08 | -0.009 | 0.002 | 1.80E-05 | smoking | 0.005 | 0.007 | 0.010 | 0.009 | -0.012 | 0.019 | -0.003 | 0.016 | 0.016 | 0.027 | -0.007 | 0.017 |
| rs4949465 | 1 | 32178489 | T | C | -0.012 | 0.002 | 1.70E-08 | -0.012 | 0.003 | 3.00E-04 | smoking | 0.001 | 0.009 | 0.014 | 0.012 | -0.050 | 0.026 | -0.011 | 0.023 | 0.030 | 0.039 | -0.009 | 0.023 |
| rs11810109 | 1 | 44186812 | A | T | 0.010 | 0.002 | 5.10E-11 | 0.016 | 0.002 | 5.40E-12 | TV | 0.005 | 0.007 | -0.001 | 0.009 | 0.020 | 0.019 | 0.016 | 0.016 | 0.000 | 0.028 | 0.026 | 0.017 |
| rs12725114 | 1 | 62432641 | G | A | -0.001 | 0.002 | 4.70E-01 | 0.015 | 0.003 | 1.60E-08 | TV | 0.007 | 0.007 | 0.016 | 0.010 | 0.029 | 0.022 | 0.002 | 0.018 | 0.040 | 0.032 | -0.004 | 0.019 |
| rs984409 | 1 | 67020440 | G | A | -0.002 | 0.001 | 1.80E-01 | -0.015 | 0.002 | 4.10E-11 | TV | -0.015 | 0.006 | -0.019 | 0.008 | -0.006 | 0.019 | -0.032 | 0.016 | 0.022 | 0.027 | -0.025 | 0.016 |
| rs71658797 | 1 | 77967507 | T | A | -0.007 | 0.002 | 5.80E-04 | -0.020 | 0.003 | 6.30E-10 | TV | -0.004 | 0.010 | -0.003 | 0.013 | -0.034 | 0.029 | 0.059 | 0.025 | -0.038 | 0.042 | 0.002 | 0.025 |
| rs114328297 | 1 | 91190854 | T | G | 0.010 | 0.002 | 1.30E-08 | 0.014 | 0.003 | 4.40E-08 | TV | -0.006 | 0.008 | 0.001 | 0.010 | -0.021 | 0.023 | -0.019 | 0.020 | 0.053 | 0.034 | -0.033 | 0.020 |
| rs17379561 | 1 | 98340139 | A | T | -0.006 | 0.002 | 5.30E-03 | -0.025 | 0.003 | 1.10E-16 | TV | -0.002 | 0.009 | -0.001 | 0.011 | 0.052 | 0.025 | -0.029 | 0.021 | 0.010 | 0.036 | -0.008 | 0.021 |
| rs55909997 | 1 | 107581039 | G | A | -0.008 | 0.001 | 1.10E-07 | -0.014 | 0.002 | 2.40E-09 | TV | -0.002 | 0.006 | 0.002 | 0.008 | 0.016 | 0.019 | -0.024 | 0.016 | -0.005 | 0.027 | -0.024 | 0.016 |
| rs1278847 | 1 | 110037483 | C | A | 0.002 | 0.002 | 2.80E-01 | 0.016 | 0.002 | 1.20E-11 | TV | -0.012 | 0.007 | -0.016 | 0.009 | -0.008 | 0.019 | -0.004 | 0.017 | -0.017 | 0.028 | 0.011 | 0.017 |
| rs4845364 | 1 | 154141908 | A | G | 0.003 | 0.001 | 1.60E-02 | -0.015 | 0.002 | 1.30E-12 | TV | 0.005 | 0.006 | 0.007 | 0.008 | 0.002 | 0.018 | -0.002 | 0.015 | 0.018 | 0.026 | 0.020 | 0.015 |
| rs6673341 | 1 | 184655649 | T | G | -0.003 | 0.001 | 3.30E-02 | -0.015 | 0.002 | 2.20E-11 | TV | 0.000 | 0.006 | -0.006 | 0.008 | -0.004 | 0.018 | 0.007 | 0.015 | -0.005 | 0.026 | -0.026 | 0.015 |
| rs10737620 | 1 | 193057971 | T | A | 0.001 | 0.002 | 6.20E-01 | 0.014 | 0.002 | 2.60E-09 | TV | 0.011 | 0.007 | 0.022 | 0.009 | 0.017 | 0.020 | 0.012 | 0.017 | 0.012 | 0.029 | -0.017 | 0.017 |
| rs2867112 | 2 | 651349 | T | G | 0.015 | 0.002 | 4.80E-15 | 0.005 | 0.003 | 9.10E-02 | smoking | -0.030 | 0.008 | -0.017 | 0.010 | -0.013 | 0.023 | -0.022 | 0.020 | -0.099 | 0.032 | -0.034 | 0.020 |
| rs6741228 | 2 | 22548774 | T | C | 0.008 | 0.001 | 1.60E-08 | 0.004 | 0.002 | 9.70E-02 | smoking | -0.004 | 0.006 | -0.006 | 0.008 | 0.011 | 0.018 | -0.005 | 0.015 | 0.027 | 0.026 | 0.005 | 0.015 |
| rs72781699 | 2 | 24277709 | G | A | -0.003 | 0.002 | 4.40E-02 | -0.019 | 0.003 | 3.00E-12 | TV | 0.011 | 0.008 | 0.006 | 0.010 | 0.016 | 0.022 | -0.015 | 0.019 | -0.007 | 0.032 | 0.043 | 0.019 |
| rs12105701 | 2 | 41737200 | C | T | -0.001 | 0.001 | 6.50E-01 | -0.013 | 0.002 | 5.40E-09 | TV | -0.003 | 0.006 | -0.004 | 0.008 | -0.020 | 0.018 | 0.043 | 0.016 | 0.026 | 0.026 | -0.027 | 0.016 |
| rs7564130 | 2 | 50606642 | T | C | -0.005 | 0.001 | 5.10E-04 | -0.015 | 0.002 | 2.80E-11 | TV | -0.002 | 0.006 | -0.010 | 0.008 | -0.016 | 0.018 | -0.005 | 0.016 | 0.005 | 0.026 | 0.006 | 0.016 |
| rs13016665 | 2 | 57995348 | C | A | -0.008 | 0.001 | 1.80E-09 | -0.004 | 0.002 | 9.90E-02 | smoking | 0.012 | 0.006 | 0.012 | 0.008 | 0.014 | 0.018 | 0.019 | 0.015 | 0.014 | 0.026 | 0.022 | 0.016 |
| rs10189857 | 2 | 60713235 | A | G | -0.003 | 0.001 | 1.80E-02 | -0.020 | 0.002 | 6.20E-21 | TV | -0.017 | 0.006 | -0.019 | 0.008 | -0.011 | 0.018 | -0.022 | 0.015 | -0.008 | 0.026 | -0.015 | 0.015 |
| rs62641636 | 2 | 68425427 | A | G | 0.000 | 0.002 | 8.90E-01 | 0.014 | 0.002 | 7.00E-10 | TV | 0.001 | 0.007 | -0.003 | 0.009 | 0.039 | 0.019 | 0.009 | 0.016 | -0.042 | 0.028 | 0.016 | 0.017 |
| rs11689199 | 2 | 100820421 | A | G | 0.003 | 0.001 | 1.60E-02 | 0.018 | 0.002 | 5.50E-17 | TV | -0.010 | 0.006 | -0.008 | 0.008 | -0.016 | 0.018 | -0.007 | 0.016 | -0.014 | 0.027 | -0.017 | 0.016 |
| rs2678670 | 2 | 104469564 | A | T | 0.009 | 0.001 | 3.10E-10 | 0.015 | 0.002 | 1.60E-11 | smoking | 0.015 | 0.006 | 0.012 | 0.008 | -0.004 | 0.018 | 0.023 | 0.015 | -0.001 | 0.026 | 0.032 | 0.015 |
| rs1451533 | 2 | 105466005 | G | A | -0.001 | 0.002 | 7.30E-01 | -0.016 | 0.002 | 1.10E-10 | TV | -0.004 | 0.007 | -0.015 | 0.009 | 0.032 | 0.021 | -0.001 | 0.018 | 0.018 | 0.031 | -0.005 | 0.018 |
| rs12476388 | 2 | 116330983 | C | T | 0.001 | 0.002 | 4.30E-01 | 0.013 | 0.002 | 2.10E-08 | TV | -0.002 | 0.007 | 0.002 | 0.009 | 0.009 | 0.019 | -0.004 | 0.017 | -0.035 | 0.028 | -0.014 | 0.017 |
| rs374722 | 2 | 147839830 | G | A | 0.000 | 0.002 | 8.80E-01 | 0.024 | 0.003 | 5.50E-16 | TV | 0.011 | 0.009 | 0.018 | 0.012 | 0.036 | 0.025 | -0.012 | 0.022 | -0.080 | 0.039 | 0.001 | 0.022 |
| rs3754970 | 2 | 162091836 | T | C | -0.006 | 0.001 | 1.80E-05 | -0.015 | 0.002 | 4.80E-12 | TV | 0.007 | 0.006 | 0.000 | 0.008 | -0.003 | 0.018 | 0.006 | 0.015 | -0.017 | 0.026 | 0.023 | 0.016 |
| rs2060199 | 2 | 166196681 | T | A | -0.001 | 0.001 | 3.30E-01 | 0.012 | 0.002 | 2.40E-08 | TV | -0.007 | 0.006 | -0.007 | 0.008 | 0.010 | 0.018 | 0.019 | 0.016 | 0.011 | 0.026 | -0.021 | 0.016 |
| rs13009008 | 2 | 174043233 | A | G | 0.009 | 0.001 | 4.60E-09 | 0.010 | 0.002 | 1.70E-05 | smoking | 0.007 | 0.006 | 0.006 | 0.008 | 0.005 | 0.019 | 0.006 | 0.016 | -0.037 | 0.027 | 0.025 | 0.016 |
| rs4473348 | 2 | 182073742 | A | T | -0.010 | 0.002 | 6.40E-11 | -0.012 | 0.002 | 1.20E-06 | smoking | 0.003 | 0.007 | 0.000 | 0.009 | -0.023 | 0.021 | -0.008 | 0.018 | -0.040 | 0.030 | 0.024 | 0.018 |
| rs4577309 | 2 | 191288833 | A | G | 0.001 | 0.001 | 3.50E-01 | 0.016 | 0.002 | 1.60E-13 | TV | -0.001 | 0.006 | 0.004 | 0.008 | -0.014 | 0.018 | 0.008 | 0.015 | -0.006 | 0.026 | -0.015 | 0.015 |
| rs34224261 | 2 | 193855452 | G | C | 0.001 | 0.001 | 6.00E-01 | -0.018 | 0.002 | 4.60E-16 | TV | 0.000 | 0.006 | -0.006 | 0.008 | 0.004 | 0.017 | -0.026 | 0.015 | -0.060 | 0.026 | -0.001 | 0.015 |
| rs4675246 | 2 | 202864487 | G | T | -0.006 | 0.002 | 2.70E-04 | -0.015 | 0.003 | 2.90E-08 | TV | 0.006 | 0.008 | 0.013 | 0.010 | -0.005 | 0.022 | 0.038 | 0.019 | -0.077 | 0.032 | -0.040 | 0.019 |
| rs13029509 | 2 | 215374209 | G | A | -0.002 | 0.001 | 1.60E-01 | -0.018 | 0.002 | 2.00E-17 | TV | -0.009 | 0.006 | -0.008 | 0.008 | 0.008 | 0.018 | -0.019 | 0.015 | 0.017 | 0.026 | -0.017 | 0.015 |
| rs10932837 | 2 | 221044785 | C | T | 0.000 | 0.001 | 7.80E-01 | -0.013 | 0.002 | 1.30E-09 | TV | 0.001 | 0.006 | 0.003 | 0.008 | -0.011 | 0.018 | 0.009 | 0.015 | -0.023 | 0.026 | 0.000 | 0.015 |
| rs4973576 | 2 | 233806771 | C | A | 0.002 | 0.002 | 2.20E-01 | -0.015 | 0.002 | 1.00E-09 | TV | 0.012 | 0.007 | 0.022 | 0.009 | 0.031 | 0.020 | -0.015 | 0.017 | 0.018 | 0.029 | 0.026 | 0.017 |
| rs138256022 | 3 | 11603090 | C | T | -0.006 | 0.004 | 1.00E-01 | -0.031 | 0.006 | 2.90E-08 | TV | -0.016 | 0.017 | -0.025 | 0.023 | -0.024 | 0.050 | -0.043 | 0.043 | -0.061 | 0.072 | -0.005 | 0.044 |
| rs9834970 | 3 | 36856030 | T | C | 0.002 | 0.001 | 7.10E-02 | 0.013 | 0.002 | 3.30E-09 | TV | 0.001 | 0.006 | 0.001 | 0.008 | 0.005 | 0.018 | 0.004 | 0.016 | -0.035 | 0.027 | 0.011 | 0.016 |
| rs3796386 | 3 | 49899795 | G | A | -0.006 | 0.001 | 3.80E-05 | -0.026 | 0.002 | 3.20E-33 | TV | -0.018 | 0.006 | -0.014 | 0.008 | -0.038 | 0.018 | -0.012 | 0.015 | -0.063 | 0.026 | -0.043 | 0.015 |
| rs11130793 | 3 | 60884659 | C | T | 0.002 | 0.001 | 1.30E-01 | 0.013 | 0.002 | 5.00E-09 | TV | 0.001 | 0.006 | 0.006 | 0.008 | 0.012 | 0.018 | -0.021 | 0.016 | 0.004 | 0.027 | 0.002 | 0.016 |
| rs11714337 | 3 | 71582521 | G | A | 0.005 | 0.001 | 7.60E-04 | 0.014 | 0.002 | 4.70E-11 | TV | 0.032 | 0.006 | 0.045 | 0.008 | 0.036 | 0.018 | 0.029 | 0.015 | 0.000 | 0.026 | 0.014 | 0.015 |
| rs775758 | 3 | 77582005 | A | T | 0.008 | 0.001 | 1.10E-08 | 0.003 | 0.002 | 1.90E-01 | smoking | 0.002 | 0.006 | 0.008 | 0.009 | 0.010 | 0.019 | 0.019 | 0.016 | -0.002 | 0.027 | 0.007 | 0.016 |
| rs421983 | 3 | 84892866 | T | C | 0.009 | 0.001 | 3.30E-10 | 0.005 | 0.002 | 2.40E-02 | smoking | 0.002 | 0.006 | 0.001 | 0.008 | -0.011 | 0.018 | 0.010 | 0.015 | 0.005 | 0.026 | 0.010 | 0.016 |
| rs6797840 | 3 | 85656569 | A | C | -0.004 | 0.001 | 7.30E-03 | -0.016 | 0.002 | 1.70E-13 | TV | -0.004 | 0.006 | -0.007 | 0.008 | -0.015 | 0.018 | -0.022 | 0.015 | 0.024 | 0.026 | 0.005 | 0.015 |
| rs2034768 | 3 | 93986371 | A | G | 0.006 | 0.001 | 4.50E-06 | 0.015 | 0.002 | 8.60E-12 | TV | -0.003 | 0.006 | -0.006 | 0.008 | 0.010 | 0.018 | 0.016 | 0.015 | 0.016 | 0.025 | -0.025 | 0.015 |
| rs326341 | 3 | 107811142 | G | A | 0.009 | 0.001 | 1.20E-11 | 0.003 | 0.002 | 1.60E-01 | smoking | 0.000 | 0.006 | 0.000 | 0.008 | 0.004 | 0.018 | 0.002 | 0.015 | -0.003 | 0.026 | -0.009 | 0.016 |
| rs9867121 | 3 | 114631548 | C | A | 0.002 | 0.002 | 2.30E-01 | 0.019 | 0.003 | 3.90E-12 | TV | 0.013 | 0.008 | 0.015 | 0.010 | 0.020 | 0.023 | 0.001 | 0.020 | 0.052 | 0.034 | 0.009 | 0.020 |
| rs73220544 | 3 | 131074511 | A | C | -0.011 | 0.002 | 1.50E-08 | 0.000 | 0.003 | 9.80E-01 | smoking | -0.002 | 0.009 | -0.002 | 0.011 | -0.013 | 0.024 | 0.001 | 0.021 | 0.056 | 0.036 | 0.011 | 0.021 |
| rs12491503 | 3 | 165706855 | G | A | -0.003 | 0.001 | 5.50E-02 | -0.014 | 0.002 | 5.50E-10 | TV | -0.001 | 0.007 | 0.008 | 0.009 | -0.018 | 0.019 | -0.029 | 0.016 | -0.025 | 0.028 | 0.009 | 0.017 |
| rs114600294 | 3 | 181419367 | G | C | 0.002 | 0.002 | 3.10E-01 | -0.016 | 0.003 | 7.90E-10 | TV | 0.017 | 0.010 | 0.002 | 0.013 | 0.029 | 0.028 | -0.015 | 0.024 | 0.035 | 0.042 | 0.007 | 0.025 |
| rs66852340 | 4 | 3311070 | C | T | -0.005 | 0.002 | 1.20E-03 | -0.018 | 0.003 | 7.90E-12 | TV | -0.016 | 0.007 | -0.019 | 0.009 | 0.027 | 0.021 | -0.046 | 0.018 | -0.011 | 0.031 | -0.022 | 0.018 |
| rs7700107 | 4 | 17880416 | A | C | 0.001 | 0.002 | 7.80E-01 | -0.021 | 0.003 | 1.90E-11 | TV | 0.005 | 0.009 | 0.033 | 0.012 | -0.019 | 0.025 | -0.026 | 0.022 | -0.066 | 0.036 | -0.009 | 0.022 |
| rs317021 | 4 | 35418368 | T | A | -0.012 | 0.002 | 1.10E-10 | -0.001 | 0.003 | 7.50E-01 | smoking | -0.008 | 0.009 | -0.009 | 0.011 | 0.035 | 0.025 | -0.053 | 0.021 | -0.012 | 0.036 | -0.015 | 0.022 |
| rs28971796 | 4 | 49151982 | A | G | 0.004 | 0.002 | 6.30E-03 | 0.015 | 0.002 | 2.10E-09 | TV | 0.010 | 0.009 | 0.017 | 0.012 | 0.042 | 0.026 | 0.005 | 0.022 | 0.049 | 0.037 | 0.017 | 0.022 |
| rs6850494 | 4 | 82291771 | A | C | -0.004 | 0.001 | 9.20E-03 | -0.014 | 0.002 | 1.10E-10 | TV | 0.009 | 0.006 | 0.004 | 0.008 | 0.030 | 0.018 | 0.002 | 0.015 | -0.045 | 0.026 | 0.020 | 0.016 |
| rs13107325 | 4 | 103188709 | C | T | 0.004 | 0.003 | 1.50E-01 | -0.029 | 0.004 | 1.50E-12 | TV | -0.019 | 0.012 | -0.021 | 0.016 | -0.051 | 0.034 | -0.007 | 0.030 | 0.051 | 0.051 | -0.025 | 0.030 |
| rs7693703 | 4 | 118357405 | G | A | 0.001 | 0.002 | 5.80E-01 | 0.023 | 0.004 | 2.70E-09 | TV | -0.007 | 0.010 | -0.015 | 0.014 | -0.012 | 0.030 | 0.009 | 0.026 | 0.052 | 0.046 | 0.005 | 0.026 |
| rs4334769 | 4 | 130245233 | G | T | 0.001 | 0.001 | 7.00E-01 | 0.012 | 0.002 | 2.10E-08 | TV | -0.008 | 0.006 | -0.007 | 0.008 | -0.016 | 0.018 | -0.032 | 0.015 | 0.012 | 0.026 | 0.015 | 0.015 |
| rs17576594 | 4 | 147952241 | G | A | 0.011 | 0.002 | 1.70E-12 | 0.002 | 0.002 | 5.00E-01 | smoking | -0.001 | 0.007 | -0.005 | 0.009 | -0.013 | 0.020 | -0.020 | 0.017 | -0.002 | 0.029 | -0.006 | 0.017 |
| rs6825241 | 4 | 152543783 | C | A | 0.000 | 0.001 | 7.40E-01 | -0.017 | 0.002 | 4.90E-15 | TV | 0.002 | 0.006 | 0.009 | 0.008 | -0.008 | 0.018 | -0.018 | 0.015 | 0.008 | 0.026 | 0.006 | 0.016 |
| rs7693082 | 4 | 159857819 | G | C | 0.002 | 0.002 | 2.80E-01 | 0.015 | 0.002 | 1.70E-10 | TV | -0.004 | 0.007 | -0.013 | 0.009 | -0.023 | 0.020 | -0.001 | 0.017 | 0.044 | 0.028 | -0.013 | 0.017 |
| rs11948770 | 5 | 13246336 | T | C | -0.010 | 0.002 | 4.90E-10 | -0.007 | 0.003 | 6.50E-03 | smoking | -0.007 | 0.007 | 0.000 | 0.009 | 0.016 | 0.021 | 0.012 | 0.018 | -0.023 | 0.030 | -0.021 | 0.018 |
| rs71627581 | 5 | 43161351 | G | A | 0.013 | 0.002 | 1.60E-09 | 0.001 | 0.003 | 7.10E-01 | smoking | 0.014 | 0.010 | 0.016 | 0.013 | 0.002 | 0.029 | 0.020 | 0.025 | 0.007 | 0.042 | -0.018 | 0.025 |
| rs10940659 | 5 | 59360341 | A | G | 0.000 | 0.001 | 9.70E-01 | -0.013 | 0.002 | 1.50E-08 | TV | 0.008 | 0.006 | 0.020 | 0.008 | -0.061 | 0.018 | 0.014 | 0.015 | -0.028 | 0.026 | 0.003 | 0.015 |
| rs262890 | 5 | 62930015 | A | G | -0.005 | 0.002 | 2.10E-03 | -0.019 | 0.002 | 3.20E-15 | TV | -0.003 | 0.007 | -0.002 | 0.009 | 0.032 | 0.019 | -0.006 | 0.016 | -0.021 | 0.028 | -0.008 | 0.017 |
| rs7716447 | 5 | 88800331 | A | G | -0.007 | 0.001 | 1.10E-06 | -0.013 | 0.002 | 3.60E-09 | TV | 0.007 | 0.007 | 0.009 | 0.009 | -0.013 | 0.019 | 0.011 | 0.016 | 0.034 | 0.028 | -0.011 | 0.017 |
| rs1031423 | 5 | 93276883 | T | C | -0.004 | 0.002 | 1.70E-02 | -0.019 | 0.003 | 1.80E-12 | TV | -0.002 | 0.008 | -0.007 | 0.010 | 0.013 | 0.022 | 0.013 | 0.019 | -0.010 | 0.032 | 0.021 | 0.019 |
| rs57585211 | 5 | 107197725 | T | G | -0.002 | 0.002 | 3.30E-01 | -0.017 | 0.003 | 4.30E-09 | TV | 0.001 | 0.008 | 0.000 | 0.011 | 0.021 | 0.024 | -0.036 | 0.020 | 0.044 | 0.035 | -0.026 | 0.020 |
| rs9326862 | 5 | 112054547 | A | T | 0.006 | 0.001 | 4.20E-05 | 0.012 | 0.002 | 2.60E-08 | TV | 0.005 | 0.006 | 0.008 | 0.008 | 0.008 | 0.018 | -0.002 | 0.015 | 0.025 | 0.026 | 0.005 | 0.015 |
| rs10041724 | 5 | 124273520 | T | C | 0.005 | 0.002 | 2.80E-03 | 0.018 | 0.003 | 3.90E-11 | TV | 0.008 | 0.008 | 0.006 | 0.010 | 0.020 | 0.022 | -0.011 | 0.019 | 0.065 | 0.033 | -0.021 | 0.019 |
| rs329120 | 5 | 133861756 | C | T | 0.010 | 0.001 | 6.30E-12 | 0.012 | 0.002 | 7.10E-08 | smoking | 0.004 | 0.006 | 0.007 | 0.008 | 0.028 | 0.018 | 0.017 | 0.015 | -0.018 | 0.026 | 0.027 | 0.015 |
| rs62379379 | 5 | 141082015 | G | T | -0.004 | 0.003 | 1.70E-01 | -0.026 | 0.004 | 7.80E-10 | TV | -0.001 | 0.012 | -0.014 | 0.016 | 0.074 | 0.037 | 0.019 | 0.031 | 0.122 | 0.055 | -0.031 | 0.030 |
| rs10054327 | 5 | 147871137 | G | A | 0.002 | 0.001 | 2.00E-01 | 0.017 | 0.002 | 3.40E-15 | TV | -0.010 | 0.006 | -0.013 | 0.008 | 0.000 | 0.018 | 0.004 | 0.015 | 0.003 | 0.026 | -0.022 | 0.016 |
| rs42210 | 5 | 166408788 | G | C | -0.006 | 0.002 | 9.00E-05 | -0.014 | 0.002 | 7.30E-09 | TV | -0.020 | 0.007 | -0.008 | 0.010 | -0.046 | 0.021 | -0.046 | 0.018 | -0.083 | 0.031 | -0.019 | 0.018 |
| rs72828890 | 5 | 167806836 | C | T | 0.003 | 0.002 | 1.10E-01 | 0.019 | 0.003 | 4.90E-09 | TV | -0.005 | 0.011 | 0.006 | 0.014 | -0.010 | 0.032 | 0.027 | 0.028 | 0.002 | 0.047 | 0.012 | 0.028 |
| rs245774 | 5 | 170530930 | A | G | -0.009 | 0.002 | 7.40E-09 | -0.008 | 0.002 | 1.60E-03 | smoking | 0.000 | 0.007 | -0.006 | 0.009 | 0.020 | 0.020 | -0.003 | 0.017 | -0.010 | 0.029 | 0.020 | 0.017 |
| rs72834698 | 6 | 26176517 | G | A | 0.006 | 0.002 | 1.30E-03 | 0.023 | 0.003 | 2.70E-13 | TV | 0.007 | 0.009 | 0.019 | 0.012 | -0.001 | 0.026 | -0.022 | 0.022 | 0.030 | 0.038 | 0.019 | 0.023 |
| rs9471333 | 6 | 40362023 | C | T | 0.001 | 0.001 | 6.10E-01 | 0.013 | 0.002 | 1.50E-09 | TV | 0.000 | 0.006 | 0.012 | 0.009 | -0.008 | 0.019 | -0.039 | 0.016 | -0.044 | 0.027 | 0.011 | 0.016 |
| rs2894808 | 6 | 52861990 | T | A | -0.015 | 0.003 | 3.50E-09 | -0.016 | 0.004 | 1.00E-04 | smoking | -0.021 | 0.011 | -0.021 | 0.015 | 0.027 | 0.033 | -0.009 | 0.028 | -0.040 | 0.047 | -0.013 | 0.028 |
| rs4523073 | 6 | 67550288 | A | G | -0.008 | 0.001 | 3.80E-09 | -0.014 | 0.002 | 5.00E-10 | TV | -0.002 | 0.006 | -0.004 | 0.008 | 0.025 | 0.018 | -0.006 | 0.016 | -0.003 | 0.027 | 0.008 | 0.016 |
| rs6905544 | 6 | 98411631 | A | G | -0.002 | 0.001 | 9.70E-02 | -0.019 | 0.002 | 8.50E-18 | TV | -0.011 | 0.006 | -0.016 | 0.008 | -0.007 | 0.018 | -0.012 | 0.015 | 0.005 | 0.026 | -0.012 | 0.016 |
| rs17789218 | 6 | 100600097 | T | C | 0.001 | 0.002 | 5.60E-01 | 0.019 | 0.003 | 1.40E-13 | TV | -0.013 | 0.008 | -0.025 | 0.011 | -0.002 | 0.023 | 0.030 | 0.020 | 0.068 | 0.034 | 0.014 | 0.020 |
| rs7766610 | 6 | 111707821 | C | A | 0.013 | 0.002 | 2.20E-12 | 0.002 | 0.003 | 4.90E-01 | smoking | -0.004 | 0.008 | -0.004 | 0.010 | -0.011 | 0.022 | -0.030 | 0.019 | -0.003 | 0.032 | -0.015 | 0.019 |
| rs2184364 | 6 | 143183596 | A | G | 0.004 | 0.002 | 1.40E-02 | 0.016 | 0.003 | 3.00E-09 | TV | 0.007 | 0.007 | 0.009 | 0.010 | 0.009 | 0.022 | -0.004 | 0.018 | -0.035 | 0.031 | 0.023 | 0.019 |
| rs9718104 | 6 | 166170539 | T | G | 0.001 | 0.003 | 7.50E-01 | -0.041 | 0.005 | 9.30E-19 | TV | -0.010 | 0.014 | -0.009 | 0.018 | 0.009 | 0.041 | 0.004 | 0.034 | 0.041 | 0.060 | -0.054 | 0.034 |
| rs17568389 | 7 | 8091876 | T | A | 0.001 | 0.001 | 5.20E-01 | 0.015 | 0.002 | 8.60E-13 | TV | -0.003 | 0.006 | -0.008 | 0.008 | -0.012 | 0.018 | -0.014 | 0.015 | 0.038 | 0.026 | 0.010 | 0.016 |
| rs10226228 | 7 | 32315613 | A | G | -0.011 | 0.001 | 2.00E-15 | -0.007 | 0.002 | 2.30E-03 | smoking | 0.013 | 0.006 | 0.014 | 0.008 | 0.034 | 0.018 | 0.007 | 0.016 | -0.016 | 0.027 | 0.005 | 0.016 |
| rs10234444 | 7 | 41828527 | G | A | 0.001 | 0.002 | 7.00E-01 | 0.016 | 0.003 | 2.90E-08 | TV | 0.011 | 0.008 | 0.001 | 0.011 | 0.031 | 0.025 | 0.005 | 0.021 | -0.069 | 0.035 | 0.021 | 0.022 |
| rs6973656 | 7 | 77422583 | A | G | 0.000 | 0.001 | 8.90E-01 | -0.013 | 0.002 | 1.00E-09 | TV | 0.000 | 0.006 | 0.006 | 0.009 | -0.013 | 0.019 | -0.019 | 0.016 | -0.013 | 0.027 | 0.003 | 0.016 |
| rs973734 | 7 | 89387578 | C | A | 0.004 | 0.002 | 5.60E-02 | 0.017 | 0.003 | 1.50E-08 | TV | 0.013 | 0.009 | 0.015 | 0.011 | 0.032 | 0.025 | 0.015 | 0.021 | 0.017 | 0.036 | 0.023 | 0.022 |
| rs11768481 | 7 | 96629103 | C | A | 0.009 | 0.001 | 9.90E-10 | 0.007 | 0.002 | 4.30E-03 | smoking | 0.005 | 0.007 | 0.000 | 0.010 | 0.026 | 0.021 | -0.006 | 0.018 | -0.010 | 0.030 | 0.030 | 0.018 |
| rs6962772 | 7 | 99081730 | A | G | 0.011 | 0.002 | 7.80E-09 | 0.017 | 0.003 | 1.60E-08 | smoking | 0.010 | 0.008 | 0.002 | 0.011 | 0.019 | 0.024 | 0.003 | 0.021 | 0.037 | 0.035 | 0.020 | 0.021 |
| rs62471080 | 7 | 109128060 | G | C | -0.001 | 0.001 | 4.30E-01 | -0.013 | 0.002 | 3.70E-09 | TV | 0.005 | 0.006 | 0.006 | 0.008 | 0.027 | 0.018 | 0.008 | 0.015 | 0.004 | 0.026 | -0.004 | 0.015 |
| rs2401924 | 7 | 115057862 | G | C | 0.011 | 0.001 | 2.70E-14 | 0.000 | 0.002 | 8.20E-01 | smoking | 0.002 | 0.006 | -0.003 | 0.008 | -0.011 | 0.018 | 0.004 | 0.016 | 0.012 | 0.027 | -0.015 | 0.016 |
| rs10246289 | 7 | 115544763 | A | G | 0.001 | 0.002 | 5.10E-01 | 0.019 | 0.003 | 1.70E-08 | TV | 0.021 | 0.009 | 0.018 | 0.012 | 0.021 | 0.027 | 0.014 | 0.024 | 0.032 | 0.040 | 0.048 | 0.024 |
| rs11763734 | 7 | 126400910 | A | C | -0.004 | 0.001 | 2.20E-03 | -0.012 | 0.002 | 8.20E-09 | TV | 0.003 | 0.006 | -0.003 | 0.008 | 0.011 | 0.018 | 0.009 | 0.015 | -0.006 | 0.026 | 0.024 | 0.015 |
| rs6957896 | 7 | 132309592 | C | T | -0.008 | 0.001 | 4.50E-08 | 0.002 | 0.002 | 2.50E-01 | smoking | 0.004 | 0.006 | 0.007 | 0.009 | -0.001 | 0.019 | -0.008 | 0.016 | 0.015 | 0.027 | -0.005 | 0.016 |
| rs4731925 | 7 | 132664757 | C | T | -0.008 | 0.001 | 2.60E-08 | -0.009 | 0.002 | 4.70E-05 | smoking | 0.008 | 0.007 | 0.006 | 0.009 | -0.007 | 0.019 | 0.011 | 0.017 | 0.021 | 0.028 | 0.012 | 0.017 |
| rs7834121 | 8 | 10770255 | G | T | 0.007 | 0.001 | 1.30E-06 | -0.014 | 0.002 | 1.10E-10 | TV | -0.008 | 0.007 | -0.009 | 0.009 | 0.008 | 0.021 | 0.005 | 0.017 | -0.013 | 0.028 | -0.008 | 0.018 |
| rs11783093 | 8 | 27425349 | C | T | 0.016 | 0.002 | 1.20E-16 | 0.000 | 0.003 | 8.90E-01 | smoking | 0.032 | 0.009 | 0.044 | 0.011 | 0.038 | 0.025 | 0.034 | 0.021 | 0.003 | 0.036 | 0.008 | 0.021 |
| rs1421334 | 8 | 30865733 | A | C | 0.003 | 0.001 | 5.70E-02 | 0.017 | 0.002 | 2.20E-15 | TV | 0.005 | 0.006 | 0.002 | 0.008 | 0.031 | 0.018 | -0.006 | 0.015 | 0.018 | 0.026 | 0.009 | 0.016 |
| rs6996198 | 8 | 65463442 | C | T | 0.001 | 0.002 | 6.10E-01 | -0.016 | 0.003 | 3.50E-08 | TV | -0.001 | 0.008 | -0.001 | 0.011 | -0.002 | 0.024 | -0.002 | 0.020 | 0.065 | 0.036 | -0.036 | 0.020 |
| rs6472942 | 8 | 76827190 | T | C | -0.005 | 0.001 | 1.80E-04 | -0.013 | 0.002 | 1.80E-09 | TV | -0.012 | 0.006 | -0.020 | 0.008 | 0.023 | 0.018 | -0.013 | 0.015 | -0.031 | 0.025 | -0.006 | 0.015 |
| rs11985394 | 8 | 92667521 | T | A | 0.005 | 0.001 | 9.80E-04 | 0.012 | 0.002 | 1.30E-08 | TV | 0.005 | 0.006 | 0.010 | 0.008 | 0.002 | 0.018 | 0.005 | 0.015 | -0.012 | 0.026 | 0.001 | 0.016 |
| rs12541615 | 8 | 118870151 | T | C | -0.004 | 0.002 | 2.00E-02 | -0.018 | 0.003 | 3.90E-10 | TV | 0.029 | 0.008 | 0.037 | 0.011 | 0.035 | 0.023 | 0.048 | 0.020 | 0.039 | 0.034 | 0.004 | 0.020 |
| rs2717559 | 8 | 143882420 | A | G | 0.002 | 0.001 | 2.20E-01 | 0.012 | 0.002 | 1.50E-08 | TV | 0.000 | 0.007 | -0.009 | 0.009 | 0.018 | 0.019 | -0.022 | 0.016 | 0.030 | 0.028 | -0.003 | 0.017 |
| rs2616830 | 9 | 1721385 | G | A | 0.001 | 0.001 | 3.40E-01 | 0.016 | 0.002 | 2.90E-14 | TV | 0.003 | 0.006 | 0.006 | 0.008 | -0.001 | 0.018 | 0.012 | 0.015 | -0.052 | 0.026 | 0.001 | 0.015 |
| rs34864022 | 9 | 22609110 | A | G | -0.008 | 0.003 | 3.80E-03 | -0.026 | 0.004 | 1.40E-09 | TV | 0.008 | 0.013 | 0.002 | 0.017 | 0.001 | 0.038 | 0.043 | 0.032 | -0.046 | 0.054 | 0.011 | 0.033 |
| rs12554512 | 9 | 23352293 | T | C | 0.004 | 0.001 | 8.50E-03 | 0.021 | 0.002 | 3.80E-21 | TV | -0.006 | 0.006 | -0.001 | 0.008 | -0.038 | 0.019 | -0.006 | 0.016 | 0.017 | 0.027 | -0.018 | 0.016 |
| rs2791574 | 9 | 82212094 | T | G | -0.003 | 0.001 | 7.70E-02 | -0.013 | 0.002 | 3.00E-08 | TV | 0.003 | 0.006 | 0.011 | 0.008 | 0.009 | 0.018 | -0.004 | 0.016 | 0.026 | 0.027 | -0.029 | 0.016 |
| rs870151 | 9 | 92230747 | T | A | -0.002 | 0.001 | 9.40E-02 | -0.016 | 0.002 | 8.80E-13 | TV | 0.005 | 0.006 | -0.008 | 0.008 | 0.014 | 0.018 | -0.005 | 0.015 | 0.043 | 0.026 | 0.021 | 0.015 |
| rs2787374 | 9 | 103054951 | T | C | 0.003 | 0.001 | 3.20E-02 | 0.012 | 0.002 | 1.20E-08 | TV | -0.004 | 0.006 | -0.010 | 0.008 | 0.001 | 0.018 | 0.042 | 0.015 | -0.015 | 0.026 | 0.000 | 0.015 |
| rs7043521 | 9 | 124602779 | A | T | 0.005 | 0.001 | 9.70E-04 | 0.014 | 0.002 | 6.50E-11 | TV | 0.003 | 0.006 | 0.003 | 0.008 | -0.019 | 0.018 | 0.016 | 0.015 | 0.037 | 0.026 | -0.019 | 0.015 |
| rs4382592 | 9 | 134870755 | T | G | 0.007 | 0.002 | 1.10E-06 | 0.014 | 0.002 | 6.70E-09 | TV | 0.011 | 0.007 | 0.004 | 0.009 | 0.030 | 0.019 | 0.002 | 0.016 | 0.045 | 0.028 | 0.006 | 0.017 |
| rs2073869 | 9 | 135763816 | C | T | 0.004 | 0.002 | 3.00E-02 | 0.019 | 0.003 | 1.50E-10 | TV | 0.011 | 0.008 | 0.001 | 0.011 | 0.012 | 0.023 | 0.029 | 0.020 | 0.019 | 0.034 | -0.013 | 0.020 |
| rs1623025 | 10 | 11164813 | G | C | -0.002 | 0.001 | 1.20E-01 | -0.012 | 0.002 | 1.40E-08 | TV | -0.009 | 0.006 | -0.012 | 0.008 | -0.011 | 0.018 | -0.013 | 0.015 | -0.044 | 0.026 | 0.000 | 0.015 |
| rs1243182 | 10 | 21916728 | C | T | -0.006 | 0.001 | 1.30E-04 | -0.019 | 0.002 | 2.00E-15 | TV | -0.056 | 0.006 | -0.081 | 0.008 | -0.059 | 0.019 | -0.051 | 0.016 | -0.016 | 0.027 | 0.042 | 0.016 |
| rs2045147 | 10 | 56660437 | A | G | 0.007 | 0.001 | 9.60E-08 | 0.013 | 0.002 | 5.90E-09 | TV | 0.002 | 0.006 | -0.002 | 0.008 | 0.023 | 0.018 | 0.032 | 0.015 | 0.045 | 0.026 | 0.001 | 0.015 |
| rs10994943 | 10 | 63591413 | T | G | 0.008 | 0.001 | 1.50E-08 | 0.013 | 0.002 | 3.10E-09 | TV | 0.011 | 0.006 | 0.010 | 0.008 | -0.009 | 0.018 | 0.015 | 0.015 | 0.028 | 0.026 | 0.032 | 0.015 |
| rs10823968 | 10 | 74738269 | A | T | 0.008 | 0.001 | 2.10E-08 | 0.002 | 0.002 | 3.00E-01 | smoking | 0.003 | 0.006 | -0.009 | 0.008 | 0.004 | 0.018 | 0.012 | 0.015 | 0.000 | 0.026 | 0.000 | 0.016 |
| rs11201422 | 10 | 86924483 | T | C | 0.002 | 0.001 | 2.40E-01 | 0.013 | 0.002 | 3.40E-08 | TV | -0.015 | 0.007 | -0.007 | 0.009 | -0.025 | 0.019 | -0.027 | 0.016 | -0.059 | 0.028 | 0.007 | 0.017 |
| rs17553262 | 10 | 92912773 | A | C | -0.013 | 0.002 | 5.30E-09 | -0.009 | 0.003 | 9.50E-03 | smoking | -0.016 | 0.010 | -0.011 | 0.013 | 0.023 | 0.029 | -0.041 | 0.024 | 0.014 | 0.042 | -0.050 | 0.024 |
| rs10786658 | 10 | 103737193 | A | T | -0.005 | 0.001 | 3.70E-04 | -0.014 | 0.002 | 4.60E-11 | TV | 0.001 | 0.006 | 0.002 | 0.008 | -0.032 | 0.018 | 0.022 | 0.015 | -0.011 | 0.026 | 0.014 | 0.016 |
| rs3896224 | 10 | 106467853 | A | G | 0.010 | 0.001 | 1.10E-11 | 0.007 | 0.002 | 1.50E-03 | smoking | 0.015 | 0.006 | 0.018 | 0.008 | 0.020 | 0.018 | 0.013 | 0.015 | 0.037 | 0.026 | 0.019 | 0.015 |
| rs7089973 | 10 | 116569565 | C | A | -0.003 | 0.001 | 3.50E-02 | -0.013 | 0.002 | 2.40E-09 | TV | -0.005 | 0.006 | -0.008 | 0.009 | 0.025 | 0.019 | -0.007 | 0.016 | -0.045 | 0.027 | 0.002 | 0.016 |
| rs11245482 | 10 | 126733546 | T | C | -0.003 | 0.001 | 2.20E-02 | -0.013 | 0.002 | 2.60E-09 | TV | -0.005 | 0.006 | -0.003 | 0.008 | -0.037 | 0.018 | 0.004 | 0.015 | -0.016 | 0.026 | 0.022 | 0.016 |
| rs17727474 | 10 | 127182959 | C | T | 0.003 | 0.002 | 1.00E-01 | 0.018 | 0.003 | 3.10E-09 | TV | -0.013 | 0.008 | -0.020 | 0.011 | 0.021 | 0.025 | -0.048 | 0.021 | -0.078 | 0.035 | 0.025 | 0.022 |
| rs12289262 | 11 | 12894758 | C | T | 0.000 | 0.002 | 9.30E-01 | -0.014 | 0.002 | 2.00E-08 | TV | 0.002 | 0.007 | 0.008 | 0.009 | -0.064 | 0.021 | -0.013 | 0.018 | -0.001 | 0.030 | 0.012 | 0.018 |
| rs34866095 | 11 | 16377356 | A | G | -0.009 | 0.002 | 1.20E-08 | 0.000 | 0.002 | 8.40E-01 | smoking | 0.001 | 0.006 | 0.001 | 0.009 | 0.024 | 0.019 | -0.003 | 0.016 | 0.000 | 0.028 | 0.025 | 0.016 |
| rs75742406 | 11 | 17070365 | G | A | 0.010 | 0.002 | 1.30E-09 | 0.004 | 0.002 | 1.40E-01 | smoking | -0.004 | 0.007 | -0.010 | 0.009 | -0.013 | 0.021 | -0.017 | 0.018 | -0.001 | 0.030 | -0.008 | 0.018 |
| rs17309874 | 11 | 27667236 | G | A | -0.011 | 0.002 | 9.70E-13 | -0.003 | 0.002 | 1.60E-01 | smoking | 0.009 | 0.007 | 0.014 | 0.009 | -0.016 | 0.021 | 0.028 | 0.018 | -0.016 | 0.030 | -0.005 | 0.018 |
| rs4391802 | 11 | 28674592 | A | G | 0.010 | 0.002 | 1.40E-11 | 0.005 | 0.002 | 2.40E-02 | smoking | -0.004 | 0.007 | -0.006 | 0.009 | -0.009 | 0.020 | 0.009 | 0.017 | 0.022 | 0.029 | -0.005 | 0.017 |
| rs112282219 | 11 | 46632809 | G | A | -0.023 | 0.004 | 3.80E-11 | -0.005 | 0.005 | 3.70E-01 | smoking | -0.034 | 0.016 | -0.053 | 0.021 | -0.038 | 0.046 | 0.017 | 0.041 | -0.010 | 0.069 | -0.030 | 0.041 |
| rs801733 | 11 | 65934549 | A | C | 0.003 | 0.001 | 5.30E-02 | 0.017 | 0.002 | 7.30E-14 | TV | 0.023 | 0.006 | 0.031 | 0.008 | 0.016 | 0.019 | 0.001 | 0.016 | 0.057 | 0.027 | -0.002 | 0.016 |
| rs116175338 | 11 | 72467875 | G | T | -0.006 | 0.002 | 3.00E-04 | -0.014 | 0.003 | 1.40E-08 | TV | -0.017 | 0.007 | -0.028 | 0.009 | 0.007 | 0.021 | -0.004 | 0.018 | -0.016 | 0.030 | -0.025 | 0.018 |
| rs12272012 | 11 | 84861286 | G | C | 0.000 | 0.003 | 9.10E-01 | -0.030 | 0.005 | 1.90E-08 | TV | -0.023 | 0.015 | -0.017 | 0.019 | 0.098 | 0.046 | -0.003 | 0.037 | -0.045 | 0.063 | -0.040 | 0.037 |
| rs11020045 | 11 | 92487907 | A | C | -0.004 | 0.001 | 3.20E-03 | -0.013 | 0.002 | 1.60E-08 | TV | -0.009 | 0.006 | -0.003 | 0.008 | 0.000 | 0.019 | -0.017 | 0.016 | -0.019 | 0.027 | -0.022 | 0.016 |
| rs17207890 | 11 | 95490754 | G | A | 0.005 | 0.001 | 1.80E-03 | 0.016 | 0.002 | 6.70E-12 | TV | 0.017 | 0.006 | 0.033 | 0.008 | 0.000 | 0.019 | 0.039 | 0.016 | -0.011 | 0.027 | 0.019 | 0.016 |
| rs11218575 | 11 | 122154576 | C | T | 0.002 | 0.001 | 1.50E-01 | 0.015 | 0.002 | 2.10E-12 | TV | 0.009 | 0.006 | 0.000 | 0.009 | 0.024 | 0.019 | 0.001 | 0.016 | 0.063 | 0.027 | 0.008 | 0.016 |
| rs4937842 | 11 | 133734542 | G | C | 0.001 | 0.001 | 4.50E-01 | -0.013 | 0.002 | 1.40E-08 | TV | 0.009 | 0.006 | 0.012 | 0.008 | -0.005 | 0.018 | 0.017 | 0.016 | 0.020 | 0.027 | 0.010 | 0.016 |
| rs10772643 | 12 | 13415288 | C | T | 0.005 | 0.002 | 2.90E-02 | 0.025 | 0.003 | 1.30E-12 | TV | 0.008 | 0.010 | 0.022 | 0.013 | -0.012 | 0.030 | -0.005 | 0.025 | -0.044 | 0.044 | -0.017 | 0.026 |
| rs10771746 | 12 | 30791864 | C | T | 0.001 | 0.002 | 5.10E-01 | -0.014 | 0.002 | 2.50E-09 | TV | -0.024 | 0.007 | -0.015 | 0.009 | -0.037 | 0.019 | -0.053 | 0.016 | -0.018 | 0.028 | 0.013 | 0.017 |
| rs74086911 | 12 | 50015942 | G | A | 0.015 | 0.003 | 2.10E-08 | 0.008 | 0.004 | 4.30E-02 | smoking | 0.012 | 0.012 | 0.004 | 0.015 | 0.024 | 0.034 | 0.024 | 0.029 | 0.026 | 0.049 | 0.046 | 0.030 |
| rs10876864 | 12 | 56401085 | G | A | -0.007 | 0.001 | 1.50E-07 | -0.013 | 0.002 | 1.00E-09 | TV | 0.004 | 0.006 | 0.008 | 0.008 | 0.040 | 0.018 | -0.011 | 0.015 | 0.008 | 0.026 | -0.024 | 0.015 |
| rs8756 | 12 | 66359752 | C | A | -0.004 | 0.001 | 1.30E-03 | -0.013 | 0.002 | 5.30E-10 | TV | -0.003 | 0.006 | 0.004 | 0.008 | 0.009 | 0.018 | -0.007 | 0.015 | 0.019 | 0.025 | -0.023 | 0.015 |
| rs12831617 | 12 | 84758368 | C | T | -0.009 | 0.002 | 1.90E-08 | -0.003 | 0.003 | 1.80E-01 | smoking | -0.028 | 0.007 | -0.020 | 0.009 | -0.040 | 0.021 | -0.030 | 0.018 | -0.002 | 0.030 | -0.041 | 0.018 |
| rs2164744 | 12 | 1.1E+08 | T | C | -0.004 | 0.001 | 6.50E-03 | -0.013 | 0.002 | 1.10E-08 | TV | 0.019 | 0.006 | 0.009 | 0.008 | 0.024 | 0.018 | 0.042 | 0.016 | 0.072 | 0.026 | 0.011 | 0.016 |
| rs2173650 | 12 | 1.18E+08 | G | T | 0.006 | 0.002 | 1.40E-03 | 0.018 | 0.003 | 4.60E-09 | TV | 0.016 | 0.009 | 0.014 | 0.014 | 0.012 | 0.028 | 0.009 | 0.025 | 0.036 | 0.042 | 0.043 | 0.026 |
| rs9563168 | 13 | 54247827 | G | A | 0.003 | 0.002 | 1.10E-01 | 0.018 | 0.003 | 4.30E-11 | TV | 0.003 | 0.008 | 0.011 | 0.010 | 0.010 | 0.022 | 0.003 | 0.019 | -0.004 | 0.032 | -0.013 | 0.019 |
| rs9569734 | 13 | 58319476 | A | G | 0.002 | 0.002 | 2.10E-01 | 0.019 | 0.003 | 3.40E-10 | TV | 0.002 | 0.008 | 0.010 | 0.011 | -0.034 | 0.024 | 0.014 | 0.021 | 0.002 | 0.036 | 0.023 | 0.021 |
| rs6562474 | 13 | 67332812 | C | G | 0.008 | 0.001 | 1.00E-08 | -0.001 | 0.002 | 7.90E-01 | smoking | -0.012 | 0.006 | -0.005 | 0.008 | -0.033 | 0.018 | -0.034 | 0.016 | -0.069 | 0.027 | 0.007 | 0.016 |
| rs56858768 | 13 | 86511730 | G | A | -0.001 | 0.002 | 3.90E-01 | -0.015 | 0.002 | 3.40E-10 | TV | 0.012 | 0.007 | 0.008 | 0.009 | 0.012 | 0.020 | 0.017 | 0.017 | -0.002 | 0.029 | 0.004 | 0.017 |
| rs7333559 | 13 | 100546450 | G | A | 0.011 | 0.002 | 3.20E-10 | 0.005 | 0.003 | 4.30E-02 | smoking | 0.016 | 0.008 | 0.015 | 0.010 | 0.001 | 0.023 | 0.000 | 0.020 | 0.002 | 0.033 | 0.030 | 0.020 |
| rs7991062 | 13 | 100713194 | C | G | -0.007 | 0.001 | 4.00E-07 | -0.018 | 0.002 | 8.80E-15 | TV | -0.004 | 0.006 | -0.012 | 0.008 | -0.001 | 0.019 | -0.001 | 0.016 | -0.036 | 0.027 | -0.005 | 0.016 |
| rs405797 | 14 | 26972834 | T | A | -0.004 | 0.002 | 8.00E-03 | -0.015 | 0.003 | 2.80E-09 | TV | 0.015 | 0.008 | 0.013 | 0.010 | 0.017 | 0.022 | 0.036 | 0.019 | 0.027 | 0.032 | 0.004 | 0.019 |
| rs1022785 | 14 | 29571653 | G | A | 0.002 | 0.002 | 4.30E-01 | 0.018 | 0.003 | 7.10E-09 | TV | 0.008 | 0.009 | 0.009 | 0.011 | 0.007 | 0.025 | -0.014 | 0.022 | -0.026 | 0.037 | 0.032 | 0.022 |
| rs860326 | 14 | 57342912 | C | T | 0.008 | 0.001 | 2.70E-09 | 0.010 | 0.002 | 8.60E-06 | smoking | 0.001 | 0.006 | -0.003 | 0.008 | -0.024 | 0.018 | -0.007 | 0.016 | -0.026 | 0.027 | 0.036 | 0.016 |
| rs7155595 | 14 | 77502546 | A | C | -0.009 | 0.001 | 2.50E-09 | -0.008 | 0.002 | 1.10E-03 | smoking | -0.012 | 0.006 | -0.002 | 0.008 | 0.001 | 0.019 | -0.029 | 0.016 | -0.035 | 0.027 | 0.022 | 0.016 |
| rs10145592 | 14 | 94287215 | C | G | 0.001 | 0.001 | 6.20E-01 | -0.015 | 0.002 | 1.80E-11 | TV | 0.003 | 0.006 | -0.015 | 0.009 | -0.009 | 0.019 | 0.006 | 0.016 | 0.030 | 0.027 | 0.020 | 0.016 |
| rs7157001 | 14 | 99749484 | A | G | -0.002 | 0.002 | 2.90E-01 | -0.014 | 0.002 | 2.30E-08 | TV | -0.003 | 0.008 | -0.007 | 0.011 | -0.020 | 0.024 | 0.027 | 0.021 | 0.018 | 0.035 | 0.013 | 0.021 |
| rs3742365 | 14 | 104198251 | T | C | -0.011 | 0.001 | 2.50E-14 | 0.003 | 0.002 | 1.90E-01 | smoking | 0.009 | 0.006 | 0.007 | 0.008 | 0.015 | 0.018 | 0.023 | 0.015 | 0.037 | 0.026 | 0.005 | 0.016 |
| rs8043253 | 15 | 41503730 | C | T | 0.003 | 0.001 | 7.20E-02 | -0.012 | 0.002 | 3.40E-08 | TV | -0.004 | 0.006 | -0.018 | 0.008 | 0.016 | 0.018 | 0.012 | 0.015 | -0.004 | 0.026 | -0.008 | 0.015 |
| rs2460 | 15 | 53073084 | G | A | -0.003 | 0.002 | 4.00E-02 | -0.015 | 0.002 | 5.00E-10 | TV | 0.000 | 0.007 | -0.006 | 0.010 | -0.001 | 0.021 | -0.007 | 0.018 | 0.024 | 0.031 | 0.024 | 0.019 |
| rs4775373 | 15 | 61476936 | T | C | 0.001 | 0.001 | 3.60E-01 | 0.013 | 0.002 | 4.70E-09 | TV | 0.002 | 0.006 | 0.000 | 0.008 | -0.001 | 0.019 | 0.004 | 0.016 | 0.034 | 0.027 | -0.026 | 0.016 |
| rs28485305 | 15 | 74044197 | C | T | 0.008 | 0.001 | 2.60E-08 | 0.002 | 0.002 | 3.00E-01 | smoking | 0.005 | 0.006 | -0.002 | 0.008 | -0.002 | 0.018 | 0.017 | 0.015 | -0.021 | 0.026 | 0.027 | 0.016 |
| rs61331678 | 15 | 77995949 | G | C | 0.003 | 0.001 | 4.10E-02 | 0.015 | 0.002 | 6.10E-12 | TV | 0.002 | 0.006 | 0.002 | 0.008 | 0.027 | 0.018 | -0.034 | 0.015 | -0.027 | 0.026 | 0.017 | 0.016 |
| rs8042134 | 15 | 97514404 | T | G | -0.010 | 0.001 | 1.30E-12 | 0.001 | 0.002 | 7.60E-01 | smoking | 0.006 | 0.007 | 0.005 | 0.009 | 0.017 | 0.019 | 0.011 | 0.016 | -0.008 | 0.028 | 0.013 | 0.017 |
| rs6598539 | 15 | 99204483 | T | C | -0.008 | 0.001 | 4.50E-09 | -0.006 | 0.002 | 5.10E-03 | smoking | -0.001 | 0.006 | 0.002 | 0.008 | -0.003 | 0.018 | 0.014 | 0.015 | -0.002 | 0.026 | 0.004 | 0.016 |
| rs11861214 | 16 | 746611 | G | T | 0.009 | 0.002 | 2.00E-08 | 0.008 | 0.003 | 4.30E-03 | smoking | -0.003 | 0.007 | 0.001 | 0.009 | -0.007 | 0.021 | 0.009 | 0.018 | -0.051 | 0.030 | 0.010 | 0.018 |
| rs35574015 | 16 | 15146061 | T | C | -0.003 | 0.002 | 3.40E-02 | -0.013 | 0.002 | 3.00E-08 | TV | -0.007 | 0.007 | 0.000 | 0.009 | -0.023 | 0.020 | -0.008 | 0.017 | 0.026 | 0.030 | -0.015 | 0.018 |
| rs7189927 | 16 | 28913787 | T | C | 0.003 | 0.001 | 2.40E-02 | 0.015 | 0.002 | 3.40E-11 | TV | 0.001 | 0.006 | 0.006 | 0.008 | -0.010 | 0.019 | -0.001 | 0.016 | 0.014 | 0.027 | -0.026 | 0.016 |
| rs749671 | 16 | 31088347 | G | A | 0.007 | 0.001 | 4.90E-06 | 0.016 | 0.002 | 2.70E-12 | TV | -0.017 | 0.006 | -0.014 | 0.008 | -0.010 | 0.018 | -0.010 | 0.015 | -0.002 | 0.026 | -0.015 | 0.016 |
| rs7184800 | 16 | 53509131 | G | A | 0.004 | 0.002 | 1.60E-02 | 0.017 | 0.002 | 8.20E-13 | TV | -0.005 | 0.006 | -0.010 | 0.008 | -0.014 | 0.019 | 0.005 | 0.016 | 0.026 | 0.028 | -0.011 | 0.016 |
| rs889398 | 16 | 69556715 | C | T | 0.009 | 0.001 | 6.30E-11 | 0.002 | 0.002 | 4.30E-01 | smoking | 0.015 | 0.006 | 0.010 | 0.008 | 0.028 | 0.018 | -0.006 | 0.015 | 0.017 | 0.026 | 0.031 | 0.015 |
| rs60952428 | 16 | 75640521 | T | C | 0.013 | 0.002 | 3.00E-08 | 0.010 | 0.004 | 1.10E-02 | smoking | 0.001 | 0.011 | -0.002 | 0.014 | -0.024 | 0.031 | 0.033 | 0.027 | 0.026 | 0.046 | 0.008 | 0.027 |
| rs1050847 | 16 | 87443734 | C | T | 0.008 | 0.001 | 1.40E-08 | 0.005 | 0.002 | 3.50E-02 | smoking | -0.007 | 0.006 | -0.005 | 0.008 | 0.017 | 0.018 | -0.011 | 0.016 | 0.012 | 0.026 | -0.025 | 0.016 |
| rs369230 | 16 | 89645437 | G | T | -0.009 | 0.002 | 1.80E-09 | -0.006 | 0.002 | 9.40E-03 | smoking | -0.021 | 0.007 | -0.018 | 0.009 | -0.040 | 0.020 | -0.017 | 0.017 | -0.034 | 0.029 | -0.028 | 0.017 |
| rs2447098 | 17 | 2277720 | C | A | -0.004 | 0.001 | 1.70E-03 | -0.015 | 0.002 | 6.80E-12 | TV | 0.008 | 0.006 | 0.009 | 0.009 | 0.012 | 0.019 | 0.036 | 0.016 | -0.005 | 0.027 | 0.020 | 0.016 |
| rs11654952 | 17 | 4813799 | T | G | -0.001 | 0.002 | 7.60E-01 | -0.017 | 0.003 | 4.30E-08 | TV | 0.007 | 0.009 | 0.009 | 0.012 | 0.031 | 0.026 | 0.037 | 0.022 | 0.017 | 0.038 | -0.027 | 0.022 |
| rs8614 | 17 | 27588806 | C | A | -0.011 | 0.002 | 1.80E-10 | 0.001 | 0.003 | 6.90E-01 | smoking | -0.016 | 0.008 | -0.016 | 0.011 | -0.067 | 0.024 | -0.013 | 0.021 | 0.013 | 0.035 | 0.003 | 0.021 |
| rs732083 | 17 | 37834367 | G | A | 0.008 | 0.001 | 1.50E-08 | 0.007 | 0.002 | 3.10E-03 | smoking | -0.004 | 0.006 | -0.011 | 0.008 | 0.006 | 0.019 | -0.021 | 0.016 | 0.021 | 0.027 | 0.031 | 0.016 |
| rs67596067 | 17 | 50333733 | G | A | -0.009 | 0.001 | 1.20E-09 | -0.009 | 0.002 | 6.90E-05 | smoking | 0.006 | 0.007 | 0.001 | 0.009 | 0.006 | 0.019 | 0.013 | 0.016 | -0.014 | 0.027 | 0.012 | 0.016 |
| rs9902312 | 17 | 65070304 | T | C | 0.000 | 0.001 | 8.30E-01 | 0.015 | 0.002 | 4.50E-11 | TV | -0.009 | 0.006 | -0.024 | 0.008 | -0.026 | 0.019 | 0.016 | 0.016 | -0.021 | 0.027 | 0.009 | 0.016 |
| rs11657730 | 17 | 79372489 | C | T | 0.003 | 0.001 | 1.70E-02 | 0.013 | 0.002 | 1.10E-08 | TV | 0.021 | 0.007 | 0.021 | 0.009 | 0.007 | 0.019 | 0.024 | 0.017 | 0.062 | 0.029 | 0.023 | 0.017 |
| rs303753 | 18 | 21074922 | G | A | 0.000 | 0.001 | 9.20E-01 | -0.014 | 0.002 | 2.70E-10 | TV | -0.004 | 0.006 | -0.010 | 0.009 | -0.033 | 0.019 | 0.005 | 0.016 | 0.026 | 0.028 | -0.051 | 0.016 |
| rs9964724 | 18 | 35159124 | C | T | 0.007 | 0.001 | 6.50E-07 | 0.018 | 0.002 | 3.30E-14 | TV | 0.007 | 0.007 | 0.002 | 0.009 | 0.029 | 0.019 | 0.007 | 0.017 | -0.043 | 0.029 | 0.009 | 0.017 |
| rs1156541 | 18 | 39952989 | C | T | 0.006 | 0.002 | 3.40E-04 | 0.014 | 0.003 | 2.80E-08 | TV | -0.018 | 0.007 | -0.012 | 0.009 | -0.043 | 0.021 | -0.029 | 0.018 | -0.010 | 0.030 | -0.044 | 0.018 |
| rs62098013 | 18 | 50863861 | G | A | -0.009 | 0.001 | 4.10E-09 | -0.006 | 0.002 | 9.10E-03 | smoking | -0.009 | 0.006 | -0.008 | 0.008 | -0.023 | 0.018 | -0.002 | 0.016 | -0.056 | 0.027 | 0.007 | 0.016 |
| rs17512836 | 18 | 53194961 | T | C | 0.002 | 0.004 | 6.90E-01 | 0.042 | 0.007 | 7.00E-10 | TV | -0.008 | 0.019 | -0.034 | 0.025 | 0.048 | 0.056 | -0.022 | 0.047 | -0.007 | 0.079 | 0.035 | 0.049 |
| rs71367545 | 18 | 77576337 | G | A | -0.010 | 0.002 | 1.40E-09 | -0.007 | 0.003 | 1.00E-02 | smoking | 0.006 | 0.008 | 0.022 | 0.011 | -0.038 | 0.024 | 0.001 | 0.021 | 0.005 | 0.035 | 0.011 | 0.021 |
| rs7248205 | 19 | 10770305 | C | T | -0.002 | 0.001 | 1.40E-01 | 0.014 | 0.002 | 3.40E-10 | TV | 0.012 | 0.006 | 0.012 | 0.008 | 0.030 | 0.019 | 0.021 | 0.016 | -0.012 | 0.027 | -0.022 | 0.016 |
| rs35343344 | 19 | 18471610 | C | A | 0.009 | 0.002 | 8.80E-09 | 0.005 | 0.002 | 7.00E-02 | smoking | -0.004 | 0.007 | 0.005 | 0.009 | 0.007 | 0.021 | -0.016 | 0.018 | 0.021 | 0.030 | -0.012 | 0.018 |
| rs111901094 | 19 | 19513570 | G | T | -0.005 | 0.002 | 1.00E-02 | -0.017 | 0.003 | 2.00E-09 | TV | -0.032 | 0.009 | -0.038 | 0.012 | -0.011 | 0.027 | -0.024 | 0.023 | -0.092 | 0.039 | -0.064 | 0.023 |
| rs6131281 | 20 | 11891724 | C | T | -0.001 | 0.001 | 7.20E-01 | 0.016 | 0.002 | 3.10E-13 | TV | 0.002 | 0.007 | -0.006 | 0.009 | -0.007 | 0.019 | 0.008 | 0.016 | 0.016 | 0.027 | 0.010 | 0.016 |
| rs4814873 | 20 | 19616429 | C | T | 0.010 | 0.002 | 2.90E-09 | 0.009 | 0.003 | 4.80E-04 | smoking | 0.007 | 0.007 | -0.006 | 0.009 | 0.023 | 0.021 | 0.007 | 0.018 | -0.021 | 0.030 | 0.027 | 0.018 |
| rs6141814 | 20 | 31368960 | C | A | -0.007 | 0.001 | 1.50E-06 | -0.014 | 0.002 | 1.30E-09 | TV | 0.004 | 0.006 | 0.005 | 0.008 | 0.029 | 0.018 | 0.000 | 0.015 | 0.006 | 0.026 | -0.006 | 0.016 |
| rs55700114 | 20 | 43717080 | G | A | -0.003 | 0.002 | 3.00E-02 | -0.014 | 0.002 | 2.10E-09 | TV | -0.002 | 0.006 | -0.007 | 0.009 | 0.035 | 0.019 | -0.019 | 0.016 | 0.018 | 0.027 | -0.008 | 0.016 |
| rs56103247 | 20 | 62483184 | C | T | 0.017 | 0.003 | 4.60E-08 | 0.030 | 0.005 | 3.80E-10 | TV | -0.006 | 0.015 | -0.007 | 0.020 | 0.020 | 0.045 | -0.013 | 0.037 | -0.068 | 0.061 | 0.014 | 0.038 |
| rs10427502 | 21 | 40654840 | G | A | -0.005 | 0.001 | 1.50E-04 | 0.014 | 0.002 | 8.60E-10 | TV | -0.012 | 0.006 | -0.013 | 0.008 | -0.003 | 0.018 | -0.021 | 0.016 | -0.067 | 0.027 | -0.014 | 0.016 |
| rs2838834 | 21 | 46665208 | C | T | -0.009 | 0.002 | 6.30E-10 | -0.001 | 0.002 | 5.30E-01 | smoking | -0.003 | 0.007 | 0.002 | 0.009 | -0.051 | 0.019 | 0.018 | 0.017 | 0.011 | 0.028 | 0.014 | 0.017 |
| rs2092829 | 22 | 31780514 | G | A | 0.003 | 0.002 | 6.60E-02 | 0.014 | 0.002 | 1.30E-08 | TV | -0.002 | 0.007 | -0.010 | 0.009 | -0.005 | 0.019 | -0.014 | 0.017 | -0.001 | 0.028 | 0.010 | 0.017 |
| rs202645 | 22 | 41798520 | A | G | -0.010 | 0.002 | 3.90E-09 | 0.005 | 0.003 | 6.50E-02 | smoking | -0.002 | 0.007 | 0.014 | 0.010 | 0.031 | 0.022 | -0.039 | 0.019 | -0.006 | 0.032 | -0.030 | 0.019 |

| **Table S25. Beta estimates for the SNPs included in the multivariable MR of smoking, leisure television use, and colorectal cancer risk** | | | | | | | | | | | | | | | | | | | | | | |
| --- | --- | --- | --- | --- | --- | --- | --- | --- | --- | --- | --- | --- | --- | --- | --- | --- | --- | --- | --- | --- | --- | --- |
| SNP | CHR | POS | EA | OA | EAF | beta_smk | se_smk | pval_smk | beta_tv | se_tv | pval_tv | Phenotype | beta_crc | se_crc | beta_crc_men | se_crc_men | beta_crc_women | se_crc_women | beta_co | se_co | beta_re | se_re |
| rs1193237 | 1 | 7526486 | G | C | 0.439176 | -0.008 | 0.001 | 2.80E-08 | -0.009 | 0.002 | 1.80E-05 | smoking | 0.004 | 0.010 | -0.004 | 0.014 | 0.009 | 0.014 | 0.007 | 0.012 | 0.015 | 0.015 |
| rs4949465 | 1 | 32178489 | T | C | 0.869552 | -0.012 | 0.002 | 1.70E-08 | -0.012 | 0.003 | 3.00E-04 | smoking | 0.015 | 0.014 | 0.044 | 0.020 | -0.011 | 0.020 | 0.021 | 0.017 | 0.032 | 0.022 |
| rs11810109 | 1 | 44186812 | A | T | 0.701 | 0.010 | 0.002 | 5.10E-11 | 0.016 | 0.002 | 5.40E-12 | TV | 0.011 | 0.011 | 0.000 | 0.015 | 0.022 | 0.015 | 0.004 | 0.013 | 0.016 | 0.017 |
| rs12725114 | 1 | 62432641 | G | A | 0.802 | -0.001 | 0.002 | 4.70E-01 | 0.015 | 0.003 | 1.60E-08 | TV | -0.016 | 0.012 | -0.023 | 0.017 | -0.007 | 0.017 | 0.003 | 0.014 | -0.060 | 0.018 |
| rs984409 | 1 | 67020440 | G | A | 0.363 | -0.002 | 0.001 | 1.80E-01 | -0.015 | 0.002 | 4.10E-11 | TV | -0.019 | 0.010 | -0.010 | 0.015 | -0.028 | 0.015 | -0.011 | 0.012 | -0.030 | 0.016 |
| rs71658797 | 1 | 77967507 | T | A | 0.877 | -0.007 | 0.002 | 5.80E-04 | -0.020 | 0.003 | 6.30E-10 | TV | -0.005 | 0.016 | -0.002 | 0.023 | -0.009 | 0.023 | -0.009 | 0.019 | -0.009 | 0.025 |
| rs114328297 | 1 | 91190854 | T | G | 0.778 | 0.010 | 0.002 | 1.30E-08 | 0.014 | 0.003 | 4.40E-08 | TV | 0.002 | 0.012 | 0.018 | 0.017 | -0.018 | 0.018 | 0.016 | 0.015 | -0.056 | 0.019 |
| rs17379561 | 1 | 98340139 | A | T | 0.856 | -0.006 | 0.002 | 5.30E-03 | -0.025 | 0.003 | 1.10E-16 | TV | 0.016 | 0.014 | 0.004 | 0.020 | 0.024 | 0.020 | 0.016 | 0.017 | 0.016 | 0.022 |
| rs55909997 | 1 | 107581039 | G | A | 0.65 | -0.008 | 0.001 | 1.10E-07 | -0.014 | 0.002 | 2.40E-09 | TV | -0.027 | 0.010 | -0.037 | 0.014 | -0.017 | 0.014 | -0.011 | 0.012 | -0.033 | 0.016 |
| rs1278847 | 1 | 110037483 | C | A | 0.694 | 0.002 | 0.002 | 2.80E-01 | 0.016 | 0.002 | 1.20E-11 | TV | -0.006 | 0.011 | 0.008 | 0.015 | -0.018 | 0.015 | -0.006 | 0.013 | -0.004 | 0.017 |
| rs4845364 | 1 | 154141908 | A | G | 0.495 | 0.003 | 0.001 | 1.60E-02 | -0.015 | 0.002 | 1.30E-12 | TV | -0.006 | 0.010 | -0.014 | 0.014 | 0.001 | 0.014 | -0.008 | 0.012 | -0.013 | 0.015 |
| rs6673341 | 1 | 184655649 | T | G | 0.465 | -0.003 | 0.001 | 3.30E-02 | -0.015 | 0.002 | 2.20E-11 | TV | 0.000 | 0.010 | -0.012 | 0.014 | 0.012 | 0.014 | 0.002 | 0.012 | -0.014 | 0.015 |
| rs10737620 | 1 | 193057971 | T | A | 0.274 | 0.001 | 0.002 | 6.20E-01 | 0.014 | 0.002 | 2.60E-09 | TV | -0.010 | 0.011 | -0.008 | 0.015 | -0.011 | 0.015 | 0.000 | 0.013 | -0.024 | 0.017 |
| rs2867112 | 2 | 651349 | T | G | 0.834596 | 0.015 | 0.002 | 4.80E-15 | 0.005 | 0.003 | 9.10E-02 | smoking | 0.012 | 0.013 | 0.029 | 0.018 | -0.002 | 0.018 | 0.018 | 0.016 | 0.019 | 0.020 |
| rs6741228 | 2 | 22548774 | T | C | 0.433309 | 0.008 | 0.001 | 1.60E-08 | 0.004 | 0.002 | 9.70E-02 | smoking | 0.002 | 0.010 | 0.008 | 0.014 | -0.006 | 0.014 | 0.003 | 0.012 | 0.005 | 0.015 |
| rs72781699 | 2 | 24277709 | G | A | 0.797 | -0.003 | 0.002 | 4.40E-02 | -0.019 | 0.003 | 3.00E-12 | TV | 0.009 | 0.012 | 0.008 | 0.017 | 0.008 | 0.017 | -0.012 | 0.014 | 0.042 | 0.019 |
| rs12105701 | 2 | 41737200 | C | T | 0.396 | -0.001 | 0.001 | 6.50E-01 | -0.013 | 0.002 | 5.40E-09 | TV | -0.009 | 0.010 | 0.013 | 0.014 | -0.030 | 0.014 | -0.007 | 0.012 | -0.017 | 0.015 |
| rs7564130 | 2 | 50606642 | T | C | 0.641 | -0.005 | 0.001 | 5.10E-04 | -0.015 | 0.002 | 2.80E-11 | TV | -0.021 | 0.010 | -0.019 | 0.014 | -0.022 | 0.014 | -0.032 | 0.012 | -0.002 | 0.016 |
| rs13016665 | 2 | 57995348 | C | A | 0.576725 | -0.008 | 0.001 | 1.80E-09 | -0.004 | 0.002 | 9.90E-02 | smoking | -0.010 | 0.010 | -0.033 | 0.014 | 0.014 | 0.014 | -0.012 | 0.012 | -0.023 | 0.015 |
| rs10189857 | 2 | 60713235 | A | G | 0.568 | -0.003 | 0.001 | 1.80E-02 | -0.020 | 0.002 | 6.20E-21 | TV | -0.022 | 0.010 | -0.022 | 0.014 | -0.021 | 0.014 | -0.020 | 0.012 | -0.019 | 0.015 |
| rs62641636 | 2 | 68425427 | A | G | 0.692 | 0.000 | 0.002 | 8.90E-01 | 0.014 | 0.002 | 7.00E-10 | TV | 0.010 | 0.010 | 0.000 | 0.015 | 0.018 | 0.015 | 0.022 | 0.012 | 0.015 | 0.016 |
| rs11689199 | 2 | 100820421 | A | G | 0.598 | 0.003 | 0.001 | 1.60E-02 | 0.018 | 0.002 | 5.50E-17 | TV | 0.025 | 0.010 | 0.039 | 0.014 | 0.012 | 0.014 | 0.020 | 0.012 | 0.034 | 0.016 |
| rs2678670 | 2 | 104469564 | A | T | 0.485528 | 0.009 | 0.001 | 3.10E-10 | 0.015 | 0.002 | 1.60E-11 | smoking | 0.004 | 0.010 | 0.012 | 0.014 | -0.005 | 0.014 | 0.011 | 0.012 | 0.005 | 0.015 |
| rs1451533 | 2 | 105466005 | G | A | 0.725 | -0.001 | 0.002 | 7.30E-01 | -0.016 | 0.002 | 1.10E-10 | TV | 0.009 | 0.011 | 0.009 | 0.015 | 0.011 | 0.015 | 0.002 | 0.013 | 0.013 | 0.017 |
| rs12476388 | 2 | 116330983 | C | T | 0.709 | 0.001 | 0.002 | 4.30E-01 | 0.013 | 0.002 | 2.10E-08 | TV | -0.002 | 0.010 | -0.005 | 0.015 | 0.000 | 0.015 | -0.012 | 0.013 | 0.001 | 0.017 |
| rs374722 | 2 | 147839830 | G | A | 0.15 | 0.000 | 0.002 | 8.80E-01 | 0.024 | 0.003 | 5.50E-16 | TV | 0.026 | 0.013 | 0.002 | 0.019 | 0.049 | 0.019 | 0.038 | 0.016 | 0.017 | 0.021 |
| rs3754970 | 2 | 162091836 | T | C | 0.496 | -0.006 | 0.001 | 1.80E-05 | -0.015 | 0.002 | 4.80E-12 | TV | 0.006 | 0.010 | 0.003 | 0.014 | 0.007 | 0.014 | 0.011 | 0.012 | -0.011 | 0.015 |
| rs2060199 | 2 | 166196681 | T | A | 0.446 | -0.001 | 0.001 | 3.30E-01 | 0.012 | 0.002 | 2.40E-08 | TV | 0.016 | 0.010 | 0.039 | 0.014 | -0.002 | 0.014 | 0.012 | 0.012 | 0.028 | 0.015 |
| rs13009008 | 2 | 174043233 | A | G | 0.327713 | 0.009 | 0.001 | 4.60E-09 | 0.010 | 0.002 | 1.70E-05 | smoking | 0.012 | 0.010 | 0.026 | 0.014 | 0.005 | 0.014 | 0.015 | 0.012 | 0.013 | 0.016 |
| rs4473348 | 2 | 182073742 | A | T | 0.249978 | -0.010 | 0.002 | 6.40E-11 | -0.012 | 0.002 | 1.20E-06 | smoking | -0.021 | 0.011 | -0.003 | 0.016 | -0.038 | 0.016 | -0.038 | 0.014 | 0.009 | 0.018 |
| rs4577309 | 2 | 191288833 | A | G | 0.469 | 0.001 | 0.001 | 3.50E-01 | 0.016 | 0.002 | 1.60E-13 | TV | 0.001 | 0.010 | 0.015 | 0.014 | -0.012 | 0.014 | 0.004 | 0.012 | 0.009 | 0.015 |
| rs34224261 | 2 | 193855452 | G | C | 0.469 | 0.001 | 0.001 | 6.00E-01 | -0.018 | 0.002 | 4.60E-16 | TV | 0.001 | 0.010 | -0.002 | 0.014 | 0.005 | 0.014 | -0.002 | 0.012 | 0.000 | 0.015 |
| rs4675246 | 2 | 202864487 | G | T | 0.797 | -0.006 | 0.002 | 2.70E-04 | -0.015 | 0.003 | 2.90E-08 | TV | 0.037 | 0.013 | 0.043 | 0.018 | 0.031 | 0.018 | 0.028 | 0.015 | 0.047 | 0.020 |
| rs13029509 | 2 | 215374209 | G | A | 0.52 | -0.002 | 0.001 | 1.60E-01 | -0.018 | 0.002 | 2.00E-17 | TV | -0.005 | 0.010 | -0.007 | 0.014 | -0.004 | 0.014 | -0.001 | 0.012 | -0.011 | 0.015 |
| rs10932837 | 2 | 221044785 | C | T | 0.494 | 0.000 | 0.001 | 7.80E-01 | -0.013 | 0.002 | 1.30E-09 | TV | -0.003 | 0.010 | 0.013 | 0.014 | -0.020 | 0.014 | -0.005 | 0.012 | -0.016 | 0.015 |
| rs4973576 | 2 | 233806771 | C | A | 0.298 | 0.002 | 0.002 | 2.20E-01 | -0.015 | 0.002 | 1.00E-09 | TV | 0.013 | 0.011 | -0.005 | 0.015 | 0.034 | 0.015 | 0.013 | 0.013 | -0.001 | 0.017 |
| rs138256022 | 3 | 11603090 | C | T | 0.959 | -0.006 | 0.004 | 1.00E-01 | -0.031 | 0.006 | 2.90E-08 | TV | 0.043 | 0.031 | 0.025 | 0.045 | 0.065 | 0.044 | 0.031 | 0.037 | 0.046 | 0.049 |
| rs9834970 | 3 | 36856030 | T | C | 0.501 | 0.002 | 0.001 | 7.10E-02 | 0.013 | 0.002 | 3.30E-09 | TV | 0.013 | 0.010 | 0.002 | 0.014 | 0.024 | 0.014 | 0.010 | 0.012 | 0.017 | 0.015 |
| rs3796386 | 3 | 49899795 | G | A | 0.572 | -0.006 | 0.001 | 3.80E-05 | -0.026 | 0.002 | 3.20E-33 | TV | -0.015 | 0.010 | -0.014 | 0.014 | -0.018 | 0.014 | -0.012 | 0.012 | -0.040 | 0.015 |
| rs11130793 | 3 | 60884659 | C | T | 0.602 | 0.002 | 0.001 | 1.30E-01 | 0.013 | 0.002 | 5.00E-09 | TV | 0.008 | 0.010 | 0.018 | 0.014 | -0.003 | 0.014 | -0.004 | 0.012 | 0.011 | 0.016 |
| rs11714337 | 3 | 71582521 | G | A | 0.569 | 0.005 | 0.001 | 7.60E-04 | 0.014 | 0.002 | 4.70E-11 | TV | -0.021 | 0.010 | -0.010 | 0.014 | -0.029 | 0.014 | -0.029 | 0.012 | -0.011 | 0.015 |
| rs775758 | 3 | 77582005 | A | T | 0.432859 | 0.008 | 0.001 | 1.10E-08 | 0.003 | 0.002 | 1.90E-01 | smoking | -0.010 | 0.010 | -0.014 | 0.014 | -0.006 | 0.014 | -0.011 | 0.012 | 0.003 | 0.015 |
| rs421983 | 3 | 84892866 | T | C | 0.519028 | 0.009 | 0.001 | 3.30E-10 | 0.005 | 0.002 | 2.40E-02 | smoking | -0.010 | 0.010 | -0.017 | 0.014 | -0.005 | 0.014 | -0.023 | 0.012 | -0.016 | 0.015 |
| rs6797840 | 3 | 85656569 | A | C | 0.456 | -0.004 | 0.001 | 7.30E-03 | -0.016 | 0.002 | 1.70E-13 | TV | 0.009 | 0.010 | -0.004 | 0.014 | 0.018 | 0.014 | 0.016 | 0.012 | 0.015 | 0.015 |
| rs2034768 | 3 | 93986371 | A | G | 0.487 | 0.006 | 0.001 | 4.50E-06 | 0.015 | 0.002 | 8.60E-12 | TV | -0.013 | 0.010 | -0.017 | 0.014 | -0.007 | 0.014 | -0.014 | 0.012 | -0.007 | 0.015 |
| rs326341 | 3 | 107811142 | G | A | 0.524943 | 0.009 | 0.001 | 1.20E-11 | 0.003 | 0.002 | 1.60E-01 | smoking | 0.003 | 0.010 | 0.005 | 0.014 | 0.002 | 0.014 | 0.005 | 0.012 | 0.012 | 0.015 |
| rs9867121 | 3 | 114631548 | C | A | 0.818 | 0.002 | 0.002 | 2.30E-01 | 0.019 | 0.003 | 3.90E-12 | TV | 0.020 | 0.012 | 0.041 | 0.017 | -0.004 | 0.017 | 0.018 | 0.015 | 0.014 | 0.019 |
| rs73220544 | 3 | 131074511 | A | C | 0.842295 | -0.011 | 0.002 | 1.50E-08 | 0.000 | 0.003 | 9.80E-01 | smoking | -0.010 | 0.013 | -0.028 | 0.019 | 0.008 | 0.019 | -0.006 | 0.016 | -0.017 | 0.021 |
| rs12491503 | 3 | 165706855 | G | A | 0.67 | -0.003 | 0.001 | 5.50E-02 | -0.014 | 0.002 | 5.50E-10 | TV | -0.001 | 0.010 | -0.011 | 0.014 | 0.008 | 0.014 | -0.005 | 0.012 | -0.006 | 0.016 |
| rs114600294 | 3 | 181419367 | G | C | 0.789 | 0.002 | 0.002 | 3.10E-01 | -0.016 | 0.003 | 7.90E-10 | TV | 0.013 | 0.014 | 0.022 | 0.020 | 0.005 | 0.020 | 0.021 | 0.017 | 0.007 | 0.022 |
| rs66852340 | 4 | 3311070 | C | T | 0.776 | -0.005 | 0.002 | 1.20E-03 | -0.018 | 0.003 | 7.90E-12 | TV | -0.004 | 0.011 | -0.013 | 0.016 | 0.002 | 0.016 | 0.000 | 0.014 | -0.030 | 0.018 |
| rs7700107 | 4 | 17880416 | A | C | 0.863 | 0.001 | 0.002 | 7.80E-01 | -0.021 | 0.003 | 1.90E-11 | TV | -0.003 | 0.014 | -0.025 | 0.019 | 0.017 | 0.019 | 0.018 | 0.017 | -0.011 | 0.022 |
| rs317021 | 4 | 35418368 | T | A | 0.814287 | -0.012 | 0.002 | 1.10E-10 | -0.001 | 0.003 | 7.50E-01 | smoking | -0.004 | 0.013 | 0.014 | 0.019 | -0.022 | 0.019 | -0.008 | 0.016 | 0.000 | 0.021 |
| rs28971796 | 4 | 49151982 | A | G | 0.378 | 0.004 | 0.002 | 6.30E-03 | 0.015 | 0.002 | 2.10E-09 | TV | 0.010 | 0.012 | 0.004 | 0.017 | 0.018 | 0.017 | 0.003 | 0.015 | 0.037 | 0.019 |
| rs6850494 | 4 | 82291771 | A | C | 0.616 | -0.004 | 0.001 | 9.20E-03 | -0.014 | 0.002 | 1.10E-10 | TV | -0.011 | 0.010 | -0.019 | 0.014 | -0.002 | 0.014 | -0.029 | 0.012 | -0.008 | 0.016 |
| rs13107325 | 4 | 103188709 | C | T | 0.926 | 0.004 | 0.003 | 1.50E-01 | -0.029 | 0.004 | 1.50E-12 | TV | 0.008 | 0.019 | -0.009 | 0.026 | 0.028 | 0.027 | 0.012 | 0.022 | -0.006 | 0.029 |
| rs7693703 | 4 | 118357405 | G | A | 0.91 | 0.001 | 0.002 | 5.80E-01 | 0.023 | 0.004 | 2.70E-09 | TV | 0.022 | 0.017 | -0.004 | 0.024 | 0.050 | 0.024 | 0.018 | 0.020 | 0.070 | 0.027 |
| rs4334769 | 4 | 130245233 | G | T | 0.474 | 0.001 | 0.001 | 7.00E-01 | 0.012 | 0.002 | 2.10E-08 | TV | -0.015 | 0.010 | -0.010 | 0.014 | -0.016 | 0.014 | -0.023 | 0.012 | -0.015 | 0.015 |
| rs17576594 | 4 | 147952241 | G | A | 0.7235 | 0.011 | 0.002 | 1.70E-12 | 0.002 | 0.002 | 5.00E-01 | smoking | 0.010 | 0.011 | 0.011 | 0.015 | 0.012 | 0.015 | 0.009 | 0.013 | 0.012 | 0.017 |
| rs6825241 | 4 | 152543783 | C | A | 0.536 | 0.000 | 0.001 | 7.40E-01 | -0.017 | 0.002 | 4.90E-15 | TV | -0.008 | 0.010 | -0.003 | 0.014 | -0.012 | 0.014 | -0.005 | 0.012 | -0.016 | 0.015 |
| rs7693082 | 4 | 159857819 | G | C | 0.3 | 0.002 | 0.002 | 2.80E-01 | 0.015 | 0.002 | 1.70E-10 | TV | 0.017 | 0.011 | 0.012 | 0.015 | 0.020 | 0.015 | 0.026 | 0.013 | 0.020 | 0.017 |
| rs11948770 | 5 | 13246336 | T | C | 0.768248 | -0.010 | 0.002 | 4.90E-10 | -0.007 | 0.003 | 6.50E-03 | smoking | -0.006 | 0.011 | -0.018 | 0.016 | 0.006 | 0.016 | -0.016 | 0.014 | -0.015 | 0.018 |
| rs71627581 | 5 | 43161351 | G | A | 0.888811 | 0.013 | 0.002 | 1.60E-09 | 0.001 | 0.003 | 7.10E-01 | smoking | 0.029 | 0.017 | 0.042 | 0.023 | 0.021 | 0.024 | 0.048 | 0.020 | 0.041 | 0.025 |
| rs10940659 | 5 | 59360341 | A | G | 0.531 | 0.000 | 0.001 | 9.70E-01 | -0.013 | 0.002 | 1.50E-08 | TV | -0.011 | 0.010 | -0.027 | 0.014 | 0.003 | 0.014 | -0.011 | 0.012 | -0.020 | 0.015 |
| rs262890 | 5 | 62930015 | A | G | 0.699 | -0.005 | 0.002 | 2.10E-03 | -0.019 | 0.002 | 3.20E-15 | TV | 0.031 | 0.011 | 0.036 | 0.015 | 0.028 | 0.015 | 0.028 | 0.013 | 0.043 | 0.017 |
| rs7716447 | 5 | 88800331 | A | G | 0.639 | -0.007 | 0.001 | 1.10E-06 | -0.013 | 0.002 | 3.60E-09 | TV | -0.016 | 0.010 | -0.018 | 0.014 | -0.009 | 0.014 | -0.007 | 0.012 | -0.021 | 0.016 |
| rs1031423 | 5 | 93276883 | T | C | 0.215 | -0.004 | 0.002 | 1.70E-02 | -0.019 | 0.003 | 1.80E-12 | TV | 0.020 | 0.012 | 0.023 | 0.017 | 0.017 | 0.017 | 0.022 | 0.014 | 0.017 | 0.019 |
| rs57585211 | 5 | 107197725 | T | G | 0.827 | -0.002 | 0.002 | 3.30E-01 | -0.017 | 0.003 | 4.30E-09 | TV | -0.003 | 0.013 | -0.020 | 0.018 | 0.012 | 0.018 | -0.008 | 0.016 | -0.009 | 0.021 |
| rs9326862 | 5 | 112054547 | A | T | 0.468 | 0.006 | 0.001 | 4.20E-05 | 0.012 | 0.002 | 2.60E-08 | TV | 0.001 | 0.010 | -0.011 | 0.014 | 0.014 | 0.014 | 0.006 | 0.012 | -0.010 | 0.015 |
| rs10041724 | 5 | 124273520 | T | C | 0.808 | 0.005 | 0.002 | 2.80E-03 | 0.018 | 0.003 | 3.90E-11 | TV | 0.015 | 0.012 | 0.021 | 0.017 | 0.010 | 0.017 | 0.016 | 0.014 | 0.012 | 0.019 |
| rs329120 | 5 | 133861756 | C | T | 0.580672 | 0.010 | 0.001 | 6.30E-12 | 0.012 | 0.002 | 7.10E-08 | smoking | 0.005 | 0.010 | 0.003 | 0.014 | 0.006 | 0.014 | 0.008 | 0.012 | 0.024 | 0.015 |
| rs62379379 | 5 | 141082015 | G | T | 0.929 | -0.004 | 0.003 | 1.70E-01 | -0.026 | 0.004 | 7.80E-10 | TV | -0.032 | 0.019 | -0.025 | 0.027 | -0.038 | 0.026 | -0.029 | 0.023 | -0.022 | 0.031 |
| rs10054327 | 5 | 147871137 | G | A | 0.576 | 0.002 | 0.001 | 2.00E-01 | 0.017 | 0.002 | 3.40E-15 | TV | -0.002 | 0.010 | 0.003 | 0.014 | -0.005 | 0.014 | -0.013 | 0.012 | -0.002 | 0.016 |
| rs42210 | 5 | 166408788 | G | C | 0.289 | -0.006 | 0.002 | 9.00E-05 | -0.014 | 0.002 | 7.30E-09 | TV | -0.007 | 0.011 | -0.016 | 0.015 | 0.004 | 0.015 | 0.004 | 0.013 | -0.024 | 0.017 |
| rs72828890 | 5 | 167806836 | C | T | 0.869 | 0.003 | 0.002 | 1.10E-01 | 0.019 | 0.003 | 4.90E-09 | TV | 0.012 | 0.016 | 0.026 | 0.023 | -0.002 | 0.023 | 0.017 | 0.019 | 0.003 | 0.025 |
| rs245774 | 5 | 170530930 | A | G | 0.271691 | -0.009 | 0.002 | 7.40E-09 | -0.008 | 0.002 | 1.60E-03 | smoking | 0.003 | 0.011 | -0.004 | 0.015 | 0.008 | 0.016 | 0.003 | 0.013 | -0.008 | 0.017 |
| rs72834698 | 6 | 26176517 | G | A | 0.858 | 0.006 | 0.002 | 1.30E-03 | 0.023 | 0.003 | 2.70E-13 | TV | 0.044 | 0.014 | 0.060 | 0.020 | 0.031 | 0.020 | 0.059 | 0.017 | 0.069 | 0.022 |
| rs9471333 | 6 | 40362023 | C | T | 0.45 | 0.001 | 0.001 | 6.10E-01 | 0.013 | 0.002 | 1.50E-09 | TV | 0.010 | 0.010 | 0.020 | 0.014 | 0.001 | 0.014 | 0.007 | 0.012 | 0.018 | 0.015 |
| rs2894808 | 6 | 52861990 | T | A | 0.922121 | -0.015 | 0.003 | 3.50E-09 | -0.016 | 0.004 | 1.00E-04 | smoking | 0.003 | 0.017 | 0.000 | 0.024 | 0.006 | 0.024 | 0.009 | 0.021 | -0.025 | 0.027 |
| rs4523073 | 6 | 67550288 | A | G | 0.591 | -0.008 | 0.001 | 3.80E-09 | -0.014 | 0.002 | 5.00E-10 | TV | 0.006 | 0.010 | 0.002 | 0.014 | 0.009 | 0.014 | 0.002 | 0.012 | 0.022 | 0.016 |
| rs6905544 | 6 | 98411631 | A | G | 0.399 | -0.002 | 0.001 | 9.70E-02 | -0.019 | 0.002 | 8.50E-18 | TV | -0.029 | 0.010 | -0.037 | 0.014 | -0.023 | 0.014 | -0.024 | 0.012 | -0.035 | 0.015 |
| rs17789218 | 6 | 100600097 | T | C | 0.756 | 0.001 | 0.002 | 5.60E-01 | 0.019 | 0.003 | 1.40E-13 | TV | 0.004 | 0.012 | 0.016 | 0.016 | -0.006 | 0.016 | -0.006 | 0.014 | 0.011 | 0.018 |
| rs7766610 | 6 | 111707821 | C | A | 0.182634 | 0.013 | 0.002 | 2.20E-12 | 0.002 | 0.003 | 4.90E-01 | smoking | 0.016 | 0.012 | 0.038 | 0.017 | -0.006 | 0.017 | 0.020 | 0.015 | 0.007 | 0.019 |
| rs2184364 | 6 | 143183596 | A | G | 0.782 | 0.004 | 0.002 | 1.40E-02 | 0.016 | 0.003 | 3.00E-09 | TV | 0.023 | 0.012 | 0.025 | 0.017 | 0.022 | 0.017 | 0.024 | 0.015 | 0.017 | 0.019 |
| rs9718104 | 6 | 166170539 | T | G | 0.942 | 0.001 | 0.003 | 7.50E-01 | -0.041 | 0.005 | 9.30E-19 | TV | -0.002 | 0.022 | 0.002 | 0.032 | -0.013 | 0.031 | 0.009 | 0.027 | 0.005 | 0.036 |
| rs17568389 | 7 | 8091876 | T | A | 0.487 | 0.001 | 0.001 | 5.20E-01 | 0.015 | 0.002 | 8.60E-13 | TV | 0.001 | 0.010 | 0.012 | 0.014 | -0.012 | 0.014 | 0.000 | 0.012 | 0.012 | 0.015 |
| rs10226228 | 7 | 32315613 | A | G | 0.62964 | -0.011 | 0.001 | 2.00E-15 | -0.007 | 0.002 | 2.30E-03 | smoking | 0.003 | 0.010 | 0.004 | 0.014 | 0.001 | 0.014 | 0.008 | 0.012 | 0.014 | 0.016 |
| rs10234444 | 7 | 41828527 | G | A | 0.823 | 0.001 | 0.002 | 7.00E-01 | 0.016 | 0.003 | 2.90E-08 | TV | -0.017 | 0.013 | -0.018 | 0.018 | -0.018 | 0.018 | -0.023 | 0.016 | -0.004 | 0.021 |
| rs6973656 | 7 | 77422583 | A | G | 0.603 | 0.000 | 0.001 | 8.90E-01 | -0.013 | 0.002 | 1.00E-09 | TV | 0.029 | 0.010 | 0.025 | 0.014 | 0.031 | 0.014 | 0.028 | 0.012 | 0.036 | 0.015 |
| rs973734 | 7 | 89387578 | C | A | 0.152 | 0.004 | 0.002 | 5.60E-02 | 0.017 | 0.003 | 1.50E-08 | TV | 0.002 | 0.014 | -0.022 | 0.020 | 0.031 | 0.020 | 0.013 | 0.016 | -0.009 | 0.021 |
| rs11768481 | 7 | 96629103 | C | A | 0.666168 | 0.009 | 0.001 | 9.90E-10 | 0.007 | 0.002 | 4.30E-03 | smoking | 0.000 | 0.011 | 0.023 | 0.015 | -0.018 | 0.015 | 0.012 | 0.013 | -0.001 | 0.017 |
| rs6962772 | 7 | 99081730 | A | G | 0.845551 | 0.011 | 0.002 | 7.80E-09 | 0.017 | 0.003 | 1.60E-08 | smoking | 0.011 | 0.013 | -0.007 | 0.018 | 0.034 | 0.018 | 0.011 | 0.016 | -0.005 | 0.021 |
| rs62471080 | 7 | 109128060 | G | C | 0.541 | -0.001 | 0.001 | 4.30E-01 | -0.013 | 0.002 | 3.70E-09 | TV | -0.005 | 0.010 | -0.007 | 0.014 | -0.004 | 0.014 | 0.010 | 0.012 | -0.012 | 0.015 |
| rs2401924 | 7 | 115057862 | G | C | 0.501955 | 0.011 | 0.001 | 2.70E-14 | 0.000 | 0.002 | 8.20E-01 | smoking | -0.004 | 0.010 | -0.020 | 0.014 | 0.014 | 0.014 | 0.001 | 0.012 | -0.017 | 0.015 |
| rs10246289 | 7 | 115544763 | A | G | 0.113 | 0.001 | 0.002 | 5.10E-01 | 0.019 | 0.003 | 1.70E-08 | TV | 0.041 | 0.015 | 0.039 | 0.022 | 0.042 | 0.022 | 0.033 | 0.019 | 0.033 | 0.024 |
| rs11763734 | 7 | 126400910 | A | C | 0.51 | -0.004 | 0.001 | 2.20E-03 | -0.012 | 0.002 | 8.20E-09 | TV | -0.001 | 0.010 | 0.001 | 0.014 | -0.005 | 0.014 | -0.002 | 0.012 | 0.005 | 0.015 |
| rs6957896 | 7 | 132309592 | C | T | 0.503261 | -0.008 | 0.001 | 4.50E-08 | 0.002 | 0.002 | 2.50E-01 | smoking | 0.006 | 0.010 | -0.003 | 0.014 | 0.015 | 0.014 | -0.003 | 0.012 | 0.007 | 0.015 |
| rs4731925 | 7 | 132664757 | C | T | 0.315607 | -0.008 | 0.001 | 2.60E-08 | -0.009 | 0.002 | 4.70E-05 | smoking | -0.007 | 0.010 | -0.007 | 0.015 | -0.007 | 0.015 | -0.006 | 0.012 | -0.032 | 0.016 |
| rs7834121 | 8 | 10770255 | G | T | 0.504 | 0.007 | 0.001 | 1.30E-06 | -0.014 | 0.002 | 1.10E-10 | TV | 0.007 | 0.010 | 0.015 | 0.015 | 0.000 | 0.015 | -0.007 | 0.012 | 0.028 | 0.016 |
| rs11783093 | 8 | 27425349 | C | T | 0.838811 | 0.016 | 0.002 | 1.20E-16 | 0.000 | 0.003 | 8.90E-01 | smoking | 0.012 | 0.013 | 0.002 | 0.019 | 0.020 | 0.019 | 0.017 | 0.016 | 0.009 | 0.021 |
| rs1421334 | 8 | 30865733 | A | C | 0.45 | 0.003 | 0.001 | 5.70E-02 | 0.017 | 0.002 | 2.20E-15 | TV | 0.017 | 0.010 | 0.017 | 0.014 | 0.018 | 0.014 | 0.022 | 0.012 | -0.005 | 0.015 |
| rs6996198 | 8 | 65463442 | C | T | 0.843 | 0.001 | 0.002 | 6.10E-01 | -0.016 | 0.003 | 3.50E-08 | TV | -0.016 | 0.013 | -0.009 | 0.018 | -0.025 | 0.018 | -0.011 | 0.016 | -0.017 | 0.020 |
| rs6472942 | 8 | 76827190 | T | C | 0.568 | -0.005 | 0.001 | 1.80E-04 | -0.013 | 0.002 | 1.80E-09 | TV | 0.000 | 0.010 | -0.006 | 0.014 | 0.004 | 0.014 | -0.001 | 0.012 | 0.015 | 0.015 |
| rs11985394 | 8 | 92667521 | T | A | 0.545 | 0.005 | 0.001 | 9.80E-04 | 0.012 | 0.002 | 1.30E-08 | TV | 0.010 | 0.010 | 0.017 | 0.014 | -0.001 | 0.014 | 0.020 | 0.012 | 0.000 | 0.015 |
| rs12541615 | 8 | 118870151 | T | C | 0.817 | -0.004 | 0.002 | 2.00E-02 | -0.018 | 0.003 | 3.90E-10 | TV | -0.003 | 0.012 | 0.001 | 0.017 | -0.008 | 0.018 | -0.013 | 0.015 | 0.006 | 0.019 |
| rs2717559 | 8 | 143882420 | A | G | 0.559 | 0.002 | 0.001 | 2.20E-01 | 0.012 | 0.002 | 1.50E-08 | TV | 0.000 | 0.010 | -0.018 | 0.014 | 0.018 | 0.014 | -0.006 | 0.012 | 0.003 | 0.015 |
| rs2616830 | 9 | 1721385 | G | A | 0.462 | 0.001 | 0.001 | 3.40E-01 | 0.016 | 0.002 | 2.90E-14 | TV | 0.013 | 0.010 | 0.014 | 0.014 | 0.008 | 0.014 | 0.016 | 0.012 | 0.031 | 0.015 |
| rs34864022 | 9 | 22609110 | A | G | 0.934 | -0.008 | 0.003 | 3.80E-03 | -0.026 | 0.004 | 1.40E-09 | TV | 0.013 | 0.021 | 0.033 | 0.030 | -0.010 | 0.030 | 0.015 | 0.025 | -0.026 | 0.033 |
| rs12554512 | 9 | 23352293 | T | C | 0.584 | 0.004 | 0.001 | 8.50E-03 | 0.021 | 0.002 | 3.80E-21 | TV | -0.002 | 0.010 | -0.001 | 0.014 | -0.002 | 0.014 | 0.011 | 0.012 | -0.005 | 0.016 |
| rs2791574 | 9 | 82212094 | T | G | 0.657 | -0.003 | 0.001 | 7.70E-02 | -0.013 | 0.002 | 3.00E-08 | TV | -0.020 | 0.010 | -0.017 | 0.014 | -0.019 | 0.014 | -0.021 | 0.012 | -0.014 | 0.016 |
| rs870151 | 9 | 92230747 | T | A | 0.528 | -0.002 | 0.001 | 9.40E-02 | -0.016 | 0.002 | 8.80E-13 | TV | 0.010 | 0.010 | 0.005 | 0.014 | 0.018 | 0.014 | 0.011 | 0.012 | 0.005 | 0.015 |
| rs2787374 | 9 | 103054951 | T | C | 0.408 | 0.003 | 0.001 | 3.20E-02 | 0.012 | 0.002 | 1.20E-08 | TV | 0.012 | 0.010 | -0.002 | 0.014 | 0.025 | 0.014 | 0.005 | 0.012 | 0.028 | 0.015 |
| rs7043521 | 9 | 124602779 | A | T | 0.431 | 0.005 | 0.001 | 9.70E-04 | 0.014 | 0.002 | 6.50E-11 | TV | -0.021 | 0.010 | -0.014 | 0.014 | -0.027 | 0.014 | -0.021 | 0.012 | -0.043 | 0.015 |
| rs4382592 | 9 | 134870755 | T | G | 0.301 | 0.007 | 0.002 | 1.10E-06 | 0.014 | 0.002 | 6.70E-09 | TV | 0.009 | 0.010 | 0.013 | 0.015 | 0.005 | 0.015 | 0.006 | 0.013 | 0.000 | 0.016 |
| rs2073869 | 9 | 135763816 | C | T | 0.834 | 0.004 | 0.002 | 3.00E-02 | 0.019 | 0.003 | 1.50E-10 | TV | -0.011 | 0.013 | -0.002 | 0.018 | -0.018 | 0.018 | -0.018 | 0.015 | -0.019 | 0.020 |
| rs1623025 | 10 | 11164813 | G | C | 0.529 | -0.002 | 0.001 | 1.20E-01 | -0.012 | 0.002 | 1.40E-08 | TV | 0.018 | 0.010 | 0.019 | 0.014 | 0.017 | 0.014 | 0.019 | 0.012 | 0.006 | 0.015 |
| rs1243182 | 10 | 21916728 | C | T | 0.69 | -0.006 | 0.001 | 1.30E-04 | -0.019 | 0.002 | 2.00E-15 | TV | -0.029 | 0.011 | -0.039 | 0.015 | -0.020 | 0.015 | -0.025 | 0.013 | -0.054 | 0.016 |
| rs2045147 | 10 | 56660437 | A | G | 0.449 | 0.007 | 0.001 | 9.60E-08 | 0.013 | 0.002 | 5.90E-09 | TV | 0.009 | 0.010 | 0.018 | 0.014 | -0.001 | 0.014 | 0.006 | 0.012 | 0.019 | 0.015 |
| rs10994943 | 10 | 63591413 | T | G | 0.581 | 0.008 | 0.001 | 1.50E-08 | 0.013 | 0.002 | 3.10E-09 | TV | 0.007 | 0.010 | 0.017 | 0.014 | -0.006 | 0.014 | 0.000 | 0.012 | 0.014 | 0.015 |
| rs10823968 | 10 | 74738269 | A | T | 0.632969 | 0.008 | 0.001 | 2.10E-08 | 0.002 | 0.002 | 3.00E-01 | smoking | -0.009 | 0.010 | -0.015 | 0.014 | 0.001 | 0.014 | -0.014 | 0.012 | 0.016 | 0.016 |
| rs11201422 | 10 | 86924483 | T | C | 0.669 | 0.002 | 0.001 | 2.40E-01 | 0.013 | 0.002 | 3.40E-08 | TV | 0.019 | 0.011 | 0.005 | 0.015 | 0.036 | 0.015 | 0.022 | 0.012 | 0.027 | 0.016 |
| rs17553262 | 10 | 92912773 | A | C | 0.884605 | -0.013 | 0.002 | 5.30E-09 | -0.009 | 0.003 | 9.50E-03 | smoking | -0.015 | 0.016 | -0.005 | 0.023 | -0.028 | 0.022 | -0.013 | 0.019 | -0.032 | 0.025 |
| rs10786658 | 10 | 103737193 | A | T | 0.414 | -0.005 | 0.001 | 3.70E-04 | -0.014 | 0.002 | 4.60E-11 | TV | 0.003 | 0.010 | 0.011 | 0.014 | -0.007 | 0.014 | 0.002 | 0.012 | -0.001 | 0.015 |
| rs3896224 | 10 | 106467853 | A | G | 0.585286 | 0.010 | 0.001 | 1.10E-11 | 0.007 | 0.002 | 1.50E-03 | smoking | -0.002 | 0.010 | -0.011 | 0.014 | 0.004 | 0.014 | 0.002 | 0.012 | -0.017 | 0.015 |
| rs7089973 | 10 | 116569565 | C | A | 0.619 | -0.003 | 0.001 | 3.50E-02 | -0.013 | 0.002 | 2.40E-09 | TV | -0.021 | 0.010 | -0.022 | 0.015 | -0.020 | 0.014 | -0.028 | 0.012 | -0.036 | 0.016 |
| rs11245482 | 10 | 126733546 | T | C | 0.613 | -0.003 | 0.001 | 2.20E-02 | -0.013 | 0.002 | 2.60E-09 | TV | -0.004 | 0.010 | 0.006 | 0.014 | -0.016 | 0.014 | -0.002 | 0.012 | -0.008 | 0.015 |
| rs17727474 | 10 | 127182959 | C | T | 0.832 | 0.003 | 0.002 | 1.00E-01 | 0.018 | 0.003 | 3.10E-09 | TV | 0.001 | 0.013 | -0.001 | 0.019 | 0.005 | 0.019 | -0.009 | 0.016 | 0.010 | 0.021 |
| rs12289262 | 11 | 12894758 | C | T | 0.731 | 0.000 | 0.002 | 9.30E-01 | -0.014 | 0.002 | 2.00E-08 | TV | -0.028 | 0.011 | -0.029 | 0.016 | -0.029 | 0.016 | -0.030 | 0.014 | -0.010 | 0.018 |
| rs34866095 | 11 | 16377356 | A | G | 0.686187 | -0.009 | 0.002 | 1.20E-08 | 0.000 | 0.002 | 8.40E-01 | smoking | -0.002 | 0.011 | -0.013 | 0.015 | 0.008 | 0.015 | 0.008 | 0.013 | -0.005 | 0.016 |
| rs75742406 | 11 | 17070365 | G | A | 0.738854 | 0.010 | 0.002 | 1.30E-09 | 0.004 | 0.002 | 1.40E-01 | smoking | 0.002 | 0.011 | -0.011 | 0.016 | 0.012 | 0.016 | 0.004 | 0.014 | -0.006 | 0.018 |
| rs17309874 | 11 | 27667236 | G | A | 0.740357 | -0.011 | 0.002 | 9.70E-13 | -0.003 | 0.002 | 1.60E-01 | smoking | 0.018 | 0.012 | 0.016 | 0.017 | 0.016 | 0.017 | 0.015 | 0.014 | 0.030 | 0.018 |
| rs4391802 | 11 | 28674592 | A | G | 0.707464 | 0.010 | 0.002 | 1.40E-11 | 0.005 | 0.002 | 2.40E-02 | smoking | 0.025 | 0.011 | 0.013 | 0.015 | 0.038 | 0.015 | 0.016 | 0.013 | 0.047 | 0.017 |
| rs112282219 | 11 | 46632809 | G | A | 0.958949 | -0.023 | 0.004 | 3.80E-11 | -0.005 | 0.005 | 3.70E-01 | smoking | 0.003 | 0.026 | 0.024 | 0.038 | -0.017 | 0.037 | -0.017 | 0.032 | -0.001 | 0.041 |
| rs801733 | 11 | 65934549 | A | C | 0.641 | 0.003 | 0.001 | 5.30E-02 | 0.017 | 0.002 | 7.30E-14 | TV | 0.027 | 0.010 | 0.028 | 0.014 | 0.026 | 0.014 | 0.023 | 0.012 | 0.048 | 0.016 |
| rs116175338 | 11 | 72467875 | G | T | 0.754 | -0.006 | 0.002 | 3.00E-04 | -0.014 | 0.003 | 1.40E-08 | TV | -0.001 | 0.011 | 0.011 | 0.016 | -0.011 | 0.016 | -0.002 | 0.014 | 0.020 | 0.018 |
| rs12272012 | 11 | 84861286 | G | C | 0.956 | 0.000 | 0.003 | 9.10E-01 | -0.030 | 0.005 | 1.90E-08 | TV | 0.031 | 0.024 | 0.000 | 0.034 | 0.062 | 0.033 | 0.036 | 0.028 | 0.064 | 0.037 |
| rs11020045 | 11 | 92487907 | A | C | 0.669 | -0.004 | 0.001 | 3.20E-03 | -0.013 | 0.002 | 1.60E-08 | TV | 0.007 | 0.010 | 0.021 | 0.015 | -0.006 | 0.015 | 0.018 | 0.012 | -0.003 | 0.016 |
| rs17207890 | 11 | 95490754 | G | A | 0.657 | 0.005 | 0.001 | 1.80E-03 | 0.016 | 0.002 | 6.70E-12 | TV | 0.000 | 0.010 | 0.012 | 0.015 | -0.010 | 0.015 | 0.016 | 0.012 | -0.021 | 0.016 |
| rs11218575 | 11 | 122154576 | C | T | 0.566 | 0.002 | 0.001 | 1.50E-01 | 0.015 | 0.002 | 2.10E-12 | TV | 0.001 | 0.010 | 0.013 | 0.014 | -0.010 | 0.014 | 0.001 | 0.012 | -0.001 | 0.015 |
| rs4937842 | 11 | 133734542 | G | C | 0.634 | 0.001 | 0.001 | 4.50E-01 | -0.013 | 0.002 | 1.40E-08 | TV | -0.012 | 0.010 | -0.018 | 0.014 | -0.008 | 0.014 | -0.018 | 0.012 | -0.023 | 0.016 |
| rs10772643 | 12 | 13415288 | C | T | 0.108 | 0.005 | 0.002 | 2.90E-02 | 0.025 | 0.003 | 1.30E-12 | TV | 0.027 | 0.016 | 0.041 | 0.022 | 0.014 | 0.022 | 0.024 | 0.019 | 0.056 | 0.024 |
| rs10771746 | 12 | 30791864 | C | T | 0.717 | 0.001 | 0.002 | 5.10E-01 | -0.014 | 0.002 | 2.50E-09 | TV | 0.006 | 0.010 | -0.008 | 0.015 | 0.017 | 0.015 | 0.001 | 0.013 | 0.031 | 0.017 |
| rs74086911 | 12 | 50015942 | G | A | 0.925459 | 0.015 | 0.003 | 2.10E-08 | 0.008 | 0.004 | 4.30E-02 | smoking | -0.006 | 0.019 | 0.005 | 0.027 | -0.016 | 0.027 | -0.013 | 0.022 | 0.036 | 0.030 |
| rs10876864 | 12 | 56401085 | G | A | 0.427 | -0.007 | 0.001 | 1.50E-07 | -0.013 | 0.002 | 1.00E-09 | TV | -0.010 | 0.010 | -0.005 | 0.014 | -0.018 | 0.014 | -0.029 | 0.012 | 0.021 | 0.015 |
| rs8756 | 12 | 66359752 | C | A | 0.485 | -0.004 | 0.001 | 1.30E-03 | -0.013 | 0.002 | 5.30E-10 | TV | -0.009 | 0.010 | -0.008 | 0.014 | -0.009 | 0.014 | -0.005 | 0.012 | -0.016 | 0.015 |
| rs12831617 | 12 | 84758368 | C | T | 0.764305 | -0.009 | 0.002 | 1.90E-08 | -0.003 | 0.003 | 1.80E-01 | smoking | 0.007 | 0.011 | -0.002 | 0.016 | 0.015 | 0.016 | 0.002 | 0.013 | 0.003 | 0.018 |
| rs2164744 | 12 | 109872039 | T | C | 0.635 | -0.004 | 0.001 | 6.50E-03 | -0.013 | 0.002 | 1.10E-08 | TV | 0.014 | 0.010 | 0.013 | 0.014 | 0.015 | 0.014 | 0.007 | 0.012 | 0.027 | 0.015 |
| rs2173650 | 12 | 117525235 | G | T | 0.852 | 0.006 | 0.002 | 1.40E-03 | 0.018 | 0.003 | 4.60E-09 | TV | -0.006 | 0.013 | 0.016 | 0.019 | -0.028 | 0.019 | -0.002 | 0.016 | 0.010 | 0.021 |
| rs9563168 | 13 | 54247827 | G | A | 0.791 | 0.003 | 0.002 | 1.10E-01 | 0.018 | 0.003 | 4.30E-11 | TV | 0.006 | 0.012 | -0.004 | 0.017 | 0.016 | 0.017 | 0.010 | 0.014 | -0.013 | 0.018 |
| rs9569734 | 13 | 58319476 | A | G | 0.844 | 0.002 | 0.002 | 2.10E-01 | 0.019 | 0.003 | 3.40E-10 | TV | 0.010 | 0.013 | 0.024 | 0.019 | -0.011 | 0.019 | 0.012 | 0.016 | 0.037 | 0.021 |
| rs6562474 | 13 | 67332812 | C | G | 0.650606 | 0.008 | 0.001 | 1.00E-08 | -0.001 | 0.002 | 7.90E-01 | smoking | 0.010 | 0.010 | 0.010 | 0.014 | 0.008 | 0.014 | 0.016 | 0.012 | -0.004 | 0.016 |
| rs56858768 | 13 | 86511730 | G | A | 0.701 | -0.001 | 0.002 | 3.90E-01 | -0.015 | 0.002 | 3.40E-10 | TV | -0.010 | 0.011 | -0.006 | 0.015 | -0.015 | 0.015 | -0.012 | 0.013 | -0.019 | 0.017 |
| rs7333559 | 13 | 100546450 | G | A | 0.211711 | 0.011 | 0.002 | 3.20E-10 | 0.005 | 0.003 | 4.30E-02 | smoking | -0.012 | 0.012 | -0.024 | 0.017 | 0.003 | 0.017 | 0.002 | 0.014 | -0.038 | 0.019 |
| rs7991062 | 13 | 100713194 | C | G | 0.659 | -0.007 | 0.001 | 4.00E-07 | -0.018 | 0.002 | 8.80E-15 | TV | -0.013 | 0.010 | -0.011 | 0.014 | -0.015 | 0.014 | -0.012 | 0.012 | -0.017 | 0.016 |
| rs405797 | 14 | 26972834 | T | A | 0.247 | -0.004 | 0.002 | 8.00E-03 | -0.015 | 0.003 | 2.80E-09 | TV | -0.014 | 0.013 | -0.009 | 0.016 | -0.023 | 0.016 | -0.017 | 0.013 | -0.006 | 0.017 |
| rs1022785 | 14 | 29571653 | G | A | 0.142 | 0.002 | 0.002 | 4.30E-01 | 0.018 | 0.003 | 7.10E-09 | TV | -0.003 | 0.016 | 0.027 | 0.019 | -0.024 | 0.019 | -0.005 | 0.016 | 0.035 | 0.021 |
| rs860326 | 14 | 57342912 | C | T | 0.427696 | 0.008 | 0.001 | 2.70E-09 | 0.010 | 0.002 | 8.60E-06 | smoking | 0.010 | 0.012 | 0.011 | 0.014 | 0.005 | 0.014 | 0.009 | 0.012 | 0.007 | 0.015 |
| rs7155595 | 14 | 77502546 | A | C | 0.674282 | -0.009 | 0.001 | 2.50E-09 | -0.008 | 0.002 | 1.10E-03 | smoking | -0.021 | 0.012 | -0.026 | 0.015 | -0.024 | 0.015 | -0.020 | 0.012 | -0.026 | 0.016 |
| rs10145592 | 14 | 94287215 | C | G | 0.409 | 0.001 | 0.001 | 6.20E-01 | -0.015 | 0.002 | 1.80E-11 | TV | -0.023 | 0.012 | -0.017 | 0.014 | -0.014 | 0.014 | -0.019 | 0.012 | -0.029 | 0.015 |
| rs7157001 | 14 | 99749484 | A | G | 0.744 | -0.002 | 0.002 | 2.90E-01 | -0.014 | 0.002 | 2.30E-08 | TV | -0.007 | 0.014 | -0.005 | 0.017 | -0.013 | 0.017 | -0.003 | 0.014 | -0.051 | 0.019 |
| rs3742365 | 14 | 104198251 | T | C | 0.595136 | -0.011 | 0.001 | 2.50E-14 | 0.003 | 0.002 | 1.90E-01 | smoking | -0.021 | 0.012 | -0.036 | 0.014 | 0.014 | 0.014 | -0.013 | 0.012 | -0.034 | 0.016 |
| rs8043253 | 15 | 41503730 | C | T | 0.573 | 0.003 | 0.001 | 7.20E-02 | -0.012 | 0.002 | 3.40E-08 | TV | 0.017 | 0.010 | 0.013 | 0.014 | 0.019 | 0.014 | 0.028 | 0.012 | -0.002 | 0.015 |
| rs2460 | 15 | 53073084 | G | A | 0.736 | -0.003 | 0.002 | 4.00E-02 | -0.015 | 0.002 | 5.00E-10 | TV | -0.015 | 0.011 | 0.004 | 0.016 | -0.032 | 0.016 | -0.021 | 0.013 | 0.015 | 0.018 |
| rs4775373 | 15 | 61476936 | T | C | 0.361 | 0.001 | 0.001 | 3.60E-01 | 0.013 | 0.002 | 4.70E-09 | TV | 0.006 | 0.010 | 0.002 | 0.014 | 0.009 | 0.014 | 0.007 | 0.012 | 0.017 | 0.016 |
| rs28485305 | 15 | 74044197 | C | T | 0.631482 | 0.008 | 0.001 | 2.60E-08 | 0.002 | 0.002 | 3.00E-01 | smoking | -0.014 | 0.010 | -0.001 | 0.014 | -0.029 | 0.014 | -0.009 | 0.012 | -0.032 | 0.015 |
| rs61331678 | 15 | 77995949 | G | C | 0.568 | 0.003 | 0.001 | 4.10E-02 | 0.015 | 0.002 | 6.10E-12 | TV | -0.011 | 0.010 | 0.004 | 0.014 | -0.025 | 0.014 | -0.015 | 0.012 | -0.011 | 0.015 |
| rs8042134 | 15 | 97514404 | T | G | 0.541083 | -0.010 | 0.001 | 1.30E-12 | 0.001 | 0.002 | 7.60E-01 | smoking | -0.007 | 0.010 | 0.000 | 0.014 | -0.012 | 0.014 | -0.007 | 0.012 | -0.015 | 0.016 |
| rs6598539 | 15 | 99204483 | T | C | 0.488672 | -0.008 | 0.001 | 4.50E-09 | -0.006 | 0.002 | 5.10E-03 | smoking | -0.014 | 0.010 | -0.017 | 0.014 | -0.012 | 0.014 | -0.015 | 0.012 | -0.022 | 0.015 |
| rs11861214 | 16 | 746611 | G | T | 0.78364 | 0.009 | 0.002 | 2.00E-08 | 0.008 | 0.003 | 4.30E-03 | smoking | 0.010 | 0.012 | 0.008 | 0.017 | 0.009 | 0.016 | 0.015 | 0.014 | 0.010 | 0.019 |
| rs35574015 | 16 | 15146061 | T | C | 0.706 | -0.003 | 0.002 | 3.40E-02 | -0.013 | 0.002 | 3.00E-08 | TV | 0.020 | 0.010 | 0.028 | 0.015 | 0.011 | 0.015 | 0.018 | 0.013 | -0.001 | 0.016 |
| rs7189927 | 16 | 28913787 | T | C | 0.356 | 0.003 | 0.001 | 2.40E-02 | 0.015 | 0.002 | 3.40E-11 | TV | 0.009 | 0.010 | 0.006 | 0.015 | 0.012 | 0.015 | 0.016 | 0.012 | 0.009 | 0.016 |
| rs749671 | 16 | 31088347 | G | A | 0.629 | 0.007 | 0.001 | 4.90E-06 | 0.016 | 0.002 | 2.70E-12 | TV | 0.023 | 0.010 | 0.018 | 0.014 | 0.028 | 0.014 | 0.023 | 0.012 | 0.015 | 0.016 |
| rs7184800 | 16 | 53509131 | G | A | 0.697 | 0.004 | 0.002 | 1.60E-02 | 0.017 | 0.002 | 8.20E-13 | TV | 0.005 | 0.011 | -0.003 | 0.015 | 0.011 | 0.015 | 0.003 | 0.013 | 0.024 | 0.016 |
| rs889398 | 16 | 69556715 | C | T | 0.588005 | 0.009 | 0.001 | 6.30E-11 | 0.002 | 0.002 | 4.30E-01 | smoking | 0.016 | 0.010 | 0.014 | 0.014 | 0.016 | 0.014 | 0.020 | 0.012 | -0.003 | 0.015 |
| rs60952428 | 16 | 75640521 | T | C | 0.909149 | 0.013 | 0.002 | 3.00E-08 | 0.010 | 0.004 | 1.10E-02 | smoking | 0.022 | 0.017 | 0.011 | 0.024 | 0.032 | 0.024 | 0.028 | 0.021 | 0.001 | 0.027 |
| rs1050847 | 16 | 87443734 | C | T | 0.425845 | 0.008 | 0.001 | 1.40E-08 | 0.005 | 0.002 | 3.50E-02 | smoking | -0.017 | 0.010 | -0.024 | 0.014 | -0.010 | 0.014 | -0.021 | 0.012 | 0.008 | 0.016 |
| rs369230 | 16 | 89645437 | G | T | 0.307565 | -0.009 | 0.002 | 1.80E-09 | -0.006 | 0.002 | 9.40E-03 | smoking | -0.023 | 0.011 | -0.010 | 0.016 | -0.036 | 0.016 | -0.037 | 0.013 | -0.003 | 0.018 |
| rs2447098 | 17 | 2277720 | C | A | 0.475 | -0.004 | 0.001 | 1.70E-03 | -0.015 | 0.002 | 6.80E-12 | TV | -0.011 | 0.010 | -0.034 | 0.014 | 0.009 | 0.014 | -0.010 | 0.012 | -0.003 | 0.015 |
| rs11654952 | 17 | 4813799 | T | G | 0.861 | -0.001 | 0.002 | 7.60E-01 | -0.017 | 0.003 | 4.30E-08 | TV | 0.025 | 0.014 | 0.005 | 0.019 | 0.047 | 0.020 | 0.018 | 0.017 | 0.001 | 0.022 |
| rs8614 | 17 | 27588806 | C | A | 0.817489 | -0.011 | 0.002 | 1.80E-10 | 0.001 | 0.003 | 6.90E-01 | smoking | -0.025 | 0.013 | -0.030 | 0.018 | -0.025 | 0.018 | -0.016 | 0.015 | -0.060 | 0.020 |
| rs732083 | 17 | 37834367 | G | A | 0.333362 | 0.008 | 0.001 | 1.50E-08 | 0.007 | 0.002 | 3.10E-03 | smoking | 0.030 | 0.010 | 0.020 | 0.014 | 0.042 | 0.014 | 0.018 | 0.012 | 0.056 | 0.016 |
| rs67596067 | 17 | 50333733 | G | A | 0.648812 | -0.009 | 0.001 | 1.20E-09 | -0.009 | 0.002 | 6.90E-05 | smoking | -0.002 | 0.010 | -0.006 | 0.015 | -0.004 | 0.015 | -0.004 | 0.012 | -0.013 | 0.016 |
| rs9902312 | 17 | 65070304 | T | C | 0.683 | 0.000 | 0.001 | 8.30E-01 | 0.015 | 0.002 | 4.50E-11 | TV | -0.001 | 0.010 | -0.005 | 0.015 | 0.004 | 0.015 | 0.000 | 0.012 | 0.010 | 0.016 |
| rs11657730 | 17 | 79372489 | C | T | 0.637 | 0.003 | 0.001 | 1.70E-02 | 0.013 | 0.002 | 1.10E-08 | TV | 0.010 | 0.011 | 0.007 | 0.015 | 0.012 | 0.015 | 0.010 | 0.012 | 0.016 | 0.016 |
| rs303753 | 18 | 21074922 | G | A | 0.653 | 0.000 | 0.001 | 9.20E-01 | -0.014 | 0.002 | 2.70E-10 | TV | -0.013 | 0.010 | -0.029 | 0.015 | 0.001 | 0.015 | -0.008 | 0.012 | -0.022 | 0.016 |
| rs9964724 | 18 | 35159124 | C | T | 0.318 | 0.007 | 0.001 | 6.50E-07 | 0.018 | 0.002 | 3.30E-14 | TV | 0.009 | 0.010 | 0.014 | 0.015 | 0.004 | 0.015 | 0.011 | 0.012 | 0.012 | 0.016 |
| rs1156541 | 18 | 39952989 | C | T | 0.223 | 0.006 | 0.002 | 3.40E-04 | 0.014 | 0.003 | 2.80E-08 | TV | 0.017 | 0.011 | 0.016 | 0.016 | 0.019 | 0.016 | 0.015 | 0.014 | 0.043 | 0.018 |
| rs62098013 | 18 | 50863861 | G | A | 0.63991 | -0.009 | 0.001 | 4.10E-09 | -0.006 | 0.002 | 9.10E-03 | smoking | -0.016 | 0.010 | -0.015 | 0.015 | -0.016 | 0.015 | -0.010 | 0.012 | -0.014 | 0.016 |
| rs17512836 | 18 | 53194961 | T | C | 0.974 | 0.002 | 0.004 | 6.90E-01 | 0.042 | 0.007 | 7.00E-10 | TV | -0.014 | 0.028 | -0.018 | 0.040 | -0.009 | 0.040 | 0.019 | 0.033 | -0.075 | 0.043 |
| rs71367545 | 18 | 77576337 | G | A | 0.790533 | -0.010 | 0.002 | 1.40E-09 | -0.007 | 0.003 | 1.00E-02 | smoking | -0.024 | 0.012 | -0.005 | 0.018 | -0.038 | 0.018 | -0.016 | 0.015 | -0.052 | 0.019 |
| rs7248205 | 19 | 10770305 | C | T | 0.398 | -0.002 | 0.001 | 1.40E-01 | 0.014 | 0.002 | 3.40E-10 | TV | -0.029 | 0.010 | -0.046 | 0.014 | -0.010 | 0.014 | -0.021 | 0.012 | -0.023 | 0.016 |
| rs35343344 | 19 | 18471610 | C | A | 0.732713 | 0.009 | 0.002 | 8.80E-09 | 0.005 | 0.002 | 7.00E-02 | smoking | 0.000 | 0.011 | -0.022 | 0.016 | 0.023 | 0.016 | -0.004 | 0.013 | -0.002 | 0.018 |
| rs111901094 | 19 | 19513570 | G | T | 0.817 | -0.005 | 0.002 | 1.00E-02 | -0.017 | 0.003 | 2.00E-09 | TV | -0.011 | 0.013 | 0.022 | 0.018 | -0.044 | 0.018 | 0.003 | 0.016 | -0.022 | 0.020 |
| rs6131281 | 20 | 11891724 | C | T | 0.597 | -0.001 | 0.001 | 7.20E-01 | 0.016 | 0.002 | 3.10E-13 | TV | 0.017 | 0.010 | 0.021 | 0.014 | 0.011 | 0.014 | 0.020 | 0.012 | 0.014 | 0.015 |
| rs4814873 | 20 | 19616429 | C | T | 0.76659 | 0.010 | 0.002 | 2.90E-09 | 0.009 | 0.003 | 4.80E-04 | smoking | -0.006 | 0.012 | -0.005 | 0.016 | -0.009 | 0.016 | 0.000 | 0.014 | -0.018 | 0.018 |
| rs6141814 | 20 | 31368960 | C | A | 0.613 | -0.007 | 0.001 | 1.50E-06 | -0.014 | 0.002 | 1.30E-09 | TV | 0.013 | 0.010 | 0.020 | 0.014 | 0.004 | 0.014 | 0.015 | 0.012 | 0.003 | 0.016 |
| rs55700114 | 20 | 43717080 | G | A | 0.713 | -0.003 | 0.002 | 3.00E-02 | -0.014 | 0.002 | 2.10E-09 | TV | -0.001 | 0.011 | -0.006 | 0.015 | 0.006 | 0.015 | -0.002 | 0.013 | -0.006 | 0.016 |
| rs56103247 | 20 | 62483184 | C | T | 0.944 | 0.017 | 0.003 | 4.60E-08 | 0.030 | 0.005 | 3.80E-10 | TV | -0.077 | 0.024 | -0.075 | 0.035 | -0.080 | 0.034 | -0.074 | 0.029 | -0.074 | 0.038 |
| rs10427502 | 21 | 40654840 | G | A | 0.622 | -0.005 | 0.001 | 1.50E-04 | 0.014 | 0.002 | 8.60E-10 | TV | 0.001 | 0.010 | 0.024 | 0.014 | -0.017 | 0.014 | -0.006 | 0.012 | 0.000 | 0.016 |
| rs2838834 | 21 | 46665208 | C | T | 0.699459 | -0.009 | 0.002 | 6.30E-10 | -0.001 | 0.002 | 5.30E-01 | smoking | -0.002 | 0.010 | -0.005 | 0.015 | -0.002 | 0.015 | -0.001 | 0.013 | -0.014 | 0.017 |
| rs2092829 | 22 | 31780514 | G | A | 0.706 | 0.003 | 0.002 | 6.60E-02 | 0.014 | 0.002 | 1.30E-08 | TV | -0.023 | 0.011 | -0.027 | 0.015 | -0.016 | 0.015 | -0.026 | 0.013 | -0.029 | 0.017 |
| rs202645 | 22 | 41798520 | A | G | 0.202939 | -0.010 | 0.002 | 3.90E-09 | 0.005 | 0.003 | 6.50E-02 | smoking | 0.001 | 0.012 | -0.012 | 0.017 | 0.018 | 0.017 | 0.002 | 0.014 | 0.003 | 0.019 |

| **Table S26. Betas of the bidirectional MR between alcohol and leisure computer use** | | | | | | | | | | | |
| --- | --- | --- | --- | --- | --- | --- | --- | --- | --- | --- | --- |
| 1) MR of alcohol consumption on leisure computer use | | | | | | | | | | | |
| SNP | Chr | Position | EA | OA | beta_alc | se_alc | pval_alc | beta_pc | se_pc | pval_pc |  |
| rs705687 | 1 | 4548453 | G | A | -0.011 | 0.002 | 8.15E-10 | 0.005 | 0.003 | 8.60E-02 |  |
| rs58107686 | 1 | 33837334 | A | C | -0.010 | 0.002 | 7.79E-10 | 0.000 | 0.002 | 9.40E-01 |  |
| rs12088813 | 1 | 66407700 | C | A | -0.009 | 0.002 | 1.58E-08 | 0.004 | 0.002 | 7.30E-02 |  |
| rs5024204 | 1 | 71491890 | T | A | 0.010 | 0.002 | 2.55E-09 | 0.002 | 0.002 | 5.10E-01 |  |
| rs10753661 | 1 | 165119792 | A | G | -0.009 | 0.002 | 3.76E-08 | 0.003 | 0.002 | 1.80E-01 |  |
| rs28680958 | 1 | 173848808 | A | G | -0.011 | 0.002 | 5.13E-10 | -0.005 | 0.003 | 4.50E-02 |  |
| rs823114 | 1 | 205719532 | A | G | 0.009 | 0.001 | 2.31E-09 | -0.005 | 0.002 | 1.70E-02 |  |
| rs77165542 | 2 | 430975 | T | C | -0.026 | 0.004 | 5.63E-11 | -0.013 | 0.006 | 3.60E-02 |  |
| rs1260326 | 2 | 27730940 | C | T | 0.021 | 0.001 | 8.05E-45 | 0.001 | 0.002 | 5.40E-01 |  |
| rs2178197 | 2 | 27860551 | G | A | -0.009 | 0.001 | 2.45E-09 | -0.003 | 0.002 | 2.00E-01 |  |
| rs13383034 | 2 | 45155276 | T | C | 0.015 | 0.002 | 6.31E-22 | 0.000 | 0.002 | 8.60E-01 |  |
| rs1004787 | 2 | 45159091 | A | G | 0.008 | 0.001 | 8.40E-09 | 0.002 | 0.002 | 3.90E-01 |  |
| rs13032049 | 2 | 63581507 | G | A | 0.010 | 0.002 | 3.00E-10 | 0.002 | 0.002 | 3.40E-01 |  |
| rs828867 | 2 | 74334462 | A | G | 0.009 | 0.001 | 2.15E-09 | 0.002 | 0.002 | 4.30E-01 |  |
| rs11692435 | 2 | 98275354 | A | G | 0.017 | 0.003 | 2.53E-11 | -0.010 | 0.004 | 1.70E-02 |  |
| rs13024996 | 2 | 144225215 | A | C | -0.011 | 0.002 | 5.72E-13 | 0.003 | 0.002 | 1.60E-01 |  |
| rs72859280 | 2 | 147956293 | T | G | 0.023 | 0.004 | 4.44E-09 | -0.007 | 0.006 | 2.20E-01 |  |
| rs56337305 | 2 | 225475560 | C | T | -0.010 | 0.001 | 1.63E-10 | 0.000 | 0.002 | 8.40E-01 |  |
| rs13094887 | 3 | 70968431 | T | A | -0.010 | 0.002 | 8.57E-11 | 0.000 | 0.002 | 9.10E-01 |  |
| rs62250685 | 3 | 85457240 | G | A | -0.014 | 0.002 | 1.05E-21 | -0.005 | 0.002 | 4.10E-02 |  |
| rs74664784 | 3 | 85475292 | C | T | -0.013 | 0.002 | 1.58E-14 | 0.149 | 0.063 | 1.80E-02 |  |
| rs13066454 | 3 | 93994255 | T | C | -0.009 | 0.001 | 4.13E-09 | 0.005 | 0.002 | 2.30E-02 |  |
| rs9838144 | 3 | 131576287 | C | G | -0.010 | 0.002 | 2.65E-08 | -0.002 | 0.003 | 3.90E-01 |  |
| rs2011092 | 3 | 141124607 | C | T | -0.009 | 0.002 | 7.35E-09 | 0.011 | 0.002 | 2.40E-06 |  |
| rs60654199 | 3 | 141267295 | A | C | -0.017 | 0.003 | 2.85E-08 | 0.006 | 0.004 | 2.10E-01 |  |
| rs6787172 | 3 | 158187811 | G | T | -0.008 | 0.001 | 4.27E-08 | -0.001 | 0.002 | 7.40E-01 |  |
| rs3748034 | 4 | 3446091 | T | G | -0.012 | 0.002 | 1.67E-08 | -0.006 | 0.003 | 8.10E-02 |  |
| rs7682824 | 4 | 39406254 | T | C | 0.008 | 0.002 | 2.77E-08 | 0.006 | 0.003 | 4.30E-02 |  |
| rs11940694 | 4 | 39414993 | G | A | 0.026 | 0.001 | 3.03E-68 | -0.010 | 0.002 | 1.40E-05 |  |
| rs35538052 | 4 | 39418965 | A | G | -0.009 | 0.002 | 1.39E-08 | 0.011 | 0.002 | 2.60E-06 |  |
| rs4501255 | 4 | 42151306 | G | C | 0.011 | 0.002 | 4.83E-10 | 0.002 | 0.003 | 5.00E-01 |  |
| rs12499107 | 4 | 99678691 | G | A | 0.013 | 0.002 | 4.45E-09 | -0.008 | 0.003 | 1.20E-02 |  |
| rs144198753 | 4 | 99713350 | T | C | -0.042 | 0.006 | 1.35E-12 | 0.035 | 0.012 | 3.10E-03 |  |
| rs1154414 | 4 | 100000136 | C | T | 0.018 | 0.002 | 3.74E-17 | 0.004 | 0.003 | 1.80E-01 |  |
| rs1229984 | 4 | 100239319 | C | T | 0.151 | 0.004 | 0.00E+00 | -0.031 | 0.007 | 2.40E-05 |  |
| rs10028756 | 4 | 100254520 | A | G | -0.019 | 0.002 | 1.16E-17 | 0.008 | 0.003 | 1.20E-02 |  |
| rs561222871 | 4 | 100260679 | T | C | -0.039 | 0.004 | 6.56E-27 | 0.002 | 0.005 | 6.20E-01 |  |
| rs36052336 | 4 | 100273594 | G | A | -0.018 | 0.003 | 1.23E-09 | -0.003 | 0.005 | 5.70E-01 |  |
| rs2165670 | 4 | 100286085 | A | G | 0.023 | 0.002 | 1.67E-22 | -0.009 | 0.004 | 1.00E-02 |  |
| rs17029090 | 4 | 100443853 | G | A | -0.049 | 0.005 | 4.75E-21 | 0.007 | 0.008 | 3.60E-01 |  |
| rs79139602 | 4 | 100444363 | T | A | 0.060 | 0.005 | 1.80E-32 | 0.007 | 0.008 | 3.80E-01 |  |
| rs4699791 | 4 | 101243023 | A | G | 0.019 | 0.002 | 6.58E-14 | -0.003 | 0.004 | 4.00E-01 |  |
| rs13107325 | 4 | 103188709 | T | C | -0.028 | 0.003 | 1.53E-22 | 0.001 | 0.004 | 7.60E-01 |  |
| rs4690727 | 4 | 143648579 | G | C | 0.011 | 0.002 | 2.43E-11 | -0.005 | 0.002 | 3.20E-02 |  |
| rs10004020 | 4 | 152968372 | A | G | 0.009 | 0.002 | 2.43E-08 | 0.006 | 0.002 | 1.00E-02 |  |
| rs12651313 | 4 | 171086393 | G | C | -0.009 | 0.001 | 3.79E-09 | -0.001 | 0.002 | 5.20E-01 |  |
| rs4916723 | 5 | 87854395 | C | A | -0.010 | 0.001 | 1.72E-11 | -0.011 | 0.002 | 6.20E-07 |  |
| rs12655091 | 5 | 144412335 | A | G | -0.008 | 0.001 | 1.25E-08 | 0.008 | 0.002 | 2.40E-04 |  |
| rs55872084 | 5 | 155902003 | T | G | 0.010 | 0.002 | 6.32E-09 | 0.000 | 0.003 | 1.00E+00 |  |
| rs11739827 | 5 | 166803321 | T | G | -0.008 | 0.001 | 1.18E-08 | 0.006 | 0.002 | 7.50E-03 |  |
| rs10085696 | 7 | 69783020 | G | A | -0.011 | 0.002 | 1.12E-09 | -0.012 | 0.003 | 3.30E-05 |  |
| rs6460047 | 7 | 73042443 | C | T | 0.012 | 0.002 | 9.69E-11 | -0.006 | 0.003 | 1.80E-02 |  |
| rs10236149 | 7 | 98977515 | G | A | -0.013 | 0.002 | 1.18E-09 | -0.013 | 0.003 | 5.50E-05 |  |
| rs35034355 | 7 | 103840115 | A | G | -0.008 | 0.001 | 2.87E-08 | -0.003 | 0.002 | 1.80E-01 |  |
| rs6951574 | 7 | 153489744 | C | T | 0.013 | 0.001 | 1.58E-19 | -0.002 | 0.002 | 4.40E-01 |  |
| rs13250583 | 8 | 20949917 | T | C | -0.010 | 0.002 | 4.70E-08 | -0.006 | 0.003 | 2.10E-02 |  |
| rs1217091 | 8 | 64527399 | C | T | 0.012 | 0.002 | 7.05E-11 | -0.005 | 0.003 | 6.30E-02 |  |
| rs28601761 | 8 | 126500031 | G | C | 0.009 | 0.001 | 7.17E-10 | -0.001 | 0.002 | 7.00E-01 |  |
| rs55932213 | 9 | 108755622 | G | A | 0.009 | 0.002 | 9.55E-09 | -0.004 | 0.003 | 9.70E-02 |  |
| rs10978550 | 9 | 109345993 | C | T | -0.012 | 0.002 | 7.15E-11 | 0.004 | 0.003 | 1.40E-01 |  |
| rs7074871 | 10 | 110507806 | A | G | -0.009 | 0.002 | 1.86E-08 | 0.005 | 0.003 | 2.90E-02 |  |
| rs17665139 | 10 | 125093880 | T | C | -0.012 | 0.002 | 1.59E-08 | 0.001 | 0.003 | 7.20E-01 |  |
| rs7950166 | 11 | 8642218 | T | C | -0.010 | 0.002 | 9.89E-11 | 0.000 | 0.002 | 1.00E+00 |  |
| rs11030084 | 11 | 27643725 | T | C | -0.011 | 0.002 | 1.72E-08 | -0.012 | 0.003 | 3.70E-05 |  |
| rs56030824 | 11 | 47397353 | A | G | -0.012 | 0.002 | 1.15E-13 | 0.010 | 0.002 | 3.80E-05 |  |
| rs10750025 | 11 | 113424042 | T | C | 0.010 | 0.002 | 4.89E-11 | -0.003 | 0.002 | 1.40E-01 |  |
| rs1713676 | 11 | 113660576 | G | A | -0.008 | 0.001 | 4.29E-08 | -0.002 | 0.002 | 2.70E-01 |  |
| rs4938230 | 11 | 116075001 | A | C | 0.013 | 0.002 | 1.48E-10 | -0.002 | 0.003 | 4.30E-01 |  |
| rs682011 | 11 | 121544285 | C | T | 0.008 | 0.001 | 2.22E-08 | -0.003 | 0.002 | 2.20E-01 |  |
| rs12795042 | 11 | 133658168 | C | A | -0.008 | 0.002 | 3.25E-08 | -0.001 | 0.002 | 8.00E-01 |  |
| rs10876188 | 12 | 51895882 | T | C | -0.008 | 0.001 | 4.84E-08 | 0.003 | 0.002 | 2.40E-01 |  |
| rs3809162 | 12 | 54674235 | G | A | 0.009 | 0.001 | 1.19E-09 | 0.004 | 0.002 | 8.30E-02 |  |
| rs10506274 | 12 | 81601464 | T | G | -0.009 | 0.001 | 5.78E-10 | 0.005 | 0.002 | 1.40E-02 |  |
| rs4842786 | 12 | 92170791 | A | G | -0.009 | 0.001 | 2.73E-09 | -0.004 | 0.002 | 9.70E-02 |  |
| rs500321 | 13 | 27124360 | T | A | -0.010 | 0.002 | 4.92E-09 | 0.000 | 0.002 | 9.00E-01 |  |
| rs1123285 | 14 | 57274519 | G | C | -0.009 | 0.002 | 8.14E-09 | 0.006 | 0.002 | 6.60E-03 |  |
| rs2180870 | 14 | 58782779 | C | T | -0.012 | 0.002 | 1.12E-08 | -0.005 | 0.003 | 1.60E-01 |  |
| rs28929474 | 14 | 94844947 | T | C | -0.037 | 0.005 | 1.34E-11 | 0.016 | 0.008 | 3.60E-02 |  |
| rs11625650 | 14 | 104610138 | A | G | -0.010 | 0.002 | 2.89E-08 | -0.002 | 0.003 | 4.30E-01 |  |
| rs2472297 | 15 | 75027880 | T | C | 0.011 | 0.002 | 3.10E-10 | -0.007 | 0.002 | 7.50E-03 |  |
| rs12907323 | 15 | 86796012 | G | A | 0.008 | 0.001 | 9.93E-09 | 0.004 | 0.002 | 5.20E-02 |  |
| rs2764771 | 16 | 20013793 | A | G | 0.010 | 0.002 | 4.02E-10 | -0.002 | 0.002 | 5.30E-01 |  |
| rs17177078 | 16 | 24810681 | T | C | -0.022 | 0.003 | 1.27E-13 | 0.004 | 0.005 | 4.10E-01 |  |
| rs378421 | 16 | 28754684 | A | G | -0.011 | 0.001 | 4.83E-14 | -0.005 | 0.002 | 2.90E-02 |  |
| rs113443718 | 16 | 29892184 | A | G | -0.010 | 0.002 | 1.19E-10 | -0.002 | 0.002 | 4.00E-01 |  |
| rs62044525 | 16 | 64872590 | G | C | -0.012 | 0.002 | 1.03E-10 | 0.002 | 0.003 | 3.80E-01 |  |
| rs7185555 | 16 | 69131281 | C | G | -0.011 | 0.002 | 4.24E-08 | -0.007 | 0.003 | 3.00E-02 |  |
| rs79616692 | 16 | 72338507 | C | G | 0.016 | 0.002 | 4.11E-12 | 0.004 | 0.004 | 2.90E-01 |  |
| rs1104608 | 16 | 73912588 | C | G | -0.011 | 0.001 | 1.05E-13 | 0.008 | 0.002 | 2.50E-04 |  |
| rs4548913 | 17 | 2209888 | A | G | -0.008 | 0.002 | 3.11E-08 | -0.002 | 0.002 | 3.20E-01 |  |
| rs3803800 | 17 | 7462969 | G | A | 0.011 | 0.002 | 1.50E-10 | 0.007 | 0.003 | 7.90E-03 |  |
| rs2854334 | 17 | 29715500 | G | A | 0.009 | 0.001 | 7.51E-10 | -0.002 | 0.002 | 3.40E-01 |  |
| rs10438820 | 17 | 78524597 | T | C | 0.009 | 0.002 | 1.76E-08 | 0.002 | 0.002 | 3.80E-01 |  |
| rs9950000 | 18 | 53052169 | T | C | -0.009 | 0.001 | 9.38E-10 | -0.006 | 0.002 | 6.90E-03 |  |
| rs4092465 | 18 | 55080437 | G | A | -0.008 | 0.002 | 4.39E-08 | 0.000 | 0.002 | 8.50E-01 |  |
| rs281379 | 19 | 49214274 | A | G | 0.014 | 0.001 | 4.91E-21 | 0.004 | 0.002 | 1.00E-01 |  |
| rs4815364 | 20 | 25035711 | A | G | 0.009 | 0.001 | 1.02E-08 | -0.001 | 0.002 | 5.80E-01 |  |
| rs9607814 | 22 | 41946519 | A | C | -0.010 | 0.002 | 4.31E-08 | 0.005 | 0.003 | 8.80E-02 |  |
| 2) MR of leisure computer use on alcohol consumption | | | | | | | | | | | |
| SNP | Chr | Position | EA | OA | beta_pc | se_pc | pval_pc | beta_alc | se_alc | pval_alc |  |
| rs2748985 | 1 | 1853184 | T | C | -0.015 | 0.002 | 4.10E-12 | 0.002 | 0.002 | 2.94E-01 |  |
| rs12145677 | 1 | 110023610 | G | A | -0.017 | 0.002 | 1.20E-12 | 0.005 | 0.002 | 2.43E-02 |  |
| rs7526456 | 1 | 197773734 | T | C | -0.013 | 0.002 | 1.20E-08 | 0.000 | 0.002 | 9.48E-01 |  |
| rs10754920 | 2 | 146456888 | C | G | 0.020 | 0.003 | 6.70E-09 | 0.004 | 0.003 | 1.53E-01 |  |
| rs6744254 | 2 | 155986195 | C | T | -0.016 | 0.002 | 5.40E-13 | -0.003 | 0.002 | 1.11E-01 |  |
| rs11274218 | 2 | 215035001 | C | T | -0.014 | 0.002 | 2.50E-09 | -0.004 | 0.002 | 6.92E-02 |  |
| rs4592851 | 2 | 215364232 | G | T | -0.014 | 0.003 | 1.40E-08 | -0.004 | 0.002 | 5.09E-02 |  |
| rs10208088 | 2 | 221055873 | C | T | 0.013 | 0.002 | 4.40E-09 | 0.000 | 0.002 | 8.85E-01 |  |
| rs11708955 | 3 | 49540114 | T | C | -0.016 | 0.002 | 4.20E-11 | 0.000 | 0.002 | 9.85E-01 |  |
| rs78082503 | 3 | 116556913 | G | C | -0.015 | 0.003 | 1.10E-08 | 0.001 | 0.002 | 7.55E-01 |  |
| rs6857629 | 4 | 39684977 | G | A | 0.015 | 0.003 | 1.90E-09 | -0.001 | 0.002 | 5.62E-01 |  |
| rs2068625 | 4 | 159856739 | T | C | -0.016 | 0.002 | 4.00E-11 | 0.003 | 0.002 | 1.14E-01 |  |
| rs2220599 | 5 | 7378854 | C | G | -0.016 | 0.002 | 2.40E-12 | 0.000 | 0.002 | 8.88E-01 |  |
| rs11749912 | 5 | 88065628 | A | G | 0.014 | 0.002 | 4.90E-10 | 0.003 | 0.002 | 1.59E-01 |  |
| rs1469249 | 5 | 113837198 | G | A | 0.016 | 0.003 | 9.50E-09 | 0.002 | 0.002 | 4.19E-01 |  |
| rs162894 | 5 | 131611872 | T | G | 0.013 | 0.002 | 1.10E-08 | 0.001 | 0.002 | 6.32E-01 |  |
| rs56158102 | 5 | 144027803 | C | T | 0.013 | 0.002 | 3.10E-08 | -0.003 | 0.002 | 1.95E-01 |  |
| rs9477970 | 6 | 19082663 | T | A | -0.016 | 0.003 | 1.00E-08 | 0.000 | 0.002 | 9.08E-01 |  |
| rs2179517 | 6 | 26198845 | G | C | -0.012 | 0.002 | 1.90E-08 | 0.000 | 0.002 | 8.38E-01 |  |
| rs9372625 | 6 | 98344031 | G | A | -0.018 | 0.002 | 5.70E-16 | -0.008 | 0.002 | 2.17E-05 |  |
| rs17789218 | 6 | 100600097 | T | C | -0.015 | 0.003 | 4.20E-09 | 0.003 | 0.002 | 2.33E-01 |  |
| rs6935828 | 6 | 140811367 | C | T | -0.012 | 0.002 | 4.30E-08 | 0.004 | 0.002 | 3.72E-02 |  |
| rs55772938 | 7 | 70025113 | A | G | -0.015 | 0.002 | 3.90E-10 | -0.003 | 0.002 | 1.37E-01 |  |
| rs12706626 | 7 | 124531370 | G | A | -0.013 | 0.002 | 1.40E-08 | 0.000 | 0.002 | 8.44E-01 |  |
| rs3944151 | 7 | 126970005 | A | G | 0.014 | 0.002 | 3.10E-08 | -0.003 | 0.002 | 2.24E-01 |  |
| rs2345941 | 7 | 133337635 | A | G | 0.015 | 0.002 | 4.00E-11 | -0.002 | 0.002 | 3.62E-01 |  |
| rs13262595 | 8 | 143316970 | A | G | -0.016 | 0.002 | 1.20E-12 | -0.005 | 0.002 | 1.45E-02 |  |
| rs4977839 | 9 | 23355310 | G | A | -0.020 | 0.002 | 4.70E-19 | 0.000 | 0.002 | 9.45E-01 |  |
| rs113851275 | 9 | 98297220 | G | A | -0.021 | 0.004 | 3.20E-09 | 0.004 | 0.003 | 1.75E-01 |  |
| rs7020477 | 9 | 116827760 | A | G | 0.014 | 0.002 | 1.20E-08 | -0.002 | 0.002 | 3.27E-01 |  |
| rs73578186 | 9 | 126334485 | C | T | 0.015 | 0.002 | 2.30E-10 | 0.002 | 0.002 | 4.03E-01 |  |
| rs2734849 | 11 | 113270160 | A | G | -0.014 | 0.002 | 8.20E-10 | 0.011 | 0.002 | 6.73E-09 |  |
| rs1448355 | 11 | 131286685 | C | T | -0.015 | 0.002 | 1.30E-10 | 0.000 | 0.002 | 9.03E-01 |  |
| rs35933007 | 12 | 38929341 | G | A | -0.015 | 0.003 | 9.20E-09 | 0.010 | 0.002 | 1.10E-05 |  |
| rs1319734 | 12 | 40367517 | A | G | 0.013 | 0.002 | 1.90E-08 | -0.002 | 0.002 | 3.74E-01 |  |
| rs206965 | 12 | 120856332 | T | C | 0.016 | 0.003 | 9.10E-09 | -0.003 | 0.002 | 2.90E-01 |  |
| rs12874776 | 13 | 57794345 | C | A | -0.023 | 0.004 | 8.00E-11 | -0.002 | 0.003 | 4.62E-01 |  |
| rs198262 | 14 | 57283283 | C | T | 0.030 | 0.005 | 3.90E-08 | -0.012 | 0.005 | 9.85E-03 |  |
| rs166835 | 15 | 47716037 | C | T | 0.013 | 0.002 | 3.40E-09 | 0.005 | 0.002 | 1.69E-02 |  |
| rs4702 | 15 | 91426560 | G | A | 0.012 | 0.002 | 3.80E-08 | 0.005 | 0.002 | 1.34E-02 |  |
| rs6498759 | 16 | 18020295 | T | C | 0.013 | 0.002 | 2.70E-08 | 0.003 | 0.002 | 1.48E-01 |  |
| rs3730399 | 16 | 67229019 | A | G | 0.025 | 0.004 | 3.30E-08 | -0.002 | 0.004 | 6.45E-01 |  |
| rs4073003 | 17 | 19148305 | A | G | 0.020 | 0.003 | 1.20E-09 | -0.001 | 0.003 | 8.61E-01 |  |
| rs7209653 | 17 | 19882084 | T | C | 0.016 | 0.002 | 9.10E-11 | 0.001 | 0.002 | 5.16E-01 |  |
| rs12603813 | 17 | 43196584 | T | C | 0.014 | 0.003 | 1.30E-08 | -0.002 | 0.002 | 3.69E-01 |  |
| rs66643547 | 17 | 79375315 | C | T | -0.015 | 0.002 | 3.20E-11 | 0.001 | 0.002 | 4.71E-01 |  |
| rs784256 | 18 | 53398626 | G | A | 0.019 | 0.003 | 6.20E-11 | 0.006 | 0.002 | 2.39E-02 |  |
| rs6129084 | 20 | 59828857 | A | T | -0.014 | 0.002 | 9.30E-10 | -0.007 | 0.002 | 5.53E-04 |  |
| rs9608249 | 22 | 24613952 | G | A | 0.017 | 0.003 | 2.20E-08 | -0.003 | 0.003 | 2.24E-01 |  |
| rs136553 | 22 | 27255675 | C | T | -0.015 | 0.002 | 2.30E-11 | 0.003 | 0.002 | 9.73E-02 |  |
| rs7288455 | 22 | 39966547 | A | G | 0.013 | 0.002 | 4.00E-09 | 0.000 | 0.002 | 8.19E-01 |  |

| **Table S27. Betas of the bidirectional MR between alcohol and leisure television use** | | | | | | | | | | |
| --- | --- | --- | --- | --- | --- | --- | --- | --- | --- | --- |
| 1) MR of alcohol consumption on leisure television use | | | | | | | | | | |
| SNP | Chr | Position | EA | OA | beta_alc | se_alc | pval_alc | beta_tv | se_tv | pval_tv |
| rs705687 | 1 | 4548453 | G | A | -0.011 | 0.002 | 8.15E-10 | 0.0016 | 0.0026 | 5.30E-01 |
| rs58107686 | 1 | 33837334 | A | C | -0.010 | 0.002 | 7.79E-10 | 0.0011 | 0.0023 | 6.40E-01 |
| rs12088813 | 1 | 66407700 | C | A | -0.009 | 0.002 | 1.58E-08 | -0.0030 | 0.0024 | 2.10E-01 |
| rs5024204 | 1 | 71491890 | T | A | 0.010 | 0.002 | 2.55E-09 | -0.0077 | 0.0024 | 1.70E-03 |
| rs10753661 | 1 | 165119792 | A | G | -0.009 | 0.002 | 3.76E-08 | 0.0024 | 0.0023 | 3.00E-01 |
| rs28680958 | 1 | 173848808 | A | G | -0.011 | 0.002 | 5.13E-10 | -0.0002 | 0.0026 | 9.50E-01 |
| rs823114 | 1 | 205719532 | A | G | 0.009 | 0.001 | 2.31E-09 | -0.0015 | 0.0022 | 5.00E-01 |
| rs77165542 | 2 | 430975 | T | C | -0.026 | 0.004 | 5.63E-11 | 0.0032 | 0.0059 | 5.90E-01 |
| rs1260326 | 2 | 27730940 | C | T | 0.021 | 0.001 | 8.05E-45 | -0.0060 | 0.0022 | 6.60E-03 |
| rs2178197 | 2 | 27860551 | G | A | -0.009 | 0.001 | 2.45E-09 | -0.0075 | 0.0022 | 6.30E-04 |
| rs13383034 | 2 | 45155276 | T | C | 0.015 | 0.002 | 6.31E-22 | 0.0002 | 0.0023 | 9.30E-01 |
| rs1004787 | 2 | 45159091 | A | G | 0.008 | 0.001 | 8.40E-09 | 0.0017 | 0.0022 | 4.40E-01 |
| rs13032049 | 2 | 63581507 | G | A | 0.010 | 0.002 | 3.00E-10 | -0.0010 | 0.0024 | 6.80E-01 |
| rs828867 | 2 | 74334462 | A | G | 0.009 | 0.001 | 2.15E-09 | 0.0013 | 0.0022 | 5.70E-01 |
| rs11692435 | 2 | 98275354 | A | G | 0.017 | 0.003 | 2.53E-11 | 0.0213 | 0.0040 | 1.40E-07 |
| rs13024996 | 2 | 144225215 | A | C | -0.011 | 0.002 | 5.72E-13 | -0.0061 | 0.0022 | 6.00E-03 |
| rs72859280 | 2 | 147956293 | T | G | 0.023 | 0.004 | 4.44E-09 | -0.0050 | 0.0058 | 3.80E-01 |
| rs56337305 | 2 | 225475560 | C | T | -0.010 | 0.001 | 1.63E-10 | 0.0018 | 0.0022 | 4.30E-01 |
| rs13094887 | 3 | 70968431 | T | A | -0.010 | 0.002 | 8.57E-11 | -0.0089 | 0.0024 | 1.50E-04 |
| rs62250685 | 3 | 85457240 | G | A | -0.014 | 0.002 | 1.05E-21 | -0.0054 | 0.0022 | 1.60E-02 |
| rs74664784 | 3 | 85475292 | C | T | -0.013 | 0.002 | 1.58E-14 | 0.0456 | 0.0617 | 4.60E-01 |
| rs13066454 | 3 | 93994255 | T | C | -0.009 | 0.001 | 4.13E-09 | -0.0125 | 0.0022 | 1.30E-08 |
| rs9838144 | 3 | 131576287 | C | G | -0.010 | 0.002 | 2.65E-08 | 0.0046 | 0.0027 | 8.80E-02 |
| rs2011092 | 3 | 141124607 | C | T | -0.009 | 0.002 | 7.35E-09 | -0.0037 | 0.0023 | 1.00E-01 |
| rs60654199 | 3 | 141267295 | A | C | -0.017 | 0.003 | 2.85E-08 | 0.0134 | 0.0043 | 1.90E-03 |
| rs6787172 | 3 | 158187811 | G | T | -0.008 | 0.001 | 4.27E-08 | 0.0007 | 0.0022 | 7.40E-01 |
| rs3748034 | 4 | 3446091 | T | G | -0.012 | 0.002 | 1.67E-08 | 0.0092 | 0.0031 | 3.00E-03 |
| rs7682824 | 4 | 39406254 | T | C | 0.008 | 0.002 | 2.77E-08 | -0.0073 | 0.0031 | 1.70E-02 |
| rs11940694 | 4 | 39414993 | G | A | 0.026 | 0.001 | 3.03E-68 | 0.0037 | 0.0022 | 9.50E-02 |
| rs35538052 | 4 | 39418965 | A | G | -0.009 | 0.002 | 1.39E-08 | -0.0031 | 0.0022 | 1.70E-01 |
| rs4501255 | 4 | 42151306 | G | C | 0.011 | 0.002 | 4.83E-10 | 0.0102 | 0.0025 | 5.50E-05 |
| rs12499107 | 4 | 99678691 | G | A | 0.013 | 0.002 | 4.45E-09 | 0.0017 | 0.0032 | 5.90E-01 |
| rs144198753 | 4 | 99713350 | T | C | -0.042 | 0.006 | 1.35E-12 | -0.0180 | 0.0116 | 1.20E-01 |
| rs1154414 | 4 | 100000136 | C | T | 0.018 | 0.002 | 3.74E-17 | -0.0072 | 0.0032 | 2.50E-02 |
| rs1229984 | 4 | 100239319 | C | T | 0.151 | 0.004 | 0.00E+00 | 0.0226 | 0.0073 | 2.00E-03 |
| rs10028756 | 4 | 100254520 | A | G | -0.019 | 0.002 | 1.16E-17 | -0.0064 | 0.0032 | 4.30E-02 |
| rs561222871 | 4 | 100260679 | T | C | -0.039 | 0.004 | 6.56E-27 | -0.0061 | 0.0045 | 1.70E-01 |
| rs36052336 | 4 | 100273594 | G | A | -0.018 | 0.003 | 1.23E-09 | 0.0023 | 0.0044 | 6.10E-01 |
| rs2165670 | 4 | 100286085 | A | G | 0.023 | 0.002 | 1.67E-22 | 0.0035 | 0.0036 | 3.30E-01 |
| rs17029090 | 4 | 100443853 | G | A | -0.049 | 0.005 | 4.75E-21 | -0.0039 | 0.0079 | 6.20E-01 |
| rs79139602 | 4 | 100444363 | T | A | 0.060 | 0.005 | 1.80E-32 | -0.0047 | 0.0077 | 5.40E-01 |
| rs4699791 | 4 | 101243023 | A | G | 0.019 | 0.002 | 6.58E-14 | -0.0090 | 0.0037 | 1.40E-02 |
| rs13107325 | 4 | 103188709 | T | C | -0.028 | 0.003 | 1.53E-22 | 0.0292 | 0.0041 | 1.50E-12 |
| rs4690727 | 4 | 143648579 | G | C | 0.011 | 0.002 | 2.43E-11 | 0.0007 | 0.0024 | 7.90E-01 |
| rs10004020 | 4 | 152968372 | A | G | 0.009 | 0.002 | 2.43E-08 | -0.0026 | 0.0024 | 2.90E-01 |
| rs12651313 | 4 | 171086393 | G | C | -0.009 | 0.001 | 3.79E-09 | 0.0064 | 0.0022 | 3.10E-03 |
| rs4916723 | 5 | 87854395 | C | A | -0.010 | 0.001 | 1.72E-11 | 0.0086 | 0.0022 | 1.10E-04 |
| rs12655091 | 5 | 144412335 | A | G | -0.008 | 0.001 | 1.25E-08 | 0.0045 | 0.0022 | 3.80E-02 |
| rs55872084 | 5 | 155902003 | T | G | 0.010 | 0.002 | 6.32E-09 | 0.0032 | 0.0025 | 2.10E-01 |
| rs11739827 | 5 | 166803321 | T | G | -0.008 | 0.001 | 1.18E-08 | 0.0015 | 0.0022 | 4.80E-01 |
| rs10085696 | 7 | 69783020 | G | A | -0.011 | 0.002 | 1.12E-09 | 0.0084 | 0.0028 | 2.60E-03 |
| rs6460047 | 7 | 73042443 | C | T | 0.012 | 0.002 | 9.69E-11 | -0.0002 | 0.0027 | 9.50E-01 |
| rs10236149 | 7 | 98977515 | G | A | -0.013 | 0.002 | 1.18E-09 | -0.0142 | 0.0033 | 1.40E-05 |
| rs35034355 | 7 | 103840115 | A | G | -0.008 | 0.001 | 2.87E-08 | -0.0050 | 0.0022 | 2.00E-02 |
| rs6951574 | 7 | 153489744 | C | T | 0.013 | 0.001 | 1.58E-19 | 0.0039 | 0.0022 | 7.20E-02 |
| rs13250583 | 8 | 20949917 | T | C | -0.010 | 0.002 | 4.70E-08 | 0.0022 | 0.0026 | 4.00E-01 |
| rs1217091 | 8 | 64527399 | C | T | 0.012 | 0.002 | 7.05E-11 | -0.0035 | 0.0028 | 2.00E-01 |
| rs28601761 | 8 | 126500031 | G | C | 0.009 | 0.001 | 7.17E-10 | 0.0009 | 0.0022 | 6.90E-01 |
| rs55932213 | 9 | 108755622 | G | A | 0.009 | 0.002 | 9.55E-09 | 0.0044 | 0.0025 | 8.40E-02 |
| rs10978550 | 9 | 109345993 | C | T | -0.012 | 0.002 | 7.15E-11 | -0.0017 | 0.0027 | 5.30E-01 |
| rs7074871 | 10 | 110507806 | A | G | -0.009 | 0.002 | 1.86E-08 | 0.0026 | 0.0025 | 2.90E-01 |
| rs17665139 | 10 | 125093880 | T | C | -0.012 | 0.002 | 1.59E-08 | -0.0064 | 0.0030 | 3.30E-02 |
| rs7950166 | 11 | 8642218 | T | C | -0.010 | 0.002 | 9.89E-11 | 0.0031 | 0.0023 | 1.70E-01 |
| rs11030084 | 11 | 27643725 | T | C | -0.011 | 0.002 | 1.72E-08 | 0.0038 | 0.0028 | 1.70E-01 |
| rs56030824 | 11 | 47397353 | A | G | -0.012 | 0.002 | 1.15E-13 | 0.0054 | 0.0023 | 2.00E-02 |
| rs10750025 | 11 | 113424042 | T | C | 0.010 | 0.002 | 4.89E-11 | -0.0047 | 0.0023 | 4.40E-02 |
| rs1713676 | 11 | 113660576 | G | A | -0.008 | 0.001 | 4.29E-08 | 0.0059 | 0.0022 | 6.30E-03 |
| rs4938230 | 11 | 116075001 | A | C | 0.013 | 0.002 | 1.48E-10 | 0.0040 | 0.0030 | 1.80E-01 |
| rs682011 | 11 | 121544285 | C | T | 0.008 | 0.001 | 2.22E-08 | 0.0079 | 0.0022 | 2.70E-04 |
| rs12795042 | 11 | 133658168 | C | A | -0.008 | 0.002 | 3.25E-08 | 0.0009 | 0.0023 | 6.70E-01 |
| rs10876188 | 12 | 51895882 | T | C | -0.008 | 0.001 | 4.84E-08 | -0.0058 | 0.0022 | 7.10E-03 |
| rs3809162 | 12 | 54674235 | G | A | 0.009 | 0.001 | 1.19E-09 | -0.0021 | 0.0022 | 3.40E-01 |
| rs10506274 | 12 | 81601464 | T | G | -0.009 | 0.001 | 5.78E-10 | 0.0028 | 0.0022 | 1.90E-01 |
| rs4842786 | 12 | 92170791 | A | G | -0.009 | 0.001 | 2.73E-09 | 0.0046 | 0.0022 | 3.70E-02 |
| rs500321 | 13 | 27124360 | T | A | -0.010 | 0.002 | 4.92E-09 | -0.0013 | 0.0024 | 5.90E-01 |
| rs1123285 | 14 | 57274519 | G | C | -0.009 | 0.002 | 8.14E-09 | -0.0058 | 0.0023 | 1.30E-02 |
| rs2180870 | 14 | 58782779 | C | T | -0.012 | 0.002 | 1.12E-08 | 0.0032 | 0.0032 | 3.20E-01 |
| rs28929474 | 14 | 94844947 | T | C | -0.037 | 0.005 | 1.34E-11 | -0.0031 | 0.0077 | 6.90E-01 |
| rs11625650 | 14 | 104610138 | A | G | -0.010 | 0.002 | 2.89E-08 | -0.0040 | 0.0025 | 1.10E-01 |
| rs2472297 | 15 | 75027880 | T | C | 0.011 | 0.002 | 3.10E-10 | 0.0029 | 0.0024 | 2.30E-01 |
| rs12907323 | 15 | 86796012 | G | A | 0.008 | 0.001 | 9.93E-09 | -0.0073 | 0.0022 | 1.10E-03 |
| rs2764771 | 16 | 20013793 | A | G | 0.010 | 0.002 | 4.02E-10 | 0.0019 | 0.0024 | 4.20E-01 |
| rs17177078 | 16 | 24810681 | T | C | -0.022 | 0.003 | 1.27E-13 | -0.0079 | 0.0047 | 9.30E-02 |
| rs378421 | 16 | 28754684 | A | G | -0.011 | 0.001 | 4.83E-14 | 0.0126 | 0.0022 | 1.20E-08 |
| rs113443718 | 16 | 29892184 | A | G | -0.010 | 0.002 | 1.19E-10 | -0.0023 | 0.0023 | 3.20E-01 |
| rs62044525 | 16 | 64872590 | G | C | -0.012 | 0.002 | 1.03E-10 | -0.0008 | 0.0028 | 7.70E-01 |
| rs7185555 | 16 | 69131281 | C | G | -0.011 | 0.002 | 4.24E-08 | -0.0064 | 0.0030 | 3.20E-02 |
| rs79616692 | 16 | 72338507 | C | G | 0.016 | 0.002 | 4.11E-12 | -0.0084 | 0.0035 | 1.70E-02 |
| rs1104608 | 16 | 73912588 | C | G | -0.011 | 0.001 | 1.05E-13 | -0.0043 | 0.0022 | 5.10E-02 |
| rs4548913 | 17 | 2209888 | A | G | -0.008 | 0.002 | 3.11E-08 | 0.0014 | 0.0023 | 5.30E-01 |
| rs3803800 | 17 | 7462969 | G | A | 0.011 | 0.002 | 1.50E-10 | -0.0015 | 0.0026 | 5.70E-01 |
| rs2854334 | 17 | 29715500 | G | A | 0.009 | 0.001 | 7.51E-10 | -0.0012 | 0.0022 | 6.00E-01 |
| rs10438820 | 17 | 78524597 | T | C | 0.009 | 0.002 | 1.76E-08 | -0.0029 | 0.0024 | 2.20E-01 |
| rs9950000 | 18 | 53052169 | T | C | -0.009 | 0.001 | 9.38E-10 | -0.0014 | 0.0022 | 5.30E-01 |
| rs4092465 | 18 | 55080437 | G | A | -0.008 | 0.002 | 4.39E-08 | 0.0005 | 0.0023 | 8.20E-01 |
| rs281379 | 19 | 49214274 | A | G | 0.014 | 0.001 | 4.91E-21 | 0.0013 | 0.0022 | 5.60E-01 |
| rs4815364 | 20 | 25035711 | A | G | 0.009 | 0.001 | 1.02E-08 | 0.0025 | 0.0022 | 2.60E-01 |
| rs9607814 | 22 | 41946519 | A | C | -0.010 | 0.002 | 4.31E-08 | 0.0060 | 0.0027 | 2.90E-02 |
| **2) MR of leisure television use on alcohol consumption** | | | | | | | | | | |
| SNP | Chr | Position | EA | OA | beta_tv | se_tv | pval_tv | beta_alc | se_alc | pval_alc |
| rs11810109 | 1 | 44186812 | A | T | 0.016 | 0.002 | 5.40E-12 | 0.003 | 0.002 | 1.44E-01 |
| rs112632642 | 1 | 51115634 | G | A | -0.024 | 0.004 | 3.60E-08 | 0.008 | 0.004 | 4.24E-02 |
| rs6670915 | 1 | 61109968 | C | T | 0.014 | 0.002 | 2.40E-10 | 0.007 | 0.002 | 5.43E-04 |
| rs12725114 | 1 | 62432641 | G | A | 0.015 | 0.003 | 1.60E-08 | 0.001 | 0.002 | 7.98E-01 |
| rs984409 | 1 | 67020440 | G | A | -0.015 | 0.002 | 4.10E-11 | -0.002 | 0.002 | 2.14E-01 |
| rs71658797 | 1 | 77967507 | T | A | -0.02 | 0.003 | 6.30E-10 | 0.003 | 0.003 | 3.40E-01 |
| rs114328297 | 1 | 91190854 | T | G | 0.014 | 0.003 | 4.40E-08 | 0.008 | 0.002 | 3.24E-04 |
| rs631130 | 1 | 96043010 | T | A | -0.014 | 0.002 | 5.00E-09 | 0.005 | 0.002 | 1.79E-02 |
| rs148648822 | 1 | 97815048 | G | T | -0.018 | 0.003 | 4.20E-09 | -0.003 | 0.003 | 2.50E-01 |
| rs17379561 | 1 | 98340139 | A | T | -0.025 | 0.003 | 1.10E-16 | -0.003 | 0.003 | 3.25E-01 |
| rs55909997 | 1 | 107581039 | G | A | -0.014 | 0.002 | 2.40E-09 | -0.002 | 0.002 | 3.61E-01 |
| rs1278847 | 1 | 110037483 | C | A | 0.016 | 0.002 | 1.20E-11 | 0.004 | 0.002 | 5.09E-02 |
| rs141425667 | 1 | 114930465 | G | A | -0.024 | 0.004 | 4.00E-08 | 0.001 | 0.004 | 8.61E-01 |
| rs4845364 | 1 | 154141908 | A | G | -0.015 | 0.002 | 1.30E-12 | -0.001 | 0.002 | 7.34E-01 |
| rs6673341 | 1 | 184655649 | T | G | -0.015 | 0.002 | 2.20E-11 | -0.001 | 0.002 | 5.32E-01 |
| rs72725224 | 1 | 188115858 | A | C | -0.046 | 0.008 | 3.70E-09 | -0.016 | 0.007 | 2.69E-02 |
| rs10737620 | 1 | 193057971 | T | A | 0.014 | 0.002 | 2.60E-09 | 0.001 | 0.002 | 6.71E-01 |
| rs11896615 | 2 | 17407744 | G | A | 0.017 | 0.003 | 3.10E-08 | -0.007 | 0.003 | 1.73E-02 |
| rs72781699 | 2 | 24277709 | G | A | -0.019 | 0.003 | 3.00E-12 | 0.007 | 0.002 | 3.84E-03 |
| rs12105701 | 2 | 41737200 | C | T | -0.013 | 0.002 | 5.40E-09 | 0.000 | 0.002 | 9.36E-01 |
| rs7564130 | 2 | 50606642 | T | C | -0.015 | 0.002 | 2.80E-11 | -0.005 | 0.002 | 1.89E-02 |
| rs11125515 | 2 | 53886799 | C | A | -0.014 | 0.002 | 2.20E-08 | 0.002 | 0.002 | 3.42E-01 |
| rs12475168 | 2 | 58992704 | G | T | -0.018 | 0.003 | 1.70E-08 | -0.001 | 0.003 | 6.19E-01 |
| rs183566425 | 2 | 59325356 | A | T | 0.046 | 0.008 | 1.90E-08 | -0.003 | 0.008 | 6.82E-01 |
| rs10189857 | 2 | 60713235 | A | G | -0.02 | 0.002 | 6.20E-21 | 0.006 | 0.002 | 1.57E-03 |
| rs62641636 | 2 | 68425427 | A | G | 0.014 | 0.002 | 7.00E-10 | 0.002 | 0.002 | 2.65E-01 |
| rs59210467 | 2 | 97097291 | C | T | -0.013 | 0.002 | 1.00E-08 | -0.002 | 0.002 | 2.41E-01 |
| rs11689199 | 2 | 100820421 | A | G | 0.018 | 0.002 | 5.50E-17 | 0.004 | 0.002 | 6.86E-02 |
| rs2916251 | 2 | 104447052 | G | T | -0.017 | 0.002 | 1.50E-13 | -0.003 | 0.002 | 1.15E-01 |
| rs1451533 | 2 | 105466005 | G | A | -0.016 | 0.002 | 1.10E-10 | 0.000 | 0.002 | 9.17E-01 |
| rs2971640 | 2 | 107624618 | G | A | -0.013 | 0.002 | 4.20E-09 | 0.003 | 0.002 | 1.80E-01 |
| rs12476388 | 2 | 116330983 | C | T | 0.013 | 0.002 | 2.10E-08 | 0.000 | 0.002 | 9.96E-01 |
| rs374722 | 2 | 147839830 | G | A | 0.024 | 0.003 | 5.50E-16 | -0.005 | 0.003 | 8.71E-02 |
| rs3754970 | 2 | 162091836 | T | C | -0.015 | 0.002 | 4.80E-12 | -0.006 | 0.002 | 1.14E-03 |
| rs1913808 | 2 | 162804678 | G | A | 0.015 | 0.002 | 3.90E-10 | 0.003 | 0.002 | 2.18E-01 |
| rs2060199 | 2 | 166196681 | T | A | 0.012 | 0.002 | 2.40E-08 | -0.003 | 0.002 | 1.82E-01 |
| rs263771 | 2 | 185921692 | C | A | -0.017 | 0.003 | 3.10E-11 | 0.000 | 0.002 | 8.38E-01 |
| rs4577309 | 2 | 191288833 | A | G | 0.016 | 0.002 | 1.60E-13 | -0.001 | 0.002 | 7.79E-01 |
| rs34224261 | 2 | 193855452 | G | C | -0.018 | 0.002 | 4.60E-16 | 0.004 | 0.002 | 3.27E-02 |
| rs4675246 | 2 | 202864487 | G | T | -0.015 | 0.003 | 2.90E-08 | -0.005 | 0.002 | 3.61E-02 |
| rs13029509 | 2 | 215374209 | G | A | -0.018 | 0.002 | 2.00E-17 | 0.001 | 0.002 | 5.73E-01 |
| rs10932837 | 2 | 221044785 | C | T | -0.013 | 0.002 | 1.30E-09 | 0.000 | 0.002 | 9.44E-01 |
| rs4973576 | 2 | 233806771 | C | A | -0.015 | 0.002 | 1.00E-09 | 0.005 | 0.002 | 3.73E-02 |
| rs138256022 | 3 | 11603090 | C | T | -0.031 | 0.006 | 2.90E-08 | 0.004 | 0.005 | 4.32E-01 |
| rs9834970 | 3 | 36856030 | T | C | 0.013 | 0.002 | 3.30E-09 | -0.001 | 0.002 | 5.90E-01 |
| rs1352890 | 3 | 49652156 | C | T | -0.012 | 0.002 | 1.30E-08 | 0.003 | 0.002 | 1.39E-01 |
| rs3796386 | 3 | 49899795 | G | A | -0.026 | 0.002 | 3.20E-33 | 0.001 | 0.002 | 5.67E-01 |
| rs182355396 | 3 | 50920457 | C | A | -0.08 | 0.013 | 2.10E-10 | 0.005 | 0.011 | 6.24E-01 |
| rs11917871 | 3 | 54233476 | T | C | -0.017 | 0.002 | 4.50E-14 | 0.000 | 0.002 | 8.26E-01 |
| rs11130793 | 3 | 60884659 | C | T | 0.013 | 0.002 | 5.00E-09 | -0.002 | 0.002 | 2.82E-01 |
| rs11714337 | 3 | 71582521 | G | A | 0.014 | 0.002 | 4.70E-11 | -0.006 | 0.002 | 3.41E-03 |
| rs12496084 | 3 | 75174777 | A | G | -0.014 | 0.003 | 4.60E-08 | 0.000 | 0.002 | 9.68E-01 |
| rs6797840 | 3 | 85656569 | A | C | -0.016 | 0.002 | 1.70E-13 | -0.007 | 0.002 | 3.54E-04 |
| rs56398417 | 3 | 88024986 | C | T | 0.014 | 0.002 | 5.90E-10 | 0.002 | 0.002 | 2.74E-01 |
| rs2034768 | 3 | 93986371 | A | G | 0.015 | 0.002 | 8.60E-12 | 0.005 | 0.002 | 6.70E-03 |
| rs12632778 | 3 | 106452359 | T | G | -0.028 | 0.005 | 3.90E-08 | 0.010 | 0.004 | 3.12E-02 |
| rs9867121 | 3 | 114631548 | C | A | 0.019 | 0.003 | 3.90E-12 | 0.004 | 0.002 | 9.28E-02 |
| rs12491503 | 3 | 165706855 | G | A | -0.014 | 0.002 | 5.50E-10 | 0.001 | 0.002 | 6.46E-01 |
| rs114600294 | 3 | 181419367 | G | C | -0.016 | 0.003 | 7.90E-10 | 0.006 | 0.002 | 2.37E-02 |
| rs66852340 | 4 | 3311070 | C | T | -0.018 | 0.003 | 7.90E-12 | 0.004 | 0.002 | 8.69E-02 |
| rs7700107 | 4 | 17880416 | A | C | -0.021 | 0.003 | 1.90E-11 | -0.002 | 0.003 | 4.30E-01 |
| rs34811474 | 4 | 25408838 | G | A | 0.015 | 0.003 | 2.30E-09 | -0.005 | 0.002 | 2.06E-02 |
| rs77215114 | 4 | 45187658 | A | T | 0.024 | 0.004 | 1.50E-08 | 0.003 | 0.004 | 4.87E-01 |
| rs28971796 | 4 | 49151982 | A | G | 0.015 | 0.002 | 2.10E-09 | -0.004 | 0.002 | 5.96E-02 |
| rs6850494 | 4 | 82291771 | A | C | -0.014 | 0.002 | 1.10E-10 | -0.001 | 0.002 | 6.62E-01 |
| rs13107325 | 4 | 103188709 | C | T | -0.029 | 0.004 | 1.50E-12 | 0.036 | 0.004 | 1.23E-20 |
| rs62332760 | 4 | 106135316 | G | T | -0.014 | 0.002 | 8.00E-11 | 0.007 | 0.002 | 2.60E-04 |
| rs7693703 | 4 | 118357405 | G | A | 0.023 | 0.004 | 2.70E-09 | -0.008 | 0.003 | 1.32E-02 |
| rs4334769 | 4 | 130245233 | G | T | 0.012 | 0.002 | 2.10E-08 | -0.003 | 0.002 | 1.23E-01 |
| rs6825241 | 4 | 152543783 | C | A | -0.017 | 0.002 | 4.90E-15 | 0.002 | 0.002 | 2.41E-01 |
| rs7693082 | 4 | 159857819 | G | C | 0.015 | 0.002 | 1.70E-10 | 0.003 | 0.002 | 1.08E-01 |
| rs10940659 | 5 | 59360341 | A | G | -0.013 | 0.002 | 1.50E-08 | 0.005 | 0.002 | 1.36E-02 |
| rs2409838 | 5 | 60204410 | A | T | 0.017 | 0.002 | 3.40E-15 | 0.001 | 0.002 | 6.10E-01 |
| rs262890 | 5 | 62930015 | A | G | -0.019 | 0.002 | 3.20E-15 | -0.003 | 0.002 | 1.60E-01 |
| rs7716447 | 5 | 88800331 | A | G | -0.013 | 0.002 | 3.60E-09 | 0.001 | 0.002 | 7.36E-01 |
| rs1031423 | 5 | 93276883 | T | C | -0.019 | 0.003 | 1.80E-12 | 0.003 | 0.002 | 2.70E-01 |
| rs57585211 | 5 | 107197725 | T | G | -0.017 | 0.003 | 4.30E-09 | 0.004 | 0.003 | 1.71E-01 |
| rs9326862 | 5 | 112054547 | A | T | 0.012 | 0.002 | 2.60E-08 | 0.002 | 0.002 | 2.05E-01 |
| rs10041724 | 5 | 124273520 | T | C | 0.018 | 0.003 | 3.90E-11 | -0.001 | 0.002 | 8.33E-01 |
| rs10057545 | 5 | 138431008 | G | A | -0.014 | 0.002 | 2.00E-10 | -0.004 | 0.002 | 4.54E-02 |
| rs62379379 | 5 | 141082015 | G | T | -0.026 | 0.004 | 7.80E-10 | 0.001 | 0.004 | 8.54E-01 |
| rs10054327 | 5 | 147871137 | G | A | 0.017 | 0.002 | 3.40E-15 | -0.002 | 0.002 | 3.26E-01 |
| rs42210 | 5 | 166408788 | G | C | -0.014 | 0.002 | 7.30E-09 | -0.002 | 0.002 | 3.85E-01 |
| rs2336895 | 5 | 166990695 | G | A | 0.012 | 0.002 | 4.60E-08 | 0.007 | 0.002 | 3.62E-04 |
| rs72828890 | 5 | 167806836 | C | T | 0.019 | 0.003 | 4.90E-09 | 0.004 | 0.003 | 1.27E-01 |
| rs255292 | 5 | 172580866 | C | A | -0.012 | 0.002 | 1.70E-08 | 0.003 | 0.002 | 1.05E-01 |
| rs72834698 | 6 | 26176517 | G | A | 0.023 | 0.003 | 2.70E-13 | 0.001 | 0.003 | 7.42E-01 |
| rs3135044 | 6 | 31592820 | C | T | -0.015 | 0.002 | 8.70E-11 | 0.002 | 0.002 | 2.98E-01 |
| rs10947452 | 6 | 33803752 | T | C | 0.014 | 0.002 | 6.40E-10 | 0.002 | 0.002 | 3.69E-01 |
| rs9471333 | 6 | 40362023 | C | T | 0.013 | 0.002 | 1.50E-09 | 0.002 | 0.002 | 2.09E-01 |
| rs4523073 | 6 | 67550288 | A | G | -0.014 | 0.002 | 5.00E-10 | -0.005 | 0.002 | 1.59E-02 |
| rs6905544 | 6 | 98411631 | A | G | -0.019 | 0.002 | 8.50E-18 | 0.008 | 0.002 | 8.95E-05 |
| rs17789218 | 6 | 100600097 | T | C | 0.019 | 0.003 | 1.40E-13 | 0.003 | 0.002 | 2.33E-01 |
| rs78394231 | 6 | 107649123 | T | C | -0.021 | 0.004 | 9.70E-09 | 0.000 | 0.003 | 9.68E-01 |
| rs2184364 | 6 | 143183596 | A | G | 0.016 | 0.003 | 3.00E-09 | 0.006 | 0.002 | 1.69E-02 |
| rs9718104 | 6 | 166170539 | T | G | -0.041 | 0.005 | 9.30E-19 | 0.013 | 0.004 | 1.68E-03 |
| rs17568389 | 7 | 8091876 | T | A | 0.015 | 0.002 | 8.60E-13 | 0.004 | 0.002 | 4.20E-02 |
| rs10234444 | 7 | 41828527 | G | A | 0.016 | 0.003 | 2.90E-08 | 0.002 | 0.003 | 5.06E-01 |
| rs6973656 | 7 | 77422583 | A | G | -0.013 | 0.002 | 1.00E-09 | -0.001 | 0.002 | 6.44E-01 |
| rs973734 | 7 | 89387578 | C | A | 0.017 | 0.003 | 1.50E-08 | 0.004 | 0.003 | 1.88E-01 |
| rs3137 | 7 | 99130875 | G | A | 0.017 | 0.003 | 1.40E-08 | 0.010 | 0.003 | 2.06E-04 |
| rs2694151 | 7 | 101902180 | C | T | 0.026 | 0.005 | 2.30E-08 | -0.004 | 0.004 | 3.01E-01 |
| rs62471080 | 7 | 109128060 | G | C | -0.013 | 0.002 | 3.70E-09 | -0.001 | 0.002 | 7.32E-01 |
| rs7788008 | 7 | 112972483 | G | A | 0.013 | 0.002 | 5.90E-09 | 0.000 | 0.002 | 9.92E-01 |
| rs10246289 | 7 | 115544763 | A | G | 0.019 | 0.003 | 1.70E-08 | 0.001 | 0.003 | 7.01E-01 |
| rs11763734 | 7 | 126400910 | A | C | -0.012 | 0.002 | 8.20E-09 | -0.005 | 0.002 | 5.86E-03 |
| rs7834121 | 8 | 10770255 | G | T | -0.014 | 0.002 | 1.10E-10 | -0.005 | 0.002 | 1.89E-02 |
| rs1421334 | 8 | 30865733 | A | C | 0.017 | 0.002 | 2.20E-15 | -0.005 | 0.002 | 9.72E-03 |
| rs6996198 | 8 | 65463442 | C | T | -0.016 | 0.003 | 3.50E-08 | 0.007 | 0.003 | 1.30E-02 |
| rs6472942 | 8 | 76827190 | T | C | -0.013 | 0.002 | 1.80E-09 | 0.000 | 0.002 | 8.98E-01 |
| rs11985394 | 8 | 92667521 | T | A | 0.012 | 0.002 | 1.30E-08 | 0.005 | 0.002 | 1.63E-02 |
| rs72671494 | 8 | 93195457 | T | C | -0.017 | 0.003 | 4.50E-08 | -0.005 | 0.003 | 1.06E-01 |
| rs12541615 | 8 | 118870151 | T | C | -0.018 | 0.003 | 3.90E-10 | -0.002 | 0.002 | 3.69E-01 |
| rs2717559 | 8 | 143882420 | A | G | 0.012 | 0.002 | 1.50E-08 | -0.001 | 0.002 | 5.86E-01 |
| rs2616830 | 9 | 1721385 | G | A | 0.016 | 0.002 | 2.90E-14 | -0.004 | 0.002 | 3.60E-02 |
| rs2291681 | 9 | 14790841 | G | T | 0.013 | 0.002 | 7.90E-09 | -0.003 | 0.002 | 1.72E-01 |
| rs34864022 | 9 | 22609110 | A | G | -0.026 | 0.004 | 1.40E-09 | 0.011 | 0.004 | 4.36E-03 |
| rs12554512 | 9 | 23352293 | T | C | 0.021 | 0.002 | 3.80E-21 | 0.000 | 0.002 | 9.84E-01 |
| rs2791574 | 9 | 82212094 | T | G | -0.013 | 0.002 | 3.00E-08 | 0.004 | 0.002 | 4.65E-02 |
| rs870151 | 9 | 92230747 | T | A | -0.016 | 0.002 | 8.80E-13 | 0.005 | 0.002 | 1.14E-02 |
| rs2787374 | 9 | 103054951 | T | C | 0.012 | 0.002 | 1.20E-08 | 0.001 | 0.002 | 6.60E-01 |
| rs10739499 | 9 | 120514261 | C | G | 0.014 | 0.002 | 1.50E-09 | 0.003 | 0.002 | 9.62E-02 |
| rs7043521 | 9 | 124602779 | A | T | 0.014 | 0.002 | 6.50E-11 | 0.001 | 0.002 | 7.61E-01 |
| rs12342024 | 9 | 126134232 | G | T | -0.021 | 0.003 | 1.70E-09 | 0.002 | 0.003 | 5.60E-01 |
| rs4382592 | 9 | 134870755 | T | G | 0.014 | 0.002 | 6.70E-09 | 0.002 | 0.002 | 3.75E-01 |
| rs2073869 | 9 | 135763816 | C | T | 0.019 | 0.003 | 1.50E-10 | 0.001 | 0.003 | 6.77E-01 |
| 9:139926402_T_C | 9 | 139926402 | T | C | -0.013 | 0.002 | 2.00E-08 | 0.006 | 0.002 | 5.00E-03 |
| rs1623025 | 10 | 11164813 | G | C | -0.012 | 0.002 | 1.40E-08 | -0.004 | 0.002 | 6.59E-02 |
| rs1243182 | 10 | 21916728 | C | T | -0.019 | 0.002 | 2.00E-15 | 0.000 | 0.002 | 9.78E-01 |
| rs2045147 | 10 | 56660437 | A | G | 0.013 | 0.002 | 5.90E-09 | 0.004 | 0.002 | 3.23E-02 |
| rs10994943 | 10 | 63591413 | T | G | 0.013 | 0.002 | 3.10E-09 | 0.001 | 0.002 | 4.50E-01 |
| rs11201422 | 10 | 86924483 | T | C | 0.013 | 0.002 | 3.40E-08 | -0.003 | 0.002 | 2.21E-01 |
| rs10786658 | 10 | 103737193 | A | T | -0.014 | 0.002 | 4.60E-11 | 0.001 | 0.002 | 6.64E-01 |
| rs7089973 | 10 | 116569565 | C | A | -0.013 | 0.002 | 2.40E-09 | 0.005 | 0.002 | 2.30E-02 |
| rs11245482 | 10 | 126733546 | T | C | -0.013 | 0.002 | 2.60E-09 | 0.002 | 0.002 | 4.27E-01 |
| rs17727474 | 10 | 127182959 | C | T | 0.018 | 0.003 | 3.10E-09 | 0.006 | 0.003 | 1.28E-02 |
| rs12289262 | 11 | 12894758 | C | T | -0.014 | 0.002 | 2.00E-08 | 0.000 | 0.002 | 8.95E-01 |
| rs801733 | 11 | 65934549 | A | C | 0.017 | 0.002 | 7.30E-14 | -0.004 | 0.002 | 3.86E-02 |
| rs116175338 | 11 | 72467875 | G | T | -0.014 | 0.003 | 1.40E-08 | 0.003 | 0.002 | 1.36E-01 |
| rs12272012 | 11 | 84861286 | G | C | -0.03 | 0.005 | 1.90E-08 | 0.000 | 0.005 | 9.22E-01 |
| rs11020045 | 11 | 92487907 | A | C | -0.013 | 0.002 | 1.60E-08 | 0.000 | 0.002 | 8.94E-01 |
| rs17207890 | 11 | 95490754 | G | A | 0.016 | 0.002 | 6.70E-12 | 0.002 | 0.002 | 2.74E-01 |
| rs10791546 | 11 | 101830126 | C | T | 0.012 | 0.002 | 4.10E-08 | -0.002 | 0.002 | 2.96E-01 |
| rs648044 | 11 | 114030799 | A | G | -0.014 | 0.002 | 1.00E-09 | 0.004 | 0.002 | 3.36E-02 |
| rs11218575 | 11 | 122154576 | C | T | 0.015 | 0.002 | 2.10E-12 | 0.000 | 0.002 | 9.54E-01 |
| rs74802478 | 11 | 131966907 | G | A | 0.017 | 0.003 | 3.50E-09 | 0.003 | 0.003 | 3.17E-01 |
| rs4937842 | 11 | 133734542 | G | C | -0.013 | 0.002 | 1.40E-08 | 0.002 | 0.002 | 3.05E-01 |
| rs10772643 | 12 | 13415288 | C | T | 0.025 | 0.003 | 1.30E-12 | 0.003 | 0.003 | 2.82E-01 |
| rs74996610 | 12 | 24075007 | C | G | -0.031 | 0.005 | 4.10E-09 | 0.003 | 0.005 | 5.04E-01 |
| rs10771746 | 12 | 30791864 | C | T | -0.014 | 0.002 | 2.50E-09 | 0.001 | 0.002 | 6.00E-01 |
| rs10876864 | 12 | 56401085 | G | A | -0.013 | 0.002 | 1.00E-09 | 0.004 | 0.002 | 3.26E-02 |
| rs8756 | 12 | 66359752 | C | A | -0.013 | 0.002 | 5.30E-10 | 0.003 | 0.002 | 9.39E-02 |
| rs11179838 | 12 | 74321123 | C | G | 0.013 | 0.002 | 2.10E-08 | 0.003 | 0.002 | 8.31E-02 |
| rs2164744 | 12 | 109872039 | T | C | -0.013 | 0.002 | 1.10E-08 | -0.003 | 0.002 | 1.26E-01 |
| rs2173650 | 12 | 117525235 | G | T | 0.018 | 0.003 | 4.60E-09 | 0.001 | 0.003 | 6.30E-01 |
| rs1790135 | 12 | 123669235 | C | T | -0.014 | 0.003 | 2.60E-08 | 0.006 | 0.002 | 5.05E-03 |
| rs9563168 | 13 | 54247827 | G | A | 0.018 | 0.003 | 4.30E-11 | -0.005 | 0.002 | 2.96E-02 |
| rs9569734 | 13 | 58319476 | A | G | 0.019 | 0.003 | 3.40E-10 | 0.003 | 0.003 | 1.97E-01 |
| rs9528023 | 13 | 60226991 | C | T | 0.013 | 0.002 | 3.80E-08 | 0.003 | 0.002 | 1.89E-01 |
| rs56858768 | 13 | 86511730 | G | A | -0.015 | 0.002 | 3.40E-10 | 0.000 | 0.002 | 8.78E-01 |
| rs553397860 | 13 | 99051118 | A | G | 0.017 | 0.003 | 8.90E-09 | 0.000 | 0.003 | 9.25E-01 |
| rs7991062 | 13 | 100713194 | C | G | -0.018 | 0.002 | 8.80E-15 | -0.008 | 0.002 | 1.45E-04 |
| rs11629120 | 14 | 23458457 | T | C | -0.013 | 0.002 | 1.10E-08 | 0.000 | 0.002 | 9.32E-01 |
| rs405797 | 14 | 26972834 | T | A | -0.015 | 0.003 | 2.80E-09 | 0.005 | 0.002 | 3.65E-02 |
| rs1022785 | 14 | 29571653 | G | A | 0.018 | 0.003 | 7.10E-09 | 0.002 | 0.003 | 3.71E-01 |
| rs10145592 | 14 | 94287215 | C | G | -0.015 | 0.002 | 1.80E-11 | 0.008 | 0.002 | 5.41E-05 |
| rs7157001 | 14 | 99749484 | A | G | -0.014 | 0.002 | 2.30E-08 | -0.003 | 0.002 | 1.16E-01 |
| rs8043253 | 15 | 41503730 | C | T | -0.012 | 0.002 | 3.40E-08 | 0.006 | 0.002 | 1.66E-03 |
| rs2460 | 15 | 53073084 | G | A | -0.015 | 0.002 | 5.00E-10 | 0.000 | 0.002 | 9.83E-01 |
| rs4775373 | 15 | 61476936 | T | C | 0.013 | 0.002 | 4.70E-09 | -0.002 | 0.002 | 3.92E-01 |
| rs61331678 | 15 | 77995949 | G | C | 0.015 | 0.002 | 6.10E-12 | 0.000 | 0.002 | 8.35E-01 |
| rs35574015 | 16 | 15146061 | T | C | -0.013 | 0.002 | 3.00E-08 | -0.001 | 0.002 | 7.98E-01 |
| rs7189927 | 16 | 28913787 | T | C | 0.015 | 0.002 | 3.40E-11 | -0.013 | 0.002 | 5.93E-11 |
| rs749671 | 16 | 31088347 | G | A | 0.016 | 0.002 | 2.70E-12 | -0.004 | 0.002 | 2.94E-02 |
| rs7184800 | 16 | 53509131 | G | A | 0.017 | 0.002 | 8.20E-13 | -0.002 | 0.002 | 2.51E-01 |
| rs2447098 | 17 | 2277720 | C | A | -0.015 | 0.002 | 6.80E-12 | 0.000 | 0.002 | 9.08E-01 |
| rs11654952 | 17 | 4813799 | T | G | -0.017 | 0.003 | 4.30E-08 | 0.003 | 0.003 | 2.49E-01 |
| rs9902312 | 17 | 65070304 | T | C | 0.015 | 0.002 | 4.50E-11 | 0.002 | 0.002 | 2.98E-01 |
| rs11657730 | 17 | 79372489 | C | T | 0.013 | 0.002 | 1.10E-08 | 0.001 | 0.002 | 4.96E-01 |
| rs303753 | 18 | 21074922 | G | A | -0.014 | 0.002 | 2.70E-10 | 0.007 | 0.002 | 3.24E-04 |
| rs11081851 | 18 | 31783262 | G | A | -0.013 | 0.002 | 1.00E-08 | 0.003 | 0.002 | 1.61E-01 |
| rs9964724 | 18 | 35159124 | C | T | 0.018 | 0.002 | 3.30E-14 | 0.002 | 0.002 | 3.50E-01 |
| rs1156541 | 18 | 39952989 | C | T | 0.014 | 0.003 | 2.80E-08 | 0.000 | 0.002 | 9.54E-01 |
| rs148544378 | 18 | 40323567 | C | T | 0.053 | 0.008 | 2.20E-12 | 0.015 | 0.007 | 4.12E-02 |
| rs17512836 | 18 | 53194961 | T | C | 0.042 | 0.007 | 7.00E-10 | -0.005 | 0.006 | 4.40E-01 |
| rs7248205 | 19 | 10770305 | C | T | 0.014 | 0.002 | 3.40E-10 | 0.002 | 0.002 | 3.57E-01 |
| rs111901094 | 19 | 19513570 | G | T | -0.017 | 0.003 | 2.00E-09 | -0.003 | 0.003 | 2.16E-01 |
| rs6131281 | 20 | 11891724 | C | T | 0.016 | 0.002 | 3.10E-13 | 0.006 | 0.002 | 1.36E-03 |
| rs6141814 | 20 | 31368960 | C | A | -0.014 | 0.002 | 1.30E-09 | -0.005 | 0.002 | 1.55E-02 |
| rs4810312 | 20 | 39968707 | C | G | -0.013 | 0.002 | 1.20E-08 | 0.000 | 0.002 | 9.04E-01 |
| rs6124476 | 20 | 41202830 | T | C | 0.012 | 0.002 | 1.80E-08 | 0.001 | 0.002 | 7.04E-01 |
| rs6031440 | 20 | 42809317 | A | T | 0.012 | 0.002 | 1.80E-08 | 0.003 | 0.002 | 1.75E-01 |
| rs55700114 | 20 | 43717080 | G | A | -0.014 | 0.002 | 2.10E-09 | -0.001 | 0.002 | 5.01E-01 |
| rs6125906 | 20 | 48729803 | C | A | -0.021 | 0.004 | 3.90E-08 | -0.005 | 0.003 | 1.26E-01 |
| rs56103247 | 20 | 62483184 | C | T | 0.03 | 0.005 | 3.80E-10 | 0.005 | 0.004 | 2.74E-01 |
| rs10427502 | 21 | 40654840 | G | A | 0.014 | 0.002 | 8.60E-10 | -0.002 | 0.002 | 2.27E-01 |
| rs2092829 | 22 | 31780514 | G | A | 0.014 | 0.002 | 1.30E-08 | 0.004 | 0.002 | 3.89E-02 |

| **Table S28. Betas of the bidirectional MR between BMI and leisure computer use** | | | | | | | | | | |
| --- | --- | --- | --- | --- | --- | --- | --- | --- | --- | --- |
| **1) MR of BMI (men and women combined) on leisure computer use** | | | | | | | | | | |
| SNP | CHR | POS | EA | OA | beta_bmi | se_bmi | pval_bmi | beta_pc | se_pc | pval_pc |
| rs79113395 | 1 | 1590521 | A | G | -0.020 | 0.002 | 2.03E-20 | 0.001 | 0.003 | 5.70E-01 |
| rs10909880 | 1 | 2727804 | T | C | -0.014 | 0.002 | 2.21E-16 | -0.006 | 0.002 | 7.30E-03 |
| rs11121210 | 1 | 8708529 | T | C | -0.011 | 0.002 | 2.37E-10 | 0.007 | 0.002 | 2.60E-03 |
| rs10779751 | 1 | 11284336 | A | G | 0.013 | 0.002 | 2.66E-13 | 0.003 | 0.002 | 2.00E-01 |
| rs61740466 | 1 | 19934900 | A | G | -0.014 | 0.002 | 2.24E-10 | 0.001 | 0.003 | 6.80E-01 |
| rs4655141 | 1 | 23312025 | T | C | -0.017 | 0.002 | 6.20E-15 | 0.010 | 0.003 | 1.00E-03 |
| rs2228552 | 1 | 32165495 | T | G | 0.012 | 0.002 | 1.80E-11 | -0.001 | 0.002 | 7.50E-01 |
| rs4653017 | 1 | 33776728 | T | C | 0.012 | 0.002 | 1.11E-10 | 0.000 | 0.002 | 9.00E-01 |
| rs7512146 | 1 | 34283008 | T | G | -0.010 | 0.002 | 1.29E-08 | 0.000 | 0.002 | 8.80E-01 |
| rs11577094 | 1 | 38026600 | T | C | 0.019 | 0.003 | 3.28E-10 | -0.009 | 0.004 | 2.10E-02 |
| rs112646560 | 1 | 39560250 | T | C | 0.018 | 0.002 | 1.29E-14 | 0.003 | 0.003 | 3.40E-01 |
| rs2984618 | 1 | 47690438 | T | G | 0.017 | 0.002 | 3.80E-24 | 0.004 | 0.002 | 9.30E-02 |
| rs657452 | 1 | 49589847 | A | G | 0.019 | 0.002 | 3.17E-30 | -0.005 | 0.002 | 4.00E-02 |
| rs587271 | 1 | 54743111 | T | C | 0.012 | 0.002 | 6.21E-11 | 0.010 | 0.002 | 7.00E-05 |
| rs79906980 | 1 | 57887985 | T | C | 0.016 | 0.003 | 1.69E-09 | 0.001 | 0.003 | 7.30E-01 |
| rs12140153 | 1 | 62579891 | T | G | -0.035 | 0.003 | 1.44E-25 | 0.004 | 0.004 | 3.20E-01 |
| rs2503185 | 1 | 66461401 | A | G | 0.013 | 0.002 | 1.33E-14 | 0.000 | 0.002 | 8.70E-01 |
| rs3101336 | 1 | 72751185 | T | C | -0.025 | 0.002 | 4.80E-54 | -0.007 | 0.002 | 1.20E-03 |
| rs6656785 | 1 | 75005776 | A | G | -0.018 | 0.002 | 3.06E-27 | 0.004 | 0.002 | 1.10E-01 |
| rs34517439 | 1 | 78450517 | A | C | 0.039 | 0.003 | 3.40E-39 | 0.003 | 0.003 | 3.90E-01 |
| rs6696828 | 1 | 80812020 | C | G | 0.012 | 0.002 | 6.42E-11 | 0.000 | 0.002 | 1.00E+00 |
| rs321237 | 1 | 96478125 | A | G | 0.013 | 0.002 | 1.49E-10 | 0.010 | 0.003 | 1.10E-04 |
| rs11165643 | 1 | 96924097 | T | C | 0.019 | 0.002 | 4.49E-30 | 0.003 | 0.002 | 1.30E-01 |
| rs12072739 | 1 | 98315893 | A | G | -0.017 | 0.002 | 1.50E-13 | 0.002 | 0.003 | 4.50E-01 |
| rs17024393 | 1 | 110154688 | T | C | -0.064 | 0.005 | 7.11E-39 | -0.024 | 0.007 | 4.80E-04 |
| rs197374 | 1 | 112289983 | T | C | 0.014 | 0.002 | 3.16E-16 | 0.004 | 0.002 | 8.80E-02 |
| rs17544384 | 1 | 115295160 | T | C | -0.013 | 0.002 | 2.28E-08 | -0.001 | 0.003 | 6.90E-01 |
| rs7534091 | 1 | 118864616 | A | G | -0.012 | 0.002 | 6.65E-11 | 0.003 | 0.003 | 2.00E-01 |
| rs74887628 | 1 | 147032779 | A | G | 0.031 | 0.005 | 1.76E-08 | -0.008 | 0.006 | 2.00E-01 |
| rs4970991 | 1 | 151004003 | T | C | 0.012 | 0.002 | 1.81E-08 | 0.007 | 0.003 | 1.20E-02 |
| rs61813324 | 1 | 156049877 | T | C | 0.029 | 0.003 | 3.20E-24 | 0.000 | 0.003 | 9.50E-01 |
| rs1750307 | 1 | 156488420 | A | T | 0.013 | 0.002 | 2.78E-13 | -0.002 | 0.002 | 3.00E-01 |
| rs10733051 | 1 | 167280354 | A | G | 0.009 | 0.002 | 6.96E-09 | -0.003 | 0.002 | 1.60E-01 |
| rs61828641 | 1 | 174321997 | A | G | 0.022 | 0.003 | 2.49E-13 | 0.014 | 0.004 | 1.10E-04 |
| rs543874 | 1 | 177889480 | A | G | -0.048 | 0.002 | 3.06E-125 | 0.002 | 0.003 | 4.50E-01 |
| rs10920678 | 1 | 190239907 | A | G | 0.015 | 0.002 | 7.15E-20 | 0.001 | 0.002 | 7.70E-01 |
| rs2400414 | 1 | 194965200 | T | C | -0.013 | 0.002 | 5.52E-13 | -0.002 | 0.002 | 3.60E-01 |
| rs2820295 | 1 | 201800868 | A | G | 0.024 | 0.002 | 5.56E-39 | -0.006 | 0.002 | 1.00E-02 |
| rs10920336 | 1 | 202115945 | A | G | -0.010 | 0.002 | 4.20E-09 | -0.001 | 0.002 | 7.80E-01 |
| rs1006317 | 1 | 209552636 | T | G | 0.016 | 0.003 | 3.75E-10 | -0.009 | 0.003 | 6.50E-03 |
| rs6661316 | 1 | 210095527 | T | C | 0.012 | 0.002 | 1.72E-13 | 0.000 | 0.002 | 9.60E-01 |
| rs10864728 | 1 | 230304914 | A | G | 0.011 | 0.002 | 1.20E-08 | 0.000 | 0.002 | 8.40E-01 |
| rs946824 | 1 | 243684019 | T | C | 0.020 | 0.003 | 4.82E-15 | -0.007 | 0.003 | 3.10E-02 |
| rs6710091 | 2 | 239597 | C | G | 0.010 | 0.002 | 5.78E-09 | 0.000 | 0.002 | 1.00E+00 |
| rs77165542 | 2 | 430975 | T | C | -0.094 | 0.005 | 1.51E-70 | -0.013 | 0.006 | 3.60E-02 |
| rs13021737 | 2 | 632348 | A | G | -0.058 | 0.002 | 2.89E-161 | -0.009 | 0.003 | 2.80E-03 |
| rs10929925 | 2 | 6155557 | A | C | -0.014 | 0.002 | 3.05E-18 | 0.002 | 0.002 | 4.80E-01 |
| rs11902450 | 2 | 12845368 | T | C | 0.017 | 0.003 | 1.06E-09 | 0.002 | 0.004 | 6.60E-01 |
| rs10182181 | 2 | 25150296 | A | G | -0.033 | 0.002 | 2.45E-91 | -0.004 | 0.002 | 1.10E-01 |
| rs935166 | 2 | 26949366 | A | G | -0.015 | 0.002 | 8.69E-16 | -0.004 | 0.002 | 5.50E-02 |
| rs10168563 | 2 | 35552173 | A | G | 0.013 | 0.002 | 9.33E-12 | -0.005 | 0.002 | 3.30E-02 |
| rs3770890 | 2 | 36657992 | T | G | -0.030 | 0.005 | 2.10E-08 | -0.010 | 0.007 | 1.40E-01 |
| rs10185199 | 2 | 40282202 | A | G | -0.014 | 0.002 | 3.18E-13 | -0.002 | 0.003 | 4.50E-01 |
| rs10169594 | 2 | 41637688 | T | C | -0.012 | 0.002 | 1.34E-11 | 0.001 | 0.002 | 6.90E-01 |
| rs7561278 | 2 | 48954905 | T | C | 0.017 | 0.002 | 4.89E-16 | -0.001 | 0.003 | 7.30E-01 |
| rs930295 | 2 | 50233352 | A | C | 0.021 | 0.002 | 2.03E-19 | -0.001 | 0.003 | 7.10E-01 |
| rs805412 | 2 | 54120820 | A | G | -0.010 | 0.002 | 1.14E-08 | 0.000 | 0.002 | 9.90E-01 |
| rs4671328 | 2 | 58935282 | T | G | 0.021 | 0.002 | 3.42E-36 | 0.002 | 0.002 | 4.70E-01 |
| rs6545714 | 2 | 59307725 | A | G | -0.019 | 0.002 | 4.01E-32 | 0.001 | 0.002 | 7.30E-01 |
| rs2861685 | 2 | 67837553 | T | C | 0.017 | 0.002 | 7.82E-18 | 0.003 | 0.002 | 1.40E-01 |
| rs17020497 | 2 | 81826131 | A | G | 0.014 | 0.003 | 2.04E-08 | -0.001 | 0.003 | 6.90E-01 |
| rs12714199 | 2 | 86812549 | T | C | -0.014 | 0.002 | 3.22E-16 | -0.001 | 0.002 | 7.90E-01 |
| rs4303732 | 2 | 100830040 | T | C | 0.017 | 0.002 | 1.26E-22 | -0.006 | 0.002 | 4.20E-03 |
| rs264941 | 2 | 104297420 | A | C | -0.012 | 0.002 | 1.84E-13 | 0.004 | 0.002 | 8.90E-02 |
| rs10197031 | 2 | 105454590 | T | C | -0.016 | 0.002 | 5.05E-18 | 0.007 | 0.002 | 3.60E-03 |
| rs13033310 | 2 | 133523605 | A | G | 0.015 | 0.002 | 3.40E-11 | 0.000 | 0.003 | 9.60E-01 |
| rs4988235 | 2 | 136608646 | A | G | 0.012 | 0.002 | 7.09E-13 | 0.002 | 0.003 | 5.30E-01 |
| rs17814208 | 2 | 144037998 | A | G | -0.013 | 0.002 | 7.24E-11 | -0.003 | 0.003 | 2.20E-01 |
| rs1451077 | 2 | 147901207 | A | G | -0.017 | 0.002 | 1.43E-18 | 0.000 | 0.002 | 9.60E-01 |
| rs2119753 | 2 | 151224579 | A | G | 0.010 | 0.002 | 1.00E-08 | -0.001 | 0.002 | 8.00E-01 |
| rs11695013 | 2 | 157057487 | T | C | -0.010 | 0.002 | 2.43E-09 | -0.005 | 0.002 | 1.70E-02 |
| rs3764835 | 2 | 159519368 | A | G | -0.013 | 0.002 | 3.84E-08 | -0.008 | 0.003 | 5.70E-03 |
| rs12692596 | 2 | 161265910 | T | C | 0.012 | 0.002 | 1.03E-12 | 0.001 | 0.002 | 5.50E-01 |
| rs62176243 | 2 | 166190881 | A | T | 0.015 | 0.002 | 2.41E-11 | 0.001 | 0.003 | 7.40E-01 |
| rs56133507 | 2 | 172818467 | T | G | -0.013 | 0.002 | 4.31E-08 | 0.004 | 0.003 | 1.10E-01 |
| rs34234296 | 2 | 175166636 | A | G | -0.015 | 0.002 | 2.01E-13 | -0.005 | 0.002 | 1.90E-02 |
| rs7588437 | 2 | 181575281 | A | G | -0.017 | 0.002 | 2.32E-22 | 0.002 | 0.002 | 3.50E-01 |
| rs6716898 | 2 | 198944271 | A | G | 0.013 | 0.002 | 2.33E-11 | -0.008 | 0.002 | 2.70E-04 |
| rs7593917 | 2 | 203931012 | A | G | -0.012 | 0.002 | 9.67E-13 | -0.003 | 0.002 | 1.50E-01 |
| rs4482463 | 2 | 205375909 | A | C | -0.031 | 0.003 | 4.85E-23 | -0.005 | 0.004 | 2.60E-01 |
| rs11692326 | 2 | 208263279 | T | C | 0.015 | 0.002 | 1.69E-14 | -0.013 | 0.003 | 4.70E-07 |
| rs73985439 | 2 | 212299249 | A | C | -0.013 | 0.002 | 1.84E-10 | 0.001 | 0.002 | 6.40E-01 |
| rs7599312 | 2 | 213413231 | A | G | -0.018 | 0.002 | 1.52E-23 | 0.009 | 0.003 | 5.70E-04 |
| rs12987009 | 2 | 219658170 | A | T | -0.011 | 0.002 | 1.83E-10 | 0.001 | 0.002 | 5.60E-01 |
| rs6725931 | 2 | 220205146 | T | C | 0.019 | 0.002 | 2.30E-15 | 0.001 | 0.003 | 7.30E-01 |
| rs4973618 | 2 | 229002620 | A | G | -0.015 | 0.002 | 1.25E-16 | 0.002 | 0.002 | 2.90E-01 |
| rs6720868 | 2 | 230663576 | T | C | 0.015 | 0.002 | 1.82E-17 | 0.000 | 0.002 | 9.40E-01 |
| rs7568228 | 2 | 236848488 | C | G | -0.010 | 0.002 | 1.00E-09 | 0.001 | 0.002 | 7.30E-01 |
| rs59302296 | 3 | 9507314 | A | T | 0.022 | 0.003 | 9.12E-12 | 0.000 | 0.004 | 1.00E+00 |
| rs6783054 | 3 | 11672805 | A | C | 0.010 | 0.002 | 6.22E-09 | 0.001 | 0.002 | 5.00E-01 |
| rs10510419 | 3 | 12426936 | T | G | -0.017 | 0.002 | 2.23E-13 | 0.000 | 0.003 | 8.90E-01 |
| rs2600226 | 3 | 12928762 | T | C | -0.012 | 0.002 | 1.42E-10 | 0.001 | 0.002 | 6.80E-01 |
| rs1048637 | 3 | 13358171 | T | G | -0.009 | 0.002 | 2.94E-08 | -0.004 | 0.002 | 4.50E-02 |
| rs4858193 | 3 | 20441050 | T | C | 0.013 | 0.002 | 2.44E-12 | -0.001 | 0.002 | 6.80E-01 |
| rs6804842 | 3 | 25106437 | A | G | -0.014 | 0.002 | 7.57E-18 | 0.001 | 0.002 | 5.80E-01 |
| rs11921432 | 3 | 35117776 | T | C | -0.019 | 0.003 | 5.38E-12 | 0.003 | 0.003 | 3.20E-01 |
| rs1799923 | 3 | 42306294 | A | G | -0.022 | 0.003 | 1.12E-17 | -0.006 | 0.003 | 6.20E-02 |
| rs28350 | 3 | 42418446 | A | G | 0.017 | 0.002 | 1.07E-14 | -0.003 | 0.003 | 3.10E-01 |
| rs4017425 | 3 | 44028764 | T | C | -0.012 | 0.002 | 2.91E-12 | -0.001 | 0.002 | 6.20E-01 |
| rs11919665 | 3 | 48085349 | A | T | 0.012 | 0.002 | 7.30E-09 | -0.009 | 0.002 | 3.10E-04 |
| rs11713193 | 3 | 49924424 | A | G | 0.025 | 0.002 | 3.02E-48 | -0.009 | 0.002 | 3.00E-05 |
| rs2365389 | 3 | 61236462 | T | C | -0.017 | 0.002 | 6.49E-25 | 0.002 | 0.002 | 3.70E-01 |
| rs6445258 | 3 | 62112198 | T | C | 0.013 | 0.002 | 2.48E-08 | 0.004 | 0.003 | 1.60E-01 |
| rs925018 | 3 | 62713143 | C | G | -0.013 | 0.002 | 4.33E-14 | 0.002 | 0.002 | 5.10E-01 |
| rs11915371 | 3 | 70539559 | A | C | -0.015 | 0.002 | 2.29E-13 | -0.006 | 0.003 | 1.90E-02 |
| rs12636480 | 3 | 82719412 | T | G | 0.013 | 0.002 | 6.96E-13 | -0.003 | 0.002 | 1.80E-01 |
| rs9827823 | 3 | 84221774 | T | C | 0.018 | 0.002 | 3.39E-15 | -0.005 | 0.003 | 8.60E-02 |
| rs9818122 | 3 | 85861064 | T | C | -0.023 | 0.002 | 3.97E-30 | -0.006 | 0.003 | 2.70E-02 |
| rs34184235 | 3 | 86192846 | T | C | -0.012 | 0.002 | 2.14E-09 | -0.001 | 0.002 | 6.10E-01 |
| rs11128021 | 3 | 88139016 | A | G | -0.018 | 0.002 | 1.63E-14 | -0.004 | 0.003 | 1.40E-01 |
| rs1492014 | 3 | 94071481 | T | C | -0.017 | 0.002 | 1.44E-23 | 0.002 | 0.002 | 2.80E-01 |
| rs1436344 | 3 | 104606144 | C | G | 0.015 | 0.002 | 1.07E-17 | -0.004 | 0.002 | 7.60E-02 |
| rs7640424 | 3 | 107820063 | T | C | -0.014 | 0.002 | 1.23E-14 | 0.005 | 0.002 | 2.70E-02 |
| rs17681451 | 3 | 114399296 | A | G | -0.023 | 0.003 | 7.37E-13 | -0.001 | 0.004 | 7.90E-01 |
| rs6808814 | 3 | 116852469 | T | C | 0.012 | 0.002 | 1.31E-09 | -0.003 | 0.003 | 2.30E-01 |
| rs7631156 | 3 | 131751628 | A | G | 0.022 | 0.002 | 3.33E-32 | -0.002 | 0.002 | 4.80E-01 |
| rs687339 | 3 | 135932359 | T | C | 0.019 | 0.002 | 4.32E-22 | 0.006 | 0.003 | 1.60E-02 |
| rs16851483 | 3 | 141275436 | T | G | 0.035 | 0.003 | 4.87E-25 | 0.006 | 0.004 | 1.80E-01 |
| rs355777 | 3 | 154034950 | C | G | 0.015 | 0.002 | 2.13E-18 | -0.004 | 0.002 | 1.20E-01 |
| rs9826775 | 3 | 156295341 | A | G | 0.016 | 0.002 | 6.61E-11 | -0.001 | 0.003 | 8.70E-01 |
| rs2047648 | 3 | 157033438 | A | T | -0.013 | 0.002 | 7.95E-12 | -0.004 | 0.003 | 1.00E-01 |
| rs3732927 | 3 | 170586057 | T | C | 0.010 | 0.002 | 1.13E-08 | -0.002 | 0.002 | 3.60E-01 |
| rs12635553 | 3 | 171113714 | A | T | 0.010 | 0.002 | 4.10E-09 | 0.003 | 0.002 | 2.20E-01 |
| rs39654 | 3 | 173095123 | A | G | -0.016 | 0.002 | 1.75E-21 | 0.006 | 0.002 | 1.20E-02 |
| rs6443750 | 3 | 181329682 | T | C | -0.015 | 0.002 | 7.25E-13 | -0.001 | 0.003 | 6.50E-01 |
| rs865809 | 3 | 183997735 | A | G | 0.012 | 0.002 | 6.50E-10 | 0.006 | 0.003 | 1.50E-02 |
| rs9816226 | 3 | 185834499 | A | T | -0.032 | 0.002 | 1.45E-50 | 0.002 | 0.003 | 4.90E-01 |
| rs7616009 | 3 | 194881756 | A | G | -0.016 | 0.002 | 4.33E-11 | 0.000 | 0.003 | 9.80E-01 |
| rs6803161 | 3 | 196205694 | T | C | 0.011 | 0.002 | 2.71E-08 | 0.001 | 0.002 | 8.00E-01 |
| rs2051559 | 4 | 3298800 | T | C | -0.017 | 0.003 | 3.78E-11 | 0.009 | 0.003 | 8.30E-03 |
| rs12642970 | 4 | 16601492 | C | G | -0.011 | 0.002 | 1.71E-08 | -0.005 | 0.002 | 1.60E-02 |
| rs994596 | 4 | 18459828 | T | C | 0.013 | 0.002 | 1.06E-12 | 0.002 | 0.002 | 4.40E-01 |
| rs34811474 | 4 | 25408838 | A | G | -0.029 | 0.002 | 8.50E-38 | 0.003 | 0.003 | 2.80E-01 |
| rs73213484 | 4 | 28489339 | A | T | 0.021 | 0.003 | 2.04E-14 | 0.003 | 0.003 | 3.20E-01 |
| rs337637 | 4 | 38604470 | A | G | -0.014 | 0.002 | 6.24E-16 | 0.005 | 0.002 | 1.90E-02 |
| rs10938397 | 4 | 45182527 | A | G | -0.032 | 0.002 | 2.42E-86 | 0.006 | 0.002 | 8.30E-03 |
| rs2271046 | 4 | 52752812 | A | T | -0.012 | 0.002 | 3.03E-10 | 0.004 | 0.002 | 6.50E-02 |
| rs1492767 | 4 | 55221467 | T | C | 0.010 | 0.002 | 3.55E-09 | 0.002 | 0.002 | 4.00E-01 |
| rs2192158 | 4 | 55505360 | A | G | 0.014 | 0.002 | 7.42E-16 | 0.003 | 0.002 | 1.70E-01 |
| rs1346841 | 4 | 65651730 | A | G | -0.013 | 0.002 | 3.18E-13 | -0.001 | 0.002 | 5.50E-01 |
| rs10002111 | 4 | 67815504 | A | G | 0.013 | 0.002 | 1.26E-09 | -0.001 | 0.003 | 7.50E-01 |
| rs10033843 | 4 | 77028783 | A | G | 0.014 | 0.002 | 1.07E-11 | 0.001 | 0.003 | 7.30E-01 |
| rs72649373 | 4 | 80609966 | T | C | -0.017 | 0.003 | 3.20E-09 | 0.005 | 0.003 | 1.10E-01 |
| rs4148155 | 4 | 89054667 | A | G | 0.019 | 0.003 | 1.34E-13 | -0.009 | 0.003 | 6.90E-03 |
| rs4286488 | 4 | 94440026 | A | G | 0.012 | 0.002 | 1.70E-09 | -0.007 | 0.003 | 1.00E-02 |
| rs7678054 | 4 | 95093855 | A | G | -0.010 | 0.002 | 4.57E-09 | 0.004 | 0.002 | 7.90E-02 |
| rs3796432 | 4 | 96030402 | T | G | -0.011 | 0.002 | 2.21E-10 | 0.002 | 0.002 | 4.30E-01 |
| rs13107325 | 4 | 103188709 | T | C | 0.047 | 0.003 | 3.81E-47 | 0.001 | 0.004 | 7.60E-01 |
| rs326893 | 4 | 112691776 | T | C | 0.012 | 0.002 | 1.89E-12 | -0.001 | 0.002 | 6.40E-01 |
| rs7696649 | 4 | 120322177 | A | G | 0.012 | 0.002 | 4.73E-10 | 0.000 | 0.002 | 8.50E-01 |
| rs4864201 | 4 | 130731284 | T | C | 0.014 | 0.002 | 4.30E-16 | 0.000 | 0.002 | 9.30E-01 |
| rs1296328 | 4 | 137083193 | A | C | 0.017 | 0.002 | 3.49E-22 | -0.002 | 0.002 | 2.90E-01 |
| rs17367750 | 4 | 140782542 | T | C | -0.012 | 0.002 | 1.97E-11 | -0.006 | 0.002 | 1.50E-02 |
| rs9992189 | 4 | 144060728 | C | G | -0.010 | 0.002 | 2.04E-08 | 0.000 | 0.002 | 9.50E-01 |
| rs3914628 | 4 | 147438019 | T | C | 0.017 | 0.002 | 6.91E-13 | 0.005 | 0.003 | 9.10E-02 |
| rs750090 | 4 | 152931436 | T | C | 0.011 | 0.002 | 2.50E-10 | 0.004 | 0.002 | 7.00E-02 |
| rs13110266 | 4 | 162129844 | A | G | -0.012 | 0.002 | 3.96E-14 | -0.001 | 0.002 | 7.30E-01 |
| rs7685628 | 4 | 165310133 | A | T | 0.010 | 0.002 | 6.15E-09 | 0.004 | 0.002 | 9.30E-02 |
| rs1522569 | 4 | 171632637 | T | G | 0.014 | 0.002 | 1.60E-10 | 0.002 | 0.003 | 5.80E-01 |
| rs1437842 | 4 | 173597016 | A | G | -0.011 | 0.002 | 8.49E-10 | 0.002 | 0.002 | 4.10E-01 |
| rs6850421 | 4 | 180187034 | A | G | 0.011 | 0.002 | 3.66E-09 | 0.007 | 0.002 | 2.60E-03 |
| rs35408866 | 4 | 187743245 | A | G | 0.016 | 0.003 | 1.50E-08 | 0.000 | 0.003 | 9.10E-01 |
| rs698147 | 5 | 3513485 | A | G | 0.012 | 0.002 | 9.67E-12 | 0.005 | 0.002 | 2.60E-02 |
| rs6890310 | 5 | 27193573 | A | G | -0.012 | 0.002 | 3.29E-10 | 0.001 | 0.002 | 7.10E-01 |
| rs7730004 | 5 | 43191033 | T | C | 0.014 | 0.002 | 1.46E-14 | -0.002 | 0.002 | 5.10E-01 |
| rs116374395 | 5 | 50723410 | A | G | 0.032 | 0.005 | 7.05E-10 | 0.002 | 0.006 | 7.50E-01 |
| rs150215901 | 5 | 50935903 | A | T | -0.028 | 0.005 | 1.54E-08 | 0.002 | 0.005 | 6.60E-01 |
| rs4865796 | 5 | 53272664 | A | G | -0.010 | 0.002 | 4.21E-08 | -0.001 | 0.002 | 8.10E-01 |
| rs13186194 | 5 | 60795485 | T | C | 0.010 | 0.002 | 2.20E-09 | 0.003 | 0.002 | 2.00E-01 |
| rs4700608 | 5 | 63026280 | T | C | -0.016 | 0.002 | 4.32E-20 | -0.002 | 0.002 | 3.90E-01 |
| rs10050620 | 5 | 63927239 | T | C | -0.013 | 0.002 | 1.20E-10 | 0.003 | 0.002 | 2.80E-01 |
| rs1159692 | 5 | 63977815 | A | C | 0.014 | 0.002 | 5.54E-15 | 0.003 | 0.002 | 1.20E-01 |
| rs2112347 | 5 | 75015242 | T | G | 0.028 | 0.002 | 1.17E-61 | -0.005 | 0.002 | 4.70E-02 |
| rs10942267 | 5 | 80841914 | A | G | 0.015 | 0.002 | 6.53E-16 | 0.007 | 0.002 | 3.60E-03 |
| rs2962334 | 5 | 86879056 | T | G | 0.040 | 0.006 | 1.78E-11 | 0.011 | 0.008 | 1.80E-01 |
| rs1501673 | 5 | 87963600 | A | G | 0.029 | 0.003 | 2.73E-31 | 0.012 | 0.003 | 3.70E-04 |
| rs12652212 | 5 | 88808594 | A | G | -0.013 | 0.002 | 1.63E-15 | 0.003 | 0.002 | 1.40E-01 |
| rs7713317 | 5 | 95716722 | A | G | -0.017 | 0.002 | 1.96E-20 | 0.002 | 0.002 | 4.50E-01 |
| rs6882366 | 5 | 95864693 | T | C | -0.013 | 0.002 | 4.39E-14 | -0.005 | 0.002 | 4.40E-02 |
| rs11739877 | 5 | 105876806 | T | C | 0.012 | 0.002 | 4.01E-11 | 0.001 | 0.002 | 6.70E-01 |
| rs6888194 | 5 | 106910657 | T | C | -0.013 | 0.002 | 3.58E-08 | -0.003 | 0.003 | 3.80E-01 |
| rs40067 | 5 | 107439012 | A | G | -0.025 | 0.002 | 9.73E-29 | -0.002 | 0.003 | 4.00E-01 |
| rs459552 | 5 | 112176756 | A | T | -0.013 | 0.002 | 8.36E-12 | 0.003 | 0.003 | 2.40E-01 |
| rs6893539 | 5 | 122705737 | A | C | -0.012 | 0.002 | 6.17E-11 | -0.001 | 0.002 | 5.70E-01 |
| rs6864049 | 5 | 124330522 | A | G | -0.012 | 0.002 | 1.48E-13 | 0.003 | 0.002 | 2.50E-01 |
| rs6886072 | 5 | 136598460 | T | C | -0.010 | 0.002 | 4.13E-09 | 0.002 | 0.002 | 4.10E-01 |
| rs13174863 | 5 | 139080745 | A | G | -0.020 | 0.002 | 1.94E-17 | -0.003 | 0.003 | 3.50E-01 |
| rs17405603 | 5 | 144608340 | A | T | -0.013 | 0.002 | 4.43E-11 | -0.004 | 0.002 | 1.10E-01 |
| rs2910026 | 5 | 152529936 | T | C | -0.013 | 0.002 | 5.69E-10 | 0.004 | 0.002 | 1.20E-01 |
| rs7715256 | 5 | 153537893 | T | G | -0.016 | 0.002 | 3.98E-22 | 0.004 | 0.002 | 1.10E-01 |
| rs7734385 | 5 | 158460212 | A | G | -0.010 | 0.002 | 6.08E-10 | 0.002 | 0.002 | 4.90E-01 |
| rs4921301 | 5 | 159984492 | T | C | -0.013 | 0.002 | 4.03E-08 | 0.001 | 0.003 | 7.50E-01 |
| rs2861089 | 5 | 164557954 | A | T | 0.011 | 0.002 | 1.55E-09 | 0.004 | 0.002 | 1.00E-01 |
| rs7727781 | 5 | 165185571 | T | C | 0.009 | 0.002 | 4.27E-08 | 0.004 | 0.002 | 6.70E-02 |
| rs2053682 | 5 | 170599327 | A | C | 0.017 | 0.002 | 2.59E-20 | 0.004 | 0.002 | 6.30E-02 |
| rs6556301 | 5 | 176527577 | T | G | -0.011 | 0.002 | 8.14E-11 | -0.001 | 0.002 | 7.70E-01 |
| rs2228213 | 6 | 12124855 | A | G | -0.014 | 0.002 | 5.50E-17 | -0.003 | 0.002 | 2.70E-01 |
| rs11757278 | 6 | 13180454 | T | C | 0.013 | 0.002 | 6.92E-13 | 0.003 | 0.002 | 1.80E-01 |
| rs3806114 | 6 | 20482335 | A | G | -0.012 | 0.002 | 1.46E-11 | 0.003 | 0.002 | 2.60E-01 |
| rs2066295 | 6 | 26168903 | A | G | 0.014 | 0.002 | 2.29E-12 | -0.009 | 0.003 | 9.80E-04 |
| rs3115667 | 6 | 31643399 | T | C | -0.018 | 0.002 | 5.92E-21 | 0.004 | 0.002 | 6.60E-02 |
| rs2281819 | 6 | 33771673 | A | T | -0.015 | 0.002 | 2.09E-14 | -0.003 | 0.003 | 2.80E-01 |
| rs2744974 | 6 | 34579431 | T | C | 0.026 | 0.002 | 1.28E-51 | 0.005 | 0.002 | 3.00E-02 |
| rs2436728 | 6 | 40365601 | A | G | 0.019 | 0.002 | 1.97E-29 | -0.004 | 0.002 | 9.30E-02 |
| rs1358980 | 6 | 43764551 | T | C | -0.013 | 0.002 | 5.15E-15 | -0.004 | 0.002 | 1.10E-01 |
| rs2206277 | 6 | 50798526 | T | C | 0.041 | 0.002 | 1.82E-83 | 0.002 | 0.003 | 4.90E-01 |
| rs1327259 | 6 | 51177811 | A | G | 0.016 | 0.002 | 1.47E-19 | -0.001 | 0.002 | 7.40E-01 |
| rs6915002 | 6 | 54028069 | T | C | 0.010 | 0.002 | 8.71E-09 | 0.009 | 0.002 | 6.00E-05 |
| rs9370410 | 6 | 55171842 | A | G | 0.011 | 0.002 | 2.58E-08 | -0.002 | 0.002 | 3.50E-01 |
| rs2622274 | 6 | 64240516 | T | G | -0.011 | 0.002 | 3.23E-10 | -0.003 | 0.002 | 1.80E-01 |
| rs1293037 | 6 | 70248345 | T | C | 0.013 | 0.002 | 5.73E-09 | -0.004 | 0.003 | 9.70E-02 |
| rs6921533 | 6 | 73742334 | T | C | 0.010 | 0.002 | 1.37E-08 | 0.001 | 0.002 | 7.00E-01 |
| rs9294260 | 6 | 83433228 | A | G | 0.014 | 0.002 | 8.16E-18 | 0.000 | 0.002 | 9.40E-01 |
| rs6909685 | 6 | 97753952 | T | C | -0.015 | 0.002 | 2.78E-16 | 0.001 | 0.002 | 6.30E-01 |
| rs9320823 | 6 | 98429337 | T | C | -0.017 | 0.002 | 2.07E-21 | 0.017 | 0.002 | 2.30E-14 |
| rs57989773 | 6 | 100629078 | T | C | -0.014 | 0.002 | 5.63E-10 | -0.014 | 0.003 | 5.20E-08 |
| rs156201 | 6 | 104847441 | C | G | 0.012 | 0.002 | 1.65E-10 | 0.006 | 0.003 | 2.40E-02 |
| rs768023 | 6 | 108876002 | A | G | 0.016 | 0.002 | 1.13E-22 | 0.001 | 0.002 | 6.60E-01 |
| rs2357760 | 6 | 120213880 | A | G | 0.014 | 0.002 | 2.11E-16 | 0.004 | 0.002 | 9.60E-02 |
| rs2875762 | 6 | 124925032 | C | G | 0.013 | 0.002 | 1.07E-10 | 0.001 | 0.003 | 7.40E-01 |
| rs1268065 | 6 | 126042783 | A | G | -0.010 | 0.002 | 7.12E-10 | -0.003 | 0.002 | 1.70E-01 |
| rs9375702 | 6 | 130384187 | T | C | -0.011 | 0.002 | 5.52E-09 | -0.005 | 0.002 | 5.10E-02 |
| rs2246012 | 6 | 131898208 | T | C | -0.016 | 0.002 | 1.15E-13 | -0.009 | 0.003 | 2.90E-03 |
| rs6922607 | 6 | 142703483 | A | G | -0.013 | 0.002 | 1.85E-09 | 0.004 | 0.003 | 1.10E-01 |
| rs765875 | 6 | 143185683 | T | C | -0.013 | 0.002 | 1.08E-14 | 0.004 | 0.002 | 1.10E-01 |
| rs7760482 | 6 | 147354276 | A | G | -0.010 | 0.002 | 6.25E-09 | 0.003 | 0.002 | 1.60E-01 |
| rs12527426 | 6 | 153392002 | A | G | 0.015 | 0.002 | 5.54E-16 | 0.002 | 0.002 | 5.20E-01 |
| rs9478496 | 6 | 154333183 | T | C | -0.016 | 0.002 | 8.17E-12 | -0.003 | 0.003 | 3.80E-01 |
| rs13191362 | 6 | 163033350 | A | G | 0.024 | 0.003 | 4.08E-21 | -0.005 | 0.003 | 1.10E-01 |
| rs9458814 | 6 | 163771305 | T | C | -0.011 | 0.002 | 2.33E-08 | -0.005 | 0.003 | 3.90E-02 |
| rs6950388 | 7 | 1270699 | A | G | 0.014 | 0.002 | 1.59E-09 | -0.001 | 0.003 | 7.50E-01 |
| rs4721089 | 7 | 1872921 | T | C | 0.017 | 0.002 | 5.60E-13 | 0.003 | 0.003 | 2.10E-01 |
| rs6463489 | 7 | 5542513 | T | C | 0.017 | 0.003 | 2.50E-10 | 0.003 | 0.003 | 3.20E-01 |
| rs4307239 | 7 | 24354300 | A | G | -0.012 | 0.002 | 1.47E-11 | 0.003 | 0.002 | 1.40E-01 |
| rs213518 | 7 | 26941065 | T | C | -0.015 | 0.002 | 2.49E-10 | -0.008 | 0.003 | 1.40E-02 |
| rs215669 | 7 | 32378979 | A | G | -0.015 | 0.002 | 8.94E-18 | 0.004 | 0.002 | 8.20E-02 |
| rs2108719 | 7 | 39481056 | A | G | 0.011 | 0.002 | 2.28E-08 | -0.003 | 0.003 | 2.90E-01 |
| rs217433 | 7 | 44553496 | T | C | -0.012 | 0.002 | 3.00E-08 | -0.002 | 0.003 | 3.60E-01 |
| rs2289379 | 7 | 44804225 | T | C | -0.014 | 0.002 | 7.02E-15 | 0.007 | 0.002 | 2.50E-03 |
| rs10499694 | 7 | 50614173 | A | G | 0.013 | 0.002 | 1.29E-15 | 0.001 | 0.002 | 5.50E-01 |
| rs11772246 | 7 | 71603692 | T | C | 0.015 | 0.002 | 3.69E-11 | 0.003 | 0.003 | 3.60E-01 |
| rs17207196 | 7 | 75101065 | T | C | -0.022 | 0.002 | 1.58E-36 | 0.001 | 0.002 | 6.10E-01 |
| rs17149254 | 7 | 76634463 | T | C | 0.024 | 0.002 | 2.99E-25 | -0.003 | 0.003 | 3.70E-01 |
| rs6973656 | 7 | 77422583 | A | G | -0.010 | 0.002 | 6.31E-09 | 0.001 | 0.002 | 7.40E-01 |
| rs1965529 | 7 | 77825707 | A | G | 0.016 | 0.002 | 7.66E-14 | -0.005 | 0.003 | 4.20E-02 |
| rs274628 | 7 | 86265855 | A | C | -0.010 | 0.002 | 1.36E-08 | -0.009 | 0.002 | 1.80E-04 |
| rs2283006 | 7 | 93085722 | A | G | 0.013 | 0.002 | 8.06E-15 | 0.000 | 0.002 | 8.80E-01 |
| rs13240600 | 7 | 99064466 | A | G | 0.018 | 0.002 | 7.90E-16 | 0.012 | 0.003 | 5.80E-05 |
| rs1721447 | 7 | 109214139 | T | G | -0.010 | 0.002 | 3.79E-09 | 0.000 | 0.002 | 9.10E-01 |
| rs2396625 | 7 | 113028634 | A | T | -0.018 | 0.002 | 2.81E-24 | 0.004 | 0.002 | 8.30E-02 |
| rs13245051 | 7 | 113362799 | A | G | 0.015 | 0.002 | 1.12E-18 | -0.002 | 0.002 | 4.80E-01 |
| rs10261050 | 7 | 114337652 | T | C | 0.011 | 0.002 | 4.53E-11 | -0.006 | 0.002 | 1.20E-02 |
| rs2283093 | 7 | 126721231 | T | C | 0.012 | 0.002 | 1.10E-08 | 0.007 | 0.003 | 7.90E-03 |
| rs7802342 | 7 | 137435925 | T | G | -0.012 | 0.002 | 6.23E-11 | -0.001 | 0.002 | 6.90E-01 |
| rs11525873 | 7 | 138817193 | T | C | 0.023 | 0.003 | 2.98E-13 | 0.003 | 0.004 | 4.50E-01 |
| rs11773362 | 7 | 147668180 | T | C | -0.011 | 0.002 | 6.39E-09 | 0.001 | 0.002 | 6.60E-01 |
| rs2907948 | 7 | 150638484 | A | G | -0.015 | 0.002 | 1.95E-14 | -0.003 | 0.003 | 3.10E-01 |
| rs56211164 | 7 | 158016764 | A | G | -0.013 | 0.002 | 8.90E-09 | -0.003 | 0.003 | 1.90E-01 |
| rs1700082 | 8 | 4121727 | C | G | 0.009 | 0.002 | 3.38E-08 | -0.001 | 0.002 | 7.20E-01 |
| rs4240673 | 8 | 10787612 | T | C | 0.018 | 0.002 | 1.58E-26 | 0.002 | 0.002 | 4.40E-01 |
| rs13263601 | 8 | 14095900 | A | C | -0.015 | 0.002 | 4.87E-16 | -0.003 | 0.002 | 1.50E-01 |
| rs10110189 | 8 | 15393380 | T | C | -0.016 | 0.003 | 2.20E-08 | 0.004 | 0.004 | 3.30E-01 |
| rs10101364 | 8 | 20634888 | T | C | 0.012 | 0.002 | 5.61E-11 | 0.005 | 0.002 | 2.60E-02 |
| rs73225274 | 8 | 21088909 | A | G | -0.016 | 0.003 | 2.33E-08 | 0.002 | 0.003 | 6.30E-01 |
| rs13249650 | 8 | 25664336 | A | T | 0.011 | 0.002 | 1.02E-08 | -0.007 | 0.002 | 1.80E-03 |
| rs1421334 | 8 | 30865733 | A | C | 0.014 | 0.002 | 3.11E-15 | -0.002 | 0.002 | 4.40E-01 |
| rs2466103 | 8 | 32412304 | T | G | -0.012 | 0.002 | 8.00E-12 | -0.002 | 0.002 | 5.00E-01 |
| rs6468266 | 8 | 34380276 | A | T | -0.011 | 0.002 | 7.71E-11 | 0.004 | 0.002 | 9.10E-02 |
| rs12681792 | 8 | 62054463 | A | C | 0.015 | 0.002 | 2.88E-12 | 0.005 | 0.003 | 5.80E-02 |
| rs12334877 | 8 | 67194171 | A | G | -0.014 | 0.002 | 2.17E-11 | -0.003 | 0.003 | 2.20E-01 |
| rs1808629 | 8 | 73435964 | A | G | -0.020 | 0.002 | 2.19E-23 | -0.002 | 0.002 | 3.30E-01 |
| rs17405819 | 8 | 76806584 | T | C | 0.021 | 0.002 | 6.04E-33 | 0.002 | 0.002 | 4.60E-01 |
| rs2196618 | 8 | 85089437 | A | G | -0.014 | 0.002 | 1.49E-12 | -0.003 | 0.003 | 2.10E-01 |
| rs12386885 | 8 | 87766769 | T | C | 0.015 | 0.003 | 9.90E-09 | -0.003 | 0.003 | 3.10E-01 |
| rs2120710 | 8 | 93210803 | A | G | 0.010 | 0.002 | 1.98E-08 | -0.003 | 0.002 | 1.60E-01 |
| rs12680842 | 8 | 95582606 | A | G | 0.014 | 0.002 | 3.41E-16 | 0.000 | 0.002 | 8.60E-01 |
| rs1383592 | 8 | 106430676 | A | G | 0.012 | 0.002 | 4.92E-09 | 0.001 | 0.003 | 6.00E-01 |
| rs3808477 | 8 | 116670347 | T | C | -0.018 | 0.002 | 8.73E-22 | -0.001 | 0.002 | 6.70E-01 |
| rs72673947 | 8 | 118884379 | A | G | -0.022 | 0.003 | 4.11E-13 | 0.000 | 0.004 | 9.60E-01 |
| rs6470144 | 8 | 124152245 | T | G | 0.010 | 0.002 | 3.06E-08 | -0.001 | 0.002 | 7.20E-01 |
| rs7842934 | 8 | 132838921 | T | C | -0.018 | 0.003 | 1.55E-08 | 0.001 | 0.004 | 7.40E-01 |
| rs305256 | 8 | 137568252 | T | C | -0.012 | 0.002 | 2.79E-08 | 0.005 | 0.003 | 4.00E-02 |
| rs16906838 | 8 | 138213836 | T | C | -0.025 | 0.004 | 4.31E-10 | 0.005 | 0.005 | 3.20E-01 |
| rs11782074 | 8 | 142617096 | T | G | 0.012 | 0.002 | 4.24E-12 | -0.006 | 0.002 | 4.70E-03 |
| rs10099330 | 8 | 143383694 | A | G | -0.012 | 0.002 | 3.22E-12 | -0.003 | 0.002 | 2.10E-01 |
| rs10975933 | 9 | 6954557 | C | G | 0.011 | 0.002 | 2.07E-10 | -0.003 | 0.002 | 1.40E-01 |
| rs1948080 | 9 | 11852043 | T | G | 0.014 | 0.002 | 1.13E-14 | 0.003 | 0.002 | 2.30E-01 |
| rs10961649 | 9 | 14670949 | T | C | 0.010 | 0.002 | 1.51E-08 | 0.002 | 0.002 | 3.70E-01 |
| rs4740619 | 9 | 15634326 | T | C | 0.019 | 0.002 | 3.15E-31 | 0.000 | 0.002 | 9.70E-01 |
| rs10962550 | 9 | 16720329 | C | G | 0.019 | 0.002 | 5.68E-17 | -0.002 | 0.003 | 4.00E-01 |
| rs10811868 | 9 | 23199959 | A | G | -0.010 | 0.002 | 3.18E-08 | -0.003 | 0.002 | 2.50E-01 |
| rs10968114 | 9 | 27800007 | A | C | 0.011 | 0.002 | 3.30E-11 | 0.001 | 0.002 | 6.30E-01 |
| rs1412235 | 9 | 28410996 | C | G | 0.024 | 0.002 | 2.28E-42 | 0.005 | 0.002 | 4.60E-02 |
| rs10121187 | 9 | 29737191 | C | G | 0.010 | 0.002 | 2.02E-09 | 0.002 | 0.002 | 5.00E-01 |
| rs2275003 | 9 | 34124860 | A | G | 0.011 | 0.002 | 9.44E-12 | -0.001 | 0.002 | 7.90E-01 |
| rs13296413 | 9 | 37258105 | T | C | -0.015 | 0.002 | 2.49E-17 | 0.000 | 0.002 | 8.60E-01 |
| rs2134858 | 9 | 73837155 | T | C | -0.012 | 0.002 | 5.87E-12 | 0.004 | 0.002 | 5.10E-02 |
| rs7861160 | 9 | 80799579 | T | C | 0.010 | 0.002 | 3.68E-08 | 0.002 | 0.002 | 3.10E-01 |
| rs1999433 | 9 | 81371441 | T | C | -0.011 | 0.002 | 2.90E-10 | 0.000 | 0.002 | 8.70E-01 |
| rs2777768 | 9 | 84186734 | A | G | 0.012 | 0.002 | 6.38E-10 | -0.001 | 0.002 | 5.50E-01 |
| rs7357754 | 9 | 92207308 | A | G | -0.012 | 0.002 | 1.81E-12 | 0.005 | 0.002 | 2.90E-02 |
| rs10992867 | 9 | 96461013 | A | G | 0.016 | 0.002 | 3.20E-17 | -0.001 | 0.003 | 7.00E-01 |
| rs7025938 | 9 | 103088321 | C | G | -0.016 | 0.002 | 1.47E-19 | 0.002 | 0.002 | 3.60E-01 |
| rs7024334 | 9 | 109072075 | T | G | 0.014 | 0.002 | 4.71E-12 | 0.001 | 0.003 | 7.90E-01 |
| rs1928295 | 9 | 120378483 | T | C | 0.013 | 0.002 | 2.23E-16 | 0.004 | 0.002 | 8.00E-02 |
| rs1877875 | 9 | 120664469 | T | C | -0.011 | 0.002 | 2.51E-10 | -0.004 | 0.002 | 8.50E-02 |
| rs13298487 | 9 | 126112104 | T | C | 0.012 | 0.002 | 1.43E-08 | -0.002 | 0.002 | 3.90E-01 |
| rs10733682 | 9 | 129460914 | A | G | 0.015 | 0.002 | 1.76E-19 | -0.002 | 0.002 | 4.50E-01 |
| rs2267958 | 9 | 131015279 | A | G | -0.013 | 0.002 | 2.01E-13 | -0.003 | 0.002 | 2.50E-01 |
| rs4740383 | 9 | 133783566 | A | G | 0.013 | 0.002 | 7.03E-14 | 0.001 | 0.002 | 5.80E-01 |
| rs10858334 | 9 | 137989785 | C | G | -0.015 | 0.003 | 4.91E-09 | -0.011 | 0.003 | 7.10E-04 |
| rs7907470 | 10 | 10268989 | A | G | -0.018 | 0.003 | 7.36E-09 | 0.003 | 0.004 | 5.30E-01 |
| rs7893571 | 10 | 16750129 | T | G | 0.013 | 0.002 | 5.83E-12 | 0.006 | 0.002 | 1.30E-02 |
| rs76638898 | 10 | 21099584 | A | G | -0.038 | 0.007 | 8.15E-09 | -0.001 | 0.007 | 8.80E-01 |
| rs7084454 | 10 | 21821274 | A | G | 0.020 | 0.002 | 4.51E-27 | 0.011 | 0.002 | 6.40E-06 |
| rs10829164 | 10 | 27318370 | T | C | 0.015 | 0.002 | 2.37E-10 | 0.008 | 0.003 | 9.20E-03 |
| rs4097319 | 10 | 33860515 | T | G | 0.011 | 0.002 | 4.32E-10 | -0.007 | 0.002 | 1.80E-03 |
| rs12765914 | 10 | 34013507 | T | C | 0.023 | 0.003 | 1.96E-13 | 0.009 | 0.004 | 2.40E-02 |
| rs1624134 | 10 | 34834482 | C | G | 0.010 | 0.002 | 6.51E-09 | 0.004 | 0.002 | 9.70E-02 |
| rs12259464 | 10 | 53680099 | A | G | 0.011 | 0.002 | 1.48E-10 | -0.005 | 0.002 | 3.80E-02 |
| rs7070670 | 10 | 61842645 | T | C | -0.013 | 0.002 | 7.14E-10 | -0.002 | 0.002 | 3.20E-01 |
| rs10761785 | 10 | 65318766 | T | G | -0.013 | 0.002 | 3.47E-16 | 0.001 | 0.002 | 6.30E-01 |
| rs12098284 | 10 | 76047464 | T | C | 0.018 | 0.003 | 9.87E-13 | 0.006 | 0.003 | 6.10E-02 |
| rs11001259 | 10 | 76807650 | A | T | -0.014 | 0.002 | 1.22E-08 | -0.001 | 0.003 | 7.50E-01 |
| rs7899106 | 10 | 87410904 | A | G | -0.033 | 0.004 | 1.72E-18 | -0.003 | 0.005 | 5.20E-01 |
| rs10788494 | 10 | 88110925 | C | G | 0.013 | 0.002 | 5.51E-15 | 0.000 | 0.002 | 9.70E-01 |
| rs147568678 | 10 | 93061851 | T | C | 0.013 | 0.002 | 3.42E-09 | 0.002 | 0.003 | 3.40E-01 |
| rs2439823 | 10 | 99778226 | A | G | -0.017 | 0.002 | 6.51E-22 | 0.000 | 0.002 | 9.20E-01 |
| rs17094222 | 10 | 102395440 | T | C | -0.017 | 0.002 | 4.04E-18 | 0.000 | 0.003 | 9.60E-01 |
| rs11594179 | 10 | 104392580 | T | C | -0.011 | 0.002 | 2.16E-08 | 0.007 | 0.003 | 8.60E-03 |
| rs79780963 | 10 | 104952499 | T | C | 0.024 | 0.004 | 2.63E-12 | 0.004 | 0.004 | 2.80E-01 |
| rs7903146 | 10 | 114758349 | T | C | -0.018 | 0.002 | 1.67E-23 | 0.000 | 0.002 | 8.40E-01 |
| rs2257791 | 10 | 118643670 | A | G | -0.014 | 0.002 | 1.63E-12 | -0.005 | 0.003 | 6.90E-02 |
| rs845084 | 10 | 125220036 | A | G | 0.014 | 0.002 | 3.22E-12 | 0.001 | 0.003 | 7.20E-01 |
| rs17636031 | 10 | 126594078 | T | C | -0.015 | 0.002 | 3.87E-17 | -0.002 | 0.002 | 5.10E-01 |
| rs4880341 | 10 | 133992689 | T | C | -0.013 | 0.002 | 3.06E-14 | -0.003 | 0.002 | 2.40E-01 |
| rs11246136 | 11 | 371265 | A | C | -0.017 | 0.003 | 2.03E-08 | -0.002 | 0.004 | 6.10E-01 |
| rs12421848 | 11 | 891338 | A | G | -0.014 | 0.002 | 3.99E-13 | -0.005 | 0.002 | 2.00E-02 |
| rs4256980 | 11 | 8673939 | C | G | -0.019 | 0.002 | 8.63E-29 | 0.000 | 0.002 | 9.30E-01 |
| rs900144 | 11 | 13294268 | T | C | 0.015 | 0.002 | 1.53E-18 | 0.006 | 0.002 | 1.20E-02 |
| rs2074314 | 11 | 17411821 | T | C | 0.011 | 0.002 | 1.37E-09 | 0.002 | 0.002 | 4.10E-01 |
| rs6265 | 11 | 27679916 | T | C | -0.041 | 0.002 | 7.40E-89 | -0.011 | 0.003 | 4.70E-05 |
| rs570463 | 11 | 28739318 | A | C | -0.012 | 0.002 | 4.33E-11 | 0.005 | 0.002 | 3.00E-02 |
| rs11030618 | 11 | 29243293 | T | C | 0.011 | 0.002 | 1.67E-10 | -0.002 | 0.002 | 3.80E-01 |
| rs2065418 | 11 | 30422068 | T | G | 0.014 | 0.002 | 6.07E-15 | 0.001 | 0.002 | 7.50E-01 |
| rs2862996 | 11 | 43653833 | T | G | -0.022 | 0.002 | 3.60E-35 | 0.000 | 0.002 | 9.50E-01 |
| rs10742752 | 11 | 45438374 | T | C | -0.012 | 0.002 | 1.20E-13 | -0.003 | 0.002 | 1.30E-01 |
| rs118081010 | 11 | 46174948 | T | C | 0.052 | 0.008 | 5.28E-12 | -0.005 | 0.009 | 5.70E-01 |
| rs7124681 | 11 | 47529947 | A | C | 0.026 | 0.002 | 3.96E-55 | 0.009 | 0.002 | 7.20E-05 |
| rs6591407 | 11 | 56914157 | A | C | -0.012 | 0.002 | 3.58E-09 | 0.001 | 0.003 | 7.20E-01 |
| rs562664 | 11 | 63823619 | T | C | -0.014 | 0.002 | 4.15E-10 | 0.000 | 0.003 | 8.70E-01 |
| rs7102454 | 11 | 65594820 | T | C | -0.017 | 0.002 | 3.84E-21 | -0.002 | 0.002 | 4.20E-01 |
| rs592483 | 11 | 69445173 | T | C | -0.014 | 0.002 | 1.53E-16 | 0.000 | 0.002 | 9.90E-01 |
| rs12282785 | 11 | 76476030 | A | C | -0.016 | 0.002 | 1.43E-11 | 0.000 | 0.003 | 9.70E-01 |
| rs349088 | 11 | 84814393 | A | C | -0.013 | 0.002 | 3.50E-14 | -0.002 | 0.002 | 4.50E-01 |
| rs10741329 | 11 | 89997796 | A | G | 0.012 | 0.002 | 3.91E-10 | 0.000 | 0.002 | 8.40E-01 |
| rs3019466 | 11 | 92476178 | T | C | -0.013 | 0.002 | 4.91E-08 | 0.001 | 0.003 | 6.30E-01 |
| rs2605603 | 11 | 93221105 | A | G | -0.010 | 0.002 | 2.04E-10 | 0.001 | 0.002 | 7.70E-01 |
| rs2513999 | 11 | 103019633 | A | G | -0.015 | 0.003 | 3.07E-09 | 0.005 | 0.003 | 1.00E-01 |
| rs12286929 | 11 | 115022404 | A | G | -0.018 | 0.002 | 1.93E-27 | 0.002 | 0.002 | 3.40E-01 |
| rs76942203 | 11 | 116973247 | A | G | 0.026 | 0.004 | 9.08E-11 | -0.010 | 0.005 | 4.60E-02 |
| rs3825061 | 11 | 118944675 | T | C | 0.014 | 0.002 | 6.15E-16 | -0.005 | 0.002 | 4.00E-02 |
| rs11218510 | 11 | 121922587 | A | G | -0.014 | 0.002 | 6.79E-13 | -0.005 | 0.002 | 1.70E-02 |
| rs35483388 | 11 | 122545146 | T | C | 0.013 | 0.002 | 2.61E-10 | -0.004 | 0.002 | 8.90E-02 |
| rs7944782 | 11 | 130795698 | T | G | -0.014 | 0.002 | 3.61E-17 | 0.002 | 0.002 | 4.80E-01 |
| rs2007518 | 11 | 132639606 | A | G | -0.013 | 0.002 | 1.96E-14 | -0.006 | 0.002 | 3.40E-03 |
| rs1941213 | 11 | 133125329 | A | C | 0.011 | 0.002 | 1.59E-08 | 0.000 | 0.003 | 8.70E-01 |
| rs329651 | 11 | 133767622 | T | G | 0.016 | 0.002 | 2.13E-14 | -0.009 | 0.003 | 1.90E-03 |
| rs12364470 | 11 | 134601012 | T | G | -0.019 | 0.002 | 2.18E-17 | -0.001 | 0.003 | 7.50E-01 |
| rs11611246 | 12 | 939480 | T | G | 0.022 | 0.002 | 2.04E-28 | 0.002 | 0.003 | 3.80E-01 |
| rs765125 | 12 | 2156207 | T | C | -0.010 | 0.002 | 1.27E-08 | -0.003 | 0.002 | 1.50E-01 |
| rs10772983 | 12 | 17141582 | T | C | -0.010 | 0.002 | 6.21E-10 | 0.002 | 0.002 | 2.90E-01 |
| rs11044430 | 12 | 19287416 | A | T | 0.016 | 0.002 | 3.77E-12 | 0.000 | 0.003 | 9.20E-01 |
| rs11046972 | 12 | 23705969 | T | C | 0.018 | 0.003 | 3.72E-08 | -0.004 | 0.004 | 3.20E-01 |
| rs10842240 | 12 | 24060075 | C | G | 0.020 | 0.003 | 1.21E-14 | 0.003 | 0.003 | 4.20E-01 |
| rs11170468 | 12 | 39430048 | A | C | 0.013 | 0.002 | 1.12E-11 | -0.005 | 0.003 | 5.10E-02 |
| rs1350430 | 12 | 41819215 | T | C | -0.013 | 0.002 | 2.08E-13 | -0.003 | 0.002 | 2.20E-01 |
| rs1126930 | 12 | 49399132 | C | G | 0.034 | 0.005 | 1.00E-10 | -0.003 | 0.006 | 6.00E-01 |
| rs7138803 | 12 | 50247468 | A | G | 0.030 | 0.002 | 3.10E-71 | 0.000 | 0.002 | 9.80E-01 |
| rs2271189 | 12 | 56494991 | A | G | -0.014 | 0.002 | 9.26E-16 | 0.005 | 0.002 | 1.60E-02 |
| rs7975187 | 12 | 60964108 | A | G | -0.014 | 0.002 | 3.86E-11 | 0.002 | 0.003 | 5.30E-01 |
| rs650198 | 12 | 69674595 | T | C | -0.014 | 0.002 | 6.43E-13 | 0.003 | 0.002 | 2.40E-01 |
| rs11115176 | 12 | 82465797 | T | C | 0.013 | 0.002 | 6.79E-12 | -0.002 | 0.003 | 4.60E-01 |
| rs10506971 | 12 | 89757937 | A | G | -0.014 | 0.002 | 4.69E-17 | 0.001 | 0.002 | 6.90E-01 |
| rs11105839 | 12 | 91237920 | A | T | -0.011 | 0.002 | 1.25E-11 | -0.001 | 0.002 | 6.30E-01 |
| rs2712665 | 12 | 99594947 | T | C | -0.011 | 0.002 | 5.40E-09 | 0.001 | 0.002 | 7.60E-01 |
| rs6539064 | 12 | 103706754 | C | G | 0.019 | 0.002 | 1.14E-23 | -0.002 | 0.003 | 5.30E-01 |
| rs1860561 | 12 | 110783241 | A | G | 0.016 | 0.002 | 1.71E-16 | -0.006 | 0.003 | 2.10E-02 |
| rs11066188 | 12 | 112610714 | A | G | -0.011 | 0.002 | 3.05E-12 | 0.001 | 0.002 | 5.70E-01 |
| rs2707183 | 12 | 116957607 | T | G | -0.009 | 0.002 | 4.89E-08 | -0.004 | 0.002 | 6.20E-02 |
| rs11615578 | 12 | 121714935 | T | C | 0.012 | 0.002 | 3.04E-09 | 0.004 | 0.003 | 1.20E-01 |
| rs12369179 | 12 | 122963550 | T | C | -0.034 | 0.003 | 2.32E-28 | 0.000 | 0.004 | 9.40E-01 |
| rs11060853 | 12 | 123424071 | A | G | -0.011 | 0.002 | 3.10E-08 | -0.002 | 0.002 | 2.70E-01 |
| rs11614340 | 12 | 133426483 | T | C | -0.012 | 0.002 | 1.87E-10 | 0.000 | 0.002 | 9.50E-01 |
| rs9512648 | 13 | 27933910 | A | G | 0.010 | 0.002 | 4.11E-08 | 0.003 | 0.002 | 1.10E-01 |
| rs7323 | 13 | 28009031 | C | G | -0.017 | 0.002 | 2.18E-18 | -0.005 | 0.002 | 2.60E-02 |
| rs4771218 | 13 | 28655311 | A | G | -0.014 | 0.002 | 3.18E-15 | -0.004 | 0.002 | 7.00E-02 |
| rs9595908 | 13 | 33184288 | T | C | 0.015 | 0.002 | 3.73E-20 | -0.001 | 0.002 | 8.00E-01 |
| rs9603697 | 13 | 40783323 | T | C | 0.013 | 0.002 | 1.69E-13 | -0.001 | 0.002 | 7.00E-01 |
| rs12429545 | 13 | 54102206 | A | G | 0.031 | 0.002 | 1.42E-37 | -0.005 | 0.003 | 1.20E-01 |
| rs962796 | 13 | 54385284 | T | C | 0.014 | 0.002 | 1.88E-11 | -0.002 | 0.003 | 4.00E-01 |
| rs9569777 | 13 | 58484786 | T | G | -0.020 | 0.002 | 3.43E-21 | -0.003 | 0.003 | 3.60E-01 |
| rs9527895 | 13 | 59367767 | T | C | -0.016 | 0.002 | 3.69E-12 | 0.001 | 0.003 | 7.80E-01 |
| rs8181823 | 13 | 65477940 | A | C | -0.013 | 0.002 | 4.36E-10 | -0.004 | 0.003 | 1.60E-01 |
| rs9599161 | 13 | 67434016 | T | C | 0.010 | 0.002 | 2.32E-09 | 0.005 | 0.002 | 3.10E-02 |
| rs1441264 | 13 | 79580919 | A | G | 0.017 | 0.002 | 7.60E-25 | 0.002 | 0.002 | 3.20E-01 |
| rs9531786 | 13 | 85983968 | C | G | -0.011 | 0.002 | 4.11E-10 | -0.006 | 0.002 | 6.70E-03 |
| rs77432547 | 13 | 86494817 | A | G | -0.017 | 0.002 | 1.44E-15 | 0.000 | 0.002 | 9.90E-01 |
| rs1927790 | 13 | 96922191 | T | C | -0.014 | 0.002 | 1.57E-17 | -0.004 | 0.002 | 4.70E-02 |
| rs9168 | 13 | 99101583 | A | C | -0.014 | 0.002 | 1.08E-13 | 0.003 | 0.002 | 2.80E-01 |
| rs9888533 | 13 | 107854612 | T | C | 0.013 | 0.002 | 1.38E-10 | -0.001 | 0.002 | 6.50E-01 |
| rs9522183 | 13 | 111977280 | T | G | -0.014 | 0.002 | 2.10E-12 | 0.003 | 0.002 | 1.60E-01 |
| rs12868881 | 13 | 112218924 | A | T | 0.014 | 0.002 | 1.40E-15 | -0.007 | 0.002 | 1.60E-03 |
| rs10132280 | 14 | 25928179 | A | C | -0.021 | 0.002 | 2.28E-33 | 0.000 | 0.002 | 9.40E-01 |
| rs4981693 | 14 | 29680331 | A | G | 0.020 | 0.002 | 7.89E-24 | 0.000 | 0.003 | 8.70E-01 |
| rs225882 | 14 | 30480123 | T | C | 0.011 | 0.002 | 8.56E-10 | -0.005 | 0.003 | 5.20E-02 |
| rs1958898 | 14 | 40886886 | C | G | -0.015 | 0.002 | 4.23E-12 | -0.001 | 0.003 | 6.10E-01 |
| rs61983990 | 14 | 41475003 | A | G | 0.020 | 0.004 | 1.91E-08 | -0.005 | 0.004 | 2.60E-01 |
| rs4900714 | 14 | 47302219 | T | G | 0.015 | 0.002 | 1.00E-18 | -0.002 | 0.002 | 3.50E-01 |
| rs217669 | 14 | 62360075 | T | C | -0.017 | 0.002 | 6.27E-16 | -0.002 | 0.002 | 4.80E-01 |
| rs4430672 | 14 | 63094407 | T | C | 0.012 | 0.002 | 5.00E-09 | 0.001 | 0.003 | 8.20E-01 |
| rs3902951 | 14 | 69789755 | T | G | -0.013 | 0.002 | 2.88E-12 | 0.005 | 0.003 | 6.60E-02 |
| rs17182027 | 14 | 73348130 | A | G | -0.011 | 0.002 | 1.46E-10 | 0.002 | 0.002 | 3.10E-01 |
| rs7144011 | 14 | 79940383 | T | G | 0.026 | 0.002 | 2.37E-40 | -0.007 | 0.003 | 5.80E-03 |
| rs4517716 | 14 | 83013876 | C | G | -0.012 | 0.002 | 4.27E-09 | -0.001 | 0.003 | 6.50E-01 |
| rs12888545 | 14 | 88308044 | A | G | -0.013 | 0.002 | 1.76E-11 | -0.002 | 0.003 | 5.50E-01 |
| rs1951455 | 14 | 91512339 | T | C | -0.015 | 0.002 | 6.05E-15 | -0.003 | 0.002 | 2.80E-01 |
| rs942066 | 14 | 94031914 | A | G | -0.020 | 0.002 | 2.36E-24 | 0.003 | 0.002 | 2.10E-01 |
| rs7161194 | 14 | 101529005 | A | G | 0.019 | 0.002 | 2.23E-24 | 0.000 | 0.002 | 9.30E-01 |
| rs10145749 | 14 | 102782109 | T | C | 0.016 | 0.003 | 8.33E-10 | -0.007 | 0.003 | 3.70E-02 |
| rs4906263 | 14 | 103249127 | C | G | -0.018 | 0.002 | 8.11E-23 | 0.003 | 0.002 | 1.60E-01 |
| rs2273175 | 14 | 104160141 | T | C | -0.012 | 0.002 | 3.69E-11 | -0.006 | 0.002 | 1.50E-02 |
| rs12594043 | 15 | 27034988 | C | G | 0.010 | 0.002 | 1.31E-09 | 0.001 | 0.002 | 5.50E-01 |
| rs7172627 | 15 | 31877690 | A | G | -0.011 | 0.002 | 1.81E-11 | -0.002 | 0.002 | 3.40E-01 |
| rs11636611 | 15 | 36391965 | T | C | 0.010 | 0.002 | 8.85E-10 | -0.001 | 0.002 | 6.70E-01 |
| rs9944219 | 15 | 46500612 | A | G | -0.012 | 0.002 | 8.14E-13 | -0.005 | 0.002 | 3.80E-02 |
| rs12912198 | 15 | 47103953 | T | C | -0.010 | 0.002 | 3.44E-08 | 0.001 | 0.002 | 5.80E-01 |
| rs6493498 | 15 | 51754451 | T | C | 0.014 | 0.002 | 4.84E-17 | 0.004 | 0.002 | 8.70E-02 |
| rs10518694 | 15 | 53072673 | A | C | 0.014 | 0.002 | 3.43E-09 | 0.000 | 0.003 | 9.60E-01 |
| rs12439632 | 15 | 59103963 | C | G | 0.015 | 0.002 | 3.68E-10 | 0.007 | 0.003 | 1.50E-02 |
| rs339991 | 15 | 60913637 | A | G | -0.013 | 0.002 | 3.51E-13 | -0.001 | 0.002 | 8.00E-01 |
| rs1559673 | 15 | 62156514 | A | C | 0.036 | 0.005 | 2.21E-13 | 0.004 | 0.008 | 5.80E-01 |
| rs2241423 | 15 | 68086838 | A | G | -0.030 | 0.002 | 3.60E-54 | 0.006 | 0.003 | 3.30E-02 |
| rs7171864 | 15 | 73227249 | A | G | 0.013 | 0.002 | 2.60E-14 | 0.001 | 0.002 | 7.80E-01 |
| rs2470893 | 15 | 75019449 | T | C | 0.011 | 0.002 | 9.43E-10 | -0.005 | 0.002 | 2.30E-02 |
| rs56151256 | 15 | 78024806 | A | C | 0.017 | 0.002 | 6.19E-14 | -0.001 | 0.003 | 7.20E-01 |
| rs12914623 | 15 | 80993570 | C | G | -0.016 | 0.002 | 2.00E-16 | -0.004 | 0.002 | 1.10E-01 |
| rs72757415 | 15 | 92572762 | T | G | -0.016 | 0.002 | 1.34E-11 | 0.002 | 0.003 | 5.30E-01 |
| rs11633626 | 15 | 95271378 | A | C | -0.016 | 0.002 | 7.27E-19 | 0.001 | 0.002 | 5.40E-01 |
| rs6496248 | 15 | 98262923 | A | T | 0.010 | 0.002 | 2.93E-09 | 0.002 | 0.002 | 4.90E-01 |
| rs12591120 | 15 | 99236869 | T | C | 0.012 | 0.002 | 3.84E-08 | 0.000 | 0.003 | 8.50E-01 |
| rs2715423 | 15 | 99511873 | A | G | -0.012 | 0.002 | 1.90E-09 | 0.000 | 0.002 | 9.50E-01 |
| rs214249 | 16 | 348687 | T | G | 0.014 | 0.002 | 2.72E-15 | -0.003 | 0.002 | 1.30E-01 |
| rs12448257 | 16 | 3599655 | A | G | 0.016 | 0.002 | 9.04E-16 | 0.005 | 0.003 | 5.70E-02 |
| rs879620 | 16 | 4015729 | T | C | 0.023 | 0.002 | 8.45E-39 | -0.003 | 0.002 | 1.70E-01 |
| rs2058527 | 16 | 6704749 | T | G | -0.012 | 0.002 | 1.80E-09 | -0.002 | 0.002 | 4.20E-01 |
| rs9926784 | 16 | 19941968 | T | C | 0.024 | 0.002 | 1.08E-30 | 0.000 | 0.003 | 9.40E-01 |
| rs194809 | 16 | 23804956 | A | G | 0.013 | 0.002 | 4.86E-09 | 0.008 | 0.003 | 2.90E-03 |
| rs2342892 | 16 | 24540806 | T | G | 0.013 | 0.002 | 1.33E-13 | 0.000 | 0.002 | 9.40E-01 |
| rs7498665 | 16 | 28883241 | A | G | -0.029 | 0.002 | 1.14E-66 | 0.005 | 0.002 | 1.90E-02 |
| rs3814883 | 16 | 29994922 | T | C | 0.023 | 0.002 | 1.47E-40 | 0.000 | 0.002 | 9.50E-01 |
| rs2193101 | 16 | 49035172 | C | G | 0.014 | 0.002 | 3.33E-11 | 0.002 | 0.003 | 6.00E-01 |
| rs16952482 | 16 | 53771583 | T | C | -0.015 | 0.003 | 3.06E-08 | 0.001 | 0.004 | 7.70E-01 |
| rs9937053 | 16 | 53799507 | A | G | 0.072 | 0.002 | 0.00E+00 | -0.004 | 0.002 | 6.80E-02 |
| rs8057911 | 16 | 54143352 | T | C | 0.013 | 0.002 | 3.66E-08 | 0.002 | 0.003 | 4.90E-01 |
| rs889398 | 16 | 69556715 | T | C | -0.020 | 0.002 | 3.23E-32 | -0.002 | 0.002 | 4.90E-01 |
| rs35949039 | 16 | 70572605 | T | G | -0.022 | 0.003 | 6.30E-12 | -0.008 | 0.004 | 2.00E-02 |
| rs12926250 | 16 | 72213316 | T | G | 0.018 | 0.003 | 1.78E-10 | 0.005 | 0.004 | 1.80E-01 |
| rs756717 | 16 | 72996162 | A | G | -0.013 | 0.002 | 2.38E-15 | -0.001 | 0.002 | 5.60E-01 |
| rs4390583 | 16 | 81694835 | A | C | -0.010 | 0.002 | 1.63E-08 | -0.001 | 0.002 | 7.50E-01 |
| rs12922346 | 16 | 82438337 | C | G | 0.013 | 0.002 | 1.48E-11 | 0.001 | 0.003 | 6.80E-01 |
| rs7206608 | 16 | 82872628 | C | G | -0.013 | 0.002 | 1.20E-12 | -0.003 | 0.002 | 2.60E-01 |
| rs3923783 | 17 | 1843189 | A | C | -0.022 | 0.002 | 4.12E-23 | 0.003 | 0.003 | 3.20E-01 |
| rs11078883 | 17 | 2138828 | C | G | -0.013 | 0.002 | 1.30E-12 | -0.003 | 0.002 | 2.00E-01 |
| rs7774 | 17 | 4801163 | A | C | 0.013 | 0.002 | 4.22E-10 | 0.001 | 0.002 | 5.90E-01 |
| rs1075901 | 17 | 15943910 | T | C | -0.012 | 0.002 | 4.43E-13 | -0.001 | 0.002 | 5.80E-01 |
| rs4986044 | 17 | 21261560 | T | C | -0.018 | 0.002 | 1.27E-27 | -0.002 | 0.002 | 2.70E-01 |
| rs8065172 | 17 | 31456969 | A | G | -0.012 | 0.002 | 5.29E-10 | -0.003 | 0.003 | 2.90E-01 |
| rs12150665 | 17 | 34914787 | T | C | 0.017 | 0.002 | 1.74E-24 | 0.001 | 0.002 | 8.00E-01 |
| rs6607337 | 17 | 35057373 | T | C | -0.012 | 0.002 | 2.55E-11 | 0.005 | 0.002 | 2.50E-02 |
| rs56161855 | 17 | 46288649 | A | T | -0.023 | 0.003 | 1.41E-16 | 0.001 | 0.003 | 7.90E-01 |
| rs9299 | 17 | 46669430 | T | C | 0.012 | 0.002 | 1.87E-11 | 0.001 | 0.002 | 6.90E-01 |
| rs11655587 | 17 | 47140794 | T | C | -0.021 | 0.002 | 6.87E-26 | 0.000 | 0.002 | 8.90E-01 |
| rs11649864 | 17 | 56093061 | A | G | 0.019 | 0.003 | 2.52E-10 | -0.010 | 0.004 | 1.30E-02 |
| rs12602912 | 17 | 65870073 | T | C | 0.017 | 0.002 | 2.90E-16 | 0.003 | 0.003 | 2.90E-01 |
| rs312750 | 17 | 68343539 | A | G | 0.010 | 0.002 | 2.57E-09 | -0.004 | 0.002 | 9.20E-02 |
| rs12939549 | 17 | 78611724 | A | G | 0.018 | 0.002 | 3.68E-28 | -0.004 | 0.002 | 1.10E-01 |
| rs35867081 | 17 | 79047278 | A | G | 0.015 | 0.002 | 6.65E-15 | 0.001 | 0.002 | 5.30E-01 |
| rs8097672 | 18 | 1839601 | A | T | -0.021 | 0.002 | 3.26E-18 | 0.008 | 0.003 | 8.50E-03 |
| rs478707 | 18 | 7543207 | T | C | -0.015 | 0.002 | 1.98E-10 | -0.001 | 0.003 | 8.60E-01 |
| rs891387 | 18 | 21103909 | T | C | 0.021 | 0.002 | 9.26E-35 | -0.007 | 0.002 | 6.80E-04 |
| rs1945160 | 18 | 22164216 | A | G | -0.010 | 0.002 | 5.65E-09 | 0.002 | 0.002 | 4.60E-01 |
| rs1941696 | 18 | 31252129 | A | G | 0.011 | 0.002 | 4.74E-11 | 0.004 | 0.002 | 8.50E-02 |
| rs474605 | 18 | 39612720 | A | G | -0.013 | 0.002 | 2.72E-13 | 0.001 | 0.002 | 6.40E-01 |
| rs1356506 | 18 | 40708038 | T | C | 0.014 | 0.002 | 8.39E-15 | 0.004 | 0.002 | 9.50E-02 |
| rs2612576 | 18 | 42967950 | A | T | -0.011 | 0.002 | 8.49E-09 | -0.003 | 0.002 | 2.80E-01 |
| rs9951619 | 18 | 56882326 | T | G | -0.015 | 0.002 | 2.33E-15 | -0.001 | 0.003 | 7.90E-01 |
| rs6567160 | 18 | 57829135 | T | C | -0.055 | 0.002 | 7.82E-184 | -0.004 | 0.003 | 1.60E-01 |
| rs17066856 | 18 | 58049656 | T | C | 0.035 | 0.003 | 7.10E-38 | 0.006 | 0.004 | 1.40E-01 |
| rs66595146 | 18 | 58204315 | A | C | 0.014 | 0.002 | 9.09E-12 | -0.006 | 0.002 | 8.30E-03 |
| rs17783165 | 18 | 63461638 | T | C | -0.013 | 0.002 | 2.74E-13 | 0.003 | 0.002 | 2.20E-01 |
| rs11150911 | 18 | 73498528 | A | C | 0.012 | 0.002 | 3.72E-11 | -0.005 | 0.002 | 3.10E-02 |
| rs12981256 | 19 | 1865901 | A | G | 0.015 | 0.002 | 1.43E-18 | -0.005 | 0.002 | 4.00E-02 |
| rs45486197 | 19 | 2244849 | A | G | 0.028 | 0.004 | 1.80E-12 | 0.007 | 0.004 | 1.20E-01 |
| rs895330 | 19 | 4060707 | C | G | 0.020 | 0.002 | 1.59E-19 | 0.005 | 0.003 | 5.10E-02 |
| rs273512 | 19 | 18224729 | T | C | 0.016 | 0.002 | 4.48E-19 | 0.002 | 0.002 | 3.20E-01 |
| rs17724992 | 19 | 18454825 | A | G | 0.017 | 0.002 | 5.23E-21 | -0.006 | 0.002 | 1.20E-02 |
| rs7258722 | 19 | 18808915 | A | T | -0.020 | 0.002 | 1.81E-30 | 0.001 | 0.002 | 6.60E-01 |
| rs12611148 | 19 | 19865077 | A | C | -0.014 | 0.002 | 1.42E-08 | -0.001 | 0.003 | 8.20E-01 |
| rs12462975 | 19 | 30272202 | A | G | 0.019 | 0.002 | 1.47E-25 | 0.003 | 0.002 | 2.00E-01 |
| rs7245985 | 19 | 30710410 | T | G | 0.012 | 0.002 | 1.88E-08 | 0.004 | 0.003 | 1.80E-01 |
| rs11882409 | 19 | 34019685 | A | C | 0.012 | 0.002 | 3.31E-10 | 0.009 | 0.002 | 4.00E-04 |
| rs185350 | 19 | 34306816 | T | C | 0.014 | 0.002 | 9.10E-17 | -0.001 | 0.002 | 6.00E-01 |
| rs429358 | 19 | 45411941 | T | C | 0.026 | 0.003 | 2.60E-22 | 0.011 | 0.003 | 2.60E-04 |
| rs11672660 | 19 | 46180184 | T | C | -0.034 | 0.002 | 6.83E-60 | -0.004 | 0.003 | 1.80E-01 |
| rs9304665 | 19 | 47602577 | A | T | 0.023 | 0.002 | 2.20E-31 | 0.004 | 0.003 | 1.10E-01 |
| rs3764625 | 19 | 49649051 | T | G | 0.010 | 0.002 | 2.42E-08 | -0.004 | 0.002 | 5.90E-02 |
| rs1884389 | 20 | 1410582 | T | C | -0.011 | 0.002 | 3.72E-10 | -0.002 | 0.002 | 3.30E-01 |
| rs4813619 | 20 | 2815715 | T | G | -0.011 | 0.002 | 1.70E-09 | -0.005 | 0.002 | 1.90E-02 |
| rs1884897 | 20 | 6612832 | A | G | -0.018 | 0.002 | 2.70E-28 | -0.001 | 0.002 | 6.40E-01 |
| rs2423668 | 20 | 12430673 | T | C | 0.011 | 0.002 | 7.84E-09 | 0.001 | 0.002 | 5.50E-01 |
| rs852056 | 20 | 17102860 | T | C | 0.012 | 0.002 | 2.15E-10 | 0.005 | 0.003 | 5.60E-02 |
| rs1409818 | 20 | 21381121 | T | C | 0.020 | 0.003 | 2.59E-12 | 0.003 | 0.004 | 4.90E-01 |
| rs8122855 | 20 | 25192049 | A | G | 0.014 | 0.002 | 4.11E-14 | 0.003 | 0.002 | 2.10E-01 |
| rs4812405 | 20 | 35276585 | A | C | -0.020 | 0.003 | 1.28E-09 | -0.004 | 0.004 | 2.90E-01 |
| rs742748 | 20 | 39293397 | T | C | -0.011 | 0.002 | 1.13E-11 | 0.000 | 0.002 | 8.50E-01 |
| rs79186842 | 20 | 47689036 | A | G | 0.020 | 0.003 | 2.60E-13 | 0.007 | 0.003 | 2.40E-02 |
| rs17806224 | 20 | 51065854 | A | G | -0.026 | 0.002 | 7.91E-32 | -0.007 | 0.003 | 1.00E-02 |
| rs1304549 | 20 | 54378256 | A | G | -0.012 | 0.002 | 1.35E-08 | -0.004 | 0.003 | 1.10E-01 |
| rs6010784 | 20 | 61540319 | T | C | 0.011 | 0.002 | 6.91E-11 | 0.010 | 0.002 | 5.40E-06 |
| rs6512302 | 20 | 62691550 | C | G | 0.013 | 0.002 | 1.52E-11 | -0.004 | 0.003 | 1.10E-01 |
| rs2064044 | 21 | 22119890 | A | C | -0.012 | 0.002 | 1.02E-08 | 0.003 | 0.003 | 2.80E-01 |
| rs2832283 | 21 | 30690558 | A | G | 0.012 | 0.002 | 4.72E-09 | -0.003 | 0.003 | 2.90E-01 |
| rs13047416 | 21 | 40309436 | C | G | 0.015 | 0.002 | 6.93E-18 | 0.000 | 0.002 | 8.50E-01 |
| rs8134638 | 21 | 40644170 | T | C | -0.013 | 0.002 | 1.57E-11 | 0.003 | 0.002 | 2.20E-01 |
| rs8126575 | 21 | 46435610 | T | G | 0.015 | 0.003 | 5.88E-10 | -0.001 | 0.003 | 7.20E-01 |
| rs427943 | 21 | 46570896 | A | C | -0.018 | 0.002 | 3.60E-25 | 0.001 | 0.002 | 5.00E-01 |
| rs11538 | 22 | 18220831 | A | G | -0.014 | 0.002 | 1.09E-09 | 0.002 | 0.003 | 4.90E-01 |
| rs2238799 | 22 | 20109325 | A | G | 0.010 | 0.002 | 8.65E-09 | 0.001 | 0.002 | 5.90E-01 |
| rs12628891 | 22 | 38317137 | T | C | -0.012 | 0.002 | 5.85E-10 | 0.006 | 0.002 | 9.80E-03 |
| rs12628051 | 22 | 40654276 | T | C | 0.016 | 0.002 | 2.90E-19 | -0.002 | 0.002 | 3.30E-01 |
| rs28489620 | 22 | 41804716 | A | G | -0.015 | 0.002 | 1.42E-12 | -0.001 | 0.002 | 6.60E-01 |
| rs9615337 | 22 | 48898559 | C | G | -0.011 | 0.002 | 2.05E-08 | -0.002 | 0.002 | 4.80E-01 |
| **2) MR of leisure computer use on BMI (men and women combined)** | | | | | | | | | | |
| SNP | CHR | POS | EA | OA | beta_bmi | se_bmi | pval_bmi | beta_pc | se_pc | pval_pc |
| rs2748985 | 1 | 1853184 | T | C | -0.014 | 0.002 | 5.53E-13 | -0.015 | 0.002 | 4.10E-12 |
| rs12145677 | 1 | 110023610 | G | A | -0.011 | 0.002 | 4.93E-09 | -0.017 | 0.002 | 1.20E-12 |
| rs7526456 | 1 | 197773734 | T | C | 0.007 | 0.002 | 1.71E-04 | -0.013 | 0.002 | 1.20E-08 |
| rs10754920 | 2 | 146456888 | C | G | 0.007 | 0.003 | 1.13E-02 | 0.020 | 0.003 | 6.70E-09 |
| rs6744254 | 2 | 155986195 | C | T | -0.006 | 0.002 | 8.39E-04 | -0.016 | 0.002 | 5.40E-13 |
| rs11274218 | 2 | 215035001 | C | T | 0.004 | 0.002 | 1.46E-02 | -0.014 | 0.002 | 2.50E-09 |
| rs4592851 | 2 | 215364232 | G | T | 0.008 | 0.002 | 6.60E-05 | -0.014 | 0.003 | 1.40E-08 |
| rs10208088 | 2 | 221055873 | C | T | 0.002 | 0.002 | 3.66E-01 | 0.013 | 0.002 | 4.40E-09 |
| rs11708955 | 3 | 49540114 | T | C | 0.015 | 0.002 | 3.34E-15 | -0.016 | 0.002 | 4.20E-11 |
| rs6774533 | 3 | 62471086 | C | T | -0.012 | 0.002 | 5.87E-09 | -0.015 | 0.002 | 1.20E-09 |
| rs78082503 | 3 | 116556913 | G | C | -0.001 | 0.002 | 6.07E-01 | -0.015 | 0.003 | 1.10E-08 |
| rs6857629 | 4 | 39684977 | G | A | -0.004 | 0.002 | 8.48E-02 | 0.015 | 0.003 | 1.90E-09 |
| rs2068625 | 4 | 159856739 | T | C | 0.007 | 0.002 | 1.39E-04 | -0.016 | 0.002 | 4.00E-11 |
| rs2220599 | 5 | 7378854 | C | G | 0.004 | 0.002 | 1.27E-02 | -0.016 | 0.002 | 2.40E-12 |
| rs11749912 | 5 | 88065628 | A | G | 0.008 | 0.002 | 2.34E-05 | 0.014 | 0.002 | 4.90E-10 |
| rs1469249 | 5 | 113837198 | G | A | -0.010 | 0.002 | 1.08E-06 | 0.016 | 0.003 | 9.50E-09 |
| rs162894 | 5 | 131611872 | T | G | 0.004 | 0.002 | 3.71E-02 | 0.013 | 0.002 | 1.10E-08 |
| rs56158102 | 5 | 144027803 | C | T | -0.001 | 0.002 | 4.22E-01 | 0.013 | 0.002 | 3.10E-08 |
| rs9477970 | 6 | 19082663 | T | A | -0.010 | 0.002 | 2.41E-06 | -0.016 | 0.003 | 1.00E-08 |
| rs2179517 | 6 | 26198845 | G | C | 0.008 | 0.002 | 3.29E-05 | -0.012 | 0.002 | 1.90E-08 |
| rs9372625 | 6 | 98344031 | G | A | 0.015 | 0.002 | 5.41E-17 | -0.018 | 0.002 | 5.70E-16 |
| rs17789218 | 6 | 100600097 | T | C | -0.011 | 0.002 | 4.78E-09 | -0.015 | 0.003 | 4.20E-09 |
| rs6935828 | 6 | 140811367 | C | T | -0.001 | 0.002 | 7.11E-01 | -0.012 | 0.002 | 4.30E-08 |
| rs55772938 | 7 | 70025113 | A | G | -0.009 | 0.002 | 4.43E-05 | -0.015 | 0.002 | 3.90E-10 |
| rs12706626 | 7 | 124531370 | G | A | -0.002 | 0.002 | 4.25E-01 | -0.013 | 0.002 | 1.40E-08 |
| rs3944151 | 7 | 126970005 | A | G | 0.000 | 0.002 | 9.81E-01 | 0.014 | 0.002 | 3.10E-08 |
| rs2345941 | 7 | 133337635 | A | G | -0.002 | 0.002 | 3.27E-01 | 0.015 | 0.002 | 4.00E-11 |
| rs13262595 | 8 | 143316970 | A | G | -0.005 | 0.002 | 4.97E-03 | -0.016 | 0.002 | 1.20E-12 |
| rs4977839 | 9 | 23355310 | G | A | -0.010 | 0.002 | 7.01E-07 | -0.020 | 0.002 | 4.70E-19 |
| rs113851275 | 9 | 98297220 | G | A | -0.002 | 0.003 | 6.23E-01 | -0.021 | 0.004 | 3.20E-09 |
| rs7020477 | 9 | 116827760 | A | G | 0.002 | 0.002 | 4.96E-01 | 0.014 | 0.002 | 1.20E-08 |
| rs73578186 | 9 | 126334485 | C | T | -0.005 | 0.002 | 2.79E-02 | 0.015 | 0.002 | 2.30E-10 |
| rs2734849 | 11 | 113270160 | A | G | 0.009 | 0.002 | 2.61E-08 | -0.014 | 0.002 | 8.20E-10 |
| rs1448355 | 11 | 131286685 | C | T | 0.001 | 0.002 | 4.80E-01 | -0.015 | 0.002 | 1.30E-10 |
| rs35933007 | 12 | 38929341 | G | A | 0.008 | 0.002 | 4.91E-04 | -0.015 | 0.003 | 9.20E-09 |
| rs1319734 | 12 | 40367517 | A | G | 0.003 | 0.002 | 9.89E-02 | 0.013 | 0.002 | 1.90E-08 |
| rs206965 | 12 | 120856332 | T | C | 0.003 | 0.002 | 2.07E-01 | 0.016 | 0.003 | 9.10E-09 |
| rs12874776 | 13 | 57794345 | C | A | -0.001 | 0.003 | 6.51E-01 | -0.023 | 0.004 | 8.00E-11 |
| rs198262 | 14 | 57283283 | C | T | -0.001 | 0.004 | 8.31E-01 | 0.030 | 0.005 | 3.90E-08 |
| rs166835 | 15 | 47716037 | C | T | 0.001 | 0.002 | 6.55E-01 | 0.013 | 0.002 | 3.40E-09 |
| rs4702 | 15 | 91426560 | G | A | 0.005 | 0.002 | 4.15E-03 | 0.012 | 0.002 | 3.80E-08 |
| rs6498759 | 16 | 18020295 | T | C | -0.001 | 0.002 | 7.31E-01 | 0.013 | 0.002 | 2.70E-08 |
| rs3730399 | 16 | 67229019 | A | G | -0.011 | 0.004 | 2.45E-03 | 0.025 | 0.004 | 3.30E-08 |
| rs4073003 | 17 | 19148305 | A | G | -0.003 | 0.002 | 1.64E-01 | 0.020 | 0.003 | 1.20E-09 |
| rs7209653 | 17 | 19882084 | T | C | 0.003 | 0.002 | 1.01E-01 | 0.016 | 0.002 | 9.10E-11 |
| rs12603813 | 17 | 43196584 | T | C | -0.001 | 0.002 | 6.96E-01 | 0.014 | 0.003 | 1.30E-08 |
| rs66643547 | 17 | 79375315 | C | T | 0.002 | 0.002 | 2.59E-01 | -0.015 | 0.002 | 3.20E-11 |
| rs784256 | 18 | 53398626 | G | A | -0.015 | 0.002 | 5.35E-11 | 0.019 | 0.003 | 6.20E-11 |
| rs6129084 | 20 | 59828857 | A | T | -0.005 | 0.002 | 1.68E-02 | -0.014 | 0.002 | 9.30E-10 |
| rs9608249 | 22 | 24613952 | G | A | -0.001 | 0.003 | 8.52E-01 | 0.017 | 0.003 | 2.20E-08 |
| rs136553 | 22 | 27255675 | C | T | -0.007 | 0.002 | 3.19E-04 | -0.015 | 0.002 | 2.30E-11 |
| rs7288455 | 22 | 39966547 | A | G | -0.001 | 0.002 | 5.60E-01 | 0.013 | 0.002 | 4.00E-09 |
| **3) MR of BMI (men) on leisure computer use** | | | | | | | | | | |
| SNP | CHR | POS | EA | OA | beta_bmi | se_bmi | pval_bmi | beta_pc | se_pc | pval_pc |
| rs2803316 | 1 | 1865298 | A | G | -0.015 | 0.003 | 4.03E-09 | -0.0148 | 0.0022 | 2.20E-11 |
| rs3762444 | 1 | 2427712 | T | C | -0.019 | 0.003 | 1.47E-14 | -0.0030 | 0.0022 | 1.70E-01 |
| rs11121210 | 1 | 8708529 | T | C | -0.015 | 0.003 | 7.86E-09 | 0.0069 | 0.0023 | 2.60E-03 |
| rs11581010 | 1 | 11212458 | A | G | -0.018 | 0.003 | 1.01E-09 | -0.0041 | 0.0026 | 1.10E-01 |
| rs2235549 | 1 | 23391775 | T | G | -0.021 | 0.003 | 8.27E-11 | 0.0098 | 0.0030 | 9.90E-04 |
| rs2271928 | 1 | 32127953 | A | G | -0.014 | 0.002 | 9.92E-09 | -0.0014 | 0.0022 | 5.40E-01 |
| rs12061406 | 1 | 40073479 | T | C | -0.027 | 0.005 | 1.70E-09 | -0.0031 | 0.0039 | 4.30E-01 |
| rs1768808 | 1 | 46503217 | T | C | 0.014 | 0.003 | 3.54E-08 | -0.0007 | 0.0023 | 7.50E-01 |
| rs977747 | 1 | 47684677 | T | G | 0.016 | 0.002 | 1.35E-11 | 0.0037 | 0.0022 | 1.00E-01 |
| rs7531656 | 1 | 49828663 | A | G | 0.020 | 0.003 | 2.32E-15 | -0.0026 | 0.0024 | 2.60E-01 |
| rs2481665 | 1 | 62594677 | T | C | 0.019 | 0.002 | 2.03E-15 | -0.0037 | 0.0022 | 9.00E-02 |
| rs11208779 | 1 | 66417145 | C | G | 0.019 | 0.003 | 1.53E-11 | -0.0004 | 0.0022 | 8.60E-01 |
| rs1993709 | 1 | 72838529 | A | G | -0.041 | 0.003 | 1.49E-42 | -0.0004 | 0.0028 | 8.80E-01 |
| rs12566985 | 1 | 75002193 | A | G | -0.018 | 0.002 | 1.09E-14 | 0.0041 | 0.0022 | 6.80E-02 |
| rs12049202 | 1 | 77967523 | T | C | 0.023 | 0.003 | 5.52E-13 | 0.0046 | 0.0028 | 9.60E-02 |
| rs12034762 | 1 | 96290845 | T | C | 0.017 | 0.003 | 4.80E-11 | 0.0006 | 0.0023 | 8.10E-01 |
| rs11165643 | 1 | 96924097 | T | C | 0.020 | 0.002 | 1.69E-17 | 0.0034 | 0.0022 | 1.30E-01 |
| rs17024393 | 1 | 110154688 | T | C | -0.071 | 0.007 | 1.70E-23 | -0.0242 | 0.0069 | 4.80E-04 |
| rs61813324 | 1 | 156049877 | T | C | 0.028 | 0.004 | 1.29E-10 | -0.0002 | 0.0033 | 9.50E-01 |
| rs76194251 | 1 | 173931908 | C | G | -0.060 | 0.010 | 4.04E-10 | -0.0037 | 0.0074 | 6.20E-01 |
| rs543874 | 1 | 177889480 | A | G | -0.036 | 0.003 | 3.70E-35 | 0.0020 | 0.0027 | 4.50E-01 |
| rs10920678 | 1 | 190239907 | A | G | 0.013 | 0.002 | 1.90E-08 | 0.0006 | 0.0022 | 7.70E-01 |
| rs2172935 | 1 | 201826340 | T | C | 0.027 | 0.003 | 3.56E-24 | -0.0057 | 0.0023 | 1.50E-02 |
| rs12217091 | 1 | 204813050 | T | C | 0.027 | 0.005 | 4.44E-08 | -0.0001 | 0.0039 | 9.80E-01 |
| rs823094 | 1 | 205689807 | T | G | 0.015 | 0.002 | 3.77E-10 | 0.0038 | 0.0022 | 8.80E-02 |
| rs4653942 | 1 | 228505204 | A | G | -0.016 | 0.003 | 4.41E-08 | 0.0037 | 0.0028 | 1.90E-01 |
| rs2125231 | 1 | 243762299 | T | C | 0.017 | 0.003 | 5.50E-11 | -0.0046 | 0.0024 | 5.20E-02 |
| rs76111507 | 2 | 455477 | T | C | -0.081 | 0.008 | 1.13E-25 | -0.0106 | 0.0058 | 6.80E-02 |
| rs12714415 | 2 | 651430 | T | C | 0.060 | 0.003 | 1.09E-80 | 0.0083 | 0.0030 | 6.30E-03 |
| rs2693826 | 2 | 6160943 | A | G | -0.016 | 0.003 | 4.96E-11 | 0.0020 | 0.0022 | 3.60E-01 |
| rs10182181 | 2 | 25150296 | A | G | -0.027 | 0.002 | 5.55E-32 | -0.0035 | 0.0022 | 1.10E-01 |
| rs1260326 | 2 | 27730940 | T | C | -0.018 | 0.002 | 2.05E-14 | -0.0014 | 0.0022 | 5.40E-01 |
| rs2888172 | 2 | 35439812 | A | G | -0.014 | 0.003 | 1.42E-08 | 0.0006 | 0.0022 | 7.80E-01 |
| rs10185199 | 2 | 40282202 | A | G | -0.016 | 0.003 | 3.79E-08 | -0.0019 | 0.0025 | 4.50E-01 |
| rs7561278 | 2 | 48954905 | T | C | 0.018 | 0.003 | 1.06E-08 | -0.0009 | 0.0027 | 7.30E-01 |
| rs12986742 | 2 | 58975143 | T | C | -0.020 | 0.003 | 3.22E-15 | -0.0020 | 0.0022 | 3.70E-01 |
| rs887912 | 2 | 59302877 | T | C | 0.023 | 0.003 | 2.75E-19 | -0.0014 | 0.0024 | 5.60E-01 |
| rs11685481 | 2 | 67736749 | T | C | 0.020 | 0.003 | 8.99E-09 | -0.0012 | 0.0030 | 6.90E-01 |
| rs4410328 | 2 | 69600683 | T | C | -0.020 | 0.003 | 7.75E-12 | -0.0031 | 0.0022 | 1.70E-01 |
| rs10209325 | 2 | 86806356 | T | C | -0.018 | 0.003 | 1.49E-09 | -0.0006 | 0.0023 | 7.90E-01 |
| rs1437377 | 2 | 100744924 | T | G | -0.024 | 0.004 | 1.47E-11 | 0.0015 | 0.0032 | 6.40E-01 |
| rs10197031 | 2 | 105454590 | T | C | -0.017 | 0.003 | 7.69E-10 | 0.0071 | 0.0024 | 3.60E-03 |
| rs13405033 | 2 | 113994595 | T | C | 0.026 | 0.004 | 4.37E-11 | -0.0010 | 0.0031 | 7.40E-01 |
| rs11687120 | 2 | 119579558 | A | G | 0.014 | 0.003 | 4.74E-08 | -0.0017 | 0.0022 | 4.30E-01 |
| rs961360 | 2 | 136393658 | A | G | 0.021 | 0.003 | 3.75E-10 | 0.0016 | 0.0035 | 6.50E-01 |
| rs453520 | 2 | 147907202 | T | C | -0.016 | 0.003 | 1.19E-10 | 0.0001 | 0.0022 | 9.80E-01 |
| rs34234296 | 2 | 175166636 | A | G | -0.018 | 0.003 | 2.86E-09 | -0.0053 | 0.0023 | 1.90E-02 |
| rs1019612 | 2 | 181602526 | T | C | 0.018 | 0.003 | 1.40E-11 | -0.0010 | 0.0023 | 6.70E-01 |
| rs10497807 | 2 | 198585087 | C | G | -0.015 | 0.002 | 4.95E-10 | 0.0107 | 0.0022 | 1.20E-06 |
| rs7569376 | 2 | 205385322 | T | C | 0.020 | 0.003 | 1.33E-10 | -0.0003 | 0.0027 | 9.10E-01 |
| rs17203016 | 2 | 208255518 | A | G | -0.017 | 0.003 | 9.91E-09 | 0.0127 | 0.0027 | 3.80E-06 |
| rs7599312 | 2 | 213413231 | A | G | -0.022 | 0.003 | 1.51E-16 | 0.0086 | 0.0025 | 5.70E-04 |
| rs1541777 | 2 | 219587291 | A | G | -0.016 | 0.002 | 1.82E-11 | -0.0008 | 0.0022 | 7.10E-01 |
| rs7600417 | 2 | 220169492 | A | G | 0.021 | 0.004 | 2.88E-09 | 0.0020 | 0.0031 | 5.30E-01 |
| rs11692572 | 2 | 227774503 | C | G | -0.014 | 0.003 | 3.72E-08 | -0.0024 | 0.0022 | 2.80E-01 |
| rs2596902 | 3 | 12955982 | A | G | -0.015 | 0.003 | 1.30E-08 | -0.0009 | 0.0023 | 7.00E-01 |
| rs6804842 | 3 | 25106437 | A | G | -0.018 | 0.002 | 1.29E-14 | 0.0012 | 0.0022 | 5.80E-01 |
| rs762318 | 3 | 38516369 | A | C | 0.015 | 0.003 | 2.13E-09 | 0.0040 | 0.0023 | 7.80E-02 |
| rs1524887 | 3 | 44016371 | T | C | -0.014 | 0.003 | 3.60E-08 | -0.0020 | 0.0022 | 3.60E-01 |
| rs6803741 | 3 | 48123540 | T | C | -0.015 | 0.003 | 1.99E-08 | 0.0086 | 0.0024 | 2.80E-04 |
| rs2188151 | 3 | 50201924 | T | G | 0.028 | 0.003 | 2.73E-29 | -0.0075 | 0.0022 | 8.10E-04 |
| rs6787805 | 3 | 51204788 | A | G | -0.021 | 0.004 | 2.31E-09 | 0.0021 | 0.0033 | 5.20E-01 |
| rs2365389 | 3 | 61236462 | T | C | -0.018 | 0.002 | 5.27E-14 | 0.0020 | 0.0022 | 3.70E-01 |
| rs11915371 | 3 | 70539559 | A | C | -0.022 | 0.003 | 1.06E-12 | -0.0064 | 0.0027 | 1.90E-02 |
| rs13078509 | 3 | 70881429 | T | C | 0.048 | 0.008 | 8.37E-09 | -0.0086 | 0.0072 | 2.40E-01 |
| rs3849570 | 3 | 81792112 | A | C | 0.015 | 0.003 | 2.56E-09 | -0.0054 | 0.0023 | 1.90E-02 |
| rs12495178 | 3 | 85886077 | T | C | 0.023 | 0.002 | 1.54E-20 | -0.0024 | 0.0023 | 2.90E-01 |
| rs73140133 | 3 | 88067498 | T | C | -0.021 | 0.003 | 2.56E-12 | 0.0010 | 0.0023 | 6.80E-01 |
| rs1492014 | 3 | 94071481 | T | C | -0.021 | 0.003 | 1.64E-17 | 0.0024 | 0.0022 | 2.80E-01 |
| rs1436351 | 3 | 104617973 | T | G | 0.018 | 0.003 | 3.39E-11 | -0.0037 | 0.0025 | 1.50E-01 |
| rs6768228 | 3 | 108120008 | A | G | -0.015 | 0.003 | 6.77E-09 | 0.0041 | 0.0023 | 7.40E-02 |
| rs2918217 | 3 | 115546380 | T | C | 0.022 | 0.004 | 7.18E-10 | 0.0022 | 0.0032 | 4.90E-01 |
| rs1320903 | 3 | 131758077 | A | G | 0.026 | 0.003 | 1.65E-21 | -0.0016 | 0.0024 | 4.90E-01 |
| rs645040 | 3 | 135926622 | T | G | 0.019 | 0.003 | 3.16E-11 | 0.0064 | 0.0026 | 1.40E-02 |
| rs2035935 | 3 | 141306013 | A | G | -0.033 | 0.005 | 3.99E-13 | -0.0062 | 0.0044 | 1.60E-01 |
| rs355777 | 3 | 154034950 | C | G | 0.017 | 0.003 | 1.81E-11 | -0.0035 | 0.0022 | 1.20E-01 |
| rs9870575 | 3 | 156296559 | A | G | -0.024 | 0.004 | 1.48E-11 | 0.0006 | 0.0031 | 8.50E-01 |
| rs7356034 | 3 | 170732599 | A | G | 0.022 | 0.003 | 6.24E-12 | 0.0013 | 0.0024 | 6.00E-01 |
| rs2606227 | 3 | 183536836 | T | C | 0.014 | 0.003 | 4.61E-08 | -0.0054 | 0.0023 | 1.90E-02 |
| rs9816226 | 3 | 185834499 | A | T | -0.034 | 0.003 | 5.05E-29 | 0.0020 | 0.0028 | 4.90E-01 |
| rs13135416 | 4 | 18346297 | T | C | 0.018 | 0.003 | 1.56E-08 | 0.0005 | 0.0024 | 8.30E-01 |
| rs34811474 | 4 | 25408838 | A | G | -0.028 | 0.003 | 6.48E-16 | 0.0028 | 0.0026 | 2.80E-01 |
| rs2242189 | 4 | 38691024 | T | C | 0.017 | 0.003 | 5.55E-11 | -0.0062 | 0.0023 | 6.90E-03 |
| rs13130484 | 4 | 45175691 | T | C | 0.035 | 0.002 | 6.44E-50 | -0.0059 | 0.0022 | 8.00E-03 |
| rs2237025 | 4 | 55541879 | T | C | 0.014 | 0.003 | 3.10E-08 | 0.0009 | 0.0022 | 6.80E-01 |
| rs13107325 | 4 | 103188709 | T | C | 0.050 | 0.005 | 3.22E-26 | 0.0013 | 0.0042 | 7.60E-01 |
| rs2613726 | 4 | 112597321 | A | G | -0.016 | 0.003 | 4.34E-10 | -0.0012 | 0.0023 | 6.20E-01 |
| rs2391518 | 4 | 130817060 | T | C | 0.016 | 0.003 | 1.74E-10 | -0.0044 | 0.0024 | 6.70E-02 |
| rs1296328 | 4 | 137083193 | A | C | 0.020 | 0.003 | 2.07E-15 | -0.0023 | 0.0022 | 2.90E-01 |
| rs6537285 | 4 | 145310147 | A | G | -0.018 | 0.003 | 1.24E-10 | -0.0042 | 0.0024 | 8.30E-02 |
| rs6827083 | 4 | 153075491 | A | G | -0.015 | 0.002 | 1.34E-10 | -0.0073 | 0.0022 | 9.80E-04 |
| rs828544 | 5 | 3520064 | T | G | 0.014 | 0.003 | 4.45E-08 | 0.0044 | 0.0022 | 4.50E-02 |
| rs1503526 | 5 | 63020706 | T | C | -0.014 | 0.002 | 5.74E-09 | -0.0017 | 0.0022 | 4.40E-01 |
| rs253414 | 5 | 74956517 | T | C | 0.026 | 0.003 | 1.10E-24 | -0.0059 | 0.0023 | 1.20E-02 |
| rs4532349 | 5 | 77473165 | A | G | 0.016 | 0.003 | 6.64E-09 | 0.0075 | 0.0026 | 3.70E-03 |
| rs7733438 | 5 | 87986284 | T | G | -0.035 | 0.004 | 2.07E-21 | -0.0103 | 0.0032 | 1.60E-03 |
| rs6235 | 5 | 95728898 | C | G | -0.021 | 0.003 | 1.92E-13 | 0.0021 | 0.0025 | 4.00E-01 |
| rs11951673 | 5 | 95861012 | T | C | -0.019 | 0.002 | 7.83E-15 | -0.0043 | 0.0022 | 5.30E-02 |
| rs289228 | 5 | 107405834 | C | G | 0.022 | 0.003 | 9.78E-12 | 0.0016 | 0.0029 | 5.90E-01 |
| rs7711753 | 5 | 122733317 | A | G | -0.013 | 0.002 | 2.64E-08 | 0.0009 | 0.0022 | 7.00E-01 |
| rs7715256 | 5 | 153537893 | T | G | -0.016 | 0.002 | 4.52E-12 | 0.0036 | 0.0022 | 1.10E-01 |
| rs7730898 | 5 | 170459675 | A | G | 0.017 | 0.003 | 9.94E-11 | 0.0021 | 0.0025 | 3.90E-01 |
| rs6556301 | 5 | 176527577 | T | G | -0.014 | 0.003 | 1.57E-08 | -0.0007 | 0.0023 | 7.70E-01 |
| rs2228213 | 6 | 12124855 | A | G | -0.015 | 0.003 | 6.77E-10 | -0.0025 | 0.0023 | 2.70E-01 |
| rs9366639 | 6 | 26167613 | C | G | 0.019 | 0.003 | 3.82E-09 | -0.0084 | 0.0029 | 3.30E-03 |
| rs2269553 | 6 | 28876509 | A | G | -0.019 | 0.003 | 2.23E-11 | 0.0065 | 0.0027 | 1.60E-02 |
| rs9277992 | 6 | 33312455 | A | G | 0.028 | 0.004 | 8.69E-14 | 0.0042 | 0.0028 | 1.40E-01 |
| rs2744974 | 6 | 34579431 | T | C | 0.023 | 0.003 | 8.48E-20 | 0.0051 | 0.0023 | 3.00E-02 |
| rs34045288 | 6 | 40369081 | T | C | 0.025 | 0.003 | 3.95E-16 | -0.0018 | 0.0023 | 4.50E-01 |
| rs2206277 | 6 | 50798526 | T | C | 0.045 | 0.003 | 5.35E-50 | 0.0020 | 0.0029 | 4.90E-01 |
| rs1327259 | 6 | 51177811 | A | G | 0.016 | 0.003 | 5.96E-10 | -0.0007 | 0.0023 | 7.40E-01 |
| rs7773045 | 6 | 81443423 | T | G | 0.026 | 0.004 | 1.60E-09 | -0.0056 | 0.0040 | 1.60E-01 |
| rs12663742 | 6 | 97792735 | A | G | -0.018 | 0.003 | 1.69E-12 | 0.0028 | 0.0023 | 2.20E-01 |
| rs4548017 | 6 | 98399521 | A | G | -0.024 | 0.003 | 1.13E-15 | 0.0172 | 0.0023 | 2.10E-14 |
| rs17789218 | 6 | 100600097 | T | C | -0.015 | 0.003 | 3.33E-08 | -0.0151 | 0.0026 | 4.20E-09 |
| rs768023 | 6 | 108876002 | A | G | 0.013 | 0.002 | 3.05E-08 | 0.0010 | 0.0023 | 6.60E-01 |
| rs1159974 | 6 | 126090277 | T | C | -0.014 | 0.003 | 2.03E-08 | -0.0033 | 0.0022 | 1.30E-01 |
| rs6569648 | 6 | 130349119 | T | C | -0.015 | 0.003 | 3.66E-08 | -0.0063 | 0.0026 | 1.40E-02 |
| rs765875 | 6 | 143185683 | T | C | -0.014 | 0.003 | 3.43E-08 | 0.0036 | 0.0022 | 1.10E-01 |
| rs13191362 | 6 | 163033350 | A | G | 0.027 | 0.004 | 2.34E-13 | -0.0053 | 0.0033 | 1.10E-01 |
| rs9364687 | 6 | 163817911 | T | G | -0.014 | 0.002 | 3.97E-09 | -0.0088 | 0.0022 | 8.40E-05 |
| rs9638713 | 7 | 14645949 | A | G | 0.045 | 0.008 | 2.32E-09 | -0.0041 | 0.0070 | 5.60E-01 |
| rs215635 | 7 | 32370220 | T | C | -0.018 | 0.003 | 1.16E-12 | 0.0032 | 0.0023 | 1.70E-01 |
| rs77095142 | 7 | 39096572 | A | C | 0.046 | 0.008 | 2.37E-08 | 0.0157 | 0.0061 | 1.00E-02 |
| rs10269783 | 7 | 49616203 | A | G | 0.017 | 0.002 | 7.59E-13 | 0.0037 | 0.0023 | 1.00E-01 |
| rs1167827 | 7 | 75163169 | A | G | -0.022 | 0.002 | 2.88E-19 | 0.0015 | 0.0022 | 5.10E-01 |
| rs2245368 | 7 | 76608143 | T | C | -0.033 | 0.003 | 4.39E-23 | 0.0014 | 0.0029 | 6.40E-01 |
| rs1965529 | 7 | 77825707 | A | G | 0.018 | 0.003 | 4.22E-08 | -0.0055 | 0.0027 | 4.20E-02 |
| rs1701829 | 7 | 113033426 | A | G | 0.021 | 0.003 | 6.76E-16 | -0.0021 | 0.0023 | 3.70E-01 |
| rs13228237 | 7 | 138780222 | C | G | -0.016 | 0.003 | 4.59E-08 | -0.0034 | 0.0027 | 2.00E-01 |
| rs4841504 | 8 | 11024663 | A | C | -0.019 | 0.002 | 4.24E-16 | -0.0011 | 0.0022 | 6.30E-01 |
| rs4123853 | 8 | 14091025 | T | C | 0.017 | 0.003 | 1.10E-10 | 0.0023 | 0.0023 | 3.10E-01 |
| rs354510 | 8 | 15534749 | T | C | 0.024 | 0.004 | 1.98E-08 | -0.0054 | 0.0033 | 1.00E-01 |
| rs7827210 | 8 | 30871656 | A | G | 0.018 | 0.003 | 2.34E-12 | -0.0074 | 0.0023 | 9.80E-04 |
| rs7012923 | 8 | 33630937 | A | C | 0.016 | 0.003 | 4.92E-11 | 0.0045 | 0.0022 | 4.40E-02 |
| rs35957544 | 8 | 73440371 | T | G | -0.018 | 0.003 | 4.14E-10 | -0.0049 | 0.0022 | 2.80E-02 |
| rs2588785 | 8 | 76636191 | A | G | -0.021 | 0.003 | 1.74E-15 | 0.0019 | 0.0025 | 4.50E-01 |
| rs957447 | 8 | 95541223 | A | C | -0.023 | 0.003 | 8.56E-14 | -0.0011 | 0.0026 | 6.70E-01 |
| rs2960151 | 8 | 116718620 | A | C | 0.023 | 0.003 | 3.22E-11 | -0.0007 | 0.0026 | 7.90E-01 |
| rs72673947 | 8 | 118884379 | A | G | -0.028 | 0.005 | 1.76E-09 | -0.0002 | 0.0036 | 9.60E-01 |
| rs7826388 | 8 | 128109776 | T | C | -0.037 | 0.007 | 1.40E-08 | -0.0013 | 0.0061 | 8.30E-01 |
| rs4740619 | 9 | 15634326 | T | C | 0.019 | 0.002 | 1.50E-15 | -0.0001 | 0.0022 | 9.70E-01 |
| rs10738451 | 9 | 16713666 | T | C | -0.016 | 0.003 | 2.22E-09 | -0.0012 | 0.0025 | 6.10E-01 |
| rs10968114 | 9 | 27800007 | A | C | 0.015 | 0.003 | 8.70E-10 | 0.0011 | 0.0022 | 6.30E-01 |
| rs1412235 | 9 | 28410996 | C | G | 0.023 | 0.003 | 9.85E-21 | 0.0047 | 0.0024 | 4.60E-02 |
| rs10746862 | 9 | 73780912 | T | G | -0.016 | 0.003 | 8.63E-10 | 0.0031 | 0.0022 | 1.60E-01 |
| rs1187351 | 9 | 87295077 | A | C | -0.014 | 0.003 | 4.51E-08 | -0.0031 | 0.0022 | 1.70E-01 |
| rs6559921 | 9 | 88872039 | A | T | 0.018 | 0.003 | 1.19E-09 | -0.0043 | 0.0027 | 1.00E-01 |
| rs6477694 | 9 | 111932342 | T | C | -0.014 | 0.002 | 8.89E-09 | -0.0041 | 0.0023 | 7.40E-02 |
| rs7038943 | 9 | 120377178 | T | C | 0.015 | 0.003 | 6.54E-09 | 0.0030 | 0.0023 | 2.00E-01 |
| rs10760279 | 9 | 126105291 | T | G | 0.015 | 0.003 | 7.49E-09 | -0.0046 | 0.0023 | 4.50E-02 |
| rs3902840 | 9 | 129419025 | A | G | 0.031 | 0.004 | 2.49E-12 | -0.0085 | 0.0039 | 2.90E-02 |
| rs4740383 | 9 | 133783566 | A | G | 0.015 | 0.003 | 2.67E-09 | 0.0012 | 0.0022 | 5.80E-01 |
| rs11251291 | 10 | 2561766 | A | C | -0.014 | 0.003 | 1.65E-08 | 0.0014 | 0.0022 | 5.40E-01 |
| rs12251016 | 10 | 21821918 | A | T | -0.019 | 0.003 | 1.43E-10 | -0.0116 | 0.0023 | 6.30E-07 |
| rs3758438 | 10 | 27400661 | T | C | 0.027 | 0.005 | 7.27E-09 | 0.0060 | 0.0041 | 1.40E-01 |
| rs12761779 | 10 | 63782043 | C | G | 0.017 | 0.003 | 1.48E-08 | -0.0015 | 0.0023 | 5.20E-01 |
| rs7072873 | 10 | 77210191 | T | C | -0.015 | 0.003 | 5.45E-09 | 0.0004 | 0.0022 | 8.40E-01 |
| rs10788494 | 10 | 88110925 | C | G | 0.016 | 0.003 | 5.39E-10 | -0.0001 | 0.0022 | 9.70E-01 |
| rs10883026 | 10 | 99793865 | T | C | -0.019 | 0.003 | 6.23E-11 | 0.0014 | 0.0022 | 5.40E-01 |
| rs17094222 | 10 | 102395440 | T | C | -0.026 | 0.003 | 1.14E-18 | 0.0001 | 0.0027 | 9.60E-01 |
| rs11191356 | 10 | 104393489 | A | G | -0.026 | 0.004 | 2.48E-10 | 0.0036 | 0.0041 | 3.70E-01 |
| rs79780963 | 10 | 104952499 | T | C | 0.037 | 0.005 | 1.55E-12 | 0.0045 | 0.0041 | 2.80E-01 |
| rs7903146 | 10 | 114758349 | T | C | -0.023 | 0.003 | 3.57E-19 | -0.0005 | 0.0024 | 8.40E-01 |
| rs845084 | 10 | 125220036 | A | G | 0.016 | 0.003 | 3.85E-08 | 0.0009 | 0.0025 | 7.20E-01 |
| rs7113874 | 11 | 8688016 | T | C | -0.014 | 0.002 | 3.30E-09 | -0.0004 | 0.0023 | 8.60E-01 |
| rs900144 | 11 | 13294268 | T | C | 0.015 | 0.003 | 5.54E-10 | 0.0056 | 0.0022 | 1.20E-02 |
| rs6265 | 11 | 27679916 | T | C | -0.048 | 0.003 | 6.25E-58 | -0.0114 | 0.0028 | 4.70E-05 |
| rs1765139 | 11 | 30353725 | T | C | 0.022 | 0.003 | 1.80E-13 | 0.0036 | 0.0026 | 1.60E-01 |
| rs2862996 | 11 | 43653833 | T | G | -0.020 | 0.003 | 2.49E-15 | 0.0001 | 0.0024 | 9.50E-01 |
| rs7124681 | 11 | 47529947 | A | C | 0.028 | 0.002 | 5.10E-31 | 0.0089 | 0.0022 | 7.20E-05 |
| rs7102454 | 11 | 65594820 | T | C | -0.018 | 0.003 | 1.29E-11 | -0.0019 | 0.0023 | 4.20E-01 |
| rs11228371 | 11 | 68594174 | A | C | 0.017 | 0.003 | 1.31E-08 | 0.0017 | 0.0023 | 4.70E-01 |
| rs349071 | 11 | 84776849 | A | G | -0.018 | 0.003 | 7.53E-10 | -0.0017 | 0.0022 | 4.50E-01 |
| rs647248 | 11 | 89917699 | A | G | 0.016 | 0.003 | 2.59E-10 | 0.0013 | 0.0022 | 5.60E-01 |
| rs12286929 | 11 | 115022404 | A | G | -0.018 | 0.002 | 2.34E-14 | 0.0021 | 0.0022 | 3.40E-01 |
| rs7933085 | 11 | 130796248 | A | G | -0.017 | 0.003 | 1.62E-09 | 0.0012 | 0.0022 | 5.80E-01 |
| rs10894670 | 11 | 133221987 | A | C | -0.014 | 0.002 | 1.65E-09 | -0.0008 | 0.0022 | 7.10E-01 |
| rs11611246 | 12 | 939480 | T | G | 0.025 | 0.003 | 7.99E-17 | 0.0024 | 0.0027 | 3.80E-01 |
| rs76895963 | 12 | 4384844 | T | G | -0.077 | 0.011 | 1.07E-11 | -0.0179 | 0.0085 | 3.50E-02 |
| rs10772983 | 12 | 17141582 | T | C | -0.014 | 0.002 | 2.04E-09 | 0.0023 | 0.0022 | 2.90E-01 |
| rs10842240 | 12 | 24060075 | C | G | 0.026 | 0.004 | 4.56E-12 | 0.0028 | 0.0035 | 4.20E-01 |
| rs11170468 | 12 | 39430048 | A | C | 0.016 | 0.003 | 7.84E-09 | -0.0051 | 0.0026 | 5.10E-02 |
| rs11181001 | 12 | 41948196 | A | G | 0.014 | 0.002 | 1.25E-09 | 0.0024 | 0.0022 | 2.70E-01 |
| rs7132908 | 12 | 50263148 | A | G | 0.035 | 0.003 | 1.35E-38 | -0.0009 | 0.0023 | 6.90E-01 |
| rs705704 | 12 | 56435412 | A | G | -0.016 | 0.003 | 1.52E-10 | 0.0072 | 0.0023 | 1.80E-03 |
| rs1689437 | 12 | 89758046 | A | G | -0.030 | 0.005 | 2.04E-09 | -0.0058 | 0.0046 | 2.00E-01 |
| rs7488867 | 12 | 103699685 | T | C | -0.022 | 0.003 | 5.69E-14 | 0.0017 | 0.0025 | 5.00E-01 |
| rs10850184 | 12 | 114064977 | T | G | 0.017 | 0.003 | 3.53E-10 | 0.0048 | 0.0024 | 4.30E-02 |
| rs11067700 | 12 | 116083562 | T | C | -0.017 | 0.003 | 8.28E-09 | -0.0025 | 0.0022 | 2.60E-01 |
| rs12369179 | 12 | 122963550 | T | C | -0.035 | 0.005 | 1.37E-14 | 0.0003 | 0.0038 | 9.40E-01 |
| rs10902480 | 12 | 132414769 | T | G | 0.022 | 0.004 | 5.60E-09 | 0.0020 | 0.0029 | 4.90E-01 |
| rs2012835 | 13 | 27987643 | T | G | -0.021 | 0.003 | 3.06E-10 | -0.0055 | 0.0025 | 2.70E-02 |
| rs2504236 | 13 | 28613298 | T | C | -0.015 | 0.003 | 1.94E-09 | -0.0044 | 0.0022 | 4.90E-02 |
| rs9595893 | 13 | 33175793 | T | G | -0.020 | 0.003 | 1.24E-14 | 0.0005 | 0.0023 | 8.10E-01 |
| rs9603697 | 13 | 40783323 | T | C | 0.020 | 0.003 | 1.34E-13 | -0.0009 | 0.0023 | 7.00E-01 |
| rs12429545 | 13 | 54102206 | A | G | 0.034 | 0.004 | 2.03E-21 | -0.0051 | 0.0033 | 1.20E-01 |
| rs7338127 | 13 | 59260511 | A | T | 0.020 | 0.003 | 1.39E-13 | 0.0007 | 0.0024 | 7.50E-01 |
| rs1441264 | 13 | 79580919 | A | G | 0.018 | 0.002 | 1.35E-13 | 0.0023 | 0.0023 | 3.20E-01 |
| rs912690 | 13 | 97020705 | C | G | -0.017 | 0.003 | 6.92E-12 | -0.0026 | 0.0022 | 2.50E-01 |
| rs9168 | 13 | 99101583 | A | C | -0.015 | 0.003 | 4.62E-08 | 0.0026 | 0.0024 | 2.80E-01 |
| rs9522279 | 13 | 112221296 | T | C | 0.022 | 0.003 | 1.65E-14 | -0.0071 | 0.0022 | 1.40E-03 |
| rs10132280 | 14 | 25928179 | A | C | -0.030 | 0.003 | 1.38E-31 | -0.0002 | 0.0024 | 9.40E-01 |
| rs1811859 | 14 | 29677727 | T | C | -0.020 | 0.004 | 6.48E-09 | -0.0008 | 0.0027 | 7.60E-01 |
| rs1955540 | 14 | 40801751 | T | C | -0.020 | 0.003 | 2.89E-10 | -0.0003 | 0.0028 | 9.10E-01 |
| rs4900714 | 14 | 47302219 | T | G | 0.020 | 0.003 | 1.17E-15 | -0.0021 | 0.0022 | 3.50E-01 |
| rs217672 | 14 | 62361021 | A | C | -0.020 | 0.003 | 4.40E-10 | -0.0011 | 0.0025 | 6.50E-01 |
| rs3902951 | 14 | 69789755 | T | G | -0.015 | 0.003 | 4.40E-08 | 0.0048 | 0.0026 | 6.60E-02 |
| rs17109256 | 14 | 79939993 | A | G | 0.031 | 0.003 | 2.81E-27 | -0.0074 | 0.0027 | 5.60E-03 |
| rs34918753 | 14 | 88312341 | A | G | 0.020 | 0.003 | 1.77E-09 | 0.0026 | 0.0026 | 3.20E-01 |
| rs11160119 | 14 | 93932258 | A | G | -0.022 | 0.003 | 2.85E-14 | 0.0014 | 0.0022 | 5.40E-01 |
| rs7161194 | 14 | 101529005 | A | G | 0.022 | 0.003 | 3.32E-15 | 0.0002 | 0.0024 | 9.30E-01 |
| rs7143963 | 14 | 103304425 | T | C | 0.021 | 0.003 | 3.35E-12 | 0.0010 | 0.0029 | 7.30E-01 |
| rs11636536 | 15 | 36391772 | T | C | 0.015 | 0.003 | 2.80E-09 | -0.0009 | 0.0022 | 6.80E-01 |
| rs10851458 | 15 | 47939956 | A | G | 0.015 | 0.003 | 5.95E-09 | 0.0006 | 0.0022 | 7.90E-01 |
| rs4776970 | 15 | 68080886 | A | T | 0.028 | 0.002 | 1.52E-30 | -0.0046 | 0.0023 | 4.40E-02 |
| rs58429335 | 15 | 70199927 | T | C | -0.031 | 0.006 | 4.85E-08 | -0.0005 | 0.0044 | 9.10E-01 |
| rs4777541 | 15 | 73082240 | T | C | 0.021 | 0.003 | 1.15E-12 | -0.0017 | 0.0026 | 5.10E-01 |
| rs8041222 | 15 | 79465549 | A | G | -0.019 | 0.003 | 3.28E-11 | 0.0011 | 0.0022 | 6.20E-01 |
| rs8036171 | 15 | 95266810 | A | C | 0.015 | 0.003 | 4.26E-10 | -0.0016 | 0.0023 | 5.00E-01 |
| rs12448257 | 16 | 3599655 | A | G | 0.019 | 0.003 | 1.17E-10 | 0.0051 | 0.0027 | 5.70E-02 |
| rs879620 | 16 | 4015729 | T | C | 0.021 | 0.003 | 3.11E-16 | -0.0031 | 0.0023 | 1.70E-01 |
| rs9926784 | 16 | 19941968 | T | C | 0.022 | 0.003 | 5.74E-14 | -0.0002 | 0.0028 | 9.40E-01 |
| rs3888190 | 16 | 28889486 | A | C | 0.032 | 0.002 | 6.96E-40 | -0.0054 | 0.0022 | 1.70E-02 |
| rs57149692 | 16 | 30142021 | C | G | -0.027 | 0.003 | 1.11E-19 | 0.0035 | 0.0023 | 1.20E-01 |
| rs1558902 | 16 | 53803574 | A | T | 0.082 | 0.002 | 1.19E-257 | -0.0045 | 0.0022 | 4.60E-02 |
| rs889398 | 16 | 69556715 | T | C | -0.021 | 0.002 | 1.54E-18 | -0.0016 | 0.0022 | 4.90E-01 |
| rs75950518 | 16 | 70378917 | T | C | -0.027 | 0.005 | 3.51E-09 | -0.0093 | 0.0034 | 7.10E-03 |
| rs28483459 | 16 | 71998398 | T | C | -0.018 | 0.003 | 2.92E-09 | 0.0000 | 0.0024 | 9.90E-01 |
| rs7206608 | 16 | 82872628 | C | G | -0.015 | 0.003 | 1.85E-08 | -0.0027 | 0.0024 | 2.60E-01 |
| rs3923783 | 17 | 1843189 | A | C | -0.023 | 0.003 | 7.47E-12 | 0.0028 | 0.0028 | 3.20E-01 |
| rs12450045 | 17 | 4742112 | A | G | 0.014 | 0.003 | 1.68E-08 | -0.0001 | 0.0022 | 9.60E-01 |
| rs4986044 | 17 | 21261560 | T | C | -0.018 | 0.002 | 1.45E-14 | -0.0025 | 0.0022 | 2.70E-01 |
| rs8065172 | 17 | 31456969 | A | G | -0.017 | 0.003 | 1.23E-08 | -0.0028 | 0.0026 | 2.90E-01 |
| rs12150665 | 17 | 34914787 | T | C | 0.018 | 0.002 | 3.43E-14 | 0.0006 | 0.0022 | 8.00E-01 |
| rs676387 | 17 | 40706273 | A | C | 0.016 | 0.003 | 4.10E-09 | -0.0003 | 0.0024 | 8.90E-01 |
| rs208015 | 17 | 46252346 | T | C | 0.037 | 0.005 | 8.72E-14 | -0.0069 | 0.0044 | 1.20E-01 |
| rs78369934 | 17 | 61739101 | T | C | 0.037 | 0.007 | 1.47E-08 | -0.0183 | 0.0049 | 1.80E-04 |
| rs12939549 | 17 | 78611724 | A | G | 0.020 | 0.002 | 1.29E-17 | -0.0035 | 0.0022 | 1.10E-01 |
| rs7226371 | 18 | 1850771 | A | G | -0.019 | 0.003 | 7.73E-09 | 0.0054 | 0.0031 | 8.20E-02 |
| rs891387 | 18 | 21103909 | T | C | 0.023 | 0.003 | 6.81E-20 | -0.0075 | 0.0022 | 6.80E-04 |
| rs11874191 | 18 | 36164084 | T | C | -0.017 | 0.003 | 3.06E-09 | 0.0022 | 0.0025 | 3.90E-01 |
| rs2612575 | 18 | 42967340 | A | G | -0.016 | 0.003 | 1.45E-08 | 0.0000 | 0.0025 | 9.90E-01 |
| rs9951619 | 18 | 56882326 | T | G | -0.019 | 0.003 | 1.75E-11 | -0.0007 | 0.0026 | 7.90E-01 |
| rs6567160 | 18 | 57829135 | T | C | -0.053 | 0.003 | 2.11E-82 | -0.0036 | 0.0026 | 1.60E-01 |
| rs17066856 | 18 | 58049656 | T | C | 0.030 | 0.004 | 2.65E-14 | 0.0056 | 0.0038 | 1.40E-01 |
| rs17710386 | 18 | 63461201 | T | C | -0.016 | 0.003 | 3.75E-11 | 0.0035 | 0.0024 | 1.40E-01 |
| rs1787267 | 18 | 76742544 | C | G | -0.033 | 0.005 | 2.49E-10 | 0.0031 | 0.0046 | 5.10E-01 |
| rs45486197 | 19 | 2244849 | A | G | 0.035 | 0.006 | 3.49E-09 | 0.0069 | 0.0045 | 1.20E-01 |
| rs7258309 | 19 | 4016005 | T | G | 0.023 | 0.003 | 6.54E-14 | 0.0067 | 0.0027 | 1.40E-02 |
| rs273496 | 19 | 18241857 | T | C | 0.020 | 0.003 | 2.50E-11 | 0.0021 | 0.0023 | 3.40E-01 |
| rs7258722 | 19 | 18808915 | A | T | -0.020 | 0.003 | 8.61E-15 | 0.0010 | 0.0023 | 6.60E-01 |
| rs17513613 | 19 | 30286822 | T | C | -0.018 | 0.003 | 1.50E-12 | -0.0029 | 0.0024 | 2.20E-01 |
| rs11084553 | 19 | 31019780 | A | G | 0.024 | 0.004 | 9.41E-12 | -0.0022 | 0.0030 | 4.60E-01 |
| rs769449 | 19 | 45410002 | A | G | -0.024 | 0.004 | 1.58E-09 | -0.0100 | 0.0033 | 2.50E-03 |
| rs1800437 | 19 | 46181392 | C | G | -0.037 | 0.003 | 1.73E-33 | -0.0039 | 0.0028 | 1.60E-01 |
| rs9304665 | 19 | 47602577 | A | T | 0.027 | 0.003 | 4.78E-21 | 0.0042 | 0.0026 | 1.10E-01 |
| rs7249149 | 19 | 51774538 | A | G | 0.015 | 0.003 | 4.41E-08 | 0.0014 | 0.0024 | 5.50E-01 |
| rs2145270 | 20 | 6621685 | T | C | 0.021 | 0.002 | 6.97E-18 | 0.0004 | 0.0023 | 8.60E-01 |
| rs8123881 | 20 | 15819495 | A | G | -0.019 | 0.003 | 2.53E-08 | 0.0077 | 0.0033 | 1.90E-02 |
| rs7272093 | 20 | 49810724 | A | G | -0.017 | 0.003 | 1.10E-09 | 0.0028 | 0.0024 | 2.40E-01 |
| rs35388084 | 20 | 50960490 | A | G | -0.028 | 0.004 | 1.12E-13 | -0.0066 | 0.0028 | 2.00E-02 |
| rs6092143 | 20 | 54181875 | T | C | 0.015 | 0.003 | 1.10E-08 | 0.0017 | 0.0023 | 4.70E-01 |
| rs13047416 | 21 | 40309436 | C | G | 0.016 | 0.003 | 9.84E-10 | -0.0004 | 0.0023 | 8.50E-01 |
| rs427943 | 21 | 46570896 | A | C | -0.018 | 0.003 | 2.19E-12 | 0.0015 | 0.0022 | 5.00E-01 |
| rs138383 | 22 | 38799861 | T | C | -0.016 | 0.003 | 1.39E-09 | -0.0056 | 0.0022 | 1.20E-02 |
| rs733381 | 22 | 40669648 | A | G | 0.021 | 0.003 | 5.20E-13 | -0.0049 | 0.0027 | 7.10E-02 |
| **4) MR of leisure computer use on BMI (men)** | | | | | | | | | | |
| SNP | CHR | POS | EA | OA | beta_bmi | se_bmi | pval_bmi | beta_pc | se_pc | pval_pc |
| rs2748985 | 1 | 1853184 | T | C | -0.013 | 0.003 | 3.29E-06 | -0.0153 | 0.0022 | 4.10E-12 |
| rs12145677 | 1 | 110023610 | G | A | -0.011 | 0.003 | 1.18E-05 | -0.0171 | 0.0024 | 1.20E-12 |
| rs7526456 | 1 | 197773734 | T | C | 0.003 | 0.003 | 2.67E-01 | -0.0127 | 0.0022 | 1.20E-08 |
| rs10754920 | 2 | 146456888 | C | G | 0.006 | 0.004 | 2.03E-01 | 0.0202 | 0.0035 | 6.70E-09 |
| rs6744254 | 2 | 155986195 | C | T | -0.007 | 0.003 | 4.61E-03 | -0.0159 | 0.0022 | 5.40E-13 |
| rs11274218 | 2 | 215035001 | C | T | 0.005 | 0.003 | 4.16E-02 | -0.0138 | 0.0023 | 2.50E-09 |
| rs4592851 | 2 | 215364232 | G | T | 0.010 | 0.003 | 3.74E-04 | -0.0144 | 0.0025 | 1.40E-08 |
| rs10208088 | 2 | 221055873 | C | T | 0.006 | 0.003 | 5.84E-02 | 0.0131 | 0.0022 | 4.40E-09 |
| rs11708955 | 3 | 49540114 | T | C | 0.012 | 0.003 | 3.85E-06 | -0.0157 | 0.0024 | 4.20E-11 |
| rs6774533 | 3 | 62471086 | C | T | -0.008 | 0.003 | 1.04E-02 | -0.0149 | 0.0025 | 1.20E-09 |
| rs78082503 | 3 | 116556913 | G | C | -0.002 | 0.004 | 5.92E-01 | -0.0154 | 0.0027 | 1.10E-08 |
| rs6857629 | 4 | 39684977 | G | A | -0.004 | 0.003 | 2.32E-01 | 0.0154 | 0.0026 | 1.90E-09 |
| rs2068625 | 4 | 159856739 | T | C | 0.004 | 0.003 | 1.61E-01 | -0.0158 | 0.0024 | 4.00E-11 |
| rs2220599 | 5 | 7378854 | C | G | 0.002 | 0.003 | 4.96E-01 | -0.0161 | 0.0023 | 2.40E-12 |
| rs11749912 | 5 | 88065628 | A | G | 0.011 | 0.003 | 1.69E-04 | 0.0139 | 0.0022 | 4.90E-10 |
| rs1469249 | 5 | 113837198 | G | A | -0.008 | 0.003 | 7.37E-03 | 0.0156 | 0.0027 | 9.50E-09 |
| rs162894 | 5 | 131611872 | T | G | 0.005 | 0.003 | 8.13E-02 | 0.0134 | 0.0023 | 1.10E-08 |
| rs56158102 | 5 | 144027803 | C | T | -0.003 | 0.003 | 2.02E-01 | 0.0132 | 0.0024 | 3.10E-08 |
| rs9477970 | 6 | 19082663 | T | A | -0.004 | 0.003 | 1.88E-01 | -0.0160 | 0.0028 | 1.00E-08 |
| rs2179517 | 6 | 26198845 | G | C | 0.009 | 0.003 | 1.33E-03 | -0.0124 | 0.0022 | 1.90E-08 |
| rs9372625 | 6 | 98344031 | G | A | 0.019 | 0.003 | 5.05E-14 | -0.0184 | 0.0023 | 5.70E-16 |
| rs17789218 | 6 | 100600097 | T | C | -0.015 | 0.003 | 3.33E-08 | -0.0151 | 0.0026 | 4.20E-09 |
| rs6935828 | 6 | 140811367 | C | T | 0.001 | 0.003 | 7.27E-01 | -0.0121 | 0.0022 | 4.30E-08 |
| rs55772938 | 7 | 70025113 | A | G | -0.008 | 0.003 | 7.68E-03 | -0.0151 | 0.0024 | 3.90E-10 |
| rs12706626 | 7 | 124531370 | G | A | -0.004 | 0.003 | 1.57E-01 | -0.0129 | 0.0023 | 1.40E-08 |
| rs3944151 | 7 | 126970005 | A | G | 0.002 | 0.003 | 6.06E-01 | 0.0137 | 0.0025 | 3.10E-08 |
| rs2345941 | 7 | 133337635 | A | G | -0.002 | 0.003 | 4.07E-01 | 0.0146 | 0.0022 | 4.00E-11 |
| rs13262595 | 8 | 143316970 | A | G | -0.004 | 0.003 | 1.15E-01 | -0.0157 | 0.0022 | 1.20E-12 |
| rs4977839 | 9 | 23355310 | G | A | -0.008 | 0.003 | 5.51E-03 | -0.0199 | 0.0022 | 4.70E-19 |
| rs113851275 | 9 | 98297220 | G | A | -0.009 | 0.005 | 6.28E-02 | -0.0209 | 0.0035 | 3.20E-09 |
| rs7020477 | 9 | 116827760 | A | G | -0.001 | 0.003 | 7.63E-01 | 0.0142 | 0.0025 | 1.20E-08 |
| rs73578186 | 9 | 126334485 | C | T | 0.002 | 0.003 | 4.41E-01 | 0.0150 | 0.0024 | 2.30E-10 |
| rs2734849 | 11 | 113270160 | A | G | 0.011 | 0.003 | 5.34E-06 | -0.0135 | 0.0022 | 8.20E-10 |
| rs1448355 | 11 | 131286685 | C | T | 0.001 | 0.003 | 7.52E-01 | -0.0146 | 0.0023 | 1.30E-10 |
| rs35933007 | 12 | 38929341 | G | A | 0.009 | 0.004 | 1.17E-02 | -0.0152 | 0.0026 | 9.20E-09 |
| rs1319734 | 12 | 40367517 | A | G | 0.001 | 0.003 | 6.02E-01 | 0.0127 | 0.0023 | 1.90E-08 |
| rs206965 | 12 | 120856332 | T | C | 0.004 | 0.003 | 1.57E-01 | 0.0156 | 0.0027 | 9.10E-09 |
| rs12874776 | 13 | 57794345 | C | A | -0.001 | 0.004 | 8.32E-01 | -0.0233 | 0.0036 | 8.00E-11 |
| rs198262 | 14 | 57283283 | C | T | -0.007 | 0.006 | 3.00E-01 | 0.0300 | 0.0055 | 3.90E-08 |
| rs166835 | 15 | 47716037 | C | T | 0.003 | 0.003 | 2.77E-01 | 0.0131 | 0.0022 | 3.40E-09 |
| rs4702 | 15 | 91426560 | G | A | 0.011 | 0.002 | 9.88E-06 | 0.0122 | 0.0022 | 3.80E-08 |
| rs6498759 | 16 | 18020295 | T | C | 0.000 | 0.003 | 9.24E-01 | 0.0133 | 0.0024 | 2.70E-08 |
| rs3730399 | 16 | 67229019 | A | G | -0.013 | 0.006 | 2.48E-02 | 0.0247 | 0.0045 | 3.30E-08 |
| rs4073003 | 17 | 19148305 | A | G | 0.001 | 0.003 | 7.58E-01 | 0.0202 | 0.0033 | 1.20E-09 |
| rs7209653 | 17 | 19882084 | T | C | 0.004 | 0.003 | 1.43E-01 | 0.0156 | 0.0024 | 9.10E-11 |
| rs12603813 | 17 | 43196584 | T | C | 0.002 | 0.003 | 5.39E-01 | 0.0144 | 0.0025 | 1.30E-08 |
| rs66643547 | 17 | 79375315 | C | T | 0.005 | 0.003 | 1.42E-01 | -0.0154 | 0.0023 | 3.20E-11 |
| rs784256 | 18 | 53398626 | G | A | -0.011 | 0.003 | 9.05E-04 | 0.0185 | 0.0028 | 6.20E-11 |
| rs6129084 | 20 | 59828857 | A | T | -0.004 | 0.003 | 2.09E-01 | -0.0141 | 0.0023 | 9.30E-10 |
| rs9608249 | 22 | 24613952 | G | A | 0.000 | 0.004 | 9.97E-01 | 0.0174 | 0.0031 | 2.20E-08 |
| rs136553 | 22 | 27255675 | C | T | -0.002 | 0.003 | 5.13E-01 | -0.0152 | 0.0023 | 2.30E-11 |
| rs7288455 | 22 | 39966547 | A | G | -0.004 | 0.003 | 1.28E-01 | 0.0131 | 0.0022 | 4.00E-09 |
| **5) MR of BMI (women) on leisure computer use** | | | | | | | | | | |
| SNP | CHR | POS | EA | OA | beta_bmi | se_bmi | pval_bmi | beta_pc | se_pc | pval_pc |
| rs79113395 | 1 | 1590521 | A | G | -0.021 | 0.003 | 1.45E-12 | 0.001 | 0.003 | 5.70E-01 |
| rs10909880 | 1 | 2727804 | T | C | -0.012 | 0.002 | 1.97E-08 | -0.006 | 0.002 | 7.30E-03 |
| rs78886584 | 1 | 16859325 | A | G | -0.015 | 0.003 | 1.30E-08 | 0.002 | 0.002 | 4.30E-01 |
| rs4295917 | 1 | 32173360 | A | G | 0.014 | 0.002 | 1.62E-08 | 0.000 | 0.002 | 9.10E-01 |
| rs112646560 | 1 | 39560250 | T | C | 0.020 | 0.003 | 1.14E-09 | 0.003 | 0.003 | 3.40E-01 |
| rs2984618 | 1 | 47690438 | T | G | 0.017 | 0.002 | 2.21E-14 | 0.004 | 0.002 | 9.30E-02 |
| rs657452 | 1 | 49589847 | A | G | 0.020 | 0.002 | 1.14E-19 | -0.005 | 0.002 | 4.00E-02 |
| rs12140153 | 1 | 62579891 | T | G | -0.033 | 0.005 | 2.87E-12 | 0.004 | 0.004 | 3.20E-01 |
| rs2568958 | 1 | 72765116 | A | G | 0.023 | 0.002 | 1.32E-24 | 0.007 | 0.002 | 1.20E-03 |
| rs6669189 | 1 | 75011358 | T | C | 0.021 | 0.002 | 4.69E-21 | -0.004 | 0.002 | 5.80E-02 |
| rs34517439 | 1 | 78450517 | A | C | 0.039 | 0.004 | 2.00E-21 | 0.003 | 0.003 | 3.90E-01 |
| rs12089815 | 1 | 91189933 | A | G | -0.015 | 0.003 | 6.02E-09 | 0.003 | 0.002 | 1.90E-01 |
| rs11165643 | 1 | 96924097 | T | C | 0.018 | 0.002 | 1.71E-16 | 0.003 | 0.002 | 1.30E-01 |
| rs4421623 | 1 | 98316285 | T | G | 0.018 | 0.003 | 1.13E-11 | -0.002 | 0.003 | 4.70E-01 |
| rs7550711 | 1 | 110082886 | T | C | 0.064 | 0.007 | 3.37E-21 | 0.023 | 0.007 | 9.90E-04 |
| rs197374 | 1 | 112289983 | T | C | 0.017 | 0.002 | 2.82E-13 | 0.004 | 0.002 | 8.80E-02 |
| rs61813324 | 1 | 156049877 | T | C | 0.030 | 0.004 | 3.60E-14 | 0.000 | 0.003 | 9.50E-01 |
| rs1750307 | 1 | 156488420 | A | T | 0.014 | 0.002 | 8.22E-09 | -0.002 | 0.002 | 3.00E-01 |
| rs12564992 | 1 | 174478100 | A | G | -0.021 | 0.004 | 2.37E-09 | -0.013 | 0.004 | 1.70E-04 |
| rs543874 | 1 | 177889480 | A | G | -0.058 | 0.003 | 8.70E-100 | 0.002 | 0.003 | 4.50E-01 |
| rs10920678 | 1 | 190239907 | A | G | 0.016 | 0.002 | 7.46E-14 | 0.001 | 0.002 | 7.70E-01 |
| rs4474229 | 1 | 195037001 | A | G | -0.017 | 0.003 | 1.64E-10 | -0.002 | 0.002 | 4.80E-01 |
| rs2820295 | 1 | 201800868 | A | G | 0.021 | 0.002 | 8.03E-19 | -0.006 | 0.002 | 1.00E-02 |
| rs17014375 | 1 | 209543560 | T | G | -0.019 | 0.003 | 1.46E-08 | 0.009 | 0.003 | 6.10E-03 |
| rs12042959 | 1 | 243533273 | A | G | 0.019 | 0.003 | 2.33E-09 | -0.008 | 0.003 | 1.70E-02 |
| rs62106258 | 2 | 417167 | T | C | 0.099 | 0.006 | 8.72E-58 | 0.009 | 0.005 | 7.80E-02 |
| rs6548237 | 2 | 621461 | A | C | -0.058 | 0.003 | 4.43E-89 | -0.008 | 0.003 | 3.50E-03 |
| rs7571496 | 2 | 6169351 | A | G | 0.015 | 0.003 | 1.45E-08 | -0.002 | 0.003 | 3.80E-01 |
| rs7601870 | 2 | 15384392 | A | G | -0.013 | 0.002 | 1.23E-08 | 0.003 | 0.002 | 2.10E-01 |
| rs10182181 | 2 | 25150296 | A | G | -0.036 | 0.002 | 5.00E-63 | -0.004 | 0.002 | 1.10E-01 |
| rs10048652 | 2 | 35449306 | T | C | 0.014 | 0.002 | 3.47E-09 | 0.000 | 0.002 | 9.30E-01 |
| rs786420 | 2 | 44719893 | T | C | 0.016 | 0.003 | 8.84E-09 | 0.007 | 0.003 | 4.70E-03 |
| rs35722922 | 2 | 47002226 | A | G | 0.019 | 0.003 | 3.65E-12 | 0.002 | 0.002 | 4.40E-01 |
| rs1421598 | 2 | 50264710 | A | C | -0.019 | 0.003 | 6.78E-10 | 0.001 | 0.003 | 7.80E-01 |
| rs7601895 | 2 | 55281901 | C | G | 0.016 | 0.003 | 9.61E-11 | 0.004 | 0.002 | 9.70E-02 |
| rs1861412 | 2 | 58893065 | A | G | -0.022 | 0.002 | 9.13E-21 | -0.005 | 0.002 | 1.90E-02 |
| rs6728037 | 2 | 59297346 | A | G | 0.021 | 0.002 | 6.49E-19 | -0.001 | 0.002 | 6.50E-01 |
| rs2861685 | 2 | 67837553 | T | C | 0.018 | 0.003 | 1.05E-11 | 0.003 | 0.002 | 1.40E-01 |
| rs12714199 | 2 | 86812549 | T | C | -0.015 | 0.002 | 5.12E-10 | -0.001 | 0.002 | 7.90E-01 |
| rs11677607 | 2 | 100751150 | T | C | -0.024 | 0.003 | 2.87E-19 | 0.006 | 0.003 | 3.10E-02 |
| rs1457457 | 2 | 104340148 | A | T | 0.014 | 0.002 | 3.79E-10 | -0.004 | 0.002 | 7.60E-02 |
| rs6739199 | 2 | 105452326 | T | C | 0.016 | 0.003 | 3.20E-10 | -0.007 | 0.002 | 4.30E-03 |
| rs1984559 | 2 | 135678888 | A | G | 0.016 | 0.003 | 3.27E-08 | 0.001 | 0.003 | 6.70E-01 |
| rs13002158 | 2 | 144021712 | A | G | -0.021 | 0.003 | 1.15E-12 | -0.006 | 0.003 | 3.60E-02 |
| rs76900466 | 2 | 145057760 | T | G | 0.023 | 0.004 | 4.53E-08 | -0.001 | 0.003 | 8.00E-01 |
| rs12692596 | 2 | 161265910 | T | C | 0.015 | 0.002 | 9.74E-11 | 0.001 | 0.002 | 5.50E-01 |
| rs12692738 | 2 | 165558252 | T | C | -0.016 | 0.003 | 2.82E-10 | -0.001 | 0.003 | 8.30E-01 |
| rs7588437 | 2 | 181575281 | A | G | -0.018 | 0.002 | 8.38E-15 | 0.002 | 0.002 | 3.50E-01 |
| rs7594237 | 2 | 198187164 | A | T | -0.014 | 0.003 | 1.81E-08 | 0.003 | 0.002 | 1.90E-01 |
| rs4482463 | 2 | 205375909 | A | C | -0.037 | 0.004 | 5.21E-18 | -0.005 | 0.004 | 2.60E-01 |
| rs11692326 | 2 | 208263279 | T | C | 0.016 | 0.003 | 2.04E-09 | -0.013 | 0.003 | 4.70E-07 |
| rs4673553 | 2 | 211608379 | T | G | -0.015 | 0.002 | 2.59E-11 | 0.000 | 0.002 | 9.00E-01 |
| rs12612009 | 2 | 228982603 | T | G | -0.014 | 0.002 | 3.78E-09 | 0.003 | 0.002 | 2.80E-01 |
| rs6720868 | 2 | 230663576 | T | C | 0.017 | 0.002 | 4.65E-12 | 0.000 | 0.002 | 9.40E-01 |
| rs1801282 | 3 | 12393125 | C | G | -0.018 | 0.003 | 4.72E-08 | 0.000 | 0.003 | 9.80E-01 |
| rs2470520 | 3 | 15762920 | T | C | -0.013 | 0.002 | 4.16E-08 | -0.002 | 0.002 | 3.50E-01 |
| rs4858223 | 3 | 20581149 | T | C | -0.016 | 0.003 | 8.26E-11 | 0.000 | 0.002 | 9.50E-01 |
| rs13062093 | 3 | 35667057 | T | G | -0.014 | 0.002 | 8.00E-09 | 0.000 | 0.002 | 8.40E-01 |
| rs1799923 | 3 | 42306294 | A | G | -0.024 | 0.004 | 5.10E-12 | -0.006 | 0.003 | 6.20E-02 |
| rs13076052 | 3 | 44456573 | C | G | -0.017 | 0.003 | 1.30E-08 | 0.005 | 0.002 | 5.00E-02 |
| rs6446187 | 3 | 49907111 | A | C | 0.028 | 0.003 | 1.22E-26 | -0.010 | 0.002 | 1.40E-05 |
| rs1916801 | 3 | 61187046 | A | T | 0.017 | 0.002 | 1.75E-14 | -0.001 | 0.002 | 7.70E-01 |
| rs1452075 | 3 | 62481063 | T | C | 0.016 | 0.003 | 1.47E-10 | 0.014 | 0.002 | 6.20E-09 |
| rs925018 | 3 | 62713143 | C | G | -0.014 | 0.002 | 9.81E-10 | 0.002 | 0.002 | 5.10E-01 |
| rs7647242 | 3 | 82642734 | T | C | 0.015 | 0.002 | 3.92E-10 | -0.003 | 0.002 | 1.80E-01 |
| rs13068138 | 3 | 85809425 | T | C | 0.023 | 0.003 | 3.60E-17 | 0.005 | 0.003 | 7.70E-02 |
| rs1949197 | 3 | 86273850 | A | G | 0.014 | 0.002 | 3.04E-09 | 0.003 | 0.002 | 1.40E-01 |
| rs1492014 | 3 | 94071481 | T | C | -0.015 | 0.002 | 2.84E-10 | 0.002 | 0.002 | 2.80E-01 |
| rs1436348 | 3 | 104612668 | A | G | -0.013 | 0.002 | 6.31E-09 | 0.004 | 0.002 | 6.90E-02 |
| rs1289736 | 3 | 107950892 | T | C | 0.014 | 0.002 | 1.43E-09 | -0.004 | 0.002 | 7.80E-02 |
| rs17619973 | 3 | 114417675 | A | G | 0.024 | 0.004 | 1.29E-08 | 0.000 | 0.004 | 9.20E-01 |
| rs7428670 | 3 | 131579810 | C | G | 0.020 | 0.003 | 3.09E-15 | -0.004 | 0.002 | 1.30E-01 |
| rs7621025 | 3 | 136272246 | T | C | -0.020 | 0.003 | 1.62E-15 | -0.008 | 0.003 | 1.10E-03 |
| rs16851483 | 3 | 141275436 | T | G | 0.036 | 0.005 | 2.28E-15 | 0.006 | 0.004 | 1.80E-01 |
| rs355754 | 3 | 154024165 | T | C | -0.014 | 0.002 | 4.26E-10 | 0.004 | 0.002 | 1.20E-01 |
| rs247975 | 3 | 173107443 | T | C | -0.018 | 0.002 | 3.62E-15 | 0.006 | 0.002 | 5.60E-03 |
| rs10513801 | 3 | 185822353 | T | G | 0.035 | 0.003 | 1.26E-27 | -0.004 | 0.003 | 2.50E-01 |
| rs4677812 | 3 | 194863794 | A | C | -0.014 | 0.003 | 9.38E-09 | 0.000 | 0.002 | 9.90E-01 |
| rs994596 | 4 | 18459828 | T | C | 0.015 | 0.003 | 1.02E-09 | 0.002 | 0.002 | 4.40E-01 |
| rs34811474 | 4 | 25408838 | A | G | -0.032 | 0.003 | 5.19E-24 | 0.003 | 0.003 | 2.80E-01 |
| rs73213484 | 4 | 28489339 | A | T | 0.025 | 0.004 | 2.28E-11 | 0.003 | 0.003 | 3.20E-01 |
| rs337637 | 4 | 38604470 | A | G | -0.013 | 0.002 | 3.04E-08 | 0.005 | 0.002 | 1.90E-02 |
| rs10938397 | 4 | 45182527 | A | G | -0.032 | 0.002 | 1.66E-45 | 0.006 | 0.002 | 8.30E-03 |
| rs2271046 | 4 | 52752812 | A | T | -0.014 | 0.003 | 2.38E-08 | 0.004 | 0.002 | 6.50E-02 |
| rs11727273 | 4 | 55509748 | T | C | -0.017 | 0.003 | 1.28E-10 | -0.002 | 0.002 | 3.00E-01 |
| rs11945861 | 4 | 65700865 | A | G | -0.015 | 0.003 | 3.13E-08 | 0.004 | 0.003 | 9.50E-02 |
| rs10002111 | 4 | 67815504 | A | G | 0.017 | 0.003 | 9.26E-10 | -0.001 | 0.003 | 7.50E-01 |
| rs4148155 | 4 | 89054667 | A | G | 0.020 | 0.004 | 6.20E-09 | -0.009 | 0.003 | 6.90E-03 |
| rs12645001 | 4 | 96018337 | A | G | -0.014 | 0.002 | 4.11E-09 | 0.002 | 0.002 | 3.30E-01 |
| rs13107325 | 4 | 103188709 | T | C | 0.045 | 0.004 | 9.33E-25 | 0.001 | 0.004 | 7.60E-01 |
| rs4834272 | 4 | 113313986 | T | C | -0.014 | 0.002 | 8.24E-10 | 0.000 | 0.002 | 8.80E-01 |
| rs1403846 | 4 | 119101723 | T | C | 0.016 | 0.003 | 1.62E-08 | 0.000 | 0.003 | 9.80E-01 |
| rs4864201 | 4 | 130731284 | T | C | 0.013 | 0.002 | 6.34E-09 | 0.000 | 0.002 | 9.30E-01 |
| rs57800857 | 4 | 140863365 | A | C | 0.019 | 0.003 | 1.07E-11 | 0.006 | 0.002 | 1.40E-02 |
| rs35107973 | 4 | 143125948 | T | C | 0.023 | 0.004 | 7.66E-09 | -0.004 | 0.003 | 2.70E-01 |
| rs750090 | 4 | 152931436 | T | C | 0.016 | 0.002 | 7.40E-11 | 0.004 | 0.002 | 7.00E-02 |
| rs13110266 | 4 | 162129844 | A | G | -0.014 | 0.002 | 1.55E-10 | -0.001 | 0.002 | 7.30E-01 |
| rs1491333 | 4 | 172186288 | T | G | -0.015 | 0.003 | 9.85E-09 | 0.002 | 0.003 | 4.60E-01 |
| rs6890310 | 5 | 27193573 | A | G | -0.015 | 0.003 | 1.38E-09 | 0.001 | 0.002 | 7.10E-01 |
| rs782971 | 5 | 43124688 | A | G | -0.017 | 0.003 | 4.89E-12 | -0.002 | 0.003 | 4.70E-01 |
| rs1812587 | 5 | 62991802 | T | G | 0.015 | 0.003 | 6.95E-09 | 0.002 | 0.002 | 4.60E-01 |
| rs10471636 | 5 | 63979856 | A | G | -0.017 | 0.002 | 4.66E-13 | -0.004 | 0.002 | 8.40E-02 |
| rs2112347 | 5 | 75015242 | T | G | 0.031 | 0.002 | 2.13E-42 | -0.005 | 0.002 | 4.70E-02 |
| rs6870983 | 5 | 87697533 | T | C | -0.021 | 0.003 | 9.61E-16 | 0.009 | 0.003 | 8.80E-04 |
| rs141729694 | 5 | 87999371 | T | C | 0.039 | 0.005 | 2.53E-14 | 0.012 | 0.004 | 3.70E-03 |
| rs159032 | 5 | 94206202 | T | C | 0.015 | 0.003 | 4.52E-08 | -0.001 | 0.003 | 7.60E-01 |
| rs2161097 | 5 | 103945178 | T | C | 0.013 | 0.002 | 2.31E-08 | 0.008 | 0.002 | 2.10E-04 |
| rs158186 | 5 | 107440856 | A | G | -0.029 | 0.003 | 3.23E-20 | -0.003 | 0.003 | 3.90E-01 |
| rs4895231 | 5 | 119388659 | C | G | 0.014 | 0.002 | 9.26E-10 | 0.000 | 0.002 | 9.30E-01 |
| rs13174863 | 5 | 139080745 | A | G | -0.027 | 0.003 | 4.53E-18 | -0.003 | 0.003 | 3.50E-01 |
| rs4569924 | 5 | 153540025 | T | C | 0.016 | 0.002 | 7.44E-13 | -0.004 | 0.002 | 1.00E-01 |
| rs6556810 | 5 | 164585949 | T | C | -0.012 | 0.002 | 4.98E-08 | -0.002 | 0.002 | 3.20E-01 |
| rs2053682 | 5 | 170599327 | A | C | 0.018 | 0.003 | 1.89E-13 | 0.004 | 0.002 | 6.30E-02 |
| rs2228213 | 6 | 12124855 | A | G | -0.015 | 0.002 | 9.21E-11 | -0.003 | 0.002 | 2.70E-01 |
| rs9367369 | 6 | 13189941 | A | G | 0.017 | 0.003 | 2.32E-09 | 0.003 | 0.002 | 2.00E-01 |
| rs3806114 | 6 | 20482335 | A | G | -0.014 | 0.002 | 4.49E-09 | 0.003 | 0.002 | 2.60E-01 |
| rs2178899 | 6 | 31606756 | A | T | 0.025 | 0.003 | 7.16E-14 | 0.001 | 0.003 | 7.20E-01 |
| rs11751591 | 6 | 33794215 | A | G | -0.019 | 0.003 | 1.16E-08 | -0.002 | 0.003 | 4.60E-01 |
| rs2814992 | 6 | 34617144 | A | G | -0.029 | 0.002 | 1.25E-35 | -0.005 | 0.002 | 3.90E-02 |
| rs1579557 | 6 | 40371918 | T | C | 0.021 | 0.003 | 3.09E-17 | -0.001 | 0.002 | 8.30E-01 |
| rs943005 | 6 | 50865820 | T | C | 0.040 | 0.003 | 1.99E-43 | 0.003 | 0.003 | 3.30E-01 |
| rs2465043 | 6 | 51180765 | A | G | -0.014 | 0.002 | 3.22E-09 | 0.001 | 0.002 | 6.50E-01 |
| rs62412826 | 6 | 61881167 | T | C | 0.033 | 0.006 | 7.98E-09 | -0.003 | 0.005 | 5.40E-01 |
| rs209416 | 6 | 83534729 | A | T | 0.017 | 0.002 | 8.71E-12 | -0.001 | 0.002 | 6.10E-01 |
| rs13209872 | 6 | 97753223 | C | G | -0.014 | 0.002 | 6.07E-09 | 0.002 | 0.002 | 4.00E-01 |
| rs12202969 | 6 | 98576223 | A | G | -0.015 | 0.002 | 1.93E-10 | 0.017 | 0.002 | 3.40E-14 |
| rs1417665 | 6 | 104816944 | T | C | 0.020 | 0.003 | 5.68E-12 | 0.004 | 0.003 | 1.10E-01 |
| rs768023 | 6 | 108876002 | A | G | 0.019 | 0.002 | 1.38E-16 | 0.001 | 0.002 | 6.60E-01 |
| rs2357760 | 6 | 120213880 | A | G | 0.016 | 0.002 | 1.09E-11 | 0.004 | 0.002 | 9.60E-02 |
| rs11754747 | 6 | 141494602 | T | C | 0.015 | 0.003 | 1.27E-08 | -0.004 | 0.003 | 1.40E-01 |
| rs198665 | 6 | 143154886 | A | C | 0.013 | 0.002 | 4.42E-09 | -0.002 | 0.002 | 4.30E-01 |
| rs2185027 | 6 | 153381622 | A | C | -0.015 | 0.002 | 1.11E-10 | -0.002 | 0.002 | 5.20E-01 |
| rs12207125 | 6 | 154301575 | A | G | 0.018 | 0.003 | 7.67E-09 | 0.006 | 0.003 | 3.50E-02 |
| rs13213408 | 6 | 162998049 | A | T | 0.020 | 0.003 | 4.33E-09 | -0.004 | 0.003 | 1.80E-01 |
| rs6973700 | 7 | 1856669 | A | G | 0.019 | 0.003 | 1.77E-09 | 0.004 | 0.003 | 2.00E-01 |
| rs836525 | 7 | 6465909 | T | C | 0.018 | 0.003 | 9.75E-09 | 0.004 | 0.003 | 2.20E-01 |
| rs4307239 | 7 | 24354300 | A | G | -0.014 | 0.002 | 1.31E-09 | 0.003 | 0.002 | 1.40E-01 |
| rs11971098 | 7 | 26699061 | A | G | -0.024 | 0.004 | 6.49E-09 | -0.009 | 0.004 | 2.10E-02 |
| rs10499694 | 7 | 50614173 | A | G | 0.014 | 0.002 | 3.99E-10 | 0.001 | 0.002 | 5.50E-01 |
| rs10237317 | 7 | 70045941 | A | G | -0.014 | 0.002 | 6.82E-10 | -0.008 | 0.002 | 2.30E-04 |
| rs73124396 | 7 | 71579606 | T | C | 0.019 | 0.003 | 1.05E-08 | 0.003 | 0.003 | 3.50E-01 |
| rs2267812 | 7 | 74138121 | A | C | -0.017 | 0.003 | 1.74E-09 | -0.001 | 0.003 | 6.90E-01 |
| rs1167800 | 7 | 75176196 | A | G | 0.020 | 0.002 | 3.66E-19 | -0.001 | 0.002 | 5.70E-01 |
| rs7805441 | 7 | 78121458 | T | C | 0.015 | 0.002 | 2.01E-10 | 0.000 | 0.002 | 9.50E-01 |
| rs2528531 | 7 | 93236510 | A | C | -0.015 | 0.002 | 1.35E-09 | 0.000 | 0.002 | 8.90E-01 |
| rs10271582 | 7 | 99121950 | A | G | 0.019 | 0.003 | 8.41E-09 | 0.012 | 0.003 | 7.40E-05 |
| rs2299383 | 7 | 103418846 | T | C | 0.018 | 0.002 | 2.73E-16 | 0.007 | 0.002 | 3.00E-03 |
| rs7788008 | 7 | 112972483 | A | G | -0.017 | 0.002 | 1.09E-13 | 0.004 | 0.002 | 5.20E-02 |
| rs2045293 | 7 | 114350102 | T | C | 0.019 | 0.002 | 4.06E-16 | -0.005 | 0.002 | 2.30E-02 |
| rs2289705 | 7 | 121983843 | A | G | -0.015 | 0.003 | 1.47E-08 | -0.002 | 0.003 | 5.50E-01 |
| rs2968864 | 7 | 150622162 | T | C | 0.016 | 0.003 | 9.35E-10 | 0.003 | 0.003 | 2.50E-01 |
| rs4240673 | 8 | 10787612 | T | C | 0.018 | 0.002 | 1.03E-15 | 0.002 | 0.002 | 4.40E-01 |
| rs2959592 | 8 | 17002725 | T | C | 0.017 | 0.003 | 6.26E-10 | -0.001 | 0.003 | 7.00E-01 |
| rs1362910 | 8 | 30856464 | A | G | 0.013 | 0.002 | 3.03E-09 | -0.001 | 0.002 | 7.10E-01 |
| rs4320543 | 8 | 34422967 | A | G | -0.013 | 0.002 | 2.95E-08 | 0.004 | 0.002 | 1.20E-01 |
| rs12681792 | 8 | 62054463 | A | C | 0.018 | 0.003 | 2.61E-10 | 0.005 | 0.003 | 5.80E-02 |
| rs7827848 | 8 | 64790774 | A | T | 0.015 | 0.002 | 1.88E-11 | 0.001 | 0.002 | 5.40E-01 |
| rs1808629 | 8 | 73435964 | A | G | -0.023 | 0.003 | 5.76E-16 | -0.002 | 0.002 | 3.30E-01 |
| rs17405819 | 8 | 76806584 | T | C | 0.022 | 0.002 | 7.81E-20 | 0.002 | 0.002 | 4.60E-01 |
| rs12680842 | 8 | 95582606 | A | G | 0.013 | 0.002 | 3.36E-08 | 0.000 | 0.002 | 8.60E-01 |
| rs2721965 | 8 | 116662038 | A | C | 0.019 | 0.002 | 2.10E-14 | 0.002 | 0.002 | 3.50E-01 |
| rs10099330 | 8 | 143383694 | A | G | -0.013 | 0.002 | 9.94E-09 | -0.003 | 0.002 | 2.10E-01 |
| rs10124645 | 9 | 11321119 | A | G | 0.014 | 0.002 | 4.98E-09 | 0.002 | 0.002 | 4.10E-01 |
| rs10756714 | 9 | 15885041 | A | G | 0.021 | 0.002 | 1.46E-19 | -0.002 | 0.002 | 3.70E-01 |
| rs10962550 | 9 | 16720329 | C | G | 0.019 | 0.003 | 1.70E-10 | -0.002 | 0.003 | 4.00E-01 |
| rs16912921 | 9 | 28413461 | A | C | 0.025 | 0.002 | 1.56E-26 | 0.005 | 0.002 | 4.30E-02 |
| rs973345 | 9 | 29713169 | T | C | 0.014 | 0.002 | 1.09E-09 | 0.003 | 0.002 | 2.00E-01 |
| rs563132 | 9 | 37245476 | A | T | 0.018 | 0.002 | 1.96E-14 | 0.000 | 0.002 | 9.80E-01 |
| rs10116186 | 9 | 80532374 | A | G | -0.015 | 0.003 | 4.10E-08 | 0.000 | 0.002 | 9.90E-01 |
| rs10797115 | 9 | 92191256 | T | C | 0.016 | 0.002 | 4.07E-12 | -0.006 | 0.002 | 7.40E-03 |
| rs10992867 | 9 | 96461013 | A | G | 0.019 | 0.003 | 8.89E-13 | -0.001 | 0.003 | 7.00E-01 |
| rs10118701 | 9 | 103061366 | A | G | -0.018 | 0.002 | 2.20E-13 | 0.002 | 0.002 | 3.00E-01 |
| rs7024334 | 9 | 109072075 | T | G | 0.016 | 0.003 | 1.63E-09 | 0.001 | 0.003 | 7.90E-01 |
| rs1928295 | 9 | 120378483 | T | C | 0.016 | 0.002 | 2.85E-13 | 0.004 | 0.002 | 8.00E-02 |
| rs3829849 | 9 | 129390800 | T | C | 0.014 | 0.002 | 1.93E-09 | 0.001 | 0.002 | 6.60E-01 |
| rs10733682 | 9 | 129460914 | A | G | 0.016 | 0.002 | 1.85E-12 | -0.002 | 0.002 | 4.50E-01 |
| rs2267958 | 9 | 131015279 | A | G | -0.016 | 0.002 | 1.88E-11 | -0.003 | 0.002 | 2.50E-01 |
| rs1009473 | 9 | 138777312 | T | C | -0.013 | 0.002 | 3.36E-08 | -0.001 | 0.002 | 6.40E-01 |
| rs7084454 | 10 | 21821274 | A | G | 0.023 | 0.003 | 7.43E-20 | 0.011 | 0.002 | 6.40E-06 |
| rs34930419 | 10 | 33987329 | T | C | -0.046 | 0.008 | 1.37E-09 | -0.008 | 0.006 | 1.90E-01 |
| rs10823893 | 10 | 53677313 | A | G | 0.018 | 0.003 | 5.22E-11 | -0.004 | 0.002 | 5.10E-02 |
| rs10761785 | 10 | 65318766 | T | G | -0.017 | 0.002 | 4.17E-15 | 0.001 | 0.002 | 6.30E-01 |
| rs10824347 | 10 | 77645229 | A | T | 0.018 | 0.003 | 5.47E-11 | -0.003 | 0.003 | 3.10E-01 |
| rs7899106 | 10 | 87410904 | A | G | -0.038 | 0.005 | 7.65E-14 | -0.003 | 0.005 | 5.20E-01 |
| rs563296 | 10 | 99772404 | A | G | 0.018 | 0.002 | 8.85E-14 | -0.001 | 0.002 | 7.60E-01 |
| rs3977755 | 10 | 104420210 | T | C | -0.014 | 0.003 | 8.50E-09 | 0.010 | 0.002 | 3.10E-05 |
| rs6585198 | 10 | 114762237 | A | G | -0.015 | 0.002 | 1.22E-12 | -0.004 | 0.002 | 8.60E-02 |
| rs1681743 | 10 | 118565501 | T | C | -0.013 | 0.002 | 3.70E-08 | -0.004 | 0.002 | 1.20E-01 |
| rs1561589 | 10 | 126695673 | A | G | 0.016 | 0.002 | 4.24E-12 | 0.003 | 0.002 | 2.50E-01 |
| rs4880341 | 10 | 133992689 | T | C | -0.014 | 0.002 | 4.53E-10 | -0.003 | 0.002 | 2.40E-01 |
| rs7395632 | 11 | 958789 | T | C | -0.013 | 0.002 | 3.87E-08 | -0.003 | 0.002 | 1.80E-01 |
| rs4929923 | 11 | 8639200 | T | C | -0.022 | 0.002 | 2.51E-22 | 0.000 | 0.002 | 9.70E-01 |
| rs11022762 | 11 | 13335926 | T | C | -0.015 | 0.002 | 5.56E-11 | -0.008 | 0.002 | 2.20E-04 |
| rs6265 | 11 | 27679916 | T | C | -0.037 | 0.003 | 7.95E-40 | -0.011 | 0.003 | 4.70E-05 |
| rs2862961 | 11 | 43656535 | A | G | -0.024 | 0.003 | 5.30E-22 | 0.000 | 0.002 | 1.00E+00 |
| rs10742752 | 11 | 45438374 | T | C | -0.013 | 0.002 | 3.98E-09 | -0.003 | 0.002 | 1.30E-01 |
| rs7124681 | 11 | 47529947 | A | C | 0.025 | 0.002 | 9.27E-29 | 0.009 | 0.002 | 7.20E-05 |
| rs893006 | 11 | 64365796 | A | C | 0.017 | 0.002 | 4.05E-13 | -0.001 | 0.002 | 7.00E-01 |
| rs625652 | 11 | 65641931 | A | T | -0.020 | 0.003 | 9.05E-13 | -0.002 | 0.002 | 4.00E-01 |
| rs7107409 | 11 | 76492195 | T | C | -0.018 | 0.003 | 2.05E-08 | 0.000 | 0.003 | 9.10E-01 |
| rs2605603 | 11 | 93221105 | A | G | -0.013 | 0.002 | 1.40E-09 | 0.001 | 0.002 | 7.70E-01 |
| rs7105462 | 11 | 112912048 | A | G | 0.014 | 0.002 | 4.40E-10 | 0.007 | 0.002 | 2.90E-03 |
| rs1048932 | 11 | 115044850 | A | C | -0.018 | 0.002 | 2.52E-16 | 0.004 | 0.002 | 4.70E-02 |
| rs1003081 | 11 | 118913993 | T | C | 0.014 | 0.002 | 6.27E-11 | -0.004 | 0.002 | 6.20E-02 |
| rs4459316 | 11 | 130756176 | T | C | -0.015 | 0.002 | 2.33E-11 | 0.002 | 0.002 | 3.10E-01 |
| rs4936175 | 11 | 132641959 | T | C | -0.016 | 0.002 | 6.69E-12 | -0.006 | 0.002 | 3.60E-03 |
| rs329651 | 11 | 133767622 | T | G | 0.018 | 0.003 | 1.96E-10 | -0.009 | 0.003 | 1.90E-03 |
| rs12364470 | 11 | 134601012 | T | G | -0.020 | 0.003 | 2.54E-11 | -0.001 | 0.003 | 7.50E-01 |
| rs11611246 | 12 | 939480 | T | G | 0.021 | 0.003 | 1.27E-14 | 0.002 | 0.003 | 3.80E-01 |
| rs7976757 | 12 | 19207948 | T | C | -0.019 | 0.003 | 2.94E-10 | -0.002 | 0.003 | 5.30E-01 |
| rs16926318 | 12 | 23678745 | A | G | -0.023 | 0.004 | 4.23E-08 | 0.001 | 0.004 | 7.60E-01 |
| rs16926778 | 12 | 24002125 | C | G | 0.026 | 0.004 | 1.41E-09 | 0.006 | 0.004 | 1.30E-01 |
| rs7138803 | 12 | 50247468 | A | G | 0.029 | 0.002 | 9.70E-38 | 0.000 | 0.002 | 9.80E-01 |
| rs2271189 | 12 | 56494991 | A | G | -0.015 | 0.002 | 6.35E-10 | 0.005 | 0.002 | 1.60E-02 |
| rs11173522 | 12 | 60953472 | A | C | 0.017 | 0.003 | 9.01E-10 | -0.002 | 0.003 | 5.40E-01 |
| rs645026 | 12 | 69757931 | A | G | -0.016 | 0.003 | 4.79E-10 | 0.001 | 0.002 | 7.30E-01 |
| rs10746306 | 12 | 84477374 | T | G | 0.018 | 0.003 | 8.74E-09 | 0.002 | 0.003 | 4.00E-01 |
| rs2279574 | 12 | 89745477 | A | C | -0.017 | 0.002 | 1.96E-12 | 0.001 | 0.002 | 6.90E-01 |
| rs2712643 | 12 | 99638276 | A | C | -0.015 | 0.003 | 1.47E-09 | 0.004 | 0.003 | 1.00E-01 |
| rs17033633 | 12 | 103656343 | T | C | -0.021 | 0.003 | 7.26E-12 | 0.001 | 0.003 | 6.50E-01 |
| rs6606686 | 12 | 110903380 | C | G | -0.018 | 0.002 | 2.50E-14 | 0.001 | 0.002 | 6.40E-01 |
| rs17508240 | 12 | 117577358 | A | G | 0.019 | 0.003 | 2.83E-09 | 0.002 | 0.003 | 3.60E-01 |
| rs6489156 | 12 | 123100397 | T | C | 0.022 | 0.003 | 9.97E-18 | -0.001 | 0.002 | 6.40E-01 |
| rs7133378 | 12 | 124409502 | A | G | 0.016 | 0.002 | 1.85E-11 | -0.001 | 0.002 | 5.80E-01 |
| rs9579775 | 13 | 20616557 | A | C | -0.025 | 0.004 | 3.66E-10 | -0.001 | 0.003 | 8.80E-01 |
| rs12872889 | 13 | 28674628 | T | C | -0.021 | 0.003 | 1.52E-11 | -0.009 | 0.003 | 9.80E-04 |
| rs9595630 | 13 | 33065443 | T | G | 0.015 | 0.002 | 2.04E-10 | 0.000 | 0.002 | 1.00E+00 |
| rs12429545 | 13 | 54102206 | A | G | 0.029 | 0.003 | 1.09E-18 | -0.005 | 0.003 | 1.20E-01 |
| rs6561937 | 13 | 58257667 | A | T | -0.020 | 0.003 | 5.95E-13 | 0.006 | 0.003 | 2.30E-02 |
| rs4055791 | 13 | 59266053 | T | C | -0.017 | 0.003 | 1.77E-10 | -0.003 | 0.002 | 2.30E-01 |
| rs9571687 | 13 | 67472713 | A | C | -0.015 | 0.002 | 3.24E-09 | -0.007 | 0.002 | 3.50E-03 |
| rs1441264 | 13 | 79580919 | A | G | 0.017 | 0.002 | 5.19E-14 | 0.002 | 0.002 | 3.20E-01 |
| rs9531786 | 13 | 85983968 | C | G | -0.014 | 0.002 | 8.39E-09 | -0.006 | 0.002 | 6.70E-03 |
| rs77432547 | 13 | 86494817 | A | G | -0.018 | 0.003 | 2.28E-09 | 0.000 | 0.002 | 9.90E-01 |
| rs1927790 | 13 | 96922191 | T | C | -0.014 | 0.002 | 1.27E-09 | -0.004 | 0.002 | 4.70E-02 |
| rs4586287 | 13 | 99245334 | C | G | 0.014 | 0.003 | 2.95E-08 | -0.005 | 0.002 | 3.00E-02 |
| rs7335249 | 13 | 109962579 | T | C | 0.014 | 0.002 | 4.91E-09 | 0.003 | 0.002 | 1.70E-01 |
| rs2479958 | 13 | 111984244 | A | G | 0.014 | 0.002 | 1.38E-08 | -0.003 | 0.002 | 1.60E-01 |
| rs2754079 | 14 | 25886140 | A | G | -0.016 | 0.002 | 2.31E-11 | -0.002 | 0.002 | 2.90E-01 |
| rs1569979 | 14 | 29681294 | A | G | 0.023 | 0.003 | 1.57E-17 | 0.000 | 0.003 | 9.10E-01 |
| rs17522122 | 14 | 33302882 | T | G | 0.017 | 0.002 | 6.46E-14 | -0.001 | 0.002 | 5.10E-01 |
| rs35020117 | 14 | 73341655 | T | G | 0.017 | 0.003 | 1.23E-09 | 0.000 | 0.002 | 9.90E-01 |
| rs7141420 | 14 | 79899454 | T | C | 0.021 | 0.002 | 7.84E-22 | -0.006 | 0.002 | 1.00E-02 |
| rs1285997 | 14 | 91513029 | C | G | -0.015 | 0.003 | 3.18E-09 | -0.002 | 0.002 | 3.20E-01 |
| rs942066 | 14 | 94031914 | A | G | -0.020 | 0.003 | 1.04E-13 | 0.003 | 0.002 | 2.10E-01 |
| rs3803286 | 14 | 103246470 | A | G | 0.017 | 0.002 | 8.29E-13 | -0.004 | 0.002 | 7.50E-02 |
| rs709400 | 14 | 104149475 | A | G | 0.018 | 0.002 | 4.41E-16 | -0.001 | 0.002 | 5.20E-01 |
| rs12101625 | 15 | 26972431 | C | G | 0.014 | 0.002 | 5.54E-09 | 0.004 | 0.002 | 5.60E-02 |
| rs8038522 | 15 | 46585105 | T | G | -0.014 | 0.002 | 1.27E-09 | -0.001 | 0.002 | 5.50E-01 |
| rs6493498 | 15 | 51754451 | T | C | 0.016 | 0.002 | 1.53E-13 | 0.004 | 0.002 | 8.70E-02 |
| rs8024806 | 15 | 53473990 | T | C | 0.027 | 0.005 | 9.76E-09 | -0.006 | 0.005 | 2.00E-01 |
| rs8033510 | 15 | 61445514 | T | C | 0.014 | 0.002 | 1.13E-08 | -0.003 | 0.002 | 2.00E-01 |
| rs4776982 | 15 | 68114974 | A | G | 0.031 | 0.003 | 4.25E-33 | -0.006 | 0.003 | 3.00E-02 |
| rs12914489 | 15 | 74187937 | A | G | 0.023 | 0.004 | 1.64E-10 | 0.000 | 0.004 | 9.10E-01 |
| rs62007782 | 15 | 78029797 | A | G | -0.018 | 0.003 | 1.94E-09 | 0.001 | 0.002 | 6.20E-01 |
| rs12593036 | 15 | 81058652 | A | G | 0.015 | 0.003 | 6.65E-10 | 0.006 | 0.002 | 1.90E-02 |
| rs7498044 | 15 | 92573639 | A | G | -0.018 | 0.003 | 2.73E-08 | 0.002 | 0.003 | 5.70E-01 |
| rs11073383 | 15 | 95274349 | A | G | -0.016 | 0.002 | 3.26E-12 | 0.003 | 0.002 | 1.60E-01 |
| rs57221746 | 15 | 99230035 | A | G | -0.018 | 0.003 | 2.08E-08 | 0.001 | 0.003 | 8.40E-01 |
| rs214249 | 16 | 348687 | T | G | 0.017 | 0.002 | 1.37E-12 | -0.003 | 0.002 | 1.30E-01 |
| rs12448257 | 16 | 3599655 | A | G | 0.015 | 0.003 | 3.13E-08 | 0.005 | 0.003 | 5.70E-02 |
| rs879620 | 16 | 4015729 | T | C | 0.025 | 0.002 | 5.45E-26 | -0.003 | 0.002 | 1.70E-01 |
| rs9926784 | 16 | 19941968 | T | C | 0.025 | 0.003 | 6.01E-20 | 0.000 | 0.003 | 9.40E-01 |
| rs17767765 | 16 | 24539793 | T | C | -0.013 | 0.002 | 2.60E-08 | 0.000 | 0.002 | 9.30E-01 |
| rs7498665 | 16 | 28883241 | A | G | -0.026 | 0.002 | 1.94E-32 | 0.005 | 0.002 | 1.90E-02 |
| rs2289292 | 16 | 30097630 | T | C | -0.019 | 0.003 | 1.13E-14 | 0.001 | 0.002 | 5.90E-01 |
| rs4889606 | 16 | 31011183 | A | G | 0.027 | 0.002 | 1.25E-32 | 0.001 | 0.002 | 6.70E-01 |
| rs1558902 | 16 | 53803574 | A | T | 0.070 | 0.002 | 1.10E-215 | -0.004 | 0.002 | 4.60E-02 |
| rs4783718 | 16 | 69547741 | T | C | 0.020 | 0.002 | 1.77E-19 | 0.001 | 0.002 | 6.40E-01 |
| rs811054 | 16 | 72251132 | T | C | 0.016 | 0.002 | 3.18E-13 | 0.002 | 0.002 | 3.60E-01 |
| rs2012502 | 16 | 81728081 | A | C | 0.015 | 0.002 | 2.97E-11 | -0.004 | 0.002 | 9.40E-02 |
| rs4790292 | 17 | 1824305 | A | C | -0.026 | 0.003 | 1.65E-15 | 0.004 | 0.003 | 1.60E-01 |
| rs3826408 | 17 | 7101292 | T | C | 0.012 | 0.002 | 4.48E-08 | -0.003 | 0.002 | 1.60E-01 |
| rs1075901 | 17 | 15943910 | T | C | -0.014 | 0.002 | 4.36E-11 | -0.001 | 0.002 | 5.80E-01 |
| rs4986044 | 17 | 21261560 | T | C | -0.017 | 0.002 | 2.20E-15 | -0.002 | 0.002 | 2.70E-01 |
| rs12150665 | 17 | 34914787 | T | C | 0.017 | 0.002 | 5.92E-14 | 0.001 | 0.002 | 8.00E-01 |
| rs56161855 | 17 | 46288649 | A | T | -0.026 | 0.004 | 1.48E-11 | 0.001 | 0.003 | 7.90E-01 |
| rs9906944 | 17 | 47091420 | T | C | -0.019 | 0.002 | 7.25E-15 | 0.003 | 0.002 | 2.00E-01 |
| rs7211966 | 17 | 54374611 | T | C | -0.047 | 0.008 | 4.89E-09 | 0.003 | 0.008 | 7.10E-01 |
| rs8075273 | 17 | 61728881 | A | C | -0.016 | 0.002 | 5.56E-11 | 0.003 | 0.002 | 2.30E-01 |
| rs2619976 | 17 | 71754545 | T | C | 0.015 | 0.002 | 6.17E-10 | 0.001 | 0.002 | 6.00E-01 |
| rs12949279 | 17 | 78558411 | T | C | 0.018 | 0.002 | 6.09E-16 | -0.003 | 0.002 | 1.20E-01 |
| rs4969387 | 17 | 79081724 | C | G | -0.015 | 0.003 | 6.90E-09 | -0.001 | 0.002 | 7.60E-01 |
| rs9955276 | 18 | 1839339 | T | C | 0.022 | 0.003 | 8.02E-12 | -0.008 | 0.003 | 1.10E-02 |
| rs1808579 | 18 | 21104888 | T | C | -0.020 | 0.002 | 9.44E-20 | 0.007 | 0.002 | 3.10E-03 |
| rs16975921 | 18 | 39914870 | A | T | -0.016 | 0.003 | 3.87E-11 | 0.006 | 0.002 | 7.80E-03 |
| rs1518159 | 18 | 40760146 | T | C | -0.019 | 0.003 | 6.66E-13 | -0.003 | 0.003 | 2.70E-01 |
| rs7239114 | 18 | 45921214 | A | G | 0.016 | 0.002 | 3.90E-12 | -0.002 | 0.002 | 3.10E-01 |
| rs6567160 | 18 | 57829135 | T | C | -0.057 | 0.003 | 8.74E-108 | -0.004 | 0.003 | 1.60E-01 |
| rs17066856 | 18 | 58049656 | T | C | 0.040 | 0.004 | 1.39E-27 | 0.006 | 0.004 | 1.40E-01 |
| rs12981256 | 19 | 1865901 | A | G | 0.018 | 0.002 | 3.93E-15 | -0.005 | 0.002 | 4.00E-02 |
| rs895330 | 19 | 4060707 | C | G | 0.018 | 0.003 | 6.65E-10 | 0.005 | 0.003 | 5.10E-02 |
| rs273504 | 19 | 18215247 | A | G | -0.015 | 0.002 | 1.27E-10 | -0.003 | 0.002 | 2.50E-01 |
| rs17724992 | 19 | 18454825 | A | G | 0.017 | 0.003 | 6.65E-12 | -0.006 | 0.002 | 1.20E-02 |
| rs11880870 | 19 | 18830704 | A | G | 0.020 | 0.002 | 3.31E-18 | 0.001 | 0.002 | 7.20E-01 |
| rs17751061 | 19 | 19413092 | T | C | -0.018 | 0.003 | 1.29E-08 | -0.001 | 0.003 | 7.20E-01 |
| rs12462975 | 19 | 30272202 | A | G | 0.020 | 0.003 | 2.63E-16 | 0.003 | 0.002 | 2.00E-01 |
| rs11667280 | 19 | 30705747 | C | G | 0.016 | 0.003 | 3.14E-08 | 0.004 | 0.003 | 1.40E-01 |
| rs7250833 | 19 | 33937277 | T | C | 0.015 | 0.002 | 7.22E-10 | 0.009 | 0.002 | 3.00E-04 |
| rs185350 | 19 | 34306816 | T | C | 0.013 | 0.002 | 7.14E-09 | -0.001 | 0.002 | 6.00E-01 |
| rs429358 | 19 | 45411941 | T | C | 0.028 | 0.004 | 3.17E-14 | 0.011 | 0.003 | 2.60E-04 |
| rs11672660 | 19 | 46180184 | T | C | -0.032 | 0.003 | 5.93E-31 | -0.004 | 0.003 | 1.80E-01 |
| rs9304665 | 19 | 47602577 | A | T | 0.020 | 0.003 | 1.84E-14 | 0.004 | 0.003 | 1.10E-01 |
| rs1884389 | 20 | 1410582 | T | C | -0.014 | 0.002 | 1.20E-09 | -0.002 | 0.002 | 3.30E-01 |
| rs1884897 | 20 | 6612832 | A | G | -0.017 | 0.002 | 9.06E-14 | -0.001 | 0.002 | 6.40E-01 |
| rs1409818 | 20 | 21381121 | T | C | 0.025 | 0.004 | 1.58E-11 | 0.003 | 0.004 | 4.90E-01 |
| rs6050446 | 20 | 25195509 | A | G | -0.037 | 0.006 | 6.20E-09 | -0.014 | 0.006 | 2.30E-02 |
| rs6142096 | 20 | 32686658 | A | G | 0.013 | 0.002 | 4.17E-09 | 0.001 | 0.002 | 7.40E-01 |
| rs16989232 | 20 | 39291784 | A | G | 0.013 | 0.002 | 1.52E-08 | -0.001 | 0.002 | 7.30E-01 |
| rs17806224 | 20 | 51065854 | A | G | -0.029 | 0.003 | 3.52E-22 | -0.007 | 0.003 | 1.00E-02 |
| rs6010784 | 20 | 61540319 | T | C | 0.013 | 0.002 | 1.08E-08 | 0.010 | 0.002 | 5.40E-06 |
| rs2836754 | 21 | 40291740 | T | C | -0.015 | 0.002 | 3.73E-11 | 0.001 | 0.002 | 8.20E-01 |
| rs915814 | 21 | 46493003 | A | G | -0.018 | 0.003 | 5.88E-09 | 0.001 | 0.003 | 5.70E-01 |
| rs427943 | 21 | 46570896 | A | C | -0.018 | 0.002 | 1.37E-14 | 0.001 | 0.002 | 5.00E-01 |
| rs28489620 | 22 | 41804716 | A | G | -0.018 | 0.003 | 6.90E-10 | -0.001 | 0.002 | 6.60E-01 |
| rs13053342 | 22 | 48871624 | A | G | 0.015 | 0.002 | 1.32E-10 | -0.001 | 0.002 | 7.60E-01 |
| **6) MR of leisure computer use on BMI (women)** | | | | | | | | | | |
| SNP | CHR | POS | EA | OA | beta_bmi | se_bmi | pval_bmi | beta_pc | se_pc | pval_pc |
| rs2748985 | 1 | 1853184 | T | C | -0.015 | 0.003 | 3.64E-08 | -0.015 | 0.002 | 4.10E-12 |
| rs12145677 | 1 | 110023610 | G | A | -0.011 | 0.002 | 1.50E-05 | -0.017 | 0.002 | 1.20E-12 |
| rs7526456 | 1 | 197773734 | T | C | 0.010 | 0.002 | 3.75E-05 | -0.013 | 0.002 | 1.20E-08 |
| rs10754920 | 2 | 146456888 | C | G | 0.008 | 0.004 | 3.86E-02 | 0.020 | 0.003 | 6.70E-09 |
| rs6744254 | 2 | 155986195 | C | T | -0.005 | 0.002 | 4.60E-02 | -0.016 | 0.002 | 5.40E-13 |
| rs11274218 | 2 | 215035001 | C | T | 0.004 | 0.002 | 8.24E-02 | -0.014 | 0.002 | 2.50E-09 |
| rs4592851 | 2 | 215364232 | G | T | 0.006 | 0.003 | 3.34E-02 | -0.014 | 0.003 | 1.40E-08 |
| rs10208088 | 2 | 221055873 | C | T | 0.000 | 0.003 | 9.66E-01 | 0.013 | 0.002 | 4.40E-09 |
| rs11708955 | 3 | 49540114 | T | C | 0.015 | 0.003 | 2.36E-09 | -0.016 | 0.002 | 4.20E-11 |
| rs6774533 | 3 | 62471086 | C | T | -0.016 | 0.003 | 2.30E-08 | -0.015 | 0.002 | 1.20E-09 |
| rs78082503 | 3 | 116556913 | G | C | -0.001 | 0.003 | 7.24E-01 | -0.015 | 0.003 | 1.10E-08 |
| rs6857629 | 4 | 39684977 | G | A | -0.004 | 0.003 | 1.99E-01 | 0.015 | 0.003 | 1.90E-09 |
| rs2068625 | 4 | 159856739 | T | C | 0.010 | 0.003 | 3.92E-05 | -0.016 | 0.002 | 4.00E-11 |
| rs2220599 | 5 | 7378854 | C | G | 0.006 | 0.002 | 8.26E-03 | -0.016 | 0.002 | 2.40E-12 |
| rs11749912 | 5 | 88065628 | A | G | 0.006 | 0.003 | 1.79E-02 | 0.014 | 0.002 | 4.90E-10 |
| rs1469249 | 5 | 113837198 | G | A | -0.012 | 0.003 | 3.12E-05 | 0.016 | 0.003 | 9.50E-09 |
| rs162894 | 5 | 131611872 | T | G | 0.003 | 0.002 | 2.43E-01 | 0.013 | 0.002 | 1.10E-08 |
| rs56158102 | 5 | 144027803 | C | T | 0.000 | 0.002 | 9.11E-01 | 0.013 | 0.002 | 3.10E-08 |
| rs9477970 | 6 | 19082663 | T | A | -0.013 | 0.003 | 7.03E-06 | -0.016 | 0.003 | 1.00E-08 |
| rs2179517 | 6 | 26198845 | G | C | 0.009 | 0.003 | 7.75E-04 | -0.012 | 0.002 | 1.90E-08 |
| rs9372625 | 6 | 98344031 | G | A | 0.013 | 0.002 | 1.20E-07 | -0.018 | 0.002 | 5.70E-16 |
| rs17789218 | 6 | 100600097 | T | C | -0.007 | 0.003 | 1.07E-02 | -0.015 | 0.003 | 4.20E-09 |
| rs6935828 | 6 | 140811367 | C | T | -0.002 | 0.002 | 3.92E-01 | -0.012 | 0.002 | 4.30E-08 |
| rs55772938 | 7 | 70025113 | A | G | -0.008 | 0.003 | 4.34E-03 | -0.015 | 0.002 | 3.90E-10 |
| rs12706626 | 7 | 124531370 | G | A | 0.001 | 0.003 | 8.39E-01 | -0.013 | 0.002 | 1.40E-08 |
| rs3944151 | 7 | 126970005 | A | G | -0.001 | 0.003 | 7.41E-01 | 0.014 | 0.002 | 3.10E-08 |
| rs2345941 | 7 | 133337635 | A | G | -0.001 | 0.002 | 8.00E-01 | 0.015 | 0.002 | 4.00E-11 |
| rs13262595 | 8 | 143316970 | A | G | -0.006 | 0.002 | 1.61E-02 | -0.016 | 0.002 | 1.20E-12 |
| rs4977839 | 9 | 23355310 | G | A | -0.011 | 0.003 | 6.06E-05 | -0.020 | 0.002 | 4.70E-19 |
| rs113851275 | 9 | 98297220 | G | A | 0.005 | 0.004 | 2.79E-01 | -0.021 | 0.004 | 3.20E-09 |
| rs7020477 | 9 | 116827760 | A | G | 0.003 | 0.003 | 3.01E-01 | 0.014 | 0.002 | 1.20E-08 |
| rs73578186 | 9 | 126334485 | C | T | -0.009 | 0.003 | 1.79E-03 | 0.015 | 0.002 | 2.30E-10 |
| rs2734849 | 11 | 113270160 | A | G | 0.008 | 0.002 | 2.10E-04 | -0.014 | 0.002 | 8.20E-10 |
| rs1448355 | 11 | 131286685 | C | T | 0.001 | 0.002 | 6.03E-01 | -0.015 | 0.002 | 1.30E-10 |
| rs35933007 | 12 | 38929341 | G | A | 0.007 | 0.003 | 3.97E-02 | -0.015 | 0.003 | 9.20E-09 |
| rs1319734 | 12 | 40367517 | A | G | 0.004 | 0.002 | 1.19E-01 | 0.013 | 0.002 | 1.90E-08 |
| rs206965 | 12 | 120856332 | T | C | 0.000 | 0.003 | 9.16E-01 | 0.016 | 0.003 | 9.10E-09 |
| rs12874776 | 13 | 57794345 | C | A | 0.001 | 0.003 | 8.91E-01 | -0.023 | 0.004 | 8.00E-11 |
| rs198262 | 14 | 57283283 | C | T | 0.003 | 0.006 | 6.11E-01 | 0.030 | 0.005 | 3.90E-08 |
| rs166835 | 15 | 47716037 | C | T | -0.001 | 0.002 | 8.32E-01 | 0.013 | 0.002 | 3.40E-09 |
| rs4702 | 15 | 91426560 | G | A | 0.000 | 0.002 | 9.14E-01 | 0.012 | 0.002 | 3.80E-08 |
| rs6498759 | 16 | 18020295 | T | C | -0.001 | 0.003 | 5.94E-01 | 0.013 | 0.002 | 2.70E-08 |
| rs3730399 | 16 | 67229019 | A | G | -0.009 | 0.005 | 7.35E-02 | 0.025 | 0.004 | 3.30E-08 |
| rs4073003 | 17 | 19148305 | A | G | -0.006 | 0.003 | 5.15E-02 | 0.020 | 0.003 | 1.20E-09 |
| rs7209653 | 17 | 19882084 | T | C | 0.002 | 0.003 | 4.02E-01 | 0.016 | 0.002 | 9.10E-11 |
| rs12603813 | 17 | 43196584 | T | C | -0.003 | 0.003 | 2.94E-01 | 0.014 | 0.003 | 1.30E-08 |
| rs66643547 | 17 | 79375315 | C | T | 0.001 | 0.003 | 6.52E-01 | -0.015 | 0.002 | 3.20E-11 |
| rs784256 | 18 | 53398626 | G | A | -0.018 | 0.003 | 1.77E-09 | 0.019 | 0.003 | 6.20E-11 |
| rs6129084 | 20 | 59828857 | A | T | -0.005 | 0.003 | 4.80E-02 | -0.014 | 0.002 | 9.30E-10 |
| rs9608249 | 22 | 24613952 | G | A | -0.002 | 0.004 | 6.57E-01 | 0.017 | 0.003 | 2.20E-08 |
| rs136553 | 22 | 27255675 | C | T | -0.010 | 0.003 | 1.86E-04 | -0.015 | 0.002 | 2.30E-11 |
| rs7288455 | 22 | 39966547 | A | G | 0.001 | 0.003 | 7.53E-01 | 0.013 | 0.002 | 4.00E-09 |

| **Table S29. Betas of the bidirectional MR between BMI and leisure television use** | | | | | | | | | | |
| --- | --- | --- | --- | --- | --- | --- | --- | --- | --- | --- |
| **1) MR of BMI (men and women combined) on leisure television use** | | | | | | | | | | |
| SNP | CHR | POS | EA | OA | beta_bmi | se_bmi | pval_bmi | beta_tv | se_tv | pval_tv |
| rs79113395 | 1 | 1590521 | A | G | -0.020 | 0.002 | 2.03E-20 | -0.007 | 0.002 | 4.50E-03 |
| rs10909880 | 1 | 2727804 | T | C | -0.014 | 0.002 | 2.21E-16 | 0.000 | 0.002 | 9.30E-01 |
| rs11121210 | 1 | 8708529 | T | C | -0.011 | 0.002 | 2.37E-10 | -0.003 | 0.002 | 2.40E-01 |
| rs10779751 | 1 | 11284336 | A | G | 0.013 | 0.002 | 2.66E-13 | 0.001 | 0.002 | 5.50E-01 |
| rs61740466 | 1 | 19934900 | A | G | -0.014 | 0.002 | 2.24E-10 | -0.003 | 0.003 | 2.10E-01 |
| rs4655141 | 1 | 23312025 | T | C | -0.017 | 0.002 | 6.20E-15 | 0.000 | 0.003 | 9.60E-01 |
| rs2228552 | 1 | 32165495 | T | G | 0.012 | 0.002 | 1.80E-11 | 0.006 | 0.002 | 4.00E-03 |
| rs4653017 | 1 | 33776728 | T | C | 0.012 | 0.002 | 1.11E-10 | -0.001 | 0.002 | 7.30E-01 |
| rs7512146 | 1 | 34283008 | T | G | -0.010 | 0.002 | 1.29E-08 | 0.001 | 0.002 | 6.80E-01 |
| rs11577094 | 1 | 38026600 | T | C | 0.019 | 0.003 | 3.28E-10 | 0.008 | 0.004 | 2.90E-02 |
| rs112646560 | 1 | 39560250 | T | C | 0.018 | 0.002 | 1.29E-14 | 0.005 | 0.003 | 7.60E-02 |
| rs2984618 | 1 | 47690438 | T | G | 0.017 | 0.002 | 3.80E-24 | 0.001 | 0.002 | 6.40E-01 |
| rs657452 | 1 | 49589847 | A | G | 0.019 | 0.002 | 3.17E-30 | -0.004 | 0.002 | 7.00E-02 |
| rs587271 | 1 | 54743111 | T | C | 0.012 | 0.002 | 6.21E-11 | -0.004 | 0.002 | 1.00E-01 |
| rs79906980 | 1 | 57887985 | T | C | 0.016 | 0.003 | 1.69E-09 | 0.008 | 0.003 | 7.10E-03 |
| rs12140153 | 1 | 62579891 | T | G | -0.035 | 0.003 | 1.44E-25 | -0.014 | 0.004 | 2.20E-04 |
| rs2503185 | 1 | 66461401 | A | G | 0.013 | 0.002 | 1.33E-14 | 0.006 | 0.002 | 9.20E-03 |
| rs3101336 | 1 | 72751185 | T | C | -0.025 | 0.002 | 4.80E-54 | 0.007 | 0.002 | 1.30E-03 |
| rs6656785 | 1 | 75005776 | A | G | -0.018 | 0.002 | 3.06E-27 | 0.003 | 0.002 | 2.00E-01 |
| rs34517439 | 1 | 78450517 | A | C | 0.039 | 0.003 | 3.40E-39 | 0.020 | 0.003 | 8.60E-10 |
| rs6696828 | 1 | 80812020 | C | G | 0.012 | 0.002 | 6.42E-11 | 0.002 | 0.002 | 3.60E-01 |
| rs321237 | 1 | 96478125 | A | G | 0.013 | 0.002 | 1.49E-10 | -0.009 | 0.003 | 7.00E-04 |
| rs11165643 | 1 | 96924097 | T | C | 0.019 | 0.002 | 4.49E-30 | 0.004 | 0.002 | 8.70E-02 |
| rs12072739 | 1 | 98315893 | A | G | -0.017 | 0.002 | 1.50E-13 | -0.019 | 0.003 | 2.80E-13 |
| rs17024393 | 1 | 110154688 | T | C | -0.064 | 0.005 | 7.11E-39 | 0.002 | 0.007 | 8.00E-01 |
| rs197374 | 1 | 112289983 | T | C | 0.014 | 0.002 | 3.16E-16 | 0.009 | 0.002 | 5.50E-05 |
| rs17544384 | 1 | 115295160 | T | C | -0.013 | 0.002 | 2.28E-08 | -0.007 | 0.003 | 1.00E-02 |
| rs7534091 | 1 | 118864616 | A | G | -0.012 | 0.002 | 6.65E-11 | -0.002 | 0.002 | 3.60E-01 |
| rs74887628 | 1 | 147032779 | A | G | 0.031 | 0.005 | 1.76E-08 | 0.004 | 0.006 | 5.20E-01 |
| rs4970991 | 1 | 151004003 | T | C | 0.012 | 0.002 | 1.81E-08 | 0.008 | 0.003 | 2.80E-03 |
| rs61813324 | 1 | 156049877 | T | C | 0.029 | 0.003 | 3.20E-24 | 0.015 | 0.003 | 4.30E-06 |
| rs1750307 | 1 | 156488420 | A | T | 0.013 | 0.002 | 2.78E-13 | 0.002 | 0.002 | 3.40E-01 |
| rs10733051 | 1 | 167280354 | A | G | 0.009 | 0.002 | 6.96E-09 | 0.003 | 0.002 | 2.40E-01 |
| rs61828641 | 1 | 174321997 | A | G | 0.022 | 0.003 | 2.49E-13 | -0.008 | 0.003 | 1.50E-02 |
| rs543874 | 1 | 177889480 | A | G | -0.048 | 0.002 | 3.06E-125 | -0.004 | 0.003 | 9.30E-02 |
| rs10920678 | 1 | 190239907 | A | G | 0.015 | 0.002 | 7.15E-20 | 0.002 | 0.002 | 3.20E-01 |
| rs2400414 | 1 | 194965200 | T | C | -0.013 | 0.002 | 5.52E-13 | -0.003 | 0.002 | 2.50E-01 |
| rs2820295 | 1 | 201800868 | A | G | 0.024 | 0.002 | 5.56E-39 | 0.010 | 0.002 | 8.70E-06 |
| rs10920336 | 1 | 202115945 | A | G | -0.010 | 0.002 | 4.20E-09 | -0.002 | 0.002 | 3.10E-01 |
| rs1006317 | 1 | 209552636 | T | G | 0.016 | 0.003 | 3.75E-10 | 0.001 | 0.003 | 6.90E-01 |
| rs6661316 | 1 | 210095527 | T | C | 0.012 | 0.002 | 1.72E-13 | 0.006 | 0.002 | 7.20E-03 |
| rs10864728 | 1 | 230304914 | A | G | 0.011 | 0.002 | 1.20E-08 | 0.007 | 0.002 | 3.10E-03 |
| rs946824 | 1 | 243684019 | T | C | 0.020 | 0.003 | 4.82E-15 | 0.010 | 0.003 | 2.60E-03 |
| rs6710091 | 2 | 239597 | C | G | 0.010 | 0.002 | 5.78E-09 | 0.005 | 0.002 | 4.50E-02 |
| rs77165542 | 2 | 430975 | T | C | -0.094 | 0.005 | 1.51E-70 | 0.003 | 0.006 | 5.90E-01 |
| rs13021737 | 2 | 632348 | A | G | -0.058 | 0.002 | 2.89E-161 | -0.004 | 0.003 | 1.40E-01 |
| rs10929925 | 2 | 6155557 | A | C | -0.014 | 0.002 | 3.05E-18 | -0.004 | 0.002 | 7.70E-02 |
| rs11902450 | 2 | 12845368 | T | C | 0.017 | 0.003 | 1.06E-09 | 0.012 | 0.004 | 5.30E-04 |
| rs10182181 | 2 | 25150296 | A | G | -0.033 | 0.002 | 2.45E-91 | -0.001 | 0.002 | 6.90E-01 |
| rs935166 | 2 | 26949366 | A | G | -0.015 | 0.002 | 8.69E-16 | -0.009 | 0.002 | 8.10E-05 |
| rs10168563 | 2 | 35552173 | A | G | 0.013 | 0.002 | 9.33E-12 | 0.007 | 0.002 | 3.80E-03 |
| rs3770890 | 2 | 36657992 | T | G | -0.030 | 0.005 | 2.10E-08 | -0.002 | 0.007 | 8.00E-01 |
| rs10185199 | 2 | 40282202 | A | G | -0.014 | 0.002 | 3.18E-13 | -0.006 | 0.002 | 1.00E-02 |
| rs10169594 | 2 | 41637688 | T | C | -0.012 | 0.002 | 1.34E-11 | -0.011 | 0.002 | 6.90E-07 |
| rs7561278 | 2 | 48954905 | T | C | 0.017 | 0.002 | 4.89E-16 | 0.003 | 0.003 | 1.90E-01 |
| rs930295 | 2 | 50233352 | A | C | 0.021 | 0.002 | 2.03E-19 | 0.006 | 0.003 | 5.90E-02 |
| rs805412 | 2 | 54120820 | A | G | -0.010 | 0.002 | 1.14E-08 | 0.008 | 0.002 | 3.60E-04 |
| rs4671328 | 2 | 58935282 | T | G | 0.021 | 0.002 | 3.42E-36 | 0.010 | 0.002 | 4.60E-06 |
| rs6545714 | 2 | 59307725 | A | G | -0.019 | 0.002 | 4.01E-32 | -0.006 | 0.002 | 3.50E-03 |
| rs2861685 | 2 | 67837553 | T | C | 0.017 | 0.002 | 7.82E-18 | 0.001 | 0.002 | 7.60E-01 |
| rs17020497 | 2 | 81826131 | A | G | 0.014 | 0.003 | 2.04E-08 | 0.008 | 0.003 | 7.90E-03 |
| rs12714199 | 2 | 86812549 | T | C | -0.014 | 0.002 | 3.22E-16 | -0.002 | 0.002 | 4.80E-01 |
| rs4303732 | 2 | 100830040 | T | C | 0.017 | 0.002 | 1.26E-22 | 0.018 | 0.002 | 7.10E-17 |
| rs264941 | 2 | 104297420 | A | C | -0.012 | 0.002 | 1.84E-13 | -0.012 | 0.002 | 9.30E-09 |
| rs10197031 | 2 | 105454590 | T | C | -0.016 | 0.002 | 5.05E-18 | -0.015 | 0.002 | 8.90E-10 |
| rs13033310 | 2 | 133523605 | A | G | 0.015 | 0.002 | 3.40E-11 | 0.004 | 0.002 | 1.50E-01 |
| rs4988235 | 2 | 136608646 | A | G | 0.012 | 0.002 | 7.09E-13 | 0.007 | 0.003 | 5.80E-03 |
| rs17814208 | 2 | 144037998 | A | G | -0.013 | 0.002 | 7.24E-11 | -0.007 | 0.003 | 5.90E-03 |
| rs1451077 | 2 | 147901207 | A | G | -0.017 | 0.002 | 1.43E-18 | -0.009 | 0.002 | 2.70E-05 |
| rs2119753 | 2 | 151224579 | A | G | 0.010 | 0.002 | 1.00E-08 | 0.005 | 0.002 | 3.80E-02 |
| rs11695013 | 2 | 157057487 | T | C | -0.010 | 0.002 | 2.43E-09 | 0.000 | 0.002 | 8.80E-01 |
| rs3764835 | 2 | 159519368 | A | G | -0.013 | 0.002 | 3.84E-08 | -0.006 | 0.003 | 5.00E-02 |
| rs12692596 | 2 | 161265910 | T | C | 0.012 | 0.002 | 1.03E-12 | 0.009 | 0.002 | 3.60E-05 |
| rs62176243 | 2 | 166190881 | A | T | 0.015 | 0.002 | 2.41E-11 | 0.013 | 0.002 | 1.00E-07 |
| rs56133507 | 2 | 172818467 | T | G | -0.013 | 0.002 | 4.31E-08 | -0.007 | 0.003 | 9.80E-03 |
| rs34234296 | 2 | 175166636 | A | G | -0.015 | 0.002 | 2.01E-13 | -0.004 | 0.002 | 1.10E-01 |
| rs7588437 | 2 | 181575281 | A | G | -0.017 | 0.002 | 2.32E-22 | -0.006 | 0.002 | 1.40E-02 |
| rs6716898 | 2 | 198944271 | A | G | 0.013 | 0.002 | 2.33E-11 | 0.008 | 0.002 | 1.30E-04 |
| rs7593917 | 2 | 203931012 | A | G | -0.012 | 0.002 | 9.67E-13 | 0.002 | 0.002 | 2.90E-01 |
| rs4482463 | 2 | 205375909 | A | C | -0.031 | 0.003 | 4.85E-23 | -0.001 | 0.004 | 7.80E-01 |
| rs11692326 | 2 | 208263279 | T | C | 0.015 | 0.002 | 1.69E-14 | 0.004 | 0.003 | 9.40E-02 |
| rs73985439 | 2 | 212299249 | A | C | -0.013 | 0.002 | 1.84E-10 | -0.010 | 0.002 | 3.30E-05 |
| rs7599312 | 2 | 213413231 | A | G | -0.018 | 0.002 | 1.52E-23 | -0.009 | 0.002 | 1.10E-04 |
| rs12987009 | 2 | 219658170 | A | T | -0.011 | 0.002 | 1.83E-10 | -0.004 | 0.002 | 1.00E-01 |
| rs6725931 | 2 | 220205146 | T | C | 0.019 | 0.002 | 2.30E-15 | 0.004 | 0.003 | 2.20E-01 |
| rs4973618 | 2 | 229002620 | A | G | -0.015 | 0.002 | 1.25E-16 | -0.007 | 0.002 | 1.30E-03 |
| rs6720868 | 2 | 230663576 | T | C | 0.015 | 0.002 | 1.82E-17 | 0.006 | 0.002 | 1.10E-02 |
| rs7568228 | 2 | 236848488 | C | G | -0.010 | 0.002 | 1.00E-09 | -0.006 | 0.002 | 7.00E-03 |
| rs59302296 | 3 | 9507314 | A | T | 0.022 | 0.003 | 9.12E-12 | 0.007 | 0.004 | 5.30E-02 |
| rs6783054 | 3 | 11672805 | A | C | 0.010 | 0.002 | 6.22E-09 | 0.001 | 0.002 | 7.40E-01 |
| rs10510419 | 3 | 12426936 | T | G | -0.017 | 0.002 | 2.23E-13 | -0.006 | 0.003 | 3.70E-02 |
| rs2600226 | 3 | 12928762 | T | C | -0.012 | 0.002 | 1.42E-10 | -0.002 | 0.002 | 4.90E-01 |
| rs1048637 | 3 | 13358171 | T | G | -0.009 | 0.002 | 2.94E-08 | 0.003 | 0.002 | 1.80E-01 |
| rs4858193 | 3 | 20441050 | T | C | 0.013 | 0.002 | 2.44E-12 | 0.001 | 0.002 | 5.90E-01 |
| rs6804842 | 3 | 25106437 | A | G | -0.014 | 0.002 | 7.57E-18 | -0.001 | 0.002 | 5.60E-01 |
| rs11921432 | 3 | 35117776 | T | C | -0.019 | 0.003 | 5.38E-12 | 0.000 | 0.003 | 9.60E-01 |
| rs1799923 | 3 | 42306294 | A | G | -0.022 | 0.003 | 1.12E-17 | -0.002 | 0.003 | 4.90E-01 |
| rs28350 | 3 | 42418446 | A | G | 0.017 | 0.002 | 1.07E-14 | 0.002 | 0.003 | 3.90E-01 |
| rs4017425 | 3 | 44028764 | T | C | -0.012 | 0.002 | 2.91E-12 | -0.008 | 0.002 | 2.50E-04 |
| rs11919665 | 3 | 48085349 | A | T | 0.012 | 0.002 | 7.30E-09 | 0.000 | 0.002 | 8.80E-01 |
| rs11713193 | 3 | 49924424 | A | G | 0.025 | 0.002 | 3.02E-48 | 0.025 | 0.002 | 1.20E-31 |
| rs2365389 | 3 | 61236462 | T | C | -0.017 | 0.002 | 6.49E-25 | -0.007 | 0.002 | 1.40E-03 |
| rs6445258 | 3 | 62112198 | T | C | 0.013 | 0.002 | 2.48E-08 | -0.002 | 0.003 | 4.60E-01 |
| rs925018 | 3 | 62713143 | C | G | -0.013 | 0.002 | 4.33E-14 | -0.004 | 0.002 | 7.60E-02 |
| rs11915371 | 3 | 70539559 | A | C | -0.015 | 0.002 | 2.29E-13 | -0.012 | 0.003 | 4.20E-06 |
| rs12636480 | 3 | 82719412 | T | G | 0.013 | 0.002 | 6.96E-13 | 0.001 | 0.002 | 6.80E-01 |
| rs9827823 | 3 | 84221774 | T | C | 0.018 | 0.002 | 3.39E-15 | 0.012 | 0.003 | 1.10E-04 |
| rs9818122 | 3 | 85861064 | T | C | -0.023 | 0.002 | 3.97E-30 | -0.010 | 0.003 | 1.50E-04 |
| rs34184235 | 3 | 86192846 | T | C | -0.012 | 0.002 | 2.14E-09 | -0.004 | 0.002 | 4.20E-02 |
| rs11128021 | 3 | 88139016 | A | G | -0.018 | 0.002 | 1.63E-14 | -0.009 | 0.003 | 2.90E-03 |
| rs1492014 | 3 | 94071481 | T | C | -0.017 | 0.002 | 1.44E-23 | -0.013 | 0.002 | 1.30E-09 |
| rs1436344 | 3 | 104606144 | C | G | 0.015 | 0.002 | 1.07E-17 | 0.004 | 0.002 | 1.00E-01 |
| rs7640424 | 3 | 107820063 | T | C | -0.014 | 0.002 | 1.23E-14 | -0.006 | 0.002 | 1.20E-02 |
| rs17681451 | 3 | 114399296 | A | G | -0.023 | 0.003 | 7.37E-13 | -0.017 | 0.004 | 1.80E-05 |
| rs6808814 | 3 | 116852469 | T | C | 0.012 | 0.002 | 1.31E-09 | 0.006 | 0.002 | 2.00E-02 |
| rs7631156 | 3 | 131751628 | A | G | 0.022 | 0.002 | 3.33E-32 | 0.002 | 0.002 | 5.10E-01 |
| rs687339 | 3 | 135932359 | T | C | 0.019 | 0.002 | 4.32E-22 | 0.004 | 0.003 | 1.10E-01 |
| rs16851483 | 3 | 141275436 | T | G | 0.035 | 0.003 | 4.87E-25 | 0.013 | 0.004 | 2.90E-03 |
| rs355777 | 3 | 154034950 | C | G | 0.015 | 0.002 | 2.13E-18 | 0.003 | 0.002 | 1.70E-01 |
| rs9826775 | 3 | 156295341 | A | G | 0.016 | 0.002 | 6.61E-11 | 0.002 | 0.003 | 4.60E-01 |
| rs2047648 | 3 | 157033438 | A | T | -0.013 | 0.002 | 7.95E-12 | -0.003 | 0.002 | 2.90E-01 |
| rs3732927 | 3 | 170586057 | T | C | 0.010 | 0.002 | 1.13E-08 | 0.006 | 0.002 | 7.10E-03 |
| rs12635553 | 3 | 171113714 | A | T | 0.010 | 0.002 | 4.10E-09 | 0.001 | 0.002 | 5.00E-01 |
| rs39654 | 3 | 173095123 | A | G | -0.016 | 0.002 | 1.75E-21 | -0.005 | 0.002 | 2.50E-02 |
| rs6443750 | 3 | 181329682 | T | C | -0.015 | 0.002 | 7.25E-13 | -0.002 | 0.003 | 5.20E-01 |
| rs865809 | 3 | 183997735 | A | G | 0.012 | 0.002 | 6.50E-10 | -0.006 | 0.003 | 3.30E-02 |
| rs9816226 | 3 | 185834499 | A | T | -0.032 | 0.002 | 1.45E-50 | 0.007 | 0.003 | 8.70E-03 |
| rs7616009 | 3 | 194881756 | A | G | -0.016 | 0.002 | 4.33E-11 | -0.008 | 0.003 | 5.60E-03 |
| rs6803161 | 3 | 196205694 | T | C | 0.011 | 0.002 | 2.71E-08 | 0.006 | 0.002 | 4.20E-03 |
| rs2051559 | 4 | 3298800 | T | C | -0.017 | 0.003 | 3.78E-11 | -0.021 | 0.003 | 1.10E-10 |
| rs12642970 | 4 | 16601492 | C | G | -0.011 | 0.002 | 1.71E-08 | -0.007 | 0.002 | 8.40E-04 |
| rs994596 | 4 | 18459828 | T | C | 0.013 | 0.002 | 1.06E-12 | 0.001 | 0.002 | 5.30E-01 |
| rs34811474 | 4 | 25408838 | A | G | -0.029 | 0.002 | 8.50E-38 | -0.015 | 0.003 | 2.30E-09 |
| rs73213484 | 4 | 28489339 | A | T | 0.021 | 0.003 | 2.04E-14 | 0.002 | 0.003 | 4.40E-01 |
| rs337637 | 4 | 38604470 | A | G | -0.014 | 0.002 | 6.24E-16 | -0.002 | 0.002 | 3.40E-01 |
| rs10938397 | 4 | 45182527 | A | G | -0.032 | 0.002 | 2.42E-86 | -0.011 | 0.002 | 9.30E-07 |
| rs2271046 | 4 | 52752812 | A | T | -0.012 | 0.002 | 3.03E-10 | -0.014 | 0.002 | 8.10E-09 |
| rs1492767 | 4 | 55221467 | T | C | 0.010 | 0.002 | 3.55E-09 | 0.004 | 0.002 | 7.90E-02 |
| rs2192158 | 4 | 55505360 | A | G | 0.014 | 0.002 | 7.42E-16 | 0.005 | 0.002 | 2.70E-02 |
| rs1346841 | 4 | 65651730 | A | G | -0.013 | 0.002 | 3.18E-13 | -0.001 | 0.002 | 5.70E-01 |
| rs10002111 | 4 | 67815504 | A | G | 0.013 | 0.002 | 1.26E-09 | 0.007 | 0.003 | 4.30E-03 |
| rs10033843 | 4 | 77028783 | A | G | 0.014 | 0.002 | 1.07E-11 | 0.013 | 0.003 | 2.80E-06 |
| rs72649373 | 4 | 80609966 | T | C | -0.017 | 0.003 | 3.20E-09 | -0.008 | 0.003 | 1.00E-02 |
| rs4148155 | 4 | 89054667 | A | G | 0.019 | 0.003 | 1.34E-13 | 0.009 | 0.003 | 6.60E-03 |
| rs4286488 | 4 | 94440026 | A | G | 0.012 | 0.002 | 1.70E-09 | 0.010 | 0.003 | 1.20E-04 |
| rs7678054 | 4 | 95093855 | A | G | -0.010 | 0.002 | 4.57E-09 | -0.002 | 0.002 | 2.50E-01 |
| rs3796432 | 4 | 96030402 | T | G | -0.011 | 0.002 | 2.21E-10 | 0.000 | 0.002 | 9.30E-01 |
| rs13107325 | 4 | 103188709 | T | C | 0.047 | 0.003 | 3.81E-47 | 0.029 | 0.004 | 1.50E-12 |
| rs326893 | 4 | 112691776 | T | C | 0.012 | 0.002 | 1.89E-12 | 0.003 | 0.002 | 1.40E-01 |
| rs7696649 | 4 | 120322177 | A | G | 0.012 | 0.002 | 4.73E-10 | 0.003 | 0.002 | 1.50E-01 |
| rs4864201 | 4 | 130731284 | T | C | 0.014 | 0.002 | 4.30E-16 | 0.004 | 0.002 | 1.10E-01 |
| rs1296328 | 4 | 137083193 | A | C | 0.017 | 0.002 | 3.49E-22 | 0.006 | 0.002 | 1.00E-02 |
| rs17367750 | 4 | 140782542 | T | C | -0.012 | 0.002 | 1.97E-11 | -0.009 | 0.002 | 4.00E-05 |
| rs9992189 | 4 | 144060728 | C | G | -0.010 | 0.002 | 2.04E-08 | -0.002 | 0.002 | 4.70E-01 |
| rs3914628 | 4 | 147438019 | T | C | 0.017 | 0.002 | 6.91E-13 | 0.006 | 0.003 | 3.90E-02 |
| rs750090 | 4 | 152931436 | T | C | 0.011 | 0.002 | 2.50E-10 | -0.004 | 0.002 | 1.00E-01 |
| rs13110266 | 4 | 162129844 | A | G | -0.012 | 0.002 | 3.96E-14 | -0.006 | 0.002 | 7.90E-03 |
| rs7685628 | 4 | 165310133 | A | T | 0.010 | 0.002 | 6.15E-09 | 0.006 | 0.002 | 1.10E-02 |
| rs1522569 | 4 | 171632637 | T | G | 0.014 | 0.002 | 1.60E-10 | 0.004 | 0.003 | 1.10E-01 |
| rs1437842 | 4 | 173597016 | A | G | -0.011 | 0.002 | 8.49E-10 | -0.007 | 0.002 | 5.40E-04 |
| rs6850421 | 4 | 180187034 | A | G | 0.011 | 0.002 | 3.66E-09 | 0.000 | 0.002 | 9.20E-01 |
| rs35408866 | 4 | 187743245 | A | G | 0.016 | 0.003 | 1.50E-08 | 0.008 | 0.003 | 1.10E-02 |
| rs698147 | 5 | 3513485 | A | G | 0.012 | 0.002 | 9.67E-12 | -0.003 | 0.002 | 1.60E-01 |
| rs6890310 | 5 | 27193573 | A | G | -0.012 | 0.002 | 3.29E-10 | -0.003 | 0.002 | 1.90E-01 |
| rs7730004 | 5 | 43191033 | T | C | 0.014 | 0.002 | 1.46E-14 | 0.005 | 0.002 | 3.50E-02 |
| rs116374395 | 5 | 50723410 | A | G | 0.032 | 0.005 | 7.05E-10 | -0.004 | 0.006 | 5.50E-01 |
| rs150215901 | 5 | 50935903 | A | T | -0.028 | 0.005 | 1.54E-08 | -0.006 | 0.005 | 2.80E-01 |
| rs4865796 | 5 | 53272664 | A | G | -0.010 | 0.002 | 4.21E-08 | 0.000 | 0.002 | 9.20E-01 |
| rs13186194 | 5 | 60795485 | T | C | 0.010 | 0.002 | 2.20E-09 | -0.015 | 0.002 | 2.10E-11 |
| rs4700608 | 5 | 63026280 | T | C | -0.016 | 0.002 | 4.32E-20 | -0.015 | 0.002 | 1.50E-11 |
| rs10050620 | 5 | 63927239 | T | C | -0.013 | 0.002 | 1.20E-10 | -0.005 | 0.002 | 4.10E-02 |
| rs1159692 | 5 | 63977815 | A | C | 0.014 | 0.002 | 5.54E-15 | -0.003 | 0.002 | 2.20E-01 |
| rs2112347 | 5 | 75015242 | T | G | 0.028 | 0.002 | 1.17E-61 | 0.007 | 0.002 | 2.10E-03 |
| rs10942267 | 5 | 80841914 | A | G | 0.015 | 0.002 | 6.53E-16 | 0.000 | 0.002 | 8.60E-01 |
| rs2962334 | 5 | 86879056 | T | G | 0.040 | 0.006 | 1.78E-11 | 0.001 | 0.008 | 8.70E-01 |
| rs1501673 | 5 | 87963600 | A | G | 0.029 | 0.003 | 2.73E-31 | 0.004 | 0.003 | 2.30E-01 |
| rs12652212 | 5 | 88808594 | A | G | -0.013 | 0.002 | 1.63E-15 | -0.009 | 0.002 | 5.10E-05 |
| rs7713317 | 5 | 95716722 | A | G | -0.017 | 0.002 | 1.96E-20 | -0.001 | 0.002 | 5.30E-01 |
| rs6882366 | 5 | 95864693 | T | C | -0.013 | 0.002 | 4.39E-14 | 0.000 | 0.002 | 9.90E-01 |
| rs11739877 | 5 | 105876806 | T | C | 0.012 | 0.002 | 4.01E-11 | 0.003 | 0.002 | 1.60E-01 |
| rs6888194 | 5 | 106910657 | T | C | -0.013 | 0.002 | 3.58E-08 | -0.001 | 0.003 | 6.20E-01 |
| rs40067 | 5 | 107439012 | A | G | -0.025 | 0.002 | 9.73E-29 | -0.002 | 0.003 | 5.40E-01 |
| rs459552 | 5 | 112176756 | A | T | -0.013 | 0.002 | 8.36E-12 | -0.010 | 0.003 | 1.80E-04 |
| rs6893539 | 5 | 122705737 | A | C | -0.012 | 0.002 | 6.17E-11 | 0.004 | 0.002 | 6.50E-02 |
| rs6864049 | 5 | 124330522 | A | G | -0.012 | 0.002 | 1.48E-13 | -0.010 | 0.002 | 5.20E-06 |
| rs6886072 | 5 | 136598460 | T | C | -0.010 | 0.002 | 4.13E-09 | -0.004 | 0.002 | 8.20E-02 |
| rs13174863 | 5 | 139080745 | A | G | -0.020 | 0.002 | 1.94E-17 | -0.002 | 0.003 | 6.00E-01 |
| rs17405603 | 5 | 144608340 | A | T | -0.013 | 0.002 | 4.43E-11 | -0.001 | 0.002 | 5.40E-01 |
| rs2910026 | 5 | 152529936 | T | C | -0.013 | 0.002 | 5.69E-10 | -0.011 | 0.002 | 4.80E-06 |
| rs7715256 | 5 | 153537893 | T | G | -0.016 | 0.002 | 3.98E-22 | 0.003 | 0.002 | 2.00E-01 |
| rs7734385 | 5 | 158460212 | A | G | -0.010 | 0.002 | 6.08E-10 | 0.000 | 0.002 | 8.80E-01 |
| rs4921301 | 5 | 159984492 | T | C | -0.013 | 0.002 | 4.03E-08 | -0.003 | 0.003 | 2.70E-01 |
| rs2861089 | 5 | 164557954 | A | T | 0.011 | 0.002 | 1.55E-09 | -0.003 | 0.002 | 1.50E-01 |
| rs7727781 | 5 | 165185571 | T | C | 0.009 | 0.002 | 4.27E-08 | -0.001 | 0.002 | 6.40E-01 |
| rs2053682 | 5 | 170599327 | A | C | 0.017 | 0.002 | 2.59E-20 | 0.002 | 0.002 | 2.90E-01 |
| rs6556301 | 5 | 176527577 | T | G | -0.011 | 0.002 | 8.14E-11 | -0.001 | 0.002 | 6.00E-01 |
| rs2228213 | 6 | 12124855 | A | G | -0.014 | 0.002 | 5.50E-17 | -0.005 | 0.002 | 3.30E-02 |
| rs11757278 | 6 | 13180454 | T | C | 0.013 | 0.002 | 6.92E-13 | 0.003 | 0.002 | 1.40E-01 |
| rs3806114 | 6 | 20482335 | A | G | -0.012 | 0.002 | 1.46E-11 | -0.009 | 0.002 | 1.30E-04 |
| rs2066295 | 6 | 26168903 | A | G | 0.014 | 0.002 | 2.29E-12 | 0.017 | 0.003 | 3.50E-11 |
| rs3115667 | 6 | 31643399 | T | C | -0.018 | 0.002 | 5.92E-21 | -0.007 | 0.002 | 2.00E-03 |
| rs2281819 | 6 | 33771673 | A | T | -0.015 | 0.002 | 2.09E-14 | -0.010 | 0.003 | 7.40E-05 |
| rs2744974 | 6 | 34579431 | T | C | 0.026 | 0.002 | 1.28E-51 | 0.007 | 0.002 | 2.70E-03 |
| rs2436728 | 6 | 40365601 | A | G | 0.019 | 0.002 | 1.97E-29 | 0.012 | 0.002 | 5.70E-08 |
| rs1358980 | 6 | 43764551 | T | C | -0.013 | 0.002 | 5.15E-15 | -0.003 | 0.002 | 1.30E-01 |
| rs2206277 | 6 | 50798526 | T | C | 0.041 | 0.002 | 1.82E-83 | 0.001 | 0.003 | 6.00E-01 |
| rs1327259 | 6 | 51177811 | A | G | 0.016 | 0.002 | 1.47E-19 | 0.001 | 0.002 | 6.60E-01 |
| rs6915002 | 6 | 54028069 | T | C | 0.010 | 0.002 | 8.71E-09 | 0.001 | 0.002 | 5.90E-01 |
| rs9370410 | 6 | 55171842 | A | G | 0.011 | 0.002 | 2.58E-08 | 0.004 | 0.002 | 8.00E-02 |
| rs2622274 | 6 | 64240516 | T | G | -0.011 | 0.002 | 3.23E-10 | 0.000 | 0.002 | 9.60E-01 |
| rs1293037 | 6 | 70248345 | T | C | 0.013 | 0.002 | 5.73E-09 | 0.005 | 0.003 | 6.70E-02 |
| rs6921533 | 6 | 73742334 | T | C | 0.010 | 0.002 | 1.37E-08 | 0.002 | 0.002 | 4.10E-01 |
| rs9294260 | 6 | 83433228 | A | G | 0.014 | 0.002 | 8.16E-18 | -0.002 | 0.002 | 3.50E-01 |
| rs6909685 | 6 | 97753952 | T | C | -0.015 | 0.002 | 2.78E-16 | -0.001 | 0.002 | 5.80E-01 |
| rs9320823 | 6 | 98429337 | T | C | -0.017 | 0.002 | 2.07E-21 | -0.019 | 0.002 | 1.50E-17 |
| rs57989773 | 6 | 100629078 | T | C | -0.014 | 0.002 | 5.63E-10 | 0.018 | 0.003 | 3.50E-12 |
| rs156201 | 6 | 104847441 | C | G | 0.012 | 0.002 | 1.65E-10 | 0.002 | 0.003 | 4.50E-01 |
| rs768023 | 6 | 108876002 | A | G | 0.016 | 0.002 | 1.13E-22 | 0.005 | 0.002 | 2.00E-02 |
| rs2357760 | 6 | 120213880 | A | G | 0.014 | 0.002 | 2.11E-16 | 0.006 | 0.002 | 1.60E-02 |
| rs2875762 | 6 | 124925032 | C | G | 0.013 | 0.002 | 1.07E-10 | 0.008 | 0.003 | 1.80E-03 |
| rs1268065 | 6 | 126042783 | A | G | -0.010 | 0.002 | 7.12E-10 | 0.002 | 0.002 | 4.50E-01 |
| rs9375702 | 6 | 130384187 | T | C | -0.011 | 0.002 | 5.52E-09 | -0.001 | 0.002 | 7.80E-01 |
| rs2246012 | 6 | 131898208 | T | C | -0.016 | 0.002 | 1.15E-13 | 0.000 | 0.003 | 9.30E-01 |
| rs6922607 | 6 | 142703483 | A | G | -0.013 | 0.002 | 1.85E-09 | 0.001 | 0.003 | 7.40E-01 |
| rs765875 | 6 | 143185683 | T | C | -0.013 | 0.002 | 1.08E-14 | -0.006 | 0.002 | 5.20E-03 |
| rs7760482 | 6 | 147354276 | A | G | -0.010 | 0.002 | 6.25E-09 | -0.001 | 0.002 | 6.10E-01 |
| rs12527426 | 6 | 153392002 | A | G | 0.015 | 0.002 | 5.54E-16 | 0.009 | 0.002 | 2.00E-04 |
| rs9478496 | 6 | 154333183 | T | C | -0.016 | 0.002 | 8.17E-12 | -0.006 | 0.003 | 5.80E-02 |
| rs13191362 | 6 | 163033350 | A | G | 0.024 | 0.003 | 4.08E-21 | 0.001 | 0.003 | 7.40E-01 |
| rs9458814 | 6 | 163771305 | T | C | -0.011 | 0.002 | 2.33E-08 | -0.004 | 0.003 | 7.50E-02 |
| rs6950388 | 7 | 1270699 | A | G | 0.014 | 0.002 | 1.59E-09 | 0.006 | 0.003 | 2.70E-02 |
| rs4721089 | 7 | 1872921 | T | C | 0.017 | 0.002 | 5.60E-13 | 0.007 | 0.003 | 6.00E-03 |
| rs6463489 | 7 | 5542513 | T | C | 0.017 | 0.003 | 2.50E-10 | 0.004 | 0.003 | 3.10E-01 |
| rs4307239 | 7 | 24354300 | A | G | -0.012 | 0.002 | 1.47E-11 | -0.001 | 0.002 | 8.00E-01 |
| rs213518 | 7 | 26941065 | T | C | -0.015 | 0.002 | 2.49E-10 | 0.005 | 0.003 | 8.10E-02 |
| rs215669 | 7 | 32378979 | A | G | -0.015 | 0.002 | 8.94E-18 | -0.006 | 0.002 | 4.50E-03 |
| rs2108719 | 7 | 39481056 | A | G | 0.011 | 0.002 | 2.28E-08 | 0.003 | 0.002 | 2.00E-01 |
| rs217433 | 7 | 44553496 | T | C | -0.012 | 0.002 | 3.00E-08 | -0.002 | 0.003 | 4.20E-01 |
| rs2289379 | 7 | 44804225 | T | C | -0.014 | 0.002 | 7.02E-15 | -0.004 | 0.002 | 4.50E-02 |
| rs10499694 | 7 | 50614173 | A | G | 0.013 | 0.002 | 1.29E-15 | -0.003 | 0.002 | 1.30E-01 |
| rs11772246 | 7 | 71603692 | T | C | 0.015 | 0.002 | 3.69E-11 | 0.003 | 0.003 | 2.80E-01 |
| rs17207196 | 7 | 75101065 | T | C | -0.022 | 0.002 | 1.58E-36 | -0.009 | 0.002 | 2.30E-05 |
| rs17149254 | 7 | 76634463 | T | C | 0.024 | 0.002 | 2.99E-25 | -0.003 | 0.003 | 3.50E-01 |
| rs6973656 | 7 | 77422583 | A | G | -0.010 | 0.002 | 6.31E-09 | -0.013 | 0.002 | 1.00E-09 |
| rs1965529 | 7 | 77825707 | A | G | 0.016 | 0.002 | 7.66E-14 | 0.008 | 0.003 | 2.30E-03 |
| rs274628 | 7 | 86265855 | A | C | -0.010 | 0.002 | 1.36E-08 | -0.002 | 0.002 | 4.30E-01 |
| rs2283006 | 7 | 93085722 | A | G | 0.013 | 0.002 | 8.06E-15 | -0.002 | 0.002 | 2.80E-01 |
| rs13240600 | 7 | 99064466 | A | G | 0.018 | 0.002 | 7.90E-16 | 0.017 | 0.003 | 3.00E-08 |
| rs1721447 | 7 | 109214139 | T | G | -0.010 | 0.002 | 3.79E-09 | -0.008 | 0.002 | 1.00E-04 |
| rs2396625 | 7 | 113028634 | A | T | -0.018 | 0.002 | 2.81E-24 | -0.012 | 0.002 | 2.50E-08 |
| rs13245051 | 7 | 113362799 | A | G | 0.015 | 0.002 | 1.12E-18 | 0.001 | 0.002 | 8.10E-01 |
| rs10261050 | 7 | 114337652 | T | C | 0.011 | 0.002 | 4.53E-11 | 0.005 | 0.002 | 3.60E-02 |
| rs2283093 | 7 | 126721231 | T | C | 0.012 | 0.002 | 1.10E-08 | 0.003 | 0.003 | 3.10E-01 |
| rs7802342 | 7 | 137435925 | T | G | -0.012 | 0.002 | 6.23E-11 | 0.001 | 0.002 | 5.50E-01 |
| rs11525873 | 7 | 138817193 | T | C | 0.023 | 0.003 | 2.98E-13 | -0.003 | 0.004 | 4.70E-01 |
| rs11773362 | 7 | 147668180 | T | C | -0.011 | 0.002 | 6.39E-09 | 0.000 | 0.002 | 9.50E-01 |
| rs2907948 | 7 | 150638484 | A | G | -0.015 | 0.002 | 1.95E-14 | -0.004 | 0.003 | 7.40E-02 |
| rs56211164 | 7 | 158016764 | A | G | -0.013 | 0.002 | 8.90E-09 | 0.002 | 0.003 | 5.20E-01 |
| rs1700082 | 8 | 4121727 | C | G | 0.009 | 0.002 | 3.38E-08 | 0.002 | 0.002 | 2.90E-01 |
| rs4240673 | 8 | 10787612 | T | C | 0.018 | 0.002 | 1.58E-26 | -0.011 | 0.002 | 1.70E-07 |
| rs13263601 | 8 | 14095900 | A | C | -0.015 | 0.002 | 4.87E-16 | 0.006 | 0.002 | 1.20E-02 |
| rs10110189 | 8 | 15393380 | T | C | -0.016 | 0.003 | 2.20E-08 | 0.004 | 0.004 | 2.70E-01 |
| rs10101364 | 8 | 20634888 | T | C | 0.012 | 0.002 | 5.61E-11 | 0.004 | 0.002 | 9.20E-02 |
| rs73225274 | 8 | 21088909 | A | G | -0.016 | 0.003 | 2.33E-08 | -0.006 | 0.003 | 6.10E-02 |
| rs13249650 | 8 | 25664336 | A | T | 0.011 | 0.002 | 1.02E-08 | 0.005 | 0.002 | 2.10E-02 |
| rs1421334 | 8 | 30865733 | A | C | 0.014 | 0.002 | 3.11E-15 | 0.017 | 0.002 | 2.20E-15 |
| rs2466103 | 8 | 32412304 | T | G | -0.012 | 0.002 | 8.00E-12 | 0.001 | 0.002 | 8.10E-01 |
| rs6468266 | 8 | 34380276 | A | T | -0.011 | 0.002 | 7.71E-11 | -0.010 | 0.002 | 9.70E-06 |
| rs12681792 | 8 | 62054463 | A | C | 0.015 | 0.002 | 2.88E-12 | 0.007 | 0.003 | 1.50E-02 |
| rs12334877 | 8 | 67194171 | A | G | -0.014 | 0.002 | 2.17E-11 | -0.002 | 0.003 | 5.10E-01 |
| rs1808629 | 8 | 73435964 | A | G | -0.020 | 0.002 | 2.19E-23 | -0.009 | 0.002 | 1.80E-04 |
| rs17405819 | 8 | 76806584 | T | C | 0.021 | 0.002 | 6.04E-33 | -0.008 | 0.002 | 3.50E-04 |
| rs2196618 | 8 | 85089437 | A | G | -0.014 | 0.002 | 1.49E-12 | 0.006 | 0.002 | 2.30E-02 |
| rs12386885 | 8 | 87766769 | T | C | 0.015 | 0.003 | 9.90E-09 | 0.007 | 0.003 | 1.90E-02 |
| rs2120710 | 8 | 93210803 | A | G | 0.010 | 0.002 | 1.98E-08 | 0.009 | 0.002 | 1.40E-04 |
| rs12680842 | 8 | 95582606 | A | G | 0.014 | 0.002 | 3.41E-16 | 0.007 | 0.002 | 3.60E-03 |
| rs1383592 | 8 | 106430676 | A | G | 0.012 | 0.002 | 4.92E-09 | 0.005 | 0.003 | 6.40E-02 |
| rs3808477 | 8 | 116670347 | T | C | -0.018 | 0.002 | 8.73E-22 | -0.006 | 0.002 | 8.50E-03 |
| rs72673947 | 8 | 118884379 | A | G | -0.022 | 0.003 | 4.11E-13 | -0.012 | 0.004 | 6.90E-04 |
| rs6470144 | 8 | 124152245 | T | G | 0.010 | 0.002 | 3.06E-08 | 0.003 | 0.002 | 1.40E-01 |
| rs7842934 | 8 | 132838921 | T | C | -0.018 | 0.003 | 1.55E-08 | -0.001 | 0.004 | 8.80E-01 |
| rs305256 | 8 | 137568252 | T | C | -0.012 | 0.002 | 2.79E-08 | -0.010 | 0.003 | 6.70E-05 |
| rs16906838 | 8 | 138213836 | T | C | -0.025 | 0.004 | 4.31E-10 | 0.000 | 0.005 | 9.40E-01 |
| rs11782074 | 8 | 142617096 | T | G | 0.012 | 0.002 | 4.24E-12 | 0.009 | 0.002 | 6.90E-05 |
| rs10099330 | 8 | 143383694 | A | G | -0.012 | 0.002 | 3.22E-12 | -0.007 | 0.002 | 9.80E-04 |
| rs10975933 | 9 | 6954557 | C | G | 0.011 | 0.002 | 2.07E-10 | -0.005 | 0.002 | 4.70E-02 |
| rs1948080 | 9 | 11852043 | T | G | 0.014 | 0.002 | 1.13E-14 | 0.010 | 0.002 | 1.10E-05 |
| rs10961649 | 9 | 14670949 | T | C | 0.010 | 0.002 | 1.51E-08 | 0.006 | 0.002 | 8.20E-03 |
| rs4740619 | 9 | 15634326 | T | C | 0.019 | 0.002 | 3.15E-31 | 0.007 | 0.002 | 6.40E-04 |
| rs10962550 | 9 | 16720329 | C | G | 0.019 | 0.002 | 5.68E-17 | 0.005 | 0.003 | 1.10E-01 |
| rs10811868 | 9 | 23199959 | A | G | -0.010 | 0.002 | 3.18E-08 | 0.007 | 0.002 | 2.70E-03 |
| rs10968114 | 9 | 27800007 | A | C | 0.011 | 0.002 | 3.30E-11 | -0.001 | 0.002 | 6.80E-01 |
| rs1412235 | 9 | 28410996 | C | G | 0.024 | 0.002 | 2.28E-42 | 0.005 | 0.002 | 4.80E-02 |
| rs10121187 | 9 | 29737191 | C | G | 0.010 | 0.002 | 2.02E-09 | 0.006 | 0.002 | 6.20E-03 |
| rs2275003 | 9 | 34124860 | A | G | 0.011 | 0.002 | 9.44E-12 | -0.006 | 0.002 | 3.80E-03 |
| rs13296413 | 9 | 37258105 | T | C | -0.015 | 0.002 | 2.49E-17 | 0.006 | 0.002 | 5.10E-03 |
| rs2134858 | 9 | 73837155 | T | C | -0.012 | 0.002 | 5.87E-12 | -0.004 | 0.002 | 6.00E-02 |
| rs7861160 | 9 | 80799579 | T | C | 0.010 | 0.002 | 3.68E-08 | 0.002 | 0.002 | 3.10E-01 |
| rs1999433 | 9 | 81371441 | T | C | -0.011 | 0.002 | 2.90E-10 | 0.000 | 0.002 | 8.60E-01 |
| rs2777768 | 9 | 84186734 | A | G | 0.012 | 0.002 | 6.38E-10 | 0.002 | 0.002 | 3.10E-01 |
| rs7357754 | 9 | 92207308 | A | G | -0.012 | 0.002 | 1.81E-12 | -0.015 | 0.002 | 9.10E-12 |
| rs10992867 | 9 | 96461013 | A | G | 0.016 | 0.002 | 3.20E-17 | 0.006 | 0.002 | 1.90E-02 |
| rs7025938 | 9 | 103088321 | C | G | -0.016 | 0.002 | 1.47E-19 | -0.011 | 0.002 | 8.70E-07 |
| rs7024334 | 9 | 109072075 | T | G | 0.014 | 0.002 | 4.71E-12 | 0.000 | 0.003 | 8.90E-01 |
| rs1928295 | 9 | 120378483 | T | C | 0.013 | 0.002 | 2.23E-16 | 0.006 | 0.002 | 3.90E-03 |
| rs1877875 | 9 | 120664469 | T | C | -0.011 | 0.002 | 2.51E-10 | -0.002 | 0.002 | 3.10E-01 |
| rs13298487 | 9 | 126112104 | T | C | 0.012 | 0.002 | 1.43E-08 | 0.007 | 0.002 | 3.00E-03 |
| rs10733682 | 9 | 129460914 | A | G | 0.015 | 0.002 | 1.76E-19 | -0.002 | 0.002 | 3.80E-01 |
| rs2267958 | 9 | 131015279 | A | G | -0.013 | 0.002 | 2.01E-13 | 0.001 | 0.002 | 5.20E-01 |
| rs4740383 | 9 | 133783566 | A | G | 0.013 | 0.002 | 7.03E-14 | 0.003 | 0.002 | 1.70E-01 |
| rs10858334 | 9 | 137989785 | C | G | -0.015 | 0.003 | 4.91E-09 | 0.000 | 0.003 | 8.90E-01 |
| rs7907470 | 10 | 10268989 | A | G | -0.018 | 0.003 | 7.36E-09 | -0.013 | 0.004 | 1.10E-03 |
| rs7893571 | 10 | 16750129 | T | G | 0.013 | 0.002 | 5.83E-12 | 0.004 | 0.002 | 8.50E-02 |
| rs76638898 | 10 | 21099584 | A | G | -0.038 | 0.007 | 8.15E-09 | -0.003 | 0.007 | 6.90E-01 |
| rs7084454 | 10 | 21821274 | A | G | 0.020 | 0.002 | 4.51E-27 | 0.016 | 0.002 | 1.00E-11 |
| rs10829164 | 10 | 27318370 | T | C | 0.015 | 0.002 | 2.37E-10 | -0.005 | 0.003 | 9.10E-02 |
| rs4097319 | 10 | 33860515 | T | G | 0.011 | 0.002 | 4.32E-10 | 0.007 | 0.002 | 6.40E-04 |
| rs12765914 | 10 | 34013507 | T | C | 0.023 | 0.003 | 1.96E-13 | 0.006 | 0.004 | 1.30E-01 |
| rs1624134 | 10 | 34834482 | C | G | 0.010 | 0.002 | 6.51E-09 | 0.005 | 0.002 | 4.10E-02 |
| rs12259464 | 10 | 53680099 | A | G | 0.011 | 0.002 | 1.48E-10 | 0.007 | 0.002 | 1.40E-03 |
| rs7070670 | 10 | 61842645 | T | C | -0.013 | 0.002 | 7.14E-10 | -0.001 | 0.002 | 6.60E-01 |
| rs10761785 | 10 | 65318766 | T | G | -0.013 | 0.002 | 3.47E-16 | -0.008 | 0.002 | 1.70E-04 |
| rs12098284 | 10 | 76047464 | T | C | 0.018 | 0.003 | 9.87E-13 | 0.003 | 0.003 | 2.90E-01 |
| rs11001259 | 10 | 76807650 | A | T | -0.014 | 0.002 | 1.22E-08 | -0.008 | 0.003 | 4.10E-03 |
| rs7899106 | 10 | 87410904 | A | G | -0.033 | 0.004 | 1.72E-18 | -0.011 | 0.005 | 2.50E-02 |
| rs10788494 | 10 | 88110925 | C | G | 0.013 | 0.002 | 5.51E-15 | 0.006 | 0.002 | 9.60E-03 |
| rs147568678 | 10 | 93061851 | T | C | 0.013 | 0.002 | 3.42E-09 | 0.000 | 0.003 | 9.90E-01 |
| rs2439823 | 10 | 99778226 | A | G | -0.017 | 0.002 | 6.51E-22 | -0.007 | 0.002 | 1.30E-03 |
| rs17094222 | 10 | 102395440 | T | C | -0.017 | 0.002 | 4.04E-18 | 0.002 | 0.003 | 5.00E-01 |
| rs11594179 | 10 | 104392580 | T | C | -0.011 | 0.002 | 2.16E-08 | 0.001 | 0.003 | 6.80E-01 |
| rs79780963 | 10 | 104952499 | T | C | 0.024 | 0.004 | 2.63E-12 | 0.011 | 0.004 | 6.60E-03 |
| rs7903146 | 10 | 114758349 | T | C | -0.018 | 0.002 | 1.67E-23 | 0.000 | 0.002 | 9.00E-01 |
| rs2257791 | 10 | 118643670 | A | G | -0.014 | 0.002 | 1.63E-12 | -0.006 | 0.003 | 2.80E-02 |
| rs845084 | 10 | 125220036 | A | G | 0.014 | 0.002 | 3.22E-12 | 0.003 | 0.002 | 2.10E-01 |
| rs17636031 | 10 | 126594078 | T | C | -0.015 | 0.002 | 3.87E-17 | -0.003 | 0.002 | 2.70E-01 |
| rs4880341 | 10 | 133992689 | T | C | -0.013 | 0.002 | 3.06E-14 | 0.001 | 0.002 | 5.90E-01 |
| rs11246136 | 11 | 371265 | A | C | -0.017 | 0.003 | 2.03E-08 | 0.003 | 0.004 | 4.60E-01 |
| rs12421848 | 11 | 891338 | A | G | -0.014 | 0.002 | 3.99E-13 | -0.001 | 0.002 | 7.30E-01 |
| rs4256980 | 11 | 8673939 | C | G | -0.019 | 0.002 | 8.63E-29 | -0.003 | 0.002 | 2.20E-01 |
| rs900144 | 11 | 13294268 | T | C | 0.015 | 0.002 | 1.53E-18 | 0.006 | 0.002 | 3.40E-03 |
| rs2074314 | 11 | 17411821 | T | C | 0.011 | 0.002 | 1.37E-09 | 0.002 | 0.002 | 4.90E-01 |
| rs6265 | 11 | 27679916 | T | C | -0.041 | 0.002 | 7.40E-89 | 0.003 | 0.003 | 2.20E-01 |
| rs570463 | 11 | 28739318 | A | C | -0.012 | 0.002 | 4.33E-11 | -0.002 | 0.002 | 4.20E-01 |
| rs11030618 | 11 | 29243293 | T | C | 0.011 | 0.002 | 1.67E-10 | 0.002 | 0.002 | 3.40E-01 |
| rs2065418 | 11 | 30422068 | T | G | 0.014 | 0.002 | 6.07E-15 | 0.005 | 0.002 | 3.30E-02 |
| rs2862996 | 11 | 43653833 | T | G | -0.022 | 0.002 | 3.60E-35 | -0.004 | 0.002 | 9.40E-02 |
| rs10742752 | 11 | 45438374 | T | C | -0.012 | 0.002 | 1.20E-13 | 0.002 | 0.002 | 3.80E-01 |
| rs118081010 | 11 | 46174948 | T | C | 0.052 | 0.008 | 5.28E-12 | 0.019 | 0.009 | 3.00E-02 |
| rs7124681 | 11 | 47529947 | A | C | 0.026 | 0.002 | 3.96E-55 | 0.004 | 0.002 | 5.10E-02 |
| rs6591407 | 11 | 56914157 | A | C | -0.012 | 0.002 | 3.58E-09 | -0.005 | 0.003 | 6.40E-02 |
| rs562664 | 11 | 63823619 | T | C | -0.014 | 0.002 | 4.15E-10 | -0.003 | 0.003 | 2.80E-01 |
| rs7102454 | 11 | 65594820 | T | C | -0.017 | 0.002 | 3.84E-21 | -0.009 | 0.002 | 2.40E-05 |
| rs592483 | 11 | 69445173 | T | C | -0.014 | 0.002 | 1.53E-16 | -0.002 | 0.002 | 2.60E-01 |
| rs12282785 | 11 | 76476030 | A | C | -0.016 | 0.002 | 1.43E-11 | -0.011 | 0.003 | 3.10E-05 |
| rs349088 | 11 | 84814393 | A | C | -0.013 | 0.002 | 3.50E-14 | -0.002 | 0.002 | 3.00E-01 |
| rs10741329 | 11 | 89997796 | A | G | 0.012 | 0.002 | 3.91E-10 | 0.004 | 0.002 | 5.40E-02 |
| rs3019466 | 11 | 92476178 | T | C | -0.013 | 0.002 | 4.91E-08 | -0.011 | 0.003 | 1.90E-04 |
| rs2605603 | 11 | 93221105 | A | G | -0.010 | 0.002 | 2.04E-10 | -0.003 | 0.002 | 2.20E-01 |
| rs2513999 | 11 | 103019633 | A | G | -0.015 | 0.003 | 3.07E-09 | -0.001 | 0.003 | 6.50E-01 |
| rs12286929 | 11 | 115022404 | A | G | -0.018 | 0.002 | 1.93E-27 | -0.006 | 0.002 | 5.50E-03 |
| rs76942203 | 11 | 116973247 | A | G | 0.026 | 0.004 | 9.08E-11 | 0.017 | 0.005 | 3.10E-04 |
| rs3825061 | 11 | 118944675 | T | C | 0.014 | 0.002 | 6.15E-16 | 0.003 | 0.002 | 2.10E-01 |
| rs11218510 | 11 | 121922587 | A | G | -0.014 | 0.002 | 6.79E-13 | -0.001 | 0.002 | 5.90E-01 |
| rs35483388 | 11 | 122545146 | T | C | 0.013 | 0.002 | 2.61E-10 | 0.002 | 0.002 | 2.90E-01 |
| rs7944782 | 11 | 130795698 | T | G | -0.014 | 0.002 | 3.61E-17 | -0.003 | 0.002 | 2.40E-01 |
| rs2007518 | 11 | 132639606 | A | G | -0.013 | 0.002 | 1.96E-14 | -0.003 | 0.002 | 2.20E-01 |
| rs1941213 | 11 | 133125329 | A | C | 0.011 | 0.002 | 1.59E-08 | 0.007 | 0.002 | 3.80E-03 |
| rs329651 | 11 | 133767622 | T | G | 0.016 | 0.002 | 2.13E-14 | 0.011 | 0.003 | 1.10E-04 |
| rs12364470 | 11 | 134601012 | T | G | -0.019 | 0.002 | 2.18E-17 | -0.007 | 0.003 | 1.40E-02 |
| rs11611246 | 12 | 939480 | T | G | 0.022 | 0.002 | 2.04E-28 | -0.002 | 0.003 | 4.20E-01 |
| rs765125 | 12 | 2156207 | T | C | -0.010 | 0.002 | 1.27E-08 | -0.006 | 0.002 | 3.40E-03 |
| rs10772983 | 12 | 17141582 | T | C | -0.010 | 0.002 | 6.21E-10 | 0.000 | 0.002 | 8.70E-01 |
| rs11044430 | 12 | 19287416 | A | T | 0.016 | 0.002 | 3.77E-12 | -0.001 | 0.003 | 7.30E-01 |
| rs11046972 | 12 | 23705969 | T | C | 0.018 | 0.003 | 3.72E-08 | 0.009 | 0.004 | 2.90E-02 |
| rs10842240 | 12 | 24060075 | C | G | 0.020 | 0.003 | 1.21E-14 | 0.006 | 0.003 | 6.20E-02 |
| rs11170468 | 12 | 39430048 | A | C | 0.013 | 0.002 | 1.12E-11 | 0.003 | 0.003 | 2.70E-01 |
| rs1350430 | 12 | 41819215 | T | C | -0.013 | 0.002 | 2.08E-13 | 0.001 | 0.002 | 6.80E-01 |
| rs1126930 | 12 | 49399132 | C | G | 0.034 | 0.005 | 1.00E-10 | 0.003 | 0.006 | 6.10E-01 |
| rs7138803 | 12 | 50247468 | A | G | 0.030 | 0.002 | 3.10E-71 | 0.004 | 0.002 | 1.10E-01 |
| rs2271189 | 12 | 56494991 | A | G | -0.014 | 0.002 | 9.26E-16 | -0.010 | 0.002 | 2.30E-06 |
| rs7975187 | 12 | 60964108 | A | G | -0.014 | 0.002 | 3.86E-11 | -0.004 | 0.003 | 9.90E-02 |
| rs650198 | 12 | 69674595 | T | C | -0.014 | 0.002 | 6.43E-13 | 0.000 | 0.002 | 8.70E-01 |
| rs11115176 | 12 | 82465797 | T | C | 0.013 | 0.002 | 6.79E-12 | 0.003 | 0.003 | 3.00E-01 |
| rs10506971 | 12 | 89757937 | A | G | -0.014 | 0.002 | 4.69E-17 | -0.006 | 0.002 | 4.80E-03 |
| rs11105839 | 12 | 91237920 | A | T | -0.011 | 0.002 | 1.25E-11 | -0.002 | 0.002 | 3.70E-01 |
| rs2712665 | 12 | 99594947 | T | C | -0.011 | 0.002 | 5.40E-09 | -0.005 | 0.002 | 3.80E-02 |
| rs6539064 | 12 | 103706754 | C | G | 0.019 | 0.002 | 1.14E-23 | 0.001 | 0.002 | 7.80E-01 |
| rs1860561 | 12 | 110783241 | A | G | 0.016 | 0.002 | 1.71E-16 | 0.005 | 0.003 | 3.60E-02 |
| rs11066188 | 12 | 112610714 | A | G | -0.011 | 0.002 | 3.05E-12 | -0.002 | 0.002 | 3.30E-01 |
| rs2707183 | 12 | 116957607 | T | G | -0.009 | 0.002 | 4.89E-08 | -0.009 | 0.002 | 2.40E-05 |
| rs11615578 | 12 | 121714935 | T | C | 0.012 | 0.002 | 3.04E-09 | -0.001 | 0.002 | 5.90E-01 |
| rs12369179 | 12 | 122963550 | T | C | -0.034 | 0.003 | 2.32E-28 | 0.000 | 0.004 | 9.30E-01 |
| rs11060853 | 12 | 123424071 | A | G | -0.011 | 0.002 | 3.10E-08 | -0.007 | 0.002 | 1.30E-03 |
| rs11614340 | 12 | 133426483 | T | C | -0.012 | 0.002 | 1.87E-10 | 0.003 | 0.002 | 1.60E-01 |
| rs9512648 | 13 | 27933910 | A | G | 0.010 | 0.002 | 4.11E-08 | 0.005 | 0.002 | 3.60E-02 |
| rs7323 | 13 | 28009031 | C | G | -0.017 | 0.002 | 2.18E-18 | -0.002 | 0.002 | 4.90E-01 |
| rs4771218 | 13 | 28655311 | A | G | -0.014 | 0.002 | 3.18E-15 | 0.000 | 0.002 | 9.30E-01 |
| rs9595908 | 13 | 33184288 | T | C | 0.015 | 0.002 | 3.73E-20 | 0.008 | 0.002 | 2.20E-04 |
| rs9603697 | 13 | 40783323 | T | C | 0.013 | 0.002 | 1.69E-13 | 0.005 | 0.002 | 2.20E-02 |
| rs12429545 | 13 | 54102206 | A | G | 0.031 | 0.002 | 1.42E-37 | -0.003 | 0.003 | 3.40E-01 |
| rs962796 | 13 | 54385284 | T | C | 0.014 | 0.002 | 1.88E-11 | 0.003 | 0.003 | 3.50E-01 |
| rs9569777 | 13 | 58484786 | T | G | -0.020 | 0.002 | 3.43E-21 | -0.014 | 0.003 | 9.70E-07 |
| rs9527895 | 13 | 59367767 | T | C | -0.016 | 0.002 | 3.69E-12 | -0.005 | 0.003 | 8.40E-02 |
| rs8181823 | 13 | 65477940 | A | C | -0.013 | 0.002 | 4.36E-10 | 0.000 | 0.003 | 9.30E-01 |
| rs9599161 | 13 | 67434016 | T | C | 0.010 | 0.002 | 2.32E-09 | 0.004 | 0.002 | 8.10E-02 |
| rs1441264 | 13 | 79580919 | A | G | 0.017 | 0.002 | 7.60E-25 | -0.003 | 0.002 | 1.40E-01 |
| rs9531786 | 13 | 85983968 | C | G | -0.011 | 0.002 | 4.11E-10 | -0.005 | 0.002 | 4.30E-02 |
| rs77432547 | 13 | 86494817 | A | G | -0.017 | 0.002 | 1.44E-15 | -0.014 | 0.002 | 1.60E-08 |
| rs1927790 | 13 | 96922191 | T | C | -0.014 | 0.002 | 1.57E-17 | -0.005 | 0.002 | 3.20E-02 |
| rs9168 | 13 | 99101583 | A | C | -0.014 | 0.002 | 1.08E-13 | -0.006 | 0.002 | 1.80E-02 |
| rs9888533 | 13 | 107854612 | T | C | 0.013 | 0.002 | 1.38E-10 | 0.002 | 0.002 | 4.80E-01 |
| rs9522183 | 13 | 111977280 | T | G | -0.014 | 0.002 | 2.10E-12 | -0.005 | 0.002 | 1.80E-02 |
| rs12868881 | 13 | 112218924 | A | T | 0.014 | 0.002 | 1.40E-15 | 0.006 | 0.002 | 5.50E-03 |
| rs10132280 | 14 | 25928179 | A | C | -0.021 | 0.002 | 2.28E-33 | -0.001 | 0.002 | 5.90E-01 |
| rs4981693 | 14 | 29680331 | A | G | 0.020 | 0.002 | 7.89E-24 | 0.012 | 0.003 | 3.30E-06 |
| rs225882 | 14 | 30480123 | T | C | 0.011 | 0.002 | 8.56E-10 | 0.001 | 0.002 | 6.90E-01 |
| rs1958898 | 14 | 40886886 | C | G | -0.015 | 0.002 | 4.23E-12 | -0.004 | 0.003 | 1.60E-01 |
| rs61983990 | 14 | 41475003 | A | G | 0.020 | 0.004 | 1.91E-08 | 0.019 | 0.004 | 9.00E-07 |
| rs4900714 | 14 | 47302219 | T | G | 0.015 | 0.002 | 1.00E-18 | 0.002 | 0.002 | 3.20E-01 |
| rs217669 | 14 | 62360075 | T | C | -0.017 | 0.002 | 6.27E-16 | -0.010 | 0.002 | 6.00E-05 |
| rs4430672 | 14 | 63094407 | T | C | 0.012 | 0.002 | 5.00E-09 | 0.009 | 0.003 | 7.20E-04 |
| rs3902951 | 14 | 69789755 | T | G | -0.013 | 0.002 | 2.88E-12 | -0.008 | 0.003 | 1.30E-03 |
| rs17182027 | 14 | 73348130 | A | G | -0.011 | 0.002 | 1.46E-10 | -0.003 | 0.002 | 1.20E-01 |
| rs7144011 | 14 | 79940383 | T | G | 0.026 | 0.002 | 2.37E-40 | 0.000 | 0.003 | 9.40E-01 |
| rs4517716 | 14 | 83013876 | C | G | -0.012 | 0.002 | 4.27E-09 | -0.004 | 0.003 | 1.00E-01 |
| rs12888545 | 14 | 88308044 | A | G | -0.013 | 0.002 | 1.76E-11 | -0.004 | 0.002 | 9.10E-02 |
| rs1951455 | 14 | 91512339 | T | C | -0.015 | 0.002 | 6.05E-15 | 0.000 | 0.002 | 8.40E-01 |
| rs942066 | 14 | 94031914 | A | G | -0.020 | 0.002 | 2.36E-24 | -0.004 | 0.002 | 6.20E-02 |
| rs7161194 | 14 | 101529005 | A | G | 0.019 | 0.002 | 2.23E-24 | 0.000 | 0.002 | 8.60E-01 |
| rs10145749 | 14 | 102782109 | T | C | 0.016 | 0.003 | 8.33E-10 | 0.007 | 0.003 | 2.50E-02 |
| rs4906263 | 14 | 103249127 | C | G | -0.018 | 0.002 | 8.11E-23 | 0.001 | 0.002 | 7.60E-01 |
| rs2273175 | 14 | 104160141 | T | C | -0.012 | 0.002 | 3.69E-11 | 0.001 | 0.002 | 7.80E-01 |
| rs12594043 | 15 | 27034988 | C | G | 0.010 | 0.002 | 1.31E-09 | 0.002 | 0.002 | 2.90E-01 |
| rs7172627 | 15 | 31877690 | A | G | -0.011 | 0.002 | 1.81E-11 | -0.004 | 0.002 | 9.30E-02 |
| rs11636611 | 15 | 36391965 | T | C | 0.010 | 0.002 | 8.85E-10 | 0.008 | 0.002 | 3.10E-04 |
| rs9944219 | 15 | 46500612 | A | G | -0.012 | 0.002 | 8.14E-13 | 0.002 | 0.002 | 4.60E-01 |
| rs12912198 | 15 | 47103953 | T | C | -0.010 | 0.002 | 3.44E-08 | 0.000 | 0.002 | 9.20E-01 |
| rs6493498 | 15 | 51754451 | T | C | 0.014 | 0.002 | 4.84E-17 | 0.004 | 0.002 | 4.20E-02 |
| rs10518694 | 15 | 53072673 | A | C | 0.014 | 0.002 | 3.43E-09 | 0.012 | 0.003 | 8.90E-05 |
| rs12439632 | 15 | 59103963 | C | G | 0.015 | 0.002 | 3.68E-10 | 0.002 | 0.003 | 5.30E-01 |
| rs339991 | 15 | 60913637 | A | G | -0.013 | 0.002 | 3.51E-13 | -0.006 | 0.002 | 9.10E-03 |
| rs1559673 | 15 | 62156514 | A | C | 0.036 | 0.005 | 2.21E-13 | 0.006 | 0.007 | 3.90E-01 |
| rs2241423 | 15 | 68086838 | A | G | -0.030 | 0.002 | 3.60E-54 | -0.005 | 0.003 | 8.00E-02 |
| rs7171864 | 15 | 73227249 | A | G | 0.013 | 0.002 | 2.60E-14 | 0.007 | 0.002 | 1.20E-03 |
| rs2470893 | 15 | 75019449 | T | C | 0.011 | 0.002 | 9.43E-10 | 0.004 | 0.002 | 7.60E-02 |
| rs56151256 | 15 | 78024806 | A | C | 0.017 | 0.002 | 6.19E-14 | 0.016 | 0.002 | 4.30E-10 |
| rs12914623 | 15 | 80993570 | C | G | -0.016 | 0.002 | 2.00E-16 | -0.009 | 0.002 | 4.60E-04 |
| rs72757415 | 15 | 92572762 | T | G | -0.016 | 0.002 | 1.34E-11 | -0.011 | 0.003 | 3.40E-05 |
| rs11633626 | 15 | 95271378 | A | C | -0.016 | 0.002 | 7.27E-19 | -0.003 | 0.002 | 1.40E-01 |
| rs6496248 | 15 | 98262923 | A | T | 0.010 | 0.002 | 2.93E-09 | 0.004 | 0.002 | 5.70E-02 |
| rs12591120 | 15 | 99236869 | T | C | 0.012 | 0.002 | 3.84E-08 | 0.007 | 0.002 | 7.10E-03 |
| rs2715423 | 15 | 99511873 | A | G | -0.012 | 0.002 | 1.90E-09 | -0.003 | 0.002 | 2.50E-01 |
| rs214249 | 16 | 348687 | T | G | 0.014 | 0.002 | 2.72E-15 | 0.007 | 0.002 | 1.30E-03 |
| rs12448257 | 16 | 3599655 | A | G | 0.016 | 0.002 | 9.04E-16 | -0.004 | 0.003 | 1.20E-01 |
| rs879620 | 16 | 4015729 | T | C | 0.023 | 0.002 | 8.45E-39 | 0.006 | 0.002 | 1.20E-02 |
| rs2058527 | 16 | 6704749 | T | G | -0.012 | 0.002 | 1.80E-09 | 0.000 | 0.002 | 9.90E-01 |
| rs9926784 | 16 | 19941968 | T | C | 0.024 | 0.002 | 1.08E-30 | -0.002 | 0.003 | 5.10E-01 |
| rs194809 | 16 | 23804956 | A | G | 0.013 | 0.002 | 4.86E-09 | 0.001 | 0.003 | 6.80E-01 |
| rs2342892 | 16 | 24540806 | T | G | 0.013 | 0.002 | 1.33E-13 | 0.000 | 0.002 | 9.00E-01 |
| rs7498665 | 16 | 28883241 | A | G | -0.029 | 0.002 | 1.14E-66 | -0.014 | 0.002 | 4.10E-10 |
| rs3814883 | 16 | 29994922 | T | C | 0.023 | 0.002 | 1.47E-40 | -0.001 | 0.002 | 6.80E-01 |
| rs2193101 | 16 | 49035172 | C | G | 0.014 | 0.002 | 3.33E-11 | 0.003 | 0.003 | 3.00E-01 |
| rs16952482 | 16 | 53771583 | T | C | -0.015 | 0.003 | 3.06E-08 | 0.005 | 0.004 | 1.80E-01 |
| rs9937053 | 16 | 53799507 | A | G | 0.072 | 0.002 | 0.00E+00 | 0.001 | 0.002 | 8.00E-01 |
| rs8057911 | 16 | 54143352 | T | C | 0.013 | 0.002 | 3.66E-08 | 0.007 | 0.003 | 4.40E-03 |
| rs889398 | 16 | 69556715 | T | C | -0.020 | 0.002 | 3.23E-32 | -0.002 | 0.002 | 4.30E-01 |
| rs35949039 | 16 | 70572605 | T | G | -0.022 | 0.003 | 6.30E-12 | -0.008 | 0.004 | 3.20E-02 |
| rs12926250 | 16 | 72213316 | T | G | 0.018 | 0.003 | 1.78E-10 | 0.006 | 0.004 | 9.80E-02 |
| rs756717 | 16 | 72996162 | A | G | -0.013 | 0.002 | 2.38E-15 | -0.004 | 0.002 | 8.70E-02 |
| rs4390583 | 16 | 81694835 | A | C | -0.010 | 0.002 | 1.63E-08 | -0.001 | 0.002 | 7.70E-01 |
| rs12922346 | 16 | 82438337 | C | G | 0.013 | 0.002 | 1.48E-11 | 0.000 | 0.002 | 8.60E-01 |
| rs7206608 | 16 | 82872628 | C | G | -0.013 | 0.002 | 1.20E-12 | -0.010 | 0.002 | 3.40E-05 |
| rs3923783 | 17 | 1843189 | A | C | -0.022 | 0.002 | 4.12E-23 | -0.008 | 0.003 | 2.30E-03 |
| rs11078883 | 17 | 2138828 | C | G | -0.013 | 0.002 | 1.30E-12 | 0.001 | 0.002 | 7.80E-01 |
| rs7774 | 17 | 4801163 | A | C | 0.013 | 0.002 | 4.22E-10 | 0.003 | 0.002 | 1.60E-01 |
| rs1075901 | 17 | 15943910 | T | C | -0.012 | 0.002 | 4.43E-13 | 0.001 | 0.002 | 6.60E-01 |
| rs4986044 | 17 | 21261560 | T | C | -0.018 | 0.002 | 1.27E-27 | -0.009 | 0.002 | 1.80E-05 |
| rs8065172 | 17 | 31456969 | A | G | -0.012 | 0.002 | 5.29E-10 | -0.006 | 0.003 | 3.10E-02 |
| rs12150665 | 17 | 34914787 | T | C | 0.017 | 0.002 | 1.74E-24 | 0.006 | 0.002 | 4.50E-03 |
| rs6607337 | 17 | 35057373 | T | C | -0.012 | 0.002 | 2.55E-11 | -0.007 | 0.002 | 2.00E-03 |
| rs56161855 | 17 | 46288649 | A | T | -0.023 | 0.003 | 1.41E-16 | -0.007 | 0.003 | 2.00E-02 |
| rs9299 | 17 | 46669430 | T | C | 0.012 | 0.002 | 1.87E-11 | 0.002 | 0.002 | 2.90E-01 |
| rs11655587 | 17 | 47140794 | T | C | -0.021 | 0.002 | 6.87E-26 | -0.009 | 0.002 | 1.00E-04 |
| rs11649864 | 17 | 56093061 | A | G | 0.019 | 0.003 | 2.52E-10 | 0.002 | 0.004 | 5.80E-01 |
| rs12602912 | 17 | 65870073 | T | C | 0.017 | 0.002 | 2.90E-16 | 0.011 | 0.003 | 2.80E-05 |
| rs312750 | 17 | 68343539 | A | G | 0.010 | 0.002 | 2.57E-09 | 0.004 | 0.002 | 5.90E-02 |
| rs12939549 | 17 | 78611724 | A | G | 0.018 | 0.002 | 3.68E-28 | 0.001 | 0.002 | 5.80E-01 |
| rs35867081 | 17 | 79047278 | A | G | 0.015 | 0.002 | 6.65E-15 | -0.004 | 0.002 | 1.00E-01 |
| rs8097672 | 18 | 1839601 | A | T | -0.021 | 0.002 | 3.26E-18 | -0.007 | 0.003 | 2.50E-02 |
| rs478707 | 18 | 7543207 | T | C | -0.015 | 0.002 | 1.98E-10 | 0.005 | 0.003 | 6.60E-02 |
| rs891387 | 18 | 21103909 | T | C | 0.021 | 0.002 | 9.26E-35 | 0.013 | 0.002 | 3.00E-09 |
| rs1945160 | 18 | 22164216 | A | G | -0.010 | 0.002 | 5.65E-09 | -0.001 | 0.002 | 7.80E-01 |
| rs1941696 | 18 | 31252129 | A | G | 0.011 | 0.002 | 4.74E-11 | 0.000 | 0.002 | 8.60E-01 |
| rs474605 | 18 | 39612720 | A | G | -0.013 | 0.002 | 2.72E-13 | -0.003 | 0.002 | 1.70E-01 |
| rs1356506 | 18 | 40708038 | T | C | 0.014 | 0.002 | 8.39E-15 | 0.004 | 0.002 | 6.40E-02 |
| rs2612576 | 18 | 42967950 | A | T | -0.011 | 0.002 | 8.49E-09 | -0.004 | 0.002 | 1.10E-01 |
| rs9951619 | 18 | 56882326 | T | G | -0.015 | 0.002 | 2.33E-15 | 0.004 | 0.003 | 1.00E-01 |
| rs6567160 | 18 | 57829135 | T | C | -0.055 | 0.002 | 7.82E-184 | 0.006 | 0.003 | 2.50E-02 |
| rs17066856 | 18 | 58049656 | T | C | 0.035 | 0.003 | 7.10E-38 | 0.002 | 0.004 | 6.60E-01 |
| rs66595146 | 18 | 58204315 | A | C | 0.014 | 0.002 | 9.09E-12 | 0.007 | 0.002 | 8.70E-04 |
| rs17783165 | 18 | 63461638 | T | C | -0.013 | 0.002 | 2.74E-13 | -0.003 | 0.002 | 2.50E-01 |
| rs11150911 | 18 | 73498528 | A | C | 0.012 | 0.002 | 3.72E-11 | 0.001 | 0.002 | 7.10E-01 |
| rs12981256 | 19 | 1865901 | A | G | 0.015 | 0.002 | 1.43E-18 | 0.005 | 0.002 | 1.20E-02 |
| rs45486197 | 19 | 2244849 | A | G | 0.028 | 0.004 | 1.80E-12 | 0.001 | 0.004 | 9.00E-01 |
| rs895330 | 19 | 4060707 | C | G | 0.020 | 0.002 | 1.59E-19 | 0.006 | 0.003 | 1.80E-02 |
| rs273512 | 19 | 18224729 | T | C | 0.016 | 0.002 | 4.48E-19 | 0.010 | 0.002 | 4.10E-06 |
| rs17724992 | 19 | 18454825 | A | G | 0.017 | 0.002 | 5.23E-21 | 0.004 | 0.002 | 1.10E-01 |
| rs7258722 | 19 | 18808915 | A | T | -0.020 | 0.002 | 1.81E-30 | -0.004 | 0.002 | 5.80E-02 |
| rs12611148 | 19 | 19865077 | A | C | -0.014 | 0.002 | 1.42E-08 | -0.002 | 0.003 | 5.00E-01 |
| rs12462975 | 19 | 30272202 | A | G | 0.019 | 0.002 | 1.47E-25 | 0.003 | 0.002 | 2.40E-01 |
| rs7245985 | 19 | 30710410 | T | G | 0.012 | 0.002 | 1.88E-08 | 0.007 | 0.003 | 1.30E-02 |
| rs11882409 | 19 | 34019685 | A | C | 0.012 | 0.002 | 3.31E-10 | 0.001 | 0.002 | 6.80E-01 |
| rs185350 | 19 | 34306816 | T | C | 0.014 | 0.002 | 9.10E-17 | 0.004 | 0.002 | 4.40E-02 |
| rs429358 | 19 | 45411941 | T | C | 0.026 | 0.003 | 2.60E-22 | 0.014 | 0.003 | 3.80E-06 |
| rs11672660 | 19 | 46180184 | T | C | -0.034 | 0.002 | 6.83E-60 | -0.004 | 0.003 | 1.40E-01 |
| rs9304665 | 19 | 47602577 | A | T | 0.023 | 0.002 | 2.20E-31 | -0.006 | 0.003 | 1.90E-02 |
| rs3764625 | 19 | 49649051 | T | G | 0.010 | 0.002 | 2.42E-08 | -0.002 | 0.002 | 3.00E-01 |
| rs1884389 | 20 | 1410582 | T | C | -0.011 | 0.002 | 3.72E-10 | -0.001 | 0.002 | 5.80E-01 |
| rs4813619 | 20 | 2815715 | T | G | -0.011 | 0.002 | 1.70E-09 | -0.002 | 0.002 | 2.70E-01 |
| rs1884897 | 20 | 6612832 | A | G | -0.018 | 0.002 | 2.70E-28 | 0.000 | 0.002 | 8.80E-01 |
| rs2423668 | 20 | 12430673 | T | C | 0.011 | 0.002 | 7.84E-09 | 0.001 | 0.002 | 5.50E-01 |
| rs852056 | 20 | 17102860 | T | C | 0.012 | 0.002 | 2.15E-10 | 0.002 | 0.003 | 5.40E-01 |
| rs1409818 | 20 | 21381121 | T | C | 0.020 | 0.003 | 2.59E-12 | -0.008 | 0.004 | 2.60E-02 |
| rs8122855 | 20 | 25192049 | A | G | 0.014 | 0.002 | 4.11E-14 | 0.000 | 0.002 | 8.80E-01 |
| rs4812405 | 20 | 35276585 | A | C | -0.020 | 0.003 | 1.28E-09 | 0.006 | 0.004 | 1.20E-01 |
| rs742748 | 20 | 39293397 | T | C | -0.011 | 0.002 | 1.13E-11 | -0.001 | 0.002 | 8.20E-01 |
| rs79186842 | 20 | 47689036 | A | G | 0.020 | 0.003 | 2.60E-13 | 0.011 | 0.003 | 2.50E-04 |
| rs17806224 | 20 | 51065854 | A | G | -0.026 | 0.002 | 7.91E-32 | -0.009 | 0.003 | 7.70E-04 |
| rs1304549 | 20 | 54378256 | A | G | -0.012 | 0.002 | 1.35E-08 | 0.004 | 0.003 | 1.90E-01 |
| rs6010784 | 20 | 61540319 | T | C | 0.011 | 0.002 | 6.91E-11 | -0.008 | 0.002 | 1.30E-04 |
| rs6512302 | 20 | 62691550 | C | G | 0.013 | 0.002 | 1.52E-11 | 0.005 | 0.002 | 3.00E-02 |
| rs2064044 | 21 | 22119890 | A | C | -0.012 | 0.002 | 1.02E-08 | 0.005 | 0.003 | 5.50E-02 |
| rs2832283 | 21 | 30690558 | A | G | 0.012 | 0.002 | 4.72E-09 | 0.005 | 0.003 | 4.70E-02 |
| rs13047416 | 21 | 40309436 | C | G | 0.015 | 0.002 | 6.93E-18 | 0.002 | 0.002 | 3.80E-01 |
| rs8134638 | 21 | 40644170 | T | C | -0.013 | 0.002 | 1.57E-11 | -0.013 | 0.002 | 6.20E-09 |
| rs8126575 | 21 | 46435610 | T | G | 0.015 | 0.003 | 5.88E-10 | 0.002 | 0.003 | 5.10E-01 |
| rs427943 | 21 | 46570896 | A | C | -0.018 | 0.002 | 3.60E-25 | -0.009 | 0.002 | 3.70E-05 |
| rs11538 | 22 | 18220831 | A | G | -0.014 | 0.002 | 1.09E-09 | -0.004 | 0.003 | 1.20E-01 |
| rs2238799 | 22 | 20109325 | A | G | 0.010 | 0.002 | 8.65E-09 | 0.002 | 0.002 | 2.80E-01 |
| rs12628891 | 22 | 38317137 | T | C | -0.012 | 0.002 | 5.85E-10 | 0.003 | 0.002 | 2.70E-01 |
| rs12628051 | 22 | 40654276 | T | C | 0.016 | 0.002 | 2.90E-19 | -0.004 | 0.002 | 6.10E-02 |
| rs28489620 | 22 | 41804716 | A | G | -0.015 | 0.002 | 1.42E-12 | -0.012 | 0.002 | 1.30E-06 |
| rs9615337 | 22 | 48898559 | C | G | -0.011 | 0.002 | 2.05E-08 | -0.005 | 0.002 | 2.40E-02 |
| **2) MR of leisure television use on BMI (men and women combined)** | | | | | | | | | | |
| SNP | CHR | POS | EA | OA | beta_bmi | se_bmi | pval_bmi | beta_tv | se_tv | pval_tv |
| rs11810109 | 1 | 44186812 | A | T | 0.001 | 0.002 | 6.53E-01 | 0.016 | 0.002 | 5.40E-12 |
| rs112632642 | 1 | 51115634 | G | A | -0.016 | 0.004 | 4.21E-05 | -0.024 | 0.004 | 3.60E-08 |
| rs6670915 | 1 | 61109968 | C | T | 0.005 | 0.002 | 7.27E-03 | 0.014 | 0.002 | 2.40E-10 |
| rs12725114 | 1 | 62432641 | G | A | 0.012 | 0.002 | 3.32E-07 | 0.015 | 0.003 | 1.60E-08 |
| rs984409 | 1 | 67020440 | G | A | -0.005 | 0.002 | 1.05E-02 | -0.015 | 0.002 | 4.10E-11 |
| rs71658797 | 1 | 77967507 | T | A | -0.037 | 0.003 | 8.42E-36 | -0.02 | 0.003 | 6.30E-10 |
| rs114328297 | 1 | 91190854 | T | G | -0.001 | 0.002 | 6.96E-01 | 0.014 | 0.003 | 4.40E-08 |
| rs631130 | 1 | 96043010 | T | A | 0.002 | 0.002 | 2.31E-01 | -0.014 | 0.002 | 5.00E-09 |
| rs148648822 | 1 | 97815048 | G | T | -0.002 | 0.002 | 4.68E-01 | -0.018 | 0.003 | 4.20E-09 |
| rs17379561 | 1 | 98340139 | A | T | -0.018 | 0.002 | 5.04E-13 | -0.025 | 0.003 | 1.10E-16 |
| rs55909997 | 1 | 107581039 | G | A | -0.012 | 0.002 | 1.55E-11 | -0.014 | 0.002 | 2.40E-09 |
| rs1278847 | 1 | 110037483 | C | A | -0.011 | 0.002 | 9.45E-08 | 0.016 | 0.002 | 1.20E-11 |
| rs141425667 | 1 | 114930465 | G | A | -0.007 | 0.004 | 7.73E-02 | -0.024 | 0.004 | 4.00E-08 |
| rs4845364 | 1 | 154141908 | A | G | -0.001 | 0.002 | 6.38E-01 | -0.015 | 0.002 | 1.30E-12 |
| rs6673341 | 1 | 184655649 | T | G | -0.008 | 0.002 | 4.45E-06 | -0.015 | 0.002 | 2.20E-11 |
| rs72725224 | 1 | 188115858 | A | C | -0.017 | 0.007 | 1.71E-02 | -0.046 | 0.008 | 3.70E-09 |
| rs10737620 | 1 | 193057971 | T | A | 0.010 | 0.002 | 8.36E-08 | 0.014 | 0.002 | 2.60E-09 |
| rs11896615 | 2 | 17407744 | G | A | 0.005 | 0.003 | 5.78E-02 | 0.017 | 0.003 | 3.10E-08 |
| rs72781699 | 2 | 24277709 | G | A | 0.000 | 0.002 | 9.01E-01 | -0.019 | 0.003 | 3.00E-12 |
| rs12105701 | 2 | 41737200 | C | T | -0.010 | 0.002 | 3.12E-08 | -0.013 | 0.002 | 5.40E-09 |
| rs7564130 | 2 | 50606642 | T | C | -0.002 | 0.002 | 2.62E-01 | -0.015 | 0.002 | 2.80E-11 |
| rs11125515 | 2 | 53886799 | C | A | 0.004 | 0.002 | 1.98E-02 | -0.014 | 0.002 | 2.20E-08 |
| rs12475168 | 2 | 58992704 | G | T | -0.014 | 0.002 | 4.19E-09 | -0.018 | 0.003 | 1.70E-08 |
| rs183566425 | 2 | 59325356 | A | T | 0.024 | 0.008 | 1.22E-03 | 0.046 | 0.008 | 1.90E-08 |
| rs10189857 | 2 | 60713235 | A | G | -0.007 | 0.002 | 3.23E-05 | -0.02 | 0.002 | 6.20E-21 |
| rs62641636 | 2 | 68425427 | A | G | 0.006 | 0.002 | 5.59E-03 | 0.014 | 0.002 | 7.00E-10 |
| rs59210467 | 2 | 97097291 | C | T | 0.005 | 0.002 | 2.83E-02 | -0.013 | 0.002 | 1.00E-08 |
| rs11689199 | 2 | 100820421 | A | G | 0.017 | 0.002 | 1.85E-22 | 0.018 | 0.002 | 5.50E-17 |
| rs2916251 | 2 | 104447052 | G | T | -0.012 | 0.002 | 6.57E-13 | -0.017 | 0.002 | 1.50E-13 |
| rs1451533 | 2 | 105466005 | G | A | -0.016 | 0.002 | 3.03E-17 | -0.016 | 0.002 | 1.10E-10 |
| rs2971640 | 2 | 107624618 | G | A | -0.005 | 0.002 | 3.16E-03 | -0.013 | 0.002 | 4.20E-09 |
| rs12476388 | 2 | 116330983 | C | T | 0.002 | 0.002 | 2.37E-01 | 0.013 | 0.002 | 2.10E-08 |
| rs374722 | 2 | 147839830 | G | A | 0.014 | 0.002 | 9.78E-10 | 0.024 | 0.003 | 5.50E-16 |
| rs3754970 | 2 | 162091836 | T | C | -0.005 | 0.002 | 1.37E-02 | -0.015 | 0.002 | 4.80E-12 |
| rs1913808 | 2 | 162804678 | G | A | -0.001 | 0.002 | 6.94E-01 | 0.015 | 0.002 | 3.90E-10 |
| rs2060199 | 2 | 166196681 | T | A | 0.005 | 0.002 | 4.69E-03 | 0.012 | 0.002 | 2.40E-08 |
| rs263771 | 2 | 185921692 | C | A | 0.001 | 0.002 | 6.10E-01 | -0.017 | 0.003 | 3.10E-11 |
| rs4577309 | 2 | 191288833 | A | G | 0.002 | 0.002 | 2.74E-01 | 0.016 | 0.002 | 1.60E-13 |
| rs34224261 | 2 | 193855452 | G | C | -0.010 | 0.002 | 4.43E-07 | -0.018 | 0.002 | 4.60E-16 |
| rs4675246 | 2 | 202864487 | G | T | 0.002 | 0.002 | 4.43E-01 | -0.015 | 0.003 | 2.90E-08 |
| rs13029509 | 2 | 215374209 | G | A | -0.007 | 0.002 | 7.53E-05 | -0.018 | 0.002 | 2.00E-17 |
| rs10932837 | 2 | 221044785 | C | T | 0.002 | 0.002 | 2.98E-01 | -0.013 | 0.002 | 1.30E-09 |
| rs4973576 | 2 | 233806771 | C | A | 0.002 | 0.002 | 2.55E-01 | -0.015 | 0.002 | 1.00E-09 |
| rs138256022 | 3 | 11603090 | C | T | 0.002 | 0.005 | 6.40E-01 | -0.031 | 0.006 | 2.90E-08 |
| rs9834970 | 3 | 36856030 | T | C | 0.007 | 0.002 | 1.23E-04 | 0.013 | 0.002 | 3.30E-09 |
| rs1352890 | 3 | 49652156 | C | T | -0.006 | 0.002 | 8.16E-04 | -0.012 | 0.002 | 1.30E-08 |
| rs3796386 | 3 | 49899795 | G | A | -0.018 | 0.002 | 1.11E-25 | -0.026 | 0.002 | 3.20E-33 |
| rs182355396 | 3 | 50920457 | C | A | -0.005 | 0.011 | 6.34E-01 | -0.08 | 0.013 | 2.10E-10 |
| rs11917871 | 3 | 54233476 | T | C | -0.002 | 0.002 | 2.36E-01 | -0.017 | 0.002 | 4.50E-14 |
| rs11130793 | 3 | 60884659 | C | T | 0.008 | 0.002 | 4.42E-05 | 0.013 | 0.002 | 5.00E-09 |
| rs11714337 | 3 | 71582521 | G | A | 0.007 | 0.002 | 4.38E-04 | 0.014 | 0.002 | 4.70E-11 |
| rs12496084 | 3 | 75174777 | A | G | -0.002 | 0.002 | 2.37E-01 | -0.014 | 0.003 | 4.60E-08 |
| rs6797840 | 3 | 85656569 | A | C | -0.013 | 0.002 | 1.02E-11 | -0.016 | 0.002 | 1.70E-13 |
| rs56398417 | 3 | 88024986 | C | T | 0.014 | 0.002 | 1.31E-11 | 0.014 | 0.002 | 5.90E-10 |
| rs2034768 | 3 | 93986371 | A | G | 0.020 | 0.002 | 2.09E-25 | 0.015 | 0.002 | 8.60E-12 |
| rs12632778 | 3 | 106452359 | T | G | 0.001 | 0.004 | 8.22E-01 | -0.028 | 0.005 | 3.90E-08 |
| rs9867121 | 3 | 114631548 | C | A | 0.008 | 0.002 | 1.28E-03 | 0.019 | 0.003 | 3.90E-12 |
| rs12491503 | 3 | 165706855 | G | A | -0.004 | 0.002 | 3.48E-02 | -0.014 | 0.002 | 5.50E-10 |
| rs114600294 | 3 | 181419367 | G | C | -0.006 | 0.002 | 1.31E-02 | -0.016 | 0.003 | 7.90E-10 |
| rs66852340 | 4 | 3311070 | C | T | -0.009 | 0.002 | 9.47E-06 | -0.018 | 0.003 | 7.90E-12 |
| rs7700107 | 4 | 17880416 | A | C | -0.010 | 0.003 | 2.46E-05 | -0.021 | 0.003 | 1.90E-11 |
| rs34811474 | 4 | 25408838 | G | A | 0.029 | 0.002 | 8.50E-38 | 0.015 | 0.003 | 2.30E-09 |
| rs77215114 | 4 | 45187658 | A | T | 0.013 | 0.004 | 7.74E-04 | 0.024 | 0.004 | 1.50E-08 |
| rs28971796 | 4 | 49151982 | A | G | 0.011 | 0.002 | 1.02E-07 | 0.015 | 0.002 | 2.10E-09 |
| rs6850494 | 4 | 82291771 | A | C | 0.001 | 0.002 | 5.16E-01 | -0.014 | 0.002 | 1.10E-10 |
| rs13107325 | 4 | 103188709 | C | T | -0.047 | 0.003 | 3.81E-47 | -0.029 | 0.004 | 1.50E-12 |
| rs62332760 | 4 | 106135316 | G | T | 0.002 | 0.002 | 2.54E-01 | -0.014 | 0.002 | 8.00E-11 |
| rs7693703 | 4 | 118357405 | G | A | 0.005 | 0.003 | 9.29E-02 | 0.023 | 0.004 | 2.70E-09 |
| rs4334769 | 4 | 130245233 | G | T | 0.002 | 0.002 | 2.03E-01 | 0.012 | 0.002 | 2.10E-08 |
| rs6825241 | 4 | 152543783 | C | A | -0.004 | 0.002 | 3.23E-02 | -0.017 | 0.002 | 4.90E-15 |
| rs7693082 | 4 | 159857819 | G | C | 0.008 | 0.002 | 3.20E-04 | 0.015 | 0.002 | 1.70E-10 |
| rs10940659 | 5 | 59360341 | A | G | -0.007 | 0.002 | 5.49E-05 | -0.013 | 0.002 | 1.50E-08 |
| rs2409838 | 5 | 60204410 | A | T | -0.004 | 0.002 | 2.03E-02 | 0.017 | 0.002 | 3.40E-15 |
| rs262890 | 5 | 62930015 | A | G | -0.014 | 0.002 | 2.95E-11 | -0.019 | 0.002 | 3.20E-15 |
| rs7716447 | 5 | 88800331 | A | G | -0.015 | 0.002 | 8.41E-17 | -0.013 | 0.002 | 3.60E-09 |
| rs1031423 | 5 | 93276883 | T | C | 0.004 | 0.002 | 9.21E-02 | -0.019 | 0.003 | 1.80E-12 |
| rs57585211 | 5 | 107197725 | T | G | -0.005 | 0.003 | 7.14E-02 | -0.017 | 0.003 | 4.30E-09 |
| rs9326862 | 5 | 112054547 | A | T | 0.004 | 0.002 | 2.09E-02 | 0.012 | 0.002 | 2.60E-08 |
| rs10041724 | 5 | 124273520 | T | C | 0.006 | 0.002 | 2.64E-03 | 0.018 | 0.003 | 3.90E-11 |
| rs10057545 | 5 | 138431008 | G | A | -0.005 | 0.002 | 2.07E-02 | -0.014 | 0.002 | 2.00E-10 |
| rs62379379 | 5 | 141082015 | G | T | -0.005 | 0.004 | 1.61E-01 | -0.026 | 0.004 | 7.80E-10 |
| rs10054327 | 5 | 147871137 | G | A | 0.003 | 0.002 | 1.74E-01 | 0.017 | 0.002 | 3.40E-15 |
| rs42210 | 5 | 166408788 | G | C | -0.003 | 0.002 | 1.02E-01 | -0.014 | 0.002 | 7.30E-09 |
| rs2336895 | 5 | 166990695 | G | A | 0.004 | 0.002 | 6.56E-02 | 0.012 | 0.002 | 4.60E-08 |
| rs72828890 | 5 | 167806836 | C | T | 0.011 | 0.003 | 4.31E-04 | 0.019 | 0.003 | 4.90E-09 |
| rs255292 | 5 | 172580866 | C | A | -0.001 | 0.002 | 7.45E-01 | -0.012 | 0.002 | 1.70E-08 |
| rs72834698 | 6 | 26176517 | G | A | 0.015 | 0.003 | 3.33E-08 | 0.023 | 0.003 | 2.70E-13 |
| rs3135044 | 6 | 31592820 | C | T | -0.009 | 0.002 | 4.16E-06 | -0.015 | 0.002 | 8.70E-11 |
| rs10947452 | 6 | 33803752 | T | C | 0.012 | 0.002 | 2.99E-09 | 0.014 | 0.002 | 6.40E-10 |
| rs9471333 | 6 | 40362023 | C | T | 0.021 | 0.002 | 6.90E-29 | 0.013 | 0.002 | 1.50E-09 |
| rs4523073 | 6 | 67550288 | A | G | -0.003 | 0.002 | 1.75E-01 | -0.014 | 0.002 | 5.00E-10 |
| rs6905544 | 6 | 98411631 | A | G | -0.017 | 0.002 | 2.59E-21 | -0.019 | 0.002 | 8.50E-18 |
| rs17789218 | 6 | 100600097 | T | C | -0.011 | 0.002 | 4.78E-09 | 0.019 | 0.003 | 1.40E-13 |
| rs78394231 | 6 | 107649123 | T | C | -0.005 | 0.003 | 1.49E-01 | -0.021 | 0.004 | 9.70E-09 |
| rs2184364 | 6 | 143183596 | A | G | 0.010 | 0.002 | 3.38E-06 | 0.016 | 0.003 | 3.00E-09 |
| rs9718104 | 6 | 166170539 | T | G | 0.000 | 0.004 | 9.54E-01 | -0.041 | 0.005 | 9.30E-19 |
| rs17568389 | 7 | 8091876 | T | A | 0.003 | 0.002 | 5.75E-02 | 0.015 | 0.002 | 8.60E-13 |
| rs10234444 | 7 | 41828527 | G | A | 0.005 | 0.002 | 1.80E-02 | 0.016 | 0.003 | 2.90E-08 |
| rs6973656 | 7 | 77422583 | A | G | -0.010 | 0.002 | 6.31E-09 | -0.013 | 0.002 | 1.00E-09 |
| rs973734 | 7 | 89387578 | C | A | 0.004 | 0.002 | 1.16E-01 | 0.017 | 0.003 | 1.50E-08 |
| rs3137 | 7 | 99130875 | G | A | 0.017 | 0.002 | 2.61E-15 | 0.017 | 0.003 | 1.40E-08 |
| rs2694151 | 7 | 101902180 | C | T | 0.007 | 0.004 | 6.38E-02 | 0.026 | 0.005 | 2.30E-08 |
| rs62471080 | 7 | 109128060 | G | C | -0.009 | 0.002 | 4.18E-06 | -0.013 | 0.002 | 3.70E-09 |
| rs7788008 | 7 | 112972483 | G | A | 0.017 | 0.002 | 3.79E-23 | 0.013 | 0.002 | 5.90E-09 |
| rs10246289 | 7 | 115544763 | A | G | 0.005 | 0.003 | 7.85E-02 | 0.019 | 0.003 | 1.70E-08 |
| rs11763734 | 7 | 126400910 | A | C | -0.003 | 0.002 | 1.35E-01 | -0.012 | 0.002 | 8.20E-09 |
| rs7834121 | 8 | 10770255 | G | T | 0.017 | 0.002 | 8.18E-19 | -0.014 | 0.002 | 1.10E-10 |
| rs1421334 | 8 | 30865733 | A | C | 0.014 | 0.002 | 3.11E-15 | 0.017 | 0.002 | 2.20E-15 |
| rs6996198 | 8 | 65463442 | C | T | -0.007 | 0.002 | 5.33E-03 | -0.016 | 0.003 | 3.50E-08 |
| rs6472942 | 8 | 76827190 | T | C | 0.017 | 0.002 | 3.49E-22 | -0.013 | 0.002 | 1.80E-09 |
| rs11985394 | 8 | 92667521 | T | A | 0.001 | 0.002 | 4.61E-01 | 0.012 | 0.002 | 1.30E-08 |
| rs72671494 | 8 | 93195457 | T | C | -0.010 | 0.003 | 2.60E-04 | -0.017 | 0.003 | 4.50E-08 |
| rs12541615 | 8 | 118870151 | T | C | -0.015 | 0.003 | 4.97E-09 | -0.018 | 0.003 | 3.90E-10 |
| rs2717559 | 8 | 143882420 | A | G | 0.004 | 0.002 | 4.06E-02 | 0.012 | 0.002 | 1.50E-08 |
| rs2616830 | 9 | 1721385 | G | A | 0.001 | 0.002 | 6.54E-01 | 0.016 | 0.002 | 2.90E-14 |
| rs2291681 | 9 | 14790841 | G | T | 0.004 | 0.002 | 2.94E-02 | 0.013 | 0.002 | 7.90E-09 |
| rs34864022 | 9 | 22609110 | A | G | 0.000 | 0.004 | 9.91E-01 | -0.026 | 0.004 | 1.40E-09 |
| rs12554512 | 9 | 23352293 | T | C | -0.010 | 0.002 | 1.67E-07 | 0.021 | 0.002 | 3.80E-21 |
| rs2791574 | 9 | 82212094 | T | G | -0.004 | 0.002 | 2.17E-02 | -0.013 | 0.002 | 3.00E-08 |
| rs870151 | 9 | 92230747 | T | A | -0.011 | 0.002 | 1.14E-09 | -0.016 | 0.002 | 8.80E-13 |
| rs2787374 | 9 | 103054951 | T | C | 0.011 | 0.002 | 1.36E-10 | 0.012 | 0.002 | 1.20E-08 |
| rs10739499 | 9 | 120514261 | C | G | 0.005 | 0.002 | 3.72E-03 | 0.014 | 0.002 | 1.50E-09 |
| rs7043521 | 9 | 124602779 | A | T | 0.009 | 0.002 | 1.09E-06 | 0.014 | 0.002 | 6.50E-11 |
| rs12342024 | 9 | 126134232 | G | T | -0.007 | 0.003 | 2.44E-02 | -0.021 | 0.003 | 1.70E-09 |
| rs4382592 | 9 | 134870755 | T | G | 0.009 | 0.002 | 1.81E-06 | 0.014 | 0.002 | 6.70E-09 |
| rs2073869 | 9 | 135763816 | C | T | 0.007 | 0.002 | 1.80E-03 | 0.019 | 0.003 | 1.50E-10 |
| 9:139926402_T_C | 9 | 139926402 | T | C | 0.001 | 0.002 | 6.77E-01 | -0.013 | 0.002 | 2.00E-08 |
| rs1623025 | 10 | 11164813 | G | C | -0.004 | 0.002 | 3.50E-02 | -0.012 | 0.002 | 1.40E-08 |
| rs1243182 | 10 | 21916728 | C | T | -0.017 | 0.002 | 4.11E-21 | -0.019 | 0.002 | 2.00E-15 |
| rs2045147 | 10 | 56660437 | A | G | 0.005 | 0.002 | 1.82E-03 | 0.013 | 0.002 | 5.90E-09 |
| rs10994943 | 10 | 63591413 | T | G | 0.003 | 0.002 | 7.68E-02 | 0.013 | 0.002 | 3.10E-09 |
| rs11201422 | 10 | 86924483 | T | C | 0.008 | 0.002 | 5.59E-06 | 0.013 | 0.002 | 3.40E-08 |
| rs10786658 | 10 | 103737193 | A | T | 0.000 | 0.002 | 8.76E-01 | -0.014 | 0.002 | 4.60E-11 |
| rs7089973 | 10 | 116569565 | C | A | 0.000 | 0.002 | 8.68E-01 | -0.013 | 0.002 | 2.40E-09 |
| rs11245482 | 10 | 126733546 | T | C | -0.010 | 0.002 | 5.11E-08 | -0.013 | 0.002 | 2.60E-09 |
| rs17727474 | 10 | 127182959 | C | T | 0.000 | 0.002 | 9.42E-01 | 0.018 | 0.003 | 3.10E-09 |
| rs12289262 | 11 | 12894758 | C | T | -0.007 | 0.002 | 3.82E-04 | -0.014 | 0.002 | 2.00E-08 |
| rs801733 | 11 | 65934549 | A | C | 0.011 | 0.002 | 1.49E-10 | 0.017 | 0.002 | 7.30E-14 |
| rs116175338 | 11 | 72467875 | G | T | -0.012 | 0.002 | 2.92E-10 | -0.014 | 0.003 | 1.40E-08 |
| rs12272012 | 11 | 84861286 | G | C | -0.009 | 0.005 | 6.78E-02 | -0.03 | 0.005 | 1.90E-08 |
| rs11020045 | 11 | 92487907 | A | C | -0.001 | 0.002 | 7.82E-01 | -0.013 | 0.002 | 1.60E-08 |
| rs17207890 | 11 | 95490754 | G | A | 0.001 | 0.002 | 4.81E-01 | 0.016 | 0.002 | 6.70E-12 |
| rs10791546 | 11 | 101830126 | C | T | 0.008 | 0.002 | 9.38E-05 | 0.012 | 0.002 | 4.10E-08 |
| rs328277 | 11 | 110414195 | C | A | -0.002 | 0.002 | 2.61E-01 | 0.012 | 0.002 | 4.50E-08 |
| rs648044 | 11 | 114030799 | A | G | -0.009 | 0.002 | 6.63E-06 | -0.014 | 0.002 | 1.00E-09 |
| rs11218575 | 11 | 122154576 | C | T | 0.003 | 0.002 | 9.06E-02 | 0.015 | 0.002 | 2.10E-12 |
| rs74802478 | 11 | 131966907 | G | A | 0.011 | 0.003 | 1.40E-05 | 0.017 | 0.003 | 3.50E-09 |
| rs4937842 | 11 | 133734542 | G | C | -0.008 | 0.002 | 9.11E-06 | -0.013 | 0.002 | 1.40E-08 |
| rs10772643 | 12 | 13415288 | C | T | 0.010 | 0.003 | 1.35E-03 | 0.025 | 0.003 | 1.30E-12 |
| rs74996610 | 12 | 24075007 | C | G | 0.000 | 0.005 | 9.55E-01 | -0.031 | 0.005 | 4.10E-09 |
| rs10771746 | 12 | 30791864 | C | T | -0.002 | 0.002 | 3.44E-01 | -0.014 | 0.002 | 2.50E-09 |
| rs10876864 | 12 | 56401085 | G | A | -0.011 | 0.002 | 1.70E-10 | -0.013 | 0.002 | 1.00E-09 |
| rs8756 | 12 | 66359752 | C | A | 0.005 | 0.002 | 5.18E-03 | -0.013 | 0.002 | 5.30E-10 |
| rs11179838 | 12 | 74321123 | C | G | 0.005 | 0.002 | 1.09E-02 | 0.013 | 0.002 | 2.10E-08 |
| rs2164744 | 12 | 109872039 | T | C | -0.005 | 0.002 | 1.05E-02 | -0.013 | 0.002 | 1.10E-08 |
| rs2173650 | 12 | 117525235 | G | T | 0.004 | 0.003 | 1.44E-01 | 0.018 | 0.003 | 4.60E-09 |
| rs1790135 | 12 | 123669235 | C | T | -0.002 | 0.002 | 2.99E-01 | -0.014 | 0.003 | 2.60E-08 |
| rs9563168 | 13 | 54247827 | G | A | 0.008 | 0.002 | 5.86E-05 | 0.018 | 0.003 | 4.30E-11 |
| rs9569734 | 13 | 58319476 | A | G | 0.018 | 0.002 | 3.82E-14 | 0.019 | 0.003 | 3.40E-10 |
| rs9528023 | 13 | 60226991 | C | T | 0.005 | 0.002 | 1.26E-02 | 0.013 | 0.002 | 3.80E-08 |
| rs56858768 | 13 | 86511730 | G | A | -0.017 | 0.002 | 5.13E-15 | -0.015 | 0.002 | 3.40E-10 |
| rs553397860 | 13 | 99051118 | A | G | 0.010 | 0.002 | 4.18E-05 | 0.017 | 0.003 | 8.90E-09 |
| rs7991062 | 13 | 100713194 | C | G | -0.001 | 0.002 | 4.84E-01 | -0.018 | 0.002 | 8.80E-15 |
| rs11629120 | 14 | 23458457 | T | C | 0.000 | 0.002 | 9.67E-01 | -0.013 | 0.002 | 1.10E-08 |
| rs405797 | 14 | 26972834 | T | A | -0.007 | 0.002 | 6.68E-04 | -0.015 | 0.003 | 2.80E-09 |
| rs1022785 | 14 | 29571653 | G | A | 0.008 | 0.002 | 9.68E-04 | 0.018 | 0.003 | 7.10E-09 |
| rs10145592 | 14 | 94287215 | C | G | -0.006 | 0.002 | 3.14E-04 | -0.015 | 0.002 | 1.80E-11 |
| rs7157001 | 14 | 99749484 | A | G | -0.007 | 0.002 | 2.72E-03 | -0.014 | 0.002 | 2.30E-08 |
| rs8043253 | 15 | 41503730 | C | T | -0.005 | 0.002 | 8.17E-03 | -0.012 | 0.002 | 3.40E-08 |
| rs2460 | 15 | 53073084 | G | A | -0.011 | 0.002 | 4.05E-09 | -0.015 | 0.002 | 5.00E-10 |
| rs4775373 | 15 | 61476936 | T | C | 0.005 | 0.002 | 1.10E-02 | 0.013 | 0.002 | 4.70E-09 |
| rs61331678 | 15 | 77995949 | G | C | 0.009 | 0.002 | 4.87E-06 | 0.015 | 0.002 | 6.10E-12 |
| rs35574015 | 16 | 15146061 | T | C | 0.008 | 0.002 | 7.78E-05 | -0.013 | 0.002 | 3.00E-08 |
| rs7189927 | 16 | 28913787 | T | C | 0.026 | 0.002 | 1.13E-51 | 0.015 | 0.002 | 3.40E-11 |
| rs749671 | 16 | 31088347 | G | A | 0.021 | 0.002 | 2.03E-35 | 0.016 | 0.002 | 2.70E-12 |
| rs7184800 | 16 | 53509131 | G | A | -0.006 | 0.002 | 3.56E-03 | 0.017 | 0.002 | 8.20E-13 |
| rs2447098 | 17 | 2277720 | C | A | -0.001 | 0.002 | 6.77E-01 | -0.015 | 0.002 | 6.80E-12 |
| rs11654952 | 17 | 4813799 | T | G | -0.004 | 0.003 | 1.22E-01 | -0.017 | 0.003 | 4.30E-08 |
| rs9902312 | 17 | 65070304 | T | C | 0.001 | 0.002 | 6.21E-01 | 0.015 | 0.002 | 4.50E-11 |
| rs11657730 | 17 | 79372489 | C | T | 0.003 | 0.002 | 2.17E-01 | 0.013 | 0.002 | 1.10E-08 |
| rs303753 | 18 | 21074922 | G | A | -0.020 | 0.002 | 1.84E-27 | -0.014 | 0.002 | 2.70E-10 |
| rs11081851 | 18 | 31783262 | G | A | -0.004 | 0.002 | 2.51E-02 | -0.013 | 0.002 | 1.00E-08 |
| rs9964724 | 18 | 35159124 | C | T | 0.000 | 0.002 | 9.88E-01 | 0.018 | 0.002 | 3.30E-14 |
| rs1156541 | 18 | 39952989 | C | T | 0.011 | 0.002 | 4.90E-08 | 0.014 | 0.003 | 2.80E-08 |
| rs148544378 | 18 | 40323567 | C | T | -0.015 | 0.007 | 3.38E-02 | 0.053 | 0.008 | 2.20E-12 |
| rs17512836 | 18 | 53194961 | T | C | 0.016 | 0.006 | 8.62E-03 | 0.042 | 0.007 | 7.00E-10 |
| rs7248205 | 19 | 10770305 | C | T | 0.008 | 0.002 | 3.95E-05 | 0.014 | 0.002 | 3.40E-10 |
| rs111901094 | 19 | 19513570 | G | T | -0.009 | 0.003 | 2.96E-04 | -0.017 | 0.003 | 2.00E-09 |
| rs6131281 | 20 | 11891724 | C | T | 0.007 | 0.002 | 5.17E-04 | 0.016 | 0.002 | 3.10E-13 |
| rs6141814 | 20 | 31368960 | C | A | 0.002 | 0.002 | 3.74E-01 | -0.014 | 0.002 | 1.30E-09 |
| rs4810312 | 20 | 39968707 | C | G | -0.001 | 0.002 | 7.67E-01 | -0.013 | 0.002 | 1.20E-08 |
| rs6124476 | 20 | 41202830 | T | C | 0.007 | 0.002 | 2.09E-05 | 0.012 | 0.002 | 1.80E-08 |
| rs6031440 | 20 | 42809317 | A | T | 0.003 | 0.002 | 6.20E-02 | 0.012 | 0.002 | 1.80E-08 |
| rs55700114 | 20 | 43717080 | G | A | -0.005 | 0.002 | 1.00E-02 | -0.014 | 0.002 | 2.10E-09 |
| rs6125906 | 20 | 48729803 | C | A | 0.001 | 0.003 | 7.93E-01 | -0.021 | 0.004 | 3.90E-08 |
| rs56103247 | 20 | 62483184 | C | T | 0.023 | 0.004 | 1.18E-07 | 0.03 | 0.005 | 3.80E-10 |
| rs10427502 | 21 | 40654840 | G | A | 0.012 | 0.002 | 3.56E-09 | 0.014 | 0.002 | 8.60E-10 |
| rs7609 | 22 | 29951650 | A | T | 0.001 | 0.002 | 4.87E-01 | 0.015 | 0.003 | 3.60E-09 |
| rs2092829 | 22 | 31780514 | G | A | -0.005 | 0.002 | 5.75E-03 | 0.014 | 0.002 | 1.30E-08 |
| **3) MR of BMI (men) on leisure television use** | | | | | | | | | | |
| SNP | CHR | POS | EA | OA | beta_bmi | se_bmi | pval_bmi | beta_tv | se_tv | pval_tv |
| rs2803316 | 1 | 1865298 | A | G | -0.015 | 0.003 | 4.03E-09 | -0.006 | 0.002 | 6.20E-03 |
| rs3762444 | 1 | 2427712 | T | C | -0.019 | 0.003 | 1.47E-14 | -0.001 | 0.002 | 5.90E-01 |
| rs11121210 | 1 | 8708529 | T | C | -0.015 | 0.003 | 7.86E-09 | -0.003 | 0.002 | 2.40E-01 |
| rs11581010 | 1 | 11212458 | A | G | -0.018 | 0.003 | 1.01E-09 | 0.001 | 0.003 | 8.10E-01 |
| rs2235549 | 1 | 23391775 | T | G | -0.021 | 0.003 | 8.27E-11 | 0.000 | 0.003 | 9.80E-01 |
| rs2271928 | 1 | 32127953 | A | G | -0.014 | 0.002 | 9.92E-09 | 0.002 | 0.002 | 2.70E-01 |
| rs12061406 | 1 | 40073479 | T | C | -0.027 | 0.005 | 1.70E-09 | 0.000 | 0.004 | 9.20E-01 |
| rs1768808 | 1 | 46503217 | T | C | 0.014 | 0.003 | 3.54E-08 | 0.004 | 0.002 | 5.00E-02 |
| rs977747 | 1 | 47684677 | T | G | 0.016 | 0.002 | 1.35E-11 | 0.001 | 0.002 | 5.60E-01 |
| rs7531656 | 1 | 49828663 | A | G | 0.020 | 0.003 | 2.32E-15 | -0.001 | 0.002 | 5.80E-01 |
| rs2481665 | 1 | 62594677 | T | C | 0.019 | 0.002 | 2.03E-15 | 0.004 | 0.002 | 8.40E-02 |
| rs11208779 | 1 | 66417145 | C | G | 0.019 | 0.003 | 1.53E-11 | 0.006 | 0.002 | 6.70E-03 |
| rs1993709 | 1 | 72838529 | A | G | -0.041 | 0.003 | 1.49E-42 | 0.000 | 0.003 | 9.60E-01 |
| rs12566985 | 1 | 75002193 | A | G | -0.018 | 0.002 | 1.09E-14 | 0.004 | 0.002 | 4.30E-02 |
| rs12049202 | 1 | 77967523 | T | C | 0.023 | 0.003 | 5.52E-13 | 0.012 | 0.003 | 4.90E-06 |
| rs12034762 | 1 | 96290845 | T | C | 0.017 | 0.003 | 4.80E-11 | -0.005 | 0.002 | 1.50E-02 |
| rs11165643 | 1 | 96924097 | T | C | 0.020 | 0.002 | 1.69E-17 | 0.004 | 0.002 | 8.70E-02 |
| rs17024393 | 1 | 110154688 | T | C | -0.071 | 0.007 | 1.70E-23 | 0.002 | 0.007 | 8.00E-01 |
| rs61813324 | 1 | 156049877 | T | C | 0.028 | 0.004 | 1.29E-10 | 0.015 | 0.003 | 4.30E-06 |
| rs76194251 | 1 | 173931908 | C | G | -0.060 | 0.010 | 4.04E-10 | -0.025 | 0.007 | 6.20E-04 |
| rs543874 | 1 | 177889480 | A | G | -0.036 | 0.003 | 3.70E-35 | -0.004 | 0.003 | 9.30E-02 |
| rs10920678 | 1 | 190239907 | A | G | 0.013 | 0.002 | 1.90E-08 | 0.002 | 0.002 | 3.20E-01 |
| rs2172935 | 1 | 201826340 | T | C | 0.027 | 0.003 | 3.56E-24 | 0.010 | 0.002 | 7.20E-06 |
| rs12217091 | 1 | 204813050 | T | C | 0.027 | 0.005 | 4.44E-08 | 0.004 | 0.004 | 2.90E-01 |
| rs823094 | 1 | 205689807 | T | G | 0.015 | 0.002 | 3.77E-10 | 0.001 | 0.002 | 5.50E-01 |
| rs4653942 | 1 | 228505204 | A | G | -0.016 | 0.003 | 4.41E-08 | 0.005 | 0.003 | 6.70E-02 |
| rs2125231 | 1 | 243762299 | T | C | 0.017 | 0.003 | 5.50E-11 | -0.004 | 0.002 | 5.50E-02 |
| rs76111507 | 2 | 455477 | T | C | -0.081 | 0.008 | 1.13E-25 | 0.003 | 0.006 | 6.20E-01 |
| rs12714415 | 2 | 651430 | T | C | 0.060 | 0.003 | 1.09E-80 | 0.005 | 0.003 | 1.20E-01 |
| rs2693826 | 2 | 6160943 | A | G | -0.016 | 0.003 | 4.96E-11 | -0.003 | 0.002 | 1.10E-01 |
| rs10182181 | 2 | 25150296 | A | G | -0.027 | 0.002 | 5.55E-32 | -0.001 | 0.002 | 6.90E-01 |
| rs1260326 | 2 | 27730940 | T | C | -0.018 | 0.002 | 2.05E-14 | 0.006 | 0.002 | 6.60E-03 |
| rs2888172 | 2 | 35439812 | A | G | -0.014 | 0.003 | 1.42E-08 | -0.001 | 0.002 | 7.20E-01 |
| rs10185199 | 2 | 40282202 | A | G | -0.016 | 0.003 | 3.79E-08 | -0.006 | 0.002 | 1.00E-02 |
| rs7561278 | 2 | 48954905 | T | C | 0.018 | 0.003 | 1.06E-08 | 0.003 | 0.003 | 1.90E-01 |
| rs12986742 | 2 | 58975143 | T | C | -0.020 | 0.003 | 3.22E-15 | -0.008 | 0.002 | 1.00E-04 |
| rs887912 | 2 | 59302877 | T | C | 0.023 | 0.003 | 2.75E-19 | 0.000 | 0.002 | 9.40E-01 |
| rs11685481 | 2 | 67736749 | T | C | 0.020 | 0.003 | 8.99E-09 | 0.003 | 0.003 | 2.50E-01 |
| rs4410328 | 2 | 69600683 | T | C | -0.020 | 0.003 | 7.75E-12 | 0.003 | 0.002 | 2.00E-01 |
| rs10209325 | 2 | 86806356 | T | C | -0.018 | 0.003 | 1.49E-09 | -0.002 | 0.002 | 3.90E-01 |
| rs1437377 | 2 | 100744924 | T | G | -0.024 | 0.004 | 1.47E-11 | -0.010 | 0.003 | 1.70E-03 |
| rs10197031 | 2 | 105454590 | T | C | -0.017 | 0.003 | 7.69E-10 | -0.015 | 0.002 | 8.90E-10 |
| rs13405033 | 2 | 113994595 | T | C | 0.026 | 0.004 | 4.37E-11 | 0.004 | 0.003 | 2.40E-01 |
| rs11687120 | 2 | 119579558 | A | G | 0.014 | 0.003 | 4.74E-08 | -0.006 | 0.002 | 8.00E-03 |
| rs961360 | 2 | 136393658 | A | G | 0.021 | 0.003 | 3.75E-10 | 0.015 | 0.003 | 2.40E-05 |
| rs453520 | 2 | 147907202 | T | C | -0.016 | 0.003 | 1.19E-10 | -0.009 | 0.002 | 5.10E-05 |
| rs34234296 | 2 | 175166636 | A | G | -0.018 | 0.003 | 2.86E-09 | -0.004 | 0.002 | 1.10E-01 |
| rs1019612 | 2 | 181602526 | T | C | 0.018 | 0.003 | 1.40E-11 | 0.008 | 0.002 | 5.70E-04 |
| rs10497807 | 2 | 198585087 | C | G | -0.015 | 0.002 | 4.95E-10 | -0.009 | 0.002 | 2.40E-05 |
| rs7569376 | 2 | 205385322 | T | C | 0.020 | 0.003 | 1.33E-10 | 0.003 | 0.003 | 2.20E-01 |
| rs17203016 | 2 | 208255518 | A | G | -0.017 | 0.003 | 9.91E-09 | -0.003 | 0.003 | 2.20E-01 |
| rs7599312 | 2 | 213413231 | A | G | -0.022 | 0.003 | 1.51E-16 | -0.009 | 0.002 | 1.10E-04 |
| rs1541777 | 2 | 219587291 | A | G | -0.016 | 0.002 | 1.82E-11 | -0.005 | 0.002 | 3.50E-02 |
| rs7600417 | 2 | 220169492 | A | G | 0.021 | 0.004 | 2.88E-09 | 0.005 | 0.003 | 1.30E-01 |
| rs11692572 | 2 | 227774503 | C | G | -0.014 | 0.003 | 3.72E-08 | 0.004 | 0.002 | 8.80E-02 |
| rs2596902 | 3 | 12955982 | A | G | -0.015 | 0.003 | 1.30E-08 | -0.003 | 0.002 | 2.50E-01 |
| rs6804842 | 3 | 25106437 | A | G | -0.018 | 0.002 | 1.29E-14 | -0.001 | 0.002 | 5.60E-01 |
| rs762318 | 3 | 38516369 | A | C | 0.015 | 0.003 | 2.13E-09 | 0.003 | 0.002 | 1.60E-01 |
| rs1524887 | 3 | 44016371 | T | C | -0.014 | 0.003 | 3.60E-08 | -0.008 | 0.002 | 5.00E-04 |
| rs6803741 | 3 | 48123540 | T | C | -0.015 | 0.003 | 1.99E-08 | 0.000 | 0.002 | 9.50E-01 |
| rs2188151 | 3 | 50201924 | T | G | 0.028 | 0.003 | 2.73E-29 | 0.018 | 0.002 | 2.40E-16 |
| rs6787805 | 3 | 51204788 | A | G | -0.021 | 0.004 | 2.31E-09 | 0.017 | 0.003 | 1.30E-07 |
| rs2365389 | 3 | 61236462 | T | C | -0.018 | 0.002 | 5.27E-14 | -0.007 | 0.002 | 1.40E-03 |
| rs11915371 | 3 | 70539559 | A | C | -0.022 | 0.003 | 1.06E-12 | -0.012 | 0.003 | 4.20E-06 |
| rs13078509 | 3 | 70881429 | T | C | 0.048 | 0.008 | 8.37E-09 | 0.012 | 0.007 | 8.50E-02 |
| rs3849570 | 3 | 81792112 | A | C | 0.015 | 0.003 | 2.56E-09 | 0.000 | 0.002 | 9.20E-01 |
| rs12495178 | 3 | 85886077 | T | C | 0.023 | 0.002 | 1.54E-20 | 0.012 | 0.002 | 1.10E-07 |
| rs73140133 | 3 | 88067498 | T | C | -0.021 | 0.003 | 2.56E-12 | -0.013 | 0.002 | 3.60E-09 |
| rs1492014 | 3 | 94071481 | T | C | -0.021 | 0.003 | 1.64E-17 | -0.013 | 0.002 | 1.30E-09 |
| rs1436351 | 3 | 104617973 | T | G | 0.018 | 0.003 | 3.39E-11 | 0.005 | 0.002 | 3.20E-02 |
| rs6768228 | 3 | 108120008 | A | G | -0.015 | 0.003 | 6.77E-09 | 0.000 | 0.002 | 8.30E-01 |
| rs2918217 | 3 | 115546380 | T | C | 0.022 | 0.004 | 7.18E-10 | 0.004 | 0.003 | 2.40E-01 |
| rs1320903 | 3 | 131758077 | A | G | 0.026 | 0.003 | 1.65E-21 | 0.001 | 0.002 | 5.40E-01 |
| rs645040 | 3 | 135926622 | T | G | 0.019 | 0.003 | 3.16E-11 | 0.004 | 0.003 | 9.70E-02 |
| rs2035935 | 3 | 141306013 | A | G | -0.033 | 0.005 | 3.99E-13 | -0.014 | 0.004 | 1.70E-03 |
| rs355777 | 3 | 154034950 | C | G | 0.017 | 0.003 | 1.81E-11 | 0.003 | 0.002 | 1.70E-01 |
| rs9870575 | 3 | 156296559 | A | G | -0.024 | 0.004 | 1.48E-11 | -0.002 | 0.003 | 4.30E-01 |
| rs7356034 | 3 | 170732599 | A | G | 0.022 | 0.003 | 6.24E-12 | 0.007 | 0.002 | 2.30E-03 |
| rs2606227 | 3 | 183536836 | T | C | 0.014 | 0.003 | 4.61E-08 | 0.001 | 0.002 | 7.60E-01 |
| rs9816226 | 3 | 185834499 | A | T | -0.034 | 0.003 | 5.05E-29 | 0.007 | 0.003 | 8.70E-03 |
| rs13135416 | 4 | 18346297 | T | C | 0.018 | 0.003 | 1.56E-08 | 0.001 | 0.002 | 5.90E-01 |
| rs34811474 | 4 | 25408838 | A | G | -0.028 | 0.003 | 6.48E-16 | -0.015 | 0.003 | 2.30E-09 |
| rs2242189 | 4 | 38691024 | T | C | 0.017 | 0.003 | 5.55E-11 | 0.001 | 0.002 | 5.40E-01 |
| rs13130484 | 4 | 45175691 | T | C | 0.035 | 0.002 | 6.44E-50 | 0.011 | 0.002 | 3.70E-07 |
| rs2237025 | 4 | 55541879 | T | C | 0.014 | 0.003 | 3.10E-08 | 0.005 | 0.002 | 2.10E-02 |
| rs13107325 | 4 | 103188709 | T | C | 0.050 | 0.005 | 3.22E-26 | 0.029 | 0.004 | 1.50E-12 |
| rs2613726 | 4 | 112597321 | A | G | -0.016 | 0.003 | 4.34E-10 | -0.002 | 0.002 | 3.80E-01 |
| rs2391518 | 4 | 130817060 | T | C | 0.016 | 0.003 | 1.74E-10 | 0.001 | 0.002 | 6.60E-01 |
| rs1296328 | 4 | 137083193 | A | C | 0.020 | 0.003 | 2.07E-15 | 0.006 | 0.002 | 1.00E-02 |
| rs6537285 | 4 | 145310147 | A | G | -0.018 | 0.003 | 1.24E-10 | 0.005 | 0.002 | 4.90E-02 |
| rs6827083 | 4 | 153075491 | A | G | -0.015 | 0.002 | 1.34E-10 | 0.003 | 0.002 | 1.40E-01 |
| rs828544 | 5 | 3520064 | T | G | 0.014 | 0.003 | 4.45E-08 | -0.003 | 0.002 | 1.60E-01 |
| rs1503526 | 5 | 63020706 | T | C | -0.014 | 0.002 | 5.74E-09 | -0.015 | 0.002 | 1.10E-11 |
| rs253414 | 5 | 74956517 | T | C | 0.026 | 0.003 | 1.10E-24 | 0.007 | 0.002 | 1.40E-03 |
| rs4532349 | 5 | 77473165 | A | G | 0.016 | 0.003 | 6.64E-09 | 0.012 | 0.003 | 4.60E-06 |
| rs7733438 | 5 | 87986284 | T | G | -0.035 | 0.004 | 2.07E-21 | -0.004 | 0.003 | 1.60E-01 |
| rs6235 | 5 | 95728898 | C | G | -0.021 | 0.003 | 1.92E-13 | -0.002 | 0.002 | 4.90E-01 |
| rs11951673 | 5 | 95861012 | T | C | -0.019 | 0.002 | 7.83E-15 | 0.000 | 0.002 | 8.50E-01 |
| rs289228 | 5 | 107405834 | C | G | 0.022 | 0.003 | 9.78E-12 | 0.001 | 0.003 | 8.10E-01 |
| rs7711753 | 5 | 122733317 | A | G | -0.013 | 0.002 | 2.64E-08 | 0.007 | 0.002 | 1.10E-03 |
| rs7715256 | 5 | 153537893 | T | G | -0.016 | 0.002 | 4.52E-12 | 0.003 | 0.002 | 2.00E-01 |
| rs7730898 | 5 | 170459675 | A | G | 0.017 | 0.003 | 9.94E-11 | 0.008 | 0.002 | 1.50E-03 |
| rs6556301 | 5 | 176527577 | T | G | -0.014 | 0.003 | 1.57E-08 | -0.001 | 0.002 | 6.00E-01 |
| rs2228213 | 6 | 12124855 | A | G | -0.015 | 0.003 | 6.77E-10 | -0.005 | 0.002 | 3.30E-02 |
| rs9366639 | 6 | 26167613 | C | G | 0.019 | 0.003 | 3.82E-09 | 0.018 | 0.003 | 2.00E-10 |
| rs2269553 | 6 | 28876509 | A | G | -0.019 | 0.003 | 2.23E-11 | 0.010 | 0.003 | 1.10E-04 |
| rs9277992 | 6 | 33312455 | A | G | 0.028 | 0.004 | 8.69E-14 | 0.013 | 0.003 | 2.30E-06 |
| rs2744974 | 6 | 34579431 | T | C | 0.023 | 0.003 | 8.48E-20 | 0.007 | 0.002 | 2.70E-03 |
| rs34045288 | 6 | 40369081 | T | C | 0.025 | 0.003 | 3.95E-16 | 0.014 | 0.002 | 3.20E-09 |
| rs2206277 | 6 | 50798526 | T | C | 0.045 | 0.003 | 5.35E-50 | 0.001 | 0.003 | 6.00E-01 |
| rs1327259 | 6 | 51177811 | A | G | 0.016 | 0.003 | 5.96E-10 | 0.001 | 0.002 | 6.60E-01 |
| rs7773045 | 6 | 81443423 | T | G | 0.026 | 0.004 | 1.60E-09 | -0.002 | 0.004 | 6.20E-01 |
| rs12663742 | 6 | 97792735 | A | G | -0.018 | 0.003 | 1.69E-12 | -0.003 | 0.002 | 1.20E-01 |
| rs4548017 | 6 | 98399521 | A | G | -0.024 | 0.003 | 1.13E-15 | -0.019 | 0.002 | 1.00E-17 |
| rs17789218 | 6 | 100600097 | T | C | -0.015 | 0.003 | 3.33E-08 | 0.019 | 0.003 | 1.40E-13 |
| rs768023 | 6 | 108876002 | A | G | 0.013 | 0.002 | 3.05E-08 | 0.005 | 0.002 | 2.00E-02 |
| rs1159974 | 6 | 126090277 | T | C | -0.014 | 0.003 | 2.03E-08 | 0.000 | 0.002 | 8.80E-01 |
| rs6569648 | 6 | 130349119 | T | C | -0.015 | 0.003 | 3.66E-08 | -0.002 | 0.003 | 3.40E-01 |
| rs765875 | 6 | 143185683 | T | C | -0.014 | 0.003 | 3.43E-08 | -0.006 | 0.002 | 5.20E-03 |
| rs13191362 | 6 | 163033350 | A | G | 0.027 | 0.004 | 2.34E-13 | 0.001 | 0.003 | 7.40E-01 |
| rs9364687 | 6 | 163817911 | T | G | -0.014 | 0.002 | 3.97E-09 | 0.001 | 0.002 | 5.80E-01 |
| rs9638713 | 7 | 14645949 | A | G | 0.045 | 0.008 | 2.32E-09 | 0.003 | 0.007 | 6.30E-01 |
| rs215635 | 7 | 32370220 | T | C | -0.018 | 0.003 | 1.16E-12 | -0.007 | 0.002 | 1.80E-03 |
| rs77095142 | 7 | 39096572 | A | C | 0.046 | 0.008 | 2.37E-08 | 0.007 | 0.006 | 2.20E-01 |
| rs10269783 | 7 | 49616203 | A | G | 0.017 | 0.002 | 7.59E-13 | 0.000 | 0.002 | 8.80E-01 |
| rs1167827 | 7 | 75163169 | A | G | -0.022 | 0.002 | 2.88E-19 | -0.008 | 0.002 | 2.10E-04 |
| rs2245368 | 7 | 76608143 | T | C | -0.033 | 0.003 | 4.39E-23 | 0.004 | 0.003 | 1.40E-01 |
| rs1965529 | 7 | 77825707 | A | G | 0.018 | 0.003 | 4.22E-08 | 0.008 | 0.003 | 2.30E-03 |
| rs1701829 | 7 | 113033426 | A | G | 0.021 | 0.003 | 6.76E-16 | 0.012 | 0.002 | 1.70E-07 |
| rs13228237 | 7 | 138780222 | C | G | -0.016 | 0.003 | 4.59E-08 | -0.003 | 0.003 | 2.60E-01 |
| rs4841504 | 8 | 11024663 | A | C | -0.019 | 0.002 | 4.24E-16 | 0.011 | 0.002 | 2.90E-07 |
| rs4123853 | 8 | 14091025 | T | C | 0.017 | 0.003 | 1.10E-10 | -0.006 | 0.002 | 1.30E-02 |
| rs354510 | 8 | 15534749 | T | C | 0.024 | 0.004 | 1.98E-08 | -0.003 | 0.003 | 4.30E-01 |
| rs7827210 | 8 | 30871656 | A | G | 0.018 | 0.003 | 2.34E-12 | 0.017 | 0.002 | 9.90E-14 |
| rs7012923 | 8 | 33630937 | A | C | 0.016 | 0.003 | 4.92E-11 | -0.004 | 0.002 | 7.30E-02 |
| rs35957544 | 8 | 73440371 | T | G | -0.018 | 0.003 | 4.14E-10 | -0.007 | 0.002 | 8.20E-04 |
| rs2588785 | 8 | 76636191 | A | G | -0.021 | 0.003 | 1.74E-15 | 0.008 | 0.002 | 1.90E-03 |
| rs957447 | 8 | 95541223 | A | C | -0.023 | 0.003 | 8.56E-14 | -0.005 | 0.003 | 4.70E-02 |
| rs2960151 | 8 | 116718620 | A | C | 0.023 | 0.003 | 3.22E-11 | 0.007 | 0.003 | 9.40E-03 |
| rs72673947 | 8 | 118884379 | A | G | -0.028 | 0.005 | 1.76E-09 | -0.012 | 0.004 | 6.90E-04 |
| rs7826388 | 8 | 128109776 | T | C | -0.037 | 0.007 | 1.40E-08 | 0.006 | 0.006 | 3.40E-01 |
| rs4740619 | 9 | 15634326 | T | C | 0.019 | 0.002 | 1.50E-15 | 0.007 | 0.002 | 6.40E-04 |
| rs10738451 | 9 | 16713666 | T | C | -0.016 | 0.003 | 2.22E-09 | -0.007 | 0.002 | 2.20E-03 |
| rs10968114 | 9 | 27800007 | A | C | 0.015 | 0.003 | 8.70E-10 | -0.001 | 0.002 | 6.80E-01 |
| rs1412235 | 9 | 28410996 | C | G | 0.023 | 0.003 | 9.85E-21 | 0.005 | 0.002 | 4.80E-02 |
| rs10746862 | 9 | 73780912 | T | G | -0.016 | 0.003 | 8.63E-10 | -0.003 | 0.002 | 1.50E-01 |
| rs1187351 | 9 | 87295077 | A | C | -0.014 | 0.003 | 4.51E-08 | 0.005 | 0.002 | 2.60E-02 |
| rs6559921 | 9 | 88872039 | A | T | 0.018 | 0.003 | 1.19E-09 | 0.001 | 0.003 | 8.40E-01 |
| rs6477694 | 9 | 111932342 | T | C | -0.014 | 0.002 | 8.89E-09 | 0.000 | 0.002 | 8.60E-01 |
| rs7038943 | 9 | 120377178 | T | C | 0.015 | 0.003 | 6.54E-09 | 0.007 | 0.002 | 2.90E-03 |
| rs10760279 | 9 | 126105291 | T | G | 0.015 | 0.003 | 7.49E-09 | 0.009 | 0.002 | 3.70E-05 |
| rs3902840 | 9 | 129419025 | A | G | 0.031 | 0.004 | 2.49E-12 | 0.009 | 0.004 | 2.10E-02 |
| rs4740383 | 9 | 133783566 | A | G | 0.015 | 0.003 | 2.67E-09 | 0.003 | 0.002 | 1.70E-01 |
| rs11251291 | 10 | 2561766 | A | C | -0.014 | 0.003 | 1.65E-08 | 0.000 | 0.002 | 9.70E-01 |
| rs12251016 | 10 | 21821918 | A | T | -0.019 | 0.003 | 1.43E-10 | -0.017 | 0.002 | 8.60E-14 |
| rs3758438 | 10 | 27400661 | T | C | 0.027 | 0.005 | 7.27E-09 | -0.006 | 0.004 | 1.30E-01 |
| rs12761779 | 10 | 63782043 | C | G | 0.017 | 0.003 | 1.48E-08 | 0.004 | 0.002 | 1.20E-01 |
| rs7072873 | 10 | 77210191 | T | C | -0.015 | 0.003 | 5.45E-09 | -0.002 | 0.002 | 4.50E-01 |
| rs10788494 | 10 | 88110925 | C | G | 0.016 | 0.003 | 5.39E-10 | 0.006 | 0.002 | 9.60E-03 |
| rs10883026 | 10 | 99793865 | T | C | -0.019 | 0.003 | 6.23E-11 | -0.005 | 0.002 | 1.30E-02 |
| rs17094222 | 10 | 102395440 | T | C | -0.026 | 0.003 | 1.14E-18 | 0.002 | 0.003 | 5.00E-01 |
| rs11191356 | 10 | 104393489 | A | G | -0.026 | 0.004 | 2.48E-10 | -0.007 | 0.004 | 9.20E-02 |
| rs79780963 | 10 | 104952499 | T | C | 0.037 | 0.005 | 1.55E-12 | 0.011 | 0.004 | 6.60E-03 |
| rs7903146 | 10 | 114758349 | T | C | -0.023 | 0.003 | 3.57E-19 | 0.000 | 0.002 | 9.00E-01 |
| rs845084 | 10 | 125220036 | A | G | 0.016 | 0.003 | 3.85E-08 | 0.003 | 0.002 | 2.10E-01 |
| rs7113874 | 11 | 8688016 | T | C | -0.014 | 0.002 | 3.30E-09 | -0.003 | 0.002 | 2.20E-01 |
| rs900144 | 11 | 13294268 | T | C | 0.015 | 0.003 | 5.54E-10 | 0.006 | 0.002 | 3.40E-03 |
| rs6265 | 11 | 27679916 | T | C | -0.048 | 0.003 | 6.25E-58 | 0.003 | 0.003 | 2.20E-01 |
| rs1765139 | 11 | 30353725 | T | C | 0.022 | 0.003 | 1.80E-13 | 0.008 | 0.003 | 1.40E-03 |
| rs2862996 | 11 | 43653833 | T | G | -0.020 | 0.003 | 2.49E-15 | -0.004 | 0.002 | 9.40E-02 |
| rs7124681 | 11 | 47529947 | A | C | 0.028 | 0.002 | 5.10E-31 | 0.004 | 0.002 | 5.10E-02 |
| rs7102454 | 11 | 65594820 | T | C | -0.018 | 0.003 | 1.29E-11 | -0.009 | 0.002 | 2.40E-05 |
| rs11228371 | 11 | 68594174 | A | C | 0.017 | 0.003 | 1.31E-08 | -0.002 | 0.002 | 3.50E-01 |
| rs349071 | 11 | 84776849 | A | G | -0.018 | 0.003 | 7.53E-10 | -0.002 | 0.002 | 2.50E-01 |
| rs647248 | 11 | 89917699 | A | G | 0.016 | 0.003 | 2.59E-10 | 0.003 | 0.002 | 1.60E-01 |
| rs12286929 | 11 | 115022404 | A | G | -0.018 | 0.002 | 2.34E-14 | -0.006 | 0.002 | 5.50E-03 |
| rs7933085 | 11 | 130796248 | A | G | -0.017 | 0.003 | 1.62E-09 | -0.002 | 0.002 | 2.70E-01 |
| rs10894670 | 11 | 133221987 | A | C | -0.014 | 0.002 | 1.65E-09 | -0.002 | 0.002 | 4.20E-01 |
| rs11611246 | 12 | 939480 | T | G | 0.025 | 0.003 | 7.99E-17 | -0.002 | 0.003 | 4.20E-01 |
| rs76895963 | 12 | 4384844 | T | G | -0.077 | 0.011 | 1.07E-11 | 0.011 | 0.008 | 1.80E-01 |
| rs10772983 | 12 | 17141582 | T | C | -0.014 | 0.002 | 2.04E-09 | 0.000 | 0.002 | 8.70E-01 |
| rs10842240 | 12 | 24060075 | C | G | 0.026 | 0.004 | 4.56E-12 | 0.006 | 0.003 | 6.20E-02 |
| rs11170468 | 12 | 39430048 | A | C | 0.016 | 0.003 | 7.84E-09 | 0.003 | 0.003 | 2.70E-01 |
| rs11181001 | 12 | 41948196 | A | G | 0.014 | 0.002 | 1.25E-09 | -0.002 | 0.002 | 3.50E-01 |
| rs7132908 | 12 | 50263148 | A | G | 0.035 | 0.003 | 1.35E-38 | 0.003 | 0.002 | 1.90E-01 |
| rs705704 | 12 | 56435412 | A | G | -0.016 | 0.003 | 1.52E-10 | -0.012 | 0.002 | 1.30E-07 |
| rs1689437 | 12 | 89758046 | A | G | -0.030 | 0.005 | 2.04E-09 | -0.009 | 0.004 | 4.90E-02 |
| rs7488867 | 12 | 103699685 | T | C | -0.022 | 0.003 | 5.69E-14 | 0.000 | 0.002 | 8.70E-01 |
| rs10850184 | 12 | 114064977 | T | G | 0.017 | 0.003 | 3.53E-10 | 0.007 | 0.002 | 3.10E-03 |
| rs11067700 | 12 | 116083562 | T | C | -0.017 | 0.003 | 8.28E-09 | -0.001 | 0.002 | 7.00E-01 |
| rs12369179 | 12 | 122963550 | T | C | -0.035 | 0.005 | 1.37E-14 | 0.000 | 0.004 | 9.30E-01 |
| rs10902480 | 12 | 132414769 | T | G | 0.022 | 0.004 | 5.60E-09 | 0.002 | 0.003 | 5.70E-01 |
| rs2012835 | 13 | 27987643 | T | G | -0.021 | 0.003 | 3.06E-10 | -0.001 | 0.002 | 7.00E-01 |
| rs2504236 | 13 | 28613298 | T | C | -0.015 | 0.003 | 1.94E-09 | 0.001 | 0.002 | 6.30E-01 |
| rs9595893 | 13 | 33175793 | T | G | -0.020 | 0.003 | 1.24E-14 | -0.008 | 0.002 | 2.40E-04 |
| rs9603697 | 13 | 40783323 | T | C | 0.020 | 0.003 | 1.34E-13 | 0.005 | 0.002 | 2.20E-02 |
| rs12429545 | 13 | 54102206 | A | G | 0.034 | 0.004 | 2.03E-21 | -0.003 | 0.003 | 3.40E-01 |
| rs7338127 | 13 | 59260511 | A | T | 0.020 | 0.003 | 1.39E-13 | 0.005 | 0.002 | 3.20E-02 |
| rs1441264 | 13 | 79580919 | A | G | 0.018 | 0.002 | 1.35E-13 | -0.003 | 0.002 | 1.40E-01 |
| rs912690 | 13 | 97020705 | C | G | -0.017 | 0.003 | 6.92E-12 | -0.006 | 0.002 | 4.40E-03 |
| rs9168 | 13 | 99101583 | A | C | -0.015 | 0.003 | 4.62E-08 | -0.006 | 0.002 | 1.80E-02 |
| rs9522279 | 13 | 112221296 | T | C | 0.022 | 0.003 | 1.65E-14 | 0.006 | 0.002 | 5.30E-03 |
| rs10132280 | 14 | 25928179 | A | C | -0.030 | 0.003 | 1.38E-31 | -0.001 | 0.002 | 5.90E-01 |
| rs1811859 | 14 | 29677727 | T | C | -0.020 | 0.004 | 6.48E-09 | -0.012 | 0.003 | 3.60E-06 |
| rs1955540 | 14 | 40801751 | T | C | -0.020 | 0.003 | 2.89E-10 | -0.005 | 0.003 | 5.60E-02 |
| rs4900714 | 14 | 47302219 | T | G | 0.020 | 0.003 | 1.17E-15 | 0.002 | 0.002 | 3.20E-01 |
| rs217672 | 14 | 62361021 | A | C | -0.020 | 0.003 | 4.40E-10 | -0.010 | 0.002 | 8.50E-05 |
| rs3902951 | 14 | 69789755 | T | G | -0.015 | 0.003 | 4.40E-08 | -0.008 | 0.003 | 1.30E-03 |
| rs17109256 | 14 | 79939993 | A | G | 0.031 | 0.003 | 2.81E-27 | 0.000 | 0.003 | 9.60E-01 |
| rs34918753 | 14 | 88312341 | A | G | 0.020 | 0.003 | 1.77E-09 | 0.003 | 0.003 | 1.90E-01 |
| rs11160119 | 14 | 93932258 | A | G | -0.022 | 0.003 | 2.85E-14 | -0.001 | 0.002 | 7.50E-01 |
| rs7161194 | 14 | 101529005 | A | G | 0.022 | 0.003 | 3.32E-15 | 0.000 | 0.002 | 8.60E-01 |
| rs7143963 | 14 | 103304425 | T | C | 0.021 | 0.003 | 3.35E-12 | 0.000 | 0.003 | 9.40E-01 |
| rs11636536 | 15 | 36391772 | T | C | 0.015 | 0.003 | 2.80E-09 | 0.008 | 0.002 | 3.50E-04 |
| rs10851458 | 15 | 47939956 | A | G | 0.015 | 0.003 | 5.95E-09 | -0.008 | 0.002 | 2.10E-04 |
| rs4776970 | 15 | 68080886 | A | T | 0.028 | 0.002 | 1.52E-30 | 0.007 | 0.002 | 3.50E-03 |
| rs58429335 | 15 | 70199927 | T | C | -0.031 | 0.006 | 4.85E-08 | -0.004 | 0.004 | 3.00E-01 |
| rs4777541 | 15 | 73082240 | T | C | 0.021 | 0.003 | 1.15E-12 | 0.010 | 0.003 | 6.90E-05 |
| rs8041222 | 15 | 79465549 | A | G | -0.019 | 0.003 | 3.28E-11 | -0.007 | 0.002 | 8.30E-04 |
| rs8036171 | 15 | 95266810 | A | C | 0.015 | 0.003 | 4.26E-10 | 0.003 | 0.002 | 1.40E-01 |
| rs12448257 | 16 | 3599655 | A | G | 0.019 | 0.003 | 1.17E-10 | -0.004 | 0.003 | 1.20E-01 |
| rs879620 | 16 | 4015729 | T | C | 0.021 | 0.003 | 3.11E-16 | 0.006 | 0.002 | 1.20E-02 |
| rs9926784 | 16 | 19941968 | T | C | 0.022 | 0.003 | 5.74E-14 | -0.002 | 0.003 | 5.10E-01 |
| rs3888190 | 16 | 28889486 | A | C | 0.032 | 0.002 | 6.96E-40 | 0.014 | 0.002 | 3.00E-10 |
| rs57149692 | 16 | 30142021 | C | G | -0.027 | 0.003 | 1.11E-19 | 0.000 | 0.002 | 9.00E-01 |
| rs1558902 | 16 | 53803574 | A | T | 0.082 | 0.002 | 1.19E-257 | 0.001 | 0.002 | 6.60E-01 |
| rs889398 | 16 | 69556715 | T | C | -0.021 | 0.002 | 1.54E-18 | -0.002 | 0.002 | 4.30E-01 |
| rs75950518 | 16 | 70378917 | T | C | -0.027 | 0.005 | 3.51E-09 | -0.006 | 0.003 | 6.70E-02 |
| rs28483459 | 16 | 71998398 | T | C | -0.018 | 0.003 | 2.92E-09 | -0.003 | 0.002 | 1.90E-01 |
| rs7206608 | 16 | 82872628 | C | G | -0.015 | 0.003 | 1.85E-08 | -0.010 | 0.002 | 3.40E-05 |
| rs3923783 | 17 | 1843189 | A | C | -0.023 | 0.003 | 7.47E-12 | -0.008 | 0.003 | 2.30E-03 |
| rs12450045 | 17 | 4742112 | A | G | 0.014 | 0.003 | 1.68E-08 | 0.006 | 0.002 | 7.40E-03 |
| rs4986044 | 17 | 21261560 | T | C | -0.018 | 0.002 | 1.45E-14 | -0.009 | 0.002 | 1.80E-05 |
| rs8065172 | 17 | 31456969 | A | G | -0.017 | 0.003 | 1.23E-08 | -0.006 | 0.003 | 3.10E-02 |
| rs12150665 | 17 | 34914787 | T | C | 0.018 | 0.002 | 3.43E-14 | 0.006 | 0.002 | 4.50E-03 |
| rs676387 | 17 | 40706273 | A | C | 0.016 | 0.003 | 4.10E-09 | 0.010 | 0.002 | 2.10E-05 |
| rs208015 | 17 | 46252346 | T | C | 0.037 | 0.005 | 8.72E-14 | 0.008 | 0.004 | 7.90E-02 |
| rs78369934 | 17 | 61739101 | T | C | 0.037 | 0.007 | 1.47E-08 | 0.022 | 0.005 | 3.90E-06 |
| rs12939549 | 17 | 78611724 | A | G | 0.020 | 0.002 | 1.29E-17 | 0.001 | 0.002 | 5.80E-01 |
| rs7226371 | 18 | 1850771 | A | G | -0.019 | 0.003 | 7.73E-09 | -0.006 | 0.003 | 4.20E-02 |
| rs891387 | 18 | 21103909 | T | C | 0.023 | 0.003 | 6.81E-20 | 0.013 | 0.002 | 3.00E-09 |
| rs11874191 | 18 | 36164084 | T | C | -0.017 | 0.003 | 3.06E-09 | -0.001 | 0.002 | 7.10E-01 |
| rs2612575 | 18 | 42967340 | A | G | -0.016 | 0.003 | 1.45E-08 | -0.004 | 0.002 | 6.50E-02 |
| rs9951619 | 18 | 56882326 | T | G | -0.019 | 0.003 | 1.75E-11 | 0.004 | 0.003 | 1.00E-01 |
| rs6567160 | 18 | 57829135 | T | C | -0.053 | 0.003 | 2.11E-82 | 0.006 | 0.003 | 2.50E-02 |
| rs17066856 | 18 | 58049656 | T | C | 0.030 | 0.004 | 2.65E-14 | 0.002 | 0.004 | 6.60E-01 |
| rs17710386 | 18 | 63461201 | T | C | -0.016 | 0.003 | 3.75E-11 | -0.003 | 0.002 | 1.60E-01 |
| rs1787267 | 18 | 76742544 | C | G | -0.033 | 0.005 | 2.49E-10 | 0.001 | 0.005 | 7.50E-01 |
| rs45486197 | 19 | 2244849 | A | G | 0.035 | 0.006 | 3.49E-09 | 0.001 | 0.004 | 9.00E-01 |
| rs7258309 | 19 | 4016005 | T | G | 0.023 | 0.003 | 6.54E-14 | 0.009 | 0.003 | 1.40E-03 |
| rs273496 | 19 | 18241857 | T | C | 0.020 | 0.003 | 2.50E-11 | 0.010 | 0.002 | 1.40E-05 |
| rs7258722 | 19 | 18808915 | A | T | -0.020 | 0.003 | 8.61E-15 | -0.004 | 0.002 | 5.80E-02 |
| rs17513613 | 19 | 30286822 | T | C | -0.018 | 0.003 | 1.50E-12 | -0.004 | 0.002 | 8.50E-02 |
| rs11084553 | 19 | 31019780 | A | G | 0.024 | 0.004 | 9.41E-12 | 0.004 | 0.003 | 2.00E-01 |
| rs769449 | 19 | 45410002 | A | G | -0.024 | 0.004 | 1.58E-09 | -0.014 | 0.003 | 1.70E-05 |
| rs1800437 | 19 | 46181392 | C | G | -0.037 | 0.003 | 1.73E-33 | -0.004 | 0.003 | 1.30E-01 |
| rs9304665 | 19 | 47602577 | A | T | 0.027 | 0.003 | 4.78E-21 | -0.006 | 0.003 | 1.90E-02 |
| rs7249149 | 19 | 51774538 | A | G | 0.015 | 0.003 | 4.41E-08 | 0.001 | 0.002 | 5.80E-01 |
| rs2145270 | 20 | 6621685 | T | C | 0.021 | 0.002 | 6.97E-18 | 0.001 | 0.002 | 5.90E-01 |
| rs8123881 | 20 | 15819495 | A | G | -0.019 | 0.003 | 2.53E-08 | -0.003 | 0.003 | 3.60E-01 |
| rs7272093 | 20 | 49810724 | A | G | -0.017 | 0.003 | 1.10E-09 | -0.006 | 0.002 | 2.00E-02 |
| rs35388084 | 20 | 50960490 | A | G | -0.028 | 0.004 | 1.12E-13 | -0.010 | 0.003 | 4.70E-04 |
| rs6092143 | 20 | 54181875 | T | C | 0.015 | 0.003 | 1.10E-08 | 0.002 | 0.002 | 4.80E-01 |
| rs13047416 | 21 | 40309436 | C | G | 0.016 | 0.003 | 9.84E-10 | 0.002 | 0.002 | 3.80E-01 |
| rs427943 | 21 | 46570896 | A | C | -0.018 | 0.003 | 2.19E-12 | -0.009 | 0.002 | 3.70E-05 |
| rs138383 | 22 | 38799861 | T | C | -0.016 | 0.003 | 1.39E-09 | -0.004 | 0.002 | 8.10E-02 |
| rs733381 | 22 | 40669648 | A | G | 0.021 | 0.003 | 5.20E-13 | 0.001 | 0.003 | 8.20E-01 |
| **4) MR of leisure television use on BMI (men)** | | | | | | | | | | |
| SNP | CHR | POS | EA | OA | beta_bmi | se_bmi | pval_bmi | beta_tv | se_tv | pval_tv |
| rs11810109 | 1 | 44186812 | A | T | 0.007 | 0.003 | 3.11E-02 | 0.016 | 0.002 | 5.40E-12 |
| rs112632642 | 1 | 51115634 | G | A | -0.015 | 0.006 | 8.48E-03 | -0.024 | 0.004 | 3.60E-08 |
| rs6670915 | 1 | 61109968 | C | T | 0.008 | 0.003 | 7.66E-04 | 0.014 | 0.002 | 2.40E-10 |
| rs12725114 | 1 | 62432641 | G | A | 0.015 | 0.004 | 4.97E-05 | 0.015 | 0.003 | 1.60E-08 |
| rs984409 | 1 | 67020440 | G | A | 0.002 | 0.003 | 5.13E-01 | -0.015 | 0.002 | 4.10E-11 |
| rs71658797 | 1 | 77967507 | T | A | -0.036 | 0.005 | 1.04E-15 | -0.02 | 0.003 | 6.30E-10 |
| rs114328297 | 1 | 91190854 | T | G | -0.003 | 0.004 | 3.45E-01 | 0.014 | 0.003 | 4.40E-08 |
| rs631130 | 1 | 96043010 | T | A | 0.006 | 0.003 | 5.36E-02 | -0.014 | 0.002 | 5.00E-09 |
| rs148648822 | 1 | 97815048 | G | T | -0.007 | 0.004 | 3.48E-02 | -0.018 | 0.003 | 4.20E-09 |
| rs17379561 | 1 | 98340139 | A | T | -0.010 | 0.004 | 5.83E-03 | -0.025 | 0.003 | 1.10E-16 |
| rs55909997 | 1 | 107581039 | G | A | -0.011 | 0.003 | 2.87E-05 | -0.014 | 0.002 | 2.40E-09 |
| rs1278847 | 1 | 110037483 | C | A | -0.013 | 0.003 | 1.77E-05 | 0.016 | 0.002 | 1.20E-11 |
| rs141425667 | 1 | 114930465 | G | A | -0.006 | 0.006 | 2.95E-01 | -0.024 | 0.004 | 4.00E-08 |
| rs4845364 | 1 | 154141908 | A | G | 0.000 | 0.003 | 9.54E-01 | -0.015 | 0.002 | 1.30E-12 |
| rs6673341 | 1 | 184655649 | T | G | -0.003 | 0.003 | 2.03E-01 | -0.015 | 0.002 | 2.20E-11 |
| rs72725224 | 1 | 188115858 | A | C | -0.025 | 0.011 | 1.63E-02 | -0.046 | 0.008 | 3.70E-09 |
| rs10737620 | 1 | 193057971 | T | A | 0.006 | 0.003 | 2.31E-02 | 0.014 | 0.002 | 2.60E-09 |
| rs11896615 | 2 | 17407744 | G | A | 0.007 | 0.004 | 7.15E-02 | 0.017 | 0.003 | 3.10E-08 |
| rs72781699 | 2 | 24277709 | G | A | 0.002 | 0.004 | 5.00E-01 | -0.019 | 0.003 | 3.00E-12 |
| rs12105701 | 2 | 41737200 | C | T | -0.010 | 0.003 | 8.44E-05 | -0.013 | 0.002 | 5.40E-09 |
| rs7564130 | 2 | 50606642 | T | C | -0.002 | 0.003 | 3.59E-01 | -0.015 | 0.002 | 2.80E-11 |
| rs11125515 | 2 | 53886799 | C | A | 0.008 | 0.003 | 3.68E-03 | -0.014 | 0.002 | 2.20E-08 |
| rs12475168 | 2 | 58992704 | G | T | -0.019 | 0.004 | 8.48E-08 | -0.018 | 0.003 | 1.70E-08 |
| rs183566425 | 2 | 59325356 | A | T | 0.031 | 0.011 | 5.60E-03 | 0.046 | 0.008 | 1.90E-08 |
| rs10189857 | 2 | 60713235 | A | G | -0.009 | 0.003 | 3.02E-04 | -0.02 | 0.002 | 6.20E-21 |
| rs62641636 | 2 | 68425427 | A | G | 0.010 | 0.003 | 1.29E-03 | 0.014 | 0.002 | 7.00E-10 |
| rs59210467 | 2 | 97097291 | C | T | 0.007 | 0.003 | 3.73E-02 | -0.013 | 0.002 | 1.00E-08 |
| rs11689199 | 2 | 100820421 | A | G | 0.014 | 0.003 | 7.23E-08 | 0.018 | 0.002 | 5.50E-17 |
| rs2916251 | 2 | 104447052 | G | T | -0.011 | 0.003 | 1.04E-05 | -0.017 | 0.002 | 1.50E-13 |
| rs1451533 | 2 | 105466005 | G | A | -0.017 | 0.003 | 1.15E-09 | -0.016 | 0.002 | 1.10E-10 |
| rs2971640 | 2 | 107624618 | G | A | -0.005 | 0.003 | 5.55E-02 | -0.013 | 0.002 | 4.20E-09 |
| rs12476388 | 2 | 116330983 | C | T | -0.001 | 0.003 | 6.84E-01 | 0.013 | 0.002 | 2.10E-08 |
| rs374722 | 2 | 147839830 | G | A | 0.016 | 0.004 | 1.99E-06 | 0.024 | 0.003 | 5.50E-16 |
| rs3754970 | 2 | 162091836 | T | C | -0.009 | 0.003 | 2.10E-03 | -0.015 | 0.002 | 4.80E-12 |
| rs1913808 | 2 | 162804678 | G | A | 0.000 | 0.003 | 9.07E-01 | 0.015 | 0.002 | 3.90E-10 |
| rs2060199 | 2 | 166196681 | T | A | 0.008 | 0.003 | 2.39E-03 | 0.012 | 0.002 | 2.40E-08 |
| rs263771 | 2 | 185921692 | C | A | 0.001 | 0.003 | 8.79E-01 | -0.017 | 0.003 | 3.10E-11 |
| rs4577309 | 2 | 191288833 | A | G | 0.001 | 0.003 | 7.52E-01 | 0.016 | 0.002 | 1.60E-13 |
| rs34224261 | 2 | 193855452 | G | C | -0.009 | 0.003 | 2.01E-03 | -0.018 | 0.002 | 4.60E-16 |
| rs4675246 | 2 | 202864487 | G | T | 0.002 | 0.003 | 5.96E-01 | -0.015 | 0.003 | 2.90E-08 |
| rs13029509 | 2 | 215374209 | G | A | -0.005 | 0.003 | 2.91E-02 | -0.018 | 0.002 | 2.00E-17 |
| rs10932837 | 2 | 221044785 | C | T | 0.004 | 0.003 | 8.62E-02 | -0.013 | 0.002 | 1.30E-09 |
| rs4973576 | 2 | 233806771 | C | A | 0.007 | 0.003 | 2.79E-02 | -0.015 | 0.002 | 1.00E-09 |
| rs138256022 | 3 | 11603090 | C | T | 0.005 | 0.008 | 5.23E-01 | -0.031 | 0.006 | 2.90E-08 |
| rs9834970 | 3 | 36856030 | T | C | 0.008 | 0.003 | 2.09E-03 | 0.013 | 0.002 | 3.30E-09 |
| rs1352890 | 3 | 49652156 | C | T | -0.005 | 0.003 | 7.52E-02 | -0.012 | 0.002 | 1.30E-08 |
| rs3796386 | 3 | 49899795 | G | A | -0.019 | 0.003 | 1.15E-13 | -0.026 | 0.002 | 3.20E-33 |
| rs182355396 | 3 | 50920457 | C | A | -0.008 | 0.017 | 6.65E-01 | -0.08 | 0.013 | 2.10E-10 |
| rs11917871 | 3 | 54233476 | T | C | -0.001 | 0.003 | 7.80E-01 | -0.017 | 0.002 | 4.50E-14 |
| rs11130793 | 3 | 60884659 | C | T | 0.005 | 0.003 | 9.55E-02 | 0.013 | 0.002 | 5.00E-09 |
| rs11714337 | 3 | 71582521 | G | A | 0.006 | 0.003 | 4.17E-02 | 0.014 | 0.002 | 4.70E-11 |
| rs12496084 | 3 | 75174777 | A | G | 0.000 | 0.003 | 8.83E-01 | -0.014 | 0.003 | 4.60E-08 |
| rs6797840 | 3 | 85656569 | A | C | -0.017 | 0.003 | 2.24E-09 | -0.016 | 0.002 | 1.70E-13 |
| rs56398417 | 3 | 88024986 | C | T | 0.022 | 0.003 | 1.34E-12 | 0.014 | 0.002 | 5.90E-10 |
| rs2034768 | 3 | 93986371 | A | G | 0.021 | 0.003 | 2.43E-13 | 0.015 | 0.002 | 8.60E-12 |
| rs12632778 | 3 | 106452359 | T | G | 0.003 | 0.006 | 6.09E-01 | -0.028 | 0.005 | 3.90E-08 |
| rs9867121 | 3 | 114631548 | C | A | 0.005 | 0.004 | 1.63E-01 | 0.019 | 0.003 | 3.90E-12 |
| rs12491503 | 3 | 165706855 | G | A | -0.001 | 0.003 | 6.70E-01 | -0.014 | 0.002 | 5.50E-10 |
| rs114600294 | 3 | 181419367 | G | C | -0.005 | 0.004 | 2.08E-01 | -0.016 | 0.003 | 7.90E-10 |
| rs66852340 | 4 | 3311070 | C | T | -0.010 | 0.003 | 5.26E-04 | -0.018 | 0.003 | 7.90E-12 |
| rs7700107 | 4 | 17880416 | A | C | -0.003 | 0.004 | 3.59E-01 | -0.021 | 0.003 | 1.90E-11 |
| rs34811474 | 4 | 25408838 | G | A | 0.028 | 0.003 | 6.48E-16 | 0.015 | 0.003 | 2.30E-09 |
| rs77215114 | 4 | 45187658 | A | T | 0.011 | 0.006 | 5.95E-02 | 0.024 | 0.004 | 1.50E-08 |
| rs28971796 | 4 | 49151982 | A | G | 0.010 | 0.003 | 1.39E-03 | 0.015 | 0.002 | 2.10E-09 |
| rs6850494 | 4 | 82291771 | A | C | -0.002 | 0.003 | 4.67E-01 | -0.014 | 0.002 | 1.10E-10 |
| rs13107325 | 4 | 103188709 | C | T | -0.050 | 0.005 | 3.22E-26 | -0.029 | 0.004 | 1.50E-12 |
| rs62332760 | 4 | 106135316 | G | T | -0.001 | 0.003 | 8.00E-01 | -0.014 | 0.002 | 8.00E-11 |
| rs7693703 | 4 | 118357405 | G | A | 0.005 | 0.004 | 2.19E-01 | 0.023 | 0.004 | 2.70E-09 |
| rs4334769 | 4 | 130245233 | G | T | 0.000 | 0.003 | 9.22E-01 | 0.012 | 0.002 | 2.10E-08 |
| rs6825241 | 4 | 152543783 | C | A | -0.004 | 0.003 | 1.02E-01 | -0.017 | 0.002 | 4.90E-15 |
| rs7693082 | 4 | 159857819 | G | C | 0.003 | 0.003 | 3.51E-01 | 0.015 | 0.002 | 1.70E-10 |
| rs10940659 | 5 | 59360341 | A | G | -0.008 | 0.003 | 2.66E-03 | -0.013 | 0.002 | 1.50E-08 |
| rs2409838 | 5 | 60204410 | A | T | -0.005 | 0.003 | 6.85E-02 | 0.017 | 0.002 | 3.40E-15 |
| rs262890 | 5 | 62930015 | A | G | -0.014 | 0.003 | 8.98E-06 | -0.019 | 0.002 | 3.20E-15 |
| rs7716447 | 5 | 88800331 | A | G | -0.014 | 0.003 | 1.40E-07 | -0.013 | 0.002 | 3.60E-09 |
| rs1031423 | 5 | 93276883 | T | C | -0.001 | 0.003 | 7.28E-01 | -0.019 | 0.003 | 1.80E-12 |
| rs57585211 | 5 | 107197725 | T | G | -0.005 | 0.004 | 1.72E-01 | -0.017 | 0.003 | 4.30E-09 |
| rs9326862 | 5 | 112054547 | A | T | 0.004 | 0.003 | 9.99E-02 | 0.012 | 0.002 | 2.60E-08 |
| rs10041724 | 5 | 124273520 | T | C | 0.008 | 0.003 | 8.30E-03 | 0.018 | 0.003 | 3.90E-11 |
| rs10057545 | 5 | 138431008 | G | A | -0.002 | 0.003 | 5.63E-01 | -0.014 | 0.002 | 2.00E-10 |
| rs62379379 | 5 | 141082015 | G | T | 0.003 | 0.006 | 6.20E-01 | -0.026 | 0.004 | 7.80E-10 |
| rs10054327 | 5 | 147871137 | G | A | 0.002 | 0.003 | 4.71E-01 | 0.017 | 0.002 | 3.40E-15 |
| rs42210 | 5 | 166408788 | G | C | -0.003 | 0.003 | 2.94E-01 | -0.014 | 0.002 | 7.30E-09 |
| rs2336895 | 5 | 166990695 | G | A | 0.006 | 0.003 | 4.52E-02 | 0.012 | 0.002 | 4.60E-08 |
| rs72828890 | 5 | 167806836 | C | T | 0.005 | 0.005 | 2.90E-01 | 0.019 | 0.003 | 4.90E-09 |
| rs255292 | 5 | 172580866 | C | A | 0.001 | 0.003 | 7.31E-01 | -0.012 | 0.002 | 1.70E-08 |
| rs72834698 | 6 | 26176517 | G | A | 0.020 | 0.004 | 2.97E-06 | 0.023 | 0.003 | 2.70E-13 |
| rs3135044 | 6 | 31592820 | C | T | -0.014 | 0.003 | 2.84E-06 | -0.015 | 0.002 | 8.70E-11 |
| rs10947452 | 6 | 33803752 | T | C | 0.013 | 0.003 | 2.93E-05 | 0.014 | 0.002 | 6.40E-10 |
| rs9471333 | 6 | 40362023 | C | T | 0.023 | 0.003 | 4.20E-15 | 0.013 | 0.002 | 1.50E-09 |
| rs4523073 | 6 | 67550288 | A | G | -0.001 | 0.003 | 8.57E-01 | -0.014 | 0.002 | 5.00E-10 |
| rs6905544 | 6 | 98411631 | A | G | -0.020 | 0.003 | 5.21E-15 | -0.019 | 0.002 | 8.50E-18 |
| rs17789218 | 6 | 100600097 | T | C | -0.015 | 0.003 | 3.33E-08 | 0.019 | 0.003 | 1.40E-13 |
| rs78394231 | 6 | 107649123 | T | C | -0.001 | 0.005 | 9.15E-01 | -0.021 | 0.004 | 9.70E-09 |
| rs2184364 | 6 | 143183596 | A | G | 0.007 | 0.003 | 3.28E-02 | 0.016 | 0.003 | 3.00E-09 |
| rs9718104 | 6 | 166170539 | T | G | -0.004 | 0.006 | 5.44E-01 | -0.041 | 0.005 | 9.30E-19 |
| rs17568389 | 7 | 8091876 | T | A | 0.005 | 0.003 | 2.94E-02 | 0.015 | 0.002 | 8.60E-13 |
| rs10234444 | 7 | 41828527 | G | A | 0.005 | 0.003 | 1.33E-01 | 0.016 | 0.003 | 2.90E-08 |
| rs6973656 | 7 | 77422583 | A | G | -0.009 | 0.003 | 7.23E-04 | -0.013 | 0.002 | 1.00E-09 |
| rs973734 | 7 | 89387578 | C | A | 0.007 | 0.004 | 5.80E-02 | 0.017 | 0.003 | 1.50E-08 |
| rs3137 | 7 | 99130875 | G | A | 0.016 | 0.003 | 7.52E-07 | 0.017 | 0.003 | 1.40E-08 |
| rs2694151 | 7 | 101902180 | C | T | 0.012 | 0.006 | 4.91E-02 | 0.026 | 0.005 | 2.30E-08 |
| rs62471080 | 7 | 109128060 | G | C | -0.009 | 0.003 | 1.55E-03 | -0.013 | 0.002 | 3.70E-09 |
| rs7788008 | 7 | 112972483 | G | A | 0.017 | 0.003 | 1.16E-11 | 0.013 | 0.002 | 5.90E-09 |
| rs10246289 | 7 | 115544763 | A | G | 0.009 | 0.004 | 4.56E-02 | 0.019 | 0.003 | 1.70E-08 |
| rs11763734 | 7 | 126400910 | A | C | -0.005 | 0.003 | 4.36E-02 | -0.012 | 0.002 | 8.20E-09 |
| rs7834121 | 8 | 10770255 | G | T | 0.017 | 0.003 | 1.56E-09 | -0.014 | 0.002 | 1.10E-10 |
| rs1421334 | 8 | 30865733 | A | C | 0.016 | 0.003 | 1.03E-10 | 0.017 | 0.002 | 2.20E-15 |
| rs6996198 | 8 | 65463442 | C | T | -0.014 | 0.003 | 7.41E-05 | -0.016 | 0.003 | 3.50E-08 |
| rs6472942 | 8 | 76827190 | T | C | 0.016 | 0.003 | 1.76E-10 | -0.013 | 0.002 | 1.80E-09 |
| rs11985394 | 8 | 92667521 | T | A | 0.000 | 0.003 | 8.98E-01 | 0.012 | 0.002 | 1.30E-08 |
| rs72671494 | 8 | 93195457 | T | C | -0.007 | 0.004 | 9.67E-02 | -0.017 | 0.003 | 4.50E-08 |
| rs12541615 | 8 | 118870151 | T | C | -0.020 | 0.004 | 6.59E-08 | -0.018 | 0.003 | 3.90E-10 |
| rs2717559 | 8 | 143882420 | A | G | 0.005 | 0.003 | 1.27E-01 | 0.012 | 0.002 | 1.50E-08 |
| rs2616830 | 9 | 1721385 | G | A | -0.003 | 0.003 | 2.90E-01 | 0.016 | 0.002 | 2.90E-14 |
| rs2291681 | 9 | 14790841 | G | T | 0.002 | 0.003 | 5.57E-01 | 0.013 | 0.002 | 7.90E-09 |
| rs34864022 | 9 | 22609110 | A | G | 0.001 | 0.006 | 8.62E-01 | -0.026 | 0.004 | 1.40E-09 |
| rs12554512 | 9 | 23352293 | T | C | -0.007 | 0.003 | 1.07E-02 | 0.021 | 0.002 | 3.80E-21 |
| rs2791574 | 9 | 82212094 | T | G | -0.003 | 0.003 | 2.65E-01 | -0.013 | 0.002 | 3.00E-08 |
| rs870151 | 9 | 92230747 | T | A | -0.007 | 0.003 | 6.68E-03 | -0.016 | 0.002 | 8.80E-13 |
| rs2787374 | 9 | 103054951 | T | C | 0.009 | 0.003 | 6.60E-04 | 0.012 | 0.002 | 1.20E-08 |
| rs10739499 | 9 | 120514261 | C | G | 0.005 | 0.003 | 6.07E-02 | 0.014 | 0.002 | 1.50E-09 |
| rs7043521 | 9 | 124602779 | A | T | 0.008 | 0.003 | 7.13E-03 | 0.014 | 0.002 | 6.50E-11 |
| rs12342024 | 9 | 126134232 | G | T | -0.007 | 0.005 | 1.04E-01 | -0.021 | 0.003 | 1.70E-09 |
| rs4382592 | 9 | 134870755 | T | G | 0.010 | 0.003 | 2.84E-04 | 0.014 | 0.002 | 6.70E-09 |
| rs2073869 | 9 | 135763816 | C | T | 0.007 | 0.003 | 3.42E-02 | 0.019 | 0.003 | 1.50E-10 |
| 9:139926402_T_C | 9 | 139926402 | T | C | 0.000 | 0.003 | 9.02E-01 | -0.013 | 0.002 | 2.00E-08 |
| rs1623025 | 10 | 11164813 | G | C | -0.005 | 0.003 | 8.37E-02 | -0.012 | 0.002 | 1.40E-08 |
| rs1243182 | 10 | 21916728 | C | T | -0.015 | 0.003 | 6.12E-08 | -0.019 | 0.002 | 2.00E-15 |
| rs2045147 | 10 | 56660437 | A | G | 0.008 | 0.003 | 1.69E-03 | 0.013 | 0.002 | 5.90E-09 |
| rs10994943 | 10 | 63591413 | T | G | -0.001 | 0.003 | 8.58E-01 | 0.013 | 0.002 | 3.10E-09 |
| rs11201422 | 10 | 86924483 | T | C | 0.003 | 0.003 | 2.01E-01 | 0.013 | 0.002 | 3.40E-08 |
| rs10786658 | 10 | 103737193 | A | T | -0.003 | 0.003 | 3.99E-01 | -0.014 | 0.002 | 4.60E-11 |
| rs7089973 | 10 | 116569565 | C | A | -0.001 | 0.003 | 8.12E-01 | -0.013 | 0.002 | 2.40E-09 |
| rs11245482 | 10 | 126733546 | T | C | -0.007 | 0.003 | 9.03E-03 | -0.013 | 0.002 | 2.60E-09 |
| rs17727474 | 10 | 127182959 | C | T | 0.006 | 0.003 | 9.72E-02 | 0.018 | 0.003 | 3.10E-09 |
| rs12289262 | 11 | 12894758 | C | T | -0.005 | 0.003 | 7.18E-02 | -0.014 | 0.002 | 2.00E-08 |
| rs801733 | 11 | 65934549 | A | C | 0.014 | 0.003 | 3.00E-08 | 0.017 | 0.002 | 7.30E-14 |
| rs116175338 | 11 | 72467875 | G | T | -0.017 | 0.003 | 3.38E-10 | -0.014 | 0.003 | 1.40E-08 |
| rs12272012 | 11 | 84861286 | G | C | -0.007 | 0.007 | 3.08E-01 | -0.03 | 0.005 | 1.90E-08 |
| rs11020045 | 11 | 92487907 | A | C | -0.005 | 0.003 | 7.87E-02 | -0.013 | 0.002 | 1.60E-08 |
| rs17207890 | 11 | 95490754 | G | A | 0.004 | 0.003 | 9.60E-02 | 0.016 | 0.002 | 6.70E-12 |
| rs10791546 | 11 | 101830126 | C | T | 0.005 | 0.003 | 1.25E-01 | 0.012 | 0.002 | 4.10E-08 |
| rs328277 | 11 | 110414195 | C | A | -0.002 | 0.003 | 6.02E-01 | 0.012 | 0.002 | 4.50E-08 |
| rs648044 | 11 | 114030799 | A | G | -0.012 | 0.003 | 9.49E-05 | -0.014 | 0.002 | 1.00E-09 |
| rs11218575 | 11 | 122154576 | C | T | 0.002 | 0.003 | 5.43E-01 | 0.015 | 0.002 | 2.10E-12 |
| rs74802478 | 11 | 131966907 | G | A | 0.015 | 0.004 | 8.94E-05 | 0.017 | 0.003 | 3.50E-09 |
| rs4937842 | 11 | 133734542 | G | C | -0.007 | 0.003 | 7.97E-03 | -0.013 | 0.002 | 1.40E-08 |
| rs10772643 | 12 | 13415288 | C | T | 0.005 | 0.005 | 3.32E-01 | 0.025 | 0.003 | 1.30E-12 |
| rs74996610 | 12 | 24075007 | C | G | -0.007 | 0.007 | 3.27E-01 | -0.031 | 0.005 | 4.10E-09 |
| rs10771746 | 12 | 30791864 | C | T | -0.002 | 0.003 | 6.07E-01 | -0.014 | 0.002 | 2.50E-09 |
| rs10876864 | 12 | 56401085 | G | A | -0.013 | 0.003 | 3.82E-07 | -0.013 | 0.002 | 1.00E-09 |
| rs8756 | 12 | 66359752 | C | A | 0.009 | 0.002 | 9.38E-05 | -0.013 | 0.002 | 5.30E-10 |
| rs11179838 | 12 | 74321123 | C | G | 0.008 | 0.003 | 8.23E-03 | 0.013 | 0.002 | 2.10E-08 |
| rs2164744 | 12 | 109872039 | T | C | -0.003 | 0.003 | 4.07E-01 | -0.013 | 0.002 | 1.10E-08 |
| rs2173650 | 12 | 117525235 | G | T | 0.005 | 0.004 | 2.16E-01 | 0.018 | 0.003 | 4.60E-09 |
| rs1790135 | 12 | 123669235 | C | T | -0.006 | 0.003 | 4.99E-02 | -0.014 | 0.003 | 2.60E-08 |
| rs9563168 | 13 | 54247827 | G | A | 0.008 | 0.003 | 9.40E-03 | 0.018 | 0.003 | 4.30E-11 |
| rs9569734 | 13 | 58319476 | A | G | 0.017 | 0.003 | 6.37E-07 | 0.019 | 0.003 | 3.40E-10 |
| rs9528023 | 13 | 60226991 | C | T | 0.004 | 0.003 | 1.40E-01 | 0.013 | 0.002 | 3.80E-08 |
| rs56858768 | 13 | 86511730 | G | A | -0.016 | 0.003 | 8.41E-07 | -0.015 | 0.002 | 3.40E-10 |
| rs553397860 | 13 | 99051118 | A | G | 0.007 | 0.004 | 4.17E-02 | 0.017 | 0.003 | 8.90E-09 |
| rs7991062 | 13 | 100713194 | C | G | 0.002 | 0.003 | 3.93E-01 | -0.018 | 0.002 | 8.80E-15 |
| rs11629120 | 14 | 23458457 | T | C | 0.002 | 0.003 | 4.16E-01 | -0.013 | 0.002 | 1.10E-08 |
| rs405797 | 14 | 26972834 | T | A | -0.009 | 0.003 | 2.78E-03 | -0.015 | 0.003 | 2.80E-09 |
| rs1022785 | 14 | 29571653 | G | A | 0.004 | 0.004 | 2.36E-01 | 0.018 | 0.003 | 7.10E-09 |
| rs10145592 | 14 | 94287215 | C | G | -0.007 | 0.003 | 5.31E-03 | -0.015 | 0.002 | 1.80E-11 |
| rs7157001 | 14 | 99749484 | A | G | -0.012 | 0.003 | 3.59E-04 | -0.014 | 0.002 | 2.30E-08 |
| rs8043253 | 15 | 41503730 | C | T | -0.004 | 0.003 | 1.07E-01 | -0.012 | 0.002 | 3.40E-08 |
| rs2460 | 15 | 53073084 | G | A | -0.013 | 0.003 | 3.33E-06 | -0.015 | 0.002 | 5.00E-10 |
| rs4775373 | 15 | 61476936 | T | C | 0.006 | 0.003 | 4.06E-02 | 0.013 | 0.002 | 4.70E-09 |
| rs61331678 | 15 | 77995949 | G | C | 0.009 | 0.003 | 1.25E-03 | 0.015 | 0.002 | 6.10E-12 |
| rs35574015 | 16 | 15146061 | T | C | 0.013 | 0.003 | 3.97E-05 | -0.013 | 0.002 | 3.00E-08 |
| rs7189927 | 16 | 28913787 | T | C | 0.029 | 0.003 | 1.16E-29 | 0.015 | 0.002 | 3.40E-11 |
| rs749671 | 16 | 31088347 | G | A | 0.014 | 0.002 | 2.72E-09 | 0.016 | 0.002 | 2.70E-12 |
| rs7184800 | 16 | 53509131 | G | A | -0.005 | 0.003 | 1.46E-01 | 0.017 | 0.002 | 8.20E-13 |
| rs2447098 | 17 | 2277720 | C | A | 0.000 | 0.003 | 9.21E-01 | -0.015 | 0.002 | 6.80E-12 |
| rs11654952 | 17 | 4813799 | T | G | -0.007 | 0.004 | 4.93E-02 | -0.017 | 0.003 | 4.30E-08 |
| rs9902312 | 17 | 65070304 | T | C | 0.004 | 0.003 | 1.59E-01 | 0.015 | 0.002 | 4.50E-11 |
| rs11657730 | 17 | 79372489 | C | T | 0.004 | 0.003 | 1.63E-01 | 0.013 | 0.002 | 1.10E-08 |
| rs303753 | 18 | 21074922 | G | A | -0.021 | 0.003 | 5.82E-15 | -0.014 | 0.002 | 2.70E-10 |
| rs11081851 | 18 | 31783262 | G | A | -0.001 | 0.003 | 7.09E-01 | -0.013 | 0.002 | 1.00E-08 |
| rs9964724 | 18 | 35159124 | C | T | -0.003 | 0.003 | 2.88E-01 | 0.018 | 0.002 | 3.30E-14 |
| rs1156541 | 18 | 39952989 | C | T | 0.007 | 0.003 | 1.56E-02 | 0.014 | 0.003 | 2.80E-08 |
| rs148544378 | 18 | 40323567 | C | T | -0.005 | 0.010 | 6.59E-01 | 0.053 | 0.008 | 2.20E-12 |
| rs17512836 | 18 | 53194961 | T | C | 0.013 | 0.009 | 1.69E-01 | 0.042 | 0.007 | 7.00E-10 |
| rs7248205 | 19 | 10770305 | C | T | 0.007 | 0.003 | 2.55E-02 | 0.014 | 0.002 | 3.40E-10 |
| rs111901094 | 19 | 19513570 | G | T | -0.006 | 0.004 | 1.14E-01 | -0.017 | 0.003 | 2.00E-09 |
| rs6131281 | 20 | 11891724 | C | T | 0.005 | 0.003 | 1.13E-01 | 0.016 | 0.002 | 3.10E-13 |
| rs6141814 | 20 | 31368960 | C | A | 0.005 | 0.003 | 7.01E-02 | -0.014 | 0.002 | 1.30E-09 |
| rs4810312 | 20 | 39968707 | C | G | 0.000 | 0.003 | 9.63E-01 | -0.013 | 0.002 | 1.20E-08 |
| rs6124476 | 20 | 41202830 | T | C | 0.008 | 0.003 | 9.86E-04 | 0.012 | 0.002 | 1.80E-08 |
| rs6031440 | 20 | 42809317 | A | T | 0.005 | 0.003 | 3.37E-02 | 0.012 | 0.002 | 1.80E-08 |
| rs55700114 | 20 | 43717080 | G | A | -0.007 | 0.003 | 3.08E-02 | -0.014 | 0.002 | 2.10E-09 |
| rs6125906 | 20 | 48729803 | C | A | 0.003 | 0.005 | 5.42E-01 | -0.021 | 0.004 | 3.90E-08 |
| rs56103247 | 20 | 62483184 | C | T | 0.027 | 0.007 | 3.07E-05 | 0.03 | 0.005 | 3.80E-10 |
| rs10427502 | 21 | 40654840 | G | A | 0.014 | 0.003 | 1.40E-06 | 0.014 | 0.002 | 8.60E-10 |
| rs7609 | 22 | 29951650 | A | T | -0.001 | 0.003 | 6.81E-01 | 0.015 | 0.003 | 3.60E-09 |
| rs2092829 | 22 | 31780514 | G | A | -0.001 | 0.003 | 6.28E-01 | 0.014 | 0.002 | 1.30E-08 |
| **5) MR of BMI (women) on leisure television use** | | | | | | | | | | |
| SNP | CHR | POS | EA | OA | beta_bmi | se_bmi | pval_bmi | beta_tv | se_tv | pval_tv |
| rs79113395 | 1 | 1590521 | A | G | -0.021 | 0.003 | 1.45E-12 | -0.007 | 0.002 | 4.50E-03 |
| rs10909880 | 1 | 2727804 | T | C | -0.012 | 0.002 | 1.97E-08 | 0.000 | 0.002 | 9.30E-01 |
| rs78886584 | 1 | 16859325 | A | G | -0.015 | 0.003 | 1.30E-08 | -0.007 | 0.002 | 2.40E-03 |
| rs4295917 | 1 | 32173360 | A | G | 0.014 | 0.002 | 1.62E-08 | 0.006 | 0.002 | 8.50E-03 |
| rs112646560 | 1 | 39560250 | T | C | 0.020 | 0.003 | 1.14E-09 | 0.005 | 0.003 | 7.60E-02 |
| rs2984618 | 1 | 47690438 | T | G | 0.017 | 0.002 | 2.21E-14 | 0.001 | 0.002 | 6.40E-01 |
| rs657452 | 1 | 49589847 | A | G | 0.020 | 0.002 | 1.14E-19 | -0.004 | 0.002 | 7.00E-02 |
| rs12140153 | 1 | 62579891 | T | G | -0.033 | 0.005 | 2.87E-12 | -0.014 | 0.004 | 2.20E-04 |
| rs2568958 | 1 | 72765116 | A | G | 0.023 | 0.002 | 1.32E-24 | -0.007 | 0.002 | 1.50E-03 |
| rs6669189 | 1 | 75011358 | T | C | 0.021 | 0.002 | 4.69E-21 | -0.002 | 0.002 | 3.30E-01 |
| rs34517439 | 1 | 78450517 | A | C | 0.039 | 0.004 | 2.00E-21 | 0.020 | 0.003 | 8.60E-10 |
| rs12089815 | 1 | 91189933 | A | G | -0.015 | 0.003 | 6.02E-09 | -0.011 | 0.002 | 6.10E-07 |
| rs11165643 | 1 | 96924097 | T | C | 0.018 | 0.002 | 1.71E-16 | 0.004 | 0.002 | 8.70E-02 |
| rs4421623 | 1 | 98316285 | T | G | 0.018 | 0.003 | 1.13E-11 | 0.019 | 0.003 | 3.40E-13 |
| rs7550711 | 1 | 110082886 | T | C | 0.064 | 0.007 | 3.37E-21 | -0.001 | 0.007 | 8.90E-01 |
| rs197374 | 1 | 112289983 | T | C | 0.017 | 0.002 | 2.82E-13 | 0.009 | 0.002 | 5.50E-05 |
| rs61813324 | 1 | 156049877 | T | C | 0.030 | 0.004 | 3.60E-14 | 0.015 | 0.003 | 4.30E-06 |
| rs1750307 | 1 | 156488420 | A | T | 0.014 | 0.002 | 8.22E-09 | 0.002 | 0.002 | 3.40E-01 |
| rs12564992 | 1 | 174478100 | A | G | -0.021 | 0.004 | 2.37E-09 | 0.008 | 0.003 | 2.00E-02 |
| rs543874 | 1 | 177889480 | A | G | -0.058 | 0.003 | 8.70E-100 | -0.004 | 0.003 | 9.30E-02 |
| rs10920678 | 1 | 190239907 | A | G | 0.016 | 0.002 | 7.46E-14 | 0.002 | 0.002 | 3.20E-01 |
| rs4474229 | 1 | 195037001 | A | G | -0.017 | 0.003 | 1.64E-10 | -0.003 | 0.002 | 1.20E-01 |
| rs2820295 | 1 | 201800868 | A | G | 0.021 | 0.002 | 8.03E-19 | 0.010 | 0.002 | 8.70E-06 |
| rs17014375 | 1 | 209543560 | T | G | -0.019 | 0.003 | 1.46E-08 | -0.001 | 0.003 | 7.20E-01 |
| rs12042959 | 1 | 243533273 | A | G | 0.019 | 0.003 | 2.33E-09 | 0.004 | 0.003 | 2.20E-01 |
| rs62106258 | 2 | 417167 | T | C | 0.099 | 0.006 | 8.72E-58 | -0.007 | 0.005 | 1.90E-01 |
| rs6548237 | 2 | 621461 | A | C | -0.058 | 0.003 | 4.43E-89 | -0.005 | 0.003 | 1.00E-01 |
| rs7571496 | 2 | 6169351 | A | G | 0.015 | 0.003 | 1.45E-08 | 0.004 | 0.002 | 1.20E-01 |
| rs7601870 | 2 | 15384392 | A | G | -0.013 | 0.002 | 1.23E-08 | -0.006 | 0.002 | 9.10E-03 |
| rs10182181 | 2 | 25150296 | A | G | -0.036 | 0.002 | 5.00E-63 | -0.001 | 0.002 | 6.90E-01 |
| rs10048652 | 2 | 35449306 | T | C | 0.014 | 0.002 | 3.47E-09 | 0.001 | 0.002 | 7.30E-01 |
| rs786420 | 2 | 44719893 | T | C | 0.016 | 0.003 | 8.84E-09 | -0.001 | 0.003 | 6.90E-01 |
| rs35722922 | 2 | 47002226 | A | G | 0.019 | 0.003 | 3.65E-12 | -0.002 | 0.002 | 4.10E-01 |
| rs1421598 | 2 | 50264710 | A | C | -0.019 | 0.003 | 6.78E-10 | -0.005 | 0.003 | 9.40E-02 |
| rs7601895 | 2 | 55281901 | C | G | 0.016 | 0.003 | 9.61E-11 | 0.010 | 0.002 | 3.70E-05 |
| rs1861412 | 2 | 58893065 | A | G | -0.022 | 0.002 | 9.13E-21 | -0.007 | 0.002 | 6.90E-04 |
| rs6728037 | 2 | 59297346 | A | G | 0.021 | 0.002 | 6.49E-19 | 0.006 | 0.002 | 7.80E-03 |
| rs2861685 | 2 | 67837553 | T | C | 0.018 | 0.003 | 1.05E-11 | 0.001 | 0.002 | 7.60E-01 |
| rs12714199 | 2 | 86812549 | T | C | -0.015 | 0.002 | 5.12E-10 | -0.002 | 0.002 | 4.80E-01 |
| rs11677607 | 2 | 100751150 | T | C | -0.024 | 0.003 | 2.87E-19 | -0.018 | 0.003 | 9.50E-13 |
| rs1457457 | 2 | 104340148 | A | T | 0.014 | 0.002 | 3.79E-10 | 0.012 | 0.002 | 1.20E-08 |
| rs6739199 | 2 | 105452326 | T | C | 0.016 | 0.003 | 3.20E-10 | 0.014 | 0.002 | 3.40E-09 |
| rs1984559 | 2 | 135678888 | A | G | 0.016 | 0.003 | 3.27E-08 | 0.007 | 0.003 | 9.40E-03 |
| rs13002158 | 2 | 144021712 | A | G | -0.021 | 0.003 | 1.15E-12 | -0.003 | 0.003 | 2.90E-01 |
| rs76900466 | 2 | 145057760 | T | G | 0.023 | 0.004 | 4.53E-08 | 0.015 | 0.003 | 7.50E-06 |
| rs12692596 | 2 | 161265910 | T | C | 0.015 | 0.002 | 9.74E-11 | 0.009 | 0.002 | 3.60E-05 |
| rs12692738 | 2 | 165558252 | T | C | -0.016 | 0.003 | 2.82E-10 | 0.000 | 0.003 | 9.80E-01 |
| rs7588437 | 2 | 181575281 | A | G | -0.018 | 0.002 | 8.38E-15 | -0.006 | 0.002 | 1.40E-02 |
| rs7594237 | 2 | 198187164 | A | T | -0.014 | 0.003 | 1.81E-08 | 0.001 | 0.002 | 6.20E-01 |
| rs4482463 | 2 | 205375909 | A | C | -0.037 | 0.004 | 5.21E-18 | -0.001 | 0.004 | 7.80E-01 |
| rs11692326 | 2 | 208263279 | T | C | 0.016 | 0.003 | 2.04E-09 | 0.004 | 0.003 | 9.40E-02 |
| rs4673553 | 2 | 211608379 | T | G | -0.015 | 0.002 | 2.59E-11 | -0.006 | 0.002 | 3.50E-03 |
| rs12612009 | 2 | 228982603 | T | G | -0.014 | 0.002 | 3.78E-09 | -0.007 | 0.002 | 1.30E-03 |
| rs6720868 | 2 | 230663576 | T | C | 0.017 | 0.002 | 4.65E-12 | 0.006 | 0.002 | 1.10E-02 |
| rs1801282 | 3 | 12393125 | C | G | -0.018 | 0.003 | 4.72E-08 | -0.006 | 0.003 | 8.00E-02 |
| rs2470520 | 3 | 15762920 | T | C | -0.013 | 0.002 | 4.16E-08 | -0.001 | 0.002 | 8.00E-01 |
| rs4858223 | 3 | 20581149 | T | C | -0.016 | 0.003 | 8.26E-11 | -0.001 | 0.002 | 6.90E-01 |
| rs13062093 | 3 | 35667057 | T | G | -0.014 | 0.002 | 8.00E-09 | 0.001 | 0.002 | 6.20E-01 |
| rs1799923 | 3 | 42306294 | A | G | -0.024 | 0.004 | 5.10E-12 | -0.002 | 0.003 | 4.90E-01 |
| rs13076052 | 3 | 44456573 | C | G | -0.017 | 0.003 | 1.30E-08 | -0.009 | 0.002 | 4.50E-04 |
| rs6446187 | 3 | 49907111 | A | C | 0.028 | 0.003 | 1.22E-26 | 0.025 | 0.002 | 3.60E-32 |
| rs1916801 | 3 | 61187046 | A | T | 0.017 | 0.002 | 1.75E-14 | 0.007 | 0.002 | 9.70E-04 |
| rs1452075 | 3 | 62481063 | T | C | 0.016 | 0.003 | 1.47E-10 | 0.002 | 0.002 | 3.70E-01 |
| rs925018 | 3 | 62713143 | C | G | -0.014 | 0.002 | 9.81E-10 | -0.004 | 0.002 | 7.60E-02 |
| rs7647242 | 3 | 82642734 | T | C | 0.015 | 0.002 | 3.92E-10 | 0.002 | 0.002 | 4.50E-01 |
| rs13068138 | 3 | 85809425 | T | C | 0.023 | 0.003 | 3.60E-17 | 0.010 | 0.003 | 1.60E-04 |
| rs1949197 | 3 | 86273850 | A | G | 0.014 | 0.002 | 3.04E-09 | 0.006 | 0.002 | 4.30E-03 |
| rs1492014 | 3 | 94071481 | T | C | -0.015 | 0.002 | 2.84E-10 | -0.013 | 0.002 | 1.30E-09 |
| rs1436348 | 3 | 104612668 | A | G | -0.013 | 0.002 | 6.31E-09 | -0.004 | 0.002 | 9.70E-02 |
| rs1289736 | 3 | 107950892 | T | C | 0.014 | 0.002 | 1.43E-09 | 0.007 | 0.002 | 9.50E-04 |
| rs17619973 | 3 | 114417675 | A | G | 0.024 | 0.004 | 1.29E-08 | 0.019 | 0.004 | 4.40E-06 |
| rs7428670 | 3 | 131579810 | C | G | 0.020 | 0.003 | 3.09E-15 | 0.003 | 0.002 | 2.20E-01 |
| rs7621025 | 3 | 136272246 | T | C | -0.020 | 0.003 | 1.62E-15 | -0.004 | 0.002 | 1.30E-01 |
| rs16851483 | 3 | 141275436 | T | G | 0.036 | 0.005 | 2.28E-15 | 0.013 | 0.004 | 2.90E-03 |
| rs355754 | 3 | 154024165 | T | C | -0.014 | 0.002 | 4.26E-10 | -0.003 | 0.002 | 1.60E-01 |
| rs247975 | 3 | 173107443 | T | C | -0.018 | 0.002 | 3.62E-15 | -0.005 | 0.002 | 1.80E-02 |
| rs10513801 | 3 | 185822353 | T | G | 0.035 | 0.003 | 1.26E-27 | -0.009 | 0.003 | 3.70E-03 |
| rs4677812 | 3 | 194863794 | A | C | -0.014 | 0.003 | 9.38E-09 | -0.009 | 0.002 | 1.10E-04 |
| rs994596 | 4 | 18459828 | T | C | 0.015 | 0.003 | 1.02E-09 | 0.001 | 0.002 | 5.30E-01 |
| rs34811474 | 4 | 25408838 | A | G | -0.032 | 0.003 | 5.19E-24 | -0.015 | 0.003 | 2.30E-09 |
| rs73213484 | 4 | 28489339 | A | T | 0.025 | 0.004 | 2.28E-11 | 0.002 | 0.003 | 4.40E-01 |
| rs337637 | 4 | 38604470 | A | G | -0.013 | 0.002 | 3.04E-08 | -0.002 | 0.002 | 3.40E-01 |
| rs10938397 | 4 | 45182527 | A | G | -0.032 | 0.002 | 1.66E-45 | -0.011 | 0.002 | 9.30E-07 |
| rs2271046 | 4 | 52752812 | A | T | -0.014 | 0.003 | 2.38E-08 | -0.014 | 0.002 | 8.10E-09 |
| rs11727273 | 4 | 55509748 | T | C | -0.017 | 0.003 | 1.28E-10 | -0.005 | 0.002 | 1.70E-02 |
| rs11945861 | 4 | 65700865 | A | G | -0.015 | 0.003 | 3.13E-08 | 0.002 | 0.003 | 3.40E-01 |
| rs10002111 | 4 | 67815504 | A | G | 0.017 | 0.003 | 9.26E-10 | 0.007 | 0.003 | 4.30E-03 |
| rs4148155 | 4 | 89054667 | A | G | 0.020 | 0.004 | 6.20E-09 | 0.009 | 0.003 | 6.60E-03 |
| rs12645001 | 4 | 96018337 | A | G | -0.014 | 0.002 | 4.11E-09 | 0.001 | 0.002 | 7.30E-01 |
| rs13107325 | 4 | 103188709 | T | C | 0.045 | 0.004 | 9.33E-25 | 0.029 | 0.004 | 1.50E-12 |
| rs4834272 | 4 | 113313986 | T | C | -0.014 | 0.002 | 8.24E-10 | 0.002 | 0.002 | 4.40E-01 |
| rs1403846 | 4 | 119101723 | T | C | 0.016 | 0.003 | 1.62E-08 | 0.008 | 0.003 | 3.50E-03 |
| rs4864201 | 4 | 130731284 | T | C | 0.013 | 0.002 | 6.34E-09 | 0.004 | 0.002 | 1.10E-01 |
| rs57800857 | 4 | 140863365 | A | C | 0.019 | 0.003 | 1.07E-11 | 0.004 | 0.002 | 5.20E-02 |
| rs35107973 | 4 | 143125948 | T | C | 0.023 | 0.004 | 7.66E-09 | 0.002 | 0.003 | 5.50E-01 |
| rs750090 | 4 | 152931436 | T | C | 0.016 | 0.002 | 7.40E-11 | -0.004 | 0.002 | 1.00E-01 |
| rs13110266 | 4 | 162129844 | A | G | -0.014 | 0.002 | 1.55E-10 | -0.006 | 0.002 | 7.90E-03 |
| rs1491333 | 4 | 172186288 | T | G | -0.015 | 0.003 | 9.85E-09 | -0.002 | 0.003 | 4.90E-01 |
| rs6890310 | 5 | 27193573 | A | G | -0.015 | 0.003 | 1.38E-09 | -0.003 | 0.002 | 1.90E-01 |
| rs782971 | 5 | 43124688 | A | G | -0.017 | 0.003 | 4.89E-12 | -0.001 | 0.002 | 5.90E-01 |
| rs1812587 | 5 | 62991802 | T | G | 0.015 | 0.003 | 6.95E-09 | 0.016 | 0.002 | 5.10E-13 |
| rs10471636 | 5 | 63979856 | A | G | -0.017 | 0.002 | 4.66E-13 | 0.002 | 0.002 | 4.60E-01 |
| rs2112347 | 5 | 75015242 | T | G | 0.031 | 0.002 | 2.13E-42 | 0.007 | 0.002 | 2.10E-03 |
| rs6870983 | 5 | 87697533 | T | C | -0.021 | 0.003 | 9.61E-16 | -0.009 | 0.003 | 4.70E-04 |
| rs141729694 | 5 | 87999371 | T | C | 0.039 | 0.005 | 2.53E-14 | 0.000 | 0.004 | 9.90E-01 |
| rs159032 | 5 | 94206202 | T | C | 0.015 | 0.003 | 4.52E-08 | 0.001 | 0.003 | 8.10E-01 |
| rs2161097 | 5 | 103945178 | T | C | 0.013 | 0.002 | 2.31E-08 | 0.004 | 0.002 | 8.40E-02 |
| rs158186 | 5 | 107440856 | A | G | -0.029 | 0.003 | 3.23E-20 | -0.002 | 0.003 | 5.30E-01 |
| rs4895231 | 5 | 119388659 | C | G | 0.014 | 0.002 | 9.26E-10 | 0.008 | 0.002 | 4.60E-04 |
| rs13174863 | 5 | 139080745 | A | G | -0.027 | 0.003 | 4.53E-18 | -0.002 | 0.003 | 6.00E-01 |
| rs4569924 | 5 | 153540025 | T | C | 0.016 | 0.002 | 7.44E-13 | -0.003 | 0.002 | 2.10E-01 |
| rs6556810 | 5 | 164585949 | T | C | -0.012 | 0.002 | 4.98E-08 | -0.001 | 0.002 | 6.40E-01 |
| rs2053682 | 5 | 170599327 | A | C | 0.018 | 0.003 | 1.89E-13 | 0.002 | 0.002 | 2.90E-01 |
| rs2228213 | 6 | 12124855 | A | G | -0.015 | 0.002 | 9.21E-11 | -0.005 | 0.002 | 3.30E-02 |
| rs9367369 | 6 | 13189941 | A | G | 0.017 | 0.003 | 2.32E-09 | 0.003 | 0.002 | 2.40E-01 |
| rs3806114 | 6 | 20482335 | A | G | -0.014 | 0.002 | 4.49E-09 | -0.009 | 0.002 | 1.30E-04 |
| rs2178899 | 6 | 31606756 | A | T | 0.025 | 0.003 | 7.16E-14 | 0.005 | 0.003 | 1.30E-01 |
| rs11751591 | 6 | 33794215 | A | G | -0.019 | 0.003 | 1.16E-08 | -0.007 | 0.003 | 2.70E-02 |
| rs2814992 | 6 | 34617144 | A | G | -0.029 | 0.002 | 1.25E-35 | -0.007 | 0.002 | 2.60E-03 |
| rs1579557 | 6 | 40371918 | T | C | 0.021 | 0.003 | 3.09E-17 | 0.012 | 0.002 | 6.60E-07 |
| rs943005 | 6 | 50865820 | T | C | 0.040 | 0.003 | 1.99E-43 | 0.003 | 0.003 | 3.10E-01 |
| rs2465043 | 6 | 51180765 | A | G | -0.014 | 0.002 | 3.22E-09 | -0.002 | 0.002 | 2.90E-01 |
| rs62412826 | 6 | 61881167 | T | C | 0.033 | 0.006 | 7.98E-09 | -0.003 | 0.005 | 5.10E-01 |
| rs209416 | 6 | 83534729 | A | T | 0.017 | 0.002 | 8.71E-12 | -0.003 | 0.002 | 2.50E-01 |
| rs13209872 | 6 | 97753223 | C | G | -0.014 | 0.002 | 6.07E-09 | -0.003 | 0.002 | 2.60E-01 |
| rs12202969 | 6 | 98576223 | A | G | -0.015 | 0.002 | 1.93E-10 | -0.017 | 0.002 | 1.90E-15 |
| rs1417665 | 6 | 104816944 | T | C | 0.020 | 0.003 | 5.68E-12 | 0.005 | 0.003 | 5.10E-02 |
| rs768023 | 6 | 108876002 | A | G | 0.019 | 0.002 | 1.38E-16 | 0.005 | 0.002 | 2.00E-02 |
| rs2357760 | 6 | 120213880 | A | G | 0.016 | 0.002 | 1.09E-11 | 0.006 | 0.002 | 1.60E-02 |
| rs11754747 | 6 | 141494602 | T | C | 0.015 | 0.003 | 1.27E-08 | 0.004 | 0.002 | 1.60E-01 |
| rs198665 | 6 | 143154886 | A | C | 0.013 | 0.002 | 4.42E-09 | 0.003 | 0.002 | 1.20E-01 |
| rs2185027 | 6 | 153381622 | A | C | -0.015 | 0.002 | 1.11E-10 | -0.009 | 0.002 | 2.10E-04 |
| rs12207125 | 6 | 154301575 | A | G | 0.018 | 0.003 | 7.67E-09 | -0.003 | 0.003 | 2.00E-01 |
| rs13213408 | 6 | 162998049 | A | T | 0.020 | 0.003 | 4.33E-09 | 0.001 | 0.003 | 7.00E-01 |
| rs6973700 | 7 | 1856669 | A | G | 0.019 | 0.003 | 1.77E-09 | 0.010 | 0.003 | 1.60E-04 |
| rs836525 | 7 | 6465909 | T | C | 0.018 | 0.003 | 9.75E-09 | 0.003 | 0.003 | 3.60E-01 |
| rs4307239 | 7 | 24354300 | A | G | -0.014 | 0.002 | 1.31E-09 | -0.001 | 0.002 | 8.00E-01 |
| rs11971098 | 7 | 26699061 | A | G | -0.024 | 0.004 | 6.49E-09 | 0.005 | 0.004 | 2.40E-01 |
| rs10499694 | 7 | 50614173 | A | G | 0.014 | 0.002 | 3.99E-10 | -0.003 | 0.002 | 1.30E-01 |
| rs10237317 | 7 | 70045941 | A | G | -0.014 | 0.002 | 6.82E-10 | 0.000 | 0.002 | 9.20E-01 |
| rs73124396 | 7 | 71579606 | T | C | 0.019 | 0.003 | 1.05E-08 | 0.003 | 0.003 | 2.90E-01 |
| rs2267812 | 7 | 74138121 | A | C | -0.017 | 0.003 | 1.74E-09 | -0.006 | 0.003 | 3.50E-02 |
| rs1167800 | 7 | 75176196 | A | G | 0.020 | 0.002 | 3.66E-19 | 0.008 | 0.002 | 1.10E-04 |
| rs7805441 | 7 | 78121458 | T | C | 0.015 | 0.002 | 2.01E-10 | 0.007 | 0.002 | 1.60E-03 |
| rs2528531 | 7 | 93236510 | A | C | -0.015 | 0.002 | 1.35E-09 | 0.004 | 0.002 | 1.00E-01 |
| rs10271582 | 7 | 99121950 | A | G | 0.019 | 0.003 | 8.41E-09 | 0.017 | 0.003 | 1.90E-08 |
| rs2299383 | 7 | 103418846 | T | C | 0.018 | 0.002 | 2.73E-16 | -0.002 | 0.002 | 2.90E-01 |
| rs7788008 | 7 | 112972483 | A | G | -0.017 | 0.002 | 1.09E-13 | -0.013 | 0.002 | 5.90E-09 |
| rs2045293 | 7 | 114350102 | T | C | 0.019 | 0.002 | 4.06E-16 | 0.004 | 0.002 | 6.70E-02 |
| rs2289705 | 7 | 121983843 | A | G | -0.015 | 0.003 | 1.47E-08 | 0.002 | 0.003 | 5.20E-01 |
| rs2968864 | 7 | 150622162 | T | C | 0.016 | 0.003 | 9.35E-10 | 0.004 | 0.002 | 7.70E-02 |
| rs4240673 | 8 | 10787612 | T | C | 0.018 | 0.002 | 1.03E-15 | -0.011 | 0.002 | 1.70E-07 |
| rs2959592 | 8 | 17002725 | T | C | 0.017 | 0.003 | 6.26E-10 | 0.006 | 0.003 | 3.30E-02 |
| rs1362910 | 8 | 30856464 | A | G | 0.013 | 0.002 | 3.03E-09 | 0.016 | 0.002 | 1.30E-13 |
| rs4320543 | 8 | 34422967 | A | G | -0.013 | 0.002 | 2.95E-08 | -0.010 | 0.002 | 6.30E-06 |
| rs12681792 | 8 | 62054463 | A | C | 0.018 | 0.003 | 2.61E-10 | 0.007 | 0.003 | 1.50E-02 |
| rs7827848 | 8 | 64790774 | A | T | 0.015 | 0.002 | 1.88E-11 | 0.009 | 0.002 | 8.20E-05 |
| rs1808629 | 8 | 73435964 | A | G | -0.023 | 0.003 | 5.76E-16 | -0.009 | 0.002 | 1.80E-04 |
| rs17405819 | 8 | 76806584 | T | C | 0.022 | 0.002 | 7.81E-20 | -0.008 | 0.002 | 3.50E-04 |
| rs12680842 | 8 | 95582606 | A | G | 0.013 | 0.002 | 3.36E-08 | 0.007 | 0.002 | 3.60E-03 |
| rs2721965 | 8 | 116662038 | A | C | 0.019 | 0.002 | 2.10E-14 | 0.006 | 0.002 | 6.60E-03 |
| rs10099330 | 8 | 143383694 | A | G | -0.013 | 0.002 | 9.94E-09 | -0.007 | 0.002 | 9.80E-04 |
| rs10124645 | 9 | 11321119 | A | G | 0.014 | 0.002 | 4.98E-09 | 0.007 | 0.002 | 2.70E-03 |
| rs10756714 | 9 | 15885041 | A | G | 0.021 | 0.002 | 1.46E-19 | 0.008 | 0.002 | 4.20E-04 |
| rs10962550 | 9 | 16720329 | C | G | 0.019 | 0.003 | 1.70E-10 | 0.005 | 0.003 | 1.10E-01 |
| rs16912921 | 9 | 28413461 | A | C | 0.025 | 0.002 | 1.56E-26 | 0.005 | 0.002 | 4.70E-02 |
| rs973345 | 9 | 29713169 | T | C | 0.014 | 0.002 | 1.09E-09 | 0.005 | 0.002 | 2.20E-02 |
| rs563132 | 9 | 37245476 | A | T | 0.018 | 0.002 | 1.96E-14 | -0.006 | 0.002 | 4.50E-03 |
| rs10116186 | 9 | 80532374 | A | G | -0.015 | 0.003 | 4.10E-08 | -0.003 | 0.002 | 1.70E-01 |
| rs10797115 | 9 | 92191256 | T | C | 0.016 | 0.002 | 4.07E-12 | 0.014 | 0.002 | 3.10E-10 |
| rs10992867 | 9 | 96461013 | A | G | 0.019 | 0.003 | 8.89E-13 | 0.006 | 0.002 | 1.90E-02 |
| rs10118701 | 9 | 103061366 | A | G | -0.018 | 0.002 | 2.20E-13 | -0.012 | 0.002 | 6.80E-07 |
| rs7024334 | 9 | 109072075 | T | G | 0.016 | 0.003 | 1.63E-09 | 0.000 | 0.003 | 8.90E-01 |
| rs1928295 | 9 | 120378483 | T | C | 0.016 | 0.002 | 2.85E-13 | 0.006 | 0.002 | 3.90E-03 |
| rs3829849 | 9 | 129390800 | T | C | 0.014 | 0.002 | 1.93E-09 | 0.001 | 0.002 | 6.30E-01 |
| rs10733682 | 9 | 129460914 | A | G | 0.016 | 0.002 | 1.85E-12 | -0.002 | 0.002 | 3.80E-01 |
| rs2267958 | 9 | 131015279 | A | G | -0.016 | 0.002 | 1.88E-11 | 0.001 | 0.002 | 5.20E-01 |
| rs1009473 | 9 | 138777312 | T | C | -0.013 | 0.002 | 3.36E-08 | -0.002 | 0.002 | 4.40E-01 |
| rs7084454 | 10 | 21821274 | A | G | 0.023 | 0.003 | 7.43E-20 | 0.016 | 0.002 | 1.00E-11 |
| rs34930419 | 10 | 33987329 | T | C | -0.046 | 0.008 | 1.37E-09 | 0.000 | 0.006 | 9.70E-01 |
| rs10823893 | 10 | 53677313 | A | G | 0.018 | 0.003 | 5.22E-11 | 0.009 | 0.002 | 7.00E-05 |
| rs10761785 | 10 | 65318766 | T | G | -0.017 | 0.002 | 4.17E-15 | -0.008 | 0.002 | 1.70E-04 |
| rs10824347 | 10 | 77645229 | A | T | 0.018 | 0.003 | 5.47E-11 | 0.006 | 0.003 | 1.10E-02 |
| rs7899106 | 10 | 87410904 | A | G | -0.038 | 0.005 | 7.65E-14 | -0.011 | 0.005 | 2.50E-02 |
| rs563296 | 10 | 99772404 | A | G | 0.018 | 0.002 | 8.85E-14 | 0.007 | 0.002 | 9.30E-04 |
| rs3977755 | 10 | 104420210 | T | C | -0.014 | 0.003 | 8.50E-09 | -0.002 | 0.002 | 4.50E-01 |
| rs6585198 | 10 | 114762237 | A | G | -0.015 | 0.002 | 1.22E-12 | -0.001 | 0.002 | 6.80E-01 |
| rs1681743 | 10 | 118565501 | T | C | -0.013 | 0.002 | 3.70E-08 | -0.002 | 0.002 | 3.10E-01 |
| rs1561589 | 10 | 126695673 | A | G | 0.016 | 0.002 | 4.24E-12 | 0.006 | 0.002 | 4.10E-03 |
| rs4880341 | 10 | 133992689 | T | C | -0.014 | 0.002 | 4.53E-10 | 0.001 | 0.002 | 5.90E-01 |
| rs7395632 | 11 | 958789 | T | C | -0.013 | 0.002 | 3.87E-08 | 0.000 | 0.002 | 9.00E-01 |
| rs4929923 | 11 | 8639200 | T | C | -0.022 | 0.002 | 2.51E-22 | -0.003 | 0.002 | 1.30E-01 |
| rs11022762 | 11 | 13335926 | T | C | -0.015 | 0.002 | 5.56E-11 | -0.005 | 0.002 | 1.30E-02 |
| rs6265 | 11 | 27679916 | T | C | -0.037 | 0.003 | 7.95E-40 | 0.003 | 0.003 | 2.20E-01 |
| rs2862961 | 11 | 43656535 | A | G | -0.024 | 0.003 | 5.30E-22 | -0.004 | 0.002 | 1.10E-01 |
| rs10742752 | 11 | 45438374 | T | C | -0.013 | 0.002 | 3.98E-09 | 0.002 | 0.002 | 3.80E-01 |
| rs7124681 | 11 | 47529947 | A | C | 0.025 | 0.002 | 9.27E-29 | 0.004 | 0.002 | 5.10E-02 |
| rs893006 | 11 | 64365796 | A | C | 0.017 | 0.002 | 4.05E-13 | 0.001 | 0.002 | 6.30E-01 |
| rs625652 | 11 | 65641931 | A | T | -0.020 | 0.003 | 9.05E-13 | -0.009 | 0.002 | 8.40E-05 |
| rs7107409 | 11 | 76492195 | T | C | -0.018 | 0.003 | 2.05E-08 | -0.011 | 0.003 | 2.80E-05 |
| rs2605603 | 11 | 93221105 | A | G | -0.013 | 0.002 | 1.40E-09 | -0.003 | 0.002 | 2.20E-01 |
| rs7105462 | 11 | 112912048 | A | G | 0.014 | 0.002 | 4.40E-10 | -0.005 | 0.002 | 3.80E-02 |
| rs1048932 | 11 | 115044850 | A | C | -0.018 | 0.002 | 2.52E-16 | -0.006 | 0.002 | 3.40E-03 |
| rs1003081 | 11 | 118913993 | T | C | 0.014 | 0.002 | 6.27E-11 | 0.003 | 0.002 | 2.40E-01 |
| rs4459316 | 11 | 130756176 | T | C | -0.015 | 0.002 | 2.33E-11 | -0.003 | 0.002 | 1.20E-01 |
| rs4936175 | 11 | 132641959 | T | C | -0.016 | 0.002 | 6.69E-12 | -0.003 | 0.002 | 1.90E-01 |
| rs329651 | 11 | 133767622 | T | G | 0.018 | 0.003 | 1.96E-10 | 0.011 | 0.003 | 1.10E-04 |
| rs12364470 | 11 | 134601012 | T | G | -0.020 | 0.003 | 2.54E-11 | -0.007 | 0.003 | 1.40E-02 |
| rs11611246 | 12 | 939480 | T | G | 0.021 | 0.003 | 1.27E-14 | -0.002 | 0.003 | 4.20E-01 |
| rs7976757 | 12 | 19207948 | T | C | -0.019 | 0.003 | 2.94E-10 | -0.006 | 0.003 | 2.90E-02 |
| rs16926318 | 12 | 23678745 | A | G | -0.023 | 0.004 | 4.23E-08 | -0.011 | 0.004 | 5.50E-03 |
| rs16926778 | 12 | 24002125 | C | G | 0.026 | 0.004 | 1.41E-09 | 0.003 | 0.004 | 5.30E-01 |
| rs7138803 | 12 | 50247468 | A | G | 0.029 | 0.002 | 9.70E-38 | 0.004 | 0.002 | 1.10E-01 |
| rs2271189 | 12 | 56494991 | A | G | -0.015 | 0.002 | 6.35E-10 | -0.010 | 0.002 | 2.30E-06 |
| rs11173522 | 12 | 60953472 | A | C | 0.017 | 0.003 | 9.01E-10 | 0.004 | 0.003 | 1.20E-01 |
| rs645026 | 12 | 69757931 | A | G | -0.016 | 0.003 | 4.79E-10 | 0.001 | 0.002 | 7.80E-01 |
| rs10746306 | 12 | 84477374 | T | G | 0.018 | 0.003 | 8.74E-09 | 0.003 | 0.003 | 2.70E-01 |
| rs2279574 | 12 | 89745477 | A | C | -0.017 | 0.002 | 1.96E-12 | -0.006 | 0.002 | 7.80E-03 |
| rs2712643 | 12 | 99638276 | A | C | -0.015 | 0.003 | 1.47E-09 | -0.004 | 0.003 | 1.40E-01 |
| rs17033633 | 12 | 103656343 | T | C | -0.021 | 0.003 | 7.26E-12 | -0.003 | 0.003 | 2.30E-01 |
| rs6606686 | 12 | 110903380 | C | G | -0.018 | 0.002 | 2.50E-14 | -0.005 | 0.002 | 4.90E-02 |
| rs17508240 | 12 | 117577358 | A | G | 0.019 | 0.003 | 2.83E-09 | 0.003 | 0.003 | 3.30E-01 |
| rs6489156 | 12 | 123100397 | T | C | 0.022 | 0.003 | 9.97E-18 | -0.005 | 0.002 | 3.90E-02 |
| rs7133378 | 12 | 124409502 | A | G | 0.016 | 0.002 | 1.85E-11 | -0.009 | 0.002 | 1.90E-04 |
| rs9579775 | 13 | 20616557 | A | C | -0.025 | 0.004 | 3.66E-10 | -0.003 | 0.003 | 3.20E-01 |
| rs12872889 | 13 | 28674628 | T | C | -0.021 | 0.003 | 1.52E-11 | -0.002 | 0.003 | 5.50E-01 |
| rs9595630 | 13 | 33065443 | T | G | 0.015 | 0.002 | 2.04E-10 | 0.008 | 0.002 | 6.00E-04 |
| rs12429545 | 13 | 54102206 | A | G | 0.029 | 0.003 | 1.09E-18 | -0.003 | 0.003 | 3.40E-01 |
| rs6561937 | 13 | 58257667 | A | T | -0.020 | 0.003 | 5.95E-13 | -0.012 | 0.003 | 4.30E-06 |
| rs4055791 | 13 | 59266053 | T | C | -0.017 | 0.003 | 1.77E-10 | -0.004 | 0.002 | 6.70E-02 |
| rs9571687 | 13 | 67472713 | A | C | -0.015 | 0.002 | 3.24E-09 | -0.005 | 0.002 | 3.40E-02 |
| rs1441264 | 13 | 79580919 | A | G | 0.017 | 0.002 | 5.19E-14 | -0.003 | 0.002 | 1.40E-01 |
| rs9531786 | 13 | 85983968 | C | G | -0.014 | 0.002 | 8.39E-09 | -0.005 | 0.002 | 4.30E-02 |
| rs77432547 | 13 | 86494817 | A | G | -0.018 | 0.003 | 2.28E-09 | -0.014 | 0.002 | 1.60E-08 |
| rs1927790 | 13 | 96922191 | T | C | -0.014 | 0.002 | 1.27E-09 | -0.005 | 0.002 | 3.20E-02 |
| rs4586287 | 13 | 99245334 | C | G | 0.014 | 0.003 | 2.95E-08 | 0.006 | 0.002 | 6.50E-03 |
| rs7335249 | 13 | 109962579 | T | C | 0.014 | 0.002 | 4.91E-09 | 0.003 | 0.002 | 1.20E-01 |
| rs2479958 | 13 | 111984244 | A | G | 0.014 | 0.002 | 1.38E-08 | 0.004 | 0.002 | 4.50E-02 |
| rs2754079 | 14 | 25886140 | A | G | -0.016 | 0.002 | 2.31E-11 | -0.003 | 0.002 | 1.90E-01 |
| rs1569979 | 14 | 29681294 | A | G | 0.023 | 0.003 | 1.57E-17 | 0.012 | 0.003 | 4.00E-06 |
| rs17522122 | 14 | 33302882 | T | G | 0.017 | 0.002 | 6.46E-14 | 0.011 | 0.002 | 7.90E-07 |
| rs35020117 | 14 | 73341655 | T | G | 0.017 | 0.003 | 1.23E-09 | 0.003 | 0.002 | 2.20E-01 |
| rs7141420 | 14 | 79899454 | T | C | 0.021 | 0.002 | 7.84E-22 | 0.006 | 0.002 | 6.50E-03 |
| rs1285997 | 14 | 91513029 | C | G | -0.015 | 0.003 | 3.18E-09 | -0.002 | 0.002 | 5.20E-01 |
| rs942066 | 14 | 94031914 | A | G | -0.020 | 0.003 | 1.04E-13 | -0.004 | 0.002 | 6.20E-02 |
| rs3803286 | 14 | 103246470 | A | G | 0.017 | 0.002 | 8.29E-13 | 0.000 | 0.002 | 9.90E-01 |
| rs709400 | 14 | 104149475 | A | G | 0.018 | 0.002 | 4.41E-16 | 0.008 | 0.002 | 5.50E-04 |
| rs12101625 | 15 | 26972431 | C | G | 0.014 | 0.002 | 5.54E-09 | 0.002 | 0.002 | 4.40E-01 |
| rs8038522 | 15 | 46585105 | T | G | -0.014 | 0.002 | 1.27E-09 | 0.000 | 0.002 | 9.90E-01 |
| rs6493498 | 15 | 51754451 | T | C | 0.016 | 0.002 | 1.53E-13 | 0.004 | 0.002 | 4.20E-02 |
| rs8024806 | 15 | 53473990 | T | C | 0.027 | 0.005 | 9.76E-09 | 0.010 | 0.005 | 2.60E-02 |
| rs8033510 | 15 | 61445514 | T | C | 0.014 | 0.002 | 1.13E-08 | 0.008 | 0.002 | 2.60E-04 |
| rs4776982 | 15 | 68114974 | A | G | 0.031 | 0.003 | 4.25E-33 | 0.004 | 0.003 | 8.40E-02 |
| rs12914489 | 15 | 74187937 | A | G | 0.023 | 0.004 | 1.64E-10 | 0.010 | 0.003 | 3.50E-03 |
| rs62007782 | 15 | 78029797 | A | G | -0.018 | 0.003 | 1.94E-09 | -0.015 | 0.002 | 7.60E-10 |
| rs12593036 | 15 | 81058652 | A | G | 0.015 | 0.003 | 6.65E-10 | 0.007 | 0.002 | 1.70E-03 |
| rs7498044 | 15 | 92573639 | A | G | -0.018 | 0.003 | 2.73E-08 | -0.011 | 0.003 | 6.40E-05 |
| rs11073383 | 15 | 95274349 | A | G | -0.016 | 0.002 | 3.26E-12 | -0.003 | 0.002 | 2.30E-01 |
| rs57221746 | 15 | 99230035 | A | G | -0.018 | 0.003 | 2.08E-08 | -0.008 | 0.003 | 1.00E-03 |
| rs214249 | 16 | 348687 | T | G | 0.017 | 0.002 | 1.37E-12 | 0.007 | 0.002 | 1.30E-03 |
| rs12448257 | 16 | 3599655 | A | G | 0.015 | 0.003 | 3.13E-08 | -0.004 | 0.003 | 1.20E-01 |
| rs879620 | 16 | 4015729 | T | C | 0.025 | 0.002 | 5.45E-26 | 0.006 | 0.002 | 1.20E-02 |
| rs9926784 | 16 | 19941968 | T | C | 0.025 | 0.003 | 6.01E-20 | -0.002 | 0.003 | 5.10E-01 |
| rs17767765 | 16 | 24539793 | T | C | -0.013 | 0.002 | 2.60E-08 | 0.000 | 0.002 | 8.60E-01 |
| rs7498665 | 16 | 28883241 | A | G | -0.026 | 0.002 | 1.94E-32 | -0.014 | 0.002 | 4.10E-10 |
| rs2289292 | 16 | 30097630 | T | C | -0.019 | 0.003 | 1.13E-14 | 0.003 | 0.002 | 1.30E-01 |
| rs4889606 | 16 | 31011183 | A | G | 0.027 | 0.002 | 1.25E-32 | 0.015 | 0.002 | 5.10E-11 |
| rs1558902 | 16 | 53803574 | A | T | 0.070 | 0.002 | 1.10E-215 | 0.001 | 0.002 | 6.60E-01 |
| rs4783718 | 16 | 69547741 | T | C | 0.020 | 0.002 | 1.77E-19 | 0.003 | 0.002 | 1.20E-01 |
| rs811054 | 16 | 72251132 | T | C | 0.016 | 0.002 | 3.18E-13 | -0.006 | 0.002 | 8.00E-03 |
| rs2012502 | 16 | 81728081 | A | C | 0.015 | 0.002 | 2.97E-11 | 0.002 | 0.002 | 2.80E-01 |
| rs4790292 | 17 | 1824305 | A | C | -0.026 | 0.003 | 1.65E-15 | -0.011 | 0.003 | 2.90E-04 |
| rs3826408 | 17 | 7101292 | T | C | 0.012 | 0.002 | 4.48E-08 | 0.006 | 0.002 | 9.60E-03 |
| rs1075901 | 17 | 15943910 | T | C | -0.014 | 0.002 | 4.36E-11 | 0.001 | 0.002 | 6.60E-01 |
| rs4986044 | 17 | 21261560 | T | C | -0.017 | 0.002 | 2.20E-15 | -0.009 | 0.002 | 1.80E-05 |
| rs12150665 | 17 | 34914787 | T | C | 0.017 | 0.002 | 5.92E-14 | 0.006 | 0.002 | 4.50E-03 |
| rs56161855 | 17 | 46288649 | A | T | -0.026 | 0.004 | 1.48E-11 | -0.007 | 0.003 | 2.00E-02 |
| rs9906944 | 17 | 47091420 | T | C | -0.019 | 0.002 | 7.25E-15 | -0.010 | 0.002 | 2.40E-05 |
| rs7211966 | 17 | 54374611 | T | C | -0.047 | 0.008 | 4.89E-09 | -0.005 | 0.008 | 5.00E-01 |
| rs8075273 | 17 | 61728881 | A | C | -0.016 | 0.002 | 5.56E-11 | -0.014 | 0.002 | 2.80E-09 |
| rs2619976 | 17 | 71754545 | T | C | 0.015 | 0.002 | 6.17E-10 | 0.006 | 0.002 | 9.30E-03 |
| rs12949279 | 17 | 78558411 | T | C | 0.018 | 0.002 | 6.09E-16 | 0.001 | 0.002 | 6.20E-01 |
| rs4969387 | 17 | 79081724 | C | G | -0.015 | 0.003 | 6.90E-09 | 0.001 | 0.002 | 6.40E-01 |
| rs9955276 | 18 | 1839339 | T | C | 0.022 | 0.003 | 8.02E-12 | 0.007 | 0.003 | 1.90E-02 |
| rs1808579 | 18 | 21104888 | T | C | -0.020 | 0.002 | 9.44E-20 | -0.012 | 0.002 | 1.10E-08 |
| rs16975921 | 18 | 39914870 | A | T | -0.016 | 0.003 | 3.87E-11 | -0.009 | 0.002 | 1.20E-04 |
| rs1518159 | 18 | 40760146 | T | C | -0.019 | 0.003 | 6.66E-13 | -0.002 | 0.002 | 3.20E-01 |
| rs7239114 | 18 | 45921214 | A | G | 0.016 | 0.002 | 3.90E-12 | -0.002 | 0.002 | 3.00E-01 |
| rs6567160 | 18 | 57829135 | T | C | -0.057 | 0.003 | 8.74E-108 | 0.006 | 0.003 | 2.50E-02 |
| rs17066856 | 18 | 58049656 | T | C | 0.040 | 0.004 | 1.39E-27 | 0.002 | 0.004 | 6.60E-01 |
| rs12981256 | 19 | 1865901 | A | G | 0.018 | 0.002 | 3.93E-15 | 0.005 | 0.002 | 1.20E-02 |
| rs895330 | 19 | 4060707 | C | G | 0.018 | 0.003 | 6.65E-10 | 0.006 | 0.003 | 1.80E-02 |
| rs273504 | 19 | 18215247 | A | G | -0.015 | 0.002 | 1.27E-10 | -0.009 | 0.002 | 9.50E-05 |
| rs17724992 | 19 | 18454825 | A | G | 0.017 | 0.003 | 6.65E-12 | 0.004 | 0.002 | 1.10E-01 |
| rs11880870 | 19 | 18830704 | A | G | 0.020 | 0.002 | 3.31E-18 | 0.002 | 0.002 | 3.20E-01 |
| rs17751061 | 19 | 19413092 | T | C | -0.018 | 0.003 | 1.29E-08 | -0.010 | 0.003 | 7.50E-04 |
| rs12462975 | 19 | 30272202 | A | G | 0.020 | 0.003 | 2.63E-16 | 0.003 | 0.002 | 2.40E-01 |
| rs11667280 | 19 | 30705747 | C | G | 0.016 | 0.003 | 3.14E-08 | 0.007 | 0.003 | 9.10E-03 |
| rs7250833 | 19 | 33937277 | T | C | 0.015 | 0.002 | 7.22E-10 | 0.001 | 0.002 | 7.40E-01 |
| rs185350 | 19 | 34306816 | T | C | 0.013 | 0.002 | 7.14E-09 | 0.004 | 0.002 | 4.40E-02 |
| rs429358 | 19 | 45411941 | T | C | 0.028 | 0.004 | 3.17E-14 | 0.014 | 0.003 | 3.80E-06 |
| rs11672660 | 19 | 46180184 | T | C | -0.032 | 0.003 | 5.93E-31 | -0.004 | 0.003 | 1.40E-01 |
| rs9304665 | 19 | 47602577 | A | T | 0.020 | 0.003 | 1.84E-14 | -0.006 | 0.003 | 1.90E-02 |
| rs1884389 | 20 | 1410582 | T | C | -0.014 | 0.002 | 1.20E-09 | -0.001 | 0.002 | 5.80E-01 |
| rs1884897 | 20 | 6612832 | A | G | -0.017 | 0.002 | 9.06E-14 | 0.000 | 0.002 | 8.80E-01 |
| rs1409818 | 20 | 21381121 | T | C | 0.025 | 0.004 | 1.58E-11 | -0.008 | 0.004 | 2.60E-02 |
| rs6050446 | 20 | 25195509 | A | G | -0.037 | 0.006 | 6.20E-09 | -0.014 | 0.006 | 2.10E-02 |
| rs6142096 | 20 | 32686658 | A | G | 0.013 | 0.002 | 4.17E-09 | 0.001 | 0.002 | 7.30E-01 |
| rs16989232 | 20 | 39291784 | A | G | 0.013 | 0.002 | 1.52E-08 | 0.000 | 0.002 | 9.80E-01 |
| rs17806224 | 20 | 51065854 | A | G | -0.029 | 0.003 | 3.52E-22 | -0.009 | 0.003 | 7.70E-04 |
| rs6010784 | 20 | 61540319 | T | C | 0.013 | 0.002 | 1.08E-08 | -0.008 | 0.002 | 1.30E-04 |
| rs2836754 | 21 | 40291740 | T | C | -0.015 | 0.002 | 3.73E-11 | -0.003 | 0.002 | 2.60E-01 |
| rs915814 | 21 | 46493003 | A | G | -0.018 | 0.003 | 5.88E-09 | -0.006 | 0.003 | 1.10E-02 |
| rs427943 | 21 | 46570896 | A | C | -0.018 | 0.002 | 1.37E-14 | -0.009 | 0.002 | 3.70E-05 |
| rs28489620 | 22 | 41804716 | A | G | -0.018 | 0.003 | 6.90E-10 | -0.012 | 0.002 | 1.30E-06 |
| rs13053342 | 22 | 48871624 | A | G | 0.015 | 0.002 | 1.32E-10 | 0.006 | 0.002 | 5.90E-03 |
| **6) MR of leisure television use on BMI (women)** | | | | | | | | | | |
| SNP | CHR | POS | EA | OA | beta_bmi | se_bmi | pval_bmi | beta_tv | se_tv | pval_tv |
| rs11810109 | 1 | 44186812 | A | T | -0.002 | 0.003 | 5.76E-01 | 0.016 | 0.002 | 5.40E-12 |
| rs112632642 | 1 | 51115634 | G | A | -0.017 | 0.005 | 9.31E-04 | -0.024 | 0.004 | 3.60E-08 |
| rs6670915 | 1 | 61109968 | C | T | 0.002 | 0.002 | 3.66E-01 | 0.014 | 0.002 | 2.40E-10 |
| rs12725114 | 1 | 62432641 | G | A | 0.008 | 0.003 | 1.01E-02 | 0.015 | 0.003 | 1.60E-08 |
| rs984409 | 1 | 67020440 | G | A | -0.010 | 0.003 | 2.25E-04 | -0.015 | 0.002 | 4.10E-11 |
| rs71658797 | 1 | 77967507 | T | A | -0.038 | 0.004 | 1.24E-20 | -0.020 | 0.003 | 6.30E-10 |
| rs114328297 | 1 | 91190854 | T | G | 0.003 | 0.003 | 4.34E-01 | 0.014 | 0.003 | 4.40E-08 |
| rs631130 | 1 | 96043010 | T | A | -0.001 | 0.003 | 8.09E-01 | -0.014 | 0.002 | 5.00E-09 |
| rs148648822 | 1 | 97815048 | G | T | 0.003 | 0.003 | 3.15E-01 | -0.018 | 0.003 | 4.20E-09 |
| rs17379561 | 1 | 98340139 | A | T | -0.022 | 0.003 | 1.48E-11 | -0.025 | 0.003 | 1.10E-16 |
| rs55909997 | 1 | 107581039 | G | A | -0.013 | 0.002 | 1.72E-07 | -0.014 | 0.002 | 2.40E-09 |
| rs1278847 | 1 | 110037483 | C | A | -0.010 | 0.003 | 4.05E-04 | 0.016 | 0.002 | 1.20E-11 |
| rs141425667 | 1 | 114930465 | G | A | -0.008 | 0.005 | 1.24E-01 | -0.024 | 0.004 | 4.00E-08 |
| rs4845364 | 1 | 154141908 | A | G | -0.002 | 0.002 | 5.13E-01 | -0.015 | 0.002 | 1.30E-12 |
| rs6673341 | 1 | 184655649 | T | G | -0.011 | 0.002 | 1.15E-06 | -0.015 | 0.002 | 2.20E-11 |
| rs72725224 | 1 | 188115858 | A | C | -0.008 | 0.010 | 3.96E-01 | -0.046 | 0.008 | 3.70E-09 |
| rs10737620 | 1 | 193057971 | T | A | 0.013 | 0.003 | 1.16E-07 | 0.014 | 0.002 | 2.60E-09 |
| rs11896615 | 2 | 17407744 | G | A | 0.003 | 0.004 | 4.51E-01 | 0.017 | 0.003 | 3.10E-08 |
| rs72781699 | 2 | 24277709 | G | A | -0.002 | 0.003 | 4.85E-01 | -0.019 | 0.003 | 3.00E-12 |
| rs12105701 | 2 | 41737200 | C | T | -0.009 | 0.002 | 6.21E-05 | -0.013 | 0.002 | 5.40E-09 |
| rs7564130 | 2 | 50606642 | T | C | -0.001 | 0.002 | 5.70E-01 | -0.015 | 0.002 | 2.80E-11 |
| rs11125515 | 2 | 53886799 | C | A | 0.004 | 0.003 | 1.45E-01 | -0.014 | 0.002 | 2.20E-08 |
| rs12475168 | 2 | 58992704 | G | T | -0.012 | 0.003 | 3.95E-04 | -0.018 | 0.003 | 1.70E-08 |
| rs183566425 | 2 | 59325356 | A | T | 0.024 | 0.010 | 2.05E-02 | 0.046 | 0.008 | 1.90E-08 |
| rs10189857 | 2 | 60713235 | A | G | -0.005 | 0.002 | 2.16E-02 | -0.020 | 0.002 | 6.20E-21 |
| rs62641636 | 2 | 68425427 | A | G | 0.003 | 0.003 | 2.52E-01 | 0.014 | 0.002 | 7.00E-10 |
| rs59210467 | 2 | 97097291 | C | T | 0.003 | 0.003 | 3.08E-01 | -0.013 | 0.002 | 1.00E-08 |
| rs11689199 | 2 | 100820421 | A | G | 0.019 | 0.002 | 1.45E-15 | 0.018 | 0.002 | 5.50E-17 |
| rs2916251 | 2 | 104447052 | G | T | -0.014 | 0.002 | 2.70E-09 | -0.017 | 0.002 | 1.50E-13 |
| rs1451533 | 2 | 105466005 | G | A | -0.015 | 0.003 | 3.95E-09 | -0.016 | 0.002 | 1.10E-10 |
| rs2971640 | 2 | 107624618 | G | A | -0.005 | 0.002 | 1.78E-02 | -0.013 | 0.002 | 4.20E-09 |
| rs12476388 | 2 | 116330983 | C | T | 0.006 | 0.003 | 1.70E-02 | 0.013 | 0.002 | 2.10E-08 |
| rs374722 | 2 | 147839830 | G | A | 0.013 | 0.003 | 5.05E-05 | 0.024 | 0.003 | 5.50E-16 |
| rs3754970 | 2 | 162091836 | T | C | -0.003 | 0.003 | 2.07E-01 | -0.015 | 0.002 | 4.80E-12 |
| rs1913808 | 2 | 162804678 | G | A | -0.001 | 0.003 | 7.48E-01 | 0.015 | 0.002 | 3.90E-10 |
| rs2060199 | 2 | 166196681 | T | A | 0.003 | 0.002 | 1.88E-01 | 0.012 | 0.002 | 2.40E-08 |
| rs263771 | 2 | 185921692 | C | A | 0.002 | 0.003 | 5.48E-01 | -0.017 | 0.003 | 3.10E-11 |
| rs4577309 | 2 | 191288833 | A | G | 0.002 | 0.003 | 3.61E-01 | 0.016 | 0.002 | 1.60E-13 |
| rs34224261 | 2 | 193855452 | G | C | -0.010 | 0.003 | 1.97E-04 | -0.018 | 0.002 | 4.60E-16 |
| rs4675246 | 2 | 202864487 | G | T | 0.002 | 0.003 | 4.91E-01 | -0.015 | 0.003 | 2.90E-08 |
| rs13029509 | 2 | 215374209 | G | A | -0.007 | 0.002 | 2.60E-03 | -0.018 | 0.002 | 2.00E-17 |
| rs10932837 | 2 | 221044785 | C | T | 0.001 | 0.002 | 7.82E-01 | -0.013 | 0.002 | 1.30E-09 |
| rs4973576 | 2 | 233806771 | C | A | 0.000 | 0.003 | 8.92E-01 | -0.015 | 0.002 | 1.00E-09 |
| rs138256022 | 3 | 11603090 | C | T | 0.000 | 0.007 | 9.75E-01 | -0.031 | 0.006 | 2.90E-08 |
| rs9834970 | 3 | 36856030 | T | C | 0.006 | 0.002 | 1.14E-02 | 0.013 | 0.002 | 3.30E-09 |
| rs1352890 | 3 | 49652156 | C | T | -0.007 | 0.003 | 8.47E-03 | -0.012 | 0.002 | 1.30E-08 |
| rs3796386 | 3 | 49899795 | G | A | -0.017 | 0.002 | 9.78E-14 | -0.026 | 0.002 | 3.20E-33 |
| rs182355396 | 3 | 50920457 | C | A | -0.007 | 0.015 | 6.42E-01 | -0.080 | 0.013 | 2.10E-10 |
| rs11917871 | 3 | 54233476 | T | C | -0.004 | 0.003 | 1.52E-01 | -0.017 | 0.002 | 4.50E-14 |
| rs11130793 | 3 | 60884659 | C | T | 0.011 | 0.003 | 6.46E-05 | 0.013 | 0.002 | 5.00E-09 |
| rs11714337 | 3 | 71582521 | G | A | 0.009 | 0.003 | 1.28E-03 | 0.014 | 0.002 | 4.70E-11 |
| rs12496084 | 3 | 75174777 | A | G | -0.004 | 0.003 | 1.50E-01 | -0.014 | 0.003 | 4.60E-08 |
| rs6797840 | 3 | 85656569 | A | C | -0.012 | 0.003 | 8.70E-06 | -0.016 | 0.002 | 1.70E-13 |
| rs56398417 | 3 | 88024986 | C | T | 0.009 | 0.003 | 2.80E-03 | 0.014 | 0.002 | 5.90E-10 |
| rs2034768 | 3 | 93986371 | A | G | 0.019 | 0.003 | 9.80E-14 | 0.015 | 0.002 | 8.60E-12 |
| rs12632778 | 3 | 106452359 | T | G | 0.000 | 0.005 | 9.66E-01 | -0.028 | 0.005 | 3.90E-08 |
| rs9867121 | 3 | 114631548 | C | A | 0.009 | 0.003 | 5.94E-03 | 0.019 | 0.003 | 3.90E-12 |
| rs12491503 | 3 | 165706855 | G | A | -0.005 | 0.002 | 3.29E-02 | -0.014 | 0.002 | 5.50E-10 |
| rs114600294 | 3 | 181419367 | G | C | -0.007 | 0.003 | 3.65E-02 | -0.016 | 0.003 | 7.90E-10 |
| rs66852340 | 4 | 3311070 | C | T | -0.009 | 0.003 | 1.10E-03 | -0.018 | 0.003 | 7.90E-12 |
| rs7700107 | 4 | 17880416 | A | C | -0.016 | 0.003 | 3.40E-06 | -0.021 | 0.003 | 1.90E-11 |
| rs34811474 | 4 | 25408838 | G | A | 0.032 | 0.003 | 5.19E-24 | 0.015 | 0.003 | 2.30E-09 |
| rs77215114 | 4 | 45187658 | A | T | 0.015 | 0.005 | 5.67E-03 | 0.024 | 0.004 | 1.50E-08 |
| rs28971796 | 4 | 49151982 | A | G | 0.012 | 0.003 | 3.93E-05 | 0.015 | 0.002 | 2.10E-09 |
| rs6850494 | 4 | 82291771 | A | C | 0.003 | 0.003 | 2.38E-01 | -0.014 | 0.002 | 1.10E-10 |
| rs13107325 | 4 | 103188709 | C | T | -0.045 | 0.004 | 9.33E-25 | -0.029 | 0.004 | 1.50E-12 |
| rs62332760 | 4 | 106135316 | G | T | 0.004 | 0.003 | 1.50E-01 | -0.014 | 0.002 | 8.00E-11 |
| rs7693703 | 4 | 118357405 | G | A | 0.004 | 0.004 | 3.03E-01 | 0.023 | 0.004 | 2.70E-09 |
| rs4334769 | 4 | 130245233 | G | T | 0.004 | 0.002 | 1.22E-01 | 0.012 | 0.002 | 2.10E-08 |
| rs6825241 | 4 | 152543783 | C | A | -0.003 | 0.002 | 2.30E-01 | -0.017 | 0.002 | 4.90E-15 |
| rs7693082 | 4 | 159857819 | G | C | 0.012 | 0.003 | 3.71E-05 | 0.015 | 0.002 | 1.70E-10 |
| rs10940659 | 5 | 59360341 | A | G | -0.007 | 0.002 | 4.25E-03 | -0.013 | 0.002 | 1.50E-08 |
| rs2409838 | 5 | 60204410 | A | T | -0.002 | 0.002 | 3.88E-01 | 0.017 | 0.002 | 3.40E-15 |
| rs262890 | 5 | 62930015 | A | G | -0.013 | 0.003 | 7.07E-06 | -0.019 | 0.002 | 3.20E-15 |
| rs7716447 | 5 | 88800331 | A | G | -0.015 | 0.002 | 1.46E-10 | -0.013 | 0.002 | 3.60E-09 |
| rs1031423 | 5 | 93276883 | T | C | 0.007 | 0.003 | 1.55E-02 | -0.019 | 0.003 | 1.80E-12 |
| rs57585211 | 5 | 107197725 | T | G | -0.003 | 0.003 | 3.66E-01 | -0.017 | 0.003 | 4.30E-09 |
| rs9326862 | 5 | 112054547 | A | T | 0.005 | 0.002 | 4.43E-02 | 0.012 | 0.002 | 2.60E-08 |
| rs10041724 | 5 | 124273520 | T | C | 0.005 | 0.003 | 1.17E-01 | 0.018 | 0.003 | 3.90E-11 |
| rs10057545 | 5 | 138431008 | G | A | -0.007 | 0.003 | 6.09E-03 | -0.014 | 0.002 | 2.00E-10 |
| rs62379379 | 5 | 141082015 | G | T | -0.011 | 0.005 | 3.92E-02 | -0.026 | 0.004 | 7.80E-10 |
| rs10054327 | 5 | 147871137 | G | A | 0.004 | 0.003 | 1.83E-01 | 0.017 | 0.002 | 3.40E-15 |
| rs42210 | 5 | 166408788 | G | C | -0.004 | 0.003 | 1.81E-01 | -0.014 | 0.002 | 7.30E-09 |
| rs2336895 | 5 | 166990695 | G | A | 0.002 | 0.003 | 5.38E-01 | 0.012 | 0.002 | 4.60E-08 |
| rs72828890 | 5 | 167806836 | C | T | 0.016 | 0.004 | 1.25E-04 | 0.019 | 0.003 | 4.90E-09 |
| rs255292 | 5 | 172580866 | C | A | -0.002 | 0.002 | 4.51E-01 | -0.012 | 0.002 | 1.70E-08 |
| rs72834698 | 6 | 26176517 | G | A | 0.013 | 0.004 | 9.74E-04 | 0.023 | 0.003 | 2.70E-13 |
| rs3135044 | 6 | 31592820 | C | T | -0.006 | 0.003 | 1.70E-02 | -0.015 | 0.002 | 8.70E-11 |
| rs10947452 | 6 | 33803752 | T | C | 0.011 | 0.003 | 1.21E-04 | 0.014 | 0.002 | 6.40E-10 |
| rs9471333 | 6 | 40362023 | C | T | 0.021 | 0.003 | 4.04E-15 | 0.013 | 0.002 | 1.50E-09 |
| rs4523073 | 6 | 67550288 | A | G | -0.004 | 0.003 | 1.07E-01 | -0.014 | 0.002 | 5.00E-10 |
| rs6905544 | 6 | 98411631 | A | G | -0.015 | 0.002 | 1.28E-10 | -0.019 | 0.002 | 8.50E-18 |
| rs17789218 | 6 | 100600097 | T | C | -0.007 | 0.003 | 1.07E-02 | 0.019 | 0.003 | 1.40E-13 |
| rs78394231 | 6 | 107649123 | T | C | -0.006 | 0.005 | 1.71E-01 | -0.021 | 0.004 | 9.70E-09 |
| rs2184364 | 6 | 143183596 | A | G | 0.011 | 0.003 | 2.43E-04 | 0.016 | 0.003 | 3.00E-09 |
| rs9718104 | 6 | 166170539 | T | G | 0.002 | 0.006 | 7.51E-01 | -0.041 | 0.005 | 9.30E-19 |
| rs17568389 | 7 | 8091876 | T | A | 0.002 | 0.002 | 3.46E-01 | 0.015 | 0.002 | 8.60E-13 |
| rs10234444 | 7 | 41828527 | G | A | 0.006 | 0.003 | 5.90E-02 | 0.016 | 0.003 | 2.90E-08 |
| rs6973656 | 7 | 77422583 | A | G | -0.011 | 0.002 | 1.55E-06 | -0.013 | 0.002 | 1.00E-09 |
| rs973734 | 7 | 89387578 | C | A | 0.001 | 0.003 | 7.34E-01 | 0.017 | 0.003 | 1.50E-08 |
| rs3137 | 7 | 99130875 | G | A | 0.017 | 0.003 | 1.95E-08 | 0.017 | 0.003 | 1.40E-08 |
| rs2694151 | 7 | 101902180 | C | T | 0.006 | 0.005 | 2.39E-01 | 0.026 | 0.005 | 2.30E-08 |
| rs62471080 | 7 | 109128060 | G | C | -0.009 | 0.003 | 9.05E-04 | -0.013 | 0.002 | 3.70E-09 |
| rs7788008 | 7 | 112972483 | G | A | 0.017 | 0.002 | 1.09E-13 | 0.013 | 0.002 | 5.90E-09 |
| rs10246289 | 7 | 115544763 | A | G | 0.004 | 0.004 | 3.87E-01 | 0.019 | 0.003 | 1.70E-08 |
| rs11763734 | 7 | 126400910 | A | C | 0.000 | 0.002 | 9.14E-01 | -0.012 | 0.002 | 8.20E-09 |
| rs7834121 | 8 | 10770255 | G | T | 0.017 | 0.003 | 1.79E-10 | -0.014 | 0.002 | 1.10E-10 |
| rs1421334 | 8 | 30865733 | A | C | 0.013 | 0.002 | 2.39E-08 | 0.017 | 0.002 | 2.20E-15 |
| rs6996198 | 8 | 65463442 | C | T | -0.002 | 0.003 | 4.82E-01 | -0.016 | 0.003 | 3.50E-08 |
| rs6472942 | 8 | 76827190 | T | C | 0.017 | 0.002 | 3.53E-13 | -0.013 | 0.002 | 1.80E-09 |
| rs11985394 | 8 | 92667521 | T | A | 0.003 | 0.002 | 2.74E-01 | 0.012 | 0.002 | 1.30E-08 |
| rs72671494 | 8 | 93195457 | T | C | -0.012 | 0.004 | 2.39E-03 | -0.017 | 0.003 | 4.50E-08 |
| rs12541615 | 8 | 118870151 | T | C | -0.010 | 0.003 | 3.99E-03 | -0.018 | 0.003 | 3.90E-10 |
| rs2717559 | 8 | 143882420 | A | G | 0.005 | 0.003 | 8.28E-02 | 0.012 | 0.002 | 1.50E-08 |
| rs2616830 | 9 | 1721385 | G | A | 0.003 | 0.002 | 2.41E-01 | 0.016 | 0.002 | 2.90E-14 |
| rs2291681 | 9 | 14790841 | G | T | 0.005 | 0.002 | 2.84E-02 | 0.013 | 0.002 | 7.90E-09 |
| rs34864022 | 9 | 22609110 | A | G | 0.001 | 0.005 | 9.19E-01 | -0.026 | 0.004 | 1.40E-09 |
| rs12554512 | 9 | 23352293 | T | C | -0.012 | 0.003 | 4.61E-06 | 0.021 | 0.002 | 3.80E-21 |
| rs2791574 | 9 | 82212094 | T | G | -0.004 | 0.002 | 8.01E-02 | -0.013 | 0.002 | 3.00E-08 |
| rs870151 | 9 | 92230747 | T | A | -0.014 | 0.002 | 4.62E-09 | -0.016 | 0.002 | 8.80E-13 |
| rs2787374 | 9 | 103054951 | T | C | 0.013 | 0.002 | 5.57E-08 | 0.012 | 0.002 | 1.20E-08 |
| rs10739499 | 9 | 120514261 | C | G | 0.005 | 0.002 | 3.34E-02 | 0.014 | 0.002 | 1.50E-09 |
| rs7043521 | 9 | 124602779 | A | T | 0.013 | 0.003 | 1.97E-06 | 0.014 | 0.002 | 6.50E-11 |
| rs12342024 | 9 | 126134232 | G | T | -0.006 | 0.004 | 1.58E-01 | -0.021 | 0.003 | 1.70E-09 |
| rs4382592 | 9 | 134870755 | T | G | 0.007 | 0.003 | 4.41E-03 | 0.014 | 0.002 | 6.70E-09 |
| rs2073869 | 9 | 135763816 | C | T | 0.008 | 0.003 | 1.17E-02 | 0.019 | 0.003 | 1.50E-10 |
| 9:139926402_T_C | 9 | 139926402 | T | C | 0.001 | 0.003 | 7.79E-01 | -0.013 | 0.002 | 2.00E-08 |
| rs1623025 | 10 | 11164813 | G | C | -0.003 | 0.003 | 2.47E-01 | -0.012 | 0.002 | 1.40E-08 |
| rs1243182 | 10 | 21916728 | C | T | -0.020 | 0.003 | 3.15E-15 | -0.019 | 0.002 | 2.00E-15 |
| rs2045147 | 10 | 56660437 | A | G | 0.003 | 0.002 | 1.72E-01 | 0.013 | 0.002 | 5.90E-09 |
| rs10994943 | 10 | 63591413 | T | G | 0.007 | 0.003 | 1.48E-02 | 0.013 | 0.002 | 3.10E-09 |
| rs11201422 | 10 | 86924483 | T | C | 0.012 | 0.003 | 1.45E-06 | 0.013 | 0.002 | 3.40E-08 |
| rs10786658 | 10 | 103737193 | A | T | 0.002 | 0.003 | 3.88E-01 | -0.014 | 0.002 | 4.60E-11 |
| rs7089973 | 10 | 116569565 | C | A | 0.000 | 0.002 | 9.06E-01 | -0.013 | 0.002 | 2.40E-09 |
| rs11245482 | 10 | 126733546 | T | C | -0.011 | 0.002 | 4.63E-06 | -0.013 | 0.002 | 2.60E-09 |
| rs17727474 | 10 | 127182959 | C | T | -0.004 | 0.003 | 2.43E-01 | 0.018 | 0.003 | 3.10E-09 |
| rs12289262 | 11 | 12894758 | C | T | -0.009 | 0.003 | 4.88E-04 | -0.014 | 0.002 | 2.00E-08 |
| rs801733 | 11 | 65934549 | A | C | 0.009 | 0.002 | 1.67E-04 | 0.017 | 0.002 | 7.30E-14 |
| rs116175338 | 11 | 72467875 | G | T | -0.008 | 0.003 | 2.76E-03 | -0.014 | 0.003 | 1.40E-08 |
| rs12272012 | 11 | 84861286 | G | C | -0.010 | 0.007 | 1.20E-01 | -0.030 | 0.005 | 1.90E-08 |
| rs11020045 | 11 | 92487907 | A | C | 0.002 | 0.002 | 5.41E-01 | -0.013 | 0.002 | 1.60E-08 |
| rs17207890 | 11 | 95490754 | G | A | -0.002 | 0.002 | 5.51E-01 | 0.016 | 0.002 | 6.70E-12 |
| rs10791546 | 11 | 101830126 | C | T | 0.009 | 0.003 | 1.31E-03 | 0.012 | 0.002 | 4.10E-08 |
| rs328277 | 11 | 110414195 | C | A | -0.002 | 0.003 | 5.21E-01 | 0.012 | 0.002 | 4.50E-08 |
| rs648044 | 11 | 114030799 | A | G | -0.007 | 0.003 | 1.12E-02 | -0.014 | 0.002 | 1.00E-09 |
| rs11218575 | 11 | 122154576 | C | T | 0.005 | 0.002 | 4.42E-02 | 0.015 | 0.002 | 2.10E-12 |
| rs74802478 | 11 | 131966907 | G | A | 0.009 | 0.003 | 1.22E-02 | 0.017 | 0.003 | 3.50E-09 |
| rs4937842 | 11 | 133734542 | G | C | -0.009 | 0.002 | 2.80E-04 | -0.013 | 0.002 | 1.40E-08 |
| rs10772643 | 12 | 13415288 | C | T | 0.013 | 0.004 | 1.86E-03 | 0.025 | 0.003 | 1.30E-12 |
| rs74996610 | 12 | 24075007 | C | G | 0.003 | 0.006 | 5.96E-01 | -0.031 | 0.005 | 4.10E-09 |
| rs10771746 | 12 | 30791864 | C | T | -0.003 | 0.003 | 3.36E-01 | -0.014 | 0.002 | 2.50E-09 |
| rs10876864 | 12 | 56401085 | G | A | -0.010 | 0.002 | 6.64E-06 | -0.013 | 0.002 | 1.00E-09 |
| rs8756 | 12 | 66359752 | C | A | 0.001 | 0.002 | 5.52E-01 | -0.013 | 0.002 | 5.30E-10 |
| rs11179838 | 12 | 74321123 | C | G | 0.003 | 0.003 | 2.98E-01 | 0.013 | 0.002 | 2.10E-08 |
| rs2164744 | 12 | 109872039 | T | C | -0.006 | 0.003 | 4.25E-02 | -0.013 | 0.002 | 1.10E-08 |
| rs2173650 | 12 | 117525235 | G | T | 0.003 | 0.004 | 4.77E-01 | 0.018 | 0.003 | 4.60E-09 |
| rs1790135 | 12 | 123669235 | C | T | 0.001 | 0.003 | 8.71E-01 | -0.014 | 0.003 | 2.60E-08 |
| rs9563168 | 13 | 54247827 | G | A | 0.010 | 0.003 | 4.82E-04 | 0.018 | 0.003 | 4.30E-11 |
| rs9569734 | 13 | 58319476 | A | G | 0.019 | 0.003 | 3.51E-09 | 0.019 | 0.003 | 3.40E-10 |
| rs9528023 | 13 | 60226991 | C | T | 0.005 | 0.003 | 5.05E-02 | 0.013 | 0.002 | 3.80E-08 |
| rs56858768 | 13 | 86511730 | G | A | -0.017 | 0.003 | 1.04E-08 | -0.015 | 0.002 | 3.40E-10 |
| rs553397860 | 13 | 99051118 | A | G | 0.012 | 0.003 | 2.57E-04 | 0.017 | 0.003 | 8.90E-09 |
| rs7991062 | 13 | 100713194 | C | G | -0.004 | 0.002 | 1.25E-01 | -0.018 | 0.002 | 8.80E-15 |
| rs11629120 | 14 | 23458457 | T | C | -0.001 | 0.003 | 6.20E-01 | -0.013 | 0.002 | 1.10E-08 |
| rs405797 | 14 | 26972834 | T | A | -0.005 | 0.003 | 4.22E-02 | -0.015 | 0.003 | 2.80E-09 |
| rs1022785 | 14 | 29571653 | G | A | 0.010 | 0.003 | 1.48E-03 | 0.018 | 0.003 | 7.10E-09 |
| rs10145592 | 14 | 94287215 | C | G | -0.004 | 0.002 | 6.48E-02 | -0.015 | 0.002 | 1.80E-11 |
| rs7157001 | 14 | 99749484 | A | G | -0.003 | 0.003 | 3.52E-01 | -0.014 | 0.002 | 2.30E-08 |
| rs8043253 | 15 | 41503730 | C | T | -0.006 | 0.002 | 9.24E-03 | -0.012 | 0.002 | 3.40E-08 |
| rs2460 | 15 | 53073084 | G | A | -0.011 | 0.003 | 1.94E-05 | -0.015 | 0.002 | 5.00E-10 |
| rs4775373 | 15 | 61476936 | T | C | 0.005 | 0.003 | 7.06E-02 | 0.013 | 0.002 | 4.70E-09 |
| rs61331678 | 15 | 77995949 | G | C | 0.008 | 0.003 | 2.59E-03 | 0.015 | 0.002 | 6.10E-12 |
| rs35574015 | 16 | 15146061 | T | C | 0.004 | 0.003 | 2.01E-01 | -0.013 | 0.002 | 3.00E-08 |
| rs7189927 | 16 | 28913787 | T | C | 0.025 | 0.002 | 3.50E-26 | 0.015 | 0.002 | 3.40E-11 |
| rs749671 | 16 | 31088347 | G | A | 0.026 | 0.002 | 1.78E-31 | 0.016 | 0.002 | 2.70E-12 |
| rs7184800 | 16 | 53509131 | G | A | -0.006 | 0.003 | 4.26E-02 | 0.017 | 0.002 | 8.20E-13 |
| rs2447098 | 17 | 2277720 | C | A | -0.002 | 0.002 | 3.60E-01 | -0.015 | 0.002 | 6.80E-12 |
| rs11654952 | 17 | 4813799 | T | G | -0.001 | 0.003 | 7.62E-01 | -0.017 | 0.003 | 4.30E-08 |
| rs9902312 | 17 | 65070304 | T | C | -0.001 | 0.002 | 5.61E-01 | 0.015 | 0.002 | 4.50E-11 |
| rs11657730 | 17 | 79372489 | C | T | 0.002 | 0.003 | 4.99E-01 | 0.013 | 0.002 | 1.10E-08 |
| rs303753 | 18 | 21074922 | G | A | -0.019 | 0.002 | 1.99E-15 | -0.014 | 0.002 | 2.70E-10 |
| rs11081851 | 18 | 31783262 | G | A | -0.006 | 0.002 | 1.79E-02 | -0.013 | 0.002 | 1.00E-08 |
| rs9964724 | 18 | 35159124 | C | T | 0.003 | 0.003 | 2.32E-01 | 0.018 | 0.002 | 3.30E-14 |
| rs1156541 | 18 | 39952989 | C | T | 0.014 | 0.003 | 2.81E-07 | 0.014 | 0.003 | 2.80E-08 |
| rs148544378 | 18 | 40323567 | C | T | -0.021 | 0.010 | 2.82E-02 | 0.053 | 0.008 | 2.20E-12 |
| rs17512836 | 18 | 53194961 | T | C | 0.021 | 0.008 | 1.30E-02 | 0.042 | 0.007 | 7.00E-10 |
| rs7248205 | 19 | 10770305 | C | T | 0.010 | 0.003 | 3.27E-04 | 0.014 | 0.002 | 3.40E-10 |
| rs111901094 | 19 | 19513570 | G | T | -0.011 | 0.003 | 1.57E-03 | -0.017 | 0.003 | 2.00E-09 |
| rs6131281 | 20 | 11891724 | C | T | 0.009 | 0.003 | 6.80E-04 | 0.016 | 0.002 | 3.10E-13 |
| rs6141814 | 20 | 31368960 | C | A | 0.001 | 0.003 | 7.05E-01 | -0.014 | 0.002 | 1.30E-09 |
| rs4810312 | 20 | 39968707 | C | G | 0.000 | 0.002 | 8.98E-01 | -0.013 | 0.002 | 1.20E-08 |
| rs6124476 | 20 | 41202830 | T | C | 0.007 | 0.002 | 2.59E-03 | 0.012 | 0.002 | 1.80E-08 |
| rs6031440 | 20 | 42809317 | A | T | 0.002 | 0.002 | 4.65E-01 | 0.012 | 0.002 | 1.80E-08 |
| rs55700114 | 20 | 43717080 | G | A | -0.003 | 0.003 | 3.02E-01 | -0.014 | 0.002 | 2.10E-09 |
| rs6125906 | 20 | 48729803 | C | A | -0.001 | 0.005 | 8.25E-01 | -0.021 | 0.004 | 3.90E-08 |
| rs56103247 | 20 | 62483184 | C | T | 0.020 | 0.006 | 7.71E-04 | 0.030 | 0.005 | 3.80E-10 |
| rs10427502 | 21 | 40654840 | G | A | 0.010 | 0.003 | 3.20E-04 | 0.014 | 0.002 | 8.60E-10 |
| rs7609 | 22 | 29951650 | A | T | 0.003 | 0.003 | 2.68E-01 | 0.015 | 0.003 | 3.60E-09 |
| rs2092829 | 22 | 31780514 | G | A | -0.007 | 0.003 | 4.45E-03 | 0.014 | 0.002 | 1.30E-08 |

| **Table S30. Betas of the bidirectional MR between years of education and leisure computer use** | | | | | | | | | | |
| --- | --- | --- | --- | --- | --- | --- | --- | --- | --- | --- |
| **1) MR of years of education on leisure computer use** | | | | | | | | | | |
| SNP | Chr | Position | EA | OA | beta_ed | se_ed | pval_ed | beta_pc | se_pc | pval_pc |
| rs34394051 | 1 | 6853091 | A | G | -0.012 | 0.002 | 2.30E-09 | -0.011 | 0.003 | 0.00021 |
| rs11121177 | 1 | 8445946 | A | G | 0.015 | 0.002 | 1.44E-16 | 0.007 | 0.003 | 0.012 |
| rs10799615 | 1 | 20541370 | A | G | -0.009 | 0.002 | 4.11E-08 | 0.001 | 0.003 | 0.73 |
| rs590013 | 1 | 29155738 | T | C | 0.010 | 0.002 | 2.47E-11 | 0.004 | 0.002 | 0.087 |
| rs10798888 | 1 | 32199099 | T | G | -0.014 | 0.002 | 5.15E-14 | -0.008 | 0.003 | 0.0073 |
| rs2364544 | 1 | 41833162 | A | G | -0.011 | 0.001 | 2.91E-13 | -0.006 | 0.002 | 0.008 |
| rs12076635 | 1 | 44026656 | C | G | 0.021 | 0.002 | 3.33E-34 | -0.002 | 0.003 | 0.37 |
| rs1738050 | 1 | 44707295 | C | G | -0.009 | 0.001 | 1.42E-10 | 0.000 | 0.002 | 0.91 |
| rs11211123 | 1 | 45957609 | A | G | 0.010 | 0.002 | 7.62E-10 | 0.003 | 0.003 | 0.2 |
| rs2764684 | 1 | 58537919 | T | C | 0.014 | 0.002 | 9.99E-15 | 0.004 | 0.003 | 0.21 |
| rs2989476 | 1 | 61059259 | C | G | 0.008 | 0.001 | 3.73E-08 | -0.001 | 0.002 | 0.61 |
| rs4915735 | 1 | 61808444 | A | G | 0.012 | 0.002 | 9.56E-09 | 0.004 | 0.003 | 0.19 |
| rs34305371 | 1 | 72733610 | A | G | 0.031 | 0.002 | 1.10E-39 | 0.015 | 0.004 | 0.000036 |
| rs79994730 | 1 | 75562222 | T | C | 0.024 | 0.004 | 5.88E-12 | 0.000 | 0.005 | 0.98 |
| rs1008078 | 1 | 91189731 | T | C | -0.018 | 0.001 | 4.50E-37 | -0.003 | 0.002 | 0.14 |
| rs6690195 | 1 | 96221653 | T | C | -0.011 | 0.001 | 7.89E-16 | -0.007 | 0.002 | 0.0023 |
| rs10875121 | 1 | 98417446 | C | G | 0.017 | 0.002 | 4.46E-20 | 0.004 | 0.003 | 0.2 |
| rs575113 | 1 | 110046373 | A | G | 0.013 | 0.002 | 7.52E-18 | 0.016 | 0.002 | 1.2E-10 |
| rs76577427 | 1 | 110761099 | C | G | 0.020 | 0.002 | 7.78E-18 | 0.013 | 0.004 | 0.00028 |
| rs4839155 | 1 | 112161489 | T | G | 0.012 | 0.002 | 4.66E-12 | 0.007 | 0.003 | 0.0092 |
| rs3026996 | 1 | 159167290 | A | C | 0.014 | 0.002 | 5.42E-17 | 0.011 | 0.003 | 8.2E-06 |
| rs34720381 | 1 | 171455322 | T | C | -0.018 | 0.002 | 1.90E-13 | 0.006 | 0.004 | 0.12 |
| rs6697584 | 1 | 181509718 | T | C | -0.013 | 0.002 | 1.43E-13 | -0.001 | 0.003 | 0.66 |
| rs1747817 | 1 | 197711492 | T | C | -0.009 | 0.002 | 2.29E-08 | 0.004 | 0.003 | 0.12 |
| rs1890132 | 1 | 199427321 | T | C | -0.009 | 0.002 | 6.56E-09 | 0.000 | 0.002 | 0.9 |
| rs8024 | 1 | 201845575 | A | C | -0.012 | 0.002 | 1.13E-14 | -0.006 | 0.002 | 0.014 |
| rs11588857 | 1 | 204587047 | A | G | 0.020 | 0.002 | 4.45E-31 | 0.006 | 0.003 | 0.038 |
| rs3111251 | 1 | 211409844 | T | C | -0.009 | 0.001 | 3.88E-10 | -0.002 | 0.002 | 0.36 |
| rs4846724 | 1 | 221967817 | A | G | 0.010 | 0.001 | 2.89E-12 | 0.005 | 0.002 | 0.032 |
| rs1329125 | 1 | 234740880 | T | C | -0.010 | 0.002 | 8.37E-11 | -0.003 | 0.002 | 0.21 |
| rs114593137 | 1 | 241876565 | A | T | -0.012 | 0.002 | 1.65E-12 | -0.011 | 0.003 | 0.00006 |
| rs3897821 | 1 | 243420388 | A | G | 0.015 | 0.002 | 3.58E-25 | 0.009 | 0.002 | 0.00012 |
| rs12614263 | 2 | 4123797 | A | G | 0.008 | 0.001 | 3.38E-09 | 0.002 | 0.002 | 0.26 |
| rs7603132 | 2 | 4951548 | A | G | 0.014 | 0.002 | 1.20E-14 | 0.005 | 0.003 | 0.092 |
| rs76076331 | 2 | 10977585 | T | C | 0.021 | 0.002 | 1.71E-23 | 0.003 | 0.003 | 0.4 |
| rs1991585 | 2 | 16647170 | T | C | -0.010 | 0.002 | 6.26E-11 | -0.002 | 0.002 | 0.34 |
| rs4358081 | 2 | 29100642 | A | C | -0.010 | 0.001 | 1.28E-11 | -0.004 | 0.002 | 0.11 |
| rs12468040 | 2 | 44854981 | T | G | 0.014 | 0.001 | 3.12E-21 | -0.001 | 0.002 | 0.76 |
| rs17502934 | 2 | 50994255 | T | G | -0.017 | 0.002 | 2.12E-17 | -0.007 | 0.003 | 0.032 |
| rs3948495 | 2 | 57383133 | T | G | -0.009 | 0.001 | 3.95E-10 | -0.003 | 0.002 | 0.15 |
| rs10189857 | 2 | 60713235 | A | G | 0.016 | 0.001 | 4.69E-29 | 0.005 | 0.002 | 0.032 |
| rs6731373 | 2 | 68503044 | A | G | -0.012 | 0.002 | 1.39E-14 | -0.009 | 0.002 | 0.000079 |
| rs112806496 | 2 | 80421805 | C | G | -0.016 | 0.003 | 1.72E-10 | -0.006 | 0.004 | 0.11 |
| rs11894424 | 2 | 80588091 | A | C | 0.021 | 0.003 | 2.14E-10 | -0.001 | 0.005 | 0.89 |
| rs62155350 | 2 | 98932980 | A | G | 0.034 | 0.005 | 1.69E-10 | 0.019 | 0.008 | 0.017 |
| rs13018640 | 2 | 100821545 | T | C | -0.022 | 0.001 | 1.05E-50 | -0.006 | 0.002 | 0.0045 |
| rs2570497 | 2 | 104441546 | T | C | -0.012 | 0.002 | 1.27E-16 | -0.007 | 0.002 | 0.0023 |
| rs62155873 | 2 | 105969362 | T | C | -0.015 | 0.002 | 1.65E-12 | -0.002 | 0.003 | 0.57 |
| rs13402497 | 2 | 115826318 | A | T | -0.008 | 0.001 | 1.33E-08 | -0.001 | 0.002 | 0.78 |
| rs4848924 | 2 | 124991887 | A | C | -0.012 | 0.002 | 3.56E-14 | -0.006 | 0.002 | 0.024 |
| rs74787922 | 2 | 141598681 | A | C | 0.018 | 0.003 | 8.97E-11 | 0.002 | 0.004 | 0.7 |
| rs77609760 | 2 | 142358950 | A | G | 0.024 | 0.003 | 7.71E-17 | 0.009 | 0.004 | 0.034 |
| rs1320139 | 2 | 144158177 | C | G | -0.015 | 0.001 | 7.62E-26 | 0.000 | 0.002 | 0.87 |
| rs7575637 | 2 | 145796251 | A | G | 0.011 | 0.001 | 3.43E-14 | 0.007 | 0.002 | 0.0031 |
| rs13422673 | 2 | 155474355 | T | C | -0.011 | 0.001 | 6.48E-15 | -0.006 | 0.002 | 0.0035 |
| rs11678980 | 2 | 162101261 | A | G | -0.017 | 0.001 | 1.60E-31 | -0.008 | 0.002 | 0.0008 |
| rs17428076 | 2 | 172851936 | C | G | 0.014 | 0.002 | 6.41E-17 | 0.002 | 0.003 | 0.49 |
| rs10193498 | 2 | 174094345 | A | T | 0.010 | 0.002 | 3.10E-09 | 0.005 | 0.003 | 0.078 |
| rs72917504 | 2 | 175225018 | T | C | -0.018 | 0.003 | 2.91E-09 | -1.12E-06 | 0.005 | 1 |
| rs62174974 | 2 | 185053385 | A | G | -0.011 | 0.002 | 2.76E-09 | -0.008 | 0.003 | 0.0058 |
| rs62179650 | 2 | 189135884 | A | G | 0.011 | 0.002 | 5.66E-13 | 0.006 | 0.002 | 0.014 |
| rs66721975 | 2 | 191701185 | A | G | -0.009 | 0.002 | 1.51E-08 | -0.009 | 0.002 | 0.00044 |
| rs10931821 | 2 | 199495830 | A | T | -0.014 | 0.001 | 8.65E-24 | -0.011 | 0.002 | 1.4E-06 |
| rs4675248 | 2 | 202880230 | A | G | -0.009 | 0.001 | 4.50E-11 | -0.007 | 0.002 | 0.0014 |
| rs62182994 | 2 | 212599303 | T | C | 0.014 | 0.002 | 2.55E-19 | 0.012 | 0.002 | 4.7E-07 |
| rs4673840 | 2 | 215363241 | T | C | -0.014 | 0.002 | 5.86E-13 | -0.015 | 0.003 | 7.2E-07 |
| rs1542354 | 2 | 221814085 | A | G | -0.008 | 0.001 | 2.72E-09 | -0.003 | 0.002 | 0.24 |
| rs4500930 | 2 | 228985505 | T | C | -0.011 | 0.002 | 1.67E-13 | -0.003 | 0.002 | 0.26 |
| rs6704768 | 2 | 233592501 | A | G | -0.012 | 0.001 | 1.84E-16 | -0.006 | 0.002 | 0.0095 |
| rs4663617 | 2 | 236744626 | A | T | 0.010 | 0.002 | 4.12E-10 | 0.001 | 0.003 | 0.71 |
| rs72993796 | 2 | 240321051 | T | C | 0.013 | 0.002 | 1.12E-08 | -0.001 | 0.003 | 0.69 |
| rs17048855 | 3 | 8258174 | A | G | 0.011 | 0.002 | 3.10E-13 | 0.010 | 0.002 | 6.5E-06 |
| rs382196 | 3 | 9145554 | T | G | -0.008 | 0.001 | 1.47E-08 | -0.008 | 0.002 | 0.00057 |
| rs9882532 | 3 | 16865845 | T | C | 0.012 | 0.002 | 2.15E-17 | -0.003 | 0.002 | 0.18 |
| rs13085461 | 3 | 24950387 | C | G | 0.010 | 0.001 | 2.40E-13 | 0.002 | 0.002 | 0.43 |
| rs73055556 | 3 | 28027538 | A | G | 0.011 | 0.002 | 4.95E-08 | 0.006 | 0.003 | 0.068 |
| rs4328757 | 3 | 36938180 | T | C | 0.010 | 0.001 | 2.95E-11 | 0.001 | 0.002 | 0.59 |
| rs9859556 | 3 | 49455986 | T | G | 0.029 | 0.002 | 4.61E-82 | 0.015 | 0.002 | 2.4E-10 |
| rs77719387 | 3 | 49917021 | A | T | -0.040 | 0.006 | 3.78E-12 | -0.008 | 0.009 | 0.39 |
| rs11130380 | 3 | 53758950 | T | G | 0.009 | 0.001 | 4.17E-10 | 0.006 | 0.002 | 0.014 |
| rs12638072 | 3 | 65654480 | A | G | -0.011 | 0.002 | 3.13E-12 | -0.004 | 0.002 | 0.086 |
| rs115000530 | 3 | 69930625 | A | T | -0.025 | 0.003 | 1.09E-15 | -0.012 | 0.005 | 0.014 |
| rs55736314 | 3 | 71586293 | C | G | -0.015 | 0.001 | 4.59E-27 | 0.001 | 0.002 | 0.69 |
| rs66568921 | 3 | 85672018 | T | G | -0.018 | 0.002 | 7.60E-34 | -0.004 | 0.002 | 0.11 |
| rs9853928 | 3 | 103295384 | T | C | -0.013 | 0.002 | 1.59E-13 | -0.003 | 0.003 | 0.3 |
| rs28513882 | 3 | 107296628 | A | G | -0.011 | 0.002 | 2.03E-09 | -0.005 | 0.003 | 0.055 |
| rs2290601 | 3 | 108112760 | T | C | -0.015 | 0.002 | 4.49E-19 | -0.003 | 0.003 | 0.24 |
| rs1717204 | 3 | 118461145 | A | C | -0.012 | 0.002 | 1.20E-10 | -0.003 | 0.003 | 0.22 |
| rs34067381 | 3 | 123594419 | T | G | -0.010 | 0.002 | 9.77E-13 | -0.004 | 0.002 | 0.13 |
| rs9289300 | 3 | 127144988 | T | C | -0.016 | 0.002 | 1.00E-17 | -0.005 | 0.003 | 0.1 |
| rs7650602 | 3 | 141147414 | T | C | -0.009 | 0.001 | 2.77E-10 | -0.010 | 0.002 | 2.9E-06 |
| rs2885198 | 3 | 143636421 | A | G | 0.009 | 0.001 | 4.63E-10 | 0.001 | 0.002 | 0.63 |
| rs12054166 | 3 | 150093445 | C | G | 0.010 | 0.002 | 2.30E-09 | -0.001 | 0.003 | 0.77 |
| rs7617204 | 3 | 160821969 | A | G | -0.010 | 0.001 | 4.33E-12 | -0.001 | 0.002 | 0.6 |
| rs72622559 | 3 | 175672897 | T | C | -0.012 | 0.002 | 5.50E-13 | 0.000 | 0.003 | 0.97 |
| rs77025239 | 3 | 180734185 | A | G | -0.014 | 0.002 | 4.65E-13 | -0.011 | 0.003 | 0.00024 |
| rs13327482 | 3 | 196788363 | A | G | -0.012 | 0.002 | 4.47E-11 | -0.003 | 0.003 | 0.26 |
| rs150537577 | 4 | 705629 | A | G | -0.015 | 0.003 | 9.50E-09 | -0.013 | 0.004 | 0.0026 |
| rs363096 | 4 | 3180021 | T | C | -0.014 | 0.001 | 5.77E-24 | -0.008 | 0.002 | 0.00017 |
| rs13133213 | 4 | 15646794 | A | G | 0.010 | 0.001 | 7.57E-12 | -0.002 | 0.002 | 0.41 |
| rs2011603 | 4 | 18025484 | A | G | -0.009 | 0.002 | 1.12E-08 | -0.004 | 0.002 | 0.083 |
| rs4467547 | 4 | 21945933 | T | G | 0.012 | 0.001 | 4.88E-17 | 0.003 | 0.002 | 0.24 |
| rs10021733 | 4 | 28609476 | T | C | -0.016 | 0.003 | 9.45E-10 | 0.005 | 0.003 | 0.072 |
| rs6812533 | 4 | 39687838 | T | C | 0.011 | 0.002 | 4.85E-11 | 0.015 | 0.003 | 9.7E-09 |
| rs13130765 | 4 | 45163333 | C | G | -0.009 | 0.001 | 1.89E-10 | -0.006 | 0.002 | 0.0068 |
| rs2055940 | 4 | 46997913 | A | G | 0.008 | 0.002 | 4.95E-08 | 0.003 | 0.002 | 0.23 |
| rs1505676 | 4 | 62127291 | C | G | 0.008 | 0.002 | 6.67E-09 | 0.001 | 0.002 | 0.71 |
| rs12506221 | 4 | 67899235 | T | G | -0.013 | 0.001 | 4.23E-20 | -0.007 | 0.002 | 0.0027 |
| rs2034631 | 4 | 82225529 | T | C | 0.009 | 0.002 | 2.64E-09 | 0.005 | 0.002 | 0.023 |
| rs7692359 | 4 | 83209346 | T | C | -0.011 | 0.002 | 1.40E-10 | -0.002 | 0.003 | 0.37 |
| rs12503522 | 4 | 94543233 | T | C | -0.011 | 0.002 | 3.93E-13 | -0.004 | 0.002 | 0.11 |
| rs7683416 | 4 | 106152984 | T | C | 0.014 | 0.001 | 6.05E-23 | 0.005 | 0.002 | 0.024 |
| rs1955250 | 4 | 118514236 | A | C | -0.014 | 0.003 | 2.26E-08 | -0.001 | 0.004 | 0.77 |
| rs13145650 | 4 | 122963344 | T | C | -0.017 | 0.003 | 8.99E-12 | -0.010 | 0.004 | 0.0091 |
| rs10006235 | 4 | 130670108 | T | C | -0.009 | 0.002 | 4.48E-09 | -0.003 | 0.002 | 0.16 |
| rs12643771 | 4 | 140753103 | T | C | 0.013 | 0.002 | 1.32E-17 | -0.006 | 0.002 | 0.017 |
| rs969512 | 4 | 147872742 | A | T | -0.011 | 0.002 | 9.84E-13 | -0.004 | 0.002 | 0.1 |
| rs7672622 | 4 | 157705551 | A | G | -0.009 | 0.002 | 4.72E-08 | -0.001 | 0.003 | 0.84 |
| rs2081652 | 4 | 159862913 | A | T | 0.012 | 0.002 | 1.09E-16 | 0.015 | 0.002 | 1.1E-10 |
| rs12646523 | 4 | 170944698 | T | C | -0.013 | 0.002 | 3.79E-16 | -0.004 | 0.003 | 0.087 |
| rs17598675 | 4 | 176647637 | T | C | -0.012 | 0.001 | 1.37E-16 | -0.006 | 0.002 | 0.0098 |
| rs72709560 | 4 | 178540163 | A | G | 0.009 | 0.002 | 2.03E-08 | 0.002 | 0.002 | 0.42 |
| rs1128956 | 4 | 183724005 | T | G | -0.014 | 0.002 | 5.34E-14 | -0.007 | 0.003 | 0.022 |
| rs11724690 | 4 | 186764747 | T | G | 0.009 | 0.002 | 2.08E-09 | 0.006 | 0.002 | 0.013 |
| rs13163845 | 5 | 3264389 | T | C | -0.015 | 0.002 | 4.66E-14 | -0.001 | 0.003 | 0.66 |
| rs162445 | 5 | 7943246 | A | G | 0.015 | 0.003 | 5.72E-09 | 0.005 | 0.004 | 0.24 |
| rs2434672 | 5 | 11471879 | A | C | 0.009 | 0.001 | 1.06E-10 | 0.003 | 0.002 | 0.21 |
| rs17563464 | 5 | 26913774 | A | C | -0.015 | 0.002 | 1.74E-17 | -0.008 | 0.003 | 0.005 |
| rs10940921 | 5 | 30808645 | T | G | 0.011 | 0.001 | 1.31E-14 | 0.003 | 0.002 | 0.16 |
| rs2964199 | 5 | 57532775 | T | C | -0.010 | 0.002 | 4.61E-11 | -0.003 | 0.002 | 0.28 |
| rs6449503 | 5 | 60095272 | A | G | 0.019 | 0.001 | 1.84E-39 | 0.009 | 0.002 | 0.000062 |
| rs10805383 | 5 | 63034606 | A | G | -0.010 | 0.001 | 2.22E-13 | 0.002 | 0.002 | 0.39 |
| rs42302 | 5 | 74965554 | A | G | 0.009 | 0.002 | 5.96E-09 | 0.006 | 0.002 | 0.011 |
| rs61104616 | 5 | 88163771 | A | G | -0.017 | 0.001 | 1.56E-34 | -0.009 | 0.002 | 0.000062 |
| rs10046069 | 5 | 89115576 | A | G | 0.013 | 0.002 | 4.40E-09 | 0.006 | 0.003 | 0.097 |
| rs12332731 | 5 | 93033082 | A | T | 0.014 | 0.002 | 5.96E-14 | 0.003 | 0.003 | 0.36 |
| rs252991 | 5 | 106767346 | A | G | 0.010 | 0.002 | 6.30E-11 | 0.004 | 0.002 | 0.059 |
| rs17489649 | 5 | 109156184 | A | G | 0.013 | 0.002 | 2.77E-17 | 0.003 | 0.002 | 0.15 |
| rs660001 | 5 | 113866598 | A | G | -0.016 | 0.002 | 1.66E-21 | -0.015 | 0.003 | 1.7E-08 |
| rs62379838 | 5 | 120102028 | T | C | 0.012 | 0.002 | 1.77E-15 | -0.002 | 0.002 | 0.34 |
| rs10060023 | 5 | 124304677 | T | C | 0.011 | 0.002 | 1.88E-13 | 0.002 | 0.002 | 0.47 |
| rs1434630 | 5 | 136557055 | T | G | -0.014 | 0.002 | 4.63E-12 | -0.003 | 0.003 | 0.29 |
| rs12519073 | 5 | 136776762 | T | C | -0.011 | 0.002 | 1.16E-10 | -0.007 | 0.003 | 0.0048 |
| rs2964255 | 5 | 152052332 | A | G | 0.009 | 0.002 | 6.34E-09 | -0.006 | 0.002 | 0.016 |
| rs182902112 | 5 | 153304758 | A | C | -0.030 | 0.005 | 1.17E-08 | -0.016 | 0.008 | 0.049 |
| rs42210 | 5 | 166408788 | C | G | -0.010 | 0.002 | 2.74E-10 | 0.002 | 0.002 | 0.35 |
| rs2545795 | 5 | 176887106 | A | C | 0.013 | 0.001 | 1.03E-18 | 0.008 | 0.002 | 0.00033 |
| rs12524795 | 6 | 3464074 | T | C | 0.010 | 0.001 | 5.28E-13 | 0.006 | 0.002 | 0.012 |
| rs112603734 | 6 | 6069588 | A | C | -0.013 | 0.002 | 2.72E-09 | -0.004 | 0.003 | 0.13 |
| rs72829857 | 6 | 16966052 | A | G | -0.015 | 0.002 | 1.52E-19 | -0.002 | 0.003 | 0.48 |
| rs72828517 | 6 | 19036035 | T | C | -0.017 | 0.002 | 1.38E-19 | -0.016 | 0.003 | 1.7E-08 |
| rs2179152 | 6 | 26325888 | T | C | -0.013 | 0.002 | 1.68E-19 | -0.004 | 0.002 | 0.052 |
| rs2256965 | 6 | 31555130 | A | G | 0.011 | 0.001 | 2.40E-13 | 0.005 | 0.002 | 0.027 |
| rs57349798 | 6 | 37486052 | A | G | 0.009 | 0.001 | 8.63E-11 | -0.003 | 0.002 | 0.26 |
| rs2436760 | 6 | 40374288 | T | C | 0.023 | 0.004 | 4.52E-08 | 0.007 | 0.006 | 0.31 |
| rs912883 | 6 | 41552040 | T | C | 0.009 | 0.002 | 6.20E-09 | 0.001 | 0.002 | 0.79 |
| rs78648104 | 6 | 50683009 | T | C | -0.014 | 0.003 | 1.70E-08 | 0.008 | 0.004 | 0.036 |
| rs7449561 | 6 | 66215549 | A | G | 0.010 | 0.002 | 9.73E-10 | 0.010 | 0.003 | 0.00024 |
| rs13212041 | 6 | 78171124 | T | C | 0.010 | 0.002 | 7.42E-09 | 0.005 | 0.003 | 0.046 |
| rs4352658 | 6 | 88279872 | T | C | -0.019 | 0.003 | 1.71E-13 | -0.007 | 0.004 | 0.091 |
| rs9359939 | 6 | 92133241 | A | C | -0.011 | 0.002 | 1.51E-10 | -0.006 | 0.003 | 0.024 |
| rs9375188 | 6 | 98555272 | T | C | 0.021 | 0.001 | 8.78E-52 | 0.017 | 0.002 | 4.4E-15 |
| rs10080647 | 6 | 114742335 | A | C | 0.012 | 0.002 | 7.05E-10 | 0.001 | 0.003 | 0.74 |
| rs11542663 | 6 | 119215402 | A | C | 0.011 | 0.002 | 2.44E-13 | -0.001 | 0.002 | 0.76 |
| rs13197257 | 6 | 128333682 | T | G | 0.011 | 0.002 | 2.83E-12 | 0.003 | 0.002 | 0.21 |
| rs9492774 | 6 | 131302489 | C | G | 0.008 | 0.002 | 4.33E-08 | 0.004 | 0.002 | 0.083 |
| rs9373363 | 6 | 143150043 | A | G | -0.011 | 0.002 | 5.54E-12 | -0.001 | 0.003 | 0.56 |
| rs4895650 | 6 | 145024122 | T | C | 0.009 | 0.001 | 5.31E-11 | 0.001 | 0.002 | 0.81 |
| rs6557171 | 6 | 152234593 | T | C | -0.016 | 0.002 | 5.48E-25 | -0.006 | 0.002 | 0.0061 |
| rs1933264 | 6 | 153391618 | T | C | 0.010 | 0.002 | 5.85E-10 | -0.002 | 0.003 | 0.34 |
| rs6917154 | 6 | 167135156 | T | C | 0.013 | 0.002 | 2.55E-09 | -0.004 | 0.003 | 0.28 |
| rs6924023 | 6 | 170076041 | A | G | 0.011 | 0.002 | 5.17E-11 | 0.006 | 0.003 | 0.016 |
| rs13240401 | 7 | 2194396 | T | C | 0.018 | 0.002 | 2.36E-24 | 0.008 | 0.003 | 0.0029 |
| rs4719944 | 7 | 3496032 | T | C | -0.012 | 0.001 | 9.25E-18 | -0.003 | 0.002 | 0.12 |
| rs929511 | 7 | 8003017 | T | C | -0.016 | 0.002 | 3.91E-14 | -0.011 | 0.003 | 0.0013 |
| rs118134876 | 7 | 11500372 | T | C | -0.022 | 0.003 | 1.50E-13 | -0.003 | 0.005 | 0.59 |
| rs6977237 | 7 | 14027991 | T | C | 0.008 | 0.001 | 5.59E-09 | -0.001 | 0.002 | 0.63 |
| rs35929923 | 7 | 21400525 | A | G | -0.011 | 0.002 | 1.77E-12 | 0.000 | 0.003 | 0.94 |
| rs79265434 | 7 | 24621381 | A | G | -0.020 | 0.002 | 1.98E-19 | -0.011 | 0.003 | 0.0017 |
| rs10951590 | 7 | 39091043 | T | C | -0.011 | 0.002 | 3.62E-13 | -0.005 | 0.002 | 0.038 |
| rs12670376 | 7 | 48823154 | A | G | 0.009 | 0.001 | 5.99E-11 | 0.002 | 0.002 | 0.47 |
| rs17551064 | 7 | 49869771 | A | G | 0.014 | 0.002 | 1.53E-13 | -0.001 | 0.003 | 0.65 |
| rs1220779 | 7 | 54734673 | A | G | -0.008 | 0.001 | 1.58E-08 | -0.004 | 0.002 | 0.1 |
| rs35417702 | 7 | 71739916 | T | C | -0.015 | 0.001 | 2.40E-27 | -0.007 | 0.002 | 0.00072 |
| rs1167827 | 7 | 75163169 | A | G | 0.010 | 0.001 | 2.93E-13 | 0.001 | 0.002 | 0.51 |
| rs10215082 | 7 | 92657985 | A | G | -0.015 | 0.001 | 7.03E-26 | -0.008 | 0.002 | 0.00014 |
| rs401966 | 7 | 101695817 | C | G | -0.010 | 0.001 | 1.72E-11 | 0.006 | 0.002 | 0.0053 |
| rs9655780 | 7 | 104667334 | A | G | -0.015 | 0.002 | 3.70E-16 | -0.002 | 0.003 | 0.51 |
| rs12113634 | 7 | 111996952 | T | C | 0.009 | 0.002 | 2.50E-09 | 0.003 | 0.002 | 0.15 |
| rs113615161 | 7 | 114519339 | T | C | -0.012 | 0.002 | 2.98E-09 | -0.009 | 0.003 | 0.0052 |
| rs6969783 | 7 | 117593308 | A | T | -0.010 | 0.001 | 2.87E-12 | 0.001 | 0.002 | 0.62 |
| rs17144467 | 7 | 122098782 | A | C | 0.010 | 0.002 | 8.74E-11 | -0.001 | 0.002 | 0.74 |
| rs113520408 | 7 | 128402782 | A | G | 0.016 | 0.002 | 1.03E-22 | 0.006 | 0.002 | 0.02 |
| rs57352738 | 7 | 133304694 | A | T | -0.015 | 0.002 | 2.51E-18 | -0.015 | 0.003 | 7E-08 |
| rs4726070 | 7 | 151328218 | A | G | 0.012 | 0.001 | 1.36E-17 | 0.004 | 0.002 | 0.11 |
| rs73191311 | 8 | 4796094 | A | G | -0.009 | 0.002 | 2.03E-10 | -0.002 | 0.002 | 0.38 |
| rs6994287 | 8 | 9340932 | A | G | 0.010 | 0.001 | 1.11E-12 | 0.004 | 0.002 | 0.073 |
| rs2517086 | 8 | 17042416 | C | G | -0.009 | 0.001 | 8.00E-10 | 0.001 | 0.002 | 0.8 |
| rs13266287 | 8 | 17871598 | A | T | -0.009 | 0.002 | 1.75E-08 | 0.001 | 0.002 | 0.76 |
| rs34807077 | 8 | 28680769 | A | C | 0.013 | 0.002 | 3.29E-11 | -0.002 | 0.003 | 0.41 |
| rs2725370 | 8 | 30852826 | T | C | -0.014 | 0.002 | 4.96E-20 | -0.004 | 0.002 | 0.092 |
| rs2923424 | 8 | 42382222 | A | G | 0.012 | 0.001 | 6.31E-17 | 0.001 | 0.002 | 0.54 |
| rs2929032 | 8 | 56371745 | A | G | -0.008 | 0.001 | 8.40E-09 | -0.004 | 0.002 | 0.086 |
| rs1866823 | 8 | 57436577 | A | G | 0.011 | 0.001 | 3.09E-14 | 0.001 | 0.002 | 0.54 |
| rs56099375 | 8 | 77372988 | T | C | 0.012 | 0.002 | 3.10E-13 | 0.001 | 0.003 | 0.61 |
| rs4320563 | 8 | 87769961 | A | G | 0.011 | 0.001 | 2.24E-14 | 0.001 | 0.002 | 0.54 |
| rs35606437 | 8 | 93327532 | A | G | 0.011 | 0.002 | 3.98E-11 | 0.008 | 0.002 | 0.0011 |
| rs7012546 | 8 | 105067737 | T | C | 0.009 | 0.001 | 1.25E-10 | 0.002 | 0.002 | 0.41 |
| rs72672052 | 8 | 115798424 | A | T | -0.010 | 0.002 | 4.87E-08 | 0.000 | 0.003 | 0.91 |
| rs4298514 | 8 | 118932887 | T | C | 0.010 | 0.002 | 1.07E-11 | 0.008 | 0.002 | 0.00069 |
| rs13261773 | 8 | 120187090 | C | G | 0.012 | 0.002 | 3.12E-10 | 0.008 | 0.003 | 0.012 |
| rs401526 | 8 | 130929166 | T | C | -0.010 | 0.001 | 1.15E-13 | -0.005 | 0.002 | 0.03 |
| rs746839 | 8 | 142617261 | C | G | 0.014 | 0.002 | 1.19E-20 | 0.006 | 0.002 | 0.0082 |
| rs7460106 | 8 | 143534777 | T | C | -0.012 | 0.002 | 2.06E-13 | -0.014 | 0.003 | 9.9E-08 |
| rs7849487 | 9 | 1757274 | T | G | -0.017 | 0.002 | 1.25E-29 | -0.003 | 0.002 | 0.21 |
| rs73648455 | 9 | 14077378 | T | C | -0.018 | 0.003 | 1.99E-12 | -0.006 | 0.004 | 0.13 |
| rs7875078 | 9 | 14494845 | A | C | -0.008 | 0.001 | 2.72E-08 | 0.002 | 0.002 | 0.42 |
| rs7029718 | 9 | 23358495 | A | G | 0.024 | 0.001 | 7.82E-65 | 0.020 | 0.002 | 7.5E-19 |
| rs4977885 | 9 | 23687983 | A | G | -0.008 | 0.001 | 6.78E-09 | -0.004 | 0.002 | 0.069 |
| rs7041702 | 9 | 33053430 | A | G | -0.011 | 0.002 | 4.53E-12 | -0.004 | 0.003 | 0.12 |
| rs11138947 | 9 | 72110562 | T | C | 0.011 | 0.002 | 2.36E-11 | 0.006 | 0.002 | 0.011 |
| rs9886703 | 9 | 82246351 | A | T | -0.014 | 0.002 | 4.46E-13 | 0.001 | 0.003 | 0.83 |
| rs7040995 | 9 | 92226172 | C | G | 0.010 | 0.001 | 5.39E-12 | 0.006 | 0.002 | 0.004 |
| rs10761251 | 9 | 96421546 | A | T | 0.011 | 0.002 | 2.52E-14 | 0.001 | 0.002 | 0.72 |
| rs10979613 | 9 | 111687584 | T | C | -0.012 | 0.002 | 1.25E-15 | -0.002 | 0.002 | 0.34 |
| rs76235882 | 9 | 121096681 | A | G | 0.024 | 0.004 | 4.52E-10 | 0.013 | 0.007 | 0.05 |
| rs12375949 | 9 | 124617900 | T | C | -0.014 | 0.001 | 1.61E-22 | -0.008 | 0.002 | 0.00069 |
| rs2039204 | 9 | 130371107 | A | T | 0.008 | 0.001 | 5.23E-09 | 0.002 | 0.002 | 0.45 |
| rs9411331 | 9 | 134883419 | A | C | 0.015 | 0.002 | 7.63E-22 | 0.006 | 0.002 | 0.015 |
| rs73581580 | 9 | 140251458 | A | G | -0.018 | 0.002 | 5.78E-16 | 0.008 | 0.003 | 0.011 |
| rs7894722 | 10 | 9972046 | T | C | 0.009 | 0.001 | 5.41E-09 | 0.002 | 0.002 | 0.47 |
| rs17148998 | 10 | 10909401 | A | G | 0.010 | 0.002 | 6.98E-09 | 0.010 | 0.003 | 0.00042 |
| rs1747714 | 10 | 11161316 | T | C | 0.011 | 0.001 | 3.82E-14 | 0.004 | 0.002 | 0.076 |
| rs2183271 | 10 | 21957229 | T | C | 0.008 | 0.002 | 1.91E-08 | -0.008 | 0.002 | 0.00043 |
| rs11598765 | 10 | 23953725 | A | G | -0.011 | 0.002 | 1.73E-09 | -0.002 | 0.003 | 0.57 |
| rs806816 | 10 | 32285942 | A | T | 0.010 | 0.002 | 4.80E-10 | -0.001 | 0.003 | 0.58 |
| rs2007655 | 10 | 34586689 | T | G | 0.009 | 0.001 | 8.64E-10 | -0.001 | 0.002 | 0.7 |
| rs11003463 | 10 | 51801793 | T | G | 0.009 | 0.002 | 5.58E-10 | 0.005 | 0.002 | 0.041 |
| rs7924036 | 10 | 65191645 | T | G | 0.013 | 0.001 | 2.55E-21 | 0.002 | 0.002 | 0.36 |
| rs10996167 | 10 | 66813928 | C | G | 0.011 | 0.002 | 7.47E-09 | 0.001 | 0.002 | 0.72 |
| rs7920624 | 10 | 67963186 | A | T | 0.012 | 0.001 | 1.06E-17 | 0.009 | 0.002 | 0.00007 |
| rs7910403 | 10 | 87039251 | T | G | 0.012 | 0.002 | 5.08E-12 | 0.001 | 0.003 | 0.71 |
| rs1426619 | 10 | 90091540 | T | C | 0.010 | 0.001 | 1.40E-11 | 0.000 | 0.002 | 0.85 |
| rs17882802 | 10 | 101983413 | A | G | 0.008 | 0.001 | 3.00E-09 | -0.005 | 0.002 | 0.028 |
| rs73344830 | 10 | 103816828 | A | G | 0.017 | 0.001 | 1.00E-32 | 0.005 | 0.002 | 0.014 |
| rs790647 | 10 | 106776484 | A | C | -0.015 | 0.002 | 3.81E-18 | 0.001 | 0.003 | 0.8 |
| rs12571549 | 10 | 111770013 | A | G | 0.015 | 0.002 | 1.45E-13 | 0.005 | 0.003 | 0.15 |
| rs1865955 | 10 | 118837990 | T | C | 0.012 | 0.002 | 4.06E-11 | -0.003 | 0.003 | 0.26 |
| rs4384309 | 10 | 133110596 | A | G | 0.010 | 0.001 | 4.89E-13 | 0.004 | 0.002 | 0.059 |
| rs12761761 | 10 | 133775375 | T | C | 0.015 | 0.002 | 4.79E-20 | -0.001 | 0.003 | 0.77 |
| rs12765185 | 10 | 134977077 | A | T | -0.009 | 0.002 | 2.96E-08 | -0.003 | 0.002 | 0.24 |
| rs4757957 | 11 | 12881398 | C | G | 0.013 | 0.002 | 1.79E-18 | 0.005 | 0.002 | 0.046 |
| rs11023764 | 11 | 15910939 | A | G | 0.009 | 0.002 | 5.32E-10 | 0.007 | 0.002 | 0.0023 |
| rs12789313 | 11 | 25058937 | T | C | 0.009 | 0.001 | 6.79E-11 | 0.004 | 0.002 | 0.055 |
| rs11030102 | 11 | 27681596 | C | G | 0.010 | 0.002 | 4.60E-10 | -0.006 | 0.002 | 0.01 |
| rs2199409 | 11 | 39915234 | T | C | 0.010 | 0.002 | 3.14E-09 | 0.003 | 0.003 | 0.26 |
| rs79728014 | 11 | 46062245 | A | G | -0.013 | 0.002 | 2.06E-11 | 0.001 | 0.003 | 0.71 |
| rs75033012 | 11 | 61347033 | C | G | -0.026 | 0.004 | 1.38E-11 | -0.018 | 0.006 | 0.0012 |
| rs628993 | 11 | 61539691 | A | G | 0.013 | 0.002 | 3.83E-08 | 0.001 | 0.004 | 0.8 |
| rs76878669 | 11 | 66092567 | C | G | 0.014 | 0.002 | 3.10E-17 | 0.000 | 0.003 | 0.93 |
| rs72962169 | 11 | 72365669 | T | C | -0.017 | 0.002 | 1.26E-19 | -0.003 | 0.003 | 0.3 |
| rs580652 | 11 | 84904742 | T | C | -0.015 | 0.002 | 4.65E-10 | 0.007 | 0.004 | 0.06 |
| rs10830858 | 11 | 91896638 | T | C | 0.009 | 0.001 | 5.31E-11 | 0.006 | 0.002 | 0.01 |
| rs488476 | 11 | 95543715 | C | G | -0.011 | 0.002 | 8.29E-15 | -0.002 | 0.002 | 0.48 |
| rs1301838 | 11 | 99670514 | T | C | -0.010 | 0.002 | 1.29E-10 | -0.001 | 0.002 | 0.74 |
| rs72486027 | 11 | 107167486 | T | C | -0.010 | 0.002 | 1.61E-10 | -0.005 | 0.003 | 0.042 |
| rs2212430 | 11 | 109289538 | T | C | -0.010 | 0.002 | 1.32E-10 | 1.97E-05 | 0.002 | 0.99 |
| rs11213482 | 11 | 110435636 | A | G | -0.011 | 0.002 | 2.68E-08 | 0.000 | 0.003 | 0.97 |
| rs17565975 | 11 | 111586950 | A | G | -0.011 | 0.001 | 5.54E-14 | -0.005 | 0.002 | 0.036 |
| rs10892807 | 11 | 121998254 | T | C | 0.011 | 0.001 | 1.09E-14 | 0.001 | 0.002 | 0.51 |
| rs12574281 | 11 | 131205421 | A | C | -0.008 | 0.002 | 3.04E-08 | -0.001 | 0.002 | 0.68 |
| rs9633970 | 11 | 132502212 | T | C | 0.009 | 0.002 | 2.81E-08 | 0.002 | 0.003 | 0.37 |
| rs12273435 | 11 | 133825855 | A | G | -0.013 | 0.002 | 4.72E-14 | -0.004 | 0.003 | 0.16 |
| rs4766424 | 12 | 1954096 | C | G | 0.014 | 0.002 | 2.47E-11 | 0.002 | 0.003 | 0.46 |
| rs10772644 | 12 | 13417617 | C | G | 0.014 | 0.002 | 1.69E-10 | -0.001 | 0.004 | 0.75 |
| rs1861786 | 12 | 14000467 | A | G | -0.008 | 0.001 | 2.39E-08 | -0.003 | 0.002 | 0.2 |
| rs7974852 | 12 | 14511679 | A | C | 0.012 | 0.001 | 1.14E-16 | 0.004 | 0.002 | 0.067 |
| rs1603460 | 12 | 23092626 | T | G | 0.009 | 0.001 | 2.20E-10 | 0.002 | 0.002 | 0.42 |
| rs117398064 | 12 | 26523628 | C | G | -0.014 | 0.003 | 1.16E-08 | -0.001 | 0.004 | 0.72 |
| rs7972246 | 12 | 27209328 | T | C | 0.011 | 0.002 | 1.01E-13 | -0.003 | 0.002 | 0.26 |
| rs10844179 | 12 | 32469601 | A | G | 0.010 | 0.002 | 5.03E-10 | 0.007 | 0.003 | 0.013 |
| rs80257979 | 12 | 49402562 | T | G | -0.025 | 0.004 | 3.31E-10 | -0.010 | 0.007 | 0.14 |
| rs17110109 | 12 | 54668908 | T | C | -0.009 | 0.001 | 1.47E-10 | -0.005 | 0.002 | 0.046 |
| rs1689510 | 12 | 56396768 | C | G | 0.019 | 0.002 | 1.35E-38 | 0.007 | 0.002 | 0.0017 |
| rs3751331 | 12 | 58290278 | A | G | -0.010 | 0.001 | 1.75E-11 | -0.004 | 0.002 | 0.12 |
| rs57661533 | 12 | 78629917 | T | C | -0.011 | 0.002 | 3.24E-08 | -0.010 | 0.003 | 0.0016 |
| rs1245829 | 12 | 79648170 | A | T | -0.010 | 0.001 | 3.29E-12 | -0.001 | 0.002 | 0.58 |
| rs717996 | 12 | 84133820 | T | C | -0.012 | 0.001 | 1.89E-17 | -0.002 | 0.002 | 0.48 |
| rs2216144 | 12 | 97666478 | T | C | 0.010 | 0.001 | 2.58E-12 | 0.006 | 0.002 | 0.004 |
| rs2131167 | 12 | 99633189 | A | G | 0.008 | 0.001 | 1.52E-08 | 0.005 | 0.002 | 0.036 |
| rs7977614 | 12 | 110115286 | A | G | -0.013 | 0.002 | 6.84E-17 | -0.002 | 0.002 | 0.39 |
| rs10773208 | 12 | 122970156 | T | C | -0.011 | 0.002 | 8.09E-12 | 0.002 | 0.003 | 0.5 |
| rs10773002 | 12 | 123746961 | A | T | 0.019 | 0.002 | 2.50E-30 | -6.6E-05 | 0.003 | 0.98 |
| rs6490618 | 13 | 21338343 | T | C | 0.009 | 0.002 | 3.48E-09 | 0.008 | 0.002 | 0.00082 |
| rs9529146 | 13 | 31785404 | T | C | 0.013 | 0.002 | 3.48E-14 | 0.007 | 0.003 | 0.0045 |
| rs936496 | 13 | 36100541 | A | G | -0.008 | 0.001 | 4.38E-08 | -0.005 | 0.002 | 0.04 |
| rs12875339 | 13 | 55722033 | A | C | -0.011 | 0.002 | 1.71E-13 | -0.002 | 0.002 | 0.41 |
| rs1334297 | 13 | 58335375 | A | G | 0.026 | 0.002 | 2.53E-58 | 0.007 | 0.002 | 0.0034 |
| rs7321274 | 13 | 69146186 | A | G | 0.011 | 0.002 | 8.21E-11 | 0.008 | 0.003 | 0.0054 |
| rs2478208 | 13 | 81471113 | C | G | -0.010 | 0.001 | 2.89E-13 | -0.003 | 0.002 | 0.22 |
| rs7326331 | 13 | 91615838 | A | G | -0.013 | 0.002 | 3.42E-16 | -0.007 | 0.002 | 0.0032 |
| rs11620355 | 13 | 92044587 | A | G | 0.018 | 0.003 | 1.09E-13 | 0.005 | 0.004 | 0.21 |
| rs9556958 | 13 | 99100046 | T | C | -0.011 | 0.001 | 5.96E-15 | -0.007 | 0.002 | 0.00098 |
| rs11157931 | 14 | 23403193 | A | C | -0.014 | 0.001 | 3.86E-22 | -0.005 | 0.002 | 0.035 |
| rs178183 | 14 | 26979412 | T | C | 0.015 | 0.002 | 4.81E-19 | 0.007 | 0.003 | 0.0037 |
| rs176218 | 14 | 29600506 | T | G | 0.020 | 0.002 | 1.10E-29 | 0.003 | 0.003 | 0.31 |
| rs61997667 | 14 | 57280046 | T | C | -0.014 | 0.002 | 2.24E-13 | -0.008 | 0.003 | 0.0086 |
| rs4899012 | 14 | 61003889 | C | G | -0.010 | 0.001 | 8.06E-13 | -0.007 | 0.002 | 0.0015 |
| rs12888615 | 14 | 72435705 | T | C | 0.012 | 0.002 | 9.42E-12 | 0.000 | 0.003 | 0.9 |
| rs730384 | 14 | 74889870 | A | G | 0.010 | 0.001 | 9.38E-13 | -0.003 | 0.002 | 0.17 |
| rs2998299 | 14 | 84997363 | T | G | -0.014 | 0.002 | 1.51E-15 | -0.005 | 0.003 | 0.044 |
| rs4904523 | 14 | 89723630 | A | G | -0.008 | 0.001 | 6.27E-09 | -0.002 | 0.002 | 0.32 |
| rs736281 | 14 | 94287830 | T | C | 0.009 | 0.001 | 2.49E-09 | 0.008 | 0.002 | 0.00082 |
| rs2496482 | 14 | 99750164 | T | C | 0.012 | 0.002 | 9.20E-17 | 0.009 | 0.002 | 0.00011 |
| rs8008382 | 14 | 104054425 | T | C | -0.011 | 0.002 | 4.07E-12 | -0.005 | 0.002 | 0.048 |
| rs7167688 | 15 | 26813137 | T | C | -0.009 | 0.001 | 1.71E-11 | -0.007 | 0.002 | 0.0017 |
| rs891793 | 15 | 27256021 | C | G | -0.010 | 0.001 | 1.87E-12 | -0.003 | 0.002 | 0.19 |
| rs6493265 | 15 | 47513253 | T | C | -0.012 | 0.001 | 2.26E-17 | -0.006 | 0.002 | 0.014 |
| rs12438177 | 15 | 55951432 | A | G | 0.009 | 0.002 | 1.48E-10 | 0.008 | 0.002 | 0.00069 |
| rs7171405 | 15 | 61466893 | A | G | -0.010 | 0.002 | 7.53E-10 | 8.42E-05 | 0.003 | 0.97 |
| rs12591647 | 15 | 65959729 | T | C | -0.015 | 0.002 | 7.78E-17 | -0.009 | 0.003 | 0.0017 |
| rs1564347 | 15 | 73438848 | T | G | 0.009 | 0.002 | 2.39E-09 | 0.004 | 0.002 | 0.082 |
| rs2469226 | 15 | 77246990 | A | T | 0.010 | 0.002 | 1.28E-08 | 0.006 | 0.003 | 0.035 |
| rs56391344 | 15 | 78006899 | A | G | 0.015 | 0.002 | 7.49E-20 | 0.002 | 0.003 | 0.34 |
| rs4778058 | 15 | 93456069 | T | C | -0.009 | 0.001 | 4.92E-11 | 0.000 | 0.002 | 0.86 |
| rs4984541 | 15 | 96911139 | A | G | -0.014 | 0.002 | 3.40E-16 | -0.008 | 0.003 | 0.0018 |
| rs8030487 | 15 | 97119595 | A | G | 0.009 | 0.002 | 2.52E-08 | 0.004 | 0.002 | 0.065 |
| rs9933256 | 16 | 1246748 | A | G | 0.012 | 0.001 | 6.06E-17 | 0.006 | 0.002 | 0.0055 |
| rs11076962 | 16 | 5811367 | T | C | 0.009 | 0.002 | 4.29E-08 | 0.004 | 0.002 | 0.092 |
| rs34098770 | 16 | 12237812 | A | G | -0.016 | 0.002 | 2.95E-16 | -0.005 | 0.003 | 0.079 |
| rs9927842 | 16 | 15153717 | T | C | -0.013 | 0.002 | 6.22E-11 | 0.004 | 0.003 | 0.22 |
| rs4787457 | 16 | 28555400 | A | G | 0.016 | 0.002 | 1.23E-28 | 0.004 | 0.002 | 0.048 |
| rs9927137 | 16 | 30634300 | A | G | 0.008 | 0.001 | 5.11E-09 | 0.004 | 0.002 | 0.057 |
| rs2052285 | 16 | 51188432 | A | G | 0.011 | 0.001 | 7.25E-15 | 0.006 | 0.002 | 0.0065 |
| rs3809634 | 16 | 53538157 | A | G | -0.011 | 0.002 | 6.82E-13 | -0.009 | 0.002 | 0.0001 |
| rs17248751 | 16 | 61579618 | A | G | -0.014 | 0.002 | 1.28E-15 | -0.008 | 0.003 | 0.0017 |
| rs9929556 | 16 | 62193640 | T | G | -0.010 | 0.001 | 2.31E-12 | -0.002 | 0.002 | 0.46 |
| rs17604349 | 16 | 72210865 | A | G | 0.013 | 0.002 | 2.36E-12 | 0.008 | 0.003 | 0.0055 |
| rs9929762 | 16 | 78169675 | A | G | 0.010 | 0.001 | 1.03E-11 | 0.000 | 0.002 | 0.88 |
| rs61527214 | 16 | 83608599 | A | G | 0.011 | 0.001 | 1.13E-14 | 0.005 | 0.002 | 0.025 |
| rs74415461 | 16 | 89990843 | T | C | 0.018 | 0.003 | 6.25E-12 | 0.002 | 0.004 | 0.66 |
| rs2447097 | 17 | 2278064 | T | G | 0.010 | 0.001 | 1.10E-11 | 0.005 | 0.002 | 0.042 |
| rs17732878 | 17 | 7362359 | T | C | -0.010 | 0.002 | 4.06E-08 | -0.004 | 0.003 | 0.11 |
| rs12602286 | 17 | 19236954 | T | G | 0.016 | 0.002 | 4.62E-14 | 0.020 | 0.003 | 3.2E-09 |
| rs11657979 | 17 | 32900837 | A | G | -0.009 | 0.002 | 4.57E-08 | -0.002 | 0.003 | 0.54 |
| rs1842713 | 17 | 33200385 | A | G | -0.012 | 0.002 | 9.67E-12 | -0.009 | 0.003 | 0.00069 |
| rs4793090 | 17 | 40686342 | A | G | 0.008 | 0.002 | 1.67E-08 | -0.001 | 0.002 | 0.58 |
| rs11871429 | 17 | 42920929 | A | G | 0.013 | 0.002 | 2.52E-14 | 0.005 | 0.003 | 0.064 |
| rs56319902 | 17 | 43871982 | T | C | -0.021 | 0.002 | 5.81E-33 | 0.003 | 0.003 | 0.3 |
| rs56794817 | 17 | 60001978 | A | G | 0.012 | 0.002 | 1.69E-10 | 2.59E-06 | 0.003 | 1 |
| rs68145588 | 17 | 75864805 | T | G | -0.013 | 0.002 | 7.53E-10 | -0.001 | 0.003 | 0.75 |
| rs11657342 | 17 | 79355294 | A | G | 0.014 | 0.002 | 2.32E-13 | 0.014 | 0.002 | 9.6E-10 |
| rs12967010 | 18 | 9645035 | T | C | 0.011 | 0.002 | 1.33E-11 | 0.001 | 0.003 | 0.64 |
| rs8097125 | 18 | 13005473 | T | C | 0.009 | 0.001 | 2.31E-09 | 0.005 | 0.002 | 0.022 |
| rs303752 | 18 | 21074255 | A | G | -0.011 | 0.001 | 1.47E-14 | -0.010 | 0.002 | 4.5E-06 |
| rs10460095 | 18 | 22624708 | A | G | -0.012 | 0.001 | 2.61E-18 | -0.002 | 0.002 | 0.41 |
| rs111852224 | 18 | 25596884 | T | C | 0.016 | 0.002 | 4.85E-13 | 0.013 | 0.003 | 0.00014 |
| rs7226824 | 18 | 27676827 | T | C | -0.008 | 0.001 | 2.72E-08 | 0.001 | 0.002 | 0.81 |
| rs11082011 | 18 | 35145122 | T | C | 0.019 | 0.002 | 2.06E-38 | 0.005 | 0.002 | 0.044 |
| rs12957463 | 18 | 37412228 | A | G | 0.015 | 0.002 | 9.20E-17 | 0.014 | 0.003 | 7.7E-07 |
| rs17411339 | 18 | 50807359 | A | G | 0.014 | 0.001 | 5.10E-23 | 0.002 | 0.002 | 0.45 |
| rs2958182 | 18 | 53149021 | A | T | 0.008 | 0.002 | 3.46E-08 | 0.003 | 0.002 | 0.28 |
| rs72944064 | 18 | 63516255 | T | C | -0.010 | 0.002 | 9.39E-10 | -0.007 | 0.003 | 0.0033 |
| rs2554835 | 18 | 74141190 | A | G | 0.009 | 0.001 | 8.95E-10 | 0.005 | 0.002 | 0.015 |
| rs11081529 | 18 | 75902735 | T | C | 0.013 | 0.002 | 5.22E-16 | 0.010 | 0.002 | 0.000036 |
| rs11663602 | 18 | 77578191 | A | C | -0.013 | 0.002 | 9.27E-17 | 0.002 | 0.002 | 0.46 |
| rs75177132 | 19 | 4386911 | T | C | 0.030 | 0.004 | 1.01E-16 | -0.004 | 0.005 | 0.48 |
| rs2287838 | 19 | 9959014 | A | G | -0.010 | 0.001 | 1.27E-13 | -0.005 | 0.002 | 0.025 |
| rs12151248 | 19 | 13212025 | T | C | -0.015 | 0.002 | 3.81E-11 | -0.015 | 0.003 | 0.000022 |
| rs12981405 | 19 | 19651577 | T | C | -0.011 | 0.002 | 2.75E-09 | 0.003 | 0.003 | 0.33 |
| rs7254263 | 19 | 30741257 | T | C | -0.012 | 0.002 | 1.84E-14 | -0.005 | 0.002 | 0.037 |
| rs3859523 | 19 | 32972296 | T | C | 0.012 | 0.002 | 9.92E-12 | 0.008 | 0.003 | 0.0065 |
| rs173003 | 19 | 36240960 | A | C | -0.008 | 0.001 | 5.50E-09 | -0.009 | 0.002 | 0.000018 |
| rs76246107 | 19 | 50121274 | A | G | -0.016 | 0.003 | 6.52E-10 | 0.004 | 0.004 | 0.38 |
| rs192436652 | 19 | 54960747 | T | C | -0.032 | 0.005 | 2.06E-12 | 0.004 | 0.007 | 0.51 |
| rs6043521 | 20 | 15739745 | T | C | -0.008 | 0.001 | 9.72E-09 | 0.009 | 0.002 | 0.000038 |
| rs175325 | 20 | 22317310 | A | T | -0.010 | 0.001 | 2.24E-12 | -0.006 | 0.002 | 0.013 |
| rs4369924 | 20 | 41954383 | A | G | 0.013 | 0.002 | 5.58E-11 | 0.005 | 0.003 | 0.1 |
| rs6065784 | 20 | 43717435 | C | G | 0.011 | 0.002 | 5.78E-13 | 0.001 | 0.002 | 0.76 |
| rs4810894 | 20 | 47532999 | A | G | -0.009 | 0.002 | 4.38E-09 | 0.001 | 0.002 | 0.76 |
| rs6123924 | 20 | 58219764 | A | G | 0.013 | 0.002 | 3.20E-11 | 0.010 | 0.003 | 0.0011 |
| rs6065080 | 20 | 59832791 | T | C | -0.013 | 0.002 | 7.49E-19 | -0.013 | 0.002 | 5.1E-09 |
| rs13050131 | 21 | 20018118 | A | C | -0.008 | 0.002 | 4.40E-08 | -0.007 | 0.002 | 0.0031 |
| rs232496 | 21 | 22734409 | T | C | 0.009 | 0.002 | 7.14E-10 | -0.001 | 0.002 | 0.6 |
| rs1544 | 21 | 33085585 | A | G | -0.010 | 0.002 | 1.76E-09 | 0.005 | 0.002 | 0.05 |
| rs743316 | 21 | 35252696 | T | C | 0.011 | 0.002 | 8.21E-11 | 0.000 | 0.003 | 0.9 |
| rs2838006 | 21 | 42653567 | T | C | 0.013 | 0.002 | 1.38E-19 | 0.001 | 0.002 | 0.61 |
| rs2297293 | 21 | 47125046 | C | G | 0.010 | 0.002 | 8.80E-11 | 0.002 | 0.002 | 0.32 |
| rs737945 | 22 | 30202774 | C | G | -0.012 | 0.001 | 3.73E-16 | 0.000 | 0.002 | 0.85 |
| rs5754753 | 22 | 34296751 | T | C | -0.013 | 0.002 | 6.15E-16 | -0.002 | 0.002 | 0.43 |
| rs143743568 | 22 | 39289014 | A | G | 0.012 | 0.002 | 4.99E-09 | -0.004 | 0.003 | 0.2 |
| rs137079 | 22 | 42977907 | T | C | 0.013 | 0.002 | 3.06E-10 | 0.001 | 0.003 | 0.7 |
| rs9616947 | 22 | 51151631 | T | C | -0.013 | 0.002 | 3.58E-20 | -0.003 | 0.002 | 0.17 |
| 2) MR of leisure computer use on years of education | | | | | | | | | | |
| SNP | Chr | Position | EA | OA | beta_pc | se_pc | pval_pc | beta_ed | se_ed | pval_ed |
| rs2748985 | 1 | 1853184 | T | C | -0.015 | 0.002 | 4.1E-12 | -0.006 | 0.002 | 4.16E-04 |
| rs12145677 | 1 | 110023610 | G | A | -0.017 | 0.002 | 1.2E-12 | -0.012 | 0.002 | 1.32E-11 |
| rs7526456 | 1 | 197773734 | T | C | -0.013 | 0.002 | 1.2E-08 | -0.003 | 0.002 | 8.28E-02 |
| rs10754920 | 2 | 146456888 | C | G | 0.020 | 0.003 | 6.7E-09 | -0.002 | 0.003 | 3.57E-01 |
| rs6744254 | 2 | 155986195 | C | T | -0.016 | 0.002 | 5.4E-13 | -0.001 | 0.002 | 5.06E-01 |
| rs11274218 | 2 | 215035001 | C | T | -0.014 | 0.002 | 2.5E-09 | -0.010 | 0.002 | 2.29E-08 |
| rs4592851 | 2 | 215364232 | G | T | -0.014 | 0.003 | 1.4E-08 | -0.009 | 0.002 | 1.48E-06 |
| rs10208088 | 2 | 221055873 | C | T | 0.013 | 0.002 | 4.4E-09 | 0.003 | 0.002 | 1.13E-01 |
| rs11708955 | 3 | 49540114 | T | C | -0.016 | 0.002 | 4.2E-11 | -0.028 | 0.002 | 3.18E-53 |
| rs6774533 | 3 | 62471086 | C | T | -0.015 | 0.002 | 1.2E-09 | -0.013 | 0.002 | 1.73E-11 |
| rs78082503 | 3 | 116556913 | G | C | -0.015 | 0.003 | 1.1E-08 | -0.006 | 0.002 | 4.59E-03 |
| rs6857629 | 4 | 39684977 | G | A | 0.015 | 0.003 | 1.9E-09 | 0.011 | 0.002 | 6.04E-08 |
| rs2068625 | 4 | 159856739 | T | C | -0.016 | 0.002 | 4E-11 | -0.014 | 0.002 | 3.08E-13 |
| rs2220599 | 5 | 7378854 | C | G | -0.016 | 0.002 | 2.4E-12 | 0.001 | 0.002 | 5.35E-01 |
| rs11749912 | 5 | 88065628 | A | G | 0.014 | 0.002 | 4.9E-10 | 0.018 | 0.002 | 1.84E-25 |
| rs1469249 | 5 | 113837198 | G | A | 0.016 | 0.003 | 9.5E-09 | 0.017 | 0.002 | 1.55E-15 |
| rs162894 | 5 | 131611872 | T | G | 0.013 | 0.002 | 1.1E-08 | 0.005 | 0.002 | 3.26E-03 |
| rs56158102 | 5 | 144027803 | C | T | 0.013 | 0.002 | 3.1E-08 | 0.005 | 0.002 | 4.09E-03 |
| rs9477970 | 6 | 19082663 | T | A | -0.016 | 0.003 | 1E-08 | -0.017 | 0.002 | 3.16E-14 |
| rs2179517 | 6 | 26198845 | G | C | -0.012 | 0.002 | 1.9E-08 | -0.013 | 0.002 | 2.92E-14 |
| rs9372625 | 6 | 98344031 | G | A | -0.018 | 0.002 | 5.7E-16 | -0.024 | 0.002 | 6.76E-42 |
| rs17789218 | 6 | 100600097 | T | C | -0.015 | 0.003 | 4.2E-09 | -0.001 | 0.002 | 6.08E-01 |
| rs6935828 | 6 | 140811367 | C | T | -0.012 | 0.002 | 4.3E-08 | -0.005 | 0.002 | 6.57E-03 |
| rs55772938 | 7 | 70025113 | A | G | -0.015 | 0.002 | 3.9E-10 | -0.007 | 0.002 | 6.27E-05 |
| rs12706626 | 7 | 124531370 | G | A | -0.013 | 0.002 | 1.4E-08 | -0.003 | 0.002 | 8.52E-02 |
| rs3944151 | 7 | 126970005 | A | G | 0.014 | 0.002 | 3.1E-08 | 0.004 | 0.002 | 2.25E-02 |
| rs2345941 | 7 | 133337635 | A | G | 0.015 | 0.002 | 4E-11 | 0.010 | 0.002 | 8.41E-09 |
| rs13262595 | 8 | 143316970 | A | G | -0.016 | 0.002 | 1.2E-12 | -0.012 | 0.002 | 1.85E-11 |
| rs4977839 | 9 | 23355310 | G | A | -0.020 | 0.002 | 4.7E-19 | -0.024 | 0.002 | 2.96E-44 |
| rs113851275 | 9 | 98297220 | G | A | -0.021 | 0.004 | 3.2E-09 | -0.003 | 0.003 | 2.96E-01 |
| rs7020477 | 9 | 116827760 | A | G | 0.014 | 0.002 | 1.2E-08 | 0.005 | 0.002 | 2.23E-02 |
| rs73578186 | 9 | 126334485 | C | T | 0.015 | 0.002 | 2.3E-10 | 0.010 | 0.002 | 1.02E-07 |
| rs2734849 | 11 | 113270160 | A | G | -0.014 | 0.002 | 8.2E-10 | -0.006 | 0.002 | 2.37E-04 |
| rs1448355 | 11 | 131286685 | C | T | -0.015 | 0.002 | 1.3E-10 | -0.010 | 0.002 | 2.47E-09 |
| rs35933007 | 12 | 38929341 | G | A | -0.015 | 0.003 | 9.2E-09 | -0.007 | 0.002 | 1.89E-03 |
| rs1319734 | 12 | 40367517 | A | G | 0.013 | 0.002 | 1.9E-08 | 0.006 | 0.002 | 4.95E-04 |
| rs206965 | 12 | 120856332 | T | C | 0.016 | 0.003 | 9.1E-09 | 0.008 | 0.002 | 9.36E-05 |
| rs12874776 | 13 | 57794345 | C | A | -0.023 | 0.004 | 8E-11 | -0.015 | 0.003 | 1.13E-07 |
| rs198262 | 14 | 57283283 | C | T | 0.030 | 0.005 | 3.9E-08 | 0.020 | 0.004 | 1.49E-06 |
| rs166835 | 15 | 47716037 | C | T | 0.013 | 0.002 | 3.4E-09 | 0.005 | 0.002 | 6.84E-03 |
| rs4702 | 15 | 91426560 | G | A | 0.012 | 0.002 | 3.8E-08 | 0.000 | 0.002 | 9.61E-01 |
| rs6498759 | 16 | 18020295 | T | C | 0.013 | 0.002 | 2.7E-08 | 0.000 | 0.002 | 8.45E-01 |
| rs3730399 | 16 | 67229019 | A | G | 0.025 | 0.004 | 3.3E-08 | 0.004 | 0.003 | 2.51E-01 |
| rs4073003 | 17 | 19148305 | A | G | 0.020 | 0.003 | 1.2E-09 | 0.016 | 0.003 | 4.27E-10 |
| rs7209653 | 17 | 19882084 | T | C | 0.016 | 0.002 | 9.1E-11 | 0.008 | 0.002 | 4.49E-05 |
| rs12603813 | 17 | 43196584 | T | C | 0.014 | 0.003 | 1.3E-08 | 0.006 | 0.002 | 4.02E-03 |
| rs66643547 | 17 | 79375315 | C | T | -0.015 | 0.002 | 3.2E-11 | -0.013 | 0.002 | 2.85E-11 |
| rs784256 | 18 | 53398626 | G | A | 0.019 | 0.003 | 6.2E-11 | 0.015 | 0.002 | 9.15E-12 |
| rs6129084 | 20 | 59828857 | A | T | -0.014 | 0.002 | 9.3E-10 | -0.012 | 0.002 | 3.05E-12 |
| rs9608249 | 22 | 24613952 | G | A | 0.017 | 0.003 | 2.2E-08 | 0.002 | 0.002 | 4.57E-01 |
| rs136553 | 22 | 27255675 | C | T | -0.015 | 0.002 | 2.3E-11 | -0.005 | 0.002 | 2.43E-03 |
| rs7288455 | 22 | 39966547 | A | G | 0.013 | 0.002 | 4E-09 | 0.010 | 0.002 | 1.85E-09 |

| **Table S31. Betas of the bidirectional MR between years of education and leisure television use** | | | | | | | | | | |
| --- | --- | --- | --- | --- | --- | --- | --- | --- | --- | --- |
| **1) MR of years of education on leisure television use** | | | | | | | | | | |
| SNP | Chr | Position | EA | OA | beta_ed | se_ed | pval_ed | beta_tv | se_tv | pval_tv |
| rs34394051 | 1 | 6853091 | A | G | -0.012 | 0.002 | 2.30E-09 | 0.009 | 0.003 | 2.50E-03 |
| rs11121177 | 1 | 8445946 | A | G | 0.015 | 0.002 | 1.44E-16 | -0.009 | 0.003 | 2.00E-03 |
| rs10799615 | 1 | 20541370 | A | G | -0.009 | 0.002 | 4.11E-08 | 0.008 | 0.002 | 2.20E-03 |
| rs590013 | 1 | 29155738 | T | C | 0.010 | 0.002 | 2.47E-11 | -0.004 | 0.002 | 1.10E-01 |
| rs10798888 | 1 | 32199099 | T | G | -0.014 | 0.002 | 5.15E-14 | 0.013 | 0.003 | 4.30E-06 |
| rs2364544 | 1 | 41833162 | A | G | -0.011 | 0.001 | 2.91E-13 | -0.003 | 0.002 | 1.70E-01 |
| rs12076635 | 1 | 44026656 | C | G | 0.021 | 0.002 | 3.33E-34 | -0.009 | 0.003 | 1.10E-03 |
| rs1738050 | 1 | 44707295 | C | G | -0.009 | 0.001 | 1.42E-10 | 0.003 | 0.002 | 1.90E-01 |
| rs11211123 | 1 | 45957609 | A | G | 0.010 | 0.002 | 7.62E-10 | 0.000 | 0.003 | 9.70E-01 |
| rs2764684 | 1 | 58537919 | T | C | 0.014 | 0.002 | 9.99E-15 | -0.006 | 0.003 | 4.70E-02 |
| rs2989476 | 1 | 61059259 | C | G | 0.008 | 0.001 | 3.73E-08 | -0.012 | 0.002 | 1.00E-07 |
| rs4915735 | 1 | 61808444 | A | G | 0.012 | 0.002 | 9.56E-09 | -0.002 | 0.003 | 5.00E-01 |
| rs34305371 | 1 | 72733610 | A | G | 0.031 | 0.002 | 1.10E-39 | -0.016 | 0.004 | 9.20E-06 |
| rs79994730 | 1 | 75562222 | T | C | 0.024 | 0.004 | 5.88E-12 | -0.010 | 0.005 | 4.10E-02 |
| rs1008078 | 1 | 91189731 | T | C | -0.018 | 0.001 | 4.50E-37 | 0.010 | 0.002 | 1.30E-05 |
| rs6690195 | 1 | 96221653 | T | C | -0.011 | 0.001 | 7.89E-16 | 0.009 | 0.002 | 5.50E-05 |
| rs10875121 | 1 | 98417446 | C | G | 0.017 | 0.002 | 4.46E-20 | -0.023 | 0.003 | 1.50E-15 |
| rs575113 | 1 | 110046373 | A | G | 0.013 | 0.002 | 7.52E-18 | -0.015 | 0.002 | 1.30E-10 |
| rs76577427 | 1 | 110761099 | C | G | 0.020 | 0.002 | 7.78E-18 | -0.010 | 0.004 | 6.50E-03 |
| rs4839155 | 1 | 112161489 | T | G | 0.012 | 0.002 | 4.66E-12 | -0.008 | 0.003 | 1.10E-03 |
| rs3026996 | 1 | 159167290 | A | C | 0.014 | 0.002 | 5.42E-17 | -0.002 | 0.003 | 4.10E-01 |
| rs34720381 | 1 | 171455322 | T | C | -0.018 | 0.002 | 1.90E-13 | 0.016 | 0.004 | 2.70E-05 |
| rs6697584 | 1 | 181509718 | T | C | -0.013 | 0.002 | 1.43E-13 | 0.002 | 0.003 | 4.00E-01 |
| rs1747817 | 1 | 197711492 | T | C | -0.009 | 0.002 | 2.29E-08 | 0.002 | 0.003 | 4.80E-01 |
| rs1890132 | 1 | 199427321 | T | C | -0.009 | 0.002 | 6.56E-09 | 0.003 | 0.002 | 2.60E-01 |
| rs8024 | 1 | 201845575 | A | C | -0.012 | 0.002 | 1.13E-14 | 0.010 | 0.002 | 7.30E-06 |
| rs11588857 | 1 | 204587047 | A | G | 0.020 | 0.002 | 4.45E-31 | -0.012 | 0.003 | 1.30E-05 |
| rs3111251 | 1 | 211409844 | T | C | -0.009 | 0.001 | 3.88E-10 | 0.003 | 0.002 | 2.40E-01 |
| rs4846724 | 1 | 221967817 | A | G | 0.010 | 0.001 | 2.89E-12 | -0.006 | 0.002 | 8.30E-03 |
| rs1329125 | 1 | 234740880 | T | C | -0.010 | 0.002 | 8.37E-11 | 0.008 | 0.002 | 6.20E-04 |
| rs114593137 | 1 | 241876565 | A | T | -0.012 | 0.002 | 1.65E-12 | 0.014 | 0.003 | 3.20E-07 |
| rs3897821 | 1 | 243420388 | A | G | 0.015 | 0.002 | 3.58E-25 | -0.007 | 0.002 | 2.00E-03 |
| rs12614263 | 2 | 4123797 | A | G | 0.008 | 0.001 | 3.38E-09 | -0.004 | 0.002 | 8.60E-02 |
| rs7603132 | 2 | 4951548 | A | G | 0.014 | 0.002 | 1.20E-14 | -0.009 | 0.003 | 1.00E-03 |
| rs76076331 | 2 | 10977585 | T | C | 0.021 | 0.002 | 1.71E-23 | -0.015 | 0.003 | 6.60E-06 |
| rs1991585 | 2 | 16647170 | T | C | -0.010 | 0.002 | 6.26E-11 | 0.009 | 0.002 | 1.20E-04 |
| rs4358081 | 2 | 29100642 | A | C | -0.010 | 0.001 | 1.28E-11 | 0.008 | 0.002 | 2.80E-04 |
| rs12468040 | 2 | 44854981 | T | G | 0.014 | 0.001 | 3.12E-21 | -0.005 | 0.002 | 1.30E-02 |
| rs17502934 | 2 | 50994255 | T | G | -0.017 | 0.002 | 2.12E-17 | 0.015 | 0.003 | 7.30E-07 |
| rs3948495 | 2 | 57383133 | T | G | -0.009 | 0.001 | 3.95E-10 | 0.009 | 0.002 | 8.60E-05 |
| rs10189857 | 2 | 60713235 | A | G | 0.016 | 0.001 | 4.69E-29 | -0.020 | 0.002 | 6.20E-21 |
| rs6731373 | 2 | 68503044 | A | G | -0.012 | 0.002 | 1.39E-14 | 0.008 | 0.002 | 6.60E-04 |
| rs112806496 | 2 | 80421805 | C | G | -0.016 | 0.003 | 1.72E-10 | 0.014 | 0.004 | 1.50E-04 |
| rs11894424 | 2 | 80588091 | A | C | 0.021 | 0.003 | 2.14E-10 | -0.002 | 0.005 | 7.50E-01 |
| rs62155350 | 2 | 98932980 | A | G | 0.034 | 0.005 | 1.69E-10 | -0.027 | 0.008 | 3.90E-04 |
| rs13018640 | 2 | 100821545 | T | C | -0.022 | 0.001 | 1.05E-50 | 0.018 | 0.002 | 7.80E-17 |
| rs2570497 | 2 | 104441546 | T | C | -0.012 | 0.002 | 1.27E-16 | 0.014 | 0.002 | 2.70E-10 |
| rs62155873 | 2 | 105969362 | T | C | -0.015 | 0.002 | 1.65E-12 | 0.010 | 0.003 | 1.80E-03 |
| rs13402497 | 2 | 115826318 | A | T | -0.008 | 0.001 | 1.33E-08 | -0.002 | 0.002 | 2.80E-01 |
| rs4848924 | 2 | 124991887 | A | C | -0.012 | 0.002 | 3.56E-14 | 0.004 | 0.002 | 1.40E-01 |
| rs74787922 | 2 | 141598681 | A | C | 0.018 | 0.003 | 8.97E-11 | -0.005 | 0.004 | 2.60E-01 |
| rs77609760 | 2 | 142358950 | A | G | 0.024 | 0.003 | 7.71E-17 | -0.016 | 0.004 | 1.80E-04 |
| rs1320139 | 2 | 144158177 | C | G | -0.015 | 0.001 | 7.62E-26 | 0.005 | 0.002 | 2.60E-02 |
| rs7575637 | 2 | 145796251 | A | G | 0.011 | 0.001 | 3.43E-14 | -0.003 | 0.002 | 1.20E-01 |
| rs13422673 | 2 | 155474355 | T | C | -0.011 | 0.001 | 6.48E-15 | 0.007 | 0.002 | 6.50E-04 |
| rs11678980 | 2 | 162101261 | A | G | -0.017 | 0.001 | 1.60E-31 | 0.015 | 0.002 | 9.10E-12 |
| rs17428076 | 2 | 172851936 | C | G | 0.014 | 0.002 | 6.41E-17 | -0.007 | 0.003 | 5.40E-03 |
| rs10193498 | 2 | 174094345 | A | T | 0.010 | 0.002 | 3.10E-09 | -0.007 | 0.003 | 5.00E-03 |
| rs72917504 | 2 | 175225018 | T | C | -0.018 | 0.003 | 2.91E-09 | 0.007 | 0.005 | 1.10E-01 |
| rs62174974 | 2 | 185053385 | A | G | -0.011 | 0.002 | 2.76E-09 | 0.009 | 0.003 | 9.50E-04 |
| rs62179650 | 2 | 189135884 | A | G | 0.011 | 0.002 | 5.66E-13 | -0.006 | 0.002 | 5.70E-03 |
| rs66721975 | 2 | 191701185 | A | G | -0.009 | 0.002 | 1.51E-08 | 0.014 | 0.002 | 5.20E-09 |
| rs10931821 | 2 | 199495830 | A | T | -0.014 | 0.001 | 8.65E-24 | 0.010 | 0.002 | 1.40E-06 |
| rs4675248 | 2 | 202880230 | A | G | -0.009 | 0.001 | 4.50E-11 | 0.009 | 0.002 | 6.10E-05 |
| rs62182994 | 2 | 212599303 | T | C | 0.014 | 0.002 | 2.55E-19 | -0.011 | 0.002 | 3.30E-06 |
| rs4673840 | 2 | 215363241 | T | C | -0.014 | 0.002 | 5.86E-13 | 0.009 | 0.003 | 2.20E-03 |
| rs1542354 | 2 | 221814085 | A | G | -0.008 | 0.001 | 2.72E-09 | 0.005 | 0.002 | 1.20E-02 |
| rs4500930 | 2 | 228985505 | T | C | -0.011 | 0.002 | 1.67E-13 | 0.007 | 0.002 | 1.20E-03 |
| rs6704768 | 2 | 233592501 | A | G | -0.012 | 0.001 | 1.84E-16 | 0.011 | 0.002 | 1.10E-06 |
| rs4663617 | 2 | 236744626 | A | T | 0.010 | 0.002 | 4.12E-10 | -0.007 | 0.003 | 4.30E-03 |
| rs72993796 | 2 | 240321051 | T | C | 0.013 | 0.002 | 1.12E-08 | -0.005 | 0.003 | 1.10E-01 |
| rs17048855 | 3 | 8258174 | A | G | 0.011 | 0.002 | 3.10E-13 | -0.004 | 0.002 | 6.20E-02 |
| rs382196 | 3 | 9145554 | T | G | -0.008 | 0.001 | 1.47E-08 | 0.004 | 0.002 | 5.60E-02 |
| rs9882532 | 3 | 16865845 | T | C | 0.012 | 0.002 | 2.15E-17 | -0.006 | 0.002 | 4.90E-03 |
| rs13085461 | 3 | 24950387 | C | G | 0.010 | 0.001 | 2.40E-13 | -0.003 | 0.002 | 2.00E-01 |
| rs73055556 | 3 | 28027538 | A | G | 0.011 | 0.002 | 4.95E-08 | -0.005 | 0.003 | 1.10E-01 |
| rs4328757 | 3 | 36938180 | T | C | 0.010 | 0.001 | 2.95E-11 | -0.012 | 0.002 | 1.50E-07 |
| rs9859556 | 3 | 49455986 | T | G | 0.029 | 0.002 | 4.61E-82 | -0.024 | 0.002 | 7.20E-25 |
| rs77719387 | 3 | 49917021 | A | T | -0.040 | 0.006 | 3.78E-12 | 0.043 | 0.009 | 7.40E-07 |
| rs11130380 | 3 | 53758950 | T | G | 0.009 | 0.001 | 4.17E-10 | 0.002 | 0.002 | 4.00E-01 |
| rs12638072 | 3 | 65654480 | A | G | -0.011 | 0.002 | 3.13E-12 | 0.008 | 0.002 | 9.40E-04 |
| rs115000530 | 3 | 69930625 | A | T | -0.025 | 0.003 | 1.09E-15 | 0.011 | 0.005 | 2.40E-02 |
| rs55736314 | 3 | 71586293 | C | G | -0.015 | 0.001 | 4.59E-27 | 0.013 | 0.002 | 1.10E-09 |
| rs66568921 | 3 | 85672018 | T | G | -0.018 | 0.002 | 7.60E-34 | 0.016 | 0.002 | 3.20E-12 |
| rs9853928 | 3 | 103295384 | T | C | -0.013 | 0.002 | 1.59E-13 | 0.005 | 0.003 | 5.60E-02 |
| rs28513882 | 3 | 107296628 | A | G | -0.011 | 0.002 | 2.03E-09 | 0.010 | 0.003 | 5.50E-04 |
| rs2290601 | 3 | 108112760 | T | C | -0.015 | 0.002 | 4.49E-19 | 0.006 | 0.003 | 2.90E-02 |
| rs1717204 | 3 | 118461145 | A | C | -0.012 | 0.002 | 1.20E-10 | 0.002 | 0.003 | 4.30E-01 |
| rs34067381 | 3 | 123594419 | T | G | -0.010 | 0.002 | 9.77E-13 | 0.011 | 0.002 | 2.30E-06 |
| rs9289300 | 3 | 127144988 | T | C | -0.016 | 0.002 | 1.00E-17 | 0.006 | 0.003 | 3.40E-02 |
| rs7650602 | 3 | 141147414 | T | C | -0.009 | 0.001 | 2.77E-10 | 0.006 | 0.002 | 7.40E-03 |
| rs2885198 | 3 | 143636421 | A | G | 0.009 | 0.001 | 4.63E-10 | -0.009 | 0.002 | 1.40E-05 |
| rs12054166 | 3 | 150093445 | C | G | 0.010 | 0.002 | 2.30E-09 | 0.004 | 0.002 | 1.30E-01 |
| rs7617204 | 3 | 160821969 | A | G | -0.010 | 0.001 | 4.33E-12 | 0.005 | 0.002 | 1.10E-02 |
| rs72622559 | 3 | 175672897 | T | C | -0.012 | 0.002 | 5.50E-13 | 0.001 | 0.003 | 6.60E-01 |
| rs77025239 | 3 | 180734185 | A | G | -0.014 | 0.002 | 4.65E-13 | 0.004 | 0.003 | 1.60E-01 |
| rs13327482 | 3 | 196788363 | A | G | -0.012 | 0.002 | 4.47E-11 | 0.005 | 0.003 | 9.60E-02 |
| rs150537577 | 4 | 705629 | A | G | -0.015 | 0.003 | 9.50E-09 | 0.006 | 0.004 | 1.50E-01 |
| rs363096 | 4 | 3180021 | T | C | -0.014 | 0.001 | 5.77E-24 | 0.012 | 0.002 | 1.50E-08 |
| rs13133213 | 4 | 15646794 | A | G | 0.010 | 0.001 | 7.57E-12 | 0.005 | 0.002 | 1.20E-02 |
| rs2011603 | 4 | 18025484 | A | G | -0.009 | 0.002 | 1.12E-08 | 0.007 | 0.002 | 2.60E-03 |
| rs4467547 | 4 | 21945933 | T | G | 0.012 | 0.001 | 4.88E-17 | -0.007 | 0.002 | 2.30E-03 |
| rs10021733 | 4 | 28609476 | T | C | -0.016 | 0.003 | 9.45E-10 | -0.001 | 0.003 | 6.10E-01 |
| rs6812533 | 4 | 39687838 | T | C | 0.011 | 0.002 | 4.85E-11 | -0.007 | 0.003 | 2.70E-03 |
| rs13130765 | 4 | 45163333 | C | G | -0.009 | 0.001 | 1.89E-10 | 0.009 | 0.002 | 1.90E-05 |
| rs2055940 | 4 | 46997913 | A | G | 0.008 | 0.002 | 4.95E-08 | -0.001 | 0.002 | 6.70E-01 |
| rs1505676 | 4 | 62127291 | C | G | 0.008 | 0.002 | 6.67E-09 | -0.008 | 0.002 | 4.20E-04 |
| rs12506221 | 4 | 67899235 | T | G | -0.013 | 0.001 | 4.23E-20 | 0.004 | 0.002 | 8.40E-02 |
| rs2034631 | 4 | 82225529 | T | C | 0.009 | 0.002 | 2.64E-09 | -0.013 | 0.002 | 5.30E-09 |
| rs7692359 | 4 | 83209346 | T | C | -0.011 | 0.002 | 1.40E-10 | 0.002 | 0.003 | 3.40E-01 |
| rs12503522 | 4 | 94543233 | T | C | -0.011 | 0.002 | 3.93E-13 | 0.007 | 0.002 | 3.40E-03 |
| rs7683416 | 4 | 106152984 | T | C | 0.014 | 0.001 | 6.05E-23 | -0.011 | 0.002 | 6.40E-07 |
| rs1955250 | 4 | 118514236 | A | C | -0.014 | 0.003 | 2.26E-08 | 0.013 | 0.004 | 6.20E-04 |
| rs13145650 | 4 | 122963344 | T | C | -0.017 | 0.003 | 8.99E-12 | 0.007 | 0.004 | 6.40E-02 |
| rs10006235 | 4 | 130670108 | T | C | -0.009 | 0.002 | 4.48E-09 | 0.004 | 0.002 | 7.20E-02 |
| rs12643771 | 4 | 140753103 | T | C | 0.013 | 0.002 | 1.32E-17 | -0.009 | 0.002 | 1.30E-04 |
| rs969512 | 4 | 147872742 | A | T | -0.011 | 0.002 | 9.84E-13 | 0.005 | 0.002 | 1.70E-02 |
| rs7672622 | 4 | 157705551 | A | G | -0.009 | 0.002 | 4.72E-08 | 0.011 | 0.003 | 7.40E-06 |
| rs2081652 | 4 | 159862913 | A | T | 0.012 | 0.002 | 1.09E-16 | -0.011 | 0.002 | 6.70E-07 |
| rs12646523 | 4 | 170944698 | T | C | -0.013 | 0.002 | 3.79E-16 | 0.005 | 0.002 | 3.10E-02 |
| rs17598675 | 4 | 176647637 | T | C | -0.012 | 0.001 | 1.37E-16 | 0.002 | 0.002 | 4.90E-01 |
| rs72709560 | 4 | 178540163 | A | G | 0.009 | 0.002 | 2.03E-08 | -0.005 | 0.002 | 1.70E-02 |
| rs1128956 | 4 | 183724005 | T | G | -0.014 | 0.002 | 5.34E-14 | 0.010 | 0.003 | 3.00E-04 |
| rs11724690 | 4 | 186764747 | T | G | 0.009 | 0.002 | 2.08E-09 | -0.006 | 0.002 | 2.10E-02 |
| rs13163845 | 5 | 3264389 | T | C | -0.015 | 0.002 | 4.66E-14 | 0.007 | 0.003 | 2.30E-02 |
| rs162445 | 5 | 7943246 | A | G | 0.015 | 0.003 | 5.72E-09 | -0.007 | 0.004 | 1.00E-01 |
| rs2434672 | 5 | 11471879 | A | C | 0.009 | 0.001 | 1.06E-10 | -0.004 | 0.002 | 4.40E-02 |
| rs17563464 | 5 | 26913774 | A | C | -0.015 | 0.002 | 1.74E-17 | 0.006 | 0.003 | 2.20E-02 |
| rs10940921 | 5 | 30808645 | T | G | 0.011 | 0.001 | 1.31E-14 | -0.009 | 0.002 | 3.40E-05 |
| rs2964199 | 5 | 57532775 | T | C | -0.010 | 0.002 | 4.61E-11 | 0.008 | 0.002 | 3.40E-04 |
| rs6449503 | 5 | 60095272 | A | G | 0.019 | 0.001 | 1.84E-39 | -0.015 | 0.002 | 1.40E-11 |
| rs10805383 | 5 | 63034606 | A | G | -0.010 | 0.001 | 2.22E-13 | 0.014 | 0.002 | 2.60E-11 |
| rs42302 | 5 | 74965554 | A | G | 0.009 | 0.002 | 5.96E-09 | -0.008 | 0.002 | 4.00E-04 |
| rs61104616 | 5 | 88163771 | A | G | -0.017 | 0.001 | 1.56E-34 | 0.007 | 0.002 | 2.60E-03 |
| rs10046069 | 5 | 89115576 | A | G | 0.013 | 0.002 | 4.40E-09 | -0.009 | 0.003 | 1.10E-02 |
| rs12332731 | 5 | 93033082 | A | T | 0.014 | 0.002 | 5.96E-14 | -0.018 | 0.003 | 1.70E-11 |
| rs252991 | 5 | 106767346 | A | G | 0.010 | 0.002 | 6.30E-11 | -0.010 | 0.002 | 1.40E-05 |
| rs17489649 | 5 | 109156184 | A | G | 0.013 | 0.002 | 2.77E-17 | 0.000 | 0.002 | 9.90E-01 |
| rs660001 | 5 | 113866598 | A | G | -0.016 | 0.002 | 1.66E-21 | 0.006 | 0.003 | 2.80E-02 |
| rs62379838 | 5 | 120102028 | T | C | 0.012 | 0.002 | 1.77E-15 | -0.011 | 0.002 | 6.90E-06 |
| rs10060023 | 5 | 124304677 | T | C | 0.011 | 0.002 | 1.88E-13 | -0.014 | 0.002 | 2.90E-09 |
| rs1434630 | 5 | 136557055 | T | G | -0.014 | 0.002 | 4.63E-12 | 0.003 | 0.003 | 2.50E-01 |
| rs12519073 | 5 | 136776762 | T | C | -0.011 | 0.002 | 1.16E-10 | 0.006 | 0.003 | 3.20E-02 |
| rs2964255 | 5 | 152052332 | A | G | 0.009 | 0.002 | 6.34E-09 | -0.012 | 0.002 | 1.40E-07 |
| rs182902112 | 5 | 153304758 | A | C | -0.030 | 0.005 | 1.17E-08 | 0.025 | 0.008 | 1.90E-03 |
| rs42210 | 5 | 166408788 | C | G | -0.010 | 0.002 | 2.74E-10 | 0.014 | 0.002 | 7.30E-09 |
| rs2545795 | 5 | 176887106 | A | C | 0.013 | 0.001 | 1.03E-18 | -0.007 | 0.002 | 6.50E-04 |
| rs12524795 | 6 | 3464074 | T | C | 0.010 | 0.001 | 5.28E-13 | -0.006 | 0.002 | 4.50E-03 |
| rs112603734 | 6 | 6069588 | A | C | -0.013 | 0.002 | 2.72E-09 | 0.000 | 0.003 | 8.60E-01 |
| rs72829857 | 6 | 16966052 | A | G | -0.015 | 0.002 | 1.52E-19 | 0.011 | 0.003 | 1.40E-05 |
| rs72828517 | 6 | 19036035 | T | C | -0.017 | 0.002 | 1.38E-19 | 0.007 | 0.003 | 2.00E-02 |
| rs2179152 | 6 | 26325888 | T | C | -0.013 | 0.002 | 1.68E-19 | 0.010 | 0.002 | 1.20E-05 |
| rs2256965 | 6 | 31555130 | A | G | 0.011 | 0.001 | 2.40E-13 | -0.005 | 0.002 | 3.10E-02 |
| rs57349798 | 6 | 37486052 | A | G | 0.009 | 0.001 | 8.63E-11 | -0.006 | 0.002 | 4.20E-03 |
| rs2436760 | 6 | 40374288 | T | C | 0.023 | 0.004 | 4.52E-08 | -0.020 | 0.006 | 1.70E-03 |
| rs912883 | 6 | 41552040 | T | C | 0.009 | 0.002 | 6.20E-09 | -0.001 | 0.002 | 5.90E-01 |
| rs78648104 | 6 | 50683009 | T | C | -0.014 | 0.003 | 1.70E-08 | 0.005 | 0.004 | 2.30E-01 |
| rs7449561 | 6 | 66215549 | A | G | 0.010 | 0.002 | 9.73E-10 | -0.006 | 0.003 | 1.40E-02 |
| rs13212041 | 6 | 78171124 | T | C | 0.010 | 0.002 | 7.42E-09 | -0.002 | 0.003 | 5.10E-01 |
| rs4352658 | 6 | 88279872 | T | C | -0.019 | 0.003 | 1.71E-13 | 0.015 | 0.004 | 2.20E-04 |
| rs9359939 | 6 | 92133241 | A | C | -0.011 | 0.002 | 1.51E-10 | 0.008 | 0.003 | 1.90E-03 |
| rs9375188 | 6 | 98555272 | T | C | 0.021 | 0.001 | 8.78E-52 | -0.017 | 0.002 | 1.70E-15 |
| rs10080647 | 6 | 114742335 | A | C | 0.012 | 0.002 | 7.05E-10 | -0.006 | 0.003 | 4.10E-02 |
| rs11542663 | 6 | 119215402 | A | C | 0.011 | 0.002 | 2.44E-13 | -0.005 | 0.002 | 2.80E-02 |
| rs13197257 | 6 | 128333682 | T | G | 0.011 | 0.002 | 2.83E-12 | 0.002 | 0.002 | 4.70E-01 |
| rs9492774 | 6 | 131302489 | C | G | 0.008 | 0.002 | 4.33E-08 | -0.004 | 0.002 | 8.10E-02 |
| rs9373363 | 6 | 143150043 | A | G | -0.011 | 0.002 | 5.54E-12 | 0.013 | 0.002 | 2.20E-07 |
| rs4895650 | 6 | 145024122 | T | C | 0.009 | 0.001 | 5.31E-11 | -0.002 | 0.002 | 4.20E-01 |
| rs6557171 | 6 | 152234593 | T | C | -0.016 | 0.002 | 5.48E-25 | 0.005 | 0.002 | 2.10E-02 |
| rs1933264 | 6 | 153391618 | T | C | 0.010 | 0.002 | 5.85E-10 | -0.010 | 0.003 | 5.60E-05 |
| rs6917154 | 6 | 167135156 | T | C | 0.013 | 0.002 | 2.55E-09 | -0.017 | 0.003 | 1.90E-07 |
| rs6924023 | 6 | 170076041 | A | G | 0.011 | 0.002 | 5.17E-11 | -0.009 | 0.003 | 6.60E-04 |
| rs13240401 | 7 | 2194396 | T | C | 0.018 | 0.002 | 2.36E-24 | -0.006 | 0.003 | 3.30E-02 |
| rs4719944 | 7 | 3496032 | T | C | -0.012 | 0.001 | 9.25E-18 | 0.000 | 0.002 | 9.60E-01 |
| rs929511 | 7 | 8003017 | T | C | -0.016 | 0.002 | 3.91E-14 | 0.016 | 0.003 | 5.70E-07 |
| rs118134876 | 7 | 11500372 | T | C | -0.022 | 0.003 | 1.50E-13 | 0.010 | 0.005 | 3.60E-02 |
| rs6977237 | 7 | 14027991 | T | C | 0.008 | 0.001 | 5.59E-09 | 0.001 | 0.002 | 5.40E-01 |
| rs35929923 | 7 | 21400525 | A | G | -0.011 | 0.002 | 1.77E-12 | 0.008 | 0.002 | 1.00E-03 |
| rs79265434 | 7 | 24621381 | A | G | -0.020 | 0.002 | 1.98E-19 | 0.010 | 0.003 | 2.80E-03 |
| rs10951590 | 7 | 39091043 | T | C | -0.011 | 0.002 | 3.62E-13 | 0.004 | 0.002 | 6.40E-02 |
| rs12670376 | 7 | 48823154 | A | G | 0.009 | 0.001 | 5.99E-11 | -0.006 | 0.002 | 3.80E-03 |
| rs17551064 | 7 | 49869771 | A | G | 0.014 | 0.002 | 1.53E-13 | -0.004 | 0.003 | 1.30E-01 |
| rs1220779 | 7 | 54734673 | A | G | -0.008 | 0.001 | 1.58E-08 | 0.003 | 0.002 | 1.20E-01 |
| rs35417702 | 7 | 71739916 | T | C | -0.015 | 0.001 | 2.40E-27 | 0.010 | 0.002 | 9.80E-06 |
| rs1167827 | 7 | 75163169 | A | G | 0.010 | 0.001 | 2.93E-13 | -0.008 | 0.002 | 2.10E-04 |
| rs10215082 | 7 | 92657985 | A | G | -0.015 | 0.001 | 7.03E-26 | 0.010 | 0.002 | 3.20E-06 |
| rs401966 | 7 | 101695817 | C | G | -0.010 | 0.001 | 1.72E-11 | 0.008 | 0.002 | 5.00E-04 |
| rs9655780 | 7 | 104667334 | A | G | -0.015 | 0.002 | 3.70E-16 | 0.010 | 0.003 | 7.80E-04 |
| rs12113634 | 7 | 111996952 | T | C | 0.009 | 0.002 | 2.50E-09 | -0.005 | 0.002 | 2.20E-02 |
| rs113615161 | 7 | 114519339 | T | C | -0.012 | 0.002 | 2.98E-09 | 0.012 | 0.003 | 1.70E-04 |
| rs6969783 | 7 | 117593308 | A | T | -0.010 | 0.001 | 2.87E-12 | 0.006 | 0.002 | 5.10E-03 |
| rs17144467 | 7 | 122098782 | A | C | 0.010 | 0.002 | 8.74E-11 | -0.009 | 0.002 | 6.10E-05 |
| rs113520408 | 7 | 128402782 | A | G | 0.016 | 0.002 | 1.03E-22 | -0.006 | 0.002 | 1.90E-02 |
| rs57352738 | 7 | 133304694 | A | T | -0.015 | 0.002 | 2.51E-18 | 0.002 | 0.003 | 5.60E-01 |
| rs4726070 | 7 | 151328218 | A | G | 0.012 | 0.001 | 1.36E-17 | -0.004 | 0.002 | 6.60E-02 |
| rs73191311 | 8 | 4796094 | A | G | -0.009 | 0.002 | 2.03E-10 | 0.007 | 0.002 | 1.10E-03 |
| rs6994287 | 8 | 9340932 | A | G | 0.010 | 0.001 | 1.11E-12 | -0.001 | 0.002 | 6.30E-01 |
| rs2517086 | 8 | 17042416 | C | G | -0.009 | 0.001 | 8.00E-10 | 0.005 | 0.002 | 1.90E-02 |
| rs13266287 | 8 | 17871598 | A | T | -0.009 | 0.002 | 1.75E-08 | 0.000 | 0.002 | 8.50E-01 |
| rs34807077 | 8 | 28680769 | A | C | 0.013 | 0.002 | 3.29E-11 | -0.006 | 0.003 | 3.10E-02 |
| rs2725370 | 8 | 30852826 | T | C | -0.014 | 0.002 | 4.96E-20 | 0.019 | 0.002 | 3.60E-15 |
| rs2923424 | 8 | 42382222 | A | G | 0.012 | 0.001 | 6.31E-17 | -0.004 | 0.002 | 1.10E-01 |
| rs2929032 | 8 | 56371745 | A | G | -0.008 | 0.001 | 8.40E-09 | 0.002 | 0.002 | 4.30E-01 |
| rs1866823 | 8 | 57436577 | A | G | 0.011 | 0.001 | 3.09E-14 | -0.003 | 0.002 | 1.70E-01 |
| rs56099375 | 8 | 77372988 | T | C | 0.012 | 0.002 | 3.10E-13 | -0.012 | 0.003 | 8.40E-07 |
| rs4320563 | 8 | 87769961 | A | G | 0.011 | 0.001 | 2.24E-14 | -0.007 | 0.002 | 2.10E-03 |
| rs35606437 | 8 | 93327532 | A | G | 0.011 | 0.002 | 3.98E-11 | -0.008 | 0.002 | 1.10E-03 |
| rs7012546 | 8 | 105067737 | T | C | 0.009 | 0.001 | 1.25E-10 | -0.004 | 0.002 | 9.30E-02 |
| rs72672052 | 8 | 115798424 | A | T | -0.010 | 0.002 | 4.87E-08 | 0.009 | 0.003 | 1.50E-03 |
| rs4298514 | 8 | 118932887 | T | C | 0.010 | 0.002 | 1.07E-11 | -0.008 | 0.002 | 1.40E-03 |
| rs13261773 | 8 | 120187090 | C | G | 0.012 | 0.002 | 3.12E-10 | -0.012 | 0.003 | 1.10E-04 |
| rs401526 | 8 | 130929166 | T | C | -0.010 | 0.001 | 1.15E-13 | 0.008 | 0.002 | 4.70E-04 |
| rs746839 | 8 | 142617261 | C | G | 0.014 | 0.002 | 1.19E-20 | -0.009 | 0.002 | 4.90E-05 |
| rs7460106 | 8 | 143534777 | T | C | -0.012 | 0.002 | 2.06E-13 | 0.004 | 0.003 | 1.00E-01 |
| rs7849487 | 9 | 1757274 | T | G | -0.017 | 0.002 | 1.25E-29 | 0.015 | 0.002 | 9.50E-11 |
| rs73648455 | 9 | 14077378 | T | C | -0.018 | 0.003 | 1.99E-12 | 0.008 | 0.004 | 6.00E-02 |
| rs7875078 | 9 | 14494845 | A | C | -0.008 | 0.001 | 2.72E-08 | 0.010 | 0.002 | 2.70E-06 |
| rs7029718 | 9 | 23358495 | A | G | 0.024 | 0.001 | 7.82E-65 | -0.021 | 0.002 | 5.50E-21 |
| rs4977885 | 9 | 23687983 | A | G | -0.008 | 0.001 | 6.78E-09 | 0.005 | 0.002 | 3.50E-02 |
| rs7041702 | 9 | 33053430 | A | G | -0.011 | 0.002 | 4.53E-12 | -0.004 | 0.002 | 1.10E-01 |
| rs11138947 | 9 | 72110562 | T | C | 0.011 | 0.002 | 2.36E-11 | -0.002 | 0.002 | 5.10E-01 |
| rs9886703 | 9 | 82246351 | A | T | -0.014 | 0.002 | 4.46E-13 | 0.012 | 0.003 | 2.40E-05 |
| rs7040995 | 9 | 92226172 | C | G | 0.010 | 0.001 | 5.39E-12 | -0.015 | 0.002 | 6.60E-12 |
| rs10761251 | 9 | 96421546 | A | T | 0.011 | 0.002 | 2.52E-14 | -0.006 | 0.002 | 1.60E-02 |
| rs10979613 | 9 | 111687584 | T | C | -0.012 | 0.002 | 1.25E-15 | 0.009 | 0.002 | 1.50E-04 |
| rs76235882 | 9 | 121096681 | A | G | 0.024 | 0.004 | 4.52E-10 | -0.012 | 0.006 | 5.20E-02 |
| rs12375949 | 9 | 124617900 | T | C | -0.014 | 0.001 | 1.61E-22 | 0.013 | 0.002 | 6.80E-10 |
| rs2039204 | 9 | 130371107 | A | T | 0.008 | 0.001 | 5.23E-09 | 0.002 | 0.002 | 4.10E-01 |
| rs9411331 | 9 | 134883419 | A | C | 0.015 | 0.002 | 7.63E-22 | -0.013 | 0.002 | 1.70E-08 |
| rs73581580 | 9 | 140251458 | A | G | -0.018 | 0.002 | 5.78E-16 | 0.014 | 0.003 | 1.70E-05 |
| rs7894722 | 10 | 9972046 | T | C | 0.009 | 0.001 | 5.41E-09 | 0.004 | 0.002 | 5.90E-02 |
| rs17148998 | 10 | 10909401 | A | G | 0.010 | 0.002 | 6.98E-09 | -0.012 | 0.003 | 1.20E-05 |
| rs1747714 | 10 | 11161316 | T | C | 0.011 | 0.001 | 3.82E-14 | -0.012 | 0.002 | 9.60E-08 |
| rs2183271 | 10 | 21957229 | T | C | 0.008 | 0.002 | 1.91E-08 | -0.017 | 0.002 | 1.10E-14 |
| rs11598765 | 10 | 23953725 | A | G | -0.011 | 0.002 | 1.73E-09 | 0.011 | 0.003 | 1.30E-04 |
| rs806816 | 10 | 32285942 | A | T | 0.010 | 0.002 | 4.80E-10 | -0.004 | 0.003 | 1.50E-01 |
| rs2007655 | 10 | 34586689 | T | G | 0.009 | 0.001 | 8.64E-10 | -0.005 | 0.002 | 2.10E-02 |
| rs11003463 | 10 | 51801793 | T | G | 0.009 | 0.002 | 5.58E-10 | -0.003 | 0.002 | 1.30E-01 |
| rs7924036 | 10 | 65191645 | T | G | 0.013 | 0.001 | 2.55E-21 | -0.009 | 0.002 | 3.40E-05 |
| rs10996167 | 10 | 66813928 | C | G | 0.011 | 0.002 | 7.47E-09 | -0.003 | 0.002 | 1.30E-01 |
| rs7920624 | 10 | 67963186 | A | T | 0.012 | 0.001 | 1.06E-17 | -0.010 | 0.002 | 3.70E-06 |
| rs7910403 | 10 | 87039251 | T | G | 0.012 | 0.002 | 5.08E-12 | -0.009 | 0.003 | 7.50E-04 |
| rs1426619 | 10 | 90091540 | T | C | 0.010 | 0.001 | 1.40E-11 | -0.006 | 0.002 | 3.80E-03 |
| rs17882802 | 10 | 101983413 | A | G | 0.008 | 0.001 | 3.00E-09 | 0.000 | 0.002 | 9.90E-01 |
| rs73344830 | 10 | 103816828 | A | G | 0.017 | 0.001 | 1.00E-32 | -0.014 | 0.002 | 5.30E-11 |
| rs790647 | 10 | 106776484 | A | C | -0.015 | 0.002 | 3.81E-18 | 0.004 | 0.003 | 1.50E-01 |
| rs12571549 | 10 | 111770013 | A | G | 0.015 | 0.002 | 1.45E-13 | -0.008 | 0.003 | 8.70E-03 |
| rs1865955 | 10 | 118837990 | T | C | 0.012 | 0.002 | 4.06E-11 | -0.003 | 0.003 | 2.80E-01 |
| rs4384309 | 10 | 133110596 | A | G | 0.010 | 0.001 | 4.89E-13 | -0.007 | 0.002 | 7.10E-04 |
| rs12761761 | 10 | 133775375 | T | C | 0.015 | 0.002 | 4.79E-20 | -0.013 | 0.002 | 2.30E-07 |
| rs12765185 | 10 | 134977077 | A | T | -0.009 | 0.002 | 2.96E-08 | 0.009 | 0.002 | 3.70E-04 |
| rs4757957 | 11 | 12881398 | C | G | 0.013 | 0.002 | 1.79E-18 | -0.011 | 0.002 | 9.70E-07 |
| rs11023764 | 11 | 15910939 | A | G | 0.009 | 0.002 | 5.32E-10 | -0.004 | 0.002 | 1.00E-01 |
| rs12789313 | 11 | 25058937 | T | C | 0.009 | 0.001 | 6.79E-11 | -0.002 | 0.002 | 3.00E-01 |
| rs11030102 | 11 | 27681596 | C | G | 0.010 | 0.002 | 4.60E-10 | -0.004 | 0.002 | 1.20E-01 |
| rs2199409 | 11 | 39915234 | T | C | 0.010 | 0.002 | 3.14E-09 | -0.009 | 0.003 | 5.10E-04 |
| rs79728014 | 11 | 46062245 | A | G | -0.013 | 0.002 | 2.06E-11 | 0.005 | 0.003 | 9.30E-02 |
| rs75033012 | 11 | 61347033 | C | G | -0.026 | 0.004 | 1.38E-11 | 0.004 | 0.006 | 4.70E-01 |
| rs628993 | 11 | 61539691 | A | G | 0.013 | 0.002 | 3.83E-08 | -0.006 | 0.004 | 1.20E-01 |
| rs76878669 | 11 | 66092567 | C | G | 0.014 | 0.002 | 3.10E-17 | -0.010 | 0.003 | 1.20E-04 |
| rs72962169 | 11 | 72365669 | T | C | -0.017 | 0.002 | 1.26E-19 | 0.014 | 0.003 | 2.00E-06 |
| rs580652 | 11 | 84904742 | T | C | -0.015 | 0.002 | 4.65E-10 | 0.014 | 0.004 | 1.70E-04 |
| rs10830858 | 11 | 91896638 | T | C | 0.009 | 0.001 | 5.31E-11 | -0.008 | 0.002 | 1.40E-04 |
| rs488476 | 11 | 95543715 | C | G | -0.011 | 0.002 | 8.29E-15 | 0.014 | 0.002 | 7.60E-11 |
| rs1301838 | 11 | 99670514 | T | C | -0.010 | 0.002 | 1.29E-10 | 0.005 | 0.002 | 3.70E-02 |
| rs72486027 | 11 | 107167486 | T | C | -0.010 | 0.002 | 1.61E-10 | 0.002 | 0.003 | 5.00E-01 |
| rs2212430 | 11 | 109289538 | T | C | -0.010 | 0.002 | 1.32E-10 | 0.007 | 0.002 | 1.60E-03 |
| rs11213482 | 11 | 110435636 | A | G | -0.011 | 0.002 | 2.68E-08 | 0.010 | 0.003 | 4.20E-04 |
| rs17565975 | 11 | 111586950 | A | G | -0.011 | 0.001 | 5.54E-14 | 0.005 | 0.002 | 2.20E-02 |
| rs10892807 | 11 | 121998254 | T | C | 0.011 | 0.001 | 1.09E-14 | -0.007 | 0.002 | 2.80E-03 |
| rs12574281 | 11 | 131205421 | A | C | -0.008 | 0.002 | 3.04E-08 | 0.006 | 0.002 | 4.40E-03 |
| rs9633970 | 11 | 132502212 | T | C | 0.009 | 0.002 | 2.81E-08 | -0.001 | 0.003 | 8.20E-01 |
| rs12273435 | 11 | 133825855 | A | G | -0.013 | 0.002 | 4.72E-14 | 0.005 | 0.003 | 5.60E-02 |
| rs4766424 | 12 | 1954096 | C | G | 0.014 | 0.002 | 2.47E-11 | -0.001 | 0.003 | 6.50E-01 |
| rs10772644 | 12 | 13417617 | C | G | 0.014 | 0.002 | 1.69E-10 | -0.025 | 0.003 | 1.50E-12 |
| rs1861786 | 12 | 14000467 | A | G | -0.008 | 0.001 | 2.39E-08 | 0.005 | 0.002 | 1.90E-02 |
| rs7974852 | 12 | 14511679 | A | C | 0.012 | 0.001 | 1.14E-16 | -0.003 | 0.002 | 1.90E-01 |
| rs1603460 | 12 | 23092626 | T | G | 0.009 | 0.001 | 2.20E-10 | -0.004 | 0.002 | 1.10E-01 |
| rs117398064 | 12 | 26523628 | C | G | -0.014 | 0.003 | 1.16E-08 | 0.007 | 0.004 | 6.20E-02 |
| rs7972246 | 12 | 27209328 | T | C | 0.011 | 0.002 | 1.01E-13 | -0.006 | 0.002 | 5.20E-03 |
| rs10844179 | 12 | 32469601 | A | G | 0.010 | 0.002 | 5.03E-10 | -0.002 | 0.003 | 3.70E-01 |
| rs80257979 | 12 | 49402562 | T | G | -0.025 | 0.004 | 3.31E-10 | -0.007 | 0.006 | 2.80E-01 |
| rs17110109 | 12 | 54668908 | T | C | -0.009 | 0.001 | 1.47E-10 | 0.002 | 0.002 | 2.70E-01 |
| rs1689510 | 12 | 56396768 | C | G | 0.019 | 0.002 | 1.35E-38 | -0.012 | 0.002 | 1.50E-07 |
| rs3751331 | 12 | 58290278 | A | G | -0.010 | 0.001 | 1.75E-11 | 0.002 | 0.002 | 3.10E-01 |
| rs57661533 | 12 | 78629917 | T | C | -0.011 | 0.002 | 3.24E-08 | 0.003 | 0.003 | 2.90E-01 |
| rs1245829 | 12 | 79648170 | A | T | -0.010 | 0.001 | 3.29E-12 | 0.011 | 0.002 | 7.50E-07 |
| rs717996 | 12 | 84133820 | T | C | -0.012 | 0.001 | 1.89E-17 | 0.007 | 0.002 | 8.60E-04 |
| rs2216144 | 12 | 97666478 | T | C | 0.010 | 0.001 | 2.58E-12 | -0.004 | 0.002 | 7.70E-02 |
| rs2131167 | 12 | 99633189 | A | G | 0.008 | 0.001 | 1.52E-08 | -0.003 | 0.002 | 1.20E-01 |
| rs7977614 | 12 | 110115286 | A | G | -0.013 | 0.002 | 6.84E-17 | 0.008 | 0.002 | 1.50E-03 |
| rs10773208 | 12 | 122970156 | T | C | -0.011 | 0.002 | 8.09E-12 | 0.006 | 0.002 | 2.40E-02 |
| rs10773002 | 12 | 123746961 | A | T | 0.019 | 0.002 | 2.50E-30 | -0.013 | 0.003 | 9.40E-08 |
| rs6490618 | 13 | 21338343 | T | C | 0.009 | 0.002 | 3.48E-09 | -0.005 | 0.002 | 3.40E-02 |
| rs9529146 | 13 | 31785404 | T | C | 0.013 | 0.002 | 3.48E-14 | -0.006 | 0.003 | 2.00E-02 |
| rs936496 | 13 | 36100541 | A | G | -0.008 | 0.001 | 4.38E-08 | 0.006 | 0.002 | 3.90E-03 |
| rs12875339 | 13 | 55722033 | A | C | -0.011 | 0.002 | 1.71E-13 | 0.007 | 0.002 | 2.20E-03 |
| rs1334297 | 13 | 58335375 | A | G | 0.026 | 0.002 | 2.53E-58 | -0.011 | 0.002 | 3.70E-06 |
| rs7321274 | 13 | 69146186 | A | G | 0.011 | 0.002 | 8.21E-11 | -0.005 | 0.003 | 6.20E-02 |
| rs2478208 | 13 | 81471113 | C | G | -0.010 | 0.001 | 2.89E-13 | 0.009 | 0.002 | 6.10E-05 |
| rs7326331 | 13 | 91615838 | A | G | -0.013 | 0.002 | 3.42E-16 | 0.009 | 0.002 | 1.00E-04 |
| rs11620355 | 13 | 92044587 | A | G | 0.018 | 0.003 | 1.09E-13 | -0.012 | 0.004 | 2.70E-03 |
| rs9556958 | 13 | 99100046 | T | C | -0.011 | 0.001 | 5.96E-15 | 0.004 | 0.002 | 6.50E-02 |
| rs11157931 | 14 | 23403193 | A | C | -0.014 | 0.001 | 3.86E-22 | 0.010 | 0.002 | 1.10E-05 |
| rs178183 | 14 | 26979412 | T | C | 0.015 | 0.002 | 4.81E-19 | -0.015 | 0.003 | 6.20E-09 |
| rs176218 | 14 | 29600506 | T | G | 0.020 | 0.002 | 1.10E-29 | -0.012 | 0.003 | 1.10E-05 |
| rs61997667 | 14 | 57280046 | T | C | -0.014 | 0.002 | 2.24E-13 | 0.014 | 0.003 | 1.60E-06 |
| rs4899012 | 14 | 61003889 | C | G | -0.010 | 0.001 | 8.06E-13 | 0.004 | 0.002 | 9.60E-02 |
| rs12888615 | 14 | 72435705 | T | C | 0.012 | 0.002 | 9.42E-12 | -0.012 | 0.003 | 8.00E-06 |
| rs730384 | 14 | 74889870 | A | G | 0.010 | 0.001 | 9.38E-13 | -0.005 | 0.002 | 1.20E-02 |
| rs2998299 | 14 | 84997363 | T | G | -0.014 | 0.002 | 1.51E-15 | 0.007 | 0.003 | 1.10E-02 |
| rs4904523 | 14 | 89723630 | A | G | -0.008 | 0.001 | 6.27E-09 | 0.004 | 0.002 | 4.80E-02 |
| rs736281 | 14 | 94287830 | T | C | 0.009 | 0.001 | 2.49E-09 | -0.015 | 0.002 | 3.80E-11 |
| rs2496482 | 14 | 99750164 | T | C | 0.012 | 0.002 | 9.20E-17 | -0.008 | 0.002 | 2.70E-04 |
| rs8008382 | 14 | 104054425 | T | C | -0.011 | 0.002 | 4.07E-12 | 0.011 | 0.002 | 1.30E-06 |
| rs7167688 | 15 | 26813137 | T | C | -0.009 | 0.001 | 1.71E-11 | 0.005 | 0.002 | 2.10E-02 |
| rs891793 | 15 | 27256021 | C | G | -0.010 | 0.001 | 1.87E-12 | 0.005 | 0.002 | 1.90E-02 |
| rs6493265 | 15 | 47513253 | T | C | -0.012 | 0.001 | 2.26E-17 | 0.001 | 0.002 | 5.20E-01 |
| rs12438177 | 15 | 55951432 | A | G | 0.009 | 0.002 | 1.48E-10 | -0.009 | 0.002 | 3.40E-05 |
| rs7171405 | 15 | 61466893 | A | G | -0.010 | 0.002 | 7.53E-10 | 0.014 | 0.003 | 6.20E-08 |
| rs12591647 | 15 | 65959729 | T | C | -0.015 | 0.002 | 7.78E-17 | 0.003 | 0.003 | 3.40E-01 |
| rs1564347 | 15 | 73438848 | T | G | 0.009 | 0.002 | 2.39E-09 | -0.010 | 0.002 | 7.10E-06 |
| rs2469226 | 15 | 77246990 | A | T | 0.010 | 0.002 | 1.28E-08 | -0.006 | 0.003 | 2.90E-02 |
| rs56391344 | 15 | 78006899 | A | G | 0.015 | 0.002 | 7.49E-20 | -0.016 | 0.002 | 3.60E-10 |
| rs4778058 | 15 | 93456069 | T | C | -0.009 | 0.001 | 4.92E-11 | 0.010 | 0.002 | 2.00E-06 |
| rs4984541 | 15 | 96911139 | A | G | -0.014 | 0.002 | 3.40E-16 | 0.006 | 0.003 | 3.00E-02 |
| rs8030487 | 15 | 97119595 | A | G | 0.009 | 0.002 | 2.52E-08 | -0.007 | 0.002 | 5.30E-03 |
| rs9933256 | 16 | 1246748 | A | G | 0.012 | 0.001 | 6.06E-17 | -0.006 | 0.002 | 2.90E-03 |
| rs11076962 | 16 | 5811367 | T | C | 0.009 | 0.002 | 4.29E-08 | -0.007 | 0.002 | 5.60E-03 |
| rs34098770 | 16 | 12237812 | A | G | -0.016 | 0.002 | 2.95E-16 | 0.007 | 0.003 | 1.40E-02 |
| rs9927842 | 16 | 15153717 | T | C | -0.013 | 0.002 | 6.22E-11 | 0.002 | 0.003 | 4.50E-01 |
| rs4787457 | 16 | 28555400 | A | G | 0.016 | 0.002 | 1.23E-28 | -0.013 | 0.002 | 2.80E-09 |
| rs9927137 | 16 | 30634300 | A | G | 0.008 | 0.001 | 5.11E-09 | -0.008 | 0.002 | 4.10E-04 |
| rs2052285 | 16 | 51188432 | A | G | 0.011 | 0.001 | 7.25E-15 | -0.008 | 0.002 | 3.50E-04 |
| rs3809634 | 16 | 53538157 | A | G | -0.011 | 0.002 | 6.82E-13 | 0.015 | 0.002 | 8.70E-11 |
| rs17248751 | 16 | 61579618 | A | G | -0.014 | 0.002 | 1.28E-15 | 0.006 | 0.003 | 1.30E-02 |
| rs9929556 | 16 | 62193640 | T | G | -0.010 | 0.001 | 2.31E-12 | 0.004 | 0.002 | 6.90E-02 |
| rs17604349 | 16 | 72210865 | A | G | 0.013 | 0.002 | 2.36E-12 | -0.013 | 0.003 | 5.10E-06 |
| rs9929762 | 16 | 78169675 | A | G | 0.010 | 0.001 | 1.03E-11 | -0.008 | 0.002 | 5.50E-04 |
| rs61527214 | 16 | 83608599 | A | G | 0.011 | 0.001 | 1.13E-14 | -0.007 | 0.002 | 3.10E-03 |
| rs74415461 | 16 | 89990843 | T | C | 0.018 | 0.003 | 6.25E-12 | -0.015 | 0.004 | 1.20E-04 |
| rs2447097 | 17 | 2278064 | T | G | 0.010 | 0.001 | 1.10E-11 | -0.015 | 0.002 | 2.50E-11 |
| rs17732878 | 17 | 7362359 | T | C | -0.010 | 0.002 | 4.06E-08 | 0.007 | 0.003 | 6.40E-03 |
| rs12602286 | 17 | 19236954 | T | G | 0.016 | 0.002 | 4.62E-14 | -0.001 | 0.003 | 8.60E-01 |
| rs11657979 | 17 | 32900837 | A | G | -0.009 | 0.002 | 4.57E-08 | 0.009 | 0.003 | 6.90E-04 |
| rs1842713 | 17 | 33200385 | A | G | -0.012 | 0.002 | 9.67E-12 | 0.010 | 0.003 | 3.00E-04 |
| rs4793090 | 17 | 40686342 | A | G | 0.008 | 0.002 | 1.67E-08 | -0.010 | 0.002 | 6.00E-06 |
| rs11871429 | 17 | 42920929 | A | G | 0.013 | 0.002 | 2.52E-14 | -0.006 | 0.003 | 1.30E-02 |
| rs56319902 | 17 | 43871982 | T | C | -0.021 | 0.002 | 5.81E-33 | 0.005 | 0.003 | 7.50E-02 |
| rs56794817 | 17 | 60001978 | A | G | 0.012 | 0.002 | 1.69E-10 | -0.005 | 0.003 | 9.90E-02 |
| rs68145588 | 17 | 75864805 | T | G | -0.013 | 0.002 | 7.53E-10 | -0.001 | 0.003 | 8.10E-01 |
| rs11657342 | 17 | 79355294 | A | G | 0.014 | 0.002 | 2.32E-13 | -0.012 | 0.002 | 7.10E-08 |
| rs12967010 | 18 | 9645035 | T | C | 0.011 | 0.002 | 1.33E-11 | -0.005 | 0.003 | 6.00E-02 |
| rs8097125 | 18 | 13005473 | T | C | 0.009 | 0.001 | 2.31E-09 | -0.005 | 0.002 | 1.40E-02 |
| rs303752 | 18 | 21074255 | A | G | -0.011 | 0.001 | 1.47E-14 | 0.013 | 0.002 | 1.20E-08 |
| rs10460095 | 18 | 22624708 | A | G | -0.012 | 0.001 | 2.61E-18 | -0.005 | 0.002 | 2.50E-02 |
| rs111852224 | 18 | 25596884 | T | C | 0.016 | 0.002 | 4.85E-13 | -0.006 | 0.003 | 5.30E-02 |
| rs7226824 | 18 | 27676827 | T | C | -0.008 | 0.001 | 2.72E-08 | 0.005 | 0.002 | 1.60E-02 |
| rs11082011 | 18 | 35145122 | T | C | 0.019 | 0.002 | 2.06E-38 | -0.017 | 0.002 | 1.60E-13 |
| rs12957463 | 18 | 37412228 | A | G | 0.015 | 0.002 | 9.20E-17 | -0.009 | 0.003 | 9.80E-04 |
| rs17411339 | 18 | 50807359 | A | G | 0.014 | 0.001 | 5.10E-23 | -0.006 | 0.002 | 7.90E-03 |
| rs2958182 | 18 | 53149021 | A | T | 0.008 | 0.002 | 3.46E-08 | -0.001 | 0.002 | 6.80E-01 |
| rs72944064 | 18 | 63516255 | T | C | -0.010 | 0.002 | 9.39E-10 | 0.010 | 0.002 | 8.70E-05 |
| rs2554835 | 18 | 74141190 | A | G | 0.009 | 0.001 | 8.95E-10 | -0.003 | 0.002 | 2.40E-01 |
| rs11081529 | 18 | 75902735 | T | C | 0.013 | 0.002 | 5.22E-16 | -0.004 | 0.002 | 1.10E-01 |
| rs11663602 | 18 | 77578191 | A | C | -0.013 | 0.002 | 9.27E-17 | 0.007 | 0.002 | 2.70E-03 |
| rs75177132 | 19 | 4386911 | T | C | 0.030 | 0.004 | 1.01E-16 | -0.021 | 0.005 | 3.80E-05 |
| rs2287838 | 19 | 9959014 | A | G | -0.010 | 0.001 | 1.27E-13 | 0.008 | 0.002 | 3.10E-04 |
| rs12151248 | 19 | 13212025 | T | C | -0.015 | 0.002 | 3.81E-11 | 0.003 | 0.003 | 3.20E-01 |
| rs12981405 | 19 | 19651577 | T | C | -0.011 | 0.002 | 2.75E-09 | 0.015 | 0.003 | 3.70E-07 |
| rs7254263 | 19 | 30741257 | T | C | -0.012 | 0.002 | 1.84E-14 | 0.004 | 0.002 | 6.80E-02 |
| rs3859523 | 19 | 32972296 | T | C | 0.012 | 0.002 | 9.92E-12 | -0.006 | 0.003 | 2.40E-02 |
| rs173003 | 19 | 36240960 | A | C | -0.008 | 0.001 | 5.50E-09 | 0.007 | 0.002 | 2.70E-03 |
| rs76246107 | 19 | 50121274 | A | G | -0.016 | 0.003 | 6.52E-10 | 0.022 | 0.004 | 7.00E-08 |
| rs192436652 | 19 | 54960747 | T | C | -0.032 | 0.005 | 2.06E-12 | 0.026 | 0.007 | 7.50E-05 |
| rs6043521 | 20 | 15739745 | T | C | -0.008 | 0.001 | 9.72E-09 | 0.001 | 0.002 | 6.60E-01 |
| rs175325 | 20 | 22317310 | A | T | -0.010 | 0.001 | 2.24E-12 | 0.005 | 0.002 | 3.70E-02 |
| rs4369924 | 20 | 41954383 | A | G | 0.013 | 0.002 | 5.58E-11 | -0.012 | 0.003 | 8.60E-05 |
| rs6065784 | 20 | 43717435 | C | G | 0.011 | 0.002 | 5.78E-13 | -0.014 | 0.002 | 2.60E-09 |
| rs4810894 | 20 | 47532999 | A | G | -0.009 | 0.002 | 4.38E-09 | 0.008 | 0.002 | 5.70E-04 |
| rs6123924 | 20 | 58219764 | A | G | 0.013 | 0.002 | 3.20E-11 | -0.010 | 0.003 | 8.20E-04 |
| rs6065080 | 20 | 59832791 | T | C | -0.013 | 0.002 | 7.49E-19 | 0.004 | 0.002 | 5.80E-02 |
| rs13050131 | 21 | 20018118 | A | C | -0.008 | 0.002 | 4.40E-08 | 0.006 | 0.002 | 1.40E-02 |
| rs232496 | 21 | 22734409 | T | C | 0.009 | 0.002 | 7.14E-10 | -0.003 | 0.002 | 2.00E-01 |
| rs1544 | 21 | 33085585 | A | G | -0.010 | 0.002 | 1.76E-09 | 0.007 | 0.002 | 4.90E-03 |
| rs743316 | 21 | 35252696 | T | C | 0.011 | 0.002 | 8.21E-11 | -0.006 | 0.003 | 1.80E-02 |
| rs2838006 | 21 | 42653567 | T | C | 0.013 | 0.002 | 1.38E-19 | -0.006 | 0.002 | 5.10E-03 |
| rs2297293 | 21 | 47125046 | C | G | 0.010 | 0.002 | 8.80E-11 | -0.004 | 0.002 | 8.20E-02 |
| rs737945 | 22 | 30202774 | C | G | -0.012 | 0.001 | 3.73E-16 | 0.011 | 0.002 | 2.90E-07 |
| rs5754753 | 22 | 34296751 | T | C | -0.013 | 0.002 | 6.15E-16 | 0.009 | 0.002 | 1.40E-04 |
| rs143743568 | 22 | 39289014 | A | G | 0.012 | 0.002 | 4.99E-09 | -0.008 | 0.003 | 9.40E-03 |
| rs137079 | 22 | 42977907 | T | C | 0.013 | 0.002 | 3.06E-10 | -0.016 | 0.003 | 1.90E-07 |
| rs9616947 | 22 | 51151631 | T | C | -0.013 | 0.002 | 3.58E-20 | 0.004 | 0.002 | 6.60E-02 |
| **2) MR of leisure television use on years of education** | | | | | | | | | | |
| SNP | Chr | Position | EA | OA | beta_tv | se_tv | pval_tv | beta_ed | se_ed | pval_ed |
| rs11810109 | 1 | 44186812 | A | T | 0.016 | 0.002 | 5.40E-12 | -0.019 | 0.002 | 1.16E-23 |
| rs112632642 | 1 | 51115634 | G | A | -0.024 | 0.004 | 3.60E-08 | 0.008 | 0.003 | 1.76E-02 |
| rs6670915 | 1 | 61109968 | C | T | 0.014 | 0.002 | 2.40E-10 | -0.006 | 0.002 | 1.96E-04 |
| rs12725114 | 1 | 62432641 | G | A | 0.015 | 0.003 | 1.60E-08 | -0.004 | 0.002 | 6.55E-02 |
| rs984409 | 1 | 67020440 | G | A | -0.015 | 0.002 | 4.10E-11 | 0.005 | 0.002 | 3.60E-03 |
| rs71658797 | 1 | 77967507 | T | A | -0.020 | 0.003 | 6.30E-10 | 0.010 | 0.003 | 1.02E-04 |
| rs114328297 | 1 | 91190854 | T | G | 0.014 | 0.003 | 4.40E-08 | -0.011 | 0.002 | 3.52E-07 |
| rs631130 | 1 | 96043010 | T | A | -0.014 | 0.002 | 5.00E-09 | 0.005 | 0.002 | 3.52E-03 |
| rs148648822 | 1 | 97815048 | G | T | -0.018 | 0.003 | 4.20E-09 | 0.008 | 0.002 | 1.24E-03 |
| rs17379561 | 1 | 98340139 | A | T | -0.025 | 0.003 | 1.10E-16 | 0.019 | 0.002 | 1.73E-15 |
| rs55909997 | 1 | 107581039 | G | A | -0.014 | 0.002 | 2.40E-09 | 0.006 | 0.002 | 6.29E-04 |
| rs1278847 | 1 | 110037483 | C | A | 0.016 | 0.002 | 1.20E-11 | -0.012 | 0.002 | 7.79E-11 |
| rs141425667 | 1 | 114930465 | G | A | -0.024 | 0.004 | 4.00E-08 | 0.010 | 0.004 | 3.12E-03 |
| rs4845364 | 1 | 154141908 | A | G | -0.015 | 0.002 | 1.30E-12 | 0.002 | 0.002 | 2.84E-01 |
| rs6673341 | 1 | 184655649 | T | G | -0.015 | 0.002 | 2.20E-11 | 0.007 | 0.002 | 1.51E-05 |
| rs72725224 | 1 | 188115858 | A | C | -0.046 | 0.008 | 3.70E-09 | 0.001 | 0.006 | 9.15E-01 |
| rs10737620 | 1 | 193057971 | T | A | 0.014 | 0.002 | 2.60E-09 | -0.007 | 0.002 | 3.12E-04 |
| rs11896615 | 2 | 17407744 | G | A | 0.017 | 0.003 | 3.10E-08 | -0.006 | 0.002 | 1.60E-02 |
| rs72781699 | 2 | 24277709 | G | A | -0.019 | 0.003 | 3.00E-12 | 0.006 | 0.002 | 2.99E-03 |
| rs12105701 | 2 | 41737200 | C | T | -0.013 | 0.002 | 5.40E-09 | 0.005 | 0.002 | 2.45E-03 |
| rs7564130 | 2 | 50606642 | T | C | -0.015 | 0.002 | 2.80E-11 | 0.009 | 0.002 | 1.49E-06 |
| rs11125515 | 2 | 53886799 | C | A | -0.014 | 0.002 | 2.20E-08 | 0.001 | 0.002 | 4.83E-01 |
| rs12475168 | 2 | 58992704 | G | T | -0.018 | 0.003 | 1.70E-08 | 0.006 | 0.002 | 2.12E-02 |
| rs183566425 | 2 | 59325356 | A | T | 0.046 | 0.008 | 1.90E-08 | -0.022 | 0.007 | 2.07E-03 |
| rs10189857 | 2 | 60713235 | A | G | -0.020 | 0.002 | 6.20E-21 | 0.017 | 0.002 | 6.70E-24 |
| rs62641636 | 2 | 68425427 | A | G | 0.014 | 0.002 | 7.00E-10 | -0.011 | 0.002 | 1.06E-09 |
| rs59210467 | 2 | 97097291 | C | T | -0.013 | 0.002 | 1.00E-08 | 0.005 | 0.002 | 9.69E-03 |
| rs11689199 | 2 | 100820421 | A | G | 0.018 | 0.002 | 5.50E-17 | -0.020 | 0.002 | 1.17E-29 |
| rs2916251 | 2 | 104447052 | G | T | -0.017 | 0.002 | 1.50E-13 | 0.010 | 0.002 | 8.79E-10 |
| rs1451533 | 2 | 105466005 | G | A | -0.016 | 0.002 | 1.10E-10 | 0.000 | 0.002 | 8.43E-01 |
| rs2971640 | 2 | 107624618 | G | A | -0.013 | 0.002 | 4.20E-09 | 0.008 | 0.002 | 3.04E-06 |
| rs12476388 | 2 | 116330983 | C | T | 0.013 | 0.002 | 2.10E-08 | 0.000 | 0.002 | 8.32E-01 |
| rs374722 | 2 | 147839830 | G | A | 0.024 | 0.003 | 5.50E-16 | -0.006 | 0.002 | 8.51E-03 |
| rs3754970 | 2 | 162091836 | T | C | -0.015 | 0.002 | 4.80E-12 | 0.016 | 0.002 | 3.53E-20 |
| rs1913808 | 2 | 162804678 | G | A | 0.015 | 0.002 | 3.90E-10 | -0.013 | 0.002 | 5.63E-12 |
| rs2060199 | 2 | 166196681 | T | A | 0.012 | 0.002 | 2.40E-08 | -0.003 | 0.002 | 6.82E-02 |
| rs263771 | 2 | 185921692 | C | A | -0.017 | 0.003 | 3.10E-11 | 0.010 | 0.002 | 4.10E-06 |
| rs4577309 | 2 | 191288833 | A | G | 0.016 | 0.002 | 1.60E-13 | -0.005 | 0.002 | 3.33E-03 |
| rs34224261 | 2 | 193855452 | G | C | -0.018 | 0.002 | 4.60E-16 | 0.011 | 0.002 | 3.79E-11 |
| rs4675246 | 2 | 202864487 | G | T | -0.015 | 0.003 | 2.90E-08 | 0.009 | 0.002 | 5.48E-05 |
| rs13029509 | 2 | 215374209 | G | A | -0.018 | 0.002 | 2.00E-17 | 0.010 | 0.002 | 7.17E-10 |
| rs10932837 | 2 | 221044785 | C | T | -0.013 | 0.002 | 1.30E-09 | 0.002 | 0.002 | 1.52E-01 |
| rs4973576 | 2 | 233806771 | C | A | -0.015 | 0.002 | 1.00E-09 | 0.008 | 0.002 | 7.60E-06 |
| rs138256022 | 3 | 11603090 | C | T | -0.031 | 0.006 | 2.90E-08 | 0.011 | 0.004 | 1.06E-02 |
| rs9834970 | 3 | 36856030 | T | C | 0.013 | 0.002 | 3.30E-09 | -0.008 | 0.002 | 7.65E-07 |
| rs1352890 | 3 | 49652156 | C | T | -0.012 | 0.002 | 1.30E-08 | 0.012 | 0.002 | 1.71E-12 |
| rs3796386 | 3 | 49899795 | G | A | -0.026 | 0.002 | 3.20E-33 | 0.026 | 0.002 | 7.19E-51 |
| rs182355396 | 3 | 50920457 | C | A | -0.080 | 0.013 | 2.10E-10 | 0.051 | 0.010 | 6.45E-07 |
| rs11917871 | 3 | 54233476 | T | C | -0.017 | 0.002 | 4.50E-14 | 0.007 | 0.002 | 8.86E-05 |
| rs11130793 | 3 | 60884659 | C | T | 0.013 | 0.002 | 5.00E-09 | -0.005 | 0.002 | 7.56E-03 |
| rs11714337 | 3 | 71582521 | G | A | 0.014 | 0.002 | 4.70E-11 | -0.013 | 0.002 | 7.66E-15 |
| rs12496084 | 3 | 75174777 | A | G | -0.014 | 0.003 | 4.60E-08 | 0.005 | 0.002 | 1.48E-02 |
| rs6797840 | 3 | 85656569 | A | C | -0.016 | 0.002 | 1.70E-13 | 0.010 | 0.002 | 1.29E-08 |
| rs56398417 | 3 | 88024986 | C | T | 0.014 | 0.002 | 5.90E-10 | -0.005 | 0.002 | 3.07E-03 |
| rs2034768 | 3 | 93986371 | A | G | 0.015 | 0.002 | 8.60E-12 | -0.006 | 0.002 | 2.08E-04 |
| rs12632778 | 3 | 106452359 | T | G | -0.028 | 0.005 | 3.90E-08 | 0.004 | 0.004 | 3.39E-01 |
| rs9867121 | 3 | 114631548 | C | A | 0.019 | 0.003 | 3.90E-12 | -0.004 | 0.002 | 9.97E-02 |
| rs12491503 | 3 | 165706855 | G | A | -0.014 | 0.002 | 5.50E-10 | 0.007 | 0.002 | 1.41E-04 |
| rs114600294 | 3 | 181419367 | G | C | -0.016 | 0.003 | 7.90E-10 | 0.011 | 0.002 | 3.21E-06 |
| rs66852340 | 4 | 3311070 | C | T | -0.018 | 0.003 | 7.90E-12 | 0.013 | 0.002 | 1.94E-10 |
| rs7700107 | 4 | 17880416 | A | C | -0.021 | 0.003 | 1.90E-11 | 0.007 | 0.003 | 5.27E-03 |
| rs34811474 | 4 | 25408838 | G | A | 0.015 | 0.003 | 2.30E-09 | -0.009 | 0.002 | 4.89E-05 |
| rs77215114 | 4 | 45187658 | A | T | 0.024 | 0.004 | 1.50E-08 | -0.008 | 0.003 | 1.74E-02 |
| rs6850494 | 4 | 82291771 | A | C | -0.014 | 0.002 | 1.10E-10 | 0.005 | 0.002 | 8.27E-03 |
| rs13107325 | 4 | 103188709 | C | T | -0.029 | 0.004 | 1.50E-12 | 0.019 | 0.003 | 4.45E-08 |
| rs62332760 | 4 | 106135316 | G | T | -0.014 | 0.002 | 8.00E-11 | 0.013 | 0.002 | 1.42E-14 |
| rs7693703 | 4 | 118357405 | G | A | 0.023 | 0.004 | 2.70E-09 | -0.008 | 0.003 | 5.22E-03 |
| rs4334769 | 4 | 130245233 | G | T | 0.012 | 0.002 | 2.10E-08 | 0.000 | 0.002 | 8.58E-01 |
| rs6825241 | 4 | 152543783 | C | A | -0.017 | 0.002 | 4.90E-15 | 0.009 | 0.002 | 2.82E-07 |
| rs7693082 | 4 | 159857819 | G | C | 0.015 | 0.002 | 1.70E-10 | -0.013 | 0.002 | 4.05E-13 |
| rs10940659 | 5 | 59360341 | A | G | -0.013 | 0.002 | 1.50E-08 | 0.009 | 0.002 | 9.56E-08 |
| rs2409838 | 5 | 60204410 | A | T | 0.017 | 0.002 | 3.40E-15 | -0.019 | 0.002 | 2.14E-28 |
| rs262890 | 5 | 62930015 | A | G | -0.019 | 0.002 | 3.20E-15 | 0.010 | 0.002 | 8.30E-08 |
| rs7716447 | 5 | 88800331 | A | G | -0.013 | 0.002 | 3.60E-09 | 0.006 | 0.002 | 2.78E-04 |
| rs1031423 | 5 | 93276883 | T | C | -0.019 | 0.003 | 1.80E-12 | 0.012 | 0.002 | 4.11E-09 |
| rs57585211 | 5 | 107197725 | T | G | -0.017 | 0.003 | 4.30E-09 | 0.010 | 0.002 | 7.87E-06 |
| rs9326862 | 5 | 112054547 | A | T | 0.012 | 0.002 | 2.60E-08 | -0.003 | 0.002 | 1.36E-01 |
| rs10041724 | 5 | 124273520 | T | C | 0.018 | 0.003 | 3.90E-11 | -0.013 | 0.002 | 1.58E-09 |
| rs10057545 | 5 | 138431008 | G | A | -0.014 | 0.002 | 2.00E-10 | 0.001 | 0.002 | 7.15E-01 |
| rs62379379 | 5 | 141082015 | G | T | -0.026 | 0.004 | 7.80E-10 | 0.015 | 0.003 | 1.86E-06 |
| rs10054327 | 5 | 147871137 | G | A | 0.017 | 0.002 | 3.40E-15 | -0.004 | 0.002 | 3.91E-02 |
| rs42210 | 5 | 166408788 | G | C | -0.014 | 0.002 | 7.30E-09 | 0.010 | 0.002 | 2.71E-07 |
| rs2336895 | 5 | 166990695 | G | A | 0.012 | 0.002 | 4.60E-08 | 0.001 | 0.002 | 4.82E-01 |
| rs72828890 | 5 | 167806836 | C | T | 0.019 | 0.003 | 4.90E-09 | -0.006 | 0.003 | 1.73E-02 |
| rs255292 | 5 | 172580866 | C | A | -0.012 | 0.002 | 1.70E-08 | 0.004 | 0.002 | 4.14E-02 |
| rs72834698 | 6 | 26176517 | G | A | 0.023 | 0.003 | 2.70E-13 | -0.014 | 0.002 | 2.15E-08 |
| rs10947452 | 6 | 33803752 | T | C | 0.014 | 0.002 | 6.40E-10 | -0.009 | 0.002 | 1.41E-06 |
| rs9471333 | 6 | 40362023 | C | T | 0.013 | 0.002 | 1.50E-09 | -0.009 | 0.002 | 3.40E-07 |
| rs4523073 | 6 | 67550288 | A | G | -0.014 | 0.002 | 5.00E-10 | 0.002 | 0.002 | 1.82E-01 |
| rs6905544 | 6 | 98411631 | A | G | -0.019 | 0.002 | 8.50E-18 | 0.023 | 0.002 | 7.33E-41 |
| rs17789218 | 6 | 100600097 | T | C | 0.019 | 0.003 | 1.40E-13 | -0.001 | 0.002 | 6.08E-01 |
| rs78394231 | 6 | 107649123 | T | C | -0.021 | 0.004 | 9.70E-09 | 0.006 | 0.003 | 4.53E-02 |
| rs2184364 | 6 | 143183596 | A | G | 0.016 | 0.003 | 3.00E-09 | -0.009 | 0.002 | 2.05E-05 |
| rs9718104 | 6 | 166170539 | T | G | -0.041 | 0.005 | 9.30E-19 | -0.003 | 0.004 | 4.30E-01 |
| rs17568389 | 7 | 8091876 | T | A | 0.015 | 0.002 | 8.60E-13 | -0.012 | 0.002 | 1.53E-12 |
| rs10234444 | 7 | 41828527 | G | A | 0.016 | 0.003 | 2.90E-08 | -0.008 | 0.002 | 8.80E-04 |
| rs6973656 | 7 | 77422583 | A | G | -0.013 | 0.002 | 1.00E-09 | 0.004 | 0.002 | 3.09E-02 |
| rs973734 | 7 | 89387578 | C | A | 0.017 | 0.003 | 1.50E-08 | -0.007 | 0.002 | 1.58E-03 |
| rs3137 | 7 | 99130875 | G | A | 0.017 | 0.003 | 1.40E-08 | -0.004 | 0.002 | 8.71E-02 |
| rs2694151 | 7 | 101902180 | C | T | 0.026 | 0.005 | 2.30E-08 | -0.009 | 0.004 | 8.53E-03 |
| rs62471080 | 7 | 109128060 | G | C | -0.013 | 0.002 | 3.70E-09 | 0.005 | 0.002 | 1.48E-03 |
| rs7788008 | 7 | 112972483 | G | A | 0.013 | 0.002 | 5.90E-09 | -0.004 | 0.002 | 9.59E-03 |
| rs10246289 | 7 | 115544763 | A | G | 0.019 | 0.003 | 1.70E-08 | -0.007 | 0.003 | 9.30E-03 |
| rs11763734 | 7 | 126400910 | A | C | -0.012 | 0.002 | 8.20E-09 | 0.007 | 0.002 | 3.27E-05 |
| rs7834121 | 8 | 10770255 | G | T | -0.014 | 0.002 | 1.10E-10 | -0.002 | 0.002 | 2.03E-01 |
| rs1421334 | 8 | 30865733 | A | C | 0.017 | 0.002 | 2.20E-15 | -0.011 | 0.002 | 5.46E-11 |
| rs6996198 | 8 | 65463442 | C | T | -0.016 | 0.003 | 3.50E-08 | 0.007 | 0.002 | 1.73E-03 |
| rs6472942 | 8 | 76827190 | T | C | -0.013 | 0.002 | 1.80E-09 | 0.000 | 0.002 | 9.62E-01 |
| rs11985394 | 8 | 92667521 | T | A | 0.012 | 0.002 | 1.30E-08 | -0.002 | 0.002 | 2.66E-01 |
| rs72671494 | 8 | 93195457 | T | C | -0.017 | 0.003 | 4.50E-08 | 0.004 | 0.002 | 1.18E-01 |
| rs12541615 | 8 | 118870151 | T | C | -0.018 | 0.003 | 3.90E-10 | 0.008 | 0.002 | 1.03E-04 |
| rs2717559 | 8 | 143882420 | A | G | 0.012 | 0.002 | 1.50E-08 | -0.009 | 0.002 | 5.93E-07 |
| rs2616830 | 9 | 1721385 | G | A | 0.016 | 0.002 | 2.90E-14 | -0.015 | 0.002 | 1.23E-17 |
| rs2291681 | 9 | 14790841 | G | T | 0.013 | 0.002 | 7.90E-09 | -0.004 | 0.002 | 1.37E-02 |
| rs34864022 | 9 | 22609110 | A | G | -0.026 | 0.004 | 1.40E-09 | 0.009 | 0.003 | 8.92E-03 |
| rs12554512 | 9 | 23352293 | T | C | 0.021 | 0.002 | 3.80E-21 | -0.024 | 0.002 | 1.69E-43 |
| rs2791574 | 9 | 82212094 | T | G | -0.013 | 0.002 | 3.00E-08 | 0.008 | 0.002 | 2.22E-06 |
| rs870151 | 9 | 92230747 | T | A | -0.016 | 0.002 | 8.80E-13 | 0.009 | 0.002 | 1.28E-07 |
| rs2787374 | 9 | 103054951 | T | C | 0.012 | 0.002 | 1.20E-08 | -0.004 | 0.002 | 1.24E-02 |
| rs10739499 | 9 | 120514261 | C | G | 0.014 | 0.002 | 1.50E-09 | -0.001 | 0.002 | 6.41E-01 |
| rs7043521 | 9 | 124602779 | A | T | 0.014 | 0.002 | 6.50E-11 | -0.014 | 0.002 | 5.88E-16 |
| rs12342024 | 9 | 126134232 | G | T | -0.021 | 0.003 | 1.70E-09 | 0.009 | 0.003 | 8.77E-04 |
| rs4382592 | 9 | 134870755 | T | G | 0.014 | 0.002 | 6.70E-09 | -0.016 | 0.002 | 1.01E-18 |
| rs2073869 | 9 | 135763816 | C | T | 0.019 | 0.003 | 1.50E-10 | -0.010 | 0.002 | 1.04E-05 |
| 9:139926402_T_C | 9 | 139926402 | T | C | -0.013 | 0.002 | 2.00E-08 | 0.008 | 0.002 | 2.40E-05 |
| rs1623025 | 10 | 11164813 | G | C | -0.012 | 0.002 | 1.40E-08 | 0.010 | 0.002 | 4.50E-09 |
| rs1243182 | 10 | 21916728 | C | T | -0.019 | 0.002 | 2.00E-15 | 0.008 | 0.002 | 4.66E-05 |
| rs2045147 | 10 | 56660437 | A | G | 0.013 | 0.002 | 5.90E-09 | -0.002 | 0.002 | 1.58E-01 |
| rs10994943 | 10 | 63591413 | T | G | 0.013 | 0.002 | 3.10E-09 | -0.008 | 0.002 | 5.94E-06 |
| rs11201422 | 10 | 86924483 | T | C | 0.013 | 0.002 | 3.40E-08 | -0.006 | 0.002 | 9.87E-04 |
| rs10786658 | 10 | 103737193 | A | T | -0.014 | 0.002 | 4.60E-11 | 0.016 | 0.002 | 1.07E-20 |
| rs7089973 | 10 | 116569565 | C | A | -0.013 | 0.002 | 2.40E-09 | 0.003 | 0.002 | 1.01E-01 |
| rs11245482 | 10 | 126733546 | T | C | -0.013 | 0.002 | 2.60E-09 | 0.006 | 0.002 | 5.32E-04 |
| rs17727474 | 10 | 127182959 | C | T | 0.018 | 0.003 | 3.10E-09 | -0.003 | 0.002 | 1.30E-01 |
| rs12289262 | 11 | 12894758 | C | T | -0.014 | 0.002 | 2.00E-08 | 0.014 | 0.002 | 9.54E-13 |
| rs801733 | 11 | 65934549 | A | C | 0.017 | 0.002 | 7.30E-14 | -0.010 | 0.002 | 1.65E-07 |
| rs116175338 | 11 | 72467875 | G | T | -0.014 | 0.003 | 1.40E-08 | 0.016 | 0.002 | 1.36E-16 |
| rs12272012 | 11 | 84861286 | G | C | -0.030 | 0.005 | 1.90E-08 | 0.009 | 0.004 | 3.62E-02 |
| rs11020045 | 11 | 92487907 | A | C | -0.013 | 0.002 | 1.60E-08 | 0.007 | 0.002 | 1.88E-04 |
| rs17207890 | 11 | 95490754 | G | A | 0.016 | 0.002 | 6.70E-12 | -0.014 | 0.002 | 3.58E-14 |
| rs10791546 | 11 | 101830126 | C | T | 0.012 | 0.002 | 4.10E-08 | -0.005 | 0.002 | 3.38E-03 |
| rs648044 | 11 | 114030799 | A | G | -0.014 | 0.002 | 1.00E-09 | 0.009 | 0.002 | 2.75E-07 |
| rs11218575 | 11 | 122154576 | C | T | 0.015 | 0.002 | 2.10E-12 | -0.006 | 0.002 | 5.66E-04 |
| rs74802478 | 11 | 131966907 | G | A | 0.017 | 0.003 | 3.50E-09 | 0.001 | 0.002 | 8.17E-01 |
| rs4937842 | 11 | 133734542 | G | C | -0.013 | 0.002 | 1.40E-08 | 0.002 | 0.002 | 1.87E-01 |
| rs10772643 | 12 | 13415288 | C | T | 0.025 | 0.003 | 1.30E-12 | -0.016 | 0.003 | 3.54E-09 |
| rs74996610 | 12 | 24075007 | C | G | -0.031 | 0.005 | 4.10E-09 | 0.005 | 0.004 | 2.15E-01 |
| rs10771746 | 12 | 30791864 | C | T | -0.014 | 0.002 | 2.50E-09 | 0.005 | 0.002 | 4.35E-03 |
| rs10876864 | 12 | 56401085 | G | A | -0.013 | 0.002 | 1.00E-09 | 0.016 | 0.002 | 1.22E-19 |
| rs8756 | 12 | 66359752 | C | A | -0.013 | 0.002 | 5.30E-10 | 0.008 | 0.002 | 1.41E-06 |
| rs11179838 | 12 | 74321123 | C | G | 0.013 | 0.002 | 2.10E-08 | -0.008 | 0.002 | 1.01E-05 |
| rs2164744 | 12 | 109872039 | T | C | -0.013 | 0.002 | 1.10E-08 | 0.004 | 0.002 | 1.28E-02 |
| rs2173650 | 12 | 117525235 | G | T | 0.018 | 0.003 | 4.60E-09 | -0.012 | 0.002 | 3.06E-07 |
| rs1790135 | 12 | 123669235 | C | T | -0.014 | 0.003 | 2.60E-08 | 0.021 | 0.002 | 2.00E-25 |
| rs9563168 | 13 | 54247827 | G | A | 0.018 | 0.003 | 4.30E-11 | -0.010 | 0.002 | 5.93E-07 |
| rs9569734 | 13 | 58319476 | A | G | 0.019 | 0.003 | 3.40E-10 | -0.009 | 0.002 | 2.77E-04 |
| rs9528023 | 13 | 60226991 | C | T | 0.013 | 0.002 | 3.80E-08 | -0.010 | 0.002 | 1.41E-07 |
| rs56858768 | 13 | 86511730 | G | A | -0.015 | 0.002 | 3.40E-10 | 0.006 | 0.002 | 1.18E-03 |
| rs553397860 | 13 | 99051118 | A | G | 0.017 | 0.003 | 8.90E-09 | -0.013 | 0.002 | 5.55E-08 |
| rs7991062 | 13 | 100713194 | C | G | -0.018 | 0.002 | 8.80E-15 | 0.008 | 0.002 | 3.09E-06 |
| rs11629120 | 14 | 23458457 | T | C | -0.013 | 0.002 | 1.10E-08 | 0.012 | 0.002 | 7.86E-13 |
| rs405797 | 14 | 26972834 | T | A | -0.015 | 0.003 | 2.80E-09 | 0.015 | 0.002 | 2.12E-14 |
| rs1022785 | 14 | 29571653 | G | A | 0.018 | 0.003 | 7.10E-09 | -0.018 | 0.002 | 2.05E-13 |
| rs10145592 | 14 | 94287215 | C | G | -0.015 | 0.002 | 1.80E-11 | 0.011 | 0.002 | 1.02E-09 |
| rs7157001 | 14 | 99749484 | A | G | -0.014 | 0.002 | 2.30E-08 | 0.009 | 0.002 | 1.29E-05 |
| rs8043253 | 15 | 41503730 | C | T | -0.012 | 0.002 | 3.40E-08 | 0.001 | 0.002 | 5.79E-01 |
| rs2460 | 15 | 53073084 | G | A | -0.015 | 0.002 | 5.00E-10 | 0.009 | 0.002 | 1.04E-05 |
| rs4775373 | 15 | 61476936 | T | C | 0.013 | 0.002 | 4.70E-09 | -0.007 | 0.002 | 2.40E-04 |
| rs61331678 | 15 | 77995949 | G | C | 0.015 | 0.002 | 6.10E-12 | -0.013 | 0.002 | 2.63E-13 |
| rs35574015 | 16 | 15146061 | T | C | -0.013 | 0.002 | 3.00E-08 | 0.002 | 0.002 | 2.21E-01 |
| rs7189927 | 16 | 28913787 | T | C | 0.015 | 0.002 | 3.40E-11 | -0.015 | 0.002 | 7.31E-18 |
| rs749671 | 16 | 31088347 | G | A | 0.016 | 0.002 | 2.70E-12 | -0.006 | 0.002 | 3.15E-04 |
| rs7184800 | 16 | 53509131 | G | A | 0.017 | 0.002 | 8.20E-13 | -0.010 | 0.002 | 2.38E-08 |
| rs2447098 | 17 | 2277720 | C | A | -0.015 | 0.002 | 6.80E-12 | 0.008 | 0.002 | 7.73E-07 |
| rs11654952 | 17 | 4813799 | T | G | -0.017 | 0.003 | 4.30E-08 | 0.003 | 0.003 | 3.08E-01 |
| rs9902312 | 17 | 65070304 | T | C | 0.015 | 0.002 | 4.50E-11 | 0.000 | 0.002 | 8.57E-01 |
| rs11657730 | 17 | 79372489 | C | T | 0.013 | 0.002 | 1.10E-08 | -0.013 | 0.002 | 4.31E-11 |
| rs303753 | 18 | 21074922 | G | A | -0.014 | 0.002 | 2.70E-10 | 0.015 | 0.002 | 2.60E-16 |
| rs11081851 | 18 | 31783262 | G | A | -0.013 | 0.002 | 1.00E-08 | 0.006 | 0.002 | 6.70E-04 |
| rs9964724 | 18 | 35159124 | C | T | 0.018 | 0.002 | 3.30E-14 | -0.020 | 0.002 | 2.66E-27 |
| rs1156541 | 18 | 39952989 | C | T | 0.014 | 0.003 | 2.80E-08 | -0.005 | 0.002 | 1.23E-02 |
| rs148544378 | 18 | 40323567 | C | T | 0.053 | 0.008 | 2.20E-12 | -0.020 | 0.007 | 2.03E-03 |
| rs17512836 | 18 | 53194961 | T | C | 0.042 | 0.007 | 7.00E-10 | -0.028 | 0.005 | 4.70E-07 |
| rs7248205 | 19 | 10770305 | C | T | 0.014 | 0.002 | 3.40E-10 | -0.005 | 0.002 | 6.35E-03 |
| rs111901094 | 19 | 19513570 | G | T | -0.017 | 0.003 | 2.00E-09 | 0.011 | 0.003 | 5.48E-05 |
| rs6131281 | 20 | 11891724 | C | T | 0.016 | 0.002 | 3.10E-13 | -0.006 | 0.002 | 2.46E-04 |
| rs6141814 | 20 | 31368960 | C | A | -0.014 | 0.002 | 1.30E-09 | 0.003 | 0.002 | 5.19E-02 |
| rs4810312 | 20 | 39968707 | C | G | -0.013 | 0.002 | 1.20E-08 | -0.001 | 0.002 | 5.84E-01 |
| rs6124476 | 20 | 41202830 | T | C | 0.012 | 0.002 | 1.80E-08 | -0.008 | 0.002 | 9.48E-06 |
| rs6031440 | 20 | 42809317 | A | T | 0.012 | 0.002 | 1.80E-08 | -0.005 | 0.002 | 3.13E-03 |
| rs55700114 | 20 | 43717080 | G | A | -0.014 | 0.002 | 2.10E-09 | 0.012 | 0.002 | 3.34E-10 |
| rs6125906 | 20 | 48729803 | C | A | -0.021 | 0.004 | 3.90E-08 | 0.006 | 0.003 | 4.48E-02 |
| rs56103247 | 20 | 62483184 | C | T | 0.030 | 0.005 | 3.80E-10 | -0.016 | 0.004 | 3.14E-05 |
| rs10427502 | 21 | 40654840 | G | A | 0.014 | 0.002 | 8.60E-10 | -0.004 | 0.002 | 1.36E-02 |
| rs2092829 | 22 | 31780514 | G | A | 0.014 | 0.002 | 1.30E-08 | -0.002 | 0.002 | 3.89E-01 |

| **Table S32. Betas of the bidirectional MR between smoking and leisure computer use** | | | | | | | | | | |
| --- | --- | --- | --- | --- | --- | --- | --- | --- | --- | --- |
| **1) MR of smoking on leisure computer use** | | | | | | | | | | |
| SNP | Chr | Position | EA | OA | beta_smk | se_smk | pval_smk | beta_pc | se_pc | pval_pc |
| rs1193237 | 1 | 7526486 | G | C | -0.008 | 0.001 | 2.80E-08 | -0.001 | 0.002 | 6.90E-01 |
| rs4949465 | 1 | 32178489 | T | C | -0.012 | 0.002 | 1.70E-08 | 0.007 | 0.003 | 4.40E-02 |
| rs549845 | 1 | 44076469 | G | A | 0.011 | 0.002 | 8.30E-14 | 0.002 | 0.002 | 3.60E-01 |
| rs1933270 | 1 | 49977965 | T | G | 0.009 | 0.001 | 1.50E-10 | -0.005 | 0.002 | 3.50E-02 |
| rs7528604 | 1 | 66407352 | G | A | 0.010 | 0.001 | 5.70E-12 | -0.001 | 0.002 | 5.20E-01 |
| rs11210229 | 1 | 73860028 | A | G | 0.012 | 0.001 | 2.00E-16 | -0.002 | 0.002 | 4.70E-01 |
| rs7553348 | 1 | 75005067 | G | A | 0.010 | 0.001 | 5.20E-12 | -0.004 | 0.002 | 6.20E-02 |
| rs10922907 | 1 | 91193049 | A | T | 0.010 | 0.001 | 3.00E-13 | -0.003 | 0.002 | 2.20E-01 |
| rs1931263 | 1 | 96175101 | G | T | -0.008 | 0.001 | 4.00E-08 | 0.007 | 0.002 | 1.90E-03 |
| rs7519626 | 1 | 99514554 | C | T | 0.008 | 0.001 | 1.20E-08 | -0.001 | 0.002 | 5.60E-01 |
| rs9435340 | 1 | 107593201 | T | A | 0.008 | 0.001 | 1.20E-08 | 0.000 | 0.002 | 9.20E-01 |
| rs10918701 | 1 | 162090536 | G | A | 0.008 | 0.001 | 2.10E-08 | -0.007 | 0.002 | 2.40E-03 |
| rs2867112 | 2 | 651349 | T | G | 0.015 | 0.002 | 4.80E-15 | 0.009 | 0.003 | 1.80E-03 |
| rs6741228 | 2 | 22548774 | T | C | 0.008 | 0.001 | 1.60E-08 | -0.002 | 0.002 | 3.70E-01 |
| rs62135536 | 2 | 44326028 | C | T | 0.024 | 0.004 | 8.00E-10 | 0.005 | 0.006 | 4.10E-01 |
| rs7569203 | 2 | 45154418 | A | C | -0.011 | 0.002 | 7.40E-13 | 0.000 | 0.002 | 8.80E-01 |
| rs13016665 | 2 | 57995348 | C | A | -0.008 | 0.001 | 1.80E-09 | 0.010 | 0.002 | 2.20E-05 |
| rs4671357 | 2 | 60136176 | T | C | -0.009 | 0.001 | 1.10E-11 | -0.001 | 0.002 | 5.10E-01 |
| rs359243 | 2 | 60475509 | T | C | -0.009 | 0.001 | 9.50E-10 | -0.010 | 0.002 | 2.50E-05 |
| rs2678670 | 2 | 104469564 | A | T | 0.009 | 0.001 | 3.10E-10 | -0.002 | 0.002 | 4.50E-01 |
| rs62155874 | 2 | 105973094 | A | G | -0.017 | 0.002 | 5.20E-16 | 0.002 | 0.003 | 5.80E-01 |
| rs3811038 | 2 | 113240183 | T | C | -0.010 | 0.002 | 8.90E-10 | -0.004 | 0.002 | 1.40E-01 |
| rs2890772 | 2 | 146175106 | G | T | -0.014 | 0.001 | 2.10E-22 | -0.008 | 0.002 | 2.20E-04 |
| rs62175972 | 2 | 161362830 | T | C | 0.022 | 0.004 | 1.70E-08 | -0.005 | 0.006 | 4.10E-01 |
| rs3769949 | 2 | 166199284 | T | A | -0.008 | 0.001 | 2.50E-09 | 0.005 | 0.002 | 2.70E-02 |
| rs13009008 | 2 | 174043233 | A | G | 0.009 | 0.001 | 4.60E-09 | 0.000 | 0.002 | 8.70E-01 |
| rs4473348 | 2 | 182073742 | A | T | -0.010 | 0.002 | 6.40E-11 | 0.003 | 0.003 | 3.10E-01 |
| rs12623702 | 2 | 202885506 | A | G | -0.010 | 0.001 | 7.70E-12 | 0.007 | 0.002 | 3.60E-03 |
| rs6779302 | 3 | 16859710 | G | T | -0.009 | 0.001 | 1.20E-09 | -0.004 | 0.002 | 7.60E-02 |
| rs6778080 | 3 | 49317338 | T | C | 0.011 | 0.002 | 1.30E-12 | -0.010 | 0.002 | 8.80E-05 |
| rs775758 | 3 | 77582005 | A | T | 0.008 | 0.001 | 1.10E-08 | 0.003 | 0.002 | 1.80E-01 |
| rs421983 | 3 | 84892866 | T | C | 0.009 | 0.001 | 3.30E-10 | 0.004 | 0.002 | 6.90E-02 |
| rs326341 | 3 | 107811142 | G | A | 0.009 | 0.001 | 1.20E-11 | -0.001 | 0.002 | 6.10E-01 |
| rs73220544 | 3 | 131074511 | A | C | -0.011 | 0.002 | 1.50E-08 | 0.001 | 0.003 | 6.90E-01 |
| rs9842947 | 3 | 157412246 | C | T | -0.009 | 0.001 | 3.10E-09 | -0.001 | 0.002 | 5.40E-01 |
| rs624833 | 4 | 2881256 | T | G | 0.009 | 0.002 | 6.60E-10 | -0.007 | 0.002 | 5.70E-03 |
| rs61796681 | 4 | 23678196 | A | T | -0.013 | 0.002 | 4.20E-08 | 0.009 | 0.004 | 1.70E-02 |
| rs317021 | 4 | 35418368 | T | A | -0.012 | 0.002 | 1.10E-10 | 0.006 | 0.003 | 2.90E-02 |
| rs72678864 | 4 | 112422145 | G | A | 0.012 | 0.002 | 1.60E-11 | -0.004 | 0.003 | 1.50E-01 |
| rs17576594 | 4 | 147952241 | G | A | 0.011 | 0.002 | 1.70E-12 | -0.002 | 0.002 | 5.10E-01 |
| rs11948770 | 5 | 13246336 | T | C | -0.010 | 0.002 | 4.90E-10 | -0.003 | 0.003 | 2.10E-01 |
| rs71627581 | 5 | 43161351 | G | A | 0.013 | 0.002 | 1.60E-09 | -0.005 | 0.003 | 1.90E-01 |
| rs10052591 | 5 | 50812738 | T | C | 0.008 | 0.001 | 2.10E-09 | 0.008 | 0.002 | 7.10E-04 |
| rs2080870 | 5 | 60388313 | A | T | 0.009 | 0.002 | 4.90E-08 | -0.010 | 0.003 | 4.20E-05 |
| rs4571506 | 5 | 87756918 | C | T | 0.008 | 0.001 | 1.50E-08 | -0.008 | 0.002 | 3.80E-04 |
| rs4957528 | 5 | 106420589 | A | C | -0.010 | 0.002 | 4.20E-09 | 0.002 | 0.003 | 5.00E-01 |
| rs329120 | 5 | 133861756 | C | T | 0.010 | 0.001 | 6.30E-12 | -0.005 | 0.002 | 1.70E-02 |
| rs986391 | 5 | 166993972 | G | A | 0.011 | 0.001 | 9.40E-15 | -0.006 | 0.002 | 1.60E-02 |
| rs13153393 | 5 | 167604213 | A | G | -0.014 | 0.002 | 2.50E-10 | -0.002 | 0.003 | 6.60E-01 |
| rs245774 | 5 | 170530930 | A | G | -0.009 | 0.002 | 7.40E-09 | -0.002 | 0.002 | 5.00E-01 |
| rs6935954 | 6 | 26255451 | A | G | 0.010 | 0.001 | 8.20E-12 | -0.008 | 0.002 | 4.40E-04 |
| rs2254710 | 6 | 37477000 | C | A | 0.009 | 0.002 | 3.50E-08 | 0.002 | 0.003 | 5.20E-01 |
| rs2894808 | 6 | 52861990 | T | A | -0.015 | 0.003 | 3.50E-09 | 0.001 | 0.004 | 7.60E-01 |
| rs12202536 | 6 | 67475273 | A | G | -0.008 | 0.001 | 2.80E-09 | 0.002 | 0.002 | 2.90E-01 |
| rs7766610 | 6 | 111707821 | C | A | 0.013 | 0.002 | 2.20E-12 | 0.000 | 0.003 | 9.20E-01 |
| rs1922018 | 7 | 3560401 | C | T | 0.010 | 0.001 | 3.00E-12 | 0.000 | 0.002 | 8.80E-01 |
| rs10226228 | 7 | 32315613 | A | G | -0.011 | 0.001 | 2.00E-15 | 0.002 | 0.002 | 4.40E-01 |
| rs11768481 | 7 | 96629103 | C | A | 0.009 | 0.001 | 9.90E-10 | 0.001 | 0.002 | 6.80E-01 |
| rs6962772 | 7 | 99081730 | A | G | 0.011 | 0.002 | 7.80E-09 | 0.012 | 0.003 | 5.30E-05 |
| rs10282292 | 7 | 111092478 | C | T | 0.009 | 0.001 | 5.90E-10 | 0.006 | 0.002 | 4.80E-03 |
| rs2401924 | 7 | 115057862 | G | C | 0.011 | 0.001 | 2.70E-14 | 0.008 | 0.002 | 6.60E-04 |
| rs7807019 | 7 | 117543063 | A | G | -0.010 | 0.001 | 6.70E-14 | -0.001 | 0.002 | 6.40E-01 |
| rs6957896 | 7 | 132309592 | C | T | -0.008 | 0.001 | 4.50E-08 | -0.001 | 0.002 | 6.90E-01 |
| rs4731925 | 7 | 132664757 | C | T | -0.008 | 0.001 | 2.60E-08 | 0.002 | 0.002 | 3.50E-01 |
| rs35169606 | 8 | 9604066 | T | G | 0.009 | 0.001 | 1.20E-09 | 0.002 | 0.002 | 4.80E-01 |
| rs11783093 | 8 | 27425349 | C | T | 0.016 | 0.002 | 1.20E-16 | -0.001 | 0.003 | 6.30E-01 |
| rs2062882 | 8 | 91839576 | G | A | -0.008 | 0.001 | 1.10E-08 | 0.008 | 0.002 | 8.60E-04 |
| rs72674867 | 8 | 95578201 | A | T | 0.009 | 0.002 | 3.80E-08 | 0.001 | 0.003 | 6.00E-01 |
| rs4543592 | 9 | 3014254 | T | C | -0.009 | 0.001 | 4.50E-10 | -0.001 | 0.002 | 8.00E-01 |
| rs7039819 | 9 | 82430418 | G | A | 0.009 | 0.001 | 5.10E-10 | -0.001 | 0.002 | 5.20E-01 |
| rs1246265 | 9 | 86761745 | T | C | -0.009 | 0.002 | 4.20E-09 | 0.002 | 0.002 | 4.40E-01 |
| rs1221148 | 9 | 122046875 | C | G | 0.009 | 0.001 | 7.30E-11 | -0.006 | 0.002 | 9.40E-03 |
| rs13296519 | 9 | 128471924 | G | T | -0.010 | 0.001 | 8.10E-12 | 0.001 | 0.002 | 7.80E-01 |
| rs113382419 | 9 | 136463019 | C | A | -0.028 | 0.002 | 3.00E-37 | 0.000 | 0.003 | 9.00E-01 |
| rs11255908 | 10 | 8802912 | T | G | -0.010 | 0.002 | 2.30E-10 | 0.003 | 0.003 | 1.80E-01 |
| rs2675638 | 10 | 63576286 | G | A | 0.008 | 0.001 | 1.30E-09 | -0.001 | 0.002 | 5.40E-01 |
| rs10823968 | 10 | 74738269 | A | T | 0.008 | 0.001 | 2.10E-08 | -0.005 | 0.002 | 5.00E-02 |
| rs17553262 | 10 | 92912773 | A | C | -0.013 | 0.002 | 5.30E-09 | -0.001 | 0.003 | 7.80E-01 |
| rs7077678 | 10 | 104438565 | C | T | 0.009 | 0.001 | 2.60E-09 | 0.005 | 0.002 | 4.10E-02 |
| rs12244388 | 10 | 104640052 | G | A | -0.013 | 0.001 | 1.40E-19 | 0.000 | 0.002 | 8.90E-01 |
| rs3896224 | 10 | 106467853 | A | G | 0.010 | 0.001 | 1.10E-11 | 0.006 | 0.002 | 1.10E-02 |
| rs34866095 | 11 | 16377356 | A | G | -0.009 | 0.002 | 1.20E-08 | -0.002 | 0.002 | 4.40E-01 |
| rs75742406 | 11 | 17070365 | G | A | 0.010 | 0.002 | 1.30E-09 | -0.003 | 0.003 | 2.70E-01 |
| rs17309874 | 11 | 27667236 | G | A | -0.011 | 0.002 | 9.70E-13 | -0.006 | 0.003 | 1.20E-02 |
| rs4391802 | 11 | 28674592 | A | G | 0.010 | 0.002 | 1.40E-11 | -0.004 | 0.002 | 1.30E-01 |
| rs112282219 | 11 | 46632809 | G | A | -0.023 | 0.004 | 3.80E-11 | 0.004 | 0.006 | 4.50E-01 |
| rs9919670 | 11 | 112877304 | G | A | -0.015 | 0.001 | 7.60E-27 | 0.006 | 0.002 | 5.00E-03 |
| rs74086911 | 12 | 50015942 | G | A | 0.015 | 0.003 | 2.10E-08 | -0.003 | 0.004 | 4.10E-01 |
| rs7297175 | 12 | 56473808 | T | C | -0.008 | 0.001 | 6.60E-09 | 0.003 | 0.002 | 1.70E-01 |
| rs10879871 | 12 | 75380511 | T | G | -0.010 | 0.001 | 5.00E-11 | 0.005 | 0.002 | 2.70E-02 |
| rs12831617 | 12 | 84758368 | C | T | -0.009 | 0.002 | 1.90E-08 | 0.004 | 0.003 | 9.30E-02 |
| rs6562474 | 13 | 67332812 | C | G | 0.008 | 0.001 | 1.00E-08 | -0.005 | 0.002 | 4.00E-02 |
| rs7333559 | 13 | 100546450 | G | A | 0.011 | 0.002 | 3.20E-10 | 0.002 | 0.003 | 4.60E-01 |
| rs860326 | 14 | 57342912 | C | T | 0.008 | 0.001 | 2.70E-09 | -0.007 | 0.002 | 2.60E-03 |
| rs7155595 | 14 | 77502546 | A | C | -0.009 | 0.001 | 2.50E-09 | 0.006 | 0.002 | 1.50E-02 |
| rs3742365 | 14 | 104198251 | T | C | -0.011 | 0.001 | 2.50E-14 | -0.004 | 0.002 | 4.70E-02 |
| rs35175834 | 15 | 47680815 | G | A | -0.016 | 0.002 | 4.60E-22 | -0.002 | 0.003 | 3.80E-01 |
| rs28485305 | 15 | 74044197 | C | T | 0.008 | 0.001 | 2.60E-08 | 0.003 | 0.002 | 1.50E-01 |
| rs8042849 | 15 | 78817929 | C | T | 0.019 | 0.001 | 1.80E-39 | -0.002 | 0.002 | 3.70E-01 |
| rs8042134 | 15 | 97514404 | T | G | -0.010 | 0.001 | 1.30E-12 | 0.000 | 0.002 | 9.80E-01 |
| rs6598539 | 15 | 99204483 | T | C | -0.008 | 0.001 | 4.50E-09 | -0.002 | 0.002 | 2.80E-01 |
| rs11861214 | 16 | 746611 | G | T | 0.009 | 0.002 | 2.00E-08 | -0.002 | 0.003 | 3.90E-01 |
| rs12708665 | 16 | 24728227 | A | G | -0.009 | 0.002 | 3.50E-09 | 0.003 | 0.002 | 1.70E-01 |
| rs57611503 | 16 | 31165795 | G | A | 0.008 | 0.001 | 4.00E-08 | 0.001 | 0.002 | 7.00E-01 |
| rs889398 | 16 | 69556715 | C | T | 0.009 | 0.001 | 6.30E-11 | 0.002 | 0.002 | 4.90E-01 |
| rs60952428 | 16 | 75640521 | T | C | 0.013 | 0.002 | 3.00E-08 | 0.002 | 0.004 | 5.70E-01 |
| rs1050847 | 16 | 87443734 | C | T | 0.008 | 0.001 | 1.40E-08 | 0.000 | 0.002 | 8.50E-01 |
| rs369230 | 16 | 89645437 | G | T | -0.009 | 0.002 | 1.80E-09 | -0.001 | 0.002 | 6.30E-01 |
| rs8614 | 17 | 27588806 | C | A | -0.011 | 0.002 | 1.80E-10 | -0.004 | 0.003 | 1.80E-01 |
| rs732083 | 17 | 37834367 | G | A | 0.008 | 0.001 | 1.50E-08 | -0.001 | 0.002 | 6.10E-01 |
| rs9904288 | 17 | 47031973 | T | C | 0.008 | 0.002 | 3.10E-08 | -0.004 | 0.002 | 9.30E-02 |
| rs67596067 | 17 | 50333733 | G | A | -0.009 | 0.001 | 1.20E-09 | 0.005 | 0.002 | 2.70E-02 |
| rs12967855 | 18 | 35138245 | A | G | 0.008 | 0.001 | 3.10E-08 | -0.005 | 0.002 | 4.50E-02 |
| rs62098013 | 18 | 50863861 | G | A | -0.009 | 0.001 | 4.10E-09 | 0.003 | 0.002 | 2.40E-01 |
| rs71367545 | 18 | 77576337 | G | A | -0.010 | 0.002 | 1.40E-09 | -0.004 | 0.003 | 1.80E-01 |
| rs76608582 | 19 | 4474725 | C | A | 0.022 | 0.003 | 3.20E-10 | 0.001 | 0.005 | 8.40E-01 |
| rs35343344 | 19 | 18471610 | C | A | 0.009 | 0.002 | 8.80E-09 | -0.005 | 0.003 | 6.20E-02 |
| rs4814873 | 20 | 19616429 | C | T | 0.010 | 0.002 | 2.90E-09 | -0.008 | 0.003 | 2.80E-03 |
| rs6119897 | 20 | 31145415 | G | A | -0.013 | 0.002 | 3.60E-15 | 0.004 | 0.003 | 1.20E-01 |
| rs12481282 | 20 | 44761377 | G | C | -0.009 | 0.002 | 7.80E-09 | -0.002 | 0.002 | 3.30E-01 |
| rs348809 | 20 | 59032097 | A | G | -0.008 | 0.001 | 1.30E-08 | -0.001 | 0.002 | 7.50E-01 |
| rs6011779 | 20 | 61984317 | C | T | 0.019 | 0.002 | 2.30E-27 | -0.007 | 0.003 | 1.10E-02 |
| rs147412694 | 21 | 40702786 | G | A | -0.012 | 0.002 | 2.90E-09 | -0.008 | 0.003 | 1.30E-02 |
| rs2838834 | 21 | 46665208 | C | T | -0.009 | 0.002 | 6.30E-10 | 0.000 | 0.002 | 9.80E-01 |
| rs136233 | 22 | 31212410 | A | G | -0.010 | 0.002 | 1.80E-08 | 0.001 | 0.003 | 6.70E-01 |
| rs202645 | 22 | 41798520 | A | G | -0.010 | 0.002 | 3.90E-09 | 0.005 | 0.003 | 7.60E-02 |
| **2) MR of leisure computer use on smoking** | | | | | | | | | | |
| SNP | Chr | Position | EA | OA | beta_pc | se_pc | pval_pc | beta_smk | se_smk | pval_smk |
| rs2748985 | 1 | 1853184 | T | C | -0.015 | 0.002 | 4.10E-12 | -0.001 | 0.001 | 4.40E-01 |
| rs12145677 | 1 | 110023610 | G | A | -0.017 | 0.002 | 1.20E-12 | 0.002 | 0.002 | 2.20E-01 |
| rs7526456 | 1 | 197773734 | T | C | -0.013 | 0.002 | 1.20E-08 | -0.001 | 0.001 | 3.00E-01 |
| rs10754920 | 2 | 146456888 | C | G | 0.020 | 0.003 | 6.70E-09 | 0.003 | 0.002 | 2.10E-01 |
| rs6744254 | 2 | 155986195 | C | T | -0.016 | 0.002 | 5.40E-13 | -0.006 | 0.001 | 9.40E-06 |
| rs11274218 | 2 | 215035001 | C | T | -0.014 | 0.002 | 2.50E-09 | -0.004 | 0.001 | 7.80E-03 |
| rs4592851 | 2 | 215364232 | G | T | -0.014 | 0.003 | 1.40E-08 | -0.001 | 0.002 | 4.80E-01 |
| rs10208088 | 2 | 221055873 | C | T | 0.013 | 0.002 | 4.40E-09 | 0.001 | 0.001 | 6.90E-01 |
| rs11708955 | 3 | 49540114 | T | C | -0.016 | 0.002 | 4.20E-11 | 0.007 | 0.002 | 1.20E-05 |
| rs6774533 | 3 | 62471086 | C | T | -0.015 | 0.002 | 1.20E-09 | 0.001 | 0.002 | 4.50E-01 |
| rs78082503 | 3 | 116556913 | G | C | -0.015 | 0.003 | 1.10E-08 | 0.004 | 0.002 | 1.30E-02 |
| rs6857629 | 4 | 39684977 | G | A | 0.015 | 0.003 | 1.90E-09 | -0.005 | 0.002 | 1.70E-03 |
| rs2068625 | 4 | 159856739 | T | C | -0.016 | 0.002 | 4.00E-11 | 0.002 | 0.002 | 2.90E-01 |
| rs2220599 | 5 | 7378854 | C | G | -0.016 | 0.002 | 2.40E-12 | 0.001 | 0.001 | 4.50E-01 |
| rs11749912 | 5 | 88065628 | A | G | 0.014 | 0.002 | 4.90E-10 | -0.004 | 0.001 | 6.00E-03 |
| rs1469249 | 5 | 113837198 | G | A | 0.016 | 0.003 | 9.50E-09 | -0.001 | 0.002 | 4.00E-01 |
| rs162894 | 5 | 131611872 | T | G | 0.013 | 0.002 | 1.10E-08 | -0.001 | 0.001 | 3.60E-01 |
| rs56158102 | 5 | 144027803 | C | T | 0.013 | 0.002 | 3.10E-08 | 0.002 | 0.001 | 1.80E-01 |
| rs9477970 | 6 | 19082663 | T | A | -0.016 | 0.003 | 1.00E-08 | -0.002 | 0.002 | 3.30E-01 |
| rs2179517 | 6 | 26198845 | G | C | -0.012 | 0.002 | 1.90E-08 | 0.006 | 0.001 | 4.50E-06 |
| rs9372625 | 6 | 98344031 | G | A | -0.018 | 0.002 | 5.70E-16 | 0.002 | 0.001 | 1.00E-01 |
| rs17789218 | 6 | 100600097 | T | C | -0.015 | 0.003 | 4.20E-09 | 0.001 | 0.002 | 5.60E-01 |
| rs6935828 | 6 | 140811367 | C | T | -0.012 | 0.002 | 4.30E-08 | 0.000 | 0.001 | 9.90E-01 |
| rs55772938 | 7 | 70025113 | A | G | -0.015 | 0.002 | 3.90E-10 | -0.002 | 0.002 | 1.40E-01 |
| rs12706626 | 7 | 124531370 | G | A | -0.013 | 0.002 | 1.40E-08 | -0.001 | 0.001 | 3.20E-01 |
| rs3944151 | 7 | 126970005 | A | G | 0.014 | 0.002 | 3.10E-08 | -0.002 | 0.002 | 2.00E-01 |
| rs2345941 | 7 | 133337635 | A | G | 0.015 | 0.002 | 4.00E-11 | -0.005 | 0.001 | 4.00E-04 |
| rs13262595 | 8 | 143316970 | A | G | -0.016 | 0.002 | 1.20E-12 | 0.000 | 0.001 | 8.00E-01 |
| rs4977839 | 9 | 23355310 | G | A | -0.020 | 0.002 | 4.70E-19 | 0.004 | 0.001 | 1.10E-02 |
| rs113851275 | 9 | 98297220 | G | A | -0.021 | 0.004 | 3.20E-09 | -0.003 | 0.002 | 2.60E-01 |
| rs7020477 | 9 | 116827760 | A | G | 0.014 | 0.002 | 1.20E-08 | 0.002 | 0.002 | 1.10E-01 |
| rs73578186 | 9 | 126334485 | C | T | 0.015 | 0.002 | 2.30E-10 | 0.000 | 0.001 | 7.60E-01 |
| rs2734849 | 11 | 113270160 | A | G | -0.014 | 0.002 | 8.20E-10 | 0.003 | 0.001 | 1.90E-02 |
| rs1448355 | 11 | 131286685 | C | T | -0.015 | 0.002 | 1.30E-10 | 0.004 | 0.001 | 1.40E-02 |
| rs35933007 | 12 | 38929341 | G | A | -0.015 | 0.003 | 9.20E-09 | 0.007 | 0.002 | 1.50E-05 |
| rs1319734 | 12 | 40367517 | A | G | 0.013 | 0.002 | 1.90E-08 | -0.001 | 0.001 | 6.20E-01 |
| rs206965 | 12 | 120856332 | T | C | 0.016 | 0.003 | 9.10E-09 | 0.000 | 0.002 | 7.90E-01 |
| rs12874776 | 13 | 57794345 | C | A | -0.023 | 0.004 | 8.00E-11 | 0.005 | 0.002 | 2.40E-02 |
| rs198262 | 14 | 57283283 | C | T | 0.030 | 0.005 | 3.90E-08 | -0.001 | 0.003 | 8.40E-01 |
| rs166835 | 15 | 47716037 | C | T | 0.013 | 0.002 | 3.40E-09 | -0.002 | 0.001 | 2.40E-01 |
| rs4702 | 15 | 91426560 | G | A | 0.012 | 0.002 | 3.80E-08 | 0.005 | 0.001 | 1.10E-04 |
| rs6498759 | 16 | 18020295 | T | C | 0.013 | 0.002 | 2.70E-08 | 0.000 | 0.002 | 7.40E-01 |
| rs3730399 | 16 | 67229019 | A | G | 0.025 | 0.004 | 3.30E-08 | -0.002 | 0.003 | 4.90E-01 |
| rs4073003 | 17 | 19148305 | A | G | 0.020 | 0.003 | 1.20E-09 | -0.004 | 0.002 | 5.90E-02 |
| rs7209653 | 17 | 19882084 | T | C | 0.016 | 0.002 | 9.10E-11 | 0.000 | 0.002 | 8.40E-01 |
| rs12603813 | 17 | 43196584 | T | C | 0.014 | 0.003 | 1.30E-08 | -0.001 | 0.002 | 3.90E-01 |
| rs66643547 | 17 | 79375315 | C | T | -0.015 | 0.002 | 3.20E-11 | 0.004 | 0.001 | 1.60E-02 |
| rs784256 | 18 | 53398626 | G | A | 0.019 | 0.003 | 6.20E-11 | -0.005 | 0.002 | 5.30E-03 |
| rs6129084 | 20 | 59828857 | A | T | -0.014 | 0.002 | 9.30E-10 | -0.001 | 0.001 | 5.10E-01 |
| rs9608249 | 22 | 24613952 | G | A | 0.017 | 0.003 | 2.20E-08 | 0.000 | 0.002 | 9.30E-01 |
| rs136553 | 22 | 27255675 | C | T | -0.015 | 0.002 | 2.30E-11 | -0.002 | 0.001 | 2.10E-01 |
| rs7288455 | 22 | 39966547 | A | G | 0.013 | 0.002 | 4.00E-09 | -0.002 | 0.001 | 1.30E-01 |

| **Table S33. Betas of the bidirectional MR between smoking and leisure television use** | | | | | | | | | | |
| --- | --- | --- | --- | --- | --- | --- | --- | --- | --- | --- |
| **1) MR of smoking on leisure television use** | | | | | | | | | | |
| SNP | Chr | Position | EA | OA | beta_smk | se_smk | pval_smk | beta_tv | se_tv | pval_tv |
| rs1193237 | 1 | 7526486 | G | C | -0.008 | 0.001 | 2.80E-08 | -0.009 | 0.002 | 1.80E-05 |
| rs4949465 | 1 | 32178489 | T | C | -0.012 | 0.002 | 1.70E-08 | -0.012 | 0.003 | 3.00E-04 |
| rs549845 | 1 | 44076469 | G | A | 0.011 | 0.002 | 8.30E-14 | 0.005 | 0.002 | 3.60E-02 |
| rs1933270 | 1 | 49977965 | T | G | 0.009 | 0.001 | 1.50E-10 | 0.000 | 0.002 | 9.70E-01 |
| rs7528604 | 1 | 66407352 | G | A | 0.010 | 0.001 | 5.70E-12 | 0.007 | 0.002 | 1.80E-03 |
| rs11210229 | 1 | 73860028 | A | G | 0.012 | 0.001 | 2.00E-16 | 0.007 | 0.002 | 2.70E-03 |
| rs7553348 | 1 | 75005067 | G | A | 0.010 | 0.001 | 5.20E-12 | -0.004 | 0.002 | 4.20E-02 |
| rs10922907 | 1 | 91193049 | A | T | 0.010 | 0.001 | 3.00E-13 | 0.011 | 0.002 | 7.40E-07 |
| rs1931263 | 1 | 96175101 | G | T | -0.008 | 0.001 | 4.00E-08 | -0.009 | 0.002 | 6.80E-05 |
| rs7519626 | 1 | 99514554 | C | T | 0.008 | 0.001 | 1.20E-08 | 0.003 | 0.002 | 1.30E-01 |
| rs9435340 | 1 | 107593201 | T | A | 0.008 | 0.001 | 1.20E-08 | 0.013 | 0.002 | 7.10E-09 |
| rs10918701 | 1 | 162090536 | G | A | 0.008 | 0.001 | 2.10E-08 | 0.005 | 0.002 | 4.20E-02 |
| rs2867112 | 2 | 651349 | T | G | 0.015 | 0.002 | 4.80E-15 | 0.005 | 0.003 | 9.10E-02 |
| rs6741228 | 2 | 22548774 | T | C | 0.008 | 0.001 | 1.60E-08 | 0.004 | 0.002 | 9.70E-02 |
| rs62135536 | 2 | 44326028 | C | T | 0.024 | 0.004 | 8.00E-10 | 0.016 | 0.006 | 1.10E-02 |
| rs7569203 | 2 | 45154418 | A | C | -0.011 | 0.002 | 7.40E-13 | 0.000 | 0.002 | 8.90E-01 |
| rs13016665 | 2 | 57995348 | C | A | -0.008 | 0.001 | 1.80E-09 | -0.004 | 0.002 | 9.90E-02 |
| rs4671357 | 2 | 60136176 | T | C | -0.009 | 0.001 | 1.10E-11 | -0.005 | 0.002 | 2.10E-02 |
| rs359243 | 2 | 60475509 | T | C | -0.009 | 0.001 | 9.50E-10 | 0.001 | 0.002 | 7.50E-01 |
| rs2678670 | 2 | 104469564 | A | T | 0.009 | 0.001 | 3.10E-10 | 0.015 | 0.002 | 1.60E-11 |
| rs62155874 | 2 | 105973094 | A | G | -0.017 | 0.002 | 5.20E-16 | -0.010 | 0.003 | 2.70E-03 |
| rs3811038 | 2 | 113240183 | T | C | -0.010 | 0.002 | 8.90E-10 | -0.002 | 0.002 | 5.00E-01 |
| rs2890772 | 2 | 146175106 | G | T | -0.014 | 0.001 | 2.10E-22 | -0.002 | 0.002 | 3.70E-01 |
| rs62175972 | 2 | 161362830 | T | C | 0.022 | 0.004 | 1.70E-08 | 0.020 | 0.006 | 1.10E-03 |
| rs3769949 | 2 | 166199284 | T | A | -0.008 | 0.001 | 2.50E-09 | -0.001 | 0.002 | 6.30E-01 |
| rs13009008 | 2 | 174043233 | A | G | 0.009 | 0.001 | 4.60E-09 | 0.010 | 0.002 | 1.70E-05 |
| rs4473348 | 2 | 182073742 | A | T | -0.010 | 0.002 | 6.40E-11 | -0.012 | 0.002 | 1.20E-06 |
| rs12623702 | 2 | 202885506 | A | G | -0.010 | 0.001 | 7.70E-12 | -0.009 | 0.002 | 5.60E-05 |
| rs6779302 | 3 | 16859710 | G | T | -0.009 | 0.001 | 1.20E-09 | -0.006 | 0.002 | 7.10E-03 |
| rs6778080 | 3 | 49317338 | T | C | 0.011 | 0.002 | 1.30E-12 | 0.009 | 0.002 | 1.70E-04 |
| rs775758 | 3 | 77582005 | A | T | 0.008 | 0.001 | 1.10E-08 | 0.003 | 0.002 | 1.90E-01 |
| rs421983 | 3 | 84892866 | T | C | 0.009 | 0.001 | 3.30E-10 | 0.005 | 0.002 | 2.40E-02 |
| rs326341 | 3 | 107811142 | G | A | 0.009 | 0.001 | 1.20E-11 | 0.003 | 0.002 | 1.60E-01 |
| rs73220544 | 3 | 131074511 | A | C | -0.011 | 0.002 | 1.50E-08 | 0.000 | 0.003 | 9.80E-01 |
| rs9842947 | 3 | 157412246 | C | T | -0.009 | 0.001 | 3.10E-09 | -0.005 | 0.002 | 2.70E-02 |
| rs624833 | 4 | 2881256 | T | G | 0.009 | 0.002 | 6.60E-10 | 0.011 | 0.002 | 5.50E-06 |
| rs61796681 | 4 | 23678196 | A | T | -0.013 | 0.002 | 4.20E-08 | 0.003 | 0.004 | 3.60E-01 |
| rs317021 | 4 | 35418368 | T | A | -0.012 | 0.002 | 1.10E-10 | -0.001 | 0.003 | 7.50E-01 |
| rs72678864 | 4 | 112422145 | G | A | 0.012 | 0.002 | 1.60E-11 | 0.013 | 0.003 | 8.70E-06 |
| rs17576594 | 4 | 147952241 | G | A | 0.011 | 0.002 | 1.70E-12 | 0.002 | 0.002 | 5.00E-01 |
| rs11948770 | 5 | 13246336 | T | C | -0.010 | 0.002 | 4.90E-10 | -0.007 | 0.003 | 6.50E-03 |
| rs71627581 | 5 | 43161351 | G | A | 0.013 | 0.002 | 1.60E-09 | 0.001 | 0.003 | 7.10E-01 |
| rs10052591 | 5 | 50812738 | T | C | 0.008 | 0.001 | 2.10E-09 | 0.004 | 0.002 | 9.90E-02 |
| rs2080870 | 5 | 60388313 | A | T | 0.009 | 0.002 | 4.90E-08 | 0.009 | 0.002 | 5.20E-04 |
| rs4571506 | 5 | 87756918 | C | T | 0.008 | 0.001 | 1.50E-08 | 0.006 | 0.002 | 2.80E-03 |
| rs4957528 | 5 | 106420589 | A | C | -0.010 | 0.002 | 4.20E-09 | -0.011 | 0.003 | 2.10E-05 |
| rs329120 | 5 | 133861756 | C | T | 0.010 | 0.001 | 6.30E-12 | 0.012 | 0.002 | 7.10E-08 |
| rs986391 | 5 | 166993972 | G | A | 0.011 | 0.001 | 9.40E-15 | 0.012 | 0.002 | 1.70E-07 |
| rs13153393 | 5 | 167604213 | A | G | -0.014 | 0.002 | 2.50E-10 | -0.011 | 0.003 | 1.10E-03 |
| rs245774 | 5 | 170530930 | A | G | -0.009 | 0.002 | 7.40E-09 | -0.008 | 0.002 | 1.60E-03 |
| rs6935954 | 6 | 26255451 | A | G | 0.010 | 0.001 | 8.20E-12 | 0.007 | 0.002 | 9.30E-04 |
| rs2254710 | 6 | 37477000 | C | A | 0.009 | 0.002 | 3.50E-08 | -0.001 | 0.003 | 6.00E-01 |
| rs2894808 | 6 | 52861990 | T | A | -0.015 | 0.003 | 3.50E-09 | -0.016 | 0.004 | 1.00E-04 |
| rs12202536 | 6 | 67475273 | A | G | -0.008 | 0.001 | 2.80E-09 | -0.010 | 0.002 | 8.40E-06 |
| rs7766610 | 6 | 111707821 | C | A | 0.013 | 0.002 | 2.20E-12 | 0.002 | 0.003 | 4.90E-01 |
| rs1922018 | 7 | 3560401 | C | T | 0.010 | 0.001 | 3.00E-12 | -0.001 | 0.002 | 6.00E-01 |
| rs10226228 | 7 | 32315613 | A | G | -0.011 | 0.001 | 2.00E-15 | -0.007 | 0.002 | 2.30E-03 |
| rs11768481 | 7 | 96629103 | C | A | 0.009 | 0.001 | 9.90E-10 | 0.007 | 0.002 | 4.30E-03 |
| rs6962772 | 7 | 99081730 | A | G | 0.011 | 0.002 | 7.80E-09 | 0.017 | 0.003 | 1.60E-08 |
| rs10282292 | 7 | 111092478 | C | T | 0.009 | 0.001 | 5.90E-10 | 0.010 | 0.002 | 1.30E-05 |
| rs2401924 | 7 | 115057862 | G | C | 0.011 | 0.001 | 2.70E-14 | 0.000 | 0.002 | 8.20E-01 |
| rs7807019 | 7 | 117543063 | A | G | -0.010 | 0.001 | 6.70E-14 | -0.006 | 0.002 | 3.90E-03 |
| rs6957896 | 7 | 132309592 | C | T | -0.008 | 0.001 | 4.50E-08 | 0.002 | 0.002 | 2.50E-01 |
| rs4731925 | 7 | 132664757 | C | T | -0.008 | 0.001 | 2.60E-08 | -0.009 | 0.002 | 4.70E-05 |
| rs35169606 | 8 | 9604066 | T | G | 0.009 | 0.001 | 1.20E-09 | -0.005 | 0.002 | 3.90E-02 |
| rs11783093 | 8 | 27425349 | C | T | 0.016 | 0.002 | 1.20E-16 | 0.000 | 0.003 | 8.90E-01 |
| rs2062882 | 8 | 91839576 | G | A | -0.008 | 0.001 | 1.10E-08 | -0.004 | 0.002 | 5.70E-02 |
| rs72674867 | 8 | 95578201 | A | T | 0.009 | 0.002 | 3.80E-08 | 0.005 | 0.003 | 3.50E-02 |
| rs4543592 | 9 | 3014254 | T | C | -0.009 | 0.001 | 4.50E-10 | -0.006 | 0.002 | 7.40E-03 |
| rs7039819 | 9 | 82430418 | G | A | 0.009 | 0.001 | 5.10E-10 | 0.010 | 0.002 | 3.10E-06 |
| rs1246265 | 9 | 86761745 | T | C | -0.009 | 0.002 | 4.20E-09 | -0.005 | 0.002 | 2.60E-02 |
| rs1221148 | 9 | 122046875 | C | G | 0.009 | 0.001 | 7.30E-11 | 0.000 | 0.002 | 8.90E-01 |
| rs13296519 | 9 | 128471924 | G | T | -0.010 | 0.001 | 8.10E-12 | -0.008 | 0.002 | 5.00E-04 |
| rs113382419 | 9 | 136463019 | C | A | -0.028 | 0.002 | 3.00E-37 | 0.006 | 0.003 | 9.00E-02 |
| rs11255908 | 10 | 8802912 | T | G | -0.010 | 0.002 | 2.30E-10 | -0.004 | 0.002 | 7.20E-02 |
| rs2675638 | 10 | 63576286 | G | A | 0.008 | 0.001 | 1.30E-09 | 0.010 | 0.002 | 1.80E-06 |
| rs10823968 | 10 | 74738269 | A | T | 0.008 | 0.001 | 2.10E-08 | 0.002 | 0.002 | 3.00E-01 |
| rs17553262 | 10 | 92912773 | A | C | -0.013 | 0.002 | 5.30E-09 | -0.009 | 0.003 | 9.50E-03 |
| rs7077678 | 10 | 104438565 | C | T | 0.009 | 0.001 | 2.60E-09 | 0.004 | 0.002 | 9.60E-02 |
| rs12244388 | 10 | 104640052 | G | A | -0.013 | 0.001 | 1.40E-19 | -0.009 | 0.002 | 5.10E-05 |
| rs3896224 | 10 | 106467853 | A | G | 0.010 | 0.001 | 1.10E-11 | 0.007 | 0.002 | 1.50E-03 |
| rs34866095 | 11 | 16377356 | A | G | -0.009 | 0.002 | 1.20E-08 | 0.000 | 0.002 | 8.40E-01 |
| rs75742406 | 11 | 17070365 | G | A | 0.010 | 0.002 | 1.30E-09 | 0.004 | 0.002 | 1.40E-01 |
| rs17309874 | 11 | 27667236 | G | A | -0.011 | 0.002 | 9.70E-13 | -0.003 | 0.002 | 1.60E-01 |
| rs4391802 | 11 | 28674592 | A | G | 0.010 | 0.002 | 1.40E-11 | 0.005 | 0.002 | 2.40E-02 |
| rs112282219 | 11 | 46632809 | G | A | -0.023 | 0.004 | 3.80E-11 | -0.005 | 0.005 | 3.70E-01 |
| rs9919670 | 11 | 112877304 | G | A | -0.015 | 0.001 | 7.60E-27 | -0.006 | 0.002 | 9.80E-03 |
| rs74086911 | 12 | 50015942 | G | A | 0.015 | 0.003 | 2.10E-08 | 0.008 | 0.004 | 4.30E-02 |
| rs7297175 | 12 | 56473808 | T | C | -0.008 | 0.001 | 6.60E-09 | -0.012 | 0.002 | 1.00E-08 |
| rs10879871 | 12 | 75380511 | T | G | -0.010 | 0.001 | 5.00E-11 | -0.006 | 0.002 | 6.10E-03 |
| rs12831617 | 12 | 84758368 | C | T | -0.009 | 0.002 | 1.90E-08 | -0.003 | 0.003 | 1.80E-01 |
| rs6562474 | 13 | 67332812 | C | G | 0.008 | 0.001 | 1.00E-08 | -0.001 | 0.002 | 7.90E-01 |
| rs7333559 | 13 | 100546450 | G | A | 0.011 | 0.002 | 3.20E-10 | 0.005 | 0.003 | 4.30E-02 |
| rs860326 | 14 | 57342912 | C | T | 0.008 | 0.001 | 2.70E-09 | 0.010 | 0.002 | 8.60E-06 |
| rs7155595 | 14 | 77502546 | A | C | -0.009 | 0.001 | 2.50E-09 | -0.008 | 0.002 | 1.10E-03 |
| rs3742365 | 14 | 104198251 | T | C | -0.011 | 0.001 | 2.50E-14 | 0.003 | 0.002 | 1.90E-01 |
| rs35175834 | 15 | 47680815 | G | A | -0.016 | 0.002 | 4.60E-22 | -0.002 | 0.003 | 4.10E-01 |
| rs28485305 | 15 | 74044197 | C | T | 0.008 | 0.001 | 2.60E-08 | 0.002 | 0.002 | 3.00E-01 |
| rs8042849 | 15 | 78817929 | C | T | 0.019 | 0.001 | 1.80E-39 | -0.003 | 0.002 | 2.70E-01 |
| rs8042134 | 15 | 97514404 | T | G | -0.010 | 0.001 | 1.30E-12 | 0.001 | 0.002 | 7.60E-01 |
| rs6598539 | 15 | 99204483 | T | C | -0.008 | 0.001 | 4.50E-09 | -0.006 | 0.002 | 5.10E-03 |
| rs11861214 | 16 | 746611 | G | T | 0.009 | 0.002 | 2.00E-08 | 0.008 | 0.003 | 4.30E-03 |
| rs12708665 | 16 | 24728227 | A | G | -0.009 | 0.002 | 3.50E-09 | -0.010 | 0.002 | 3.20E-05 |
| rs57611503 | 16 | 31165795 | G | A | 0.008 | 0.001 | 4.00E-08 | 0.007 | 0.002 | 1.90E-03 |
| rs889398 | 16 | 69556715 | C | T | 0.009 | 0.001 | 6.30E-11 | 0.002 | 0.002 | 4.30E-01 |
| rs60952428 | 16 | 75640521 | T | C | 0.013 | 0.002 | 3.00E-08 | 0.010 | 0.004 | 1.10E-02 |
| rs1050847 | 16 | 87443734 | C | T | 0.008 | 0.001 | 1.40E-08 | 0.005 | 0.002 | 3.50E-02 |
| rs369230 | 16 | 89645437 | G | T | -0.009 | 0.002 | 1.80E-09 | -0.006 | 0.002 | 9.40E-03 |
| rs8614 | 17 | 27588806 | C | A | -0.011 | 0.002 | 1.80E-10 | 0.001 | 0.003 | 6.90E-01 |
| rs732083 | 17 | 37834367 | G | A | 0.008 | 0.001 | 1.50E-08 | 0.007 | 0.002 | 3.10E-03 |
| rs9904288 | 17 | 47031973 | T | C | 0.008 | 0.002 | 3.10E-08 | 0.006 | 0.002 | 7.30E-03 |
| rs67596067 | 17 | 50333733 | G | A | -0.009 | 0.001 | 1.20E-09 | -0.009 | 0.002 | 6.90E-05 |
| rs12967855 | 18 | 35138245 | A | G | 0.008 | 0.001 | 3.10E-08 | 0.017 | 0.002 | 2.50E-13 |
| rs62098013 | 18 | 50863861 | G | A | -0.009 | 0.001 | 4.10E-09 | -0.006 | 0.002 | 9.10E-03 |
| rs71367545 | 18 | 77576337 | G | A | -0.010 | 0.002 | 1.40E-09 | -0.007 | 0.003 | 1.00E-02 |
| rs76608582 | 19 | 4474725 | C | A | 0.022 | 0.003 | 3.20E-10 | 0.023 | 0.005 | 1.10E-05 |
| rs35343344 | 19 | 18471610 | C | A | 0.009 | 0.002 | 8.80E-09 | 0.005 | 0.002 | 7.00E-02 |
| rs4814873 | 20 | 19616429 | C | T | 0.010 | 0.002 | 2.90E-09 | 0.009 | 0.003 | 4.80E-04 |
| rs6119897 | 20 | 31145415 | G | A | -0.013 | 0.002 | 3.60E-15 | -0.006 | 0.003 | 1.80E-02 |
| rs12481282 | 20 | 44761377 | G | C | -0.009 | 0.002 | 7.80E-09 | -0.001 | 0.002 | 6.20E-01 |
| rs348809 | 20 | 59032097 | A | G | -0.008 | 0.001 | 1.30E-08 | -0.006 | 0.002 | 5.40E-03 |
| rs6011779 | 20 | 61984317 | C | T | 0.019 | 0.002 | 2.30E-27 | 0.003 | 0.003 | 3.20E-01 |
| rs147412694 | 21 | 40702786 | G | A | -0.012 | 0.002 | 2.90E-09 | 0.010 | 0.003 | 1.20E-03 |
| rs2838834 | 21 | 46665208 | C | T | -0.009 | 0.002 | 6.30E-10 | -0.001 | 0.002 | 5.30E-01 |
| rs136233 | 22 | 31212410 | A | G | -0.010 | 0.002 | 1.80E-08 | -0.010 | 0.003 | 4.00E-04 |
| rs202645 | 22 | 41798520 | A | G | -0.010 | 0.002 | 3.90E-09 | 0.005 | 0.003 | 6.50E-02 |
| **2) MR of leisure television use on smoking** | | | | | | | | | | |
| SNP | Chr | Position | EA | OA | beta_tv | se_tv | pval_tv | beta_smk | se_smk | pval_smk |
| rs11810109 | 1 | 44186812 | A | T | 0.016 | 0.002 | 5.40E-12 | 0.010 | 0.002 | 5.10E-11 |
| rs112632642 | 1 | 51115634 | G | A | -0.024 | 0.004 | 3.60E-08 | 0.002 | 0.003 | 4.40E-01 |
| rs6670915 | 1 | 61109968 | C | T | 0.014 | 0.002 | 2.40E-10 | 0.001 | 0.001 | 3.60E-01 |
| rs12725114 | 1 | 62432641 | G | A | 0.015 | 0.003 | 1.60E-08 | -0.001 | 0.002 | 4.70E-01 |
| rs984409 | 1 | 67020440 | G | A | -0.015 | 0.002 | 4.10E-11 | -0.002 | 0.001 | 1.80E-01 |
| rs71658797 | 1 | 77967507 | T | A | -0.020 | 0.003 | 6.30E-10 | -0.007 | 0.002 | 5.80E-04 |
| rs114328297 | 1 | 91190854 | T | G | 0.014 | 0.003 | 4.40E-08 | 0.010 | 0.002 | 1.30E-08 |
| rs631130 | 1 | 96043010 | T | A | -0.014 | 0.002 | 5.00E-09 | 0.000 | 0.002 | 8.10E-01 |
| rs148648822 | 1 | 97815048 | G | T | -0.018 | 0.003 | 4.20E-09 | -0.003 | 0.002 | 1.00E-01 |
| rs17379561 | 1 | 98340139 | A | T | -0.025 | 0.003 | 1.10E-16 | -0.006 | 0.002 | 5.30E-03 |
| rs55909997 | 1 | 107581039 | G | A | -0.014 | 0.002 | 2.40E-09 | -0.008 | 0.001 | 1.10E-07 |
| rs1278847 | 1 | 110037483 | C | A | 0.016 | 0.002 | 1.20E-11 | 0.002 | 0.002 | 2.80E-01 |
| rs141425667 | 1 | 114930465 | G | A | -0.024 | 0.004 | 4.00E-08 | -0.002 | 0.003 | 4.40E-01 |
| rs4845364 | 1 | 154141908 | A | G | -0.015 | 0.002 | 1.30E-12 | 0.003 | 0.001 | 1.60E-02 |
| rs6673341 | 1 | 184655649 | T | G | -0.015 | 0.002 | 2.20E-11 | -0.003 | 0.001 | 3.30E-02 |
| rs72725224 | 1 | 188115858 | A | C | -0.046 | 0.008 | 3.70E-09 | -0.001 | 0.005 | 8.20E-01 |
| rs10737620 | 1 | 193057971 | T | A | 0.014 | 0.002 | 2.60E-09 | 0.001 | 0.002 | 6.20E-01 |
| rs11896615 | 2 | 17407744 | G | A | 0.017 | 0.003 | 3.10E-08 | 0.000 | 0.002 | 9.90E-01 |
| rs72781699 | 2 | 24277709 | G | A | -0.019 | 0.003 | 3.00E-12 | -0.003 | 0.002 | 4.40E-02 |
| rs12105701 | 2 | 41737200 | C | T | -0.013 | 0.002 | 5.40E-09 | -0.001 | 0.001 | 6.50E-01 |
| rs7564130 | 2 | 50606642 | T | C | -0.015 | 0.002 | 2.80E-11 | -0.005 | 0.001 | 5.10E-04 |
| rs11125515 | 2 | 53886799 | C | A | -0.014 | 0.002 | 2.20E-08 | -0.001 | 0.002 | 3.60E-01 |
| rs12475168 | 2 | 58992704 | G | T | -0.018 | 0.003 | 1.70E-08 | -0.002 | 0.002 | 4.10E-01 |
| rs183566425 | 2 | 59325356 | A | T | 0.046 | 0.008 | 1.90E-08 | 0.003 | 0.005 | 5.90E-01 |
| rs10189857 | 2 | 60713235 | A | G | -0.020 | 0.002 | 6.20E-21 | -0.003 | 0.001 | 1.80E-02 |
| rs62641636 | 2 | 68425427 | A | G | 0.014 | 0.002 | 7.00E-10 | 0.000 | 0.002 | 8.90E-01 |
| rs59210467 | 2 | 97097291 | C | T | -0.013 | 0.002 | 1.00E-08 | 0.001 | 0.002 | 6.70E-01 |
| rs11689199 | 2 | 100820421 | A | G | 0.018 | 0.002 | 5.50E-17 | 0.003 | 0.001 | 1.60E-02 |
| rs2916251 | 2 | 104447052 | G | T | -0.017 | 0.002 | 1.50E-13 | -0.009 | 0.001 | 7.30E-10 |
| rs1451533 | 2 | 105466005 | G | A | -0.016 | 0.002 | 1.10E-10 | -0.001 | 0.002 | 7.30E-01 |
| rs2971640 | 2 | 107624618 | G | A | -0.013 | 0.002 | 4.20E-09 | -0.001 | 0.001 | 3.50E-01 |
| rs12476388 | 2 | 116330983 | C | T | 0.013 | 0.002 | 2.10E-08 | 0.001 | 0.002 | 4.30E-01 |
| rs374722 | 2 | 147839830 | G | A | 0.024 | 0.003 | 5.50E-16 | 0.000 | 0.002 | 8.80E-01 |
| rs3754970 | 2 | 162091836 | T | C | -0.015 | 0.002 | 4.80E-12 | -0.006 | 0.001 | 1.80E-05 |
| rs1913808 | 2 | 162804678 | G | A | 0.015 | 0.002 | 3.90E-10 | 0.003 | 0.002 | 4.90E-02 |
| rs2060199 | 2 | 166196681 | T | A | 0.012 | 0.002 | 2.40E-08 | -0.001 | 0.001 | 3.30E-01 |
| rs263771 | 2 | 185921692 | C | A | -0.017 | 0.003 | 3.10E-11 | -0.009 | 0.002 | 8.40E-08 |
| rs4577309 | 2 | 191288833 | A | G | 0.016 | 0.002 | 1.60E-13 | 0.001 | 0.001 | 3.50E-01 |
| rs34224261 | 2 | 193855452 | G | C | -0.018 | 0.002 | 4.60E-16 | 0.001 | 0.001 | 6.00E-01 |
| rs4675246 | 2 | 202864487 | G | T | -0.015 | 0.003 | 2.90E-08 | -0.006 | 0.002 | 2.70E-04 |
| rs13029509 | 2 | 215374209 | G | A | -0.018 | 0.002 | 2.00E-17 | -0.002 | 0.001 | 1.60E-01 |
| rs10932837 | 2 | 221044785 | C | T | -0.013 | 0.002 | 1.30E-09 | 0.000 | 0.001 | 7.80E-01 |
| rs4973576 | 2 | 233806771 | C | A | -0.015 | 0.002 | 1.00E-09 | 0.002 | 0.002 | 2.20E-01 |
| rs138256022 | 3 | 11603090 | C | T | -0.031 | 0.006 | 2.90E-08 | -0.006 | 0.004 | 1.00E-01 |
| rs9834970 | 3 | 36856030 | T | C | 0.013 | 0.002 | 3.30E-09 | 0.002 | 0.001 | 7.10E-02 |
| rs1352890 | 3 | 49652156 | C | T | -0.012 | 0.002 | 1.30E-08 | 0.000 | 0.001 | 1.00E+00 |
| rs3796386 | 3 | 49899795 | G | A | -0.026 | 0.002 | 3.20E-33 | -0.006 | 0.001 | 3.80E-05 |
| rs11917871 | 3 | 54233476 | T | C | -0.017 | 0.002 | 4.50E-14 | 0.001 | 0.001 | 5.30E-01 |
| rs11130793 | 3 | 60884659 | C | T | 0.013 | 0.002 | 5.00E-09 | 0.002 | 0.001 | 1.30E-01 |
| rs11714337 | 3 | 71582521 | G | A | 0.014 | 0.002 | 4.70E-11 | 0.005 | 0.001 | 7.60E-04 |
| rs12496084 | 3 | 75174777 | A | G | -0.014 | 0.003 | 4.60E-08 | -0.002 | 0.002 | 1.90E-01 |
| rs6797840 | 3 | 85656569 | A | C | -0.016 | 0.002 | 1.70E-13 | -0.004 | 0.001 | 7.30E-03 |
| rs56398417 | 3 | 88024986 | C | T | 0.014 | 0.002 | 5.90E-10 | 0.006 | 0.001 | 3.90E-05 |
| rs2034768 | 3 | 93986371 | A | G | 0.015 | 0.002 | 8.60E-12 | 0.006 | 0.001 | 4.50E-06 |
| rs12632778 | 3 | 106452359 | T | G | -0.028 | 0.005 | 3.90E-08 | -0.001 | 0.003 | 8.30E-01 |
| rs9867121 | 3 | 114631548 | C | A | 0.019 | 0.003 | 3.90E-12 | 0.002 | 0.002 | 2.30E-01 |
| rs12491503 | 3 | 165706855 | G | A | -0.014 | 0.002 | 5.50E-10 | -0.003 | 0.001 | 5.50E-02 |
| rs114600294 | 3 | 181419367 | G | C | -0.016 | 0.003 | 7.90E-10 | 0.002 | 0.002 | 3.10E-01 |
| rs66852340 | 4 | 3311070 | C | T | -0.018 | 0.003 | 7.90E-12 | -0.005 | 0.002 | 1.20E-03 |
| rs7700107 | 4 | 17880416 | A | C | -0.021 | 0.003 | 1.90E-11 | 0.001 | 0.002 | 7.80E-01 |
| rs34811474 | 4 | 25408838 | G | A | 0.015 | 0.003 | 2.30E-09 | 0.005 | 0.002 | 3.20E-03 |
| rs77215114 | 4 | 45187658 | A | T | 0.024 | 0.004 | 1.50E-08 | -0.005 | 0.003 | 5.50E-02 |
| rs28971796 | 4 | 49151982 | A | G | 0.015 | 0.002 | 2.10E-09 | 0.004 | 0.002 | 6.30E-03 |
| rs6850494 | 4 | 82291771 | A | C | -0.014 | 0.002 | 1.10E-10 | -0.004 | 0.001 | 9.20E-03 |
| rs13107325 | 4 | 103188709 | C | T | -0.029 | 0.004 | 1.50E-12 | 0.004 | 0.003 | 1.50E-01 |
| rs62332760 | 4 | 106135316 | G | T | -0.014 | 0.002 | 8.00E-11 | 0.005 | 0.001 | 5.70E-04 |
| rs7693703 | 4 | 118357405 | G | A | 0.023 | 0.004 | 2.70E-09 | 0.001 | 0.002 | 5.80E-01 |
| rs4334769 | 4 | 130245233 | G | T | 0.012 | 0.002 | 2.10E-08 | 0.001 | 0.001 | 7.00E-01 |
| rs6825241 | 4 | 152543783 | C | A | -0.017 | 0.002 | 4.90E-15 | 0.000 | 0.001 | 7.40E-01 |
| rs7693082 | 4 | 159857819 | G | C | 0.015 | 0.002 | 1.70E-10 | 0.002 | 0.002 | 2.80E-01 |
| rs10940659 | 5 | 59360341 | A | G | -0.013 | 0.002 | 1.50E-08 | 0.000 | 0.001 | 9.70E-01 |
| rs2409838 | 5 | 60204410 | A | T | 0.017 | 0.002 | 3.40E-15 | 0.007 | 0.001 | 2.10E-06 |
| rs262890 | 5 | 62930015 | A | G | -0.019 | 0.002 | 3.20E-15 | -0.005 | 0.002 | 2.10E-03 |
| rs7716447 | 5 | 88800331 | A | G | -0.013 | 0.002 | 3.60E-09 | -0.007 | 0.001 | 1.10E-06 |
| rs1031423 | 5 | 93276883 | T | C | -0.019 | 0.003 | 1.80E-12 | -0.004 | 0.002 | 1.70E-02 |
| rs57585211 | 5 | 107197725 | T | G | -0.017 | 0.003 | 4.30E-09 | -0.002 | 0.002 | 3.30E-01 |
| rs9326862 | 5 | 112054547 | A | T | 0.012 | 0.002 | 2.60E-08 | 0.006 | 0.001 | 4.20E-05 |
| rs10041724 | 5 | 124273520 | T | C | 0.018 | 0.003 | 3.90E-11 | 0.005 | 0.002 | 2.80E-03 |
| rs10057545 | 5 | 138431008 | G | A | -0.014 | 0.002 | 2.00E-10 | 0.000 | 0.001 | 7.70E-01 |
| rs62379379 | 5 | 141082015 | G | T | -0.026 | 0.004 | 7.80E-10 | -0.004 | 0.003 | 1.70E-01 |
| rs10054327 | 5 | 147871137 | G | A | 0.017 | 0.002 | 3.40E-15 | 0.002 | 0.001 | 2.00E-01 |
| rs42210 | 5 | 166408788 | G | C | -0.014 | 0.002 | 7.30E-09 | -0.006 | 0.002 | 9.00E-05 |
| rs2336895 | 5 | 166990695 | G | A | 0.012 | 0.002 | 4.60E-08 | 0.011 | 0.001 | 2.60E-14 |
| rs72828890 | 5 | 167806836 | C | T | 0.019 | 0.003 | 4.90E-09 | 0.003 | 0.002 | 1.10E-01 |
| rs255292 | 5 | 172580866 | C | A | -0.012 | 0.002 | 1.70E-08 | 0.002 | 0.001 | 1.40E-01 |
| rs72834698 | 6 | 26176517 | G | A | 0.023 | 0.003 | 2.70E-13 | 0.006 | 0.002 | 1.30E-03 |
| rs3135044 | 6 | 31592820 | C | T | -0.015 | 0.002 | 8.70E-11 | -0.001 | 0.001 | 4.30E-01 |
| rs10947452 | 6 | 33803752 | T | C | 0.014 | 0.002 | 6.40E-10 | 0.004 | 0.001 | 4.30E-03 |
| rs9471333 | 6 | 40362023 | C | T | 0.013 | 0.002 | 1.50E-09 | 0.001 | 0.001 | 6.10E-01 |
| rs4523073 | 6 | 67550288 | A | G | -0.014 | 0.002 | 5.00E-10 | -0.008 | 0.001 | 3.80E-09 |
| rs6905544 | 6 | 98411631 | A | G | -0.019 | 0.002 | 8.50E-18 | -0.002 | 0.001 | 9.70E-02 |
| rs17789218 | 6 | 100600097 | T | C | 0.019 | 0.003 | 1.40E-13 | 0.001 | 0.002 | 5.60E-01 |
| rs78394231 | 6 | 107649123 | T | C | -0.021 | 0.004 | 9.70E-09 | -0.002 | 0.002 | 3.80E-01 |
| rs2184364 | 6 | 143183596 | A | G | 0.016 | 0.003 | 3.00E-09 | 0.004 | 0.002 | 1.40E-02 |
| rs9718104 | 6 | 166170539 | T | G | -0.041 | 0.005 | 9.30E-19 | 0.001 | 0.003 | 7.50E-01 |
| rs17568389 | 7 | 8091876 | T | A | 0.015 | 0.002 | 8.60E-13 | 0.001 | 0.001 | 5.20E-01 |
| rs10234444 | 7 | 41828527 | G | A | 0.016 | 0.003 | 2.90E-08 | 0.001 | 0.002 | 7.00E-01 |
| rs6973656 | 7 | 77422583 | A | G | -0.013 | 0.002 | 1.00E-09 | 0.000 | 0.001 | 8.90E-01 |
| rs973734 | 7 | 89387578 | C | A | 0.017 | 0.003 | 1.50E-08 | 0.004 | 0.002 | 5.60E-02 |
| rs3137 | 7 | 99130875 | G | A | 0.017 | 0.003 | 1.40E-08 | 0.011 | 0.002 | 9.90E-09 |
| rs2694151 | 7 | 101902180 | C | T | 0.026 | 0.005 | 2.30E-08 | -0.003 | 0.003 | 3.70E-01 |
| rs62471080 | 7 | 109128060 | G | C | -0.013 | 0.002 | 3.70E-09 | -0.001 | 0.001 | 4.30E-01 |
| rs7788008 | 7 | 112972483 | G | A | 0.013 | 0.002 | 5.90E-09 | 0.001 | 0.001 | 4.10E-01 |
| rs10246289 | 7 | 115544763 | A | G | 0.019 | 0.003 | 1.70E-08 | 0.001 | 0.002 | 5.10E-01 |
| rs11763734 | 7 | 126400910 | A | C | -0.012 | 0.002 | 8.20E-09 | -0.004 | 0.001 | 2.20E-03 |
| rs7834121 | 8 | 10770255 | G | T | -0.014 | 0.002 | 1.10E-10 | 0.007 | 0.001 | 1.30E-06 |
| rs1421334 | 8 | 30865733 | A | C | 0.017 | 0.002 | 2.20E-15 | 0.003 | 0.001 | 5.70E-02 |
| rs6996198 | 8 | 65463442 | C | T | -0.016 | 0.003 | 3.50E-08 | 0.001 | 0.002 | 6.10E-01 |
| rs6472942 | 8 | 76827190 | T | C | -0.013 | 0.002 | 1.80E-09 | -0.005 | 0.001 | 1.80E-04 |
| rs11985394 | 8 | 92667521 | T | A | 0.012 | 0.002 | 1.30E-08 | 0.005 | 0.001 | 9.80E-04 |
| rs72671494 | 8 | 93195457 | T | C | -0.017 | 0.003 | 4.50E-08 | -0.006 | 0.002 | 4.70E-03 |
| rs12541615 | 8 | 118870151 | T | C | -0.018 | 0.003 | 3.90E-10 | -0.004 | 0.002 | 2.00E-02 |
| rs2717559 | 8 | 143882420 | A | G | 0.012 | 0.002 | 1.50E-08 | 0.002 | 0.001 | 2.20E-01 |
| rs2616830 | 9 | 1721385 | G | A | 0.016 | 0.002 | 2.90E-14 | 0.001 | 0.001 | 3.40E-01 |
| rs2291681 | 9 | 14790841 | G | T | 0.013 | 0.002 | 7.90E-09 | 0.001 | 0.001 | 3.30E-01 |
| rs34864022 | 9 | 22609110 | A | G | -0.026 | 0.004 | 1.40E-09 | -0.008 | 0.003 | 3.80E-03 |
| rs12554512 | 9 | 23352293 | T | C | 0.021 | 0.002 | 3.80E-21 | 0.004 | 0.001 | 8.50E-03 |
| rs2791574 | 9 | 82212094 | T | G | -0.013 | 0.002 | 3.00E-08 | -0.003 | 0.001 | 7.70E-02 |
| rs870151 | 9 | 92230747 | T | A | -0.016 | 0.002 | 8.80E-13 | -0.002 | 0.001 | 9.40E-02 |
| rs2787374 | 9 | 103054951 | T | C | 0.012 | 0.002 | 1.20E-08 | 0.003 | 0.001 | 3.20E-02 |
| rs10739499 | 9 | 120514261 | C | G | 0.014 | 0.002 | 1.50E-09 | 0.008 | 0.001 | 2.30E-07 |
| rs7043521 | 9 | 124602779 | A | T | 0.014 | 0.002 | 6.50E-11 | 0.005 | 0.001 | 9.70E-04 |
| rs12342024 | 9 | 126134232 | G | T | -0.021 | 0.003 | 1.70E-09 | -0.005 | 0.002 | 1.50E-02 |
| rs4382592 | 9 | 134870755 | T | G | 0.014 | 0.002 | 6.70E-09 | 0.007 | 0.002 | 1.10E-06 |
| rs2073869 | 9 | 135763816 | C | T | 0.019 | 0.003 | 1.50E-10 | 0.004 | 0.002 | 3.00E-02 |
| 9:139926402_T_C | 9 | 139926402 | T | C | -0.013 | 0.002 | 2.00E-08 | 0.003 | 0.001 | 7.50E-02 |
| rs1623025 | 10 | 11164813 | G | C | -0.012 | 0.002 | 1.40E-08 | -0.002 | 0.001 | 1.20E-01 |
| rs1243182 | 10 | 21916728 | C | T | -0.019 | 0.002 | 2.00E-15 | -0.006 | 0.001 | 1.30E-04 |
| rs2045147 | 10 | 56660437 | A | G | 0.013 | 0.002 | 5.90E-09 | 0.007 | 0.001 | 9.60E-08 |
| rs10994943 | 10 | 63591413 | T | G | 0.013 | 0.002 | 3.10E-09 | 0.008 | 0.001 | 1.50E-08 |
| rs11201422 | 10 | 86924483 | T | C | 0.013 | 0.002 | 3.40E-08 | 0.002 | 0.001 | 2.40E-01 |
| rs10786658 | 10 | 103737193 | A | T | -0.014 | 0.002 | 4.60E-11 | -0.005 | 0.001 | 3.70E-04 |
| rs7089973 | 10 | 116569565 | C | A | -0.013 | 0.002 | 2.40E-09 | -0.003 | 0.001 | 3.50E-02 |
| rs11245482 | 10 | 126733546 | T | C | -0.013 | 0.002 | 2.60E-09 | -0.003 | 0.001 | 2.20E-02 |
| rs17727474 | 10 | 127182959 | C | T | 0.018 | 0.003 | 3.10E-09 | 0.003 | 0.002 | 1.00E-01 |
| rs12289262 | 11 | 12894758 | C | T | -0.014 | 0.002 | 2.00E-08 | 0.000 | 0.002 | 9.30E-01 |
| rs801733 | 11 | 65934549 | A | C | 0.017 | 0.002 | 7.30E-14 | 0.003 | 0.001 | 5.30E-02 |
| rs116175338 | 11 | 72467875 | G | T | -0.014 | 0.003 | 1.40E-08 | -0.006 | 0.002 | 3.00E-04 |
| rs12272012 | 11 | 84861286 | G | C | -0.030 | 0.005 | 1.90E-08 | 0.000 | 0.003 | 9.10E-01 |
| rs11020045 | 11 | 92487907 | A | C | -0.013 | 0.002 | 1.60E-08 | -0.004 | 0.001 | 3.20E-03 |
| rs17207890 | 11 | 95490754 | G | A | 0.016 | 0.002 | 6.70E-12 | 0.005 | 0.001 | 1.80E-03 |
| rs10791546 | 11 | 101830126 | C | T | 0.012 | 0.002 | 4.10E-08 | 0.001 | 0.001 | 6.80E-01 |
| rs328277 | 11 | 110414195 | C | A | 0.012 | 0.002 | 4.50E-08 | 0.000 | 0.001 | 8.10E-01 |
| rs648044 | 11 | 114030799 | A | G | -0.014 | 0.002 | 1.00E-09 | -0.005 | 0.001 | 1.30E-03 |
| rs11218575 | 11 | 122154576 | C | T | 0.015 | 0.002 | 2.10E-12 | 0.002 | 0.001 | 1.50E-01 |
| rs74802478 | 11 | 131966907 | G | A | 0.017 | 0.003 | 3.50E-09 | 0.002 | 0.002 | 3.10E-01 |
| rs4937842 | 11 | 133734542 | G | C | -0.013 | 0.002 | 1.40E-08 | 0.001 | 0.001 | 4.50E-01 |
| rs10772643 | 12 | 13415288 | C | T | 0.025 | 0.003 | 1.30E-12 | 0.005 | 0.002 | 2.90E-02 |
| rs74996610 | 12 | 24075007 | C | G | -0.031 | 0.005 | 4.10E-09 | 0.002 | 0.003 | 6.50E-01 |
| rs10771746 | 12 | 30791864 | C | T | -0.014 | 0.002 | 2.50E-09 | 0.001 | 0.002 | 5.10E-01 |
| rs10876864 | 12 | 56401085 | G | A | -0.013 | 0.002 | 1.00E-09 | -0.007 | 0.001 | 1.50E-07 |
| rs8756 | 12 | 66359752 | C | A | -0.013 | 0.002 | 5.30E-10 | -0.004 | 0.001 | 1.30E-03 |
| rs11179838 | 12 | 74321123 | C | G | 0.013 | 0.002 | 2.10E-08 | 0.003 | 0.001 | 6.90E-02 |
| rs2164744 | 12 | 109872039 | T | C | -0.013 | 0.002 | 1.10E-08 | -0.004 | 0.001 | 6.50E-03 |
| rs2173650 | 12 | 117525235 | G | T | 0.018 | 0.003 | 4.60E-09 | 0.006 | 0.002 | 1.40E-03 |
| rs1790135 | 12 | 123669235 | C | T | -0.014 | 0.003 | 2.60E-08 | -0.001 | 0.002 | 6.40E-01 |
| rs9563168 | 13 | 54247827 | G | A | 0.018 | 0.003 | 4.30E-11 | 0.003 | 0.002 | 1.10E-01 |
| rs9569734 | 13 | 58319476 | A | G | 0.019 | 0.003 | 3.40E-10 | 0.002 | 0.002 | 2.10E-01 |
| rs9528023 | 13 | 60226991 | C | T | 0.013 | 0.002 | 3.80E-08 | 0.000 | 0.002 | 8.80E-01 |
| rs56858768 | 13 | 86511730 | G | A | -0.015 | 0.002 | 3.40E-10 | -0.001 | 0.002 | 3.90E-01 |
| rs553397860 | 13 | 99051118 | A | G | 0.017 | 0.003 | 8.90E-09 | 0.000 | 0.002 | 9.30E-01 |
| rs7991062 | 13 | 100713194 | C | G | -0.018 | 0.002 | 8.80E-15 | -0.007 | 0.001 | 4.00E-07 |
| rs11629120 | 14 | 23458457 | T | C | -0.013 | 0.002 | 1.10E-08 | -0.001 | 0.001 | 3.80E-01 |
| rs405797 | 14 | 26972834 | T | A | -0.015 | 0.003 | 2.80E-09 | -0.004 | 0.002 | 8.00E-03 |
| rs1022785 | 14 | 29571653 | G | A | 0.018 | 0.003 | 7.10E-09 | 0.002 | 0.002 | 4.30E-01 |
| rs10145592 | 14 | 94287215 | C | G | -0.015 | 0.002 | 1.80E-11 | 0.001 | 0.001 | 6.20E-01 |
| rs7157001 | 14 | 99749484 | A | G | -0.014 | 0.002 | 2.30E-08 | -0.002 | 0.002 | 2.90E-01 |
| rs8043253 | 15 | 41503730 | C | T | -0.012 | 0.002 | 3.40E-08 | 0.003 | 0.001 | 7.20E-02 |
| rs2460 | 15 | 53073084 | G | A | -0.015 | 0.002 | 5.00E-10 | -0.003 | 0.002 | 4.00E-02 |
| rs4775373 | 15 | 61476936 | T | C | 0.013 | 0.002 | 4.70E-09 | 0.001 | 0.001 | 3.60E-01 |
| rs61331678 | 15 | 77995949 | G | C | 0.015 | 0.002 | 6.10E-12 | 0.003 | 0.001 | 4.10E-02 |
| rs35574015 | 16 | 15146061 | T | C | -0.013 | 0.002 | 3.00E-08 | -0.003 | 0.002 | 3.40E-02 |
| rs7189927 | 16 | 28913787 | T | C | 0.015 | 0.002 | 3.40E-11 | 0.003 | 0.001 | 2.40E-02 |
| rs749671 | 16 | 31088347 | G | A | 0.016 | 0.002 | 2.70E-12 | 0.007 | 0.001 | 4.90E-06 |
| rs7184800 | 16 | 53509131 | G | A | 0.017 | 0.002 | 8.20E-13 | 0.004 | 0.002 | 1.60E-02 |
| rs2447098 | 17 | 2277720 | C | A | -0.015 | 0.002 | 6.80E-12 | -0.004 | 0.001 | 1.70E-03 |
| rs11654952 | 17 | 4813799 | T | G | -0.017 | 0.003 | 4.30E-08 | -0.001 | 0.002 | 7.60E-01 |
| rs9902312 | 17 | 65070304 | T | C | 0.015 | 0.002 | 4.50E-11 | 0.000 | 0.001 | 8.30E-01 |
| rs11657730 | 17 | 79372489 | C | T | 0.013 | 0.002 | 1.10E-08 | 0.003 | 0.001 | 1.70E-02 |
| rs303753 | 18 | 21074922 | G | A | -0.014 | 0.002 | 2.70E-10 | 0.000 | 0.001 | 9.20E-01 |
| rs11081851 | 18 | 31783262 | G | A | -0.013 | 0.002 | 1.00E-08 | -0.005 | 0.001 | 8.20E-04 |
| rs9964724 | 18 | 35159124 | C | T | 0.018 | 0.002 | 3.30E-14 | 0.007 | 0.001 | 6.50E-07 |
| rs1156541 | 18 | 39952989 | C | T | 0.014 | 0.003 | 2.80E-08 | 0.006 | 0.002 | 3.40E-04 |
| rs148544378 | 18 | 40323567 | C | T | 0.053 | 0.008 | 2.20E-12 | 0.016 | 0.005 | 1.10E-03 |
| rs17512836 | 18 | 53194961 | T | C | 0.042 | 0.007 | 7.00E-10 | 0.002 | 0.004 | 6.90E-01 |
| rs7248205 | 19 | 10770305 | C | T | 0.014 | 0.002 | 3.40E-10 | -0.002 | 0.001 | 1.40E-01 |
| rs111901094 | 19 | 19513570 | G | T | -0.017 | 0.003 | 2.00E-09 | -0.005 | 0.002 | 1.00E-02 |
| rs6131281 | 20 | 11891724 | C | T | 0.016 | 0.002 | 3.10E-13 | -0.001 | 0.001 | 7.20E-01 |
| rs6141814 | 20 | 31368960 | C | A | -0.014 | 0.002 | 1.30E-09 | -0.007 | 0.001 | 1.50E-06 |
| rs4810312 | 20 | 39968707 | C | G | -0.013 | 0.002 | 1.20E-08 | -0.001 | 0.001 | 3.10E-01 |
| rs6124476 | 20 | 41202830 | T | C | 0.012 | 0.002 | 1.80E-08 | 0.001 | 0.001 | 7.10E-01 |
| rs6031440 | 20 | 42809317 | A | T | 0.012 | 0.002 | 1.80E-08 | 0.001 | 0.001 | 6.40E-01 |
| rs55700114 | 20 | 43717080 | G | A | -0.014 | 0.002 | 2.10E-09 | -0.003 | 0.002 | 3.00E-02 |
| rs6125906 | 20 | 48729803 | C | A | -0.021 | 0.004 | 3.90E-08 | 0.000 | 0.002 | 9.10E-01 |
| rs56103247 | 20 | 62483184 | C | T | 0.030 | 0.005 | 3.80E-10 | 0.017 | 0.003 | 4.60E-08 |
| rs10427502 | 21 | 40654840 | G | A | 0.014 | 0.002 | 8.60E-10 | -0.005 | 0.001 | 1.50E-04 |
| rs7609 | 22 | 29951650 | A | T | 0.015 | 0.003 | 3.60E-09 | 0.000 | 0.002 | 9.60E-01 |
| rs2092829 | 22 | 31780514 | G | A | 0.014 | 0.002 | 1.30E-08 | 0.003 | 0.002 | 6.60E-02 |

| **Table S34. Multivariable Mendelian randomization estimates between leisure television watching, leisure computer use and cancer risk** | | | | |
| --- | --- | --- | --- | --- |
| **OR* (95% CI) adjusted for:** | | | | |
| **Traits** | **Alcohol** | **BMI** | **Years of education** | **Smoking** |
| **Cancer Type** |  |  |  |  |
| **Television watching** | |  |  |  |
| **Breast cancer** |  |  |  |  |
| Overall | 1.15 (1.04, 1.28) | 1.27 (1.13, 1.43) | 1.08 (0.92, 1.27) | 1.12 (0.99, 1.27) |
| Luminal A | 1.21 (1.05, 1.39) | 1.30 (1.12, 1.52) | 1.11 (0.90, 1.35) | 1.19 (1.01, 1.39) |
| Luminal B | 1.09 (0.86, 1.39) | 1.17 (0.91, 1.51) | 0.93 (0.66, 1.31) | 1.13 (0.87, 1.48) |
| HER2 negative | 1.22 (1.00, 1.51) | 1.32 (1.07, 1.65) | 1.12 (0.84, 1.51) | 1.17 (0.93, 1.49) |
| HER2 enriched | 1.23 (0.89, 1.70) | 1.46 (1.05, 2.05) | 1.08 (0.66, 1.79) | 1.15 (0.79, 1.67) |
| Triple negative | 1.19 (0.97, 1.04) | 1.39 (1.12, 1.72) | 1.07 (0.81, 1.40) | 1.16 (0.92, 1.46) |
| **Colorectal cancer** |  |  |  |  |
| Overall | 1.26 (1.09, 1.46) | 1.20 (1.03, 1.38) | 1.08 (0.90, 1.31) | 1.17 (1.00, 1.39) |
| Men | 1.40 (1.17, 1.70) | 1.32 (1.09, 1.58) | 1.13 (0.88, 1.45) | 1.32 (1.08, 1.62) |
| Women | 1.17 (0.97, 1.40) | 1.11 (0.92, 1.34) | 1.07 (0.83, 1.38) | 1.08 (0.89, 1.34) |
| Colon | 1.26 (1.07, 1.49) | 1.20 (1.02, 1.40) | 1.07 (0.86, 1.32) | 1.17 (0.99, 1.40) |
| Rectal | 1.49 (1.20, 1.84) | 1.43 (1.16, 1.75) | 1.07 (0.79, 1.45) | 1.35 (1.05, 1.75) |
|  |  |  |  |  |
| **Computer use** |  |  |  |  |
| **Breast cancer** |  |  |  |  |
| Overall | 1.05 (0.93, 1.19) | 1.06 (0.87, 1.30) | 1.20 (0.99, 1.45) | 1.01 (0.84, 1.21) |
| Luminal A | 1.04 (0.90, 1.20) | 1.05 (0.84, 1.32) | 1.26 (1.00, 1.60) | 1.06 (0.85, 1.31) |
| Luminal B | 0.95 (0.73, 1.23) | 1.00 (0.70, 1.45) | 0.91 (0.60, 1.40) | 0.88 (0.59, 1.30) |
| HER2 negative | 1.01 (0.83, 1.32) | 1.14 (0.83, 1.57) | 1.32 (0.94, 1.88) | 1.09 (0.80, 1.49) |
| HER2 enriched | 1.32 (0.98, 1.79) | 0.77 (0.48, 1.26) | 1.06 (0.59, 1.90) | 0.66 (0.39, 1.08) |
| Triple negative | 1.06 (0.87, 1.30) | 0.73 (0.52, 1.01) | 0.79 (0.57, 1.09) | 0.68 (0.49, 0.95) |
| **Colorectal cancer** |  |  |  |  |
| Overall | 1.14 (0.99, 1.32) | 0.81 (0.66, 0.99) | 1.01 (0.79, 1.27) | 0.90 (0.72, 1.12) |
| Men | 1.11 (0.93, 1.32) | 0.82 (0.61, 1.09) | 0.89 (0.65, 1.20) | 0.84 (0.63, 1.09) |
| Women | 1.20 (0.99, 1.43) | 0.87 (0.66, 1.14) | 1.14 (0.84, 1.54) | 0.98 (0.72, 1.34) |
| Colon | 1.19 (1.00, 1.42) | 0.84 (0.66, 1.05) | 0.99 (0.76, 1.30) | 0.90 (0.71, 1.16) |
| Rectal | 1.09 (0.89, 1.34) | 0.63 (0.48, 0.84) | 0.92 (0.65, 1.30) | 0.64 (0.46, 0.90) |
| Abbreviations: BMI Body mass index, CI Confidence intervals, HER2 Human epidermal growth factor receptor 2, OR Odds ratio, SNPs Single nucleotide polymorphism  * The estimates correspond to a standard deviation increase in leisure television watching or computer use | | | | |

| **Table S35: Outlying SNPs identified by MR-PRESSO** | | | | | | |
| --- | --- | --- | --- | --- | --- | --- |
| **Exposure** |  | | | | | |
|  | **Colorectal cancer in women** | **Overall breast cancer** | **Luminal A breast cancer** | **Luminal B breast cancer** | **Overall prostate cancer** | |
| **Television watching** |  | rs1243182  rs11714337 | rs1243182  rs11714337 |  | rs4845364  rs62332760  rs6141814 | |
| **Computer use** | rs3730399 | rs11708955  rs162894  rs4702 | rs162894  rs113851275 | rs4702 | rs11708955 | |
| MR-PRESSO MR pleiotropy residual sum and outlier test | | | | | |  |

| **Table S36. Evidence of association (p<5*10-8) with secondary traits of the SNPs (or their proxies) used as genetic instruments for Mendelian randomization analyses of sedentary behaviours and risk of breast and colorectal cancer** | | | |
| --- | --- | --- | --- |
| SNP | Chr | Gene | Diseases & traits |
| **Computer** |  |  |  |
| rs2748985 | 1 | CFAP74 | Arm fat-free mass (UK Biobank), Arm predicted mass (UK Biobank) Basal metabolic rate (UK Biobank), Body mass index (UK Biobank), Impedance of arm/whole body (UK Biobank), Trunk fat-free mass (UK Biobank), Trunk predicted mass (UK Biobank) |
| rs12145677 | 1 | SYPL2 | Impedance of arm/leg/whole body (UK Biobank), Qualifications: college or university degree (UK Biobank), Time spent watching television (UK Biobank) |
| rs7526456 | 1 |  |  |
| rs10754920 | 2 |  |  |
| rs6744254 | 2 | KCNJ3 |  |
| rs11274218 | 2 | SPAG16 |  |
| rs4592851 | 2 | VWC2L |  |
| rs10208088 | 2 | LOC105373893 |  |
| rs11708955 | 3 | DAG1 | Mean corpuscular volume (PMID: 27863252), Reticulocyte count (PMID: 27863252), Crohns disease (PMID: 26192919), Inflammatory bowel disease (PMID: 26192919), Ulcerative colitis (PMID: 26192919), Primary sclerosing cholangitis (PMID: 27992413), Years of educational attainment (PMID: 27225129), Arm fat mass/percentage (UK Biobank), Body mass index (UK Biobank), Time spent watching television (UK Biobank), Diastolic blood pressure (UK Biobank), Leg fat mass/percentage (UK Biobank), Trunk fat mass/percentage (UK Biobank), Whole body fat mass (UK Biobank), Weight (UK Biobank), Age at menarche (PMID: 25231870), Fed-up/miserableness (UK Biobank), Fluid intelligence score (UK Biobank) |
| rs6774533 | 3 | CADPS | Arm fat mass right (UK Biobank), Hip circumference (UK Biobank), Trunk fat mass (UK Biobank), Waist circumference (UK Biobank), Whole body fat mass (UK Biobank), Qualifications: college or university degree (UK Biobank) |
| rs78082503 | 3 |  |  |
| rs6857629 | 4 |  |  |
| rs2068625 | 4 | C4orf45 | Overall health rating (UK Biobank), Pulse rate (UK Biobank) |
| rs2220599 | 5 | LINC02142 | Number of days or week walked 10+ minutes (UK Biobank), Subjective well being (PMID: 29292387) |
| rs11749912 | 5 | MEF2C | Trunk fat percentage (UK Biobank), Years of educational attainment (PMID: 27225129) |
| rs1469249 | 5 | LOC101927078 | Self-reported hypertension (UK Biobank), Years of educational attainment (PMID: 27225129) |
| rs162894 | 5 |  | Eosinophil count (PMID: 27863252), Granulocyte count (PMID: 27863252), Mean platelet volume (PMID: 27863252), Monocyte count (PMID: 27863252), Myeloid white cell count (PMID: 27863252), Height (PMID: 25282103), Asthma (UK Biobank), Basal metabolic rate (UK Biobank), Hand grip strength right (UK Biobank), Leg fat-free mass (UK Biobank), Leg predicted mass (UK Biobank), Trunk fat-free mass (UK Biobank), Trunk predicted mass (UK Biobank), Whole body fat-free mass (UK Biobank) |
| rs56158102 | 5 |  |  |
| rs9477970 | 6 | LOC101928519 | Arm fat-free mass (UK Biobank), Arm predicted mass (UK Biobank)  Basal metabolic rate (UK Biobank), Height (UK Biobank), Impedance of arm/whole body (UK Biobank), Leg predicted mass (UK Biobank), Leg fat-free mass (UK Biobank), Trunk fat-free mass (UK Biobank), Trunk predicted mass (UK Biobank), Whole body fat-free mass (UK Biobank), Qualifications: college or university degree (UK Biobank) |
| rs2179517 | 6 | H3C4 | Arm fat mass (UK Biobank), Arm fat percentage (UK Biobank), Arm fat-free mass (UK Biobank), Basal metabolic rate (UK Biobank), Body fat percentage (UK Biobank), Height (UK Biobank), Hip circumference (UK Biobank), Leg fat mass (UK Biobank), Leg fat percentage (UK Biobank), Leg fat-free mass (UK Biobank), Leg predicted mass (UK Biobank), Mean corpuscular hemoglobin (PMID: 27863252), Qualifications: college or university degree (UK Biobank), Trunk fat mass (UK Biobank), Trunk fat percentage (UK Biobank), Trunk fat-free mass (UK Biobank), Trunk predicted mass (UK Biobank), Waist circumference (UK Biobank), Weight (UK Biobank)  Whole body fat mass (UK Biobank), Whole body fat-free mass (UK Biobank) |
| rs9372625 | 6 |  | Alcohol intake frequency (UK Biobank), Arm fat mass (UK Biobank), Arm fat percentage (UK Biobank), Body fat percentage (UK Biobank), Body mass index (UK Biobank), Leg fat mass (UK Biobank), Leg fat percentage (UK Biobank), Overall health rating (UK Biobank), Time spent watching television (UK Biobank), Trunk fat mass (UK Biobank), Whole body fat mass (UK Biobank), Years of educational attainment (PMID: 27225129), Number of days or week walked 10+ minutes (UK Biobank), Sodium in urine (UK Biobank), Weight (UK Biobank), Fluid intelligence score (UK Biobank) |
| rs17789218 | 6 | LOC105377911 | Arm fat mass (UK Biobank), Basal metabolic rate (UK Biobank), Comparative height size at age 10 (UK Biobank), Diastolic blood pressure (UK Biobank), Hip circumference (UK Biobank), Impedance of leg (UK Biobank), Leg fat-free mass (UK Biobank), Leg predicted mass (UK Biobank), Trunk fat mass (UK Biobank), Weight (UK Biobank), Whole body fat mass (UK Biobank) |
| rs6935828 | 6 |  | Hayfever, allergic rhinitis or eczema (UK Biobank) |
| rs55772938 | 7 | AUTS2 | Trunk fat mass (UK Biobank), Weight (UK Biobank) |
| rs12706626 | 7 | POT1 |  |
| rs3944151 | 7 |  | Pulse rate (UK Biobank) |
| rs2345941 | 7 | EXOC4 |  |
| rs13262595 | 8 | TSNARE1 | Nervous feelings (UK Biobank), Schizophrenia (PMID: 26198764)  Tense or highly strung (UK Biobank), Qualifications-degree (UK Biobank) |
| rs4977839 | 9 |  | Bipolar disorder (PMID: 27329760), Basal metabolic rate (UK Biobank), Fluid intelligence score (UK Biobank), Impedance of leg (UK Biobank), Leg fat-free mass (UK Biobank), Leg predicted mass (UK Biobank), Time spent watching television (UK Biobank), Whole body fat-free mass (UK Biobank), Years of educational attainment (PMID: 27225129), Qualifications-degree (UK Biobank) |
| rs113851275 | 9 | LOC105376157 | Arm fat-free mass (UK Biobank), Arm predicted mass (UK Biobank), Basal metabolic rate (UK Biobank), Birth weight (UK Biobank), Forced expiratory volume in 1-second (UK Biobank), Forced vital capacity (UK Biobank), Height (UK Biobank), Impedance of leg (UK Biobank), Leg fat-free mass (UK Biobank), Leg predicted mass (UK Biobank), Trunk fat-free mass (UK Biobank), Trunk predicted mass (UK Biobank), Whole body fat-free mass (UK Biobank), Whole body water mass (UK Biobank), Sensitivity or hurt feelings (UK Biobank) |
| rs7020477 | 9 | AMBP |  |
| rs73578186 | 9 | DENND1A |  |
| rs2734849 | 11 | ANKK1 | Neuroticism score (UK Biobank) |
| rs1448355 | 11 | NTM |  |
| rs35933007 | 12 |  | Morning or evening person (UK Biobank) |
| rs1319734 | 12 | SLC2A13 |  |
| rs206965 | 12 |  |  |
| rs12874776 | 13 |  |  |
| rs198262 | 14 | OTX2-AS1 |  |
| rs166835 | 15 | SEMA6D |  |
| rs4702 | 15 | FES | Schizophrenia (PMID: 26198764), Diastolic blood pressure (UK Biobank), Systolic blood pressure (UK Biobank) |
| rs6498759 | 16 | LOC107984893 |  |
| rs3730399 | 16 | E2F4 | Mean corpuscular haemoglobin, (PMID: 27863252), High density lipoprotein (PMID: 24097068) |
| rs4073003 | 17 | EPN2 |  |
| rs7209653 | 17 | AKAP10 | Mean corpuscular haemoglobin (PMID: 27863252), Reticulocyte count (PMID: 27863252) |
| rs12603813 | 17 | PLCD3 | Arm fat-free mass (UK Biobank), Arm predicted mass (UK Biobank)  Basal metabolic rate (UK Biobank), Height (UK Biobank), Impedance of arm/leg/whole body (UK Biobank), Leg fat-free mass (UK Biobank), Leg predicted mass (UK Biobank), Sleeplessness or insomnia (UK Biobank), Systolic blood pressure (UK Biobank), Trunk fat-free mass (UK Biobank), Trunk predicted mass (UK Biobank), Whole body fat-free mass (UK Biobank) |
| rs66643547 | 17 | BAHCC1- MIR4740 | Height (UK Biobank), Forced vital capacity (UK Biobank), Qualifications-degree (UK Biobank) |
| rs784256 | 18 | LOC105372130 | Body mass index (UK Biobank), Creatinine in urine (UK Biobank), Impedance of arm/leg/whole body (UK Biobank), Mood swings (UK Biobank), Fed-up feelings (UK Biobank) Overall health rating (UK Biobank), Potassium in urine (UK Biobank), Usual walking pace (UK Biobank), Qualifications-degree (UK Biobank), Hand grip strength (UK Biobank), Fuchss corneal dystrophy (UK Biobank) |
| rs6129084 | 20 | CDH4 | Educational attainment years of education (PMID: 27225129), Qualifications-degree (UK Biobank) |
| rs9608249 | 22 |  |  |
| rs136553 | 22 | LOC110091768 |  |
| rs7288455 | 22 | CACNA1I |  |
|  |  |  |  |
| **Television** |  |  |  |
| rs11810109 | 1 | ST3GAL3 | Age at menarche (UK Biobank), Comparative height size at age 10 (UK Biobank), Fluid intelligence score (UK Biobank), Years of educational attainment (PMID:27225129) |
| rs112632642 | 1 | FAF1 |  |
| rs6670915 | 1 |  |  |
| rs12725114 | 1 | PATJ |  |
| rs984409 | 1 | SGIP1 |  |
| rs10890123 | 1 | TNNI3K |  |
| rs71658797 | 1 | AK5 | Lung cancer (PMID: 28604730), Arm fat mass (UK Biobank), Arm fat percentage (UK Biobank), Arm fat-free mass (UK Biobank), Arm predicted mass (UK Biobank), Basal metabolic rate (UK Biobank), Body mass index (UK Biobank), Creatinine in urine (UK Biobank), Height (UK Biobank), Hip circumference (UK Biobank), Impedance of leg/whole body (UK Biobank), Leg fat mass (UK Biobank), Leg fat percentage (UK Biobank), Leg fat-free mass (UK Biobank), Leg predicted mass (UK Biobank), Trunk fat mass (UK Biobank), Trunk fat percentage (UK Biobank), Trunk fat-free mass (UK Biobank), Trunk predicted mass (UK Biobank), Waist circumference (UK Biobank), Weight (UK Biobank), Whole body fat mass (UK Biobank), Whole body fat-free mass (UK Biobank) |
| rs114328297 | 1 |  | Average weekly beer plus cider intake (UK Biobank), Morning or evening person (UK Biobank), Chronotype (PMID: 27494321) |
| rs631130 | 1 |  |  |
| rs148648822 | 1 | DPYD |  |
| rs17379561 | 1 | DPYD | Arm fat mass (UK Biobank), Arm fat percentage (UK Biobank), Body fat percentage (UK Biobank), Leg fat mass (UK Biobank), Leg fat percentage (UK Biobank), Overall health rating (UK Biobank), Trunk fat mass (UK Biobank), Trunk fat percentage (UK Biobank), Waist circumference (UK Biobank), Whole body fat mass (UK Biobank), Years of educational attainment (PMID: 27225129), ), Qualifications-degree (UK Biobank) |
| rs55909997 | 1 |  | Arm fat mass (UK Biobank), Leg fat mass (UK Biobank), Trunk fat mass (UK Biobank), Waist circumference (UK Biobank), Weight (UK Biobank), Whole body fat mass (UK Biobank) |
| rs1278847 | 1 | CYB561D1 | Impedance of arm/leg/whole body (UK Biobank), Time spent using computer (UK Biobank), Low density lipoprotein (PMID: 24097068), Total cholesterol (PMID: 24097068) |
| rs141425667 | 1 |  |  |
| rs4845364 | 1 | TPM3 | Platelet count (PMID: 27863252) |
| rs6673341 | 1 |  | Leg fat percentage (UK Biobank), Trunk fat percentage (UK Biobank), Body fat percentage (UK Biobank), Body mass index (UK Biobank) |
| rs72725224 | 1 |  |  |
| rs10737620 | 1 | RO60 |  |
| rs6721975 | 2 | SOX11 |  |
| rs11896615 | 2 |  |  |
| rs72781699 | 2 | FKBP1B | Lymphocyte count (PMID: 27863252), Red blood cell count (PMID: 27863252), White blood cell count (PMID: 27863252), Height (UK Biobank), Trunk fat-free mass (UK Biobank), Trunk predicted mass (UK Biobank), Alcohol intake frequency (UK Biobank), Whole body fat-free mass (UK Biobank) |
| rs12105701 | 2 | LOC105374506 |  |
| rs7564130 | 2 | NRXN1 |  |
| rs11125515 | 2 |  |  |
| rs12475168 | 2 | LINC01122 | Mean corpuscular hemoglobin (PMID:27863252), Mean corpuscular volume (PMID:27863252), Red blood cell count (PMID:27863252) |
| rs183566425 | 2 |  |  |
| rs10189857 | 2 | BCL11A | Red blood cell count (PMID: 27863252), Height (UK Biobank), Years of educational attainment (PMID: 27225129), Cognitive ability multi trait analysis (PMID: 29186694), Qualifications-degree (UK Biobank) |
| rs62641636 | 2 | PPP3R1 |  |
| rs59210467 | 2 |  |  |
| rs11689199 | 2 |  | Arm fat mass (UK Biobank), Arm fat percentage (UK Biobank), Body fat percentage (UK Biobank), Body mass index (UK Biobank), Hip circumference (UK Biobank), Leg fat mass (UK Biobank), Leg fat percentage (UK Biobank), Trunk fat mass (UK Biobank), Trunk fat percentage, Waist circumference (UK Biobank), Weight (UK Biobank), Whole body fat mass (UK Biobank), Years of educational attainment (PMID: 27225129), Cognitive ability multi trait analysis (PMID: 29186694) |
| rs2916251 | 2 |  | Alcohol intake versus 10 years previously (UK Biobank), Body mass index (UK Biobank), Impedance of arm (UK Biobank), Leg fat mass (UK Biobank), Leg fat percentage (UK Biobank), Past tobacco smoking (UK Biobank), Trunk fat mass (UK Biobank), Weight (UK Biobank), Whole body fat mass (UK Biobank) |
| rs1451533 | 2 | PANTR1 | Arm fat mass left (UK Biobank), Arm fat percentage (UK Biobank), Body mass index (UK Biobank), Impedance of leg/whole body (UK Biobank), Leg fat mass (UK Biobank) |
| rs2971640 | 2 |  |  |
| rs12476388 | 2 | DPP10 |  |
| rs374722 | 2 |  |  |
| rs3754970 | 2 | TANK | Fluid intelligence score (UK Biobank), Years of educational attainment (PMID: 27225129), Cognitive ability multi trait analysis (PMID: 29186694), Qualifications-degree (UK Biobank) |
| rs1913808 | 2 | SLC4A10 |  |
| rs2060199 | 2 | SCN2A |  |
| rs263771 | 2 |  |  |
| rs4577309 | 2 | MFSD6 |  |
| rs34224261 | 2 |  | Leg fat mass (UK Biobank), Leg fat percentage (UK Biobank), Qualifications-degree (UK Biobank) |
| rs4675246 | 2 |  |  |
| rs13029509 | 2 | VWC2L | Average weekly champagne plus white wine intake (UK Biobank) |
| rs10932837 | 2 | LOC105373893 |  |
| rs4973576 | 2 | NGEF | Height (UK Biobank) |
| rs138256022 | 3 | VGLL4 |  |
| rs9834970 | 3 |  | Bipolar disorder (PMID: 28072414) |
| rs1352890 | 3 | BSN | High light scatter percentage of red cells (PMID: 27863252), High light scatter reticulocyte count (PMID: 27863252), Immature fraction of reticulocytes (PMID: 27863252), Reticulocyte count (PMID: 27863252), Reticulocyte fraction of red cells (PMID: 27863252), Crohns disease (PMID: 26192919), Inflammatory bowel disease (PMID: 26192919), Ulcerative colitis (PMID: 26192919), Body fat percentage (UK Biobank), Pulse rate (UK Biobank), Qualifications: college or university degree (UK Biobank), Trunk fat percentage (UK Biobank) |
| rs3796386 | 3 | CAMKV | Reticulocyte count (PMID: 27863252), Inflammatory bowel disease (PMID: 26192919), Ulcerative colitis (PMID: 23128233), Age at menarche (PMID: 25231870), Years of educational attainment (PMID: 27225129), Age at first live birth (UK Biobank), Age at menarche (UK Biobank), Arm fat mass (UK Biobank), Arm fat percentage (UK Biobank), Arm fat-free mass (UK Biobank), Arm predicted mass (UK Biobank), Average weekly beer plus cider intake (UK Biobank), Basal metabolic rate (UK Biobank), Body fat percentage (UK Biobank), Body mass index (UK Biobank), Hip circumference (UK Biobank), Impedance of arm/leg/whole body (UK Biobank), Leg fat mass (UK Biobank), Leg fat percentage (UK Biobank), Leg fat-free mass (UK Biobank), Leg predicted mass (UK Biobank), Overall health rating (UK Biobank), Trunk fat mass (UK Biobank), Trunk fat percentage (UK Biobank), Trunk fat-free mass (UK Biobank), Trunk predicted mass (UK Biobank), Waist circumference (UK Biobank), Weight (UK Biobank), Whole body fat mass (UK Biobank), Whole body fat-free mass (UK Biobank), Average weekly beer plus cider intake (UK Biobank), Qualifications-degree (UK Biobank) |
| rs11917871 | 3 | CACNA2D3 |  |
| rs11130793 | 3 | FHIT |  |
| rs11714337 | 3 | FOXP1 | Alcohol intake frequency (UK Biobank), Forced vital capacity (UK Biobank), Educational attainment years of education (PMID: 27225129), Qualifications-degree (UK Biobank) |
| rs12496084 | 3 |  |  |
| rs6797840 | 3 | CADM2 | Arm fat mass (UK Biobank), Arm fat-free mass (UK Biobank), Body fat percentage (UK Biobank), Body mass index (UK Biobank), Hip circumference (UK Biobank), Impedance of arm (UK Biobank), Leg fat mass (UK Biobank), Leg fat percentage (UK Biobank), Qualifications-degree (UK Biobank), Trunk fat mass (UK Biobank), Waist circumference (UK Biobank), Weight (UK Biobank), Whole body fat mass (UK Biobank) |
| rs56398417 | 3 | HTR1F | Arm fat mass (UK Biobank), Arm fat-free mass (UK Biobank), Arm predicted mass (UK Biobank), Basal metabolic rate (UK Biobank), Body mass index (UK Biobank),  Leg fat mass (UK Biobank), Leg fat-free mass (UK Biobank), Leg predicted mass (UK Biobank), Trunk fat mass (UK Biobank), Trunk fat-free mass (UK Biobank), Trunk predicted mass (UK Biobank), Waist circumference (UK Biobank), Weight (UK Biobank), Whole body fat mass (UK Biobank), Whole body fat-free mass (UK Biobank), Whole body water mass (UK Biobank) |
| rs2034768 | 3 |  | Arm fat mass (UK Biobank), Arm fat percentage (UK Biobank), Basal metabolic rate (UK Biobank), Body fat percentage (UK Biobank), Body mass index (UK Biobank), Hip circumference (UK Biobank), Impedance of leg/whole body (UK Biobank), Leg fat mass (UK Biobank), Leg fat percentage (UK Biobank), Leg fat-free mass (UK Biobank), Leg predicted mass (UK Biobank), Trunk fat mass (UK Biobank), Trunk fat percentage (UK Biobank), Waist circumference (UK Biobank), Weight (UK Biobank), Whole body fat mass (UK Biobank),Whole body fat-free mass (UK Biobank) |
| rs12632778 | 3 | LOC105374029 |  |
| rs9867121 | 3 | ZBTB20 |  |
| rs12491503 | 3 |  | Serum butyrylcholinesterase activity (PMID: 21862451) |
| rs114600294 | 3 | SOX2-OT |  |
| rs66852340 | 4 | RGS12 | Impedance of arm (UK Biobank), Qualifications-degree (UK Biobank) |
| rs7700107 | 4 | LCORL | Height (PMID: 25282103), Arm fat percentage (UK Biobank), Arm fat-free mass (UK Biobank), Arm predicted mass (UK Biobank), Basal metabolic rate (UK Biobank), Birth weight (UK Biobank), Body fat percentage (UK Biobank), Forced vital capacity (UK Biobank), Leg fat percentage (UK Biobank), Leg fat-free mass (UK Biobank), Leg predicted mass (UK Biobank), Self-reported hypertension (UK Biobank), Trunk fat-free mass (UK Biobank), Trunk predicted mass (UK Biobank), Weight (UK Biobank), Whole body fat-free mass (UK Biobank), Hand grip strength (UK Biobank) |
| rs34811474 | 4 | ANAPC4 | Age at menarche (UK Biobank), Arm fat mass (UK Biobank), Arm fat percentage (UK Biobank), Body fat percentage (UK Biobank), Body mass index (UK Biobank), Comparative body size at age 10 (UK Biobank), Diastolic blood pressure (UK Biobank), Forced expiratory volume in 1-second (UK Biobank), Forced vital capacity (UK Biobank), Height (UK Biobank), Hip circumference (UK Biobank), Impedance of arm (UK Biobank), Impedance of leg (UK Biobank), Impedance of whole body (UK Biobank), Leg fat mass (UK Biobank), Leg fat percentage right (UK Biobank), Overall health rating (UK Biobank), Qualifications: college or university degree (UK Biobank), Usual walking pace (UK Biobank), Waist circumference (UK Biobank), Weight (UK Biobank), Whole body fat mass (UK Biobank) |
| rs77215114 | 4 |  |  |
| rs28971796 | 4 |  | Hip circumference (UK Biobank), Weight (UK Biobank) |
| rs6850494 | 4 |  | Arm fat-free mass (UK Biobank), Arm predicted mass (UK Biobank), Basal metabolic rate (UK Biobank), Height (UK Biobank), Leg fat-free mass (UK Biobank), Leg predicted mass (UK Biobank), Trunk fat mass (UK Biobank), Trunk fat-free mass (UK Biobank), Trunk predicted mass (UK Biobank), Weight (UK Biobank), Whole body fat-free mass (UK Biobank) |
| rs13107325 | 4 | SLC39A8 | Hematocrit (PMID: 27863252), Childhood BMI (PMID: 26604143), Body mass index (PMID: 29273807), Height (PMID: 28146470), High density lipoprotein (PMID: 24097068), Diastolic blood pressure (PMID: 21909110), Systolic blood pressure (PMID: 21909110), Crohns disease (PMID: 26192919), Schizophrenia (PMID: 26198764), Alcohol intake frequency (UK Biobank), Arm fat mass (UK Biobank), Arm fat percentage (UK Biobank), Fluid intelligence score (UK Biobank), Hip circumference (UK Biobank), Impedance of leg (UK Biobank), Leg fat mass (UK Biobank), Leg fat percentage (UK Biobank), Prospective memory result (UK Biobank), Qualifications-degree (UK Biobank), Trunk fat mass (UK Biobank), Trunk fat percentage (UK Biobank), Usual walking pace (UK Biobank), Waist circumference (UK Biobank), Weight (UK Biobank), Sleep duration (UK Biobank), Overall health rating (UK Biobank) |
| rs62332760 | 4 | TET2 | Lymphocyte count (PMID: 27863252), Lymphocyte percentage of white cells (PMID: 27863252), Neutrophil percentage of white cells (PMID: 27863252), Arm fat-free mass (UK Biobank), Arm predicted mass (UK Biobank), Basal metabolic rate (UK Biobank), Birth weight of first child (UK Biobank), Comparative height size at age 10 (UK Biobank), Fluid intelligence score (UK Biobank), Forced expiratory volume in 1-second (UK Biobank), Forced vital capacity (UK Biobank), Height (UK Biobank), Leg fat-free mass (UK Biobank), Leg predicted mass (UK Biobank), Qualifications: college or university degree (UK Biobank), Trunk fat-free mass (UK Biobank), Trunk predicted mass (UK Biobank), Weight (UK Biobank), Whole body fat-free mass (UK Biobank), Whole body water mass (UK Biobank) |
| rs7693703 | 4 | LINC01378 |  |
| rs4334769 | 4 |  |  |
| rs6825241 | 4 | FAM160A1 | Platelet count (PMID: 27863252), Height (UK Biobank) |
| rs7693082 | 4 | C4orf45 | Overall health rating (UK Biobank), Pulse rate (UK Biobank), Time spent using computer (UK Biobank) |
| rs10940659 | 5 | PDE4D | Qualifications-degree (UK Biobank) |
| rs2409838 | 5 | ERCC8 | Mean platelet volume (PMID: 27863252), Qualifications: college or university degree (UK Biobank), Years of educational attainment (PMID: 27225129) |
| rs262890 | 5 |  | Qualifications-degree (UK Biobank) |
| rs7716447 | 5 |  | Body mass index (UK Biobank), Height (UK Biobank), Impedance of arm/leg/whole body (UK Biobank) |
| rs1031423 | 5 | FAM172A |  |
| rs57585211 | 5 | FBXL17 |  |
| rs9326862 | 5 | APC | Arm fat-free mass (UK Biobank), Arm predicted mass (UK Biobank), Height (UK Biobank), Trunk fat-free mass (UK Biobank), Trunk predicted mass (UK Biobank) |
| rs10041724 | 5 |  |  |
| rs10057545 | 5 | SIL1 |  |
| rs62379379 | 5 |  |  |
| rs10054327 | 5 | HTR4 |  |
| rs42210 | 5 | TENM2 | Qualifications-degree (UK Biobank) |
| rs2336895 | 5 | TENM2 | Current tobacco smoking (UK Biobank), Past tobacco smoking (UK Biobank) |
| rs72828890 | 5 | WWC1 |  |
| rs255292 | 5 | BNIP1 | Height (UK Biobank), Pulse rate (UK Biobank) |
| rs72834698 | 6 |  | Mean corpuscular hemoglobin (PMID: 27863252), Arm fat mass (UK Biobank), Arm fat percentage (UK Biobank), Arm fat-free mass (UK Biobank), Arm predicted mass (UK Biobank), Basal metabolic rate (UK Biobank), Body fat percentage (UK Biobank), Height (UK Biobank), Hip circumference (UK Biobank), Leg fat mass (UK Biobank), Leg fat percentage (UK Biobank), Leg fat-free mass (UK Biobank), Leg predicted mass (UK Biobank), Trunk fat mass (UK Biobank), Trunk fat percentage (UK Biobank), Trunk fat-free mass (UK Biobank), Trunk predicted mass (UK Biobank), Waist circumference (UK Biobank), Weight (UK Biobank), Whole body fat mass (UK Biobank), Whole body fat-free mass (UK Biobank), Qualifications-degree (UK Biobank) |
| rs3135044 | 6 | PRRC2A | Eosinophil count (PMID: 27863252), Granulocyte count (PMID: 27863252), Immature fraction of reticulocytes (PMID: 27863252), Lymphocyte count (PMID: 27863252), Mean corpuscular hemoglobin (PMID: 27863252), Mean corpuscular volume (PMID: 27863252), Monocyte count (PMID: 27863252), Myeloid white cell count (PMID: 27863252), Neutrophil count (PMID: 27863252), Platelet distribution width (PMID: 27863252), Sum basophil neutrophil counts (PMID: 27863252), Sum eosinophil basophil counts (PMID: 27863252), Sum neutrophil eosinophil counts (PMID: 27863252), White blood cell count (PMID: 27863252), IgA deficiency (PMID: 27723758), Primary sclerosing cholangitis (PMID: 27992413), Arm fat percentage (UK Biobank), Arm fat-free mass (UK Biobank), Arm predicted mass (UK Biobank), Basal metabolic rate (UK Biobank), Comparative height size at age 10 (UK Biobank), Forced vital capacity (UK Biobank), Hand grip strength (UK Biobank), Height (UK Biobank), Intestinal malabsorption (UK Biobank), Leg fat percentage (UK Biobank), Leg fat-free mass (UK Biobank), Leg predicted mass (UK Biobank), Overall health rating (UK Biobank), Self-reported high cholesterol (UK Biobank), Self-reported hypertension (UK Biobank), Self-reported hyperthyroidism or thyrotoxicosis (UK Biobank), Self-reported malabsorption or coeliac disease (UK Biobank), Self-reported multiple sclerosis (UK Biobank), Self-reported psoriasis (UK Biobank), Self-reported rheumatoid arthritis (UK Biobank), Trunk fat-free mass (UK Biobank), Trunk predicted mass (UK Biobank), Whole body fat-free mass (UK Biobank), Whole body water mass (UK Biobank), Rheumatoid arthritis (UK Biobank), |
| rs576432906 | 6 |  |  |
| rs10947452 | 6 |  | Platelet count (PMID: 27863252), Plateletcrit (PMID: 27863252) |
| rs9471333 | 6 | LRFN2 | Arm fat mass (UK Biobank), Arm fat percentage (UK Biobank), Arm fat-free mass (UK Biobank), Arm predicted mass (UK Biobank), Basal metabolic rate (UK Biobank), Body fat percentage (UK Biobank), Body mass index (UK Biobank), Hip circumference (UK Biobank), Impedance of arm/whole body (UK Biobank), Leg fat mass (UK Biobank), Leg fat percentage (UK Biobank), Leg fat-free mass (UK Biobank), Leg predicted mass (UK Biobank), Trunk fat mass (UK Biobank), Trunk fat percentage (UK Biobank), Usual walking pace (UK Biobank), Waist circumference (UK Biobank), Weight (UK Biobank), Whole body fat mass (UK Biobank), Whole body fat-free mass (UK Biobank) |
| rs4523073 | 6 |  |  |
| rs77589760 | 6 | PM20D2 |  |
| rs6905544 | 6 |  | Gut microbiota bacterial taxa (PMID: 27694959), Alcohol intake frequency (UK Biobank), Arm fat mass (UK Biobank), Arm fat percentage (UK Biobank), Body fat percentage (UK Biobank), Body mass index (UK Biobank), Fluid intelligence score (UK Biobank), Leg fat mass (UK Biobank), Leg fat percentage (UK Biobank), Overall health rating (UK Biobank), Qualifications-degree (UK Biobank), Sodium in urine (UK Biobank), Time spent using computer (UK Biobank), Trunk fat mass (UK Biobank), Trunk fat percentage (UK Biobank), Waist circumference (UK Biobank), Weight (UK Biobank), Whole body fat mass (UK Biobank), Years of educational attainment (PMID: 27225129) |
| rs17789218 | 6 | LOC105377911 | Diastolic blood pressure (UK Biobank), Hip circumference (UK Biobank), Impedance of leg (UK Biobank), Leg fat-free mass (UK Biobank), Leg predicted mass (UK Biobank), Time spent using computer (UK Biobank), Trunk fat mass (UK Biobank), Weight (UK Biobank), Whole body fat mass (UK Biobank), Arm fat mass (UK Biobank), Basal metabolic rate (UK Biobank), Comparative height size at age 10 (UK Biobank) |
| rs78394231 | 6 | PDSS2 |  |
| rs2184364 | 6 | HIVEP2 | Body fat percentage (UK Biobank), Trunk fat mass (UK Biobank), Trunk fat percentage (UK Biobank) |
| rs9718104 | 6 | PDE10A | Bipolar disorder (UK Biobank) |
| rs17568389 | 6 | GLCCI1 | Height (PMID: 25282103), Trunk fat-free mass (UK Biobank), Trunk predicted mass (UK Biobank), Qualifications-degree (UK Biobank) |
| rs10234444 | 7 | LOC107986788 |  |
| rs6973656 | 7 | TMEM60 | Chronic kidney disease (PMID: 20383146), Glomerular filtration rate in non diabetics creatinine (PMID: 26831199), Serum creatinine (PMID: 20383146), Arm fat mass (UK Biobank), Body fat percentage (UK Biobank), Hip circumference (UK Biobank), Leg fat mass (UK Biobank), Leg fat percentage left (UK Biobank), Leg fat-free mass left (UK Biobank), Trunk fat mass (UK Biobank), Trunk fat percentage (UK Biobank), Weight (UK Biobank), Whole body fat mass (UK Biobank), basal metabolic rate (UK Biobank) |
| rs973734 | 7 |  |  |
| rs3137 | 7 | ZKSCAN5 | Arm fat mass (UK Biobank), Arm fat percentage (UK Biobank), Body fat percentage (UK Biobank), Body mass index (UK Biobank), Height (UK Biobank), Leg fat mass (UK Biobank), Leg fat percentage (UK Biobank), Sitting height (UK Biobank), Waist circumference (UK Biobank), Whole body fat mass (UK Biobank), Dihydroepiandrosterone sulphate (PMID: 28887542) |
| rs2694151 | 7 | CUX1 |  |
| rs62471080 | 7 |  |  |
| rs7788008 | 7 |  | Arm fat mass (UK Biobank), Arm fat percentage (UK Biobank), Body fat percentage (UK Biobank), Body mass index (UK Biobank), Leg fat mass (UK Biobank), Leg fat percentage (UK Biobank), Trunk fat mass (UK Biobank), Weight (UK Biobank) |
| rs10246289 | 7 |  |  |
| rs11763734 | 7 | GRM8 | Neuroticism score (UK Biobank) |
| rs7834121 | 8 | XKR6 | Neuroticism (PMID: 29292387), Granulocyte count (PMID: 27863252), Neutrophil count (PMID: 27863252), Platelet distribution width (PMID: 27863252), Sum basophil neutrophil counts (PMID: 27863252), Sum neutrophil eosinophil counts (PMID: 27863252), White blood cell count (PMID: 27863252), Body mass index (UK Biobank), Diastolic blood pressure (UK Biobank), Heel bone mineral density (UK Biobank), Impedance of arm/leg/whole body (UK Biobank), Systolic blood pressure (UK Biobank), Nervous feelings (UK Biobank) |
| rs1421334 | 8 | PURG | Arm fat mass (UK Biobank), Arm fat percentage (UK Biobank), Body fat percentage (UK Biobank), Body mass index (UK Biobank), Leg fat mass (UK Biobank), Leg fat percentage (UK Biobank), Overall health rating (UK Biobank), Trunk fat mass (UK Biobank), Trunk fat percentage (UK Biobank), Waist circumference (UK Biobank), Whole body fat mass (UK Biobank), Qualifications-degree (UK Biobank) |
| rs6996198 | 8 |  |  |
| rs6472942 | 8 |  | Arm fat mass (UK Biobank), Arm fat percentage (UK Biobank), Body fat percentage (UK Biobank), Body mass index (UK Biobank), Hip circumference (UK Biobank), Leg fat mass (UK Biobank), Leg fat percentage (UK Biobank), Systolic blood pressure (UK Biobank),Trunk fat mass (UK Biobank), Trunk fat percentage (UK Biobank), Waist circumference (UK Biobank),Weight (UK Biobank), Whole body fat mass (UK Biobank), Comparative body size at age 10 (UK Biobank) |
| rs11985394 | 8 |  |  |
| rs72671494 | 8 |  |  |
| rs12541615 | 8 | EXT1 |  |
| rs2717559 | 8 |  |  |
| rs2616830 | 9 | LOC105375951 | Years of educational attainment (PMID: 27225129) |
| rs2291681 | 9 | FREM1 |  |
| rs72671494 | 9 |  |  |
| rs34864022 | 9 |  |  |
| rs12554512 | 9 |  | Years of educational attainment (PMID: 27225129), Bipolar disorder (PMID: 27329760), Basal metabolic rate (UK Biobank), Fluid intelligence score (UK Biobank), Impedance of leg (UK Biobank), Leg fat-free mass (UK Biobank), Leg predicted mass (UK Biobank), Time spent using computer (UK Biobank), Whole body fat-free mass (UK Biobank), Qualifications-degree (UK Biobank), Cognitive ability (UK Biobank) |
| rs2791574 | 9 | TLE4 | Lymphocyte count (PMID: 27863252) |
| rs141184308 | 9 | KIF27 |  |
| rs870151 | 9 |  | Age at menarche (UK Biobank), Arm fat percentage (UK Biobank), Body fat percentage (UK Biobank), Body mass index (UK Biobank), Forced vital capacity (UK Biobank), Leg fat percentage (UK Biobank), Qualifications-degree (UK Biobank), Fibrinogen levels (PMID: 28107422), Trunk fat percentage (UK Biobank), Whole body fat mass (UK Biobank) |
| rs2787374 | 9 | INVS | Hip circumference (UK Biobank) |
| rs10739499 | 9 |  |  |
| rs7043521 | 9 | TTLL11 | Age completed full time education (UK Biobank), Body fat percentage (UK Biobank), Body mass index (UK Biobank), Leg fat mass (UK Biobank), Leg fat percentage (UK Biobank), Qualifications-degree (UK Biobank) |
| rs12342024 | 9 | CRB2 |  |
| rs4382592 | 9 | MED27 |  |
| rs2073869 | 9 | SPACA9 |  |
| rs7469569 | 9 | FUT7 | Lymphocyte count (PMID: 27863252), Monocyte percentage of white cells (PMID: 27863252) |
| rs1623025 | 10 | CELF2 | Qualifications-degree (UK Biobank) |
| rs1243182 | 10 | MLLT10 | Arm fat mass (UK Biobank), Arm fat percentage (UK Biobank), Body fat percentage (UK Biobank), Body mass index (UK Biobank), Creatinine in urine (UK Biobank), Hip circumference (UK Biobank), Leg fat mass (UK Biobank), Leg fat percentage (UK Biobank), Sodium in urine (UK Biobank), Trunk fat mass (UK Biobank), Trunk fat percentage (UK Biobank), Waist circumference (UK Biobank), Weight (UK Biobank), Whole body fat mass (UK Biobank) |
| rs2045147 | 10 | PCDH15 |  |
| rs10994943 | 10 | LINC02625 | Age completed full time education (UK Biobank) |
| rs11201422 | 10 |  |  |
| rs10786658 | 10 | ARMH3 | Years of educational attainment (PMID: 27225129) |
| rs7089973 | 10 | ABLIM1 | Bipolar disorder or attention deficit hyperactivity disorder |
| rs11245482 | 10 | CTBP2 |  |
| rs17727474 | 10 |  |  |
| rs12289262 | 11 | TEAD1 |  |
| rs801733 | 11 | PACS1 | Arm fat mass (UK Biobank), Arm fat percentage (UK Biobank), Arm fat-free mass (UK Biobank), Arm predicted mass (UK Biobank), Basal metabolic rate (UK Biobank), Body fat percentage (UK Biobank), Body mass index (UK Biobank), Hip circumference (UK Biobank), Leg fat mass (UK Biobank), Leg fat percentage (UK Biobank), Trunk fat mass (UK Biobank), Trunk fat percentage (UK Biobank), Waist circumference (UK Biobank), Weight (UK Biobank), Whole body fat mass (UK Biobank) |
| rs116175338 | 11 | STARD10 | Leg fat-free mass (UK Biobank), Leg predicted mass (UK Biobank) |
| rs12272012 | 11 | DLG2 |  |
| rs11020045 | 11 | FAT3 |  |
| rs17207890 | 11 |  | Qualifications-degree (UK Biobank) |
| rs10791546 | 11 | CEP126 |  |
| rs113584870 | 11 | CWF19L2 |  |
| rs328277 | 11 |  |  |
| rs648044 | 11 | ZBTB16 | Non glioblastoma glioma (PMID: 28346443) |
| rs11218575 | 11 | MIR100HG |  |
| rs74802478 | 11 | NTM |  |
| rs4937842 | 11 |  |  |
| rs10772643 | 12 |  | Years of educational attainment (PMID: 27225129) |
| rs74996610 | 12 | SOX5 |  |
| rs10771746 | 12 | IPO8 |  |
| rs10876864 | 12 | IKZF4 | Years of educational attainment (PMID: 27225129), Eosinophil count (PMID: 27863252), Eosinophil percentage of granulocytes (PMID: 27863252), Eosinophil percentage of white cells (PMID: 27863252), Neutrophil percentage of granulocytes (PMID: 27863252), Sum eosinophil basophil counts (PMID: 27863252), Allergic disease (PMID: 29083406), Type 1 diabetes (PMID: 18198356),Arm fat-free mass (UK Biobank), Arm predicted mass left (UK Biobank), Asthma (UK Biobank), Basal metabolic rate (UK Biobank), Body mass index (UK Biobank), Hayfever, allergic rhinitis or eczema (UK Biobank), Impedance of arm/leg/whole body (UK Biobank), Leg fat-free mass (UK Biobank), Leg predicted mass (UK Biobank), Trunk fat-free mass (UK Biobank), Trunk predicted mass (UK Biobank), Whole body fat-free mass (UK Biobank), Qualifications-degree (UK Biobank) |
| rs8756 | 12 | HMGA2 | Birthweight (PMID: 23202124), Height (PMID: 28146470), Hip circumference (PMID: 25673412), Weight (PMID: 23754948), Arm fat-free mass (UK Biobank), Arm predicted mass (UK Biobank), Basal metabolic rate (UK Biobank), Comparative body size at age 10 (UK Biobank), Comparative height size at age 10 (UK Biobank),Forced vital capacity (UK Biobank), Impedance of leg (UK Biobank), Leg fat-free mass (UK Biobank), Leg predicted mass (UK Biobank), Trunk fat mass (UK Biobank), Trunk fat-free mass (UK Biobank), Trunk predicted mass (UK Biobank), Whole body fat-free mass (UK Biobank), Pulse pressure (UK Biobank) |
| rs11179838 | 12 |  |  |
| rs2164744 | 12 | MYO1H | Red cell distribution width (PMID: 27863252) |
| rs2173650 | 12 | TESC |  |
| rs1790135 | 12 | MPHOSPH9 | Lymphocyte count (PMID: 27863252), Lymphocyte percentage of white cells (PMID: 27863252), Neutrophil percentage of white cells (PMID: 27863252), Platelet count (PMID: 27863252), Plateletcrit (PMID: 27863252), Basal metabolic rate (UK Biobank), Comparative height size at age 10 (UK Biobank), Diastolic blood pressure (UK Biobank), Height (UK Biobank), Leg fat-free mass (UK Biobank), Leg predicted mass (UK Biobank), Qualifications: college or university degree (UK Biobank), Trunk fat-free mass (UK Biobank), Trunk predicted mass (UK Biobank), Whole body fat-free mass (UK Biobank), Whole body water mass (UK Biobank), Years of educational attainment (UK Biobank) |
| rs9563168 | 13 |  |  |
| rs9569734 | 13 |  | Arm fat mass (UK Biobank), Basal metabolic rate (UK Biobank), Body mass index (UK Biobank), Impedance of leg/whole body (UK Biobank), Leg fat mass (UK Biobank), Leg fat-free mass (UK Biobank), Leg predicted mass left (UK Biobank), Waist circumference (UK Biobank), Weight (UK Biobank) |
| rs9528023 | 13 |  |  |
| rs56858768 | 13 |  | Body mass index (UK Biobank) |
| rs553397860 | 13 | FARP1 |  |
| rs7991062 | 13 |  |  |
| rs11629120 | 14 | C14orf93 | Red cell distribution width (PMID: 27863252), Reticulocyte fraction of red cells (PMID: 27863252), Systolic blood pressure (UK Biobank), Years of educational attainment (PMID: 27225129) |
| rs405797 | 14 | NOVA1 | Qualifications-degree (UK Biobank) |
| rs1022785 | 14 | LOC107984685 | Qualifications-degree (UK Biobank) |
| rs10145592 | 14 |  |  |
| rs7157001 | 14 | LOC105370660 |  |
| rs8043253 | 14 | EXD1 | Diastolic blood pressure (UK Biobank), Hayfever, allergic rhinitis or eczema (UK Biobank), Height (UK Biobank), Self-reported hypertension (UK Biobank), Systolic blood pressure (UK Biobank), Age at menopause (PMID: 26414677) |
| rs2460 | 15 | ONECUT1 |  |
| rs4775373 | 15 | RORA |  |
| rs61331678 | 15 | LINGO1 | Miserableness (UK Biobank), Neuroticism score (UK Biobank), Years of educational attainment (PMID: 27225129), Qualifications-degree (UK Biobank) |
| rs35574015 | 16 | PDXDC1 | Arm fat-free mass (UK Biobank), Arm predicted mass (UK Biobank), Basal metabolic rate (UK Biobank), Height (UK Biobank), Leg fat-free mass (UK Biobank), Leg predicted mass (UK Biobank), Trunk fat-free mass (UK Biobank), Trunk predicted mass (UK Biobank), Weight (UK Biobank), Whole body fat-free mass (UK Biobank), Arachidonic acid 20:4n6 (PMID: 24823311), Dihomo-gamma-linolenic acid (PMID: 24823311), Gamma-linolenic acid 18:3n6 (PMID: 24823311), Linoleic acid 18:2n6 (PMID: 24823311), Plasma omega 6 polyunsaturated fatty acid levels linoleic acid (PMID: 24823311), Blood metabolite ratios (PMID: 24816252) |
| rs7189927 | 16 | ATP2A1 | Red blood cell count (PMID: 27863252), Body mass index (PMID: 25673413), Hip circumference (PMID: 25673412), Waist circumference (PMID: 25673412), Weight (PMID: 23754948), Alcohol intake frequency (UK Biobank), Arm fat mass (UK Biobank), Arm fat percentage (UK Biobank), Arm fat-free mass left (UK Biobank), Arm predicted mass left (UK Biobank), Basal metabolic rate (UK Biobank), Body fat percentage (UK Biobank), Hip circumference (UK Biobank), Impedance of leg (UK Biobank), Leg fat mass (UK Biobank), Leg fat percentage (UK Biobank), Leg fat-free mass (UK Biobank), Leg predicted mass (UK Biobank), Trunk fat mass (UK Biobank), Trunk fat percentage (UK Biobank), Whole body fat mass (UK Biobank), Whole body fat-free mass (UK Biobank), Qualifications-degree (UK Biobank) |
| rs749671 | 16 | ZNF646 | Lymphocyte count (PMID: 27863252), Body mass index (PMID: 25673413), Hip circumference (PMID: 25673412), Waist circumference (PMID: 25673412), Triglycerides (PMID: 24097068), Arm fat mass (UK Biobank), Arm fat percentage (UK Biobank), Arm fat-free mass (UK Biobank), Arm predicted mass (UK Biobank), Basal metabolic rate (UK Biobank), Body fat percentage (UK Biobank), Body mass index (UK Biobank), Impedance of arm/leg/whole body (UK Biobank), Leg fat mass (UK Biobank), Leg fat percentage (UK Biobank), Leg fat-free mass (UK Biobank), Leg predicted mass (UK Biobank), Overall health rating (UK Biobank), Trunk fat mass (UK Biobank), Trunk fat percentage (UK Biobank), Trunk fat-free mass (UK Biobank), Trunk predicted mass (UK Biobank), Weight (UK Biobank), Whole body fat mass (UK Biobank), Whole body fat-free mass (UK Biobank), Alcohol intake frequency (UK Biobank) |
| rs7184800 | 16 | RBL2 | Arm fat-free mass (UK Biobank), Arm predicted mass (UK Biobank), Basal metabolic rate (UK Biobank), Fluid intelligence score (UK Biobank), Height (UK Biobank), Hip circumference (UK Biobank), Impedance of leg (UK Biobank), Leg fat-free mass (UK Biobank), Leg predicted mass (UK Biobank), Pulse rate (UK Biobank), Trunk fat-free mass (UK Biobank), Trunk predicted mass (UK Biobank), Weight (UK Biobank), Whole body fat-free mass (UK Biobank), Qualifications-degree (UK Biobank) |
| rs142710267 | 16 | PMFBP1 |  |
| rs2447098 | 17 | SGSM2 | Plateletcrit (PMID: 27863252) |
| rs11654952 | 17 |  |  |
| rs2584597 | 17 | TCAM1P |  |
| rs9902312 | 17 | HELZ |  |
| rs11657730 | 17 | BAHCC1 | Height (UK Biobank), Time spent using computer (UK Biobank), Forced vital capacity (UK Biobank), Qualifications-degree (UK Biobank) |
| rs303753 | 18 |  | Years of educational attainment (PMID: 27225129), Granulocyte count (PMID: 27863252), Myeloid white cell count (PMID: 27863252), Neutrophil count (PMID: 27863252), Alcohol intake frequency (UK Biobank), Arm fat mass (UK Biobank), Arm fat percentage (UK Biobank), Arm fat-free mass (UK Biobank), Arm predicted mass (UK Biobank), Basal metabolic rate (UK Biobank), Body fat percentage (UK Biobank), Body mass index (UK Biobank), Hip circumference (UK Biobank), Leg fat mass (UK Biobank), Leg fat percentage (UK Biobank), Leg fat-free mass (UK Biobank), Leg predicted mass (UK Biobank), Trunk fat mass (UK Biobank), Trunk fat percentage (UK Biobank), Trunk fat-free mass (UK Biobank), Trunk predicted mass (UK Biobank), Waist circumference (UK Biobank), Weight (UK Biobank), Whole body fat mass (UK Biobank), Whole body fat-free mass (UK Biobank), Qualifications-degree (UK Biobank) |
| rs71362609 | 18 |  |  |
| rs11081851 | 18 | NOL4 |  |
| rs9964724 |  |  | Educational attainment years of education (PMID: 27225129), Mood swings (UK Biobank), Neuroticism score (UK Biobank), Overall health rating (UK Biobank), Worrier or anxious feelings (UK Biobank), Qualifications-degree (UK Biobank) |
| rs1156541 | 18 | LINC00907 |  |
| rs17512836 | 18 | TCF4 | Schizophrenia (PMID: 21926974) |
| rs7248205 | 19 | ILF3 | Platelet count (PMID: 27863252), Arm fat-free mass (UK Biobank), Arm predicted mass (UK Biobank), Basal metabolic rate (UK Biobank), Height (UK Biobank), Hip circumference (UK Biobank), Leg fat-free mass (UK Biobank), Leg predicted mass (UK Biobank), Trunk fat-free mass (UK Biobank), Trunk predicted mass (UK Biobank), Weight (UK Biobank), Whole body fat-free mass (UK Biobank) |
| rs111901094 | 19 | GATAD2A | Low density lipoprotein (PMID: 24097068),Total cholesterol (PMID: 24097068), Triglycerides (PMID: 24097068), Plateletcrit (PMID: 27863252), Age at first live birth (UK Biobank), Height (UK Biobank), Leg fat percentage (UK Biobank), Leg fat-free mass (UK Biobank), Leg predicted mass (UK Biobank) |
| rs6131281 | 20 | BTBD3 |  |
| rs6141814 | 20 | DNMT3B |  |
| rs4810312 | 20 | LPIN3 | Low density lipoprotein (PMID: 24097068), Total cholesterol (PMID: 24097068) |
| rs6124476 | 20 | PTPRT |  |
| rs6031440 | 20 | JPH2 | Granulocyte percentage of myeloid white cells (PMID: 27863252), Monocyte percentage of white cells (PMID: 27863252) |
| rs55700114 | 20 |  | Qualifications-degree (UK Biobank) |
| rs6125906 | 20 | UBE2V1 |  |
| rs56103247 | 20 |  | Leg fat percentage (UK Biobank) |
| rs10427502 | 21 | BRWD1 | Age at menarche (UK Biobank) |
| rs7609 | 22 | NIPSNAP1 |  |
| rs2092829 | 22 |  |  |
| rs150399729 | 22 | RRP7BP |  |
| Abbreviations: Chr, chromosome; PMID, Pubmed ID; SNP, single nucleotide polymorphism | | | |

| **Table S37.** **Mendelian Randomization estimates for the effects of sedentary behaviour on the secondary traits** | | | | | | | | | | |
| --- | --- | --- | --- | --- | --- | --- | --- | --- | --- | --- |
| **Methods** | **Leisure television watching** | | | | | **Leisure computer use** | | | | |
|  | **Estimates** | **95% CI** | **P-value** |  | **P-value for pleiotropy^†^ or heterogeneity^‡^** | **Estimates (OR)** | **95% CI** | **P-value** |  | **P-value for pleiotropy^†^ or heterogeneity^‡^** |
| **Education** |  |  |  |  |  |  |  |  |  |  |
| Inverse-variance weighted | -0.54 | -0.58, -0.49 | 2×10^-125^ |  | 2×10^-156^ | 0.59 | 0.48, 0.70 | 7×10^-26^ |  | 4×10^-83^ |
| MR-Egger | -0.56 | -0.75, -0.36 | 2×10^-8^ |  | 0.83 | 1.02 | 0.32, 1.72 | 0.004 |  |  |
| Weighted median | -0.52 | -0.55, -0.48 | 7×10^-169^ |  |  | 0.59 | 0.51, 0.67 | 9×10^-46^ |  |  |
| Inverse-variance weighted* | -0.38 | -0.41, -0.34 | 8×10^-112^ |  | 9×10^-21^ | 0.33 | 0.25, 0.41 | 3×10^-17^ |  | 3×10^-8^ |
| **BMI** |  |  |  |  |  |  |  |  |  |  |
| Inverse-variance weighted | 0.38 | 0.32, 0.45 | 2×10^-31^ |  | 0 | 0 | -0.13, 0.12 | 0.96 |  | 5×10^-105^ |
| MR-Egger | 0.42 | 0.13, 0.72 | 0.005 |  | 0.79 | -0.09 | -0.88, 0.70 | 0.82 |  | 0.82 |
| Weighted median | 0.37 | 0.33, 0.42 | 8×10^-69^ |  |  | 0.02 | -0.06, 0.09 | 0.65 |  |  |
| Inverse-variance weighted* | 0.23 | 0.18, 0.28 | 8×10^-23^ |  | 6×10^-78^ | 0.02 | -0.08, 0.12 | 0.70 |  | 7×10^-37^ |
| **Smoking** |  |  |  |  |  |  |  |  |  |  |
| Inverse-variance weighted | 0.17 | 0.14, 0.20 | 2×10^-33^ |  | 2×10^-69^ | -0.06 | -0.12, -0.01 | 0.02 |  | 2×10^-17^ |
| MR-Egger | 0.10 | -0.03, 0.22 | 0.12 |  | 0.26 | -0.22 | -0.55, 0.12 | 0.21 |  | 0.37 |
| Weighted median | 0.16 | 0.14, 0.19 | 1×10^-38^ |  |  | -0.08 | -0.12, -0.03 | 0.002 |  |  |
| Inverse-variance weighted* | 0.15 | 0.13, 0.18 | 1×10^-31^ |  | 3×10^-45^ |  |  |  |  |  |
| **Alcohol consumption** |  |  |  |  |  |  |  |  |  |  |
| Inverse-variance weighted | -0.02 | -0.05, 0.02 | 0.39 |  | 1×10^-55^ | 0.00 | -0.07, 0.07 | 0.90 |  | 8×10^-14^ |
| MR-Egger | -0.13 | -0.28, 0.03 | 0.12 |  | 0.17 | 0.00 | -0.44, 0.44 | 1.00 |  | 0.98 |
| Weighted median | 0.00 | -0.03, 0.04 | 0.97 |  |  | -0.02 | -0.08, 0.04 | 0.57 |  |  |
| Inverse-variance weighted* | 0.00 | -0.04, 0.03 | 0.79 |  | 4×10^-37^ | 0.01 | -0.05, 0.08 | 0.72 |  | 4×10^-9^ |
| Abbreviations: CI, confidence intervals; MR: Mendelian Randomization; OR: odds ratio; MR-PRESSO: MR pleiotropy residual sum and outlier test  * Excluding leisure television watching/leisure computer use SNPs which are genome-wide significant for the secondary trait  † P-value or pleiotropy based on MR-Egger intercept  ‡ P-value for heterogeneity based on Q statistic | | | | | | | | | | |

| **Table S38: Mendelian Randomization estimates for the effects of the secondary traits on sedentary behaviour** | | | | | | | | | | |
| --- | --- | --- | --- | --- | --- | --- | --- | --- | --- | --- |
| **Methods** | **Leisure television watching** | | | | | **Leisure computer use** | | | | |
|  | **Estimates** | **95% CI** | **P-value** |  | **P-value for pleiotropy^†^ or heterogeneity^‡^** | **Estimates (OR)** | **95% CI** | **P-value** |  | **P-value for pleiotropy^†^ or heterogeneity^‡^** |
| **Education** |  |  |  |  |  |  |  |  |  |  |
| Inverse-variance weighted | -0.63 | -0.66, -0.59 | 2×10^-277^ |  | 2×10^-81^ | 0.34 | 0.30, 0.37 | 2×10^-83^ |  | 5×10^-70^ |
| MR-Egger | -0.64 | -0.77, -0.52 | 0 |  | 0.77 | 0.42 | 0.30, 0.54 | 2×10^-11^ |  | 0.17 |
| Weighted median | -0.63 | -0.67, -0.60 | 4×10^-249^ |  |  | 0.34 | 0.30, 0.37 | 1×10^-76^ |  |  |
| Inverse-variance weighted* | -0.55 | -0.58, -0.52 | 8×10^-244^ |  | 4×10^-35^ | 0.30 | 0.27, 0.33 | 8×10^-75^ |  | 5×10^-45^ |
| **BMI** |  |  |  |  |  |  |  |  |  |  |
| Inverse-variance weighted | 0.23 | 0.21, 0.26 | 3×10^-63^ |  | 1×10^-256^ | 0.02 | -0.01, 0.04 | 0.14 |  | 5×10^-80^ |
| MR-Egger | 0.09 | 0.02, 0.16 | 0.02 |  | 5×10^-6^ | 0.01 | -0.04, 0.07 | 0.62 |  | 0.94 |
| Weighted median | 0.20 | 0.18, 0.23 | 4×10^-54^ |  |  | 0.01 | -0.01, 0.03 | 0.44 |  |  |
| Inverse-variance weighted* | 0.20 | 0.17, 0.22 | 1×10^-57^ |  | 4×10^-134^ | 0.02 | 0.00, 0.04 | 0.08 |  | 3×10^-72^ |
| **Smoking** |  |  |  |  |  |  |  |  |  |  |
| Inverse-variance weighted | 0.49 | 0.40, 0.57 | 6×10^-29^ |  | 4×10^-54^ | -0.10 | -0.17, -0.02 | 0.01 |  | 1×10^-29^ |
| MR-Egger | -0.17 | -0.49, 0.15 | 0.29 |  | 3×10^-5^ | 0.08 | -0.21, 0.37 | 0.60 |  | 0.22 |
| Weighted median | 0.52 | 0.44, 0.60 | 3×10^-38^ |  |  | -0.11 | -0.18, -0.04 | 0.004 |  |  |
| Inverse-variance weighted* | 0.45 | 0.37, 0.53 | 2×10^-28^ |  | 1×10^-38^ |  |  |  |  |  |
| **Alcohol consumption** |  |  |  |  |  |  |  |  |  |  |
| Inverse-variance weighted | 0.02 | -0.07, 0.10 | 0.67 |  | 7×10^-50^ | -0.09 | -0.17, -0.01 | 0.03 |  | 2×10^-38^ |
| MR-Egger | 0.07 | -0.09, 0.24 | 0.39 |  | 0.46 | -0.19 | -0.34, -0.03 | 0.02 |  | 0.15 |
| Weighted median | 0.14 | 0.07, 0.21 | 8×10^-4^ |  |  | -0.16 | -0.24, -0.09 | 1×10^-5^ |  |  |
| Inverse-variance weighted* | 0.04 | -0.02, 0.27 | 0.09 |  | 5×10^-31^ |  |  |  |  |  |
| Abbreviations: CI, confidence intervals; MR: Mendelian Randomization; OR: odds ratio; MR-PRESSO: MR pleiotropy residual sum and outlier test  * Excluding secondary traits related SNPs which are genome-wide significant for leisure television watching/leisure computer use  † P-value or pleiotropy based on MR-Egger intercept  ‡ P-value for heterogeneity based on Q statistic | | | | | | | | | | |

| **Table S39: Multivariable Mendelian randomization estimates for years of education and cancer risk** | | | | | | |
| --- | --- | --- | --- | --- | --- | --- |
|  | **Years of education** | | | | | |
|  | **Leisure television watching analysis** | | |  | **Leisure computer use analysis** | |
|  | **Estimates (OR)** | **95% CI** |  | | **Estimates (OR)** | **95% CI** |
| **Breast cancer** | 0.92 | 0.78, 1.09 |  | | 0.89 | 0.79, 0.99 |
| **Luminal A breast cancer** | 0.90 | 0.72, 1.11 |  | | 0.81 | 0.70, 0.94 |
| **Luminal B breast cancer** | 0.84 | 0.58, 1.20 |  | | 1.11 | 0.86, 1.45 |
| **Luminal B HER2 negative breast cancer** | 0.92 | 0.68, 1.27 |  | | 0.81 | 0.66, 1.01 |
| **HER2 enriched breast cancer** | 0.76 | 0.44, 1.28 |  | | 0.82 | 0.57, 1.17 |
| **Triple negative breast cancer** | 0.81 | 0.61, 1.08 |  | | 0.90 | 0.73, 1.11 |
|  |  |  |  | |  |  |
| **Colorectal cancer** | 0.73 | 0.60, 0.90 |  | | 0.71 | 0.61, 0.82 |
| **Colorectal cancer in men** | 0.67 | 0.52, 0.88 |  | | 0.68 | 0.56, 0.82 |
| **Colorectal cancer in women** | 0.80 | 0.61, 1.05 |  | | 0.74 | 0.61, 0.89 |
| **Colon cancer** | 0.71 | 0.57, 0.90 |  | | 0.70 | 0.59, 0.83 |
| **Rectal cancer** | 0.59 | 0.43, 0.82 |  | | 0.61 | 0.49, 0.76 |
| Abbreviations: CI, confidence intervals; OR: odds ratio | | | | | | |

**Figure S1**: Scatter plots showing the correlation of genetic associations of leisure television watching with genetic associations with breast cancer. Coloured lines represent the slopes of the different regression analyses.

**Figure S2**: Scatter plots showing the correlation of genetic associations of leisure television watching with genetic associations with colorectal cancer. Coloured lines represent the slopes of the different regression analyses.

**Figure S3**: Scatter plots showing the correlation of genetic associations of leisure television watching with genetic associations with prostate cancer. Coloured lines represent the slopes of the different regression analyses.

**Figure S4**: Funnel plots of risk estimates of leisure television watching and breast cancer against instrumental strength

**Figure S5**: Funnel plots of risk estimates of leisure television watching and colorectal cancer against instrumental strength

**Figure S6**: Funnel plots of risk estimates of leisure television watching and prostate cancer against instrumental strength

**Figure S7**: Scatter plots showing the correlation of genetic associations of leisure computer use with genetic associations with breast cancer. Coloured lines represent the slopes of the different regression analyses.

**Figure S8**: Scatter plots showing the correlation of genetic associations of leisure computer use with genetic associations with colorectal cancer. Coloured lines represent the slopes of the different regression analyses.

**Figure S9**: Scatter plots showing the correlation of genetic associations of leisure computer use with genetic associations with prostate cancer. Coloured lines represent the slopes of the different regression analyses

**Figure S10**: Funnel plots of risk estimates of leisure computer use and breast cancer against instrumental strength

**Figure S11**: Funnel plots of risk estimates of leisure computer use and colorectal cancer against instrumental strength

**Figure S12**: Funnel plots of risk estimates of leisure computer use and prostate cancer against instrumental strength
